# Plant-based diet quality and risk of age-related eye diseases: evidence from multi-cohorts with multi-omics insights

**DOI:** 10.1038/s41538-026-00919-z

**Published:** 2026-06-04

**Authors:** Xuehao Cui, Jingwen Hui, Qiuchen Zhao, Quanhong Han

**Affiliations:** 1 Department of Clinical Neurosciences, University of Cambridge, Cambridge, UK; 2 John van Geest Centre for Brain Repair, University of Cambridge, Cambridge, UK; 3 Cambridge Eye Unit, Addenbrooke’s Hospital, Cambridge University Hospitals NHS Foundation Trust, Cambridge, UK; 4 Tianjin Eye Hospital, No.4 Gansu Road, Heping District, Tianjin, China; 5 Tianjin Key Lab of Ophthalmology and Visual Science, Tianjin, China; 6 School of Medicine, Nankai University, Tianjin, China; 7 Department of Pathology, University of Cambridge, Cambridge, UK; 8 Cancer Research UK Cambridge Centre and Department of Oncology, University of Cambridge, Cambridge, UK

**Keywords:** Biomarkers, Computational biology and bioinformatics, Diseases, Health care, Medical research, Risk factors

## Abstract

Plant-based diets may influence age-related eye diseases (AREDs), but whether ocular benefits depend on diet quality remains unclear. We examined associations of a healthy plant-based diet index (PDI-H) and an unhealthy plant-based diet index (PDI-U) with age-related macular degeneration (AMD), cataract, glaucoma, diabetic retinopathy (DR), and retinal vein occlusion (RVO) using the UK Biobank prospective cohort and an independent Tianjin hospital-based cross-sectional sample. Cox regression, logistic regression, propensity score matching, restricted cubic splines, subgroup analyses, multi-omics profiling, and machine learning were applied. Higher PDI-H was associated with lower risks of AMD, cataract, glaucoma, and DR, whereas higher PDI-U was associated with increased risks of AMD, cataract, and DR. Associations with RVO were weak or absent. Findings from Tianjin were directionally consistent with UK Biobank results and should be interpreted as supportive cross-sectional evidence. Proteomic and metabolomic analyses linked plant-based diet quality to inflammation, lipid metabolism, glycaemic regulation, and hormonal signalling. Predictive models incorporating dietary indices showed good discrimination for AMD and cataract, with modest but consistent AUC improvements after adding PDI-H and PDI-U to traditional risk factors. These findings suggest that plant-based diet quality, rather than plant-based eating alone, may be a modifiable determinant of major AREDs.

## Introduction

Age-related eye diseases (ARED), including age-related macular degeneration (AMD), cataract, glaucoma, diabetic retinopathy (DR), and retinal vein occlusion (RVO), are leading causes of visual impairment and blindness in older adults^1–4^. These conditions share common risk factors such as aging, smoking, hypertension, and diabetes, but there is growing interest in the role of diet and nutrition in their development^5–7^. Diet is a readily modifiable factor that influences systemic inflammation, metabolic health, and antioxidant status—processes implicated in the pathogenesis of ocular diseases^8–10^. In particular, plant-based diets rich in fruits, vegetables, and whole grains are thought to confer health benefits^11,12^. In contrast, diets high in refined carbohydrates and processed foods can exacerbate metabolic and vascular dysfunction^13,14^. However, the relevance of overall plant-based diet quality to age-related eye disease risk remains incompletely understood.

Recently, diet indices have been developed to quantify adherence to plant-based diets and to distinguish healthy from unhealthy plant-based food choices^15–17^. Two such indices are the healthy plant-based diet index (PDI-H) and the unhealthy plant-based diet index (PDI-U)^18^. PDI-H emphasizes higher intake of healthful plant foods and lower intake of both animal foods and less healthy plant foods^19^. In contrast, PDI-U emphasizes greater consumption of unhealthy plant-based foods and lower intake of nutritious plant foods and animal products^18^. These indices provide a nuanced assessment of diet quality that goes beyond a simple binary classification of diets as “plant-based” or not^20^. High PDI-H scores represent a nutritious plant-focused eating pattern, whereas high PDI-U scores reflect a plant-based diet dominated by processed or sugary foods^21^.

While adherence to healthy plant-based diets has been associated with lower risks of cardiovascular disease, type 2 diabetes, and mortality in prior studies, its relationship with eye-specific outcomes like AMD, cataract, glaucoma, DR, and RVO has not been comprehensively investigated^22,23^. There is evidence that specific nutrients found predominantly in plant foods—for example, lutein and zeaxanthin for AMD or vitamin C for cataract—can protect ocular tissues^24–26^. Yet, focusing on isolated nutrients or foods may not capture the broader impact of overall diet patterns. Using PDI-H and PDI-U allows assessment of whether diets rich in wholesome plant foods versus those high in less healthy plant-based choices differentially influence eye health^27^. Moreover, examining both “healthy” and “unhealthy” plant-based diet scores can clarify whether simply following a plant-based diet is sufficient for ocular benefits, or whether the quality of the plant foods consumed is critical.

In addition, the biological mechanisms linking diet to eye disease need elucidation. Age-related degenerative eye conditions often involve complex pathophysiology, including oxidative damage, chronic low-grade inflammation, dyslipidemia, and microvascular changes^9,28,29^. A healthy plant-based diet may mitigate these processes through high antioxidant intake, anti-inflammatory effects, improved lipid and glucose metabolism, and modulation of hormonal and growth factor pathways^30–32^. In contrast, an unhealthy plant-based diet could worsen systemic inflammation, contribute to hyperglycaemia and dyslipidemia, and thereby accelerate ocular damage^33^.

In this study, we aimed to investigate the associations of plant-based diet quality—quantified by PDI-H and PDI-U—with the risk of five major ARED: AMD, cataract, glaucoma, DR, and RVO. We leveraged data from two complementary datasets: the UK Biobank (UKB) prospective cohort and an independent hospital-based cross-sectional sample from Tianjin, China. Using these complementary datasets, we sought to determine the consistency of any diet-eye disease associations across populations. We further conducted extensive analyses to explore potential mechanisms, including restricted cubic spline dose-response analyses, subgroup analyses, propensity score matching, and multi-omics profiling. We also applied machine learning models to evaluate the contribution of diet indices to overall eye disease risk prediction and to assess the translational potential of our findings.

## Results

### Participant characteristics and distribution of PDIs

A total of 134,975 participants from the UKB prospective cohort and 5977 participants from the Tianjin hospital-based cross-sectional sample were included in the analyses. Participants who developed AREDs during follow-up in UKB or who had an ARED at baseline in the Tianjin hospital-based cross-sectional sample were generally older and more likely to have risk factors such as smoking, hypertension, and diabetes mellitus than those without the corresponding diseases (Supplementary Table 1). In UKB, individuals who went on to develop AMD had lower PDI-H scores than those who did not develop AMD (32.86 vs 33.88) and higher PDI-U scores (36.17 vs 35.19). Similarly, patients with cataract had slightly lower PDI-H (33.51 vs 33.89) and higher PDI-U (35.82 vs 35.16). DR patients had a notably lower PDI-H (32.43 vs 33.87) and a slightly higher PDI-U (35.80 vs 35.21). In contrast, no meaningful differences in baseline PDI-H or PDI-U were observed in RVO. The Tianjin hospital-based cross-sectional sample showed analogous patterns: participants with AMD, cataract, or DR had significantly lower PDI-H and higher PDI-U than disease-free individuals, whereas differences for glaucoma were minor and for RVO were not significant.

Baseline characteristics across quintiles of the plant-based diet indices are summarized in Supplementary Table 2. Higher PDI-H was consistently associated with a more favourable cardiometabolic and lifestyle profile in both cohorts, including lower BMI, lower prevalence of smoking and diabetes, and reduced systemic inflammation as reflected by lower C-reactive protein levels. Lipid profiles were modestly more favourable among participants with higher PDI-H, while glycaemic markers showed minimal variation. In contrast, higher PDI-U was generally associated with less favourable metabolic and lifestyle characteristics, including higher adiposity, greater smoking prevalence, and higher inflammatory burden. Overall, these patterns indicate that PDI-H and PDI-U capture opposing dimensions of overall diet quality and cardiometabolic risk.

In the UKB cohort, PDI-H and PDI-U followed approximately normal distributions (Supplementary Fig. 1A). PDI-H was higher among women and participants without hypertension or diabetes and showed transparent gradients across educational attainment, household income, smoking status, and alcohol consumption (Supplementary Fig. 1B–C). In contrast, PDI-U varied little by sex (Supplementary Fig. 1D) but was higher among participants with lower socioeconomic status, current smoking, and cardiometabolic comorbidities (Supplementary Fig. 1D, E). Similar distribution patterns were observed in the Tianjin cohort. Both PDI-H and PDI-U were approximately normally distributed (Supplementary Fig. 2A). PDI-H differed modestly by sex and education level and tended to be lower among current smokers (Supplementary Fig. 2B, 2E). In contrast, PDI-U showed variation primarily by income level and exhibited weaker associations with lifestyle and metabolic factors than in the UK Biobank (Supplementary Fig. 2C, D).

### Associations of PDIs with ARED risk

**Fig. 1:**
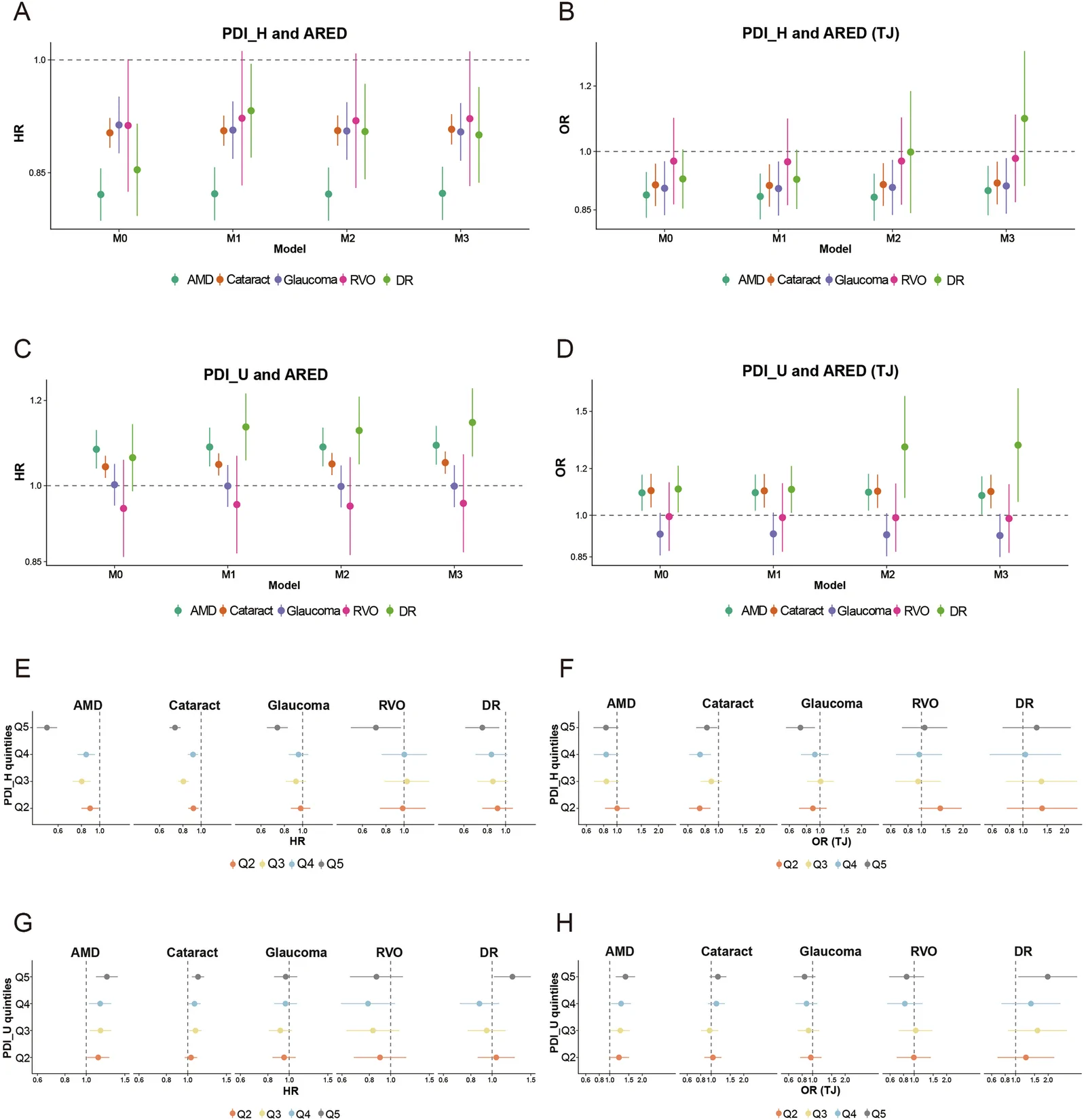
Associations of PDI-H and PDI-U with ARED risk. **A** Prospective associations between PDI-H and incident AREDs in UKB using Cox models with increasing covariate adjustment (M0–M3). **B** Cross-sectional associations between PDI-H and prevalent AREDs in the Tianjin hospital-based sample using logistic regression. **C** Prospective associations between PDI-U and incident AREDs in UKB. **D** Cross-sectional associations between PDI-U and prevalent AREDs in the Tianjin hospital-based sample. **E**, **F** Quintile analyses of PDI-H in UKB (hazard ratios) and the Tianjin sample (odds ratios). **G**, H Quintile analyses of PDI-U in UKB and the Tianjin sample. Points represent effect estimates and bars indicate 95% confidence intervals.

In the UKB cohort, higher adherence to a PDI-H was consistently associated with a lower risk of age-related eye diseases across progressively adjusted models (Fig. 1A). These inverse associations were most evident for AMD, cataract, glaucoma, and DR. They remained stable from the minimally adjusted model to the fully adjusted model (M3). In contrast, no significant association was observed for RVO. Directionally comparable associations were observed in the Tianjin hospital-based cross-sectional sample, in which higher PDI-H was associated with lower odds of AMD, cataract, and glaucoma. In contrast, associations with DR and RVO were weaker or null (Fig. 1B). Corresponding effect estimates from both cohorts are summarized in Supplementary Table 3. In contrast, higher scores on the PDI-U were associated with increased risk of several AREDs. In the UKB, higher PDI-U was positively related to the risk of AMD, cataract, and DR across all models, whereas associations with glaucoma and RVO were not statistically significant (Fig. 1C). Similar cross-sectional patterns were observed in the Tianjin sample (Fig. 1D; Supplementary Table 3). Quintile-based analyses further supported these findings. Increasing quintiles of PDI-H were associated with progressively lower risks of AMD, cataract, glaucoma, and diabetic retinopathy in the UK Biobank, with consistent gradients observed in the Tianjin sample (Fig. 1E, F). Conversely, increasing quintiles of PDI-U were associated with higher risks of AMD, cataract, and diabetic retinopathy, whereas no precise dose–response pattern was evident for glaucoma or RVO (Fig. 1G, H).

**Fig. 2:**
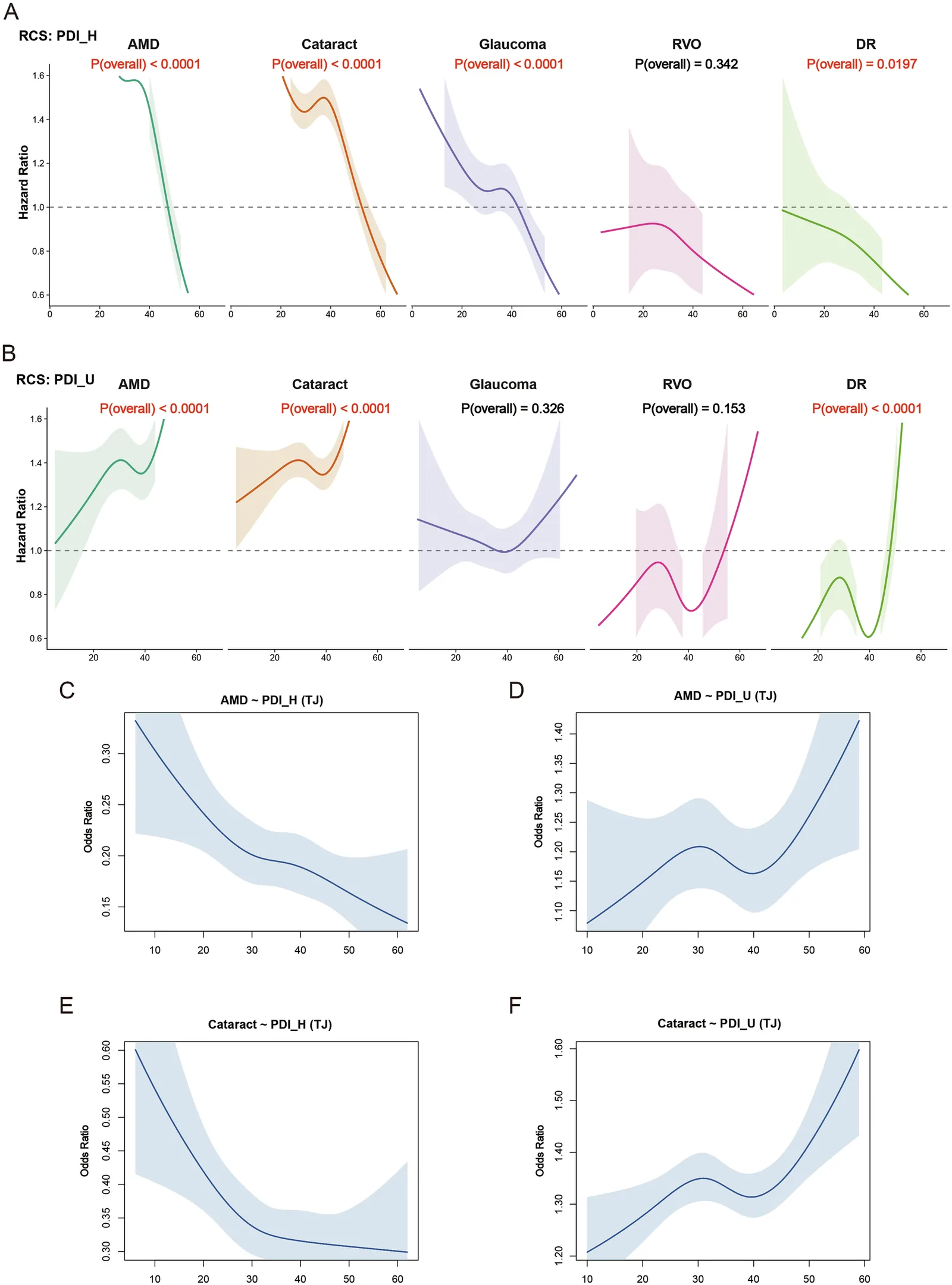
Dose–response relationships between PDIs and AREDs. **A** Restricted cubic spline (RCS) analyses showing dose–response relationships between PDI-H and ARED risk in UKB. **B** RCS analyses PDI-U and ARED risk in UKB. **C**–**F** RCS analyses for AMD and cataract in the TJ cohort. Solid lines represent estimated hazard or odds ratios, and shaded areas indicate 95% confidence intervals. *P* values for overall associations are shown.

Restricted cubic spline analyses demonstrated largely monotonic dose–response relationships. In the UK Biobank, PDI-H showed significant inverse associations with AMD, cataract, glaucoma, and diabetic retinopathy, while no significant association was observed for RVO (Fig. 2A). PDI-U exhibited significant positive dose–response relationships with AMD, cataract, and diabetic retinopathy, but not with glaucoma or RVO (Fig. 2B). In the Tianjin cohort, spline analyses for AMD and cataract revealed similar patterns, with lower risk at higher PDI-H levels and higher risk at higher PDI-U levels (Fig. 2C–F). Subgroup analyses in the UK Biobank demonstrated that the associations of PDI-H and PDI-U with AMD, cataract, diabetic retinopathy, and glaucoma were broadly consistent across strata defined by age, sex, smoking status, alcohol consumption, and hypertension status, with no firm evidence of effect modification (Supplementary Fig. 3A–G). In the Tianjin cohort, subgroup analyses restricted to age and sex for AMD and cataract showed similar directions of association for both PDI-H and PDI-U (Supplementary Fig. 4A–D).

In ROC analyses using dietary indices alone, both PDI-H and PDI-U showed moderate discriminatory performance for several ARED outcomes (Supplementary Fig. 5A–J). For PDI-H, the AUC was 0.729 for AMD (Supplementary Fig. 5A), 0.717 for cataract (Supplementary Fig. 5B), 0.701 for glaucoma (Supplementary Fig. 5C), 0.711 for RVO (Supplementary Fig. 5D), and was highest for diabetic retinopathy (AUC = 0.930; Supplementary Fig. 5E). For PDI-U, the AUC was 0.725 for AMD (Supplementary Fig. 5F), 0.716 for cataract (Supplementary Fig. 5G), 0.698 for glaucoma (Supplementary Fig. 5H), 0.929 for diabetic retinopathy (Supplementary Fig. 5I), and 0.710 for RVO (Supplementary Fig. 5J). Overall, both indices demonstrated limited discrimination for AMD and cataract, weaker performance for glaucoma and RVO, and substantially higher discrimination for DR. The substantially higher AUCs observed for diabetic retinopathy should be interpreted cautiously. Because DR cases were necessarily drawn from participants with diabetes, whereas controls were derived from the broader disease-free population, these AUCs likely capture broader diabetes-related and cardiometabolic differences rather than DR-specific discrimination by dietary indices alone.

### Propensity-matched analyses

**Fig. 3:**
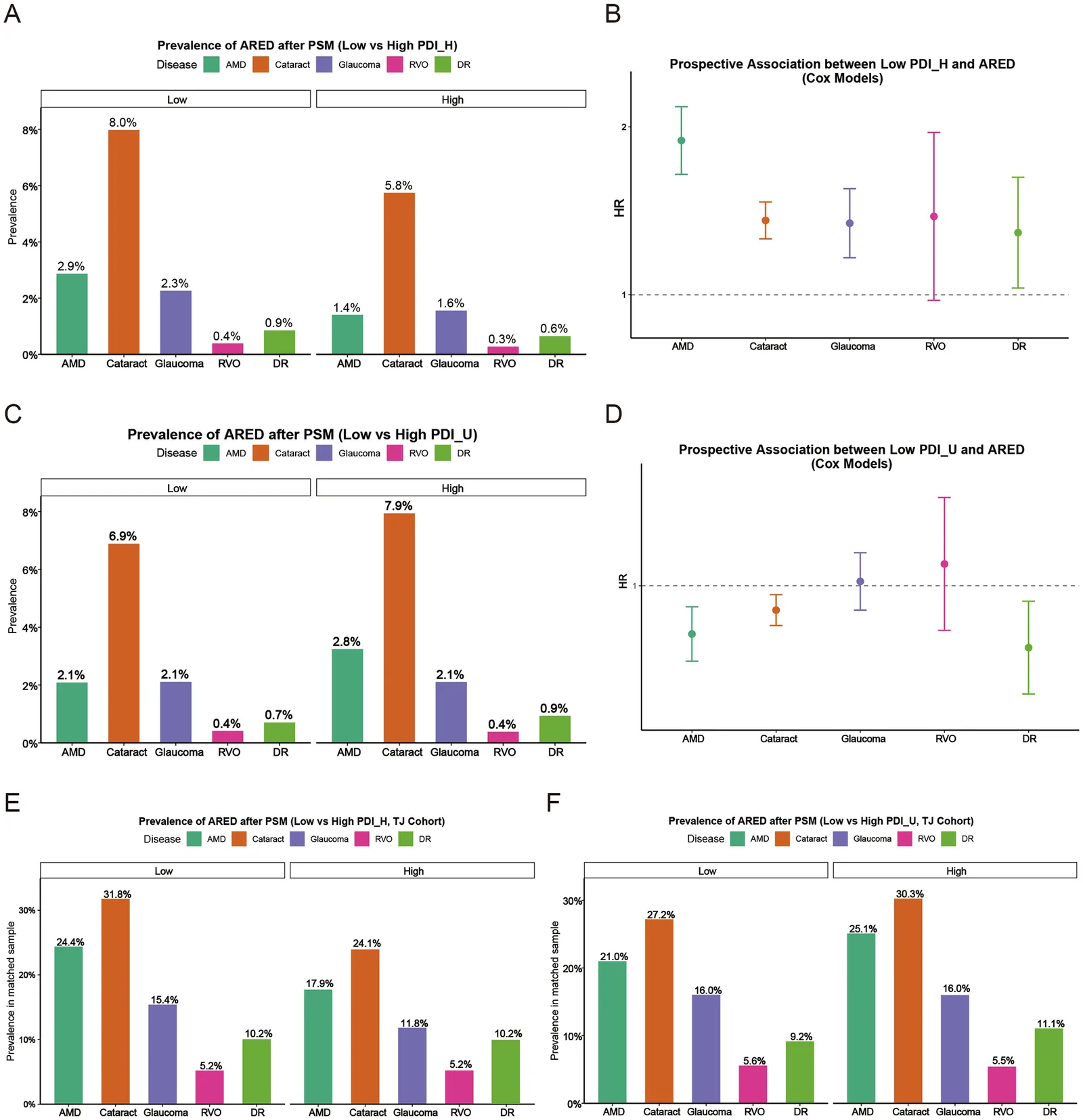
Propensity score–matched analyses. **A** Prevalence of AREDs after propensity score matching (PSM) comparing low vs high PDI-H groups in UKB. **B** Cox model estimates for low vs high PDI-H after PSM in UKB. **C** Prevalence of AREDs after PSM compared low vs high PDI-U groups in UKB. **D** Cox model estimates for low vs high PDI-U after PSM in UKB. **E**, **F** Prevalence of AREDs after PSM in the TJ cohort for PDI-H and PDI-U, respectively. Bars indicate disease prevalence in matched samples.

After propensity score matching, baseline covariates between low and high diet quality groups were well balanced in both cohorts (Supplementary Fig. 6A, C, E, G). In the UKB, participants with high PDI-H showed consistently lower prevalences of several age-related eye diseases compared with those with low PDI-H. Specifically, the prevalence of AMD decreased from 2.9% in the low PDI-H group to 1.4% in the high PDI-H group; cataract from 8.0 to 5.8%; glaucoma from 2.3 to 1.6%; and DR from 0.9 to 0.6%. In contrast, the prevalence of RVO remained low and essentially unchanged (0.4% vs 0.3%) (Fig. 3A). Correspondingly, Cox models in the matched sample showed elevated risks for AMD, cataract, glaucoma, and DR among participants with low PDI-H. At the same time, the association with RVO was weak and imprecise (Fig. 3B).

In contrast, high PDI-U was associated with higher disease prevalence after matching. In the UKB, AMD prevalence increased from 2.1% in the low PDI-U group to 2.8% in the high PDI-U group, cataract prevalence from 6.9 to 7.9%, and DR prevalence from 0.7 to 0.9%. In contrast, glaucoma prevalence was similar between the two groups (2.1%). RVO showed minimal differences (0.4% in both groups) (Fig. 3C). These patterns were mirrored in Cox regression analyses, in which low PDI-U was associated with lower risks of AMD, cataract, and DR, but not glaucoma or RVO (Fig. 3D).

Similar results were observed in the Tianjin hospital-based sample. After matching, higher PDI-H was associated with lower prevalences of AMD (24.4% vs 17.9%) and cataract (31.8% vs 24.1%), while differences for glaucoma, RVO, and DR were modest (Fig. 3E). Conversely, higher PDI-U was associated with higher prevalences of AMD (21.0% vs 25.1%), cataract (27.2% vs 30.3%), and DR (9.2% vs 11.1%) (Fig. 3F). Logistic regression analyses in the matched TJ sample showed consistent directions of association, with more potent effects for AMD and cataract and weaker or non-significant associations for glaucoma and RVO (Supplementary Fig. 6F, H).

### Proteomic associations linking PDIs to ARED

**Fig. 4:**
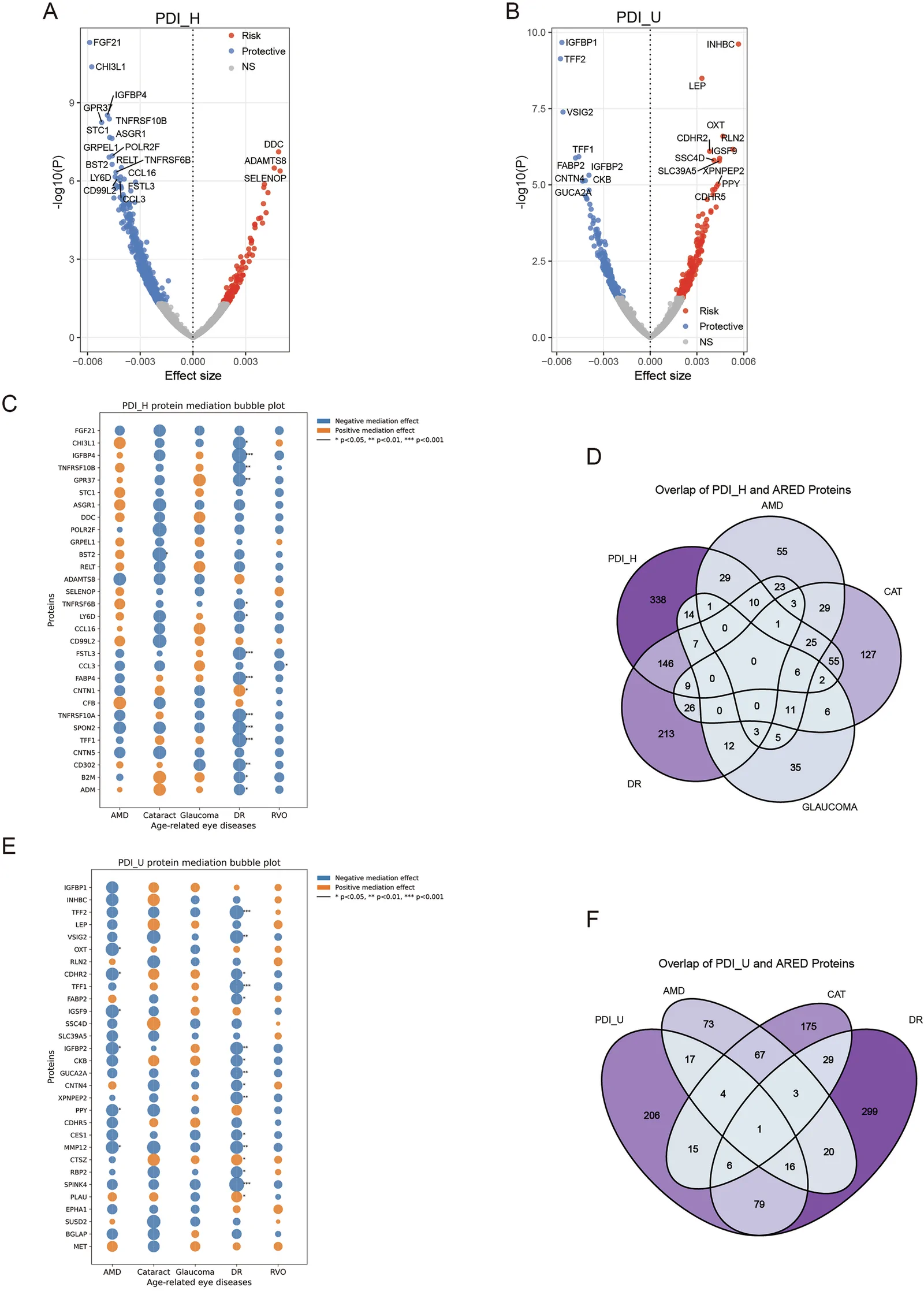
Proteomic associations of PDI-H and PDI-U. **A** Volcano plot showing associations between circulating proteins and PDI-H. The x-axis represents effect size and the y-axis represents −log10(*P* value); labelled proteins are shown using standard uppercase nomenclature. **B** Volcano plot showing associations between circulating proteins and PDI-U. The x-axis represents effect size and the y-axis represents −log10(*P* value); labelled proteins are shown using standard uppercase nomenclature. **C** Bubble plot summarizing exploratory mediation effects of the top 30 PDI-H-associated proteins on the associations between PDI-H and individual AREDs. **D** Overlap between PDI-H-associated proteins and ARED-associated proteins. **E** Bubble plot summarizing exploratory mediation effects of the top 30 PDI-U-associated proteins on the associations between PDI-U and individual AREDs. **F** Overlap between PDI-U-associated proteins and ARED-associated proteins. In panels C and E, bubble size represents the absolute mediation effect, orange indicates a positive mediation effect, blue indicates a negative mediation effect, and asterisks denote nominal mediation P values (*P < 0.05, **P < 0.01, ***P < 0.001). Full indirect effect estimates, confidence intervals, P values, and FDR-adjusted q values are provided in Supplementary Table 19.

Proteins significantly associated with PDI-H and PDI-U are summarized in Supplementary Table 4 and Supplementary Table 5, respectively, and visualized in Fig. 4A, B. Higher PDI-H was associated with a broad set of circulating proteins, showing inverse associations, whereas fewer proteins were positively associated with PDI-H. In contrast, higher PDI-U was associated with more proteins showing positive associations and fewer proteins showing inverse associations. Exploratory mediation analyses of the top 30 diet-associated proteins are summarized in Fig. 4C for PDI-H and Fig. 4E for PDI-U. Several proteins showed nominally significant indirect effects linking plant-based diet quality to AMD, cataract, glaucoma, DR, and RVO, with the largest number of mediation signals observed for AMD, cataract, and DR. Full mediation estimates, including signed indirect effects, 95% confidence intervals, nominal P values, and FDR-adjusted q values, are provided in Supplementary Table 19. The direction of mediation was heterogeneous across molecules and outcomes, supporting a biologically complex network rather than a single uniform pathway. These findings strengthen the biological plausibility of the diet–disease associations, but should be interpreted as exploratory and hypothesis-generating rather than definitive evidence of causal mediation. These patterns were consistent across multiple disease endpoints and are detailed in Supplementary Tables 8–12, which list disease-specific protein associations for AMD, cataract, glaucoma, DR, and RVO. Overlap analyses identified substantial shared proteins between diet indices and ocular diseases, notably AMD, cataract, and DR (Fig. 4D, F). The number of overlapping proteins was highest for DR, intermediate for AMD and cataract, and relatively limited for glaucoma and RVO, consistent with the overall burden of systemic protein alterations observed across diseases.

Disease-specific proteomic signatures are further illustrated in Supplementary Fig. 7A–E. AMD, cataract, and DR were associated with more significantly altered proteins, whereas glaucoma and RVO were associated with fewer proteins that were considerably changed. Functional enrichment analyses of diet–disease overlapping proteins are shown in Supplementary Fig. 8A–D. Proteins associated with higher PDI-H and lower disease risk were enriched in pathways related to immune regulation, leucocyte migration, cytokine signalling, and regulation of protease activity. In contrast, proteins associated with higher PDI-U and increased disease risk were enriched in pathways related to hormonal signalling, protein kinase activity, metabolic processes, and cell migration. These enrichment patterns were consistent across AMD and cataract, with similar but attenuated signals observed for DR.

### Metabolomic associations linking PDIs to ARED

**Fig. 5:**
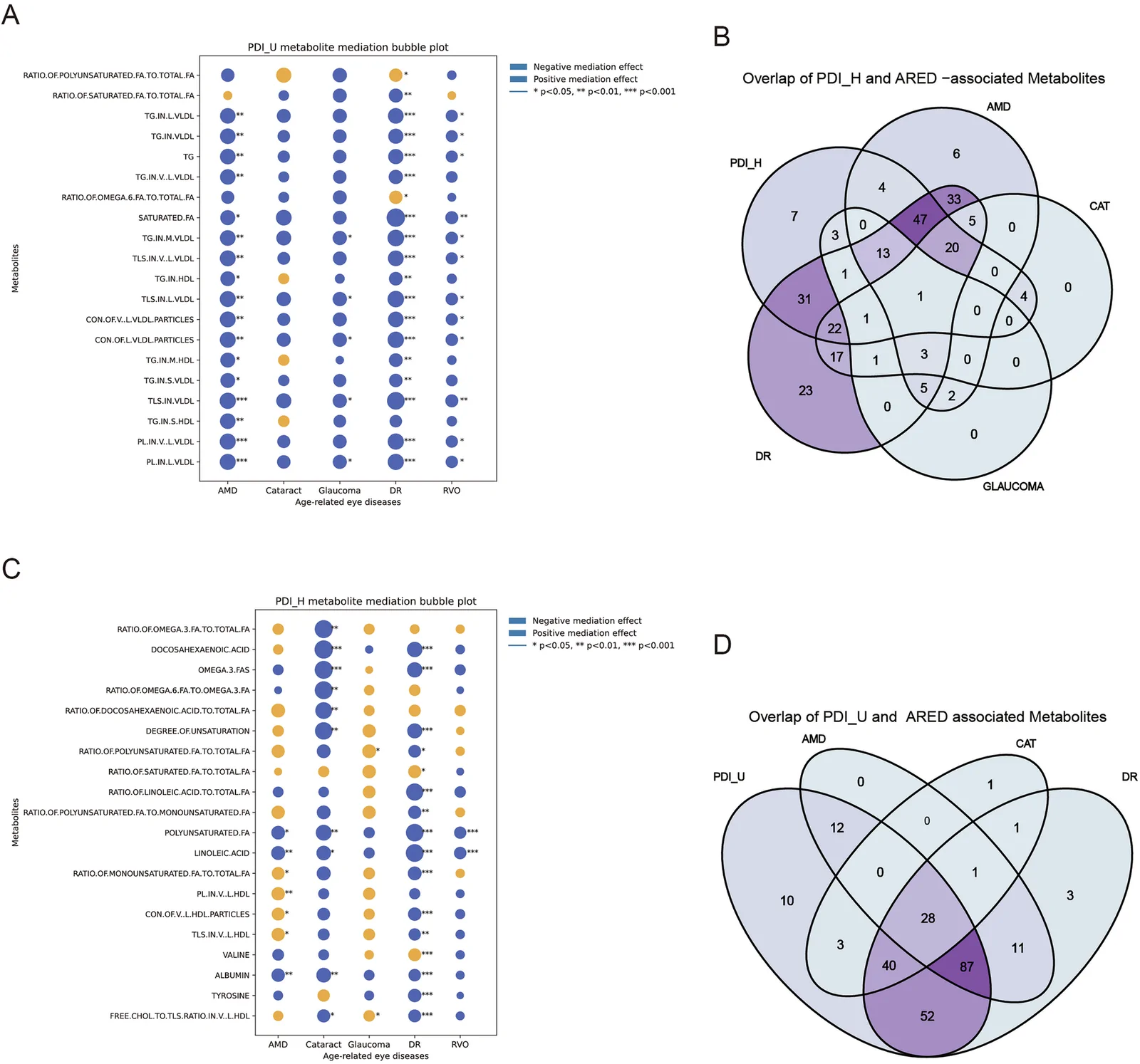
Metabolomic associations of PDI-H and PDI-U. **A** Bubble plot summarizing exploratory mediation effects of the top 20 PDI-U-associated metabolites on the associations between PDI-U and individual AREDs. **B** Overlap between PDI-H-associated metabolites and ARED-associated metabolites. **C** Bubble plot summarizing exploratory mediation effects of the top 20 PDI-H-associated metabolites on the associations between PDI-H and individual AREDs. **D** Overlap between PDI-U-associated metabolites and ARED-associated metabolites. In panels **A** and **C**, bubble size represents the absolute mediation effect, orange indicates a positive mediation effect, blue indicates a negative mediation effect, and asterisks denote nominal mediation P values (*P < 0.05, **P < 0.01, ***P < 0.001). Full indirect effect estimates, confidence intervals, nominal P values, and FDR-adjusted q values are provided in Supplementary Table 19.

Metabolites associated with PDI-H and PDI-U are summarized in Supplementary Table 6 and Supplementary Table 7, respectively. Exploratory mediation analyses of the top 20 diet-associated metabolites are shown in Fig. 5A, C. Multiple metabolites demonstrated nominally significant indirect effects linking plant-based diet quality to AMD, cataract, glaucoma, DR, and RVO, with mediation signals again being most prominent for AMD, cataract, and DR. Full metabolite-level mediation estimates, including signed indirect effects, 95% confidence intervals, nominal P values, and FDR-adjusted q values, are reported in Supplementary Table 19. As with the proteomic analyses, these metabolite-level mediation results should be interpreted conservatively as supportive and hypothesis-generating rather than causal. Higher PDI-H was associated with a favourable metabolic profile characterized by inverse associations with triglyceride-rich lipoproteins, cholesterol-related measures, and markers of dyslipidaemia. In contrast, higher PDI-U was related to positive associations with LDL- and VLDL-related metabolites, triglycerides, and markers of altered glucose–lactate metabolism. Overlap analyses revealed that a substantial proportion of diet-associated metabolites were also associated with ocular disease risk (Fig. 5B, D). The most significant overlap was observed between AMD and DR, followed by cataract; fewer overlapping metabolites were identified for glaucoma and RVO.

Disease-specific metabolite associations are detailed in Supplementary Fig. 9A–G, Supplementary Tables 13–17. AMD and DR were strongly associated with lipid particle composition, fatty acid balance, and lipoprotein subclass measures, whereas cataract was additionally related to amino acid- and glucose-related metabolites. In contrast, glaucoma and RVO exhibited fewer significant metabolite associations, with smaller effect sizes and less consistent patterns across metabolic pathways. Collectively, the proteomic and metabolomic analyses demonstrate that plant-based diet quality is linked to coordinated alterations in circulating proteins and metabolites across multiple biological systems, with the most pronounced molecular signatures observed for AMD, cataract, and DR.

### Associations between PDIs, ocular metrics, and ARED

**Fig. 6:**
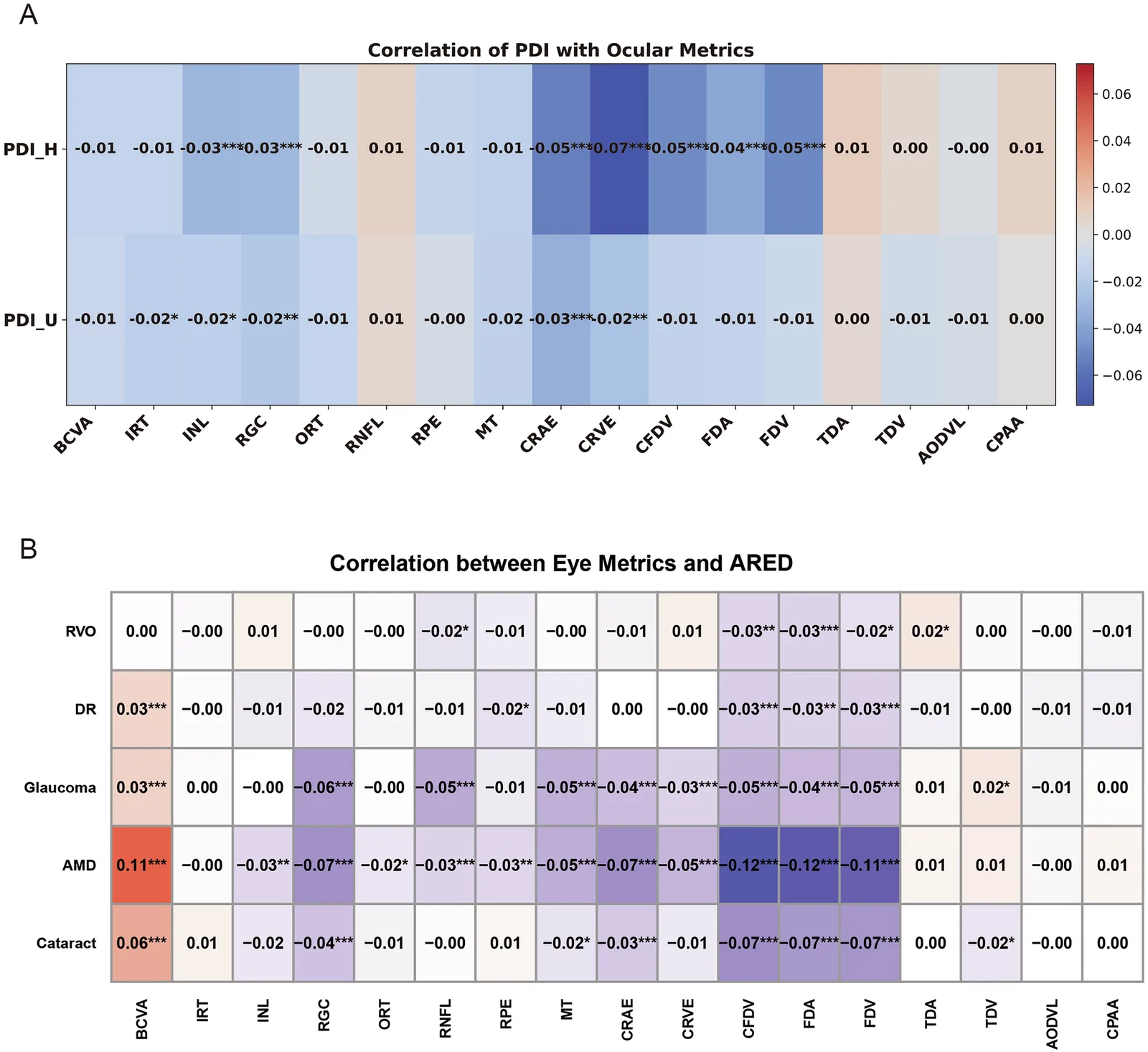
Associations between ocular metrics, PDIs, and AREDs. Multivariable-adjusted associations between PDI-H/PDI-U and ocular structural and vascular metrics. Values shown in each cell are standardized association coefficients. Asterisks denote FDR-adjusted significance after Benjamini–Hochberg correction across ocular metrics (*q < 0.05, **q < 0.01, ***q < 0.001). Associations between ocular metrics and ARED status. Colour intensity represents the magnitude and direction of standardized association coefficients. Asterisks denote FDR-adjusted significance using the same thresholds. Full estimates, nominal P values, and FDR-adjusted q values are provided in Supplementary Table 20.

Multivariable-adjusted analyses of ocular metrics are shown in Fig. 6A and detailed in Supplementary Table 20. Both PDI-H and PDI-U were associated with only modest differences in selected ocular structural and vascular parameters. After FDR correction, PDI-H was inversely associated with INL (β = −0.03, q = 5.08 × 10 − 4), CRAE (β = −0.05, q = 4.49 × 10 − 4), CRVE (β = −0.07, q = 7.61 × 10 − 5), CFDV (β = −0.05, q = 4.31 × 10 − 4), FDA (β = −0.04, q = 2.89 × 10 − 4), and FDV (β = −0.05, q = 6.34 × 10 − 5), and positively associated with RGC (β = 0.03, q = 4.29 × 10 − 4). PDI-U showed weaker but significant inverse associations with IRT, INL, RGC, CRAE, and CRVE. These relatively small effect sizes are consistent with the expectation that dietary quality is only one of several determinants of ocular imaging phenotypes.

Figure 6B addresses a complementary rather than competing question by summarizing which ocular metrics were associated with manifest ARED status. AMD and cataract showed broad associations with visual acuity, retinal ganglion cell metrics, macular structure, and retinal vascular parameters, whereas DR and RVO were characterized mainly by vascular alterations. Glaucoma showed prominent associations with retinal ganglion cell and nerve fibre layer metrics as well as vascular parameters. Full standardized coefficients, confidence intervals, nominal P values, and FDR-adjusted q values are provided in Supplementary Table 20.

Disease-specific comparisons of ocular parameters are presented in Supplementary Fig. 10A–E. In AMD, significant differences were observed for visual acuity, retinal ganglion cell metrics, macular thickness, and retinal vascular measures (Supplementary Fig. 10A). Cataract cases showed widespread differences across both structural and vascular parameters (Supplementary Fig. 10B). DR was primarily characterized by marked alterations in retinal vascular indices, including fractal dimensions and vessel density (Supplementary Fig. 10C). Glaucoma cases exhibited substantial reductions in retinal nerve fibre layer thickness, ganglion cell metrics, and associated vascular parameters (Supplementary Fig. 10D). In RVO, significant differences were confined mainly to vascular measures, with relatively limited changes in retinal thickness parameters (Supplementary Fig. 10E).

### Predictive machine learning model and translational implications

**Fig. 7:**
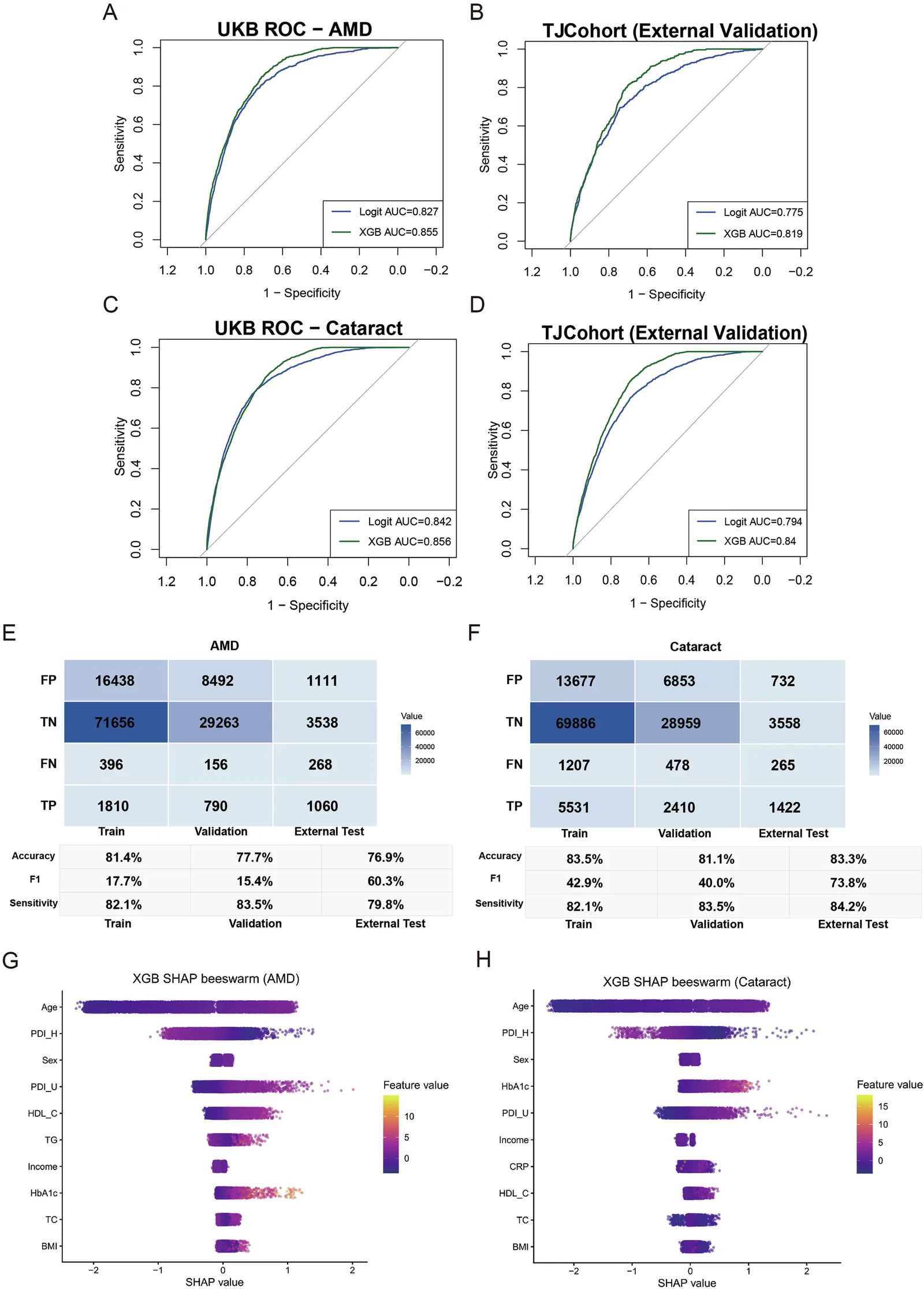
Predictive machine learning models for AMD and cataract. **A**–**D** ROC curves comparing logistic regression and XGBoost models for AMD and cataract in the UKB validation dataset and the Tianjin hospital-based cross-sectional sample. **E**, **F** Confusion matrices and performance metrics (accuracy, F1 score, and sensitivity) for AMD and cataract prediction across the training, validation, and Tianjin sample sets. **G**, **H** SHAP summary plots showing feature importance and directionality in XGBoost models for AMD and cataract. Each point represents an individual, coloured by feature value.

In the UKB validation dataset, both logistic regression and XGBoost models demonstrated good discriminative performance for AMD (Fig. 7A). The logistic regression model achieved an AUC of 0.827, while the XGBoost model showed improved discrimination with an AUC of 0.855. When evaluated in the Tianjin hospital-based cross-sectional sample, model performance remained acceptable (Fig. 7B), with AUCs of 0.775 for logistic regression and 0.819 for XGBoost, supporting cross-setting transportability rather than prospective external validation. Similar results were observed for cataract prediction. In UKB, the logistic regression model yielded an AUC of 0.842, and the XGBoost model achieved an AUC of 0.856 (Fig. 7C). Evaluation in the Tianjin sample showed sustained discrimination, with AUCs of 0.794 for logistic regression and 0.840 for XGBoost (Fig. 7D).

Confusion matrices and performance metrics further illustrated model behaviour (Fig. 7E, F). For AMD prediction, overall accuracy was 81.4% in the training set, 77.7% in the validation set, and 76.9% in the external test set, with sensitivities of 82.1%, 83.5%, and 79.8%, respectively (Fig. 7E). For cataract, accuracy reached 83.5% in the training set, 81.1% in the validation set, and 83.3% in the external test set, with sensitivities of 82.1%, 83.5%, and 84.2% (Fig. 7F). F1 scores were modest on the training and validation sets but increased substantially on the external test set, indicating improved balance between sensitivity and precision.

We further quantified the incremental predictive contribution of dietary indices by comparing otherwise identical models with and without PDI-H and PDI-U (Supplementary Fig. 11 and Supplementary Table 21). For AMD, adding PDI-H and PDI-U improved the AUC of logistic regression from 0.786 to 0.827 in UKB (ΔAUC = 0.041, 95% CI 0.024–0.058; DeLong P = 1.94 × 10 − 6) and from 0.736 to 0.775 in the Tianjin sample (ΔAUC = 0.039, 95% CI 0.023–0.055; DeLong P = 2.09 × 10 − 6). For XGBoost, the corresponding AUC increased from 0.812 to 0.855 in UKB (ΔAUC = 0.043, 95% CI 0.027–0.059; DeLong P = 1.55 × 10 − 7) and from 0.778 to 0.819 in the Tianjin sample (ΔAUC = 0.041, 95% CI 0.026–0.056; DeLong P = 1.08 × 10 − 7).

For cataract, adding PDI-H and PDI-U improved the AUC of logistic regression from 0.800 to 0.842 in UKB (ΔAUC = 0.042, 95% CI 0.032–0.052; DeLong P = 4.89 × 10 − 18) and from 0.754 to 0.794 in the Tianjin sample (ΔAUC = 0.040, 95% CI 0.026–0.054; DeLong P = 3.61 × 10 − 8). For XGBoost, the AUC increased from 0.813 to 0.856 in UKB (ΔAUC = 0.043, 95% CI 0.034–0.052; DeLong P = 8.48 × 10 − 20) and from 0.798 to 0.840 in the Tianjin sample (ΔAUC = 0.042, 95% CI 0.029–0.055; DeLong P = 3.65 × 10 − 10). These results indicate that PDI-H and PDI-U provided modest but consistent incremental discrimination beyond traditional demographic, lifestyle, metabolic, and clinical predictors.

Model interpretability was assessed using SHAP values for the XGBoost models (Fig. 7G, H). Age was the most influential predictor for both AMD and cataract. Notably, both PDI-H and PDI-U consistently ranked among the top contributors, alongside established clinical and metabolic variables, including sex, HbA1c, lipid markers, body mass index, and C-reactive protein. Higher PDI-H values were associated with lower predicted risk, whereas higher PDI-U values were associated with higher predicted risk, consistent with the main epidemiological findings. Together with the formal with-versus-without PDI comparisons, these SHAP results suggest that dietary quality provides complementary predictive information beyond traditional risk factors, although the absolute incremental gain was modest.

## Discussion

In this extensive, comprehensive investigation, we found that adherence to a healthy plant-based diet was significantly associated with a lower risk of AMD, cataract, glaucoma, and DR. In contrast, dietary patterns characterized by unhealthy plant-based foods were associated with higher risks of AMD, cataract, and DR; no clear association was observed with RVO. Importantly, the prospective associations observed in the UK Biobank were directionally supported by findings from an independent hospital-based cross-sectional sample in Tianjin. The TJ dataset therefore provides complementary external support across a different ethnic and dietary setting, but it should not be interpreted as confirmatory prospective replication.

Our findings are broadly consistent with, but extend beyond, existing epidemiological evidence suggesting that diets rich in fruits and vegetables are associated with lower risks of AMD and cataract. By explicitly distinguishing healthy from unhealthy plant-based dietary patterns, our study offers a more nuanced perspective on the role of plant-derived foods in ocular health. We demonstrate that not all plant-based diets confer equivalent benefits: protective associations were evident only when diets were rich in high-quality plant foods, whereas plant-based diets dominated by refined grains, sweets, and fried or highly processed foods were not protective and were associated with increased risk for several eye diseases. This distinction is clinically and publicly important, as generic recommendations promoting “plant-based diets” may be misinterpreted to include nutritionally poor plant-derived foods. Our results emphasize that dietary quality, rather than plant origin alone, is the critical determinant of ocular benefit. The directionally consistent associations across two markedly different settings are compatible with the involvement of shared biological processes, such as inflammation and metabolic regulation, rather than reliance on region-specific foods. However, because the Tianjin dataset was hospital-based and cross-sectional, these findings should be interpreted as supportive rather than confirmatory prospective replication. From a global health perspective, this supports the notion that dietary guidance for eye health may be broadly applicable across populations, with an emphasis on improving the quality of plant-based food intake.

Our multi-omics analyses, including exploratory mediation analyses of representative proteins and metabolites, provide biologically plausible clues to these associations rather than definitive mechanistic proof. Chronic inflammation appears to be a central pathway. We observed that higher PDI-H was associated with lower levels of systemic inflammatory markers, including CRP and inflammation-related circulating proteins. In contrast, higher PDI-U was associated with more pro-inflammatory profiles. Chronic inflammation has been implicated as a key driver of the pathogenesis of AMD and DR^34,35^. By reducing the systemic inflammatory burden, healthier dietary patterns may alleviate inflammatory stress in ocular tissues and thereby slow disease progression^36^. Proteomic analyses further showed that higher PDI-H was associated with lower circulating levels of several inflammatory proteins, including CXCL10. Because CXCL10 has been implicated in inflammatory responses relevant to glaucoma, this pattern may represent one possible pathway linking diet quality to glaucoma-related biology; however, our observational data do not establish that CXCL10 directly mediates a neuroprotective effect^37^.

Oxidative stress and lipid peroxidation represent another important mechanistic axis. The lens and retina are particularly vulnerable to oxidative damage due to lifelong exposure to light and high metabolic activity^38,39^. Diets rich in antioxidants and unsaturated fatty acids can enhance endogenous antioxidant capacity^40^. PDI-H captures high intake of fruits, vegetables, and nuts, which are significant sources of dietary antioxidants. We therefore hypothesize that higher PDI-H reflects increased systemic antioxidant potential. In contrast, PDI-U typically reflects diets with low antioxidant content and high pro-oxidative load. Consistent with this, we observed elevated levels of oxidative lipid–related markers, including Lp-PLA2 and advanced glycation end products, among individuals with poorer dietary quality^41^. These factors are known to damage retinal pigment epithelial cells and lens proteins, suggesting that healthy dietary patterns may protect ocular tissues by both supplying antioxidants and reducing oxidative stress.

In cataract, non-enzymatic glycation and cross-linking of lens proteins are key pathogenic mechanisms^42^. Diets high in refined carbohydrates can induce frequent hyperglycaemia and accelerate glycation of crystallins, leading to lens opacity^43^. Our findings suggest that higher PDI-U exacerbates glycaemic toxicity to the lens. In contrast, higher PDI-H, characterized by higher fibre intake and lower glycaemic load, promotes glycaemic stability and improved insulin sensitivity. This may partially explain the lower risks of cataract and DR observed among individuals with healthier dietary patterns. Indeed, in the UK cohort, higher PDI-H was associated with a lower prevalence of diabetes, and even among participants with diabetes, more nutritious diets were associated with a reduced risk of DR. Importantly, dietary associations with DR persisted after adjustment for diabetes status, indicating that diet may also exert direct effects on retinal microvasculature through anti-inflammatory or lipid-modulating pathways. At the same time, the apparently high AUCs of the PDI-only models for DR should be interpreted cautiously. In our study design, DR cases were necessarily drawn from participants with diabetes, whereas the control group was derived from the broader disease-free population. Accordingly, the high discriminatory performance likely reflects not only DR-related dietary variation, but also broader diet-linked differences in diabetic and cardiometabolic status between DR cases and controls. Therefore, these AUCs should not be interpreted as indicating that a single dietary index alone provides highly specific prediction of DR.

Lipid metabolism constitutes another critical link between diet and ocular disease. Elevated systemic LDL cholesterol and triglycerides can accumulate in Bruch’s membrane, whereas HDL facilitates lipid clearance^44,45^. We observed that higher PDI-H was associated with higher HDL levels, lower LDL levels, and greater intake of plant sterols that competitively inhibit cholesterol absorption. Such dietary patterns are also typically rich in omega-3 fatty acids, which have been shown to benefit retinal health. Our data suggest that higher PDI-H is associated with more favourable omega-3 profiles, potentially supporting retinal cell membrane integrity and exerting anti-inflammatory effects relevant to AMD.

Hormonal pathways emerged as an additional mechanistic theme in our enrichment analyses. Higher PDI-U was associated with pathways related to peptide hormone responses and insulin signalling. Dietary patterns influence circulating levels of insulin, IGF-1, and sex hormones^46,47^. Diets high in refined carbohydrates and specific protein sources can increase IGF-1 levels, which have been implicated in DR severity and choroidal neovascularization in AMD^48^. Hyperinsulinemia associated with insulin resistance may also impair ocular blood flow and potentially influence intraocular pressure^49^. Conversely, plant-based diets rich in soy and flaxseed may increase phytoestrogen intake, which could represent one hypothesis for hormonal modulation relevant to cataract or glaucoma^50^. Because hormone levels were not directly measured in this study, these interpretations should be regarded as biologically plausible explanations rather than firm conclusions.

These findings have important practical implications. As populations age globally, the burden of AMD, cataract, and glaucoma continues to rise^51^. Unlike age or genetic predisposition, diet represents a modifiable risk factor at both individual and population levels. Our results support dietary patterns emphasizing vegetables, fruits, whole grains, legumes, and nuts, while minimizing refined starches, sugary foods and beverages, and fried or highly processed items. Such dietary changes may simultaneously reduce the risk of eye diseases and common comorbidities, such as cardiovascular disease and diabetes, thereby enhancing feasibility and overall health benefits. In clinical practice, ophthalmologists may consider incorporating dietary counselling into patient management, particularly for individuals with early disease or elevated risk. Beyond specific supplements, such as lutein and zeaxanthin for AMD, improving overall dietary quality may offer broader preventive benefits and could be implemented even before clinical disease onset. For patients with diabetes, our findings underscore the importance of carbohydrate and fat quality, rather than glycaemic control alone, in mitigating the risk of DR.

Our multi-omics results also point to potential future therapeutic and preventive targets. For example, if CXCL10 is confirmed as a key mediator linking diet and glaucoma risk, interventions targeting this pathway may mimic some of the protective effects of healthier dietary patterns. Similarly, inhibitors of PLA2G7 (Lp-PLA2), currently under investigation for cardiovascular disease, may reduce oxidative lipid stress relevant to AMD. Proteomic and metabolomic signatures may also serve as biomarkers for risk stratification or monitoring responses to dietary interventions.

Several limitations should be acknowledged. First, dietary intake was self-reported at baseline and may be subject to measurement error and temporal change. Although we excluded participants with baseline blindness or severe visual loss, some residual heterogeneity in pre-existing ocular status may remain. In addition, because the PDI was harmonized across two cohort-specific dietary instruments, some degree of food-group misclassification or imperfect cross-cohort comparability cannot be excluded. Second, residual confounding is still possible despite multivariable adjustment and propensity score matching, as healthier diets may cluster with other favourable behaviours or healthcare-related factors. This is particularly relevant for diabetic retinopathy, where broader diabetic and cardiometabolic differences may also influence apparent discrimination. Third, the two datasets differed fundamentally in design: UKB was prospective, whereas the Tianjin dataset was hospital-based and cross-sectional. The TJ sample may therefore be affected by selection bias and should be interpreted as supportive cross-sectional evidence rather than prospective confirmation. Fourth, some outcome definitions may have introduced heterogeneity. In particular, glaucoma in the UKB was defined using both diagnostic codes and treatment/self-report information to improve sensitivity, which may reduce comparability with outcomes defined only by diagnostic coding. Statistical power was also limited for some outcomes, especially RVO. Fifth, the multi-omics mediation analyses and ocular metric analyses were exploratory. They support biological plausibility, but do not establish causal mediation or a definitive mechanistic pathway, and some molecular or imaging changes may reflect early disease rather than true intermediates. Finally, the relatively high AUCs of the PDI-only models for DR should be interpreted cautiously, as DR cases were restricted to participants with diabetes whereas controls were drawn from the broader disease-free population. Thus, the observed discrimination likely reflects not only DR-related dietary variation but also broader diet-linked differences in diabetic and cardiometabolic status. Although our study included both Western and Asian populations, generalizability to other settings still requires confirmation in interventional and longitudinal studies.

In this extensive multi-cohort study, greater adherence to a healthy plant-based diet was consistently associated with a lower risk of AMD, cataract, glaucoma, and DR. In contrast, unhealthy plant-based dietary patterns were associated with increased risks of AMD, cataract, and DR, whereas no apparent association was observed with RVO. These associations were robust in the prospective UKB analyses and were directionally supported by an independent Chinese hospital-based cross-sectional sample. Integrative multi-omics analyses provided biological support for these relationships, and predictive modelling suggested a modest incremental contribution of dietary quality to risk stratification for AMD and cataract. Together, these findings emphasize that dietary quality, rather than plant-based eating per se, is a key modifiable determinant of ocular health.

## Methods

### Study population and design

This research utilized two complementary datasets: a population-based prospective cohort (UKB) and a hospital-based cross-sectional sample (TJ). The primary analyses were conducted in the UKB cohort. The UKB recruited ~500,000 participants aged 40–69 years between 2006 and 2010^52^. For the present study, we included participants who had complete baseline dietary data and no missing information on key covariates. We excluded participants with implausibly low baseline energy intake, defined a priori as <500 kcal/day for women and <800 kcal/day for men, and those with prevalent end-stage ocular disease at baseline. End-stage ocular disease was operationally defined as documented blindness or severe visual loss, including ICD-10 H54.x diagnoses, or baseline self-reported vision loss due to eye injury or another serious eye condition. We then restricted the UKB analyses to participants with follow-up data available for incident ARED ascertainment. In parallel, we analyzed data from an independent hospital-based cross-sectional sample in Tianjin, China. This Tianjin sample enroled middle-aged and older adults during routine health examinations and specialized ophthalmology clinics. For the Tianjin sample, we included individuals who underwent comprehensive eye evaluations and baseline dietary assessments. Unlike UKB, the Tianjin data are cross-sectional with respect to eye disease status. The two cohorts thus allow examination of associations in both prospective and cross-sectional contexts.

**Fig. 8:**
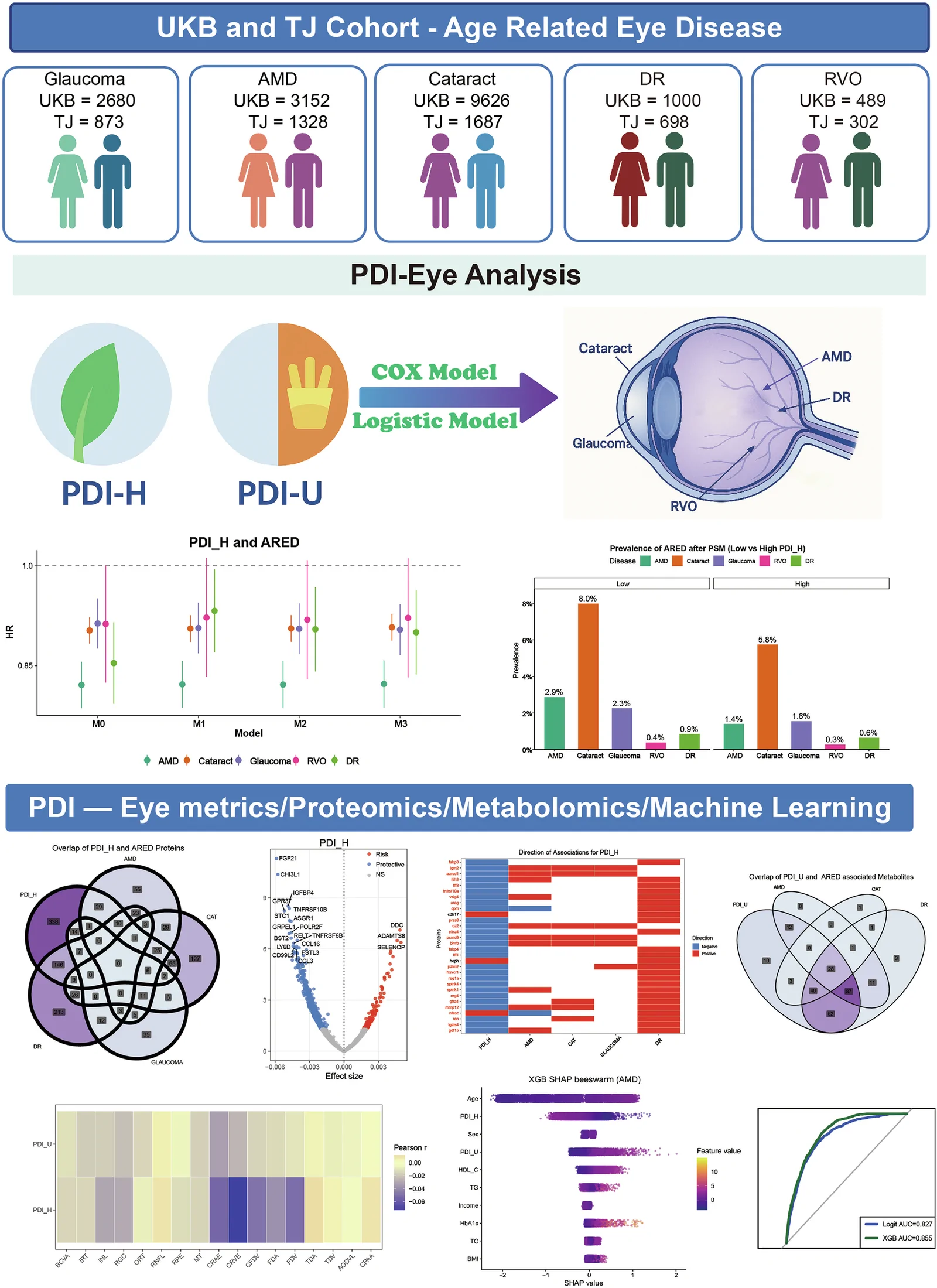
Study design and analytical framework. Overview of the study design integrating the UK Biobank (UKB) prospective cohort and the Tianjin hospital-based cross-sectional sample to investigate associations between plant-based diet quality and age-related eye diseases (AREDs). The five outcomes included AMD, cataract, glaucoma, DR, and RVO, with sample sizes shown for each cohort. PDI-H and PDI-U were derived from food-frequency questionnaires. Prospective Cox models were applied in UKB, and logistic regression models in TJ. Downstream analyses integrated ocular metrics, proteomics, metabolomics, and machine learning to explore biological mechanisms and predictive value.

Figure 8 summarizes the study design, participant flow, and analytical framework for each cohort. In the UKB, baseline characteristics were collected through questionnaires, physical examinations, and blood assays. Participants were prospectively followed for incident eye diseases via linkage to hospital inpatient records, death registries, and follow-up self-reports, with outcomes defined using standardized diagnostic or procedure codes. Follow-up time was calculated from enrolment to the first occurrence of the outcome, death, or end of follow-up. In the TJ cohort, participants underwent a single comprehensive ophthalmic examination to identify prevalent eye diseases. Owing to its cross-sectional nature, TJ data were analysed using a case–control framework with logistic models.

### Ethics approval and informed consent

All research procedures adhered to the principles of the Declaration of Helsinki and to the governance frameworks of the contributing cohorts. The UK Biobank obtained ethics approval from its institutional ethics committee, and all participants provided written informed consent. Data usage in this study complied fully with UK Biobank resource access policies. The Tianjin cohort received ethical approval from the Ethics Committee of Tianjin Eye Hospital (approval number: KY2025048), and all participants provided written informed consent before study participation.

### Dietary assessment and plant-based diet index construction

Dietary intake was assessed in both cohorts using cohort-specific dietary questionnaires, and individual food items were subsequently harmonized into predefined plant-based diet food groups. In the UKB, dietary variables were coded according to the UKB plant-based diet framework used in prior UKB analyses. In the TJ dataset, food items were grouped according to a locally adapted food-frequency questionnaire and then mapped to the same conceptual plant-based diet domains. The detailed definitions of each food group, the cohort-specific item mapping, and the UKB dietary coding scheme are provided in Supplementary Table 18.

Based on dietary data, two plant-based diet indices were constructed for each participant: the PDI-H and the PDI-U. All food items were first classified into three categories: healthy plant foods, unhealthy plant foods, and animal foods. Intake of each food group was ranked within each cohort and assigned a score. Food group intakes were categorized into quintiles and scored on a 1–5 scale. For PDI-H, higher scores were assigned to greater consumption of healthy plant foods, whereas higher consumption of unhealthy plant foods and animal foods received lower scores. For PDI-U, higher scores were assigned to greater consumption of unhealthy plant foods, while higher consumption of healthy plant foods and animal foods received lower scores^53^. Scores across all food groups were summed to derive the overall PDI-H and PDI-U values. Both indices were energy-adjusted using the density method, with food group intakes standardized per 1,000 kcal to account for dietary composition. Total energy intake was additionally included as a covariate in Model 2 to control for residual confounding related to absolute caloric consumption, because density standardization does not fully remove variation associated with total energy intake. Due to differences in dietary data availability, a total of 17 food groups were included in the UKB cohort and 15 food groups in the TJ cohort. To improve transparency and cross-cohort comparability, the operational definitions of these food groups and their cohort-specific mappings are summarized in Supplementary Table 18.

### Ascertainment of eye disease outcomes

Five ocular diseases were investigated as study outcomes: AMD, cataract, glaucoma, DR, and RVO. In the UK Biobank cohort, AMD cases were identified through hospital inpatient diagnostic records. Cataract cases were defined as individuals with an ICD diagnosis code for age-related cataract. Glaucoma cases were identified using ICD diagnostic codes and, additionally, participants who self-reported use of glaucoma medications or intraocular pressure-lowering treatment were classified as glaucoma cases to improve capture of treated disease that may be under-ascertained in inpatient coding alone. We acknowledge that this broader definition may increase sensitivity at the expense of reduced comparability with outcomes defined only by diagnostic codes. For DR, cases were defined among participants with diabetes at baseline who received an ICD diagnosis code for non-proliferative or proliferative diabetic retinopathy or diabetic macular oedema during follow-up. RVO cases were identified using ICD diagnostic codes from hospital inpatient records. For all outcomes, only incident cases occurring after baseline were included, and the time to first diagnosis was used in the analysis.

In the TJ cohort, the presence of the above ocular diseases was determined at baseline through comprehensive ophthalmic examinations. AMD was diagnosed based on dilated fundus examination, optical coherence tomography (OCT), and fundus photography. Cataract was diagnosed using slit-lamp examination. Glaucoma was diagnosed based on a combination of intraocular pressure measurements, optic nerve head assessment, and visual field testing. DR was assessed in participants with diabetes using fundoscopy or retinal photography. RVO was diagnosed by fundus examination, characterized by venous dilation, retinal haemorrhages, and retinal oedema. Experienced ophthalmologists made all diagnoses in the Tianjin cohort, and these diagnoses were subsequently reviewed by senior retinal specialists as needed.

### Statistical Analysis

PDIs and AREDs first described baseline characteristics of participants. Differences between groups were assessed using t-tests for continuous variables and chi-square tests for categorical variables. Trends in baseline characteristics across quintiles of dietary indices were further examined using linear regression for continuous variables and the Mantel–Haenszel chi-square test for trend for categorical variables.

The primary analyses evaluated the associations between dietary indices (PDI-H and PDI-U) and each ocular disease outcome. In the prospective UK Biobank cohort, Cox proportional hazards regression models were used to estimate hazard ratios (HRs) and 95% confidence intervals (CIs) for incident ocular diseases. In the Tianjin cross-sectional cohort, logistic regression models were applied to estimate odds ratios (ORs) and 95% CIs for disease prevalence. PDI-H and PDI-U were primarily analyzed as continuous variables to maximize statistical power and facilitate comparison of effect estimates across cohorts. In addition, dietary indices were categorized into quintiles, with the lowest quintile used as the reference group, to assess potential non-linear associations or threshold effects.

A series of progressively adjusted models was constructed. Model 0 (M0) was minimally adjusted for age, sex, and ethnicity (UKB only). Model 1 (M1) additionally adjusted for socioeconomic and lifestyle factors, including educational attainment, household income, smoking status (never, former, current), and alcohol consumption (none, moderate, heavy). Model 2 (M2) further included body mass index (BMI) and total energy intake. Model 3 (M3), the fully adjusted model, additionally incorporated medical history and clinical factors, including hypertension (yes/no) and diabetes mellitus (yes/no). For Cox models, the proportional hazards assumption was evaluated using Schoenfeld residuals and log–log survival plots, with no substantial violations observed.

Restricted cubic spline (RCS) functions were used to model potential dose–response relationships between dietary indices and ocular disease risk, providing a flexible framework for nonlinear associations. In the UKB cohort, four knots were specified for PDI-H and PDI-U in Cox models, typically placed at selected percentiles. In the TJ cohort, analogous spline functions were incorporated into logistic regression models. All spline analyses were conducted using the fully adjusted model (M3).

To assess the consistency of associations across population subgroups, stratified analyses and interaction tests were performed. UKB participants were stratified by age (<60 vs ≥60 years), sex, smoking status (never vs ever), alcohol consumption (no vs any), and hypertension status (no vs yes). Within each subgroup, associations between dietary indices and ocular outcomes were estimated separately using HRs or ORs as appropriate. Due to the smaller sample size, subgroup analyses in the Tianjin cohort were restricted to age- and sex-stratified analyses for AMD and cataract.

Given the potential for residual confounding in observational studies, propensity score matching (PSM) was conducted as a sensitivity analysis to further control baseline differences. Propensity scores were estimated using logistic regression models including age, sex, BMI, smoking status, alcohol consumption, education, income, and comorbidities as covariates. For the PSM analyses, low- and high-dietary-index groups were defined as participants in the lowest and highest quintiles of PDI-H or PDI-U, respectively. These participants were then matched 1:1 using nearest-neighbour matching with a caliper width of 0.2 standard deviations of the logit of the propensity score. After matching, differences in ocular disease prevalence between dietary groups were compared using McNemar’s test and conditional logistic regression. In addition, for the UKB cohort, participants in the highest and lowest quintiles of the dietary indices were compared using cumulative incidence curves to illustrate differences in disease incidence over follow-up visually.

Proteomic analyses were conducted in the UKB to identify circulating proteins associated with dietary indices and ocular disease outcomes. For each protein, linear regression models were used to evaluate associations with PDI-H and PDI-U, adjusting for age, sex, and technical covariates. Proteins significantly associated with dietary indices were identified using a false discovery rate (FDR) threshold of <0.05. Case–control comparisons were then performed for each ocular disease to identify disease-associated proteins, and overlapping diet–disease proteins were summarized using overlap analyses. To move beyond overlap-based interpretation, we further performed exploratory regression-based mediation analyses for representative molecules, focusing on the top 30 diet-associated proteins for PDI-H and PDI-U. For each candidate protein, we estimated the direction and magnitude of the indirect effect of the dietary index on each ocular outcome through the protein mediator, and summarized these signed mediation effects in bubble plots (Fig. 4C, E). These mediation analyses were intended to prioritize plausible intermediates and should be interpreted as hypothesis-generating rather than causal proof. Gene Ontology (GO) enrichment analyses were then performed on overlapping protein sets, focusing on biological process terms.

Metabolomic analyses were also performed in the UKB using nuclear magnetic resonance (NMR) spectroscopy–derived biomarkers, including lipid fractions, fatty acids, amino acids, and glycaemic markers. Associations between dietary indices and circulating metabolites were examined using linear regression models adjusted for age and sex. In addition to overlap analyses, we conducted exploratory regression-based mediation analyses for representative metabolites, focusing on the top 20 diet-associated metabolites for PDI-H and PDI-U. For each candidate metabolite, we estimated the signed indirect effect of the dietary index on each ocular outcome through the metabolite mediator and summarized these mediation effects in bubble plots (Fig. 5A, C). These analyses were performed to evaluate whether selected metabolites might plausibly intermediate diet–disease associations, but they were interpreted conservatively as exploratory rather than causal.

Exploratory mediation analyses were conducted using a regression-based framework. For each candidate protein or metabolite, we fitted two models: first, a mediator model estimating the association between the dietary index and the candidate mediator; and second, an outcome model estimating the association between the dietary index, the mediator, and the ocular outcome. The indirect effect was calculated as the product of the exposure–mediator and mediator–outcome coefficients. Covariate adjustment was aligned with the fully adjusted model used in the main epidemiological analyses, including age, sex, ethnicity where applicable, educational attainment, household income, smoking status, alcohol consumption, body mass index, total energy intake, hypertension, and diabetes status. For proteomic analyses, additional technical covariates related to assay processing were included where available. Confidence intervals and P values for indirect effects were estimated using non-parametric bootstrap resampling with 1000 iterations. Given the exploratory nature of these analyses, nominal P values are shown in the bubble plots, while Benjamini–Hochberg FDR-adjusted q values are reported in Supplementary Table 19. These mediation analyses were intended to prioritize plausible biological intermediates and should not be interpreted as evidence of causal mediation.

Association analyses of ocular parameters were conducted in a subset of UKB participants who underwent ocular imaging, including OCT-derived retinal thickness, macular volume, and retinal vascular measurements. Associations between PDI-H/PDI-U and ocular parameters were re-estimated using multivariable-adjusted models, with covariate adjustment based on the fully adjusted framework of the main analyses where applicable. Associations between ocular parameters and disease status were summarized separately to evaluate whether these imaging phenotypes might represent structural or vascular correlates of the main diet–ARED associations. Nominal P values were adjusted for multiple testing using the Benjamini–Hochberg FDR procedure separately for the PDI–ocular metric analyses and the disease–ocular metric analyses. Figure 6 displays FDR-adjusted significance markers, and full standardized coefficients, standard errors, 95% confidence intervals, nominal P values, and FDR-adjusted q values are reported in Supplementary Table 20. These analyses were exploratory and intended to provide supportive phenotypic context rather than formal mediation evidence.

To evaluate the predictive value of dietary indices and integrate multiple risk factors, machine learning models were developed for two outcomes with sufficient case numbers: AMD and cataract. The UKB dataset was used as the training set, including predictors such as age, sex, smoking status, alcohol consumption, BMI, diabetes status, hypertension status, selected laboratory measurements, and dietary indices (PDI-H and PDI-U). Multivariable logistic regression models were first trained and evaluated using 10-fold cross-validation. Gradient-boosting decision tree models (XGBoost) were subsequently trained to capture nonlinear relationships and interactions. Model performance was externally validated in the Tianjin cohort. To quantify the incremental predictive contribution of dietary indices, we additionally compared otherwise identical models fitted with and without PDI-H/PDI-U. Discriminative performance was assessed using the area under the receiver operating characteristic curve (AUC), accuracy, sensitivity, specificity, precision, and F1 score in the validation and external validation datasets (Supplementary Fig. 11). Feature importance in XGBoost models was evaluated based on gain values.

All statistical analyses were performed using R version 4.0 and SAS version 9.4. Two-sided p values < 0.05 were considered statistically significant. No adjustment for multiple comparisons was made in the primary hypothesis-driven analyses of dietary indices and ocular disease outcomes. For high-dimensional proteomic and metabolomic analyses, multiple-testing correction was performed using an FDR approach. The study was reported in accordance with the STROBE guidelines for observational studies.

## Supplementary information

Supplementary information

## Data Availability

Data used in this study are available upon reasonable request and subject to institutional and ethical restrictions. The primary dataset was derived from the UK Biobank, which is not publicly accessible due to data governance policies. Data from the Tianjin cohort is not publicly available due to local institutional and ethical restrictions involving sensitive clinical and ophthalmic information; access to de-identified data may be granted upon reasonable request to the corresponding author, with approval from the relevant institutional review boards.

## Code availability

Analytical code supporting the findings of this study may be available from the corresponding author on reasonable request.
