# Supplementary material for: Plant-based diet quality and risk of age-related eye diseases: evidence from multi-cohorts with multi-omics insights
Source: NPJ Sci Food. 2026 Jun 4;10:268. doi: 10.1038/s41538-026-00919-z (PMC13547330; doi:10.1038/s41538-026-00919-z)
Supplement: Supplementary file 1 [file 41538_2026_919_MOESM1_ESM.pdf]

Supplementary Information

Supplementary Tables 1-21 and Supplementary Figures 1-11

# Plant-Based Diet Quality and Risk of Age-Related Eye Diseases: Evidence from Multi-Cohorts with Multi-Omics Insights

## Supplementary Table 1. Baseline characteristics of participants stratified by five major ocular diseases in the UK Biobank and Tianjin cohorts

| Cohort | Disease | Status | n | PDI_H_mean | PDI_H_sd | PDI_U_mean | PDI_U_sd | Age_mean | Age_sd | BMI_mean |
|---|---|---|---|---|---|---|---|---|---|---|
| UKB | AMD | 0 | 125849 | 33.88 | 9.51 | 35.19 | 8.71 | 56.91 | 7.77 | 27.2 |
| UKB | AMD | 1 | 3152 | 32.86 | 8.9 | 36.17 | 8.84 | 62.46 | 5.38 | 27.28 |
| UKB | Cataract | 0 | 119375 | 33.89 | 9.52 | 35.16 | 8.71 | 56.65 | 7.78 | 27.19 |
| UKB | Cataract | 1 | 9626 | 33.51 | 9.21 | 35.82 | 8.81 | 61.96 | 5.7 | 27.43 |
| UKB | Glaucoma | 0 | 126321 | 33.86 | 9.5 | 35.2 | 8.72 | 56.94 | 7.77 | 27.21 |
| UKB | Glaucoma | 1 | 2680 | 33.56 | 9.17 | 35.55 | 8.7 | 61.66 | 5.88 | 26.99 |
| UKB | DR | 0 | 128001 | 33.87 | 9.5 | 35.21 | 8.71 | 57.02 | 7.77 | 27.18 |
| UKB | DR | 1 | 1000 | 32.43 | 9.27 | 35.8 | 9.92 | 60.13 | 6.72 | 30.86 |
| UKB | RVO | 0 | 128512 | 33.86 | 9.5 | 35.21 | 8.72 | 57.02 | 7.77 | 27.2 |
| UKB | RVO | 1 | 489 | 33.46 | 9.26 | 35.19 | 8.75 | 61.63 | 6.08 | 28.08 |
| TJ | AMD | 0 | 4649 | 34.96 | 9.59 | 35.46 | 8.63 | 56.89 | 7.57 | 24.51 |
| TJ | AMD | 1 | 1328 | 33.82 | 9.64 | 35.79 | 8.85 | 56.85 | 7.6 | 24.59 |
| TJ | Cataract | 0 | 4290 | 34.99 | 9.48 | 35.34 | 8.63 | 57.05 | 7.62 | 24.53 |
| TJ | Cataract | 1 | 1687 | 33.98 | 9.9 | 36.04 | 8.78 | 56.46 | 7.43 | 24.51 |
| TJ | Glaucoma | 0 | 5104 | 34.84 | 9.7 | 35.61 | 8.68 | 56.9 | 7.62 | 24.53 |
| TJ | Glaucoma | 1 | 873 | 33.89 | 9.05 | 35.07 | 8.67 | 56.73 | 7.27 | 24.53 |
| TJ | DR | 0 | 5279 | 34.78 | 9.64 | 35.44 | 8.69 | 56.94 | 7.58 | 24.55 |
| TJ | DR | 1 | 698 | 34.1 | 9.37 | 36.24 | 8.53 | 56.42 | 7.5 | 24.32 |
| TJ | RVO | 0 | 5675 | 34.72 | 9.61 | 35.53 | 8.67 | 56.88 | 7.55 | 24.53 |
| TJ | RVO | 1 | 302 | 34.47 | 9.7 | 35.69 | 8.87 | 56.86 | 7.95 | 24.42 |

**Supplementary Table 1. Baseline characteristics of participants stratified by five major ocular diseases in the UK Biobank and Tianjin cohorts (continued, column block 2 of 5)**

| Cohort | Disease | Status | BMI_sd | HbA1c_mean | HbA1c_sd | TC_mean | TC_sd | LDL_C_mean | LDL_C_sd | HDL_C_mean |
|---|---|---|---|---|---|---|---|---|---|---|
| UKB | AMD | 0 | 4.69 | 35.83 | 5.98 | 4.7 | 0.95 | 1.78 | 0.44 | 1.33 |
| UKB | AMD | 1 | 4.7 | 37.1 | 7.01 | 4.72 | 1.01 | 1.77 | 0.46 | 1.37 |
| UKB | Cataract | 0 | 4.69 | 35.74 | 5.87 | 4.71 | 0.94 | 1.78 | 0.44 | 1.33 |
| UKB | Cataract | 1 | 4.74 | 37.38 | 7.41 | 4.7 | 1.02 | 1.77 | 0.46 | 1.34 |
| UKB | Glaucoma | 0 | 4.69 | 35.84 | 5.99 | 4.71 | 0.95 | 1.78 | 0.44 | 1.33 |
| UKB | Glaucoma | 1 | 4.51 | 36.79 | 6.86 | 4.67 | 0.98 | 1.76 | 0.45 | 1.34 |
| UKB | DR | 0 | 4.67 | 35.71 | 5.61 | 4.71 | 0.94 | 1.79 | 0.44 | 1.34 |
| UKB | DR | 1 | 5.74 | 55.42 | 15.87 | 3.75 | 0.93 | 1.39 | 0.43 | 1.13 |
| UKB | RVO | 0 | 4.69 | 35.86 | 6.01 | 4.71 | 0.95 | 1.78 | 0.44 | 1.33 |
| UKB | RVO | 1 | 4.97 | 37.56 | 7.29 | 4.5 | 0.98 | 1.7 | 0.44 | 1.3 |
| TJ | AMD | 0 | 3.47 | 35.18 | 4.09 | 3.84 | 0.51 | 1.85 | 0.42 | 1.37 |
| TJ | AMD | 1 | 3.35 | 34.71 | 3.67 | 4.09 | 0.51 | 2.07 | 0.41 | 1.26 |
| TJ | Cataract | 0 | 3.51 | 35.05 | 3.96 | 3.89 | 0.52 | 1.89 | 0.42 | 1.35 |
| TJ | Cataract | 1 | 3.28 | 35.15 | 4.11 | 3.91 | 0.53 | 1.93 | 0.43 | 1.34 |
| TJ | Glaucoma | 0 | 3.44 | 35.12 | 4.01 | 3.9 | 0.52 | 1.9 | 0.42 | 1.35 |
| TJ | Glaucoma | 1 | 3.5 | 34.83 | 3.93 | 3.89 | 0.52 | 1.89 | 0.43 | 1.28 |
| TJ | DR | 0 | 3.46 | 34.16 | 3.14 | 3.85 | 0.51 | 1.85 | 0.41 | 1.36 |
| TJ | DR | 1 | 3.34 | 42 | 2.94 | 4.25 | 0.5 | 2.23 | 0.41 | 1.23 |
| TJ | RVO | 0 | 3.45 | 35.1 | 4.02 | 3.9 | 0.52 | 1.9 | 0.42 | 1.35 |
| TJ | RVO | 1 | 3.37 | 34.74 | 3.56 | 3.86 | 0.54 | 1.86 | 0.44 | 1.26 |

**Supplementary Table 1. Baseline characteristics of participants stratified by five major ocular diseases in the UK Biobank and Tianjin cohorts (continued, column block 3 of 5)**

| Cohort | Disease | Status | HDL_C_sd | TG_mean | TG_sd | CRP_mean | CRP_sd | Sex_0 | Sex_1 | Smoke_0 |
|---|---|---|---|---|---|---|---|---|---|---|
| UKB | AMD | 0 | 0.33 | 1.35 | 0.59 | 2.45 | 4.07 | 59094 | 66755 | 69198 |
| UKB | AMD | 1 | 0.35 | 1.34 | 0.59 | 2.65 | 4.45 | 1307 | 1845 | 1594 |
| UKB | Cataract | 0 | 0.33 | 1.35 | 0.59 | 2.43 | 4.07 | 56184 | 63191 | 65867 |
| UKB | Cataract | 1 | 0.34 | 1.37 | 0.58 | 2.7 | 4.27 | 4217 | 5409 | 4925 |
| UKB | Glaucoma | 0 | 0.33 | 1.35 | 0.59 | 2.45 | 4.07 | 58981 | 67340 | 69392 |
| UKB | Glaucoma | 1 | 0.33 | 1.36 | 0.59 | 2.6 | 4.58 | 1420 | 1260 | 1400 |
| UKB | DR | 0 | 0.33 | 1.35 | 0.59 | 2.45 | 4.07 | 59748 | 68253 | 70364 |
| UKB | DR | 1 | 0.3 | 1.51 | 0.71 | 3.29 | 5.1 | 653 | 347 | 428 |
| UKB | RVO | 0 | 0.33 | 1.35 | 0.59 | 2.45 | 4.08 | 60126 | 68386 | 70546 |
| UKB | RVO | 1 | 0.31 | 1.38 | 0.59 | 2.74 | 4.11 | 275 | 214 | 246 |
| TJ | AMD | 0 | 0.25 | 1.54 | 0.8 | 2.9 | 2.14 | 2199 | 2450 | 2563 |
| TJ | AMD | 1 | 0.26 | 1.51 | 0.79 | 2.98 | 2.16 | 613 | 715 | 725 |
| TJ | Cataract | 0 | 0.26 | 1.52 | 0.79 | 2.89 | 2.13 | 2006 | 2284 | 2366 |
| TJ | Cataract | 1 | 0.26 | 1.55 | 0.81 | 2.98 | 2.17 | 806 | 881 | 922 |
| TJ | Glaucoma | 0 | 0.26 | 1.54 | 0.8 | 2.91 | 2.14 | 2392 | 2712 | 2788 |
| TJ | Glaucoma | 1 | 0.25 | 1.48 | 0.77 | 2.93 | 2.15 | 420 | 453 | 500 |
| TJ | DR | 0 | 0.26 | 1.53 | 0.8 | 2.92 | 2.14 | 2486 | 2793 | 2894 |
| TJ | DR | 1 | 0.26 | 1.55 | 0.77 | 2.86 | 2.14 | 326 | 372 | 394 |
| TJ | RVO | 0 | 0.26 | 1.53 | 0.8 | 2.92 | 2.15 | 2664 | 3011 | 3133 |
| TJ | RVO | 1 | 0.23 | 1.55 | 0.78 | 2.77 | 2.01 | 148 | 154 | 155 |

**Supplementary Table 1. Baseline characteristics of participants stratified by five major ocular diseases in the UK Biobank and Tianjin cohorts (continued, column block 4 of 5)**

| Cohort | Disease | Status | Smoke_1 | Smoke_2 | Alcohol_0 | Alcohol_1 | Alcohol_2 | HBP_0 | HBP_1 | DM_0 |
|---|---|---|---|---|---|---|---|---|---|---|
| UKB | AMD | 0 | 46391 | 10260 | 4196 | 4267 | 117386 | 71567 | 54282 | 122007 |
| UKB | AMD | 1 | 1350 | 208 | 152 | 115 | 2885 | 1576 | 1576 | 3004 |
| UKB | Cataract | 0 | 43758 | 9750 | 3927 | 4007 | 111441 | 68200 | 51175 | 115893 |
| UKB | Cataract | 1 | 3983 | 718 | 421 | 375 | 8830 | 4943 | 4683 | 9118 |
| UKB | Glaucoma | 0 | 46638 | 10291 | 4244 | 4282 | 117795 | 71905 | 54416 | 122452 |
| UKB | Glaucoma | 1 | 1103 | 177 | 104 | 100 | 2476 | 1238 | 1442 | 2559 |
| UKB | DR | 0 | 47269 | 10368 | 4282 | 4314 | 119405 | 72691 | 55310 | 124516 |
| UKB | DR | 1 | 472 | 100 | 66 | 68 | 866 | 452 | 548 | 495 |
| UKB | RVO | 0 | 47535 | 10431 | 4324 | 4364 | 119824 | 72957 | 55555 | 124546 |
| UKB | RVO | 1 | 206 | 37 | 24 | 18 | 447 | 186 | 303 | 465 |
| TJ | AMD | 0 | 1655 | 431 | 133 | 175 | 4341 | 2988 | 1661 | 3874 |
| TJ | AMD | 1 | 488 | 115 | 39 | 52 | 1237 | 550 | 778 | 1170 |
| TJ | Cataract | 0 | 1536 | 388 | 124 | 148 | 4018 | 2594 | 1696 | 3651 |
| TJ | Cataract | 1 | 607 | 158 | 48 | 79 | 1560 | 944 | 743 | 1393 |
| TJ | Glaucoma | 0 | 1850 | 466 | 145 | 193 | 4766 | 3198 | 1906 | 4295 |
| TJ | Glaucoma | 1 | 293 | 80 | 27 | 34 | 812 | 340 | 533 | 749 |
| TJ | DR | 0 | 1900 | 485 | 156 | 187 | 4936 | 3099 | 2180 | 5035 |
| TJ | DR | 1 | 243 | 61 | 16 | 40 | 642 | 439 | 259 | 9 |
| TJ | RVO | 0 | 2030 | 512 | 164 | 212 | 5299 | 3406 | 2269 | 4777 |
| TJ | RVO | 1 | 113 | 34 | 8 | 15 | 279 | 132 | 170 | 267 |

## Supplementary Table 1. Baseline characteristics of participants stratified by five major ocular diseases in the UK Biobank and Tianjin cohorts (continued, column block 5 of 5)

| Cohort | Disease | Status | DM_1 |
|---|---|---|---|
| UKB | AMD | 0 | 3842 |
| UKB | AMD | 1 | 148 |
| UKB | Cataract | 0 | 3482 |
| UKB | Cataract | 1 | 508 |
| UKB | Glaucoma | 0 | 3869 |
| UKB | Glaucoma | 1 | 121 |
| UKB | DR | 0 | 3485 |
| UKB | DR | 1 | 505 |
| UKB | RVO | 0 | 3966 |
| UKB | RVO | 1 | 24 |
| TJ | AMD | 0 | 775 |
| TJ | AMD | 1 | 158 |
| TJ | Cataract | 0 | 639 |
| TJ | Cataract | 1 | 294 |
| TJ | Glaucoma | 0 | 809 |
| TJ | Glaucoma | 1 | 124 |
| TJ | DR | 0 | 244 |
| TJ | DR | 1 | 689 |
| TJ | RVO | 0 | 898 |
| TJ | RVO | 1 | 35 |

## Supplementary Table 2. Participant baseline characteristics by quintiles of PDI-H and PDI-U in the UKB and TJ cohorts

| Cohort | Quintile | n | Age_mean | Age_sd | BMI_mean | BMI_sd | HbA1c_mean | HbA1c_sd | TC_mean | TC_sd |
|---|---|---|---|---|---|---|---|---|---|---|
| TJ_PDI-U | 1 | 1284 | 56.99 | 7.45 | 24.49 | 3.47 | 35.03 | 3.88 | 3.89 | 0.52 |
| TJ_PDI-U | 2 | 1192 | 56.93 | 7.6 | 24.68 | 3.36 | 35.01 | 4.06 | 3.87 | 0.51 |
| TJ_PDI-U | 3 | 1339 | 56.67 | 7.53 | 24.38 | 3.49 | 35.08 | 4.23 | 3.9 | 0.53 |
| TJ_PDI-U | 4 | 1083 | 56.65 | 7.57 | 24.55 | 3.49 | 35.11 | 3.89 | 3.91 | 0.53 |
| TJ_PDI-U | 5 | 1079 | 57.18 | 7.74 | 24.57 | 3.4 | 35.18 | 3.92 | 3.9 | 0.51 |
| TJ_PDI-H | 1 | 1201 | 57.17 | 7.51 | 24.34 | 3.41 | 35.21 | 4.08 | 3.92 | 0.54 |
| TJ_PDI-H | 2 | 1219 | 56.99 | 7.57 | 24.52 | 3.47 | 35.36 | 4.17 | 3.89 | 0.54 |
| TJ_PDI-H | 3 | 1236 | 56.56 | 7.69 | 24.49 | 3.47 | 34.94 | 3.96 | 3.9 | 0.5 |
| TJ_PDI-H | 4 | 1245 | 56.69 | 7.4 | 24.55 | 3.5 | 34.98 | 3.92 | 3.88 | 0.52 |
| TJ_PDI-H | 5 | 1076 | 57 | 7.7 | 24.77 | 3.34 | 34.89 | 3.84 | 3.89 | 0.51 |
| UKB_PDI-U | 1 | 28607 | 56.19 | 7.87 | 27.57 | 4.94 | 35.93 | 6.22 | 4.66 | 0.95 |
| UKB_PDI-U | 2 | 25620 | 57.1 | 7.74 | 27.32 | 4.77 | 35.97 | 6.09 | 4.69 | 0.95 |
| UKB_PDI-U | 3 | 23306 | 57.37 | 7.73 | 27.17 | 4.63 | 35.96 | 6.11 | 4.71 | 0.94 |
| UKB_PDI-U | 4 | 29388 | 57.41 | 7.68 | 27.05 | 4.55 | 35.8 | 5.93 | 4.73 | 0.95 |
| UKB_PDI-U | 5 | 22080 | 57.25 | 7.75 | 26.86 | 4.46 | 35.64 | 5.64 | 4.74 | 0.95 |
| UKB_PDI-H | 1 | 29228 | 55.72 | 8.12 | 27.74 | 4.93 | 35.9 | 6.21 | 4.67 | 0.95 |
| UKB_PDI-H | 2 | 22846 | 56.82 | 7.9 | 27.45 | 4.74 | 35.94 | 6 | 4.69 | 0.95 |
| UKB_PDI-H | 3 | 25621 | 57.12 | 7.72 | 27.23 | 4.71 | 35.93 | 6.26 | 4.69 | 0.95 |
| UKB_PDI-H | 4 | 27214 | 57.63 | 7.53 | 26.94 | 4.54 | 35.86 | 5.87 | 4.72 | 0.95 |
| UKB_PDI-H | 5 | 24092 | 58.12 | 7.27 | 26.6 | 4.4 | 35.68 | 5.66 | 4.76 | 0.94 |

**Supplementary Table 2. Participant baseline characteristics by quintiles of PDI-H and PDI-U in the UKB and TJ cohorts (continued, column block 2 of 4)**

| Cohort | Quintile | n | LDL_C_mean | LDL_C_sd | HDL_C_mean | HDL_C_sd | TG_mean | TG_sd | CRP_mean | CRP_sd |
|---|---|---|---|---|---|---|---|---|---|---|
| TJ_PDI-U | 1 | 1284 | 1.9 | 0.43 | 1.34 | 0.26 | 1.51 | 0.8 | 2.9 | 2.13 |
| TJ_PDI-U | 2 | 1192 | 1.88 | 0.41 | 1.36 | 0.25 | 1.54 | 0.79 | 2.92 | 2.18 |
| TJ_PDI-U | 3 | 1339 | 1.9 | 0.43 | 1.35 | 0.26 | 1.55 | 0.8 | 2.98 | 2.14 |
| TJ_PDI-U | 4 | 1083 | 1.89 | 0.43 | 1.34 | 0.26 | 1.54 | 0.81 | 2.91 | 2.15 |
| TJ_PDI-U | 5 | 1079 | 1.91 | 0.42 | 1.34 | 0.26 | 1.52 | 0.8 | 2.87 | 2.12 |
| TJ_PDI-H | 1 | 1201 | 1.93 | 0.43 | 1.34 | 0.26 | 1.5 | 0.78 | 2.85 | 2.17 |
| TJ_PDI-H | 2 | 1219 | 1.9 | 0.43 | 1.34 | 0.26 | 1.55 | 0.8 | 2.99 | 2.13 |
| TJ_PDI-H | 3 | 1236 | 1.9 | 0.42 | 1.34 | 0.26 | 1.49 | 0.76 | 2.97 | 2.23 |
| TJ_PDI-H | 4 | 1245 | 1.88 | 0.42 | 1.35 | 0.26 | 1.57 | 0.85 | 2.92 | 2.07 |
| TJ_PDI-H | 5 | 1076 | 1.88 | 0.41 | 1.35 | 0.26 | 1.55 | 0.8 | 2.85 | 2.11 |
| UKB_PDI-U | 1 | 28607 | 1.77 | 0.45 | 1.32 | 0.33 | 1.33 | 0.59 | 2.54 | 4.26 |
| UKB_PDI-U | 2 | 25620 | 1.78 | 0.45 | 1.33 | 0.32 | 1.35 | 0.59 | 2.43 | 3.99 |
| UKB_PDI-U | 3 | 23306 | 1.78 | 0.44 | 1.34 | 0.33 | 1.36 | 0.59 | 2.46 | 4.11 |
| UKB_PDI-U | 4 | 29388 | 1.79 | 0.44 | 1.34 | 0.33 | 1.36 | 0.6 | 2.43 | 4.03 |
| UKB_PDI-U | 5 | 22080 | 1.8 | 0.44 | 1.35 | 0.33 | 1.36 | 0.59 | 2.39 | 3.98 |
| UKB_PDI-H | 1 | 29228 | 1.78 | 0.45 | 1.32 | 0.32 | 1.36 | 0.6 | 2.67 | 4.41 |
| UKB_PDI-H | 2 | 22846 | 1.78 | 0.45 | 1.32 | 0.32 | 1.37 | 0.6 | 2.52 | 4.11 |
| UKB_PDI-H | 3 | 25621 | 1.78 | 0.44 | 1.33 | 0.33 | 1.36 | 0.59 | 2.45 | 4 |
| UKB_PDI-H | 4 | 27214 | 1.79 | 0.44 | 1.34 | 0.33 | 1.35 | 0.58 | 2.37 | 4.03 |
| UKB_PDI-H | 5 | 24092 | 1.79 | 0.44 | 1.36 | 0.33 | 1.33 | 0.58 | 2.22 | 3.76 |

## Supplementary Table 2. Participant baseline characteristics by quintiles of PDI-H and PDI-U in the UKB and TJ cohorts (continued, column block 3 of 4)

| Cohort | Quintile | n | Sex_0 | Sex_1 | Smoke_0 | Smoke_1 | Smoke_2 | Alcohol_0 | Alcohol_1 | Alcohol_2 |
|---|---|---|---|---|---|---|---|---|---|---|
| TJ_PDI-U | 1 | 1284 | 587 | 697 | 685 | 490 | 109 | 38 | 51 | 1195 |
| TJ_PDI-U | 2 | 1192 | 543 | 649 | 663 | 410 | 119 | 35 | 51 | 1106 |
| TJ_PDI-U | 3 | 1339 | 669 | 670 | 771 | 457 | 111 | 44 | 42 | 1253 |
| TJ_PDI-U | 4 | 1083 | 516 | 567 | 578 | 410 | 95 | 26 | 43 | 1014 |
| TJ_PDI-U | 5 | 1079 | 497 | 582 | 591 | 376 | 112 | 29 | 40 | 1010 |
| TJ_PDI-H | 1 | 1201 | 540 | 661 | 627 | 449 | 125 | 40 | 44 | 1117 |
| TJ_PDI-H | 2 | 1219 | 556 | 663 | 696 | 408 | 115 | 42 | 50 | 1127 |
| TJ_PDI-H | 3 | 1236 | 585 | 651 | 648 | 473 | 115 | 33 | 45 | 1158 |
| TJ_PDI-H | 4 | 1245 | 595 | 650 | 699 | 435 | 111 | 29 | 48 | 1168 |
| TJ_PDI-H | 5 | 1076 | 536 | 540 | 618 | 378 | 80 | 28 | 40 | 1008 |
| UKB_PDI-U | 1 | 28607 | 13305 | 15302 | 14689 | 10939 | 2979 | 1144 | 1374 | 26089 |
| UKB_PDI-U | 2 | 25620 | 12112 | 13508 | 13735 | 9735 | 2150 | 864 | 914 | 23842 |
| UKB_PDI-U | 3 | 23306 | 10973 | 12333 | 12839 | 8744 | 1723 | 723 | 713 | 21870 |
| UKB_PDI-U | 4 | 29388 | 13744 | 15644 | 16685 | 10594 | 2109 | 934 | 788 | 27666 |
| UKB_PDI-U | 5 | 22080 | 10267 | 11813 | 12844 | 7729 | 1507 | 683 | 593 | 20804 |
| UKB_PDI-H | 1 | 29228 | 13622 | 15606 | 15404 | 10585 | 3239 | 1278 | 1287 | 26663 |
| UKB_PDI-H | 2 | 22846 | 10966 | 11880 | 12244 | 8506 | 2096 | 850 | 813 | 21183 |
| UKB_PDI-H | 3 | 25621 | 12243 | 13378 | 13989 | 9695 | 1937 | 822 | 873 | 23926 |
| UKB_PDI-H | 4 | 27214 | 12718 | 14496 | 15237 | 10155 | 1822 | 781 | 792 | 25641 |
| UKB_PDI-H | 5 | 24092 | 10852 | 13240 | 13918 | 8800 | 1374 | 617 | 617 | 22858 |

## Supplementary Table 2. Participant baseline characteristics by quintiles of PDI-H and PDI-U in the UKB and TJ cohorts (continued, column block 4 of 4)

| Cohort | Quintile | n | HBP_0 | HBP_1 | DM_0 | DM_1 |
|---|---|---|---|---|---|---|
| TJ_PDI-U | 1 | 1284 | 734 | 550 | 1100 | 184 |
| TJ_PDI-U | 2 | 1192 | 741 | 451 | 1009 | 183 |
| TJ_PDI-U | 3 | 1339 | 802 | 537 | 1117 | 222 |
| TJ_PDI-U | 4 | 1083 | 657 | 426 | 910 | 173 |
| TJ_PDI-U | 5 | 1079 | 604 | 475 | 908 | 171 |
| TJ_PDI-H | 1 | 1201 | 698 | 503 | 997 | 204 |
| TJ_PDI-H | 2 | 1219 | 728 | 491 | 1005 | 214 |
| TJ_PDI-H | 3 | 1236 | 718 | 518 | 1057 | 179 |
| TJ_PDI-H | 4 | 1245 | 737 | 508 | 1067 | 178 |
| TJ_PDI-H | 5 | 1076 | 657 | 419 | 918 | 158 |
| UKB_PDI-U | 1 | 28607 | 16657 | 11950 | 27667 | 940 |
| UKB_PDI-U | 2 | 25620 | 14570 | 11050 | 24784 | 836 |
| UKB_PDI-U | 3 | 23306 | 13015 | 10291 | 22568 | 738 |
| UKB_PDI-U | 4 | 29388 | 16399 | 12989 | 28526 | 862 |
| UKB_PDI-U | 5 | 22080 | 12502 | 9578 | 21466 | 614 |
| UKB_PDI-H | 1 | 29228 | 16946 | 12282 | 28286 | 942 |
| UKB_PDI-H | 2 | 22846 | 13035 | 9811 | 22144 | 702 |
| UKB_PDI-H | 3 | 25621 | 14497 | 11124 | 24739 | 882 |
| UKB_PDI-H | 4 | 27214 | 15256 | 11958 | 26381 | 833 |
| UKB_PDI-H | 5 | 24092 | 13409 | 10683 | 23461 | 631 |

## Supplementary Table 3. Associations between plant-based diet indexes and ocular disease risk across two population-based cohorts

| Model | OR | LCL | UCL | pvalue | Disease | Exposure | Cohort |
|---|---|---|---|---|---|---|---|
| M0 | 0.8860705572 | 0.8314207505 | 0.94431253 | 0.000196005775 | AMD | PDI_H | TJ Cohort |
| M1 | 0.8823854924 | 0.8278104686 | 0.9405584814 | 0.0001223811548 | AMD | PDI_H | TJ Cohort |
| M2 | 0.8805971033 | 0.8248479952 | 0.9401141336 | 0.0001385734976 | AMD | PDI_H | TJ Cohort |
| M3 | 0.8969627537 | 0.8373887503 | 0.9607750061 | 0.001927435071 | AMD | PDI_H | TJ Cohort |
| M0 | 0.9113097553 | 0.8592332026 | 0.9665425727 | 0.00197802548 | Cataract | PDI_H | TJ Cohort |
| M1 | 0.9098025364 | 0.8576890586 | 0.9650824466 | 0.00168377782 | Cataract | PDI_H | TJ Cohort |
| M2 | 0.9121647527 | 0.8598103065 | 0.9677070975 | 0.002300044174 | Cataract | PDI_H | TJ Cohort |
| M3 | 0.9159828696 | 0.8632526866 | 0.9719339776 | 0.003718935203 | Cataract | PDI_H | TJ Cohort |
| M0 | 0.9027574186 | 0.8376665598 | 0.972906161 | 0.007375076005 | Glaucoma | PDI_H | TJ Cohort |
| M1 | 0.9020404949 | 0.8367992497 | 0.9723682887 | 0.007112311976 | Glaucoma | PDI_H | TJ Cohort |
| M2 | 0.9048141496 | 0.838177171 | 0.9767489186 | 0.01038473218 | Glaucoma | PDI_H | TJ Cohort |
| M3 | 0.9086252245 | 0.8411614558 | 0.9814997975 | 0.01491624727 | Glaucoma | PDI_H | TJ Cohort |
| M0 | 0.9736206006 | 0.8630856887 | 1.098311658 | 0.6637012465 | RVO | PDI_H | TJ Cohort |
| M1 | 0.9717444982 | 0.8612695017 | 1.096390117 | 0.6415783385 | RVO | PDI_H | TJ Cohort |
| M2 | 0.9737479206 | 0.8623744815 | 1.099504952 | 0.6677197777 | RVO | PDI_H | TJ Cohort |
| M3 | 0.9808836694 | 0.8682769129 | 1.108094386 | 0.7563839947 | RVO | PDI_H | TJ Cohort |
| M0 | 0.9265203315 | 0.8533174262 | 1.00600304 | 0.06914506536 | DR | PDI_H | TJ Cohort |
| M1 | 0.9254042761 | 0.8521150062 | 1.004997058 | 0.06553502013 | DR | PDI_H | TJ Cohort |
| M2 | 0.9984476657 | 0.8425920537 | 1.183132142 | 0.9856858555 | DR | PDI_H | TJ Cohort |
| M3 | 1.096396068 | 0.9087527734 | 1.322784781 | 0.3365899963 | DR | PDI_H | TJ Cohort |
| M0 | 1.091718502 | 1.017584053 | 1.171253897 | 0.01445146458 | AMD | PDI_U | TJ Cohort |
| M1 | 1.092460467 | 1.018153801 | 1.172190165 | 0.0138708559 | AMD | PDI_U | TJ Cohort |
| M2 | 1.094372653 | 1.018809237 | 1.175540484 | 0.01349292509 | AMD | PDI_U | TJ Cohort |
| M3 | 1.08041819 | 1.002658552 | 1.164208357 | 0.0423901271 | AMD | PDI_U | TJ Cohort |
| M0 | 1.1011176 | 1.031759531 | 1.175138134 | 0.003708963539 | Cataract | PDI_U | TJ Cohort |
| M1 | 1.100197629 | 1.030814113 | 1.174251311 | 0.004063936007 | Cataract | PDI_U | TJ Cohort |
| M2 | 1.098461129 | 1.029111533 | 1.172484043 | 0.004765893192 | Cataract | PDI_U | TJ Cohort |
| M3 | 1.097251879 | 1.027851365 | 1.171338316 | 0.005368379742 | Cataract | PDI_U | TJ Cohort |
| M0 | 0.9291654332 | 0.8552991455 | 1.009411043 | 0.08214681533 | Glaucoma | PDI_U | TJ Cohort |
| M1 | 0.9301028799 | 0.8560563685 | 1.01055421 | 0.08690560611 | Glaucoma | PDI_U | TJ Cohort |
| M2 | 0.9269192762 | 0.8525217241 | 1.007809326 | 0.07544172027 | Glaucoma | PDI_U | TJ Cohort |
| M3 | 0.9241140727 | 0.8495339063 | 1.005241595 | 0.06602858161 | Glaucoma | PDI_U | TJ Cohort |
| M0 | 0.9949086015 | 0.8707088819 | 1.136824426 | 0.9401914862 | RVO | PDI_U | TJ Cohort |
| M1 | 0.9912912089 | 0.8673740778 | 1.132911723 | 0.8978459891 | RVO | PDI_U | TJ Cohort |
| M2 | 0.990773959 | 0.8673342002 | 1.131781772 | 0.8914022295 | RVO | PDI_U | TJ Cohort |
| M3 | 0.9873658669 | 0.8638452394 | 1.128548623 | 0.8520791407 | RVO | PDI_U | TJ Cohort |
| M0 | 1.107997453 | 1.011582646 | 1.213601638 | 0.02724883519 | DR | PDI_U | TJ Cohort |
| M1 | 1.106350535 | 1.009919043 | 1.211989728 | 0.02984586081 | DR | PDI_U | TJ Cohort |
| M2 | 1.305839689 | 1.070519698 | 1.592887359 | 0.008483997037 | DR | PDI_U | TJ Cohort |
| M3 | 1.31508283 | 1.053924625 | 1.640954968 | 0.01530840065 | DR | PDI_U | TJ Cohort |
| Model | HR | LCL | UCL | pvalue | Disease | Exposure | Cohort |
| M0 | 0.8237828785 | 0.7933025839 | 0.8554342878 | 6.95107e-24 | AMD | PDI_H | UKB |
| M1 | 0.8246027212 | 0.7937205021 | 0.8566865112 | 4.04802e-23 | AMD | PDI_H | UKB |
| M2 | 0.8242777115 | 0.7933884432 | 0.8563696025 | 3.52056e-23 | AMD | PDI_H | UKB |
| M3 | 0.825150425 | 0.7941629937 | 0.8573469543 | 7.48103e-23 | AMD | PDI_H | UKB |
| M0 | 0.9002440543 | 0.8811690141 | 0.9197320199 | 6.73738e-22 | Cataract | PDI_H | UKB |
| M1 | 0.903011682 | 0.8836120285 | 0.9228372537 | 3.34414e-20 | Cataract | PDI_H | UKB |
| M2 | 0.9031529681 | 0.8837348191 | 0.9229977887 | 4.08531e-20 | Cataract | PDI_H | UKB |
| M3 | 0.9048661733 | 0.8853588172 | 0.9248033404 | 2.46284e-19 | Cataract | PDI_H | UKB |
| M0 | 0.9105282501 | 0.8741537023 | 0.9484163848 | 6.60023e-06 | Glaucoma | PDI_H | UKB |
| M1 | 0.9037895221 | 0.8672094895 | 0.9419125484 | 1.59536e-06 | Glaucoma | PDI_H | UKB |
| M2 | 0.9026526885 | 0.8661091378 | 0.9407381132 | 1.18985e-06 | Glaucoma | PDI_H | UKB |
| M3 | 0.9014656688 | 0.8648777986 | 0.9396013556 | 9.24577e-07 | Glaucoma | PDI_H | UKB |
| M0 | 0.9098378993 | 0.8270090583 | 1.000962438 | 0.05234971854 | RVO | PDI_H | UKB |
| M1 | 0.919390136 | 0.8345911055 | 1.012805213 | 0.08870224693 | RVO | PDI_H | UKB |
| M2 | 0.9161212669 | 0.8315870515 | 1.00924873 | 0.07612642775 | RVO | PDI_H | UKB |
| M3 | 0.9188493243 | 0.8339118316 | 1.012438065 | 0.08722703034 | RVO | PDI_H | UKB |
| M0 | 0.8536233845 | 0.7988347114 | 0.9121697796 | 2.92257e-06 | DR | PDI_H | UKB |
| M1 | 0.9294063629 | 0.8688042154 | 0.9942357231 | 0.03333373571 | DR | PDI_H | UKB |
| M2 | 0.9019227536 | 0.8420045799 | 0.9661047846 | 0.00324867453 | DR | PDI_H | UKB |
| M3 | 0.8975507861 | 0.8379259696 | 0.9614183626 | 0.002057032972 | DR | PDI_H | UKB |
| M0 | 1.080967145 | 1.037316276 | 1.126454869 | 0.0002138186168 | AMD | PDI_U | UKB |
| M1 | 1.086253402 | 1.042290261 | 1.132070881 | 8.67125e-05 | AMD | PDI_U | UKB |
| M2 | 1.086343296 | 1.042380709 | 1.132160013 | 8.51736e-05 | AMD | PDI_U | UKB |
| M3 | 1.090307813 | 1.046112103 | 1.136370685 | 4.21615e-05 | AMD | PDI_U | UKB |
| M0 | 1.041377963 | 1.017175606 | 1.066156183 | 0.0007263158314 | Cataract | PDI_U | UKB |
| M1 | 1.046437064 | 1.022044426 | 1.071411869 | 0.0001619553102 | Cataract | PDI_U | UKB |
| M2 | 1.047527728 | 1.023111949 | 1.072526171 | 0.0001138917387 | Cataract | PDI_U | UKB |
| M3 | 1.050591694 | 1.026060021 | 1.075709886 | 4.23775e-05 | Cataract | PDI_U | UKB |
| M0 | 1.002115646 | 0.9585154817 | 1.047699059 | 0.9258076633 | Glaucoma | PDI_U | UKB |
| M1 | 0.9993892724 | 0.9557283328 | 1.045044793 | 0.9786153619 | Glaucoma | PDI_U | UKB |
| M2 | 0.9985002117 | 0.9548522483 | 1.0441434 | 0.94752495 | Glaucoma | PDI_U | UKB |

| Model | OR | LCL | UCL | pvalue | Disease | Exposure | Cohort |
|---|---|---|---|---|---|---|---|
| M3 | 0.9990565112 | 0.9552650206 | 1.044855502 | 0.9670755823 | Glaucoma | PDI_U | UKB |
| M0 | 0.9525443024 | 0.8584578468 | 1.056942576 | 0.3595183598 | RVO | PDI_U | UKB |
| M1 | 0.960434321 | 0.8652982922 | 1.066030169 | 0.4481262103 | RVO | PDI_U | UKB |
| M2 | 0.9573509127 | 0.8623931307 | 1.06276446 | 0.4134672598 | RVO | PDI_U | UKB |
| M3 | 0.963050142 | 0.8674257797 | 1.069216062 | 0.4804064943 | RVO | PDI_U | UKB |
| M0 | 1.061830527 | 0.9882290861 | 1.140913665 | 0.1016453683 | DR | PDI_U | UKB |
| M1 | 1.133862414 | 1.055494966 | 1.218048418 | 0.0005858823508 | DR | PDI_U | UKB |
| M2 | 1.125302302 | 1.046870142 | 1.209610647 | 0.001361687804 | DR | PDI_U | UKB |
| M3 | 1.144864467 | 1.06450561 | 1.231289563 | 0.0002689197653 | DR | PDI_U | UKB |

**Supplementary Table 4. Proteome-wide association analysis of the unhealthy plant-based diet index (PDI-U) in the UK Biobank**

| Protein | N_used | term | beta | se | t | p_val |
|---|---|---|---|---|---|---|
| igfbp1 | 13785 | PDI_U | -0.00567083373 | 0.0008923340891 | -6.355056698 | 2.14909e-10 |
| inhbc | 13632 | PDI_U | 0.00566285105 | 0.0008937639916 | 6.335957929 | 2.43293e-10 |
| tff2 | 13691 | PDI_U | -0.005764815788 | 0.0009354280725 | -6.162756878 | 7.34961e-10 |
| lep | 13618 | PDI_U | 0.003315965541 | 0.0005597302938 | 5.924220249 | 3.21362e-09 |
| vsig2 | 11730 | PDI_U | -0.005601161977 | 0.001020015817 | -5.49125012 | 4.07419e-08 |
| oxt | 13784 | PDI_U | 0.00466387154 | 0.0009043913727 | 5.156917327 | 2.5453e-07 |
| rln2 | 11512 | PDI_U | 0.005307510311 | 0.001068285584 | 4.968250428 | 6.85315e-07 |
| cdhr2 | 13579 | PDI_U | 0.003811637605 | 0.0007716041502 | 4.939887382 | 7.90991e-07 |
| tff1 | 13564 | PDI_U | -0.00458917731 | 0.0009444869568 | -4.858910202 | 1.19354e-06 |
| fabp2 | 13618 | PDI_U | -0.004796193984 | 0.0009910079852 | -4.839712752 | 1.31454e-06 |
| igsf9 | 11781 | PDI_U | 0.004453765001 | 0.0009212390007 | 4.834538049 | 1.35143e-06 |
| ssc4d | 13599 | PDI_U | 0.004091426173 | 0.0008518090512 | 4.803219885 | 1.57801e-06 |
| slc39a5 | 13621 | PDI_U | 0.004435227347 | 0.0009249217504 | 4.795246025 | 1.64196e-06 |
| igfbp2 | 13777 | PDI_U | -0.003934007089 | 0.0008599725007 | -4.574573124 | 4.81358e-06 |
| ckb | 11790 | PDI_U | -0.004166149526 | 0.000928486652 | -4.487032223 | 7.29067e-06 |
| guca2a | 13544 | PDI_U | -0.004319052882 | 0.0009634501405 | -4.482902332 | 7.42396e-06 |
| cntn4 | 13632 | PDI_U | -0.004356209548 | 0.0009718157591 | -4.482546724 | 7.43594e-06 |
| xpnpep2 | 13699 | PDI_U | 0.004347362054 | 0.0009813309779 | 4.430067075 | 9.4935e-06 |
| ppy | 13617 | PDI_U | 0.004259518865 | 0.0009696192541 | 4.392980902 | 1.12652e-05 |
| cdhr5 | 13618 | PDI_U | 0.004147248309 | 0.0009541927839 | 4.346342143 | 1.39431e-05 |
| ces1 | 13687 | PDI_U | 0.004006683742 | 0.0009236723581 | 4.337775951 | 1.44963e-05 |
| mmp12 | 13646 | PDI_U | -0.003929307984 | 0.0009069996762 | -4.332204395 | 1.4868e-05 |
| ctsz | 13653 | PDI_U | 0.004028983388 | 0.0009459615071 | 4.259140946 | 2.06589e-05 |
| rbp2 | 13579 | PDI_U | -0.004162099695 | 0.0009820751864 | -4.238066243 | 2.26944e-05 |
| spink4 | 13621 | PDI_U | -0.004086401087 | 0.0009732085517 | -4.198895581 | 2.69926e-05 |
| plau | 13632 | PDI_U | -0.004064581384 | 0.0009723318272 | -4.180241014 | 2.9302e-05 |
| epha1 | 13695 | PDI_U | 0.003656766602 | 0.0008759827051 | 4.174473515 | 3.00524e-05 |
| susd2 | 13632 | PDI_U | -0.003877618841 | 0.0009478952519 | -4.090767237 | 4.32422e-05 |
| bglap | 11790 | PDI_U | 0.004231858864 | 0.001046973541 | 4.04199218 | 5.33361e-05 |
| met | 13688 | PDI_U | -0.00379623527 | 0.0009479239009 | -4.004789062 | 6.2401e-05 |
| ghr | 11537 | PDI_U | 0.003894572097 | 0.0009797562425 | 3.975041881 | 7.07961e-05 |
| cbln4 | 13753 | PDI_U | 0.003893220028 | 0.0009856267565 | 3.949994258 | 7.85433e-05 |
| nppc | 13621 | PDI_U | 0.003367837559 | 0.0008598135503 | 3.916939385 | 9.01183e-05 |
| mrc1 | 11794 | PDI_U | -0.00385031043 | 0.0009903893055 | -3.887673675 | 0.0001017647258 |
| cd38 | 13579 | PDI_U | 0.003108142818 | 0.0008018851937 | 3.876044654 | 0.0001066681246 |
| kit | 13669 | PDI_U | -0.003667269121 | 0.0009528198791 | -3.848858741 | 0.0001192099356 |
| ctso | 13801 | PDI_U | 0.003619416667 | 0.0009489189865 | 3.814252553 | 0.0001371912799 |
| adipoq | 11817 | PDI_U | -0.003345209902 | 0.0008798101081 | -3.80219535 | 0.0001441414757 |
| dsg2 | 13632 | PDI_U | -0.003616882821 | 0.0009537714845 | -3.792190141 | 0.000149969388 |
| ddc | 13599 | PDI_U | -0.00367966115 | 0.0009836198361 | -3.740938333 | 0.0001840885213 |
| crlf1 | 13710 | PDI_U | 0.003530464654 | 0.0009440477279 | 3.739709921 | 0.0001849833719 |
| igfbp6 | 13573 | PDI_U | 0.003040889014 | 0.0008215938218 | 3.701207256 | 0.0002154255053 |
| hbegf | 13715 | PDI_U | 0.003635145844 | 0.0009853892508 | 3.689045564 | 0.0002259678535 |
| f9 | 13669 | PDI_U | 0.003319644913 | 0.0009043238241 | 3.670858629 | 0.0002426582447 |
| c9 | 11794 | PDI_U | -0.003830320548 | 0.001048365821 | -3.653610669 | 0.0002597006748 |
| lepr | 13618 | PDI_U | -0.003407294946 | 0.0009382413432 | -3.631576215 | 0.0002827303122 |
| psg1 | 13552 | PDI_U | -0.003498145639 | 0.0009652910717 | -3.623928307 | 0.0002912248116 |
| tgfbi | 13551 | PDI_U | 0.003529103471 | 0.0009786812698 | 3.605978351 | 0.0003120971295 |
| gas6 | 13707 | PDI_U | -0.003478277993 | 0.0009698052015 | -3.586573869 | 0.0003362182249 |
| mepe | 13658 | PDI_U | 0.003346101312 | 0.0009387525771 | 3.564412384 | 0.0003659177788 |
| plat | 13750 | PDI_U | 0.003053335552 | 0.0008579149171 | 3.559019072 | 0.0003734982436 |
| fabp1 | 13658 | PDI_U | -0.003297329477 | 0.0009275033074 | -3.555059535 | 0.0003791715862 |
| tcn2 | 13777 | PDI_U | -0.00340315562 | 0.0009628025428 | -3.534635057 | 0.0004096786044 |
| apof | 11794 | PDI_U | -0.003333353126 | 0.0009441847205 | -3.53040359 | 0.000416510053 |
| lgals4 | 13658 | PDI_U | -0.003332480145 | 0.0009477624752 | -3.516155399 | 0.0004392657825 |
| lypd8 | 13308 | PDI_U | -0.00352787915 | 0.001008072402 | -3.499628741 | 0.0004674315763 |
| scgb1a1 | 13663 | PDI_U | 0.003327867345 | 0.0009516823107 | 3.496825892 | 0.0004723255386 |
| kitlg | 13618 | PDI_U | 0.003431338443 | 0.0009832246844 | 3.489882321 | 0.0004847629354 |
| ccl25 | 13801 | PDI_U | -0.00335101495 | 0.0009667576906 | -3.466240799 | 0.0005293975743 |
| ssh3 | 11304 | PDI_U | -0.00377319876 | 0.001091200008 | -3.457843414 | 0.000546524783 |
| adamts15 | 13579 | PDI_U | 0.003077465112 | 0.0008955171746 | 3.436522715 | 0.0005909990581 |
| cd109 | 13632 | PDI_U | -0.003382982225 | 0.0009847038992 | -3.435532476 | 0.000593154361 |
| gusb | 13685 | PDI_U | 0.002987784193 | 0.0008747608565 | 3.415543998 | 0.0006383979048 |
| klk6 | 13682 | PDI_U | 0.003329712547 | 0.0009767682488 | 3.408907437 | 0.0006541188664 |
| nrcam | 13618 | PDI_U | 0.003337425685 | 0.0009843214018 | 3.39058531 | 0.0006994180579 |
| itih3 | 13689 | PDI_U | -0.003153104638 | 0.0009318043788 | -3.383869737 | 0.0007167253835 |
| f7 | 13666 | PDI_U | 0.00323449751 | 0.0009572344365 | 3.379002455 | 0.0007295283227 |
| pon3 | 13710 | PDI_U | -0.002927041484 | 0.0008665417409 | -3.377842458 | 0.0007326033968 |

| Protein | N_used | term | beta | se | t | p_val |
|---|---|---|---|---|---|---|
| cant1 | 13564 | PDI_U | 0.003306391529 | 0.0009798707078 | 3.374314084 | 0.0007420734025 |
| cntn3 | 13632 | PDI_U | 0.00297209679 | 0.0008830274707 | 3.365803317 | 0.0007653201383 |
| boc | 13388 | PDI_U | -0.003298350311 | 0.0009806804113 | -3.363328433 | 0.0007722478809 |
| col4a1 | 13618 | PDI_U | -0.003241531145 | 0.000966122753 | -3.355195947 | 0.0007952561939 |
| amot | 11537 | PDI_U | 0.003262959269 | 0.000977006319 | 3.339752472 | 0.0008411918938 |
| pkd1 | 11781 | PDI_U | -0.003276390365 | 0.0009847796534 | -3.327028898 | 0.0008804620386 |
| pla2g7 | 13632 | PDI_U | 0.003178404605 | 0.0009558228817 | 3.32530709 | 0.0008855454335 |
| lpl | 13618 | PDI_U | -0.00286466557 | 0.0008636328049 | -3.316994854 | 0.0009122971867 |
| stc1 | 13784 | PDI_U | 0.003025902108 | 0.0009165607715 | 3.301365498 | 0.0009646020684 |
| cep152 | 11304 | PDI_U | -0.0035973277 | 0.001094060271 | -3.288052583 | 0.001011913843 |
| scg2 | 13692 | PDI_U | 0.003204928649 | 0.0009764095674 | 3.282360964 | 0.001031997863 |
| wfikkn1 | 13510 | PDI_U | 0.00306628617 | 0.0009382022819 | 3.268256994 | 0.001084826332 |
| ceacam5 | 13118 | PDI_U | -0.003169578493 | 0.0009853962137 | -3.216552336 | 0.00130055865 |
| mamdc4 | 11730 | PDI_U | 0.003283619541 | 0.001026638899 | 3.198417228 | 0.001385517156 |
| sfrp4 | 11537 | PDI_U | 0.003299935061 | 0.001038130278 | 3.178729232 | 0.001483132115 |
| ccl11 | 13801 | PDI_U | -0.002972771543 | 0.0009389218649 | -3.166154346 | 0.001548050937 |
| creg1 | 13488 | PDI_U | 0.003057008032 | 0.0009659928457 | 3.164628026 | 0.001556266289 |
| hs6st1 | 13753 | PDI_U | 0.003091628811 | 0.0009787453369 | 3.158767347 | 0.001587810853 |
| serpina12 | 13212 | PDI_U | 0.003120171601 | 0.0009895338726 | 3.153173113 | 0.001618677917 |
| serpina1 | 11561 | PDI_U | -0.003382331945 | 0.001076415442 | -3.142217971 | 0.0016809704 |
| lgals3 | 13663 | PDI_U | -0.002942044591 | 0.0009397677791 | -3.130608068 | 0.001748128107 |
| anxa10 | 13320 | PDI_U | -0.003089014852 | 0.0009868621376 | -3.130138177 | 0.001751019907 |
| edn1 | 11474 | PDI_U | -0.003213636608 | 0.00103101765 | -3.11695596 | 0.001831807066 |
| prss27 | 13568 | PDI_U | 0.003106541896 | 0.001000542377 | 3.104857892 | 0.001907627982 |
| crtac1 | 13800 | PDI_U | -0.002900225946 | 0.0009372410143 | -3.094429183 | 0.001975866511 |
| s100g | 11757 | PDI_U | -0.003260762196 | 0.001056522224 | -3.08631671 | 0.002031244498 |
| kir3dl2 | 11304 | PDI_U | -0.003372337483 | 0.001094006512 | -3.082557047 | 0.00205724951 |
| galnt2 | 13753 | PDI_U | 0.002973203542 | 0.0009673780158 | 3.073466104 | 0.002120021679 |
| cd86 | 11724 | PDI_U | -0.003204634725 | 0.00105554025 | -3.036013761 | 0.002402551933 |
| npy | 13753 | PDI_U | 0.002945451053 | 0.0009705646971 | 3.034780743 | 0.002411607275 |
| ca9 | 13753 | PDI_U | -0.002893837748 | 0.0009537006412 | -3.034325052 | 0.002415250822 |
| spink1 | 13632 | PDI_U | -0.002927319925 | 0.000965751777 | -3.031130767 | 0.00244097331 |
| pgf | 13801 | PDI_U | 0.002573851066 | 0.0008494043337 | 3.030183581 | 0.00244858048 |
| mstn | 13388 | PDI_U | 0.002727356474 | 0.0009070681765 | 3.006782229 | 0.00264519138 |
| cdh17 | 13618 | PDI_U | -0.002949887713 | 0.0009817941435 | -3.004588826 | 0.002664243006 |
| itpr1 | 11474 | PDI_U | 0.003208335066 | 0.00107764537 | 2.977171484 | 0.002915293735 |
| csf1r | 11794 | PDI_U | -0.003052991399 | 0.001025705872 | -2.976478427 | 0.002921720403 |
| pga4 | 11794 | PDI_U | -0.00305974173 | 0.001028566158 | -2.974764148 | 0.002938084053 |
| fstl3 | 13650 | PDI_U | 0.002560413034 | 0.0008620872165 | 2.970016241 | 0.002983025512 |
| ghrl | 13618 | PDI_U | -0.002722510831 | 0.0009180797488 | -2.965440458 | 0.003027744507 |
| ckmt1a_ckmt1b | 13608 | PDI_U | -0.002959259056 | 0.0009987333441 | -2.963012172 | 0.003051720932 |
| agt | 11794 | PDI_U | 0.003122819351 | 0.001055656707 | 2.958176963 | 0.003100792128 |
| ptgds | 13531 | PDI_U | 0.00272992349 | 0.0009262536629 | 2.947274164 | 0.003211364465 |
| ccl4 | 13661 | PDI_U | 0.002870113635 | 0.0009747757993 | 2.944383352 | 0.003241444452 |
| c1ql2 | 11411 | PDI_U | -0.003015162303 | 0.001030322069 | -2.926426982 | 0.003435569483 |
| bst1 | 13784 | PDI_U | -0.002852515799 | 0.0009784890809 | -2.915224968 | 0.003560049694 |
| tp53bp1 | 11411 | PDI_U | 0.003133957286 | 0.001075959828 | 2.912708453 | 0.003590044047 |
| furin | 13699 | PDI_U | 0.002562334334 | 0.0008819240279 | 2.905391228 | 0.003673817743 |
| cd28 | 13308 | PDI_U | -0.002841927179 | 0.0009849916005 | -2.885229861 | 0.00391756737 |
| rtn4r | 13753 | PDI_U | 0.002535773915 | 0.0008795133145 | 2.883155801 | 0.003943234778 |
| robo1 | 13677 | PDI_U | -0.002772792229 | 0.0009630574901 | -2.879155457 | 0.003993606669 |
| grpel1 | 13579 | PDI_U | -0.002811909158 | 0.0009774587838 | -2.876754708 | 0.004024140637 |
| adgre5 | 13727 | PDI_U | 0.002721031925 | 0.0009478707128 | 2.870678341 | 0.004102188039 |
| ppp1r14d | 11757 | PDI_U | -0.003033383075 | 0.001057221921 | -2.869201835 | 0.004122430623 |
| ret | 13579 | PDI_U | 0.002701243231 | 0.0009428770351 | 2.864894499 | 0.004177887493 |
| timp4 | 13632 | PDI_U | -0.002673363453 | 0.0009334130889 | -2.864073244 | 0.004188703447 |
| atf2 | 13320 | PDI_U | -0.00289654098 | 0.001012126157 | -2.861837883 | 0.004218490978 |
| fgl1 | 11794 | PDI_U | -0.002830979876 | 0.0009908121156 | -2.857231791 | 0.004281020234 |
| vgf | 11757 | PDI_U | 0.002962036412 | 0.001039039618 | 2.850744438 | 0.004369300604 |
| omg | 13579 | PDI_U | 0.002792556657 | 0.0009803315196 | 2.848583975 | 0.0043980315 |
| retn | 13601 | PDI_U | 0.002776573922 | 0.0009800519682 | 2.833088461 | 0.004616850202 |
| stc2 | 13613 | PDI_U | 0.002680251691 | 0.000946880135 | 2.830613498 | 0.004652695155 |
| sele | 13800 | PDI_U | 0.002620997934 | 0.0009289333482 | 2.821513448 | 0.004786597253 |
| ermap | 11411 | PDI_U | 0.002915374504 | 0.001034840724 | 2.817220501 | 0.004852457716 |
| lrp1 | 13579 | PDI_U | -0.002692737685 | 0.0009570568814 | -2.813560758 | 0.004906659779 |
| gpr101 | 11383 | PDI_U | -0.00306370226 | 0.001089044264 | -2.813202697 | 0.004913486286 |
| pnpt1 | 13378 | PDI_U | -0.002810312146 | 0.001000569593 | -2.808712324 | 0.004981223813 |
| calb1 | 13579 | PDI_U | 0.002697951168 | 0.0009632535673 | 2.800873269 | 0.005103664539 |
| gcg | 13308 | PDI_U | 0.002774243019 | 0.0009908671024 | 2.799813429 | 0.005120587803 |
| reg1a | 13560 | PDI_U | -0.002715263514 | 0.0009724765245 | -2.792112144 | 0.005243857197 |

| Protein | N_used | term | beta | se | t | p_val |
|---|---|---|---|---|---|---|
| prkd2 | 11355 | PDI_U | -0.003027853105 | 0.001087036071 | -2.785421006 | 0.00535469578 |
| tnxb | 13632 | PDI_U | -0.002709130487 | 0.0009798266718 | -2.764907882 | 0.005701547508 |
| ttr | 11671 | PDI_U | 0.002766955961 | 0.001002898741 | 2.758958455 | 0.005807633894 |
| shbg | 11794 | PDI_U | -0.002545889544 | 0.0009258596518 | -2.749757525 | 0.005973031881 |
| cpa2 | 13746 | PDI_U | 0.002716036834 | 0.0009890858745 | 2.7460071 | 0.006040397994 |
| cemip2 | 11411 | PDI_U | 0.002922309232 | 0.001065670228 | 2.742226588 | 0.006111946481 |
| igf2bp3 | 11355 | PDI_U | -0.002977009918 | 0.001088318443 | -2.735421728 | 0.006239723879 |
| lrrc25 | 13579 | PDI_U | -0.00262950126 | 0.0009617663808 | -2.734033246 | 0.006264487983 |
| osmr | 13712 | PDI_U | -0.002598264134 | 0.0009524256018 | -2.728049444 | 0.006379125313 |
| cda | 11750 | PDI_U | 0.002772234394 | 0.00101739319 | 2.724840721 | 0.006442780794 |
| cd163 | 13689 | PDI_U | -0.002575598019 | 0.0009473437178 | -2.718757691 | 0.00656102251 |
| mthfd2 | 11767 | PDI_U | -0.002904144926 | 0.001068397408 | -2.718225357 | 0.006572935475 |
| sftpa1 | 13308 | PDI_U | -0.002728998016 | 0.001004137476 | -2.717753376 | 0.006581195673 |
| ace | 11794 | PDI_U | -0.002879881 | 0.001064879779 | -2.704418899 | 0.006852225584 |
| nomo1 | 13632 | PDI_U | 0.002516358269 | 0.0009322493326 | 2.699233114 | 0.00695852373 |
| ca4 | 13800 | PDI_U | -0.002620935152 | 0.0009753050301 | -2.687297893 | 0.007211910091 |
| hs6st2 | 11411 | PDI_U | 0.002741463182 | 0.001021113975 | 2.684776869 | 0.007268321968 |
| st3gal1 | 13579 | PDI_U | -0.00253788388 | 0.0009460695947 | -2.682555167 | 0.007315062315 |
| afm | 11817 | PDI_U | 0.002635005824 | 0.000982864038 | 2.68094642 | 0.007351628028 |
| reg3a | 13800 | PDI_U | -0.00256488509 | 0.0009570439546 | -2.680007619 | 0.007370795556 |
| nxph1 | 13320 | PDI_U | 0.002686775405 | 0.001002855724 | 2.679124565 | 0.007390568735 |
| adam23 | 13801 | PDI_U | 0.002623140326 | 0.0009830654082 | 2.66832736 | 0.00763191036 |
| actn2 | 11355 | PDI_U | -0.002799936881 | 0.001052821404 | -2.659460447 | 0.007837588931 |
| adh4 | 13388 | PDI_U | 0.002377762114 | 0.000897602668 | 2.649014089 | 0.008082180653 |
| klk14 | 13753 | PDI_U | -0.002557031181 | 0.0009653343742 | -2.648855411 | 0.008085722121 |
| clec4g | 13621 | PDI_U | 0.002561693935 | 0.0009671849927 | 2.648608027 | 0.008091729077 |
| gpr15l | 11724 | PDI_U | -0.002672700528 | 0.001010673592 | -2.644474486 | 0.008192698667 |
| vwa1 | 13732 | PDI_U | 0.002500099768 | 0.0009476270857 | 2.638273858 | 0.008342332507 |
| tf | 11817 | PDI_U | -0.002775421478 | 0.001054067222 | -2.633059277 | 0.008472982054 |
| art5 | 11383 | PDI_U | -0.002865834872 | 0.00108915659 | -2.631242282 | 0.008518814566 |
| adam22 | 13552 | PDI_U | 0.002588614507 | 0.0009842793783 | 2.629959099 | 0.008549172863 |
| tnfsf14 | 13552 | PDI_U | 0.00259933016 | 0.0009892758349 | 2.627507989 | 0.008610978473 |
| marco | 13618 | PDI_U | -0.002528834865 | 0.00096372551 | -2.624019847 | 0.008699574655 |
| calca | 13535 | PDI_U | 0.002093042324 | 0.0007977738616 | 2.623603536 | 0.008710268163 |
| pdia5 | 11304 | PDI_U | 0.002864335112 | 0.001092815073 | 2.621061131 | 0.008777400202 |
| pepd | 11794 | PDI_U | -0.002764864779 | 0.001060276703 | -2.607682287 | 0.00912724734 |
| bcan | 13552 | PDI_U | 0.002423452476 | 0.0009310492834 | 2.602926096 | 0.009253264224 |
| cpb1 | 13800 | PDI_U | 0.002546994018 | 0.0009852979017 | 2.584998926 | 0.009748101766 |
| cd300lg | 13632 | PDI_U | -0.002463375358 | 0.0009529720181 | -2.584939863 | 0.009749896458 |
| gast | 11767 | PDI_U | -0.002652606108 | 0.001028960911 | -2.577946429 | 0.009951000398 |
| ptprr | 11767 | PDI_U | 0.002677338632 | 0.001039510678 | 2.575575882 | 0.01001943447 |
| tgfbr2 | 13699 | PDI_U | 0.002311205582 | 0.0009027098056 | 2.560297415 | 0.0104688975 |
| megf10 | 13658 | PDI_U | -0.002523919243 | 0.0009871637069 | -2.556738285 | 0.01057659215 |
| bst2 | 13784 | PDI_U | -0.002444560979 | 0.0009635710891 | -2.536980412 | 0.01119226112 |
| spint1 | 13575 | PDI_U | -0.002506477644 | 0.0009910557855 | -2.529098443 | 0.01144683156 |
| rcor1 | 13388 | PDI_U | -0.002520222225 | 0.001001297319 | -2.51695693 | 0.01184892492 |
| reck | 11474 | PDI_U | -0.002552324635 | 0.001014454202 | -2.515958463 | 0.0118844683 |
| krt19 | 13604 | PDI_U | -0.002449095812 | 0.0009737983049 | -2.514992889 | 0.01191492137 |
| pnlip | 11794 | PDI_U | 0.002678608363 | 0.001065319105 | 2.514371845 | 0.01193767614 |
| ereg | 13535 | PDI_U | 0.002496341415 | 0.0009984595982 | 2.500192717 | 0.01242432188 |
| plin1 | 13320 | PDI_U | 0.00229000865 | 0.0009227065966 | 2.481838385 | 0.01308289975 |
| bgn | 13291 | PDI_U | -0.002472038433 | 0.0009983073103 | -2.476229922 | 0.01329017177 |
| gzmb | 13621 | PDI_U | 0.002410878077 | 0.0009749246332 | 2.472886616 | 0.01341478917 |
| nell1 | 13699 | PDI_U | -0.002456194857 | 0.0009933208951 | -2.472710348 | 0.01342133471 |
| dmp1 | 11474 | PDI_U | 0.002536181909 | 0.001027535387 | 2.468218557 | 0.01359322554 |
| ism1 | 13621 | PDI_U | 0.002355809365 | 0.0009571309089 | 2.461324092 | 0.01385484229 |
| acy3 | 11355 | PDI_U | -0.002627246581 | 0.001067811669 | -2.460402575 | 0.01389292023 |
| mfap5 | 13800 | PDI_U | 0.002216878197 | 0.0009015436355 | 2.458980475 | 0.01394541456 |
| apoa4 | 11817 | PDI_U | 0.002586538713 | 0.001052035726 | 2.458603496 | 0.01396210797 |
| nphs1 | 11411 | PDI_U | -0.002657757344 | 0.001084094563 | -2.451591804 | 0.01423748924 |
| il1rn | 13629 | PDI_U | 0.002102902717 | 0.0008579199463 | 2.451164268 | 0.01425197438 |
| itm2a | 13621 | PDI_U | -0.002441864962 | 0.0009966920885 | -2.449969244 | 0.01429934821 |
| sord | 13695 | PDI_U | 0.002309790855 | 0.0009427913179 | 2.449949221 | 0.01430007536 |
| ptprn2 | 13513 | PDI_U | 0.002426460526 | 0.000990432695 | 2.449899461 | 0.0143022185 |
| phospho1 | 13552 | PDI_U | 0.002274600153 | 0.0009285615056 | 2.449595573 | 0.01431425145 |
| mog | 13753 | PDI_U | 0.002307215913 | 0.0009434522212 | 2.445503716 | 0.01447745633 |
| sod3 | 11794 | PDI_U | -0.002424558373 | 0.000993018168 | -2.441605251 | 0.01463673381 |
| cdh15 | 13767 | PDI_U | 0.002018062883 | 0.0008265671376 | 2.441499052 | 0.01463894122 |
| hmmr | 11278 | PDI_U | -0.002673784198 | 0.001096144695 | -2.439262089 | 0.01473261526 |
| cd300a | 11537 | PDI_U | 0.002535343296 | 0.001047390586 | 2.420628301 | 0.01550906288 |

| Protein | N_used | term | beta | se | t | p_val |
|---|---|---|---|---|---|---|
| lsp1 | 13621 | PDI_U | 0.002320744627 | 0.0009591099917 | 2.419685591 | 0.01554694847 |
| tmprss15 | 13579 | PDI_U | 0.002368200866 | 0.0009834935647 | 2.407947496 | 0.01605574466 |
| rarres2 | 13712 | PDI_U | 0.00215491478 | 0.0008967524352 | 2.403020828 | 0.01627347231 |
| angpt2 | 13753 | PDI_U | -0.002314426882 | 0.0009634212011 | -2.402300136 | 0.01630551859 |
| hs3st3b1 | 13579 | PDI_U | -0.002382694559 | 0.0009973802066 | -2.388953123 | 0.01691003865 |
| oxct1 | 11474 | PDI_U | -0.002585162104 | 0.001082190672 | -2.388823126 | 0.01691851518 |
| dner | 13641 | PDI_U | -0.002269372008 | 0.0009511282702 | -2.385978926 | 0.01704729648 |
| inhbb | 11537 | PDI_U | 0.00242111717 | 0.001015233502 | 2.384788491 | 0.01710501417 |
| fabp6 | 13371 | PDI_U | -0.00239025712 | 0.001005030015 | -2.378294265 | 0.01740691675 |
| brme1 | 11383 | PDI_U | -0.002592132337 | 0.001090198752 | -2.377669515 | 0.01743886677 |
| podxl2 | 13699 | PDI_U | 0.002305131685 | 0.0009707894213 | 2.374491969 | 0.01758682503 |
| angptl7 | 13753 | PDI_U | 0.002290058974 | 0.000964697892 | 2.373861282 | 0.01761682578 |
| rab37 | 13378 | PDI_U | -0.002385228981 | 0.001006031753 | -2.370928129 | 0.01775757867 |
| slamf7 | 13621 | PDI_U | -0.002350294035 | 0.0009919070474 | -2.369470044 | 0.01782746889 |
| pcare | 11383 | PDI_U | -0.002578610169 | 0.001089250936 | -2.367324263 | 0.01793387199 |
| hgf | 13801 | PDI_U | 0.002061480927 | 0.0008722410325 | 2.363430348 | 0.01812042145 |
| copb2 | 11355 | PDI_U | 0.002567945144 | 0.001088902035 | 2.358288497 | 0.01837628983 |
| cd36 | 11411 | PDI_U | 0.002509694743 | 0.001067054012 | 2.351984729 | 0.01869045858 |
| serpina5 | 11611 | PDI_U | 0.002446463625 | 0.001042031077 | 2.347783747 | 0.01890219206 |
| chga | 11790 | PDI_U | -0.002431923018 | 0.001036892971 | -2.345394448 | 0.01902346222 |
| pnliprp1 | 11767 | PDI_U | 0.00250407125 | 0.001068462235 | 2.343621671 | 0.01911410231 |
| adam15 | 13388 | PDI_U | -0.002343513533 | 0.001000925583 | -2.341346424 | 0.01922893062 |
| ostn | 11483 | PDI_U | -0.002527369608 | 0.001079518737 | -2.341200315 | 0.01923888042 |
| pbxip1 | 11724 | PDI_U | 0.002409976077 | 0.001030115248 | 2.339520827 | 0.01932520748 |
| faslg | 13801 | PDI_U | 0.002181303343 | 0.0009333564637 | 2.337052806 | 0.01945067499 |
| crip2 | 13552 | PDI_U | 0.002113663928 | 0.000908473637 | 2.326610088 | 0.02000070497 |
| pigr | 13463 | PDI_U | -0.002223721747 | 0.000956611496 | -2.324581878 | 0.02010913879 |
| cd99l2 | 13632 | PDI_U | 0.002195115793 | 0.0009456605532 | 2.321251305 | 0.0202879678 |
| ftcd | 11741 | PDI_U | 0.00226763905 | 0.0009805102833 | 2.312713175 | 0.02075563423 |
| fcgr2b | 13308 | PDI_U | 0.002312884644 | 0.001001183834 | 2.310149809 | 0.02089509684 |
| ggt1 | 13602 | PDI_U | 0.002029735555 | 0.0008803358536 | 2.305637726 | 0.0211458662 |
| heph | 11781 | PDI_U | -0.002375470358 | 0.00103435615 | -2.296569085 | 0.02166084422 |
| folr2 | 13784 | PDI_U | -0.002139720777 | 0.0009341842187 | -2.290469839 | 0.02200913556 |
| serpina4 | 11570 | PDI_U | 0.002440311866 | 0.001066594662 | 2.287946821 | 0.02215859077 |
| rnaset2 | 13736 | PDI_U | 0.002174389318 | 0.0009553435898 | 2.276028584 | 0.02285963586 |
| il18rap | 13469 | PDI_U | -0.002259565582 | 0.0009933953184 | -2.274588515 | 0.0229462918 |
| il12a_il12b | 13579 | PDI_U | 0.002178945042 | 0.0009590113365 | 2.272074332 | 0.02309760306 |
| cpa1 | 13800 | PDI_U | 0.002243420503 | 0.0009880215746 | 2.270618943 | 0.02318541232 |
| cfhr5 | 11612 | PDI_U | -0.002405118483 | 0.001059287297 | -2.270506301 | 0.02319513716 |
| mrpl52 | 11278 | PDI_U | -0.002487121787 | 0.001096282479 | -2.26868698 | 0.02330620229 |
| gh1 | 13599 | PDI_U | -0.002106617153 | 0.0009297198735 | -2.265862237 | 0.02347548405 |
| b4galt1 | 13710 | PDI_U | 0.002178727779 | 0.0009623627911 | 2.263936012 | 0.02359362087 |
| vamp5 | 13388 | PDI_U | -0.002266634525 | 0.00100176817 | -2.262633804 | 0.0236742401 |
| nptx2 | 11790 | PDI_U | -0.002041761619 | 0.0009037248699 | -2.259273466 | 0.02388457948 |
| nagpa | 11537 | PDI_U | 0.002394243021 | 0.001061086973 | 2.256406009 | 0.02406387268 |
| ids | 13801 | PDI_U | 0.002205306568 | 0.0009786503752 | 2.253416157 | 0.02424856516 |
| pdcd6 | 13618 | PDI_U | -0.002244467645 | 0.001000082472 | -2.244282555 | 0.02483023552 |
| ripk4 | 11355 | PDI_U | -0.002441847811 | 0.001089898391 | -2.240436201 | 0.02508189857 |
| ctla4 | 11790 | PDI_U | -0.002390700714 | 0.001069054288 | -2.23627625 | 0.0253523303 |
| galnt3 | 13378 | PDI_U | 0.002250497514 | 0.001006401332 | 2.236182965 | 0.02535622342 |
| aspn | 11781 | PDI_U | -0.002310086987 | 0.001033087251 | -2.236100565 | 0.02536385124 |
| gpc5 | 13552 | PDI_U | 0.002229565681 | 0.000997260669 | 2.235689976 | 0.02538831807 |
| lilrb4 | 13378 | PDI_U | 0.002093381712 | 0.0009369186245 | 2.234326074 | 0.02547809258 |
| cgb3_cgb5_cgb8 | 11794 | PDI_U | -0.002230175525 | 0.0009983264256 | -2.233914146 | 0.0255074113 |
| duox2 | 13388 | PDI_U | -0.002254024355 | 0.001009710209 | -2.232347792 | 0.0256084737 |
| tap1 | 11570 | PDI_U | -0.002404999711 | 0.001079978316 | -2.226896296 | 0.02597339529 |
| fcn2 | 13572 | PDI_U | 0.002188997245 | 0.0009832676332 | 2.226247637 | 0.02601396606 |
| gip | 11474 | PDI_U | 0.002276095135 | 0.001025548715 | 2.219392508 | 0.02647953289 |
| glp1r | 11383 | PDI_U | -0.002417298787 | 0.001089464501 | -2.218795366 | 0.02652031741 |
| fgfbp3 | 11767 | PDI_U | 0.002308528564 | 0.001041404223 | 2.216745921 | 0.026659524 |
| mcemp1 | 11411 | PDI_U | 0.002396927368 | 0.001082796698 | 2.213644882 | 0.02687293983 |
| morf4l2 | 11304 | PDI_U | -0.00241794337 | 0.001093362245 | -2.211475091 | 0.02702290797 |
| art3 | 13689 | PDI_U | 0.001961211747 | 0.0008872096781 | 2.210539172 | 0.02708425395 |
| myom3 | 11537 | PDI_U | -0.002228482156 | 0.001008266642 | -2.210211133 | 0.02711008726 |
| pcdh17 | 13618 | PDI_U | 0.002172143358 | 0.0009831624291 | 2.209343333 | 0.02716736417 |
| pzp | 11817 | PDI_U | 0.001690625881 | 0.0007658699925 | 2.207458051 | 0.0273012377 |
| ces2 | 13579 | PDI_U | 0.002119175155 | 0.0009614823027 | 2.204070891 | 0.02753608967 |
| fas | 13750 | PDI_U | 0.001963320175 | 0.0008941143962 | 2.1958266 | 0.02812101504 |
| anxa1 | 11467 | PDI_U | 0.002314845358 | 0.001056100786 | 2.191879212 | 0.02840828552 |
| f11r | 13632 | PDI_U | 0.002157889254 | 0.0009885502229 | 2.182882776 | 0.02906145994 |

| Protein | N_used | term | beta | se | t | p_val |
|---|---|---|---|---|---|---|
| cxcl8 | 13539 | PDI_U | -0.002150896848 | 0.0009865645926 | -2.180188569 | 0.02926066433 |
| shc1 | 11304 | PDI_U | -0.002369680483 | 0.001088228493 | -2.177557836 | 0.02945960165 |
| pglyrp1 | 13727 | PDI_U | 0.002103645901 | 0.000967967108 | 2.17326176 | 0.02977767697 |
| gdf15 | 13800 | PDI_U | -0.001729054446 | 0.0007966107566 | -2.170513555 | 0.0299849721 |
| serping1 | 11790 | PDI_U | -0.002257487955 | 0.001043404465 | -2.163578967 | 0.03051672023 |
| cdh2 | 13618 | PDI_U | 0.001914788286 | 0.0008867593585 | 2.159309928 | 0.03084354999 |
| coro6 | 11383 | PDI_U | -0.002321836252 | 0.001081183676 | -2.147494736 | 0.03177498845 |
| cd276 | 13658 | PDI_U | -0.002079476683 | 0.000968564273 | -2.146968189 | 0.03181338202 |
| smad5 | 13579 | PDI_U | 0.002041211093 | 0.0009510100647 | 2.146361189 | 0.03186185315 |
| siglec8 | 11474 | PDI_U | 0.002235870112 | 0.001042897179 | 2.143902732 | 0.03206165527 |
| cnpy2 | 13388 | PDI_U | -0.002158999289 | 0.001009375161 | -2.138946323 | 0.03245806949 |
| top1mt | 11304 | PDI_U | -0.002340436211 | 0.001094745187 | -2.137882166 | 0.03254771441 |
| cdh22 | 11570 | PDI_U | -0.002292404765 | 0.001078228174 | -2.126085018 | 0.03351731655 |
| gja8 | 11304 | PDI_U | -0.002320081536 | 0.001093971452 | -2.120788007 | 0.03396138086 |
| chp1 | 11383 | PDI_U | -0.002311942648 | 0.00109039305 | -2.120283734 | 0.0340037176 |
| pxn | 13320 | PDI_U | 0.002068879112 | 0.0009829614674 | 2.104740807 | 0.03533257608 |
| nell2 | 13710 | PDI_U | 0.002023744074 | 0.0009615464256 | 2.104676405 | 0.03533765409 |
| ncan | 13784 | PDI_U | 0.001857002253 | 0.0008832869651 | 2.10237706 | 0.03553836522 |
| cpxm1 | 13699 | PDI_U | 0.002088273225 | 0.0009938430269 | 2.10121032 | 0.0356407446 |
| tnfsf11 | 13621 | PDI_U | 0.002061711781 | 0.0009812855035 | 2.101031528 | 0.03565654279 |
| gga1 | 13320 | PDI_U | -0.002117246006 | 0.001008476877 | -2.099449234 | 0.03579610267 |
| cdan1 | 11767 | PDI_U | -0.002245417818 | 0.001069741523 | -2.099028381 | 0.03583567246 |
| aldh1a1 | 13632 | PDI_U | 0.001957501637 | 0.000935736645 | 2.091936494 | 0.03646266712 |
| edar | 13621 | PDI_U | 0.002088326052 | 0.000999693373 | 2.088966586 | 0.036729262 |
| hspg2 | 13531 | PDI_U | 0.001876034174 | 0.0008984470387 | 2.088085433 | 0.03680879673 |
| tcl1a | 13538 | PDI_U | -0.002049956973 | 0.0009839374626 | -2.083422017 | 0.03723150339 |
| gpd1 | 11730 | PDI_U | 0.002003603541 | 0.0009644943597 | 2.077361594 | 0.03778992601 |
| nbl1 | 13699 | PDI_U | 0.001987215082 | 0.0009579132569 | 2.074525086 | 0.03804921635 |
| dlk1 | 13800 | PDI_U | 0.00198213476 | 0.0009560187914 | 2.073321966 | 0.03816085208 |
| il22 | 11730 | PDI_U | -0.002115798852 | 0.001020689323 | -2.072911713 | 0.03820230324 |
| gsr | 11709 | PDI_U | -0.002162514791 | 0.001043423231 | -2.072519306 | 0.03823889024 |
| rgl2 | 11570 | PDI_U | -0.002231271679 | 0.001079610686 | -2.066737303 | 0.03878113235 |
| ment | 11537 | PDI_U | -0.002059070925 | 0.0009977191732 | -2.063778045 | 0.03906110004 |
| prap1 | 11790 | PDI_U | 0.001851922649 | 0.0008973530033 | 2.063761577 | 0.03906218271 |
| calcb | 11767 | PDI_U | -0.002173767325 | 0.001054896122 | -2.060645858 | 0.03935879024 |
| ceacam18 | 11570 | PDI_U | -0.002223150555 | 0.001079453227 | -2.05951541 | 0.03946723627 |
| cdon | 13677 | PDI_U | -0.002011446784 | 0.0009795852355 | -2.053365762 | 0.04005610875 |
| il12b | 13801 | PDI_U | 0.001945767528 | 0.0009483199009 | 2.051805015 | 0.04020745935 |
| clec1b | 13784 | PDI_U | 0.002025475608 | 0.0009884345965 | 2.049175146 | 0.0404638968 |
| treh | 11790 | PDI_U | 0.002131096068 | 0.001040009169 | 2.049112768 | 0.04047319641 |
| nid1 | 13639 | PDI_U | 0.001989048155 | 0.0009708972764 | 2.048670032 | 0.04051350539 |
| agrp | 13801 | PDI_U | 0.001980651365 | 0.0009681120516 | 2.045890619 | 0.04078606332 |
| defa1_defa1b | 13351 | PDI_U | 0.002020506962 | 0.000990434651 | 2.040020469 | 0.04136794339 |
| adgrv1 | 11383 | PDI_U | -0.002220859254 | 0.001089807803 | -2.037844882 | 0.04158854479 |
| clec4d | 13621 | PDI_U | 0.001951279853 | 0.0009576345802 | 2.037603793 | 0.04160887405 |
| adgrf5 | 11781 | PDI_U | 0.00211199759 | 0.001039389043 | 2.031960607 | 0.04218005845 |
| flt3lg | 13801 | PDI_U | -0.001917014088 | 0.0009456736155 | -2.027141348 | 0.04266715866 |
| ung | 11730 | PDI_U | -0.002169961283 | 0.001071856322 | -2.024488952 | 0.04294250457 |
| islr2 | 13134 | PDI_U | -0.002046308138 | 0.00101305932 | -2.019929235 | 0.04341101076 |
| clic5 | 11483 | PDI_U | -0.002183807988 | 0.001081528981 | -2.019185825 | 0.0434911235 |
| igsf8 | 13618 | PDI_U | 0.00197166835 | 0.0009786287978 | 2.014725455 | 0.0439530585 |
| pfdn6 | 11483 | PDI_U | -0.002183771871 | 0.001083965502 | -2.014613811 | 0.04396842079 |
| prss2 | 13689 | PDI_U | 0.001991173678 | 0.0009902021138 | 2.010876012 | 0.04435814184 |
| bche | 11548 | PDI_U | -0.002063687113 | 0.001029666296 | -2.004229061 | 0.04506886149 |
| pdcl2 | 11304 | PDI_U | -0.002189709687 | 0.001094025444 | -2.001516235 | 0.04536066827 |
| sirt5 | 13320 | PDI_U | -0.002020908881 | 0.001012745115 | -1.995476306 | 0.04601130569 |
| cxcl6 | 13801 | PDI_U | 0.001956200124 | 0.0009844646264 | 1.987069999 | 0.04693439401 |
| tnfrsf10b | 13552 | PDI_U | -0.0017462948 | 0.0008794659031 | -1.985631045 | 0.04709444341 |
| gsta1 | 13593 | PDI_U | 0.001782170791 | 0.000899952415 | 1.98029447 | 0.04769059811 |
| nop56 | 11355 | PDI_U | -0.002154248733 | 0.00108921809 | -1.977793752 | 0.04797614276 |
| epha10 | 13320 | PDI_U | 0.001997528096 | 0.001013117419 | 1.971664942 | 0.04866854807 |
| lysmd3 | 11483 | PDI_U | 0.002130921414 | 0.001081235339 | 1.970821094 | 0.04876834794 |
| ighmbp2 | 11355 | PDI_U | -0.002145996807 | 0.001089276117 | -1.970112787 | 0.04884973284 |
| mmp15 | 11781 | PDI_U | -0.002099761305 | 0.001067547097 | -1.966902734 | 0.04921788226 |
| cntn1 | 13800 | PDI_U | -0.001793312178 | 0.000913147751 | -1.963879533 | 0.04956412528 |
| dscam | 11757 | PDI_U | 0.002069395061 | 0.001055257983 | 1.961032368 | 0.04989884148 |
| penk | 11537 | PDI_U | 0.002067166719 | 0.001055380673 | 1.958692983 | 0.05017283335 |
| dpt | 13800 | PDI_U | 0.001683828555 | 0.0008625706114 | 1.952105176 | 0.05094593124 |
| tex33 | 11570 | PDI_U | -0.002104710719 | 0.001079090358 | -1.95044901 | 0.05114676255 |
| plaur | 13557 | PDI_U | -0.00180539584 | 0.000925688011 | -1.950328641 | 0.05115755897 |

| Protein | N_used | term | beta | se | t | p_val |
|---|---|---|---|---|---|---|
| acadm | 11570 | PDI_U | -0.002103875152 | 0.001078877283 | -1.950059738 | 0.05119314252 |
| meltf | 11781 | PDI_U | -0.002043861159 | 0.001049441139 | -1.947571029 | 0.05149006026 |
| serpina6 | 11817 | PDI_U | -0.001842415808 | 0.0009472007295 | -1.945116543 | 0.05178466403 |
| ssc5d | 13689 | PDI_U | 0.00181213988 | 0.0009326591826 | 1.942981867 | 0.05203884146 |
| ccn2 | 13644 | PDI_U | 0.001929483628 | 0.0009933811527 | 1.942339677 | 0.05211656069 |
| vegfc | 13699 | PDI_U | 0.001923167572 | 0.0009926285995 | 1.937449287 | 0.05271099327 |
| morn4 | 11304 | PDI_U | -0.002119198032 | 0.001093880222 | -1.937321829 | 0.05273092189 |
| tmem25 | 11757 | PDI_U | -0.002052336745 | 0.001060206448 | -1.935789722 | 0.05291742822 |
| erbb4 | 13699 | PDI_U | -0.00184904084 | 0.0009557804666 | -1.934587391 | 0.05306153054 |
| st6gal1 | 13800 | PDI_U | -0.001840399506 | 0.0009521559516 | -1.932876125 | 0.05327191199 |
| notch3 | 13800 | PDI_U | -0.001797231606 | 0.0009298621831 | -1.932793524 | 0.05328209172 |
| asgr1 | 13552 | PDI_U | 0.001731096153 | 0.0008961641499 | 1.931673069 | 0.05342071275 |
| col3a1 | 11790 | PDI_U | -0.002040418217 | 0.001056839721 | -1.930678964 | 0.05354674007 |
| lama1 | 11355 | PDI_U | -0.002096181514 | 0.001087868789 | -1.926869798 | 0.05402080805 |
| myl3 | 11474 | PDI_U | -0.001923395049 | 0.0009983386119 | -1.926595873 | 0.05405470821 |
| lrtm2 | 11757 | PDI_U | 0.001911741449 | 0.0009924261836 | 1.926331128 | 0.05408714457 |
| arg2 | 11730 | PDI_U | -0.002056248651 | 0.00106776994 | -1.92574128 | 0.05416085419 |
| cdh1 | 13181 | PDI_U | -0.001910225419 | 0.000993112548 | -1.923473249 | 0.05444217614 |
| adgrg1 | 13579 | PDI_U | 0.001770392908 | 0.0009220610566 | 1.920038695 | 0.05487395516 |
| gsta3 | 13308 | PDI_U | 0.001795495828 | 0.0009353043251 | 1.919691569 | 0.05491824427 |
| itih4 | 11817 | PDI_U | -0.001916696911 | 0.0009993521819 | -1.917939386 | 0.0551427959 |
| spp1 | 13777 | PDI_U | 0.001844467027 | 0.0009626172747 | 1.916095914 | 0.05537357734 |
| c1qbp | 11757 | PDI_U | -0.002044346446 | 0.001069836672 | -1.910895841 | 0.05604228701 |
| lcat | 11794 | PDI_U | 0.001919345621 | 0.00100658185 | 1.906795379 | 0.05657138892 |
| ntf3 | 13621 | PDI_U | -0.001886602344 | 0.0009911555718 | -1.903437158 | 0.05700460253 |
| lrp11 | 13618 | PDI_U | 0.001828636444 | 0.0009613767288 | 1.902101839 | 0.05717893843 |
| il18r1 | 13801 | PDI_U | 0.001753454471 | 0.0009252416812 | 1.895131301 | 0.0580959047 |
| ipcef1 | 13526 | PDI_U | 0.001876477593 | 0.0009920324811 | 1.891548542 | 0.05857251993 |
| serpinf1 | 11620 | PDI_U | 0.001758055384 | 0.0009295584838 | 1.891280016 | 0.05861184859 |
| ca12 | 13579 | PDI_U | -0.001876759584 | 0.0009939470706 | -1.888188656 | 0.0590219453 |
| slitrk2 | 13753 | PDI_U | -0.001778328713 | 0.0009427613633 | -1.886297829 | 0.05927589996 |
| rac3 | 11483 | PDI_U | -0.002040498169 | 0.001082990957 | -1.884132233 | 0.05957235765 |
| skiv2l | 11355 | PDI_U | -0.002050530561 | 0.001089793191 | -1.881577696 | 0.05991896253 |
| rbp5 | 13579 | PDI_U | 0.001679885504 | 0.0008934755583 | 1.880169512 | 0.06010638643 |
| osm | 13551 | PDI_U | 0.001813257749 | 0.0009654620553 | 1.878124302 | 0.06038563662 |
| cntnap4 | 11483 | PDI_U | 0.002028122381 | 0.001081638729 | 1.875046008 | 0.06081177991 |
| kir2dl3 | 13308 | PDI_U | -0.001874272048 | 0.001001157961 | -1.872104225 | 0.06121411959 |
| cd58 | 13643 | PDI_U | -0.001850838946 | 0.0009909103613 | -1.867816725 | 0.06180906251 |
| il12rb2 | 11411 | PDI_U | 0.002018138611 | 0.00108070007 | 1.867436365 | 0.06186631982 |
| was | 13378 | PDI_U | -0.001884978026 | 0.001009705185 | -1.866859807 | 0.06194304471 |
| susd5 | 11459 | PDI_U | 0.001806672707 | 0.0009680232208 | 1.866352654 | 0.06201759318 |
| cst6 | 13287 | PDI_U | 0.001804989593 | 0.0009674080155 | 1.865799708 | 0.06209143142 |
| eci2 | 11355 | PDI_U | -0.002024381135 | 0.001086660061 | -1.862938749 | 0.06249671277 |
| angpt1 | 13637 | PDI_U | 0.001856752397 | 0.0009971203878 | 1.862114565 | 0.06260845468 |
| vsig10l | 11355 | PDI_U | -0.00201678606 | 0.001083918046 | -1.860644416 | 0.06282026576 |
| prtn3 | 13287 | PDI_U | 0.001851430538 | 0.000996292345 | 1.85832055 | 0.06314562669 |
| ly9 | 13483 | PDI_U | -0.001841338891 | 0.000991397879 | -1.857315746 | 0.06328805373 |
| sod2 | 13632 | PDI_U | -0.001803131123 | 0.0009730839505 | -1.853006745 | 0.06390300625 |
| sdc4 | 13618 | PDI_U | 0.001850750308 | 0.0009991654705 | 1.852296104 | 0.06400495876 |
| kazald1 | 13579 | PDI_U | 0.001824017369 | 0.0009849789987 | 1.851833766 | 0.06407140814 |
| anpep | 13671 | PDI_U | -0.001837015182 | 0.0009921828999 | -1.851488452 | 0.06412088268 |
| hrc | 11474 | PDI_U | 0.001982511125 | 0.001071302769 | 1.850561 | 0.06425844839 |
| tdp1 | 11730 | PDI_U | 0.001944137086 | 0.001051807239 | 1.848377738 | 0.06457289477 |
| ltbr | 13594 | PDI_U | 0.001783611772 | 0.0009667373461 | 1.844980727 | 0.06506209913 |
| dsc2 | 13475 | PDI_U | -0.001759100152 | 0.0009562618374 | -1.839559086 | 0.06585499981 |
| selp | 13689 | PDI_U | 0.001817656683 | 0.0009883843403 | 1.839018091 | 0.06593419036 |
| amigo2 | 13579 | PDI_U | -0.00182134697 | 0.0009908987864 | -1.838075689 | 0.06607310316 |
| lag3 | 13579 | PDI_U | -0.001772800037 | 0.00096651507 | -1.834218723 | 0.06664342157 |
| esr1 | 11411 | PDI_U | -0.001984170329 | 0.001084674627 | -1.829276983 | 0.06738423351 |
| bmp4 | 13526 | PDI_U | -0.001812924427 | 0.0009914569454 | -1.828545794 | 0.06748970899 |
| anxa5 | 13286 | PDI_U | 0.001829780191 | 0.001005397963 | 1.819956136 | 0.06878819643 |
| hhex | 11767 | PDI_U | 0.001899427072 | 0.001043839517 | 1.819654306 | 0.06883708964 |
| prame | 11304 | PDI_U | -0.001989907247 | 0.001094569787 | -1.817981157 | 0.06909349771 |
| mybpc1 | 11781 | PDI_U | -0.001768065263 | 0.0009736212639 | -1.815968209 | 0.06940068193 |
| igfbp3 | 13689 | PDI_U | 0.001737672138 | 0.0009585245683 | 1.812861345 | 0.06987512298 |
| got1 | 11790 | PDI_U | -0.001883905469 | 0.001039462012 | -1.812385105 | 0.06995216426 |
| vcan | 13784 | PDI_U | -0.001746479669 | 0.0009637710921 | -1.812131203 | 0.06998769483 |
| cxcl13 | 13784 | PDI_U | 0.00176712939 | 0.0009761417251 | 1.810320515 | 0.07026788261 |
| lipf | 11474 | PDI_U | -0.001910187763 | 0.001055851292 | -1.809144694 | 0.07045471533 |
| chgb | 13750 | PDI_U | 0.001702971197 | 0.000942005851 | 1.807813821 | 0.07065734341 |

| Protein | N_used | term | beta | se | t | p_val |
|---|---|---|---|---|---|---|
| acox1 | 13618 | PDI_U | -0.001805097417 | 0.001000635698 | -1.803950649 | 0.07126115198 |
| erp44 | 13561 | PDI_U | 0.001769188336 | 0.0009839593612 | 1.798029884 | 0.07219452543 |
| hmox1 | 13461 | PDI_U | 0.001766797051 | 0.0009828181928 | 1.797684519 | 0.07224943777 |
| serpine1 | 13800 | PDI_U | 0.001750132083 | 0.0009753073537 | 1.794441595 | 0.07276461103 |
| cd226 | 11411 | PDI_U | 0.001941927565 | 0.001084757862 | 1.790194507 | 0.07344917862 |
| thrap3 | 11355 | PDI_U | 0.001947589123 | 0.001087966174 | 1.790119187 | 0.07346141543 |
| f12 | 11817 | PDI_U | -0.001888156507 | 0.001056138247 | -1.787792945 | 0.07383506103 |
| prkag3 | 11483 | PDI_U | -0.001931155716 | 0.001083307246 | -1.782648204 | 0.07467002641 |
| efemp1 | 13663 | PDI_U | -0.001586102515 | 0.0008923107546 | -1.77752258 | 0.0755045572 |
| klk12 | 13308 | PDI_U | -0.001793426993 | 0.001009506146 | -1.776538955 | 0.07566698758 |
| ccdc50 | 11757 | PDI_U | -0.001898123495 | 0.001068845232 | -1.775863744 | 0.07578126364 |
| spint2 | 13710 | PDI_U | 0.001736463885 | 0.0009801898421 | 1.771558743 | 0.07649006211 |
| tslp | 13388 | PDI_U | -0.001787435357 | 0.001010311091 | -1.769193047 | 0.07688444686 |
| cpped1 | 13784 | PDI_U | 0.001721626068 | 0.0009741542422 | 1.767303362 | 0.07719957913 |
| btnl10 | 11304 | PDI_U | -0.001934146929 | 0.001094765986 | -1.766721796 | 0.0773018417 |
| reg1b | 13800 | PDI_U | -0.001712640244 | 0.0009700978798 | -1.765430355 | 0.07751359614 |
| nfasc | 13801 | PDI_U | 0.001630730361 | 0.0009241522295 | 1.764568985 | 0.07765836731 |
| fabp4 | 13800 | PDI_U | 0.001284071441 | 0.0007280424629 | 1.763731522 | 0.07779933476 |
| thbd | 13618 | PDI_U | 0.001682016677 | 0.0009545520826 | 1.762100474 | 0.07807477509 |
| arsa | 13632 | PDI_U | 0.001694444691 | 0.0009617608856 | 1.761814934 | 0.07812300165 |
| slc1a4 | 11483 | PDI_U | -0.001907331305 | 0.001083112224 | -1.760972929 | 0.07826962573 |
| gamt | 11355 | PDI_U | -0.001915828979 | 0.001089452543 | -1.758524491 | 0.07868527153 |
| mep1a | 11724 | PDI_U | -0.00180269483 | 0.00102567859 | -1.757563089 | 0.07884800049 |
| ift20 | 11483 | PDI_U | -0.001902075788 | 0.0010827918 | -1.75664037 | 0.07900580637 |
| gal | 13710 | PDI_U | 0.001663537837 | 0.00094806888 | 1.754659258 | 0.07933996481 |
| igfbpl1 | 13618 | PDI_U | 0.001622105483 | 0.0009246913123 | 1.754212959 | 0.07941653384 |
| prcp | 13712 | PDI_U | 0.001589842719 | 0.0009065096799 | 1.753806665 | 0.07948599943 |
| gask1a | 11355 | PDI_U | 0.001865212997 | 0.001063698306 | 1.753516939 | 0.07954032191 |
| sat1 | 11355 | PDI_U | -0.001904106095 | 0.001087433533 | -1.751009175 | 0.07997135866 |
| il2ra | 13531 | PDI_U | -0.001703081845 | 0.0009744202059 | -1.747789953 | 0.08052310738 |
| egfr | 13800 | PDI_U | -0.001697603204 | 0.0009714346791 | -1.747521723 | 0.08056914069 |
| mst1 | 11593 | PDI_U | -0.001872923659 | 0.001072184724 | -1.746829271 | 0.08069346602 |
| myoc | 13632 | PDI_U | -0.001690481765 | 0.000968220929 | -1.745966973 | 0.080839237 |
| ncs1 | 13753 | PDI_U | -0.001577583762 | 0.0009037178194 | -1.745659683 | 0.08089245443 |
| igsf3 | 13579 | PDI_U | 0.00160168259 | 0.00091907553 | 1.742710515 | 0.0814068509 |
| scarf2 | 13784 | PDI_U | -0.001508028555 | 0.0008669829468 | -1.739398174 | 0.08198709116 |
| pcdhb15 | 11730 | PDI_U | -0.001853933278 | 0.001068139131 | -1.735666472 | 0.08264911243 |
| psmc3 | 11304 | PDI_U | -0.001894128405 | 0.001092924803 | -1.733082092 | 0.08310837855 |
| scn4b | 11474 | PDI_U | 0.001811363226 | 0.001045615357 | 1.732341835 | 0.08323961805 |
| lefty2 | 13732 | PDI_U | 0.001618069863 | 0.0009342024465 | 1.732033425 | 0.0832900867 |
| sfrp1 | 13504 | PDI_U | 0.001603129238 | 0.0009263261934 | 1.730631444 | 0.08354038405 |
| slc12a2 | 11355 | PDI_U | -0.001884597407 | 0.001089352926 | -1.730015463 | 0.08365471358 |
| il1rl2 | 13378 | PDI_U | 0.001665994441 | 0.0009631386492 | 1.729755568 | 0.08369704174 |
| s100a13 | 11411 | PDI_U | 0.001796195654 | 0.001041363798 | 1.724849335 | 0.08458173485 |
| dctpp1 | 13618 | PDI_U | -0.001676449617 | 0.0009724088492 | -1.724017237 | 0.08472744656 |
| capn3 | 11730 | PDI_U | -0.001837021022 | 0.001066285044 | -1.722823585 | 0.0849468159 |
| dgkz | 13378 | PDI_U | -0.001738481776 | 0.00100957029 | -1.722001721 | 0.0850923424 |
| ifnw1 | 11355 | PDI_U | -0.00187653761 | 0.001089914407 | -1.721729338 | 0.08514582405 |
| c19orf12 | 13320 | PDI_U | 0.001643112269 | 0.0009575983605 | 1.71586788 | 0.0862095014 |
| il31ra | 11368 | PDI_U | -0.001859705048 | 0.001085080292 | -1.713887038 | 0.08657676355 |
| grk5 | 13388 | PDI_U | -0.001727315143 | 0.001010308814 | -1.709690264 | 0.08734635741 |
| wfdc12 | 13735 | PDI_U | -0.001520343749 | 0.0008895276647 | -1.709158477 | 0.08744420786 |
| stoml2 | 11304 | PDI_U | -0.001867274534 | 0.001093809933 | -1.707128888 | 0.08782559024 |
| lair2 | 13552 | PDI_U | 0.001691796583 | 0.0009941144498 | 1.701812687 | 0.08881344434 |
| sbsn | 11765 | PDI_U | -0.001799240306 | 0.001060210064 | -1.697060202 | 0.08971175675 |
| csf3 | 13378 | PDI_U | -0.00165411508 | 0.0009753263403 | -1.695960636 | 0.08991662304 |
| tigit | 11570 | PDI_U | -0.001823391768 | 0.001075507326 | -1.695378286 | 0.09003061687 |
| ccne1 | 11411 | PDI_U | 0.001837488765 | 0.001084722766 | 1.693970868 | 0.090298138 |
| cdhr1 | 13320 | PDI_U | -0.001689436387 | 0.0009973406752 | -1.693941126 | 0.0902998713 |
| tpt1 | 13378 | PDI_U | -0.001705403253 | 0.001009123654 | -1.68998442 | 0.09105425489 |
| plxna4 | 13621 | PDI_U | 0.001684505285 | 0.0009977571146 | 1.68829193 | 0.09137811524 |
| fhip2a | 11355 | PDI_U | -0.00183896112 | 0.001089804626 | -1.687422751 | 0.09154958612 |
| rgs8 | 13378 | PDI_U | -0.001704711729 | 0.001010784394 | -1.686523594 | 0.09171833183 |
| fgf5 | 13378 | PDI_U | -0.001573025258 | 0.0009348954132 | -1.682568163 | 0.09248207602 |
| tprkb | 11304 | PDI_U | -0.001842088301 | 0.00109482289 | -1.682544563 | 0.09249093808 |
| f2r | 13691 | PDI_U | 0.001672507255 | 0.0009949795484 | 1.680946365 | 0.09279616311 |
| bmp10 | 11537 | PDI_U | -0.001687545895 | 0.001004618387 | -1.679787984 | 0.09302567728 |
| tfrc | 13694 | PDI_U | -0.001641538708 | 0.0009780694885 | -1.678345687 | 0.09330247113 |
| sh3gl3 | 11493 | PDI_U | -0.001814119104 | 0.001083031997 | -1.675037403 | 0.0939541267 |
| ctsh | 13346 | PDI_U | 0.001652682055 | 0.0009897689305 | 1.669765543 | 0.09498923303 |

| Protein | N_used | term | beta | se | t | p_val |
|---|---|---|---|---|---|---|
| efcab14 | 11724 | PDI_U | 0.001705815023 | 0.001022166358 | 1.668823288 | 0.09517913424 |
| cd274 | 13552 | PDI_U | 0.001624458496 | 0.0009761836436 | 1.664091082 | 0.09611736885 |
| pqbp1 | 13308 | PDI_U | 0.001661560489 | 0.001002535996 | 1.657357437 | 0.09747082353 |
| calb2 | 13320 | PDI_U | 0.001535148418 | 0.0009268130961 | 1.656373248 | 0.09766983011 |
| pdgfb | 13710 | PDI_U | 0.001644282337 | 0.0009928568088 | 1.656112264 | 0.09772199089 |
| dll1 | 13699 | PDI_U | -0.001565282086 | 0.0009501702615 | -1.647370107 | 0.099504951 |
| dda1 | 11355 | PDI_U | 0.001793080026 | 0.001090226235 | 1.644686183 | 0.1000622537 |
| gtf2ird1 | 11570 | PDI_U | -0.001774120799 | 0.001079479576 | -1.643496402 | 0.1003074686 |
| cdk5rap3 | 11757 | PDI_U | -0.0017538637 | 0.001067238653 | -1.643365985 | 0.1003340003 |
| nfkbie | 13308 | PDI_U | 0.001646095726 | 0.001003060488 | 1.64107324 | 0.1008058651 |
| npdc1 | 13618 | PDI_U | 0.001553131843 | 0.0009466822804 | 1.640605169 | 0.1009025163 |
| ulbp2 | 13552 | PDI_U | -0.001586575938 | 0.0009674413736 | -1.639971146 | 0.1010343963 |
| klb | 13320 | PDI_U | -0.00164225426 | 0.001001635748 | -1.639572334 | 0.1011177554 |
| itgb7 | 13753 | PDI_U | -0.001621146247 | 0.0009894345538 | -1.638457279 | 0.1013492337 |
| csf2rb | 11411 | PDI_U | -0.00176455688 | 0.001077513386 | -1.637619452 | 0.1015287046 |
| cd48 | 13691 | PDI_U | -0.001609953798 | 0.0009832914594 | -1.637310873 | 0.1015885324 |
| myo6 | 11757 | PDI_U | -0.00175193286 | 0.001070043496 | -1.637253875 | 0.1016042243 |
| tnfrsf9 | 13552 | PDI_U | -0.001552363043 | 0.0009503601109 | -1.63344718 | 0.1023982314 |
| lyve1 | 11817 | PDI_U | -0.001699736985 | 0.001041332236 | -1.632271551 | 0.102648965 |
| efhd1 | 11757 | PDI_U | 0.001624033085 | 0.0009956994869 | 1.631047426 | 0.1029071246 |
| atp1b4 | 11304 | PDI_U | -0.001782000247 | 0.001094060655 | -1.628794746 | 0.1033843727 |
| asah2 | 13552 | PDI_U | 0.001563276751 | 0.0009598335283 | 1.628695712 | 0.1034007154 |
| apoh | 13632 | PDI_U | 0.001611646203 | 0.0009902677128 | 1.627485358 | 0.1036571869 |
| kirrel1 | 11383 | PDI_U | -0.001771260776 | 0.001089303669 | -1.626048663 | 0.1039670144 |
| folr3 | 13660 | PDI_U | -0.001583285077 | 0.0009738295627 | -1.62583386 | 0.1040080914 |
| clec14a | 13632 | PDI_U | -0.001550424664 | 0.0009556270815 | -1.622415996 | 0.1047374511 |
| sparc | 13699 | PDI_U | 0.001610932808 | 0.0009934305499 | 1.621585734 | 0.1049151128 |
| lilra2 | 13632 | PDI_U | -0.001535919184 | 0.0009474516993 | -1.621105525 | 0.1050181575 |
| lrrc38 | 11483 | PDI_U | 0.001700754689 | 0.001049230089 | 1.620954934 | 0.1050547893 |
| ranbp2 | 11383 | PDI_U | -0.001761154049 | 0.001088051048 | -1.618631821 | 0.105554238 |
| coch | 11781 | PDI_U | 0.001675575855 | 0.001036263239 | 1.616940361 | 0.1059179559 |
| kir2dl2 | 11730 | PDI_U | -0.001712576843 | 0.001059215371 | -1.616835339 | 0.1059407467 |
| tnfrsf6b | 13552 | PDI_U | -0.001526054463 | 0.0009442770333 | -1.616108842 | 0.1060940802 |
| gp5 | 11537 | PDI_U | 0.001745583069 | 0.001081168294 | 1.614534091 | 0.1064390034 |
| septin8 | 11755 | PDI_U | -0.001630184552 | 0.001009955006 | -1.614116018 | 0.1065291315 |
| mcts1 | 11724 | PDI_U | -0.001725774201 | 0.001069421532 | -1.613745515 | 0.1066095769 |
| mcam | 13689 | PDI_U | -0.001532497802 | 0.0009504786768 | -1.61234317 | 0.1069103568 |
| brd2 | 11383 | PDI_U | -0.001757904475 | 0.001090268909 | -1.612358622 | 0.1069116752 |
| arntl | 11355 | PDI_U | -0.001750157818 | 0.001089298869 | -1.606682856 | 0.1081517908 |
| tg | 11730 | PDI_U | 0.001691878013 | 0.001053457049 | 1.606024674 | 0.1082954341 |
| rln1 | 11757 | PDI_U | 0.001702874168 | 0.001062423438 | 1.602820595 | 0.109001159 |
| micb_mica | 13621 | PDI_U | -0.001585289238 | 0.0009894600125 | -1.602176155 | 0.1091398701 |
| gpihbp1 | 11724 | PDI_U | -0.00171094296 | 0.001067963032 | -1.602061972 | 0.1091688741 |
| mfap4 | 11537 | PDI_U | 0.00166787287 | 0.001041426179 | 1.601527697 | 0.1092874971 |
| tp53inp1 | 13388 | PDI_U | -0.001610202641 | 0.001007908991 | -1.597567494 | 0.1101628867 |
| gkn1 | 13075 | PDI_U | -0.001608313917 | 0.001009106438 | -1.593800075 | 0.1110050142 |
| sumf1 | 11411 | PDI_U | -0.001728002001 | 0.0010860111 | -1.5911458 | 0.1116044971 |
| serpina3 | 11772 | PDI_U | -0.001674603352 | 0.00105314456 | -1.590098278 | 0.1118395342 |
| esm1 | 13658 | PDI_U | 0.001523873996 | 0.0009600726436 | 1.587248638 | 0.1124795119 |
| chchd6 | 11730 | PDI_U | -0.001652959868 | 0.001041712056 | -1.586772332 | 0.112591199 |
| dkk1 | 13784 | PDI_U | 0.001565295392 | 0.0009867741611 | 1.586275212 | 0.112699851 |
| adamtsl4 | 11474 | PDI_U | -0.001664658514 | 0.001050914595 | -1.58400932 | 0.1132191865 |
| hla_a | 11757 | PDI_U | 0.001686432789 | 0.001066054423 | 1.581938739 | 0.1136904824 |
| nmrk2 | 11304 | PDI_U | -0.001727087588 | 0.001091850236 | -1.581798978 | 0.1137234751 |
| adm | 13753 | PDI_U | 0.001281869182 | 0.0008114630679 | 1.5797012 | 0.114198328 |
| mapre3 | 11304 | PDI_U | 0.001722993808 | 0.001091792618 | 1.578132861 | 0.1145631012 |
| mzt1 | 13308 | PDI_U | 0.001591013289 | 0.001012206413 | 1.571826921 | 0.1160144908 |
| enpp2 | 13800 | PDI_U | -0.001260195449 | 0.0008017434017 | -1.571818922 | 0.1160154974 |
| scp2 | 13308 | PDI_U | -0.001582024814 | 0.001008081486 | -1.569342196 | 0.1165920211 |
| il17ra | 13632 | PDI_U | -0.001565295745 | 0.0009977062313 | -1.568894426 | 0.1166957712 |
| cxcl17 | 13801 | PDI_U | -0.001284849706 | 0.0008193589032 | -1.568115878 | 0.1168770377 |
| nectin4 | 13699 | PDI_U | -0.001529639671 | 0.0009766788146 | -1.566164483 | 0.1173332332 |
| cd6 | 13779 | PDI_U | 0.001506714981 | 0.0009633170181 | 1.564090484 | 0.117819305 |
| selenop | 11817 | PDI_U | -0.001650856561 | 0.001055653852 | -1.563823745 | 0.117885771 |
| nmnat1 | 13552 | PDI_U | 0.00152048676 | 0.000974298031 | 1.560597181 | 0.1186421955 |
| ltbp2 | 13800 | PDI_U | -0.001307018539 | 0.0008387420835 | -1.558308048 | 0.1191831932 |
| timm10 | 11781 | PDI_U | 0.001660751004 | 0.001066496503 | 1.557202485 | 0.1194493073 |
| ppbp | 11717 | PDI_U | 0.001666346171 | 0.001070621285 | 1.556429144 | 0.1196331152 |
| egf | 13633 | PDI_U | 0.00155417543 | 0.0009987740031 | 1.556083183 | 0.1197115462 |
| gad1 | 11304 | PDI_U | -0.001699926863 | 0.001093626735 | -1.554394026 | 0.1201184821 |

| Protein | N_used | term | beta | se | t | p_val |
|---|---|---|---|---|---|---|
| rtbdn | 13579 | PDI_U | 0.001515131465 | 0.0009755532598 | 1.55309969 | 0.1204226456 |
| nudt10 | 11474 | PDI_U | -0.001684584684 | 0.001085092991 | -1.552479554 | 0.1205751301 |
| reg3g | 11730 | PDI_U | -0.001647698457 | 0.001061924202 | -1.551615882 | 0.1207811666 |
| nxpe4 | 11355 | PDI_U | -0.001690413366 | 0.001089959638 | -1.550895379 | 0.1209546566 |
| kcnh2 | 11483 | PDI_U | -0.001677628077 | 0.001083563312 | -1.548251088 | 0.1215894423 |
| cr1 | 11737 | PDI_U | -0.001605850934 | 0.001037635091 | -1.547606618 | 0.121744026 |
| cd84 | 13621 | PDI_U | 0.001544659361 | 0.0009983535794 | 1.547206714 | 0.1218366602 |
| s100a14 | 11757 | PDI_U | 0.001649505017 | 0.001066900451 | 1.546072096 | 0.1221140994 |
| ptx3 | 13621 | PDI_U | -0.001537651631 | 0.0009950561654 | -1.545291296 | 0.122299072 |
| adamts4 | 11411 | PDI_U | 0.001633762467 | 0.001057433073 | 1.545026828 | 0.1223675341 |
| cenpj | 11570 | PDI_U | -0.001667526298 | 0.001079504713 | -1.544714236 | 0.1224427777 |
| grin2b | 11383 | PDI_U | -0.001680382768 | 0.001088900422 | -1.543192319 | 0.1228119479 |
| gpkow | 13320 | PDI_U | 0.001524285741 | 0.0009880706763 | 1.542688977 | 0.1229300346 |
| stx5 | 11411 | PDI_U | 0.001674564493 | 0.001085556619 | 1.542586046 | 0.1229590046 |
| eng | 13800 | PDI_U | -0.00151310874 | 0.0009809348874 | -1.542517 | 0.1229709593 |
| mocs2 | 11537 | PDI_U | 0.00165379472 | 0.001073221422 | 1.540963204 | 0.1233531961 |
| osbpl2 | 11767 | PDI_U | -0.001643559599 | 0.001068489847 | -1.538207971 | 0.1240246882 |
| mln | 13621 | PDI_U | 0.001428836927 | 0.0009302323211 | 1.536000088 | 0.1245616061 |
| amdhd2 | 11790 | PDI_U | -0.001638769919 | 0.001069151487 | -1.532776168 | 0.1253578731 |
| gucy2c | 11570 | PDI_U | -0.001648975135 | 0.001076722535 | -1.531476385 | 0.125679067 |
| birc2 | 13308 | PDI_U | 0.001550300758 | 0.00101232189 | 1.531430638 | 0.1256867877 |
| angptl4 | 13691 | PDI_U | -0.00145177862 | 0.0009525741316 | -1.524058413 | 0.1275172374 |
| cntf | 11383 | PDI_U | -0.001659948439 | 0.001090200683 | -1.522608145 | 0.1278845785 |
| cfi | 11817 | PDI_U | -0.001440062699 | 0.000945967775 | -1.522316866 | 0.1279564888 |
| apex1 | 13705 | PDI_U | 0.001480119916 | 0.0009723485949 | 1.522211194 | 0.1279792628 |
| susd1 | 13388 | PDI_U | 0.001531879055 | 0.001008350487 | 1.519193053 | 0.1287375444 |
| neb | 11355 | PDI_U | -0.001638473734 | 0.001080026948 | -1.517067456 | 0.1292775213 |
| pfdn4 | 11304 | PDI_U | -0.001659576293 | 0.001094046635 | -1.516915495 | 0.1293160139 |
| sftpa2 | 13308 | PDI_U | -0.001503774385 | 0.0009921165538 | -1.51572351 | 0.1296130525 |
| ptprk | 11724 | PDI_U | -0.001603601518 | 0.001058062416 | -1.515602004 | 0.129647007 |
| hjv | 11757 | PDI_U | 0.00154392791 | 0.001018943653 | 1.515224033 | 0.1297425895 |
| fam171b | 11730 | PDI_U | -0.001609380026 | 0.001063621406 | -1.51311361 | 0.1302777719 |
| ctrc | 13658 | PDI_U | 0.001488104514 | 0.000983903888 | 1.512449063 | 0.1304428189 |
| rfc4 | 11730 | PDI_U | -0.001613605498 | 0.001068293102 | -1.510452043 | 0.1309550814 |
| cep164 | 13378 | PDI_U | -0.00152546905 | 0.001010347943 | -1.509845258 | 0.1311065517 |
| glb1 | 13552 | PDI_U | 0.001449754898 | 0.000960605759 | 1.509209043 | 0.1312687034 |
| defb4a_defb4b | 13447 | PDI_U | 0.001406240053 | 0.0009379459851 | 1.499276159 | 0.1338255011 |
| sowaha | 11483 | PDI_U | -0.00162394486 | 0.001084488238 | -1.497429666 | 0.1343090085 |
| chrdl2 | 13618 | PDI_U | 0.001456440635 | 0.0009764157195 | 1.491619406 | 0.1358221318 |
| dipk1c | 11483 | PDI_U | -0.001614618821 | 0.001083351108 | -1.49039292 | 0.1361484483 |
| m6pr | 11474 | PDI_U | 0.00161561109 | 0.001084848718 | 1.489250126 | 0.1364490289 |
| eps8l2 | 13726 | PDI_U | -0.001409382941 | 0.0009468976074 | -1.488421695 | 0.1366627122 |
| il2 | 13378 | PDI_U | -0.001504488157 | 0.001010939055 | -1.488208562 | 0.1367194915 |
| lamp3 | 13779 | PDI_U | -0.001362555737 | 0.0009157253403 | -1.487952421 | 0.1367863454 |
| sort1 | 13599 | PDI_U | 0.001465949153 | 0.0009864228165 | 1.486126566 | 0.1372688489 |
| naa10 | 11355 | PDI_U | -0.001617339485 | 0.001088359648 | -1.48603404 | 0.1372979097 |
| cptp | 11355 | PDI_U | -0.001615941026 | 0.001088063085 | -1.4851538 | 0.1375308765 |
| fap | 13800 | PDI_U | 0.001442244585 | 0.000971757969 | 1.484160286 | 0.1377892618 |
| fgfr4 | 11537 | PDI_U | 0.001595324305 | 0.001075212681 | 1.483729065 | 0.1379081583 |
| slurp1 | 11699 | PDI_U | -0.001545826935 | 0.001042015152 | -1.483497559 | 0.1379692306 |
| smndc1 | 11724 | PDI_U | 0.001540330581 | 0.001038356734 | 1.483431012 | 0.1379868415 |
| crnn | 13204 | PDI_U | -0.001425740626 | 0.000961214339 | -1.483270243 | 0.1380265144 |
| slitrk6 | 13388 | PDI_U | -0.001482470447 | 0.0009996807399 | -1.482943892 | 0.1381128783 |
| ddr1 | 13784 | PDI_U | -0.00144314342 | 0.0009732940763 | -1.482741398 | 0.1381660128 |
| cacna1c | 11383 | PDI_U | -0.001615211093 | 0.001090416911 | -1.481278469 | 0.1385600917 |
| aif1 | 13308 | PDI_U | 0.001490465755 | 0.001007694232 | 1.479085329 | 0.1391412011 |
| il17rb | 13452 | PDI_U | -0.001419513294 | 0.0009598488558 | -1.478892521 | 0.1391924784 |
| dpep2 | 13579 | PDI_U | -0.001468416676 | 0.0009930793538 | -1.478649889 | 0.1392571276 |
| snu13 | 11355 | PDI_U | -0.001606221259 | 0.001087443711 | -1.477061518 | 0.1396869096 |
| ccn1 | 13561 | PDI_U | 0.001442431975 | 0.0009766251687 | 1.476955562 | 0.1397107913 |
| ccn5 | 13632 | PDI_U | 0.001262746129 | 0.0008551414056 | 1.476651839 | 0.1397921078 |
| zbtb17 | 13388 | PDI_U | -0.001454770168 | 0.000985256672 | -1.476539271 | 0.1398227218 |
| adcyap1r1 | 13308 | PDI_U | 0.001494364291 | 0.001012757086 | 1.475540692 | 0.1400909161 |
| cacnb1 | 11383 | PDI_U | -0.001600604389 | 0.001090379849 | -1.467932841 | 0.1421501257 |
| nrtn | 13378 | PDI_U | -0.001476422477 | 0.001007810849 | -1.464979741 | 0.1429499658 |
| icam5 | 13618 | PDI_U | -0.001424978942 | 0.0009753054433 | -1.46105915 | 0.1440223016 |
| tspan15 | 11355 | PDI_U | -0.001590381576 | 0.001088673785 | -1.46084309 | 0.1440862057 |
| arid3a | 11383 | PDI_U | -0.001591346126 | 0.001089473646 | -1.460655915 | 0.1441375221 |
| asah1 | 11673 | PDI_U | -0.001542003922 | 0.001055847161 | -1.460442363 | 0.1441954783 |
| megf9 | 13750 | PDI_U | 0.001413889245 | 0.000970610274 | 1.456701297 | 0.1452217059 |

| Protein | N_used | term | beta | se | t | p_val |
|---|---|---|---|---|---|---|
| slk | 11570 | PDI_U | 0.001573375996 | 0.001080256632 | 1.456483533 | 0.1452861625 |
| clec4m | 11775 | PDI_U | -0.001544719582 | 0.001060662535 | -1.456372344 | 0.1453164066 |
| ogn | 13632 | PDI_U | 0.001318935632 | 0.0009058947501 | 1.45594798 | 0.1454300499 |
| idua | 13753 | PDI_U | 0.001422915827 | 0.000977355338 | 1.455883824 | 0.1454475844 |
| ddx39a | 11411 | PDI_U | 0.001582439905 | 0.001086934238 | 1.45587456 | 0.1454548396 |
| xcl1 | 13753 | PDI_U | 0.001419713316 | 0.0009763392949 | 1.454118791 | 0.1459362141 |
| serpini2 | 11724 | PDI_U | 0.001547848608 | 0.001064743442 | 1.453729177 | 0.1460482001 |
| tgfb2 | 11730 | PDI_U | 0.001556076823 | 0.001070554325 | 1.453524391 | 0.1461049924 |
| fam3c | 13599 | PDI_U | 0.001381431135 | 0.0009507683882 | 1.452962838 | 0.1462571664 |
| guk1 | 11355 | PDI_U | -0.001579919487 | 0.001089254339 | -1.450459668 | 0.146958029 |
| s100a11 | 13514 | PDI_U | 0.001407703087 | 0.0009715407322 | 1.448938825 | 0.1473778906 |
| sirpb1 | 13643 | PDI_U | 0.001406030879 | 0.0009704414075 | 1.448857054 | 0.1474005097 |
| crim1 | 13710 | PDI_U | -0.001389516178 | 0.0009594956882 | -1.448173447 | 0.1475914365 |
| scarf1 | 13800 | PDI_U | 0.001427193994 | 0.000986950028 | 1.446065103 | 0.1481816723 |
| camkk1 | 13308 | PDI_U | 0.001456994699 | 0.001008163919 | 1.445196234 | 0.148426341 |
| fbp1 | 13618 | PDI_U | 0.001384897096 | 0.0009583681345 | 1.445057537 | 0.1484647547 |
| dcc | 11483 | PDI_U | 0.001536865312 | 0.001063992874 | 1.44443196 | 0.1486448188 |
| angptl3 | 13639 | PDI_U | -0.001314865651 | 0.0009117336936 | -1.442159766 | 0.1492802896 |
| csf3r | 11411 | PDI_U | 0.001534922137 | 0.00106506006 | 1.44116017 | 0.1495669065 |
| lrrn1 | 13378 | PDI_U | 0.001429071163 | 0.0009917120243 | 1.441014254 | 0.1496040814 |
| bin2 | 13632 | PDI_U | 0.001436277895 | 0.000996884573 | 1.440766498 | 0.1496736487 |
| ccar2 | 11767 | PDI_U | -0.001537265917 | 0.001067177944 | -1.440496334 | 0.1497536596 |
| tnfrsf17 | 11537 | PDI_U | -0.001534113367 | 0.001065286509 | -1.440094617 | 0.1498677946 |
| ptpn9 | 11397 | PDI_U | 0.001564908604 | 0.00108678563 | 1.439942305 | 0.1499112188 |
| brk1 | 13320 | PDI_U | -0.001440447544 | 0.001000571126 | -1.439625337 | 0.1499969486 |
| bdnf | 11790 | PDI_U | 0.001528725974 | 0.001063756857 | 1.437100935 | 0.1507158792 |
| chmp1a | 13552 | PDI_U | 0.001441443847 | 0.001003174121 | 1.436883006 | 0.1507743453 |
| cd164 | 13632 | PDI_U | 0.001415361561 | 0.0009857030804 | 1.43589037 | 0.1510565048 |
| phldb1 | 11724 | PDI_U | 0.001485242277 | 0.001034490698 | 1.435723182 | 0.1511078323 |
| siglec10 | 13621 | PDI_U | -0.001384735944 | 0.0009645438083 | -1.435638083 | 0.1511283355 |
| fcer2 | 13632 | PDI_U | -0.001379783188 | 0.0009619476995 | -1.43436404 | 0.1514913628 |
| ntf4 | 13308 | PDI_U | 0.001444076383 | 0.001010682247 | 1.428813445 | 0.1530813516 |
| prdx3 | 13378 | PDI_U | -0.001429734473 | 0.001004523526 | -1.423296156 | 0.1546736615 |
| l1cam | 13699 | PDI_U | -0.001379484218 | 0.0009693559729 | -1.423093529 | 0.1547318363 |
| fgf21 | 13732 | PDI_U | 0.00131074078 | 0.0009220066956 | 1.42161742 | 0.1551600749 |
| atp6ap2 | 13308 | PDI_U | 0.001427997274 | 0.001004622138 | 1.421427241 | 0.1552160456 |
| cpm | 13721 | PDI_U | 0.001234083191 | 0.0008701064004 | 1.418312967 | 0.1561221437 |
| kdr | 13699 | PDI_U | 0.001392065982 | 0.0009818949507 | 1.417734128 | 0.1562911669 |
| pcna | 11304 | PDI_U | -0.001549269954 | 0.00109319256 | -1.417197675 | 0.156452731 |
| enox2 | 11355 | PDI_U | -0.001539192523 | 0.001088444804 | -1.414120879 | 0.1573538595 |
| cga | 13632 | PDI_U | -0.001048858006 | 0.0007418761374 | -1.41379127 | 0.1574460533 |
| cr2 | 13689 | PDI_U | -0.001387625431 | 0.0009815102116 | -1.413765659 | 0.1574534801 |
| il22ra1 | 13378 | PDI_U | -0.001417593061 | 0.001002987042 | -1.413371263 | 0.1575698784 |
| jchain | 13378 | PDI_U | -0.001414977835 | 0.001001625007 | -1.412682217 | 0.1577724665 |
| tmod4 | 11304 | PDI_U | 0.001546164396 | 0.001094535103 | 1.412622028 | 0.1577944487 |
| rbfox3 | 11757 | PDI_U | -0.00132070423 | 0.0009358946293 | -1.411167656 | 0.1582216725 |
| acan | 13497 | PDI_U | 0.001311120564 | 0.0009312588099 | 1.40790138 | 0.1591833261 |
| fus | 13283 | PDI_U | 0.001397060136 | 0.0009933235658 | 1.406450209 | 0.159613895 |
| gngt1 | 11483 | PDI_U | -0.00152289607 | 0.001082872593 | -1.406348337 | 0.1596478018 |
| naga | 11388 | PDI_U | 0.001523328049 | 0.001083357378 | 1.40611776 | 0.1597164725 |
| enpep | 11355 | PDI_U | 0.00153145903 | 0.001089527261 | 1.405617909 | 0.1598650043 |
| asgr2 | 11794 | PDI_U | 0.001477172709 | 0.001051035297 | 1.405445386 | 0.1599152462 |
| atp1b2 | 11355 | PDI_U | -0.001528887319 | 0.001088325571 | -1.404806943 | 0.1601060775 |
| traf3 | 11411 | PDI_U | 0.001509986018 | 0.001075184566 | 1.404397037 | 0.1602278988 |
| cgref1 | 13618 | PDI_U | 0.001378125166 | 0.0009817832237 | 1.403695982 | 0.1604322222 |
| nmt1 | 11757 | PDI_U | 0.001500182518 | 0.001069291267 | 1.402969017 | 0.1606525121 |
| atox1 | 13699 | PDI_U | 0.00138841748 | 0.0009899156109 | 1.402561455 | 0.1607703336 |
| reg4 | 13677 | PDI_U | -0.001325051108 | 0.0009457214063 | -1.401100895 | 0.1612066131 |
| hrg | 11740 | PDI_U | -0.00148058803 | 0.001057005502 | -1.400738243 | 0.1613188193 |
| itgbl1 | 11790 | PDI_U | 0.001407088945 | 0.001004667348 | 1.400552081 | 0.1613744023 |
| postn | 11790 | PDI_U | -0.001481820982 | 0.001058482568 | -1.399948404 | 0.161555109 |
| serpina11 | 13800 | PDI_U | -0.001244256871 | 0.000888993912 | -1.399623613 | 0.1616485568 |
| flt4 | 13699 | PDI_U | 0.001356191079 | 0.0009691747762 | 1.399325604 | 0.1617380271 |
| tex101 | 11483 | PDI_U | -0.001051497921 | 0.0007514689685 | -1.399256609 | 0.1617630858 |
| znf830 | 11730 | PDI_U | -0.001483344399 | 0.001060874531 | -1.39822793 | 0.1620710945 |
| pdcd1 | 13735 | PDI_U | -0.001364765143 | 0.0009789438234 | -1.39411998 | 0.1633039481 |
| atrn | 11817 | PDI_U | 0.001440888133 | 0.001033593158 | 1.394057345 | 0.1633265265 |
| vipr1 | 11474 | PDI_U | -0.001510755612 | 0.001084191227 | -1.393440174 | 0.163513743 |
| khk | 11474 | PDI_U | 0.00144803906 | 0.001039974305 | 1.392379651 | 0.163834474 |
| ahcy | 13618 | PDI_U | 0.001376929199 | 0.0009903808122 | 1.390302783 | 0.1644596948 |

| Protein | N_used | term | beta | se | t | p_val |
|---|---|---|---|---|---|---|
| timp1 | 13475 | PDI_U | 0.001326738719 | 0.0009548526379 | 1.389469606 | 0.1647129748 |
| ror1 | 13618 | PDI_U | 0.001344788685 | 0.0009688300949 | 1.388054202 | 0.1651432681 |
| myom1 | 11383 | PDI_U | 0.001509815333 | 0.001088308992 | 1.387303922 | 0.1653762963 |
| pecr | 11474 | PDI_U | -0.001501426216 | 0.001082952236 | -1.386419609 | 0.1656457781 |
| csnk1d | 11411 | PDI_U | 0.001499757032 | 0.001082247059 | 1.385780649 | 0.1658410032 |
| podxl | 13579 | PDI_U | -0.001387290288 | 0.001001283648 | -1.385511779 | 0.1659188097 |
| colec12 | 13658 | PDI_U | 0.001197183325 | 0.0008640746951 | 1.385509067 | 0.1659195065 |
| cd99 | 13632 | PDI_U | -0.001164724912 | 0.0008407947619 | -1.385266613 | 0.1659936449 |
| itgb6 | 13378 | PDI_U | 0.001302289068 | 0.00094014732 | 1.38519681 | 0.1660154127 |
| fn1 | 11817 | PDI_U | 0.001421775254 | 0.001027042992 | 1.384338596 | 0.1662809681 |
| fut1 | 11550 | PDI_U | 0.001494773987 | 0.001080195741 | 1.383799186 | 0.1664467208 |
| bcl2 | 11724 | PDI_U | -0.001436638138 | 0.001040159399 | -1.381171136 | 0.167252698 |
| robo2 | 13784 | PDI_U | -0.001292822448 | 0.0009368191543 | -1.380012825 | 0.1676050976 |
| ceacam8 | 13388 | PDI_U | 0.001343651871 | 0.0009742145481 | 1.379215568 | 0.1678513588 |
| acvrl1 | 13526 | PDI_U | -0.001267258359 | 0.0009188433091 | -1.379188755 | 0.1678593879 |
| siglec1 | 13710 | PDI_U | -0.001304393896 | 0.0009458420154 | -1.379082209 | 0.1678919247 |
| ddx25 | 11483 | PDI_U | -0.001490811579 | 0.001082173083 | -1.377609184 | 0.1683508595 |
| hmgcs1 | 11355 | PDI_U | -0.001501209644 | 0.001090150505 | -1.377066411 | 0.168518891 |
| tnfrsf1b | 13632 | PDI_U | -0.001280312107 | 0.000930953302 | -1.375269956 | 0.1690703866 |
| nptxr | 13618 | PDI_U | 0.00133176851 | 0.000968377714 | 1.375257289 | 0.1690743355 |
| skap1 | 13535 | PDI_U | 0.001344779252 | 0.0009780305012 | 1.374987028 | 0.169158246 |
| myh7b | 11304 | PDI_U | -0.001501976893 | 0.001093413739 | -1.373658332 | 0.1695750477 |
| klhl41 | 11757 | PDI_U | -0.001423883308 | 0.001038496019 | -1.371101364 | 0.1703695568 |
| fam20a | 11781 | PDI_U | 0.001327571461 | 0.0009698955321 | 1.368777788 | 0.1710948705 |
| mdk | 13651 | PDI_U | -0.001344346268 | 0.0009825649951 | -1.368200857 | 0.1712717521 |
| ctbs | 11616 | PDI_U | 0.001420842347 | 0.001039238527 | 1.367195605 | 0.1715904852 |
| sirt1 | 11411 | PDI_U | 0.001483426305 | 0.001085329416 | 1.366798212 | 0.1717155188 |
| aldh5a1 | 11757 | PDI_U | -0.001456423281 | 0.001066582302 | -1.365504826 | 0.172120588 |
| pcdh1 | 13757 | PDI_U | -0.001312620537 | 0.0009623275271 | -1.364006017 | 0.1725880068 |
| cdkl5 | 11383 | PDI_U | -0.001485544902 | 0.001089344282 | -1.363705605 | 0.1726872345 |
| cxcl9 | 13710 | PDI_U | -0.001263675799 | 0.0009278202443 | -1.361983431 | 0.173225511 |
| sptlc1 | 11767 | PDI_U | -0.001450038535 | 0.001065464119 | -1.36094544 | 0.1735570218 |
| arhgap25 | 13308 | PDI_U | 0.001369546648 | 0.001007379422 | 1.359514219 | 0.1740067552 |
| mrpl28 | 11757 | PDI_U | -0.001453870633 | 0.001070206241 | -1.358495753 | 0.1743325201 |
| ntrk3 | 13784 | PDI_U | -0.001245851927 | 0.0009178790701 | -1.357315978 | 0.1747030748 |
| sh2d1a | 13621 | PDI_U | 0.001334132657 | 0.0009845642953 | 1.35504879 | 0.1754245041 |
| ceacam6 | 11790 | PDI_U | -0.00141289178 | 0.001043023705 | -1.35461138 | 0.1755673897 |
| maneal | 11304 | PDI_U | 0.001480267766 | 0.001094287277 | 1.35272318 | 0.1761711668 |
| ptprs | 13800 | PDI_U | -0.001330115209 | 0.0009850766815 | -1.350265654 | 0.1769529482 |
| adh1b | 11537 | PDI_U | 0.001346921303 | 0.0009986209272 | 1.34878137 | 0.1774337012 |
| msr1 | 13784 | PDI_U | -0.001218934533 | 0.0009041864039 | -1.348100932 | 0.1776480938 |
| dnajb8 | 13388 | PDI_U | -0.001360795427 | 0.001009802697 | -1.347585455 | 0.1778145755 |
| clps | 13597 | PDI_U | 0.001334915037 | 0.0009910008808 | 1.347037185 | 0.1779907256 |
| mbl2 | 11632 | PDI_U | -0.001433338063 | 0.001065321743 | -1.345450866 | 0.1785059317 |
| proc | 13669 | PDI_U | 0.001317224359 | 0.0009796212136 | 1.344626209 | 0.1787683063 |
| mdga1 | 13552 | PDI_U | -0.001309212349 | 0.0009739129529 | -1.344280661 | 0.178880168 |
| dpp4 | 13359 | PDI_U | -0.001343025128 | 0.0009995120932 | -1.343680719 | 0.1790744971 |
| slitrk1 | 11411 | PDI_U | 0.001318777635 | 0.000981608918 | 1.343485792 | 0.1791414673 |
| vstm1 | 13320 | PDI_U | 0.001353527757 | 0.001008885372 | 1.341607079 | 0.1797463217 |
| gmpr | 13801 | PDI_U | 0.001306399342 | 0.0009737765396 | 1.341580218 | 0.1797542371 |
| fut3_fut5 | 13784 | PDI_U | -0.001257460641 | 0.0009373254284 | -1.341541159 | 0.179766936 |
| tlr2 | 11570 | PDI_U | -0.001444988213 | 0.001078067608 | -1.340350273 | 0.1801578403 |
| eln | 11781 | PDI_U | -0.001075477257 | 0.000804291611 | -1.337173286 | 0.181191923 |
| ntrk2 | 13618 | PDI_U | 0.001297503637 | 0.0009703818082 | 1.337106308 | 0.1812102922 |
| omd | 13710 | PDI_U | 0.00126279312 | 0.0009445597144 | 1.336911897 | 0.1812735988 |
| tfap2a | 11383 | PDI_U | 0.001452812767 | 0.001089982836 | 1.332876738 | 0.1825989624 |
| cdsn | 13760 | PDI_U | -0.00130915613 | 0.0009825669922 | -1.332383584 | 0.1827562576 |
| atp6v1f | 13303 | PDI_U | 0.00134614635 | 0.001010398583 | 1.332292397 | 0.1827869669 |
| lypd3 | 13582 | PDI_U | -0.001317236933 | 0.0009891013363 | -1.331751242 | 0.1829643129 |
| mtr | 11355 | PDI_U | -0.001451554763 | 0.001090133426 | -1.331538625 | 0.183038602 |
| mxra8 | 11537 | PDI_U | -0.001278346116 | 0.0009600909314 | -1.331484419 | 0.1830560028 |
| fam3d | 11724 | PDI_U | -0.001415735132 | 0.001064793088 | -1.32958708 | 0.1836802525 |
| plg | 11488 | PDI_U | -0.001402643703 | 0.001056094236 | -1.328142561 | 0.1841574285 |
| pros1 | 11817 | PDI_U | 0.00137025096 | 0.001032673209 | 1.326896977 | 0.184568429 |
| mitd1 | 13552 | PDI_U | 0.001328437381 | 0.001001378228 | 1.326609011 | 0.1846604269 |
| klrf1 | 11411 | PDI_U | 0.001378613714 | 0.001039672441 | 1.32600775 | 0.1848636997 |
| slirp | 11304 | PDI_U | -0.00145073592 | 0.001094529646 | -1.325442326 | 0.1850513015 |
| lrtm1 | 11483 | PDI_U | -0.001435915218 | 0.001084450724 | -1.32409448 | 0.1854980516 |
| atraid | 11724 | PDI_U | 0.001373527352 | 0.001037529969 | 1.323843545 | 0.1855808484 |
| zbtb16 | 13308 | PDI_U | 0.001333194819 | 0.001007180359 | 1.323690247 | 0.1856286967 |

| Protein | N_used | term | beta | se | t | p_val |
|---|---|---|---|---|---|---|
| dcun1d2 | 11304 | PDI_U | -0.001447447062 | 0.001093652536 | -1.323498108 | 0.18569658 |
| edem2 | 11355 | PDI_U | -0.001440071994 | 0.001089614774 | -1.321634056 | 0.1863166918 |
| timp3 | 13710 | PDI_U | 0.0013004135 | 0.000986104939 | 1.318737437 | 0.1872789529 |
| rrm2 | 13308 | PDI_U | 0.001316969856 | 0.0009988338032 | 1.318507495 | 0.1873565287 |
| fcrl2 | 13741 | PDI_U | -0.001292775455 | 0.0009839345243 | -1.31388362 | 0.1889073251 |
| sdhb | 11724 | PDI_U | -0.001406696999 | 0.001070789718 | -1.31370051 | 0.1889727413 |
| prrt3 | 11474 | PDI_U | 0.001360062572 | 0.001035884749 | 1.312947771 | 0.1892268335 |
| tpm3 | 11355 | PDI_U | -0.001415212122 | 0.001079066275 | -1.311515479 | 0.189710213 |
| angptl1 | 13411 | PDI_U | 0.001289891648 | 0.0009838277131 | 1.311095054 | 0.1898481198 |
| ccl21 | 13710 | PDI_U | -0.001269103588 | 0.0009697616031 | -1.308675848 | 0.1906661318 |
| il7 | 13621 | PDI_U | 0.001306653692 | 0.000998549904 | 1.308551217 | 0.1907085128 |
| c5 | 11817 | PDI_U | -0.001378481846 | 0.001053439116 | -1.308553884 | 0.1907109857 |
| tie1 | 13800 | PDI_U | -0.001290914963 | 0.0009865287037 | -1.30854273 | 0.1907111022 |
| gla | 11517 | PDI_U | 0.001320794086 | 0.001009845107 | 1.307917498 | 0.1909274258 |
| slamf6 | 13308 | PDI_U | 0.001304854303 | 0.0009986432387 | 1.306627084 | 0.1913619933 |
| ncam2 | 13632 | PDI_U | -0.001246905293 | 0.0009543780506 | -1.306510866 | 0.1914009475 |
| fgfbp2 | 11790 | PDI_U | 0.001374324648 | 0.001052411285 | 1.305881709 | 0.191618292 |
| ccl23 | 13710 | PDI_U | 0.001256502139 | 0.0009624424628 | 1.305534811 | 0.191732728 |
| psmd1 | 11757 | PDI_U | -0.001382519146 | 0.001059544517 | -1.30482403 | 0.1919783392 |
| clmp | 13753 | PDI_U | 0.001087092082 | 0.0008332817602 | 1.304591236 | 0.192053923 |
| vit | 11781 | PDI_U | 0.001244124006 | 0.0009539534033 | 1.304176915 | 0.1921987745 |
| pag1 | 13388 | PDI_U | 0.001275727811 | 0.0009786703574 | 1.303531676 | 0.1924157445 |
| nampt | 13308 | PDI_U | 0.001308693418 | 0.001004079683 | 1.303376057 | 0.1924689751 |
| tpsd1 | 11757 | PDI_U | -0.001387969041 | 0.001066012281 | -1.302019747 | 0.1929351689 |
| abca2 | 11355 | PDI_U | 0.001398094082 | 0.001074206491 | 1.301513344 | 0.193109233 |
| epgn | 11730 | PDI_U | -0.001392191838 | 0.001070852057 | -1.300078595 | 0.193599604 |
| larp1 | 11730 | PDI_U | 0.001389641345 | 0.001069260842 | 1.299628015 | 0.1937540593 |
| prss22 | 11411 | PDI_U | 0.001394864444 | 0.001073284331 | 1.29962248 | 0.1937566725 |
| sgsh | 11586 | PDI_U | 0.001357535861 | 0.001045036168 | 1.299032419 | 0.1939586813 |
| csf1 | 13710 | PDI_U | 0.001222326887 | 0.0009416721256 | 1.298038727 | 0.194295883 |
| psca | 11724 | PDI_U | -0.001383773078 | 0.001066243638 | -1.297801955 | 0.1943809565 |
| ntprobnp | 13618 | PDI_U | -0.001202069477 | 0.0009262775648 | -1.297742192 | 0.1943979405 |
| lhb | 13308 | PDI_U | -0.001232761874 | 0.00095002566 | -1.297609029 | 0.19444423 |
| fcgr2a | 13572 | PDI_U | -0.00127661204 | 0.000984445201 | -1.296783242 | 0.1947278438 |
| bcl2l11 | 13621 | PDI_U | 0.001283928712 | 0.0009902085917 | 1.296624491 | 0.194782406 |
| gbp2 | 13378 | PDI_U | -0.001305594679 | 0.001007165175 | -1.296306417 | 0.1948923199 |
| clec4a | 13621 | PDI_U | -0.001266688285 | 0.0009778276247 | -1.295410616 | 0.1952005907 |
| rtn4ip1 | 11757 | PDI_U | -0.001380027471 | 0.001065666918 | -1.294989502 | 0.1953493063 |
| pyy | 11355 | PDI_U | 0.001370762245 | 0.001060334283 | 1.292764242 | 0.1961189425 |
| pde1c | 11304 | PDI_U | -0.001413835296 | 0.001093693107 | -1.292716656 | 0.1961355244 |
| cd2ap | 13618 | PDI_U | 0.001277238364 | 0.0009896220815 | 1.290632442 | 0.1968530916 |
| immt | 11730 | PDI_U | -0.001313545044 | 0.001018656838 | -1.289487289 | 0.1972541833 |
| amfr | 13320 | PDI_U | -0.001300577513 | 0.001008856821 | -1.289159657 | 0.1973649937 |
| wif1 | 13753 | PDI_U | -0.001237209572 | 0.0009599193626 | -1.288868233 | 0.1974655975 |
| pi3 | 13441 | PDI_U | 0.001190239195 | 0.0009237787016 | 1.28844624 | 0.1976128688 |
| tshb | 13618 | PDI_U | -0.001279094838 | 0.0009929848504 | -1.288131272 | 0.197722177 |
| ttn | 11355 | PDI_U | -0.0013844617 | 0.001074845876 | -1.288056018 | 0.197752739 |
| igf1r | 13579 | PDI_U | 0.001246030974 | 0.0009673982778 | 1.288022734 | 0.1977600173 |
| mep1b | 13618 | PDI_U | 0.001266788663 | 0.0009839399099 | 1.287465474 | 0.197953995 |
| dag1 | 13710 | PDI_U | 0.001266976754 | 0.0009845945923 | 1.286800439 | 0.1981855992 |
| pcdh12 | 11757 | PDI_U | -0.00137374247 | 0.00106828779 | -1.285929206 | 0.1984931206 |
| hbq1 | 13753 | PDI_U | -0.001257787271 | 0.00097829454 | -1.285693847 | 0.1985715957 |
| chchd10 | 11767 | PDI_U | -0.001198997 | 0.0009330814944 | -1.284986367 | 0.1988223664 |
| adgre2 | 13710 | PDI_U | 0.001224938531 | 0.0009541988482 | 1.283735076 | 0.1992563816 |
| il2rb | 13378 | PDI_U | -0.001291658071 | 0.001007446498 | -1.282110836 | 0.1998260055 |
| pts | 13272 | PDI_U | 0.001219804194 | 0.0009523370163 | 1.280853493 | 0.2002675322 |
| tspan7 | 11383 | PDI_U | 0.001391379461 | 0.001086770317 | 1.280288428 | 0.2004698319 |
| cgn | 11483 | PDI_U | -0.001388089695 | 0.001084660531 | -1.279745741 | 0.2006604537 |
| ptprz1 | 11781 | PDI_U | -0.001235789815 | 0.0009662323138 | -1.278977941 | 0.2009300413 |
| cdcp1 | 13552 | PDI_U | -0.001118927995 | 0.000874927344 | -1.278881044 | 0.2009608669 |
| gabarapl1 | 11570 | PDI_U | 0.001378409368 | 0.001078927925 | 1.277573168 | 0.2014256269 |
| enpp7 | 13229 | PDI_U | 0.00126290602 | 0.0009893879952 | 1.276451732 | 0.2018183068 |
| tlr1 | 11411 | PDI_U | 0.001374005951 | 0.001076510183 | 1.276352025 | 0.2018571105 |
| ca7 | 11570 | PDI_U | -0.001374257974 | 0.001077169313 | -1.27580498 | 0.2020501054 |
| b3gnt7 | 11474 | PDI_U | -0.001330038058 | 0.001043068555 | -1.275120463 | 0.2022924527 |
| lcn2 | 13249 | PDI_U | 0.00123593609 | 0.0009712458618 | 1.272526493 | 0.2032084687 |
| axl | 13689 | PDI_U | -0.001253805135 | 0.0009860415188 | -1.271554099 | 0.2035532165 |
| cd3e | 11570 | PDI_U | -0.001372949967 | 0.0010798066 | -1.271477658 | 0.203584356 |
| txn | 11600 | PDI_U | -0.001355639951 | 0.001067670946 | -1.269717 | 0.2042109523 |
| klk11 | 13562 | PDI_U | -0.001249717817 | 0.0009846628793 | -1.269183436 | 0.2043974468 |

| Protein | N_used | term | beta | se | t | p_val |
|---|---|---|---|---|---|---|
| fam13a | 11724 | PDI_U | 0.001351245645 | 0.00106655952 | 1.266920055 | 0.2052090694 |
| spag1 | 11757 | PDI_U | 0.001351096347 | 0.001067137122 | 1.266094411 | 0.2055043946 |
| il19 | 13290 | PDI_U | 0.00124339094 | 0.0009847976216 | 1.262585238 | 0.2067604491 |
| s100a4 | 13699 | PDI_U | 0.001250087058 | 0.0009909071322 | 1.261558241 | 0.2071292902 |
| cd34 | 13744 | PDI_U | -0.001238102697 | 0.0009825877034 | -1.260042938 | 0.2076752858 |
| ggh | 13413 | PDI_U | 0.00118033191 | 0.0009378893076 | 1.258498098 | 0.208233599 |
| rnase4 | 11399 | PDI_U | -0.001243797733 | 0.0009888762566 | -1.257789056 | 0.2084938475 |
| lamp2 | 13278 | PDI_U | 0.001188150859 | 0.0009450629529 | 1.257218745 | 0.2086965704 |
| crybb2 | 11767 | PDI_U | -0.001316879904 | 0.001047586362 | -1.257060946 | 0.2087565433 |
| col4a4 | 11554 | PDI_U | -0.001357589431 | 0.001080566612 | -1.256368109 | 0.2090079663 |
| arhgef12 | 13621 | PDI_U | 0.001253567496 | 0.0009979299268 | 1.256167856 | 0.2090766818 |
| casp2 | 13621 | PDI_U | 0.001252264979 | 0.0009981400592 | 1.254598458 | 0.2096461097 |
| celsr2 | 11537 | PDI_U | 0.001328782275 | 0.001059874979 | 1.253716053 | 0.2099706614 |
| crisp3 | 11679 | PDI_U | 0.001324983374 | 0.001059023426 | 1.251136982 | 0.210909592 |
| letm1 | 11757 | PDI_U | -0.001321738819 | 0.00105955465 | -1.247447519 | 0.2122583242 |
| traf2 | 13621 | PDI_U | 0.001245258887 | 0.0009984020506 | 1.247251933 | 0.2123265995 |
| crhbp | 13658 | PDI_U | 0.001171749718 | 0.0009398349472 | 1.24676117 | 0.2125064818 |
| cst3 | 13659 | PDI_U | 0.001091331325 | 0.0008755390975 | 1.246467837 | 0.2126140845 |
| cd22 | 13801 | PDI_U | -0.001161271594 | 0.0009322188899 | -1.245706997 | 0.2128931497 |
| arid4b | 13320 | PDI_U | 0.001258006201 | 0.00101153031 | 1.243666343 | 0.2136442949 |
| cd80 | 11781 | PDI_U | -0.001268070464 | 0.001020849093 | -1.242172298 | 0.2141977495 |
| ptpn6 | 13691 | PDI_U | 0.001238471151 | 0.0009971160737 | 1.24205314 | 0.2142382502 |
| ceacam1 | 13678 | PDI_U | -0.001224274701 | 0.0009861830333 | -1.241427463 | 0.2144691861 |
| usp47 | 11355 | PDI_U | -0.001350665315 | 0.001089079728 | -1.240189567 | 0.21493094 |
| serpinb9 | 13552 | PDI_U | 0.001242749484 | 0.001002665273 | 1.239446023 | 0.215201849 |
| chi3l1 | 13317 | PDI_U | -0.001174738209 | 0.0009489818077 | -1.237893287 | 0.215777475 |
| bcl7a | 11304 | PDI_U | -0.001352139605 | 0.001094492706 | -1.235403029 | 0.2167062479 |
| cd177 | 13614 | PDI_U | -0.001227405228 | 0.0009960568681 | -1.232264209 | 0.2178717049 |
| fbn2 | 11483 | PDI_U | -0.001335659974 | 0.001084433491 | -1.231666105 | 0.2180990844 |
| mia | 13699 | PDI_U | 0.001200689398 | 0.0009755053142 | 1.230838398 | 0.2184044619 |
| agr3 | 13291 | PDI_U | -0.001212740978 | 0.000987281892 | -1.228363437 | 0.2193323372 |
| wwp2 | 13552 | PDI_U | 0.001220028034 | 0.0009958043713 | 1.225168386 | 0.2205330953 |
| sugt1 | 13308 | PDI_U | 0.001237389207 | 0.001012380549 | 1.222256994 | 0.2216321236 |
| cd74 | 13784 | PDI_U | 0.001121071089 | 0.000918714197 | 1.220260983 | 0.2223868494 |
| selplg | 13621 | PDI_U | 0.001192745068 | 0.0009779466258 | 1.219642296 | 0.2226216417 |
| cfp | 11817 | PDI_U | 0.0012486291 | 0.001024441399 | 1.21883897 | 0.2229296724 |
| pla2g10 | 13552 | PDI_U | -0.001167021029 | 0.0009586220946 | -1.217394254 | 0.2234754647 |
| acsl1 | 11355 | PDI_U | -0.001326122607 | 0.00108932935 | -1.217375266 | 0.2234867945 |
| rnf168 | 11411 | PDI_U | 0.001322068346 | 0.001086456697 | 1.216862438 | 0.2236817524 |
| plxdc2 | 11767 | PDI_U | -0.001288645673 | 0.001060198563 | -1.215475777 | 0.2242090923 |
| sirpa | 13675 | PDI_U | -0.001207795472 | 0.0009941016057 | -1.214961796 | 0.2244016582 |
| sez6l2 | 13579 | PDI_U | -0.001091188716 | 0.000899822469 | -1.212671114 | 0.2252767033 |
| slamf1 | 13378 | PDI_U | -0.001211228096 | 0.0009990779395 | -1.212345952 | 0.2254014094 |
| araf | 11570 | PDI_U | -0.001308140699 | 0.001079177093 | -1.212164998 | 0.225474005 |
| tmprss11d | 11755 | PDI_U | 0.001255961145 | 0.001036148133 | 1.212144388 | 0.2254815024 |
| palld | 11411 | PDI_U | -0.001313644088 | 0.001085385609 | -1.210301737 | 0.2261882271 |
| cd93 | 13141 | PDI_U | -0.001220809289 | 0.001009140344 | -1.209751742 | 0.2263959506 |
| neo1 | 11757 | PDI_U | 0.001276607903 | 0.001055585377 | 1.209383847 | 0.2265397546 |
| luzp2 | 11730 | PDI_U | 0.001263546022 | 0.001044836368 | 1.209324312 | 0.2265626723 |
| siae | 13674 | PDI_U | 0.001183076068 | 0.0009787982685 | 1.208702658 | 0.2267980192 |
| clec11a | 13615 | PDI_U | 0.001190931927 | 0.0009860355621 | 1.207798149 | 0.2271459116 |
| usp28 | 11767 | PDI_U | -0.001269964784 | 0.001052025657 | -1.207161417 | 0.2273942758 |
| eno3 | 11474 | PDI_U | 0.00119347376 | 0.0009901027153 | 1.205403986 | 0.2280722676 |
| tmsb10 | 13632 | PDI_U | 0.001174019477 | 0.0009743981249 | 1.204866314 | 0.2282758496 |
| gp2 | 13220 | PDI_U | -0.00117204938 | 0.0009733015653 | -1.204199625 | 0.2285340098 |
| msmb | 13592 | PDI_U | -0.001095440952 | 0.0009103207306 | -1.203357142 | 0.2288591225 |
| mamdc2 | 11781 | PDI_U | 0.001245269044 | 0.001035061003 | 1.203087586 | 0.2289666297 |
| ssbp1 | 11411 | PDI_U | -0.001304505253 | 0.001086460044 | -1.200693261 | 0.2298951591 |
| stat2 | 11411 | PDI_U | 0.001298903748 | 0.001082110144 | 1.20034338 | 0.2300309531 |
| csf2ra | 13552 | PDI_U | -0.001190795228 | 0.0009938577389 | -1.198154606 | 0.2308778055 |
| lbr | 13784 | PDI_U | 0.00116013359 | 0.0009694717407 | 1.196665712 | 0.2314574762 |
| scrib | 11474 | PDI_U | 0.001288068765 | 0.001076500592 | 1.196533261 | 0.2315132756 |
| sprr3 | 11767 | PDI_U | 0.001183198244 | 0.0009895188049 | 1.195730933 | 0.2318256959 |
| wfikkn2 | 13677 | PDI_U | 0.001105110351 | 0.0009247841714 | 1.194992719 | 0.2321106141 |
| efcab2 | 11570 | PDI_U | 0.001287655436 | 0.001077746878 | 1.194766102 | 0.2322029442 |
| slc51b | 11355 | PDI_U | 0.001290817186 | 0.001081106279 | 1.193978068 | 0.2325115158 |
| icam1 | 13565 | PDI_U | -0.00113921311 | 0.0009551350238 | -1.192724674 | 0.2329980998 |
| hcls1 | 13801 | PDI_U | 0.001176978371 | 0.0009869036182 | 1.192597078 | 0.2330477325 |
| crym | 11383 | PDI_U | -0.001294528437 | 0.001086208572 | -1.191786246 | 0.2333699426 |
| esam | 13800 | PDI_U | 0.001154150831 | 0.0009691045658 | 1.190945613 | 0.2336954384 |

| Protein | N_used | term | beta | se | t | p_val |
|---|---|---|---|---|---|---|
| col2a1 | 11474 | PDI_U | 0.001288439713 | 0.001081870194 | 1.190937434 | 0.2337028095 |
| frzb | 13503 | PDI_U | 0.001158257765 | 0.0009726320421 | 1.190848867 | 0.2337338728 |
| cd40lg | 13801 | PDI_U | 0.001179514806 | 0.000991460608 | 1.189673898 | 0.234195072 |
| smpd1 | 13727 | PDI_U | -0.001155527508 | 0.0009715016001 | -1.189424194 | 0.2342933759 |
| dynlt3 | 11730 | PDI_U | 0.001271906136 | 0.001069404691 | 1.18935904 | 0.2343225106 |
| c1rl | 11786 | PDI_U | -0.001198491346 | 0.001007825169 | -1.189185766 | 0.2343905561 |
| hexim1 | 13621 | PDI_U | 0.001177751664 | 0.00099211262 | 1.187114891 | 0.2352030263 |
| dpp6 | 13579 | PDI_U | 0.001110770142 | 0.0009390464013 | 1.182870346 | 0.2368812638 |
| lrg1 | 11817 | PDI_U | -0.001203210094 | 0.001019250929 | -1.180484667 | 0.2378312902 |
| umod | 13750 | PDI_U | -0.001103971838 | 0.0009356450302 | -1.179904561 | 0.2380586064 |
| grik2 | 11757 | PDI_U | 0.001259117799 | 0.00106730778 | 1.179713877 | 0.2381379273 |
| ltb | 11730 | PDI_U | 0.00124555417 | 0.001057640694 | 1.17767232 | 0.2389511833 |
| vim | 13388 | PDI_U | 0.001187169006 | 0.001008926055 | 1.17666602 | 0.2393497743 |
| pvr | 13784 | PDI_U | -0.001157210184 | 0.0009840285759 | -1.175992458 | 0.2396182177 |
| lmnb1 | 11689 | PDI_U | 0.001241871386 | 0.001056133762 | 1.175865624 | 0.2396725502 |
| snx18 | 11724 | PDI_U | -0.001255320223 | 0.001067974222 | -1.175421838 | 0.2398498849 |
| spaca5_spaca5b | 11757 | PDI_U | -0.001227048586 | 0.00104394396 | -1.175396988 | 0.2398597547 |
| cpq | 11773 | PDI_U | -0.001236458301 | 0.001052491923 | -1.174791249 | 0.2401020265 |
| tnn | 11790 | PDI_U | -0.001241660691 | 0.001057060801 | -1.174635073 | 0.2401644929 |
| stx7 | 11411 | PDI_U | 0.001245354422 | 0.001061235647 | 1.173494714 | 0.2406219943 |
| iglc2 | 11803 | PDI_U | -0.001221858714 | 0.001043022743 | -1.17145932 | 0.2414378716 |
| hsd17b14 | 11767 | PDI_U | -0.001245708071 | 0.00106523713 | -1.16941856 | 0.2422587469 |
| ccl7 | 13621 | PDI_U | -0.001099305775 | 0.0009406318158 | -1.168688701 | 0.2425495472 |
| ddx4 | 11411 | PDI_U | -0.001269493189 | 0.001086350284 | -1.168585499 | 0.2425951115 |
| b2m | 11817 | PDI_U | -0.001163957163 | 0.0009962621059 | -1.168324236 | 0.2426995965 |
| fmr1 | 13308 | PDI_U | 0.001180623407 | 0.001012647096 | 1.16587843 | 0.2436845143 |
| plcb1 | 11411 | PDI_U | -0.001263772717 | 0.001084780162 | -1.165003529 | 0.2440419432 |
| srp14 | 13579 | PDI_U | 0.001123012854 | 0.0009642352331 | 1.164666894 | 0.244174333 |
| shmt1 | 13691 | PDI_U | 0.00115337908 | 0.0009905660501 | 1.164363628 | 0.2442969877 |
| hmox2 | 13320 | PDI_U | -0.001160138899 | 0.0009965634481 | -1.164139524 | 0.2443883429 |
| scrn1 | 13621 | PDI_U | 0.001161268525 | 0.0009976675173 | 1.163983496 | 0.2444511058 |
| mpo | 13632 | PDI_U | 0.001127694833 | 0.0009691229892 | 1.163624066 | 0.2445967783 |
| upb1 | 11781 | PDI_U | 0.001140127245 | 0.0009805685047 | 1.162720645 | 0.244966438 |
| pdcd1lg2 | 13579 | PDI_U | -0.001152001168 | 0.0009930152727 | -1.160104179 | 0.2460268074 |
| s100a16 | 13301 | PDI_U | 0.001172513457 | 0.001010778143 | 1.160010697 | 0.2460652912 |
| evpl | 11570 | PDI_U | -0.001250264024 | 0.001079859044 | -1.157802985 | 0.2469683696 |
| ppif | 11790 | PDI_U | -0.001236672961 | 0.001068329842 | -1.157575978 | 0.247060591 |
| cdkn1a | 13579 | PDI_U | 0.001148739244 | 0.0009924319032 | 1.157499311 | 0.2470887982 |
| flt1 | 13559 | PDI_U | -0.001145586786 | 0.000990031306 | -1.157121779 | 0.2472430121 |
| qdpr | 13547 | PDI_U | 0.001146342108 | 0.0009909525714 | 1.156808248 | 0.2473711274 |
| eri1 | 11570 | PDI_U | -0.001232571023 | 0.001066672118 | -1.155529428 | 0.2478975755 |
| fgfr2 | 13699 | PDI_U | 0.001053299269 | 0.0009115454755 | 1.155509295 | 0.2479020996 |
| ceacam21 | 13621 | PDI_U | 0.001146022453 | 0.0009922255227 | 1.155001989 | 0.2481098912 |
| ccl20 | 13801 | PDI_U | -0.001105360983 | 0.0009582599 | -1.153508545 | 0.2487217061 |
| megf11 | 11355 | PDI_U | -0.001250574911 | 0.001084940825 | -1.152666471 | 0.2490715993 |
| tyro3 | 13618 | PDI_U | -0.001143083538 | 0.0009924136422 | -1.151821669 | 0.2494146002 |
| fgf3 | 11411 | PDI_U | 0.001251242733 | 0.00108652906 | 1.151596196 | 0.2495111983 |
| chmp6 | 11781 | PDI_U | 0.001221559326 | 0.001063863601 | 1.14822927 | 0.2508972668 |
| psapl1 | 11724 | PDI_U | -0.001204669143 | 0.001049963101 | -1.147344266 | 0.2512628024 |
| ankmy2 | 11411 | PDI_U | 0.001242281348 | 0.001086179461 | 1.143716478 | 0.2527652548 |
| hao1 | 13753 | PDI_U | 0.001073271812 | 0.000939216673 | 1.142730791 | 0.2531702913 |
| bmper | 11537 | PDI_U | 0.001201668558 | 0.001052358969 | 1.141880854 | 0.2535272714 |
| cnst | 13618 | PDI_U | 0.001140479433 | 0.0009994809275 | 1.141071731 | 0.253860157 |
| il10rb | 13710 | PDI_U | 0.001072336095 | 0.0009402238781 | 1.140511446 | 0.2540932271 |
| lbp | 13689 | PDI_U | -0.001100135459 | 0.0009670750851 | -1.13759053 | 0.255311431 |
| vstm2b | 11570 | PDI_U | -0.001225629585 | 0.00107887482 | -1.136025758 | 0.2559693391 |
| il21r | 11411 | PDI_U | -0.001233512086 | 0.001085862759 | -1.135974207 | 0.2559912415 |
| cntnap2 | 13621 | PDI_U | 0.001098018512 | 0.0009677229784 | 1.134641356 | 0.2565456022 |
| relt | 13632 | PDI_U | 0.0009771664102 | 0.0008618579841 | 1.133790518 | 0.256902385 |
| tnfrsf19 | 13753 | PDI_U | 0.001051808786 | 0.0009292523325 | 1.131887163 | 0.2577016284 |
| cxcl3 | 13642 | PDI_U | 0.001127813927 | 0.0009966715651 | 1.131580318 | 0.2578308269 |
| ikbkg | 13621 | PDI_U | 0.00112819185 | 0.0009971293982 | 1.131439763 | 0.2578899805 |
| dcbld2 | 13699 | PDI_U | -0.001081980847 | 0.0009567694322 | -1.130868954 | 0.258130067 |
| cntn5 | 13703 | PDI_U | 0.001032706337 | 0.0009139811481 | 1.129898947 | 0.2585386025 |
| mucl3 | 11304 | PDI_U | -0.001234462172 | 0.001093131498 | -1.129289728 | 0.2587996218 |
| zp4 | 11304 | PDI_U | -0.001233341135 | 0.001093060623 | -1.128337357 | 0.2592014444 |
| psmd9 | 13579 | PDI_U | 0.00111858316 | 0.0009917216383 | 1.127920493 | 0.2593734442 |
| mmp9 | 13602 | PDI_U | 0.001089769287 | 0.0009667538041 | 1.127245926 | 0.2596584203 |
| fcrl1 | 13618 | PDI_U | -0.001090265499 | 0.0009683673698 | -1.125880046 | 0.2602361547 |
| igf2r | 13632 | PDI_U | 0.001078966988 | 0.0009586420153 | 1.125516064 | 0.2603902463 |

| Protein | N_used | term | beta | se | t | p_val |
|---|---|---|---|---|---|---|
| ccn4 | 13753 | PDI_U | 0.001087787552 | 0.0009671600685 | 1.124723391 | 0.2607259126 |
| clta | 13388 | PDI_U | -0.001129475036 | 0.001006202851 | -1.122512259 | 0.2616648464 |
| glyr1 | 11730 | PDI_U | -0.001196031414 | 0.001065558003 | -1.12244609 | 0.2616958151 |
| shpk | 11570 | PDI_U | -0.001210454264 | 0.001079221377 | -1.1215996 | 0.262056023 |
| fli1 | 13308 | PDI_U | 0.001130595084 | 0.001008033844 | 1.12158445 | 0.2620594215 |
| vwf | 13800 | PDI_U | -0.001096137251 | 0.0009779581394 | -1.12084271 | 0.2623743421 |
| il31 | 11411 | PDI_U | 0.001216268356 | 0.001086175364 | 1.119771628 | 0.2628346856 |
| snapin | 11757 | PDI_U | 0.001190934787 | 0.001064305551 | 1.118978273 | 0.2631722924 |
| cthrc1 | 11790 | PDI_U | -0.001019396614 | 0.0009114345501 | -1.118452898 | 0.2633964235 |
| sdk2 | 11537 | PDI_U | 0.001188421478 | 0.001063149626 | 1.117830876 | 0.2636625323 |
| aldh3a1 | 13604 | PDI_U | -0.001061686957 | 0.0009509908654 | -1.116400794 | 0.2642703492 |
| txlna | 13552 | PDI_U | 0.001108509103 | 0.0009934401569 | 1.115828764 | 0.2645152412 |
| gimap8 | 11304 | PDI_U | 0.001202856085 | 0.001078415617 | 1.115391939 | 0.264706239 |
| id4 | 11570 | PDI_U | 0.001202737628 | 0.001078969351 | 1.114709724 | 0.2649980107 |
| cdh3 | 13784 | PDI_U | 0.001094714391 | 0.0009823139027 | 1.114424206 | 0.2651166913 |
| hsp90b1 | 13254 | PDI_U | -0.001130409162 | 0.00101534389 | -1.113326405 | 0.2655884787 |
| susd4 | 11730 | PDI_U | 0.001159384217 | 0.001041412679 | 1.113280298 | 0.2656109014 |
| dpep1 | 13535 | PDI_U | -0.001087022028 | 0.0009767279331 | -1.112922024 | 0.2657617027 |
| gabarap | 11730 | PDI_U | -0.001183336321 | 0.00106485888 | -1.111261166 | 0.2664787498 |
| rgmb | 13784 | PDI_U | 0.001062180949 | 0.0009564236244 | 1.110575818 | 0.2667703657 |
| gnas | 11757 | PDI_U | -0.001173259941 | 0.001056648152 | -1.110360094 | 0.2668666187 |
| adam8 | 13767 | PDI_U | -0.001074769368 | 0.0009680856607 | -1.11020069 | 0.266931963 |
| ccl3 | 13621 | PDI_U | 0.001021425108 | 0.0009205441626 | 1.109588382 | 0.2671960461 |
| fcamr | 11781 | PDI_U | 0.001069450463 | 0.0009653260505 | 1.107864501 | 0.2679429794 |
| ctsd | 12770 | PDI_U | 0.001039665281 | 0.0009406642332 | 1.105245893 | 0.2690738981 |
| sct | 11483 | PDI_U | 0.001185374571 | 0.001073001127 | 1.10472817 | 0.2693005644 |
| acp1 | 11411 | PDI_U | 0.001190498098 | 0.001077726907 | 1.104638003 | 0.269339793 |
| fam171a2 | 11757 | PDI_U | 0.001179998938 | 0.00106911382 | 1.10371685 | 0.2697385962 |
| cpa4 | 11301 | PDI_U | 0.00119797206 | 0.001085433926 | 1.103680317 | 0.2697553619 |
| ace2 | 13388 | PDI_U | 0.0009582686886 | 0.0008684363374 | 1.103441493 | 0.269855337 |
| pikfyve | 11411 | PDI_U | 0.001191802673 | 0.001080718963 | 1.102786861 | 0.2701430167 |
| sorcs2 | 13308 | PDI_U | 0.00105624779 | 0.0009593606785 | 1.100991331 | 0.2709203615 |
| akt1s1 | 13320 | PDI_U | 0.001106732698 | 0.001006721989 | 1.099342927 | 0.2716384018 |
| cd69 | 13550 | PDI_U | 0.0011028664 | 0.001003566157 | 1.098947381 | 0.271810561 |
| mpig6b | 13691 | PDI_U | 0.001094564272 | 0.0009972277473 | 1.097607116 | 0.2723954045 |
| nlgn1 | 11483 | PDI_U | -0.001189906914 | 0.001084213974 | -1.097483469 | 0.27245314 |
| procr | 11355 | PDI_U | -0.001193338495 | 0.001087560381 | -1.097261832 | 0.2725502434 |
| eif4b | 13552 | PDI_U | 0.001092811942 | 0.0009976640537 | 1.095370669 | 0.2733737621 |
| myl1 | 11355 | PDI_U | -0.001170830007 | 0.001070766302 | -1.093450555 | 0.2742192476 |
| acy1 | 13618 | PDI_U | 0.0009582719931 | 0.0008769576858 | 1.092723182 | 0.2745347052 |
| btc | 13308 | PDI_U | 0.001102766422 | 0.001009779763 | 1.092086079 | 0.2748150554 |
| cd8a | 13784 | PDI_U | 0.001064757872 | 0.0009750376168 | 1.092017225 | 0.2748446329 |
| mvk | 13378 | PDI_U | 0.001056559969 | 0.0009688431283 | 1.090537713 | 0.2754960092 |
| pdgfa | 13800 | PDI_U | 0.001079868114 | 0.0009903452339 | 1.090395628 | 0.2755579619 |
| clstn3 | 11767 | PDI_U | -0.001137869036 | 0.001044075408 | -1.089834151 | 0.2758085478 |
| prkra | 13308 | PDI_U | 0.001077997859 | 0.0009924765516 | 1.0861696 | 0.2774236557 |
| smpd3 | 11411 | PDI_U | 0.001173936585 | 0.001081676271 | 1.085293832 | 0.2778144885 |
| nudc | 13801 | PDI_U | 0.001073789036 | 0.0009899068687 | 1.084737433 | 0.2780569281 |
| cxadr | 13621 | PDI_U | 0.001071379722 | 0.000989863792 | 1.082350653 | 0.2791159237 |
| dkk3 | 13800 | PDI_U | 0.001013692711 | 0.0009385239588 | 1.080092523 | 0.2801198734 |
| ruvbl1 | 13308 | PDI_U | -0.001092628464 | 0.001012056222 | -1.079612417 | 0.2803343932 |
| slc28a1 | 11304 | PDI_U | -0.001180875456 | 0.001093835365 | -1.079573301 | 0.2803552941 |
| polr2f | 13579 | PDI_U | 0.001010378396 | 0.000936230402 | 1.079198447 | 0.2805184574 |
| arsb | 13579 | PDI_U | 0.001064359077 | 0.0009863048928 | 1.079137987 | 0.280545404 |
| phykpl | 11537 | PDI_U | -0.001155083045 | 0.00107104499 | -1.078463609 | 0.2808494845 |
| sdc1 | 13618 | PDI_U | 0.00104058569 | 0.000965256471 | 1.078040626 | 0.2810347385 |
| msra | 13699 | PDI_U | 0.001071672583 | 0.0009946564702 | 1.077429862 | 0.2813072583 |
| lrrc37a2 | 11724 | PDI_U | -0.001149441904 | 0.001068105976 | -1.076149679 | 0.2818824874 |
| gfap | 13293 | PDI_U | -0.0009971478677 | 0.0009297377113 | -1.072504488 | 0.2835129868 |
| bsg | 13779 | PDI_U | 0.001007526305 | 0.0009403339747 | 1.071455814 | 0.2839833106 |
| cep20 | 13308 | PDI_U | 0.001077010523 | 0.001005515121 | 1.07110326 | 0.2841424447 |
| tst | 13300 | PDI_U | 0.001084905481 | 0.001013816135 | 1.070120551 | 0.2845844872 |
| hip1 | 11355 | PDI_U | -0.00116601583 | 0.001089883524 | -1.069853617 | 0.2847079689 |
| tnc | 13689 | PDI_U | -0.001059430124 | 0.0009908387928 | -1.069225521 | 0.2849869316 |
| fga | 11530 | PDI_U | -0.001071356065 | 0.001002259561 | -1.068940728 | 0.2851187795 |
| iqgap2 | 13579 | PDI_U | -0.001060203217 | 0.000992690234 | -1.068010121 | 0.2855349495 |
| vtcn1 | 13308 | PDI_U | 0.001074346793 | 0.00100825025 | 1.065555691 | 0.2866438868 |
| smoc1 | 13753 | PDI_U | 0.0009656589794 | 0.0009084364801 | 1.063073975 | 0.2877670875 |
| cxcl16 | 13773 | PDI_U | 0.001009455334 | 0.0009496168812 | 1.063013257 | 0.2877945931 |
| dll4 | 11474 | PDI_U | 0.00114698918 | 0.001080486825 | 1.06154851 | 0.2884630658 |

| Protein | N_used | term | beta | se | t | p_val |
|---|---|---|---|---|---|---|
| spon1 | 13677 | PDI_U | -0.0009982285704 | 0.0009405278031 | -1.061349348 | 0.2885499243 |
| gclm | 11355 | PDI_U | -0.001150449746 | 0.001086317704 | -1.059036175 | 0.289605856 |
| alcam | 13800 | PDI_U | -0.0009693659619 | 0.0009156507631 | -1.058663413 | 0.2897716421 |
| nrn1 | 11757 | PDI_U | -0.001132397474 | 0.001070079811 | -1.058236463 | 0.2899694154 |
| pmm2 | 11355 | PDI_U | -0.001150129441 | 0.001087024956 | -1.058052471 | 0.2900540525 |
| siglec15 | 13320 | PDI_U | 0.00106870733 | 0.00101172694 | 1.056319929 | 0.2908412491 |
| metap1d | 13621 | PDI_U | -0.001052009268 | 0.000997735345 | -1.054397113 | 0.2917198621 |
| skap2 | 13710 | PDI_U | 0.001048675418 | 0.0009948542992 | 1.054099498 | 0.2918559581 |
| adamts1 | 11411 | PDI_U | 0.001124430116 | 0.00106891589 | 1.051935075 | 0.2928516371 |
| tjp3 | 11383 | PDI_U | -0.001145859518 | 0.00109056519 | -1.050702451 | 0.293417599 |
| sarg | 11723 | PDI_U | 0.001120209233 | 0.001067296963 | 1.049575958 | 0.2939347742 |
| fcrl3 | 13378 | PDI_U | -0.001054291948 | 0.001004623702 | -1.049439652 | 0.2939947912 |
| steap4 | 11383 | PDI_U | -0.001143140439 | 0.001090255055 | -1.048507351 | 0.2944272021 |
| parp1 | 13621 | PDI_U | 0.001013339642 | 0.0009667715035 | 1.048168712 | 0.2945794973 |
| c7 | 11510 | PDI_U | -0.001102153745 | 0.001051988993 | -1.047685624 | 0.2948054953 |
| eif5a | 13378 | PDI_U | -0.00105877913 | 0.001010686416 | -1.047584208 | 0.2948491589 |
| fosb | 13320 | PDI_U | 0.001060459525 | 0.001012310234 | 1.04756377 | 0.2948586619 |
| ifit1 | 11570 | PDI_U | 0.001130107752 | 0.001079259882 | 1.047113649 | 0.2950690617 |
| ahsa1 | 11730 | PDI_U | 0.001120884286 | 0.001070566976 | 1.047000618 | 0.2951208883 |
| ghrhr | 13320 | PDI_U | 0.001060829761 | 0.001013321675 | 1.04688352 | 0.2951723169 |
| bag6 | 13388 | PDI_U | -0.00104962852 | 0.001002847212 | -1.04664849 | 0.2952806415 |
| tnfaip8l2 | 11537 | PDI_U | 0.001129006213 | 0.00107915224 | 1.046197349 | 0.2954918674 |
| apod | 11285 | PDI_U | -0.001054979758 | 0.001008606489 | -1.045977564 | 0.2955938182 |
| milr1 | 13378 | PDI_U | -0.001035630593 | 0.0009906870506 | -1.045366034 | 0.2958727336 |
| apcs | 11694 | PDI_U | 0.0009522531207 | 0.0009110928721 | 1.045176787 | 0.2959628959 |
| hdac9 | 11383 | PDI_U | 0.001139050464 | 0.001090403946 | 1.044613299 | 0.2962239365 |
| rida | 11537 | PDI_U | 0.001038034722 | 0.0009937637734 | 1.044548765 | 0.2962534779 |
| tnfrsf11b | 13658 | PDI_U | -0.0009379810416 | 0.0008986886269 | -1.043721945 | 0.2966325427 |
| prss53 | 11767 | PDI_U | -0.001111354927 | 0.001066068387 | -1.042479957 | 0.2972106521 |
| nlgn2 | 11383 | PDI_U | -0.001135785687 | 0.001089571592 | -1.042414923 | 0.2972415124 |
| pf4 | 11717 | PDI_U | 0.001116208403 | 0.001071808944 | 1.041424788 | 0.2976999526 |
| sema3f | 13618 | PDI_U | 0.0009570497649 | 0.0009208679727 | 1.039290966 | 0.2986879032 |
| cuzd1 | 11483 | PDI_U | -0.001126068328 | 0.001083612227 | -1.03918016 | 0.2987428589 |
| hif1a | 11411 | PDI_U | 0.001126091412 | 0.001085246182 | 1.037636834 | 0.2994611728 |
| pi16 | 11794 | PDI_U | -0.001075259861 | 0.001037495602 | -1.03639944 | 0.3000371018 |
| atp1b3 | 11355 | PDI_U | -0.001128173921 | 0.00108953206 | -1.035466475 | 0.3004731895 |
| il7r | 13632 | PDI_U | 0.001009715062 | 0.0009756770592 | 1.034886546 | 0.3007402723 |
| sv2a | 11483 | PDI_U | -0.001119570812 | 0.001082868625 | -1.033893481 | 0.3012077591 |
| ifngr2 | 13552 | PDI_U | 0.001018460919 | 0.0009851743514 | 1.033787489 | 0.3012539853 |
| pla2g2a | 13629 | PDI_U | -0.0009752779237 | 0.0009440832096 | -1.033042335 | 0.3016024335 |
| ofd1 | 11570 | PDI_U | 0.001113721956 | 0.001079607972 | 1.031598492 | 0.3022818327 |
| cd209 | 13680 | PDI_U | -0.00102311191 | 0.0009928904706 | -1.030437838 | 0.3028227521 |
| nfatc1 | 13621 | PDI_U | 0.001022758232 | 0.0009937822946 | 1.029157228 | 0.3034240879 |
| cd160 | 13801 | PDI_U | 0.001006912214 | 0.0009785412573 | 1.028993113 | 0.3035009601 |
| pak4 | 13320 | PDI_U | 0.001036814587 | 0.001008925865 | 1.027641993 | 0.3041369406 |
| nadk | 13618 | PDI_U | 0.0009920027113 | 0.0009654222001 | 1.027532525 | 0.3041880445 |
| ifnlr1 | 13621 | PDI_U | 0.0009845117181 | 0.0009586830638 | 1.026941807 | 0.3044661188 |
| pdzd2 | 11355 | PDI_U | -0.001111054295 | 0.00108554991 | -1.023494438 | 0.3060959553 |
| odam | 13301 | PDI_U | 0.001018342106 | 0.0009955618359 | 1.022881823 | 0.3063823446 |
| eef1d | 11781 | PDI_U | 0.001083518058 | 0.001059953102 | 1.022232075 | 0.3066920847 |
| ctrl | 11767 | PDI_U | -0.00107283806 | 0.001049616074 | -1.022124267 | 0.3067431232 |
| sla2 | 11757 | PDI_U | 0.001089677687 | 0.001066870165 | 1.021377973 | 0.3070964342 |
| pon2 | 13388 | PDI_U | 0.001011237112 | 0.0009908038703 | 1.020622892 | 0.307451598 |
| satb1 | 11757 | PDI_U | -0.001090905581 | 0.00106913927 | -1.020358723 | 0.3075793791 |
| gfod2 | 13308 | PDI_U | 0.001033574571 | 0.001012981116 | 1.020329554 | 0.3075907559 |
| gnpda1 | 11474 | PDI_U | -0.001101204971 | 0.001079541783 | -1.020067021 | 0.3077182056 |
| tnfrsf10a | 13552 | PDI_U | -0.0009478644869 | 0.0009293429047 | -1.019929761 | 0.3077799966 |
| smpdl3b | 11730 | PDI_U | -0.001092607883 | 0.001071674909 | -1.019532952 | 0.307971066 |
| itgb1bp1 | 13308 | PDI_U | 0.001032287345 | 0.001012883279 | 1.019157258 | 0.3081468584 |
| muc2 | 11790 | PDI_U | -0.001081672991 | 0.001063141863 | -1.017430531 | 0.3089695626 |
| dtx2 | 11483 | PDI_U | -0.001098491228 | 0.001079812025 | -1.01729857 | 0.3090328722 |
| coq7 | 11474 | PDI_U | -0.001092642184 | 0.001074252873 | -1.017118233 | 0.309118656 |
| fst | 13691 | PDI_U | -0.000976681943 | 0.0009606427201 | -1.016696346 | 0.3093158864 |
| pde4d | 11355 | PDI_U | -0.00110797058 | 0.001089787461 | -1.016685015 | 0.3093249813 |
| pdgfc | 13308 | PDI_U | 0.001020187537 | 0.001004061769 | 1.016060533 | 0.3096190497 |
| fyb1 | 13597 | PDI_U | 0.001010028239 | 0.0009943671441 | 1.015749811 | 0.3097666308 |
| gsn | 11744 | PDI_U | -0.001074762646 | 0.001059566547 | -1.014341807 | 0.3104405997 |
| padi2 | 13378 | PDI_U | 0.001016647444 | 0.001002366207 | 1.014247525 | 0.3104830158 |
| med18 | 13308 | PDI_U | 0.001026257674 | 0.001011848204 | 1.014240743 | 0.3104863476 |
| icam2 | 13800 | PDI_U | 0.001006473028 | 0.0009927622913 | 1.013810694 | 0.3106908812 |

| Protein | N_used | term | beta | se | t | p_val |
|---|---|---|---|---|---|---|
| ankra2 | 11304 | PDI_U | -0.00110936353 | 0.001095000931 | -1.013116518 | 0.3110262166 |
| vwc2l | 11757 | PDI_U | 0.001010192508 | 0.0009989100722 | 1.011294746 | 0.3118962127 |
| tpsab1 | 13779 | PDI_U | -0.0009939344848 | 0.0009834146524 | -1.01069725 | 0.3121791165 |
| cfb | 11581 | PDI_U | -0.0009958248371 | 0.0009853297104 | -1.010651386 | 0.312204449 |
| tp73 | 11483 | PDI_U | -0.001093815477 | 0.001085039612 | -1.00808806 | 0.3134334529 |
| anxa2 | 11291 | PDI_U | -0.001069080716 | 0.001061483806 | -1.007156878 | 0.3138809974 |
| sf3b4 | 13579 | PDI_U | 0.001005974449 | 0.001001085456 | 1.004883692 | 0.3149707798 |
| adgre1 | 11724 | PDI_U | -0.00105302007 | 0.001048373565 | -1.004432108 | 0.3151911313 |
| akr1b1 | 13308 | PDI_U | 0.001015994115 | 0.001011555698 | 1.004387714 | 0.3152100477 |
| eif2ak3 | 11355 | PDI_U | -0.001093020925 | 0.001088473894 | -1.004177437 | 0.3153145163 |
| ly96 | 13552 | PDI_U | -0.0009735004433 | 0.000971351835 | -1.002211977 | 0.3162591342 |
| nedd9 | 11411 | PDI_U | 0.001082338674 | 0.001080645612 | 1.001566714 | 0.3165741641 |
| adamts13 | 13800 | PDI_U | 0.0009827577756 | 0.0009812564576 | 1.001529996 | 0.3165882219 |
| rps10 | 11355 | PDI_U | -0.00108962644 | 0.001088203568 | -1.001307542 | 0.3166995072 |
| serpina7 | 11817 | PDI_U | -0.001037734821 | 0.001036576037 | -1.001117897 | 0.3167903335 |
| scarb1 | 13320 | PDI_U | -0.001013466077 | 0.001012551256 | -1.000903482 | 0.3168916715 |
| arhgap30 | 11570 | PDI_U | -0.001076835561 | 0.001078095949 | -0.9988309136 | 0.3178975184 |
| mslnl | 11483 | PDI_U | -0.001081209662 | 0.001083112394 | -0.998243274 | 0.3181824637 |
| hsd17b3 | 11355 | PDI_U | -0.001085968248 | 0.001088215115 | -0.9979352734 | 0.3183320338 |
| rasa1 | 13320 | PDI_U | -0.001010655236 | 0.001013114835 | -0.9975722412 | 0.318504965 |
| med21 | 11355 | PDI_U | 0.001085359722 | 0.001089621946 | 0.9960883454 | 0.3192284702 |
| brap | 11610 | PDI_U | 0.001070884834 | 0.001075149642 | 0.9960332893 | 0.3192547507 |
| nme3 | 13677 | PDI_U | -0.0009871996169 | 0.0009912404376 | -0.9959234706 | 0.3193049652 |
| pik3ap1 | 13621 | PDI_U | -0.000993728938 | 0.0009984492918 | -0.995272315 | 0.3196215345 |
| ca1 | 13352 | PDI_U | 0.0009805396381 | 0.000985503128 | 0.9949634966 | 0.3197720653 |
| olr1 | 13492 | PDI_U | 0.0009897756409 | 0.0009950889432 | 0.9946604749 | 0.319919278 |
| zcchc8 | 11355 | PDI_U | -0.001083904984 | 0.001089723936 | -0.9946601595 | 0.319922795 |
| plxnb3 | 13580 | PDI_U | 0.0009951047747 | 0.001001316486 | 0.9937964553 | 0.3203396949 |
| gbp6 | 11355 | PDI_U | -0.00108289134 | 0.00109006175 | -0.9934220151 | 0.3205255283 |
| stx16 | 13308 | PDI_U | 0.001004565488 | 0.001011898376 | 0.9927533357 | 0.3208482419 |
| fgf7 | 11304 | PDI_U | -0.001085360331 | 0.001093406613 | -0.9926410895 | 0.3209061629 |
| gpha2 | 11724 | PDI_U | 0.001030753953 | 0.001038462083 | 0.9925773609 | 0.3209364677 |
| scgb2a2 | 11355 | PDI_U | -0.001081309139 | 0.001089571816 | -0.9924165833 | 0.321015522 |
| vsir | 13593 | PDI_U | 0.0009875261511 | 0.0009989223353 | 0.9885915213 | 0.3228806483 |
| cep85 | 13308 | PDI_U | 0.0009951960281 | 0.001006772165 | 0.988501731 | 0.3229249751 |
| fen1 | 13308 | PDI_U | 0.0009861229937 | 0.0009980261361 | 0.9880733159 | 0.3231347232 |
| scg3 | 13677 | PDI_U | 0.0009449309549 | 0.0009565499078 | 0.9878532706 | 0.3232420046 |
| kiaa1549l | 11570 | PDI_U | -0.001064571783 | 0.001078541766 | -0.9870473415 | 0.3236400928 |
| diablo | 13568 | PDI_U | 0.0009903223929 | 0.001003379988 | 0.9869863912 | 0.3236669257 |
| dsg4 | 13534 | PDI_U | 0.0009611288359 | 0.0009740125004 | 0.9867725881 | 0.3237717918 |
| crkl | 13676 | PDI_U | 0.0009824743712 | 0.0009960299343 | 0.9863904059 | 0.3239590369 |
| fbln2 | 11790 | PDI_U | 0.0009955888413 | 0.001011858797 | 0.9839207251 | 0.3251747036 |
| tmem106a | 11730 | PDI_U | 0.001051106687 | 0.001068478116 | 0.9837418952 | 0.3252627457 |
| ifngr1 | 13710 | PDI_U | 0.0009605142329 | 0.0009767782048 | 0.9833493705 | 0.3254528871 |
| app | 13611 | PDI_U | 0.0009792394111 | 0.0009959627058 | 0.9832089147 | 0.3255221199 |
| actn4 | 13378 | PDI_U | -0.0009919123662 | 0.001008948196 | -0.9831152578 | 0.3255685106 |
| pltp | 13760 | PDI_U | -0.0009176403402 | 0.0009338024923 | -0.9826921086 | 0.3257762887 |
| slc39a14 | 13320 | PDI_U | 0.0009759340884 | 0.0009932587191 | 0.9825577864 | 0.3258429914 |
| clec4c | 13621 | PDI_U | -0.0009728333154 | 0.0009905756406 | -0.9820888739 | 0.3260735249 |
| pfkfb2 | 13561 | PDI_U | 0.0009760454218 | 0.0009940648469 | 0.9818729883 | 0.3261799566 |
| defb116 | 11383 | PDI_U | -0.001068675149 | 0.001088542998 | -0.9817482187 | 0.3262447913 |
| nrxn3 | 11483 | PDI_U | -0.001062230427 | 0.001083210003 | -0.9806320321 | 0.3267949135 |
| serpinb1 | 13575 | PDI_U | 0.0009750982341 | 0.0009957259629 | 0.9792837291 | 0.3274572746 |
| pnma2 | 11383 | PDI_U | 0.001065902521 | 0.001089694616 | 0.978166273 | 0.3280129041 |
| zbp1 | 11411 | PDI_U | 0.001047483158 | 0.00107177283 | 0.977336283 | 0.3284234366 |
| map4k5 | 13535 | PDI_U | 0.0009791012552 | 0.001002595075 | 0.976566991 | 0.3288010364 |
| sytl4 | 11781 | PDI_U | 0.001038574898 | 0.001064598213 | 0.9755557405 | 0.3293047176 |
| tbcc | 13552 | PDI_U | 0.000977194396 | 0.001001807836 | 0.9754309768 | 0.3293639504 |
| pagr1 | 11474 | PDI_U | 0.001052402072 | 0.001079914934 | 0.9745231208 | 0.3298174307 |
| nptx1 | 13632 | PDI_U | -0.0009251381129 | 0.0009494990633 | -0.9743433656 | 0.3299033836 |
| mansc4 | 11355 | PDI_U | -0.001044449829 | 0.001072186209 | -0.9741310045 | 0.3300122699 |
| gpc1 | 13753 | PDI_U | 0.0009395186167 | 0.0009645502238 | 0.974048415 | 0.3300496471 |
| trim26 | 11304 | PDI_U | -0.001065958603 | 0.001094467762 | -0.9739515773 | 0.3301014456 |
| tff3 | 13610 | PDI_U | -0.0009370870455 | 0.0009642032334 | -0.9718771033 | 0.3311289789 |
| rab39b | 11355 | PDI_U | -0.001056365771 | 0.001088684539 | -0.9703139276 | 0.3319107265 |
| muc13 | 13320 | PDI_U | -0.0009606814088 | 0.0009926339787 | -0.9678103202 | 0.3331566964 |
| rp2 | 13579 | PDI_U | -0.0009647063462 | 0.0009982773551 | -0.9663710604 | 0.3338757653 |
| havcr2 | 13632 | PDI_U | -0.000898637283 | 0.000930247791 | -0.9660192603 | 0.3340516955 |
| psmd5 | 11483 | PDI_U | 0.001046538927 | 0.00108335345 | 0.966017994 | 0.3340555424 |
| ca5a | 13388 | PDI_U | 0.0009046406821 | 0.0009375898122 | 0.9648576278 | 0.3346335702 |

| Protein | N_used | term | beta | se | t | p_val |
|---|---|---|---|---|---|---|
| bag4 | 11411 | PDI_U | 0.0010349764 | 0.001073737313 | 0.963900935 | 0.3351160425 |
| tpr | 11355 | PDI_U | 0.001022624695 | 0.001063658009 | 0.9614224562 | 0.3363602914 |
| tgfb1 | 13779 | PDI_U | 0.0009311238633 | 0.0009685915178 | 0.9613173832 | 0.3364094937 |
| vegfa | 13759 | PDI_U | 0.0009288445779 | 0.0009663945293 | 0.961144284 | 0.336496531 |
| ccl14 | 13590 | PDI_U | 0.0009402996612 | 0.0009785008823 | 0.9609594413 | 0.3365896733 |
| scgb3a2 | 13691 | PDI_U | -0.0008979870104 | 0.0009352856673 | -0.9601205726 | 0.3370115058 |
| il32 | 13621 | PDI_U | -0.0009295656609 | 0.0009691489133 | -0.959156687 | 0.3374968563 |
| fis1 | 13646 | PDI_U | 0.0009559522621 | 0.0009975299332 | 0.958319375 | 0.3379187298 |
| ppp1r12a | 13699 | PDI_U | 0.0009514114041 | 0.0009932356434 | 0.9578909198 | 0.338134685 |
| rnaseh2a | 11757 | PDI_U | 0.001021360895 | 0.001066823999 | 0.9573846251 | 0.3383928619 |
| pibf1 | 11757 | PDI_U | 0.001020421577 | 0.001066180156 | 0.9570817571 | 0.3385456903 |
| ccl8 | 13736 | PDI_U | 0.0009373101776 | 0.0009819706462 | 0.9545195482 | 0.339837531 |
| il17a | 13359 | PDI_U | -0.000963677126 | 0.001010611486 | -0.9535584538 | 0.340324463 |
| nos3 | 13320 | PDI_U | 0.0009088187255 | 0.0009538553888 | 0.9527846005 | 0.3407165229 |
| gp1ba | 13575 | PDI_U | 0.0009508787907 | 0.0009989935884 | 0.9518367303 | 0.3411967526 |
| caps | 11767 | PDI_U | -0.000965883592 | 0.001016558606 | -0.9501504251 | 0.3420553584 |
| artn | 13378 | PDI_U | -0.0009580354092 | 0.00101009832 | -0.9484575812 | 0.3429137054 |
| tnpo1 | 11570 | PDI_U | -0.00102395034 | 0.001079932516 | -0.9481614126 | 0.3430671115 |
| acrbp | 11730 | PDI_U | 0.001014107109 | 0.001069607531 | 0.9481114141 | 0.3430922899 |
| pgm2 | 11483 | PDI_U | -0.001027826364 | 0.001084664738 | -0.9475982092 | 0.3433539996 |
| anxa3 | 13767 | PDI_U | 0.000937415915 | 0.0009896174107 | 0.9472508313 | 0.3435276195 |
| traf3ip2 | 11355 | PDI_U | -0.001031948211 | 0.001090058442 | -0.9466907199 | 0.343816567 |
| pafah1b3 | 11757 | PDI_U | 0.001008916555 | 0.001066788151 | 0.945751557 | 0.344294775 |
| lat | 13801 | PDI_U | 0.0009342612119 | 0.0009881347681 | 0.945479546 | 0.3444306753 |
| gzma | 13801 | PDI_U | 0.0009138665248 | 0.0009666847245 | 0.9453615038 | 0.3444909134 |
| lifr | 13621 | PDI_U | -0.0009308185716 | 0.0009856456837 | -0.9443744207 | 0.3449951135 |
| tacstd2 | 12097 | PDI_U | 0.0009856116981 | 0.001043769725 | 0.944280788 | 0.3450450585 |
| impg1 | 11383 | PDI_U | -0.001027923911 | 0.001089617557 | -0.9433804592 | 0.3455063774 |
| nectin1 | 11411 | PDI_U | 0.001022901023 | 0.00108527095 | 0.9425305472 | 0.3459410539 |
| sned1 | 11474 | PDI_U | -0.001010753486 | 0.001073306552 | -0.9417192912 | 0.3463562226 |
| tagln3 | 11304 | PDI_U | -0.001030757693 | 0.001095144376 | -0.941207128 | 0.3466188587 |
| lama4 | 13710 | PDI_U | 0.0009239109034 | 0.0009825534826 | 0.9403161455 | 0.3470720014 |
| fgf20 | 11411 | PDI_U | 0.001021361458 | 0.001086383664 | 0.9401480272 | 0.347161558 |
| gstt2b | 11767 | PDI_U | 0.001003477415 | 0.001068285373 | 0.9393346012 | 0.3475782793 |
| paep | 13552 | PDI_U | 0.0009001619627 | 0.0009607028235 | 0.9369827388 | 0.3487841488 |
| tsc1 | 11411 | PDI_U | -0.001017165964 | 0.001085736238 | -0.9368444459 | 0.3488584266 |
| fgf19 | 13710 | PDI_U | 0.0009239539809 | 0.0009864525676 | 0.9366430899 | 0.3489586919 |
| eif4g3 | 11757 | PDI_U | 0.0009984453154 | 0.001068294763 | 0.9346159416 | 0.3500054749 |
| adgrg2 | 13618 | PDI_U | -0.0008232264614 | 0.0008811268873 | -0.9342882089 | 0.3501718251 |
| smad1 | 13308 | PDI_U | 0.0009366350672 | 0.001003598936 | 0.9332762657 | 0.3506942935 |
| metap2 | 13732 | PDI_U | 0.0009217336085 | 0.0009884410888 | 0.9325124369 | 0.3510881717 |
| lgmn | 13710 | PDI_U | -0.0009144062485 | 0.0009806410887 | -0.9324576127 | 0.3511165172 |
| siglec9 | 13736 | PDI_U | -0.0009104403544 | 0.0009791625587 | -0.9298153267 | 0.3524830635 |
| nppb | 13388 | PDI_U | -0.0009118972911 | 0.0009813212407 | -0.9292546144 | 0.3527739188 |
| col1a1 | 13800 | PDI_U | 0.0009175494145 | 0.0009879778553 | 0.9287145553 | 0.3530532933 |
| tpp1 | 13564 | PDI_U | 0.0008882519833 | 0.0009564883828 | 0.9286594581 | 0.3530821374 |
| itpa | 11355 | PDI_U | -0.00100592481 | 0.001083200239 | -0.9286600706 | 0.3530850408 |
| hdac8 | 11411 | PDI_U | 0.001008030946 | 0.001085632077 | 0.9285198621 | 0.3531576303 |
| yju2 | 11730 | PDI_U | -0.0009902526306 | 0.001068482851 | -0.9267838316 | 0.3540578686 |
| gage2a | 11304 | PDI_U | -0.001011810274 | 0.001093708542 | -0.9251187445 | 0.3549239133 |
| akt3 | 13239 | PDI_U | 0.0009387815424 | 0.001015297862 | 0.9246365792 | 0.3551718471 |
| nefl | 13285 | PDI_U | -0.0008029468995 | 0.0008694478339 | -0.9235136005 | 0.3557564039 |
| ufd1 | 11724 | PDI_U | 0.0009861347425 | 0.001069102073 | 0.9223953145 | 0.3563414135 |
| casc3 | 11483 | PDI_U | 0.000992964657 | 0.001078177987 | 0.9209654337 | 0.3570878418 |
| prss8 | 13598 | PDI_U | 0.0007953886549 | 0.0008646164867 | 0.9199323251 | 0.3576244584 |
| nudt15 | 11355 | PDI_U | -0.001001700573 | 0.001089329615 | -0.9195569086 | 0.357823908 |
| c1qtnf1 | 13689 | PDI_U | 0.0008846501316 | 0.0009632232602 | 0.9184268779 | 0.3584116205 |
| il20 | 13378 | PDI_U | -0.0009252423005 | 0.001008027613 | -0.9178739637 | 0.358701416 |
| cd3d | 11383 | PDI_U | -0.0009998149218 | 0.001089731882 | -0.9174870792 | 0.3589069195 |
| c2cd2l | 13303 | PDI_U | -0.0009277075388 | 0.001011390719 | -0.917259296 | 0.359023425 |
| sumf2 | 13784 | PDI_U | 0.0008935789238 | 0.0009746298164 | 0.9168393053 | 0.3592429071 |
| ddx1 | 11724 | PDI_U | -0.0009817014609 | 0.001071017467 | -0.9166063964 | 0.3593678043 |
| mapt | 13320 | PDI_U | -0.0009281822206 | 0.001013175669 | -0.9161118347 | 0.3596248412 |
| tjap1 | 13699 | PDI_U | 0.0009102983212 | 0.0009939669798 | 0.9158235029 | 0.3597756091 |
| psme2 | 13499 | PDI_U | -0.0008975463475 | 0.0009802728144 | -0.9156087309 | 0.3598885204 |
| palm3 | 11355 | PDI_U | -0.0009958608584 | 0.00108801972 | -0.9152966994 | 0.3600553484 |
| il1b | 13324 | PDI_U | -0.0009224870899 | 0.001007963792 | -0.9151986383 | 0.3601039366 |
| snap29 | 13699 | PDI_U | 0.0009080652223 | 0.0009926920345 | 0.914750185 | 0.3603389064 |
| cxcl10 | 13710 | PDI_U | 0.000860047466 | 0.000940474557 | 0.9144824382 | 0.3604794982 |
| zp3 | 11474 | PDI_U | -0.0009869237861 | 0.001080025139 | -0.9137970502 | 0.3608427178 |

| Protein | N_used | term | beta | se | t | p_val |
|---|---|---|---|---|---|---|
| ak1 | 13618 | PDI_U | 0.0008948900859 | 0.000979782138 | 0.9133561954 | 0.36107142 |
| bank1 | 13621 | PDI_U | 0.0009085759858 | 0.000995174802 | 0.9129813014 | 0.3612685492 |
| ndst1 | 11730 | PDI_U | 0.0009771692673 | 0.001071140207 | 0.9122701776 | 0.3616452767 |
| snap23 | 13618 | PDI_U | 0.0009096050978 | 0.0009973790841 | 0.9119953609 | 0.3617873177 |
| atp6v1g2 | 11355 | PDI_U | -0.0009924042999 | 0.001089633393 | -0.9107689856 | 0.3624364606 |
| lyar | 13308 | PDI_U | 0.0009207598759 | 0.001011180348 | 0.910579283 | 0.3625335974 |
| mertk | 13801 | PDI_U | 0.0008608641025 | 0.0009454293082 | 0.9105536449 | 0.3625465207 |
| preb | 13378 | PDI_U | -0.0009118490169 | 0.001001824033 | -0.9101888026 | 0.3627393615 |
| epo | 13621 | PDI_U | -0.0008949560287 | 0.000983746577 | -0.9097424577 | 0.3629744588 |
| gpa33 | 13579 | PDI_U | 0.0008948971351 | 0.0009860524564 | 0.9075553022 | 0.3641293383 |
| smad3 | 11730 | PDI_U | 0.0009722944386 | 0.001071987644 | 0.9070015347 | 0.3644246363 |
| serpina9 | 13523 | PDI_U | 0.0008919358874 | 0.000985034858 | 0.9054866233 | 0.3652237898 |
| eif1ax | 11767 | PDI_U | -0.0009643026449 | 0.001065670375 | -0.904878908 | 0.365548087 |
| lilrb1 | 13800 | PDI_U | -0.0008642649191 | 0.0009559499498 | -0.904090135 | 0.3659634045 |
| mapk13 | 11304 | PDI_U | 0.0009876797197 | 0.001092866928 | 0.9037511286 | 0.3661466605 |
| lilrb5 | 13800 | PDI_U | 0.0008902242708 | 0.0009874169447 | 0.9015687604 | 0.36730174 |
| rab3gap1 | 11383 | PDI_U | -0.0009825461782 | 0.001090061037 | -0.9013680382 | 0.3674117599 |
| edil3 | 13388 | PDI_U | -0.0008634757266 | 0.0009585094568 | -0.9008525899 | 0.3676829206 |
| septin3 | 11757 | PDI_U | -0.0009406808826 | 0.00104721959 | -0.89826517 | 0.3690626104 |
| bag3 | 13526 | PDI_U | 0.0008697558553 | 0.0009684907647 | 0.8980528127 | 0.3691733934 |
| icoslg | 13579 | PDI_U | 0.0008866517837 | 0.0009873672919 | 0.8979959039 | 0.3692036689 |
| fxn | 13753 | PDI_U | -0.0008893800398 | 0.0009912638819 | -0.8972182443 | 0.3696181908 |
| gabra4 | 11383 | PDI_U | -0.000977762991 | 0.001089965775 | -0.897058434 | 0.3697067286 |
| creld2 | 13691 | PDI_U | 0.0008819102171 | 0.000983903709 | 0.8963379332 | 0.3700880788 |
| plekho1 | 11767 | PDI_U | 0.0009543512615 | 0.001065528221 | 0.895660239 | 0.3704525923 |
| kazn | 11724 | PDI_U | 0.0009573842705 | 0.001069769525 | 0.8949444231 | 0.3708351895 |
| ap1g2 | 11730 | PDI_U | 0.0009545625077 | 0.001066719574 | 0.8948579655 | 0.3708813993 |
| sema3g | 11537 | PDI_U | 0.000959851417 | 0.001073931132 | 0.8937737142 | 0.371461639 |
| pla2g4a | 13710 | PDI_U | 0.0008886902568 | 0.0009968742312 | 0.8914768072 | 0.3726890871 |
| bid | 13621 | PDI_U | 0.0008791031313 | 0.0009879493267 | 0.8898261354 | 0.3735749799 |
| paip2b | 11271 | PDI_U | -0.0009735134875 | 0.001095398487 | -0.8887299911 | 0.3741671953 |
| numb | 11411 | PDI_U | 0.00095747121 | 0.001078893098 | 0.8874569797 | 0.3748516419 |
| pttg1 | 11570 | PDI_U | 0.0009580945948 | 0.001080032677 | 0.8870977846 | 0.3750447149 |
| bhmt2 | 11355 | PDI_U | -0.0009671587215 | 0.00109031105 | -0.8870484449 | 0.3750716258 |
| usp8 | 13388 | PDI_U | 0.000889349782 | 0.001002749621 | 0.8869111123 | 0.3751427042 |
| ptn | 13388 | PDI_U | -0.0008440405819 | 0.0009522601865 | -0.8863550046 | 0.3754421924 |
| myom2 | 11355 | PDI_U | -0.0009410848604 | 0.001062206332 | -0.8859718044 | 0.3756515029 |
| ctsv | 13717 | PDI_U | -0.0008274177562 | 0.0009340123324 | -0.8858745517 | 0.3757006738 |
| lpcat2 | 13579 | PDI_U | 0.0008761192931 | 0.0009904984579 | 0.8845236316 | 0.3764292864 |
| fuca1 | 13722 | PDI_U | 0.0008696963233 | 0.0009844283862 | 0.883453114 | 0.3770069952 |
| ccdc134 | 11730 | PDI_U | 0.0009456274953 | 0.001070397534 | 0.8834357942 | 0.3770189823 |
| mmp1 | 13139 | PDI_U | 0.0008910303459 | 0.001009896158 | 0.8822989758 | 0.3776313059 |
| siglec7 | 13618 | PDI_U | -0.0008299780185 | 0.0009428532848 | -0.8802833186 | 0.378721395 |
| tnfrsf14 | 13677 | PDI_U | 0.000839134152 | 0.0009547651932 | 0.8788905984 | 0.3794760506 |
| dok1 | 11767 | PDI_U | 0.0009389440843 | 0.001068739354 | 0.8785529237 | 0.3796616875 |
| ahsg | 11794 | PDI_U | -0.0009288780825 | 0.001058762305 | -0.877324474 | 0.3803283112 |
| ank2 | 11355 | PDI_U | -0.0009551106431 | 0.001088954865 | -0.8770892842 | 0.3804567199 |
| znf174 | 11411 | PDI_U | -0.0009510759622 | 0.001086648557 | -0.8752378643 | 0.381462936 |
| nfatc3 | 13378 | PDI_U | 0.0008733327939 | 0.0009980529201 | 0.87503656 | 0.3815697367 |
| cd63 | 13526 | PDI_U | 0.0008660170581 | 0.0009903647655 | 0.8744425167 | 0.3818928514 |
| trpv3 | 11355 | PDI_U | -0.0009527995305 | 0.001089918094 | -0.8741936992 | 0.3820312846 |
| lypla2 | 11383 | PDI_U | -0.0009514091018 | 0.001088952258 | -0.8736922073 | 0.3823043464 |
| eppk1 | 11474 | PDI_U | -0.0009425430951 | 0.001079590755 | -0.8730559157 | 0.3826508904 |
| gimap7 | 11537 | PDI_U | -0.0009277454059 | 0.001063075319 | -0.8726996003 | 0.3828450169 |
| rictor | 11411 | PDI_U | -0.0009475908606 | 0.001086143242 | -0.8724363636 | 0.3829887462 |
| cd33 | 13699 | PDI_U | -0.0008639763243 | 0.0009916144508 | -0.8712825066 | 0.3836152016 |
| pdrg1 | 11483 | PDI_U | -0.0009446483102 | 0.001084430591 | -0.8711007587 | 0.3837173698 |
| suox | 11757 | PDI_U | 0.0008877140257 | 0.001019151441 | 0.871032498 | 0.3837542121 |
| psrc1 | 13308 | PDI_U | 0.0008735047156 | 0.001005385657 | 0.8688255198 | 0.3849582548 |
| akr1c4 | 13388 | PDI_U | 0.0008720491941 | 0.001004572577 | 0.868079832 | 0.3853662047 |
| cd300c | 13784 | PDI_U | 0.0008300764677 | 0.000958328778 | 0.8661708661 | 0.3864115567 |
| pam | 13742 | PDI_U | -0.0008482630633 | 0.0009797105787 | -0.8658302582 | 0.386598382 |
| treml1 | 11794 | PDI_U | 0.0009259257335 | 0.001069839374 | 0.8654810771 | 0.3867924198 |
| irak4 | 13621 | PDI_U | 0.0008636311329 | 0.0009982840128 | 0.8651156603 | 0.3869905594 |
| stxbp3 | 13308 | PDI_U | -0.0008758015682 | 0.001012986922 | -0.8645734203 | 0.3872885638 |
| casp9 | 11411 | PDI_U | -0.0009364972219 | 0.001086435798 | -0.8619903941 | 0.3887109367 |
| omp | 11383 | PDI_U | -0.0009373726832 | 0.001090289974 | -0.8597462198 | 0.3899470756 |
| mmp13 | 13320 | PDI_U | 0.0008622189628 | 0.001003018325 | 0.859624337 | 0.3900116395 |
| pmvk | 13292 | PDI_U | 0.000868597605 | 0.001011601763 | 0.8586359142 | 0.3905569146 |
| rnase6 | 11698 | PDI_U | 0.0008338278014 | 0.0009718132019 | 0.8580124243 | 0.3909031998 |

| Protein | N_used | term | beta | se | t | p_val |
|---|---|---|---|---|---|---|
| rnase10 | 11474 | PDI_U | 0.0009181804781 | 0.001070657987 | 0.8575852323 | 0.3911394632 |
| pcdh9 | 11724 | PDI_U | 0.0008934302761 | 0.001042563788 | 0.8569550245 | 0.3914872749 |
| ankrd54 | 13308 | PDI_U | 0.0008607370494 | 0.00100470091 | 0.8567097344 | 0.3916207622 |
| comp | 13489 | PDI_U | -0.0008106262325 | 0.000947738714 | -0.8553267061 | 0.3923855121 |
| il1a | 13378 | PDI_U | 0.0008630460451 | 0.001010568631 | 0.8540202212 | 0.3931090908 |
| hla_dra | 13571 | PDI_U | -0.000848613421 | 0.0009942658117 | -0.8535075943 | 0.3933929556 |
| akap12 | 11537 | PDI_U | -0.0009171256365 | 0.001074565187 | -0.853485342 | 0.3934079508 |
| slamf8 | 13308 | PDI_U | -0.0008458945958 | 0.0009919217066 | -0.8527836321 | 0.3937946636 |
| tcp11 | 11570 | PDI_U | -0.0009195712642 | 0.001079115009 | -0.8521531595 | 0.3941467468 |
| pof1b | 11411 | PDI_U | -0.0009152451083 | 0.001074776148 | -0.8515681242 | 0.3944717268 |
| morc3 | 11767 | PDI_U | -0.0009108475627 | 0.001070017674 | -0.8512453438 | 0.3946504192 |
| rbp7 | 11724 | PDI_U | 0.0008693792101 | 0.001021496239 | 0.8510841025 | 0.3947400357 |
| tacc3 | 13579 | PDI_U | 0.0008510240342 | 0.001000959056 | 0.8502086365 | 0.3952241046 |
| foxo1 | 13621 | PDI_U | 0.0008482498291 | 0.0009984478235 | 0.8495685094 | 0.3955799707 |
| scrg1 | 11781 | PDI_U | 0.0007859661365 | 0.0009253078922 | 0.8494103889 | 0.395670252 |
| klk3 | 11790 | PDI_U | -0.0005650471725 | 0.0006654702904 | -0.8490945135 | 0.3958459604 |
| klk7 | 11253 | PDI_U | -0.0009042548704 | 0.001065805374 | -0.8484240107 | 0.3962199411 |
| rrm2b | 13308 | PDI_U | 0.0008554735736 | 0.001008590569 | 0.8481871631 | 0.396349014 |
| fmnl1 | 13320 | PDI_U | 0.0008384073135 | 0.0009887658478 | 0.8479331233 | 0.3964904664 |
| sptbn2 | 11383 | PDI_U | -0.0009241542109 | 0.001090078108 | -0.8477871484 | 0.3965743658 |
| ambn | 13378 | PDI_U | -0.0008524191722 | 0.001006674388 | -0.8467675171 | 0.3971398776 |
| gdf2 | 13618 | PDI_U | -0.0007706141885 | 0.000911222794 | -0.8456923966 | 0.3977392378 |
| cep350 | 11304 | PDI_U | 0.0009259873612 | 0.001095106788 | 0.8455680953 | 0.3978116466 |
| dkk4 | 13530 | PDI_U | 0.0008272535365 | 0.0009785506312 | 0.8453865442 | 0.3979100174 |
| dand5 | 11411 | PDI_U | -0.0009182593425 | 0.001087048257 | -0.8447273035 | 0.39828084 |
| cert | 13320 | PDI_U | 0.0008536414819 | 0.001010854613 | 0.8444750325 | 0.3984191899 |
| treml2 | 13632 | PDI_U | 0.0008247588962 | 0.000976659623 | 0.8444691239 | 0.3984221425 |
| pdp1 | 13308 | PDI_U | 0.000854338965 | 0.001011939993 | 0.844258524 | 0.3985401473 |
| prkcq | 13378 | PDI_U | -0.0008527114933 | 0.001010475006 | -0.8438719295 | 0.398756078 |
| defb104a_defb104b | 11383 | PDI_U | 0.0008573465722 | 0.00101601817 | 0.8438299605 | 0.398782182 |
| camlg | 11483 | PDI_U | -0.0009126012055 | 0.001082729906 | -0.8428705997 | 0.3993183919 |
| rad23b | 13308 | PDI_U | 0.0008526590108 | 0.001012629225 | 0.8420248889 | 0.3997891669 |
| il9 | 11570 | PDI_U | -0.0009087742254 | 0.001080100621 | -0.8413792271 | 0.4001529261 |
| il12rb1 | 13378 | PDI_U | -0.000835868585 | 0.0009941295457 | -0.8408044894 | 0.4004725126 |
| dnaja1 | 11304 | PDI_U | -0.0009203568984 | 0.001095259372 | -0.8403095397 | 0.4007526457 |
| thpo | 13388 | PDI_U | 0.0008437032257 | 0.001004524949 | 0.8399027091 | 0.4009779502 |
| b3gat3 | 11355 | PDI_U | -0.0009143972095 | 0.001089771004 | -0.8390728019 | 0.4014461399 |
| lrfn2 | 11483 | PDI_U | -0.000907520169 | 0.001083053515 | -0.8379273564 | 0.4020889619 |
| prdx6 | 13753 | PDI_U | 0.0008320371735 | 0.0009929937426 | 0.837907771 | 0.402097078 |
| aplp1 | 13618 | PDI_U | 0.0008032857931 | 0.0009587978569 | 0.8378051613 | 0.4021548561 |
| nucb2 | 13730 | PDI_U | 0.0008154638916 | 0.0009734298616 | 0.8377222887 | 0.4022012871 |
| clspn | 13303 | PDI_U | -0.0008488594825 | 0.001013505342 | -0.8375481087 | 0.402299607 |
| nit2 | 11730 | PDI_U | 0.0008844262773 | 0.001057280784 | 0.8365103112 | 0.4028849364 |
| sh3bgrl2 | 11383 | PDI_U | -0.0009112583141 | 0.001089551495 | -0.8363609417 | 0.4029694535 |
| eif4ebp1 | 13618 | PDI_U | 0.0008218423398 | 0.0009835903798 | 0.8355534546 | 0.4034208352 |
| serpinc1 | 11790 | PDI_U | -0.0008725447163 | 0.001044586495 | -0.8353015476 | 0.4035648897 |
| bap18 | 11767 | PDI_U | 0.0008576198949 | 0.001028608111 | 0.8337673846 | 0.4044290193 |
| ikzf2 | 13308 | PDI_U | 0.0008428970733 | 0.001011151442 | 0.8336012178 | 0.4045207139 |
| mme | 13579 | PDI_U | 0.0007910938304 | 0.0009491996108 | 0.8334325272 | 0.4046155086 |
| ctsl | 13618 | PDI_U | 0.0008028628157 | 0.0009641499707 | 0.8327156979 | 0.4050197051 |
| ap2b1 | 11483 | PDI_U | -0.0009006171027 | 0.001081736154 | -0.8325663326 | 0.4051066862 |
| prelp | 13658 | PDI_U | -0.0007810180038 | 0.0009384150398 | -0.8322735364 | 0.4052691289 |
| echs1 | 11474 | PDI_U | -0.0008898036171 | 0.001069135898 | -0.832264279 | 0.405277128 |
| clec12a | 11411 | PDI_U | -0.0008987794946 | 0.001080739407 | -0.8316338695 | 0.4056330587 |
| mrpl58 | 11757 | PDI_U | -0.00088681651 | 0.001066645816 | -0.8315530262 | 0.4056781911 |
| spred2 | 11411 | PDI_U | -0.0009022958482 | 0.001086036202 | -0.8308156274 | 0.4060951941 |
| hpgds | 13308 | PDI_U | 0.0008388116207 | 0.001010521156 | 0.830078238 | 0.4065094537 |
| nagk | 11450 | PDI_U | 0.0008836985881 | 0.001065002059 | 0.8297623282 | 0.4066904908 |
| sell | 11817 | PDI_U | -0.0008544647563 | 0.001030138533 | -0.8294658716 | 0.4068576127 |
| nxph3 | 11411 | PDI_U | -0.0008595261884 | 0.001036554308 | -0.8292148149 | 0.4070002265 |
| il16 | 13801 | PDI_U | 0.0007733699416 | 0.0009327616634 | 0.8291184898 | 0.407051714 |
| tnfrsf13c | 13378 | PDI_U | -0.0008245093974 | 0.0009946242085 | -0.8289657445 | 0.4071385943 |
| ptgr1 | 11781 | PDI_U | -0.0008404316812 | 0.001014508797 | -0.8284124139 | 0.4074537776 |
| blnk | 11411 | PDI_U | -0.0008863317115 | 0.001070371498 | -0.8280598962 | 0.4076539159 |
| ahsp | 13597 | PDI_U | 0.0007935095362 | 0.0009607150997 | 0.8259571817 | 0.4088428863 |
| src | 13672 | PDI_U | 0.0008192211665 | 0.0009974062608 | 0.8213515382 | 0.4114603934 |
| lilrb2 | 13727 | PDI_U | -0.0007982324488 | 0.0009720130239 | -0.8212157956 | 0.4115376349 |
| pear1 | 13800 | PDI_U | -0.0008112891192 | 0.0009920211449 | -0.8178143413 | 0.4134773398 |
| il6st | 13618 | PDI_U | -0.0008060226614 | 0.0009874416404 | -0.8162737203 | 0.4143578918 |
| impact | 11767 | PDI_U | 0.0008653463703 | 0.001060729682 | 0.8158029182 | 0.4146293938 |

| Protein | N_used | term | beta | se | t | p_val |
|---|---|---|---|---|---|---|
| lilra5 | 13599 | PDI_U | 0.0007657132283 | 0.0009390406013 | 0.8154207893 | 0.4148457819 |
| folh1 | 11411 | PDI_U | 0.0008824259148 | 0.00108225401 | 0.8153593394 | 0.4148836872 |
| tnfrsf1a | 13632 | PDI_U | -0.0007261375156 | 0.0008907483606 | -0.8151993848 | 0.4149724444 |
| mgll | 13691 | PDI_U | 0.0008131481638 | 0.0009974993541 | 0.815186657 | 0.4149796671 |
| il4 | 13378 | PDI_U | -0.0008232420528 | 0.001010661542 | -0.814557613 | 0.4153400938 |
| pdzk1 | 11411 | PDI_U | 0.0008427178491 | 0.001036190292 | 0.8132848333 | 0.4160717571 |
| glrx5 | 11537 | PDI_U | -0.0008766617663 | 0.001078629569 | -0.812755177 | 0.4163752273 |
| apom | 13669 | PDI_U | 0.0007798381942 | 0.0009598277372 | 0.8124772435 | 0.4165320013 |
| cd207 | 13340 | PDI_U | 0.0008117527679 | 0.0009993801825 | 0.8122562185 | 0.4166591341 |
| lmnb2 | 11767 | PDI_U | 0.0008046606624 | 0.0009906567941 | 0.8122496784 | 0.4166648272 |
| arnt | 13378 | PDI_U | -0.0008188188057 | 0.00100819919 | -0.8121597538 | 0.4167144336 |
| sncg | 13552 | PDI_U | 0.000734238578 | 0.0009049260475 | 0.8113796481 | 0.4171619455 |
| tor1aip1 | 11474 | PDI_U | 0.0008743004787 | 0.001077750288 | 0.8112273207 | 0.4172519834 |
| hnf1a | 11483 | PDI_U | 0.0008797027286 | 0.001084427201 | 0.8112141858 | 0.4172595116 |
| il1r2 | 13635 | PDI_U | -0.000791338537 | 0.0009755286831 | -0.8111894101 | 0.417271078 |
| ppm1a | 13308 | PDI_U | -0.0008223292846 | 0.001013968796 | -0.811000583 | 0.4173798525 |
| fgf12 | 11411 | PDI_U | 0.0008795184857 | 0.001084674791 | 0.8108591564 | 0.4174634869 |
| fos | 11271 | PDI_U | -0.0008849705294 | 0.001095247353 | -0.8080097402 | 0.419102046 |
| lilra6 | 11781 | PDI_U | -0.000859626999 | 0.001064166185 | -0.8077939434 | 0.4192255386 |
| clec10a | 13552 | PDI_U | 0.0008006578166 | 0.0009913283058 | 0.807661611 | 0.4192995969 |
| tspan1 | 13388 | PDI_U | 0.0007638218806 | 0.0009465214718 | 0.8069778694 | 0.4196935818 |
| cd14 | 13353 | PDI_U | -0.0007858942233 | 0.0009761793598 | -0.8050715429 | 0.4207927436 |
| fzd8 | 11383 | PDI_U | -0.0008760111301 | 0.001089168772 | -0.8042932855 | 0.421244429 |
| draxin | 13552 | PDI_U | 0.000786305132 | 0.0009778528572 | 0.8041139586 | 0.4213452811 |
| bex3 | 11304 | PDI_U | -0.0008784103503 | 0.001093923175 | -0.802990896 | 0.4219968974 |
| dcdc2c | 11304 | PDI_U | -0.0008780088765 | 0.001094708778 | -0.8020478998 | 0.4225421291 |
| jmjd1c | 11304 | PDI_U | -0.000877534855 | 0.001094524943 | -0.8017495264 | 0.4227147317 |
| chit1 | 13687 | PDI_U | -0.0007672930238 | 0.0009574376579 | -0.8014025952 | 0.4229125375 |
| hla_e | 13621 | PDI_U | -0.0007725709265 | 0.0009658596043 | -0.799879116 | 0.4237948002 |
| cpe | 13649 | PDI_U | 0.0007891340875 | 0.0009876907472 | 0.7989687964 | 0.4243224203 |
| espl1 | 11304 | PDI_U | -0.0008743093594 | 0.00109461578 | -0.798736301 | 0.4244601318 |
| rpgr | 11483 | PDI_U | -0.0008659070568 | 0.001085007208 | -0.7980657182 | 0.424848875 |
| btn2a1 | 13710 | PDI_U | 0.0007614871409 | 0.0009552380117 | 0.7971700577 | 0.4253660904 |
| flrt2 | 13552 | PDI_U | -0.0007888700797 | 0.000990767171 | -0.7962214563 | 0.425917288 |
| il20ra | 13378 | PDI_U | -0.0008039455302 | 0.00101105032 | -0.795158771 | 0.4265352708 |
| lrch4 | 11355 | PDI_U | 0.0008498470851 | 0.001069888456 | 0.7943324188 | 0.4270185538 |
| crx | 13388 | PDI_U | -0.0008013555014 | 0.0010099428 | -0.7934662256 | 0.4275203142 |
| myl6b | 11483 | PDI_U | 0.0008588183548 | 0.001082780226 | 0.793160361 | 0.4277008036 |
| pla2g15 | 13699 | PDI_U | 0.0007591452262 | 0.0009609829224 | 0.7899674475 | 0.4295604651 |
| glod4 | 13627 | PDI_U | 0.0007838777182 | 0.0009928625081 | 0.7895128599 | 0.4298260655 |
| igdcc4 | 11790 | PDI_U | -0.0007856684018 | 0.0009952553589 | -0.7894138874 | 0.4298860327 |
| il11 | 13378 | PDI_U | 0.0007962367157 | 0.001009082738 | 0.7890698012 | 0.430085207 |
| cd46 | 13545 | PDI_U | 0.0007703838959 | 0.0009786974071 | 0.7871522805 | 0.4312065128 |
| calcoco2 | 11474 | PDI_U | 0.0008508542519 | 0.001081783386 | 0.7865292286 | 0.4315737631 |
| prtfdc1 | 13320 | PDI_U | 0.0007963040972 | 0.001012514545 | 0.786461885 | 0.4316109442 |
| klf4 | 11383 | PDI_U | 0.000837570227 | 0.001065265328 | 0.7862550342 | 0.4317344743 |
| pdcd5 | 13784 | PDI_U | 0.0007679868221 | 0.0009769925853 | 0.7860723139 | 0.4318386475 |
| vwa5a | 11304 | PDI_U | -0.000858700726 | 0.001093622382 | -0.7851894219 | 0.4323589899 |
| mprip | 11710 | PDI_U | -0.0008370072392 | 0.001066905129 | -0.7845189007 | 0.432751582 |
| cep112 | 11355 | PDI_U | 0.0008507666039 | 0.001085314068 | 0.7838897783 | 0.4331211563 |
| hsdl2 | 11474 | PDI_U | -0.0008326147158 | 0.001062555785 | -0.7835962377 | 0.4332932554 |
| samd9l | 13621 | PDI_U | 0.0007813872802 | 0.0009980947297 | 0.7828788761 | 0.4337118658 |
| ryr1 | 11355 | PDI_U | -0.0008511131306 | 0.001089943881 | -0.7808779381 | 0.4348905849 |
| muc16 | 13579 | PDI_U | 0.0007812732235 | 0.001000922912 | 0.7805528421 | 0.4350791551 |
| cndp1 | 13689 | PDI_U | -0.0007665978652 | 0.0009833004445 | -0.7796174418 | 0.4356295765 |
| rnase3 | 13543 | PDI_U | 0.0007735347921 | 0.0009931033312 | 0.7789066533 | 0.4360483258 |
| blvrb | 13632 | PDI_U | -0.00076130415 | 0.0009787010458 | -0.7778720104 | 0.4366579821 |
| polr2a | 11411 | PDI_U | -0.0008443043456 | 0.001086446901 | -0.7771243536 | 0.4371015334 |
| cela2a | 11790 | PDI_U | 0.0008192385563 | 0.001054962327 | 0.7765571675 | 0.4374356749 |
| pdlim5 | 11757 | PDI_U | 0.0008302349939 | 0.001069427802 | 0.7763357115 | 0.4375664258 |
| dars1 | 13535 | PDI_U | 0.0007762059964 | 0.00100244214 | 0.7743150105 | 0.4387580663 |
| gdnf | 13320 | PDI_U | 0.0007492405717 | 0.0009691118827 | 0.7731208182 | 0.439464613 |
| bach1 | 13621 | PDI_U | 0.0007695643928 | 0.0009968132816 | 0.7720246179 | 0.4401132529 |
| adra2a | 11355 | PDI_U | -0.0008396877759 | 0.001088122092 | -0.771685257 | 0.4403169404 |
| prok1 | 13621 | PDI_U | 0.0005786270449 | 0.0007499195646 | 0.7715854769 | 0.4403733757 |
| cela3a | 13779 | PDI_U | 0.0007591471688 | 0.0009840048545 | 0.7714872191 | 0.4404314365 |
| nacc1 | 11730 | PDI_U | 0.0008174034286 | 0.001063084124 | 0.7688981616 | 0.4419692654 |
| dnajb1 | 13579 | PDI_U | 0.0007598423771 | 0.0009886283947 | 0.7685823927 | 0.4421546396 |
| acta2 | 13388 | PDI_U | 0.0006858770119 | 0.0008931926995 | 0.7678936609 | 0.4425639191 |
| klk15 | 11706 | PDI_U | -0.0008179532798 | 0.001066020222 | -0.7672962136 | 0.4429209102 |

| Protein | N_used | term | beta | se | t | p_val |
|---|---|---|---|---|---|---|
| slc4a1 | 11790 | PDI_U | -0.0008130652219 | 0.00105971369 | -0.7672498991 | 0.4429483296 |
| cxcl5 | 13571 | PDI_U | 0.0007503479856 | 0.0009792312328 | 0.7662623091 | 0.4435335797 |
| tnfsf8 | 11790 | PDI_U | 0.0008105623866 | 0.001058277552 | 0.7659260892 | 0.4437356304 |
| tnfaip2 | 11724 | PDI_U | -0.0008110549494 | 0.001060236378 | -0.7649755904 | 0.4443014933 |
| fstl1 | 11474 | PDI_U | -0.0008167815111 | 0.001067911067 | -0.7648403843 | 0.4443823432 |
| prdx5 | 13801 | PDI_U | 0.0007567786249 | 0.0009898527999 | 0.7645365301 | 0.4445606591 |
| sat2 | 11570 | PDI_U | -0.0008190091326 | 0.001071763489 | -0.7641696523 | 0.4447817488 |
| dffa | 13801 | PDI_U | 0.0007535682736 | 0.0009891268443 | 0.7618520091 | 0.4461613655 |
| lcn15 | 11757 | PDI_U | -0.0008042570783 | 0.001056619137 | -0.7611608098 | 0.4465763046 |
| rab6b | 13320 | PDI_U | -0.0007701523286 | 0.001013356077 | -0.7600016871 | 0.4472670396 |
| arhgap5 | 11304 | PDI_U | 0.0008311666462 | 0.001094943799 | 0.7590952585 | 0.4478114202 |
| rapgef2 | 11570 | PDI_U | -0.0008182355267 | 0.001078279406 | -0.7588344187 | 0.4479670877 |
| cyb5a | 11304 | PDI_U | 0.0008301176144 | 0.001094361868 | 0.7585403317 | 0.4481434094 |
| mecr | 11474 | PDI_U | -0.0008169699715 | 0.001079112329 | -0.7570759314 | 0.4490199348 |
| c1qtnf9 | 11520 | PDI_U | -0.0008156187764 | 0.001078244319 | -0.7564322502 | 0.4494055627 |
| alms1 | 11570 | PDI_U | 0.0008162267558 | 0.001080130438 | 0.7556742472 | 0.4498599278 |
| cd70 | 13621 | PDI_U | -0.0007193540604 | 0.0009521586634 | -0.7554980993 | 0.4499632477 |
| cd72 | 11537 | PDI_U | 0.0007720821813 | 0.001022001822 | 0.7554606698 | 0.4499880612 |
| pth | 11483 | PDI_U | 0.0007909723177 | 0.001047608983 | 0.7550262839 | 0.4502487128 |
| atxn2 | 11383 | PDI_U | -0.0008171151682 | 0.001085924464 | -0.7524604107 | 0.4517898051 |
| wnt9a | 13378 | PDI_U | -0.0006806794506 | 0.0009056154527 | -0.7516208437 | 0.452292339 |
| ca14 | 13753 | PDI_U | -0.000670987069 | 0.0008927575158 | -0.7515893813 | 0.4523109033 |
| axin1 | 13621 | PDI_U | 0.0007477025627 | 0.0009952604707 | 0.7512631966 | 0.4525072643 |
| leo1 | 11757 | PDI_U | 0.0007982442333 | 0.001063146551 | 0.7508317954 | 0.4527689338 |
| ggt5 | 13552 | PDI_U | -0.0007432132239 | 0.0009911578533 | -0.7498434497 | 0.4533620249 |
| cd27 | 13699 | PDI_U | -0.0007214194462 | 0.0009624765507 | -0.7495449584 | 0.453541694 |
| ppme1 | 13579 | PDI_U | 0.0007454885972 | 0.0009947965831 | 0.7493879753 | 0.4536363896 |
| epha2 | 13129 | PDI_U | 0.0007184761192 | 0.0009595462083 | 0.7487665659 | 0.4540113407 |
| gprc5c | 11730 | PDI_U | -0.0007957999091 | 0.001063637274 | -0.7481873081 | 0.4543622008 |
| ctsc | 13619 | PDI_U | 0.0007265646817 | 0.0009723192393 | 0.7472491054 | 0.4549261198 |
| dnajc9 | 11724 | PDI_U | -0.0007923404294 | 0.001060894469 | -0.7468607414 | 0.455162618 |
| acp5 | 13685 | PDI_U | 0.0007117739777 | 0.0009549603523 | 0.7453440093 | 0.4560765945 |
| btla | 11304 | PDI_U | -0.0008156941982 | 0.001094435801 | -0.7453102299 | 0.4560997108 |
| itga2 | 11537 | PDI_U | 0.0007764392782 | 0.001041831953 | 0.7452634525 | 0.4561276684 |
| kcnip4 | 13320 | PDI_U | 0.0007529013273 | 0.001011463234 | 0.7443684571 | 0.4566667397 |
| il13 | 13378 | PDI_U | 0.0007518887941 | 0.001010424257 | 0.7441317729 | 0.4568098405 |
| afap1 | 11537 | PDI_U | 0.0007987241689 | 0.001075412742 | 0.7427140651 | 0.4576699501 |
| serpinh1 | 11570 | PDI_U | 0.0007972655245 | 0.001073667912 | 0.7425624959 | 0.4577616925 |
| galnt7 | 13579 | PDI_U | -0.000736054021 | 0.0009924372433 | -0.7416630381 | 0.4583043577 |
| rab27b | 11781 | PDI_U | 0.0007899038947 | 0.001065891129 | 0.7410737112 | 0.4586635341 |
| efna1 | 13602 | PDI_U | -0.0007051101149 | 0.0009532936565 | -0.7396567785 | 0.4595210564 |
| gid8 | 11757 | PDI_U | -0.0007914530149 | 0.001070499238 | -0.7393307595 | 0.4597209512 |
| pklr | 13676 | PDI_U | -0.0007352564902 | 0.0009945211568 | -0.7393070375 | 0.4597332769 |
| ldlrap1 | 11757 | PDI_U | 0.000786490936 | 0.001065611126 | 0.7380656198 | 0.4604893207 |
| cabp2 | 11483 | PDI_U | 0.0007986841245 | 0.001083407884 | 0.7371961535 | 0.4610181501 |
| heg1 | 11537 | PDI_U | -0.0007530477171 | 0.001021563664 | -0.7371520184 | 0.4610449146 |
| hepacam2 | 11767 | PDI_U | 0.000777833578 | 0.001057261661 | 0.7357058394 | 0.4619244142 |
| ccdc28a | 11483 | PDI_U | 0.0007976874776 | 0.001085026636 | 0.7351777835 | 0.4622462574 |
| fkbp5 | 13552 | PDI_U | 0.0007365464583 | 0.001001891275 | 0.7351560759 | 0.462257179 |
| grap2 | 13618 | PDI_U | 0.0007358458934 | 0.001001067827 | 0.7350609758 | 0.4623150286 |
| il25 | 11252 | PDI_U | 0.0008061468552 | 0.001097311247 | 0.7346565138 | 0.4625640367 |
| erbb2 | 13699 | PDI_U | 0.0006761246186 | 0.0009209143184 | 0.7341884093 | 0.4628464935 |
| manf | 13710 | PDI_U | 0.0007287942141 | 0.0009956436373 | 0.7319829975 | 0.4641914684 |
| fadd | 13618 | PDI_U | 0.0007321506406 | 0.001000759639 | 0.7315948924 | 0.4644284664 |
| becn1 | 11474 | PDI_U | 0.0007844109335 | 0.001072713077 | 0.7312402083 | 0.4646473927 |
| prtg | 13753 | PDI_U | 0.0007207716495 | 0.0009861393743 | 0.7309024143 | 0.4648512235 |
| mad1l1 | 13521 | PDI_U | 0.0006854631011 | 0.0009384442633 | 0.7304249469 | 0.4651431408 |
| hdgfl2 | 11767 | PDI_U | 0.0007684855875 | 0.00105253007 | 0.7301317174 | 0.4653242255 |
| apoa1 | 11817 | PDI_U | 0.0007197988431 | 0.0009861872089 | 0.7298805304 | 0.4654776967 |
| icam3 | 13800 | PDI_U | -0.0007186716955 | 0.0009854744511 | -0.7292646651 | 0.4658521697 |
| lgals1 | 13618 | PDI_U | 0.0006674404455 | 0.0009171276324 | 0.7277508843 | 0.4667786184 |
| cd59 | 13130 | PDI_U | -0.0006434874911 | 0.0008854624195 | -0.7267247903 | 0.4674075314 |
| mcee | 11757 | PDI_U | -0.0007768686132 | 0.001069887009 | -0.726122111 | 0.4677783846 |
| scn2b | 11483 | PDI_U | -0.000787064303 | 0.001084107414 | -0.7260021404 | 0.4678522705 |
| hras | 11549 | PDI_U | 0.0007839087099 | 0.001080347451 | 0.7256079599 | 0.4680938581 |
| icam4 | 13621 | PDI_U | 0.0007205755354 | 0.000993530855 | 0.7252673954 | 0.4683004744 |
| dmd | 11474 | PDI_U | -0.0007740884739 | 0.001068752188 | -0.7242918258 | 0.4689013774 |
| fh | 11383 | PDI_U | 0.0007878833222 | 0.001088659563 | 0.7237187357 | 0.4692533161 |
| il13ra2 | 11383 | PDI_U | -0.000787591848 | 0.001090225068 | -0.7224121618 | 0.4700559683 |
| pcsk9 | 13800 | PDI_U | -0.0006878213277 | 0.0009521657698 | -0.7223756088 | 0.4700758302 |

| Protein | N_used | term | beta | se | t | p_val |
|---|---|---|---|---|---|---|
| morf4l1 | 11570 | PDI_U | 0.0007797004102 | 0.001079471571 | 0.7222982345 | 0.4701257515 |
| max | 13320 | PDI_U | 0.0007268206767 | 0.001007875654 | 0.7211412177 | 0.4708353018 |
| gsap | 13308 | PDI_U | 0.0007287653475 | 0.001012684368 | 0.7196372047 | 0.4717610475 |
| hsd11b1 | 13621 | PDI_U | 0.0006829804519 | 0.000949164897 | 0.7195593243 | 0.47180872 |
| efnb2 | 11483 | PDI_U | -0.000777449563 | 0.001082252473 | -0.7183624733 | 0.4725484499 |
| bsnd | 11304 | PDI_U | -0.0007864362685 | 0.001094861421 | -0.7182975429 | 0.4725887061 |
| dnm1 | 11724 | PDI_U | -0.0007681320697 | 0.001069546653 | -0.718184726 | 0.4726577201 |
| tomm20 | 11781 | PDI_U | 0.0007588837999 | 0.0010573268 | 0.7177381675 | 0.4729329908 |
| casp1 | 13320 | PDI_U | 0.0007205098263 | 0.001003881599 | 0.7177239096 | 0.4729401361 |
| ctnna1 | 11383 | PDI_U | 0.0007811456042 | 0.001089467075 | 0.7169978997 | 0.4733901195 |
| mmp3 | 13632 | PDI_U | -0.0005479003688 | 0.0007659728549 | -0.7152999812 | 0.4744359614 |
| doc2b | 11483 | PDI_U | -0.0007759731812 | 0.00108490096 | -0.715247944 | 0.4744704054 |
| eloa | 13579 | PDI_U | 0.0006875404976 | 0.0009619632863 | 0.7147263387 | 0.4747904569 |
| cpxm2 | 11781 | PDI_U | 0.0007201814409 | 0.001008085284 | 0.7144052715 | 0.4749907822 |
| cdh6 | 13618 | PDI_U | -0.0007039693713 | 0.0009863559086 | -0.7137072584 | 0.4754204592 |
| shd | 11332 | PDI_U | -0.0007779894368 | 0.001090468381 | -0.7134452043 | 0.475585017 |
| stam | 11730 | PDI_U | -0.0007634284016 | 0.001071274289 | -0.7126357921 | 0.4760853471 |
| cst5 | 13390 | PDI_U | -0.0006926166036 | 0.0009721718756 | -0.7124425433 | 0.4762032039 |
| sap18 | 11570 | PDI_U | -0.0007679986183 | 0.001080124462 | -0.711027891 | 0.4770813005 |
| klrc1 | 11383 | PDI_U | 0.0007738012206 | 0.001090118942 | 0.7098319189 | 0.4778229284 |
| fcn1 | 11763 | PDI_U | -0.0007356704013 | 0.001036550665 | -0.7097293226 | 0.4778860873 |
| gmfg | 11781 | PDI_U | 0.0007570236323 | 0.001066647861 | 0.7097221677 | 0.4778905034 |
| mfge8 | 13632 | PDI_U | 0.0006888388207 | 0.0009756190701 | 0.7060530506 | 0.4801672236 |
| lonp1 | 11474 | PDI_U | -0.0007596741508 | 0.001077932204 | -0.7047513269 | 0.4809793229 |
| inpp1 | 13579 | PDI_U | 0.0007028369836 | 0.0009979657107 | 0.7042696718 | 0.4812769376 |
| ccl24 | 13691 | PDI_U | -0.0006914962833 | 0.0009819754148 | -0.7041889978 | 0.4813270692 |
| kcnc4 | 11383 | PDI_U | -0.0007661846 | 0.001089960414 | -0.7031770269 | 0.4819598304 |
| map2 | 11767 | PDI_U | 0.0007481217535 | 0.001063924326 | 0.7031719599 | 0.4819625174 |
| tet2 | 11549 | PDI_U | -0.0007601299666 | 0.001081097996 | -0.7031092183 | 0.4820018754 |
| pde5a | 11537 | PDI_U | 0.0007593010241 | 0.001080518486 | 0.7027191425 | 0.4822449889 |
| hnmt | 13486 | PDI_U | 0.0006428321354 | 0.0009156459273 | 0.7020531803 | 0.4826581258 |
| ifi30 | 11446 | PDI_U | -0.0006880411066 | 0.0009817747586 | -0.7008136037 | 0.4834336081 |
| dhrs4l2 | 11767 | PDI_U | -0.0007489665612 | 0.001068742023 | -0.7007926562 | 0.4834462927 |
| erbb3 | 13613 | PDI_U | 0.0006599525555 | 0.000942598039 | 0.7001420842 | 0.4838505515 |
| scly | 13715 | PDI_U | 0.0006435152695 | 0.0009199889244 | 0.6994815399 | 0.4842630187 |
| itih5 | 11730 | PDI_U | -0.0007488467162 | 0.00107103423 | -0.6991809367 | 0.4844528399 |
| gadd45b | 11570 | PDI_U | -0.000754440723 | 0.001079912281 | -0.6986129671 | 0.4848079939 |
| tnfrsf10c | 13677 | PDI_U | -0.0006926081146 | 0.0009914281852 | -0.6985963531 | 0.484816212 |
| cdc27 | 13308 | PDI_U | 0.0007059682497 | 0.001010554811 | 0.6985947142 | 0.4848175664 |
| gad2 | 11411 | PDI_U | -0.0007557175182 | 0.001085025995 | -0.6964971548 | 0.4861317403 |
| rnf41 | 13308 | PDI_U | 0.0007006127572 | 0.001006185877 | 0.6963054969 | 0.4862497031 |
| kdm3a | 11411 | PDI_U | -0.0007521854552 | 0.001082303255 | -0.6949858572 | 0.4870783306 |
| top2b | 11411 | PDI_U | 0.00074308992 | 0.001069353231 | 0.6948965958 | 0.4871342699 |
| nfu1 | 11724 | PDI_U | -0.0007418041867 | 0.001068891077 | -0.6939941799 | 0.4876996231 |
| col18a1 | 13692 | PDI_U | 0.0006386874535 | 0.0009221212956 | 0.6926284606 | 0.4885545019 |
| timd4 | 13689 | PDI_U | 0.0006674043456 | 0.0009642528421 | 0.6921466201 | 0.4888570071 |
| lgals7_lgals7b | 13602 | PDI_U | -0.0006536775546 | 0.0009466903348 | -0.6904871958 | 0.4898996514 |
| sil1 | 11474 | PDI_U | 0.0007417771078 | 0.001074645337 | 0.6902529441 | 0.4900491099 |
| spring1 | 11304 | PDI_U | -0.0007542834008 | 0.001092845831 | -0.6902011058 | 0.4900819131 |
| c2 | 13669 | PDI_U | -0.000656929831 | 0.0009521322629 | -0.6899564867 | 0.4902332755 |
| sox9 | 11483 | PDI_U | -0.0007432756657 | 0.001078151787 | -0.6893979814 | 0.4905867981 |
| klrk1 | 11767 | PDI_U | -0.0007344344276 | 0.001065474399 | -0.6893027444 | 0.4906463769 |
| stau1 | 11304 | PDI_U | -0.000753036163 | 0.00109291433 | -0.6890166432 | 0.4908269507 |
| dync1h1 | 11730 | PDI_U | -0.0007383517149 | 0.001071611769 | -0.6890104571 | 0.4908303288 |
| chac2 | 13308 | PDI_U | 0.0006928454609 | 0.001007757922 | 0.6875117982 | 0.4917722608 |
| dbnl | 13710 | PDI_U | 0.0006836770111 | 0.000994774889 | 0.6872680631 | 0.4919254559 |
| mmp10 | 13630 | PDI_U | -0.0006778764672 | 0.0009874196132 | -0.6865130671 | 0.4924013159 |
| adamts8 | 13699 | PDI_U | -0.0006735208787 | 0.0009812046088 | -0.6864224573 | 0.492458375 |
| slc44a4 | 11483 | PDI_U | 0.0007440259452 | 0.00108472405 | 0.685912648 | 0.4927820563 |
| uxs1 | 13308 | PDI_U | -0.0006836418809 | 0.0009973469279 | -0.6854604569 | 0.4930653556 |
| lcp1 | 11790 | PDI_U | 0.000713911544 | 0.001042209622 | 0.684998036 | 0.4933586402 |
| ccer2 | 11474 | PDI_U | 0.0007268947206 | 0.001063187718 | 0.6836936775 | 0.4941824374 |
| rhoc | 13477 | PDI_U | 0.0006843844496 | 0.001005404465 | 0.6807056002 | 0.4960694968 |
| cd244 | 13801 | PDI_U | -0.0006664664732 | 0.0009816756868 | -0.6789069773 | 0.4972081989 |
| lta4h | 13753 | PDI_U | -0.0006579137423 | 0.0009692545114 | -0.6787832655 | 0.4972866303 |
| spon2 | 13556 | PDI_U | 0.000650611534 | 0.0009593355748 | 0.6781897296 | 0.4976629898 |
| ccn3 | 13777 | PDI_U | 0.0006054854411 | 0.0008932686972 | 0.6778312539 | 0.4978900853 |
| thap12 | 11271 | PDI_U | 0.0007420996976 | 0.001095686964 | 0.6772917101 | 0.4982348055 |
| palm | 11643 | PDI_U | 0.0006512115039 | 0.0009650118664 | 0.6748222759 | 0.4998020993 |
| cd2 | 11355 | PDI_U | -0.0007328175715 | 0.001086314679 | -0.6745905083 | 0.4999497135 |

| Protein | N_used | term | beta | se | t | p_val |
|---|---|---|---|---|---|---|
| tmem132a | 11474 | PDI_U | 0.0007293543876 | 0.001081838307 | 0.6741805898 | 0.5002101044 |
| man2b2 | 11781 | PDI_U | 0.0006950743509 | 0.001031388119 | 0.6739212312 | 0.5003746287 |
| dusp3 | 13320 | PDI_U | -0.0006810451159 | 0.001012891288 | -0.6723773063 | 0.5013551997 |
| hyou1 | 13800 | PDI_U | 0.0006577361367 | 0.0009786012447 | 0.672118639 | 0.5015194334 |
| carhsp1 | 13320 | PDI_U | 0.0006656462524 | 0.0009950759778 | 0.6689401284 | 0.5035452745 |
| cbln1 | 11383 | PDI_U | -0.0007279018733 | 0.001089069599 | -0.6683703903 | 0.503910758 |
| ensa | 11730 | PDI_U | -0.0007155472069 | 0.001071196694 | -0.6679886253 | 0.5041540094 |
| rap1a | 11411 | PDI_U | 0.0007222807946 | 0.001081445551 | 0.6678845682 | 0.5042208004 |
| mrpl24 | 11355 | PDI_U | -0.000727389647 | 0.001089144488 | -0.6678541324 | 0.5042402962 |
| rtkn2 | 11355 | PDI_U | -0.0007271593943 | 0.001090114765 | -0.6670484776 | 0.5047547373 |
| rbm19 | 11355 | PDI_U | -0.0007266231592 | 0.001089696914 | -0.6668121657 | 0.5049056839 |
| unc5d | 11411 | PDI_U | 0.0007141350505 | 0.001072024448 | 0.6661555638 | 0.5053251531 |
| ophn1 | 11767 | PDI_U | 0.0007110305685 | 0.00106861026 | 0.6653787589 | 0.5058213221 |
| klk13 | 13340 | PDI_U | -0.0006523574483 | 0.0009806678391 | -0.6652175408 | 0.5059228736 |
| irag2 | 13388 | PDI_U | -0.0006703016932 | 0.001009181904 | -0.6642030445 | 0.5065718141 |
| btn3a2 | 13621 | PDI_U | 0.0006495392088 | 0.0009815244227 | 0.6617657123 | 0.5081325881 |
| srpk2 | 13621 | PDI_U | 0.000656253914 | 0.0009923049558 | 0.6613429775 | 0.5084035819 |
| ap3s2 | 11767 | PDI_U | -0.0007048546129 | 0.001065789929 | -0.6613447865 | 0.5084041879 |
| acaa1 | 13308 | PDI_U | 0.0006543681077 | 0.0009899043641 | 0.6610417444 | 0.5085969967 |
| scn3a | 11483 | PDI_U | -0.0007169749775 | 0.001084895896 | -0.66086984 | 0.5087090611 |
| gipr | 11411 | PDI_U | 0.0007177239004 | 0.001086103757 | 0.6608244339 | 0.5087382659 |
| ezr | 13784 | PDI_U | 0.000628144194 | 0.0009508874132 | 0.6605873474 | 0.5088880343 |
| mylpf | 11483 | PDI_U | -0.0006813009669 | 0.001032411111 | -0.6599124707 | 0.5093232502 |
| plxdc1 | 13308 | PDI_U | 0.0006629134229 | 0.001005837628 | 0.6590660407 | 0.509864775 |
| evi5 | 11411 | PDI_U | 0.0007139179946 | 0.001083476732 | 0.6589140066 | 0.5099643003 |
| daam1 | 11537 | PDI_U | 0.0007105366982 | 0.001078974733 | 0.658529506 | 0.5102110985 |
| dennd2b | 11355 | PDI_U | -0.0007146570892 | 0.001090290892 | -0.6554737772 | 0.5121760576 |
| stxbp1 | 11757 | PDI_U | -0.0007003179454 | 0.001068562625 | -0.6553831558 | 0.5122339299 |
| lta | 13621 | PDI_U | 0.0006343338023 | 0.0009680229129 | 0.6552880039 | 0.5122934191 |
| ndrg1 | 13320 | PDI_U | -0.0006621646877 | 0.001010614959 | -0.655209664 | 0.5123440982 |
| sez6 | 11483 | PDI_U | 0.0006877344175 | 0.001050739623 | 0.6545241106 | 0.5127873214 |
| cnpy4 | 13579 | PDI_U | 0.0006544420576 | 0.001000398908 | 0.6541810998 | 0.5130062253 |
| cdc123 | 11304 | PDI_U | -0.000714107679 | 0.00109440797 | -0.6525059197 | 0.5140881378 |
| metap1 | 13320 | PDI_U | 0.0006600026575 | 0.001012959769 | 0.6515586082 | 0.5146972083 |
| bnip2 | 11781 | PDI_U | -0.0006960430254 | 0.001068590653 | -0.6513654443 | 0.5148233297 |
| tmed8 | 11724 | PDI_U | 0.0006973649344 | 0.001071152633 | 0.6510416097 | 0.5150323997 |
| gata3 | 11304 | PDI_U | -0.0007125358919 | 0.001095035495 | -0.6506966167 | 0.515255587 |
| ren | 13618 | PDI_U | -0.0005978786188 | 0.0009190468073 | -0.650542077 | 0.5153531163 |
| fcrl6 | 13621 | PDI_U | 0.0006435981434 | 0.0009893693895 | 0.650513499 | 0.5153715667 |
| arf6 | 11724 | PDI_U | 0.0006948689781 | 0.001068268605 | 0.6504627905 | 0.5154060888 |
| anxa4 | 13564 | PDI_U | 0.0006527558729 | 0.001003874922 | 0.6502362583 | 0.515550646 |
| nt5e | 13699 | PDI_U | 0.0006361686165 | 0.0009783980732 | 0.6502145026 | 0.5155645877 |
| rabep1 | 11537 | PDI_U | 0.0007000420351 | 0.001077244529 | 0.6498450595 | 0.5158052615 |
| f11 | 11624 | PDI_U | -0.000676684796 | 0.001041946876 | -0.6494427032 | 0.5160651152 |
| cpvl | 13735 | PDI_U | -0.0006422250701 | 0.0009892342627 | -0.6492143412 | 0.5162107084 |
| pdgfrb | 13800 | PDI_U | -0.0006410849325 | 0.0009880001004 | -0.6488713233 | 0.5164323588 |
| col24a1 | 11724 | PDI_U | -0.0006855762387 | 0.001058020139 | -0.6479803301 | 0.5170103748 |
| clip2 | 13621 | PDI_U | 0.0006460242568 | 0.0009977144952 | 0.6475041306 | 0.5173166443 |
| il1rl1 | 13750 | PDI_U | -0.000596218164 | 0.0009213166294 | -0.6471370916 | 0.5175540339 |
| dapk2 | 11411 | PDI_U | 0.0006993398619 | 0.001082559237 | 0.6460060918 | 0.5182884147 |
| orm1 | 11768 | PDI_U | 0.000662647801 | 0.001026656679 | 0.6454424491 | 0.5186530989 |
| gba | 11483 | PDI_U | -0.0006977879587 | 0.001081336733 | -0.6453012622 | 0.5187448812 |
| gipc2 | 11767 | PDI_U | -0.0006562232415 | 0.001016963848 | -0.6452768627 | 0.5187603777 |
| acot13 | 11724 | PDI_U | -0.0006894628838 | 0.001069888017 | -0.6444252789 | 0.5193123172 |
| ifit3 | 11570 | PDI_U | 0.0006947098908 | 0.001078586291 | 0.6440930099 | 0.5195279039 |
| gnly | 13632 | PDI_U | -0.000630114644 | 0.0009798020274 | -0.6431040418 | 0.5201674186 |
| wasl | 11411 | PDI_U | 0.0006946116871 | 0.001081014492 | 0.6425553888 | 0.5205255611 |
| wdr46 | 11730 | PDI_U | 0.0006783782729 | 0.001056413564 | 0.6421521799 | 0.5207869408 |
| hmcn2 | 11516 | PDI_U | -0.0006846531676 | 0.001068142965 | -0.640975216 | 0.5215515537 |
| sh2b3 | 13308 | PDI_U | 0.0006460698479 | 0.001008627574 | 0.6405435113 | 0.5218303495 |
| nfe2 | 11474 | PDI_U | 0.0006912673862 | 0.001081288268 | 0.6392998116 | 0.5226406868 |
| gstm4 | 11355 | PDI_U | -0.0006944946772 | 0.001088598434 | -0.6379714093 | 0.5235051699 |
| pilrb | 13618 | PDI_U | -0.0006207079046 | 0.0009732450832 | -0.6377714261 | 0.523633215 |
| atp1b1 | 11355 | PDI_U | 0.0006936767518 | 0.001087657489 | 0.6377713195 | 0.5236354256 |
| gp6 | 13597 | PDI_U | 0.0006366521421 | 0.0009985488146 | 0.6375773851 | 0.5237595661 |
| vstm2l | 13388 | PDI_U | -0.0006369510322 | 0.001000072944 | -0.6369045739 | 0.524197903 |
| dnaja2 | 13751 | PDI_U | 0.0006288405121 | 0.0009889068769 | 0.6358945688 | 0.5248557349 |
| mnda | 13593 | PDI_U | 0.0006307018864 | 0.0009926080473 | 0.6353987237 | 0.5251791057 |
| amy2b | 12703 | PDI_U | 0.0006368899312 | 0.001002860734 | 0.6350731557 | 0.5253921532 |
| mri1 | 11483 | PDI_U | -0.0006846058374 | 0.001078008053 | -0.6350656062 | 0.5253982943 |

| Protein | N_used | term | beta | se | t | p_val |
|---|---|---|---|---|---|---|
| bpifb2 | 11754 | PDI_U | 0.0006109870172 | 0.0009630017093 | 0.6344609893 | 0.5257923786 |
| khdc3l | 11304 | PDI_U | -0.0006940175985 | 0.001094401076 | -0.6341528837 | 0.5259938988 |
| crtap | 11483 | PDI_U | 0.0006822461912 | 0.001078708168 | 0.6324659544 | 0.5270950545 |
| htr1b | 11383 | PDI_U | -0.0006881323942 | 0.001089233801 | -0.6317582078 | 0.5275575864 |
| pecam1 | 13632 | PDI_U | 0.0006259564692 | 0.0009908249773 | 0.6317528157 | 0.5275590132 |
| ehd3 | 11474 | PDI_U | 0.0006837682091 | 0.001083442507 | 0.6311070545 | 0.527983113 |
| tab2 | 11570 | PDI_U | 0.0006810730272 | 0.001079319144 | 0.6310209827 | 0.5280392826 |
| clstn1 | 13320 | PDI_U | -0.0006360128658 | 0.001008885203 | -0.6304115314 | 0.5284361928 |
| itga11 | 13621 | PDI_U | -0.0006118151706 | 0.0009710408134 | -0.6300612313 | 0.5286651018 |
| cd5l | 11740 | PDI_U | -0.0006697963884 | 0.001063279305 | -0.629934567 | 0.5287496677 |
| gchfr | 11260 | PDI_U | -0.0006452946482 | 0.00102547769 | -0.6292624934 | 0.5291900015 |
| naa80 | 11483 | PDI_U | 0.0006792836911 | 0.00107956497 | 0.629219834 | 0.529217676 |
| ralb | 11411 | PDI_U | -0.0006831482149 | 0.001086111398 | -0.6289854024 | 0.529371217 |
| dnajc21 | 11767 | PDI_U | -0.0006718634932 | 0.001068323286 | -0.6288952998 | 0.5294298236 |
| arhgef10 | 11757 | PDI_U | 0.0006706365538 | 0.001067333647 | 0.628328879 | 0.5298007347 |
| reep4 | 11757 | PDI_U | -0.0006706900422 | 0.001067977937 | -0.6279999043 | 0.5300162129 |
| btn1a1 | 11711 | PDI_U | -0.0006731917077 | 0.001072550111 | -0.6276552497 | 0.5302420571 |
| ccs | 13552 | PDI_U | -0.0006214431576 | 0.0009909557551 | -0.6271149387 | 0.5305944695 |
| tafa5 | 13579 | PDI_U | 0.0006060297633 | 0.000967966625 | 0.6260853914 | 0.5312694663 |
| masp1 | 13535 | PDI_U | 0.0006192257846 | 0.0009892583699 | 0.6259495026 | 0.531358628 |
| snap25 | 11383 | PDI_U | -0.0006733609602 | 0.001076638588 | -0.625428967 | 0.5317021068 |
| dtnb | 11704 | PDI_U | -0.0006246051935 | 0.0009987588237 | -0.6253814021 | 0.531732971 |
| lsm8 | 11570 | PDI_U | 0.0006711364965 | 0.001075024593 | 0.6242987377 | 0.5324437349 |
| lgals9 | 13642 | PDI_U | 0.0005485032126 | 0.0008789723504 | 0.6240278347 | 0.532619749 |
| col5a1 | 11537 | PDI_U | 0.0006663492676 | 0.00106837343 | 0.6237044548 | 0.5328340406 |
| gfer | 13308 | PDI_U | 0.00061786041 | 0.0009915001953 | 0.6231571238 | 0.5331919628 |
| inppl1 | 13579 | PDI_U | 0.0006213266016 | 0.0009988523881 | 0.6220404626 | 0.5339257152 |
| klk4 | 13753 | PDI_U | -0.0005776060915 | 0.0009286392343 | -0.6219919105 | 0.5339575071 |
| col28a1 | 11483 | PDI_U | 0.0006689908909 | 0.001075616623 | 0.6219603498 | 0.5339803015 |
| prr4 | 11448 | PDI_U | -0.000668924843 | 0.001075778335 | -0.6218054603 | 0.5340821908 |
| mnat1 | 11570 | PDI_U | 0.0006708163085 | 0.001078881959 | 0.6217698824 | 0.5341054566 |
| ppt1 | 11483 | PDI_U | -0.0006730030075 | 0.001083529583 | -0.621121027 | 0.534532337 |
| clgn | 11483 | PDI_U | -0.0006665179149 | 0.001073277435 | -0.6210117659 | 0.5346042208 |
| crebzf | 11304 | PDI_U | -0.0006774772292 | 0.001092731865 | -0.6199848753 | 0.535280255 |
| tsc22d1 | 11757 | PDI_U | -0.0006588616184 | 0.001064745575 | -0.6187972358 | 0.5360619444 |
| tpbgl | 11483 | PDI_U | 0.0006701919931 | 0.001083635646 | 0.6184661751 | 0.5362803679 |
| gigyf2 | 11757 | PDI_U | 0.0006572593329 | 0.001063762566 | 0.6178628147 | 0.5366777525 |
| kifbp | 13579 | PDI_U | 0.0006166789003 | 0.0009997132481 | 0.6168557849 | 0.537340204 |
| creld1 | 11537 | PDI_U | 0.0006201298539 | 0.001007903031 | 0.615267377 | 0.5383903065 |
| galnt10 | 13579 | PDI_U | 0.0005966431972 | 0.0009702487223 | 0.6149384003 | 0.5386057153 |
| ebag9 | 13552 | PDI_U | 0.0006148522276 | 0.001000361545 | 0.6146300111 | 0.5388094182 |
| nck2 | 13710 | PDI_U | 0.0006105072566 | 0.0009955301814 | 0.6132483655 | 0.5397223111 |
| brd3 | 11355 | PDI_U | -0.0006681155754 | 0.001090114747 | -0.6128855492 | 0.5399643064 |
| dcxr | 13699 | PDI_U | 0.0005516749241 | 0.0009001410622 | 0.6128760783 | 0.5399684646 |
| enpp6 | 11474 | PDI_U | -0.0006083794658 | 0.0009931548223 | -0.6125726344 | 0.54017111 |
| znrd2 | 11724 | PDI_U | -0.0006436784528 | 0.001050967384 | -0.612462825 | 0.5402434771 |
| nenf | 11730 | PDI_U | -0.0006537939974 | 0.001068752162 | -0.6117358358 | 0.5407244161 |
| dpp7 | 13516 | PDI_U | 0.0006093976951 | 0.0009972229371 | 0.6110947437 | 0.5411471453 |
| f3 | 13674 | PDI_U | -0.0005773258208 | 0.0009449127462 | -0.6109832079 | 0.5412208618 |
| crhr1 | 13388 | PDI_U | -0.0006166980878 | 0.001010317486 | -0.6104002916 | 0.5416070455 |
| cebpa | 11411 | PDI_U | 0.0006582223728 | 0.001079436604 | 0.6097832612 | 0.5420175458 |
| s100a3 | 11730 | PDI_U | -0.0006506192512 | 0.001067227222 | -0.6096351721 | 0.5421153276 |
| amotl2 | 11684 | PDI_U | 0.0006489347808 | 0.001067257819 | 0.6080393786 | 0.5431731875 |
| tnfsf13b | 13773 | PDI_U | -0.0005723092649 | 0.0009418289919 | -0.6076573028 | 0.5434248143 |
| ncam1 | 13689 | PDI_U | -0.0005666817353 | 0.0009329556429 | -0.6074047996 | 0.5435923881 |
| csrp3 | 11355 | PDI_U | -0.0006466820218 | 0.001064891056 | -0.6072752874 | 0.5436803916 |
| ebi3_il27 | 13732 | PDI_U | -0.000585211816 | 0.0009639408399 | -0.6071034567 | 0.5437923028 |
| hdgf | 13721 | PDI_U | 0.0006013468883 | 0.0009905873674 | 0.6070609298 | 0.5438205313 |
| ceacam19 | 11724 | PDI_U | -0.000632658326 | 0.001042773144 | -0.6067075371 | 0.5440567794 |
| nfkb2 | 11570 | PDI_U | -0.0006547925126 | 0.001079749974 | -0.6064297554 | 0.5442413249 |
| anxa11 | 13593 | PDI_U | 0.0006065094232 | 0.001000758982 | 0.6060494428 | 0.5444920504 |
| pilra | 13632 | PDI_U | -0.0005794399294 | 0.0009562110086 | -0.6059749618 | 0.5445414781 |
| cd200r1 | 13588 | PDI_U | -0.0006007904372 | 0.0009937353881 | -0.604577883 | 0.5454696095 |
| cxcl1 | 13607 | PDI_U | 0.0006002099156 | 0.000993599671 | 0.604076202 | 0.5458030605 |
| lats1 | 11411 | PDI_U | 0.0006524662471 | 0.001081044741 | 0.603551567 | 0.546153831 |
| rbpms | 11411 | PDI_U | -0.0006548033412 | 0.00108552326 | -0.6032144732 | 0.5463780222 |
| nek7 | 11570 | PDI_U | 0.0006489914167 | 0.001079745621 | 0.6010595495 | 0.5478121112 |
| mybpc2 | 11474 | PDI_U | 0.0006171452142 | 0.001027874935 | 0.6004088563 | 0.5482456576 |
| c4bpb | 13699 | PDI_U | -0.0005793228383 | 0.0009655287597 | -0.6000057818 | 0.5485123154 |
| cxcl14 | 13378 | PDI_U | -0.0005499688651 | 0.0009166478802 | -0.5999783308 | 0.5485308482 |

| Protein | N_used | term | beta | se | t | p_val |
|---|---|---|---|---|---|---|
| ly6d | 13592 | PDI_U | -0.000579955932 | 0.0009677131275 | -0.5993056367 | 0.5489790875 |
| pglyrp4 | 11355 | PDI_U | -0.0006441058322 | 0.001077920406 | -0.5975448918 | 0.5501555728 |
| tcof1 | 11781 | PDI_U | 0.0006172133926 | 0.001033466358 | 0.5972263999 | 0.550367725 |
| c8b | 11817 | PDI_U | -0.0006235746186 | 0.001044155873 | -0.5972045311 | 0.5503822883 |
| cacnb3 | 11757 | PDI_U | 0.0006340625547 | 0.001065737243 | 0.5949520474 | 0.5518869867 |
| spink5 | 13632 | PDI_U | 0.000582543361 | 0.0009797682939 | 0.5945725787 | 0.5521390906 |
| vcpkmt | 11304 | PDI_U | 0.0006495390082 | 0.001092707137 | 0.5944310112 | 0.5522357813 |
| mtus1 | 11570 | PDI_U | -0.000622010302 | 0.00104643499 | -0.5944089293 | 0.5522502723 |
| cstb | 13461 | PDI_U | -0.000564839501 | 0.0009513489062 | -0.5937248651 | 0.5527061348 |
| trem2 | 13658 | PDI_U | -0.0005421330816 | 0.0009136556361 | -0.5933669757 | 0.5529454193 |
| azu1 | 13254 | PDI_U | 0.0005903920235 | 0.000996752699 | 0.5923154501 | 0.5536494852 |
| fkbp4 | 13552 | PDI_U | 0.0005899059636 | 0.0009960401804 | 0.5922511714 | 0.5536922974 |
| mtif3 | 11724 | PDI_U | -0.0006328755415 | 0.001068865121 | -0.592100471 | 0.5537947412 |
| psma1 | 13308 | PDI_U | -0.000599746975 | 0.001013077759 | -0.5920048777 | 0.5538573882 |
| tnr | 13552 | PDI_U | -0.0005658111698 | 0.000956960124 | -0.5912588786 | 0.5543568477 |
| tp53 | 13308 | PDI_U | 0.0005964352503 | 0.00100880878 | 0.5912272596 | 0.5543782109 |
| brd1 | 11570 | PDI_U | -0.0006362793131 | 0.001078259075 | -0.5900987322 | 0.5551359949 |
| nphs2 | 11411 | PDI_U | -0.000641426597 | 0.001086987184 | -0.5900958232 | 0.5551381059 |
| aoc3 | 13689 | PDI_U | -0.0005623963157 | 0.0009533572743 | -0.5899113909 | 0.5552598022 |
| cilp | 11790 | PDI_U | -0.0005880256616 | 0.000997150312 | -0.5897061401 | 0.5553989899 |
| atp5if1 | 13710 | PDI_U | 0.000586264848 | 0.0009941998027 | 0.5896851381 | 0.5554114868 |
| galnt5 | 11781 | PDI_U | -0.0006177629629 | 0.001048396273 | -0.5892456685 | 0.555707796 |
| clns1a | 11483 | PDI_U | -0.000638813174 | 0.001084160445 | -0.5892238337 | 0.5557227345 |
| otud7b | 11757 | PDI_U | -0.0006264749217 | 0.001064436677 | -0.588550672 | 0.5561740502 |
| dxo | 11767 | PDI_U | -0.0006272420408 | 0.001065840939 | -0.5884949787 | 0.5562114101 |
| il36g | 11351 | PDI_U | -0.0006291929158 | 0.001071030185 | -0.5874651571 | 0.5569030424 |
| s100a12 | 13461 | PDI_U | 0.0005797583161 | 0.0009898552177 | 0.5857001163 | 0.5580868838 |
| pth1r | 13621 | PDI_U | 0.0005797904444 | 0.0009911544622 | 0.5849647724 | 0.5585811021 |
| ppp3r1 | 13552 | PDI_U | 0.0005742680991 | 0.0009846713906 | 0.5832078647 | 0.5597630908 |
| fabp3 | 11781 | PDI_U | -0.000532684037 | 0.0009170819595 | -0.5808467079 | 0.5613548944 |
| ssb | 13320 | PDI_U | 0.0005870292624 | 0.001012998317 | 0.5794967794 | 0.5622638284 |
| hspb6 | 13753 | PDI_U | 0.0004764138035 | 0.0008229287127 | 0.5789247552 | 0.562649435 |
| tmprss5 | 13483 | PDI_U | -0.000558702039 | 0.0009667632305 | -0.577909897 | 0.5633346127 |
| eddm3b | 11730 | PDI_U | 0.0003998330307 | 0.0006919100296 | 0.5778685286 | 0.5633639882 |
| timm8a | 11767 | PDI_U | 0.0006155500958 | 0.001067722773 | 0.5765074151 | 0.5642832977 |
| apoa2 | 11794 | PDI_U | -0.0005874844169 | 0.001020094323 | -0.5759118579 | 0.5646857601 |
| il17f | 13378 | PDI_U | 0.000581234659 | 0.001009716034 | 0.5756417049 | 0.5648670767 |
| fgfbp1 | 13699 | PDI_U | 0.0005541492121 | 0.0009637171267 | 0.575012311 | 0.5652924226 |
| pvalb | 13698 | PDI_U | 0.000571722445 | 0.0009961790801 | 0.5739153295 | 0.5660345304 |
| cwc15 | 11662 | PDI_U | 0.0006025219296 | 0.00105152977 | 0.5729955981 | 0.5666587332 |
| fgf23 | 13728 | PDI_U | 0.0005414586985 | 0.0009469889259 | 0.5717687755 | 0.5674880034 |
| tnfrsf11a | 13801 | PDI_U | 0.000525952782 | 0.0009200376226 | 0.5716644288 | 0.5675586555 |
| hyal1 | 13566 | PDI_U | 0.0005635325368 | 0.000985819523 | 0.5716386455 | 0.5675762875 |
| pon1 | 11794 | PDI_U | -0.0005991192715 | 0.001048548402 | -0.5713797003 | 0.5677531854 |
| st8sia1 | 11411 | PDI_U | 0.0006181951995 | 0.00108474783 | 0.5698976132 | 0.5687583749 |
| fabp9 | 13621 | PDI_U | -0.0005570800475 | 0.0009791579996 | -0.5689378503 | 0.5694077065 |
| optc | 13579 | PDI_U | -0.0005275505887 | 0.0009272718246 | -0.5689276593 | 0.5694146516 |
| hsbp1 | 11474 | PDI_U | -0.000615890911 | 0.001083014118 | -0.5686822553 | 0.5695829337 |
| ada | 13801 | PDI_U | -0.0005561727182 | 0.0009780600876 | -0.5686488235 | 0.5696037452 |
| ptprc | 11790 | PDI_U | -0.0005933210299 | 0.001043680239 | -0.5684892823 | 0.5697136196 |
| dbh | 11763 | PDI_U | -0.0006020313395 | 0.001059562775 | -0.5681884584 | 0.5699178642 |
| acrv1 | 11411 | PDI_U | -0.0003884467928 | 0.0006837710266 | -0.5680948413 | 0.5699817602 |
| hspb1 | 13394 | PDI_U | 0.0005702126563 | 0.001007640046 | 0.5658892365 | 0.5714785675 |
| vash1 | 13621 | PDI_U | 0.0005650964718 | 0.0009986612379 | 0.5658540157 | 0.571502353 |
| ca3 | 13785 | PDI_U | 0.0005421045337 | 0.0009585868663 | 0.5655246831 | 0.5717261525 |
| arhgap45 | 11537 | PDI_U | 0.000609566269 | 0.001079523854 | 0.5646621581 | 0.5723145676 |
| kiaa0319 | 11767 | PDI_U | 0.0005821455827 | 0.001031740936 | 0.5642361974 | 0.5726041614 |
| hk2 | 13388 | PDI_U | -0.0005677482183 | 0.001011070871 | -0.5615315745 | 0.5744446196 |
| mphosph8 | 13388 | PDI_U | -0.0005640600292 | 0.001007375448 | -0.5599302927 | 0.5755363619 |
| septin7 | 11570 | PDI_U | -0.0006041389351 | 0.001079722267 | -0.5595317919 | 0.5758096827 |
| pdia3 | 11537 | PDI_U | -0.000600054483 | 0.001073131834 | -0.5591619445 | 0.5760620674 |
| gbp4 | 13320 | PDI_U | -0.0005655714718 | 0.001011950252 | -0.5588925651 | 0.5762444479 |
| hars1 | 13632 | PDI_U | 0.0005564647041 | 0.0009958419374 | 0.5587881803 | 0.5763154766 |
| tbcb | 13552 | PDI_U | 0.0005596817076 | 0.001002679352 | 0.5581861306 | 0.57672652 |
| csnk2a1 | 11757 | PDI_U | -0.0005932928344 | 0.001065201585 | -0.5569770481 | 0.5775537238 |
| gp1bb | 11355 | PDI_U | -0.0006054071007 | 0.001087191001 | -0.5568544074 | 0.5776378936 |
| pard3 | 11570 | PDI_U | 0.0006003175165 | 0.001079621633 | 0.5560443568 | 0.5781912959 |
| snx15 | 11537 | PDI_U | 0.0005944828717 | 0.001070381525 | 0.5553934348 | 0.578636362 |
| lactb2 | 13618 | PDI_U | 0.0005535819808 | 0.0009992869605 | 0.5539769882 | 0.5796036986 |
| entpd6 | 13388 | PDI_U | 0.0005557482934 | 0.00100369386 | 0.5537029923 | 0.5797913824 |

| Protein | N_used | term | beta | se | t | p_val |
|---|---|---|---|---|---|---|
| aoc1 | 13378 | PDI_U | -0.0005582099452 | 0.001009617464 | -0.5528925211 | 0.5803462554 |
| nfic | 11757 | PDI_U | 0.0005906021031 | 0.001068275768 | 0.552855471 | 0.5803729026 |
| evi2b | 11304 | PDI_U | -0.0006047722841 | 0.00109412281 | -0.5527462537 | 0.5804481175 |
| tax1bp1 | 11767 | PDI_U | 0.0005865248027 | 0.001061165516 | 0.5527175484 | 0.5804673448 |
| slc13a1 | 11730 | PDI_U | 0.0005874032181 | 0.001071754814 | 0.5480761183 | 0.5836500781 |
| ifnar1 | 11724 | PDI_U | 0.0005832397854 | 0.001065827746 | 0.5472176793 | 0.5842396181 |
| comt | 13243 | PDI_U | 0.0005511231193 | 0.00100800304 | 0.5467474774 | 0.5845614517 |
| smoc2 | 13710 | PDI_U | 0.0004942034601 | 0.0009039565268 | 0.5467115347 | 0.5845858336 |
| crisp2 | 13753 | PDI_U | -0.0004594408036 | 0.0008403991111 | -0.5466935859 | 0.5845981386 |
| bcat2 | 11383 | PDI_U | 0.000594271803 | 0.001087957352 | 0.5462271126 | 0.5849205479 |
| ca8 | 11411 | PDI_U | 0.0005929083229 | 0.001085723677 | 0.5460950472 | 0.5850112914 |
| rabgap1l | 13378 | PDI_U | 0.0005465006371 | 0.001002938612 | 0.5448993894 | 0.5858318076 |
| micall2 | 11483 | PDI_U | 0.0005904901311 | 0.001083818451 | 0.5448238406 | 0.5858852717 |
| ogfr | 13579 | PDI_U | 0.0005291834718 | 0.0009715298677 | 0.5446908936 | 0.5859750816 |
| mdm1 | 11483 | PDI_U | -0.0005908528173 | 0.00108483215 | -0.544649066 | 0.5860054894 |
| pbk | 11304 | PDI_U | -0.0005960241449 | 0.001094569823 | -0.5445282085 | 0.586088795 |
| nfx1 | 11355 | PDI_U | 0.0005909897748 | 0.001085540202 | 0.54441998 | 0.5861632016 |
| creb3 | 11304 | PDI_U | -0.0005959801462 | 0.001094815792 | -0.5443656826 | 0.586200605 |
| dcn | 13388 | PDI_U | -0.0005142241865 | 0.000944706371 | -0.5443217091 | 0.5862291838 |
| hmbs | 13753 | PDI_U | 0.0005304428759 | 0.0009755200682 | 0.5437539352 | 0.5866196335 |
| nubp1 | 11730 | PDI_U | 0.0005801158689 | 0.001071030602 | 0.5416426646 | 0.5880749926 |
| foxo3 | 13308 | PDI_U | 0.0005468097204 | 0.001010680824 | 0.5410310632 | 0.5884952361 |
| lrrfip1 | 11730 | PDI_U | 0.0005765378457 | 0.00106657698 | 0.5405496802 | 0.5888282833 |
| nfkb1 | 11355 | PDI_U | 0.0005854848537 | 0.001083636611 | 0.5402963021 | 0.589003316 |
| sec31a | 11474 | PDI_U | 0.0005841137104 | 0.001081404224 | 0.5401437292 | 0.5891084097 |
| ddx53 | 11483 | PDI_U | 0.000585296533 | 0.001084229888 | 0.5398269678 | 0.5893268461 |
| pfdn2 | 13320 | PDI_U | -0.0005465012263 | 0.001013301921 | -0.5393271392 | 0.5896701684 |
| tfpi | 13590 | PDI_U | -0.0005215170897 | 0.00096701723 | -0.5393048578 | 0.5896853603 |
| akt2 | 11757 | PDI_U | 0.0005765762457 | 0.001069311627 | 0.5392031947 | 0.5897568769 |
| siglec5 | 13632 | PDI_U | -0.000528841708 | 0.0009838692231 | -0.5375121973 | 0.5909226435 |
| ptprf | 13800 | PDI_U | -0.0005107249268 | 0.0009503204483 | -0.5374239054 | 0.5909835072 |
| rilpl2 | 11781 | PDI_U | 0.0005718196778 | 0.001065910624 | 0.5364611863 | 0.5916499937 |
| rasgrf1 | 11304 | PDI_U | -0.0005864796787 | 0.001095140548 | -0.5355291426 | 0.592294558 |
| mapkapk2 | 11570 | PDI_U | 0.0005772439902 | 0.001079171285 | 0.534895617 | 0.5927323292 |
| phactr2 | 11757 | PDI_U | 0.0005708118321 | 0.001067727985 | 0.5346041691 | 0.5929337198 |
| pten | 13320 | PDI_U | 0.0005415301882 | 0.0010131276 | 0.5345133113 | 0.5929953718 |
| stk11 | 13388 | PDI_U | -0.0005344914018 | 0.001002671511 | -0.5330673068 | 0.5939958455 |
| vsig4 | 13613 | PDI_U | 0.0004787139073 | 0.0008982655949 | 0.5329313624 | 0.5940898013 |
| adamts16 | 13388 | PDI_U | -0.000494606329 | 0.0009281878011 | -0.5328731194 | 0.5941302664 |
| tubb3 | 11383 | PDI_U | -0.0005786122859 | 0.001090504801 | -0.5305912322 | 0.5957124345 |
| tarbp2 | 13320 | PDI_U | 0.0005340516033 | 0.001007907432 | 0.5298617577 | 0.596216622 |
| il10 | 13378 | PDI_U | -0.0005342583456 | 0.001008768897 | -0.5296142131 | 0.5963882342 |
| alpp | 13579 | PDI_U | -0.0004943830334 | 0.0009339886431 | -0.5293244592 | 0.5965890515 |
| dbn1 | 11411 | PDI_U | 0.0005736939589 | 0.001084615979 | 0.5289374027 | 0.5968591734 |
| rab10 | 11781 | PDI_U | -0.0005639392924 | 0.001066973392 | -0.528541102 | 0.5971337938 |
| cc2d1a | 13552 | PDI_U | 0.0005232576637 | 0.0009921894263 | 0.5273767789 | 0.5979406066 |
| tnfrsf21 | 13784 | PDI_U | -0.0005091371386 | 0.0009675183843 | -0.5262299372 | 0.5987369299 |
| tgfa | 13621 | PDI_U | 0.0005056261387 | 0.0009624892891 | 0.5253317044 | 0.5993611797 |
| mag | 11483 | PDI_U | 0.000568934426 | 0.001083729139 | 0.5249784336 | 0.599608329 |
| tef | 11355 | PDI_U | -0.0005715695591 | 0.001088899933 | -0.5249054958 | 0.5996591473 |
| kir3dl1 | 13308 | PDI_U | 0.0005312849499 | 0.001012806547 | 0.5245670571 | 0.5998929378 |
| tdrkh | 13320 | PDI_U | 0.0005286679163 | 0.001008001177 | 0.5244715267 | 0.5999593543 |
| yod1 | 11355 | PDI_U | 0.0005585244831 | 0.001066484755 | 0.5237060168 | 0.6004932616 |
| gfra1 | 13732 | PDI_U | -0.0004794261362 | 0.0009157337839 | -0.5235431352 | 0.6006047975 |
| vav3 | 11383 | PDI_U | 0.0005683414334 | 0.001088004832 | 0.5223703211 | 0.6014226922 |
| il15 | 13621 | PDI_U | -0.0005185539032 | 0.0009930700284 | -0.5221725441 | 0.6015586996 |
| lamp1 | 11454 | PDI_U | 0.0005547970347 | 0.001064916735 | 0.5209769146 | 0.6023929351 |
| col9a2 | 11570 | PDI_U | 0.0005605330037 | 0.00107804395 | 0.5199537584 | 0.6031057633 |
| fcer1a | 11757 | PDI_U | 0.0005556161907 | 0.001069917492 | 0.5196681857 | 0.6033046579 |
| rest | 11304 | PDI_U | -0.0005674210306 | 0.001094510728 | -0.5184243664 | 0.6041723757 |
| slc27a4 | 13320 | PDI_U | 0.0005221211983 | 0.001007144306 | 0.5184174654 | 0.6041756506 |
| cat | 11605 | PDI_U | -0.0005422112013 | 0.001046671556 | -0.5180337595 | 0.6044446009 |
| ceacam3 | 13308 | PDI_U | 0.0005239692797 | 0.001013031536 | 0.5172289915 | 0.6050049202 |
| krt5 | 13320 | PDI_U | 0.0004945059256 | 0.0009566047304 | 0.5169386163 | 0.6052076003 |
| sel1l | 11474 | PDI_U | 0.0005418849251 | 0.001048943012 | 0.5166009203 | 0.6054447399 |
| cdh4 | 11383 | PDI_U | 0.0005619548528 | 0.001090138598 | 0.515489364 | 0.6062211216 |
| c1qa | 13747 | PDI_U | -0.0005065343407 | 0.0009831006451 | -0.5152415912 | 0.6063925009 |
| stx4 | 13579 | PDI_U | -0.0005085706025 | 0.0009887890112 | -0.5143368269 | 0.6070248961 |
| rbp1 | 11483 | PDI_U | 0.0005564578402 | 0.001083188118 | 0.5137222527 | 0.6074560866 |
| kynu | 13801 | PDI_U | -0.0004806667305 | 0.0009368783041 | -0.5130514053 | 0.6079235818 |

| Protein | N_used | term | beta | se | t | p_val |
|---|---|---|---|---|---|---|
| lmod2 | 11383 | PDI_U | 0.0005588031661 | 0.001089172671 | 0.5130528711 | 0.6079243042 |
| il5 | 13378 | PDI_U | -0.0005160271359 | 0.001006142896 | -0.5128765886 | 0.6080461268 |
| mki67 | 11411 | PDI_U | 0.0005558405193 | 0.001084536806 | 0.5125142056 | 0.6083011112 |
| atp5po | 13320 | PDI_U | -0.0005185125995 | 0.001013652364 | -0.511529019 | 0.6089891609 |
| vcam1 | 13669 | PDI_U | -0.0005013492048 | 0.0009808708917 | -0.5111266009 | 0.609270673 |
| ppp1r2 | 13618 | PDI_U | 0.0005101000226 | 0.0009993540445 | 0.5104297375 | 0.6097587083 |
| fuom | 11537 | PDI_U | 0.0005070505736 | 0.0009938461643 | 0.5101902003 | 0.6099279875 |
| ppl | 11411 | PDI_U | 0.0005421517384 | 0.001065360663 | 0.5088903291 | 0.6108389485 |
| wfdc2 | 12993 | PDI_U | 0.0004574854234 | 0.0009005782163 | 0.507990772 | 0.6114684463 |
| il24 | 13378 | PDI_U | -0.000513461779 | 0.001011090189 | -0.50782985 | 0.6115810539 |
| fam3b | 13579 | PDI_U | 0.0004993343871 | 0.0009847854366 | 0.507048915 | 0.6121287387 |
| il13ra1 | 13501 | PDI_U | 0.0004916834403 | 0.0009698869409 | 0.5069492325 | 0.612198727 |
| acyp1 | 11487 | PDI_U | -0.0005445229978 | 0.001075161916 | -0.5064567387 | 0.6125457825 |
| stx3 | 11767 | PDI_U | -0.0005405191649 | 0.001068800173 | -0.5057251847 | 0.6130590686 |
| bcat1 | 11781 | PDI_U | 0.0005292732614 | 0.001047060736 | 0.5054847758 | 0.613227855 |
| il18 | 13632 | PDI_U | 0.0004837455719 | 0.0009583697411 | 0.504758812 | 0.6137364143 |
| eif2ak2 | 11684 | PDI_U | 0.0005401350196 | 0.001070771792 | 0.5044352343 | 0.6139650856 |
| ehbp1 | 11781 | PDI_U | 0.0005358731875 | 0.001064750124 | 0.5032853958 | 0.6147730516 |
| prkar1a | 13618 | PDI_U | 0.0005025332718 | 0.001000037163 | 0.5025145967 | 0.6153137202 |
| calcoco1 | 13703 | PDI_U | 0.0005000585853 | 0.0009954708936 | 0.5023337081 | 0.6154408809 |
| ckap4 | 13729 | PDI_U | 0.0004797396557 | 0.0009550335633 | 0.502327535 | 0.615445207 |
| lztfl1 | 11537 | PDI_U | 0.0005417655047 | 0.001078942887 | 0.5021262121 | 0.6155883358 |
| ubqln3 | 11355 | PDI_U | -0.0005462299969 | 0.001089621785 | -0.5013023825 | 0.6161680571 |
| lap3 | 13378 | PDI_U | 0.0005018056987 | 0.001004130348 | 0.4997415919 | 0.6172652686 |
| psmg3 | 13621 | PDI_U | 0.0004870795756 | 0.0009757886928 | 0.4991650131 | 0.6176712088 |
| hmgcl | 11474 | PDI_U | -0.0005391394516 | 0.001081689923 | -0.4984232914 | 0.6181952874 |
| sema6c | 11411 | PDI_U | -0.0005395586609 | 0.001082561807 | -0.4984091045 | 0.6182053372 |
| afp | 13510 | PDI_U | 0.0004848621496 | 0.0009741196414 | 0.4977439413 | 0.618672641 |
| nrgn | 11794 | PDI_U | 0.0005313266302 | 0.001068830537 | 0.497110264 | 0.6191205729 |
| il15ra | 13378 | PDI_U | -0.0004912104224 | 0.0009883394714 | -0.4970057724 | 0.6191931553 |
| tarm1 | 11483 | PDI_U | -0.0005382784695 | 0.001084222615 | -0.4964648977 | 0.6195759605 |
| jam3 | 11537 | PDI_U | 0.0005350499527 | 0.001078934828 | 0.4959057199 | 0.6199703867 |
| pnma1 | 11474 | PDI_U | 0.00053637586 | 0.001083045924 | 0.4952475681 | 0.6204348706 |
| kyat1 | 13591 | PDI_U | -0.0004935503978 | 0.000997388657 | -0.4948426016 | 0.6207192361 |
| cd200 | 13621 | PDI_U | 0.0004747759762 | 0.0009603338224 | 0.4943863948 | 0.6210413007 |
| krt17 | 11355 | PDI_U | 0.000538673781 | 0.001090105814 | 0.4941481591 | 0.6212111173 |
| tmed1 | 11570 | PDI_U | -0.0005330623324 | 0.001079768584 | -0.4936820169 | 0.6215401482 |
| angptl2 | 13681 | PDI_U | 0.0004603364746 | 0.0009324917362 | 0.4936627926 | 0.6215522782 |
| slmap | 11724 | PDI_U | -0.0005245641262 | 0.001064664795 | -0.4927035522 | 0.6222313038 |
| hebp1 | 13388 | PDI_U | 0.0004969844182 | 0.001010055332 | 0.4920368246 | 0.6227013862 |
| ccl28 | 13587 | PDI_U | -0.0004826139277 | 0.0009810517007 | -0.4919352643 | 0.6227730625 |
| sez6l | 13699 | PDI_U | 0.000454332888 | 0.0009235745287 | 0.4919287766 | 0.6227775837 |
| upk3bl1 | 11544 | PDI_U | -0.0005310476057 | 0.001081408169 | -0.491070459 | 0.6233859592 |
| nub1 | 13621 | PDI_U | 0.0004902599643 | 0.0009983536353 | 0.491068442 | 0.6233859595 |
| inpp5j | 11757 | PDI_U | 0.0005160931958 | 0.001052052502 | 0.4905584034 | 0.6237479772 |
| trim5 | 13621 | PDI_U | 0.0004895857137 | 0.0009982736811 | 0.4904323564 | 0.623835892 |
| cetn2 | 13320 | PDI_U | -0.0004954487942 | 0.001010267191 | -0.4904136239 | 0.6238493234 |
| ly75 | 13621 | PDI_U | 0.0004869250988 | 0.0009931333755 | 0.4902917481 | 0.6239353697 |
| lrp2bp | 11483 | PDI_U | 0.0005307902202 | 0.001083810079 | 0.4897446801 | 0.6243239463 |
| clec2l | 11483 | PDI_U | 0.0005298798591 | 0.001083602444 | 0.4889983981 | 0.6248521801 |
| apbb1ip | 13579 | PDI_U | 0.0004680101916 | 0.0009571947803 | 0.4889393478 | 0.6248925411 |
| dcun1d1 | 11383 | PDI_U | -0.000531849669 | 0.001087890373 | -0.4888816761 | 0.6249348979 |
| nudt5 | 13632 | PDI_U | 0.0004816610802 | 0.0009856673887 | 0.4886649247 | 0.6250868078 |
| tlr4 | 11411 | PDI_U | 0.0005303543412 | 0.001086100978 | 0.488310343 | 0.6253394294 |
| yap1 | 11767 | PDI_U | -0.000444738763 | 0.0009107972094 | -0.4882961415 | 0.6253492023 |
| ep300 | 11411 | PDI_U | -0.0005293765128 | 0.001086155563 | -0.4873855375 | 0.6259945132 |
| ppie | 11570 | PDI_U | 0.0005258226601 | 0.001079157767 | 0.4872528151 | 0.6260884222 |
| ang | 13590 | PDI_U | -0.0004569285766 | 0.0009380929945 | -0.4870823887 | 0.6262078092 |
| spink6 | 13753 | PDI_U | 0.000457224502 | 0.0009398857971 | 0.4864681469 | 0.6266430425 |
| bambi | 13308 | PDI_U | 0.0004833463097 | 0.0009963618556 | 0.4851112144 | 0.6276054499 |
| cyth3 | 11304 | PDI_U | -0.0005302391932 | 0.001093267167 | -0.4850042234 | 0.6276827581 |
| rab11fip3 | 11781 | PDI_U | 0.0005163326098 | 0.001065303358 | 0.4846812939 | 0.6279114579 |
| nectin2 | 13599 | PDI_U | 0.0004717123016 | 0.0009734092194 | 0.4845981446 | 0.6279692419 |
| azi2 | 11757 | PDI_U | 0.0005168612469 | 0.001066877579 | 0.4844616263 | 0.6280673256 |
| rbm17 | 11757 | PDI_U | 0.0005174332724 | 0.001068840204 | 0.4841072317 | 0.6283187957 |
| dynlt1 | 11483 | PDI_U | 0.0005190630078 | 0.001072970004 | 0.4837628321 | 0.6285634302 |
| il17d | 13378 | PDI_U | 0.0004474781673 | 0.0009250474388 | 0.4837353724 | 0.6285816109 |
| klrb1 | 13801 | PDI_U | 0.0004667235824 | 0.0009650347337 | 0.4836339731 | 0.6286533396 |
| il34 | 13301 | PDI_U | -0.0004888867321 | 0.00101289125 | -0.4826645824 | 0.6293418654 |
| gli2 | 11411 | PDI_U | -0.000522616189 | 0.001084559981 | -0.4818693276 | 0.6299080276 |

| Protein | N_used | term | beta | se | t | p_val |
|---|---|---|---|---|---|---|
| cfhr2 | 11728 | PDI_U | 0.0005111492927 | 0.001062089208 | 0.4812677586 | 0.6303351967 |
| chek2 | 13388 | PDI_U | -0.0004837123121 | 0.001010915711 | -0.4784892618 | 0.6323098359 |
| otud6b | 11724 | PDI_U | -0.0005122554891 | 0.001070870414 | -0.4783543202 | 0.632406969 |
| lrig3 | 11379 | PDI_U | 0.0005075348406 | 0.001061618084 | 0.4780766723 | 0.6326048285 |
| nrp1 | 13712 | PDI_U | -0.0004686934328 | 0.0009829650579 | -0.4768159652 | 0.6335007812 |
| syngap1 | 11383 | PDI_U | -0.0005195805598 | 0.00108987217 | -0.4767353217 | 0.6335597684 |
| csf2 | 11355 | PDI_U | -0.0005092291546 | 0.001069993742 | -0.4759178812 | 0.6341420482 |
| egflam | 11724 | PDI_U | -0.0005046259287 | 0.001060758807 | -0.4757216489 | 0.6342815683 |
| mydgf | 11781 | PDI_U | 0.0005032825388 | 0.001061674022 | 0.4740462028 | 0.6354757544 |
| fkbpl | 11724 | PDI_U | 0.0005056449936 | 0.001067482473 | 0.4736799025 | 0.6357370162 |
| gadd45gip1 | 11474 | PDI_U | -0.0005117134571 | 0.001080629472 | -0.4735327607 | 0.6358421524 |
| gart | 11757 | PDI_U | -0.0005028121599 | 0.0010634975 | -0.4727911067 | 0.6363710051 |
| adamtsl2 | 11790 | PDI_U | 0.0004679481373 | 0.0009911256556 | 0.472138053 | 0.6368369997 |
| tnfsf12 | 13801 | PDI_U | 0.0004630757107 | 0.0009813324787 | 0.471884627 | 0.6370166084 |
| rab33a | 11355 | PDI_U | -0.0005128008536 | 0.001089040553 | -0.4708739744 | 0.6377397891 |
| sost | 13599 | PDI_U | 0.0004330086916 | 0.0009217656314 | 0.4697600744 | 0.6385339812 |
| cmip | 11730 | PDI_U | 0.0005011376175 | 0.001070414283 | 0.4681716466 | 0.6396705542 |
| paxx | 11537 | PDI_U | 0.000494688599 | 0.001059591882 | 0.4668671094 | 0.6406037836 |
| pcbd1 | 11512 | PDI_U | 0.0004618254799 | 0.0009893471542 | 0.4667982092 | 0.6406531002 |
| smc3 | 11304 | PDI_U | -0.0005101247811 | 0.00109509555 | -0.4658267319 | 0.6413485136 |
| ahnak | 11474 | PDI_U | -0.0004640746478 | 0.0009965405839 | -0.4656856482 | 0.641449375 |
| fetub | 13669 | PDI_U | -0.0004387232318 | 0.0009424551949 | -0.4655109698 | 0.641573006 |
| lelp1 | 11383 | PDI_U | 0.0005058076068 | 0.001090102692 | 0.4639999611 | 0.6426566558 |
| sema7a | 13750 | PDI_U | 0.000450875837 | 0.0009732355905 | 0.4632751221 | 0.6431745144 |
| ssna1 | 11724 | PDI_U | 0.000490621882 | 0.001063437824 | 0.461354553 | 0.6445528195 |
| gipc3 | 11724 | PDI_U | -0.000493098454 | 0.001071638393 | -0.4601351139 | 0.6454277833 |
| btd | 11817 | PDI_U | 0.0004814772925 | 0.001050722109 | 0.4582346641 | 0.6467922932 |
| txnl1 | 11363 | PDI_U | -0.00049781055 | 0.001088128582 | -0.4574923939 | 0.6473259241 |
| bola2_bola2b | 11474 | PDI_U | 0.0004884440577 | 0.001070431948 | 0.4563055678 | 0.6481789064 |
| ccl19 | 13784 | PDI_U | 0.0004410825349 | 0.0009669433133 | 0.4561617303 | 0.6482808758 |
| atxn2l | 11757 | PDI_U | 0.0004860847054 | 0.001066183281 | 0.4559110182 | 0.6484623951 |
| vps37a | 13579 | PDI_U | 0.0004532561184 | 0.0009955442427 | 0.4552847568 | 0.6489116768 |
| ak2 | 11767 | PDI_U | 0.0004861327203 | 0.00106817616 | 0.455105383 | 0.6490418306 |
| bpifb1 | 13604 | PDI_U | -0.0004305127216 | 0.0009466795499 | -0.4547607705 | 0.6492886185 |
| hcg22 | 11355 | PDI_U | -0.0004948694561 | 0.001088501374 | -0.454633745 | 0.6493814536 |
| il20rb | 11411 | PDI_U | 0.0004936733984 | 0.001086233237 | 0.4544819487 | 0.6494906355 |
| dhps | 11730 | PDI_U | 0.0004802947745 | 0.001056825575 | 0.4544692956 | 0.6494995048 |
| c1galt1c1 | 11355 | PDI_U | -0.0004946386259 | 0.001088848266 | -0.4542769101 | 0.6496382252 |
| ppp1r9b | 13801 | PDI_U | 0.0004495341029 | 0.0009896046666 | 0.4542562481 | 0.6496515526 |
| dnaja4 | 11767 | PDI_U | -0.0004799891497 | 0.001062182638 | -0.4518894703 | 0.6513569481 |
| spock1 | 13552 | PDI_U | 0.0004437337014 | 0.000982353943 | 0.4517045049 | 0.6514891076 |
| clc | 13593 | PDI_U | 0.0004437995984 | 0.0009834855706 | 0.4512517638 | 0.6518153108 |
| vsig10 | 11730 | PDI_U | 0.0004801963766 | 0.001067240178 | 0.4499421839 | 0.6527604527 |
| dipk2b | 11537 | PDI_U | 0.0004557482846 | 0.001013679975 | 0.4495977979 | 0.6530089313 |
| ocln | 11757 | PDI_U | -0.0004485058028 | 0.001000648219 | -0.4482152609 | 0.6540061177 |
| decr1 | 13621 | PDI_U | -0.0004468174094 | 0.0009970891283 | -0.4481218345 | 0.6540724045 |
| golga3 | 11757 | PDI_U | 0.0004771732735 | 0.001065404737 | 0.4478798123 | 0.6542481994 |
| adgrb3 | 13526 | PDI_U | 0.0004445668002 | 0.0009926702838 | 0.4478494092 | 0.6542690608 |
| commd9 | 11730 | PDI_U | 0.0004794344961 | 0.001070967342 | 0.4476649076 | 0.6544033269 |
| impa1 | 13552 | PDI_U | -0.0004487365683 | 0.001003219936 | -0.4472963027 | 0.6546682912 |
| ptp4a3 | 11411 | PDI_U | 0.0004674219214 | 0.001045507493 | 0.447076587 | 0.6548282574 |
| pamr1 | 13632 | PDI_U | -0.0004107272241 | 0.0009197861521 | -0.4465464316 | 0.6552096802 |
| nmi | 11681 | PDI_U | -0.0004781195984 | 0.001070913451 | -0.446459607 | 0.6552735688 |
| krt18 | 13579 | PDI_U | 0.0003987716658 | 0.0008938044166 | 0.4461509234 | 0.6554953507 |
| eno2 | 13784 | PDI_U | 0.0004418673036 | 0.0009909934747 | 0.4458831615 | 0.6556886554 |
| tsnax | 11355 | PDI_U | 0.0004839600244 | 0.001085652549 | 0.4457780023 | 0.6557661214 |
| mdh1 | 11716 | PDI_U | -0.0004725421392 | 0.001060629333 | -0.4455299553 | 0.6559450574 |
| bcl2l15 | 11411 | PDI_U | 0.0004775638658 | 0.001072741828 | 0.4451806143 | 0.6561976884 |
| glo1 | 13388 | PDI_U | -0.0004428298038 | 0.0009960646271 | -0.4445793895 | 0.6566309378 |
| adamtsl5 | 11781 | PDI_U | -0.000470678884 | 0.001059090308 | -0.4444180828 | 0.6567485139 |
| asrgl1 | 11474 | PDI_U | -0.0004812134371 | 0.001084180749 | -0.4438498263 | 0.6571595402 |
| layn | 13535 | PDI_U | -0.0004176607125 | 0.0009417603871 | -0.4434893612 | 0.6574189077 |
| ngfr | 11781 | PDI_U | 0.0004727353944 | 0.001066148728 | 0.443404735 | 0.6574811623 |
| notch1 | 13800 | PDI_U | 0.0004318121498 | 0.0009743663591 | 0.4431722686 | 0.6576480888 |
| s100p | 13388 | PDI_U | 0.0004338482109 | 0.0009843347622 | 0.4407527069 | 0.6593991577 |
| igfbp7 | 13800 | PDI_U | -0.0004066058193 | 0.0009241180652 | -0.4399933673 | 0.6599488078 |
| klk10 | 13652 | PDI_U | -0.0004369220417 | 0.0009932479461 | -0.4398922177 | 0.6600221423 |
| mesd | 13632 | PDI_U | 0.0004376783688 | 0.0009964369154 | 0.4392434303 | 0.6604921273 |
| dkkl1 | 13579 | PDI_U | 0.000424543644 | 0.000967041132 | 0.439013016 | 0.6606590965 |
| rad51 | 11570 | PDI_U | -0.0004717896549 | 0.001076881283 | -0.4381073963 | 0.6613166205 |

| Protein | N_used | term | beta | se | t | p_val |
|---|---|---|---|---|---|---|
| slc9a3r1 | 11537 | PDI_U | 0.0004711450483 | 0.00107982912 | 0.4363144498 | 0.6626167723 |
| cd79b | 13801 | PDI_U | -0.0004179603871 | 0.0009587099121 | -0.4359612661 | 0.6628716576 |
| c7orf50 | 11767 | PDI_U | 0.000460736772 | 0.001059474375 | 0.4348729735 | 0.663662616 |
| rwdd1 | 13784 | PDI_U | 0.0004297213102 | 0.0009883060786 | 0.4348058961 | 0.6637101376 |
| ctrb1 | 13632 | PDI_U | 0.0004319703588 | 0.0009951816693 | 0.4340618122 | 0.6642504309 |
| smpdl3a | 13695 | PDI_U | -0.0004276267455 | 0.0009864515485 | -0.4334999993 | 0.6646584005 |
| gorasp2 | 11724 | PDI_U | -0.0004631119126 | 0.001068383253 | -0.4334698352 | 0.6646814591 |
| pla2g1b | 13777 | PDI_U | 0.0004094949203 | 0.0009474999347 | 0.4321846422 | 0.6656139904 |
| cxcl12 | 13378 | PDI_U | -0.0004327163229 | 0.001001772737 | -0.4319505879 | 0.665784294 |
| ca11 | 13308 | PDI_U | -0.0004331740426 | 0.001008206278 | -0.4296482299 | 0.66745851 |
| c1qtnf6 | 11757 | PDI_U | 0.000455626881 | 0.001065170827 | 0.4277500562 | 0.6688409446 |
| exosc10 | 11355 | PDI_U | 0.0004659438555 | 0.001089314328 | 0.4277405002 | 0.6688481803 |
| sit1 | 13378 | PDI_U | 0.0004138809131 | 0.00096877212 | 0.4272221553 | 0.6692244075 |
| yes1 | 13579 | PDI_U | 0.0004264173998 | 0.0009983512959 | 0.4271215969 | 0.6692975413 |
| palm2 | 11767 | PDI_U | 0.0003963282953 | 0.0009303279111 | 0.4260092496 | 0.670108906 |
| ranbp1 | 11474 | PDI_U | -0.0004590079011 | 0.001077721094 | -0.4259060195 | 0.6701843256 |
| dusp13 | 11730 | PDI_U | 0.0004471769965 | 0.001053038529 | 0.4246539745 | 0.6710967368 |
| prkar2a | 11750 | PDI_U | 0.0004509469214 | 0.001064428797 | 0.4236515611 | 0.6718277088 |
| sult1a1 | 13784 | PDI_U | 0.0004203630954 | 0.0009922978909 | 0.4236259084 | 0.6718452731 |
| tdo2 | 11483 | PDI_U | -0.0004540338458 | 0.001072791533 | -0.4232265373 | 0.6721379212 |
| dok2 | 13618 | PDI_U | 0.0004220154633 | 0.0009994517588 | 0.4222469565 | 0.6728514426 |
| npl | 11781 | PDI_U | 0.0003941358878 | 0.0009344921084 | 0.4217648113 | 0.673204396 |
| ada2 | 13547 | PDI_U | -0.0004076766568 | 0.0009696150594 | -0.4204520679 | 0.6741619129 |
| park7 | 13632 | PDI_U | 0.0004154116699 | 0.000988252901 | 0.4203495578 | 0.674236743 |
| itga6 | 13621 | PDI_U | 0.0004189096239 | 0.0009994538024 | 0.4191385564 | 0.6751214891 |
| ism2 | 11790 | PDI_U | -0.0004474106951 | 0.001068990467 | -0.4185357202 | 0.6755631071 |
| chrdl1 | 13691 | PDI_U | 0.0003666717957 | 0.0008777568026 | 0.4177373444 | 0.6761457219 |
| ceacam20 | 11570 | PDI_U | -0.0004485095113 | 0.001075809812 | -0.4169040904 | 0.676756304 |
| scgn | 13378 | PDI_U | -0.0004128709883 | 0.0009916430168 | -0.4163504218 | 0.6771602868 |
| tnip1 | 11767 | PDI_U | 0.0004430805281 | 0.001064309525 | 0.416307961 | 0.6771922684 |
| txnrd1 | 13632 | PDI_U | 0.0004098729487 | 0.0009846001818 | 0.4162836411 | 0.6772090211 |
| apoe | 11817 | PDI_U | -0.0004388250905 | 0.001054970001 | -0.4159597808 | 0.677446995 |
| rgcc | 11757 | PDI_U | -0.0004437097925 | 0.001067414062 | -0.415686666 | 0.6776468939 |
| bcl7b | 11730 | PDI_U | -0.0004447714354 | 0.001071527269 | -0.41508177 | 0.6780896437 |
| gpr158 | 11757 | PDI_U | 0.0004312967174 | 0.001040250328 | 0.4146085859 | 0.678436034 |
| rangap1 | 13308 | PDI_U | -0.000419563151 | 0.001012260693 | -0.414481323 | 0.6785283291 |
| slc16a1 | 13320 | PDI_U | 0.0004146409132 | 0.00100170814 | 0.4139338562 | 0.678929219 |
| rbpms2 | 11474 | PDI_U | 0.0004472842846 | 0.001080837004 | 0.4138313947 | 0.679005333 |
| erc2 | 11483 | PDI_U | -0.0004477197058 | 0.001083866578 | -0.4130764014 | 0.6795583594 |
| armcx2 | 11383 | PDI_U | -0.0004489261125 | 0.001089129851 | -0.412187869 | 0.6802094981 |
| insl5 | 11724 | PDI_U | -0.0004151067459 | 0.001007157696 | -0.4121566539 | 0.680232149 |
| il33 | 13378 | PDI_U | -0.0004162413862 | 0.001010372289 | -0.4119683314 | 0.6803692416 |
| ern1 | 11730 | PDI_U | 0.0004292335474 | 0.001043225671 | 0.4114484137 | 0.680751285 |
| havcr1 | 13736 | PDI_U | -0.0003615317913 | 0.0008796763704 | -0.4109827244 | 0.6810916191 |
| map2k6 | 13621 | PDI_U | 0.0004106073624 | 0.0009993027257 | 0.4108938681 | 0.6811568284 |
| cyp24a1 | 11355 | PDI_U | -0.0004476037232 | 0.001090276188 | -0.4105415932 | 0.6814164532 |
| hpcal1 | 13801 | PDI_U | 0.0004057485603 | 0.0009906562645 | 0.4095755257 | 0.6821237155 |
| ager | 13647 | PDI_U | 0.0003935852563 | 0.0009609616504 | 0.4095743635 | 0.6821246401 |
| mavs | 13308 | PDI_U | 0.0004140751547 | 0.001011680771 | 0.409294282 | 0.6823303042 |
| il18bp | 13689 | PDI_U | 0.0003878041254 | 0.0009509681397 | 0.4077992829 | 0.6834274232 |
| il3ra | 13378 | PDI_U | -0.000397224646 | 0.0009740754494 | -0.4077965893 | 0.6834295494 |
| arl13b | 11355 | PDI_U | -0.0004430867009 | 0.001087159379 | -0.4075637019 | 0.6836017091 |
| qsox1 | 11560 | PDI_U | -0.0004298287029 | 0.001054632912 | -0.407562383 | 0.683602541 |
| tnf | 13369 | PDI_U | -0.000397345644 | 0.0009752754239 | -0.4074189037 | 0.6837068744 |
| tank | 13378 | PDI_U | 0.0004101879793 | 0.001006898133 | 0.4073778328 | 0.6837370294 |
| insl4 | 11730 | PDI_U | 0.0004314945844 | 0.001059682672 | 0.4071922623 | 0.6838742219 |
| cdh23 | 11767 | PDI_U | -0.0004318038971 | 0.001061470721 | -0.4067977462 | 0.6841639485 |
| trim58 | 11730 | PDI_U | -0.0004333679162 | 0.001065327826 | -0.4067930132 | 0.6841674483 |
| tspyl1 | 11411 | PDI_U | 0.0004409970535 | 0.001085309431 | 0.4063330152 | 0.6845055581 |
| ltbp3 | 13308 | PDI_U | 0.0003892381295 | 0.0009585226239 | 0.406081317 | 0.6846893879 |
| itgax | 11570 | PDI_U | -0.0004378186753 | 0.001079529138 | -0.4055644815 | 0.6850701379 |
| fshb | 11474 | PDI_U | -0.0003072115014 | 0.0007577150982 | -0.4054446085 | 0.6851582941 |
| plin3 | 13618 | PDI_U | -0.0004018476233 | 0.0009929968977 | -0.4046816503 | 0.6857178935 |
| lair1 | 13658 | PDI_U | -0.0003648810644 | 0.0009022975223 | -0.4043910743 | 0.6859315005 |
| il17c | 13378 | PDI_U | -0.0003989614956 | 0.0009869262368 | -0.4042465189 | 0.6860379168 |
| arhgef1 | 11767 | PDI_U | 0.0004296047778 | 0.001063069745 | 0.4041172086 | 0.6861338829 |
| nt5c1a | 11767 | PDI_U | -0.0003813531416 | 0.0009441988922 | -0.4038906896 | 0.6863004503 |
| cinp | 11570 | PDI_U | 0.0004359861374 | 0.001080004696 | 0.4036891128 | 0.6864488148 |
| tbl1x | 13308 | PDI_U | 0.0004078369981 | 0.001012802888 | 0.402681512 | 0.6871890103 |
| pomc | 11474 | PDI_U | -0.0004158231474 | 0.001033123673 | -0.4024911617 | 0.6873300974 |

| Protein | N_used | term | beta | se | t | p_val |
|---|---|---|---|---|---|---|
| cntn2 | 13753 | PDI_U | -0.0003887055433 | 0.0009660536464 | -0.4023643457 | 0.6874221646 |
| cd5 | 13736 | PDI_U | 0.000383893278 | 0.000954229397 | 0.4023071173 | 0.6874642828 |
| cct5 | 13308 | PDI_U | 0.0004064283523 | 0.001010753636 | 0.4021042692 | 0.6876137546 |
| epha4 | 11537 | PDI_U | 0.0004323520484 | 0.001075927251 | 0.4018413401 | 0.687808247 |
| ambp | 13736 | PDI_U | 0.0003653409184 | 0.0009103700402 | 0.4013103488 | 0.6881978934 |
| dnlz | 11383 | PDI_U | -0.0004365687318 | 0.00108934609 | -0.4007621964 | 0.6886027437 |
| taldo1 | 11742 | PDI_U | -0.0004172609273 | 0.001041862334 | -0.400495261 | 0.688799067 |
| ptpn1 | 13552 | PDI_U | -0.0003998312372 | 0.001000046693 | -0.3998125689 | 0.6893008792 |
| ube2z | 11411 | PDI_U | 0.0004333663666 | 0.001085225533 | 0.3993329989 | 0.6896553367 |
| stk24 | 13320 | PDI_U | 0.0004039482244 | 0.001011978924 | 0.3991666377 | 0.689776829 |
| cd82 | 11570 | PDI_U | -0.0004307765871 | 0.001079352599 | -0.3991064526 | 0.6898221416 |
| aldh2 | 11730 | PDI_U | -0.0004257045695 | 0.001067330464 | -0.3988498256 | 0.690011129 |
| grp | 11474 | PDI_U | 0.0004290030315 | 0.001076078731 | 0.3986725311 | 0.6901419368 |
| ccl16 | 13800 | PDI_U | -0.0003587203381 | 0.0009017334966 | -0.397811925 | 0.6907749851 |
| lyn | 13579 | PDI_U | -0.0003956443848 | 0.0009977538259 | -0.3965350716 | 0.691716577 |
| izumo1 | 11304 | PDI_U | -0.0004280647979 | 0.001079659408 | -0.39648133 | 0.6917574703 |
| lrrc59 | 11781 | PDI_U | 0.0004217586037 | 0.001064321595 | 0.3962698921 | 0.6919131199 |
| dut | 11724 | PDI_U | -0.0004193451521 | 0.001058540536 | -0.3961540798 | 0.6919985816 |
| insr | 11411 | PDI_U | -0.0004276374408 | 0.001081701234 | -0.3953378504 | 0.6926009669 |
| fgf2 | 13621 | PDI_U | 0.0003944857913 | 0.000998107876 | 0.3952336224 | 0.692676676 |
| il5ra | 13621 | PDI_U | -0.0003900425142 | 0.0009869169333 | -0.3952131137 | 0.6926918098 |
| dnajb14 | 11724 | PDI_U | 0.0004219869511 | 0.001067835189 | 0.3951798513 | 0.6927173583 |
| serpinf2 | 11565 | PDI_U | -0.0004127181468 | 0.001044609665 | -0.3950931727 | 0.6927814215 |
| capg | 13523 | PDI_U | -0.0003816659954 | 0.0009694242794 | -0.3937037719 | 0.693805969 |
| npc2 | 11595 | PDI_U | 0.0003799654051 | 0.0009668376193 | 0.3929981596 | 0.694328073 |
| nars1 | 11757 | PDI_U | 0.0004182835854 | 0.001066851343 | 0.3920729802 | 0.6950114051 |
| f13b | 11740 | PDI_U | 0.0004113766424 | 0.001051980216 | 0.3910497899 | 0.6957675368 |
| lgals8 | 13784 | PDI_U | 0.0003861238169 | 0.0009885137866 | 0.3906104519 | 0.6960912405 |
| atp6v1g1 | 11474 | PDI_U | 0.0004217111551 | 0.001080276113 | 0.3903734889 | 0.6962676431 |
| ncf2 | 13607 | PDI_U | 0.0003844408669 | 0.0009859406616 | 0.3899229252 | 0.6965996495 |
| ldlr | 13668 | PDI_U | 0.0003622445169 | 0.000930723762 | 0.389207337 | 0.6971288451 |
| nt5c | 11781 | PDI_U | -0.0004126940958 | 0.001066763478 | -0.3868656028 | 0.6988627018 |
| caly | 11355 | PDI_U | -0.0004210461451 | 0.001089225452 | -0.3865555513 | 0.6990925204 |
| nrp2 | 13632 | PDI_U | -0.0003703886118 | 0.0009601336441 | -0.3857677669 | 0.6996746943 |
| itih1 | 11817 | PDI_U | -0.0004119836159 | 0.001068344549 | -0.3856280414 | 0.6997791111 |
| ddi2 | 11537 | PDI_U | 0.000411574692 | 0.001068937225 | 0.3850316767 | 0.7002210519 |
| ube2l6 | 11781 | PDI_U | -0.0004070384848 | 0.001057835068 | -0.3847844498 | 0.7004040739 |
| prdx2 | 11584 | PDI_U | -0.0004084529793 | 0.001061580851 | -0.3847591814 | 0.7004229145 |
| pdxdc1 | 11304 | PDI_U | -0.0004208258916 | 0.001094344302 | -0.3845461532 | 0.7005809368 |
| ppib | 13641 | PDI_U | 0.0003838646854 | 0.0009984128981 | 0.3844748862 | 0.7006325048 |
| mtpn | 13388 | PDI_U | 0.0003882546608 | 0.001010198123 | 0.3843351636 | 0.7007361585 |
| mzb1 | 13696 | PDI_U | 0.0003695644621 | 0.0009624757165 | 0.383972765 | 0.7010045994 |
| hbz | 11781 | PDI_U | 0.0004100551009 | 0.001068114 | 0.383905745 | 0.7010552434 |
| ptprh | 11724 | PDI_U | 0.0004030750302 | 0.001051934369 | 0.3831750745 | 0.7015969123 |
| rab2b | 11730 | PDI_U | -0.0004100502583 | 0.001071462638 | -0.3827014062 | 0.701948113 |
| lgals3bp | 11817 | PDI_U | 0.0003839639752 | 0.001003315181 | 0.382695271 | 0.7019526112 |
| stx1b | 11483 | PDI_U | -0.0004136533867 | 0.001081287173 | -0.3825564542 | 0.7020557506 |
| l3hypdh | 11781 | PDI_U | 0.0004062929027 | 0.001064858422 | 0.3815464049 | 0.7028047325 |
| eif4g1 | 13801 | PDI_U | 0.0003751409384 | 0.0009856333423 | 0.3806090179 | 0.7034992518 |
| efna4 | 13526 | PDI_U | -0.000357492643 | 0.0009398055762 | -0.3803899999 | 0.7036619152 |
| ptprm | 13602 | PDI_U | 0.0003741409053 | 0.0009836415719 | 0.3803630468 | 0.7036818859 |
| hs1bp3 | 11474 | PDI_U | 0.0004092518326 | 0.001080513293 | 0.3787568698 | 0.7048754334 |
| tgfbr1 | 11411 | PDI_U | -0.0004102086909 | 0.001083824453 | -0.3784825944 | 0.7050791698 |
| dtymk | 11781 | PDI_U | 0.0003977300193 | 0.001051689175 | 0.3781820987 | 0.7053021436 |
| nbn | 13378 | PDI_U | 0.0003724781851 | 0.0009871738406 | 0.3773177223 | 0.705943494 |
| c1r | 11817 | PDI_U | -0.0003932274618 | 0.001043288776 | -0.3769114274 | 0.7062462035 |
| rab44 | 11730 | PDI_U | 0.0004004351413 | 0.001062435991 | 0.3769028391 | 0.7062526363 |
| vegfd | 13782 | PDI_U | -0.0003557733855 | 0.0009474159252 | -0.3755197438 | 0.7072797498 |
| serpinb5 | 13388 | PDI_U | 0.0003778392265 | 0.001008120753 | 0.3747956041 | 0.7078184257 |
| ecscr | 11383 | PDI_U | -0.0004083033844 | 0.001089743056 | -0.3746785832 | 0.7079065092 |
| lrig1 | 13715 | PDI_U | -0.0003648948186 | 0.0009744737786 | -0.3744531937 | 0.708072968 |
| rexo2 | 11355 | PDI_U | -0.0004072304823 | 0.001089031135 | -0.3739383284 | 0.7084571888 |
| vps4b | 11730 | PDI_U | 0.000399656247 | 0.001070816997 | 0.3732255353 | 0.7089873439 |
| scn3b | 11383 | PDI_U | -0.0004064542742 | 0.001090256873 | -0.3728059728 | 0.7092998058 |
| aprt | 13532 | PDI_U | -0.0003718445383 | 0.0009985910768 | -0.3723691779 | 0.7096238356 |
| rpa2 | 11411 | PDI_U | 0.0004043584869 | 0.001086813832 | 0.3720586498 | 0.7098560986 |
| rilp | 13715 | PDI_U | -0.0003678343386 | 0.0009894492298 | -0.3717566576 | 0.7100797848 |
| csh1 | 11794 | PDI_U | 0.0003979032859 | 0.001070407573 | 0.3717306342 | 0.7101000989 |
| entr1 | 11474 | PDI_U | 0.0003909163862 | 0.001055353858 | 0.3704126188 | 0.7110819176 |
| cd248 | 11790 | PDI_U | 0.000378049772 | 0.001021064701 | 0.3702505548 | 0.7112024693 |

| Protein | N_used | term | beta | se | t | p_val |
|---|---|---|---|---|---|---|
| kif1c | 11355 | PDI_U | -0.0003947608893 | 0.001066211989 | -0.3702461551 | 0.7112060024 |
| serpinb6 | 13745 | PDI_U | 0.0003674673791 | 0.0009927203771 | 0.370162019 | 0.7112674826 |
| lrpap1 | 13784 | PDI_U | -0.0003575041471 | 0.0009659868511 | -0.3700921464 | 0.7113195247 |
| mif | 13605 | PDI_U | 0.0003676976454 | 0.0009947948944 | 0.3696215646 | 0.711670239 |
| top1 | 11724 | PDI_U | -0.0003877059529 | 0.001050928664 | -0.3689174787 | 0.7121959034 |
| aamdc | 11474 | PDI_U | -0.0003977403736 | 0.001079305744 | -0.368515016 | 0.7124960565 |
| lsm1 | 13308 | PDI_U | 0.0003729055128 | 0.001013067228 | 0.3680955245 | 0.7128078677 |
| ercc1 | 11304 | PDI_U | -0.0004019701448 | 0.001094185471 | -0.3673692947 | 0.7133504567 |
| tinagl1 | 13585 | PDI_U | 0.0003552052871 | 0.0009673933822 | 0.3671777104 | 0.7134921888 |
| ica1 | 13378 | PDI_U | -0.0003702414295 | 0.00100840847 | -0.3671542241 | 0.7135097949 |
| dsg3 | 13697 | PDI_U | -0.0003436292155 | 0.0009378493069 | -0.366401311 | 0.7140713059 |
| uros | 11483 | PDI_U | -0.0003966487391 | 0.001082714949 | -0.366346414 | 0.7141133562 |
| ap3b1 | 11724 | PDI_U | -0.0003906144164 | 0.001066859581 | -0.3661347973 | 0.7142711063 |
| insl3 | 11767 | PDI_U | -0.0002425666166 | 0.0006629279868 | -0.36590191 | 0.7144448554 |
| pkd2 | 11355 | PDI_U | 0.0003932224143 | 0.001074698294 | 0.3658909822 | 0.7144532489 |
| cfc1 | 13308 | PDI_U | -0.0003639624374 | 0.0009953861111 | -0.3656495036 | 0.7146324476 |
| il4r | 13621 | PDI_U | -0.0003581527171 | 0.0009810241464 | -0.3650804299 | 0.7150570422 |
| rpl14 | 11474 | PDI_U | 0.0003873644156 | 0.001061396818 | 0.3649572046 | 0.7151500853 |
| ctag1a_ctag1b | 11535 | PDI_U | -0.0003929289596 | 0.00108141288 | -0.3633477711 | 0.7163517773 |
| dab2 | 13753 | PDI_U | 0.0003595870488 | 0.000991953095 | 0.3625040847 | 0.7169809468 |
| krt8 | 11383 | PDI_U | -0.0003889627499 | 0.001073862587 | -0.3622090523 | 0.7172025465 |
| cnp | 11450 | PDI_U | -0.0003906290469 | 0.001079856881 | -0.3617414992 | 0.7175518945 |
| htra2 | 13699 | PDI_U | 0.0003596198961 | 0.0009948540029 | 0.3614800715 | 0.7177461796 |
| ccl13 | 13782 | PDI_U | -0.0003528787001 | 0.0009762622735 | -0.3614589129 | 0.7177619601 |
| htr1a | 11383 | PDI_U | -0.0003926617244 | 0.001087295508 | -0.3611361598 | 0.7180043722 |
| tpd52l2 | 11537 | PDI_U | 0.0003894643159 | 0.00107946818 | 0.3607927709 | 0.7182609799 |
| necap2 | 11355 | PDI_U | 0.0003915657383 | 0.00108646803 | 0.3604024486 | 0.7185529064 |
| serpind1 | 11790 | PDI_U | -0.0003605216194 | 0.001001731442 | -0.3598984763 | 0.7189295094 |
| mn1 | 11483 | PDI_U | 0.0003900785044 | 0.001084214575 | 0.3597798015 | 0.7190184324 |
| msln | 13555 | PDI_U | -0.000344310304 | 0.0009570798828 | -0.3597708528 | 0.7190390689 |
| gcc1 | 11767 | PDI_U | 0.0003829688975 | 0.001064783932 | 0.3596681786 | 0.7191017524 |
| trim25 | 11724 | PDI_U | 0.0003849198775 | 0.001070916923 | 0.3594301941 | 0.7192797701 |
| arhgap1 | 13308 | PDI_U | 0.0003616485115 | 0.001009042339 | 0.3584076677 | 0.7200439444 |
| defb103a_defb103b | 11317 | PDI_U | 0.0003870380932 | 0.001084687404 | 0.3568199388 | 0.7212332682 |
| cdc25a | 11304 | PDI_U | -0.0003894581641 | 0.001094414176 | -0.355859941 | 0.7219521046 |
| scn2a | 11383 | PDI_U | -0.0003866584676 | 0.001089920306 | -0.3547584768 | 0.7227771173 |
| lilra4 | 11355 | PDI_U | 0.0003851070223 | 0.001087075904 | 0.3542595517 | 0.7231509628 |
| tars1 | 11730 | PDI_U | 0.000378990988 | 0.001071512328 | 0.3536972726 | 0.7235721301 |
| hnrnpul1 | 11757 | PDI_U | -0.0003664587638 | 0.001037484855 | -0.3532184226 | 0.7239310369 |
| ythdf3 | 13378 | PDI_U | 0.0003537119732 | 0.001004163783 | 0.3522453004 | 0.72465986 |
| cdh5 | 13800 | PDI_U | -0.0003483928227 | 0.0009894310105 | -0.3521143152 | 0.7247579145 |
| lmod1 | 11757 | PDI_U | 0.0003365369282 | 0.0009582985074 | 0.3511817306 | 0.7254583144 |
| agr2 | 13219 | PDI_U | -0.0003510570459 | 0.001000061777 | -0.3510353601 | 0.72556742 |
| pcdh7 | 11724 | PDI_U | 0.0003686221134 | 0.001050280091 | 0.3509750557 | 0.7256133749 |
| znf75d | 11570 | PDI_U | 0.0003772989595 | 0.001080113351 | 0.3493142261 | 0.7268597832 |
| chm | 11483 | PDI_U | 0.000378163291 | 0.001084174275 | 0.3488030474 | 0.7272435772 |
| rnf149 | 11474 | PDI_U | -0.000361987656 | 0.00103924576 | -0.3483176644 | 0.7276080262 |
| kif22 | 11355 | PDI_U | 0.0003745557915 | 0.001077461416 | 0.3476280318 | 0.7281260015 |
| tmed10 | 11355 | PDI_U | -0.0003784275264 | 0.00108927089 | -0.3474136048 | 0.7282870597 |
| rspo1 | 13552 | PDI_U | 0.0003400764732 | 0.0009802075439 | 0.3469433339 | 0.7286392823 |
| dapp1 | 13621 | PDI_U | 0.0003466527106 | 0.001000171456 | 0.3465932849 | 0.72890225 |
| hspa2 | 11730 | PDI_U | 0.0003628770645 | 0.001047579378 | 0.3463957692 | 0.7290515237 |
| tmed4 | 11355 | PDI_U | -0.0003773786211 | 0.001089895647 | -0.3462520673 | 0.72915971 |
| tgfbr3 | 13800 | PDI_U | -0.000339417393 | 0.0009844172097 | -0.3447901862 | 0.7302573704 |
| gpr37 | 13388 | PDI_U | -0.0003339792423 | 0.0009689213678 | -0.3446917917 | 0.7303315089 |
| casp8 | 13715 | PDI_U | 0.0003400042196 | 0.0009866495743 | 0.344604841 | 0.7303967544 |
| gopc | 13621 | PDI_U | 0.0003440115222 | 0.0009984555784 | 0.3445436428 | 0.7304428045 |
| serpinb8 | 13801 | PDI_U | -0.0003281163508 | 0.0009529942156 | -0.3443004642 | 0.7306255856 |
| scpep1 | 11430 | PDI_U | 0.0003652352149 | 0.001061693383 | 0.3440119536 | 0.7308436313 |
| cep290 | 11570 | PDI_U | 0.0003714417873 | 0.001080037408 | 0.3439156686 | 0.7309159642 |
| gfral | 11450 | PDI_U | -0.0003585322272 | 0.001043539136 | -0.3435733408 | 0.7311734915 |
| grn | 13559 | PDI_U | -0.0003340396054 | 0.0009760012763 | -0.3422532465 | 0.7321656265 |
| defb118 | 11383 | PDI_U | -0.0003730095003 | 0.00109059334 | -0.3420243702 | 0.7323388732 |
| sdccag8 | 11730 | PDI_U | 0.0003651641671 | 0.001067746094 | 0.3419953201 | 0.7323605473 |
| hddc2 | 11724 | PDI_U | -0.0003651009732 | 0.001070076735 | -0.3411913942 | 0.732965622 |
| acadsb | 11781 | PDI_U | 0.0003637738523 | 0.001066972319 | 0.3409402903 | 0.7331546187 |
| pik3ip1 | 13632 | PDI_U | -0.0003133698675 | 0.0009208117515 | -0.3403191445 | 0.7336214499 |
| thsd1 | 11321 | PDI_U | -0.0003707788814 | 0.001089768602 | -0.3402363408 | 0.7336848721 |
| casp7 | 11767 | PDI_U | 0.0003631828216 | 0.001068160832 | 0.3400076194 | 0.733856865 |
| atp5f1d | 11355 | PDI_U | -0.0003703555772 | 0.001089849246 | -0.3398227587 | 0.7339962998 |

| Protein | N_used | term | beta | se | t | p_val |
|---|---|---|---|---|---|---|
| stip1 | 13605 | PDI_U | -0.0003358697842 | 0.0009895836453 | -0.3394051486 | 0.734309784 |
| ywhaq | 11537 | PDI_U | 0.0003648283684 | 0.001078987569 | 0.3381210117 | 0.7352781558 |
| pdia4 | 11790 | PDI_U | -0.0003598522294 | 0.001065741498 | -0.337654328 | 0.7356297136 |
| egln1 | 13621 | PDI_U | 0.0003218246065 | 0.0009537893702 | 0.3374168517 | 0.7358078867 |
| spry2 | 13378 | PDI_U | -0.0003395888843 | 0.001007714054 | -0.3369893304 | 0.7361302344 |
| cd300e | 13706 | PDI_U | 0.0003071409015 | 0.000911652921 | 0.3369055201 | 0.7361932868 |
| cplx2 | 11304 | PDI_U | -0.0003684042021 | 0.001093612606 | -0.3368690157 | 0.7362219039 |
| hagh | 13715 | PDI_U | -0.0003263344658 | 0.000968871853 | -0.3368190177 | 0.736258494 |
| fdx2 | 11355 | PDI_U | -0.0003648359464 | 0.001085023683 | -0.3362469892 | 0.7366908411 |
| tcl1b | 13388 | PDI_U | -0.0003392959492 | 0.001010372442 | -0.3358127509 | 0.7370173412 |
| ncr3lg1 | 11724 | PDI_U | 0.0003384777247 | 0.001008081315 | 0.3357643076 | 0.7370546219 |
| add1 | 11537 | PDI_U | 0.0003595708053 | 0.001073335573 | 0.3350031569 | 0.7376288017 |
| mansc1 | 13699 | PDI_U | -0.0003315940472 | 0.0009903914128 | -0.3348111089 | 0.7377727106 |
| fgr | 13320 | PDI_U | -0.0003331425292 | 0.0009955870502 | -0.3346191869 | 0.7379176429 |
| lto1 | 13378 | PDI_U | -0.0003374844177 | 0.001010494728 | -0.3339793949 | 0.7384003469 |
| c2orf69 | 11483 | PDI_U | -0.0003609832568 | 0.00108260311 | -0.33344007 | 0.738808215 |
| urod | 11537 | PDI_U | 0.0003572733713 | 0.001071502074 | 0.3334322722 | 0.7388140716 |
| vegfb | 11411 | PDI_U | 0.0003444398048 | 0.001034170081 | 0.3330591467 | 0.739095762 |
| sms | 11383 | PDI_U | -0.0003621796937 | 0.001087880908 | -0.3329221894 | 0.7391991569 |
| prr5 | 11411 | PDI_U | 0.0003605770571 | 0.001086539137 | 0.3318583243 | 0.7400023423 |
| sema4c | 13579 | PDI_U | -0.0003249822691 | 0.0009794203581 | -0.3318108169 | 0.7400372414 |
| prg2 | 11790 | PDI_U | 0.0003448723673 | 0.001044314191 | 0.3302381317 | 0.7412259101 |
| chl1 | 13666 | PDI_U | -0.0003241028186 | 0.0009820085286 | -0.3300407371 | 0.7413742446 |
| psmg4 | 11411 | PDI_U | -0.0003522679187 | 0.001069302571 | -0.3294370821 | 0.741831396 |
| tpmt | 13308 | PDI_U | 0.0003334806387 | 0.001012498505 | 0.3293640802 | 0.7418857024 |
| ampd3 | 11767 | PDI_U | 0.0003493958509 | 0.001061507677 | 0.3291505644 | 0.7420477507 |
| vnn1 | 11794 | PDI_U | 0.0003374052215 | 0.001025229654 | 0.3291020896 | 0.7420843745 |
| bcl2l1 | 11767 | PDI_U | -0.0003515583986 | 0.001068569429 | -0.3289991171 | 0.7421622164 |
| aspscr1 | 11757 | PDI_U | 0.0003480743838 | 0.001061367088 | 0.3279491022 | 0.7429559924 |
| fcar | 13621 | PDI_U | -0.0003217757938 | 0.000984320762 | -0.326901358 | 0.7437475215 |
| atxn10 | 13320 | PDI_U | 0.0003310546177 | 0.001012831027 | 0.3268606597 | 0.7437784177 |
| map3k5 | 13308 | PDI_U | 0.0003297655348 | 0.001012642686 | 0.3256484635 | 0.744695465 |
| rnf43 | 11570 | PDI_U | -0.0003491102371 | 0.001072595614 | -0.3254816938 | 0.744822427 |
| ppp2r5a | 11724 | PDI_U | 0.0003474758799 | 0.00106878434 | 0.325113184 | 0.7451012183 |
| cd40 | 13642 | PDI_U | 0.0003211213104 | 0.00098820926 | 0.3249527437 | 0.7452218247 |
| uso1 | 13579 | PDI_U | -0.0003243800616 | 0.0009983802475 | -0.3249063294 | 0.7452569761 |
| ephb4 | 13800 | PDI_U | -0.0003163967672 | 0.0009742883263 | -0.3247465444 | 0.7453778316 |
| il2rg | 11730 | PDI_U | 0.0003448846977 | 0.001065066672 | 0.3238151252 | 0.7460837834 |
| dgcr6 | 11383 | PDI_U | -0.0003515274426 | 0.001085604337 | -0.3238080678 | 0.746089303 |
| itgam | 13632 | PDI_U | 0.0003143875571 | 0.0009718830162 | 0.3234829211 | 0.7463345063 |
| rgs10 | 11757 | PDI_U | -0.0003457976857 | 0.001069117077 | -0.3234422995 | 0.7463660578 |
| mrpl46 | 13320 | PDI_U | -0.000326486117 | 0.001012362981 | -0.3224990672 | 0.7470797104 |
| tgm2 | 13600 | PDI_U | 0.0003180801818 | 0.0009933723755 | 0.3202023628 | 0.7488198556 |
| cadps | 11483 | PDI_U | 0.0003463950167 | 0.001084862259 | 0.3192986151 | 0.749505897 |
| atp6v1d | 13579 | PDI_U | 0.0003195035022 | 0.001001975387 | 0.3188736035 | 0.7498272705 |
| lilra3 | 11790 | PDI_U | 0.0003356543058 | 0.001055662821 | 0.3179559791 | 0.7505239659 |
| farsa | 11483 | PDI_U | -0.0003432597299 | 0.001080406064 | -0.3177136276 | 0.7507079562 |
| fgf6 | 11411 | PDI_U | 0.0003451467838 | 0.001086704793 | 0.3176085963 | 0.7507876701 |
| lyzl2 | 11304 | PDI_U | 0.0003124724889 | 0.0009846253871 | 0.3173516477 | 0.7509826593 |
| tnfsf10 | 13801 | PDI_U | 0.0003126318736 | 0.0009861868061 | 0.3170108053 | 0.7512402027 |
| bnip3l | 11411 | PDI_U | -0.0003387954902 | 0.001069985724 | -0.3166355238 | 0.7515259758 |
| stat5b | 13579 | PDI_U | 0.0003160228703 | 0.0009997625196 | 0.3160979374 | 0.751933035 |
| nptn | 13308 | PDI_U | -0.000316644181 | 0.001004698823 | -0.3151632844 | 0.7526426268 |
| cetn3 | 11724 | PDI_U | 0.0003363305626 | 0.001068357154 | 0.3148109801 | 0.7529107825 |
| relb | 11411 | PDI_U | 0.000341814926 | 0.001086851204 | 0.3145002046 | 0.753146917 |
| ephb6 | 13535 | PDI_U | 0.0003117864758 | 0.0009922946419 | 0.3142075576 | 0.75336825 |
| lhpp | 13678 | PDI_U | 0.0003079779538 | 0.000980556158 | 0.3140849723 | 0.753461297 |
| trim40 | 11483 | PDI_U | -0.0003398277332 | 0.001082317039 | -0.3139816901 | 0.7535406548 |
| egfl7 | 13308 | PDI_U | 0.0003055881404 | 0.0009736433308 | 0.3138604567 | 0.7536319487 |
| fhit | 13320 | PDI_U | 0.0003144633947 | 0.001010117826 | 0.3113135781 | 0.7555671222 |
| tnfrsf12a | 13699 | PDI_U | 0.0002821040862 | 0.00090872319 | 0.310440065 | 0.7562310575 |
| jpt2 | 11724 | PDI_U | 0.0003321707911 | 0.001070819005 | 0.3102025548 | 0.7564124473 |
| stab2 | 11790 | PDI_U | -0.0003131505517 | 0.001010059701 | -0.3100317253 | 0.7565423161 |
| cdc37 | 13699 | PDI_U | 0.0003083296992 | 0.0009962761772 | 0.3094821559 | 0.756959495 |
| mrps16 | 11411 | PDI_U | -0.0003346457251 | 0.001084434283 | -0.308590138 | 0.7576389629 |
| il3 | 11304 | PDI_U | 0.0003374639387 | 0.001094935738 | 0.3082043329 | 0.7579325406 |
| psme1 | 13532 | PDI_U | -0.0003031415076 | 0.0009845588525 | -0.3078957717 | 0.7581663863 |
| trdmt1 | 11570 | PDI_U | 0.000332280357 | 0.001079237299 | 0.3078844266 | 0.7581758242 |
| arl2bp | 11355 | PDI_U | 0.0003350280803 | 0.001088961358 | 0.3076583736 | 0.7583479459 |
| rgma | 13784 | PDI_U | 0.0002977694736 | 0.0009680132546 | 0.3076088806 | 0.7583846135 |

| Protein | N_used | term | beta | se | t | p_val |
|---|---|---|---|---|---|---|
| nos1 | 13320 | PDI_U | -0.0002879058555 | 0.0009372104818 | -0.3071944468 | 0.7587001789 |
| mmut | 11355 | PDI_U | 0.0003341570283 | 0.001089564702 | 0.3066885589 | 0.7590860685 |
| sprr1b | 11570 | PDI_U | 0.0003308509432 | 0.001079062903 | 0.3066095055 | 0.7591461407 |
| gpi | 11794 | PDI_U | 0.0003257604542 | 0.001062774093 | 0.3065190018 | 0.7592149307 |
| agbl2 | 11383 | PDI_U | -0.0003334524602 | 0.001090002954 | -0.3059188592 | 0.7596720267 |
| fcgr3b | 13640 | PDI_U | -0.0003010617897 | 0.0009864011436 | -0.3052123284 | 0.7602091046 |
| clint1 | 11304 | PDI_U | 0.0003340266224 | 0.001094513585 | 0.3051827104 | 0.7602326247 |
| a1bg | 11817 | PDI_U | 0.0003025783571 | 0.0009926978727 | 0.3048040752 | 0.7605207508 |
| padi4 | 13301 | PDI_U | 0.0003052408721 | 0.00100157135 | 0.3047619843 | 0.7605522096 |
| dhodh | 11355 | PDI_U | 0.0003290679491 | 0.001090192968 | 0.3018437643 | 0.7627767005 |
| cbx2 | 11355 | PDI_U | -0.0003286650021 | 0.001088891176 | -0.301834572 | 0.7627837081 |
| cspg5 | 11383 | PDI_U | -0.0003272325318 | 0.001084684132 | -0.3016846309 | 0.7628980024 |
| xrcc4 | 13320 | PDI_U | -0.000305044811 | 0.001011180185 | -0.3016720616 | 0.7629067819 |
| stx8 | 13621 | PDI_U | 0.0002990003436 | 0.0009942524947 | 0.3007287838 | 0.7636259119 |
| plb1 | 11767 | PDI_U | -0.0003034263697 | 0.001009057499 | -0.3007027549 | 0.7636464859 |
| crybb1 | 11781 | PDI_U | 0.0003193708128 | 0.001062617079 | 0.3005511761 | 0.7637620755 |
| ddah1 | 13579 | PDI_U | 0.0002926825311 | 0.0009763823898 | 0.2997621978 | 0.7643631441 |
| dlgap5 | 11757 | PDI_U | -0.0003201119317 | 0.001069832431 | -0.2992168888 | 0.7647798581 |
| uhrf2 | 11355 | PDI_U | -0.0003249418364 | 0.001089753372 | -0.2981792438 | 0.7655718242 |
| tnfrsf4 | 13779 | PDI_U | 0.0002827596064 | 0.000951576663 | 0.2971485298 | 0.7663575984 |
| cd3g | 11411 | PDI_U | -0.0003225643407 | 0.001086577936 | -0.2968625903 | 0.7665768268 |
| thop1 | 13557 | PDI_U | 0.0002826718536 | 0.0009548837949 | 0.2960274905 | 0.7672136256 |
| adam12 | 11411 | PDI_U | -0.0003004861158 | 0.001016754456 | -0.2955345944 | 0.7675909123 |
| vasp | 11794 | PDI_U | 0.0003146995684 | 0.001068169576 | 0.2946157385 | 0.7682926285 |
| ervv_1 | 11355 | PDI_U | 0.0003204519911 | 0.00108894614 | 0.2942771724 | 0.7685514993 |
| klk8 | 13201 | PDI_U | -0.0002846028985 | 0.000968707241 | -0.2937966048 | 0.7689179526 |
| dctd | 11537 | PDI_U | 0.0003164610046 | 0.001080896097 | 0.2927765264 | 0.7696982425 |
| nt5c3a | 13593 | PDI_U | -0.0002925046893 | 0.001001088978 | -0.2921865048 | 0.7701484925 |
| ephx2 | 13388 | PDI_U | 0.0002941858138 | 0.001007173104 | 0.2920906175 | 0.7702218702 |
| kiaa1549 | 11570 | PDI_U | -0.0003149071618 | 0.001080045616 | -0.2915683903 | 0.7706218791 |
| cd55 | 13162 | PDI_U | -0.0002869746475 | 0.0009846376899 | -0.2914520239 | 0.7707102283 |
| pcsk7 | 11730 | PDI_U | 0.0003102917719 | 0.001067416567 | 0.2906941689 | 0.7712903761 |
| cdc42bpb | 11724 | PDI_U | -0.0003106063646 | 0.00107020529 | -0.2902306385 | 0.7716449367 |
| tymp | 13555 | PDI_U | -0.0002891914102 | 0.001001999325 | -0.2886143764 | 0.7728809099 |
| cep43 | 13289 | PDI_U | 0.000290169192 | 0.001005974679 | 0.2884458207 | 0.7730100003 |
| itgb2 | 13558 | PDI_U | 0.0002746742558 | 0.0009546775424 | 0.287714169 | 0.7735699445 |
| tnfrsf8 | 13784 | PDI_U | -0.0002810681531 | 0.0009802185214 | -0.2867403002 | 0.7743154911 |
| eif2s2 | 11474 | PDI_U | -0.0003105219017 | 0.001083539443 | -0.286581078 | 0.7744382843 |
| crygd | 11767 | PDI_U | 0.0003051553044 | 0.001067415095 | 0.285882508 | 0.7749731489 |
| nid2 | 13632 | PDI_U | 0.0002836358727 | 0.0009938578729 | 0.2853887668 | 0.7753506523 |
| prc1 | 11730 | PDI_U | -0.0003051965515 | 0.001071967447 | -0.2847069211 | 0.7758737189 |
| stx6 | 13308 | PDI_U | -0.0002869115873 | 0.001010280838 | -0.2839919126 | 0.7764210006 |
| bcr | 13621 | PDI_U | 0.0002812199426 | 0.0009962949596 | 0.2822657486 | 0.7777440392 |
| apob | 11794 | PDI_U | -0.0002988545501 | 0.001070527759 | -0.2791656242 | 0.7801226231 |
| sftpd | 13618 | PDI_U | -0.0002729917801 | 0.0009795698666 | -0.2786853592 | 0.7804905345 |
| fnta | 11730 | PDI_U | -0.0002944367871 | 0.001061292285 | -0.2774323259 | 0.781453057 |
| pglyrp2 | 11647 | PDI_U | -0.0002966577857 | 0.001070071284 | -0.2772317976 | 0.7816070503 |
| prl | 13784 | PDI_U | 0.0002681150422 | 0.0009679738072 | 0.2769858442 | 0.7817951328 |
| thtpa | 11716 | PDI_U | -0.0002963525017 | 0.001070679593 | -0.2767891566 | 0.7819468956 |
| clec5a | 13568 | PDI_U | 0.0002649642978 | 0.0009582119697 | 0.2765195032 | 0.7821532976 |
| ca6 | 13597 | PDI_U | 0.0002533137109 | 0.0009176762074 | 0.2760382244 | 0.7825229064 |
| myh4 | 11355 | PDI_U | -0.0003003418074 | 0.001089824239 | -0.2755873807 | 0.7828700232 |
| ing1 | 13303 | PDI_U | 0.0002766214678 | 0.001008304999 | 0.274343049 | 0.7838252685 |
| ces3 | 13579 | PDI_U | 0.0002669614345 | 0.0009762168752 | 0.2734652937 | 0.7844997302 |
| dnmbp | 13552 | PDI_U | 0.0002711785406 | 0.00099263229 | 0.2731913351 | 0.7847103069 |
| ccl22 | 13535 | PDI_U | -0.0002612920746 | 0.000956622235 | -0.2731402899 | 0.7847495479 |
| xg | 13721 | PDI_U | 0.0002001810619 | 0.0007331900304 | 0.2730275285 | 0.7848361671 |
| plscr3 | 11747 | PDI_U | -0.000290489709 | 0.001064818486 | -0.2728067863 | 0.7850065429 |
| cd4 | 13604 | PDI_U | 0.0002598052588 | 0.0009525586865 | 0.2727446219 | 0.7850536747 |
| hpse | 11619 | PDI_U | 0.000291221363 | 0.001067818775 | 0.2727254567 | 0.7850691162 |
| znrf4 | 11730 | PDI_U | 0.0002911810821 | 0.001069698457 | 0.2722085652 | 0.7854664523 |
| rnase1 | 11794 | PDI_U | -0.0002614488665 | 0.0009629246296 | -0.2715154005 | 0.7859994135 |
| mars1 | 11517 | PDI_U | 0.0002932365809 | 0.001080006737 | 0.271513659 | 0.7860008673 |
| ubac1 | 13579 | PDI_U | -0.0002684384264 | 0.0009889341981 | -0.2714421515 | 0.7860551162 |
| cdc26 | 11698 | PDI_U | 0.0002903845826 | 0.001072242895 | 0.2708197778 | 0.7865344306 |
| jam2 | 13784 | PDI_U | 0.0002547451609 | 0.0009445473685 | 0.2697007787 | 0.7873945021 |
| dpy30 | 13705 | PDI_U | 0.0002575983607 | 0.0009555020022 | 0.2695947891 | 0.787476072 |
| ccl26 | 13621 | PDI_U | -0.000263829601 | 0.0009804519891 | -0.2690897708 | 0.7878646812 |
| itgal | 11411 | PDI_U | -0.0002870535721 | 0.001067199429 | -0.2689783787 | 0.787951189 |
| ogt | 11570 | PDI_U | -0.0002892326168 | 0.001078710245 | -0.268128182 | 0.7886054387 |

| Protein | N_used | term | beta | se | t | p_val |
|---|---|---|---|---|---|---|
| gne | 13308 | PDI_U | 0.000270535485 | 0.0010097997 | 0.2679100469 | 0.7887727159 |
| pdlim7 | 13710 | PDI_U | 0.0002663148756 | 0.0009954092144 | 0.2675431087 | 0.7890550555 |
| enah | 13378 | PDI_U | -0.0002637442547 | 0.0009862258863 | -0.2674278361 | 0.7891438957 |
| tbca | 11537 | PDI_U | -0.0002876444566 | 0.001076742838 | -0.2671431343 | 0.7893637376 |
| kif20b | 11383 | PDI_U | -0.0002911109244 | 0.001090285856 | -0.2670042199 | 0.789470754 |
| tmprss11b | 11383 | PDI_U | 0.0002902730194 | 0.001088887019 | 0.2665777205 | 0.7897991453 |
| tbc1d5 | 13621 | PDI_U | -0.0002659451433 | 0.0009985975976 | -0.2663186291 | 0.7899978617 |
| tgoln2 | 11794 | PDI_U | 0.0002777424352 | 0.001044183864 | 0.2659899705 | 0.7902515875 |
| myh9 | 11790 | PDI_U | 0.000282058099 | 0.001061222971 | 0.2657858968 | 0.7904087574 |
| hspa1a | 13695 | PDI_U | 0.0002615687999 | 0.0009865403413 | 0.2651374596 | 0.790907565 |
| ednrb | 11411 | PDI_U | 0.0002870707516 | 0.001085067059 | 0.2645649863 | 0.7913493794 |
| triap1 | 13753 | PDI_U | 0.0002607073129 | 0.0009857738199 | 0.2644697066 | 0.7914219712 |
| wars | 13632 | PDI_U | -0.0002590140772 | 0.0009809588049 | -0.2640417476 | 0.7917517448 |
| camsap1 | 11757 | PDI_U | 0.0002819670783 | 0.001068432424 | 0.2639072644 | 0.791856008 |
| clpp | 13535 | PDI_U | 0.0002631592864 | 0.000999540021 | 0.2632803899 | 0.7923384845 |
| pnliprp2 | 13658 | PDI_U | 0.0002625143053 | 0.000999058805 | 0.2627616153 | 0.7927382895 |
| matn3 | 13526 | PDI_U | 0.000257340303 | 0.0009801770916 | 0.2625447026 | 0.792905528 |
| il6 | 13579 | PDI_U | -0.0002352260553 | 0.0008993879097 | -0.2615401572 | 0.7936799561 |
| fdx1 | 11355 | PDI_U | -0.0002810401411 | 0.001075643021 | -0.2612764046 | 0.7938841047 |
| il1r1 | 13602 | PDI_U | -0.0002542837207 | 0.0009797998861 | -0.2595261791 | 0.795233217 |
| srpx | 11781 | PDI_U | -0.0002555229165 | 0.0009851534637 | -0.2593737178 | 0.7953514428 |
| eno1 | 13666 | PDI_U | 0.0002575566557 | 0.0009954804102 | 0.258725991 | 0.7958505642 |
| plpbp | 13618 | PDI_U | 0.0002577631938 | 0.001000019415 | 0.2577581896 | 0.7965974325 |
| sorbs1 | 11757 | PDI_U | 0.0002590575893 | 0.001009692943 | 0.2565706646 | 0.7975147172 |
| ppp1r12b | 11570 | PDI_U | 0.0002764571838 | 0.001078207727 | 0.25640438 | 0.7976431696 |
| dusp29 | 11483 | PDI_U | -0.0002724560079 | 0.001063578518 | -0.2561691527 | 0.7978248202 |
| gfra2 | 13579 | PDI_U | 0.0002549784961 | 0.0009960758255 | 0.2559830181 | 0.7979678311 |
| hip1r | 11738 | PDI_U | -0.0002591553908 | 0.001013688142 | -0.255655936 | 0.7982210068 |
| mtss2 | 11730 | PDI_U | 0.0002727577885 | 0.001067495075 | 0.2555119878 | 0.7983321705 |
| areg | 13579 | PDI_U | -0.0002525216428 | 0.0009898663817 | -0.2551067977 | 0.7986444809 |
| zpr1 | 11383 | PDI_U | 0.0002769803279 | 0.001088707944 | 0.2544119656 | 0.7991819082 |
| pdap1 | 11781 | PDI_U | 0.0002701704557 | 0.001066210556 | 0.2533931541 | 0.7999688434 |
| oplah | 11474 | PDI_U | 0.0002737261594 | 0.001080359819 | 0.2533657349 | 0.7999901476 |
| zfyve19 | 11724 | PDI_U | 0.0002707545273 | 0.001069949237 | 0.2530536198 | 0.8002312199 |
| rab6a | 13621 | PDI_U | 0.0002465493219 | 0.0009752559732 | 0.2528047289 | 0.8004229315 |
| sh3glb2 | 11767 | PDI_U | -0.0002674532053 | 0.001058391576 | -0.2526977835 | 0.8005061793 |
| map1lc3a | 11383 | PDI_U | -0.0002744682224 | 0.001090173023 | -0.2517657442 | 0.8012266866 |
| denr | 11411 | PDI_U | -0.0002727060063 | 0.001086359262 | -0.25102746 | 0.8017974034 |
| cacybp | 11517 | PDI_U | 0.0002701200922 | 0.001080071173 | 0.2500947151 | 0.8025185683 |
| xiap | 11411 | PDI_U | 0.0002700568428 | 0.001084487887 | 0.2490178507 | 0.8033514598 |
| snx5 | 11474 | PDI_U | -0.0002698264003 | 0.001085003296 | -0.2486871709 | 0.8036072288 |
| frmd7 | 11483 | PDI_U | 0.0002683122946 | 0.001083726383 | 0.2475830603 | 0.8044614498 |
| bloc1s2 | 11383 | PDI_U | -0.0002696676459 | 0.001089363858 | -0.2475459818 | 0.8044901797 |
| brsk2 | 11757 | PDI_U | 0.0002642707369 | 0.001067785974 | 0.2474941077 | 0.8045301773 |
| lamb1 | 11474 | PDI_U | -0.0002642009204 | 0.00107058842 | -0.2467810369 | 0.8050821066 |
| tmco5a | 11483 | PDI_U | 0.000267134616 | 0.001083508252 | 0.2465459913 | 0.8052640186 |
| pebp1 | 13632 | PDI_U | 0.0002409294212 | 0.0009813905498 | 0.2454980041 | 0.8060745523 |
| casp3 | 13800 | PDI_U | 0.0002436526733 | 0.0009928142153 | 0.2454161811 | 0.8061378539 |
| tspan8 | 11724 | PDI_U | -0.0002560792107 | 0.001043857134 | -0.2453201712 | 0.8062128328 |
| ppm1f | 11781 | PDI_U | 0.0002614307568 | 0.001066592113 | 0.2451084662 | 0.8063767213 |
| kirrel2 | 13320 | PDI_U | -0.0002417888169 | 0.0009907086852 | -0.244056422 | 0.8071908846 |
| pacs2 | 11355 | PDI_U | 0.0002647442871 | 0.001086326122 | 0.2437060858 | 0.807462867 |
| cacna1h | 11355 | PDI_U | 0.0002636324589 | 0.001088072965 | 0.2422929963 | 0.8085575207 |
| lamtor5 | 11730 | PDI_U | -0.000258681561 | 0.001070078633 | -0.2417407031 | 0.8089853183 |
| rnf5 | 11474 | PDI_U | 0.0002593251223 | 0.001082580326 | 0.2395435387 | 0.8106884232 |
| pbld | 13320 | PDI_U | -0.0002355990951 | 0.0009842682972 | -0.2393647096 | 0.8108264785 |
| enoph1 | 11730 | PDI_U | -0.000255794992 | 0.001070941121 | -0.2388506585 | 0.8112255639 |
| nos2 | 11355 | PDI_U | -0.0002586659154 | 0.001086225634 | -0.2381327666 | 0.8117824229 |
| smad2 | 11730 | PDI_U | -0.0002541219532 | 0.001067391838 | -0.2380774745 | 0.8118251679 |
| naprt | 11767 | PDI_U | 0.0002485493944 | 0.001049494608 | 0.2368277002 | 0.8127945879 |
| itga5 | 13632 | PDI_U | 0.00021906004573 | 0.000925947093 | 0.2365798856 | 0.812986284 |
| krt6c | 11355 | PDI_U | 0.0002554377003 | 0.001081128158 | 0.2362695842 | 0.8132277532 |
| adgrd1 | 11537 | PDI_U | 0.0002272880846 | 0.0009655594518 | 0.2353952252 | 0.813906174 |
| shh | 11411 | PDI_U | -0.0002554025865 | 0.001085576657 | -0.235269048 | 0.8140041433 |
| pinlyp | 11537 | PDI_U | 0.0002513693335 | 0.001073193094 | 0.2342256346 | 0.8148139755 |
| cend1 | 11383 | PDI_U | 0.0002519866743 | 0.001079345297 | 0.2334625212 | 0.8154064718 |
| fcrlb | 13308 | PDI_U | 0.000230431532 | 0.0009899728469 | 0.2327655074 | 0.8159470812 |
| spink8 | 11355 | PDI_U | -0.0002523463616 | 0.001088264439 | -0.2318796357 | 0.8166356848 |
| ugdh | 11355 | PDI_U | -0.0002518963179 | 0.001088837784 | -0.2313442107 | 0.8170515757 |
| dtd1 | 11537 | PDI_U | -0.0002494589464 | 0.001080369396 | -0.23090153 | 0.8173954007 |

| Protein | N_used | term | beta | se | t | p_val |
|---|---|---|---|---|---|---|
| fkbp14 | 11757 | PDI_U | 0.0002465229081 | 0.00106791022 | 0.2308460987 | 0.8174383876 |
| ngrn | 11767 | PDI_U | 0.0002424820013 | 0.00105197896 | 0.230500809 | 0.8177066461 |
| ceacam16 | 11724 | PDI_U | -0.000238458447 | 0.001039498869 | -0.229397505 | 0.8185639809 |
| ngf | 13552 | PDI_U | -0.0002298420048 | 0.001004278655 | -0.2288627799 | 0.8189790266 |
| cmc1 | 11781 | PDI_U | 0.0002432197427 | 0.001066371426 | 0.228081639 | 0.8195867363 |
| gpnmb | 13241 | PDI_U | -0.0002280264906 | 0.001001928413 | -0.2275876076 | 0.8199703719 |
| pgr | 11411 | PDI_U | 0.0002457891621 | 0.001083604251 | 0.2268255795 | 0.8205634399 |
| amn | 13378 | PDI_U | 0.0002254768089 | 0.0009944398788 | 0.2267374969 | 0.8206313355 |
| fgf16 | 11411 | PDI_U | 0.0002445291272 | 0.001084889023 | 0.2253955215 | 0.8216756359 |
| ube2b | 11730 | PDI_U | -0.0002408647568 | 0.001070172573 | -0.2250709491 | 0.8219280051 |
| dnph1 | 13695 | PDI_U | 0.0002217502424 | 0.0009856616931 | 0.2249760176 | 0.8220012908 |
| dbi | 13566 | PDI_U | 0.0002224613649 | 0.0009900130491 | 0.2247054876 | 0.8222117826 |
| tppp3 | 13320 | PDI_U | 0.0002249527107 | 0.001007725892 | 0.2232280747 | 0.8233614289 |
| tbc1d23 | 13579 | PDI_U | -0.0002230627463 | 0.001000539508 | -0.2229424671 | 0.8235836404 |
| ppp1cc | 11730 | PDI_U | -0.0002381456651 | 0.001069880647 | -0.222590871 | 0.8238578216 |
| vta1 | 13784 | PDI_U | 0.0002191130134 | 0.0009917628904 | 0.2209328616 | 0.8251479849 |
| ttf2 | 11483 | PDI_U | -0.0002393588802 | 0.001083634339 | -0.2208852854 | 0.8251856863 |
| cebpb | 13388 | PDI_U | 0.0002183833796 | 0.0009894919013 | 0.2207025437 | 0.8253274192 |
| gcnt1 | 13579 | PDI_U | -0.0002140636978 | 0.0009708862305 | -0.2204827827 | 0.8254984975 |
| timp2 | 11781 | PDI_U | 0.0002310901919 | 0.001050787703 | 0.2199209139 | 0.8259365624 |
| txndc5 | 13784 | PDI_U | -0.0002164314655 | 0.0009859383438 | -0.2195182558 | 0.8262496139 |
| lacrt | 11701 | PDI_U | 0.0002291375631 | 0.00104424749 | 0.2194284068 | 0.8263201751 |
| smarca2 | 13320 | PDI_U | -0.0002212621941 | 0.001010916564 | -0.2188728545 | 0.8267524491 |
| pappa | 13378 | PDI_U | 0.0002105076817 | 0.0009623133887 | 0.2187516917 | 0.8268468197 |
| tk1 | 11570 | PDI_U | 0.0002343596619 | 0.001074319018 | 0.2181471779 | 0.827318291 |
| snx2 | 11730 | PDI_U | -0.0002305834232 | 0.001069099384 | -0.2156800637 | 0.8292408962 |
| myo9b | 13378 | PDI_U | -0.000217392065 | 0.001008978033 | -0.2154576789 | 0.829413792 |
| tnfaip8 | 13378 | PDI_U | -0.0002170389028 | 0.00100976527 | -0.2149399561 | 0.8298174117 |
| clul1 | 13601 | PDI_U | 0.0002013367513 | 0.0009387389804 | 0.2144757547 | 0.8301792902 |
| frmd4b | 11355 | PDI_U | -0.0002332241882 | 0.001088944648 | -0.2141745117 | 0.8304148214 |
| prune2 | 11570 | PDI_U | 0.0002289227486 | 0.001072856476 | 0.2133768623 | 0.8310367897 |
| sparcl1 | 13632 | PDI_U | 0.0002102157747 | 0.0009876507696 | 0.2128442372 | 0.8314516433 |
| elob | 11355 | PDI_U | 0.0002306885787 | 0.001087281477 | 0.2121700623 | 0.831978174 |
| rbks | 13366 | PDI_U | 0.0002035565288 | 0.0009629484792 | 0.2113888055 | 0.832587117 |
| psap | 11683 | PDI_U | -0.0002150496079 | 0.001019474397 | -0.2109416465 | 0.8329364888 |
| npr1 | 11355 | PDI_U | -0.0002285302764 | 0.001084994002 | -0.2106281473 | 0.8331812301 |
| amigo1 | 11483 | PDI_U | 0.0002278540636 | 0.001084145637 | 0.2101692391 | 0.8335393197 |
| fgf9 | 11271 | PDI_U | -0.0002301225692 | 0.001095475264 | -0.2100664222 | 0.833619633 |
| ctf1 | 13388 | PDI_U | -0.0002118703286 | 0.001008639115 | -0.2100556338 | 0.8336274513 |
| dnpep | 11767 | PDI_U | 0.0002204959643 | 0.001051805573 | 0.2096356683 | 0.8339556718 |
| tbc1d17 | 13320 | PDI_U | -0.0002088512347 | 0.00100157595 | -0.2085226135 | 0.8348241189 |
| ca13 | 13472 | PDI_U | 0.0002096686944 | 0.001007346401 | 0.2081396173 | 0.8351231035 |
| safb2 | 11757 | PDI_U | -0.0002214444611 | 0.001067366934 | -0.2074679794 | 0.8356479942 |
| col6a3 | 13800 | PDI_U | 0.0001824266179 | 0.0008817032125 | 0.2069025215 | 0.8360890425 |
| washc3 | 11767 | PDI_U | 0.0002192540895 | 0.001062114475 | 0.2064316933 | 0.8364572914 |
| prkg1 | 11537 | PDI_U | -0.0002226788846 | 0.001080075141 | -0.2061698082 | 0.8366619125 |
| fzd10 | 11483 | PDI_U | 0.0002223916092 | 0.001084973067 | 0.2049743133 | 0.8375958308 |
| ptges2 | 11324 | PDI_U | 0.0002209323478 | 0.001081430099 | 0.2042964664 | 0.8381255065 |
| ppcdc | 13784 | PDI_U | -0.0002010955149 | 0.0009847557 | -0.204208531 | 0.8381935625 |
| epb41l5 | 11483 | PDI_U | 0.0002211802482 | 0.001084213565 | 0.204000628 | 0.8383566271 |
| casp10 | 13320 | PDI_U | 0.0002047281452 | 0.001007917198 | 0.2031200039 | 0.8390443422 |
| tppp2 | 11570 | PDI_U | -0.0002188839876 | 0.001080006422 | -0.2026691538 | 0.8393972019 |
| tek | 13699 | PDI_U | 0.000200215401 | 0.0009892136074 | 0.2023985513 | 0.8396081692 |
| stambp | 13535 | PDI_U | 0.0002016108212 | 0.0009991844464 | 0.2017753798 | 0.8400953647 |
| atp2b4 | 11355 | PDI_U | -0.0002189184013 | 0.001087661711 | -0.2012743476 | 0.8404876687 |
| fndc1 | 11767 | PDI_U | 0.0002074077093 | 0.001032133903 | 0.2009503891 | 0.8407408431 |
| tnfsf13 | 13710 | PDI_U | 0.0001899689315 | 0.0009490992169 | 0.2001570838 | 0.8413607014 |
| bcam | 13632 | PDI_U | 0.0001948858354 | 0.0009806069301 | 0.1987400144 | 0.8424690878 |
| snca | 11537 | PDI_U | 0.0002110990941 | 0.001072746078 | 0.1967838415 | 0.8440001664 |
| trim21 | 13621 | PDI_U | 0.0001939642624 | 0.0009872186532 | 0.1964754837 | 0.8442409536 |
| klrd1 | 13801 | PDI_U | 0.0001877087534 | 0.0009556624329 | 0.1964174241 | 0.844286354 |
| plxnb2 | 13735 | PDI_U | 0.000185945248 | 0.0009484481404 | 0.1960520982 | 0.8445722916 |
| scamp3 | 13579 | PDI_U | -0.0001940660006 | 0.0009927222677 | -0.1954887152 | 0.8450132988 |
| raly | 11730 | PDI_U | 0.0002069801774 | 0.001062738342 | 0.1947611836 | 0.8455832877 |
| hadh | 11355 | PDI_U | 0.0002107177541 | 0.001089035088 | 0.1934903258 | 0.8465784463 |
| gc | 11817 | PDI_U | 0.0002048952321 | 0.001062934339 | 0.1927637715 | 0.8471472934 |
| ifnl1 | 13320 | PDI_U | 0.000194253242 | 0.001008553518 | 0.1926057851 | 0.8472706529 |
| bax | 13301 | PDI_U | 0.0001946059956 | 0.001012111647 | 0.192277202 | 0.8475280134 |
| adam9 | 11724 | PDI_U | 0.0002005273526 | 0.001045203619 | 0.1918548204 | 0.847859254 |
| mmp7 | 13373 | PDI_U | 0.0001851807717 | 0.0009658916242 | 0.1917200305 | 0.8479644283 |

| Protein | N_used | term | beta | se | t | p_val |
|---|---|---|---|---|---|---|
| igfl4 | 11411 | PDI_U | -0.000208084284 | 0.001085908513 | -0.1916222973 | 0.8480414885 |
| cenpf | 11570 | PDI_U | 0.0002029503202 | 0.001065162071 | 0.1905346855 | 0.8488935122 |
| arfip1 | 11757 | PDI_U | 0.0002033084466 | 0.001069881505 | 0.1900289385 | 0.8492897381 |
| snx9 | 13618 | PDI_U | 0.0001898991561 | 0.0009996831835 | 0.1899593383 | 0.8493438274 |
| cytl1 | 11790 | PDI_U | 0.0001909980287 | 0.001007527516 | 0.1895710296 | 0.8496485572 |
| kiaa2013 | 11730 | PDI_U | 0.0002020922547 | 0.001071208091 | 0.1886582601 | 0.8503639347 |
| septin9 | 13579 | PDI_U | 0.0001881714231 | 0.001000570784 | 0.188064079 | 0.850829232 |
| clec1a | 13388 | PDI_U | -0.0001859067821 | 0.00099219054 | -0.1873700409 | 0.8513733524 |
| il10ra | 13378 | PDI_U | -0.0001875828322 | 0.00100896477 | -0.1859161368 | 0.8525133488 |
| gbp1 | 11749 | PDI_U | 0.0001940434061 | 0.001045177561 | 0.1856559242 | 0.8527178029 |
| dctn2 | 13579 | PDI_U | 0.0001851357522 | 0.001001455983 | 0.1848665896 | 0.8533364402 |
| tpsg1 | 11411 | PDI_U | -0.0002003719673 | 0.001086224555 | -0.1844664313 | 0.8536508406 |
| fxyd5 | 13378 | PDI_U | 0.0001859591759 | 0.001009532268 | 0.1842033007 | 0.8538567647 |
| tia1 | 13259 | PDI_U | 0.0001858177401 | 0.001012402854 | 0.1835413042 | 0.8543761222 |
| rspo3 | 13753 | PDI_U | -0.0001695908092 | 0.0009250058031 | -0.1833402651 | 0.8545337479 |
| akr7l | 11411 | PDI_U | -0.0001927862084 | 0.001058771392 | -0.1820848295 | 0.8555193918 |
| ccl15 | 13800 | PDI_U | 0.0001744262428 | 0.0009627359438 | 0.1811776572 | 0.8562307944 |
| map1lc3b2 | 11355 | PDI_U | -0.0001971039908 | 0.00108950258 | -0.1809119083 | 0.8564399538 |
| ppp1r14a | 11757 | PDI_U | 0.0001926207587 | 0.001067691735 | 0.1804085884 | 0.856834925 |
| fcrl5 | 13632 | PDI_U | 0.0001705117817 | 0.0009459441028 | 0.1802556633 | 0.8569545428 |
| bola1 | 11383 | PDI_U | -0.0001935200904 | 0.001076412488 | -0.179782465 | 0.8573265537 |
| fgd3 | 11757 | PDI_U | 0.0001918905537 | 0.001067878507 | 0.1796932445 | 0.8573964971 |
| jun | 13378 | PDI_U | 0.0001788798584 | 0.001002423952 | 0.1784473106 | 0.8583744015 |
| sestd1 | 13320 | PDI_U | 0.0001798673987 | 0.001012306479 | 0.177680774 | 0.8589763894 |
| irak1 | 13378 | PDI_U | 0.0001780531767 | 0.001005546852 | 0.1770709901 | 0.8594553114 |
| gm2a | 11503 | PDI_U | 0.0001781631098 | 0.001006460805 | 0.1770194217 | 0.859496254 |
| casp4 | 11570 | PDI_U | -0.0001903617382 | 0.001079867655 | -0.1762824707 | 0.8600751212 |
| ggact | 11781 | PDI_U | -0.0001876008486 | 0.001066487497 | -0.1759053427 | 0.8603713344 |
| cngb3 | 11383 | PDI_U | -0.0001915821367 | 0.001090201454 | -0.1757309496 | 0.8605084486 |
| tcn1 | 11533 | PDI_U | 0.0001878131923 | 0.001074744768 | 0.1747514367 | 0.8612780196 |
| vti1a | 11537 | PDI_U | 0.000187668644 | 0.001074177259 | 0.1747091948 | 0.8613112114 |
| ccl2 | 13526 | PDI_U | -0.0001720043277 | 0.0009869298574 | -0.1742822212 | 0.8616462815 |
| pithd1 | 11271 | PDI_U | -0.0001902331608 | 0.001095156928 | -0.17370402 | 0.8621012009 |
| gstp1 | 13246 | PDI_U | 0.0001763286304 | 0.00101513343 | 0.1736999543 | 0.8621039301 |
| thy1 | 13784 | PDI_U | -0.0001589441849 | 0.0009201834174 | -0.1727309816 | 0.8628654283 |
| cdk1 | 11304 | PDI_U | -0.0001879555343 | 0.001092336322 | -0.1720674581 | 0.8633875793 |
| txndc9 | 11767 | PDI_U | -0.000182626367 | 0.001067601738 | -0.1710622608 | 0.8641777542 |
| cfhr4 | 11791 | PDI_U | 0.0001810100846 | 0.001066077057 | 0.1697908076 | 0.8651775723 |
| txndc15 | 13699 | PDI_U | 0.0001654264639 | 0.0009906348869 | 0.1669903473 | 0.8673801109 |
| ddhd2 | 11757 | PDI_U | -0.0001778088902 | 0.001065783361 | -0.1668339896 | 0.8675035457 |
| cox5b | 13579 | PDI_U | -0.0001660329897 | 0.0009970114028 | -0.1665306828 | 0.8677418206 |
| vasn | 13420 | PDI_U | -0.0001647338275 | 0.0009916069381 | -0.1661281514 | 0.8680586053 |
| man1a2 | 11474 | PDI_U | -0.0001726597104 | 0.001044050971 | -0.1653747903 | 0.8686519114 |
| lif | 13320 | PDI_U | 0.0001663542162 | 0.001007689288 | 0.1650848313 | 0.8688797195 |
| tpk1 | 11781 | PDI_U | 0.0001746272954 | 0.001057823008 | 0.1650817708 | 0.8688824562 |
| ccnd2 | 11570 | PDI_U | 0.0001775258417 | 0.001076169703 | 0.1649608247 | 0.8689777017 |
| scarb2 | 13292 | PDI_U | 0.0001488284269 | 0.0009029134349 | 0.1648313351 | 0.8690792483 |
| dnajb6 | 11415 | PDI_U | 0.0001787821462 | 0.001085652213 | 0.1646771811 | 0.8692009975 |
| gys1 | 13388 | PDI_U | 0.0001653947905 | 0.001007380479 | 0.1641830411 | 0.8695895319 |
| klkb1 | 11544 | PDI_U | -0.00017004707 | 0.001036296087 | -0.1640912015 | 0.8696622239 |
| zhx2 | 11767 | PDI_U | 0.0001706553715 | 0.001042017401 | 0.1637740131 | 0.8699118655 |
| enpp5 | 13710 | PDI_U | 0.0001497686295 | 0.000915789721 | 0.1635404133 | 0.8700953689 |
| glt8d2 | 13320 | PDI_U | -0.0001648436759 | 0.00101320645 | -0.1626950518 | 0.8707610134 |
| tlr3 | 13801 | PDI_U | 0.0001595136247 | 0.000981799705 | 0.1624706382 | 0.870937629 |
| gapdh | 11271 | PDI_U | 0.0001756111307 | 0.0010824309 | 0.1622377288 | 0.8711215614 |
| ciapin1 | 13699 | PDI_U | 0.0001610089076 | 0.0009925026969 | 0.1622251588 | 0.8711309431 |
| ifng | 13378 | PDI_U | 0.000162521958 | 0.001004696149 | 0.1617622981 | 0.8714954888 |
| cracr2a | 13308 | PDI_U | -0.0001632341195 | 0.001011816755 | -0.161327749 | 0.8718377201 |
| tada3 | 11730 | PDI_U | -0.0001713717537 | 0.001070625042 | -0.1600670142 | 0.872831044 |
| sema4d | 13784 | PDI_U | 0.0001578688111 | 0.0009870453379 | 0.15994079 | 0.8729300619 |
| nhlrc3 | 11262 | PDI_U | 0.0001618579765 | 0.001014247884 | 0.1595842388 | 0.8732114603 |
| arg1 | 13565 | PDI_U | 0.0001593903891 | 0.001000326467 | 0.1593383703 | 0.8734046661 |
| rrp15 | 11383 | PDI_U | -0.0001735972396 | 0.001090417382 | -0.1592025609 | 0.8735121137 |
| ilkap | 13304 | PDI_U | -0.0001589405818 | 0.001010545318 | -0.1572819932 | 0.8750249999 |
| vmo1 | 13579 | PDI_U | -0.0001526175235 | 0.0009797403159 | -0.1557734443 | 0.876213921 |
| cd300lf | 13753 | PDI_U | -0.0001500226621 | 0.0009642770731 | -0.1555804512 | 0.8763660203 |
| ggct | 11763 | PDI_U | -0.0001656228805 | 0.001064646179 | -0.1555661249 | 0.8763777003 |
| dtx3 | 13579 | PDI_U | 0.0001408753495 | 0.0009110363216 | 0.1546319791 | 0.8771137577 |
| mtss1 | 11724 | PDI_U | 0.0001654407228 | 0.001070319776 | 0.1545713035 | 0.877161958 |
| dpp10 | 13621 | PDI_U | 0.0001496457419 | 0.0009682651573 | 0.1545503737 | 0.8771780877 |

| Protein | N_used | term | beta | se | t | p_val |
|---|---|---|---|---|---|---|
| cit | 11483 | PDI_U | -0.0001650275971 | 0.001071953192 | -0.1539503761 | 0.8776515713 |
| cirbp | 11724 | PDI_U | 0.0001644450984 | 0.001069437506 | 0.1537678428 | 0.8777954392 |
| serpine2 | 11781 | PDI_U | -0.0001636995634 | 0.001065355595 | -0.1536572054 | 0.8778826636 |
| syap1 | 11537 | PDI_U | 0.0001649847784 | 0.001076905108 | 0.1532027076 | 0.8782411047 |
| vnn2 | 13579 | PDI_U | -0.0001499949234 | 0.0009864800144 | -0.1520506459 | 0.8791492512 |
| apoc1 | 11790 | PDI_U | -0.0001523439047 | 0.001003346593 | -0.1518357722 | 0.8793190677 |
| cd1c | 13699 | PDI_U | -0.0001464424498 | 0.0009649157641 | -0.151767082 | 0.879372883 |
| ctsf | 13699 | PDI_U | 0.0001455108299 | 0.0009591754565 | 0.1517040797 | 0.8794225752 |
| c9orf40 | 11724 | PDI_U | 0.0001611268513 | 0.001067428731 | 0.15094858 | 0.8800188778 |
| mapk9 | 13621 | PDI_U | 0.0001494974802 | 0.0009905167673 | 0.1509287729 | 0.88003414 |
| clec6a | 13308 | PDI_U | -0.0001492133257 | 0.0009911942082 | -0.1505389403 | 0.8803417144 |
| nedd4l | 11411 | PDI_U | -0.0001627056544 | 0.00108202545 | -0.1503713748 | 0.8804742847 |
| ptk7 | 13320 | PDI_U | 0.0001517656114 | 0.001009666144 | 0.1503126675 | 0.8805202173 |
| vsnl1 | 11767 | PDI_U | -0.0001438298669 | 0.000957793724 | -0.1501678945 | 0.8806347315 |
| ece1 | 13320 | PDI_U | 0.0001515276891 | 0.001010299824 | 0.1499828917 | 0.8807803859 |
| aif1l | 11474 | PDI_U | -0.0001604567496 | 0.001077221585 | -0.1489542652 | 0.8815923419 |
| wasf3 | 13320 | PDI_U | 0.0001503098178 | 0.001013310304 | 0.1483354281 | 0.8820803045 |
| c1qtnf5 | 11537 | PDI_U | -0.0001585006262 | 0.001068749428 | -0.1483047588 | 0.8821048545 |
| gnpda2 | 11537 | PDI_U | 0.0001592857387 | 0.001078166171 | 0.1477376521 | 0.8825524 |
| pdgfra | 13618 | PDI_U | -0.0001450233669 | 0.0009849570825 | -0.1472382599 | 0.8829461443 |
| nit1 | 11474 | PDI_U | 0.0001572251828 | 0.00107361823 | 0.1464442186 | 0.8835732976 |
| ccl27 | 13618 | PDI_U | 0.0001391720004 | 0.0009543135137 | 0.1458346743 | 0.8840540628 |
| slc34a3 | 11304 | PDI_U | -0.0001580458549 | 0.001093848772 | -0.1444860195 | 0.885119274 |
| inpp5d | 11474 | PDI_U | 0.0001529521132 | 0.001063793753 | 0.1437798565 | 0.8856768374 |
| thbs4 | 13639 | PDI_U | 0.000134840708 | 0.0009387486126 | 0.1436387828 | 0.8857878379 |
| chad | 11537 | PDI_U | 0.0001518648174 | 0.001059283942 | 0.1433655429 | 0.886004001 |
| clstn2 | 13801 | PDI_U | -0.0001401797651 | 0.0009793955566 | -0.1431288555 | 0.8861905075 |
| rpe | 11570 | PDI_U | -0.000153576122 | 0.00107822809 | -0.1424337981 | 0.8867398502 |
| sult2a1 | 13621 | PDI_U | -0.0001357608864 | 0.0009556573755 | -0.1420602089 | 0.887034551 |
| golm2 | 13784 | PDI_U | 0.0001330269661 | 0.0009369179234 | 0.1419835855 | 0.8870950481 |
| magea3 | 11570 | PDI_U | -0.0001528026869 | 0.00107999428 | -0.1414847187 | 0.8874894971 |
| slit2 | 13320 | PDI_U | -0.0001411226342 | 0.001005021576 | -0.140417517 | 0.8883322435 |
| naaa | 13498 | PDI_U | -0.0001403490537 | 0.001000930294 | -0.1402186093 | 0.8884893631 |
| tp53i3 | 11271 | PDI_U | -0.0001508557657 | 0.001079235504 | -0.1397802103 | 0.8888361499 |
| fes | 13308 | PDI_U | 0.0001408327744 | 0.001007907742 | 0.1397278426 | 0.8888771433 |
| igfbp4 | 13486 | PDI_U | 0.0001237186745 | 0.0008897552408 | 0.1390479863 | 0.8894143078 |
| rras | 11483 | PDI_U | -0.000150335195 | 0.001081900763 | -0.1389546991 | 0.8894883858 |
| il36a | 11411 | PDI_U | -0.0001505485221 | 0.001086440197 | -0.1385704639 | 0.8897920322 |
| phldb2 | 11304 | PDI_U | 0.000150542735 | 0.001094804576 | 0.1375064905 | 0.8906329145 |
| dnm3 | 11757 | PDI_U | -0.0001444039729 | 0.001053302955 | -0.1370963332 | 0.8909570007 |
| fkbp7 | 13303 | PDI_U | -0.0001385944703 | 0.001012878521 | -0.136832273 | 0.8911654459 |
| lypd1 | 13320 | PDI_U | 0.0001385041575 | 0.001012545256 | 0.1367881155 | 0.891200347 |
| rarres1 | 13308 | PDI_U | 0.0001368397221 | 0.001001646803 | 0.1366147446 | 0.8913373897 |
| spart | 11537 | PDI_U | 0.0001472519448 | 0.001078239702 | 0.1365669846 | 0.8913754598 |
| erp29 | 11537 | PDI_U | -0.0001473592044 | 0.001079131358 | -0.1365535375 | 0.8913860892 |
| mllt1 | 11304 | PDI_U | -0.0001492085933 | 0.001094097634 | -0.13637594 | 0.8915265242 |
| abl1 | 13579 | PDI_U | -0.0001345498944 | 0.0009923303393 | -0.1355898223 | 0.8921475633 |
| vamp8 | 11411 | PDI_U | 0.0001463651218 | 0.001082785405 | 0.1351746349 | 0.8924761881 |
| mycbp2 | 11757 | PDI_U | -0.0001422631763 | 0.001066510619 | -0.1333912423 | 0.8938862565 |
| clu | 11817 | PDI_U | 0.000139882815 | 0.001051609364 | 0.1330178484 | 0.8941815322 |
| pm20d1 | 13547 | PDI_U | -0.00013134519 | 0.0009890923237 | -0.1327936602 | 0.8943585424 |
| pxdnl | 11730 | PDI_U | 0.0001414576499 | 0.001065322312 | 0.1327838986 | 0.8943665686 |
| tnni3 | 13388 | PDI_U | 0.0001325168002 | 0.0009995688593 | 0.1325739582 | 0.8945323231 |
| f10 | 11794 | PDI_U | 0.0001386982998 | 0.001049252045 | 0.1321877812 | 0.8948380227 |
| rcc1 | 11730 | PDI_U | -0.0001402915944 | 0.001061802808 | -0.1321258461 | 0.8948870214 |
| unc79 | 11304 | PDI_U | -0.0001438390791 | 0.001093951898 | -0.1314857438 | 0.8953934059 |
| ptprb | 11781 | PDI_U | -0.0001332497569 | 0.001017128217 | -0.1310058601 | 0.8957729109 |
| lpa | 11526 | PDI_U | -0.0001389747163 | 0.001078489457 | -0.1288605237 | 0.8974702644 |
| ido1 | 11474 | PDI_U | 0.0001354346178 | 0.001056179702 | 0.1282306577 | 0.8979686887 |
| atg4a | 13308 | PDI_U | 0.0001282853502 | 0.001002830138 | 0.1279233096 | 0.8982115988 |
| f2 | 11817 | PDI_U | 0.0001355793296 | 0.001061930884 | 0.1276724611 | 0.8984103592 |
| gfra3 | 13522 | PDI_U | 0.0001228899826 | 0.0009662601613 | 0.1271810507 | 0.8987989957 |
| chrm1 | 11483 | PDI_U | -0.0001370398816 | 0.001084425489 | -0.1263709521 | 0.8994405094 |
| atg16l1 | 11724 | PDI_U | -0.0001346021622 | 0.001068470312 | -0.1259765111 | 0.8997526802 |
| il6r | 13551 | PDI_U | -0.0001249103392 | 0.0009920007452 | -0.1259175861 | 0.8997990315 |
| gtpbp2 | 11483 | PDI_U | 0.0001363278882 | 0.001082850161 | 0.1258972784 | 0.8998154434 |
| entpd2 | 13308 | PDI_U | -0.0001262523698 | 0.001007488645 | -0.1253139383 | 0.9002769126 |
| apol1 | 11794 | PDI_U | 0.0001297796905 | 0.001039679579 | 0.1248266227 | 0.90066294 |
| foxj3 | 11411 | PDI_U | 0.0001350128175 | 0.00108338277 | 0.1246215291 | 0.9008253809 |
| ache | 11537 | PDI_U | 0.000127324943 | 0.001026479899 | 0.1240403666 | 0.9012854771 |

| Protein | N_used | term | beta | se | t | p_val |
|---|---|---|---|---|---|---|
| baiap2 | 13308 | PDI_U | 0.0001197101505 | 0.0009669367191 | 0.1238035004 | 0.9014727316 |
| aarsd1 | 13579 | PDI_U | 0.0001208786546 | 0.000982036778 | 0.1230897429 | 0.9020378567 |
| txk | 11570 | PDI_U | -0.0001320869904 | 0.001077861137 | -0.1225454615 | 0.9024691748 |
| gatd3 | 11474 | PDI_U | -0.000131850611 | 0.001078405507 | -0.1222644081 | 0.9026917622 |
| scgb3a1 | 11537 | PDI_U | 0.0001257644122 | 0.001028778388 | 0.122246359 | 0.902706044 |
| cfd | 11817 | PDI_U | 0.0001183721112 | 0.0009718297079 | 0.1218033471 | 0.9030568363 |
| prnd | 11767 | PDI_U | 0.0001284038509 | 0.001058861693 | 0.1212659328 | 0.9034824753 |
| ddx58 | 13308 | PDI_U | 0.0001217719597 | 0.001006005403 | 0.1210450355 | 0.9036571934 |
| dlg4 | 11767 | PDI_U | -0.0001281294757 | 0.001068507532 | -0.1199144338 | 0.9045529798 |
| cdnf | 13579 | PDI_U | -0.000116430341 | 0.0009719825285 | -0.1197864546 | 0.904654086 |
| pgd | 11781 | PDI_U | -0.0001256780651 | 0.001052742644 | -0.1193815657 | 0.9049751029 |
| prkab1 | 13378 | PDI_U | -0.0001180460912 | 0.001006085698 | -0.1173320438 | 0.9065986961 |
| gmpr2 | 11537 | PDI_U | -0.0001246097925 | 0.001074932206 | -0.1159233967 | 0.9077152725 |
| tdgf1 | 13320 | PDI_U | 0.0001167393487 | 0.001011792352 | 0.1153787617 | 0.908146653 |
| upk3a | 11383 | PDI_U | 0.000125289825 | 0.001090202831 | 0.1149234082 | 0.9085078601 |
| spint3 | 11724 | PDI_U | -7.5719e-05 | 0.0006636768678 | -0.1140901699 | 0.9091682702 |
| cblif | 13599 | PDI_U | 0.000110656375 | 0.0009823898807 | 0.1126399785 | 0.9103176525 |
| nap1l4 | 11698 | PDI_U | -0.0001202193956 | 0.001070945702 | -0.1122553602 | 0.9106228634 |
| hgfac | 11666 | PDI_U | -0.0001185310606 | 0.00105829085 | -0.1120023485 | 0.9108234734 |
| sart1 | 11355 | PDI_U | 0.0001195762472 | 0.001068227283 | 0.1119389563 | 0.9108737887 |
| b4gat1 | 13632 | PDI_U | -0.0001059494108 | 0.0009493563931 | -0.1116013033 | 0.9111411831 |
| abo | 11794 | PDI_U | -0.0001189763847 | 0.001070549797 | -0.1111357782 | 0.9115105706 |
| abraxas2 | 11355 | PDI_U | 0.0001203576606 | 0.001083965976 | 0.1110345373 | 0.9115909237 |
| apobr | 11474 | PDI_U | 0.0001188681226 | 0.001077660436 | 0.1103020196 | 0.9121717867 |
| lat2 | 13579 | PDI_U | -0.0001096834076 | 0.000996513989 | -0.1100671027 | 0.9123577858 |
| cryzl1 | 11474 | PDI_U | -0.0001179279803 | 0.001083003839 | -0.1088897159 | 0.9132918698 |
| ccdc80 | 13618 | PDI_U | -9.67428e-05 | 0.00088937563 | -0.1087760463 | 0.9133817274 |
| vwc2 | 13509 | PDI_U | -0.0001023810028 | 0.0009463048127 | -0.1081903013 | 0.9138463464 |
| stk4 | 13618 | PDI_U | 0.0001083086829 | 0.001001237846 | 0.1081747792 | 0.9138586459 |
| slc9a3r2 | 11724 | PDI_U | -0.0001126904134 | 0.001047408945 | -0.1075896993 | 0.9143230112 |
| pkn3 | 11570 | PDI_U | 0.0001157305655 | 0.001078939953 | 0.1072632126 | 0.91458203 |
| plcb2 | 11767 | PDI_U | -0.0001144617294 | 0.001067489949 | -0.107225112 | 0.9146122236 |
| ncr1 | 13597 | PDI_U | -0.0001049814385 | 0.0009801517063 | -0.1071073363 | 0.9147054087 |
| nudt2 | 13579 | PDI_U | -0.0001071998664 | 0.001001052714 | -0.1070871343 | 0.9147214372 |
| nme1 | 11411 | PDI_U | -0.0001158610135 | 0.00108507541 | -0.106776923 | 0.9149678346 |
| smtn | 11743 | PDI_U | -0.000113856885 | 0.00106688957 | -0.1067185285 | 0.9150141078 |
| pydc1 | 11474 | PDI_U | -0.0001118126182 | 0.00105745595 | -0.1057373767 | 0.9157925745 |
| dctn6 | 13320 | PDI_U | 0.0001066745255 | 0.001010136793 | 0.1056040392 | 0.9158981115 |
| shisa5 | 11790 | PDI_U | 0.0001015840443 | 0.0009629857847 | 0.1054886229 | 0.9159898936 |
| entpd5 | 13388 | PDI_U | -0.0001039119514 | 0.0009967137996 | -0.1042545527 | 0.916968906 |
| mcfd2 | 13547 | PDI_U | -0.0001033932603 | 0.001003010879 | -0.1030828902 | 0.9178987115 |
| igdcc3 | 11383 | PDI_U | -0.0001107980023 | 0.001089909988 | -0.1016579382 | 0.919029982 |
| mmp8 | 13767 | PDI_U | 9.77394e-05 | 0.0009714242233 | 0.1006145073 | 0.9198579464 |
| cradd | 13535 | PDI_U | 0.0001007702447 | 0.001002950486 | 0.1004737982 | 0.919969673 |
| mink1 | 11767 | PDI_U | -0.0001071660714 | 0.001067425524 | -0.1003967668 | 0.9200310486 |
| syt1 | 11767 | PDI_U | -0.0001041542387 | 0.001045616572 | -0.09961035569 | 0.9206553707 |
| alpi | 11411 | PDI_U | 0.0001074533714 | 0.001081633653 | 0.09934359118 | 0.9208672157 |
| usp25 | 11730 | PDI_U | -0.0001043212782 | 0.001067345279 | -0.09773901689 | 0.9221412045 |
| batf | 11483 | PDI_U | 0.0001054714241 | 0.001084160413 | 0.0972839654 | 0.922502589 |
| vps28 | 11570 | PDI_U | -0.000104904329 | 0.001078510785 | -0.09726776069 | 0.9225154444 |
| vps53 | 13579 | PDI_U | -9.68614e-05 | 0.0009991858373 | -0.09694029706 | 0.922775239 |
| get3 | 11781 | PDI_U | -0.000103135298 | 0.001067110377 | -0.0966491379 | 0.9230066784 |
| rnf31 | 11411 | PDI_U | 0.0001048069848 | 0.001086535945 | 0.09645974925 | 0.923157136 |
| ccl5 | 13689 | PDI_U | -9.52087e-05 | 0.0009885857375 | -0.09630793683 | 0.9232774188 |
| maged1 | 13308 | PDI_U | -9.75242e-05 | 0.00101285034 | -0.0962869053 | 0.923294162 |
| tnfrsf13b | 13677 | PDI_U | -9.2773e-05 | 0.0009667163602 | -0.09596713102 | 0.9235480849 |
| esyt2 | 11767 | PDI_U | -0.0001018562163 | 0.001061484959 | -0.09595634435 | 0.9235568804 |
| atf4 | 11304 | PDI_U | -0.0001044663086 | 0.001094860605 | -0.09541516807 | 0.9239867618 |
| spesp1 | 11781 | PDI_U | -9.22006e-05 | 0.0009757153867 | -0.09449542902 | 0.924717222 |
| gh2 | 13308 | PDI_U | -9.45313e-05 | 0.001008037957 | -0.0937775478 | 0.9252872803 |
| ubxn1 | 11537 | PDI_U | -0.0001004445333 | 0.001072804952 | -0.09362795449 | 0.9254063302 |
| serpini1 | 11517 | PDI_U | 9.80066e-05 | 0.001052681343 | 0.09310185226 | 0.925824267 |
| rnf4 | 11570 | PDI_U | -0.0001001483227 | 0.001079670154 | -0.09275825799 | 0.926097221 |
| bloc1s3 | 11767 | PDI_U | -9.88256e-05 | 0.001067326555 | -0.09259172426 | 0.9262294965 |
| lxn | 13493 | PDI_U | -9.23864e-05 | 0.0009986233599 | -0.09251374339 | 0.9262912471 |
| flt3 | 13308 | PDI_U | 9.13279e-05 | 0.0009965095509 | 0.09164776768 | 0.9269792787 |
| aida | 11483 | PDI_U | 9.91596e-05 | 0.001083770196 | 0.09149504854 | 0.9271008381 |
| c1s | 11817 | PDI_U | -9.47008e-05 | 0.001036017581 | -0.0914084451 | 0.9271696026 |
| itgb5 | 13699 | PDI_U | -8.88051e-05 | 0.0009746312386 | -0.09111661798 | 0.9274012617 |
| p4hb | 13594 | PDI_U | -8.92434e-05 | 0.0009799937142 | -0.09106523432 | 0.9274420997 |

| Protein | N_used | term | beta | se | t | p_val |
|---|---|---|---|---|---|---|
| notch2 | 11790 | PDI_U | -9.34872e-05 | 0.001040061417 | -0.08988621359 | 0.9283791652 |
| swap70 | 11724 | PDI_U | -9.44408e-05 | 0.001054688884 | -0.0895437707 | 0.9286513004 |
| wasf1 | 13388 | PDI_U | 8.88201e-05 | 0.001008844512 | 0.08804140093 | 0.9298450887 |
| cd101 | 11724 | PDI_U | -9.28531e-05 | 0.001059102638 | -0.0876715087 | 0.930139264 |
| bhlhe40 | 11383 | PDI_U | -9.50999e-05 | 0.001090612825 | -0.08719856119 | 0.9305152184 |
| yy1 | 11411 | PDI_U | 9.39993e-05 | 0.001086371139 | 0.08652594049 | 0.9310498556 |
| ninj1 | 13308 | PDI_U | 8.62824e-05 | 0.001000212919 | 0.0862640776 | 0.9312577929 |
| igsf21 | 11474 | PDI_U | -8.94056e-05 | 0.001043311419 | -0.08569402379 | 0.9317111497 |
| ndufs6 | 13308 | PDI_U | -8.56339e-05 | 0.001002120909 | -0.08545268242 | 0.9319027984 |
| ifnl2 | 11383 | PDI_U | -9.28408e-05 | 0.001090020659 | -0.08517339661 | 0.9321250396 |
| ca2 | 13567 | PDI_U | 8.30596e-05 | 0.0009766526024 | 0.08504519607 | 0.9322267156 |
| dclre1c | 11304 | PDI_U | -9.29551e-05 | 0.001094902073 | -0.08489812813 | 0.932343885 |
| h2ap | 11304 | PDI_U | 9.19165e-05 | 0.001094450679 | 0.08398411155 | 0.933070553 |
| cd302 | 13579 | PDI_U | 7.72201e-05 | 0.0009246385221 | 0.08351380905 | 0.9334442298 |
| pcyt2 | 11724 | PDI_U | 8.9018e-05 | 0.001067264411 | 0.08340763865 | 0.9335288404 |
| grhpr | 11781 | PDI_U | 8.79639e-05 | 0.001058390387 | 0.08311105765 | 0.933764647 |
| rabepk | 13308 | PDI_U | 8.40747e-05 | 0.001012221622 | 0.08305953889 | 0.9338054483 |
| ecm1 | 11475 | PDI_U | 8.87986e-05 | 0.001080560715 | 0.0821782906 | 0.9345063714 |
| git1 | 11411 | PDI_U | 8.91005e-05 | 0.00108461766 | 0.08214925227 | 0.9345294701 |
| clasp1 | 11304 | PDI_U | 8.95473e-05 | 0.001094327796 | 0.08182854531 | 0.9347845066 |
| pafah2 | 11570 | PDI_U | 8.83154e-05 | 0.00107969563 | 0.08179657822 | 0.9348098935 |
| col9a1 | 13621 | PDI_U | -7.98658e-05 | 0.0009830701434 | -0.08124118012 | 0.9352513454 |
| anp32c | 11474 | PDI_U | -8.80003e-05 | 0.0010858765 | -0.08104082733 | 0.9354108987 |
| clec7a | 13801 | PDI_U | 7.59432e-05 | 0.0009648071182 | 0.0787133649 | 0.937261757 |
| oga | 11724 | PDI_U | 8.39146e-05 | 0.001069306565 | 0.07847574187 | 0.9374509665 |
| cspg4 | 11790 | PDI_U | 7.79314e-05 | 0.0009980951593 | 0.07808017381 | 0.9377656044 |
| cope | 13320 | PDI_U | 7.91119e-05 | 0.001013706399 | 0.07804227125 | 0.9377956012 |
| tbr1 | 11483 | PDI_U | 8.4524e-05 | 0.001084592354 | 0.07793154968 | 0.937883862 |
| clec3b | 11525 | PDI_U | -8.27639e-05 | 0.001066615777 | -0.07759484057 | 0.938151695 |
| kctd5 | 11526 | PDI_U | -8.04004e-05 | 0.001036599142 | -0.07756169189 | 0.9381780637 |
| gzmh | 13388 | PDI_U | 7.53263e-05 | 0.0009761245473 | 0.07716875176 | 0.9384904542 |
| ndufa5 | 11394 | PDI_U | -8.34726e-05 | 0.001083109651 | -0.07706750366 | 0.938571199 |
| tyrp1 | 11411 | PDI_U | -8.31567e-05 | 0.001079247797 | -0.07705057959 | 0.9385846601 |
| erbin | 13579 | PDI_U | -7.6889e-05 | 0.0009995633877 | -0.07692255695 | 0.9386862871 |
| ppm1b | 11411 | PDI_U | 8.3465e-05 | 0.001085545294 | 0.07688761235 | 0.9387143017 |
| eda2r | 13753 | PDI_U | -5.91186e-05 | 0.0007787409576 | -0.07591567834 | 0.9394872885 |
| abhd14b | 13509 | PDI_U | -7.51261e-05 | 0.0009983344182 | -0.07525139513 | 0.9400158081 |
| psip1 | 13621 | PDI_U | 7.16946e-05 | 0.0009770473113 | 0.07337886854 | 0.9415057111 |
| itgb1bp2 | 13388 | PDI_U | 7.25491e-05 | 0.001008363736 | 0.07194731108 | 0.9426449145 |
| trim24 | 11535 | PDI_U | 7.64163e-05 | 0.001071685218 | 0.07130481868 | 0.9431563977 |
| mpi | 13579 | PDI_U | -7.08952e-05 | 0.0009978511142 | -0.07104791287 | 0.9433606701 |
| ivd | 13320 | PDI_U | -7.19598e-05 | 0.001013322258 | -0.07101377457 | 0.9433878597 |
| edf1 | 11757 | PDI_U | 7.48757e-05 | 0.001067741088 | 0.07012537694 | 0.9440950624 |
| ptrhd1 | 11537 | PDI_U | 7.46577e-05 | 0.001078665078 | 0.06921301387 | 0.9448212651 |
| hgs | 13308 | PDI_U | -6.96533e-05 | 0.001009423096 | -0.06900304735 | 0.9449882315 |
| iglon5 | 11304 | PDI_U | 7.54887e-05 | 0.001094563735 | 0.06896689097 | 0.9450171951 |
| glrx | 13524 | PDI_U | -6.81198e-05 | 0.000993561928 | -0.06856123715 | 0.945339889 |
| fut8 | 13552 | PDI_U | 6.77804e-05 | 0.001000110752 | 0.06777290168 | 0.9459674162 |
| folr1 | 13646 | PDI_U | -6.17544e-05 | 0.0009363600075 | -0.06595150946 | 0.9474173982 |
| hnrnpk | 13350 | PDI_U | -6.62873e-05 | 0.001005099281 | -0.06595102274 | 0.9474178071 |
| yars1 | 11724 | PDI_U | 6.93675e-05 | 0.00106875287 | 0.06490506486 | 0.9482506957 |
| csde1 | 11355 | PDI_U | 7.03327e-05 | 0.001087765465 | 0.06465798435 | 0.9484474558 |
| arhgef5 | 11383 | PDI_U | 6.96365e-05 | 0.001085854834 | 0.06413060843 | 0.9488673573 |
| twf2 | 11790 | PDI_U | 6.83289e-05 | 0.001066206813 | 0.06408593909 | 0.9489028854 |
| commd1 | 11474 | PDI_U | -6.90538e-05 | 0.001080363248 | -0.06391716036 | 0.9490373028 |
| crtam | 13552 | PDI_U | 6.24672e-05 | 0.0009835729573 | 0.0635104959 | 0.9493609391 |
| nfat5 | 11411 | PDI_U | -6.81121e-05 | 0.001084785025 | -0.06278856494 | 0.9499359726 |
| itprip | 11383 | PDI_U | -6.74688e-05 | 0.001086123337 | -0.06211895682 | 0.9504691926 |
| casq2 | 11355 | PDI_U | 6.76421e-05 | 0.001088929007 | 0.06211801534 | 0.950469945 |
| krt14 | 12884 | PDI_U | 6.34772e-05 | 0.001026570108 | 0.06183429975 | 0.9506957485 |
| cst7 | 13661 | PDI_U | 6.0193e-05 | 0.0009789552999 | 0.0614870103 | 0.9509722594 |
| obp2b | 13513 | PDI_U | 4.85758e-05 | 0.0008071683942 | 0.06018045871 | 0.9520127999 |
| wfdc1 | 11489 | PDI_U | 6.38655e-05 | 0.001070266299 | 0.05967254491 | 0.9524174777 |
| kel | 13320 | PDI_U | 5.78565e-05 | 0.0009761465388 | 0.05927033378 | 0.9527376797 |
| saa4 | 11817 | PDI_U | -6.13638e-05 | 0.001044818713 | -0.05873155299 | 0.9531669212 |
| ccl18 | 13454 | PDI_U | 5.49113e-05 | 0.0009472125676 | 0.0579714482 | 0.953772233 |
| scin | 11383 | PDI_U | 6.27087e-05 | 0.001089840986 | 0.05753932349 | 0.9541165934 |
| ccl17 | 13628 | PDI_U | 5.62223e-05 | 0.0009898512939 | 0.05679877243 | 0.9547063252 |
| blmh | 13800 | PDI_U | 5.47847e-05 | 0.0009755211435 | 0.05615936749 | 0.9552156638 |
| mtdh | 11411 | PDI_U | -6.02627e-05 | 0.001074530246 | -0.05608283932 | 0.9552767976 |

| Protein | N_used | term | beta | se | t | p_val |
|---|---|---|---|---|---|---|
| sox2 | 11732 | PDI_U | -6.00641e-05 | 0.001071771662 | -0.05604191377 | 0.9553093726 |
| mfap3l | 11483 | PDI_U | 6.05959e-05 | 0.001083830382 | 0.05590899159 | 0.9554152815 |
| nup50 | 11355 | PDI_U | -6.07517e-05 | 0.00109001741 | -0.0557346224 | 0.9555541993 |
| nsfl1c | 13784 | PDI_U | 5.41272e-05 | 0.0009780095075 | 0.05534422041 | 0.9558650361 |
| setmar | 13510 | PDI_U | 5.08714e-05 | 0.0009240089579 | 0.05505507293 | 0.9560954032 |
| acp6 | 13732 | PDI_U | -5.39547e-05 | 0.0009895195186 | -0.05452619261 | 0.9565167348 |
| sugp1 | 11724 | PDI_U | -5.74362e-05 | 0.001068713264 | -0.05374330616 | 0.9571405959 |
| thbs2 | 13632 | PDI_U | -5.18644e-05 | 0.0009735919592 | -0.05327121199 | 0.9575165984 |
| tigar | 13303 | PDI_U | 5.35896e-05 | 0.001009843524 | 0.05306727174 | 0.9576791056 |
| cyb5r2 | 11590 | PDI_U | 5.55114e-05 | 0.001063755953 | 0.05218431858 | 0.9583827281 |
| agrn | 13710 | PDI_U | 4.82479e-05 | 0.000930859403 | 0.05183153521 | 0.9586636836 |
| mindy1 | 11730 | PDI_U | 5.54568e-05 | 0.001071132397 | 0.05177399691 | 0.9587096577 |
| bmp6 | 13364 | PDI_U | -5.15857e-05 | 0.001004150712 | -0.05137251461 | 0.9590294545 |
| rbm25 | 11304 | PDI_U | 5.57412e-05 | 0.001094904835 | 0.0509096374 | 0.9593984264 |
| lpp | 11355 | PDI_U | -5.45368e-05 | 0.001088078727 | -0.05012206567 | 0.9600259985 |
| tctn3 | 11781 | PDI_U | -5.09981e-05 | 0.001021639039 | -0.0499178841 | 0.9601886727 |
| ahnak2 | 11474 | PDI_U | 5.24908e-05 | 0.001055512434 | 0.0497301384 | 0.9603383057 |
| lect2 | 11781 | PDI_U | -4.57453e-05 | 0.0009313536225 | -0.04911702955 | 0.9608268656 |
| nfya | 11355 | PDI_U | -5.26105e-05 | 0.001081987397 | -0.04862394885 | 0.9612198402 |
| cxcl11 | 13784 | PDI_U | 4.69935e-05 | 0.0009777841475 | 0.04806119137 | 0.9616681721 |
| fabp5 | 13739 | PDI_U | 4.6432e-05 | 0.0009701458539 | 0.04786083616 | 0.9618278481 |
| cd7 | 11388 | PDI_U | 4.95801e-05 | 0.001067952624 | 0.04642537295 | 0.9629720285 |
| extl1 | 11355 | PDI_U | 4.97069e-05 | 0.001080808964 | 0.04599048784 | 0.9633186411 |
| cpox | 11411 | PDI_U | 4.90867e-05 | 0.001078074176 | 0.04553181156 | 0.9636842168 |
| il1rap | 13632 | PDI_U | -4.23152e-05 | 0.0009491604805 | -0.04458169897 | 0.9644413838 |
| otoa | 11383 | PDI_U | -4.78898e-05 | 0.001090128238 | -0.04393044034 | 0.9649606222 |
| terf1 | 11411 | PDI_U | -4.75734e-05 | 0.001086405266 | -0.04378977326 | 0.965072746 |
| ctsb | 13621 | PDI_U | -4.15853e-05 | 0.0009660835229 | -0.04304524133 | 0.9656661014 |
| lpo | 13212 | PDI_U | -4.27136e-05 | 0.000998061901 | -0.0427965014 | 0.9658644002 |
| rassf2 | 13308 | PDI_U | 4.23609e-05 | 0.0009903694871 | 0.04277278124 | 0.9658833038 |
| col15a1 | 11790 | PDI_U | -3.98519e-05 | 0.0009698257071 | -0.04109180581 | 0.9672234036 |
| st13 | 11537 | PDI_U | 4.40812e-05 | 0.001074523252 | 0.04102398999 | 0.9672774813 |
| cdkn2d | 13579 | PDI_U | -4.08877e-05 | 0.001000625017 | -0.0408621166 | 0.9674064201 |
| dnajc6 | 11474 | PDI_U | 4.40065e-05 | 0.001079549853 | 0.0407637212 | 0.9674849716 |
| cd83 | 13621 | PDI_U | -3.85008e-05 | 0.0009496838445 | -0.04054070002 | 0.9676626545 |
| brdt | 11355 | PDI_U | -4.38787e-05 | 0.001090177135 | -0.04024912926 | 0.9678952182 |
| epcam | 13647 | PDI_U | -3.93443e-05 | 0.0009815664208 | -0.04008322581 | 0.9680273618 |
| spink2 | 11537 | PDI_U | -3.86203e-05 | 0.0009696046227 | -0.03983099202 | 0.9682285577 |
| mthfsd | 11570 | PDI_U | 4.27822e-05 | 0.001075789679 | 0.03976816929 | 0.9682786402 |
| mgmt | 13621 | PDI_U | -3.96968e-05 | 0.001000095833 | -0.03969300755 | 0.9683384587 |
| vat1 | 13579 | PDI_U | 3.79261e-05 | 0.0009877173799 | 0.0383977408 | 0.9693711272 |
| scara5 | 13784 | PDI_U | 3.40609e-05 | 0.0009101606736 | 0.0374229302 | 0.9701483322 |
| prg3 | 11790 | PDI_U | -3.90311e-05 | 0.00105682779 | -0.03693235418 | 0.9705395683 |
| pdia2 | 11304 | PDI_U | 4.01642e-05 | 0.001094186112 | 0.03670693394 | 0.9707193287 |
| igbp1 | 11757 | PDI_U | -3.80835e-05 | 0.001061536284 | -0.03587586567 | 0.9713819496 |
| pms1 | 11483 | PDI_U | -3.8267e-05 | 0.001081214087 | -0.03539266003 | 0.9717672533 |
| gas2 | 11570 | PDI_U | -3.77135e-05 | 0.00106765331 | -0.03532368691 | 0.9718222457 |
| tfpi2 | 13654 | PDI_U | -3.31237e-05 | 0.0009422830186 | -0.03515262104 | 0.9719585561 |
| ncln | 13378 | PDI_U | -3.538e-05 | 0.00101000695 | -0.03502943229 | 0.9720567948 |
| elavl4 | 11383 | PDI_U | 3.8123e-05 | 0.001089503295 | 0.03499113999 | 0.97208742 |
| cx3cl1 | 13552 | PDI_U | -3.41362e-05 | 0.0009901850011 | -0.0344746084 | 0.972499198 |
| eif4e | 11411 | PDI_U | -3.60006e-05 | 0.00108343671 | -0.0332281936 | 0.9734931971 |
| sh3bp1 | 11724 | PDI_U | 3.50702e-05 | 0.001064954591 | 0.03293115172 | 0.9737300519 |
| lrp2 | 11483 | PDI_U | 3.55916e-05 | 0.001084875865 | 0.03280707319 | 0.9738290084 |
| cd164l2 | 11483 | PDI_U | -3.53612e-05 | 0.001084759129 | -0.03259817643 | 0.9739955912 |
| sod1 | 13384 | PDI_U | 3.23349e-05 | 0.0009940645442 | 0.03252794843 | 0.9740515137 |
| nudt16 | 11705 | PDI_U | -3.40809e-05 | 0.00106856961 | -0.03189391643 | 0.9745571945 |
| dock9 | 11383 | PDI_U | -3.44876e-05 | 0.001090217412 | -0.0316336524 | 0.9747647613 |
| epn1 | 11570 | PDI_U | 3.41413e-05 | 0.001079456491 | 0.03162826644 | 0.9747690474 |
| sag | 11383 | PDI_U | 3.44212e-05 | 0.001090134348 | 0.03157515846 | 0.9748114084 |
| ist1 | 11635 | PDI_U | 3.37821e-05 | 0.001073396196 | 0.03147214414 | 0.9748935473 |
| dgka | 11411 | PDI_U | -3.37232e-05 | 0.001081988928 | -0.03116778867 | 0.975136274 |
| reps1 | 11411 | PDI_U | 3.3757e-05 | 0.00108631885 | 0.03107465234 | 0.9752105484 |
| cep170 | 11474 | PDI_U | -3.2312e-05 | 0.001082618739 | -0.0298461195 | 0.9761902967 |
| map2k1 | 11724 | PDI_U | 3.15006e-05 | 0.001065568086 | 0.02956223632 | 0.9764166869 |
| kir2ds4 | 11724 | PDI_U | -3.02302e-05 | 0.001072668549 | -0.02818224461 | 0.9775172785 |
| mfap3 | 13388 | PDI_U | -2.73056e-05 | 0.00101072399 | -0.02701586048 | 0.9784474868 |
| agxt | 13618 | PDI_U | -2.60652e-05 | 0.0009664073176 | -0.02697123074 | 0.9784830758 |
| amy2a | 13121 | PDI_U | 2.59889e-05 | 0.0009850576601 | 0.02638316238 | 0.9789521255 |
| fam172a | 11474 | PDI_U | 2.76135e-05 | 0.001083202105 | 0.02549247087 | 0.9796625977 |

| Protein | N_used | term | beta | se | t | p_val |
|---|---|---|---|---|---|---|
| per3 | 11411 | PDI_U | 2.70692e-05 | 0.001074929023 | 0.0251823148 | 0.979909984 |
| myl4 | 11474 | PDI_U | 2.39404e-05 | 0.00105662545 | 0.02265743649 | 0.9819239223 |
| dctn1 | 13753 | PDI_U | 2.22344e-05 | 0.0009899189287 | 0.02246087684 | 0.982080646 |
| pcbp2 | 11757 | PDI_U | -2.23453e-05 | 0.001066250407 | -0.02095687079 | 0.9832804162 |
| eif5 | 11355 | PDI_U | -2.27876e-05 | 0.001089542797 | -0.02091478474 | 0.9833140006 |
| cbs | 11474 | PDI_U | -2.18566e-05 | 0.001055136224 | -0.02071449074 | 0.9834737701 |
| itgav | 13753 | PDI_U | 1.89454e-05 | 0.0009343682148 | 0.02027619792 | 0.9838233376 |
| matn2 | 13677 | PDI_U | 1.90664e-05 | 0.0009633004557 | 0.01979279534 | 0.9842089542 |
| nexn | 11794 | PDI_U | 1.99115e-05 | 0.001070045454 | 0.0186081338 | 0.9851540292 |
| akr1b10 | 11483 | PDI_U | -1.964e-05 | 0.001078190496 | -0.01821573172 | 0.9854670694 |
| appl2 | 11537 | PDI_U | 1.9486e-05 | 0.001080845456 | 0.01802849779 | 0.9856164312 |
| pmch | 11355 | PDI_U | 1.92771e-05 | 0.001087513968 | 0.01772581058 | 0.9858579018 |
| qpct | 13644 | PDI_U | -1.65281e-05 | 0.000971143612 | -0.01701925624 | 0.9864215028 |
| aifm1 | 13560 | PDI_U | -1.61614e-05 | 0.0009829041747 | -0.01644251364 | 0.9868816055 |
| ndufb7 | 11724 | PDI_U | 1.64268e-05 | 0.001069385296 | 0.01536095585 | 0.9877444741 |
| cpb2 | 11790 | PDI_U | 1.52457e-05 | 0.001054325185 | 0.01446011541 | 0.9884631441 |
| coro1a | 13394 | PDI_U | 1.3071e-05 | 0.001005324477 | 0.0130018066 | 0.9896265453 |
| idi2 | 13320 | PDI_U | -1.15228e-05 | 0.0009805742339 | -0.01175109143 | 0.9906243775 |
| klk1 | 13542 | PDI_U | 1.15565e-05 | 0.0009952093724 | 0.011612108 | 0.9907352577 |
| echdc3 | 11781 | PDI_U | -1.18608e-05 | 0.001042211025 | -0.01138044387 | 0.9909201084 |
| atxn3 | 11767 | PDI_U | -1.10097e-05 | 0.001058515381 | -0.01040111432 | 0.9917014376 |
| ctse | 11537 | PDI_U | 1.08963e-05 | 0.001075481686 | 0.01013153106 | 0.9919165214 |
| crh | 13278 | PDI_U | 1.01085e-05 | 0.000997921775 | 0.01012955114 | 0.9919180781 |
| itgb1 | 13624 | PDI_U | 8.87444e-06 | 0.0009672328516 | 0.009175076743 | 0.9926795851 |
| maea | 13308 | PDI_U | -7.59028e-06 | 0.00101210617 | -0.007499492746 | 0.9940164391 |
| prdx1 | 13290 | PDI_U | 6.99478e-06 | 0.001013219959 | 0.006903516322 | 0.9944919384 |
| robo4 | 11474 | PDI_U | -7.27263e-06 | 0.001081344906 | -0.006725544707 | 0.9946339492 |
| snrpb2 | 11767 | PDI_U | 6.62247e-06 | 0.001030284017 | 0.0064278147 | 0.9948714903 |
| drg2 | 13579 | PDI_U | -5.70438e-06 | 0.001000825152 | -0.005699681113 | 0.9954524209 |
| siglec6 | 13753 | PDI_U | 5.19512e-06 | 0.000977504171 | 0.005314674115 | 0.9957596007 |
| mb | 13800 | PDI_U | -4.38415e-06 | 0.0008371045784 | -0.005237274207 | 0.9958213546 |
| fkbp1b | 13695 | PDI_U | -4.90536e-06 | 0.0009964799209 | -0.004922691762 | 0.9960723479 |
| ddt | 11790 | PDI_U | -4.9811e-06 | 0.001024177466 | -0.004863513776 | 0.9961195751 |
| elac1 | 11757 | PDI_U | 4.69966e-06 | 0.001068162482 | 0.004399763766 | 0.9964895825 |
| dnajb2 | 11537 | PDI_U | -3.73745e-06 | 0.001067931067 | -0.003499715261 | 0.9972076975 |
| pspn | 13621 | PDI_U | 2.4313e-06 | 0.0007184050423 | 0.003384303004 | 0.9972997716 |
| sirt2 | 13579 | PDI_U | 3.22761e-06 | 0.0009980894769 | 0.003233784743 | 0.9974198651 |
| oscar | 13677 | PDI_U | -1.99986e-06 | 0.0009781266922 | -0.002044581398 | 0.9983686911 |
| ass1 | 11355 | PDI_U | -1.56153e-06 | 0.001034885957 | -0.001508889373 | 0.9987961075 |
| babam1 | 11411 | PDI_U | -1.37572e-06 | 0.001086445948 | -0.001266255566 | 0.9989896967 |
| cox6b1 | 11781 | PDI_U | -1.12828e-06 | 0.001067038562 | -0.001057393421 | 0.9991563402 |

## Supplementary Table 5. Proteome-wide association analysis of the healthy plant-based diet index (PDI-H) in the UK Biobank

| Protein | N_used | term | beta | se | t | p_val |
|---|---|---|---|---|---|---|
| fgf21 | 13732 | PDI_H | -0.005883642 | 0.00085077 | -6.915664881 | 4.86e-12 |
| chi3l1 | 13317 | PDI_H | -0.005764028 | 0.000872949 | -6.602938033 | 4.18e-11 |
| igfbp4 | 13486 | PDI_H | -0.00486905 | 0.000820356 | -5.935288872 | 3.01e-09 |
| tnfrsf10b | 13552 | PDI_H | -0.004760495 | 0.000809507 | -5.880734871 | 4.18e-09 |
| gpr37 | 13388 | PDI_H | -0.005197376 | 0.000891467 | -5.830137919 | 5.67e-09 |
| stc1 | 13784 | PDI_H | -0.004741624 | 0.000846173 | -5.603609223 | 2.14e-08 |
| asgr1 | 13552 | PDI_H | -0.004610785 | 0.000824972 | -5.589019117 | 2.33e-08 |
| ddc | 13599 | PDI_H | 0.004875803 | 0.000906402 | 5.379294841 | 7.6e-08 |
| polr2f | 13579 | PDI_H | -0.004590215 | 0.000863636 | -5.314990203 | 1.08e-07 |
| grpel1 | 13579 | PDI_H | -0.004773826 | 0.000901911 | -5.293010857 | 1.22e-07 |
| bst2 | 13784 | PDI_H | -0.004603874 | 0.000889578 | -5.175345809 | 2.31e-07 |
| relt | 13632 | PDI_H | -0.004072957 | 0.00079525 | -5.121603227 | 3.07e-07 |
| adamts8 | 13699 | PDI_H | 0.004628563 | 0.000905094 | 5.113900466 | 3.2e-07 |
| selenop | 11817 | PDI_H | 0.004961593 | 0.000979457 | 5.065657819 | 4.13e-07 |
| tnfrsf6b | 13552 | PDI_H | -0.004385649 | 0.000869412 | -5.044383038 | 4.61e-07 |
| ly6d | 13592 | PDI_H | -0.004430931 | 0.0008921 | -4.96685209 | 6.89e-07 |
| ccl16 | 13800 | PDI_H | -0.004122969 | 0.000830516 | -4.964346171 | 6.98e-07 |
| cd99l2 | 13632 | PDI_H | -0.004305818 | 0.000872768 | -4.933518644 | 8.17e-07 |
| fstl3 | 13650 | PDI_H | -0.003914075 | 0.000794499 | -4.92646785 | 8.47e-07 |
| ccl3 | 13621 | PDI_H | -0.004159278 | 0.000848386 | -4.902580186 | 9.57e-07 |
| fabp4 | 13800 | PDI_H | -0.003269052 | 0.000670636 | -4.874554084 | 1.1e-06 |
| cntn1 | 13800 | PDI_H | 0.004069065 | 0.000841179 | 4.837333308 | 1.33e-06 |
| cfb | 11581 | PDI_H | -0.004408899 | 0.000913445 | -4.826669112 | 1.41e-06 |
| tnfrsf10a | 13552 | PDI_H | -0.004109436 | 0.000855688 | -4.802494911 | 1.58e-06 |
| spon2 | 13556 | PDI_H | -0.004240752 | 0.000883709 | -4.798811029 | 1.61e-06 |
| tff1 | 13564 | PDI_H | -0.00417769 | 0.000871784 | -4.792113702 | 1.67e-06 |
| cntn5 | 13703 | PDI_H | 0.004033887 | 0.000843728 | 4.781029502 | 1.76e-06 |
| cd302 | 13579 | PDI_H | -0.00406902 | 0.000853079 | -4.769805507 | 1.86e-06 |
| b2m | 11817 | PDI_H | -0.004401322 | 0.000924427 | -4.761137206 | 1.95e-06 |
| adm | 13753 | PDI_H | -0.003536364 | 0.000749527 | -4.718128375 | 2.4e-06 |
| prtg | 13753 | PDI_H | 0.004269531 | 0.000910815 | 4.687592781 | 2.79e-06 |
| rspo3 | 13753 | PDI_H | -0.00397365 | 0.000854346 | -4.651099752 | 3.33e-06 |
| agrn | 13710 | PDI_H | -0.00395349 | 0.000857955 | -4.608039758 | 4.1e-06 |
| xcl1 | 13753 | PDI_H | -0.004149315 | 0.000901842 | -4.600933248 | 4.24e-06 |
| timp4 | 13632 | PDI_H | -0.003955309 | 0.000861657 | -4.590352352 | 4.46e-06 |
| calcb | 11767 | PDI_H | -0.004502375 | 0.000981087 | -4.589171087 | 4.5e-06 |
| tpp1 | 13564 | PDI_H | -0.004017001 | 0.000880789 | -4.560683202 | 5.14e-06 |
| npdc1 | 13618 | PDI_H | -0.003975811 | 0.000871881 | -4.560038246 | 5.16e-06 |
| efna1 | 13602 | PDI_H | -0.004002648 | 0.000879797 | -4.549511398 | 5.42e-06 |
| gdf15 | 13800 | PDI_H | -0.003309652 | 0.000733931 | -4.509484104 | 6.55e-06 |
| cst3 | 13659 | PDI_H | -0.003619157 | 0.000806397 | -4.488057744 | 7.25e-06 |
| efna4 | 13526 | PDI_H | -0.003881557 | 0.000865665 | -4.483902905 | 7.39e-06 |
| itga11 | 13621 | PDI_H | 0.004012041 | 0.000895026 | 4.482597829 | 7.43e-06 |
| col6a3 | 13800 | PDI_H | -0.00363767 | 0.000812199 | -4.478790832 | 7.57e-06 |
| tnfrsf1a | 13632 | PDI_H | -0.003679635 | 0.000822076 | -4.47602873 | 7.67e-06 |
| cd59 | 13130 | PDI_H | -0.003637828 | 0.000815627 | -4.460163483 | 8.26e-06 |
| spink1 | 13632 | PDI_H | -0.003975445 | 0.000891581 | -4.458872926 | 8.31e-06 |
| tff3 | 13610 | PDI_H | -0.003943858 | 0.000887911 | -4.441724211 | 8.99e-06 |
| rnase4 | 11399 | PDI_H | -0.004065332 | 0.00091663 | -4.435085804 | 9.29e-06 |
| lgals9 | 13642 | PDI_H | -0.003572787 | 0.000809821 | -4.411821364 | 1.03e-05 |
| tnfrsf1b | 13632 | PDI_H | -0.003768097 | 0.000859245 | -4.385357718 | 1.17e-05 |
| glo1 | 13388 | PDI_H | -0.004019647 | 0.000916948 | -4.38372227 | 1.18e-05 |
| echdc3 | 11781 | PDI_H | -0.004226299 | 0.000968337 | -4.364490708 | 1.29e-05 |
| nectin4 | 13699 | PDI_H | -0.003930734 | 0.000901219 | -4.361573686 | 1.3e-05 |
| ceacam16 | 11724 | PDI_H | 0.004163796 | 0.000965961 | 4.310523776 | 1.64e-05 |
| gfra1 | 13732 | PDI_H | -0.003626864 | 0.000845833 | -4.287919248 | 1.82e-05 |
| prss8 | 13598 | PDI_H | -0.003411564 | 0.000796716 | -4.282034875 | 1.86e-05 |
| oxt | 13784 | PDI_H | -0.00357813 | 0.000835809 | -4.28103501 | 1.87e-05 |
| shisa5 | 11790 | PDI_H | -0.003824542 | 0.000895145 | -4.2725401 | 1.95e-05 |
| ogn | 13632 | PDI_H | -0.003557152 | 0.000836159 | -4.254159881 | 2.11e-05 |
| klk14 | 13753 | PDI_H | -0.003766859 | 0.000891944 | -4.223202466 | 2.42e-05 |
| nell1 | 13699 | PDI_H | 0.003856333 | 0.000916743 | 4.206561005 | 2.61e-05 |
| hla_e | 13621 | PDI_H | -0.003740932 | 0.000890338 | -4.201699068 | 2.67e-05 |
| lgals1 | 13618 | PDI_H | -0.003543756 | 0.000844693 | -4.195316286 | 2.74e-05 |
| pi3 | 13441 | PDI_H | -0.003571167 | 0.000851427 | -4.19432847 | 2.75e-05 |
| mxra8 | 11537 | PDI_H | 0.003740304 | 0.00089192 | 4.193543427 | 2.77e-05 |
| vsig4 | 13613 | PDI_H | -0.003455547 | 0.00082931 | -4.166773528 | 3.11e-05 |
| ctsd | 12770 | PDI_H | -0.003594129 | 0.000864007 | -4.159838312 | 3.21e-05 |

| Protein | N_used | term | beta | se | t | p_val |
|---|---|---|---|---|---|---|
| prelp | 13658 | PDI_H | -0.003579557 | 0.000864582 | -4.140215595 | 3.49e-05 |
| edn1 | 11474 | PDI_H | -0.003958321 | 0.000957616 | -4.133517212 | 3.6e-05 |
| col18a1 | 13692 | PDI_H | -0.003488605 | 0.000849351 | -4.107377265 | 4.03e-05 |
| tnfrsf17 | 11537 | PDI_H | -0.004061595 | 0.000989691 | -4.103902933 | 4.09e-05 |
| sfrp4 | 11537 | PDI_H | 0.003958783 | 0.000964798 | 4.1032253 | 4.1e-05 |
| itgav | 13753 | PDI_H | 0.003522437 | 0.000863149 | 4.080916472 | 4.51e-05 |
| col15a1 | 11790 | PDI_H | -0.003678608 | 0.000901564 | -4.080252294 | 4.53e-05 |
| tnfrsf13b | 13677 | PDI_H | -0.00362251 | 0.000890643 | -4.0672979 | 4.78e-05 |
| wfikkn1 | 13510 | PDI_H | 0.003517858 | 0.000864951 | 4.067120587 | 4.79e-05 |
| smpd1 | 13727 | PDI_H | -0.003623483 | 0.000896664 | -4.041071017 | 5.35e-05 |
| nhlrc3 | 11262 | PDI_H | -0.003808341 | 0.000943379 | -4.036916407 | 5.45e-05 |
| capg | 13523 | PDI_H | -0.003613304 | 0.000895774 | -4.033723566 | 5.52e-05 |
| septin8 | 11755 | PDI_H | -0.003774763 | 0.0009394 | -4.018268377 | 5.9e-05 |
| mad1l1 | 13521 | PDI_H | -0.003464321 | 0.000864213 | -4.008643459 | 6.14e-05 |
| erbb4 | 13699 | PDI_H | 0.003534389 | 0.000882073 | 4.006913693 | 6.18e-05 |
| cstb | 13461 | PDI_H | -0.003496027 | 0.000876081 | -3.99052961 | 6.63e-05 |
| sbsn | 11765 | PDI_H | -0.003936228 | 0.000986532 | -3.989965618 | 6.65e-05 |
| msr1 | 13784 | PDI_H | -0.003326392 | 0.000834944 | -3.983971382 | 6.81e-05 |
| acp5 | 13685 | PDI_H | -0.003477508 | 0.000879935 | -3.952006136 | 7.79e-05 |
| rbp5 | 13579 | PDI_H | -0.00324578 | 0.000824655 | -3.935924551 | 8.33e-05 |
| gm2a | 11503 | PDI_H | -0.003677786 | 0.000938395 | -3.919231488 | 8.93e-05 |
| tafa5 | 13579 | PDI_H | -0.003497406 | 0.00089331 | -3.915107694 | 9.08e-05 |
| scarb2 | 13292 | PDI_H | -0.003237679 | 0.000831378 | -3.894352133 | 9.89e-05 |
| lbp | 13689 | PDI_H | -0.003433147 | 0.000890962 | -3.8533035 | 0.000117067 |
| cxcl9 | 13710 | PDI_H | -0.003279132 | 0.000855415 | -3.83337946 | 0.00012696 |
| p4hb | 13594 | PDI_H | -0.003460582 | 0.000903925 | -3.828396606 | 0.00012956 |
| siglec1 | 13710 | PDI_H | -0.003325974 | 0.000872037 | -3.814028031 | 0.00013732 |
| ptx3 | 13621 | PDI_H | -0.003491852 | 0.000917417 | -3.806176907 | 0.00014175 |
| umod | 13750 | PDI_H | 0.003262983 | 0.000862526 | 3.783055628 | 0.000155572 |
| golm2 | 13784 | PDI_H | -0.00326732 | 0.000865163 | -3.776537343 | 0.000159692 |
| acvrl1 | 13526 | PDI_H | -0.003193825 | 0.000846595 | -3.772553138 | 0.000162275 |
| igsf9 | 11781 | PDI_H | -0.003221463 | 0.000856968 | -3.759139232 | 0.000171324 |
| tnfrsf9 | 13552 | PDI_H | -0.003287497 | 0.000875381 | -3.755503845 | 0.000173718 |
| slamf8 | 13308 | PDI_H | -0.003409898 | 0.000912966 | -3.734966957 | 0.000188524 |
| rgma | 13784 | PDI_H | 0.003336433 | 0.00089389 | 3.732487566 | 0.000190361 |
| fap | 13800 | PDI_H | 0.003333036 | 0.000895426 | 3.72228992 | 0.000198209 |
| pdcd5 | 13784 | PDI_H | -0.00334688 | 0.000902204 | -3.709669409 | 0.000208346 |
| hspg2 | 13531 | PDI_H | -0.003063886 | 0.000828107 | -3.699865742 | 0.000216569 |
| hmox2 | 13320 | PDI_H | -0.003395292 | 0.000918817 | -3.695285539 | 0.00022052 |
| cdhr2 | 13579 | PDI_H | -0.002633274 | 0.000712767 | -3.6944417 | 0.000221236 |
| grn | 13559 | PDI_H | -0.003323837 | 0.000901328 | -3.687709216 | 0.000227166 |
| egln1 | 13621 | PDI_H | -0.003242471 | 0.000879325 | -3.687452976 | 0.000227391 |
| adamts13 | 13800 | PDI_H | 0.003321355 | 0.000904151 | 3.673451697 | 0.000240202 |
| cytl1 | 11790 | PDI_H | -0.003440775 | 0.000936739 | -3.673142227 | 0.000240647 |
| cdcp1 | 13552 | PDI_H | -0.002958792 | 0.000805887 | -3.671470087 | 0.000242087 |
| pla2g15 | 13699 | PDI_H | -0.003250258 | 0.000886858 | -3.664914375 | 0.000248356 |
| parp1 | 13621 | PDI_H | -0.003256301 | 0.000891334 | -3.653287766 | 0.000259876 |
| cxcl10 | 13710 | PDI_H | -0.003154773 | 0.000867096 | -3.638319753 | 0.000275431 |
| ccdc80 | 13618 | PDI_H | -0.002969072 | 0.000819252 | -3.624125645 | 0.000290998 |
| lair1 | 13658 | PDI_H | -0.003007652 | 0.000831414 | -3.617516253 | 0.000298517 |
| klk7 | 11253 | PDI_H | -0.003569255 | 0.000991338 | -3.600444117 | 0.000319042 |
| tnf | 13369 | PDI_H | -0.003223484 | 0.000898328 | -3.588316556 | 0.000334011 |
| a1bg | 11817 | PDI_H | -0.003304398 | 0.000921452 | -3.586078258 | 0.000337044 |
| dbh | 11763 | PDI_H | 0.003485189 | 0.000982524 | 3.547178264 | 0.000390898 |
| fabp3 | 11781 | PDI_H | -0.00302192 | 0.000852324 | -3.545506319 | 0.000393381 |
| prss53 | 11767 | PDI_H | -0.003514852 | 0.000991702 | -3.544261717 | 0.000395243 |
| cd300e | 13706 | PDI_H | -0.002982376 | 0.000842601 | -3.539488849 | 0.000402232 |
| itgam | 13632 | PDI_H | 0.003175111 | 0.000897184 | 3.538973845 | 0.000403024 |
| cdsn | 13760 | PDI_H | -0.003207093 | 0.000906874 | -3.536427042 | 0.000406914 |
| fabp1 | 13658 | PDI_H | -0.003020284 | 0.000855049 | -3.532294124 | 0.000413331 |
| trem2 | 13658 | PDI_H | -0.002972059 | 0.000841904 | -3.530162275 | 0.000416673 |
| nppc | 13621 | PDI_H | -0.002785301 | 0.000793167 | -3.511621749 | 0.000446824 |
| acp6 | 13732 | PDI_H | -0.003205787 | 0.00091418 | -3.506735033 | 0.00045509 |
| iglc2 | 11803 | PDI_H | -0.003387742 | 0.000967686 | -3.500867885 | 0.00046546 |
| fgfr2 | 13699 | PDI_H | -0.002942249 | 0.000841292 | -3.49729688 | 0.000471489 |
| rnase6 | 11698 | PDI_H | -0.003148614 | 0.00090087 | -3.495080196 | 0.000475676 |
| zbtb17 | 13388 | PDI_H | -0.003166735 | 0.000907304 | -3.490267614 | 0.000484091 |
| sorcs2 | 13308 | PDI_H | -0.003064455 | 0.000883076 | -3.470204107 | 0.000521712 |
| pinlyp | 11537 | PDI_H | -0.003456346 | 0.000997158 | -3.466198123 | 0.000529798 |
| palm2 | 11767 | PDI_H | -0.002990563 | 0.00086542 | -3.455620752 | 0.000550966 |

| Protein | N_used | term | beta | se | t | p_val |
|---|---|---|---|---|---|---|
| enpp7 | 13229 | PDI_H | -0.003135526 | 0.000911372 | -3.440445991 | 0.000582551 |
| fam3c | 13599 | PDI_H | -0.003007786 | 0.000876299 | -3.432375212 | 0.000600102 |
| myom3 | 11537 | PDI_H | 0.003210846 | 0.000937039 | 3.426589666 | 0.000613346 |
| sumf2 | 13784 | PDI_H | -0.003082143 | 0.000900096 | -3.424237231 | 0.000618324 |
| ccl7 | 13621 | PDI_H | -0.002960767 | 0.000867297 | -3.413786865 | 0.000642534 |
| apcs | 11694 | PDI_H | -0.002887319 | 0.000846038 | -3.412754908 | 0.000645279 |
| scly | 13715 | PDI_H | -0.002884243 | 0.000850231 | -3.392304075 | 0.000695035 |
| yap1 | 11767 | PDI_H | -0.002874044 | 0.00084727 | -3.392124449 | 0.000695815 |
| cgref1 | 13618 | PDI_H | -0.003066508 | 0.000904493 | -3.390305405 | 0.000700132 |
| col28a1 | 11483 | PDI_H | 0.003389871 | 0.000999891 | 3.390239535 | 0.00070067 |
| il6 | 13579 | PDI_H | -0.002807178 | 0.00083013 | -3.381612821 | 0.000722651 |
| cd74 | 13784 | PDI_H | -0.002867823 | 0.000848486 | -3.379929943 | 0.000727056 |
| srp14 | 13579 | PDI_H | -0.003005609 | 0.000890027 | -3.37698685 | 0.000734904 |
| cbln4 | 13753 | PDI_H | 0.003072822 | 0.000911192 | 3.372310503 | 0.00074746 |
| adam8 | 13767 | PDI_H | -0.003014897 | 0.000894096 | -3.37200639 | 0.000748283 |
| il1rn | 13629 | PDI_H | -0.002658597 | 0.00079112 | -3.360550572 | 0.000780011 |
| coch | 11781 | PDI_H | -0.003236273 | 0.000963235 | -3.359795403 | 0.00078248 |
| adgrg2 | 13618 | PDI_H | 0.00271976 | 0.000811736 | 3.3505466 | 0.000808716 |
| rnase1 | 11794 | PDI_H | -0.002991234 | 0.000893236 | -3.348761258 | 0.000814282 |
| zhx2 | 11767 | PDI_H | -0.003236238 | 0.000969343 | -3.338587438 | 0.000844672 |
| krt18 | 13579 | PDI_H | -0.002744745 | 0.000824991 | -3.32699747 | 0.000880205 |
| tgfa | 13621 | PDI_H | -0.002949162 | 0.000887435 | -3.323244042 | 0.000892118 |
| tff2 | 13691 | PDI_H | -0.002864498 | 0.000863413 | -3.317645241 | 0.000910165 |
| kctd5 | 11526 | PDI_H | -0.003198082 | 0.000964357 | -3.316283704 | 0.000915055 |
| nectin2 | 13599 | PDI_H | -0.002972215 | 0.000897131 | -3.31302346 | 0.000925343 |
| mmp8 | 13767 | PDI_H | -0.002963965 | 0.000897154 | -3.303740931 | 0.000956476 |
| fam20a | 11781 | PDI_H | -0.002977707 | 0.000901531 | -3.30294595 | 0.000959602 |
| cd27 | 13699 | PDI_H | -0.00293333 | 0.000888316 | -3.302124275 | 0.000962014 |
| mzb1 | 13696 | PDI_H | -0.002918828 | 0.000887204 | -3.289916533 | 0.001004704 |
| mmp12 | 13646 | PDI_H | -0.002746033 | 0.000838821 | -3.273683257 | 0.001064199 |
| ccl14 | 13590 | PDI_H | -0.002945056 | 0.000902341 | -3.263794389 | 0.001102028 |
| pvr | 13784 | PDI_H | -0.002964395 | 0.00090883 | -3.261770698 | 0.00110988 |
| ccl20 | 13801 | PDI_H | -0.002881593 | 0.000884444 | -3.258083866 | 0.001124386 |
| clec3b | 11525 | PDI_H | 0.003200146 | 0.00098914 | 3.235280954 | 0.001218663 |
| rcor1 | 13388 | PDI_H | -0.00297896 | 0.000922279 | -3.229997049 | 0.001240905 |
| ang | 13590 | PDI_H | -0.002792104 | 0.000865064 | -3.227627197 | 0.001251178 |
| igfbp3 | 13689 | PDI_H | 0.002845623 | 0.000883293 | 3.221607509 | 0.001277719 |
| asah2 | 13552 | PDI_H | 0.002839985 | 0.00088423 | 3.211817119 | 0.001322066 |
| cd274 | 13552 | PDI_H | -0.002886864 | 0.000899297 | -3.210135042 | 0.001329821 |
| cwc15 | 11662 | PDI_H | -0.0031356 | 0.000977038 | -3.209292886 | 0.001334223 |
| tnfrsf12a | 13699 | PDI_H | -0.002689651 | 0.000838709 | -3.206892839 | 0.001344855 |
| pcsk9 | 13800 | PDI_H | -0.002808502 | 0.000877434 | -3.200811807 | 0.001373516 |
| vegfb | 11411 | PDI_H | -0.003066011 | 0.000959497 | -3.195436832 | 0.001399998 |
| rspo1 | 13552 | PDI_H | -0.00287562 | 0.000902921 | -3.184798503 | 0.00145183 |
| eloa | 13579 | PDI_H | -0.002819773 | 0.000887945 | -3.175615521 | 0.001498535 |
| plat | 13750 | PDI_H | -0.002511559 | 0.000790942 | -3.175401535 | 0.001499598 |
| c9 | 11794 | PDI_H | -0.003084737 | 0.00097309 | -3.170044613 | 0.001528057 |
| klk8 | 13201 | PDI_H | -0.002827477 | 0.000892982 | -3.166331031 | 0.001547265 |
| tmprss11d | 11755 | PDI_H | -0.003050443 | 0.000963968 | -3.164463388 | 0.001557657 |
| cxcl11 | 13784 | PDI_H | -0.002855185 | 0.000903038 | -3.161754075 | 0.00157162 |
| il22ra1 | 13378 | PDI_H | -0.00292341 | 0.000924693 | -3.16149142 | 0.001573141 |
| npc2 | 11595 | PDI_H | -0.002830052 | 0.000900119 | -3.144087013 | 0.001670268 |
| ccl22 | 13535 | PDI_H | -0.002767721 | 0.000882223 | -3.137213372 | 0.00170928 |
| il10rb | 13710 | PDI_H | -0.002709076 | 0.000866989 | -3.124692707 | 0.001783625 |
| tnfaip2 | 11724 | PDI_H | -0.003079011 | 0.000985624 | -3.123920233 | 0.001788942 |
| prss2 | 13689 | PDI_H | -0.002849205 | 0.00091253 | -3.122312936 | 0.001798102 |
| pon3 | 13710 | PDI_H | 0.00249147 | 0.000799343 | 3.116898292 | 0.001831427 |
| smpdl3a | 13695 | PDI_H | -0.002834022 | 0.000909643 | -3.115533639 | 0.001839921 |
| fabp5 | 13739 | PDI_H | -0.002789316 | 0.000895971 | -3.113175903 | 0.001854661 |
| thbs2 | 13632 | PDI_H | -0.002795379 | 0.000898852 | -3.109942935 | 0.001875096 |
| rrm2 | 13308 | PDI_H | -0.002854346 | 0.000919512 | -3.104197166 | 0.001911967 |
| celsr2 | 11537 | PDI_H | -0.003056289 | 0.00098495 | -3.102990304 | 0.001920393 |
| il4r | 13621 | PDI_H | -0.002805772 | 0.000904567 | -3.101786232 | 0.001927503 |
| reg3a | 13800 | PDI_H | -0.002735881 | 0.000882163 | -3.101333927 | 0.001930396 |
| ppy | 13617 | PDI_H | 0.002772 | 0.000895266 | 3.096286706 | 0.001963591 |
| ifnlr1 | 13621 | PDI_H | -0.002735476 | 0.000883998 | -3.094436723 | 0.001975868 |
| mrc1 | 11794 | PDI_H | -0.002831444 | 0.000919367 | -3.079776552 | 0.002076328 |
| apoa4 | 11817 | PDI_H | 0.003005216 | 0.000976918 | 3.076222521 | 0.002101209 |
| penk | 11537 | PDI_H | -0.003017386 | 0.000980876 | -3.076213909 | 0.002101386 |
| bax | 13301 | PDI_H | -0.002867277 | 0.000933235 | -3.072404546 | 0.002127713 |

| Protein | N_used | term | beta | se | t | p_val |
|---|---|---|---|---|---|---|
| sfrp1 | 13504 | PDI_H | -0.002622904 | 0.000853752 | -3.072208951 | 0.002129042 |
| serpina11 | 13800 | PDI_H | -0.002512275 | 0.000819288 | -3.066411531 | 0.002170643 |
| st6gal1 | 13800 | PDI_H | -0.002676849 | 0.000877557 | -3.050340995 | 0.002290157 |
| csf1 | 13710 | PDI_H | -0.00264312 | 0.000868353 | -3.043831892 | 0.002340286 |
| plaur | 13557 | PDI_H | -0.002596184 | 0.000853321 | -3.042448579 | 0.002351112 |
| prap1 | 11790 | PDI_H | -0.002539077 | 0.000834605 | -3.042250614 | 0.002353333 |
| anxa2 | 11291 | PDI_H | -0.003001126 | 0.000986671 | -3.041668485 | 0.002358114 |
| ntrk3 | 13784 | PDI_H | 0.002574283 | 0.000847793 | 3.03645152 | 0.002398281 |
| ephb6 | 13535 | PDI_H | -0.002774807 | 0.00091386 | -3.036359611 | 0.002399094 |
| psap | 11683 | PDI_H | -0.002875333 | 0.000949147 | -3.029387175 | 0.002455865 |
| rab39b | 11355 | PDI_H | -0.003059587 | 0.001010896 | -3.026609674 | 0.002478679 |
| icam1 | 13565 | PDI_H | -0.002665529 | 0.000880752 | -3.026423502 | 0.002479299 |
| enpp6 | 11474 | PDI_H | 0.002791598 | 0.000922391 | 3.026478277 | 0.002479698 |
| furin | 13699 | PDI_H | -0.002462017 | 0.000814257 | -3.023636334 | 0.002502191 |
| ddt | 11790 | PDI_H | -0.002879234 | 0.000952393 | -3.023156195 | 0.002506913 |
| gbp1 | 11749 | PDI_H | -0.002931968 | 0.000972621 | -3.014501339 | 0.00257952 |
| spink4 | 13621 | PDI_H | -0.002701719 | 0.000897956 | -3.008745082 | 0.002628091 |
| hao1 | 13753 | PDI_H | -0.002608309 | 0.000867909 | -3.005278531 | 0.002658165 |
| ltbp3 | 13308 | PDI_H | -0.002649919 | 0.00088237 | -3.003182283 | 0.002676693 |
| fgr | 13320 | PDI_H | -0.002754813 | 0.000918034 | -3.000772676 | 0.002697945 |
| marco | 13618 | PDI_H | -0.002663138 | 0.000888099 | -2.99869477 | 0.002716289 |
| sema3f | 13618 | PDI_H | -0.002536597 | 0.000848425 | -2.98977087 | 0.00279686 |
| pon2 | 13388 | PDI_H | -0.002726546 | 0.000912485 | -2.988045664 | 0.002812772 |
| spint1 | 13575 | PDI_H | -0.002726882 | 0.000915043 | -2.980058405 | 0.002887043 |
| vwc2 | 13509 | PDI_H | -0.002595029 | 0.000871794 | -2.976653395 | 0.002919302 |
| fstl1 | 11474 | PDI_H | -0.002950367 | 0.000991844 | -2.97462909 | 0.002939543 |
| rbp2 | 13579 | PDI_H | -0.002697196 | 0.000907134 | -2.973315705 | 0.00295119 |
| csf3 | 13378 | PDI_H | -0.00266928 | 0.000899261 | -2.968303054 | 0.002999799 |
| maged1 | 13308 | PDI_H | -0.002767516 | 0.000932384 | -2.968216194 | 0.003000674 |
| hsdl2 | 11474 | PDI_H | -0.002927977 | 0.000986873 | -2.96692324 | 0.00301417 |
| snrpb2 | 11767 | PDI_H | -0.002832621 | 0.000958526 | -2.955185731 | 0.003131008 |
| clmp | 13753 | PDI_H | -0.002269379 | 0.000770038 | -2.947100984 | 0.003213074 |
| psip1 | 13621 | PDI_H | -0.002654745 | 0.000900927 | -2.94668353 | 0.003217464 |
| pilra | 13632 | PDI_H | -0.002600312 | 0.00088285 | -2.945361588 | 0.003231231 |
| itih3 | 13689 | PDI_H | -0.002529527 | 0.00085898 | -2.944804601 | 0.003237026 |
| il18r1 | 13801 | PDI_H | -0.002508726 | 0.000854101 | -2.937272493 | 0.003316607 |
| adamtsl2 | 11790 | PDI_H | -0.002704747 | 0.000921687 | -2.934559805 | 0.003346659 |
| ccl25 | 13801 | PDI_H | -0.002615948 | 0.000892698 | -2.93038329 | 0.003390994 |
| tnfrsf14 | 13677 | PDI_H | -0.002576436 | 0.000879913 | -2.928057602 | 0.003416497 |
| f9 | 13669 | PDI_H | -0.002441302 | 0.000834094 | -2.926891857 | 0.003429324 |
| omg | 13579 | PDI_H | 0.002646641 | 0.000905205 | 2.923803323 | 0.003463548 |
| lmnb2 | 11767 | PDI_H | -0.002694906 | 0.000921691 | -2.923870404 | 0.003463684 |
| hyou1 | 13800 | PDI_H | -0.002627962 | 0.00090185 | -2.913968399 | 0.003574395 |
| cxcl8 | 13539 | PDI_H | -0.002644388 | 0.000911252 | -2.90192991 | 0.003714699 |
| chchd10 | 11767 | PDI_H | -0.002519225 | 0.000868166 | -2.901779212 | 0.003717388 |
| fcer2 | 13632 | PDI_H | -0.002568092 | 0.000888212 | -2.891305858 | 0.003842512 |
| ambp | 13736 | PDI_H | -0.002430004 | 0.000841484 | -2.887758639 | 0.003886037 |
| mst1 | 11593 | PDI_H | -0.002867733 | 0.000993398 | -2.886792436 | 0.003899106 |
| mdk | 13651 | PDI_H | -0.002615629 | 0.000907034 | -2.883717754 | 0.003936256 |
| havcr2 | 13632 | PDI_H | -0.00247379 | 0.000858908 | -2.880156053 | 0.003980982 |
| glrx | 13524 | PDI_H | -0.002635637 | 0.000915128 | -2.8800739 | 0.003982068 |
| dscam | 11757 | PDI_H | 0.002826061 | 0.00098192 | 2.878098254 | 0.004008021 |
| cfhr4 | 11791 | PDI_H | 0.002843507 | 0.000989505 | 2.873666883 | 0.004064623 |
| hmcn2 | 11516 | PDI_H | 0.002849007 | 0.000992666 | 2.870056652 | 0.004111464 |
| bpifb2 | 11754 | PDI_H | -0.002566805 | 0.000894725 | -2.868819541 | 0.004127415 |
| serpina1 | 11561 | PDI_H | -0.002861842 | 0.000999234 | -2.864036414 | 0.004190337 |
| efcab14 | 11724 | PDI_H | -0.002720593 | 0.000950386 | -2.862619748 | 0.004208997 |
| dpp6 | 13579 | PDI_H | 0.002475266 | 0.000866882 | 2.855367442 | 0.004305228 |
| immt | 11730 | PDI_H | -0.002702225 | 0.00094661 | -2.854633388 | 0.004316215 |
| rbfox3 | 11757 | PDI_H | -0.002471525 | 0.000870791 | -2.838251712 | 0.004543918 |
| tmsb10 | 13632 | PDI_H | -0.002551766 | 0.000899698 | -2.836247011 | 0.004571445 |
| megf10 | 13658 | PDI_H | 0.002577711 | 0.000909994 | 2.832668775 | 0.004622883 |
| clec6a | 13308 | PDI_H | -0.002584113 | 0.000912476 | -2.831980632 | 0.004633017 |
| reg4 | 13677 | PDI_H | -0.002464245 | 0.000871635 | -2.827152717 | 0.004703217 |
| mstn | 13388 | PDI_H | 0.002358807 | 0.000835648 | 2.822729013 | 0.004768709 |
| lamp3 | 13779 | PDI_H | -0.002384339 | 0.000845461 | -2.820165544 | 0.004806749 |
| ccl27 | 13618 | PDI_H | -0.002479149 | 0.000879237 | -2.819658051 | 0.004814434 |
| lgals4 | 13658 | PDI_H | -0.002457577 | 0.000873863 | -2.812314197 | 0.004925665 |
| plg | 11488 | PDI_H | -0.002754988 | 0.000980719 | -2.809151864 | 0.00497562 |
| pebp1 | 13632 | PDI_H | -0.002544589 | 0.000906113 | -2.80824494 | 0.00498832 |

| Protein | N_used | term | beta | se | t | p_val |
|---|---|---|---|---|---|---|
| ceacam19 | 11724 | PDI_H | -0.002717506 | 0.000969459 | -2.8031146 | 0.005069489 |
| dsg3 | 13697 | PDI_H | -0.00241854 | 0.000866265 | -2.791918138 | 0.005246927 |
| ggt1 | 13602 | PDI_H | -0.002268372 | 0.000812993 | -2.790151022 | 0.005275686 |
| boc | 13388 | PDI_H | 0.002520284 | 0.000903547 | 2.789322968 | 0.005289305 |
| itih4 | 11817 | PDI_H | -0.002581184 | 0.000927971 | -2.781536862 | 0.0054188 |
| mpo | 13632 | PDI_H | -0.002475468 | 0.000894837 | -2.766390215 | 0.005675707 |
| kir2dl2 | 11730 | PDI_H | -0.002722494 | 0.000984361 | -2.765747523 | 0.00568814 |
| dbi | 13566 | PDI_H | -0.002527671 | 0.000914118 | -2.765148793 | 0.005697378 |
| il19 | 13290 | PDI_H | -0.002507326 | 0.00090693 | -2.764630365 | 0.005706595 |
| nmi | 11681 | PDI_H | -0.002748406 | 0.000994516 | -2.763560628 | 0.00572641 |
| spink2 | 11537 | PDI_H | -0.002488917 | 0.000901077 | -2.762156197 | 0.005751197 |
| cfd | 11817 | PDI_H | -0.002490555 | 0.000902278 | -2.760295249 | 0.005783825 |
| lgals7_lgals7b | 13602 | PDI_H | -0.002412501 | 0.000874527 | -2.758636104 | 0.005812075 |
| creld2 | 13691 | PDI_H | -0.002500918 | 0.000907038 | -2.757237014 | 0.005836939 |
| ssc4d | 13599 | PDI_H | 0.002165928 | 0.000785817 | 2.756276903 | 0.005854144 |
| ddah1 | 13579 | PDI_H | -0.002482331 | 0.000901324 | -2.754094715 | 0.005893311 |
| lbr | 13784 | PDI_H | -0.002465787 | 0.000895486 | -2.753572654 | 0.005902596 |
| fkbp4 | 13552 | PDI_H | -0.002521624 | 0.0009176 | -2.748062641 | 0.006002792 |
| sct | 11483 | PDI_H | 0.002738304 | 0.000997668 | 2.744704058 | 0.006065972 |
| tnfrsf13c | 13378 | PDI_H | -0.002514843 | 0.000917023 | -2.742398471 | 0.006107349 |
| serpina9 | 13523 | PDI_H | -0.002494364 | 0.00090983 | -2.741571944 | 0.00612264 |
| wfdc12 | 13735 | PDI_H | -0.002254158 | 0.000822701 | -2.739947411 | 0.006152843 |
| crisp3 | 11679 | PDI_H | 0.002688941 | 0.0009822 | 2.737671864 | 0.006196956 |
| cd209 | 13680 | PDI_H | 0.002498881 | 0.000914767 | 2.73171429 | 0.006308663 |
| ulbp2 | 13552 | PDI_H | -0.002429705 | 0.000891335 | -2.725917452 | 0.006420549 |
| nme3 | 13677 | PDI_H | -0.002486849 | 0.000913575 | -2.722106771 | 0.006494942 |
| sele | 13800 | PDI_H | -0.00232724 | 0.000856345 | -2.717643565 | 0.006583075 |
| klk12 | 13308 | PDI_H | -0.002522238 | 0.000929466 | -2.713643464 | 0.006663349 |
| lep | 13618 | PDI_H | -0.001398933 | 0.000516372 | -2.709157351 | 0.006753877 |
| sdc1 | 13618 | PDI_H | -0.002408418 | 0.000889377 | -2.707982939 | 0.00677781 |
| caps | 11767 | PDI_H | -0.002561217 | 0.000945849 | -2.70785059 | 0.006781845 |
| vasn | 13420 | PDI_H | 0.002472011 | 0.000914481 | 2.703183893 | 0.006876528 |
| hgf | 13801 | PDI_H | -0.002174762 | 0.000805272 | -2.700654407 | 0.006928772 |
| slurp1 | 11699 | PDI_H | -0.002618832 | 0.00096976 | -2.700493303 | 0.006933647 |
| osmr | 13712 | PDI_H | -0.002368637 | 0.000878035 | -2.697656669 | 0.00699149 |
| sult2a1 | 13621 | PDI_H | -0.002368373 | 0.000881251 | -2.687513387 | 0.007207374 |
| mif | 13605 | PDI_H | -0.002466365 | 0.000918315 | -2.685750456 | 0.0072455 |
| cfi | 11817 | PDI_H | -0.002354115 | 0.000878369 | -2.680097465 | 0.007370288 |
| syt1 | 11767 | PDI_H | -0.002606928 | 0.000972855 | -2.679668015 | 0.007379787 |
| acyp1 | 11487 | PDI_H | -0.002675182 | 0.000998385 | -2.679508815 | 0.007383546 |
| eif4ebp1 | 13618 | PDI_H | -0.002427406 | 0.000906259 | -2.678488536 | 0.007404413 |
| psapl1 | 11724 | PDI_H | -0.002613844 | 0.000976212 | -2.677537471 | 0.007426896 |
| gla | 11517 | PDI_H | -0.002510723 | 0.000938094 | -2.676408677 | 0.00745213 |
| areg | 13579 | PDI_H | -0.002443739 | 0.000913785 | -2.674303926 | 0.007497429 |
| lhpp | 13678 | PDI_H | -0.002417614 | 0.000904279 | -2.673525865 | 0.007514769 |
| sel1l | 11474 | PDI_H | -0.002601735 | 0.000974286 | -2.670400835 | 0.007586765 |
| ecm1 | 11475 | PDI_H | 0.002673641 | 0.001003925 | 2.663188825 | 0.007751213 |
| spink6 | 13753 | PDI_H | -0.002311559 | 0.000868555 | -2.661383875 | 0.007791067 |
| inhbb | 11537 | PDI_H | -0.002505494 | 0.000943738 | -2.654861911 | 0.007944992 |
| cdnf | 13579 | PDI_H | -0.002382056 | 0.000897278 | -2.654759234 | 0.007945767 |
| dpy30 | 13705 | PDI_H | -0.002342083 | 0.000882529 | -2.653831985 | 0.007967537 |
| il18 | 13632 | PDI_H | -0.002342855 | 0.000883037 | -2.653179989 | 0.007982986 |
| dtx3 | 13579 | PDI_H | -0.002227425 | 0.000841017 | -2.64849012 | 0.00809458 |
| rassf2 | 13308 | PDI_H | -0.002408669 | 0.000911751 | -2.641805082 | 0.008256202 |
| triap1 | 13753 | PDI_H | -0.002405439 | 0.000910959 | -2.640557147 | 0.008286341 |
| il2 | 13378 | PDI_H | -0.002460236 | 0.000932138 | -2.639348674 | 0.008316189 |
| colec12 | 13658 | PDI_H | -0.002097477 | 0.000796424 | -2.633618593 | 0.008457561 |
| ssh3 | 11304 | PDI_H | -0.002663547 | 0.001012492 | -2.630684675 | 0.008532875 |
| il15 | 13621 | PDI_H | -0.002407521 | 0.000915769 | -2.628959946 | 0.00857427 |
| plxdc1 | 13308 | PDI_H | 0.002428838 | 0.00092601 | 2.622907148 | 0.008728249 |
| ppcdc | 13784 | PDI_H | -0.002385163 | 0.000909581 | -2.622265071 | 0.008744359 |
| trim26 | 11304 | PDI_H | -0.002661562 | 0.001015031 | -2.622148252 | 0.008749467 |
| rarres2 | 13712 | PDI_H | -0.002163795 | 0.000826673 | -2.617474537 | 0.008868045 |
| angptl4 | 13691 | PDI_H | -0.002295872 | 0.000878229 | -2.614205141 | 0.008953333 |
| serpinf1 | 11620 | PDI_H | -0.002250755 | 0.000861579 | -2.612362383 | 0.009003471 |
| fhip2a | 11355 | PDI_H | -0.002635487 | 0.001012127 | -2.603909709 | 0.009228714 |
| fcrl6 | 13621 | PDI_H | -0.002375622 | 0.000912366 | -2.603803262 | 0.009229578 |
| mnda | 13593 | PDI_H | -0.002376938 | 0.000914719 | -2.598545714 | 0.009372072 |
| gast | 11767 | PDI_H | -0.002486491 | 0.000957646 | -2.596460769 | 0.00943069 |
| cd48 | 13691 | PDI_H | -0.002350124 | 0.000906565 | -2.59233993 | 0.009542691 |

| Protein | N_used | term | beta | se | t | p_val |
|---|---|---|---|---|---|---|
| met | 13688 | PDI_H | 0.002246739 | 0.000874376 | 2.569535198 | 0.010194003 |
| thop1 | 13557 | PDI_H | -0.002261293 | 0.000880083 | -2.569409355 | 0.010197805 |
| pilrb | 13618 | PDI_H | -0.002302191 | 0.000896737 | -2.567297103 | 0.010260063 |
| ccl15 | 13800 | PDI_H | -0.002271856 | 0.000887278 | -2.560479019 | 0.010463352 |
| acaa1 | 13308 | PDI_H | -0.002332301 | 0.000911353 | -2.559164172 | 0.010503385 |
| il17c | 13378 | PDI_H | -0.002325116 | 0.000909942 | -2.555235953 | 0.010622557 |
| tigar | 13303 | PDI_H | -0.002377559 | 0.000931028 | -2.55369298 | 0.01066978 |
| gapdh | 11271 | PDI_H | -0.002568689 | 0.001006379 | -2.552407128 | 0.010711225 |
| aarsd1 | 13579 | PDI_H | -0.002306505 | 0.000906578 | -2.544187761 | 0.010964208 |
| spink5 | 13632 | PDI_H | -0.00230087 | 0.000904672 | -2.543318498 | 0.010991469 |
| pltp | 13760 | PDI_H | 0.00218533 | 0.000860104 | 2.540775514 | 0.01107159 |
| slc39a5 | 13621 | PDI_H | -0.002167291 | 0.000853652 | -2.53884584 | 0.011132923 |
| ckap4 | 13729 | PDI_H | -0.0022326 | 0.000881751 | -2.53200671 | 0.011352246 |
| tnn | 11790 | PDI_H | 0.002488563 | 0.000983143 | 2.531231184 | 0.011379191 |
| sod1 | 13384 | PDI_H | -0.002316223 | 0.000916083 | -2.528399363 | 0.011469802 |
| nucb2 | 13730 | PDI_H | -0.002268158 | 0.000899378 | -2.521918654 | 0.011682895 |
| taldo1 | 11742 | PDI_H | -0.002441566 | 0.000968423 | -2.521177053 | 0.011709441 |
| atg4a | 13308 | PDI_H | -0.002327455 | 0.000923245 | -2.520950786 | 0.01171542 |
| rilp | 13715 | PDI_H | -0.00230397 | 0.000914585 | -2.519142222 | 0.011775396 |
| fgfr4 | 11537 | PDI_H | 0.00251587 | 0.000999373 | 2.517448116 | 0.011834286 |
| dnph1 | 13695 | PDI_H | -0.002276341 | 0.000909024 | -2.504160291 | 0.012285785 |
| ephb4 | 13800 | PDI_H | -0.002247771 | 0.000897936 | -2.503263241 | 0.012316868 |
| cdh15 | 13767 | PDI_H | 0.001910344 | 0.000763466 | 2.502198449 | 0.012353987 |
| pnma2 | 11383 | PDI_H | 0.002522488 | 0.001010673 | 2.49585028 | 0.012579617 |
| pecr | 11474 | PDI_H | -0.002506258 | 0.001005988 | -2.491340079 | 0.012740256 |
| mmp3 | 13632 | PDI_H | -0.001761129 | 0.000707275 | -2.490021322 | 0.012785373 |
| nrtn | 13378 | PDI_H | -0.002313846 | 0.000929278 | -2.489940244 | 0.012788513 |
| cxcl16 | 13773 | PDI_H | -0.002177518 | 0.00087526 | -2.487853608 | 0.012863406 |
| raly | 11730 | PDI_H | -0.002455475 | 0.000987588 | -2.48633448 | 0.01292048 |
| pik3ip1 | 13632 | PDI_H | -0.00211282 | 0.000850236 | -2.484979702 | 0.012967795 |
| ccl18 | 13454 | PDI_H | -0.002169163 | 0.000873199 | -2.484157166 | 0.012997933 |
| ccl21 | 13710 | PDI_H | -0.002220816 | 0.000894357 | -2.483141334 | 0.013034816 |
| asgr2 | 11794 | PDI_H | -0.002418638 | 0.000975258 | -2.479997911 | 0.013152182 |
| dpp4 | 13359 | PDI_H | 0.00228524 | 0.000922091 | 2.478322421 | 0.013212449 |
| ifi30 | 11446 | PDI_H | -0.002261781 | 0.000913108 | -2.477013372 | 0.013263042 |
| ilkap | 13304 | PDI_H | -0.002308628 | 0.000932272 | -2.476346083 | 0.013285838 |
| klk10 | 13652 | PDI_H | -0.002267285 | 0.000916881 | -2.472824134 | 0.013417106 |
| psme2 | 13499 | PDI_H | -0.002233536 | 0.0009035 | -2.472092266 | 0.013444731 |
| siglec5 | 13632 | PDI_H | -0.002236465 | 0.00090847 | -2.461791054 | 0.013836816 |
| hsd11b1 | 13621 | PDI_H | -0.002152729 | 0.000875318 | -2.459368668 | 0.01393051 |
| vat1 | 13579 | PDI_H | 0.002236665 | 0.000911837 | 2.45292103 | 0.01418264 |
| hnrnpul1 | 11757 | PDI_H | -0.002366352 | 0.000965322 | -2.451359484 | 0.014246239 |
| glrx5 | 11537 | PDI_H | -0.002454141 | 0.001002497 | -2.448028568 | 0.014378833 |
| wfdc2 | 12993 | PDI_H | -0.002032447 | 0.000831017 | -2.445735019 | 0.014468906 |
| glt8d2 | 13320 | PDI_H | -0.002282613 | 0.000934385 | -2.442904795 | 0.014582491 |
| gfap | 13293 | PDI_H | -0.002088401 | 0.000855865 | -2.440104592 | 0.014695995 |
| apol1 | 11794 | PDI_H | -0.002353438 | 0.000964649 | -2.439683148 | 0.014714795 |
| agxt | 13618 | PDI_H | -0.002169695 | 0.000890445 | -2.436641046 | 0.01483712 |
| smoc1 | 13753 | PDI_H | -0.002044922 | 0.000839489 | -2.435912876 | 0.014866882 |
| arsb | 13579 | PDI_H | -0.002217221 | 0.000910575 | -2.434967137 | 0.014905941 |
| mn1 | 11483 | PDI_H | 0.002452618 | 0.001008117 | 2.432869855 | 0.014994863 |
| cda | 11750 | PDI_H | 0.002295076 | 0.000945814 | 2.426561221 | 0.015257636 |
| bcl2 | 11724 | PDI_H | -0.002342085 | 0.000967175 | -2.421572152 | 0.015468621 |
| ca2 | 13567 | PDI_H | -0.002184363 | 0.000902143 | -2.421305517 | 0.015477915 |
| echs1 | 11474 | PDI_H | -0.002403837 | 0.000993115 | -2.420501201 | 0.015514567 |
| c2 | 13669 | PDI_H | -0.002123291 | 0.000877859 | -2.418715053 | 0.015588423 |
| lilrb4 | 13378 | PDI_H | -0.002088939 | 0.000864013 | -2.417716639 | 0.015631521 |
| pfdn6 | 11483 | PDI_H | -0.002431272 | 0.001008063 | -2.411826197 | 0.015888447 |
| rangap1 | 13308 | PDI_H | -0.002246196 | 0.000931952 | -2.410206057 | 0.015957001 |
| siglec6 | 13753 | PDI_H | -0.002175327 | 0.000903353 | -2.408058352 | 0.016050704 |
| vcan | 13784 | PDI_H | -0.002142022 | 0.000890339 | -2.405851263 | 0.016147927 |
| nmnat1 | 13552 | PDI_H | -0.002157315 | 0.000897699 | -2.403161547 | 0.01626737 |
| apex1 | 13705 | PDI_H | -0.002158355 | 0.000898204 | -2.402966974 | 0.016275875 |
| notch1 | 13800 | PDI_H | 0.002156872 | 0.000898027 | 2.401787992 | 0.016328308 |
| lrpap1 | 13784 | PDI_H | -0.002143072 | 0.000892284 | -2.401782284 | 0.016328578 |
| gnly | 13632 | PDI_H | -0.002170521 | 0.000904729 | -2.39908372 | 0.016449512 |
| angpt2 | 13753 | PDI_H | -0.00213197 | 0.000890528 | -2.394052944 | 0.016676666 |
| c4bpb | 13699 | PDI_H | 0.002133073 | 0.000891295 | 2.393229419 | 0.016714184 |
| park7 | 13632 | PDI_H | -0.002182782 | 0.000912526 | -2.39202272 | 0.016769281 |
| dnm3 | 11757 | PDI_H | -0.002336355 | 0.000980049 | -2.383916399 | 0.01714528 |

| Protein | N_used | term | beta | se | t | p_val |
|---|---|---|---|---|---|---|
| nomo1 | 13632 | PDI_H | -0.002052591 | 0.000861039 | -2.383852261 | 0.017146093 |
| adam23 | 13801 | PDI_H | 0.0021624 | 0.000907692 | 2.382307414 | 0.017218002 |
| rabgap1l | 13378 | PDI_H | -0.002201254 | 0.000924739 | -2.380404773 | 0.017307563 |
| c1qtnf9 | 11520 | PDI_H | 0.002384497 | 0.001002743 | 2.377973453 | 0.017424311 |
| slamf7 | 13621 | PDI_H | -0.002173074 | 0.000914919 | -2.375154421 | 0.017555384 |
| robo2 | 13784 | PDI_H | 0.002055141 | 0.000865401 | 2.374783751 | 0.017572851 |
| bach1 | 13621 | PDI_H | -0.002178843 | 0.000919276 | -2.370172622 | 0.017793641 |
| impg1 | 11383 | PDI_H | 0.002395091 | 0.001010626 | 2.369908648 | 0.017809073 |
| c5 | 11817 | PDI_H | -0.002313096 | 0.000978202 | -2.364641679 | 0.018063609 |
| mme | 13579 | PDI_H | -0.002069767 | 0.000876315 | -2.361898513 | 0.018195662 |
| coq7 | 11474 | PDI_H | -0.002354808 | 0.000997896 | -2.359772467 | 0.018302813 |
| usp28 | 11767 | PDI_H | -0.002305358 | 0.000978946 | -2.354938283 | 0.018542129 |
| bid | 13621 | PDI_H | -0.002144005 | 0.000911111 | -2.353177801 | 0.01862788 |
| fuom | 11537 | PDI_H | -0.002171963 | 0.0009237 | -2.351372263 | 0.018721055 |
| plxdc2 | 11767 | PDI_H | 0.002318679 | 0.000986553 | 2.350282446 | 0.018775609 |
| gipc2 | 11767 | PDI_H | -0.002221514 | 0.000946279 | -2.347629838 | 0.018909778 |
| chchd6 | 11730 | PDI_H | -0.002270695 | 0.000968179 | -2.345325375 | 0.01902707 |
| nbl1 | 13699 | PDI_H | -0.002071081 | 0.0008844 | -2.341791897 | 0.019205674 |
| pqbp1 | 13308 | PDI_H | -0.00216074 | 0.000923099 | -2.340744451 | 0.019260035 |
| pmm2 | 11355 | PDI_H | -0.002360345 | 0.001009527 | -2.338069961 | 0.019400905 |
| fdx1 | 11355 | PDI_H | -0.002333541 | 0.000998911 | -2.336086136 | 0.019504079 |
| psmd9 | 13579 | PDI_H | -0.002138505 | 0.000915596 | -2.335642973 | 0.019524351 |
| adam12 | 11411 | PDI_H | -0.002199685 | 0.000943535 | -2.331322605 | 0.019753694 |
| cep164 | 13378 | PDI_H | -0.002171364 | 0.000931648 | -2.330668938 | 0.019785622 |
| ager | 13647 | PDI_H | 0.002064733 | 0.000885958 | 2.330508428 | 0.019793804 |
| ceacam8 | 13388 | PDI_H | -0.002090463 | 0.000897353 | -2.329587658 | 0.019842761 |
| itpa | 11355 | PDI_H | -0.002340371 | 0.001005966 | -2.326491577 | 0.020009865 |
| cd300lf | 13753 | PDI_H | -0.002067117 | 0.000891144 | -2.31962271 | 0.020375878 |
| cant1 | 13564 | PDI_H | -0.002094967 | 0.000903245 | -2.319378416 | 0.020389316 |
| c7orf50 | 11767 | PDI_H | -0.002279942 | 0.000985833 | -2.312706783 | 0.020755948 |
| dnajc9 | 11724 | PDI_H | -0.002278829 | 0.000986421 | -2.310199542 | 0.020894404 |
| cntn2 | 13753 | PDI_H | 0.002060256 | 0.000892792 | 2.307655225 | 0.021033104 |
| fcrl5 | 13632 | PDI_H | -0.002015078 | 0.000873467 | -2.306987075 | 0.021070467 |
| eda2r | 13753 | PDI_H | -0.001658873 | 0.00071968 | -2.305014036 | 0.021180616 |
| nfasc | 13801 | PDI_H | 0.00196605 | 0.000853183 | 2.304371412 | 0.021216591 |
| cd83 | 13621 | PDI_H | -0.002017872 | 0.000875803 | -2.304023807 | 0.021236297 |
| itgb1 | 13624 | PDI_H | 0.002049127 | 0.000890947 | 2.299942076 | 0.021466558 |
| ly96 | 13552 | PDI_H | -0.00205827 | 0.000894953 | -2.299864706 | 0.021471023 |
| krt5 | 13320 | PDI_H | -0.002025241 | 0.000882218 | -2.295625527 | 0.021712777 |
| metap1d | 13621 | PDI_H | -0.002111896 | 0.000920156 | -2.295150764 | 0.021739627 |
| nudt5 | 13632 | PDI_H | -0.002086955 | 0.000910156 | -2.29296393 | 0.021865248 |
| lgmn | 13710 | PDI_H | -0.002068237 | 0.000904394 | -2.286875758 | 0.022218259 |
| ret | 13579 | PDI_H | 0.001990238 | 0.00087073 | 2.285711387 | 0.022286498 |
| ptn | 13388 | PDI_H | -0.002002928 | 0.000877101 | -2.283576799 | 0.02241202 |
| psme1 | 13532 | PDI_H | -0.002069401 | 0.000907126 | -2.281271751 | 0.02254785 |
| clgn | 11483 | PDI_H | -0.002275914 | 0.00099799 | -2.280497023 | 0.022596487 |
| sart1 | 11355 | PDI_H | -0.002258768 | 0.000992033 | -2.276906898 | 0.022810325 |
| dpp7 | 13516 | PDI_H | -0.002088673 | 0.000917868 | -2.275570161 | 0.022887344 |
| crnn | 13204 | PDI_H | -0.002021692 | 0.000890298 | -2.27080505 | 0.023174829 |
| ren | 13618 | PDI_H | -0.001916001 | 0.000846846 | -2.26251415 | 0.023681355 |
| pbxip1 | 11724 | PDI_H | -0.002162875 | 0.000958013 | -2.257668102 | 0.023984694 |
| itgb2 | 13558 | PDI_H | 0.001987785 | 0.000880843 | 2.256684094 | 0.02404369 |
| stambp | 13535 | PDI_H | -0.002074118 | 0.000920344 | -2.253634068 | 0.024235146 |
| hebp1 | 13388 | PDI_H | -0.002095917 | 0.000930321 | -2.252896972 | 0.024281781 |
| ppl | 11411 | PDI_H | -0.002224819 | 0.000988665 | -2.250327306 | 0.024447162 |
| timp1 | 13475 | PDI_H | -0.001979218 | 0.000879755 | -2.249737317 | 0.02448171 |
| hspb1 | 13394 | PDI_H | -0.002085246 | 0.000927975 | -2.247092591 | 0.024650338 |
| cd99 | 13632 | PDI_H | -0.001741875 | 0.000776436 | -2.243423255 | 0.02488554 |
| epha1 | 13695 | PDI_H | -0.001809874 | 0.000808423 | -2.238771432 | 0.025186803 |
| cc2d1a | 13552 | PDI_H | -0.002040375 | 0.000914137 | -2.232022698 | 0.025629757 |
| hif1a | 11411 | PDI_H | 0.002242018 | 0.001007159 | 2.226080429 | 0.026028269 |
| cd79b | 13801 | PDI_H | -0.001968428 | 0.000885004 | -2.224201261 | 0.026151036 |
| egfr | 13800 | PDI_H | 0.001990958 | 0.000895445 | 2.223428428 | 0.026203068 |
| aif1l | 11474 | PDI_H | -0.002223299 | 0.001000637 | -2.221883295 | 0.026310644 |
| apbb1ip | 13579 | PDI_H | -0.001962284 | 0.000883703 | -2.220525573 | 0.026399567 |
| letm1 | 11757 | PDI_H | -0.002189381 | 0.000985962 | -2.220552866 | 0.026400269 |
| cxcl1 | 13607 | PDI_H | -0.002033466 | 0.000915861 | -2.220278251 | 0.026416311 |
| nt5c1a | 11767 | PDI_H | -0.001948988 | 0.000878585 | -2.21832716 | 0.026551568 |
| bcan | 13552 | PDI_H | 0.001902323 | 0.000858015 | 2.217122101 | 0.026631294 |
| vegfd | 13782 | PDI_H | 0.001937647 | 0.000874481 | 2.215768103 | 0.026723679 |

| Protein | N_used | term | beta | se | t | p_val |
|---|---|---|---|---|---|---|
| ptgr1 | 11781 | PDI_H | -0.002084637 | 0.000943193 | -2.210191706 | 0.027111029 |
| il18rap | 13469 | PDI_H | -0.002023428 | 0.00091577 | -2.209537925 | 0.027154023 |
| preb | 13378 | PDI_H | -0.002040086 | 0.000923757 | -2.208465381 | 0.027228756 |
| klk13 | 13340 | PDI_H | -0.002001613 | 0.000906422 | -2.20825666 | 0.027243346 |
| cntf | 11383 | PDI_H | 0.002232547 | 0.001011263 | 2.207681801 | 0.027286352 |
| ceacam21 | 13621 | PDI_H | -0.002015706 | 0.000915096 | -2.202727549 | 0.027630659 |
| ezr | 13784 | PDI_H | -0.001933854 | 0.000878376 | -2.201624743 | 0.027708348 |
| vsnl1 | 11767 | PDI_H | -0.001957233 | 0.000891233 | -2.196095128 | 0.028104599 |
| cd163 | 13689 | PDI_H | -0.001914749 | 0.000873298 | -2.192548282 | 0.028356727 |
| xg | 13721 | PDI_H | -0.001479255 | 0.000675975 | -2.188327389 | 0.02866258 |
| nid1 | 13639 | PDI_H | -0.001960154 | 0.000895762 | -2.188253982 | 0.028668026 |
| rnf149 | 11474 | PDI_H | -0.002108088 | 0.000965372 | -2.183704054 | 0.029004206 |
| tinagl1 | 13585 | PDI_H | -0.001944737 | 0.000891398 | -2.181671746 | 0.029150863 |
| ptprn2 | 13513 | PDI_H | -0.001988768 | 0.000912576 | -2.179289571 | 0.029327389 |
| f13b | 11740 | PDI_H | 0.002122755 | 0.000976427 | 2.174002451 | 0.029724882 |
| ceacam3 | 13308 | PDI_H | -0.002026824 | 0.000932703 | -2.173064909 | 0.029793028 |
| gpr158 | 11757 | PDI_H | -0.00209615 | 0.000967952 | -2.16555254 | 0.030365459 |
| slitrk6 | 13388 | PDI_H | 0.001992467 | 0.000920846 | 2.163735984 | 0.030502263 |
| agr3 | 13291 | PDI_H | -0.001967289 | 0.00090937 | -2.163353921 | 0.03053175 |
| vamp5 | 13388 | PDI_H | -0.001995229 | 0.00092287 | -2.161983789 | 0.030637101 |
| foxo1 | 13621 | PDI_H | -0.001989007 | 0.00092082 | -2.160039005 | 0.030787054 |
| il1rl1 | 13750 | PDI_H | -0.00183289 | 0.000849184 | -2.158414447 | 0.03091289 |
| comt | 13243 | PDI_H | -0.00200625 | 0.000929631 | -2.158114651 | 0.030936852 |
| stip1 | 13605 | PDI_H | -0.001970812 | 0.00091359 | -2.157218043 | 0.031006149 |
| decr1 | 13621 | PDI_H | -0.001983533 | 0.00091955 | -2.157070748 | 0.031017604 |
| grhpr | 11781 | PDI_H | -0.002121536 | 0.000983971 | -2.156095561 | 0.031096403 |
| lcn2 | 13249 | PDI_H | -0.001928575 | 0.000894659 | -2.155655045 | 0.031128577 |
| tnfrsf19 | 13753 | PDI_H | -0.001851237 | 0.000858838 | -2.155514666 | 0.031138892 |
| smndc1 | 11724 | PDI_H | -0.002080418 | 0.000965562 | -2.154619699 | 0.031211931 |
| cacnb1 | 11383 | PDI_H | -0.002176803 | 0.001011433 | -2.152197579 | 0.031402778 |
| cntn4 | 13632 | PDI_H | 0.001931295 | 0.00089804 | 2.15056683 | 0.031527949 |
| mamdc2 | 11781 | PDI_H | -0.002067225 | 0.000962343 | -2.148117209 | 0.031724794 |
| vwf | 13800 | PDI_H | -0.00193353 | 0.000901411 | -2.145004447 | 0.031969909 |
| vcam1 | 13669 | PDI_H | -0.001936036 | 0.000904391 | -2.140707602 | 0.032315279 |
| krt8 | 11383 | PDI_H | -0.002130768 | 0.000996025 | -2.139270824 | 0.032434972 |
| bank1 | 13621 | PDI_H | -0.001961427 | 0.000917808 | -2.137077264 | 0.032609482 |
| mdh1 | 11716 | PDI_H | -0.002100241 | 0.000983958 | -2.134482042 | 0.032824058 |
| bcr | 13621 | PDI_H | -0.001960442 | 0.000918816 | -2.133659667 | 0.032888479 |
| ctrb1 | 13632 | PDI_H | -0.001954719 | 0.000918964 | -2.127088786 | 0.033430626 |
| heph | 11781 | PDI_H | 0.002043064 | 0.000961848 | 2.124103194 | 0.033682308 |
| otud7b | 11757 | PDI_H | -0.002097563 | 0.000990473 | -2.117738844 | 0.034218143 |
| ncf2 | 13607 | PDI_H | -0.00192502 | 0.000909032 | -2.117658457 | 0.034222106 |
| il12rb1 | 13378 | PDI_H | -0.001941159 | 0.000916672 | -2.117616591 | 0.034225965 |
| cox5b | 13579 | PDI_H | -0.001945817 | 0.000920471 | -2.113936846 | 0.03453886 |
| ccl19 | 13784 | PDI_H | -0.001886249 | 0.000893212 | -2.111758246 | 0.034725151 |
| tcl1a | 13538 | PDI_H | -0.001912116 | 0.00090658 | -2.109153468 | 0.034949666 |
| il18bp | 13689 | PDI_H | -0.001848273 | 0.00087642 | -2.10889096 | 0.034972125 |
| eci2 | 11355 | PDI_H | -0.002126157 | 0.001009339 | -2.106485126 | 0.035184141 |
| epha2 | 13129 | PDI_H | -0.001867173 | 0.000886715 | -2.105718645 | 0.035247756 |
| wnt9a | 13378 | PDI_H | -0.001758187 | 0.000835051 | -2.10548385 | 0.035267819 |
| hmmr | 11278 | PDI_H | -0.002137866 | 0.001016346 | -2.103483663 | 0.035445625 |
| clec4d | 13621 | PDI_H | -0.001857961 | 0.000883299 | -2.103434522 | 0.03544611 |
| hnrnpk | 13350 | PDI_H | -0.001946342 | 0.000925963 | -2.101965527 | 0.035574998 |
| erbb3 | 13613 | PDI_H | -0.001825587 | 0.000868754 | -2.101384989 | 0.035625534 |
| milr1 | 13378 | PDI_H | -0.001918602 | 0.000913513 | -2.100245395 | 0.03572595 |
| ctnna1 | 11383 | PDI_H | 0.002118494 | 0.001010524 | 2.0964315 | 0.036065971 |
| omd | 13710 | PDI_H | 0.001826225 | 0.000871174 | 2.096281305 | 0.036075542 |
| spon1 | 13677 | PDI_H | -0.001814845 | 0.000866936 | -2.093401576 | 0.036331709 |
| hdgf | 13721 | PDI_H | -0.001917615 | 0.000916113 | -2.093207998 | 0.036348922 |
| rln2 | 11512 | PDI_H | 0.002075527 | 0.000993947 | 2.088165299 | 0.03680487 |
| cst6 | 13287 | PDI_H | -0.001861745 | 0.000892565 | -2.085838297 | 0.03701232 |
| otoa | 11383 | PDI_H | 0.002108538 | 0.001011116 | 2.085356246 | 0.03705922 |
| folr1 | 13646 | PDI_H | 0.001801841 | 0.00086413 | 2.085149851 | 0.037074256 |
| serpind1 | 11790 | PDI_H | -0.001933287 | 0.00093011 | -2.07855843 | 0.037679551 |
| tigit | 11570 | PDI_H | -0.002077171 | 0.000999361 | -2.078500358 | 0.037685306 |
| ltbr | 13594 | PDI_H | -0.001851939 | 0.000891113 | -2.078231213 | 0.037706801 |
| cd80 | 11781 | PDI_H | -0.001971018 | 0.000949145 | -2.076624405 | 0.037857885 |
| tgm2 | 13600 | PDI_H | -0.001899741 | 0.000915197 | -2.075771761 | 0.037933827 |
| ankrd54 | 13308 | PDI_H | -0.001919639 | 0.000925063 | -2.075142669 | 0.037992498 |
| cpped1 | 13784 | PDI_H | -0.00186442 | 0.000899974 | -2.071637283 | 0.038317853 |

| Protein | N_used | term | beta | se | t | p_val |
|---|---|---|---|---|---|---|
| prr4 | 11448 | PDI_H | -0.002072082 | 0.00100104 | -2.069928351 | 0.038481453 |
| gzmh | 13388 | PDI_H | -0.001860782 | 0.000899087 | -2.069634934 | 0.038505698 |
| pdcd6 | 13618 | PDI_H | -0.001907122 | 0.0009217 | -2.069135893 | 0.038552174 |
| cpm | 13721 | PDI_H | -0.001663225 | 0.000803917 | -2.068900655 | 0.03857411 |
| pdgfra | 13618 | PDI_H | -0.001877418 | 0.000907593 | -2.068568644 | 0.038605429 |
| ssbp1 | 11411 | PDI_H | -0.002084149 | 0.001008332 | -2.066926304 | 0.038763623 |
| enpp5 | 13710 | PDI_H | 0.001744875 | 0.000844589 | 2.065947209 | 0.038852223 |
| slc51b | 11355 | PDI_H | 0.002074449 | 0.001004097 | 2.065984789 | 0.038852569 |
| slamf6 | 13308 | PDI_H | -0.001899297 | 0.000919521 | -2.065528973 | 0.03889231 |
| cxcl17 | 13801 | PDI_H | -0.001562448 | 0.000756448 | -2.065507138 | 0.038893682 |
| il17a | 13359 | PDI_H | -0.001925004 | 0.000932125 | -2.065178199 | 0.038925406 |
| fxn | 13753 | PDI_H | -0.001891134 | 0.000916147 | -2.064224835 | 0.03901513 |
| ccar2 | 11767 | PDI_H | -0.002048789 | 0.000993126 | -2.062968401 | 0.039137542 |
| mia | 13699 | PDI_H | -0.001855157 | 0.000900591 | -2.059932456 | 0.039423862 |
| ing1 | 13303 | PDI_H | -0.001913812 | 0.000929691 | -2.058544979 | 0.039557295 |
| rpl14 | 11474 | PDI_H | -0.002027685 | 0.000985973 | -2.056532397 | 0.039753813 |
| s100a14 | 11757 | PDI_H | 0.002041714 | 0.000992863 | 2.056390681 | 0.039766918 |
| ddx58 | 13308 | PDI_H | -0.001904191 | 0.000926242 | -2.055823706 | 0.039818993 |
| mcfd2 | 13547 | PDI_H | -0.00189708 | 0.00092395 | -2.053227572 | 0.040069688 |
| urod | 11537 | PDI_H | -0.00204465 | 0.000995925 | -2.053015203 | 0.040093627 |
| fcar | 13621 | PDI_H | -0.001862526 | 0.000907786 | -2.051723845 | 0.040215602 |
| scamp3 | 13579 | PDI_H | -0.001877473 | 0.00091652 | -2.04847961 | 0.040532229 |
| nsfl1c | 13784 | PDI_H | -0.001849478 | 0.000903437 | -2.047157916 | 0.040661517 |
| cep152 | 11304 | PDI_H | -0.002077028 | 0.001015217 | -2.045895241 | 0.040789795 |
| reg1a | 13560 | PDI_H | -0.001834909 | 0.000897023 | -2.045553805 | 0.040819562 |
| gip | 11474 | PDI_H | 0.001947098 | 0.000952873 | 2.043396466 | 0.041036024 |
| rfc4 | 11730 | PDI_H | -0.002028185 | 0.00099293 | -2.04262536 | 0.04111187 |
| pigr | 13463 | PDI_H | -0.001801382 | 0.000882536 | -2.041141535 | 0.041256237 |
| extl1 | 11355 | PDI_H | 0.002048373 | 0.001003762 | 2.040695585 | 0.041304198 |
| inpp5d | 11474 | PDI_H | -0.002015965 | 0.000988197 | -2.040043045 | 0.041368914 |
| il12rb2 | 11411 | PDI_H | 0.002044892 | 0.001003082 | 2.038609909 | 0.041512002 |
| cdh17 | 13618 | PDI_H | 0.001844342 | 0.000904982 | 2.037988632 | 0.04157037 |
| pxn | 13320 | PDI_H | -0.001847815 | 0.000906704 | -2.037946558 | 0.041575011 |
| erbb2 | 13699 | PDI_H | -0.001731878 | 0.000850165 | -2.037107842 | 0.041658435 |
| mcam | 13689 | PDI_H | 0.00178324 | 0.000876056 | 2.035531858 | 0.041816622 |
| dctpp1 | 13618 | PDI_H | 0.001824029 | 0.000896132 | 2.035447396 | 0.041825214 |
| nbn | 13378 | PDI_H | -0.001852611 | 0.00091025 | -2.035276276 | 0.041842769 |
| myom2 | 11355 | PDI_H | 0.002006417 | 0.000986521 | 2.033830392 | 0.041991923 |
| kynu | 13801 | PDI_H | -0.001757955 | 0.000864879 | -2.03260244 | 0.042111826 |
| serpina3 | 11772 | PDI_H | -0.001976243 | 0.000977159 | -2.022437661 | 0.043153748 |
| mgmt | 13621 | PDI_H | -0.00186059 | 0.000922335 | -2.017259976 | 0.043687985 |
| jun | 13378 | PDI_H | -0.001864505 | 0.000924311 | -2.017183728 | 0.043696296 |
| mertk | 13801 | PDI_H | 0.001758284 | 0.000872793 | 2.014548288 | 0.043971377 |
| dtx2 | 11483 | PDI_H | 0.002013432 | 0.001004146 | 2.005118288 | 0.044973853 |
| grp | 11474 | PDI_H | -0.002003979 | 0.000999622 | -2.004737499 | 0.045014592 |
| osm | 13551 | PDI_H | -0.001784806 | 0.000890571 | -2.004113939 | 0.045077739 |
| wars | 13632 | PDI_H | -0.001814994 | 0.000905844 | -2.003650097 | 0.045127326 |
| pla2g2a | 13629 | PDI_H | -0.001742461 | 0.000870073 | -2.002659996 | 0.045233584 |
| ntrk2 | 13618 | PDI_H | 0.001790595 | 0.000894229 | 2.002389004 | 0.045262719 |
| nudt16 | 11705 | PDI_H | -0.0019901 | 0.000993849 | -2.002416114 | 0.045263046 |
| tspan1 | 13388 | PDI_H | 0.001745666 | 0.00087185 | 2.00225382 | 0.045277591 |
| hnmt | 13486 | PDI_H | -0.001688974 | 0.000843945 | -2.001284945 | 0.045381709 |
| mdga1 | 13552 | PDI_H | 0.001794064 | 0.000897382 | 1.999220386 | 0.045604463 |
| srpk2 | 13621 | PDI_H | -0.001829082 | 0.000915168 | -1.998630471 | 0.045668207 |
| oscar | 13677 | PDI_H | -0.001801899 | 0.000901569 | -1.99862627 | 0.045668581 |
| mmp9 | 13602 | PDI_H | -0.001781443 | 0.000892473 | -1.996075136 | 0.045945664 |
| ncam1 | 13689 | PDI_H | 0.001712409 | 0.000859841 | 1.991542249 | 0.04644116 |
| havcr1 | 13736 | PDI_H | -0.0016186 | 0.000813243 | -1.99030248 | 0.046577429 |
| azu1 | 13254 | PDI_H | -0.001830028 | 0.000920034 | -1.98908786 | 0.046712048 |
| ces1 | 13687 | PDI_H | -0.001692207 | 0.000851474 | -1.987385596 | 0.046899597 |
| gp6 | 13597 | PDI_H | -0.001832029 | 0.000922751 | -1.985399547 | 0.047120109 |
| fos | 11271 | PDI_H | -0.002016096 | 0.001015562 | -1.98520281 | 0.047146135 |
| smoc2 | 13710 | PDI_H | -0.001654301 | 0.000833694 | -1.984303245 | 0.047241967 |
| scpep1 | 11430 | PDI_H | -0.001958189 | 0.000987183 | -1.983612223 | 0.047322997 |
| yars1 | 11724 | PDI_H | -0.001970852 | 0.000993764 | -1.983220367 | 0.047366138 |
| hdac9 | 11383 | PDI_H | 0.002005854 | 0.001011439 | 1.983168617 | 0.047372616 |
| omp | 11383 | PDI_H | -0.002003538 | 0.001011318 | -1.981115718 | 0.047602349 |
| ripk4 | 11355 | PDI_H | -0.002005437 | 0.001012438 | -1.980798724 | 0.047637966 |
| fdx2 | 11355 | PDI_H | -0.001995701 | 0.001007692 | -1.980466192 | 0.04767529 |
| dll1 | 13699 | PDI_H | -0.001736837 | 0.000877251 | -1.979863991 | 0.047738812 |

| Protein | N_used | term | beta | se | t | p_val |
|---|---|---|---|---|---|---|
| ly9 | 13483 | PDI_H | -0.001813153 | 0.000916663 | -1.97799247 | 0.0479499 |
| ca5a | 13388 | PDI_H | -0.001705754 | 0.000863636 | -1.975084621 | 0.048279074 |
| klk11 | 13562 | PDI_H | -0.001793405 | 0.000908375 | -1.974301445 | 0.048367752 |
| slitrk2 | 13753 | PDI_H | -0.0017203 | 0.000871419 | -1.974136431 | 0.048386227 |
| blvrb | 13632 | PDI_H | -0.001783942 | 0.000903781 | -1.973866562 | 0.048417095 |
| cd28 | 13308 | PDI_H | -0.001788966 | 0.000907189 | -1.971986934 | 0.048631792 |
| tmprss15 | 13579 | PDI_H | 0.001790693 | 0.000908203 | 1.971688194 | 0.048665497 |
| cxcl13 | 13784 | PDI_H | -0.001776422 | 0.000901829 | -1.969799053 | 0.048881413 |
| hdgfl2 | 11767 | PDI_H | -0.00192755 | 0.000979447 | -1.9679987 | 0.049091655 |
| cd5 | 13736 | PDI_H | -0.001735161 | 0.000882169 | -1.966927045 | 0.049211737 |
| fabp9 | 13621 | PDI_H | -0.001775082 | 0.000903043 | -1.965667238 | 0.049357363 |
| max | 13320 | PDI_H | -0.001825031 | 0.00092956 | -1.963328471 | 0.049628812 |
| itgbl1 | 11790 | PDI_H | -0.001833868 | 0.000934538 | -1.962326006 | 0.04974805 |
| abl1 | 13579 | PDI_H | -0.001796875 | 0.00091617 | -1.961290685 | 0.049865545 |
| il2rg | 11730 | PDI_H | 0.001940747 | 0.000989853 | 1.960640886 | 0.049944584 |
| cfc1 | 13308 | PDI_H | -0.001796075 | 0.000916482 | -1.95974861 | 0.050046039 |
| tnfrsf8 | 13784 | PDI_H | -0.001773907 | 0.000905492 | -1.959053883 | 0.050126624 |
| cdh3 | 13784 | PDI_H | -0.001775392 | 0.000907466 | -1.956428527 | 0.050434875 |
| sirt2 | 13579 | PDI_H | -0.001800702 | 0.000921487 | -1.954126479 | 0.050706777 |
| gpa33 | 13579 | PDI_H | 0.001777606 | 0.000910402 | 1.952551518 | 0.050893297 |
| ahsp | 13597 | PDI_H | -0.001732476 | 0.000887802 | -1.95142053 | 0.051027564 |
| il1rap | 13632 | PDI_H | 0.001710211 | 0.000876485 | 1.951215122 | 0.051051934 |
| hagh | 13715 | PDI_H | -0.001747155 | 0.000895647 | -1.950718929 | 0.051110846 |
| irak1 | 13378 | PDI_H | -0.001806872 | 0.0009272 | -1.948740462 | 0.051347322 |
| rasa1 | 13320 | PDI_H | -0.001819092 | 0.000934411 | -1.946779357 | 0.051582204 |
| sdhb | 11724 | PDI_H | -0.00193835 | 0.000995737 | -1.946648773 | 0.051600733 |
| mmp10 | 13630 | PDI_H | -0.001771402 | 0.000910075 | -1.946434775 | 0.051623073 |
| cyb5r2 | 11590 | PDI_H | -0.001924679 | 0.000989175 | -1.945740621 | 0.051710071 |
| ccl17 | 13628 | PDI_H | -0.001774296 | 0.000912248 | -1.944970221 | 0.051799121 |
| lonp1 | 11474 | PDI_H | -0.001947476 | 0.001001368 | -1.944814302 | 0.051821757 |
| gzma | 13801 | PDI_H | -0.001730728 | 0.000892427 | -1.939349216 | 0.052479201 |
| atf2 | 13320 | PDI_H | -0.001810651 | 0.000933752 | -1.939112772 | 0.052508718 |
| igsf21 | 11474 | PDI_H | 0.001879336 | 0.000969187 | 1.939084925 | 0.052515509 |
| cdh2 | 13618 | PDI_H | -0.001582554 | 0.000817263 | -1.93640619 | 0.052838654 |
| inhbc | 13632 | PDI_H | -0.001600348 | 0.000826547 | -1.936185296 | 0.052865674 |
| hgfac | 11666 | PDI_H | -0.001900461 | 0.000981596 | -1.936093954 | 0.052880346 |
| il5 | 13378 | PDI_H | -0.00179487 | 0.000927759 | -1.934628891 | 0.053056928 |
| rtn4ip1 | 11757 | PDI_H | -0.001918604 | 0.000991705 | -1.93465128 | 0.053057087 |
| il10ra | 13378 | PDI_H | -0.001798909 | 0.000930354 | -1.933575669 | 0.05318641 |
| copb2 | 11355 | PDI_H | 0.001955658 | 0.001011545 | 1.933336918 | 0.053219561 |
| hip1r | 11738 | PDI_H | -0.001823698 | 0.000943581 | -1.932740338 | 0.053292244 |
| l1cam | 13699 | PDI_H | -0.001727493 | 0.000894948 | -1.930272575 | 0.053593708 |
| itga2 | 11537 | PDI_H | 0.001867956 | 0.000968387 | 1.928934953 | 0.053763464 |
| sirpb1 | 13643 | PDI_H | -0.001722807 | 0.000894635 | -1.925708274 | 0.054161586 |
| edf1 | 11757 | PDI_H | -0.001912334 | 0.000993566 | -1.924717098 | 0.054288887 |
| lsm1 | 13308 | PDI_H | -0.001794387 | 0.000932767 | -1.923724545 | 0.054410444 |
| f10 | 11794 | PDI_H | -0.001871648 | 0.000973624 | -1.922352629 | 0.054585486 |
| akt1s1 | 13320 | PDI_H | -0.001784212 | 0.000928525 | -1.921553834 | 0.054683255 |
| mrpl52 | 11278 | PDI_H | -0.001953056 | 0.00101647 | -1.921409756 | 0.05470527 |
| atraid | 11724 | PDI_H | -0.001853314 | 0.000964813 | -1.920903749 | 0.054768091 |
| btn1a1 | 11711 | PDI_H | 0.001912872 | 0.000996879 | 1.9188617 | 0.055026143 |
| ptprc | 11790 | PDI_H | -0.001862597 | 0.000970767 | -1.918685697 | 0.055048266 |
| kitlg | 13618 | PDI_H | 0.001738583 | 0.000906421 | 1.918074806 | 0.055122434 |
| cdh1 | 13181 | PDI_H | -0.001752417 | 0.000913826 | -1.917670377 | 0.055174426 |
| aoc3 | 13689 | PDI_H | 0.00168431 | 0.000878652 | 1.916924468 | 0.05526834 |
| morf4l1 | 11570 | PDI_H | 0.001921975 | 0.00100297 | 1.916282931 | 0.055353729 |
| pla2g10 | 13552 | PDI_H | 0.001689858 | 0.000883293 | 1.913134496 | 0.055751905 |
| fus | 13283 | PDI_H | -0.001750053 | 0.000915098 | -1.912421466 | 0.055843661 |
| amy2b | 12703 | PDI_H | 0.001769433 | 0.000925872 | 1.911099658 | 0.056014279 |
| lpo | 13212 | PDI_H | -0.001758951 | 0.00092101 | -1.909807048 | 0.056179713 |
| sod2 | 13632 | PDI_H | -0.001713876 | 0.000898695 | -1.907070657 | 0.056532455 |
| gpkow | 13320 | PDI_H | -0.001737449 | 0.000911365 | -1.90642552 | 0.056616537 |
| dpt | 13800 | PDI_H | -0.001515829 | 0.000795156 | -1.906328014 | 0.056628431 |
| tbc1d17 | 13320 | PDI_H | -0.001756435 | 0.000923741 | -1.901436328 | 0.057266462 |
| rpe | 11570 | PDI_H | -0.001904601 | 0.001001796 | -1.901186695 | 0.05730241 |
| ca14 | 13753 | PDI_H | 0.001568248 | 0.000825118 | 1.900635311 | 0.057370703 |
| serpina7 | 11817 | PDI_H | -0.001829416 | 0.000962595 | -1.900504556 | 0.057391274 |
| acy1 | 13618 | PDI_H | -0.001532845 | 0.000808131 | -1.896777614 | 0.057878453 |
| wdr46 | 11730 | PDI_H | -0.001862078 | 0.000981835 | -1.89652895 | 0.057914706 |
| hepacam2 | 11767 | PDI_H | 0.00186347 | 0.000983862 | 1.894035925 | 0.058244768 |

| Protein | N_used | term | beta | se | t | p_val |
|---|---|---|---|---|---|---|
| ptp4a3 | 11411 | PDI_H | -0.001834194 | 0.000970302 | -1.890333689 | 0.058738688 |
| gusb | 13685 | PDI_H | -0.001524893 | 0.000806719 | -1.890241746 | 0.058746765 |
| kir3dl2 | 11304 | PDI_H | -0.001916558 | 0.001015136 | -1.887980473 | 0.059054187 |
| acp1 | 11411 | PDI_H | 0.001885843 | 0.001000249 | 1.88537334 | 0.05940486 |
| ptprs | 13800 | PDI_H | 0.001711826 | 0.000908025 | 1.885218438 | 0.059421361 |
| ung | 11730 | PDI_H | -0.001876827 | 0.000996346 | -1.883709851 | 0.059628973 |
| clec11a | 13615 | PDI_H | -0.001713649 | 0.000910373 | -1.882358924 | 0.059808625 |
| pyy | 11355 | PDI_H | 0.001852791 | 0.000984847 | 1.881298722 | 0.059956884 |
| cyb5a | 11304 | PDI_H | -0.001906067 | 0.001015067 | -1.877775294 | 0.060437663 |
| clu | 11817 | PDI_H | 0.001832913 | 0.000976518 | 1.876988031 | 0.060544377 |
| fut1 | 11550 | PDI_H | 0.00188113 | 0.001004082 | 1.873481792 | 0.061027143 |
| bola1 | 11383 | PDI_H | -0.001868845 | 0.000998433 | -1.871778353 | 0.061262926 |
| igdcc4 | 11790 | PDI_H | 0.00172952 | 0.000925745 | 1.868247735 | 0.061752357 |
| rrp15 | 11383 | PDI_H | 0.001887138 | 0.001011424 | 1.865822302 | 0.062091959 |
| amot | 11537 | PDI_H | 0.001689948 | 0.00090856 | 1.86003048 | 0.062906671 |
| lif | 13320 | PDI_H | -0.001726432 | 0.000929385 | -1.857606983 | 0.063246918 |
| il5ra | 13621 | PDI_H | -0.001688318 | 0.000910207 | -1.85487209 | 0.063636107 |
| rbp7 | 11724 | PDI_H | -0.001760192 | 0.000949872 | -1.853083067 | 0.063895588 |
| tgfbr2 | 13699 | PDI_H | -0.001543826 | 0.000833564 | -1.852077437 | 0.064036222 |
| clstn2 | 13801 | PDI_H | 0.001669826 | 0.000904145 | 1.846857172 | 0.064789255 |
| cep350 | 11304 | PDI_H | 0.001875993 | 0.001015769 | 1.846869204 | 0.064792245 |
| erp44 | 13561 | PDI_H | -0.001676782 | 0.000908857 | -1.844934493 | 0.065068879 |
| ceacam1 | 13678 | PDI_H | -0.001679697 | 0.000910611 | -1.84458219 | 0.065119967 |
| cd40 | 13642 | PDI_H | -0.00168018 | 0.000910991 | -1.84434255 | 0.065154921 |
| ggct | 11763 | PDI_H | -0.001824868 | 0.00098951 | -1.844214133 | 0.065177095 |
| cst7 | 13661 | PDI_H | -0.001659081 | 0.000902741 | -1.837825463 | 0.06610985 |
| lpa | 11526 | PDI_H | 0.00183853 | 0.001000925 | 1.83683127 | 0.066260569 |
| ctse | 11537 | PDI_H | 0.001836193 | 0.000999656 | 1.836825198 | 0.066261441 |
| prss22 | 11411 | PDI_H | -0.001828836 | 0.000996154 | -1.835896296 | 0.066399027 |
| mecr | 11474 | PDI_H | -0.001838232 | 0.001002486 | -1.833672432 | 0.066728548 |
| fgl1 | 11794 | PDI_H | -0.001684974 | 0.000919727 | -1.832036829 | 0.066971163 |
| clec1a | 13388 | PDI_H | -0.00167401 | 0.000913918 | -1.831685225 | 0.067020557 |
| rida | 11537 | PDI_H | -0.001691105 | 0.000923744 | -1.830707115 | 0.067170077 |
| nt5e | 13699 | PDI_H | -0.001653405 | 0.000903255 | -1.830496099 | 0.067197522 |
| fbln2 | 11790 | PDI_H | -0.001722735 | 0.000941208 | -1.830344753 | 0.06722366 |
| nfya | 11355 | PDI_H | -0.00183864 | 0.001004893 | -1.829687056 | 0.06732298 |
| morn4 | 11304 | PDI_H | -0.001853223 | 0.001014771 | -1.826247064 | 0.067839446 |
| bap18 | 11767 | PDI_H | -0.001747793 | 0.000957214 | -1.82591582 | 0.067888298 |
| scara5 | 13784 | PDI_H | -0.001535092 | 0.000840788 | -1.825778308 | 0.067905305 |
| itgb5 | 13699 | PDI_H | 0.001641045 | 0.000899765 | 1.823859723 | 0.068195078 |
| eif2ak2 | 11684 | PDI_H | -0.001815773 | 0.00099582 | -1.823394061 | 0.068269293 |
| zpr1 | 11383 | PDI_H | 0.001841126 | 0.001009847 | 1.823172692 | 0.068303482 |
| defb103a_defb103b | 11317 | PDI_H | 0.001836994 | 0.001007856 | 1.822675232 | 0.068378995 |
| agrp | 13801 | PDI_H | 0.001628093 | 0.000893866 | 1.82140648 | 0.068566748 |
| prok1 | 13621 | PDI_H | -0.001256105 | 0.000691646 | -1.816109996 | 0.069375494 |
| f12 | 11817 | PDI_H | -0.001780502 | 0.000980865 | -1.815236121 | 0.069512994 |
| otud6b | 11724 | PDI_H | -0.001806174 | 0.000995769 | -1.813847087 | 0.06972685 |
| dnajb2 | 11537 | PDI_H | -0.001798398 | 0.000992642 | -1.811728973 | 0.070054102 |
| ltb | 11730 | PDI_H | 0.001780097 | 0.000983029 | 1.810827674 | 0.07019313 |
| eif2s2 | 11474 | PDI_H | -0.001822723 | 0.001006581 | -1.810805792 | 0.07019709 |
| lrrn1 | 13378 | PDI_H | 0.001654283 | 0.000914531 | 1.808886857 | 0.070491043 |
| gal | 13710 | PDI_H | 0.001579226 | 0.000874488 | 1.805887601 | 0.070957841 |
| znf830 | 11730 | PDI_H | 0.001779016 | 0.00098606 | 1.804165777 | 0.07123099 |
| c1galt1c1 | 11355 | PDI_H | -0.001821539 | 0.001011279 | -1.801223193 | 0.071694256 |
| serpina12 | 13212 | PDI_H | 0.001642525 | 0.000912005 | 1.801004078 | 0.071725052 |
| pklr | 13676 | PDI_H | -0.0016498 | 0.000917025 | -1.799077673 | 0.072028452 |
| cx3cl1 | 13552 | PDI_H | -0.001639481 | 0.00091234 | -1.797006368 | 0.072356886 |
| vta1 | 13784 | PDI_H | -0.001640378 | 0.000916176 | -1.790461592 | 0.073401694 |
| metap2 | 13732 | PDI_H | -0.001634893 | 0.000913515 | -1.789672768 | 0.07352858 |
| scin | 11383 | PDI_H | 0.001804536 | 0.001010902 | 1.785075807 | 0.074275669 |
| zp3 | 11474 | PDI_H | 0.001790058 | 0.001003354 | 1.784074263 | 0.074438044 |
| ica1 | 13378 | PDI_H | -0.001657916 | 0.000929864 | -1.782966705 | 0.074614411 |
| creg1 | 13488 | PDI_H | -0.001590147 | 0.000891943 | -1.78278976 | 0.074643037 |
| ccl2 | 13526 | PDI_H | -0.001620434 | 0.000909637 | -1.781407523 | 0.074868352 |
| lect2 | 11781 | PDI_H | -0.001542229 | 0.000865921 | -1.781026671 | 0.074933878 |
| tnfrsf11a | 13801 | PDI_H | -0.00151129 | 0.000849364 | -1.779318807 | 0.07520953 |
| ccl24 | 13691 | PDI_H | 0.001609918 | 0.000905397 | 1.77813473 | 0.075403937 |
| man2b2 | 11781 | PDI_H | -0.001701975 | 0.000958947 | -1.774837028 | 0.075950643 |
| vegfa | 13759 | PDI_H | -0.001582877 | 0.000892062 | -1.774402948 | 0.07601865 |
| dsg2 | 13632 | PDI_H | 0.001562917 | 0.000881229 | 1.773564559 | 0.076157545 |

| Protein | N_used | term | beta | se | t | p_val |
|---|---|---|---|---|---|---|
| lilra5 | 13599 | PDI_H | -0.001534532 | 0.000865719 | -1.772552707 | 0.076325257 |
| dda1 | 11355 | PDI_H | 0.001794033 | 0.001012675 | 1.771579063 | 0.076491301 |
| s100a3 | 11730 | PDI_H | 0.001756211 | 0.000991903 | 1.770547424 | 0.076661986 |
| thbs4 | 13639 | PDI_H | 0.001532305 | 0.000866021 | 1.769363031 | 0.076855673 |
| pof1b | 11411 | PDI_H | -0.001763777 | 0.000997507 | -1.768184055 | 0.077056882 |
| dynlt1 | 11483 | PDI_H | -0.001764267 | 0.000997788 | -1.768178321 | 0.077057673 |
| igfbpl1 | 13618 | PDI_H | -0.001506733 | 0.000852192 | -1.768067012 | 0.077072108 |
| kyat1 | 13591 | PDI_H | -0.001624766 | 0.000919119 | -1.767742409 | 0.07712643 |
| grk5 | 13388 | PDI_H | -0.001645145 | 0.000930715 | -1.767614007 | 0.07714825 |
| txlna | 13552 | PDI_H | -0.001616312 | 0.000915385 | -1.765718071 | 0.077465693 |
| ccl26 | 13621 | PDI_H | -0.001595698 | 0.000904253 | -1.764659056 | 0.077643511 |
| lrg1 | 11817 | PDI_H | -0.001669872 | 0.000946542 | -1.764182515 | 0.077727115 |
| eif4g1 | 13801 | PDI_H | -0.001603477 | 0.000909917 | -1.762223262 | 0.078053736 |
| angptl1 | 13411 | PDI_H | 0.001597038 | 0.000906697 | 1.761380108 | 0.078196894 |
| epcam | 13647 | PDI_H | 0.001592053 | 0.000904813 | 1.759537852 | 0.07850862 |
| alpi | 11411 | PDI_H | 0.001766296 | 0.001003842 | 1.759536327 | 0.078513278 |
| chrdl2 | 13618 | PDI_H | 0.001581812 | 0.000899834 | 1.75789249 | 0.078788287 |
| anxa3 | 13767 | PDI_H | -0.001607103 | 0.000914246 | -1.757845848 | 0.078795982 |
| lrp11 | 13618 | PDI_H | -0.001556738 | 0.00088602 | -1.756999989 | 0.078940301 |
| bcam | 13632 | PDI_H | 0.001590831 | 0.000905549 | 1.756759087 | 0.07898135 |
| gpr15l | 11724 | PDI_H | -0.001651095 | 0.000940074 | -1.756346415 | 0.079055409 |
| plin3 | 13618 | PDI_H | -0.001601475 | 0.000915047 | -1.750156233 | 0.080113898 |
| bmp4 | 13526 | PDI_H | 0.001599323 | 0.000913925 | 1.749949804 | 0.08014967 |
| btn3a2 | 13621 | PDI_H | -0.001584033 | 0.000905256 | -1.749817758 | 0.080172302 |
| tpsab1 | 13779 | PDI_H | -0.001585863 | 0.000907943 | -1.746654933 | 0.080719513 |
| stk11 | 13388 | PDI_H | -0.001613128 | 0.000923591 | -1.746582085 | 0.08073281 |
| thtpa | 11716 | PDI_H | -0.001736084 | 0.000994805 | -1.745149726 | 0.080985053 |
| atxn3 | 11767 | PDI_H | -0.001714929 | 0.000985029 | -1.740992305 | 0.08171109 |
| proc | 13669 | PDI_H | -0.001572423 | 0.000903341 | -1.740675631 | 0.081762974 |
| actn4 | 13378 | PDI_H | -0.001619142 | 0.000930396 | -1.740272971 | 0.081834112 |
| baiap2 | 13308 | PDI_H | -0.001548588 | 0.000890311 | -1.739378011 | 0.081991435 |
| mtpn | 13388 | PDI_H | 0.001616989 | 0.00093052 | 1.737725979 | 0.082282124 |
| glod4 | 13627 | PDI_H | -0.001590218 | 0.000915373 | -1.737234156 | 0.082368463 |
| cd300lg | 13632 | PDI_H | -0.001529181 | 0.000880246 | -1.737220828 | 0.082370806 |
| cela2a | 11790 | PDI_H | -0.001704419 | 0.0009813 | -1.736898249 | 0.082431277 |
| krt19 | 13604 | PDI_H | -0.001558079 | 0.000898311 | -1.734454486 | 0.082860146 |
| rgcc | 11757 | PDI_H | -0.001721874 | 0.000993299 | -1.733490703 | 0.083034732 |
| clec4a | 13621 | PDI_H | 0.001562044 | 0.00090189 | 1.731968395 | 0.083301849 |
| mmp15 | 11781 | PDI_H | -0.001716275 | 0.000992717 | -1.728866528 | 0.083859211 |
| crybb2 | 11767 | PDI_H | -0.001685301 | 0.000974926 | -1.728644569 | 0.083898986 |
| camkk1 | 13308 | PDI_H | -0.001603715 | 0.000928345 | -1.727498426 | 0.08410141 |
| gnas | 11757 | PDI_H | -0.001697852 | 0.000983326 | -1.726642765 | 0.084258136 |
| sost | 13599 | PDI_H | -0.001465968 | 0.000849784 | -1.72510742 | 0.084530855 |
| ptprr | 11767 | PDI_H | -0.00166921 | 0.000967619 | -1.7250686 | 0.084541395 |
| dnajc21 | 11767 | PDI_H | -0.001714295 | 0.000994176 | -1.724338254 | 0.084673087 |
| nacc1 | 11730 | PDI_H | -0.001702543 | 0.000988068 | -1.723102898 | 0.0848963 |
| tnfsf12 | 13801 | PDI_H | 0.001560464 | 0.000905954 | 1.722454348 | 0.085009669 |
| ccn1 | 13561 | PDI_H | 0.00155151 | 0.000902063 | 1.719958254 | 0.085462866 |
| comp | 13489 | PDI_H | 0.001503392 | 0.000874366 | 1.719408246 | 0.085563023 |
| eri1 | 11570 | PDI_H | -0.001704036 | 0.000991143 | -1.719262333 | 0.08559339 |
| gclm | 11355 | PDI_H | -0.001734168 | 0.001008982 | -1.718730454 | 0.08569075 |
| yy1 | 11411 | PDI_H | 0.001732724 | 0.001008245 | 1.718554645 | 0.08572265 |
| gadd45gip1 | 11474 | PDI_H | -0.00172273 | 0.001003899 | -1.716039546 | 0.086181827 |
| ssb | 13320 | PDI_H | -0.001603176 | 0.00093431 | -1.715893652 | 0.086204783 |
| bambi | 13308 | PDI_H | -0.001573607 | 0.000917415 | -1.71526169 | 0.086320558 |
| irak4 | 13621 | PDI_H | -0.001579235 | 0.000920728 | -1.715202989 | 0.086330781 |
| tymp | 13555 | PDI_H | -0.001582784 | 0.000923295 | -1.7142775 | 0.086500654 |
| txndc5 | 13784 | PDI_H | -0.001559648 | 0.000910804 | -1.712385804 | 0.086848104 |
| aifm1 | 13560 | PDI_H | -0.00155332 | 0.000907807 | -1.711067413 | 0.08709156 |
| ccl11 | 13801 | PDI_H | -0.001483322 | 0.00086711 | -1.710650034 | 0.08716823 |
| adamtsl4 | 11474 | PDI_H | -0.001668392 | 0.000976392 | -1.708731182 | 0.087527828 |
| ciapin1 | 13699 | PDI_H | -0.001564886 | 0.000916278 | -1.707873167 | 0.087682556 |
| ube2l6 | 11781 | PDI_H | -0.001679544 | 0.000983533 | -1.707663974 | 0.087725084 |
| gfer | 13308 | PDI_H | -0.001556225 | 0.000912945 | -1.70462024 | 0.088288636 |
| cep20 | 13308 | PDI_H | -0.001574752 | 0.000925877 | -1.700822166 | 0.088999769 |
| ca3 | 13785 | PDI_H | -0.001501343 | 0.000883463 | -1.699385048 | 0.089269239 |
| c1rl | 11786 | PDI_H | -0.001585596 | 0.000936656 | -1.692827589 | 0.090514745 |
| araf | 11570 | PDI_H | -0.001696911 | 0.001002773 | -1.692218618 | 0.090631252 |
| tnfrsf11b | 13658 | PDI_H | -0.001399551 | 0.000828427 | -1.689408074 | 0.091164099 |
| nfu1 | 11724 | PDI_H | -0.001678961 | 0.000993958 | -1.689166663 | 0.091214113 |

| Protein | N_used | term | beta | se | t | p_val |
|---|---|---|---|---|---|---|
| ace2 | 13388 | PDI_H | -0.001348533 | 0.000799977 | -1.685714487 | 0.091874129 |
| snx9 | 13618 | PDI_H | -0.001552664 | 0.000921211 | -1.685459233 | 0.091922935 |
| ttr | 11671 | PDI_H | 0.001570349 | 0.000932033 | 1.684864593 | 0.092041463 |
| entr1 | 11474 | PDI_H | -0.001651634 | 0.000980419 | -1.684621077 | 0.092088927 |
| igfbp1 | 13785 | PDI_H | -0.001385231 | 0.000823599 | -1.681924242 | 0.092606201 |
| igf2r | 13632 | PDI_H | -0.00148825 | 0.000885313 | -1.681042746 | 0.092777541 |
| mycbp2 | 11757 | PDI_H | -0.00166579 | 0.000992459 | -1.678446525 | 0.093286565 |
| tfpi | 13590 | PDI_H | -0.001495662 | 0.000891988 | -1.676774159 | 0.093609672 |
| ccl8 | 13736 | PDI_H | -0.001520708 | 0.000907875 | -1.675019013 | 0.093953284 |
| muc13 | 13320 | PDI_H | -0.001530324 | 0.000915553 | -1.67147431 | 0.09465154 |
| plau | 13632 | PDI_H | 0.001501454 | 0.000898491 | 1.671083207 | 0.094728218 |
| crlf1 | 13710 | PDI_H | 0.001455746 | 0.00087114 | 1.671081263 | 0.094728471 |
| rnase3 | 13543 | PDI_H | -0.001528576 | 0.000915324 | -1.669983692 | 0.09494572 |
| ids | 13801 | PDI_H | 0.001509004 | 0.000903643 | 1.669912554 | 0.094959364 |
| cd5l | 11740 | PDI_H | -0.001647341 | 0.000987007 | -1.669027452 | 0.09513863 |
| pdcd1 | 13735 | PDI_H | -0.001509072 | 0.000904952 | -1.667571172 | 0.095423702 |
| txndc15 | 13699 | PDI_H | -0.001523995 | 0.000914558 | -1.666373098 | 0.095662007 |
| tnfaip8 | 13378 | PDI_H | -0.001549901 | 0.000931126 | -1.66454455 | 0.096027095 |
| sprr3 | 11767 | PDI_H | -0.001532593 | 0.000920889 | -1.664253359 | 0.096088462 |
| mvk | 13378 | PDI_H | -0.0014855 | 0.000893429 | -1.662694773 | 0.096396996 |
| bche | 11548 | PDI_H | -0.001585638 | 0.000955381 | -1.659691112 | 0.097003789 |
| axin1 | 13621 | PDI_H | -0.00152205 | 0.00091794 | -1.658115186 | 0.097317266 |
| pcbp2 | 11757 | PDI_H | -0.001643137 | 0.00099222 | -1.656021208 | 0.097744238 |
| atg16l1 | 11724 | PDI_H | -0.001645213 | 0.000993552 | -1.655890355 | 0.097770815 |
| clec5a | 13568 | PDI_H | -0.001460229 | 0.000883007 | -1.653700273 | 0.098211581 |
| gabarap | 11730 | PDI_H | -0.001636332 | 0.000989755 | -1.653270397 | 0.098302632 |
| gart | 11757 | PDI_H | 0.001635886 | 0.000989668 | 1.652964984 | 0.098364717 |
| mtss2 | 11730 | PDI_H | -0.001638391 | 0.000992156 | -1.65134427 | 0.098695093 |
| tgfb1 | 13779 | PDI_H | -0.001476342 | 0.000894398 | -1.650653903 | 0.098832076 |
| itga5 | 13632 | PDI_H | -0.001410182 | 0.000855085 | -1.649172522 | 0.099135357 |
| cd7 | 11388 | PDI_H | -0.001633751 | 0.000990801 | -1.64891914 | 0.099191813 |
| foxo3 | 13308 | PDI_H | -0.001531211 | 0.00093061 | -1.645384911 | 0.099914097 |
| txn | 11600 | PDI_H | -0.001630164 | 0.00099074 | -1.645400372 | 0.099914394 |
| susd5 | 11459 | PDI_H | 0.001478264 | 0.000899295 | 1.643802737 | 0.100244418 |
| enpep | 11355 | PDI_H | 0.00166318 | 0.001012012 | 1.643437841 | 0.100320091 |
| nfatc3 | 13378 | PDI_H | -0.001512174 | 0.000920353 | -1.643037374 | 0.100398723 |
| anpep | 13671 | PDI_H | -0.001501651 | 0.000914443 | -1.642147609 | 0.100582437 |
| brk1 | 13320 | PDI_H | -0.001515375 | 0.000922917 | -1.64194157 | 0.100625742 |
| hgs | 13308 | PDI_H | -0.001525935 | 0.000929442 | -1.641775224 | 0.100660245 |
| cd248 | 11790 | PDI_H | 0.001558666 | 0.000949764 | 1.641109172 | 0.100801456 |
| pear1 | 13800 | PDI_H | 0.001500454 | 0.000914417 | 1.64088624 | 0.100843841 |
| s100a11 | 13514 | PDI_H | -0.001468365 | 0.000895423 | -1.639856229 | 0.101058359 |
| sell | 11817 | PDI_H | 0.001568689 | 0.000956641 | 1.639788619 | 0.10107577 |
| dnmbp | 13552 | PDI_H | -0.001499629 | 0.000914616 | -1.639626665 | 0.101106046 |
| c1r | 11817 | PDI_H | -0.001586079 | 0.000968831 | -1.637105611 | 0.101635059 |
| kir2dl3 | 13308 | PDI_H | -0.00150908 | 0.000921954 | -1.636828633 | 0.101689948 |
| gja8 | 11304 | PDI_H | -0.001659811 | 0.001014919 | -1.635412195 | 0.101990539 |
| pkd2 | 11355 | PDI_H | -0.001632281 | 0.000998159 | -1.635291739 | 0.102015651 |
| fcgr2a | 13572 | PDI_H | -0.001484901 | 0.000908219 | -1.634959948 | 0.102080655 |
| csf3r | 11411 | PDI_H | -0.001615715 | 0.000988568 | -1.634398822 | 0.102202749 |
| sort1 | 13599 | PDI_H | -0.001484983 | 0.000909469 | -1.632802479 | 0.102533717 |
| odam | 13301 | PDI_H | 0.001499163 | 0.000918244 | 1.632640467 | 0.102568327 |
| cradd | 13535 | PDI_H | -0.001508123 | 0.000923894 | -1.632354692 | 0.102628071 |
| mydgf | 11781 | PDI_H | -0.001610355 | 0.000987117 | -1.631373014 | 0.102838392 |
| wasl | 11411 | PDI_H | 0.001634383 | 0.001003304 | 1.628999889 | 0.103340675 |
| gbp2 | 13378 | PDI_H | -0.001511101 | 0.000928789 | -1.626958046 | 0.103769581 |
| mamdc4 | 11730 | PDI_H | -0.001552276 | 0.0009546 | -1.626099998 | 0.103955274 |
| sugt1 | 13308 | PDI_H | -0.001515829 | 0.000932219 | -1.626043424 | 0.103964117 |
| igsf8 | 13618 | PDI_H | -0.001466336 | 0.000901949 | -1.625740444 | 0.104028042 |
| stx4 | 13579 | PDI_H | -0.001482577 | 0.000912949 | -1.623942531 | 0.104411305 |
| afm | 11817 | PDI_H | -0.001481784 | 0.000912993 | -1.622996192 | 0.10461692 |
| gcg | 13308 | PDI_H | 0.001481082 | 0.000912627 | 1.622877607 | 0.104639279 |
| cpe | 13649 | PDI_H | 0.001480139 | 0.000912518 | 1.62203738 | 0.104818461 |
| rab3gap1 | 11383 | PDI_H | 0.00163961 | 0.001011166 | 1.62150361 | 0.104937402 |
| tnfrsf10c | 13677 | PDI_H | -0.001479253 | 0.000912878 | -1.620428715 | 0.105163289 |
| prkab1 | 13378 | PDI_H | -0.001501267 | 0.000927737 | -1.618202939 | 0.105642465 |
| tnr | 13552 | PDI_H | 0.001426524 | 0.000881759 | 1.617816958 | 0.105725344 |
| sugp1 | 11724 | PDI_H | -0.001607607 | 0.000993783 | -1.617664846 | 0.105761775 |
| tlr2 | 11570 | PDI_H | -0.001619969 | 0.001001767 | -1.617111824 | 0.105881435 |
| layn | 13535 | PDI_H | -0.001401806 | 0.000867535 | -1.615849515 | 0.10615018 |

| Protein | N_used | term | beta | se | t | p_val |
|---|---|---|---|---|---|---|
| clec4m | 11775 | PDI_H | 0.001594109 | 0.000986754 | 1.615507818 | 0.106227587 |
| cd300c | 13784 | PDI_H | -0.001428174 | 0.000885332 | -1.613150574 | 0.10673471 |
| oga | 11724 | PDI_H | -0.001604023 | 0.000994335 | -1.613161984 | 0.106736262 |
| pglyrp1 | 13727 | PDI_H | -0.001438298 | 0.000892045 | -1.612360215 | 0.106906586 |
| slamf1 | 13378 | PDI_H | -0.001484708 | 0.000921326 | -1.611490664 | 0.107096432 |
| rad51 | 11570 | PDI_H | -0.001611757 | 0.001000596 | -1.610797057 | 0.107251269 |
| jam2 | 13784 | PDI_H | -0.001403538 | 0.000872579 | -1.608493116 | 0.107750144 |
| pappa | 13378 | PDI_H | -0.0014268 | 0.000887376 | -1.607886589 | 0.107883639 |
| nit1 | 11474 | PDI_H | -0.001602766 | 0.000997392 | -1.606956131 | 0.108091537 |
| bglap | 11790 | PDI_H | -0.001564716 | 0.000974538 | -1.605598137 | 0.108389044 |
| glb1 | 13552 | PDI_H | -0.001421078 | 0.000885182 | -1.605407263 | 0.108427528 |
| tnfsf13b | 13773 | PDI_H | -0.001393454 | 0.000868172 | -1.605044887 | 0.108506874 |
| gfra3 | 13522 | PDI_H | 0.001426563 | 0.000890532 | 1.601922931 | 0.109196032 |
| ist1 | 11635 | PDI_H | -0.001598047 | 0.000997612 | -1.601872528 | 0.109210975 |
| ndufs6 | 13308 | PDI_H | -0.001477934 | 0.000922723 | -1.601708959 | 0.109243739 |
| kifbp | 13579 | PDI_H | -0.001477129 | 0.000923042 | -1.600284156 | 0.109558844 |
| f11 | 11624 | PDI_H | 0.001546898 | 0.000967623 | 1.598657444 | 0.109923946 |
| kiaa0319 | 11767 | PDI_H | -0.001533766 | 0.000960146 | -1.59742995 | 0.11019678 |
| blmh | 13800 | PDI_H | -0.00143585 | 0.00089919 | -1.596825792 | 0.110327462 |
| impact | 11767 | PDI_H | -0.001574176 | 0.000987138 | -1.594686166 | 0.110809317 |
| becn1 | 11474 | PDI_H | -0.001585992 | 0.000996576 | -1.591441505 | 0.111537827 |
| tslp | 13388 | PDI_H | -0.001477747 | 0.000930745 | -1.587702991 | 0.112377152 |
| il1rl2 | 13378 | PDI_H | 0.001408776 | 0.000888237 | 1.586036804 | 0.112754617 |
| ces2 | 13579 | PDI_H | -0.001407699 | 0.000887891 | -1.585441606 | 0.112889341 |
| cd34 | 13744 | PDI_H | 0.001438511 | 0.000907469 | 1.585190997 | 0.112945972 |
| rcc1 | 11730 | PDI_H | -0.001563312 | 0.000986873 | -1.584107169 | 0.113196319 |
| vsig2 | 11730 | PDI_H | -0.0015026 | 0.000949253 | -1.582929456 | 0.113464531 |
| vash1 | 13621 | PDI_H | -0.001457241 | 0.000921076 | -1.582107059 | 0.113648371 |
| usp8 | 13388 | PDI_H | -0.001460556 | 0.000923699 | -1.581203026 | 0.113855268 |
| tdp1 | 11730 | PDI_H | -0.001545452 | 0.000977725 | -1.58066242 | 0.113982235 |
| ca4 | 13800 | PDI_H | -0.00142054 | 0.000899228 | -1.579732752 | 0.11419102 |
| ptgds | 13531 | PDI_H | -0.001349313 | 0.000854155 | -1.57970602 | 0.114197602 |
| gatd3 | 11474 | PDI_H | -0.001581898 | 0.001001843 | -1.578987064 | 0.114366619 |
| eppk1 | 11474 | PDI_H | -0.001583492 | 0.001002977 | -1.578791435 | 0.114411499 |
| gmpr2 | 11537 | PDI_H | -0.001577499 | 0.000999184 | -1.578787289 | 0.114412299 |
| il7r | 13632 | PDI_H | 0.001422005 | 0.00090105 | 1.578163862 | 0.114551193 |
| il20rb | 11411 | PDI_H | 0.001591033 | 0.001008146 | 1.57817774 | 0.114552531 |
| pon1 | 11794 | PDI_H | -0.001535448 | 0.000973034 | -1.578001623 | 0.114592083 |
| pcyt2 | 11724 | PDI_H | -0.001562381 | 0.000992441 | -1.574280396 | 0.11544965 |
| creld1 | 11537 | PDI_H | -0.001474878 | 0.000936894 | -1.574221282 | 0.11546375 |
| cdc37 | 13699 | PDI_H | -0.001444391 | 0.000919779 | -1.570366849 | 0.116352906 |
| ptpn1 | 13552 | PDI_H | -0.001444121 | 0.000921458 | -1.567212254 | 0.117088456 |
| hcls1 | 13801 | PDI_H | -0.001426857 | 0.000911154 | -1.565989732 | 0.117373968 |
| plpbp | 13618 | PDI_H | -0.001442486 | 0.000921535 | -1.565307544 | 0.117534071 |
| aamdc | 11474 | PDI_H | -0.001569529 | 0.001002687 | -1.565323619 | 0.117534654 |
| ccl28 | 13587 | PDI_H | -0.001416317 | 0.000905213 | -1.564622851 | 0.117694677 |
| col1a1 | 13800 | PDI_H | 0.001424133 | 0.000910704 | 1.563770609 | 0.117894399 |
| ncam2 | 13632 | PDI_H | 0.001378011 | 0.000881402 | 1.563430821 | 0.117974528 |
| lmnb1 | 11689 | PDI_H | -0.001532423 | 0.000981844 | -1.56076074 | 0.118607314 |
| l3hypdh | 11781 | PDI_H | -0.00154471 | 0.000990084 | -1.560181175 | 0.118743961 |
| lyar | 13308 | PDI_H | -0.001452162 | 0.000931098 | -1.559623225 | 0.118872745 |
| lrp2 | 11483 | PDI_H | 0.001571413 | 0.00100888 | 1.557581989 | 0.119359958 |
| itgb6 | 13378 | PDI_H | 0.001350191 | 0.000867002 | 1.557310206 | 0.119420529 |
| nell2 | 13710 | PDI_H | -0.001381259 | 0.00088699 | -1.557243183 | 0.119435861 |
| ubxn1 | 11537 | PDI_H | -0.001551167 | 0.00099721 | -1.555506906 | 0.119852849 |
| ppme1 | 13579 | PDI_H | -0.001426862 | 0.000918513 | -1.553447356 | 0.120339622 |
| meltf | 11781 | PDI_H | 0.001515166 | 0.000975901 | 1.552581866 | 0.120549949 |
| rnf41 | 13308 | PDI_H | -0.001436196 | 0.000926488 | -1.550150415 | 0.121129219 |
| lypd8 | 13308 | PDI_H | -0.001438059 | 0.000928636 | -1.548570524 | 0.121508797 |
| fis1 | 13646 | PDI_H | -0.001424178 | 0.000919997 | -1.548024502 | 0.121639608 |
| eng | 13800 | PDI_H | 0.00139944 | 0.000904264 | 1.54760142 | 0.12174124 |
| bcat2 | 11383 | PDI_H | 0.001557268 | 0.001009203 | 1.543067184 | 0.122842303 |
| qdpr | 13547 | PDI_H | -0.001408575 | 0.000912859 | -1.543037322 | 0.122845099 |
| zfyve19 | 11724 | PDI_H | -0.001534949 | 0.000994945 | -1.542748097 | 0.122918924 |
| sirt5 | 13320 | PDI_H | -0.001439288 | 0.000934224 | -1.540623541 | 0.123432211 |
| rnf4 | 11570 | PDI_H | -0.00154289 | 0.001003189 | -1.53798531 | 0.124079581 |
| pcsk7 | 11730 | PDI_H | 0.001524911 | 0.000992099 | 1.537055008 | 0.124306839 |
| myl3 | 11474 | PDI_H | 0.001421968 | 0.000927616 | 1.532926574 | 0.125321546 |
| il1b | 13324 | PDI_H | -0.001422326 | 0.000929176 | -1.530738883 | 0.125857702 |
| lrrc25 | 13579 | PDI_H | -0.001357686 | 0.000888245 | -1.528503771 | 0.126410821 |

| Protein | N_used | term | beta | se | t | p_val |
|---|---|---|---|---|---|---|
| cirbp | 11724 | PDI_H | -0.001519792 | 0.000994469 | -1.528245153 | 0.126478687 |
| orm1 | 11768 | PDI_H | -0.001455499 | 0.000953694 | -1.526170056 | 0.12699442 |
| tnpo1 | 11570 | PDI_H | 0.001529266 | 0.001003473 | 1.523972958 | 0.127542826 |
| siglec9 | 13736 | PDI_H | 0.001377936 | 0.000905293 | 1.522088034 | 0.128010063 |
| nptx2 | 11790 | PDI_H | -0.001279369 | 0.000840809 | -1.521593109 | 0.128137911 |
| ccn5 | 13632 | PDI_H | -0.001201462 | 0.000789771 | -1.521278531 | 0.12821317 |
| dapp1 | 13621 | PDI_H | -0.001402037 | 0.000922469 | -1.519875062 | 0.128565611 |
| mrpl58 | 11757 | PDI_H | -0.001508044 | 0.00099246 | -1.519500603 | 0.128663454 |
| golga3 | 11757 | PDI_H | -0.001505815 | 0.000991459 | -1.518785993 | 0.128843289 |
| erbin | 13579 | PDI_H | -0.001401191 | 0.0009229 | -1.51824888 | 0.12897497 |
| arsa | 13632 | PDI_H | -0.001347278 | 0.000888271 | -1.516741962 | 0.129355053 |
| pydc1 | 11474 | PDI_H | -0.001490043 | 0.000982389 | -1.516754692 | 0.129356208 |
| jpt2 | 11724 | PDI_H | -0.001508709 | 0.000995758 | -1.515136108 | 0.129764925 |
| scrib | 11474 | PDI_H | -0.001514152 | 0.001000144 | -1.513933113 | 0.130070377 |
| inppl1 | 13579 | PDI_H | -0.001395473 | 0.000922257 | -1.513107581 | 0.130275628 |
| yju2 | 11730 | PDI_H | -0.00150177 | 0.000993126 | -1.512164356 | 0.130519023 |
| ctbs | 11616 | PDI_H | 0.001457076 | 0.000963772 | 1.511847497 | 0.130599894 |
| cd200r1 | 13588 | PDI_H | 0.001385016 | 0.000916197 | 1.51170125 | 0.130633156 |
| unc79 | 11304 | PDI_H | -0.001531656 | 0.001014717 | -1.509441317 | 0.13121402 |
| cbln1 | 11383 | PDI_H | 0.001524438 | 0.001010246 | 1.508976709 | 0.131332518 |
| cd276 | 13658 | PDI_H | -0.001346245 | 0.000892973 | -1.507598033 | 0.131680586 |
| ltbp2 | 13800 | PDI_H | -0.001165359 | 0.00077319 | -1.50720952 | 0.131779871 |
| il17d | 13378 | PDI_H | -0.001284913 | 0.000853028 | -1.506296709 | 0.132014654 |
| fcn2 | 13572 | PDI_H | 0.001362958 | 0.000906912 | 1.502855386 | 0.132899627 |
| dxo | 11767 | PDI_H | -0.001490015 | 0.000991894 | -1.502192239 | 0.13307433 |
| flrt2 | 13552 | PDI_H | 0.001370777 | 0.000912931 | 1.501512309 | 0.133246424 |
| cilp | 11790 | PDI_H | -0.001392608 | 0.000927545 | -1.501390639 | 0.133281361 |
| thy1 | 13784 | PDI_H | -0.001274186 | 0.000850081 | -1.498899907 | 0.133922521 |
| aif1 | 13308 | PDI_H | -0.001390323 | 0.000927942 | -1.498285744 | 0.134082766 |
| khk | 11474 | PDI_H | -0.001447705 | 0.000966232 | -1.498299145 | 0.134083084 |
| dut | 11724 | PDI_H | -0.001471644 | 0.000984346 | -1.495048199 | 0.134928806 |
| dcdc2c | 11304 | PDI_H | -0.001517984 | 0.00101545 | -1.49488856 | 0.134971472 |
| galnt5 | 11781 | PDI_H | 0.001456613 | 0.000974794 | 1.494277659 | 0.135129872 |
| aspscr1 | 11757 | PDI_H | -0.001475572 | 0.000987701 | -1.493945635 | 0.135216693 |
| dnajb1 | 13579 | PDI_H | -0.001362712 | 0.000912825 | -1.492850846 | 0.135499488 |
| scg3 | 13677 | PDI_H | -0.001315667 | 0.000881769 | -1.492076262 | 0.135702239 |
| ccnd2 | 11570 | PDI_H | 0.001491106 | 0.000999944 | 1.491189925 | 0.135938929 |
| nfatc1 | 13621 | PDI_H | -0.001366825 | 0.000916611 | -1.491173587 | 0.135939104 |
| bloc1s2 | 11383 | PDI_H | -0.001506819 | 0.001010504 | -1.491155569 | 0.135948395 |
| fam13a | 11724 | PDI_H | -0.00147749 | 0.000991864 | -1.489609142 | 0.136353964 |
| gpi | 11794 | PDI_H | -0.001468512 | 0.000986236 | -1.489006149 | 0.136512515 |
| ache | 11537 | PDI_H | 0.001420468 | 0.000954158 | 1.488714025 | 0.136590052 |
| psmg3 | 13621 | PDI_H | -0.001338354 | 0.000899988 | -1.487079873 | 0.137016884 |
| jchain | 13378 | PDI_H | -0.001373508 | 0.000923706 | -1.486953402 | 0.137050707 |
| cd14 | 13353 | PDI_H | -0.001338205 | 0.000900259 | -1.486466353 | 0.137179442 |
| cfhr5 | 11612 | PDI_H | -0.001458727 | 0.000983147 | -1.483732411 | 0.137907093 |
| f3 | 13674 | PDI_H | 0.001296018 | 0.000873714 | 1.483344341 | 0.138006015 |
| cacybp | 11517 | PDI_H | -0.001488468 | 0.001003475 | -1.483313641 | 0.138018492 |
| smarca2 | 13320 | PDI_H | -0.001381742 | 0.000932406 | -1.481909848 | 0.138387962 |
| ahsa1 | 11730 | PDI_H | -0.001474393 | 0.000995077 | -1.481687292 | 0.138450409 |
| ocln | 11757 | PDI_H | -0.001378955 | 0.000931202 | -1.480833429 | 0.138677791 |
| chrdl1 | 13691 | PDI_H | -0.001198175 | 0.000809325 | -1.480461841 | 0.138773068 |
| ccl23 | 13710 | PDI_H | -0.001313755 | 0.000887736 | -1.479894527 | 0.138924394 |
| gkn1 | 13075 | PDI_H | -0.001377302 | 0.000930833 | -1.479644784 | 0.138992183 |
| fgf2 | 13621 | PDI_H | -0.001358724 | 0.000920571 | -1.475957317 | 0.139978487 |
| dusp3 | 13320 | PDI_H | -0.001378282 | 0.000934242 | -1.475293858 | 0.140157214 |
| txnl1 | 11363 | PDI_H | -0.001489259 | 0.001009724 | -1.474916455 | 0.140262741 |
| slc28a1 | 11304 | PDI_H | -0.001496179 | 0.001014666 | -1.47455378 | 0.140360427 |
| il2ra | 13531 | PDI_H | -0.001320457 | 0.000897218 | -1.471723919 | 0.141118723 |
| insl3 | 11767 | PDI_H | -0.000907909 | 0.000616931 | -1.4716529 | 0.141141402 |
| ppp1r12a | 13699 | PDI_H | -0.001349438 | 0.00091701 | -1.471563294 | 0.141161835 |
| rab6a | 13621 | PDI_H | 0.001323655 | 0.000899492 | 1.471558703 | 0.141163207 |
| casc3 | 11483 | PDI_H | -0.001474629 | 0.0010027 | -1.470657975 | 0.141411066 |
| skiv2l | 11355 | PDI_H | -0.001486946 | 0.001012354 | -1.468801084 | 0.141914481 |
| tnfsf14 | 13552 | PDI_H | -0.001338984 | 0.000911771 | -1.468553109 | 0.141977272 |
| adgre1 | 11724 | PDI_H | -0.001431226 | 0.00097493 | -1.468029874 | 0.142122963 |
| ctsc | 13619 | PDI_H | 0.001318044 | 0.00089786 | 1.467984067 | 0.142131665 |
| nub1 | 13621 | PDI_H | -0.0013511 | 0.000920802 | -1.467308797 | 0.14231518 |
| adgre5 | 13727 | PDI_H | 0.001281703 | 0.000873651 | 1.467064704 | 0.142381384 |
| clpp | 13535 | PDI_H | -0.00135016 | 0.000920772 | -1.466335048 | 0.142580284 |

| Protein | N_used | term | beta | se | t | p_val |
|---|---|---|---|---|---|---|
| sdk2 | 11537 | PDI_H | 0.00144881 | 0.000988299 | 1.465962786 | 0.142685707 |
| clec14a | 13632 | PDI_H | -0.00129342 | 0.000882595 | -1.465473652 | 0.14281482 |
| tank | 13378 | PDI_H | -0.00136028 | 0.000928508 | -1.465017356 | 0.142939704 |
| ca1 | 13352 | PDI_H | -0.00132882 | 0.000908677 | -1.462366807 | 0.143664278 |
| atox1 | 13699 | PDI_H | -0.001336373 | 0.00091398 | -1.4621469 | 0.143723918 |
| cbs | 11474 | PDI_H | -0.001432831 | 0.000980241 | -1.461713408 | 0.143847176 |
| fxyd5 | 13378 | PDI_H | -0.001358268 | 0.000930933 | -1.459039848 | 0.14457764 |
| kdr | 13699 | PDI_H | 0.001320156 | 0.000906577 | 1.456198747 | 0.145360623 |
| klrc1 | 11383 | PDI_H | 0.001470969 | 0.001011229 | 1.454634737 | 0.145798011 |
| il22 | 11730 | PDI_H | -0.001378574 | 0.00094885 | -1.452889925 | 0.146281096 |
| gpc1 | 13753 | PDI_H | 0.001295273 | 0.000891532 | 1.452862338 | 0.146284814 |
| vti1a | 11537 | PDI_H | -0.001450642 | 0.0009985 | -1.452822264 | 0.146300334 |
| cd72 | 11537 | PDI_H | -0.001379824 | 0.000950022 | -1.452411928 | 0.146414325 |
| hars1 | 13632 | PDI_H | -0.001334799 | 0.00091966 | -1.451404693 | 0.146690236 |
| commd1 | 11474 | PDI_H | -0.001454606 | 0.001003679 | -1.449274848 | 0.1472882 |
| fhit | 13320 | PDI_H | -0.001348277 | 0.000931675 | -1.44715492 | 0.147877093 |
| c19orf12 | 13320 | PDI_H | -0.001277121 | 0.000883328 | -1.445805386 | 0.148255344 |
| ubqln3 | 11355 | PDI_H | -0.001463219 | 0.001012051 | -1.445796081 | 0.148262033 |
| cacna1c | 11383 | PDI_H | 0.001462226 | 0.001011582 | 1.445484462 | 0.148349412 |
| cd8a | 13784 | PDI_H | -0.001300971 | 0.000900799 | -1.444240843 | 0.148693988 |
| prdx6 | 13753 | PDI_H | 0.001324111 | 0.000917815 | 1.442676915 | 0.149134299 |
| adamts15 | 13579 | PDI_H | -0.001190813 | 0.0008272 | -1.439570964 | 0.150011892 |
| hpgds | 13308 | PDI_H | 0.001339133 | 0.000930499 | 1.439155345 | 0.150130055 |
| scp2 | 13308 | PDI_H | -0.00133419 | 0.000928315 | -1.437216921 | 0.150679898 |
| lepr | 13618 | PDI_H | -0.001243136 | 0.000865035 | -1.437093276 | 0.150714486 |
| ptprm | 13602 | PDI_H | 0.001299912 | 0.000907179 | 1.432917149 | 0.151904519 |
| dusp13 | 11730 | PDI_H | -0.001402451 | 0.000978753 | -1.432896348 | 0.151914138 |
| rwdd1 | 13784 | PDI_H | -0.001308165 | 0.000913025 | -1.432780775 | 0.151943195 |
| igf2bp3 | 11355 | PDI_H | -0.00144777 | 0.001011164 | -1.431786092 | 0.152232602 |
| spry2 | 13378 | PDI_H | -0.001329724 | 0.000929262 | -1.430946644 | 0.152468886 |
| hpse | 11619 | PDI_H | -0.00142265 | 0.000994374 | -1.430698067 | 0.152543688 |
| hmbs | 13753 | PDI_H | -0.001288079 | 0.000901652 | -1.428575789 | 0.153148926 |
| prc1 | 11730 | PDI_H | 0.001422844 | 0.000996343 | 1.428067143 | 0.153299184 |
| hsbp1 | 11474 | PDI_H | -0.001435177 | 0.001006158 | -1.426392659 | 0.153782268 |
| ak1 | 13618 | PDI_H | -0.001287173 | 0.000902926 | -1.425558111 | 0.154018875 |
| spart | 11537 | PDI_H | -0.001427654 | 0.001002279 | -1.424408052 | 0.15435546 |
| ppp1r14a | 11757 | PDI_H | -0.001414689 | 0.000993592 | -1.423812301 | 0.154527379 |
| il17ra | 13632 | PDI_H | -0.001311946 | 0.000921457 | -1.423773954 | 0.154534825 |
| chac2 | 13308 | PDI_H | -0.001320258 | 0.000927949 | -1.422770335 | 0.154826204 |
| mmp7 | 13373 | PDI_H | -0.001265288 | 0.000889654 | -1.422224306 | 0.154984489 |
| lat2 | 13579 | PDI_H | -0.001308549 | 0.000920094 | -1.422190531 | 0.154993936 |
| dcbld2 | 13699 | PDI_H | -0.001256011 | 0.000883358 | -1.421858546 | 0.155090105 |
| mtr | 11355 | PDI_H | -0.00143934 | 0.001012597 | -1.421434716 | 0.155217912 |
| scrg1 | 11781 | PDI_H | -0.001222473 | 0.000860369 | -1.420871288 | 0.155380675 |
| fgf12 | 11411 | PDI_H | -0.001430238 | 0.00100674 | -1.420662059 | 0.15544238 |
| cope | 13320 | PDI_H | -0.001327681 | 0.000934984 | -1.420004199 | 0.155629884 |
| pcdh12 | 11757 | PDI_H | 0.001410421 | 0.000994216 | 1.418625502 | 0.156034761 |
| agbl2 | 11383 | PDI_H | 0.001434287 | 0.001011108 | 1.41852957 | 0.156063618 |
| vwa1 | 13732 | PDI_H | -0.001240321 | 0.000876028 | -1.415847563 | 0.156842841 |
| anxa5 | 13286 | PDI_H | -0.00131272 | 0.000927193 | -1.415799973 | 0.15685754 |
| adgrf5 | 11781 | PDI_H | -0.001368118 | 0.000966584 | -1.415415372 | 0.156973205 |
| cnp | 11450 | PDI_H | -0.00142112 | 0.001004078 | -1.415348556 | 0.156993551 |
| pmch | 11355 | PDI_H | -0.001428669 | 0.001010086 | -1.414404213 | 0.157270701 |
| ddr1 | 13784 | PDI_H | -0.001270619 | 0.000899224 | -1.413017088 | 0.157673299 |
| gopc | 13621 | PDI_H | -0.001300343 | 0.000920897 | -1.412039919 | 0.157961072 |
| tcn1 | 11533 | PDI_H | -0.001407554 | 0.000997164 | -1.411557729 | 0.158107232 |
| nefl | 13285 | PDI_H | -0.001130511 | 0.000801635 | -1.410257424 | 0.158487119 |
| pik3ap1 | 13621 | PDI_H | -0.001297405 | 0.000920921 | -1.408812684 | 0.15891341 |
| serpinf2 | 11565 | PDI_H | 0.001364322 | 0.000969429 | 1.407345782 | 0.159351783 |
| psg1 | 13552 | PDI_H | -0.001252161 | 0.000889876 | -1.407119683 | 0.159414856 |
| angptl7 | 13753 | PDI_H | -0.001253784 | 0.000891825 | -1.405863047 | 0.159787407 |
| angptl2 | 13681 | PDI_H | -0.001209448 | 0.000860368 | -1.40573293 | 0.159826174 |
| klrd1 | 13801 | PDI_H | -0.001240116 | 0.000882281 | -1.405578937 | 0.159871725 |
| amfr | 13320 | PDI_H | -0.001307197 | 0.00093057 | -1.404726483 | 0.160125967 |
| stc2 | 13613 | PDI_H | 0.001226698 | 0.000874936 | 1.4020434 | 0.160925108 |
| kif1c | 11355 | PDI_H | -0.001387861 | 0.000990308 | -1.401444082 | 0.161108678 |
| hrg | 11740 | PDI_H | 0.001372222 | 0.000981283 | 1.39839554 | 0.162020763 |
| fcrl1 | 13618 | PDI_H | -0.001245687 | 0.000892423 | -1.39584761 | 0.162783166 |
| sil1 | 11474 | PDI_H | -0.001393269 | 0.000998394 | -1.395510536 | 0.162888974 |
| pgf | 13801 | PDI_H | -0.001093498 | 0.000784443 | -1.393979387 | 0.163346292 |

| Protein | N_used | term | beta | se | t | p_val |
|---|---|---|---|---|---|---|
| mep1b | 13618 | PDI_H | 0.001263241 | 0.000906788 | 1.393094321 | 0.163614029 |
| ppm1a | 13308 | PDI_H | -0.001300429 | 0.000933677 | -1.39280426 | 0.163702278 |
| gh1 | 13599 | PDI_H | -0.001192966 | 0.000857305 | -1.39153084 | 0.164087296 |
| eno1 | 13666 | PDI_H | -0.00127983 | 0.000919741 | -1.39151071 | 0.164093284 |
| fcrl3 | 13378 | PDI_H | -0.001288671 | 0.00092645 | -1.390977309 | 0.164255462 |
| pdia3 | 11537 | PDI_H | -0.001387455 | 0.000997548 | -1.390865636 | 0.164293027 |
| mbl2 | 11632 | PDI_H | 0.00137538 | 0.000989805 | 1.389545693 | 0.164693499 |
| fst | 13691 | PDI_H | -0.001223601 | 0.000885786 | -1.38137319 | 0.167186808 |
| amn | 13378 | PDI_H | -0.00126662 | 0.000917024 | -1.381229235 | 0.167231579 |
| dnaja4 | 11767 | PDI_H | -0.001364186 | 0.000988498 | -1.380059429 | 0.167594593 |
| cdc26 | 11698 | PDI_H | -0.001372282 | 0.00099656 | -1.37701886 | 0.168532794 |
| mri1 | 11483 | PDI_H | -0.001380324 | 0.001002534 | -1.376834804 | 0.168590197 |
| ttn | 11355 | PDI_H | -0.001372729 | 0.000998397 | -1.374932697 | 0.169179468 |
| aldh1a1 | 13632 | PDI_H | -0.001187425 | 0.000864289 | -1.373875953 | 0.169502806 |
| hddc2 | 11724 | PDI_H | -0.001366971 | 0.000995086 | -1.373721516 | 0.169554447 |
| calca | 13535 | PDI_H | -0.001005244 | 0.000735099 | -1.367495229 | 0.171492859 |
| ggact | 11781 | PDI_H | -0.001356014 | 0.000991617 | -1.367477523 | 0.171501792 |
| cntnap2 | 13621 | PDI_H | 0.00122049 | 0.000892594 | 1.36735158 | 0.171537714 |
| pfkfb2 | 13561 | PDI_H | -0.001253862 | 0.000918167 | -1.365614954 | 0.17208252 |
| tpr | 11355 | PDI_H | -0.001347314 | 0.000987975 | -1.363713081 | 0.172684947 |
| plcb1 | 11411 | PDI_H | -0.001372686 | 0.001006876 | -1.363312049 | 0.172811112 |
| mybpc1 | 11781 | PDI_H | 0.001234288 | 0.000905396 | 1.363257313 | 0.172827509 |
| sema4c | 13579 | PDI_H | 0.001232739 | 0.00090432 | 1.363166705 | 0.1728526 |
| serpini1 | 11517 | PDI_H | 0.001332354 | 0.00097804 | 1.36227025 | 0.173139281 |
| serpinb8 | 13801 | PDI_H | -0.001197868 | 0.000879825 | -1.361485524 | 0.17338255 |
| septin9 | 13579 | PDI_H | -0.001256856 | 0.000923846 | -1.360460444 | 0.173706864 |
| tomm20 | 11781 | PDI_H | -0.001337025 | 0.000983121 | -1.359980233 | 0.17386223 |
| sh2d1a | 13621 | PDI_H | -0.00123419 | 0.000908147 | -1.359020091 | 0.174162744 |
| acan | 13497 | PDI_H | 0.001166392 | 0.000858397 | 1.35880314 | 0.174231705 |
| sirpa | 13675 | PDI_H | -0.001243259 | 0.000915938 | -1.357361181 | 0.174688896 |
| zcchc8 | 11355 | PDI_H | -0.001372402 | 0.00101219 | -1.355874205 | 0.175166188 |
| susd2 | 13632 | PDI_H | -0.001187474 | 0.000875917 | -1.355691314 | 0.175219882 |
| sncg | 13552 | PDI_H | -0.001129903 | 0.000833847 | -1.355047727 | 0.175424957 |
| ngrn | 11767 | PDI_H | -0.001324679 | 0.000978999 | -1.353095743 | 0.176051067 |
| tp53 | 13308 | PDI_H | -0.001256666 | 0.000928919 | -1.352826316 | 0.176134127 |
| crym | 11383 | PDI_H | -0.001363065 | 0.001007655 | -1.352709368 | 0.176175393 |
| tarm1 | 11483 | PDI_H | 0.001363386 | 0.00100831 | 1.352150657 | 0.176353782 |
| hmgcl | 11474 | PDI_H | -0.001358123 | 0.001004934 | -1.351454696 | 0.176576494 |
| ipcef1 | 13526 | PDI_H | -0.001235313 | 0.000914506 | -1.350798373 | 0.176782646 |
| mphosph8 | 13388 | PDI_H | -0.00125255 | 0.000927968 | -1.349777466 | 0.177110217 |
| clta | 13388 | PDI_H | -0.001250886 | 0.00092692 | -1.349507332 | 0.177196906 |
| il7 | 13621 | PDI_H | -0.001242765 | 0.000921044 | -1.349300934 | 0.177262773 |
| tbca | 11537 | PDI_H | -0.001349069 | 0.001000899 | -1.347857684 | 0.177730653 |
| prrt3 | 11474 | PDI_H | -0.001297073 | 0.000962442 | -1.347689999 | 0.177784746 |
| il17f | 13378 | PDI_H | -0.001253689 | 0.000931124 | -1.34642571 | 0.178188096 |
| terf1 | 11411 | PDI_H | 0.001355437 | 0.001008327 | 1.344243291 | 0.178896478 |
| nadk | 13618 | PDI_H | -0.001195937 | 0.000889707 | -1.344192109 | 0.178908684 |
| ghrhr | 13320 | PDI_H | 0.001254949 | 0.000934675 | 1.342658494 | 0.179405481 |
| casp1 | 13320 | PDI_H | -0.001242423 | 0.000925947 | -1.3417858 | 0.179688351 |
| ap2b1 | 11483 | PDI_H | -0.001348815 | 0.001006018 | -1.3407461 | 0.180029451 |
| dtymk | 11781 | PDI_H | -0.001309634 | 0.000977866 | -1.339277907 | 0.180506082 |
| ptk7 | 13320 | PDI_H | -0.001246335 | 0.000931266 | -1.338322944 | 0.18081405 |
| dkkl1 | 13579 | PDI_H | 0.001194778 | 0.000892895 | 1.338094592 | 0.18088803 |
| cep290 | 11570 | PDI_H | -0.001342581 | 0.00100356 | -1.33781811 | 0.180982059 |
| mtdh | 11411 | PDI_H | -0.001331968 | 0.000997306 | -1.335565727 | 0.181717925 |
| mog | 13753 | PDI_H | -0.001163357 | 0.000872201 | -1.333817332 | 0.182285836 |
| dffa | 13801 | PDI_H | -0.001217491 | 0.000913201 | -1.333212746 | 0.182484023 |
| cend1 | 11383 | PDI_H | -0.001334107 | 0.00100123 | -1.332467401 | 0.182733348 |
| dpp10 | 13621 | PDI_H | 0.00118962 | 0.000893056 | 1.332078 | 0.182856866 |
| apoa2 | 11794 | PDI_H | -0.001261021 | 0.000946658 | -1.332076813 | 0.182860714 |
| mtif3 | 11724 | PDI_H | -0.001321187 | 0.000993975 | -1.3291963 | 0.183809107 |
| ap3b1 | 11724 | PDI_H | -0.001318537 | 0.0009921 | -1.329036339 | 0.183861871 |
| st13 | 11537 | PDI_H | -0.001324226 | 0.000998835 | -1.32576993 | 0.184942192 |
| hyal1 | 13566 | PDI_H | -0.001203837 | 0.000909125 | -1.324171023 | 0.185468581 |
| lypd3 | 13582 | PDI_H | -0.001207828 | 0.000912388 | -1.323810314 | 0.185588349 |
| prdx3 | 13378 | PDI_H | -0.001225275 | 0.000926396 | -1.322624535 | 0.1859829 |
| hsp90b1 | 13254 | PDI_H | -0.001237648 | 0.000936211 | -1.321974991 | 0.186199314 |
| alms1 | 11570 | PDI_H | 0.001325064 | 0.001003668 | 1.320220543 | 0.186787539 |
| atp5po | 13320 | PDI_H | -0.0012342 | 0.000934953 | -1.320066495 | 0.186835523 |
| icam3 | 13800 | PDI_H | -0.001197313 | 0.000908409 | -1.318032631 | 0.187514621 |

| Protein | N_used | term | beta | se | t | p_val |
|---|---|---|---|---|---|---|
| reg1b | 13800 | PDI_H | -0.00117784 | 0.000894319 | -1.317024342 | 0.187852353 |
| samd9l | 13621 | PDI_H | -0.001211424 | 0.00092059 | -1.315921919 | 0.188222416 |
| dnajb14 | 11724 | PDI_H | -0.001305138 | 0.00099301 | -1.314324833 | 0.188762652 |
| msra | 13699 | PDI_H | -0.00120287 | 0.000918345 | -1.309823848 | 0.190277417 |
| ptprb | 11781 | PDI_H | 0.001238546 | 0.000945729 | 1.309620528 | 0.190349805 |
| bcl2l1 | 11767 | PDI_H | -0.001300389 | 0.000994446 | -1.307651882 | 0.191016984 |
| stat5b | 13579 | PDI_H | -0.00120596 | 0.000923107 | -1.306414433 | 0.191433807 |
| cep85 | 13308 | PDI_H | -0.001210936 | 0.00092707 | -1.306197041 | 0.191508155 |
| sftpa1 | 13308 | PDI_H | -0.001207769 | 0.000924866 | -1.305884713 | 0.191614359 |
| qpct | 13644 | PDI_H | -0.001169429 | 0.000895728 | -1.305562193 | 0.191723517 |
| lama1 | 11355 | PDI_H | 0.00131658 | 0.001010594 | 1.302777871 | 0.192677055 |
| epn1 | 11570 | PDI_H | 0.001306148 | 0.001003019 | 1.302215987 | 0.192868511 |
| ctla4 | 11790 | PDI_H | -0.0012941 | 0.00099465 | -1.301060918 | 0.193263055 |
| cpa4 | 11301 | PDI_H | -0.001312328 | 0.001009912 | -1.29944862 | 0.193816551 |
| crtac1 | 13800 | PDI_H | 0.001122751 | 0.000864232 | 1.299131055 | 0.19392068 |
| cep43 | 13289 | PDI_H | -0.001203184 | 0.000926487 | -1.298652507 | 0.194085764 |
| sumf1 | 11411 | PDI_H | -0.001308699 | 0.001008078 | -1.298211693 | 0.194240866 |
| ifnl2 | 11383 | PDI_H | 0.001312511 | 0.001011135 | 1.298057108 | 0.19429404 |
| farsa | 11483 | PDI_H | -0.001302237 | 0.00100476 | -1.296067334 | 0.194978363 |
| phldb1 | 11724 | PDI_H | -0.00124479 | 0.000962083 | -1.293849185 | 0.195743034 |
| icam5 | 13618 | PDI_H | -0.001162693 | 0.000898855 | -1.293526892 | 0.195850848 |
| ceacam5 | 13118 | PDI_H | -0.001174943 | 0.000908503 | -1.293274237 | 0.195939022 |
| lelp1 | 11383 | PDI_H | 0.00130774 | 0.001011221 | 1.293228356 | 0.195958363 |
| oplah | 11474 | PDI_H | -0.001296779 | 0.001003697 | -1.292001877 | 0.196382544 |
| uso1 | 13579 | PDI_H | -0.001189032 | 0.000921832 | -1.289857181 | 0.197122236 |
| gnpda1 | 11474 | PDI_H | -0.001293151 | 0.00100298 | -1.289308421 | 0.197316902 |
| cpxm2 | 11781 | PDI_H | -0.001208456 | 0.000937342 | -1.289236229 | 0.197341311 |
| crtam | 13552 | PDI_H | -0.001166859 | 0.000906301 | -1.287496787 | 0.197943195 |
| mcee | 11757 | PDI_H | -0.0012803 | 0.000995672 | -1.285865055 | 0.198515512 |
| pga4 | 11794 | PDI_H | -0.001226485 | 0.00095487 | -1.28445303 | 0.199008742 |
| pdia4 | 11790 | PDI_H | -0.001271482 | 0.000991364 | -1.282558411 | 0.199672069 |
| per3 | 11411 | PDI_H | -0.001277412 | 0.000997683 | -1.280379226 | 0.200437849 |
| amotl2 | 11684 | PDI_H | -0.00126868 | 0.000991619 | -1.279402325 | 0.200780847 |
| itgax | 11570 | PDI_H | -0.001283241 | 0.001003097 | -1.27927962 | 0.200824288 |
| dmp1 | 11474 | PDI_H | 0.001220362 | 0.000954874 | 1.278035497 | 0.201262792 |
| lsm8 | 11570 | PDI_H | 0.001276321 | 0.000998921 | 1.277699365 | 0.201381111 |
| cdh22 | 11570 | PDI_H | -0.001280114 | 0.001002077 | -1.277460857 | 0.201465251 |
| fkbp5 | 13552 | PDI_H | -0.001179155 | 0.000923199 | -1.277248832 | 0.201536313 |
| prkra | 13308 | PDI_H | -0.001165942 | 0.000913915 | -1.275766085 | 0.202060507 |
| ifng | 13378 | PDI_H | -0.001181317 | 0.000926491 | -1.275045008 | 0.202315474 |
| defb104a_defb104b | 11383 | PDI_H | 0.001201743 | 0.00094252 | 1.275032115 | 0.202323926 |
| saa4 | 11817 | PDI_H | -0.001237126 | 0.00097029 | -1.275007264 | 0.202331764 |
| mfap5 | 13800 | PDI_H | 0.001058899 | 0.000831211 | 1.273922348 | 0.20271241 |
| gorasp2 | 11724 | PDI_H | -0.001265301 | 0.000993526 | -1.273545879 | 0.202849677 |
| adam9 | 11724 | PDI_H | -0.001234501 | 0.000971964 | -1.270109864 | 0.204070731 |
| dag1 | 13710 | PDI_H | -0.001153076 | 0.000908186 | -1.269646999 | 0.204231969 |
| tnxb | 13632 | PDI_H | 0.001149008 | 0.00090513 | 1.269439847 | 0.204305922 |
| casp7 | 11767 | PDI_H | -0.001261675 | 0.00099407 | -1.269201022 | 0.204394505 |
| crim1 | 13710 | PDI_H | -0.001123166 | 0.000885049 | -1.269043438 | 0.204447134 |
| pts | 13272 | PDI_H | -0.001115326 | 0.000879063 | -1.268767067 | 0.204546426 |
| camlg | 11483 | PDI_H | -0.001275382 | 0.001006952 | -1.266577683 | 0.205332054 |
| f7 | 13666 | PDI_H | -0.001117398 | 0.00088263 | -1.265986333 | 0.205539575 |
| lrtm2 | 11757 | PDI_H | 0.001169264 | 0.000923712 | 1.265832557 | 0.205598145 |
| map2 | 11767 | PDI_H | -0.001252884 | 0.000990144 | -1.265355116 | 0.205769138 |
| igfbp6 | 13573 | PDI_H | -0.000958712 | 0.000757728 | -1.265246254 | 0.205804806 |
| tlr4 | 11411 | PDI_H | 0.001274538 | 0.001008064 | 1.264341996 | 0.206133153 |
| gne | 13308 | PDI_H | -0.001175532 | 0.00092983 | -1.264244987 | 0.20616427 |
| tspan15 | 11355 | PDI_H | -0.001275067 | 0.001011276 | -1.260849347 | 0.207389064 |
| reep4 | 11757 | PDI_H | -0.00125247 | 0.000993893 | -1.2601658 | 0.207634594 |
| pde1c | 11304 | PDI_H | -0.001278293 | 0.001014583 | -1.259919728 | 0.207724359 |
| tnfrsf4 | 13779 | PDI_H | -0.001104199 | 0.000878697 | -1.256632562 | 0.208908039 |
| septin7 | 11570 | PDI_H | -0.00126072 | 0.001003285 | -1.256592613 | 0.208926587 |
| aprt | 13532 | PDI_H | -0.001155019 | 0.000920179 | -1.255210583 | 0.209424019 |
| adgrd1 | 11537 | PDI_H | -0.001126278 | 0.000897556 | -1.254827081 | 0.209566985 |
| naa10 | 11355 | PDI_H | -0.001267899 | 0.001010989 | -1.254117604 | 0.209825106 |
| entpd2 | 13308 | PDI_H | 0.001161663 | 0.000927701 | 1.252195508 | 0.210520658 |
| ngf | 13552 | PDI_H | -0.001157251 | 0.000925385 | -1.250561932 | 0.211115957 |
| med18 | 13308 | PDI_H | -0.001164673 | 0.000931751 | -1.24998357 | 0.21132755 |
| defb116 | 11383 | PDI_H | -0.001260858 | 0.001009812 | -1.248606468 | 0.211834751 |
| tp53bp1 | 11411 | PDI_H | 0.001247238 | 0.000999014 | 1.248468083 | 0.211885331 |

| Protein | N_used | term | beta | se | t | p_val |
|---|---|---|---|---|---|---|
| znrd2 | 11724 | PDI_H | -0.001220015 | 0.000977341 | -1.248300482 | 0.211945991 |
| heg1 | 11537 | PDI_H | -0.00118504 | 0.000949637 | -1.247888296 | 0.21209732 |
| cd38 | 13579 | PDI_H | -0.000924079 | 0.000740813 | -1.247385436 | 0.212277735 |
| hspa1a | 13695 | PDI_H | -0.001133928 | 0.000909992 | -1.24608637 | 0.212754022 |
| pbld | 13320 | PDI_H | -0.001131093 | 0.00090785 | -1.245903944 | 0.212821595 |
| trim5 | 13621 | PDI_H | -0.001146299 | 0.000920748 | -1.244964589 | 0.213166204 |
| cd55 | 13162 | PDI_H | -0.001129671 | 0.000907538 | -1.244763936 | 0.213240719 |
| akap12 | 11537 | PDI_H | 0.001242987 | 0.000998915 | 1.244336865 | 0.213400913 |
| nrcam | 13618 | PDI_H | 0.001128786 | 0.00090748 | 1.243868249 | 0.213569486 |
| il10 | 13378 | PDI_H | -0.001155821 | 0.000930258 | -1.242473783 | 0.214083605 |
| tk1 | 11570 | PDI_H | -0.001239833 | 0.000998254 | -1.242001256 | 0.2142613 |
| ntf4 | 13308 | PDI_H | -0.001153172 | 0.000930713 | -1.239019273 | 0.215360232 |
| ptrhd1 | 11537 | PDI_H | -0.001241785 | 0.001002695 | -1.238447592 | 0.215575367 |
| prdx2 | 11584 | PDI_H | -0.001216585 | 0.000983825 | -1.23658739 | 0.2162654 |
| ca9 | 13753 | PDI_H | 0.001090121 | 0.000881787 | 1.236263245 | 0.21638186 |
| dipk2b | 11537 | PDI_H | 0.001163139 | 0.000942295 | 1.23436753 | 0.217091151 |
| itgb7 | 13753 | PDI_H | 0.001125853 | 0.00091461 | 1.230965145 | 0.21835697 |
| usp25 | 11730 | PDI_H | -0.001220897 | 0.000992066 | -1.230661782 | 0.21847409 |
| mesd | 13632 | PDI_H | -0.001132038 | 0.000920225 | -1.230174846 | 0.218652884 |
| cdc123 | 11304 | PDI_H | -0.00124794 | 0.001015193 | -1.229263523 | 0.218998633 |
| ckmt1a_ckmt1b | 13608 | PDI_H | -0.00112931 | 0.000921126 | -1.226010286 | 0.220216038 |
| sh3glb2 | 11767 | PDI_H | -0.001206863 | 0.000984981 | -1.225265267 | 0.22049984 |
| fut3_fut5 | 13784 | PDI_H | -0.00106058 | 0.000865996 | -1.224693589 | 0.220711638 |
| gipc3 | 11724 | PDI_H | -0.001219691 | 0.000996559 | -1.223902438 | 0.221013654 |
| pde4d | 11355 | PDI_H | -0.001238054 | 0.001012266 | -1.223052016 | 0.221335459 |
| oxct1 | 11474 | PDI_H | -0.001227884 | 0.001005653 | -1.220980913 | 0.222118362 |
| slit2 | 13320 | PDI_H | -0.001128822 | 0.000926993 | -1.217724623 | 0.223350227 |
| nid2 | 13632 | PDI_H | -0.001117504 | 0.000917841 | -1.217535672 | 0.223421566 |
| hsd17b14 | 11767 | PDI_H | -0.001205358 | 0.000991408 | -1.215804236 | 0.22408392 |
| tia1 | 13259 | PDI_H | -0.001132834 | 0.000932629 | -1.214667209 | 0.224514695 |
| ebi3_il27 | 13732 | PDI_H | -0.001079969 | 0.000890912 | -1.212205553 | 0.225454581 |
| gsta3 | 13308 | PDI_H | -0.001043923 | 0.000861355 | -1.211954064 | 0.225551502 |
| slk | 11570 | PDI_H | 0.001214814 | 0.001003865 | 1.210137233 | 0.226250986 |
| satb1 | 11757 | PDI_H | -0.001204083 | 0.000995006 | -1.210126003 | 0.226254901 |
| gsn | 11744 | PDI_H | 0.001188467 | 0.000983061 | 1.208945707 | 0.226708068 |
| fen1 | 13308 | PDI_H | -0.001109774 | 0.000919025 | -1.207556168 | 0.227239507 |
| crh | 13278 | PDI_H | 0.001108897 | 0.000918308 | 1.207542642 | 0.227244762 |
| cit | 11483 | PDI_H | -0.001202717 | 0.000996906 | -1.206450339 | 0.227668777 |
| rgs10 | 11757 | PDI_H | -0.001198514 | 0.000994946 | -1.204601322 | 0.228381519 |
| ppif | 11790 | PDI_H | -0.001197047 | 0.000993832 | -1.204476727 | 0.228429575 |
| entpd6 | 13388 | PDI_H | -0.001111635 | 0.000924589 | -1.202301868 | 0.229267877 |
| cntn3 | 13632 | PDI_H | 0.000979284 | 0.000815825 | 1.200360227 | 0.230020346 |
| stx6 | 13308 | PDI_H | -0.00111664 | 0.000930278 | -1.200329016 | 0.23003297 |
| pnlip | 11794 | PDI_H | 0.00118549 | 0.000988892 | 1.198805994 | 0.230627502 |
| lyn | 13579 | PDI_H | -0.001103376 | 0.000921263 | -1.197676965 | 0.231063724 |
| tctn3 | 11781 | PDI_H | -0.001136107 | 0.000949934 | -1.195985292 | 0.231726394 |
| ppp1r2 | 13618 | PDI_H | -0.001101213 | 0.000920963 | -1.195718359 | 0.231827323 |
| sat2 | 11570 | PDI_H | -0.001190253 | 0.000995908 | -1.195143466 | 0.232055504 |
| bsg | 13779 | PDI_H | -0.001036862 | 0.000868353 | -1.194055999 | 0.232476637 |
| ikbkg | 13621 | PDI_H | -0.001098043 | 0.000919732 | -1.193872143 | 0.232548796 |
| cacnb3 | 11757 | PDI_H | -0.001184017 | 0.000991813 | -1.193790712 | 0.232583957 |
| iqgap2 | 13579 | PDI_H | -0.001094153 | 0.000916622 | -1.193680175 | 0.23262397 |
| tbcc | 13552 | PDI_H | -0.001101522 | 0.000923143 | -1.193230231 | 0.232800126 |
| ddhd2 | 11757 | PDI_H | -0.001183171 | 0.000991842 | -1.192902912 | 0.232931496 |
| itgal | 11411 | PDI_H | -0.001181529 | 0.000990521 | -1.19283575 | 0.232958534 |
| dab2 | 13753 | PDI_H | -0.001093631 | 0.000916856 | -1.192804899 | 0.232966387 |
| tp53i3 | 11271 | PDI_H | -0.001197016 | 0.001003651 | -1.1926616 | 0.233027064 |
| suox | 11757 | PDI_H | -0.001129928 | 0.000948475 | -1.191310823 | 0.23355566 |
| map4k5 | 13535 | PDI_H | -0.001099226 | 0.000923642 | -1.190100459 | 0.234027802 |
| cdhr1 | 13320 | PDI_H | -0.001093242 | 0.000920009 | -1.188294542 | 0.234738592 |
| upb1 | 11781 | PDI_H | -0.001081267 | 0.000911799 | -1.185860863 | 0.235701192 |
| serpina4 | 11570 | PDI_H | 0.00117563 | 0.000991712 | 1.185454057 | 0.235862341 |
| dand5 | 11411 | PDI_H | 0.001194449 | 0.001008973 | 1.183826343 | 0.236506498 |
| stk4 | 13618 | PDI_H | -0.001091644 | 0.000922692 | -1.183107523 | 0.236787209 |
| micb_mica | 13621 | PDI_H | -0.001078706 | 0.000912702 | -1.181881269 | 0.237273466 |
| lilrb2 | 13727 | PDI_H | -0.001057857 | 0.000895681 | -1.18106459 | 0.237597546 |
| ceacam20 | 11570 | PDI_H | 0.001179615 | 0.000999652 | 1.180026434 | 0.238013987 |
| thap12 | 11271 | PDI_H | 0.001197121 | 0.001016076 | 1.178180388 | 0.2387496 |
| erp29 | 11537 | PDI_H | -0.001181776 | 0.001003136 | -1.178081896 | 0.238788283 |
| psma1 | 13308 | PDI_H | -0.001098918 | 0.000932865 | -1.178003418 | 0.238816327 |

| Protein | N_used | term | beta | se | t | p_val |
|---|---|---|---|---|---|---|
| cxadr | 13621 | PDI_H | -0.001075332 | 0.000913028 | -1.177764178 | 0.238911229 |
| ruvbl1 | 13308 | PDI_H | 0.001095989 | 0.000931953 | 1.176012819 | 0.239610809 |
| chga | 11790 | PDI_H | -0.001133273 | 0.00096476 | -1.174668247 | 0.240151216 |
| vnn1 | 11794 | PDI_H | -0.00111672 | 0.00095143 | -1.173727441 | 0.240527941 |
| tgfbr1 | 11411 | PDI_H | -0.001180267 | 0.001005957 | -1.173277872 | 0.240708908 |
| col2a1 | 11474 | PDI_H | 0.001178702 | 0.001005173 | 1.172636601 | 0.240965935 |
| mrpl24 | 11355 | PDI_H | -0.001185727 | 0.001011648 | -1.172074766 | 0.241191656 |
| jmjd1c | 11304 | PDI_H | -0.001189578 | 0.001015318 | -1.171631391 | 0.2413698 |
| stx8 | 13621 | PDI_H | -0.001073442 | 0.00091704 | -1.170550103 | 0.241800161 |
| coro1a | 13394 | PDI_H | -0.001081584 | 0.000925628 | -1.168487114 | 0.24263115 |
| serpinb6 | 13745 | PDI_H | -0.001071256 | 0.000916827 | -1.16843918 | 0.242649942 |
| kirrel1 | 11383 | PDI_H | -0.001179346 | 0.001010602 | -1.166974545 | 0.243245121 |
| ifnw1 | 11355 | PDI_H | -0.001180973 | 0.001012476 | -1.166421061 | 0.24346877 |
| hs1bp3 | 11474 | PDI_H | -0.001170509 | 0.001003857 | -1.166012299 | 0.243633734 |
| nudc | 13801 | PDI_H | -0.001062557 | 0.000913955 | -1.162592224 | 0.245015107 |
| hmox1 | 13461 | PDI_H | -0.001051966 | 0.000905798 | -1.161368695 | 0.245512605 |
| ephx2 | 13388 | PDI_H | -0.001077505 | 0.00092779 | -1.161368194 | 0.245512922 |
| amigo2 | 13579 | PDI_H | 0.001062051 | 0.000915045 | 1.160654068 | 0.24580303 |
| chmp1a | 13552 | PDI_H | -0.001072941 | 0.000924443 | -1.160635781 | 0.24581051 |
| serpinb1 | 13575 | PDI_H | -0.001065811 | 0.000919097 | -1.159627474 | 0.246220924 |
| pdia2 | 11304 | PDI_H | 0.001175659 | 0.001014976 | 1.158312125 | 0.246761182 |
| cd63 | 13526 | PDI_H | -0.001057318 | 0.000912889 | -1.158210841 | 0.246798473 |
| fzd8 | 11383 | PDI_H | 0.001169546 | 0.001010389 | 1.157521164 | 0.247083811 |
| maneal | 11304 | PDI_H | 0.001174253 | 0.001015152 | 1.156726342 | 0.247408654 |
| slc27a4 | 13320 | PDI_H | -0.001073612 | 0.000928964 | -1.155708789 | 0.24782104 |
| cxcl14 | 13378 | PDI_H | 0.00097596 | 0.000845316 | 1.154550213 | 0.248295306 |
| sox9 | 11483 | PDI_H | -0.001157577 | 0.001002695 | -1.154464976 | 0.24833364 |
| lactb2 | 13618 | PDI_H | -0.00106296 | 0.000920906 | -1.154253945 | 0.248416345 |
| cd4 | 13604 | PDI_H | -0.001014351 | 0.000878969 | -1.154023795 | 0.248510704 |
| amy2a | 13121 | PDI_H | 0.00104629 | 0.000907931 | 1.152389842 | 0.249181928 |
| csde1 | 11355 | PDI_H | -0.001162522 | 0.00101035 | -1.150613744 | 0.249915431 |
| cd164 | 13632 | PDI_H | -0.001046741 | 0.000910381 | -1.149782778 | 0.250253539 |
| cenpj | 11570 | PDI_H | -0.001152141 | 0.001003184 | -1.148484634 | 0.250792321 |
| eif4b | 13552 | PDI_H | -0.001055746 | 0.000919337 | -1.148377331 | 0.250833115 |
| snx15 | 11537 | PDI_H | -0.001141045 | 0.000995018 | -1.146758329 | 0.251505323 |
| akr1b1 | 13308 | PDI_H | -0.00106606 | 0.000931489 | -1.144468548 | 0.252449976 |
| bnip3l | 11411 | PDI_H | -0.001136377 | 0.000993113 | -1.144257138 | 0.25254104 |
| vmo1 | 13579 | PDI_H | -0.001035096 | 0.000904631 | -1.144219172 | 0.252552941 |
| serpinc1 | 11790 | PDI_H | 0.001109873 | 0.000970048 | 1.144142829 | 0.25258766 |
| ninj1 | 13308 | PDI_H | -0.001052872 | 0.00092101 | -1.143171184 | 0.252988107 |
| cnpy4 | 13579 | PDI_H | -0.001053894 | 0.000923719 | -1.140923769 | 0.253921786 |
| cpa2 | 13746 | PDI_H | 0.00104234 | 0.000913857 | 1.140594722 | 0.254058504 |
| gba | 11483 | PDI_H | -0.001146528 | 0.001005656 | -1.140079562 | 0.254276959 |
| rab6b | 13320 | PDI_H | -0.001065482 | 0.000934706 | -1.139911065 | 0.25434387 |
| stau1 | 11304 | PDI_H | -0.001155609 | 0.001013819 | -1.139856607 | 0.254370223 |
| acox1 | 13618 | PDI_H | -0.001050926 | 0.00092225 | -1.139523148 | 0.254505079 |
| hip1 | 11355 | PDI_H | -0.001153214 | 0.001012369 | -1.139124646 | 0.254675225 |
| igbp1 | 11757 | PDI_H | -0.001125109 | 0.000987894 | -1.138896983 | 0.254769352 |
| afp | 13510 | PDI_H | -0.001021403 | 0.000898224 | -1.137136166 | 0.255501553 |
| sptlc1 | 11767 | PDI_H | -0.001126666 | 0.000991647 | -1.136155957 | 0.255914462 |
| vipr1 | 11474 | PDI_H | -0.001144404 | 0.001007356 | -1.136047327 | 0.25596051 |
| cenpf | 11570 | PDI_H | -0.001123688 | 0.000989756 | -1.135318032 | 0.256265632 |
| clec4c | 13621 | PDI_H | -0.001037168 | 0.000913681 | -1.135153282 | 0.256331092 |
| pak4 | 13320 | PDI_H | -0.0010552 | 0.000930637 | -1.133847677 | 0.256878872 |
| eef1d | 11781 | PDI_H | -0.001117369 | 0.000985608 | -1.133684084 | 0.256950181 |
| pagr1 | 11474 | PDI_H | -0.001135402 | 0.00100334 | -1.131622389 | 0.257816898 |
| shh | 11411 | PDI_H | 0.001139191 | 0.001007583 | 1.130616841 | 0.258240184 |
| dgka | 11411 | PDI_H | -0.001135324 | 0.001004251 | -1.13051809 | 0.258281766 |
| anxa4 | 13564 | PDI_H | -0.001043219 | 0.000924291 | -1.128669374 | 0.259057313 |
| msmb | 13592 | PDI_H | -0.000946181 | 0.000839012 | -1.127731832 | 0.259453114 |
| tnfrsf21 | 13784 | PDI_H | -0.001007946 | 0.000893849 | -1.127646753 | 0.259488779 |
| osbpl2 | 11767 | PDI_H | -0.001121161 | 0.000994486 | -1.127377125 | 0.259606078 |
| pdcl2 | 11304 | PDI_H | -0.001142795 | 0.00101501 | -1.125894704 | 0.260234019 |
| tbl1x | 13308 | PDI_H | -0.001049966 | 0.00093261 | -1.125836436 | 0.260255079 |
| cct5 | 13308 | PDI_H | -0.001046997 | 0.000930723 | -1.124928837 | 0.260639498 |
| cr2 | 13689 | PDI_H | 0.001017565 | 0.000904733 | 1.124713025 | 0.260730398 |
| wfdc1 | 11489 | PDI_H | -0.001117593 | 0.000994776 | -1.123462013 | 0.261264824 |
| dlk1 | 13800 | PDI_H | 0.000989678 | 0.000881393 | 1.122857799 | 0.261517445 |
| npy | 13753 | PDI_H | 0.001007218 | 0.000897389 | 1.12238733 | 0.2617174 |
| nap1l4 | 11698 | PDI_H | -0.001115576 | 0.000995379 | -1.120755614 | 0.262414933 |

| Protein | N_used | term | beta | se | t | p_val |
|---|---|---|---|---|---|---|
| klk15 | 11706 | PDI_H | -0.001108478 | 0.000990875 | -1.118685334 | 0.263297383 |
| atp6v1f | 13303 | PDI_H | -0.001041976 | 0.000931786 | -1.118257072 | 0.263477427 |
| scn3b | 11383 | PDI_H | -0.001130361 | 0.00101138 | -1.117642649 | 0.263743259 |
| chgb | 13750 | PDI_H | -0.000972507 | 0.000870268 | -1.117479938 | 0.263808715 |
| nagk | 11450 | PDI_H | -0.001106522 | 0.000990322 | -1.117335173 | 0.263874511 |
| cert | 13320 | PDI_H | -0.001041209 | 0.000932405 | -1.116691324 | 0.26414649 |
| fosb | 13320 | PDI_H | -0.001039554 | 0.000933762 | -1.113296769 | 0.265601101 |
| dsg4 | 13534 | PDI_H | -0.001000754 | 0.000899562 | -1.112489485 | 0.265947526 |
| cnpy2 | 13388 | PDI_H | -0.001033997 | 0.000929978 | -1.111850869 | 0.266222259 |
| acot13 | 11724 | PDI_H | -0.00110618 | 0.000994951 | -1.111792897 | 0.266250028 |
| atp2b4 | 11355 | PDI_H | -0.001122499 | 0.001010259 | -1.111100684 | 0.266548564 |
| ssna1 | 11724 | PDI_H | -0.00109781 | 0.000988944 | -1.110082674 | 0.266986193 |
| ptprz1 | 11781 | PDI_H | 0.000996435 | 0.000898486 | 1.10901604 | 0.267445924 |
| dnm1 | 11724 | PDI_H | -0.00110299 | 0.000994638 | -1.108935786 | 0.267480654 |
| cacna1h | 11355 | PDI_H | -0.001120606 | 0.001010642 | -1.108806652 | 0.267537109 |
| s100a16 | 13301 | PDI_H | 0.001032467 | 0.00093234 | 1.10739344 | 0.268143904 |
| lap3 | 13378 | PDI_H | -0.001025219 | 0.00092599 | -1.107159773 | 0.268244781 |
| vwa5a | 11304 | PDI_H | -0.001123073 | 0.001014486 | -1.10703667 | 0.268301656 |
| manf | 13710 | PDI_H | -0.001016327 | 0.000918353 | -1.106684946 | 0.2684496 |
| ccne1 | 11411 | PDI_H | 0.001114345 | 0.001006918 | 1.106689659 | 0.268451482 |
| fgfbp3 | 11767 | PDI_H | -0.001072512 | 0.000969383 | -1.1063861 | 0.26858208 |
| elac1 | 11757 | PDI_H | -0.00109975 | 0.000994063 | -1.106317829 | 0.268611637 |
| tarbp2 | 13320 | PDI_H | -0.001023537 | 0.000929673 | -1.10096459 | 0.270931982 |
| elavl4 | 11383 | PDI_H | -0.001111789 | 0.001010676 | -1.100045339 | 0.271335656 |
| fnta | 11730 | PDI_H | -0.001083403 | 0.000986455 | -1.098279276 | 0.272105124 |
| c1qtnf1 | 13689 | PDI_H | 0.000974153 | 0.000887841 | 1.097215485 | 0.272566522 |
| scgb3a2 | 13691 | PDI_H | -0.000945222 | 0.000862423 | -1.096006541 | 0.273095207 |
| ppib | 13641 | PDI_H | -0.001008065 | 0.000919869 | -1.095879082 | 0.273151059 |
| mpi | 13579 | PDI_H | -0.001009516 | 0.000921356 | -1.09568462 | 0.273236265 |
| tbcb | 13552 | PDI_H | -0.001011673 | 0.000923932 | -1.094964818 | 0.273551527 |
| khdc3l | 11304 | PDI_H | -0.001111281 | 0.0010152 | -1.094642691 | 0.273696553 |
| tcl1b | 13388 | PDI_H | -0.00101697 | 0.000930743 | -1.092643067 | 0.274570224 |
| pcdh1 | 13757 | PDI_H | 0.000971105 | 0.000888822 | 1.092576102 | 0.274599109 |
| fam172a | 11474 | PDI_H | -0.001099476 | 0.001006356 | -1.092532236 | 0.274622191 |
| nectin1 | 11411 | PDI_H | 0.001099878 | 0.00100734 | 1.091863587 | 0.274916141 |
| ifnl1 | 13320 | PDI_H | -0.001014895 | 0.000930261 | -1.090978184 | 0.275302234 |
| scarf1 | 13800 | PDI_H | -0.000992494 | 0.000909839 | -1.090845904 | 0.275359756 |
| hhex | 11767 | PDI_H | -0.00105973 | 0.000971585 | -1.090722577 | 0.27541733 |
| bloc1s3 | 11767 | PDI_H | -0.001081741 | 0.000993307 | -1.08902948 | 0.276163211 |
| tcn2 | 13777 | PDI_H | -0.00096591 | 0.000887573 | -1.088259734 | 0.276499515 |
| cd93 | 13141 | PDI_H | 0.001010617 | 0.000929493 | 1.087277593 | 0.276934108 |
| ap1g2 | 11730 | PDI_H | -0.001077506 | 0.000991532 | -1.086708365 | 0.277188069 |
| il1r1 | 13602 | PDI_H | 0.00098257 | 0.000904893 | 1.085840507 | 0.277568822 |
| ighmbp2 | 11355 | PDI_H | -0.001096559 | 0.001011932 | -1.083628653 | 0.278552514 |
| stoml2 | 11304 | PDI_H | -0.001097768 | 0.001014766 | -1.081794707 | 0.279366861 |
| prg2 | 11790 | PDI_H | -0.001048458 | 0.000971452 | -1.079269013 | 0.280489923 |
| esr1 | 11411 | PDI_H | -0.001086657 | 0.001006897 | -1.079213944 | 0.2805152 |
| tfpi2 | 13654 | PDI_H | -0.000938659 | 0.000869955 | -1.078974599 | 0.280618128 |
| tcof1 | 11781 | PDI_H | -0.001036473 | 0.000960956 | -1.078585402 | 0.280794697 |
| kit | 13669 | PDI_H | -0.000946796 | 0.000879105 | -1.076999765 | 0.281499394 |
| adgrg1 | 13579 | PDI_H | -0.00091608 | 0.000851493 | -1.075851446 | 0.282012831 |
| atp6v1g2 | 11355 | PDI_H | -0.001087846 | 0.001012129 | -1.074809454 | 0.282482909 |
| folr2 | 13784 | PDI_H | -0.000927482 | 0.000863213 | -1.074453606 | 0.282638251 |
| dhps | 11730 | PDI_H | -0.001054453 | 0.000982311 | -1.07344116 | 0.28309533 |
| snap29 | 13699 | PDI_H | -0.000983837 | 0.000916539 | -1.073426679 | 0.283098644 |
| flt1 | 13559 | PDI_H | -0.000980191 | 0.000914161 | -1.072229814 | 0.283635923 |
| myh9 | 11790 | PDI_H | -0.001058477 | 0.00098718 | -1.072222605 | 0.283642028 |
| lgals3 | 13663 | PDI_H | -0.000927032 | 0.00086645 | -1.069919252 | 0.284674574 |
| impa1 | 13552 | PDI_H | -0.000989056 | 0.000924428 | -1.069911352 | 0.284678285 |
| calcoco2 | 11474 | PDI_H | -0.001074619 | 0.001005067 | -1.069201317 | 0.285001482 |
| ca7 | 11570 | PDI_H | 0.001069504 | 0.001000989 | 1.068447671 | 0.285340938 |
| fam3d | 11724 | PDI_H | 0.00105651 | 0.000990274 | 1.066885828 | 0.286045386 |
| tet2 | 11549 | PDI_H | -0.001068883 | 0.001004497 | -1.064097998 | 0.287306578 |
| hjv | 11757 | PDI_H | -0.001009098 | 0.000948355 | -1.064051353 | 0.287327312 |
| igfbp7 | 13800 | PDI_H | -0.000906296 | 0.000851859 | -1.063903923 | 0.287390857 |
| prkar2a | 11750 | PDI_H | -0.001053043 | 0.000990351 | -1.063302617 | 0.287666613 |
| gigyf2 | 11757 | PDI_H | -0.001052597 | 0.000989988 | -1.06324209 | 0.287694039 |
| galnt2 | 13753 | PDI_H | 0.00095033 | 0.000894454 | 1.062468897 | 0.288041543 |
| drg2 | 13579 | PDI_H | -0.000981661 | 0.000924104 | -1.062283855 | 0.28812575 |
| cpb2 | 11790 | PDI_H | -0.001039823 | 0.00097907 | -1.06205137 | 0.288234141 |

| Protein | N_used | term | beta | se | t | p_val |
|---|---|---|---|---|---|---|
| abhd14b | 13509 | PDI_H | -0.000975407 | 0.000919585 | -1.060703905 | 0.288843465 |
| tbc1d5 | 13621 | PDI_H | -0.000976678 | 0.000921056 | -1.060389645 | 0.28898619 |
| art3 | 13689 | PDI_H | 0.000867291 | 0.0008179 | 1.060387964 | 0.28898686 |
| paxx | 11537 | PDI_H | -0.001044369 | 0.000984992 | -1.060281642 | 0.289038708 |
| smpdl3b | 11730 | PDI_H | -0.001055231 | 0.00099615 | -1.059309466 | 0.2894807 |
| tax1bp1 | 11767 | PDI_H | -0.001045922 | 0.000987589 | -1.059066565 | 0.289591227 |
| stx7 | 11411 | PDI_H | -0.001042701 | 0.000985055 | -1.058520284 | 0.289840738 |
| id4 | 11570 | PDI_H | 0.001060607 | 0.001002646 | 1.057808146 | 0.290165026 |
| s100a4 | 13699 | PDI_H | -0.000965534 | 0.000914917 | -1.055323541 | 0.291296006 |
| ermap | 11411 | PDI_H | 0.001013316 | 0.000960832 | 1.054624193 | 0.291619587 |
| sox2 | 11732 | PDI_H | 0.001050826 | 0.000996672 | 1.054335444 | 0.291751102 |
| ifnar1 | 11724 | PDI_H | -0.001042555 | 0.000991176 | -1.051836563 | 0.292896242 |
| xrcc4 | 13320 | PDI_H | -0.00097974 | 0.000932689 | -1.050446631 | 0.29353189 |
| traf3 | 11411 | PDI_H | 0.001047365 | 0.00099803 | 1.049433055 | 0.2940011 |
| ddx1 | 11724 | PDI_H | 0.001045245 | 0.000996025 | 1.049415964 | 0.294008368 |
| alpp | 13579 | PDI_H | -0.00090485 | 0.000862401 | -1.049221612 | 0.294094824 |
| mcts1 | 11724 | PDI_H | -0.001041085 | 0.000994616 | -1.046719925 | 0.295250372 |
| twf2 | 11790 | PDI_H | -0.001038141 | 0.000991815 | -1.046708203 | 0.295255659 |
| pten | 13320 | PDI_H | -0.000976458 | 0.000934492 | -1.044907406 | 0.296084746 |
| ghrl | 13618 | PDI_H | 0.000883909 | 0.00084634 | 1.044390202 | 0.29632345 |
| rtn4r | 13753 | PDI_H | -0.000848858 | 0.000813181 | -1.043873903 | 0.296562098 |
| sema4d | 13784 | PDI_H | -0.000951603 | 0.000911887 | -1.043553368 | 0.296710394 |
| ptpn6 | 13691 | PDI_H | -0.000959387 | 0.000919462 | -1.043421903 | 0.296771373 |
| nrn1 | 11757 | PDI_H | 0.001039047 | 0.000995901 | 1.043323844 | 0.296819805 |
| il21r | 11411 | PDI_H | -0.001051103 | 0.001007912 | -1.042851912 | 0.297039 |
| get3 | 11781 | PDI_H | 0.001034024 | 0.000992228 | 1.04212323 | 0.297375956 |
| pcare | 11383 | PDI_H | -0.001053178 | 0.001010696 | -1.042031766 | 0.297419105 |
| bmper | 11537 | PDI_H | -0.001016652 | 0.000978316 | -1.039185669 | 0.298740195 |
| morf4l2 | 11304 | PDI_H | -0.001053509 | 0.001014443 | -1.038509655 | 0.299055079 |
| siglec10 | 13621 | PDI_H | -0.000923722 | 0.000889713 | -1.038224638 | 0.299183931 |
| dbnl | 13710 | PDI_H | -0.000949673 | 0.000917555 | -1.035004102 | 0.300685267 |
| ceacam18 | 11570 | PDI_H | -0.00103755 | 0.001003228 | -1.034211757 | 0.301058817 |
| pglyrp2 | 11647 | PDI_H | -0.001027306 | 0.000994467 | -1.033022421 | 0.301614878 |
| cfhr2 | 11728 | PDI_H | 0.001017989 | 0.000985629 | 1.032831753 | 0.30170396 |
| nampt | 13308 | PDI_H | -0.000954682 | 0.000924638 | -1.032492634 | 0.301860176 |
| mink1 | 11767 | PDI_H | -0.001025237 | 0.000993404 | -1.032044309 | 0.302072594 |
| rab10 | 11781 | PDI_H | -0.001020581 | 0.000992114 | -1.028693495 | 0.303644869 |
| kazn | 11724 | PDI_H | -0.00102341 | 0.000994865 | -1.028692584 | 0.3036454 |
| kcnip4 | 13320 | PDI_H | 0.000959254 | 0.000932968 | 1.028175339 | 0.303886044 |
| scgb1a1 | 13663 | PDI_H | 0.000902756 | 0.000878108 | 1.028069003 | 0.303935585 |
| ckb | 11790 | PDI_H | -0.000888325 | 0.000864444 | -1.027626957 | 0.304146442 |
| atrn | 11817 | PDI_H | -0.000985692 | 0.000959967 | -1.02679775 | 0.304536748 |
| smad2 | 11730 | PDI_H | -0.001018233 | 0.00099213 | -1.026309137 | 0.304767077 |
| fgfbp1 | 13699 | PDI_H | 0.00091216 | 0.000889774 | 1.025160166 | 0.305305738 |
| c1s | 11817 | PDI_H | -0.000985801 | 0.00096214 | -1.024591859 | 0.305576802 |
| rbm25 | 11304 | PDI_H | 0.001040312 | 0.001015656 | 1.024276003 | 0.305726871 |
| matn3 | 13526 | PDI_H | -0.000924127 | 0.000903485 | -1.022846165 | 0.306398896 |
| syap1 | 11537 | PDI_H | -0.001023383 | 0.001001081 | -1.022278096 | 0.306670754 |
| vwc2l | 11757 | PDI_H | -0.000949105 | 0.000929663 | -1.020913056 | 0.307316661 |
| hbq1 | 13753 | PDI_H | -0.000923186 | 0.000904294 | -1.020890555 | 0.307324267 |
| cntnap4 | 11483 | PDI_H | 0.001025632 | 0.001006085 | 1.01942879 | 0.308020943 |
| dll4 | 11474 | PDI_H | -0.001023188 | 0.001003889 | -1.019223734 | 0.308118273 |
| kel | 13320 | PDI_H | -0.000916958 | 0.000900374 | -1.018419024 | 0.308497379 |
| btn2a1 | 13710 | PDI_H | -0.000896663 | 0.000881093 | -1.017670934 | 0.308852339 |
| csnk1d | 11411 | PDI_H | 0.001021815 | 0.001004586 | 1.017150102 | 0.309103617 |
| zbp1 | 11411 | PDI_H | -0.00101162 | 0.000994822 | -1.016885964 | 0.309229263 |
| cdh6 | 13618 | PDI_H | 0.000923961 | 0.000909006 | 1.016451312 | 0.309432595 |
| dhodh | 11355 | PDI_H | -0.001028811 | 0.001012621 | -1.015988111 | 0.309656718 |
| rad23b | 13308 | PDI_H | -0.000947043 | 0.000932477 | -1.015620702 | 0.309828523 |
| kdm3a | 11411 | PDI_H | -0.0010197 | 0.001004575 | -1.015056008 | 0.310100674 |
| neb | 11355 | PDI_H | -0.001017974 | 0.001003276 | -1.014649945 | 0.310294364 |
| ece1 | 13320 | PDI_H | -0.000945068 | 0.000931877 | -1.014154774 | 0.310527343 |
| bcl7a | 11304 | PDI_H | 0.001028772 | 0.001015343 | 1.013225767 | 0.310974045 |
| mag | 11483 | PDI_H | 0.001021097 | 0.001007887 | 1.013106186 | 0.311030812 |
| ctf1 | 13388 | PDI_H | -0.000940549 | 0.00092915 | -1.012267839 | 0.311428318 |
| frmd7 | 11483 | PDI_H | 0.00101985 | 0.001007875 | 1.011881213 | 0.311616196 |
| tdrkh | 13320 | PDI_H | -0.000940054 | 0.000929766 | -1.011064736 | 0.312003829 |
| c2orf69 | 11483 | PDI_H | -0.001017511 | 0.001006833 | -1.010605985 | 0.312226367 |
| apof | 11794 | PDI_H | 0.000885647 | 0.000876692 | 1.01021499 | 0.312413045 |
| arhgef12 | 13621 | PDI_H | -0.000929821 | 0.000920494 | -1.010132435 | 0.312449804 |

| Protein | N_used | term | beta | se | t | p_val |
|---|---|---|---|---|---|---|
| rgs8 | 13378 | PDI_H | -0.000941548 | 0.000932224 | -1.010001439 | 0.312512884 |
| selplg | 13621 | PDI_H | -0.00091086 | 0.000902059 | -1.009757439 | 0.312629468 |
| fabp6 | 13371 | PDI_H | -0.000934513 | 0.000925958 | -1.009239009 | 0.312878302 |
| cga | 13632 | PDI_H | -0.000690236 | 0.000685192 | -1.007361197 | 0.313779127 |
| mthfsd | 11570 | PDI_H | -0.001006623 | 0.000999642 | -1.006983347 | 0.313963857 |
| nop56 | 11355 | PDI_H | -0.001018568 | 0.001011887 | -1.006602699 | 0.314147209 |
| nrp1 | 13712 | PDI_H | 0.000910889 | 0.000906158 | 1.005221385 | 0.314808014 |
| wwp2 | 13552 | PDI_H | -0.000921297 | 0.000917644 | -1.003981239 | 0.315405598 |
| psca | 11724 | PDI_H | 0.000995351 | 0.000991625 | 1.003756728 | 0.315516624 |
| apom | 13669 | PDI_H | -0.00088807 | 0.000885117 | -1.003336217 | 0.315716444 |
| gbp6 | 11355 | PDI_H | -0.001015772 | 0.00101254 | -1.003191964 | 0.315789649 |
| plscr3 | 11747 | PDI_H | -0.000993433 | 0.000991146 | -1.002307211 | 0.316215908 |
| itih1 | 11817 | PDI_H | 0.0009937 | 0.00099217 | 1.001542465 | 0.316585149 |
| ahcy | 13618 | PDI_H | -0.000913487 | 0.000912764 | -1.000791537 | 0.3169454 |
| serpine1 | 13800 | PDI_H | -0.000899553 | 0.000899149 | -1.000450002 | 0.317110339 |
| podxl2 | 13699 | PDI_H | -0.000896717 | 0.000896479 | -1.000265827 | 0.317199563 |
| washc3 | 11767 | PDI_H | -0.000988368 | 0.000988466 | -0.999901573 | 0.317378725 |
| s100a12 | 13461 | PDI_H | -0.000914015 | 0.000914371 | -0.999610256 | 0.317517142 |
| il25 | 11252 | PDI_H | 0.001015852 | 0.001017684 | 0.998199903 | 0.318203922 |
| pdia5 | 11304 | PDI_H | 0.001010753 | 0.001014028 | 0.996769711 | 0.318897662 |
| palm3 | 11355 | PDI_H | -0.001005979 | 0.001010638 | -0.995390525 | 0.319567598 |
| clip2 | 13621 | PDI_H | -0.00091518 | 0.000920258 | -0.994482957 | 0.32000548 |
| itm2a | 13621 | PDI_H | -0.000913933 | 0.000919503 | -0.993941754 | 0.320268897 |
| ntprobnp | 13618 | PDI_H | -0.000848276 | 0.000853678 | -0.993672692 | 0.320399912 |
| lrtm1 | 11483 | PDI_H | 0.001001008 | 0.001008625 | 0.992447633 | 0.321000146 |
| gtpbp2 | 11483 | PDI_H | -0.000994603 | 0.001007061 | -0.987629809 | 0.323354813 |
| ern1 | 11730 | PDI_H | -0.000957534 | 0.000969676 | -0.987478096 | 0.323428704 |
| sec31a | 11474 | PDI_H | -0.000991271 | 0.001004708 | -0.986625609 | 0.323847037 |
| c1qtnf6 | 11757 | PDI_H | -0.000977566 | 0.000991297 | -0.986148584 | 0.32408052 |
| artn | 13378 | PDI_H | -0.00091773 | 0.000931526 | -0.985190809 | 0.324548187 |
| csf1r | 11794 | PDI_H | -0.00093803 | 0.000952242 | -0.985075387 | 0.324607271 |
| nos3 | 13320 | PDI_H | -0.00086637 | 0.000879845 | -0.98468458 | 0.324796931 |
| rbks | 13366 | PDI_H | -0.000873361 | 0.000888189 | -0.983305494 | 0.325474921 |
| pcbd1 | 11512 | PDI_H | -0.000904071 | 0.000919765 | -0.982936295 | 0.325659472 |
| ccl4 | 13661 | PDI_H | -0.000883772 | 0.000899252 | -0.9827861 | 0.325730144 |
| padi4 | 13301 | PDI_H | -0.000906085 | 0.000923813 | -0.980810127 | 0.326704238 |
| ensa | 11730 | PDI_H | -0.000976115 | 0.000995687 | -0.98034296 | 0.326937095 |
| ythdf3 | 13378 | PDI_H | -0.000906253 | 0.000926026 | -0.978646897 | 0.327772191 |
| dnpep | 11767 | PDI_H | -0.000957726 | 0.000978874 | -0.978396241 | 0.327898521 |
| abraxas2 | 11355 | PDI_H | -0.000983844 | 0.001006837 | -0.977163155 | 0.328509225 |
| crhbp | 13658 | PDI_H | -0.000846514 | 0.000866431 | -0.977012468 | 0.328580299 |
| tjap1 | 13699 | PDI_H | -0.000896158 | 0.000917723 | -0.976501603 | 0.328833212 |
| cd3g | 11411 | PDI_H | -0.000984208 | 0.001008529 | -0.975885489 | 0.329141923 |
| kif20b | 11383 | PDI_H | -0.000984932 | 0.001011417 | -0.973813855 | 0.330169688 |
| fli1 | 13308 | PDI_H | -0.000903409 | 0.000928268 | -0.973219898 | 0.330461728 |
| shd | 11332 | PDI_H | 0.000985305 | 0.001012535 | 0.973107447 | 0.330520696 |
| c7 | 11510 | PDI_H | -0.000948626 | 0.000975246 | -0.972704483 | 0.330720659 |
| crybb1 | 11781 | PDI_H | -0.00096062 | 0.00098806 | -0.972228941 | 0.330956647 |
| rnaset2 | 13736 | PDI_H | -0.000856452 | 0.00088109 | -0.972037602 | 0.331048974 |
| hmgcs1 | 11355 | PDI_H | -0.000983162 | 0.001012666 | -0.970865134 | 0.331636144 |
| rbpms2 | 11474 | PDI_H | -0.000973396 | 0.001004177 | -0.969346425 | 0.332392827 |
| carhsp1 | 13320 | PDI_H | -0.000889674 | 0.000917853 | -0.969299344 | 0.332413472 |
| smad1 | 13308 | PDI_H | -0.000893962 | 0.000924171 | -0.967312073 | 0.333405644 |
| tdgf1 | 13320 | PDI_H | 0.000901794 | 0.000933257 | 0.966287094 | 0.333918101 |
| txndc9 | 11767 | PDI_H | -0.000959713 | 0.000993575 | -0.965919051 | 0.334104559 |
| txnrd1 | 13632 | PDI_H | -0.000875351 | 0.000909313 | -0.962651577 | 0.335739495 |
| akt3 | 13239 | PDI_H | -0.00089983 | 0.000934778 | -0.962612808 | 0.335759465 |
| top2b | 11411 | PDI_H | -0.000954291 | 0.00099256 | -0.961444168 | 0.336349279 |
| tlr1 | 11411 | PDI_H | 0.000960711 | 0.000999253 | 0.961429294 | 0.336356754 |
| ncs1 | 13753 | PDI_H | 0.000802592 | 0.000835405 | 0.960722493 | 0.336708623 |
| cspg5 | 11383 | PDI_H | -0.000966293 | 0.001006222 | -0.960317372 | 0.336915929 |
| ampd3 | 11767 | PDI_H | 0.000948528 | 0.000987907 | 0.960138893 | 0.337005065 |
| cdkn1a | 13579 | PDI_H | -0.000879294 | 0.000916407 | -0.959501374 | 0.337323327 |
| itgb1bp2 | 13388 | PDI_H | -0.000891152 | 0.000928899 | -0.959364113 | 0.337392688 |
| hspa2 | 11730 | PDI_H | -0.000932782 | 0.000973723 | -0.957954272 | 0.338105581 |
| mapre3 | 11304 | PDI_H | 0.000969695 | 0.001012887 | 0.957358294 | 0.338406937 |
| fcgr2b | 13308 | PDI_H | 0.000881821 | 0.000922102 | 0.956315337 | 0.33893033 |
| eif4e | 11411 | PDI_H | -0.000960655 | 0.001005611 | -0.955295049 | 0.339448769 |
| htr1a | 11383 | PDI_H | 0.000963383 | 0.001008647 | 0.95512422 | 0.339535186 |
| cd1c | 13699 | PDI_H | -0.000848899 | 0.000890875 | -0.952882799 | 0.340666283 |

| Protein | N_used | term | beta | se | t | p_val |
|---|---|---|---|---|---|---|
| ada2 | 13547 | PDI_H | -0.000851897 | 0.000894209 | -0.952681546 | 0.340768459 |
| phykpl | 11537 | PDI_H | -0.000948256 | 0.000995689 | -0.95236156 | 0.34093362 |
| klf4 | 11383 | PDI_H | -0.000940752 | 0.000988232 | -0.951955131 | 0.341139971 |
| ncln | 13378 | PDI_H | -0.00088512 | 0.000931412 | -0.950299237 | 0.341977409 |
| galnt3 | 13378 | PDI_H | 0.000882111 | 0.000928261 | 0.950283413 | 0.341985447 |
| epo | 13621 | PDI_H | 0.000861647 | 0.000907391 | 0.949588082 | 0.342338459 |
| ctag1a_ctag1b | 11535 | PDI_H | -0.000953703 | 0.001004557 | -0.949376559 | 0.342449039 |
| tppp3 | 13320 | PDI_H | 0.000881133 | 0.000929508 | 0.947955591 | 0.343169275 |
| vim | 13388 | PDI_H | 0.000880992 | 0.000929465 | 0.947848031 | 0.343223948 |
| sdccag8 | 11730 | PDI_H | -0.000940674 | 0.000992469 | -0.94781195 | 0.343244741 |
| ces3 | 13579 | PDI_H | -0.000854201 | 0.000901393 | -0.947645864 | 0.343326648 |
| septin3 | 11757 | PDI_H | -0.000921916 | 0.00097462 | -0.945923123 | 0.344207258 |
| serpini2 | 11724 | PDI_H | -0.000936578 | 0.000990253 | -0.945796136 | 0.344272088 |
| c1qbp | 11757 | PDI_H | -0.000941126 | 0.00099579 | -0.945104739 | 0.344624848 |
| crygd | 11767 | PDI_H | -0.000938471 | 0.000993405 | -0.944701742 | 0.344830584 |
| cd70 | 13621 | PDI_H | -0.000829105 | 0.000878246 | -0.944046373 | 0.345162711 |
| ak2 | 11767 | PDI_H | -0.000937851 | 0.000994119 | -0.943399113 | 0.345496185 |
| serpine2 | 11781 | PDI_H | -0.000934129 | 0.000990606 | -0.942988225 | 0.345706282 |
| cat | 11605 | PDI_H | -0.000915184 | 0.000971055 | -0.94246284 | 0.345975366 |
| lpp | 11355 | PDI_H | -0.000952479 | 0.00101066 | -0.942433119 | 0.345991009 |
| klhl41 | 11757 | PDI_H | 0.000908595 | 0.000966546 | 0.940042889 | 0.347214894 |
| slc12a2 | 11355 | PDI_H | 0.000951167 | 0.001011977 | 0.939909674 | 0.347283909 |
| acy3 | 11355 | PDI_H | 0.000932367 | 0.0009921 | 0.939791842 | 0.347344356 |
| arhgap45 | 11537 | PDI_H | -0.000942978 | 0.001003536 | -0.939655955 | 0.347413757 |
| blnk | 11411 | PDI_H | -0.000933439 | 0.000993515 | -0.939531115 | 0.347478033 |
| c1qtnf5 | 11537 | PDI_H | 0.000932303 | 0.000993507 | 0.938396398 | 0.348060401 |
| bcl2l11 | 13621 | PDI_H | -0.000856745 | 0.00091338 | -0.937993845 | 0.348264219 |
| hk2 | 13388 | PDI_H | -0.000873212 | 0.000931405 | -0.937521806 | 0.34850714 |
| egfl7 | 13308 | PDI_H | -0.000840197 | 0.000896562 | -0.93713265 | 0.34870735 |
| rtkn2 | 11355 | PDI_H | -0.000948281 | 0.001012571 | -0.936507637 | 0.34903182 |
| nme1 | 11411 | PDI_H | 0.000942892 | 0.001007134 | 0.936212658 | 0.34918354 |
| mnat1 | 11570 | PDI_H | 0.000938376 | 0.001002538 | 0.936000309 | 0.349292585 |
| espl1 | 11304 | PDI_H | 0.000948478 | 0.001015424 | 0.934070661 | 0.350287418 |
| lipf | 11474 | PDI_H | 0.000916202 | 0.000981099 | 0.933852769 | 0.350399517 |
| mapk9 | 13621 | PDI_H | -0.000852172 | 0.000913609 | -0.932753742 | 0.350963678 |
| grap2 | 13618 | PDI_H | -0.000859992 | 0.000922571 | -0.932168463 | 0.351266011 |
| serping1 | 11790 | PDI_H | -0.000903063 | 0.000969132 | -0.931826723 | 0.351445182 |
| trim58 | 11730 | PDI_H | -0.000921733 | 0.000990224 | -0.930832071 | 0.351959613 |
| ccdc28a | 11483 | PDI_H | -0.000938738 | 0.001009113 | -0.930260701 | 0.352255696 |
| cd300a | 11537 | PDI_H | -0.000905574 | 0.000973899 | -0.92984413 | 0.352471269 |
| gpnmb | 13241 | PDI_H | -0.000857209 | 0.000922173 | -0.929553388 | 0.352619332 |
| ism1 | 13621 | PDI_H | -0.000819091 | 0.000883012 | -0.927610739 | 0.353625976 |
| dcn | 13388 | PDI_H | -0.000806346 | 0.000870269 | -0.926547968 | 0.354177998 |
| clint1 | 11304 | PDI_H | 0.000940529 | 0.001015306 | 0.92635104 | 0.354283377 |
| cadps | 11483 | PDI_H | 0.000934594 | 0.001008941 | 0.926311759 | 0.354303474 |
| fmr1 | 13308 | PDI_H | 0.000863058 | 0.000932523 | 0.925509221 | 0.354717887 |
| cd2ap | 13618 | PDI_H | -0.000843984 | 0.000912061 | -0.925359234 | 0.354795488 |
| crhr1 | 13388 | PDI_H | 0.0008597 | 0.000930713 | 0.923700357 | 0.355659008 |
| eif5 | 11355 | PDI_H | -0.000933277 | 0.001012021 | -0.92219103 | 0.356448555 |
| pdap1 | 11781 | PDI_H | -0.00091413 | 0.000991404 | -0.922055744 | 0.356518401 |
| fn1 | 11817 | PDI_H | -0.000879243 | 0.00095389 | -0.921743893 | 0.356681016 |
| cpox | 11411 | PDI_H | 0.000921652 | 0.001000637 | 0.921065596 | 0.357035673 |
| man1a2 | 11474 | PDI_H | 0.0008905 | 0.000969998 | 0.918043305 | 0.358615512 |
| aoc1 | 13378 | PDI_H | -0.000853078 | 0.000931066 | -0.916238292 | 0.359558456 |
| tpmt | 13308 | PDI_H | -0.000854243 | 0.000932343 | -0.91623315 | 0.359561239 |
| prnd | 11767 | PDI_H | 0.000901871 | 0.000985444 | 0.915192067 | 0.360109581 |
| sdc4 | 13618 | PDI_H | 0.00084241 | 0.000920917 | 0.914751133 | 0.360338504 |
| btla | 11304 | PDI_H | -0.000927855 | 0.001015255 | -0.913912721 | 0.360782222 |
| dok2 | 13618 | PDI_H | -0.000840757 | 0.000921071 | -0.912804139 | 0.361361734 |
| cracr2a | 13308 | PDI_H | -0.000849932 | 0.000931712 | -0.912225467 | 0.36166658 |
| eno2 | 13784 | PDI_H | -0.000835127 | 0.000915549 | -0.912160186 | 0.361700366 |
| klrk1 | 11767 | PDI_H | -0.000904328 | 0.000991618 | -0.911972393 | 0.361801952 |
| yes1 | 13579 | PDI_H | -0.000840411 | 0.000921836 | -0.911670103 | 0.361958605 |
| sgsh | 11586 | PDI_H | -0.000885407 | 0.00097153 | -0.911352635 | 0.362128582 |
| bag4 | 11411 | PDI_H | -0.000907697 | 0.000996653 | -0.910746118 | 0.362448416 |
| dnajc6 | 11474 | PDI_H | -0.000913397 | 0.001002979 | -0.910684207 | 0.362480938 |
| pcdh17 | 13618 | PDI_H | 0.000823479 | 0.000906216 | 0.908701342 | 0.363523891 |
| snca | 11537 | PDI_H | -0.000905232 | 0.000997225 | -0.907750354 | 0.364029113 |
| gimap8 | 11304 | PDI_H | -0.000907311 | 0.001000425 | -0.906925726 | 0.364465428 |
| fkbpl | 11724 | PDI_H | -0.000899526 | 0.000992724 | -0.906119001 | 0.36489151 |

| Protein | N_used | term | beta | se | t | p_val |
|---|---|---|---|---|---|---|
| arntl | 11355 | PDI_H | -0.00091673 | 0.001011911 | -0.905939534 | 0.364987101 |
| fadd | 13618 | PDI_H | -0.000834235 | 0.000922289 | -0.904526918 | 0.365732083 |
| serpinh1 | 11570 | PDI_H | -0.000902269 | 0.000997702 | -0.904346603 | 0.365830492 |
| sh2b3 | 13308 | PDI_H | -0.000839816 | 0.000928789 | -0.904205208 | 0.365902982 |
| cd2 | 11355 | PDI_H | -0.000911709 | 0.001009045 | -0.90353674 | 0.366260286 |
| mars1 | 11517 | PDI_H | -0.00090542 | 0.001003476 | -0.902284125 | 0.366924845 |
| dtnb | 11704 | PDI_H | -0.000835994 | 0.000927856 | -0.900995149 | 0.367609457 |
| esyt2 | 11767 | PDI_H | -0.000889511 | 0.000987886 | -0.900418334 | 0.367916114 |
| dmd | 11474 | PDI_H | -0.000894053 | 0.00099297 | -0.900382458 | 0.367935669 |
| cdh23 | 11767 | PDI_H | 0.000888901 | 0.00098788 | 0.899806986 | 0.368241408 |
| il32 | 13621 | PDI_H | 0.000803735 | 0.000893932 | 0.899101032 | 0.368614756 |
| spring1 | 11304 | PDI_H | -0.000911355 | 0.001013778 | -0.898969091 | 0.368688293 |
| arid4b | 13320 | PDI_H | -0.000838792 | 0.000933073 | -0.898956597 | 0.368692044 |
| atp6v1d | 13579 | PDI_H | -0.000830842 | 0.000925181 | -0.898031901 | 0.369184479 |
| arhgap30 | 11570 | PDI_H | -0.00089932 | 0.001001837 | -0.897670623 | 0.369379874 |
| txk | 11570 | PDI_H | -0.000897786 | 0.001001576 | -0.896373053 | 0.370072223 |
| art5 | 11383 | PDI_H | -0.000905518 | 0.00101068 | -0.895949672 | 0.370298609 |
| angpt1 | 13637 | PDI_H | 0.000823663 | 0.000920146 | 0.895143148 | 0.370726384 |
| lpcat2 | 13579 | PDI_H | -0.000818663 | 0.000914607 | -0.895098048 | 0.370750557 |
| myoc | 13632 | PDI_H | -0.00080018 | 0.000894285 | -0.89477106 | 0.370925295 |
| dctn1 | 13753 | PDI_H | -0.000818119 | 0.000914992 | -0.894126497 | 0.371269874 |
| itih5 | 11730 | PDI_H | 0.000889647 | 0.000995545 | 0.893628397 | 0.3715391 |
| thrap3 | 11355 | PDI_H | 0.000902957 | 0.001010702 | 0.893395716 | 0.37166425 |
| pdzd2 | 11355 | PDI_H | -0.000900757 | 0.001008361 | -0.8932881 | 0.37172186 |
| pglyrp4 | 11355 | PDI_H | 0.000893839 | 0.001001244 | 0.892728649 | 0.372021443 |
| rras | 11483 | PDI_H | -0.000896895 | 0.001006186 | -0.891381653 | 0.372743154 |
| cdkn2d | 13579 | PDI_H | -0.000822564 | 0.000923931 | -0.890287236 | 0.37332746 |
| nudt10 | 11474 | PDI_H | -0.000896607 | 0.001008236 | -0.88928268 | 0.37386984 |
| mrpl46 | 13320 | PDI_H | -0.000830067 | 0.000933791 | -0.888921028 | 0.374061591 |
| clul1 | 13601 | PDI_H | 0.000768295 | 0.000864859 | 0.888346746 | 0.374369986 |
| dcxr | 13699 | PDI_H | -0.000737598 | 0.000831085 | -0.887511949 | 0.374818931 |
| yod1 | 11355 | PDI_H | -0.000876493 | 0.000990619 | -0.884793488 | 0.376286777 |
| fetub | 13669 | PDI_H | 0.000767607 | 0.00086909 | 0.88323093 | 0.37712706 |
| snap23 | 13618 | PDI_H | -0.000811201 | 0.000919185 | -0.882522852 | 0.377509719 |
| aldh3a1 | 13604 | PDI_H | -0.000773966 | 0.000877179 | -0.88233557 | 0.37761097 |
| nos1 | 13320 | PDI_H | -0.000761682 | 0.000864472 | -0.881095313 | 0.378282146 |
| col4a4 | 11554 | PDI_H | -0.000884132 | 0.001003455 | -0.88108728 | 0.378288928 |
| palm | 11643 | PDI_H | -0.000789798 | 0.000896764 | -0.880719022 | 0.378488117 |
| btc | 13308 | PDI_H | -0.00081777 | 0.000929879 | -0.879436627 | 0.379180474 |
| prl | 13784 | PDI_H | 0.000786361 | 0.00089428 | 0.879322994 | 0.379241514 |
| evi2b | 11304 | PDI_H | -0.000892334 | 0.001014957 | -0.879184285 | 0.379320072 |
| vstm2b | 11570 | PDI_H | -0.000879378 | 0.001002575 | -0.877119332 | 0.380440056 |
| gstm4 | 11355 | PDI_H | -0.000886221 | 0.001011166 | -0.876435186 | 0.380812057 |
| arhgap1 | 13308 | PDI_H | -0.000813881 | 0.000929163 | -0.875929157 | 0.381084371 |
| vit | 11781 | PDI_H | -0.000776915 | 0.000887088 | -0.875804247 | 0.381154335 |
| phactr2 | 11757 | PDI_H | -0.000870081 | 0.00099369 | -0.875605606 | 0.381262383 |
| nt5c | 11781 | PDI_H | -0.000867459 | 0.000991925 | -0.874520708 | 0.381852593 |
| paip2b | 11271 | PDI_H | -0.000888348 | 0.001015851 | -0.8744866 | 0.381871967 |
| serpina6 | 11817 | PDI_H | 0.000768873 | 0.000879808 | 0.873909666 | 0.382185227 |
| cd3e | 11570 | PDI_H | -0.000875096 | 0.001003455 | -0.872082193 | 0.383181655 |
| optc | 13579 | PDI_H | -0.000746186 | 0.000856211 | -0.871497233 | 0.383498137 |
| tagln3 | 11304 | PDI_H | -0.000885283 | 0.001015931 | -0.871400865 | 0.38355384 |
| cnst | 13618 | PDI_H | -0.000801853 | 0.000921138 | -0.870502726 | 0.384041088 |
| fgf19 | 13710 | PDI_H | 0.000791804 | 0.000909902 | 0.870208119 | 0.384201929 |
| draxin | 13552 | PDI_H | -0.00078349 | 0.000901081 | -0.869499563 | 0.38458936 |
| edem2 | 11355 | PDI_H | -0.000878309 | 0.00101217 | -0.867747992 | 0.385550664 |
| lgals8 | 13784 | PDI_H | -0.000791521 | 0.000913259 | -0.866698678 | 0.386122229 |
| tnc | 13689 | PDI_H | -0.000790282 | 0.000913321 | -0.865283976 | 0.386898121 |
| fcgr3b | 13640 | PDI_H | -0.000786154 | 0.00090934 | -0.86453163 | 0.387311129 |
| trim40 | 11483 | PDI_H | -0.000869687 | 0.001006578 | -0.864003388 | 0.387604095 |
| necap2 | 11355 | PDI_H | -0.00087173 | 0.001009176 | -0.863803914 | 0.387713882 |
| gli2 | 11411 | PDI_H | 0.0008682 | 0.001006671 | 0.862446886 | 0.388459793 |
| sh3gl3 | 11493 | PDI_H | 0.000866849 | 0.001005884 | 0.861778461 | 0.388827438 |
| f11r | 13632 | PDI_H | -0.000785839 | 0.000913121 | -0.860607456 | 0.389469427 |
| ptpn9 | 11397 | PDI_H | -0.000868102 | 0.001008746 | -0.860575908 | 0.389489781 |
| pdrg1 | 11483 | PDI_H | -0.000867299 | 0.001008573 | -0.859926397 | 0.389847588 |
| galnt10 | 13579 | PDI_H | -0.000769961 | 0.000895897 | -0.859429776 | 0.390118633 |
| arhgef1 | 11767 | PDI_H | -0.000849642 | 0.000989371 | -0.858770023 | 0.39048492 |
| snx2 | 11730 | PDI_H | -0.000853129 | 0.000993731 | -0.858511754 | 0.390627503 |
| gp5 | 11537 | PDI_H | 0.000862367 | 0.00100517 | 0.857931545 | 0.390948105 |

| Protein | N_used | term | beta | se | t | p_val |
|---|---|---|---|---|---|---|
| vps28 | 11570 | PDI_H | -0.000858354 | 0.001002183 | -0.856484402 | 0.391747653 |
| atxn2l | 11757 | PDI_H | -0.000849804 | 0.000992251 | -0.856440818 | 0.391771468 |
| igsf3 | 13579 | PDI_H | -0.000725892 | 0.000848729 | -0.855269099 | 0.392417294 |
| sult1a1 | 13784 | PDI_H | -0.000783914 | 0.000916757 | -0.855095198 | 0.392513323 |
| dctn6 | 13320 | PDI_H | -0.000796135 | 0.000931737 | -0.8544635 | 0.392863606 |
| ctrc | 13658 | PDI_H | 0.000774283 | 0.000907296 | 0.85339617 | 0.393454624 |
| megf11 | 11355 | PDI_H | -0.000859903 | 0.001007811 | -0.853238393 | 0.393545133 |
| htra2 | 13699 | PDI_H | -0.000783495 | 0.000918526 | -0.852992404 | 0.393678443 |
| ccn4 | 13753 | PDI_H | -0.000762015 | 0.000894 | -0.852366264 | 0.394025694 |
| adamtsl5 | 11781 | PDI_H | 0.000839337 | 0.000984794 | 0.852297012 | 0.394066607 |
| ebag9 | 13552 | PDI_H | -0.000785531 | 0.000921815 | -0.852157339 | 0.394141843 |
| dpep2 | 13579 | PDI_H | -0.000780812 | 0.00091704 | -0.851448562 | 0.394535254 |
| anxa11 | 13593 | PDI_H | -0.00078591 | 0.000923057 | -0.851421174 | 0.394550446 |
| cmc1 | 11781 | PDI_H | -0.000843424 | 0.000991558 | -0.850604461 | 0.395006415 |
| tf | 11817 | PDI_H | -0.000832202 | 0.000979203 | -0.849877171 | 0.395410615 |
| myo9b | 13378 | PDI_H | -0.000790761 | 0.000930471 | -0.84984946 | 0.39542401 |
| fcn1 | 11763 | PDI_H | -0.000817081 | 0.000961678 | -0.849641428 | 0.395541781 |
| rpa2 | 11411 | PDI_H | 0.000855093 | 0.00100876 | 0.847667139 | 0.39664117 |
| rab2b | 11730 | PDI_H | -0.000843837 | 0.000995932 | -0.847283156 | 0.396854617 |
| gage2a | 11304 | PDI_H | -0.000859386 | 0.001014599 | -0.847020146 | 0.397001843 |
| uros | 11483 | PDI_H | 0.000852327 | 0.001006951 | 0.846443339 | 0.397323127 |
| il4 | 13378 | PDI_H | -0.000787582 | 0.000932046 | -0.845004103 | 0.398123673 |
| arf6 | 11724 | PDI_H | -0.000839353 | 0.000993468 | -0.844871659 | 0.398199757 |
| fgf7 | 11304 | PDI_H | -0.000856776 | 0.001014325 | -0.84467527 | 0.398310066 |
| lhb | 13308 | PDI_H | -0.00073872 | 0.000874871 | -0.844375334 | 0.398474893 |
| clasp1 | 11304 | PDI_H | -0.000856601 | 0.001015136 | -0.843828764 | 0.398782975 |
| acadm | 11570 | PDI_H | -0.000844072 | 0.001002689 | -0.841808252 | 0.399912709 |
| clic5 | 11483 | PDI_H | -0.000846349 | 0.001006022 | -0.841283247 | 0.40020681 |
| ift20 | 11483 | PDI_H | -0.000846792 | 0.001007153 | -0.840777795 | 0.400489954 |
| atxn10 | 13320 | PDI_H | -0.000784889 | 0.000934226 | -0.840148924 | 0.400839985 |
| add1 | 11537 | PDI_H | -0.000837987 | 0.000997782 | -0.839850502 | 0.401009635 |
| eif1ax | 11767 | PDI_H | -0.000832387 | 0.00099182 | -0.839251379 | 0.401345328 |
| tbc1d23 | 13579 | PDI_H | -0.000775059 | 0.000923857 | -0.838938992 | 0.401518327 |
| nptxr | 13618 | PDI_H | 0.000748425 | 0.000892494 | 0.838577015 | 0.401721445 |
| timm10 | 11781 | PDI_H | -0.000829876 | 0.000991776 | -0.836757375 | 0.402745951 |
| rap1a | 11411 | PDI_H | -0.00083985 | 0.001003792 | -0.836677113 | 0.402791627 |
| naprt | 11767 | PDI_H | 0.000816975 | 0.000976734 | 0.836435687 | 0.402926846 |
| gmfg | 11781 | PDI_H | -0.000828894 | 0.000991836 | -0.835716998 | 0.403331096 |
| serpinb9 | 13552 | PDI_H | -0.000771609 | 0.000923978 | -0.835094856 | 0.403679037 |
| cpa1 | 13800 | PDI_H | -0.000760385 | 0.000910944 | -0.83472148 | 0.403889007 |
| casp3 | 13800 | PDI_H | -0.000763148 | 0.000915194 | -0.833864642 | 0.404371709 |
| atxn2 | 11383 | PDI_H | -0.000839964 | 0.001007404 | -0.833790699 | 0.404416452 |
| rnf31 | 11411 | PDI_H | 0.000840527 | 0.001008498 | 0.833444611 | 0.404611487 |
| mln | 13621 | PDI_H | 0.000714647 | 0.000858085 | 0.832839429 | 0.404949909 |
| snu13 | 11355 | PDI_H | -0.000839627 | 0.001010176 | -0.831168779 | 0.405895785 |
| unc5d | 11411 | PDI_H | -0.000825397 | 0.000995048 | -0.829504742 | 0.406836225 |
| lrp2bp | 11483 | PDI_H | 0.000835846 | 0.001007976 | 0.829231807 | 0.406990505 |
| cdc42bpb | 11724 | PDI_H | -0.000823413 | 0.000995256 | -0.827338137 | 0.40806229 |
| gp2 | 13220 | PDI_H | 0.000740383 | 0.000896989 | 0.825408945 | 0.409154366 |
| clec2l | 11483 | PDI_H | 0.000831746 | 0.001007783 | 0.825322191 | 0.409205859 |
| prkcq | 13378 | PDI_H | -0.000768961 | 0.000931876 | -0.825175155 | 0.409286883 |
| mocs2 | 11537 | PDI_H | -0.000821999 | 0.000997775 | -0.823832019 | 0.41005206 |
| uxs1 | 13308 | PDI_H | -0.000756439 | 0.000918409 | -0.823641122 | 0.410158276 |
| nek7 | 11570 | PDI_H | -0.000826102 | 0.001003348 | -0.823345187 | 0.410328712 |
| nfkb1 | 11355 | PDI_H | -0.000827975 | 0.001006556 | -0.822582039 | 0.410763008 |
| scgn | 13378 | PDI_H | -0.000751708 | 0.000914491 | -0.821995861 | 0.411093915 |
| cdc27 | 13308 | PDI_H | -0.000764866 | 0.000930572 | -0.821930723 | 0.411131065 |
| tnfaip8l2 | 11537 | PDI_H | -0.000824329 | 0.001003233 | -0.821672511 | 0.411280305 |
| crkl | 13676 | PDI_H | -0.000753888 | 0.000918517 | -0.820766497 | 0.411793604 |
| f2r | 13691 | PDI_H | -0.000752986 | 0.000917549 | -0.82065008 | 0.411859914 |
| tnfsf11 | 13621 | PDI_H | 0.000742636 | 0.000905247 | 0.820368394 | 0.412020496 |
| apoa1 | 11817 | PDI_H | 0.000751357 | 0.000915898 | 0.820349718 | 0.412033335 |
| lgals3bp | 11817 | PDI_H | -0.000762877 | 0.00093179 | -0.818722042 | 0.412961545 |
| f2 | 11817 | PDI_H | 0.00080628 | 0.000986222 | 0.817544196 | 0.413634003 |
| cd46 | 13545 | PDI_H | -0.000737361 | 0.00090221 | -0.817283242 | 0.413780969 |
| map2k1 | 11724 | PDI_H | -0.000809024 | 0.00099094 | -0.816420575 | 0.414276238 |
| lilra2 | 13632 | PDI_H | -0.000714008 | 0.000875092 | -0.815923009 | 0.41455844 |
| cryzl1 | 11474 | PDI_H | -0.000819604 | 0.001006195 | -0.814557087 | 0.415342806 |
| csf2ra | 13552 | PDI_H | -0.000745257 | 0.00091586 | -0.813724088 | 0.415817341 |
| pnma1 | 11474 | PDI_H | -0.000818162 | 0.001006245 | -0.813084508 | 0.416186496 |

| Protein | N_used | term | beta | se | t | p_val |
|---|---|---|---|---|---|---|
| cd69 | 13550 | PDI_H | -0.000751434 | 0.000924756 | -0.812575234 | 0.416475924 |
| tmprss11b | 11383 | PDI_H | 0.000820584 | 0.001010132 | 0.812353565 | 0.416605789 |
| brd1 | 11570 | PDI_H | 0.000811569 | 0.001001967 | 0.809976346 | 0.417970446 |
| il36a | 11411 | PDI_H | 0.000816254 | 0.001008411 | 0.809445959 | 0.418275571 |
| tdo2 | 11483 | PDI_H | -0.000807103 | 0.000997727 | -0.80894165 | 0.418565487 |
| dnlz | 11383 | PDI_H | 0.000816012 | 0.001010562 | 0.807483032 | 0.419405137 |
| cdon | 13677 | PDI_H | 0.000728263 | 0.000903163 | 0.806347155 | 0.420056741 |
| bola2_bola2b | 11474 | PDI_H | -0.000800851 | 0.000994524 | -0.80526034 | 0.420686167 |
| mavs | 13308 | PDI_H | -0.000750049 | 0.000931598 | -0.805120727 | 0.420764413 |
| metap1 | 13320 | PDI_H | 0.000751526 | 0.000934358 | 0.804323498 | 0.421224538 |
| nck2 | 13710 | PDI_H | -0.000737431 | 0.000918262 | -0.803072114 | 0.421946988 |
| nubp1 | 11730 | PDI_H | 0.00079785 | 0.00099554 | 0.801424697 | 0.422902073 |
| frzb | 13503 | PDI_H | -0.000718394 | 0.000896537 | -0.801298732 | 0.422972837 |
| reg3g | 11730 | PDI_H | -0.000789809 | 0.000987165 | -0.800077998 | 0.423681827 |
| dok1 | 11767 | PDI_H | -0.000795633 | 0.000994677 | -0.799890089 | 0.423790644 |
| plxna4 | 13621 | PDI_H | -0.0007349 | 0.000920391 | -0.798465341 | 0.424614432 |
| nxph1 | 13320 | PDI_H | 0.000735751 | 0.000925273 | 0.795171677 | 0.426527826 |
| il20ra | 13378 | PDI_H | -0.00074117 | 0.000932406 | -0.794900136 | 0.42668571 |
| mrpl28 | 11757 | PDI_H | 0.000791494 | 0.000996069 | 0.794617992 | 0.426851805 |
| calb2 | 13320 | PDI_H | -0.000677849 | 0.000854971 | -0.792832834 | 0.427889358 |
| ddx39a | 11411 | PDI_H | 0.000798393 | 0.001008964 | 0.79129993 | 0.428785453 |
| aldh5a1 | 11757 | PDI_H | -0.000785251 | 0.000992697 | -0.791028599 | 0.428943276 |
| trdmt1 | 11570 | PDI_H | -0.000792702 | 0.001002866 | -0.79043649 | 0.429289116 |
| smad3 | 11730 | PDI_H | 0.000787529 | 0.000996453 | 0.790332677 | 0.429349502 |
| ndrg1 | 13320 | PDI_H | -0.000736395 | 0.000932196 | -0.789957396 | 0.429566725 |
| pikfyve | 11411 | PDI_H | -0.000792114 | 0.001003155 | -0.789623172 | 0.429764296 |
| plxnb2 | 13735 | PDI_H | 0.000689167 | 0.000874673 | 0.787913941 | 0.430760655 |
| clspn | 13303 | PDI_H | -0.00073484 | 0.000934636 | -0.786231446 | 0.431745924 |
| leo1 | 11757 | PDI_H | -0.000776985 | 0.000989445 | -0.785273479 | 0.432309082 |
| ncan | 13784 | PDI_H | -0.000640706 | 0.000816174 | -0.78501217 | 0.432459936 |
| klk1 | 13542 | PDI_H | -0.000720566 | 0.000918272 | -0.784697441 | 0.432644722 |
| csf2 | 11355 | PDI_H | -0.000779625 | 0.000993883 | -0.784423132 | 0.432808251 |
| cd58 | 13643 | PDI_H | 0.000716022 | 0.000913656 | 0.783688886 | 0.433236303 |
| apod | 11285 | PDI_H | 0.000734003 | 0.000937681 | 0.782786024 | 0.433769219 |
| uhrf2 | 11355 | PDI_H | -0.000791379 | 0.001012232 | -0.781816073 | 0.434338992 |
| mitd1 | 13552 | PDI_H | -0.000720288 | 0.000922803 | -0.780543892 | 0.435084448 |
| rtbdn | 13579 | PDI_H | 0.000702409 | 0.000900867 | 0.779703493 | 0.435579023 |
| gp1bb | 11355 | PDI_H | -0.000787177 | 0.001009861 | -0.779490352 | 0.435707182 |
| map2k6 | 13621 | PDI_H | -0.000717327 | 0.000921727 | -0.778242711 | 0.436439472 |
| nup50 | 11355 | PDI_H | 0.00078626 | 0.001012473 | 0.776573862 | 0.43742642 |
| rab44 | 11730 | PDI_H | -0.000766762 | 0.000987547 | -0.776430949 | 0.437510248 |
| sarg | 11723 | PDI_H | -0.000768623 | 0.00099167 | -0.77507938 | 0.438308405 |
| denr | 11411 | PDI_H | -0.000779327 | 0.00100834 | -0.772880478 | 0.439609145 |
| ccs | 13552 | PDI_H | -0.000704558 | 0.000913152 | -0.771566821 | 0.440384497 |
| spaca5_spaca5b | 11757 | PDI_H | -0.000749415 | 0.000971608 | -0.771314089 | 0.4405363 |
| arg2 | 11730 | PDI_H | -0.000764893 | 0.000992656 | -0.770551737 | 0.440988213 |
| mfap3 | 13388 | PDI_H | -0.00071737 | 0.000931084 | -0.770467327 | 0.441036337 |
| caly | 11355 | PDI_H | -0.000779229 | 0.001011745 | -0.770183327 | 0.441207193 |
| bag6 | 13388 | PDI_H | -0.000711019 | 0.000923866 | -0.769612548 | 0.44154335 |
| epha4 | 11537 | PDI_H | 0.000768359 | 0.001000198 | 0.768206797 | 0.442380076 |
| fyb1 | 13597 | PDI_H | -0.000705848 | 0.000919021 | -0.768042938 | 0.442475026 |
| calb1 | 13579 | PDI_H | -0.000682267 | 0.000889688 | -0.766861692 | 0.443177096 |
| prcp | 13712 | PDI_H | -0.00064114 | 0.000836241 | -0.76669337 | 0.443277057 |
| flt3lg | 13801 | PDI_H | -0.000669326 | 0.000873232 | -0.766491791 | 0.443396856 |
| hexim1 | 13621 | PDI_H | -0.000701212 | 0.000915137 | -0.766236731 | 0.443548747 |
| ppie | 11570 | PDI_H | 0.000766538 | 0.0010028 | 0.764397922 | 0.444645752 |
| spint3 | 11724 | PDI_H | -0.000471635 | 0.000617198 | -0.76415437 | 0.444790649 |
| tex33 | 11570 | PDI_H | -0.00076553 | 0.001002892 | -0.763321794 | 0.445287087 |
| scn4b | 11474 | PDI_H | -0.000741131 | 0.000971589 | -0.762803108 | 0.445596525 |
| psmg4 | 11411 | PDI_H | -0.000757032 | 0.000992511 | -0.762743689 | 0.445632053 |
| prg3 | 11790 | PDI_H | -0.000749145 | 0.000983112 | -0.762013285 | 0.446067334 |
| igfbp2 | 13777 | PDI_H | -0.000602365 | 0.000793038 | -0.759565972 | 0.447527073 |
| ogfr | 13579 | PDI_H | -0.000680937 | 0.000897083 | -0.759057094 | 0.447831591 |
| rab33a | 11355 | PDI_H | -0.000766783 | 0.001011577 | -0.758007694 | 0.448462124 |
| tubb3 | 11383 | PDI_H | 0.000766188 | 0.001011646 | 0.757367709 | 0.44884529 |
| irag2 | 13388 | PDI_H | -0.000703509 | 0.00092968 | -0.756722112 | 0.449229693 |
| megf9 | 13750 | PDI_H | -0.000676766 | 0.000894807 | -0.756327079 | 0.449466086 |
| pth | 11483 | PDI_H | -0.000736796 | 0.000974327 | -0.756210261 | 0.449538671 |
| pgd | 11781 | PDI_H | -0.000740055 | 0.000978891 | -0.756013562 | 0.449656196 |
| klrb1 | 13801 | PDI_H | -0.000672164 | 0.000890986 | -0.754404715 | 0.450619124 |

| Protein | N_used | term | beta | se | t | p_val |
|---|---|---|---|---|---|---|
| sap18 | 11570 | PDI_H | -0.00075647 | 0.001003711 | -0.75367273 | 0.451061113 |
| cdh5 | 13800 | PDI_H | 0.000687211 | 0.000912081 | 0.753453614 | 0.451190241 |
| atp6v1g1 | 11474 | PDI_H | -0.00075569 | 0.001003672 | -0.752925994 | 0.45150985 |
| cd109 | 13632 | PDI_H | -0.000684971 | 0.000909809 | -0.752873431 | 0.451538987 |
| acta2 | 13388 | PDI_H | -0.000619403 | 0.000822833 | -0.752769318 | 0.451601795 |
| gfra2 | 13579 | PDI_H | 0.000691184 | 0.00091974 | 0.751498853 | 0.452365526 |
| rabep1 | 11537 | PDI_H | -0.000751864 | 0.001001435 | -0.750786813 | 0.452796295 |
| mpig6b | 13691 | PDI_H | -0.000690103 | 0.000919571 | -0.750461752 | 0.45298956 |
| entpd5 | 13388 | PDI_H | -0.000688836 | 0.000918179 | -0.750219601 | 0.453135651 |
| pnliprp2 | 13658 | PDI_H | -0.000690614 | 0.000920993 | -0.749858337 | 0.453352957 |
| got1 | 11790 | PDI_H | -0.000725062 | 0.000967093 | -0.749733374 | 0.453430278 |
| dtd1 | 11537 | PDI_H | -0.000751367 | 0.001004324 | -0.748131974 | 0.454395823 |
| swap70 | 11724 | PDI_H | -0.000733345 | 0.000980828 | -0.747679972 | 0.454668226 |
| fbp1 | 13618 | PDI_H | -0.00066028 | 0.00088328 | -0.747532413 | 0.454755164 |
| pnliprp1 | 11767 | PDI_H | -0.000742974 | 0.000994623 | -0.746991193 | 0.455083818 |
| plcb2 | 11767 | PDI_H | -0.000741683 | 0.000993486 | -0.746546408 | 0.455352338 |
| dkk3 | 13800 | PDI_H | -0.000644516 | 0.000865187 | -0.744944073 | 0.456318228 |
| ca8 | 11411 | PDI_H | 0.000750411 | 0.001007763 | 0.744630939 | 0.45651021 |
| cuzd1 | 11483 | PDI_H | -0.000749489 | 0.001007835 | -0.743662933 | 0.457095651 |
| prss27 | 13568 | PDI_H | -0.000685446 | 0.000922415 | -0.743098991 | 0.457434628 |
| nfkb2 | 11570 | PDI_H | -0.000743401 | 0.001003358 | -0.740913022 | 0.458761232 |
| tex101 | 11483 | PDI_H | 0.000517612 | 0.000698945 | 0.740561203 | 0.458974697 |
| lyve1 | 11817 | PDI_H | 0.000715821 | 0.000967205 | 0.740092093 | 0.459258834 |
| fkbp14 | 11757 | PDI_H | -0.000735249 | 0.000993859 | -0.73979224 | 0.459440855 |
| tyrp1 | 11411 | PDI_H | -0.000741027 | 0.001001739 | -0.739740018 | 0.459472995 |
| cdk1 | 11304 | PDI_H | -0.000748664 | 0.001013297 | -0.738839537 | 0.460019797 |
| siglec8 | 11474 | PDI_H | -0.000715064 | 0.000969132 | -0.73783977 | 0.460626928 |
| ywhaq | 11537 | PDI_H | -0.000739026 | 0.001003043 | -0.736783979 | 0.461268725 |
| gamt | 11355 | PDI_H | -0.000745486 | 0.001012089 | -0.736581055 | 0.461392393 |
| zp4 | 11304 | PDI_H | -0.00074674 | 0.001014025 | -0.736411964 | 0.461495324 |
| kiaa2013 | 11730 | PDI_H | 0.000732926 | 0.000995698 | 0.736092119 | 0.461689373 |
| lto1 | 13378 | PDI_H | -0.000685463 | 0.000931879 | -0.735571195 | 0.462004605 |
| spag1 | 11757 | PDI_H | 0.000729735 | 0.000993206 | 0.734727319 | 0.462520248 |
| snap25 | 11383 | PDI_H | -0.000733084 | 0.000998789 | -0.733972831 | 0.46298043 |
| plin1 | 13320 | PDI_H | -0.000624716 | 0.000851295 | -0.733841413 | 0.463058325 |
| fgf20 | 11411 | PDI_H | 0.00073848 | 0.001008402 | 0.732326838 | 0.463984159 |
| cetn3 | 11724 | PDI_H | -0.000727565 | 0.000993544 | -0.732292432 | 0.464004751 |
| msln | 13555 | PDI_H | -0.000645435 | 0.000883607 | -0.730454526 | 0.465125035 |
| clc | 13593 | PDI_H | -0.000661187 | 0.000906513 | -0.729374654 | 0.465785096 |
| pcdh9 | 11724 | PDI_H | 0.000706854 | 0.000969583 | 0.729028635 | 0.465998724 |
| ca13 | 13472 | PDI_H | -0.000675507 | 0.000927277 | -0.728484781 | 0.466329555 |
| vps4b | 11730 | PDI_H | -0.000723965 | 0.00099534 | -0.72735455 | 0.467023321 |
| st3gal1 | 13579 | PDI_H | 0.000635167 | 0.000873797 | 0.726904448 | 0.467297034 |
| padi2 | 13378 | PDI_H | 0.000670237 | 0.000924415 | 0.725039491 | 0.468440496 |
| lcn15 | 11757 | PDI_H | -0.000710679 | 0.000983374 | -0.722693873 | 0.469882371 |
| srpx | 11781 | PDI_H | 0.000660998 | 0.000916047 | 0.721576205 | 0.470569408 |
| atp1b1 | 11355 | PDI_H | 0.000724466 | 0.001010303 | 0.717077467 | 0.473341064 |
| ddi2 | 11537 | PDI_H | -0.00071177 | 0.000993703 | -0.716280378 | 0.473832754 |
| clec12a | 11411 | PDI_H | -0.000717982 | 0.001003155 | -0.715724073 | 0.474176404 |
| igdcc3 | 11383 | PDI_H | 0.000723237 | 0.001011085 | 0.715308211 | 0.474433302 |
| tmed4 | 11355 | PDI_H | -0.000723599 | 0.00101237 | -0.71475806 | 0.474773264 |
| pbk | 11304 | PDI_H | -0.000725627 | 0.001015383 | -0.714633818 | 0.474850115 |
| naa80 | 11483 | PDI_H | -0.000717467 | 0.001004042 | -0.714578857 | 0.474883854 |
| slc1a4 | 11483 | PDI_H | -0.000719637 | 0.00100746 | -0.714308314 | 0.475051087 |
| slc4a1 | 11790 | PDI_H | -0.000704074 | 0.000985824 | -0.714197984 | 0.475118917 |
| csf2rb | 11411 | PDI_H | -0.000713747 | 0.001000248 | -0.713569886 | 0.475507793 |
| tmed10 | 11355 | PDI_H | -0.000718413 | 0.00101179 | -0.710041474 | 0.477693014 |
| tnip1 | 11767 | PDI_H | -0.000701815 | 0.000990535 | -0.70852113 | 0.478635745 |
| ivd | 13320 | PDI_H | 0.000662114 | 0.000934683 | 0.708383492 | 0.478719548 |
| vstm2l | 13388 | PDI_H | -0.000652342 | 0.000921289 | -0.708075401 | 0.478910773 |
| pmvk | 13292 | PDI_H | -0.000660711 | 0.000933254 | -0.707964623 | 0.478979652 |
| hdac8 | 11411 | PDI_H | 0.000712719 | 0.001007705 | 0.707269217 | 0.47941365 |
| fgf16 | 11411 | PDI_H | -0.00071165 | 0.001006979 | -0.706717363 | 0.479756582 |
| matn2 | 13677 | PDI_H | -0.000626183 | 0.000888016 | -0.705148072 | 0.480730112 |
| sned1 | 11474 | PDI_H | -0.000702487 | 0.000997231 | -0.704437796 | 0.481174487 |
| flt3 | 13308 | PDI_H | 0.000646192 | 0.000917628 | 0.704198496 | 0.481321501 |
| dctd | 11537 | PDI_H | -0.000705154 | 0.001004818 | -0.701773174 | 0.482834802 |
| casp2 | 13621 | PDI_H | -0.000644883 | 0.000920706 | -0.700421913 | 0.483675829 |
| lztfl1 | 11537 | PDI_H | -0.000702423 | 0.00100301 | -0.700315709 | 0.4837443 |
| kif22 | 11355 | PDI_H | 0.000700634 | 0.001000821 | 0.700059071 | 0.483904772 |

| Protein | N_used | term | beta | se | t | p_val |
|---|---|---|---|---|---|---|
| guk1 | 11355 | PDI_H | -0.00070665 | 0.001011863 | -0.698365221 | 0.484963137 |
| ctsl | 13618 | PDI_H | 0.000619165 | 0.000888566 | 0.696813558 | 0.485931385 |
| ralb | 11411 | PDI_H | -0.0007022 | 0.00100813 | -0.696537184 | 0.486106682 |
| ccn3 | 13777 | PDI_H | -0.00057305 | 0.000823367 | -0.69598366 | 0.486450814 |
| ceacam6 | 11790 | PDI_H | -0.000675134 | 0.000970351 | -0.695762887 | 0.486591063 |
| postn | 11790 | PDI_H | -0.000684189 | 0.000984738 | -0.694793477 | 0.487198442 |
| pard3 | 11570 | PDI_H | -0.000696148 | 0.001003239 | -0.693900342 | 0.487758654 |
| ppm1f | 11781 | PDI_H | -0.000687383 | 0.000991774 | -0.693084587 | 0.488270146 |
| bst1 | 13784 | PDI_H | -0.000625872 | 0.00090428 | -0.692121153 | 0.488872917 |
| brdt | 11355 | PDI_H | -0.000700513 | 0.001012627 | -0.691777965 | 0.489090936 |
| mtus1 | 11570 | PDI_H | 0.000672645 | 0.000972402 | 0.691735806 | 0.489117151 |
| erc2 | 11483 | PDI_H | -0.000696125 | 0.001008035 | -0.690576972 | 0.489845395 |
| esm1 | 13658 | PDI_H | 0.000611049 | 0.000885135 | 0.6903458 | 0.489988494 |
| pth1r | 13621 | PDI_H | 0.000630374 | 0.000914221 | 0.689519684 | 0.490508047 |
| gas6 | 13707 | PDI_H | 0.000613113 | 0.000894093 | 0.685736647 | 0.4928908 |
| tpm3 | 11355 | PDI_H | -0.000686584 | 0.001002383 | -0.684951606 | 0.493388455 |
| pamr1 | 13632 | PDI_H | -0.000579703 | 0.00084947 | -0.682428488 | 0.494979653 |
| rrm2b | 13308 | PDI_H | -0.000633281 | 0.000928778 | -0.681843199 | 0.495349982 |
| elob | 11355 | PDI_H | -0.00068809 | 0.00100994 | -0.681317675 | 0.495684411 |
| tor1aip1 | 11474 | PDI_H | -0.000681081 | 0.001001351 | -0.680162248 | 0.496415471 |
| sit1 | 13378 | PDI_H | -0.000607391 | 0.000893407 | -0.679859785 | 0.496605021 |
| fuca1 | 13722 | PDI_H | 0.000616127 | 0.000907476 | 0.678946016 | 0.497183529 |
| src | 13672 | PDI_H | -0.000623603 | 0.000920402 | -0.677532899 | 0.498079378 |
| pi16 | 11794 | PDI_H | 0.000651435 | 0.00096289 | 0.676541572 | 0.498710142 |
| paep | 13552 | PDI_H | -0.000598841 | 0.000885295 | -0.676430853 | 0.498778688 |
| itprip | 11383 | PDI_H | 0.000680297 | 0.001007574 | 0.675183158 | 0.499573134 |
| skap1 | 13535 | PDI_H | 0.000608162 | 0.000901075 | 0.674929685 | 0.499731979 |
| kiaa1549l | 11570 | PDI_H | 0.000676282 | 0.001002265 | 0.674753149 | 0.499846107 |
| ism2 | 11790 | PDI_H | 0.000670525 | 0.000994439 | 0.674274254 | 0.500150203 |
| prdx1 | 13290 | PDI_H | -0.000629259 | 0.000933348 | -0.674195201 | 0.500198957 |
| efemp1 | 13663 | PDI_H | -0.000554025 | 0.000822465 | -0.673614706 | 0.500567706 |
| trim25 | 11724 | PDI_H | -0.000670267 | 0.000995929 | -0.673007184 | 0.500955999 |
| ca12 | 13579 | PDI_H | 0.000616288 | 0.000917897 | 0.671413279 | 0.501968719 |
| rhoc | 13477 | PDI_H | -0.000622236 | 0.00092681 | -0.671373694 | 0.501994016 |
| nrgn | 11794 | PDI_H | -0.000664768 | 0.000991938 | -0.670170829 | 0.502762032 |
| gpr101 | 11383 | PDI_H | -0.000676604 | 0.001010636 | -0.669484154 | 0.503200279 |
| fgf23 | 13728 | PDI_H | 0.000585741 | 0.000875748 | 0.668846308 | 0.50360478 |
| vps53 | 13579 | PDI_H | -0.000616095 | 0.000922614 | -0.667770444 | 0.504291494 |
| ptprk | 11724 | PDI_H | -0.000657109 | 0.000984066 | -0.667748738 | 0.504307151 |
| b4galt1 | 13710 | PDI_H | -0.000592727 | 0.00088783 | -0.667613823 | 0.504391379 |
| gchfr | 11260 | PDI_H | -0.000633582 | 0.00095351 | -0.664473619 | 0.506400849 |
| timp3 | 13710 | PDI_H | -0.000602533 | 0.000909621 | -0.662399843 | 0.507726148 |
| krt14 | 12884 | PDI_H | -0.000626186 | 0.000947901 | -0.660602518 | 0.508879076 |
| fgf5 | 13378 | PDI_H | 0.000566303 | 0.000862252 | 0.656771959 | 0.511338865 |
| hnf1a | 11483 | PDI_H | 0.000660479 | 0.001008579 | 0.654860634 | 0.512570618 |
| cyth3 | 11304 | PDI_H | 0.000663271 | 0.001014175 | 0.6540009 | 0.513124544 |
| epgn | 11730 | PDI_H | -0.000649346 | 0.000995443 | -0.652319213 | 0.514208064 |
| nit2 | 11730 | PDI_H | -0.000640619 | 0.000983135 | -0.651609018 | 0.514666208 |
| cgb3_cgb5_cgb8 | 11794 | PDI_H | 0.000603584 | 0.000926693 | 0.651331378 | 0.514845301 |
| il36g | 11351 | PDI_H | -0.00064538 | 0.000993616 | -0.649526072 | 0.516011557 |
| ddx53 | 11483 | PDI_H | 0.000654683 | 0.00100838 | 0.64924192 | 0.516195018 |
| pla2g4a | 13710 | PDI_H | -0.000595177 | 0.000919524 | -0.647266187 | 0.517470528 |
| tyro3 | 13618 | PDI_H | 0.000591822 | 0.000914637 | 0.647057023 | 0.517605955 |
| clstn3 | 11767 | PDI_H | 0.000628774 | 0.000971749 | 0.647053588 | 0.517609897 |
| gcnt1 | 13579 | PDI_H | -0.000578784 | 0.000896486 | -0.645614791 | 0.518539775 |
| rbpms | 11411 | PDI_H | -0.000650092 | 0.001007586 | -0.645197749 | 0.51881203 |
| ctsb | 13621 | PDI_H | -0.000573653 | 0.000890372 | -0.644284474 | 0.519401841 |
| col4a1 | 13618 | PDI_H | 0.000573722 | 0.000890732 | 0.644102442 | 0.519519865 |
| tpd52l2 | 11537 | PDI_H | -0.000645546 | 0.001003496 | -0.643297115 | 0.520044127 |
| ccl5 | 13689 | PDI_H | -0.000585758 | 0.000911217 | -0.642829696 | 0.520345388 |
| fkbp1b | 13695 | PDI_H | 0.000591337 | 0.000920027 | 0.642738284 | 0.520404705 |
| atp6ap2 | 13308 | PDI_H | -0.000594066 | 0.000925171 | -0.642115129 | 0.520809504 |
| hpcal1 | 13801 | PDI_H | -0.000587281 | 0.000914644 | -0.642086569 | 0.52082765 |
| rest | 11304 | PDI_H | 0.000651059 | 0.001015331 | 0.641228722 | 0.521387105 |
| ugdh | 11355 | PDI_H | -0.000647956 | 0.001011388 | -0.640660093 | 0.521756489 |
| serpina5 | 11611 | PDI_H | 0.000620421 | 0.000968619 | 0.640521283 | 0.52184641 |
| prtfdc1 | 13320 | PDI_H | -0.000597558 | 0.000933963 | -0.639809918 | 0.522307198 |
| tgfb2 | 11730 | PDI_H | 0.000636254 | 0.000995185 | 0.639333114 | 0.522618748 |
| bgn | 13291 | PDI_H | 0.000587603 | 0.000919786 | 0.638847861 | 0.522932928 |
| daam1 | 11537 | PDI_H | -0.000638811 | 0.001003051 | -0.636867794 | 0.524223612 |

| Protein | N_used | term | beta | se | t | p_val |
|---|---|---|---|---|---|---|
| tgfbr3 | 13800 | PDI_H | 0.00057732 | 0.000907465 | 0.636189953 | 0.524663181 |
| cxcl6 | 13801 | PDI_H | -0.000578266 | 0.000909053 | -0.636119762 | 0.524708924 |
| col9a2 | 11570 | PDI_H | 0.000636678 | 0.001001775 | 0.635550385 | 0.525082099 |
| map3k5 | 13308 | PDI_H | -0.000592243 | 0.000932491 | -0.635119456 | 0.525361438 |
| dnajb6 | 11415 | PDI_H | -0.000640214 | 0.001008019 | -0.635121293 | 0.525362054 |
| tmed8 | 11724 | PDI_H | -0.000632385 | 0.000996163 | -0.634821131 | 0.525557481 |
| vsir | 13593 | PDI_H | -0.000584502 | 0.000920772 | -0.634795915 | 0.525572222 |
| foxj3 | 11411 | PDI_H | -0.000638289 | 0.001005584 | -0.634744288 | 0.525607943 |
| gucy2c | 11570 | PDI_H | -0.000633067 | 0.001000637 | -0.632664688 | 0.52696515 |
| gsta1 | 13593 | PDI_H | -0.000524422 | 0.000829635 | -0.632112288 | 0.527324147 |
| lypd1 | 13320 | PDI_H | 0.000589877 | 0.00093397 | 0.631580224 | 0.527672061 |
| stxbp1 | 11757 | PDI_H | -0.000627882 | 0.000994489 | -0.631361265 | 0.527816624 |
| exosc10 | 11355 | PDI_H | 0.000638615 | 0.001011837 | 0.631143856 | 0.527959185 |
| sytl4 | 11781 | PDI_H | -0.000623933 | 0.000989961 | -0.630260489 | 0.528536406 |
| siae | 13674 | PDI_H | -0.000569382 | 0.000903843 | -0.629957156 | 0.528733151 |
| rbm19 | 11355 | PDI_H | -0.000637438 | 0.001012205 | -0.629752315 | 0.528869333 |
| dynlt3 | 11730 | PDI_H | 0.000625459 | 0.000994087 | 0.629179333 | 0.529243923 |
| myo6 | 11757 | PDI_H | -0.000625934 | 0.000995962 | -0.62847102 | 0.529707646 |
| slc34a3 | 11304 | PDI_H | 0.000637307 | 0.001014707 | 0.628070388 | 0.529970532 |
| eif5a | 13378 | PDI_H | -0.00058324 | 0.000932095 | -0.625729847 | 0.531502837 |
| csrp3 | 11355 | PDI_H | -0.000618096 | 0.00098916 | -0.62486945 | 0.532069314 |
| upk3bl1 | 11544 | PDI_H | 0.000627331 | 0.001004345 | 0.624617373 | 0.532234572 |
| susd1 | 13388 | PDI_H | -0.000578672 | 0.000928985 | -0.622907401 | 0.533355994 |
| ctrl | 11767 | PDI_H | -0.000608404 | 0.000976901 | -0.622789325 | 0.533435055 |
| pcdh7 | 11724 | PDI_H | 0.000608283 | 0.00097674 | 0.622768776 | 0.533448605 |
| hspb6 | 13753 | PDI_H | 0.000473036 | 0.000760662 | 0.621874097 | 0.534034976 |
| asah1 | 11673 | PDI_H | -0.000610377 | 0.000983231 | -0.620787076 | 0.53475186 |
| col9a1 | 13621 | PDI_H | -0.000562314 | 0.000906756 | -0.620137427 | 0.535177697 |
| lcat | 11794 | PDI_H | -0.000578514 | 0.000934304 | -0.61919295 | 0.535801228 |
| fzd10 | 11483 | PDI_H | -0.000623851 | 0.001009062 | -0.6182485 | 0.536423819 |
| cxcl5 | 13571 | PDI_H | -0.000557694 | 0.000902424 | -0.617995667 | 0.536588575 |
| rapgef2 | 11570 | PDI_H | -0.000615777 | 0.001002008 | -0.614543616 | 0.538868257 |
| lrig1 | 13715 | PDI_H | 0.000553362 | 0.000900939 | 0.614205382 | 0.539089814 |
| sez6 | 11483 | PDI_H | -0.000599739 | 0.000977241 | -0.613707035 | 0.539421108 |
| psmd5 | 11483 | PDI_H | 0.000616902 | 0.001007595 | 0.612251379 | 0.540383589 |
| ppp1cc | 11730 | PDI_H | -0.000607917 | 0.000994472 | -0.611296564 | 0.541015124 |
| ccdc134 | 11730 | PDI_H | -0.000606309 | 0.000994984 | -0.609366072 | 0.542293636 |
| timd4 | 13689 | PDI_H | 0.000540166 | 0.000888805 | 0.607743793 | 0.543367503 |
| cd160 | 13801 | PDI_H | -0.000548876 | 0.00090349 | -0.607506628 | 0.54352475 |
| csh1 | 11794 | PDI_H | 0.000602747 | 0.0009934 | 0.60675165 | 0.544027431 |
| nmt1 | 11757 | PDI_H | -0.000603495 | 0.000995233 | -0.606385235 | 0.54427069 |
| brap | 11610 | PDI_H | -0.000605568 | 0.000999559 | -0.605835878 | 0.544635598 |
| atp5if1 | 13710 | PDI_H | -0.000555036 | 0.000917044 | -0.60524491 | 0.545026306 |
| prkd2 | 11355 | PDI_H | -0.000610735 | 0.00101006 | -0.604652368 | 0.545422098 |
| icam4 | 13621 | PDI_H | -0.000554076 | 0.000916423 | -0.604607357 | 0.545449997 |
| cd200 | 13621 | PDI_H | 0.000534992 | 0.000885794 | 0.603968814 | 0.545874443 |
| scarb1 | 13320 | PDI_H | -0.000563385 | 0.000934011 | -0.603188124 | 0.546393827 |
| fam171b | 11730 | PDI_H | -0.000594903 | 0.000988749 | -0.60167195 | 0.547404165 |
| gpc5 | 13552 | PDI_H | 0.000551819 | 0.000919127 | 0.600373331 | 0.548267505 |
| tpsd1 | 11757 | PDI_H | -0.000593426 | 0.00099217 | -0.598108749 | 0.549778906 |
| top1 | 11724 | PDI_H | 0.000583821 | 0.000977345 | 0.597354649 | 0.550282174 |
| pxdnl | 11730 | PDI_H | 0.000590281 | 0.000990235 | 0.596102114 | 0.551118526 |
| nedd4l | 11411 | PDI_H | -0.000597991 | 0.001004327 | -0.595415183 | 0.551577799 |
| lrp1 | 13579 | PDI_H | 0.000526065 | 0.000883974 | 0.595113933 | 0.551777241 |
| agt | 11794 | PDI_H | -0.000582993 | 0.000980069 | -0.594849326 | 0.551955616 |
| il3ra | 13378 | PDI_H | -0.000533655 | 0.000898301 | -0.594071347 | 0.552474448 |
| s100a13 | 11411 | PDI_H | 0.000573915 | 0.00096671 | 0.593678696 | 0.552738815 |
| col3a1 | 11790 | PDI_H | -0.000582636 | 0.000983288 | -0.592537918 | 0.553501813 |
| slc39a14 | 13320 | PDI_H | 0.000542506 | 0.000916215 | 0.592116914 | 0.553782361 |
| nexn | 11794 | PDI_H | 0.000587801 | 0.000993059 | 0.591909369 | 0.553922637 |
| tpsg1 | 11411 | PDI_H | 0.000594187 | 0.001008225 | 0.589339214 | 0.555645424 |
| dusp29 | 11483 | PDI_H | 0.000582665 | 0.000989167 | 0.589046136 | 0.555841924 |
| lmod1 | 11757 | PDI_H | -0.000525222 | 0.000891859 | -0.588906822 | 0.555935107 |
| creb3 | 11304 | PDI_H | -0.000597881 | 0.001015618 | -0.588687 | 0.556083035 |
| tie1 | 13800 | PDI_H | 0.000534432 | 0.000909466 | 0.587632931 | 0.556788325 |
| ostn | 11483 | PDI_H | -0.000589341 | 0.001004229 | -0.586859608 | 0.557309548 |
| nfe2 | 11474 | PDI_H | -0.000589387 | 0.001004633 | -0.586668858 | 0.557437681 |
| gtf2ird1 | 11570 | PDI_H | -0.000588007 | 0.001003217 | -0.586121635 | 0.557805224 |
| klkb1 | 11544 | PDI_H | 0.000563414 | 0.000961566 | 0.585933928 | 0.557931383 |
| mthfd2 | 11767 | PDI_H | -0.000582379 | 0.000994651 | -0.585510218 | 0.558215938 |

| Protein | N_used | term | beta | se | t | p_val |
|---|---|---|---|---|---|---|
| mep1a | 11724 | PDI_H | -0.000558349 | 0.000953983 | -0.585281254 | 0.558369893 |
| izumo1 | 11304 | PDI_H | 0.000585452 | 0.001001552 | 0.584544199 | 0.558865916 |
| aldh2 | 11730 | PDI_H | -0.000579709 | 0.000992108 | -0.584320112 | 0.559016212 |
| gcc1 | 11767 | PDI_H | -0.000578758 | 0.000990982 | -0.58402534 | 0.559214471 |
| tppp2 | 11570 | PDI_H | -0.000585487 | 0.001003591 | -0.583392074 | 0.559640776 |
| dkk4 | 13530 | PDI_H | -0.000525937 | 0.000901822 | -0.583194094 | 0.559772375 |
| angptl3 | 13639 | PDI_H | -0.000489807 | 0.000841249 | -0.582237985 | 0.560416024 |
| cetn2 | 13320 | PDI_H | -0.000542526 | 0.000931879 | -0.582184967 | 0.56045196 |
| pacs2 | 11355 | PDI_H | -0.000586127 | 0.001009059 | -0.580865083 | 0.561342927 |
| skap2 | 13710 | PDI_H | -0.000531648 | 0.000917674 | -0.579343363 | 0.562367039 |
| muc2 | 11790 | PDI_H | -0.000572391 | 0.00098904 | -0.578734248 | 0.56277957 |
| iglon5 | 11304 | PDI_H | 0.000587519 | 0.001015372 | 0.578624776 | 0.562853923 |
| il16 | 13801 | PDI_H | -0.000495822 | 0.000861211 | -0.575727097 | 0.564809052 |
| prkag3 | 11483 | PDI_H | -0.000579732 | 0.001007653 | -0.575328688 | 0.565080308 |
| ctso | 13801 | PDI_H | 0.00050318 | 0.000876569 | 0.57403354 | 0.565954468 |
| lifr | 13621 | PDI_H | 0.000521683 | 0.000909163 | 0.573805842 | 0.566108678 |
| fam171a2 | 11757 | PDI_H | 0.000569559 | 0.000995038 | 0.572399101 | 0.567062581 |
| dclre1c | 11304 | PDI_H | -0.000580465 | 0.001015686 | -0.571500182 | 0.567672012 |
| shmt1 | 13691 | PDI_H | -0.000521474 | 0.000913441 | -0.570889969 | 0.568083609 |
| ubac1 | 13579 | PDI_H | -0.000521269 | 0.000913154 | -0.570844492 | 0.568114515 |
| apobr | 11474 | PDI_H | -0.000569664 | 0.001001246 | -0.568955111 | 0.569397751 |
| tspyl1 | 11411 | PDI_H | -0.000573146 | 0.001007382 | -0.56894566 | 0.569404227 |
| il31ra | 11368 | PDI_H | 0.000571333 | 0.001006901 | 0.567417072 | 0.57044207 |
| klk4 | 13753 | PDI_H | -0.000487025 | 0.000858378 | -0.567378608 | 0.57046625 |
| shpk | 11570 | PDI_H | 0.000567995 | 0.001002915 | 0.566344384 | 0.57117069 |
| plekho1 | 11767 | PDI_H | -0.000561635 | 0.000991704 | -0.56633319 | 0.571178114 |
| lamb1 | 11474 | PDI_H | -0.000562896 | 0.000994678 | -0.565908148 | 0.571467301 |
| ddx4 | 11411 | PDI_H | 0.000570548 | 0.001008402 | 0.565794592 | 0.57154456 |
| arhgef5 | 11383 | PDI_H | 0.000569517 | 0.001007331 | 0.565371848 | 0.571832023 |
| serpinb5 | 13388 | PDI_H | -0.000524571 | 0.0009287 | -0.564843948 | 0.572189384 |
| arl13b | 11355 | PDI_H | -0.000570158 | 0.001009839 | -0.564602985 | 0.572354999 |
| stx3 | 11767 | PDI_H | -0.000560047 | 0.000994726 | -0.563016509 | 0.57343438 |
| tmod4 | 11304 | PDI_H | 0.000571647 | 0.001015436 | 0.562957827 | 0.573474779 |
| stam | 11730 | PDI_H | 0.00056014 | 0.00099579 | 0.562507967 | 0.573780738 |
| abca2 | 11355 | PDI_H | 0.000559965 | 0.000997874 | 0.561157939 | 0.574700959 |
| hbz | 11781 | PDI_H | -0.000557197 | 0.0009932 | -0.561011657 | 0.574800271 |
| galnt7 | 13579 | PDI_H | 0.000513577 | 0.000916406 | 0.560425416 | 0.575198553 |
| stat2 | 11411 | PDI_H | -0.000562253 | 0.00100447 | -0.559751575 | 0.575659897 |
| scg2 | 13692 | PDI_H | 0.000505255 | 0.000902867 | 0.559611679 | 0.575753499 |
| icoslg | 13579 | PDI_H | 0.000506256 | 0.000911733 | 0.555267724 | 0.578720707 |
| eddm3b | 11730 | PDI_H | -0.000356649 | 0.000643152 | -0.554533735 | 0.579224208 |
| syngap1 | 11383 | PDI_H | -0.000560623 | 0.001011068 | -0.554485426 | 0.579257582 |
| gsap | 13308 | PDI_H | 0.000516427 | 0.000932547 | 0.553781801 | 0.579737497 |
| edil3 | 13388 | PDI_H | 0.000488636 | 0.00088302 | 0.553369193 | 0.580019878 |
| efcab2 | 11570 | PDI_H | -0.000553921 | 0.001001553 | -0.55306208 | 0.580231598 |
| casp10 | 13320 | PDI_H | -0.000514135 | 0.000929705 | -0.553008602 | 0.580266809 |
| brsk2 | 11757 | PDI_H | 0.000548918 | 0.000993754 | 0.552368233 | 0.580706602 |
| bmp6 | 13364 | PDI_H | -0.000510931 | 0.000925079 | -0.552311202 | 0.580744402 |
| pfdn4 | 11304 | PDI_H | 0.000560571 | 0.001014997 | 0.552288332 | 0.580761754 |
| gp1ba | 13575 | PDI_H | -0.000507331 | 0.000922123 | -0.550177044 | 0.582207007 |
| spint2 | 13710 | PDI_H | -0.000495842 | 0.000904215 | -0.548367722 | 0.583448372 |
| gad2 | 11411 | PDI_H | 0.000552103 | 0.001007135 | 0.548192223 | 0.583570656 |
| cmip | 11730 | PDI_H | -0.000545078 | 0.000994979 | -0.547828685 | 0.583819975 |
| chmp6 | 11781 | PDI_H | -0.000541213 | 0.000989298 | -0.547068208 | 0.584342246 |
| ass1 | 11355 | PDI_H | -0.000525325 | 0.000961277 | -0.546486846 | 0.584742076 |
| mfge8 | 13632 | PDI_H | 0.000492249 | 0.00090105 | 0.546306205 | 0.58486442 |
| ifngr2 | 13552 | PDI_H | -0.000493161 | 0.000907858 | -0.543214172 | 0.586991293 |
| pla2g7 | 13632 | PDI_H | 0.000479695 | 0.000883109 | 0.54318885 | 0.587008672 |
| hras | 11549 | PDI_H | 0.000543965 | 0.001003837 | 0.541885282 | 0.587908002 |
| lat | 13801 | PDI_H | -0.000492717 | 0.000912344 | -0.540055584 | 0.589167422 |
| lair2 | 13552 | PDI_H | -0.000493906 | 0.000916158 | -0.539105674 | 0.589822805 |
| apoc1 | 11790 | PDI_H | 0.000500628 | 0.000931764 | 0.537290066 | 0.591077417 |
| fga | 11530 | PDI_H | 0.000498917 | 0.000930852 | 0.535978437 | 0.591983803 |
| rarres1 | 13308 | PDI_H | -0.000494146 | 0.000922366 | -0.535737698 | 0.592148807 |
| pcdhb15 | 11730 | PDI_H | 0.000531342 | 0.000992983 | 0.535097256 | 0.592592761 |
| cxcl3 | 13642 | PDI_H | -0.00049142 | 0.000918937 | -0.534770621 | 0.592817204 |
| nlgn2 | 11383 | PDI_H | 0.000540082 | 0.001010829 | 0.534295878 | 0.593147289 |
| klk6 | 13682 | PDI_H | -0.000481974 | 0.000902559 | -0.534007811 | 0.593344813 |
| tlr3 | 13801 | PDI_H | -0.000483807 | 0.000906467 | -0.533728362 | 0.593538087 |
| apoh | 13632 | PDI_H | -0.000487743 | 0.000914652 | -0.53325551 | 0.593865422 |

| Protein | N_used | term | beta | se | t | p_val |
|---|---|---|---|---|---|---|
| reps1 | 11411 | PDI_H | 0.000536745 | 0.001008314 | 0.532319368 | 0.594515196 |
| cgn | 11483 | PDI_H | -0.000536958 | 0.001008846 | -0.53224941 | 0.594563574 |
| il12a_il12b | 13579 | PDI_H | -0.000471383 | 0.000885692 | -0.53221974 | 0.594582528 |
| trpv3 | 11355 | PDI_H | -0.000538783 | 0.001012429 | -0.532168384 | 0.594619801 |
| rab37 | 13378 | PDI_H | -0.000493809 | 0.000927963 | -0.532142576 | 0.594636096 |
| bhmt2 | 11355 | PDI_H | -0.00053845 | 0.001012795 | -0.531647721 | 0.594980418 |
| b3gat3 | 11355 | PDI_H | 0.000537661 | 0.00101229 | 0.531133463 | 0.595336696 |
| smpd3 | 11411 | PDI_H | 0.000532897 | 0.001004057 | 0.530743788 | 0.595606677 |
| tacstd2 | 12097 | PDI_H | -0.000510234 | 0.000963043 | -0.529814549 | 0.59625025 |
| cep170 | 11474 | PDI_H | -0.000532596 | 0.001005854 | -0.529496598 | 0.596471259 |
| anp32c | 11474 | PDI_H | -0.000534198 | 0.001008881 | -0.529495756 | 0.596471843 |
| gngt1 | 11483 | PDI_H | 0.000531609 | 0.001007198 | 0.527809867 | 0.597641517 |
| adam15 | 13388 | PDI_H | -0.000484987 | 0.000922258 | -0.525868995 | 0.598987951 |
| ecscr | 11383 | PDI_H | -0.000531193 | 0.001010946 | -0.525441065 | 0.599286858 |
| kcnc4 | 11383 | PDI_H | -0.000530893 | 0.001010833 | -0.525203209 | 0.599452174 |
| lcp1 | 11790 | PDI_H | 0.000508871 | 0.000969546 | 0.524855165 | 0.599693758 |
| fgd3 | 11757 | PDI_H | -0.00052053 | 0.00099384 | -0.523755619 | 0.600458408 |
| cspg4 | 11790 | PDI_H | 0.000485992 | 0.000928489 | 0.523423065 | 0.600689725 |
| dnaja1 | 11304 | PDI_H | 0.000530841 | 0.001016051 | 0.522454701 | 0.601364028 |
| pspn | 13621 | PDI_H | 0.000345531 | 0.000662639 | 0.521447236 | 0.602063739 |
| kiaa1549 | 11570 | PDI_H | 0.000520458 | 0.001003632 | 0.518574148 | 0.604067669 |
| upk3a | 11383 | PDI_H | 0.000522191 | 0.001011367 | 0.516322133 | 0.60563948 |
| pafah1b3 | 11757 | PDI_H | -0.000512468 | 0.000992862 | -0.516152166 | 0.605757852 |
| dgkz | 13378 | PDI_H | -0.000480239 | 0.000931135 | -0.515756131 | 0.606033276 |
| npr1 | 11355 | PDI_H | 0.000519712 | 0.001007824 | 0.515677395 | 0.606089795 |
| lrrc59 | 11781 | PDI_H | -0.000509669 | 0.000989676 | -0.514985208 | 0.606573028 |
| adh1b | 11537 | PDI_H | 0.000477396 | 0.000928413 | 0.514206869 | 0.607117225 |
| acadsb | 11781 | PDI_H | 0.000508709 | 0.000992139 | 0.512739605 | 0.608143105 |
| cpq | 11773 | PDI_H | -0.000501546 | 0.000978909 | -0.512351861 | 0.608414397 |
| rnf43 | 11570 | PDI_H | 0.000509326 | 0.000996711 | 0.511006503 | 0.609356264 |
| il31 | 11411 | PDI_H | 0.000512888 | 0.001008237 | 0.50869743 | 0.610974169 |
| lmod2 | 11383 | PDI_H | -0.000513934 | 0.001010423 | -0.508632134 | 0.611019968 |
| usp47 | 11355 | PDI_H | -0.000513972 | 0.001011686 | -0.508035392 | 0.611438402 |
| mybpc2 | 11474 | PDI_H | 0.000485176 | 0.000955008 | 0.508033464 | 0.611439652 |
| anxa10 | 13320 | PDI_H | -0.000462417 | 0.000910619 | -0.50780458 | 0.611598813 |
| arhgap5 | 11304 | PDI_H | 0.000514378 | 0.001015754 | 0.50640084 | 0.612585172 |
| grik2 | 11757 | PDI_H | -0.000502905 | 0.000993368 | -0.506262902 | 0.612681605 |
| dner | 13641 | PDI_H | -0.000443591 | 0.00087719 | -0.505695448 | 0.613078643 |
| chek2 | 13388 | PDI_H | -0.000470463 | 0.00093128 | -0.505178943 | 0.613441482 |
| stk24 | 13320 | PDI_H | -0.000470767 | 0.000933458 | -0.504325689 | 0.614040877 |
| nptx1 | 13632 | PDI_H | 0.000441964 | 0.000876942 | 0.503983262 | 0.614281291 |
| batf | 11483 | PDI_H | 0.000507691 | 0.001008311 | 0.503506568 | 0.614617832 |
| gadd45b | 11570 | PDI_H | -0.000503981 | 0.001003527 | -0.502209812 | 0.615529509 |
| masp1 | 13535 | PDI_H | 0.000457348 | 0.000911375 | 0.501821523 | 0.615801241 |
| prkar1a | 13618 | PDI_H | -0.000462474 | 0.000921632 | -0.501798876 | 0.615817124 |
| ikzf2 | 13308 | PDI_H | -0.000467161 | 0.000931143 | -0.501706848 | 0.615882054 |
| crip2 | 13552 | PDI_H | -0.00041992 | 0.000837312 | -0.501509645 | 0.616020645 |
| slc9a3r1 | 11537 | PDI_H | -0.000503049 | 0.001003841 | -0.501124076 | 0.616293374 |
| enpp2 | 13800 | PDI_H | 0.000370339 | 0.000739138 | 0.501041523 | 0.616349899 |
| il20 | 13378 | PDI_H | -0.000465377 | 0.000929639 | -0.500599835 | 0.616661021 |
| appl2 | 11537 | PDI_H | -0.000502242 | 0.001004778 | -0.499853372 | 0.617187869 |
| eln | 11781 | PDI_H | -0.000372459 | 0.000747935 | -0.497983401 | 0.618505046 |
| dcun1d2 | 11304 | PDI_H | 0.000504962 | 0.001014609 | 0.49769175 | 0.618711015 |
| dync1h1 | 11730 | PDI_H | 0.000495663 | 0.000996105 | 0.497601435 | 0.618774329 |
| lrfn2 | 11483 | PDI_H | 0.000501105 | 0.001007312 | 0.497467948 | 0.618868636 |
| dlgap5 | 11757 | PDI_H | -0.000495282 | 0.000995662 | -0.497439275 | 0.618888627 |
| tmem106a | 11730 | PDI_H | 0.000492761 | 0.000993213 | 0.496127874 | 0.619813499 |
| lpl | 13618 | PDI_H | 0.000395023 | 0.000796237 | 0.4961129 | 0.619822772 |
| tg | 11730 | PDI_H | 0.000485712 | 0.000979317 | 0.495970007 | 0.619924874 |
| mylpf | 11483 | PDI_H | -0.000475042 | 0.0009602 | -0.494732398 | 0.620798504 |
| ogt | 11570 | PDI_H | 0.00049546 | 0.001002392 | 0.494277404 | 0.621119674 |
| nfkbie | 13308 | PDI_H | -0.000456555 | 0.000923762 | -0.494234405 | 0.62114881 |
| pdcd1lg2 | 13579 | PDI_H | 0.000453091 | 0.000916969 | 0.494118446 | 0.621230531 |
| ufd1 | 11724 | PDI_H | -0.000490548 | 0.000994281 | -0.493369381 | 0.621760864 |
| naaa | 13498 | PDI_H | 0.0004538 | 0.000922748 | 0.491792148 | 0.622874292 |
| mepe | 13658 | PDI_H | 0.000425533 | 0.000865809 | 0.491486116 | 0.623090574 |
| ido1 | 11474 | PDI_H | 0.000482165 | 0.000981292 | 0.491356946 | 0.623183418 |
| tsc22d1 | 11757 | PDI_H | -0.000486737 | 0.000990941 | -0.491186779 | 0.623303527 |
| ror1 | 13618 | PDI_H | 0.000438239 | 0.000892927 | 0.490789575 | 0.6235832 |
| col24a1 | 11724 | PDI_H | -0.000482748 | 0.000983956 | -0.490619299 | 0.623704926 |

| Protein | N_used | term | beta | se | t | p_val |
|---|---|---|---|---|---|---|
| il13ra1 | 13501 | PDI_H | -0.000439153 | 0.000895168 | -0.490581519 | 0.62373044 |
| mmp1 | 13139 | PDI_H | -0.000456255 | 0.00093115 | -0.489991122 | 0.62414837 |
| fcamr | 11781 | PDI_H | 0.000438218 | 0.000897665 | 0.488174902 | 0.625435055 |
| pkd1 | 11781 | PDI_H | 0.000446782 | 0.000916138 | 0.487680205 | 0.625785459 |
| ercc1 | 11304 | PDI_H | 0.000494769 | 0.001015031 | 0.487442065 | 0.625954553 |
| amdhd2 | 11790 | PDI_H | 0.000484618 | 0.00099469 | 0.487204797 | 0.626122273 |
| c2cd2l | 13303 | PDI_H | -0.000453255 | 0.000932704 | -0.485958198 | 0.627004815 |
| ccl13 | 13782 | PDI_H | -0.000437871 | 0.000901259 | -0.485844129 | 0.627085415 |
| ifngr1 | 13710 | PDI_H | -0.000437279 | 0.000900999 | -0.485326581 | 0.627452465 |
| hbegf | 13715 | PDI_H | 0.000439792 | 0.000911483 | 0.482501817 | 0.629457215 |
| jam3 | 11537 | PDI_H | -0.000483374 | 0.001003013 | -0.48192163 | 0.629870771 |
| tnfsf13 | 13710 | PDI_H | -0.000420845 | 0.000875438 | -0.480725246 | 0.630719463 |
| eif4g3 | 11757 | PDI_H | 0.000477741 | 0.000994265 | 0.480496428 | 0.630883392 |
| thpo | 13388 | PDI_H | -0.000444596 | 0.00092541 | -0.480431569 | 0.630928408 |
| tacc3 | 13579 | PDI_H | -0.000443864 | 0.000924283 | -0.480224644 | 0.631075408 |
| scrn1 | 13621 | PDI_H | -0.000440885 | 0.000920271 | -0.479081254 | 0.631888523 |
| acrbp | 11730 | PDI_H | 0.000475845 | 0.000994261 | 0.478592302 | 0.632237625 |
| ppt1 | 11483 | PDI_H | 0.000481428 | 0.001007741 | 0.477729978 | 0.632851507 |
| gzmb | 13621 | PDI_H | 0.000429286 | 0.00089945 | 0.477276323 | 0.633173033 |
| sftpd | 13618 | PDI_H | 0.000430784 | 0.000902765 | 0.477182862 | 0.633239578 |
| ptprf | 13800 | PDI_H | 0.000416875 | 0.000876044 | 0.475860403 | 0.634181373 |
| il17rb | 13452 | PDI_H | 0.000420564 | 0.000884626 | 0.475414383 | 0.634499371 |
| ctsf | 13699 | PDI_H | 0.000420793 | 0.000885597 | 0.475151773 | 0.63468638 |
| clec10a | 13552 | PDI_H | 0.000433734 | 0.000913517 | 0.474795294 | 0.634940545 |
| il11 | 13378 | PDI_H | -0.00044184 | 0.000930605 | -0.474787736 | 0.634946032 |
| rbm17 | 11757 | PDI_H | -0.000471435 | 0.000994746 | -0.473925006 | 0.635562196 |
| sv2a | 11483 | PDI_H | -0.000476833 | 0.001007157 | -0.473445073 | 0.635904689 |
| azi2 | 11757 | PDI_H | -0.000468795 | 0.00099292 | -0.472137784 | 0.636837216 |
| tmed1 | 11570 | PDI_H | -0.000473188 | 0.001003384 | -0.471592013 | 0.63722693 |
| gata3 | 11304 | PDI_H | 0.000478382 | 0.001015833 | 0.470925501 | 0.637703033 |
| slc16a1 | 13320 | PDI_H | -0.000435108 | 0.000923985 | -0.470902975 | 0.637717742 |
| capn3 | 11730 | PDI_H | 0.000464066 | 0.00099126 | 0.468157765 | 0.63968048 |
| gnpda2 | 11537 | PDI_H | 0.000468613 | 0.001002289 | 0.467542245 | 0.640120815 |
| map1lc3a | 11383 | PDI_H | 0.000472635 | 0.001011344 | 0.467333288 | 0.640270399 |
| acrv1 | 11411 | PDI_H | 0.000296601 | 0.000634682 | 0.467322491 | 0.640278101 |
| lysmd3 | 11483 | PDI_H | 0.000467623 | 0.001005762 | 0.464944539 | 0.641979999 |
| nlgn1 | 11483 | PDI_H | 0.000466291 | 0.001008415 | 0.46240016 | 0.643803155 |
| dlg4 | 11767 | PDI_H | -0.000459399 | 0.000994448 | -0.461964111 | 0.644115606 |
| bag3 | 13526 | PDI_H | 0.000411419 | 0.000892765 | 0.460836377 | 0.644923417 |
| mmut | 11355 | PDI_H | 0.000465462 | 0.001012074 | 0.459908975 | 0.645590373 |
| cemip2 | 11411 | PDI_H | 0.000454862 | 0.000989477 | 0.45969898 | 0.645741069 |
| commd9 | 11730 | PDI_H | 0.00045626 | 0.000995496 | 0.45832424 | 0.646728011 |
| tjp3 | 11383 | PDI_H | 0.000463364 | 0.001011755 | 0.45798015 | 0.646975454 |
| ranbp1 | 11474 | PDI_H | -0.000458429 | 0.001001315 | -0.457827541 | 0.647085026 |
| sparc | 13699 | PDI_H | -0.000419744 | 0.000917312 | -0.457580142 | 0.647261376 |
| gprc5c | 11730 | PDI_H | 0.00045234 | 0.000988697 | 0.45751093 | 0.64731233 |
| morc3 | 11767 | PDI_H | 0.000454378 | 0.000995883 | 0.456256503 | 0.648213968 |
| neo1 | 11757 | PDI_H | -0.000447467 | 0.000982462 | -0.455454547 | 0.64879068 |
| bcl7b | 11730 | PDI_H | -0.00045356 | 0.000996015 | -0.455374325 | 0.648848401 |
| armcx2 | 11383 | PDI_H | -0.000458792 | 0.001010382 | -0.454077409 | 0.649781779 |
| psmd1 | 11757 | PDI_H | -0.000447643 | 0.000986157 | -0.453926456 | 0.649890149 |
| kazald1 | 13579 | PDI_H | 0.000412881 | 0.000909619 | 0.453905985 | 0.649903757 |
| amigo1 | 11483 | PDI_H | -0.000456515 | 0.0010083 | -0.452756709 | 0.650732502 |
| enox2 | 11355 | PDI_H | -0.000457575 | 0.001011119 | -0.452542799 | 0.650886651 |
| akt2 | 11757 | PDI_H | -0.000450181 | 0.000995188 | -0.452358108 | 0.651019376 |
| dpep1 | 13535 | PDI_H | 0.000406928 | 0.000899861 | 0.452211496 | 0.651123881 |
| sord | 13695 | PDI_H | -0.000392424 | 0.000871769 | -0.450146642 | 0.652611839 |
| prkg1 | 11537 | PDI_H | -0.000451312 | 0.001004066 | -0.4494846 | 0.653090568 |
| rln1 | 11757 | PDI_H | 0.000443539 | 0.000988873 | 0.448529402 | 0.653779446 |
| ndst1 | 11730 | PDI_H | 0.000446497 | 0.000995684 | 0.448432521 | 0.653849367 |
| fabp2 | 13618 | PDI_H | -0.000409881 | 0.00091409 | -0.448403344 | 0.65386927 |
| clec1b | 13784 | PDI_H | -0.000408392 | 0.000913338 | -0.447141964 | 0.654779593 |
| gstp1 | 13246 | PDI_H | -0.000418379 | 0.00093635 | -0.446819136 | 0.655012965 |
| ankmy2 | 11411 | PDI_H | 0.000447334 | 0.001008246 | 0.443675717 | 0.657285476 |
| gdnf | 13320 | PDI_H | -0.000395885 | 0.000893934 | -0.442857647 | 0.657875902 |
| dnaja2 | 13751 | PDI_H | -0.000402982 | 0.000913057 | -0.441354649 | 0.658963214 |
| flt4 | 13699 | PDI_H | 0.000393508 | 0.000894894 | 0.439725765 | 0.660142682 |
| smad5 | 13579 | PDI_H | -0.000386137 | 0.000878287 | -0.439648184 | 0.660198939 |
| cthrc1 | 11790 | PDI_H | -0.00037238 | 0.000847919 | -0.439169281 | 0.660546938 |
| cd36 | 11411 | PDI_H | 0.000434684 | 0.000990677 | 0.438775011 | 0.660832885 |

| Protein | N_used | term | beta | se | t | p_val |
|---|---|---|---|---|---|---|
| ldlrap1 | 11757 | PDI_H | -0.000434634 | 0.000991755 | -0.438247547 | 0.661214905 |
| camsap1 | 11757 | PDI_H | -0.000435348 | 0.000994361 | -0.4378166 | 0.661527287 |
| psrc1 | 13308 | PDI_H | -0.000405229 | 0.000925838 | -0.437689154 | 0.661618743 |
| rpgr | 11483 | PDI_H | -0.000440329 | 0.001009128 | -0.43634561 | 0.662594207 |
| nxph3 | 11411 | PDI_H | 0.000419424 | 0.000962156 | 0.435921183 | 0.66290217 |
| scn3a | 11483 | PDI_H | -0.000439845 | 0.001009016 | -0.435914363 | 0.662907067 |
| crisp2 | 13753 | PDI_H | 0.000338031 | 0.000776815 | 0.435149306 | 0.663460891 |
| il6st | 13618 | PDI_H | -0.000394698 | 0.00091004 | -0.433714326 | 0.664502778 |
| mansc4 | 11355 | PDI_H | -0.000431146 | 0.00099597 | -0.432890125 | 0.665102825 |
| babam1 | 11411 | PDI_H | 0.000435293 | 0.001008436 | 0.43165108 | 0.666003191 |
| fmnl1 | 13320 | PDI_H | -0.000392678 | 0.000912067 | -0.430536685 | 0.666812259 |
| chl1 | 13666 | PDI_H | 0.000388849 | 0.000905349 | 0.429502191 | 0.667564577 |
| tnfsf8 | 11790 | PDI_H | 0.000422808 | 0.000984502 | 0.429463663 | 0.667593688 |
| dcc | 11483 | PDI_H | 0.000424816 | 0.000989647 | 0.429260807 | 0.667741498 |
| ncr3lg1 | 11724 | PDI_H | 0.000401775 | 0.000937503 | 0.428558658 | 0.668252318 |
| cebpb | 13388 | PDI_H | -0.000390267 | 0.000911541 | -0.428139951 | 0.668556125 |
| sptbn2 | 11383 | PDI_H | 0.000432476 | 0.001011287 | 0.427649378 | 0.668914509 |
| naga | 11388 | PDI_H | -0.000426722 | 0.001005292 | -0.424475469 | 0.671227119 |
| cdkl5 | 11383 | PDI_H | -0.000428553 | 0.001010657 | -0.4240339 | 0.671549112 |
| vamp8 | 11411 | PDI_H | -0.000424222 | 0.00100504 | -0.422095179 | 0.672963507 |
| prr5 | 11411 | PDI_H | 0.000425278 | 0.001008528 | 0.42168183 | 0.67326522 |
| casp9 | 11411 | PDI_H | -0.000424228 | 0.00100846 | -0.420668913 | 0.674004795 |
| olr1 | 13492 | PDI_H | -0.000385429 | 0.000917169 | -0.420238125 | 0.674318204 |
| brd2 | 11383 | PDI_H | -0.000424311 | 0.001011548 | -0.419467411 | 0.674882491 |
| nudt15 | 11355 | PDI_H | -0.000424235 | 0.001011891 | -0.419249823 | 0.675041503 |
| c8b | 11817 | PDI_H | -0.000406303 | 0.000969748 | -0.418977669 | 0.675240075 |
| sftpa2 | 13308 | PDI_H | 0.000382263 | 0.000913672 | 0.418380693 | 0.675675558 |
| hla_dra | 13571 | PDI_H | 0.000383567 | 0.000917304 | 0.418146317 | 0.675846765 |
| ccer2 | 11474 | PDI_H | -0.000411634 | 0.000987825 | -0.416707681 | 0.676900039 |
| duox2 | 13388 | PDI_H | -0.000387458 | 0.000930338 | -0.416469971 | 0.677072819 |
| klb | 13320 | PDI_H | 0.000383027 | 0.000924008 | 0.414527577 | 0.678494457 |
| lilra6 | 11781 | PDI_H | -0.000409821 | 0.000989556 | -0.414146377 | 0.678774457 |
| bnip2 | 11781 | PDI_H | -0.000410929 | 0.000993661 | -0.413550697 | 0.679210722 |
| alcam | 13800 | PDI_H | 0.000348598 | 0.000844112 | 0.412976479 | 0.679630268 |
| fshb | 11474 | PDI_H | 0.000289766 | 0.000703997 | 0.411601268 | 0.680639398 |
| lacrt | 11701 | PDI_H | 0.000398995 | 0.000970406 | 0.411162992 | 0.68096056 |
| nudt2 | 13579 | PDI_H | -0.000379502 | 0.000924348 | -0.410562057 | 0.681400174 |
| snx5 | 11474 | PDI_H | -0.000413391 | 0.001008077 | -0.410079117 | 0.681755575 |
| ace | 11794 | PDI_H | 0.000402937 | 0.000988579 | 0.407592358 | 0.683580381 |
| ca6 | 13597 | PDI_H | 0.000345222 | 0.000847421 | 0.407379327 | 0.683735827 |
| smtn | 11743 | PDI_H | -0.000403689 | 0.000992502 | -0.406738764 | 0.684207287 |
| c1qa | 13747 | PDI_H | -0.000369084 | 0.000908185 | -0.406397087 | 0.684457191 |
| plxnb3 | 13580 | PDI_H | -0.000374276 | 0.000922637 | -0.405658903 | 0.684999639 |
| tmco5a | 11483 | PDI_H | -0.000408383 | 0.00100771 | -0.405258259 | 0.685295243 |
| krt6c | 11355 | PDI_H | 0.000405405 | 0.001004238 | 0.40369392 | 0.686445421 |
| eps8l2 | 13726 | PDI_H | 0.000351056 | 0.000874928 | 0.40124039 | 0.688249398 |
| nptn | 13308 | PDI_H | 0.000371205 | 0.000925184 | 0.401222891 | 0.688262477 |
| pdgfc | 13308 | PDI_H | -0.000370848 | 0.000924629 | -0.401077826 | 0.688369271 |
| pgr | 11411 | PDI_H | 0.000399832 | 0.001005802 | 0.397525384 | 0.690987515 |
| hs3st3b1 | 13579 | PDI_H | 0.000365803 | 0.00092115 | 0.397116014 | 0.691288158 |
| ggh | 13413 | PDI_H | -0.000343209 | 0.00086447 | -0.397016464 | 0.691361642 |
| aspn | 11781 | PDI_H | -0.00038116 | 0.000960834 | -0.396697307 | 0.691597879 |
| cst5 | 13390 | PDI_H | 0.000354829 | 0.000896407 | 0.395835008 | 0.692233061 |
| csnk2a1 | 11757 | PDI_H | -0.000392299 | 0.000991366 | -0.395715273 | 0.692322274 |
| reck | 11474 | PDI_H | 0.00037275 | 0.000942788 | 0.395369392 | 0.692577652 |
| cplx2 | 11304 | PDI_H | -0.000400952 | 0.001014502 | -0.39522046 | 0.69268766 |
| b3gnt7 | 11474 | PDI_H | 0.000382626 | 0.000969182 | 0.394792991 | 0.693003014 |
| sf3b4 | 13579 | PDI_H | -0.000364796 | 0.000924412 | -0.394624402 | 0.693126305 |
| pkn3 | 11570 | PDI_H | -0.000395496 | 0.001002607 | -0.394467422 | 0.693243253 |
| mapt | 13320 | PDI_H | -0.000368582 | 0.000934589 | -0.394378317 | 0.69330807 |
| gad1 | 11304 | PDI_H | 0.000398272 | 0.001014619 | 0.392533116 | 0.694671756 |
| bpifb1 | 13604 | PDI_H | -0.000342118 | 0.00087243 | -0.392143852 | 0.694958076 |
| snapin | 11757 | PDI_H | -0.000388241 | 0.000990572 | -0.39193596 | 0.695112644 |
| sorbs1 | 11757 | PDI_H | 0.000368129 | 0.000939695 | 0.391753156 | 0.695247718 |
| ggt5 | 13552 | PDI_H | 0.000357753 | 0.000913359 | 0.391689054 | 0.695294143 |
| bcat1 | 11781 | PDI_H | 0.000381061 | 0.000973634 | 0.391380437 | 0.695523136 |
| ptges2 | 11324 | PDI_H | -0.000391996 | 0.001003448 | -0.390648374 | 0.69606452 |
| ap3s2 | 11767 | PDI_H | -0.000387111 | 0.000991939 | -0.390256763 | 0.696353763 |
| gabra4 | 11383 | PDI_H | -0.000393786 | 0.001011188 | -0.389428915 | 0.696966179 |
| pithd1 | 11271 | PDI_H | -0.000395509 | 0.001015621 | -0.38942548 | 0.696968792 |

| Protein | N_used | term | beta | se | t | p_val |
|---|---|---|---|---|---|---|
| rgl2 | 11570 | PDI_H | -0.000390672 | 0.001003415 | -0.389341991 | 0.697030351 |
| lta | 13621 | PDI_H | 0.000346413 | 0.000892899 | 0.387964724 | 0.698048207 |
| arhgef10 | 11757 | PDI_H | -0.000383798 | 0.000993354 | -0.386365527 | 0.699232976 |
| gsr | 11709 | PDI_H | -0.00037256 | 0.000968712 | -0.384592955 | 0.700546006 |
| cptp | 11355 | PDI_H | -0.000388737 | 0.001010776 | -0.384592821 | 0.700546324 |
| vasp | 11794 | PDI_H | -0.000380746 | 0.00099133 | -0.384075953 | 0.700929085 |
| esam | 13800 | PDI_H | -0.000342227 | 0.0008934 | -0.383061545 | 0.701680037 |
| c9orf40 | 11724 | PDI_H | -0.000379907 | 0.000992694 | -0.382703012 | 0.701946926 |
| sat1 | 11355 | PDI_H | 0.000386501 | 0.00101023 | 0.382587051 | 0.702033141 |
| inpp1 | 13579 | PDI_H | 0.000352378 | 0.000921514 | 0.382389659 | 0.702178349 |
| fbn2 | 11483 | PDI_H | 0.000385643 | 0.001008636 | 0.382341047 | 0.702215496 |
| pomc | 11474 | PDI_H | -0.000366473 | 0.000959881 | -0.381790492 | 0.702623849 |
| nedd9 | 11411 | PDI_H | -0.000382722 | 0.001003098 | -0.381539577 | 0.702810022 |
| akr1c4 | 13388 | PDI_H | 0.000352798 | 0.000925459 | 0.381214498 | 0.703050147 |
| tfrc | 13694 | PDI_H | -0.00034288 | 0.00090186 | -0.380192322 | 0.70380856 |
| dapk2 | 11411 | PDI_H | 0.000381937 | 0.001004849 | 0.380094167 | 0.703882597 |
| spink8 | 11355 | PDI_H | 0.000384195 | 0.001010868 | 0.380064211 | 0.703904868 |
| ahnak | 11474 | PDI_H | -0.000350771 | 0.000925894 | -0.378846357 | 0.704808978 |
| adam22 | 13552 | PDI_H | 0.000343151 | 0.000907234 | 0.37823902 | 0.70525897 |
| fas | 13750 | PDI_H | -0.000311051 | 0.000824379 | -0.377315856 | 0.705944718 |
| lag3 | 13579 | PDI_H | -0.00033674 | 0.000892568 | -0.37727173 | 0.70597758 |
| dctn2 | 13579 | PDI_H | -0.000347887 | 0.000924722 | -0.376207778 | 0.706768316 |
| ndufa5 | 11394 | PDI_H | -0.000378126 | 0.001005201 | -0.376169484 | 0.706797912 |
| znf174 | 11411 | PDI_H | 0.000378089 | 0.00100866 | 0.37484273 | 0.707784407 |
| pdzk1 | 11411 | PDI_H | -0.000360263 | 0.000961819 | -0.374564098 | 0.707991646 |
| micall2 | 11483 | PDI_H | -0.000376477 | 0.00100801 | -0.373485787 | 0.708793823 |
| mfap3l | 11483 | PDI_H | 0.000376454 | 0.001008008 | 0.373462702 | 0.708811002 |
| stab2 | 11790 | PDI_H | -0.000350735 | 0.000939628 | -0.373270185 | 0.708954082 |
| lamp2 | 13278 | PDI_H | -0.000325214 | 0.000874267 | -0.37198438 | 0.709910421 |
| cd226 | 11411 | PDI_H | 0.000374358 | 0.001007013 | 0.371750461 | 0.71008556 |
| hla_a | 11757 | PDI_H | 0.000368041 | 0.000992253 | 0.370914217 | 0.710708115 |
| rexo2 | 11355 | PDI_H | 0.000375196 | 0.001011584 | 0.370899745 | 0.710719131 |
| ntf3 | 13621 | PDI_H | 0.00033612 | 0.000914344 | 0.367608199 | 0.713171117 |
| bdnf | 11790 | PDI_H | -0.000363715 | 0.000989663 | -0.36751406 | 0.713242209 |
| cbx2 | 11355 | PDI_H | 0.000371299 | 0.001011452 | 0.36709496 | 0.713555034 |
| tmem25 | 11757 | PDI_H | -0.000362207 | 0.000986862 | -0.367028838 | 0.71360412 |
| clps | 13597 | PDI_H | 0.000335895 | 0.000915952 | 0.366717413 | 0.713835528 |
| clec4g | 13621 | PDI_H | 0.000325967 | 0.000892342 | 0.365294054 | 0.714897594 |
| lefty2 | 13732 | PDI_H | 0.000314968 | 0.000863551 | 0.364735679 | 0.715314344 |
| pdgfrb | 13800 | PDI_H | -0.000330671 | 0.000910787 | -0.36306089 | 0.716564965 |
| c1ql2 | 11411 | PDI_H | -0.000346785 | 0.000956704 | -0.362479166 | 0.717000709 |
| notch2 | 11790 | PDI_H | -0.00035067 | 0.000967534 | -0.362436556 | 0.717032329 |
| chrm1 | 11483 | PDI_H | 0.000365531 | 0.001008563 | 0.362427351 | 0.71703938 |
| lilrb5 | 13800 | PDI_H | 0.00032982 | 0.000910262 | 0.362335789 | 0.71710667 |
| git1 | 11411 | PDI_H | -0.000364069 | 0.001006742 | -0.361631368 | 0.717634223 |
| bsnd | 11304 | PDI_H | 0.000365791 | 0.00101568 | 0.360143689 | 0.718746418 |
| fkbp7 | 13303 | PDI_H | -0.000335712 | 0.000934051 | -0.359415463 | 0.719290019 |
| dock9 | 11383 | PDI_H | 0.00036307 | 0.001011387 | 0.358982383 | 0.719614933 |
| tspan8 | 11724 | PDI_H | 0.00034812 | 0.000970775 | 0.358600722 | 0.71990027 |
| fndc1 | 11767 | PDI_H | 0.000344129 | 0.0009606 | 0.358244375 | 0.720166875 |
| kir2ds4 | 11724 | PDI_H | -0.000356624 | 0.000997566 | -0.357493648 | 0.720728722 |
| cndp1 | 13689 | PDI_H | 0.000323845 | 0.000906375 | 0.357297594 | 0.720874544 |
| myh7b | 11304 | PDI_H | 0.00036186 | 0.001014399 | 0.356723557 | 0.721305433 |
| eno3 | 11474 | PDI_H | 0.000327991 | 0.000919962 | 0.356526336 | 0.721452995 |
| setmar | 13510 | PDI_H | -0.00030237 | 0.000852046 | -0.354875546 | 0.722688373 |
| col5a1 | 11537 | PDI_H | 0.000352135 | 0.000993206 | 0.35454388 | 0.722937812 |
| fcrl2 | 13741 | PDI_H | -0.000320525 | 0.000909059 | -0.352589675 | 0.724401492 |
| apob | 11794 | PDI_H | 0.000349748 | 0.000993519 | 0.35202894 | 0.724822855 |
| pgm2 | 11483 | PDI_H | 0.000354677 | 0.001008824 | 0.351574725 | 0.72516368 |
| gabarapl1 | 11570 | PDI_H | -0.000352173 | 0.001002667 | -0.351236278 | 0.725417498 |
| kcnh2 | 11483 | PDI_H | -0.000352965 | 0.001007866 | -0.35020986 | 0.726187637 |
| notch3 | 13800 | PDI_H | 0.000299477 | 0.000857296 | 0.349327518 | 0.726848777 |
| polr2a | 11411 | PDI_H | 0.00035106 | 0.001008467 | 0.348112424 | 0.727762182 |
| il13ra2 | 11383 | PDI_H | 0.00035172 | 0.001011417 | 0.347749439 | 0.728034801 |
| cdk5rap3 | 11757 | PDI_H | -0.000345431 | 0.000993364 | -0.347739007 | 0.728042431 |
| tab2 | 11570 | PDI_H | 0.00034853 | 0.001002977 | 0.34749535 | 0.728225538 |
| ppp3r1 | 13552 | PDI_H | -0.000314722 | 0.000907376 | -0.346848805 | 0.728710299 |
| safb2 | 11757 | PDI_H | -0.000344241 | 0.000993371 | -0.346538424 | 0.72894432 |
| ttf2 | 11483 | PDI_H | -0.000346672 | 0.001007829 | -0.343979176 | 0.730868251 |
| dkk1 | 13784 | PDI_H | -0.000313198 | 0.000911751 | -0.343513059 | 0.731217762 |

| Protein | N_used | term | beta | se | t | p_val |
|---|---|---|---|---|---|---|
| axl | 13689 | PDI_H | 0.000312183 | 0.000908935 | 0.343460113 | 0.731257622 |
| ghr | 11537 | PDI_H | 0.000312802 | 0.000911432 | 0.343197798 | 0.731455921 |
| adcyap1r1 | 13308 | PDI_H | 0.000319348 | 0.000932679 | 0.342399177 | 0.73205592 |
| podxl | 13579 | PDI_H | 0.00031595 | 0.000924628 | 0.341704721 | 0.732578411 |
| tef | 11355 | PDI_H | -0.000344827 | 0.001011469 | -0.34091733 | 0.733172132 |
| pdp1 | 13308 | PDI_H | -0.000316794 | 0.000931875 | -0.339953334 | 0.733897041 |
| ptprh | 11724 | PDI_H | 0.000331021 | 0.000978291 | 0.338366678 | 0.735092947 |
| tfap2a | 11383 | PDI_H | 0.00034118 | 0.001011249 | 0.337385079 | 0.735832858 |
| tnfsf10 | 13801 | PDI_H | 0.00030608 | 0.000910526 | 0.33615702 | 0.736757577 |
| htr1b | 11383 | PDI_H | -0.000339148 | 0.001010492 | -0.335626421 | 0.73715879 |
| siglec15 | 13320 | PDI_H | 0.000313151 | 0.000933264 | 0.335543441 | 0.73722047 |
| larp1 | 11730 | PDI_H | -0.000333421 | 0.000993977 | -0.335441053 | 0.737298409 |
| pdlim5 | 11757 | PDI_H | -0.000332919 | 0.000995313 | -0.334486218 | 0.738018662 |
| evpl | 11570 | PDI_H | -0.000335575 | 0.00100352 | -0.334397501 | 0.738085692 |
| il12b | 13801 | PDI_H | -0.000291393 | 0.000875694 | -0.332756326 | 0.739323286 |
| pdlim7 | 13710 | PDI_H | -0.000304948 | 0.000918159 | -0.332130323 | 0.739795936 |
| pdgfa | 13800 | PDI_H | -0.00030271 | 0.000912975 | -0.331564786 | 0.740222953 |
| mucl3 | 11304 | PDI_H | 0.000335908 | 0.00101411 | 0.331234551 | 0.74047347 |
| myom1 | 11383 | PDI_H | -0.000332881 | 0.001009702 | -0.329682719 | 0.741645785 |
| lrig3 | 11379 | PDI_H | -0.000323904 | 0.000985577 | -0.328644284 | 0.742430626 |
| rilpl2 | 11781 | PDI_H | -0.000325073 | 0.000991166 | -0.327970306 | 0.742939948 |
| ehd3 | 11474 | PDI_H | -0.000328706 | 0.001006644 | -0.32653614 | 0.744024712 |
| ahnak2 | 11474 | PDI_H | 0.000319913 | 0.000980677 | 0.326216086 | 0.744266827 |
| ldlr | 13668 | PDI_H | -0.000279626 | 0.000857708 | -0.326015042 | 0.74441797 |
| prune2 | 11570 | PDI_H | 0.000324802 | 0.000996957 | 0.325792984 | 0.744586886 |
| npl | 11781 | PDI_H | -0.00028246 | 0.000868959 | -0.3250558 | 0.745144619 |
| atf4 | 11304 | PDI_H | 0.00032973 | 0.001015658 | 0.324646969 | 0.745454288 |
| mzt1 | 13308 | PDI_H | -0.000301996 | 0.000932182 | -0.323966239 | 0.74596869 |
| efnb2 | 11483 | PDI_H | 0.000325483 | 0.001006565 | 0.323360132 | 0.746428414 |
| sprr1b | 11570 | PDI_H | 0.000324151 | 0.001002727 | 0.323270003 | 0.746496618 |
| palld | 11411 | PDI_H | -0.000324477 | 0.00100752 | -0.322054632 | 0.747417213 |
| sirt1 | 11411 | PDI_H | 0.000323965 | 0.001007486 | 0.321557412 | 0.747793908 |
| pzp | 11817 | PDI_H | 0.00022839 | 0.000711431 | 0.321028521 | 0.74819446 |
| cd244 | 13801 | PDI_H | 0.000290907 | 0.000906373 | 0.320957322 | 0.748247597 |
| stx16 | 13308 | PDI_H | -0.000298934 | 0.000931847 | -0.320797305 | 0.748369044 |
| atp1b4 | 11304 | PDI_H | 0.000325186 | 0.001015035 | 0.320368984 | 0.748694563 |
| nmrk2 | 11304 | PDI_H | 0.000323715 | 0.001012977 | 0.319568312 | 0.749301511 |
| arfip1 | 11757 | PDI_H | 0.000316759 | 0.000995712 | 0.318122698 | 0.750397523 |
| bhlhe40 | 11383 | PDI_H | 0.000320825 | 0.001011755 | 0.317097044 | 0.751175789 |
| cd84 | 13621 | PDI_H | -0.000290946 | 0.000920944 | -0.315921163 | 0.752067193 |
| nfx1 | 11355 | PDI_H | -0.000318452 | 0.00100835 | -0.315815127 | 0.752148646 |
| sod3 | 11794 | PDI_H | -0.000290531 | 0.000921816 | -0.315171967 | 0.75263667 |
| vsig10 | 11730 | PDI_H | 0.00031223 | 0.000992036 | 0.314736409 | 0.752967402 |
| lrrc37a2 | 11724 | PDI_H | -0.000311641 | 0.000993374 | -0.313719713 | 0.753739514 |
| cabp2 | 11483 | PDI_H | -0.000314932 | 0.001007641 | -0.312544081 | 0.754632749 |
| plb1 | 11767 | PDI_H | 0.000292506 | 0.000939126 | 0.311466565 | 0.75545148 |
| lilra3 | 11790 | PDI_H | 0.000305788 | 0.000982053 | 0.311376749 | 0.755519739 |
| il3 | 11304 | PDI_H | -0.000316094 | 0.001015732 | -0.311198544 | 0.755655435 |
| il1a | 13378 | PDI_H | 0.000288444 | 0.000931984 | 0.309494787 | 0.756950002 |
| lilrb1 | 13800 | PDI_H | -0.000271489 | 0.000881255 | -0.308070646 | 0.75803323 |
| pde5a | 11537 | PDI_H | -0.000306618 | 0.001004502 | -0.305244068 | 0.760185784 |
| sparcl1 | 13632 | PDI_H | -0.000277379 | 0.000909766 | -0.304890573 | 0.760454155 |
| chad | 11537 | PDI_H | 0.000298553 | 0.000984741 | 0.303178697 | 0.761759155 |
| dars1 | 13535 | PDI_H | -0.000279085 | 0.000923534 | -0.302192457 | 0.762510001 |
| chp1 | 11383 | PDI_H | 0.000303276 | 0.001011751 | 0.299753978 | 0.764370301 |
| pros1 | 11817 | PDI_H | -0.000286475 | 0.000959144 | -0.298677689 | 0.765191239 |
| robo4 | 11474 | PDI_H | 0.000299496 | 0.001004679 | 0.298101676 | 0.765630966 |
| numb | 11411 | PDI_H | -0.000297568 | 0.001001465 | -0.297133035 | 0.766370358 |
| fes | 13308 | PDI_H | -0.000275142 | 0.000928138 | -0.296444602 | 0.766895198 |
| cpxm1 | 13699 | PDI_H | -0.000271409 | 0.000917757 | -0.295730605 | 0.767440309 |
| sema6c | 11411 | PDI_H | -0.000296902 | 0.001004846 | -0.295470394 | 0.767639947 |
| ryr1 | 11355 | PDI_H | 0.000298862 | 0.001012455 | 0.295185698 | 0.76785743 |
| ccdc50 | 11757 | PDI_H | -0.000292268 | 0.00099488 | -0.293771738 | 0.768937523 |
| m6pr | 11474 | PDI_H | 0.000296082 | 0.001008032 | 0.293722727 | 0.768975104 |
| lamtor5 | 11730 | PDI_H | -0.000291417 | 0.000994669 | -0.29297877 | 0.769543567 |
| lrrc38 | 11483 | PDI_H | 0.000285385 | 0.000975943 | 0.292420091 | 0.769970737 |
| thsd1 | 11321 | PDI_H | -0.000294932 | 0.001010741 | -0.291797459 | 0.77044684 |
| cxcl12 | 13378 | PDI_H | -0.000269225 | 0.000923854 | -0.291415146 | 0.770738354 |
| defa1_defa1b | 13351 | PDI_H | -0.000265792 | 0.000912606 | -0.2912448 | 0.77086863 |
| myl1 | 11355 | PDI_H | 0.000289498 | 0.000994667 | 0.291050727 | 0.771017845 |

| Protein | N_used | term | beta | se | t | p_val |
|---|---|---|---|---|---|---|
| rnf168 | 11411 | PDI_H | 0.000293281 | 0.001008516 | 0.290804091 | 0.771206447 |
| trim21 | 13621 | PDI_H | -0.000264425 | 0.000910594 | -0.290387106 | 0.771524531 |
| efhd1 | 11757 | PDI_H | 0.00026899 | 0.000926777 | 0.290241985 | 0.771636243 |
| tcp11 | 11570 | PDI_H | 0.000290915 | 0.001002803 | 0.290101892 | 0.771743493 |
| cr1 | 11737 | PDI_H | 0.000278363 | 0.00096315 | 0.289013474 | 0.772576176 |
| igf1r | 13579 | PDI_H | 0.00025755 | 0.000893329 | 0.288303996 | 0.773118453 |
| enah | 13378 | PDI_H | -0.000262028 | 0.000909512 | -0.288097156 | 0.773276837 |
| cpb1 | 13800 | PDI_H | -0.000261559 | 0.000908503 | -0.287900462 | 0.77342726 |
| kir3dl1 | 13308 | PDI_H | 0.000267805 | 0.000932659 | 0.287141831 | 0.774008199 |
| ep300 | 11411 | PDI_H | 0.000288689 | 0.001008182 | 0.286345867 | 0.774618435 |
| folr3 | 13660 | PDI_H | -0.000255557 | 0.000898986 | -0.284272311 | 0.776206016 |
| shc1 | 11304 | PDI_H | 0.000286906 | 0.001009718 | 0.284144351 | 0.77630497 |
| cfp | 11817 | PDI_H | 0.000270112 | 0.000951488 | 0.283883584 | 0.776504576 |
| casp4 | 11570 | PDI_H | -0.000284195 | 0.001003473 | -0.283211169 | 0.77702004 |
| dipk1c | 11483 | PDI_H | -0.000283904 | 0.001007663 | -0.281745365 | 0.778143849 |
| psmc3 | 11304 | PDI_H | 0.000285682 | 0.001013997 | 0.281738323 | 0.77814933 |
| atp1b2 | 11355 | PDI_H | 0.00028353 | 0.001011013 | 0.280441041 | 0.779144268 |
| cblif | 13599 | PDI_H | -0.0002538 | 0.000905763 | -0.280206158 | 0.779323609 |
| retn | 13601 | PDI_H | -0.000253217 | 0.000903692 | -0.280203077 | 0.779325971 |
| cngb3 | 11383 | PDI_H | 0.000283021 | 0.001011375 | 0.279837424 | 0.779607328 |
| itpr1 | 11474 | PDI_H | 0.000277821 | 0.001001629 | 0.277369411 | 0.781501469 |
| pvalb | 13698 | PDI_H | -0.000255541 | 0.000921355 | -0.277353836 | 0.781512614 |
| gas2 | 11570 | PDI_H | -0.000274498 | 0.000992122 | -0.276677964 | 0.782032341 |
| tpk1 | 11781 | PDI_H | 0.000272007 | 0.000983636 | 0.276532697 | 0.782143805 |
| maea | 13308 | PDI_H | 0.000257393 | 0.000932004 | 0.276171306 | 0.782420787 |
| sez6l | 13699 | PDI_H | -0.000235431 | 0.000852739 | -0.276088122 | 0.782484552 |
| slirp | 11304 | PDI_H | -0.000278761 | 0.001015431 | -0.274524924 | 0.783686274 |
| stx5 | 11411 | PDI_H | 0.000276497 | 0.001007721 | 0.274378656 | 0.783798616 |
| sh3bp1 | 11724 | PDI_H | -0.000271231 | 0.000990395 | -0.273861685 | 0.784195747 |
| ctsh | 13346 | PDI_H | 0.000249391 | 0.000912045 | 0.2734419 | 0.784517783 |
| ppp1r12b | 11570 | PDI_H | -0.000272527 | 0.001001932 | -0.272001248 | 0.785625918 |
| insr | 11411 | PDI_H | 0.000272769 | 0.001004044 | 0.271670646 | 0.785880193 |
| pcna | 11304 | PDI_H | 0.000275129 | 0.001014201 | 0.271276084 | 0.786183658 |
| gask1a | 11355 | PDI_H | 0.000267292 | 0.000988183 | 0.270488009 | 0.786789762 |
| pepd | 11794 | PDI_H | 0.000264937 | 0.000984288 | 0.269165557 | 0.787806997 |
| hs6st1 | 13753 | PDI_H | 0.000243505 | 0.000905017 | 0.269061018 | 0.787886768 |
| rab11fip3 | 11781 | PDI_H | -0.00026492 | 0.0009906 | -0.267434048 | 0.789139674 |
| rp2 | 13579 | PDI_H | -0.000245688 | 0.00092182 | -0.266525235 | 0.789838778 |
| calcoco1 | 13703 | PDI_H | -0.000244001 | 0.000920101 | -0.265189275 | 0.790867649 |
| tmprss5 | 13483 | PDI_H | -0.000236119 | 0.000891052 | -0.26498865 | 0.79102226 |
| mdm1 | 11483 | PDI_H | 0.000267201 | 0.001008956 | 0.264829083 | 0.79114589 |
| nxpe4 | 11355 | PDI_H | -0.000268108 | 0.001012551 | -0.26478457 | 0.791180235 |
| egflam | 11724 | PDI_H | -0.000261103 | 0.000986502 | -0.264674989 | 0.791264504 |
| sema3g | 11537 | PDI_H | -0.000263948 | 0.000998392 | -0.264372489 | 0.791497636 |
| faslg | 13801 | PDI_H | 0.000227806 | 0.000861917 | 0.264301576 | 0.791551496 |
| mrps16 | 11411 | PDI_H | -0.000266009 | 0.001006578 | -0.264270674 | 0.791576134 |
| tp73 | 11483 | PDI_H | -0.000264596 | 0.001009181 | -0.262188696 | 0.793180676 |
| dgcr6 | 11383 | PDI_H | 0.000263374 | 0.001007114 | 0.261513193 | 0.793701514 |
| rnase10 | 11474 | PDI_H | -0.00025998 | 0.000994782 | -0.26134406 | 0.793831887 |
| pf4 | 11717 | PDI_H | 0.000258618 | 0.000996489 | 0.259528811 | 0.79523182 |
| shbg | 11794 | PDI_H | -0.000223038 | 0.000859533 | -0.25948723 | 0.795263867 |
| adamts4 | 11411 | PDI_H | 0.000254232 | 0.000981614 | 0.258993331 | 0.795645063 |
| cinp | 11570 | PDI_H | 0.000259407 | 0.001003607 | 0.258475036 | 0.796044916 |
| lyzl2 | 11304 | PDI_H | -0.000234983 | 0.000913402 | -0.257261779 | 0.796981383 |
| mki67 | 11411 | PDI_H | 0.000257812 | 0.001006681 | 0.256101182 | 0.797877331 |
| ngfr | 11781 | PDI_H | 0.000253426 | 0.000991385 | 0.255628313 | 0.798242321 |
| cela3a | 13779 | PDI_H | 0.000231456 | 0.000907293 | 0.255105826 | 0.798645175 |
| nrxn3 | 11483 | PDI_H | -0.000256672 | 0.001007477 | -0.254766752 | 0.798907824 |
| pibf1 | 11757 | PDI_H | -0.000252089 | 0.000992306 | -0.25404315 | 0.799466669 |
| atp1b3 | 11355 | PDI_H | -0.000257053 | 0.001012094 | -0.253981222 | 0.799514668 |
| luzp2 | 11730 | PDI_H | -0.000245707 | 0.000971265 | -0.252976096 | 0.800291123 |
| relb | 11411 | PDI_H | -0.000254953 | 0.001008822 | -0.252723671 | 0.800486311 |
| dbn1 | 11411 | PDI_H | 0.000253997 | 0.001006755 | 0.252292629 | 0.800819434 |
| tnni3 | 13388 | PDI_H | 0.000231984 | 0.000920827 | 0.251930379 | 0.801098748 |
| s100g | 11757 | PDI_H | -0.000246228 | 0.000983678 | -0.250313699 | 0.802349141 |
| klk3 | 11790 | PDI_H | 0.000154825 | 0.000619085 | 0.250086571 | 0.802524763 |
| scn2b | 11483 | PDI_H | 0.000251917 | 0.001008292 | 0.249844827 | 0.802711825 |
| tshb | 13618 | PDI_H | 0.000228496 | 0.000915187 | 0.249671779 | 0.802844953 |
| il1r2 | 13635 | PDI_H | 0.000224388 | 0.000899318 | 0.24950945 | 0.802970494 |
| sh3bgrl2 | 11383 | PDI_H | -0.000249917 | 0.001010803 | -0.247245745 | 0.804722509 |

| Protein | N_used | term | beta | se | t | p_val |
|---|---|---|---|---|---|---|
| gdf2 | 13618 | PDI_H | 0.000207207 | 0.000839801 | 0.246733227 | 0.805118412 |
| gfod2 | 13308 | PDI_H | -0.000229937 | 0.000932847 | -0.246489908 | 0.805306821 |
| top1mt | 11304 | PDI_H | 0.000248882 | 0.001015758 | 0.245021041 | 0.806444592 |
| rab27b | 11781 | PDI_H | -0.000242252 | 0.000991161 | -0.244412945 | 0.806915278 |
| lxn | 13493 | PDI_H | -0.000224815 | 0.000919938 | -0.244381158 | 0.806939352 |
| apoe | 11817 | PDI_H | 0.00023894 | 0.000979789 | 0.243868914 | 0.807336583 |
| lats1 | 11411 | PDI_H | -0.000244481 | 0.001003444 | -0.243642037 | 0.807512453 |
| rnf5 | 11474 | PDI_H | 0.000243554 | 0.00100583 | 0.242142033 | 0.808674441 |
| nfic | 11757 | PDI_H | 0.000240661 | 0.000994231 | 0.242057638 | 0.808739728 |
| hsd17b3 | 11355 | PDI_H | -0.000244078 | 0.001010868 | -0.241454179 | 0.80920749 |
| scarf2 | 13784 | PDI_H | -0.000193181 | 0.000801084 | -0.24114983 | 0.809442584 |
| rbp1 | 11483 | PDI_H | 0.000242917 | 0.001007426 | 0.241126457 | 0.809461417 |
| enoph1 | 11730 | PDI_H | 0.000238537 | 0.000995472 | 0.239621797 | 0.810627657 |
| atp5f1d | 11355 | PDI_H | -0.000242459 | 0.001012346 | -0.239501542 | 0.810721028 |
| tspan7 | 11383 | PDI_H | 0.000241291 | 0.001008265 | 0.239313383 | 0.810866902 |
| mgll | 13691 | PDI_H | -0.000218528 | 0.00091982 | -0.237576483 | 0.81221315 |
| aplp1 | 13618 | PDI_H | -0.000209854 | 0.000883647 | -0.237486601 | 0.812282889 |
| birc2 | 13308 | PDI_H | -0.000221199 | 0.000932286 | -0.237265162 | 0.812454743 |
| insl5 | 11724 | PDI_H | 0.00022135 | 0.000936652 | 0.236320942 | 0.813187771 |
| casp8 | 13715 | PDI_H | -0.000215476 | 0.000912206 | -0.236214023 | 0.813270129 |
| rasgrf1 | 11304 | PDI_H | 0.00023923 | 0.001015932 | 0.235477964 | 0.813842051 |
| casq2 | 11355 | PDI_H | 0.000237859 | 0.001011487 | 0.235157523 | 0.814090718 |
| lamp1 | 11454 | PDI_H | -0.000233036 | 0.000994708 | -0.23427595 | 0.814774948 |
| s100p | 13388 | PDI_H | 0.000212388 | 0.000906799 | 0.234217574 | 0.814819657 |
| med21 | 11355 | PDI_H | 0.000235942 | 0.001012175 | 0.233104043 | 0.815684822 |
| xpnpep2 | 13699 | PDI_H | 0.000208449 | 0.000906707 | 0.229896338 | 0.818175758 |
| smc3 | 11304 | PDI_H | 0.000231063 | 0.001015887 | 0.227449758 | 0.820078151 |
| selp | 13689 | PDI_H | -0.000207139 | 0.000911156 | -0.227336241 | 0.820165693 |
| h2ap | 11304 | PDI_H | 0.000230276 | 0.00101528 | 0.226809916 | 0.820575658 |
| tars1 | 11730 | PDI_H | 0.000223734 | 0.000996006 | 0.224631332 | 0.822270005 |
| tek | 13699 | PDI_H | 0.000204795 | 0.000913338 | 0.224227434 | 0.822583684 |
| gid8 | 11757 | PDI_H | 0.000223206 | 0.000996311 | 0.22403264 | 0.822735803 |
| pag1 | 13388 | PDI_H | 0.000201237 | 0.000901632 | 0.223192295 | 0.823389257 |
| adipoq | 11817 | PDI_H | 0.000181852 | 0.000817606 | 0.222419986 | 0.823990803 |
| lilra4 | 11355 | PDI_H | 0.000223981 | 0.001009771 | 0.221813682 | 0.824462922 |
| gpd1 | 11730 | PDI_H | -0.000197639 | 0.00089669 | -0.220409468 | 0.825556112 |
| rps10 | 11355 | PDI_H | -0.000222667 | 0.001010858 | -0.220274843 | 0.825661075 |
| cebpa | 11411 | PDI_H | -0.000219726 | 0.001001952 | -0.219297745 | 0.826422044 |
| mllt1 | 11304 | PDI_H | -0.000219532 | 0.001014953 | -0.216297302 | 0.828759919 |
| clstn1 | 13320 | PDI_H | -0.000201152 | 0.00093062 | -0.216147932 | 0.828875751 |
| tp53inp1 | 13388 | PDI_H | -0.000200224 | 0.000928598 | -0.21561986 | 0.829287362 |
| nppb | 13388 | PDI_H | 0.000193771 | 0.000904046 | 0.214337325 | 0.83028728 |
| vegfc | 13699 | PDI_H | -0.000195317 | 0.000916615 | -0.21308506 | 0.831263791 |
| frmd4b | 11355 | PDI_H | -0.000215456 | 0.001011504 | -0.213005262 | 0.831326686 |
| thbd | 13618 | PDI_H | -0.00018721 | 0.000879812 | -0.21278365 | 0.831498905 |
| treh | 11790 | PDI_H | 0.000205676 | 0.000967661 | 0.212550081 | 0.831681589 |
| lrch4 | 11355 | PDI_H | -0.00021065 | 0.000993828 | -0.211958025 | 0.83214359 |
| btd | 11817 | PDI_H | -0.000205397 | 0.000975846 | -0.210480917 | 0.833295977 |
| hadh | 11355 | PDI_H | -0.000212918 | 0.001011587 | -0.21047923 | 0.833297441 |
| egf | 13633 | PDI_H | -0.000194021 | 0.000921948 | -0.210446797 | 0.833322119 |
| vcpkmt | 11304 | PDI_H | 0.000213077 | 0.001013678 | 0.210201726 | 0.833514025 |
| gbp4 | 13320 | PDI_H | 0.000195676 | 0.000933444 | 0.209628221 | 0.833961061 |
| stxbp3 | 13308 | PDI_H | -0.000195153 | 0.000932843 | -0.209202931 | 0.834293029 |
| il6r | 13551 | PDI_H | 0.000190133 | 0.00091543 | 0.207698439 | 0.835467561 |
| nphs1 | 11411 | PDI_H | -0.000209046 | 0.001006525 | -0.207691003 | 0.835473953 |
| b4gat1 | 13632 | PDI_H | -0.000182059 | 0.000876787 | -0.207643747 | 0.835510248 |
| pm20d1 | 13547 | PDI_H | 0.000188989 | 0.000911524 | 0.20733358 | 0.83575247 |
| pla2g1b | 13777 | PDI_H | 0.000181106 | 0.000873887 | 0.207241559 | 0.835824278 |
| ssc5d | 13689 | PDI_H | -0.000178073 | 0.000859798 | -0.207110184 | 0.835926892 |
| tpt1 | 13378 | PDI_H | -0.000192419 | 0.000930727 | -0.206740884 | 0.836215375 |
| arg1 | 13565 | PDI_H | 0.000191096 | 0.000925 | 0.20658987 | 0.836333275 |
| asrgl1 | 11474 | PDI_H | -0.000207713 | 0.001007324 | -0.206202781 | 0.836636178 |
| ervv_1 | 11355 | PDI_H | 0.000207682 | 0.001011507 | 0.205319391 | 0.837326279 |
| adra2a | 11355 | PDI_H | -0.000206079 | 0.001010764 | -0.203883898 | 0.838447886 |
| folh1 | 11411 | PDI_H | -0.000204522 | 0.001004581 | -0.203589638 | 0.838677826 |
| clns1a | 11483 | PDI_H | -0.000204636 | 0.001008335 | -0.202944904 | 0.839181696 |
| evi5 | 11411 | PDI_H | -0.000204072 | 0.001005706 | -0.202914396 | 0.839205564 |
| il13 | 13378 | PDI_H | -0.000188259 | 0.000931846 | -0.202027495 | 0.839898304 |
| timm8a | 11767 | PDI_H | -0.000200418 | 0.000993738 | -0.201681298 | 0.840169374 |
| eif2ak3 | 11355 | PDI_H | -0.000202291 | 0.001011109 | -0.200068739 | 0.841430408 |

| Protein | N_used | term | beta | se | t | p_val |
|---|---|---|---|---|---|---|
| ifit1 | 11570 | PDI_H | -0.000200007 | 0.001002956 | -0.199417513 | 0.84193967 |
| lsp1 | 13621 | PDI_H | 0.000176439 | 0.000884858 | 0.199398485 | 0.841954024 |
| adgre2 | 13710 | PDI_H | 0.000174002 | 0.000880199 | 0.197685228 | 0.843294281 |
| insl4 | 11730 | PDI_H | -0.000194009 | 0.000985012 | -0.196960794 | 0.843861634 |
| bin2 | 13632 | PDI_H | -0.000181109 | 0.000920752 | -0.196696499 | 0.844067984 |
| itga6 | 13621 | PDI_H | -0.000179328 | 0.000921886 | -0.194523201 | 0.845769135 |
| igfl4 | 11411 | PDI_H | -0.000195912 | 0.001007945 | -0.194368019 | 0.845891193 |
| bmp10 | 11537 | PDI_H | -0.00018139 | 0.000934038 | -0.194200262 | 0.8460225 |
| ppp1r14d | 11757 | PDI_H | -0.000190863 | 0.000984276 | -0.193911966 | 0.846248168 |
| mcemp1 | 11411 | PDI_H | -0.000193562 | 0.001005271 | -0.192547038 | 0.847317156 |
| il9 | 11570 | PDI_H | -0.000192821 | 0.001003721 | -0.19210661 | 0.847662072 |
| mapkapk2 | 11570 | PDI_H | -0.000192356 | 0.001002839 | -0.19181114 | 0.847893514 |
| stx1b | 11483 | PDI_H | 0.000192602 | 0.001005654 | 0.191519191 | 0.848122237 |
| rgmb | 13784 | PDI_H | 0.000168863 | 0.00088367 | 0.191093219 | 0.848455377 |
| acsl1 | 11355 | PDI_H | -0.000192868 | 0.001011925 | -0.190594826 | 0.848846455 |
| akr1b10 | 11483 | PDI_H | -0.000189686 | 0.001002767 | -0.189162787 | 0.849968579 |
| itgb1bp1 | 13308 | PDI_H | -0.000176404 | 0.000932758 | -0.189120911 | 0.850000939 |
| ppbp | 11717 | PDI_H | 0.000188001 | 0.000995544 | 0.188842015 | 0.850219915 |
| slc9a3r2 | 11724 | PDI_H | -0.000183547 | 0.00097408 | -0.188430836 | 0.850542194 |
| ofd1 | 11570 | PDI_H | 0.00018588 | 0.001003278 | 0.185272944 | 0.853018209 |
| defb4a_defb4b | 13447 | PDI_H | -0.000160031 | 0.000865987 | -0.184795988 | 0.853391844 |
| tsnax | 11355 | PDI_H | 0.000185036 | 0.001008453 | 0.183485166 | 0.854420637 |
| cd22 | 13801 | PDI_H | 0.00015778 | 0.000860746 | 0.183306363 | 0.854560337 |
| zbtb16 | 13308 | PDI_H | -0.000166444 | 0.000927531 | -0.179448914 | 0.857587962 |
| afap1 | 11537 | PDI_H | 0.00017929 | 0.000999761 | 0.179332862 | 0.857679498 |
| edar | 13621 | PDI_H | -0.000164897 | 0.000922249 | -0.178798904 | 0.858098267 |
| vav3 | 11383 | PDI_H | -0.000180244 | 0.00100935 | -0.178573891 | 0.858275478 |
| ly75 | 13621 | PDI_H | 0.000162164 | 0.000916058 | 0.177023574 | 0.859492507 |
| tprkb | 11304 | PDI_H | 0.000179314 | 0.001015753 | 0.176532856 | 0.859878505 |
| ube2z | 11411 | PDI_H | 0.000177718 | 0.001007317 | 0.176426795 | 0.859961789 |
| ankra2 | 11304 | PDI_H | 0.00017871 | 0.001015837 | 0.175923547 | 0.86035716 |
| nos2 | 11355 | PDI_H | 0.000176492 | 0.001008979 | 0.174921314 | 0.861144584 |
| crebzf | 11304 | PDI_H | 0.000176745 | 0.001013703 | 0.174356252 | 0.861588619 |
| ahsg | 11794 | PDI_H | 0.000171182 | 0.000982633 | 0.174207298 | 0.861705543 |
| cd33 | 13699 | PDI_H | -0.000159091 | 0.000915579 | -0.173760232 | 0.862056468 |
| hrc | 11474 | PDI_H | -0.000172382 | 0.0009955 | -0.17316147 | 0.862527566 |
| susd4 | 11730 | PDI_H | 0.000167087 | 0.000968074 | 0.172597649 | 0.862970681 |
| znrf4 | 11730 | PDI_H | -0.000171044 | 0.000994319 | -0.172021523 | 0.863423579 |
| slc44a4 | 11483 | PDI_H | 0.00017346 | 0.001008865 | 0.171935542 | 0.863491237 |
| dsc2 | 13475 | PDI_H | -0.000151516 | 0.000882371 | -0.171714265 | 0.863664751 |
| siglec7 | 13618 | PDI_H | -0.00014856 | 0.000868956 | -0.170963788 | 0.864254782 |
| snx18 | 11724 | PDI_H | 0.000167287 | 0.000993264 | 0.168421403 | 0.86625468 |
| crtap | 11483 | PDI_H | 0.000166829 | 0.001003267 | 0.166285623 | 0.867935111 |
| spesp1 | 11781 | PDI_H | 0.000150784 | 0.000907288 | 0.166191905 | 0.868008785 |
| anxa1 | 11467 | PDI_H | -0.000162973 | 0.000981335 | -0.166072717 | 0.868102656 |
| arhgap25 | 13308 | PDI_H | -0.000153509 | 0.000927718 | -0.165469533 | 0.868576944 |
| ereg | 13535 | PDI_H | -0.000152027 | 0.000920059 | -0.165236227 | 0.868760522 |
| ment | 11537 | PDI_H | -0.000151478 | 0.000927682 | -0.163286927 | 0.87029539 |
| slmap | 11724 | PDI_H | -0.000161143 | 0.000990138 | -0.162748203 | 0.8707195 |
| tada3 | 11730 | PDI_H | -0.000161949 | 0.000995178 | -0.162734019 | 0.870730667 |
| hcg22 | 11355 | PDI_H | 0.000163112 | 0.0010111 | 0.161321542 | 0.87184303 |
| chit1 | 13687 | PDI_H | 0.000141862 | 0.000882591 | 0.160733857 | 0.872305396 |
| pfdn2 | 13320 | PDI_H | 0.00014989 | 0.000934691 | 0.1603629 | 0.872597647 |
| ada | 13801 | PDI_H | -0.000144596 | 0.000903032 | -0.160122862 | 0.872786638 |
| hs6st2 | 11411 | PDI_H | 0.000149778 | 0.000948102 | 0.157976329 | 0.874478249 |
| defb118 | 11383 | PDI_H | 0.000159809 | 0.001011745 | 0.157953685 | 0.874496099 |
| pecam1 | 13632 | PDI_H | -0.000142754 | 0.0009151 | -0.155998401 | 0.876036593 |
| treml1 | 11794 | PDI_H | -0.000154691 | 0.000992913 | -0.155795227 | 0.876197102 |
| fcer1a | 11757 | PDI_H | -0.000154515 | 0.000995068 | -0.155280673 | 0.87660272 |
| app | 13611 | PDI_H | -0.000142107 | 0.000919975 | -0.154468842 | 0.877242369 |
| slitrk1 | 11411 | PDI_H | 0.000139142 | 0.000911205 | 0.152700574 | 0.87863711 |
| tgoln2 | 11794 | PDI_H | -0.000147065 | 0.000969074 | -0.151758419 | 0.879380078 |
| fcrlb | 13308 | PDI_H | -0.000136827 | 0.000911626 | -0.150091593 | 0.88069463 |
| cd164l2 | 11483 | PDI_H | 0.000149661 | 0.001008877 | 0.148344165 | 0.88207377 |
| gfral | 11450 | PDI_H | -0.000143138 | 0.00096909 | -0.147703834 | 0.882579109 |
| traf2 | 13621 | PDI_H | -0.000135964 | 0.000920963 | -0.14763271 | 0.882634826 |
| scgb3a1 | 11537 | PDI_H | -0.000140419 | 0.000956385 | -0.146822446 | 0.883274736 |
| epha10 | 13320 | PDI_H | 0.000136983 | 0.000934647 | 0.146560723 | 0.883480977 |
| adgrb3 | 13526 | PDI_H | 0.000133813 | 0.00091504 | 0.146237099 | 0.883736401 |
| myl6b | 11483 | PDI_H | 0.000145059 | 0.001007064 | 0.144041002 | 0.885470623 |

| Protein | N_used | term | beta | se | t | p_val |
|---|---|---|---|---|---|---|
| nagpa | 11537 | PDI_H | 0.000142076 | 0.000986638 | 0.144000428 | 0.885502649 |
| nt5c3a | 13593 | PDI_H | 0.000131646 | 0.000923375 | 0.14256991 | 0.886631978 |
| cd207 | 13340 | PDI_H | 0.000130152 | 0.000923902 | 0.140872386 | 0.887972886 |
| prame | 11304 | PDI_H | -0.000142986 | 0.00101554 | -0.140798442 | 0.888031685 |
| myl4 | 11474 | PDI_H | -0.00013614 | 0.000981715 | -0.138675195 | 0.889709257 |
| fgf6 | 11411 | PDI_H | 0.000139293 | 0.001008688 | 0.138093122 | 0.890169261 |
| sla2 | 11757 | PDI_H | -0.000136152 | 0.000992956 | -0.137117634 | 0.890940164 |
| ccn2 | 13644 | PDI_H | 0.000125085 | 0.000916047 | 0.136548288 | 0.89138987 |
| pdxdc1 | 11304 | PDI_H | 0.00013782 | 0.001015189 | 0.135758253 | 0.892014814 |
| gga1 | 13320 | PDI_H | 0.00012625 | 0.000930384 | 0.135696166 | 0.892063531 |
| wfikkn2 | 13677 | PDI_H | -0.000115542 | 0.00085257 | -0.135521966 | 0.892201194 |
| cd6 | 13779 | PDI_H | 0.000120329 | 0.000889664 | 0.135251646 | 0.892414892 |
| brme1 | 11383 | PDI_H | 0.000136577 | 0.001011626 | 0.135007543 | 0.8926083 |
| lama4 | 13710 | PDI_H | -0.000121917 | 0.00090633 | -0.134517158 | 0.892995621 |
| traf3ip2 | 11355 | PDI_H | 0.000136101 | 0.001012577 | 0.134410611 | 0.893080276 |
| ube2b | 11730 | PDI_H | 0.00013365 | 0.000994759 | 0.134354375 | 0.893124666 |
| kirrel2 | 13320 | PDI_H | -0.000122599 | 0.000913843 | -0.134157589 | 0.89327999 |
| lypla2 | 11383 | PDI_H | -0.000132676 | 0.001010252 | -0.131329478 | 0.895516997 |
| adamts1 | 11411 | PDI_H | 0.000130224 | 0.00099222 | 0.131244685 | 0.895584064 |
| grin2b | 11383 | PDI_H | -0.000129984 | 0.001010276 | -0.12866214 | 0.89762727 |
| map1lc3b2 | 11355 | PDI_H | -0.000130151 | 0.001012023 | -0.128604367 | 0.897672991 |
| gys1 | 13388 | PDI_H | -0.000119181 | 0.000928025 | -0.128424068 | 0.897815316 |
| epb41l5 | 11483 | PDI_H | 0.000128651 | 0.001008372 | 0.127583388 | 0.898480915 |
| diablo | 13568 | PDI_H | -0.00011601 | 0.000924754 | -0.125449302 | 0.900169719 |
| chm | 11483 | PDI_H | 0.000126346 | 0.001008339 | 0.12530153 | 0.900287037 |
| cdan1 | 11767 | PDI_H | 0.000121986 | 0.00099579 | 0.122502151 | 0.902503436 |
| sestd1 | 13320 | PDI_H | 0.000112682 | 0.000933764 | 0.120675097 | 0.903950206 |
| nrp2 | 13632 | PDI_H | 0.000106811 | 0.000886746 | 0.12045289 | 0.904126173 |
| spp1 | 13777 | PDI_H | -0.000106667 | 0.000887156 | -0.120234976 | 0.904298767 |
| mindy1 | 11730 | PDI_H | 0.000118614 | 0.000995649 | 0.11913191 | 0.905172892 |
| pam | 13742 | PDI_H | -0.000107424 | 0.000902434 | -0.119037763 | 0.905247179 |
| rnaseh2a | 11757 | PDI_H | -0.000117882 | 0.000992907 | -0.118724165 | 0.905495922 |
| gh2 | 13308 | PDI_H | 0.00011017 | 0.000928261 | 0.118684536 | 0.905527081 |
| nars1 | 11757 | PDI_H | -0.000116841 | 0.000992901 | -0.11767657 | 0.906325945 |
| ifit3 | 11570 | PDI_H | 0.000117036 | 0.001002302 | 0.11676677 | 0.907046906 |
| spock1 | 13552 | PDI_H | 0.000105682 | 0.000905239 | 0.11674487 | 0.907063963 |
| fgfbp2 | 11790 | PDI_H | -0.000114011 | 0.000979098 | -0.116444847 | 0.90730198 |
| timp2 | 11781 | PDI_H | -0.00011332 | 0.000977097 | -0.115975884 | 0.907673633 |
| vgf | 11757 | PDI_H | -0.000112169 | 0.000967345 | -0.115955799 | 0.907689555 |
| gc | 11817 | PDI_H | -0.000113893 | 0.000987182 | -0.115372064 | 0.908152183 |
| obp2b | 13513 | PDI_H | -8.51e-05 | 0.000744185 | -0.11428634 | 0.90901251 |
| pnpt1 | 13378 | PDI_H | 0.000104593 | 0.000923012 | 0.113317153 | 0.90978082 |
| tpbgl | 11483 | PDI_H | 0.000113973 | 0.001007849 | 0.113085541 | 0.909964717 |
| ftcd | 11741 | PDI_H | -0.000102937 | 0.000912277 | -0.112835742 | 0.910162712 |
| clec7a | 13801 | PDI_H | -0.00010039 | 0.000890786 | -0.11269768 | 0.910271881 |
| ndufb7 | 11724 | PDI_H | -0.000111709 | 0.000994518 | -0.112325216 | 0.910567474 |
| ednrb | 11411 | PDI_H | -0.000112598 | 0.001007167 | -0.111796511 | 0.910986723 |
| cd86 | 11724 | PDI_H | -0.000109143 | 0.000982028 | -0.111140368 | 0.911506942 |
| ehbp1 | 11781 | PDI_H | 0.000109377 | 0.000990089 | 0.110472099 | 0.91203686 |
| sms | 11383 | PDI_H | -0.000110902 | 0.001009229 | -0.109887714 | 0.912500365 |
| slc13a1 | 11730 | PDI_H | 0.000108877 | 0.00099624 | 0.109287544 | 0.912976297 |
| sez6l2 | 13579 | PDI_H | 8.99e-05 | 0.000830924 | 0.108233427 | 0.913812131 |
| myh4 | 11355 | PDI_H | 0.000109071 | 0.001012324 | 0.107743247 | 0.914201269 |
| muc16 | 13579 | PDI_H | -9.93e-05 | 0.000924253 | -0.10748899 | 0.914402649 |
| mb | 13800 | PDI_H | 8.17e-05 | 0.000771675 | 0.105862703 | 0.915692827 |
| cdh4 | 11383 | PDI_H | 0.00010647 | 0.00101133 | 0.105276701 | 0.916158107 |
| cep112 | 11355 | PDI_H | 0.000104782 | 0.001008158 | 0.103934572 | 0.917223109 |
| tap1 | 11570 | PDI_H | -0.000103853 | 0.001003793 | -0.103460277 | 0.91759947 |
| il2rb | 13378 | PDI_H | 9.47e-05 | 0.000929139 | 0.101924342 | 0.918818257 |
| btnl10 | 11304 | PDI_H | 0.000103358 | 0.001015714 | 0.101759121 | 0.918949681 |
| ophn1 | 11767 | PDI_H | -9.98e-05 | 0.00099457 | -0.100314455 | 0.920096393 |
| spred2 | 11411 | PDI_H | -0.000100807 | 0.001008094 | -0.09999745 | 0.920348109 |
| pafah2 | 11570 | PDI_H | -9.9e-05 | 0.001003315 | -0.09864485 | 0.92142197 |
| dnajb8 | 13388 | PDI_H | -9.15e-05 | 0.000930319 | -0.098334317 | 0.921668303 |
| pdgfb | 13710 | PDI_H | -8.89e-05 | 0.000915897 | -0.097036803 | 0.922698588 |
| fgf3 | 11411 | PDI_H | 9.78e-05 | 0.00100858 | 0.096966631 | 0.922754599 |
| st8sia1 | 11411 | PDI_H | -9.66e-05 | 0.001006882 | -0.095984139 | 0.923534857 |
| islr2 | 13134 | PDI_H | 8.9e-05 | 0.000934168 | 0.095253839 | 0.924114662 |
| ctsv | 13717 | PDI_H | -8.2e-05 | 0.000863735 | -0.094991017 | 0.924323351 |
| ranbp2 | 11383 | PDI_H | -9.45e-05 | 0.001009498 | -0.093570977 | 0.925451614 |

| Protein | N_used | term | beta | se | t | p_val |
|---|---|---|---|---|---|---|
| guca2a | 13544 | PDI_H | -8.14e-05 | 0.000891422 | -0.091261897 | 0.927285844 |
| tgfbi | 13551 | PDI_H | 8.1e-05 | 0.000903573 | 0.089597714 | 0.928608226 |
| gstt2b | 11767 | PDI_H | 8.9e-05 | 0.000994286 | 0.089466529 | 0.928712677 |
| adamts16 | 13388 | PDI_H | 7.64e-05 | 0.000855079 | 0.089354212 | 0.928801749 |
| cd82 | 11570 | PDI_H | 8.96e-05 | 0.001003003 | 0.089325863 | 0.928824488 |
| ppp1r9b | 13801 | PDI_H | -8.13e-05 | 0.000913688 | -0.088998033 | 0.929084766 |
| fut8 | 13552 | PDI_H | -8.15e-05 | 0.000921595 | -0.088414262 | 0.929548734 |
| mansc1 | 13699 | PDI_H | 8.07e-05 | 0.000914429 | 0.088249001 | 0.929680063 |
| cdhr5 | 13618 | PDI_H | 7.76e-05 | 0.000879992 | 0.088238659 | 0.929688291 |
| il34 | 13301 | PDI_H | 7.92e-05 | 0.000934293 | 0.084766839 | 0.932448034 |
| cdc25a | 11304 | PDI_H | 8.48e-05 | 0.001015253 | 0.083486294 | 0.933466355 |
| cd3d | 11383 | PDI_H | -8.44e-05 | 0.001010979 | -0.083460039 | 0.93348722 |
| bex3 | 11304 | PDI_H | -8.39e-05 | 0.001014821 | -0.082636433 | 0.934142095 |
| adh4 | 13388 | PDI_H | -6.82e-05 | 0.000827111 | -0.082472559 | 0.934272173 |
| was | 13378 | PDI_H | 7.66e-05 | 0.000931287 | 0.082220434 | 0.934472655 |
| wif1 | 13753 | PDI_H | -7.26e-05 | 0.000887343 | -0.08185349 | 0.934764412 |
| pms1 | 11483 | PDI_H | 8.19e-05 | 0.001005581 | 0.081468189 | 0.935071044 |
| robo1 | 13677 | PDI_H | 7.22e-05 | 0.000888078 | 0.081337836 | 0.935174476 |
| wasf3 | 13320 | PDI_H | 7.43e-05 | 0.00093469 | 0.079523301 | 0.936617594 |
| fam3b | 13579 | PDI_H | -7.21e-05 | 0.00090934 | -0.079325336 | 0.936775024 |
| tbr1 | 11483 | PDI_H | 7.97e-05 | 0.001008723 | 0.078992206 | 0.9370402 |
| sowaha | 11483 | PDI_H | 7.93e-05 | 0.001008724 | 0.078661061 | 0.937303591 |
| mtss1 | 11724 | PDI_H | -7.77e-05 | 0.000995388 | -0.078044349 | 0.937794108 |
| vps37a | 13579 | PDI_H | -7.04e-05 | 0.000919273 | -0.076566385 | 0.938969631 |
| trim24 | 11535 | PDI_H | 7.62e-05 | 0.000995554 | 0.076535143 | 0.938994685 |
| crx | 13388 | PDI_H | -7.06e-05 | 0.000930407 | -0.075881701 | 0.93951435 |
| mprip | 11710 | PDI_H | 7.42e-05 | 0.000992111 | 0.074755134 | 0.940410818 |
| dennd2b | 11355 | PDI_H | 7.42e-05 | 0.001012773 | 0.073215705 | 0.94163576 |
| tmem132a | 11474 | PDI_H | 7.36e-05 | 0.001005161 | 0.07318856 | 0.941657347 |
| prdx5 | 13801 | PDI_H | 6.69e-05 | 0.00091393 | 0.073159856 | 0.941679972 |
| nfat5 | 11411 | PDI_H | -7.33e-05 | 0.001006903 | -0.072776399 | 0.941985329 |
| krt17 | 11355 | PDI_H | 7.24e-05 | 0.001012593 | 0.071516194 | 0.942988197 |
| cyp24a1 | 11355 | PDI_H | -7.2e-05 | 0.001012748 | -0.071077194 | 0.943337572 |
| vnn2 | 13579 | PDI_H | 6.45e-05 | 0.000910897 | 0.070771425 | 0.943580718 |
| lta4h | 13753 | PDI_H | -6.24e-05 | 0.000895933 | -0.069695883 | 0.944436733 |
| ppp2r5a | 11724 | PDI_H | -6.92e-05 | 0.000993964 | -0.069648701 | 0.944474462 |
| znf75d | 11570 | PDI_H | -6.92e-05 | 0.001003708 | -0.068896294 | 0.94507336 |
| nenf | 11730 | PDI_H | 6.65e-05 | 0.000993453 | 0.066954543 | 0.946619034 |
| ctsz | 13653 | PDI_H | -5.73e-05 | 0.000872388 | -0.065725201 | 0.947597572 |
| aida | 11483 | PDI_H | 6.54e-05 | 0.001007959 | 0.064891083 | 0.948261852 |
| brd3 | 11355 | PDI_H | 6.35e-05 | 0.001012607 | 0.062751735 | 0.949965305 |
| sema7a | 13750 | PDI_H | 5.59e-05 | 0.000897183 | 0.062266292 | 0.950351677 |
| rabepk | 13308 | PDI_H | 5.72e-05 | 0.000932113 | 0.061382108 | 0.951055824 |
| vtcn1 | 13308 | PDI_H | -5.61e-05 | 0.000928496 | -0.060368076 | 0.951863391 |
| fgf9 | 11271 | PDI_H | -6.13e-05 | 0.001015923 | -0.060313496 | 0.951907024 |
| ank2 | 11355 | PDI_H | -6.06e-05 | 0.001011547 | -0.059930382 | 0.952212138 |
| dhrs4l2 | 11767 | PDI_H | -5.94e-05 | 0.000994695 | -0.059765775 | 0.9523432 |
| cd40lg | 13801 | PDI_H | 5.39e-05 | 0.000915442 | 0.058840417 | 0.953080069 |
| glyr1 | 11730 | PDI_H | -5.8e-05 | 0.000990521 | -0.058538766 | 0.953320483 |
| ppm1b | 11411 | PDI_H | 5.84e-05 | 0.001007608 | 0.057989672 | 0.953757871 |
| mapk13 | 11304 | PDI_H | 5.76e-05 | 0.001013849 | 0.056850072 | 0.954665633 |
| xiap | 11411 | PDI_H | 5.71e-05 | 0.001006629 | 0.056706382 | 0.954780084 |
| pttg1 | 11570 | PDI_H | 5.56e-05 | 0.001003662 | 0.055393067 | 0.955826277 |
| il33 | 13378 | PDI_H | 5.08e-05 | 0.000931786 | 0.054490413 | 0.956545261 |
| coro6 | 11383 | PDI_H | 5.37e-05 | 0.001003215 | 0.053556304 | 0.957289611 |
| dcun1d1 | 11383 | PDI_H | 5.39e-05 | 0.001009244 | 0.053441429 | 0.957381136 |
| rictor | 11411 | PDI_H | -5.25e-05 | 0.001008197 | -0.052027066 | 0.958508039 |
| ddx25 | 11483 | PDI_H | -5.13e-05 | 0.001006556 | -0.050924194 | 0.959386813 |
| ca11 | 13308 | PDI_H | 4.58e-05 | 0.000928422 | 0.049320462 | 0.960664654 |
| mslnl | 11483 | PDI_H | 4.96e-05 | 0.00100739 | 0.049253009 | 0.960718525 |
| cd177 | 13614 | PDI_H | -4.38e-05 | 0.000919878 | -0.047580159 | 0.962051543 |
| magea3 | 11570 | PDI_H | 4.49e-05 | 0.001003593 | 0.044778294 | 0.9642848 |
| phospho1 | 13552 | PDI_H | -3.72e-05 | 0.000855853 | -0.043505501 | 0.965299221 |
| il24 | 13378 | PDI_H | 3.56e-05 | 0.000932452 | 0.038191342 | 0.969535694 |
| procr | 11355 | PDI_H | -3.76e-05 | 0.001010271 | -0.037185076 | 0.970338092 |
| cd101 | 11724 | PDI_H | 3.45e-05 | 0.000984956 | 0.035067601 | 0.972026434 |
| il15ra | 13378 | PDI_H | 3.15e-05 | 0.00091147 | 0.03456305 | 0.972428682 |
| sag | 11383 | PDI_H | -3.49e-05 | 0.001011315 | -0.034530588 | 0.972454658 |
| lrrfip1 | 11730 | PDI_H | -3.3e-05 | 0.000991427 | -0.033333697 | 0.97340905 |
| glp1r | 11383 | PDI_H | 3.36e-05 | 0.001010913 | 0.033262732 | 0.973465657 |

| Protein | N_used | term | beta | se | t | p_val |
|---|---|---|---|---|---|---|
| steap4 | 11383 | PDI_H | 3.15e-05 | 0.001011476 | 0.031145128 | 0.975154346 |
| treml2 | 13632 | PDI_H | -2.72e-05 | 0.000902028 | -0.030202804 | 0.975905755 |
| tsc1 | 11411 | PDI_H | -2.96e-05 | 0.001007824 | -0.029331172 | 0.97660098 |
| klrf1 | 11411 | PDI_H | 2.8e-05 | 0.000965103 | 0.028977413 | 0.976883112 |
| idi2 | 13320 | PDI_H | 2.61e-05 | 0.000904493 | 0.02889458 | 0.976949102 |
| doc2b | 11483 | PDI_H | 2.91e-05 | 0.001009032 | 0.028824157 | 0.977005336 |
| rac3 | 11483 | PDI_H | -2.88e-05 | 0.001007389 | -0.028630329 | 0.977159921 |
| cox6b1 | 11781 | PDI_H | 2.71e-05 | 0.000992207 | 0.027286166 | 0.978231954 |
| abo | 11794 | PDI_H | -2.65e-05 | 0.000993542 | -0.026626509 | 0.978758081 |
| adgrv1 | 11383 | PDI_H | -2.65e-05 | 0.001011197 | -0.026159215 | 0.979130806 |
| idua | 13753 | PDI_H | -2.33e-05 | 0.000903476 | -0.025840261 | 0.979385124 |
| scn2a | 11383 | PDI_H | -2.44e-05 | 0.001011122 | -0.024157202 | 0.98072764 |
| akr7l | 11411 | PDI_H | 2.35e-05 | 0.000982758 | 0.023866377 | 0.980959611 |
| ncr1 | 13597 | PDI_H | -2.04e-05 | 0.000904327 | -0.022590024 | 0.981977633 |
| qsox1 | 11560 | PDI_H | 2.13e-05 | 0.000979455 | 0.021756653 | 0.982642448 |
| arid3a | 11383 | PDI_H | 2.08e-05 | 0.001010797 | 0.020572017 | 0.983587424 |
| tst | 13300 | PDI_H | 1.9e-05 | 0.000934701 | 0.020304783 | 0.983800546 |
| nphs2 | 11411 | PDI_H | 2.03e-05 | 0.001008962 | 0.020092941 | 0.983969583 |
| arl2bp | 11355 | PDI_H | -1.93e-05 | 0.001011523 | -0.019043933 | 0.984806393 |
| gpha2 | 11724 | PDI_H | -1.78e-05 | 0.000965801 | -0.018417543 | 0.985306071 |
| prtn3 | 13287 | PDI_H | 1.64e-05 | 0.000918855 | 0.017833816 | 0.985771696 |
| gmpr | 13801 | PDI_H | -1.55e-05 | 0.000899126 | -0.017222957 | 0.986258997 |
| gipr | 11411 | PDI_H | 1.69e-05 | 0.001008146 | 0.016781554 | 0.986611179 |
| fh | 11383 | PDI_H | -1.67e-05 | 0.00100997 | -0.016575787 | 0.986775332 |
| bcl2l15 | 11411 | PDI_H | 1.48e-05 | 0.000995733 | 0.014842443 | 0.988158138 |
| phldb2 | 11304 | PDI_H | -1.5e-05 | 0.001015611 | -0.014811908 | 0.988182501 |
| actn2 | 11355 | PDI_H | -1.32e-05 | 0.000978254 | -0.013543082 | 0.989194752 |
| agr2 | 13219 | PDI_H | 1.21e-05 | 0.000923637 | 0.013113832 | 0.989537174 |
| mfap4 | 11537 | PDI_H | -1.26e-05 | 0.000968251 | -0.01305085 | 0.98958745 |
| vstm1 | 13320 | PDI_H | 1.17e-05 | 0.000930671 | 0.01259593 | 0.989950356 |
| mmp13 | 13320 | PDI_H | 8.52e-06 | 0.000925221 | 0.009205573 | 0.992655257 |
| inpp5j | 11757 | PDI_H | 8.88e-06 | 0.000979132 | 0.009070593 | 0.992762967 |
| cpvl | 13735 | PDI_H | -7.53e-06 | 0.000915084 | -0.008226992 | 0.993436003 |
| icam2 | 13800 | PDI_H | 6.45e-06 | 0.000915201 | 0.007049605 | 0.994375378 |
| gimap7 | 11537 | PDI_H | -4.89e-06 | 0.000988302 | -0.00494428 | 0.996055137 |
| vsig10l | 11355 | PDI_H | -4.96e-06 | 0.001006988 | -0.004923088 | 0.996072046 |
| scgb2a2 | 11355 | PDI_H | -4e-06 | 0.00101213 | -0.003954634 | 0.996844737 |
| gpihbp1 | 11724 | PDI_H | 3.45e-06 | 0.000993305 | 0.0034758 | 0.997226777 |
| wasf1 | 13388 | PDI_H | -3.06e-06 | 0.000929374 | -0.003296069 | 0.997370171 |
| ambn | 13378 | PDI_H | 2.31e-06 | 0.000928395 | 0.002482896 | 0.998018974 |
| arnt | 13378 | PDI_H | -1.54e-06 | 0.0009298 | -0.001654792 | 0.998679692 |

## Supplementary Table 6. Metabolome-wide associations between the unhealthy plant-based diet index (PDI-U) and circulating metabolites in the UK Biobank

| Metabolite | N_used | term | beta | se | t | p_val |
|---|---|---|---|---|---|---|
| Ratio.of.polyunsaturated.FA.to.total.FA | 129001 | PDI_U | -0.004565706738 | 0.0002880393915 | -15.85098037 | 1.56546e-56 |
| Ratio.of.saturated.FA.to.total.FA | 129001 | PDI_U | 0.00488080144 | 0.0003114226619 | 15.6725956 | 2.61969e-55 |
| TG.in.L.VLDL | 129001 | PDI_U | 0.004426038536 | 0.0002965336306 | 14.92592435 | 2.46271e-50 |
| TG.in.VLDL | 129001 | PDI_U | 0.004365956674 | 0.0002962171995 | 14.73903839 | 3.96857e-49 |
| TG | 129001 | PDI_U | 0.004387427126 | 0.000298790431 | 14.6839613 | 8.94431e-49 |
| TG.in.V..L.VLDL | 129001 | PDI_U | 0.004261248488 | 0.0002903404676 | 14.67672944 | 9.94922e-49 |
| Ratio.of.omega.6.FA.to.total.FA | 129001 | PDI_U | -0.004177050508 | 0.0002918994616 | -14.30989453 | 2.06016e-46 |
| Saturated.FA | 129001 | PDI_U | 0.004438529576 | 0.0003101730085 | 14.30985113 | 2.06144e-46 |
| TG.in.M.VLDL | 129001 | PDI_U | 0.004249229614 | 0.0003021725352 | 14.06226284 | 6.99298e-45 |
| TLs.in.V..L.VLDL | 129001 | PDI_U | 0.00407032739 | 0.0002928674132 | 13.89819149 | 6.98809e-44 |
| TG.in.HDL | 129001 | PDI_U | 0.004324059176 | 0.000311211806 | 13.89426459 | 7.38148e-44 |
| TLs.in.L.VLDL | 129001 | PDI_U | 0.004112480088 | 0.0002972150074 | 13.83671748 | 1.64415e-43 |
| Con.of.V..L.VLDL.particles | 129001 | PDI_U | 0.004022304398 | 0.0002937370709 | 13.69355385 | 1.1885e-42 |
| Con.of.L.VLDL.particles | 129001 | PDI_U | 0.004043505021 | 0.0002969056498 | 13.61882141 | 3.31079e-42 |
| TG.in.M.HDL | 129001 | PDI_U | 0.004223180702 | 0.0003106430075 | 13.59496464 | 4.58628e-42 |
| TG.in.S.VLDL | 129001 | PDI_U | 0.003997553947 | 0.0003026077147 | 13.21035041 | 8.11675e-40 |
| TLs.in.VLDL | 129001 | PDI_U | 0.004015396849 | 0.0003047289483 | 13.17694584 | 1.26362e-39 |
| TG.in.S.HDL | 129001 | PDI_U | 0.003877332271 | 0.0002948065786 | 13.15212262 | 1.75451e-39 |
| PL.in.V..L.VLDL | 129001 | PDI_U | 0.003855762456 | 0.0002946891019 | 13.08417051 | 4.2953e-39 |
| PL.in.L.VLDL | 129001 | PDI_U | 0.003823835573 | 0.0002953624095 | 12.94624993 | 2.60676e-38 |
| TG.in.S.LDL | 129001 | PDI_U | 0.003919340338 | 0.000303919405 | 12.89598582 | 5.0057e-38 |
| Free.Chol.in.L.VLDL | 129001 | PDI_U | 0.003794119353 | 0.0002965243761 | 12.79530339 | 1.83566e-37 |
| Free.Chol.in.V..L.VLDL | 129001 | PDI_U | 0.003759692893 | 0.0002972059748 | 12.65012554 | 1.17448e-36 |
| Ratio.of.polyunsaturated.FA.to.monounsaturated.FA | 129001 | PDI_U | -0.003560288057 | 0.0002831765411 | -12.57268008 | 3.1342e-36 |
| Average.diameter.for.VLDL.particles | 129001 | PDI_U | 0.003574099204 | 0.0002846575056 | 12.55578769 | 3.8794e-36 |
| Con.of.chylomicrons.and.extremely.L.VLDL.particles | 129001 | PDI_U | 0.003710536953 | 0.0002961300293 | 12.53009349 | 5.36335e-36 |
| CE.in.chylomicrons.and.extremely.L.VLDL | 129001 | PDI_U | 0.003697291467 | 0.0002957458573 | 12.50158329 | 7.67703e-36 |
| TG.in.chylomicrons.and.extremely.L.VLDL | 129001 | PDI_U | 0.003687817861 | 0.0002971293171 | 12.41149105 | 2.37185e-35 |
| Chol.to.TLs.ratio.in.V..L.VLDL | 129001 | PDI_U | -0.003621620233 | 0.0002929017088 | -12.36462651 | 4.2515e-35 |
| TLs.in.chylomicrons.and.extremely.L.VLDL | 129001 | PDI_U | 0.003632177773 | 0.000294767375 | 12.3221838 | 7.19893e-35 |
| Chol.in.chylomicrons.and.extremely.L.VLDL | 129001 | PDI_U | 0.003633091918 | 0.000295063522 | 12.3129145 | 8.07453e-35 |
| Monounsaturated.FA | 129001 | PDI_U | 0.003694936898 | 0.000305861806 | 12.08041287 | 1.39768e-33 |
| Ratio.of.TG.to.PG | 129001 | PDI_U | 0.003373347321 | 0.0002801744199 | 12.04016884 | 2.27712e-33 |
| Chol.in.V..L.VLDL | 129001 | PDI_U | 0.003599383387 | 0.0003009006563 | 11.96203236 | 5.84745e-33 |
| PL.in.chylomicrons.and.extremely.L.VLDL | 129001 | PDI_U | 0.003496342233 | 0.0002932207133 | 11.9239265 | 9.24187e-33 |
| Chol.in.L.VLDL | 129001 | PDI_U | 0.00358847724 | 0.0003028211388 | 11.85015436 | 2.23278e-32 |
| TG.to.TLs.ratio.in.V..L.HDL | 129001 | PDI_U | 0.003367281554 | 0.0002842420651 | 11.8465279 | 2.3314e-32 |
| CE.to.TLs.ratio.in.V..L.VLDL | 129001 | PDI_U | -0.003471822341 | 0.0002934568078 | -11.83077798 | 2.81239e-32 |
| PL.in.VLDL | 129001 | PDI_U | 0.003622730794 | 0.0003088461052 | 11.72988985 | 9.29698e-32 |
| Free.Chol.in.chylomicrons.and.extremely.L.VLDL | 129001 | PDI_U | 0.003458205901 | 0.000295697699 | 11.69507207 | 1.4013e-31 |
| Degree.of.unsaturation | 129001 | PDI_U | -0.003419585243 | 0.0002925775163 | -11.6877923 | 1.52659e-31 |
| TG.to.TLs.ratio.in.L.HDL | 129001 | PDI_U | 0.003368944857 | 0.0002898019069 | 11.62499203 | 3.18864e-31 |
| Total.FA | 129001 | PDI_U | 0.003629842371 | 0.0003123557145 | 11.6208611 | 3.34648e-31 |
| TLs.in.M.VLDL | 129001 | PDI_U | 0.003642871755 | 0.0003135055511 | 11.61979985 | 3.38826e-31 |
| Free.Chol.to.TLs.ratio.in.V..L.VLDL | 129001 | PDI_U | -0.003487021605 | 0.0003003665856 | -11.60921944 | 3.83424e-31 |
| TG.in.V..L.HDL | 129001 | PDI_U | 0.003646698585 | 0.0003162345755 | 11.53162515 | 9.46351e-31 |
| TG.in.L.HDL | 129001 | PDI_U | 0.003526368158 | 0.0003086019398 | 11.42691508 | 3.17277e-30 |
| CE.to.TLs.ratio.in.L.VLDL | 129001 | PDI_U | -0.003332709502 | 0.0002959063178 | -11.2627183 | 2.06925e-29 |
| TLs.in.LP.particles | 129001 | PDI_U | 0.00351776317 | 0.000313159073 | 11.23315105 | 2.8922e-29 |
| CE.to.TLs.ratio.in.L.HDL | 129001 | PDI_U | -0.003174749317 | 0.0002827103667 | -11.22968837 | 3.0077e-29 |
| TLs.in.S.VLDL | 129001 | PDI_U | 0.00349242967 | 0.0003111347812 | 11.22481279 | 3.17811e-29 |
| Free.Chol.in.VLDL | 129001 | PDI_U | 0.00348655776 | 0.0003116043285 | 11.18905433 | 4.75777e-29 |
| Con.of.VLDL.particles | 129001 | PDI_U | 0.003487628736 | 0.0003137002109 | 11.11771244 | 1.06016e-28 |
| TG.to.TLs.ratio.in.M.HDL | 129001 | PDI_U | 0.003283262709 | 0.000295642676 | 11.10551005 | 1.21526e-28 |
| TG.to.TLs.ratio.in.V..L.VLDL | 129001 | PDI_U | 0.003323958834 | 0.0003001212146 | 11.07538778 | 1.70128e-28 |
| Con.of.S.VLDL.particles | 129001 | PDI_U | 0.003429588465 | 0.0003100968475 | 11.05973341 | 2.0256e-28 |
| TG.in.M.LDL | 129001 | PDI_U | 0.003387736763 | 0.0003064364124 | 11.05526832 | 2.12886e-28 |
| CE.in.V..L.VLDL | 129001 | PDI_U | 0.00335955109 | 0.0003049640708 | 11.01621933 | 3.28575e-28 |
| CE.to.TLs.ratio.in.M.HDL | 129001 | PDI_U | -0.003276076473 | 0.0002973968816 | -11.01584003 | 3.29961e-28 |
| CE.to.TLs.ratio.in.M.VLDL | 129001 | PDI_U | -0.003112068569 | 0.0002872064891 | -10.83564852 | 2.39729e-27 |
| PL.to.TLs.ratio.in.S.LDL | 129001 | PDI_U | -0.00336696036 | 0.0003132508149 | -10.74844885 | 6.18725e-27 |
| PL.in.S.HDL | 129001 | PDI_U | 0.003330715118 | 0.000311460149 | 10.69387248 | 1.11573e-26 |
| CE.in.L.VLDL | 129001 | PDI_U | 0.003299937614 | 0.0003088847516 | 10.68339436 | 1.24903e-26 |
| CE.to.TLs.ratio.in.M.LDL | 129001 | PDI_U | 0.003305507992 | 0.0003098485849 | 10.66813971 | 1.4718e-26 |
| Free.Chol.in.S.HDL | 129001 | PDI_U | 0.003250065966 | 0.0003103955864 | 10.47072223 | 1.20566e-25 |
| TLs.in.S.HDL | 129001 | PDI_U | 0.003272755113 | 0.0003128408218 | 10.46140684 | 1.33018e-25 |
| TG.in.V..S.VLDL | 129001 | PDI_U | 0.003210053483 | 0.0003079490038 | 10.42397749 | 1.97262e-25 |
| Chol.to.TLs.ratio.in.L.HDL | 129001 | PDI_U | -0.002874682832 | 0.0002776364786 | -10.3541251 | 4.10028e-25 |

| Metabolite | N_used | term | beta | se | t | p_val |
|---|---|---|---|---|---|---|
| TG.in.LDL | 129001 | PDI_U | 0.003187619414 | 0.0003080977587 | 10.34612984 | 4.45709e-25 |
| TG.to.TLs.ratio.in.S.HDL | 129001 | PDI_U | 0.002968017697 | 0.0002904195028 | 10.21976027 | 1.65261e-24 |
| Con.of.M.VLDL.particles | 129001 | PDI_U | 0.003222484724 | 0.000316027722 | 10.19684192 | 2.09243e-24 |
| Chol.to.TLs.ratio.in.M.VLDL | 129001 | PDI_U | -0.002931798417 | 0.0002889656039 | -10.14583873 | 3.53106e-24 |
| Free.Chol.to.TLs.ratio.in.chylomicrons.and.extremely.L.VLDL | 129001 | PDI_U | -0.003068806305 | 0.0003087977935 | -9.937915263 | 2.90265e-23 |
| PL.in.M.VLDL | 129001 | PDI_U | 0.00312466091 | 0.000316979726 | 9.857604931 | 6.47444e-23 |
| Free.Chol.to.TLs.ratio.in.M.LDL | 129001 | PDI_U | -0.002869495306 | 0.0002923686651 | -9.814647217 | 9.9181e-23 |
| VLDL_C | 129001 | PDI_U | 0.003094458183 | 0.0003158373241 | 9.79763298 | 1.17374e-22 |
| Chol.to.TLs.ratio.in.L.VLDL | 129001 | PDI_U | -0.003011039219 | 0.0003082778833 | -9.767289131 | 1.58384e-22 |
| PC | 129001 | PDI_U | 0.002868399131 | 0.0002960385279 | 9.689276431 | 3.40783e-22 |
| Total.PL.in.LP.particles | 129001 | PDI_U | 0.002913213308 | 0.0003010372278 | 9.677252639 | 3.83296e-22 |
| CE.in.M.LDL | 129001 | PDI_U | 0.003065909764 | 0.0003171594569 | 9.666777064 | 4.24586e-22 |
| Average.diameter.for.LDL.particles | 129001 | PDI_U | -0.00291472025 | 0.0003043852242 | -9.5757613 | 1.02806e-21 |
| CE.to.TLs.ratio.in.S.LDL | 129001 | PDI_U | 0.002990894417 | 0.0003137674791 | 9.53220017 | 1.56522e-21 |
| PG | 129001 | PDI_U | 0.00283626402 | 0.0002991514929 | 9.481029135 | 2.55848e-21 |
| TG.to.TLs.ratio.in.M.VLDL | 129001 | PDI_U | 0.002754115007 | 0.0002908112117 | 9.470456764 | 2.83096e-21 |
| Con.of.M.LDL.particles | 129001 | PDI_U | 0.003000772265 | 0.0003180542305 | 9.434781799 | 3.98006e-21 |
| Chol.to.TLs.ratio.in.M.HDL | 129001 | PDI_U | -0.002711723771 | 0.0002880956367 | -9.412581883 | 4.91682e-21 |
| PL.to.TLs.ratio.in.M.LDL | 129001 | PDI_U | -0.002978525565 | 0.0003174966626 | -9.381281495 | 6.61835e-21 |
| TG.in.L.LDL | 129001 | PDI_U | 0.00289953315 | 0.000309258719 | 9.375752312 | 6.97437e-21 |
| PL.in.S.VLDL | 129001 | PDI_U | 0.002932300776 | 0.0003162566248 | 9.27190309 | 1.85552e-20 |
| Ratio.of.monounsaturated.FA.to.total.FA | 129001 | PDI_U | 0.002608154603 | 0.0002835517882 | 9.198159599 | 3.69338e-20 |
| TG.in.IDL | 129001 | PDI_U | 0.002848965313 | 0.0003100544378 | 9.188597115 | 4.03664e-20 |
| TLs.in.M.LDL | 129001 | PDI_U | 0.002843851921 | 0.0003180385369 | 8.94184695 | 3.87608e-19 |
| Free.Chol.in.M.VLDL | 129001 | PDI_U | 0.002800192337 | 0.0003181530619 | 8.80139993 | 1.36741e-18 |
| CE.to.TLs.ratio.in.S.HDL | 129001 | PDI_U | -0.002763871438 | 0.0003143872865 | -8.791295188 | 1.49611e-18 |
| Con.of.S.LDL.particles | 129001 | PDI_U | 0.002798606345 | 0.0003187257206 | 8.780610297 | 1.64522e-18 |
| Chol.in.M.LDL | 129001 | PDI_U | 0.002784982707 | 0.0003182619219 | 8.750599791 | 2.1471e-18 |
| CE.in.S.LDL | 129001 | PDI_U | 0.002765325111 | 0.0003180955189 | 8.693379652 | 3.55833e-18 |
| Remnant_C | 129001 | PDI_U | 0.002739336 | 0.0003159740455 | 8.669496874 | 4.38937e-18 |
| Total.cholines | 129001 | PDI_U | 0.00255483603 | 0.0002963805773 | 8.620119622 | 6.76229e-18 |
| Chol.to.TLs.ratio.in.S.HDL | 129001 | PDI_U | -0.002673000925 | 0.0003117305323 | -8.574716458 | 1.00405e-17 |
| CE.in.VLDL | 129001 | PDI_U | 0.002722189963 | 0.0003175208905 | 8.57326256 | 1.01681e-17 |
| Con.of.S.HDL.particles | 129001 | PDI_U | 0.002691869252 | 0.0003154997315 | 8.532080962 | 1.45262e-17 |
| PL.to.TLs.ratio.in.IDL | 129001 | PDI_U | -0.002714657099 | 0.0003202876623 | -8.475684266 | 2.36115e-17 |
| TG.to.TLs.ratio.in.S.LDL | 129001 | PDI_U | 0.002563552869 | 0.0003024863847 | 8.47493639 | 2.37636e-17 |
| CE.in.LDL | 129001 | PDI_U | 0.002612013567 | 0.0003178978563 | 8.216518342 | 2.1141e-16 |
| TC.minus.HDL.C | 129001 | PDI_U | 0.002594408867 | 0.0003165837282 | 8.195016472 | 2.52823e-16 |
| Total.free.Chol | 129001 | PDI_U | 0.002533757563 | 0.0003102367989 | 8.167172855 | 3.18515e-16 |
| Free.Chol.to.TLs.ratio.in.S.LDL | 129001 | PDI_U | -0.002486600542 | 0.0003045640028 | -8.164459749 | 3.25752e-16 |
| PL.in.M.LDL | 129001 | PDI_U | 0.002584535423 | 0.0003185717709 | 8.112882747 | 4.98621e-16 |
| Con.of.V..S.VLDL.particles | 129001 | PDI_U | 0.002536776179 | 0.0003141406043 | 8.075289039 | 6.78909e-16 |
| PL.to.TLs.ratio.in.S.VLDL | 129001 | PDI_U | -0.002388924642 | 0.0002970191781 | -8.042997953 | 8.84025e-16 |
| CE.in.S.VLDL | 129001 | PDI_U | 0.002535941563 | 0.0003156677276 | 8.033578798 | 9.54606e-16 |
| Chol.in.S.VLDL | 129001 | PDI_U | 0.002526235288 | 0.0003169286092 | 7.970991619 | 1.58681e-15 |
| TLs.in.V..S.VLDL | 129001 | PDI_U | 0.002483619842 | 0.0003132114052 | 7.929531941 | 2.21719e-15 |
| Spectrometer.corrected.alanine | 129001 | PDI_U | 0.002460891235 | 0.0003136038685 | 7.847132902 | 4.28896e-15 |
| TLs.in.LDL | 129001 | PDI_U | 0.00248874713 | 0.0003176563292 | 7.834716015 | 4.73458e-15 |
| TG.to.TLs.ratio.in.S.VLDL | 129001 | PDI_U | 0.002370852319 | 0.0003039499411 | 7.800140741 | 6.22979e-15 |
| TLs.in.S.LDL | 129001 | PDI_U | 0.002475105942 | 0.0003190181052 | 7.758512454 | 8.65567e-15 |
| Free.Chol.to.TLs.ratio.in.M.VLDL | 129001 | PDI_U | -0.002268209797 | 0.0002952459371 | -7.682442032 | 1.57177e-14 |
| Free.Chol.in.S.VLDL | 129001 | PDI_U | 0.002440555726 | 0.0003182709124 | 7.66817083 | 1.75679e-14 |
| Chol.in.S.LDL | 129001 | PDI_U | 0.002411605196 | 0.0003190113627 | 7.559621623 | 4.06899e-14 |
| LDL_C | 129001 | PDI_U | 0.002378588555 | 0.0003173153123 | 7.495977859 | 6.6224e-14 |
| Ratio.of.linoleic.acid.to.total.FA | 129001 | PDI_U | -0.002184109636 | 0.0002922690727 | -7.472941341 | 7.89141e-14 |
| Chol.to.TLs.ratio.in.S.VLDL | 129001 | PDI_U | -0.002293685324 | 0.0003072762409 | -7.464571024 | 8.40937e-14 |
| ApoB | 129001 | PDI_U | 0.002361522779 | 0.0003183067792 | 7.41901503 | 1.18714e-13 |
| TC | 129001 | PDI_U | 0.002269612376 | 0.0003061024905 | 7.414550506 | 1.22781e-13 |
| Linoleic.acid | 129001 | PDI_U | 0.002318492232 | 0.0003155192288 | 7.34818046 | 2.02103e-13 |
| CE.in.L.LDL | 129001 | PDI_U | 0.002318230481 | 0.0003157504695 | 7.341970019 | 2.11705e-13 |
| Free.Chol.to.TLs.ratio.in.S.VLDL | 129001 | PDI_U | -0.002168358179 | 0.0002962178264 | -7.320147491 | 2.49132e-13 |
| Chol.in.S.HDL | 129001 | PDI_U | 0.002302570732 | 0.0003157274074 | 7.292907356 | 3.05066e-13 |
| TLs.in.L.LDL | 129001 | PDI_U | 0.002275595443 | 0.0003152318951 | 7.218798221 | 5.27363e-13 |
| PL.in.V..S.VLDL | 129001 | PDI_U | 0.002259411596 | 0.0003140669866 | 7.194043605 | 6.324e-13 |
| PL.in.LDL | 129001 | PDI_U | 0.002277085888 | 0.0003178424887 | 7.164196006 | 7.86603e-13 |
| Omega.6.FA | 129001 | PDI_U | 0.002212312887 | 0.0003129527939 | 7.069158448 | 1.56662e-12 |
| Total.esterified.Chol | 129001 | PDI_U | 0.002149174098 | 0.0003044020308 | 7.060314585 | 1.6696e-12 |
| Free.Chol.in.V..S.VLDL | 129001 | PDI_U | 0.002197454438 | 0.0003128977664 | 7.022915068 | 2.18354e-12 |
| Con.of.LDL.particles | 129001 | PDI_U | 0.002187105431 | 0.0003188079053 | 6.860260973 | 6.9043e-12 |
| Polyunsaturated.FA | 129001 | PDI_U | 0.002132824115 | 0.0003111329161 | 6.855025632 | 7.16184e-12 |

| Metabolite | N_used | term | beta | se | t | p_val |
|---|---|---|---|---|---|---|
| Chol.in.L.LDL | 129001 | PDI_U | 0.002147444651 | 0.0003146366393 | 6.825157605 | 8.82147e-12 |
| PL.in.L.LDL | 129001 | PDI_U | 0.002147316587 | 0.0003152688657 | 6.811064525 | 9.73013e-12 |
| PL.to.TLs.ratio.in.L.HDL | 129001 | PDI_U | 0.001897394984 | 0.0002841653289 | 6.677081228 | 2.4473e-11 |
| Total.Con.of.LP.particles | 129001 | PDI_U | 0.0019612166 | 0.0002947395076 | 6.654067573 | 2.86235e-11 |
| CE.to.TLs.ratio.in.S.VLDL | 129001 | PDI_U | -0.002090348061 | 0.0003150683128 | -6.634586775 | 3.26692e-11 |
| TLs.in.IDL | 129001 | PDI_U | 0.001996902232 | 0.0003059863699 | 6.526114978 | 6.77462e-11 |
| PL.to.TLs.ratio.in.M.VLDL | 129001 | PDI_U | -0.001944672129 | 0.0002996051593 | -6.490783181 | 8.56993e-11 |
| Free.Chol.to.TLs.ratio.in.L.LDL | 129001 | PDI_U | -0.001854403026 | 0.0002906327749 | -6.3805709 | 1.77024e-10 |
| CE.in.IDL | 129001 | PDI_U | 0.00193938235 | 0.0003051579589 | 6.355339239 | 2.08658e-10 |
| Alanine | 129001 | PDI_U | 0.001990690538 | 0.0003161380532 | 6.296902629 | 3.04625e-10 |
| Con.of.IDL.particles | 129001 | PDI_U | 0.00197449411 | 0.0003140401826 | 6.287393205 | 3.23869e-10 |
| Clinical.LDL_C | 129001 | PDI_U | 0.001986242384 | 0.0003161934284 | 6.281732023 | 3.35884e-10 |
| Chol.to.TLs.ratio.in.chylomicrons.and.extremely.L.VLDL | 129001 | PDI_U | -0.00191725322 | 0.0003139992678 | -6.105916213 | 1.02503e-09 |
| Chol.in.IDL | 129001 | PDI_U | 0.001838166332 | 0.0003048948996 | 6.028852353 | 1.65574e-09 |
| Glycoprotein.acetyls | 129001 | PDI_U | 0.001787339173 | 0.0002971116077 | 6.015716405 | 1.79573e-09 |
| Chol.in.V..S.VLDL | 129001 | PDI_U | 0.001843485775 | 0.0003079906843 | 5.985524463 | 2.16261e-09 |
| PL.to.TLs.ratio.in.M.HDL | 129001 | PDI_U | 0.001644855737 | 0.0002825324345 | 5.821829767 | 5.83441e-09 |
| CE.in.S.HDL | 129001 | PDI_U | 0.001845295101 | 0.0003170620571 | 5.819980852 | 5.8993e-09 |
| Free.Chol.in.M.LDL | 129001 | PDI_U | 0.001846495472 | 0.0003183170312 | 5.8008064 | 6.61503e-09 |
| TG.to.TLs.ratio.in.V..S.VLDL | 129001 | PDI_U | 0.001688848568 | 0.0002930542627 | 5.762921013 | 8.28579e-09 |
| Con.of.HDL.particles | 129001 | PDI_U | 0.001688016088 | 0.0002937211976 | 5.747001243 | 9.10438e-09 |
| Chol.in.M.VLDL | 129001 | PDI_U | 0.001814442839 | 0.0003173816833 | 5.716911007 | 1.08716e-08 |
| CE.to.TLs.ratio.in.L.LDL | 129001 | PDI_U | 0.001835984051 | 0.0003212593184 | 5.714959679 | 1.09971e-08 |
| Omega.3.FAs | 129001 | PDI_U | 0.001762254161 | 0.0003083744115 | 5.714657558 | 1.10166e-08 |
| PL.in.S.LDL | 129001 | PDI_U | 0.001823291301 | 0.0003198591937 | 5.700293558 | 1.19861e-08 |
| Creatinine | 129001 | PDI_U | 0.00149383756 | 0.0002652475597 | 5.631861653 | 1.78644e-08 |
| PL.in.M.HDL | 129001 | PDI_U | 0.001650957515 | 0.0002938670552 | 5.618042191 | 1.9353e-08 |
| TG.to.TLs.ratio.in.chylomicrons.and.extremely.L.VLDL | 129001 | PDI_U | 0.001770100947 | 0.0003185606274 | 5.556559082 | 2.75686e-08 |
| Acetone | 129001 | PDI_U | -0.001768322075 | 0.0003191344312 | -5.540994332 | 3.01344e-08 |
| CE.in.V..S.VLDL | 129001 | PDI_U | 0.001665334372 | 0.0003052641803 | 5.45538743 | 4.89557e-08 |
| PL.in.IDL | 129001 | PDI_U | 0.001652880943 | 0.0003050549427 | 5.418305726 | 6.02738e-08 |
| Sphingomyelins | 129001 | PDI_U | 0.001579830646 | 0.0002940533514 | 5.372598676 | 7.77439e-08 |
| Free.Chol.in.LDL | 129001 | PDI_U | 0.001664948432 | 0.0003144030731 | 5.295585744 | 1.18829e-07 |
| Free.Chol.in.L.LDL | 129001 | PDI_U | 0.001624201688 | 0.000310999878 | 5.222515514 | 1.76782e-07 |
| PL.to.TLs.ratio.in.V..S.VLDL | 129001 | PDI_U | -0.0015759009 | 0.0003072519613 | -5.129018196 | 2.91677e-07 |
| Con.of.L.LDL.particles | 129001 | PDI_U | 0.001617648657 | 0.0003183105211 | 5.081983 | 3.74035e-07 |
| Acetate | 129001 | PDI_U | -0.001605619754 | 0.0003185479076 | -5.040434157 | 4.65103e-07 |
| Ratio.of.docosahexaenoic.acid.to.total.FA | 129001 | PDI_U | -0.001495272031 | 0.0003000148063 | -4.983994123 | 6.23654e-07 |
| TG.to.TLs.ratio.in.L.VLDL | 129001 | PDI_U | 0.001594461638 | 0.0003199901819 | 4.982845501 | 6.27368e-07 |
| Free.Chol.in.IDL | 129001 | PDI_U | 0.001508897097 | 0.0003048498308 | 4.949640593 | 7.44441e-07 |
| ApoA1 | 129001 | PDI_U | 0.001340807474 | 0.0002838696199 | 4.723321481 | 2.32267e-06 |
| TLs.in.M.HDL | 129001 | PDI_U | 0.001359042024 | 0.0002901918235 | 4.683254021 | 2.82643e-06 |
| PL.to.TLs.ratio.in.V..L.HDL | 129001 | PDI_U | -0.001289868222 | 0.0002793930717 | -4.616679341 | 3.90304e-06 |
| Glucose.lactate | 129001 | PDI_U | 0.001302195804 | 0.0002927766435 | 4.447744835 | 8.68489e-06 |
| Glycine | 129001 | PDI_U | -0.001321150273 | 0.0002973372052 | -4.443272654 | 8.86744e-06 |
| PL.to.TLs.ratio.in.L.LDL | 129001 | PDI_U | -0.001403878211 | 0.0003191152142 | -4.399283232 | 1.08696e-05 |
| Free.Chol.to.TLs.ratio.in.L.VLDL | 129001 | PDI_U | -0.001384330279 | 0.0003190268751 | -4.339227779 | 1.43093e-05 |
| Free.Chol.to.TLs.ratio.in.IDL | 129001 | PDI_U | -0.001351354646 | 0.0003139717136 | -4.304064942 | 1.67816e-05 |
| PL.to.TLs.ratio.in.L.VLDL | 129001 | PDI_U | 0.001289867205 | 0.0003018021984 | 4.273882736 | 1.92236e-05 |
| PL.to.TLs.ratio.in.chylomicrons.and.extremely.L.VLDL | 129001 | PDI_U | -0.001351975478 | 0.000319303712 | -4.234136426 | 2.2959e-05 |
| CE.in.V..L.HDL | 129001 | PDI_U | -0.001106484345 | 0.0002636361279 | -4.197013338 | 2.70639e-05 |
| Chol.to.TLs.ratio.in.V..S.VLDL | 129001 | PDI_U | -0.001215063627 | 0.0002914908893 | -4.168444614 | 3.06885e-05 |
| Con.of.M.HDL.particles | 129001 | PDI_U | 0.001189214226 | 0.000286483394 | 4.151075598 | 3.31129e-05 |
| CE.to.TLs.ratio.in.V..S.VLDL | 129001 | PDI_U | -0.001178059761 | 0.000288408155 | -4.08469643 | 4.41611e-05 |
| PL.in.HDL | 129001 | PDI_U | 0.001136155509 | 0.0002802853686 | 4.053566958 | 5.04721e-05 |
| Free.Chol.in.M.HDL | 129001 | PDI_U | 0.001141638254 | 0.0002817848352 | 4.051453845 | 5.09301e-05 |
| CE.in.L.HDL | 129001 | PDI_U | -0.001050036956 | 0.0002595858403 | -4.04504712 | 5.2343e-05 |
| Chol.in.V..L.HDL | 129001 | PDI_U | -0.001076279275 | 0.0002667361941 | -4.034995247 | 5.46348e-05 |
| Free.Chol.in.S.LDL | 129001 | PDI_U | 0.001268351841 | 0.0003189239824 | 3.976972291 | 6.98363e-05 |
| Ratio.of.ApoB.to.ApoA1 | 129001 | PDI_U | 0.001206842875 | 0.0003113651334 | 3.875973078 | 0.0001062519426 |
| X3.Hydroxybutyrate | 129001 | PDI_U | -0.001216920547 | 0.0003184752715 | -3.821083316 | 0.0001329290158 |
| CE.to.TLs.ratio.in.V..L.HDL | 129001 | PDI_U | -0.001210314308 | 0.0003172398157 | -3.815139992 | 0.000136169159 |
| Glutamine | 129001 | PDI_U | -0.001176151022 | 0.0003154596035 | -3.728372853 | 0.00019280244 |
| Chol.to.TLs.ratio.in.M.LDL | 129001 | PDI_U | 0.001135751458 | 0.0003138631041 | 3.618620487 | 0.0002962907263 |
| Chol.in.L.HDL | 129001 | PDI_U | -0.0009282890503 | 0.0002595839925 | -3.576064308 | 0.0003489333392 |
| PL.in.V..L.HDL | 129001 | PDI_U | -0.0009570072609 | 0.0002682873112 | -3.567098483 | 0.000361086515 |
| Chol.to.TLs.ratio.in.S.LDL | 129001 | PDI_U | 0.001127344271 | 0.0003164314964 | 3.56268034 | 0.0003672198373 |
| Free.Chol.to.TLs.ratio.in.V..S.VLDL | 129001 | PDI_U | -0.001119480271 | 0.0003165502438 | -3.536501054 | 0.0004056070108 |
| Ratio.of.omega.6.FA.to.omega.3.FA | 129001 | PDI_U | -0.001069876295 | 0.0003102252655 | -3.448707806 | 0.0005634556439 |
| Free.Chol.in.V..L.HDL | 129001 | PDI_U | -0.0009487057198 | 0.0002820409839 | -3.363715821 | 0.0007692305322 |

| Metabolite | N_used | term | beta | se | t | p_val |
|---|---|---|---|---|---|---|
| TLs.in.V..L.HDL | 129001 | PDI_U | -0.0008741322983 | 0.0002674815249 | -3.26801 | 0.001083348511 |
| Average.diameter.for.HDL.particles | 129001 | PDI_U | -0.0008626433885 | 0.0002646385249 | -3.259704492 | 0.001115572306 |
| Isoleucine | 129001 | PDI_U | 0.000988957504 | 0.0003077728115 | 3.213271176 | 0.001312645525 |
| TLs.in.HDL | 129001 | PDI_U | 0.0008787888822 | 0.0002761389785 | 3.182415199 | 0.001460868968 |
| CE.in.M.VLDL | 129001 | PDI_U | 0.0009665862917 | 0.0003136881494 | 3.08136056 | 0.002061003451 |
| Phenylalanine | 129001 | PDI_U | -0.0009636634248 | 0.0003173077564 | -3.036999271 | 0.002389937387 |
| TG.to.TLs.ratio.in.IDL | 129001 | PDI_U | 0.0008717843744 | 0.0002979653717 | 2.925790905 | 0.003436416522 |
| Glucose | 129001 | PDI_U | 0.0008504351606 | 0.0002945044365 | 2.887682002 | 0.003881564942 |
| CE.to.TLs.ratio.in.IDL | 129001 | PDI_U | 0.0008648352338 | 0.0003086800832 | 2.80172023 | 0.005083850476 |
| Free.Chol.in.HDL | 129001 | PDI_U | 0.0007320825588 | 0.0002700582633 | 2.710831914 | 0.006712351738 |
| Tyrosine | 129001 | PDI_U | 0.0008460513492 | 0.0003137668683 | 2.696433036 | 0.007009558395 |
| CE.to.TLs.ratio.in.chylomicrons.and.extremely.L.VLDL | 129001 | PDI_U | -0.0008558275886 | 0.0003180290968 | -2.691035497 | 0.00712397937 |
| PL.to.TLs.ratio.in.S.HDL | 129001 | PDI_U | 0.000817384935 | 0.000310034195 | 2.636434781 | 0.008379233686 |
| Docosahexaenoic.acid | 129001 | PDI_U | 0.000790636557 | 0.0003002704266 | 2.633081672 | 0.00846240843 |
| Total.Con.of.branched.chain.AAs | 129001 | PDI_U | 0.0007837443247 | 0.0003005889347 | 2.607362528 | 0.009125320298 |
| TG.to.TLs.ratio.in.M.LDL | 129001 | PDI_U | 0.000749445868 | 0.000309339477 | 2.422729472 | 0.01540576111 |
| Valine | 129001 | PDI_U | 0.0007209775881 | 0.0003002546002 | 2.40122079 | 0.01634188007 |
| Free.Chol.to.TLs.ratio.in.L.HDL | 129001 | PDI_U | -0.0006870952031 | 0.0002892789632 | -2.375199342 | 0.0175409279 |
| Citrate | 129001 | PDI_U | 0.0007249186695 | 0.0003128687869 | 2.317005402 | 0.02050499539 |
| Lactate | 129001 | PDI_U | 0.0006777770728 | 0.0003192322628 | 2.123147162 | 0.03374342066 |
| TG.to.TLs.ratio.in.L.LDL | 129001 | PDI_U | 0.0006368374993 | 0.0003027256831 | 2.103678462 | 0.03540843543 |
| Acetoacetate | 129001 | PDI_U | -0.0006491419494 | 0.0003171861937 | -2.046564328 | 0.04070290281 |
| Leucine | 129001 | PDI_U | 0.0006039103112 | 0.0003008452472 | 2.0073786 | 0.04471144767 |
| Free.Chol.in.L.HDL | 129001 | PDI_U | -0.0005120099435 | 0.000261177456 | -1.960391036 | 0.04995225197 |
| PL.to.TLs.ratio.in.V..L.VLDL | 129001 | PDI_U | -0.0006046663473 | 0.0003205003001 | -1.886632702 | 0.05921198899 |
| Free.Chol.to.TLs.ratio.in.V..L.HDL | 129001 | PDI_U | 0.0005007654251 | 0.0002659238975 | 1.883115545 | 0.0596869591 |
| Chol.in.M.HDL | 129001 | PDI_U | 0.0005284310614 | 0.0002842760064 | 1.858866206 | 0.06304838108 |
| TLs.in.L.HDL | 129001 | PDI_U | -0.0004836187423 | 0.0002614989597 | -1.849409813 | 0.06440094562 |
| Con.of.L.HDL.particles | 129001 | PDI_U | -0.000465276189 | 0.0002594497295 | -1.79331923 | 0.07292423609 |
| Histidine | 129001 | PDI_U | -0.0004351896407 | 0.0003189528904 | -1.364432347 | 0.1724339136 |
| Chol.to.TLs.ratio.in.V..L.HDL | 129001 | PDI_U | -0.0003979880554 | 0.0003025892651 | -1.315274867 | 0.1884198796 |
| CE.in.M.HDL | 129001 | PDI_U | 0.000370081664 | 0.0002853145802 | 1.297100428 | 0.1945989545 |
| Free.Chol.to.TLs.ratio.in.M.HDL | 129001 | PDI_U | 0.0003458858148 | 0.0002691852988 | 1.284935753 | 0.198817043 |
| PL.in.L.HDL | 129001 | PDI_U | -0.0003200610023 | 0.0002645699322 | -1.209740652 | 0.2263806425 |
| Con.of.V..L.HDL.particles | 129001 | PDI_U | -0.00030167189 | 0.0002688782121 | -1.121964802 | 0.2618794945 |
| Free.Chol.to.TLs.ratio.in.S.HDL | 129001 | PDI_U | 0.0002985014767 | 0.0002955178978 | 1.010096102 | 0.3124511442 |
| Albumin | 129001 | PDI_U | 0.0003114219639 | 0.0003144787891 | 0.9902797094 | 0.322039279 |
| CE.in.HDL | 129001 | PDI_U | -0.0001803838693 | 0.0002707815056 | -0.6661602272 | 0.5053098832 |
| Chol.to.TLs.ratio.in.IDL | 129001 | PDI_U | 0.0001797173585 | 0.0003058659381 | 0.5875690496 | 0.5568226191 |
| Pyruvate | 129001 | PDI_U | 0.0001873111324 | 0.0003198570743 | 0.5856088468 | 0.5581394264 |
| Ratio.of.omega.3.FA.to.total.FA | 129001 | PDI_U | 7.33641e-05 | 0.0003079740773 | 0.2382153121 | 0.81171448 |
| HDL_C | 129001 | PDI_U | 2.67702e-05 | 0.0002697887769 | 0.09922642606 | 0.9209586476 |
| Chol.to.TLs.ratio.in.L.LDL | 129001 | PDI_U | -3.87776e-06 | 0.0002999036424 | -0.01293003459 | 0.9896836325 |

## Supplementary Table 7. Metabolome-wide associations between the healthy plant-based diet index (PDI-H) and circulating metabolites in the UK Biobank

| Metabolite | N_used | term | beta | se | t | p_val |
|---|---|---|---|---|---|---|
| Ratio.of.omega.3.FA.to.total.FA | 129001 | PDI_H | 0.006163163241 | 0.0002872859242 | 21.45306373 | 6.45092e-102 |
| Docosahexaenoic.acid | 129001 | PDI_H | 0.005264914421 | 0.0002802233231 | 18.78828058 | 1.2002e-78 |
| Omega.3.FAs | 129001 | PDI_H | 0.005343721946 | 0.000287824193 | 18.56592349 | 7.64076e-77 |
| Ratio.of.omega.6.FA.to.omega.3.FA | 129001 | PDI_H | -0.005251353221 | 0.0002895460479 | -18.13650457 | 2.02627e-73 |
| Ratio.of.docosahexaenoic.acid.to.total.FA | 129001 | PDI_H | 0.005076181271 | 0.0002800306197 | 18.1272365 | 2.39725e-73 |
| Degree.of.unsaturation | 129001 | PDI_H | 0.004722467649 | 0.0002732385679 | 17.28331284 | 7.4762e-67 |
| Ratio.of.polyunsaturated.FA.to.total.FA | 129001 | PDI_H | 0.004063417759 | 0.0002691935815 | 15.09477951 | 1.93975e-51 |
| Ratio.of.saturated.FA.to.total.FA | 129001 | PDI_H | -0.004082860614 | 0.0002910756335 | -14.02680315 | 1.15266e-44 |
| Ratio.of.linoleic.acid.to.total.FA | 129001 | PDI_H | 0.003797263544 | 0.0002729762322 | 13.91060135 | 5.87691e-44 |
| Ratio.of.polyunsaturated.FA.to.monounsaturated.FA | 129001 | PDI_H | 0.003235354739 | 0.0002646337503 | 12.22578275 | 2.36537e-34 |
| Polyunsaturated.FA | 129001 | PDI_H | 0.00295942216 | 0.0002906860084 | 10.1808208 | 2.46693e-24 |
| Linoleic.acid | 129001 | PDI_H | 0.0029213411 | 0.0002947982849 | 9.909627192 | 3.85329e-23 |
| Ratio.of.monounsaturated.FA.to.total.FA | 129001 | PDI_H | -0.002451066987 | 0.0002649746149 | -9.250195489 | 2.27358e-20 |
| PL.in.V..L.HDL | 129001 | PDI_H | 0.002273846771 | 0.0002506435536 | 9.072033723 | 1.18397e-19 |
| Con.of.V..L.HDL.particles | 129001 | PDI_H | 0.00224184374 | 0.0002511870061 | 8.924998848 | 4.51355e-19 |
| TLs.in.V..L.HDL | 129001 | PDI_H | 0.002228648124 | 0.0002498914539 | 8.918464756 | 4.78774e-19 |
| Valine | 129001 | PDI_H | 0.002467083458 | 0.0002805063438 | 8.795107534 | 1.4462e-18 |
| Albumin | 129001 | PDI_H | 0.002570413047 | 0.0002937904521 | 8.74913745 | 2.17508e-18 |
| Tyrosine | 129001 | PDI_H | 0.00248594775 | 0.0002931377695 | 8.480475764 | 2.26596e-17 |
| Free.Chol.to.TLs.ratio.in.V..L.HDL | 129001 | PDI_H | -0.002099049468 | 0.0002484372363 | -8.449013115 | 2.96772e-17 |
| Average.diameter.for.HDL.particles | 129001 | PDI_H | 0.002085994459 | 0.0002472433661 | 8.437008814 | 3.28864e-17 |
| CE.in.V..L.HDL | 129001 | PDI_H | 0.002011755691 | 0.0002463178042 | 8.167317414 | 3.18134e-16 |
| Chol.in.V..L.HDL | 129001 | PDI_H | 0.002032641433 | 0.0002492131126 | 8.156237894 | 3.48687e-16 |
| Free.Chol.to.TLs.ratio.in.S.HDL | 129001 | PDI_H | 0.002214123132 | 0.0002760900519 | 8.019568677 | 1.06997e-15 |
| CE.to.TLs.ratio.in.L.LDL | 129001 | PDI_H | 0.002366870821 | 0.0003001784883 | 7.884878206 | 3.17285e-15 |
| PL.to.TLs.ratio.in.V..L.HDL | 129001 | PDI_H | 0.002042804432 | 0.0002610489435 | 7.825369468 | 5.09979e-15 |
| Free.Chol.in.V..L.HDL | 129001 | PDI_H | 0.002003784967 | 0.0002635162759 | 7.604027344 | 2.88984e-14 |
| X3.Hydroxybutyrate | 129001 | PDI_H | -0.002208948773 | 0.0002975644452 | -7.423429811 | 1.14823e-13 |
| Chol.to.TLs.ratio.in.V..L.HDL | 129001 | PDI_H | -0.0020971646 | 0.000282707483 | -7.418143225 | 1.19498e-13 |
| CE.in.S.HDL | 129001 | PDI_H | -0.002173749966 | 0.0002962676105 | -7.337116474 | 2.19519e-13 |
| TG.in.V..L.HDL | 129001 | PDI_H | 0.002114671976 | 0.0002956109128 | 7.153565327 | 8.49994e-13 |
| Con.of.L.HDL.particles | 129001 | PDI_H | 0.001702861817 | 0.0002424091857 | 7.024741294 | 2.15518e-12 |
| Free.Chol.in.L.HDL | 129001 | PDI_H | 0.001701140382 | 0.0002440247379 | 6.97118004 | 3.15794e-12 |
| Total.Con.of.branched.chain.AAs | 129001 | PDI_H | 0.001957295193 | 0.0002808511323 | 6.969155427 | 3.2037e-12 |
| Free.Chol.to.TLs.ratio.in.L.HDL | 129001 | PDI_H | 0.001851385038 | 0.0002702843321 | 6.849768256 | 7.42993e-12 |
| TLs.in.L.HDL | 129001 | PDI_H | 0.001650363854 | 0.0002443275394 | 6.754718925 | 1.43716e-11 |
| Free.Chol.to.TLs.ratio.in.M.HDL | 129001 | PDI_H | 0.001688243202 | 0.0002515079819 | 6.712483593 | 1.92127e-11 |
| Chol.to.TLs.ratio.in.L.LDL | 129001 | PDI_H | 0.001880154 | 0.0002802073061 | 6.709867871 | 1.95601e-11 |
| Omega.6.FA | 129001 | PDI_H | 0.00194800328 | 0.0002924568257 | 6.660823441 | 2.73385e-11 |
| Chol.in.L.HDL | 129001 | PDI_H | 0.001587441048 | 0.0002425497499 | 6.544805958 | 5.97947e-11 |
| TG.in.L.HDL | 129001 | PDI_H | 0.001874800022 | 0.0002884833563 | 6.498815204 | 8.12485e-11 |
| PL.to.TLs.ratio.in.L.HDL | 129001 | PDI_H | -0.001703826607 | 0.0002655524618 | -6.416158206 | 1.40238e-10 |
| PL.in.L.HDL | 129001 | PDI_H | 0.00157981185 | 0.0002471995624 | 6.390835951 | 1.65541e-10 |
| CE.in.L.HDL | 129001 | PDI_H | 0.001547890793 | 0.00024255682 | 6.381559557 | 1.75885e-10 |
| Chol.in.S.HDL | 129001 | PDI_H | -0.001859868272 | 0.0002950586957 | -6.303384038 | 2.92153e-10 |
| Free.Chol.to.TLs.ratio.in.L.VLDL | 129001 | PDI_H | -0.001844902789 | 0.0002981041285 | -6.188786444 | 6.08107e-10 |
| Histidine | 129001 | PDI_H | 0.001832380994 | 0.000298015969 | 6.148600024 | 7.8398e-10 |
| PL.to.TLs.ratio.in.L.VLDL | 129001 | PDI_H | -0.001729946341 | 0.0002820092159 | -6.13436102 | 8.57495e-10 |
| Creatinine | 129001 | PDI_H | -0.001518842504 | 0.0002478649267 | -6.127702389 | 8.94139e-10 |
| Con.of.S.HDL.particles | 129001 | PDI_H | -0.00179793144 | 0.0002948712461 | -6.097344057 | 1.0815e-09 |
| Ratio.of.omega.6.FA.to.total.FA | 129001 | PDI_H | 0.001661009117 | 0.0002729536523 | 6.085315595 | 1.16588e-09 |
| Acetate | 129001 | PDI_H | 0.001804514718 | 0.0002976659493 | 6.062214112 | 1.34631e-09 |
| Glucose | 129001 | PDI_H | 0.00163494302 | 0.0002751819579 | 5.941316183 | 2.83465e-09 |
| CE.to.TLs.ratio.in.chylomicrons.and.extremely.L.VLDL | 129001 | PDI_H | -0.001744570908 | 0.0002971628581 | -5.870756927 | 4.34867e-09 |
| Isoleucine | 129001 | PDI_H | 0.001673886682 | 0.0002875835905 | 5.820522232 | 5.88023e-09 |
| Spectrometer.corrected.alanine | 129001 | PDI_H | 0.001659150434 | 0.0002930924315 | 5.660843664 | 1.50949e-08 |
| TLs.in.S.HDL | 129001 | PDI_H | -0.001612149567 | 0.0002924353816 | -5.512840335 | 3.53762e-08 |
| CE.to.TLs.ratio.in.S.VLDL | 129001 | PDI_H | -0.001563521207 | 0.0002944454502 | -5.310053886 | 1.09774e-07 |
| CE.to.TLs.ratio.in.S.HDL | 129001 | PDI_H | -0.001501276442 | 0.0002938492607 | -5.109001937 | 3.24325e-07 |
| PL.to.TLs.ratio.in.L.LDL | 129001 | PDI_H | -0.001472346521 | 0.0002982033705 | -4.93739061 | 7.92734e-07 |
| PL.in.S.HDL | 129001 | PDI_H | -0.00142279706 | 0.0002911576579 | -4.886689465 | 1.02668e-06 |
| TG.in.S.VLDL | 129001 | PDI_H | 0.001325193287 | 0.0002829502964 | 4.683484357 | 2.82326e-06 |
| Acetone | 129001 | PDI_H | -0.001367317911 | 0.0002982383275 | -4.584648533 | 4.55177e-06 |
| TG.to.TLs.ratio.in.chylomicrons.and.extremely.L.VLDL | 129001 | PDI_H | 0.001358283328 | 0.0002977025274 | 4.562552225 | 5.0582e-06 |
| Chol.to.TLs.ratio.in.L.HDL | 129001 | PDI_H | 0.001180764564 | 0.0002595347912 | 4.549542504 | 5.38116e-06 |
| Chol.to.TLs.ratio.in.chylomicrons.and.extremely.L.VLDL | 129001 | PDI_H | -0.001301251549 | 0.0002934484253 | -4.434344972 | 9.24288e-06 |
| CE.to.TLs.ratio.in.V..L.HDL | 129001 | PDI_H | -0.001287251708 | 0.0002964517006 | -4.342197078 | 1.41172e-05 |
| Free.Chol.in.HDL | 129001 | PDI_H | 0.001088423487 | 0.0002523550622 | 4.313063815 | 1.61127e-05 |

| Metabolite | N_used | term | beta | se | t | p_val |
|---|---|---|---|---|---|---|
| TLs.in.M.VLDL | 129001 | PDI_H | 0.001234370117 | 0.0002931001591 | 4.211427659 | 2.53934e-05 |
| Lactate | 129001 | PDI_H | -0.00123930773 | 0.0002983038128 | -4.154515217 | 3.26188e-05 |
| PC | 129001 | PDI_H | 0.001135387338 | 0.0002767269023 | 4.102916371 | 4.08225e-05 |
| Free.Chol.in.M.VLDL | 129001 | PDI_H | 0.001214678931 | 0.000297380088 | 4.084600753 | 4.41793e-05 |
| Alanine | 129001 | PDI_H | 0.001139994538 | 0.0002954554109 | 3.858431749 | 0.0001141723992 |
| Chol.in.M.VLDL | 129001 | PDI_H | 0.001138991169 | 0.0002966098329 | 3.840031727 | 0.0001230771441 |
| TG.to.TLs.ratio.in.L.LDL | 129001 | PDI_H | -0.001080443226 | 0.0002828822223 | -3.819410133 | 0.0001338337673 |
| Con.of.S.VLDL.particles | 129001 | PDI_H | 0.001100054392 | 0.0002899028106 | 3.794562701 | 0.0001479714179 |
| Chol.to.TLs.ratio.in.S.VLDL | 129001 | PDI_H | -0.001089085785 | 0.0002871918088 | -3.79218958 | 0.0001493928507 |
| Leucine | 129001 | PDI_H | 0.001062231213 | 0.000281124955 | 3.778502029 | 0.0001578454478 |
| TG.in.M.VLDL | 129001 | PDI_H | 0.001056385685 | 0.000282577514 | 3.738392592 | 0.0001852805129 |
| PL.in.M.VLDL | 129001 | PDI_H | 0.001098779526 | 0.0002963093499 | 3.70821753 | 0.0002088106987 |
| TLs.in.S.VLDL | 129001 | PDI_H | 0.001073680511 | 0.0002908781687 | 3.691169109 | 0.0002233175039 |
| Con.of.M.VLDL.particles | 129001 | PDI_H | 0.001066491143 | 0.0002954280343 | 3.609986253 | 0.0003063291497 |
| Chol.to.TLs.ratio.in.M.LDL | 129001 | PDI_H | 0.001053258967 | 0.0002933013718 | 3.591046848 | 0.0003294746677 |
| Free.Chol.to.TLs.ratio.in.V..L.VLDL | 129001 | PDI_H | 0.0009755155214 | 0.0002808222831 | 3.47378246 | 0.0005133451778 |
| TG.in.L.VLDL | 129001 | PDI_H | 0.0009580523777 | 0.0002773333401 | 3.454515701 | 0.0005514591729 |
| CE.in.V..S.VLDL | 129001 | PDI_H | 0.0009804468783 | 0.0002852854054 | 3.436722874 | 0.0005889832138 |
| CE.in.M.VLDL | 129001 | PDI_H | 0.0009961585728 | 0.0002931353196 | 3.398289138 | 0.0006782920181 |
| Con.of.VLDL.particles | 129001 | PDI_H | 0.0009939818366 | 0.000293276287 | 3.389233568 | 0.0007010925167 |
| PL.in.S.VLDL | 129001 | PDI_H | 0.0009950541942 | 0.0002956233465 | 3.365952675 | 0.0007630203854 |
| Total.free.Chol | 129001 | PDI_H | 0.0009570172135 | 0.000289975116 | 3.300342549 | 0.0009659306193 |
| CE.in.L.VLDL | 129001 | PDI_H | 0.0009462142175 | 0.0002887645956 | 3.276766723 | 0.001050308058 |
| Chol.in.V..S.VLDL | 129001 | PDI_H | 0.0009355782459 | 0.0002878416228 | 3.250322997 | 0.001153035375 |
| Glucose.lactate | 129001 | PDI_H | 0.0008702615718 | 0.0002736063618 | 3.18070664 | 0.001469510741 |
| TLs.in.VLDL | 129001 | PDI_H | 0.0008988111705 | 0.0002849459985 | 3.154321082 | 0.00160908893 |
| TLs.in.V..S.VLDL | 129001 | PDI_H | 0.0009159635105 | 0.0002927523624 | 3.128799724 | 0.001755609815 |
| Con.of.S.LDL.particles | 129001 | PDI_H | 0.0009218292415 | 0.0002979231445 | 3.09418472 | 0.001973967551 |
| TG.to.TLs.ratio.in.S.VLDL | 129001 | PDI_H | 0.0008747100667 | 0.0002840939598 | 3.078946372 | 0.002077776813 |
| Free.Chol.in.VLDL | 129001 | PDI_H | 0.0008963864109 | 0.0002913209354 | 3.07697217 | 0.002091586191 |
| TG.in.VLDL | 129001 | PDI_H | 0.0008520963042 | 0.00027703411 | 3.075781189 | 0.002099957669 |
| Glutamine | 129001 | PDI_H | 0.0008988089149 | 0.0002947983163 | 3.04889433 | 0.002297317724 |
| Total.cholines | 129001 | PDI_H | 0.0008416155721 | 0.0002770337961 | 3.037952712 | 0.002382389377 |
| TLs.in.HDL | 129001 | PDI_H | 0.0007809511032 | 0.0002580493913 | 3.026362896 | 0.002475638284 |
| Pyruvate | 129001 | PDI_H | -0.0008977188859 | 0.000298892382 | -3.003485335 | 0.002669575821 |
| Free.Chol.in.S.VLDL | 129001 | PDI_H | 0.0008917090496 | 0.0002974776097 | 2.997566944 | 0.002721958805 |
| CE.to.TLs.ratio.in.L.HDL | 129001 | PDI_H | 0.0007892313715 | 0.000264309278 | 2.986014633 | 0.002826921634 |
| PL.to.TLs.ratio.in.M.LDL | 129001 | PDI_H | -0.0008832653326 | 0.0002967876714 | -2.976084985 | 0.002920080753 |
| VLDL_C | 129001 | PDI_H | 0.0008770663254 | 0.0002952457339 | 2.970631663 | 0.002972427199 |
| HDL_C | 129001 | PDI_H | 0.0007382882174 | 0.0002521058672 | 2.928484869 | 0.003406779655 |
| PL.to.TLs.ratio.in.S.HDL | 129001 | PDI_H | 0.0008480433278 | 0.0002897212621 | 2.927100764 | 0.003421977292 |
| PG | 129001 | PDI_H | 0.0008160120497 | 0.0002796414898 | 2.91806502 | 0.003522716876 |
| PL.to.TLs.ratio.in.V..S.VLDL | 129001 | PDI_H | -0.0008374023255 | 0.0002871429266 | -2.916325801 | 0.003542414379 |
| TLs.in.LP.particles | 129001 | PDI_H | 0.0008504919962 | 0.0002927767653 | 2.904916294 | 0.003674136992 |
| PL.in.VLDL | 129001 | PDI_H | 0.0008176032189 | 0.0002887577377 | 2.831450424 | 0.004634458637 |
| Con.of.V..S.VLDL.particles | 129001 | PDI_H | 0.0008297264528 | 0.0002936255732 | 2.825797643 | 0.004717029347 |
| CE.in.VLDL | 129001 | PDI_H | 0.000836988063 | 0.0002967946787 | 2.820091205 | 0.004801732357 |
| Glycoprotein.acetyls | 129001 | PDI_H | -0.0007811859716 | 0.0002776775084 | -2.813285009 | 0.004904557928 |
| PL.in.IDL | 129001 | PDI_H | 0.0008016728817 | 0.0002850937304 | 2.81196251 | 0.004924767363 |
| Remnant_C | 129001 | PDI_H | 0.0008225114757 | 0.0002953509289 | 2.784861652 | 0.005355837064 |
| PL.in.V..S.VLDL | 129001 | PDI_H | 0.0008054521683 | 0.0002935419776 | 2.743907958 | 0.006072094683 |
| TLs.in.L.VLDL | 129001 | PDI_H | 0.0007616667948 | 0.0002779416641 | 2.74038366 | 0.006137594373 |
| TG.to.TLs.ratio.in.L.VLDL | 129001 | PDI_H | 0.0008173037254 | 0.000299046943 | 2.73302819 | 0.00627635091 |
| Free.Chol.in.V..S.VLDL | 129001 | PDI_H | 0.0007981351032 | 0.0002924465032 | 2.729166171 | 0.006350330497 |
| Free.Chol.to.TLs.ratio.in.V..S.VLDL | 129001 | PDI_H | -0.0008013036941 | 0.0002958181714 | -2.708771034 | 0.006754183666 |
| PL.in.HDL | 129001 | PDI_H | 0.0007039838903 | 0.00026193252 | 2.687653638 | 0.007196522166 |
| Total.PL.in.LP.particles | 129001 | PDI_H | 0.0007459789758 | 0.0002814099621 | 2.650862003 | 0.008029644093 |
| TG.in.chylomicrons.and.extremely.L.VLDL | 129001 | PDI_H | 0.0007268004931 | 0.0002778220115 | 2.61606519 | 0.008895993442 |
| Chol.to.TLs.ratio.in.S.HDL | 129001 | PDI_H | -0.0007499629608 | 0.0002913838153 | -2.573797587 | 0.01006001588 |
| Chol.in.L.VLDL | 129001 | PDI_H | 0.0007202336458 | 0.0002831294606 | 2.543831519 | 0.0109655518 |
| Chol.to.TLs.ratio.in.S.LDL | 129001 | PDI_H | -0.0007516208649 | 0.0002957084193 | -2.541763493 | 0.01103063743 |
| Con.of.LDL.particles | 129001 | PDI_H | 0.0007546440799 | 0.0002979689376 | 2.532626676 | 0.01132232082 |
| ApoB | 129001 | PDI_H | 0.0007439709837 | 0.0002975099507 | 2.500659161 | 0.01239747211 |
| TC | 129001 | PDI_H | 0.0007112191605 | 0.0002861030473 | 2.485884604 | 0.01292424235 |
| Free.Chol.in.S.HDL | 129001 | PDI_H | -0.0007198453277 | 0.0002901771164 | -2.480710184 | 0.01311335861 |
| TG | 129001 | PDI_H | 0.0006928796827 | 0.0002794425339 | 2.47950687 | 0.01315768682 |
| CE.in.HDL | 129001 | PDI_H | 0.0006215678125 | 0.0002530364476 | 2.456335894 | 0.01403359141 |
| Con.of.L.LDL.particles | 129001 | PDI_H | 0.00072502666 | 0.0002974801293 | 2.437227191 | 0.01480171917 |
| Total.FA | 129001 | PDI_H | 0.0006907865002 | 0.0002920389331 | 2.365391808 | 0.01801247556 |
| TG.in.S.LDL | 129001 | PDI_H | 0.0006713408248 | 0.0002841857643 | 2.362330944 | 0.01816189836 |

| Metabolite | N_used | term | beta | se | t | p_val |
|---|---|---|---|---|---|---|
| PL.to.TLs.ratio.in.IDL | 129001 | PDI_H | 0.0006956296544 | 0.0002993818834 | 2.323552937 | 0.02015101342 |
| TG.to.TLs.ratio.in.IDL | 129001 | PDI_H | -0.0006448752853 | 0.0002784483637 | -2.315960046 | 0.02056201007 |
| Con.of.L.VLDL.particles | 129001 | PDI_H | 0.0006302152712 | 0.0002776484752 | 2.269831558 | 0.0232194478 |
| TG.in.L.LDL | 129001 | PDI_H | -0.0006477512883 | 0.0002890912377 | -2.240646564 | 0.02505067291 |
| TLs.in.IDL | 129001 | PDI_H | 0.0006350657998 | 0.0002859821724 | 2.220648212 | 0.02637652805 |
| Con.of.M.LDL.particles | 129001 | PDI_H | 0.0006586525617 | 0.0002973145821 | 2.215338908 | 0.02673855092 |
| CE.in.IDL | 129001 | PDI_H | 0.0006286897266 | 0.0002852055682 | 2.204338893 | 0.02750227878 |
| PL.in.S.LDL | 129001 | PDI_H | 0.0006512202802 | 0.0002989365633 | 2.178456436 | 0.02937387652 |
| Chol.in.IDL | 129001 | PDI_H | 0.0006189082588 | 0.0002849554005 | 2.171947813 | 0.02986141506 |
| Total.esterified.Chol | 129001 | PDI_H | 0.0006087745394 | 0.0002845098016 | 2.139731341 | 0.0323783554 |
| Free.Chol.in.V..L.VLDL | 129001 | PDI_H | 0.0005875608205 | 0.0002779026794 | 2.114268282 | 0.0344942732 |
| Chol.in.S.VLDL | 129001 | PDI_H | 0.0006171649557 | 0.0002962337709 | 2.083371366 | 0.03721935033 |
| TG.in.V..S.VLDL | 129001 | PDI_H | 0.0005993164753 | 0.0002878908409 | 2.081749018 | 0.03736736741 |
| Free.Chol.in.IDL | 129001 | PDI_H | 0.0005818393862 | 0.0002849008009 | 2.042252547 | 0.04112851571 |
| TC.minus.HDL.C | 129001 | PDI_H | 0.0005930125458 | 0.0002959159341 | 2.003989909 | 0.04507322842 |
| CE.to.TLs.ratio.in.M.LDL | 129001 | PDI_H | 0.0005771558901 | 0.0002896728795 | 1.9924402 | 0.04632488412 |
| CE.to.TLs.ratio.in.S.LDL | 129001 | PDI_H | -0.0005593858926 | 0.0002933109045 | -1.907143185 | 0.05650426736 |
| CE.to.TLs.ratio.in.V..S.VLDL | 129001 | PDI_H | 0.0005120047699 | 0.0002695274744 | 1.89963851 | 0.05748280307 |
| TG.to.TLs.ratio.in.V..L.HDL | 129001 | PDI_H | -0.000486901721 | 0.0002657616561 | -1.832099213 | 0.06693894492 |
| PL.to.TLs.ratio.in.S.LDL | 129001 | PDI_H | 0.0005261108363 | 0.0002928563678 | 1.796480781 | 0.07242042547 |
| TG.in.HDL | 129001 | PDI_H | 0.0005196254652 | 0.000291037493 | 1.785424482 | 0.07419483926 |
| Acetoacetate | 129001 | PDI_H | -0.0005221749814 | 0.000296407778 | -1.761677729 | 0.07812613127 |
| CE.in.L.LDL | 129001 | PDI_H | 0.0005177921652 | 0.0002951229912 | 1.754496196 | 0.07934789543 |
| CE.to.TLs.ratio.in.M.HDL | 129001 | PDI_H | -0.0004829509726 | 0.0002780411105 | -1.736976851 | 0.08239364411 |
| Free.Chol.in.L.VLDL | 129001 | PDI_H | 0.000475504705 | 0.0002772709586 | 1.71494594 | 0.08635754062 |
| Monounsaturated.FA | 129001 | PDI_H | -0.0004656116993 | 0.0002859827373 | -1.628111206 | 0.1035037501 |
| TG.in.S.HDL | 129001 | PDI_H | -0.000447235533 | 0.0002756749092 | -1.622329483 | 0.1047352629 |
| TLs.in.S.LDL | 129001 | PDI_H | 0.000482395909 | 0.0002981849573 | 1.617774127 | 0.1057137293 |
| Chol.in.V..L.VLDL | 129001 | PDI_H | 0.0004534989291 | 0.0002813409875 | 1.611919163 | 0.1069819781 |
| Free.Chol.in.M.HDL | 129001 | PDI_H | 0.0004195478547 | 0.0002633385676 | 1.593188034 | 0.1111204651 |
| PL.in.V..L.VLDL | 129001 | PDI_H | 0.0004380359191 | 0.0002755632667 | 1.589601997 | 0.1119269974 |
| Con.of.chylomicrons.and.extremely.L.VLDL.particles | 129001 | PDI_H | 0.0004354159996 | 0.0002768955194 | 1.572492038 | 0.1158389369 |
| Chol.in.L.LDL | 129001 | PDI_H | 0.0004614508 | 0.0002940742848 | 1.569164065 | 0.116612168 |
| Con.of.IDL.particles | 129001 | PDI_H | 0.0004581064776 | 0.0002935088179 | 1.560792895 | 0.1185750776 |
| Ratio.of.ApoB.to.ApoA1 | 129001 | PDI_H | 0.0004519341193 | 0.0002909810464 | 1.553139371 | 0.1203922955 |
| TLs.in.V..L.VLDL | 129001 | PDI_H | 0.0004200867196 | 0.0002738832782 | 1.533816604 | 0.1250772296 |
| CE.in.S.VLDL | 129001 | PDI_H | 0.0004439555229 | 0.0002950587424 | 1.504634363 | 0.1324205552 |
| Free.Chol.in.chylomicrons.and.extremely.L.VLDL | 129001 | PDI_H | 0.0004080869973 | 0.0002764699306 | 1.476062863 | 0.1399294583 |
| TLs.in.chylomicrons.and.extremely.L.VLDL | 129001 | PDI_H | 0.0003973053575 | 0.0002756162815 | 1.441516283 | 0.1494413083 |
| Saturated.FA | 129001 | PDI_H | -0.0004162048832 | 0.0002900804643 | -1.434791151 | 0.1513490448 |
| PL.to.TLs.ratio.in.V..L.VLDL | 129001 | PDI_H | 0.0004238205668 | 0.0002995053301 | 1.415068529 | 0.1570508185 |
| TG.in.V..L.VLDL | 129001 | PDI_H | 0.0003837186276 | 0.0002715438862 | 1.41309986 | 0.1576287773 |
| Chol.to.TLs.ratio.in.V..S.VLDL | 129001 | PDI_H | 0.0003825521881 | 0.0002724108548 | 1.404320648 | 0.1602257962 |
| Average.diameter.for.VLDL.particles | 129001 | PDI_H | 0.0003727998466 | 0.0002661693611 | 1.400611419 | 0.1613327098 |
| PL.in.L.VLDL | 129001 | PDI_H | 0.0003835836356 | 0.000276189677 | 1.38884132 | 0.1648834052 |
| TG.in.LDL | 129001 | PDI_H | -0.0003927521499 | 0.0002880308655 | -1.363576606 | 0.1727032388 |
| Phenylalanine | 129001 | PDI_H | 0.0004024764526 | 0.000296528614 | 1.357293811 | 0.1746902478 |
| CE.in.LDL | 129001 | PDI_H | 0.000402338977 | 0.0002971471911 | 1.354005654 | 0.1757369459 |
| PL.to.TLs.ratio.in.M.HDL | 129001 | PDI_H | -0.0003553876932 | 0.0002640558585 | -1.345880736 | 0.1783433477 |
| Ratio.of.TG.to.PG | 129001 | PDI_H | 0.0003416886264 | 0.0002619648472 | 1.304330066 | 0.1921233957 |
| TG.to.TLs.ratio.in.M.LDL | 129001 | PDI_H | -0.000370390957 | 0.0002890786104 | -1.28128109 | 0.2000972558 |
| Free.Chol.to.TLs.ratio.in.M.LDL | 129001 | PDI_H | 0.0003459914903 | 0.0002733151613 | 1.265906687 | 0.2055487783 |
| PL.in.chylomicrons.and.extremely.L.VLDL | 129001 | PDI_H | 0.0003453183149 | 0.0002741603786 | 1.259548578 | 0.2078345347 |
| LDL_C | 129001 | PDI_H | 0.0003678146368 | 0.0002965900021 | 1.240145097 | 0.2149239874 |
| TG.to.TLs.ratio.in.S.LDL | 129001 | PDI_H | 0.0003487596556 | 0.0002827467644 | 1.233470015 | 0.2174027049 |
| Chol.in.S.LDL | 129001 | PDI_H | 0.0003582343084 | 0.000298176492 | 1.201417006 | 0.2295916884 |
| Con.of.V..L.VLDL.particles | 129001 | PDI_H | 0.0003299610473 | 0.0002746915286 | 1.201205763 | 0.2296735999 |
| PL.to.TLs.ratio.in.S.VLDL | 129001 | PDI_H | -0.0003308722506 | 0.0002776287707 | -1.191779403 | 0.2333499468 |
| Free.Chol.in.S.LDL | 129001 | PDI_H | 0.0003522031834 | 0.0002980471314 | 1.18170298 | 0.237325754 |
| CE.to.TLs.ratio.in.L.VLDL | 129001 | PDI_H | 0.0003240585184 | 0.0002766552152 | 1.171344332 | 0.2414625621 |
| CE.in.V..L.VLDL | 129001 | PDI_H | 0.0003279171148 | 0.0002851176787 | 1.150111478 | 0.2501000918 |
| CE.in.S.LDL | 129001 | PDI_H | 0.0003375327122 | 0.000297341868 | 1.135167121 | 0.2563074121 |
| TLs.in.L.LDL | 129001 | PDI_H | 0.0003291669442 | 0.000294638337 | 1.117189798 | 0.2639152627 |
| ApoA1 | 129001 | PDI_H | 0.0002960898825 | 0.0002652942686 | 1.116080962 | 0.2643895596 |
| Citrate | 129001 | PDI_H | 0.0003092501032 | 0.0002923767852 | 1.057710868 | 0.2901892598 |
| Free.Chol.to.TLs.ratio.in.S.VLDL | 129001 | PDI_H | -0.0002877447737 | 0.0002768681859 | -1.039284354 | 0.2986744573 |
| Free.Chol.in.L.LDL | 129001 | PDI_H | 0.000296158508 | 0.0002906550519 | 1.018934665 | 0.3082358961 |
| Con.of.HDL.particles | 129001 | PDI_H | -0.000275103548 | 0.0002745128557 | -1.002151784 | 0.3162721708 |
| Free.Chol.to.TLs.ratio.in.M.VLDL | 129001 | PDI_H | 0.0002702395399 | 0.000275965724 | 0.979250379 | 0.3274581183 |
| PL.to.TLs.ratio.in.M.VLDL | 129001 | PDI_H | -0.0002617058695 | 0.0002800220458 | -0.9345902345 | 0.3500012632 |

| Metabolite | N_used | term | beta | se | t | p_val |
|---|---|---|---|---|---|---|
| TLs.in.LDL | 129001 | PDI_H | 0.0002770501328 | 0.0002969154877 | 0.9330942449 | 0.3507730569 |
| CE.to.TLs.ratio.in.V..L.VLDL | 129001 | PDI_H | -0.000247951493 | 0.0002743795941 | -0.9036805153 | 0.3661665215 |
| TG.to.TLs.ratio.in.V..S.VLDL | 129001 | PDI_H | -0.000244000035 | 0.0002738899542 | -0.8908688737 | 0.3730011822 |
| Free.Chol.in.LDL | 129001 | PDI_H | 0.0002602836184 | 0.0002938367825 | 0.8858101977 | 0.375721465 |
| PL.to.TLs.ratio.in.chylomicrons.and.extremely.L.VLDL | 129001 | PDI_H | -0.0002587905558 | 0.0002984051911 | -0.8672454888 | 0.3858091304 |
| Chol.to.TLs.ratio.in.L.VLDL | 129001 | PDI_H | -0.00024178678 | 0.0002881875532 | -0.8389910574 | 0.4014758827 |
| CE.to.TLs.ratio.in.IDL | 129001 | PDI_H | 0.0002384884839 | 0.0002884656903 | 0.8267481781 | 0.4083813373 |
| TG.in.M.LDL | 129001 | PDI_H | -0.0002363423989 | 0.000286495873 | -0.8249417222 | 0.4094062033 |
| Chol.to.TLs.ratio.in.M.VLDL | 129001 | PDI_H | 0.0002125397939 | 0.0002701418225 | 0.7867711557 | 0.431417287 |
| TG.to.TLs.ratio.in.M.HDL | 129001 | PDI_H | 0.0002117978569 | 0.0002764058024 | 0.7662569132 | 0.4435248523 |
| Free.Chol.to.TLs.ratio.in.chylomicrons.and.extremely.L.VLDL | 129001 | PDI_H | -0.0002162189048 | 0.0002886775004 | -0.7489981191 | 0.4538597049 |
| Clinical.LDL_C | 129001 | PDI_H | 0.0002158421781 | 0.0002955233865 | 0.7303725797 | 0.4651638009 |
| Total.Con.of.LP.particles | 129001 | PDI_H | -0.0001979913904 | 0.0002754771004 | -0.7187217746 | 0.4723136661 |
| Average.diameter.for.LDL.particles | 129001 | PDI_H | -0.0002006080179 | 0.0002845447217 | -0.7050140192 | 0.4808027848 |
| CE.to.TLs.ratio.in.M.VLDL | 129001 | PDI_H | 0.0001875352459 | 0.0002685124873 | 0.69842281 | 0.4849140747 |
| TG.to.TLs.ratio.in.S.HDL | 129001 | PDI_H | 0.0001828554025 | 0.0002715027688 | 0.6734936933 | 0.5006344664 |
| CE.in.M.HDL | 129001 | PDI_H | -0.0001761644773 | 0.0002666241949 | -0.6607220222 | 0.5087917781 |
| CE.in.chylomicrons.and.extremely.L.VLDL | 129001 | PDI_H | -0.000181008638 | 0.0002765377277 | -0.65455314 | 0.5127566699 |
| TG.in.M.HDL | 129001 | PDI_H | 0.0001756719302 | 0.0002904994934 | 0.6047237059 | 0.5453636579 |
| TG.to.TLs.ratio.in.V..L.VLDL | 129001 | PDI_H | -0.0001511741461 | 0.0002805925283 | -0.5387675394 | 0.5900481941 |
| PL.in.LDL | 129001 | PDI_H | 0.0001474076851 | 0.0002970786349 | 0.4961907985 | 0.6197606478 |
| Con.of.M.HDL.particles | 129001 | PDI_H | 0.0001301623634 | 0.000267732785 | 0.4861652015 | 0.6268508675 |
| Chol.to.TLs.ratio.in.IDL | 129001 | PDI_H | 0.0001289276449 | 0.0002858280513 | 0.4510671513 | 0.6519419101 |
| TG.to.TLs.ratio.in.M.VLDL | 129001 | PDI_H | -0.0001170811562 | 0.0002718537096 | -0.4306770591 | 0.6667039213 |
| PL.in.L.LDL | 129001 | PDI_H | 0.0001211962793 | 0.0002946675933 | 0.4112982971 | 0.6808545015 |
| Chol.in.M.LDL | 129001 | PDI_H | 0.0001209266268 | 0.0002974998601 | 0.4064762475 | 0.6843933803 |
| Free.Chol.in.M.LDL | 129001 | PDI_H | 0.0001198734224 | 0.0002975018949 | 0.4029333071 | 0.6869979558 |
| CE.in.M.LDL | 129001 | PDI_H | 0.0001187355599 | 0.0002964887 | 0.400472463 | 0.6888092249 |
| Sphingomyelins | 129001 | PDI_H | -0.0001074095132 | 0.000274819761 | -0.3908362077 | 0.695918955 |
| TG.to.TLs.ratio.in.L.HDL | 129001 | PDI_H | -0.0001054018192 | 0.0002709579276 | -0.3889969934 | 0.6972790126 |
| Free.Chol.to.TLs.ratio.in.IDL | 129001 | PDI_H | -7.70587e-05 | 0.0002934236297 | -0.262619399 | 0.7928443659 |
| Free.Chol.to.TLs.ratio.in.S.LDL | 129001 | PDI_H | -6.83578e-05 | 0.0002846847189 | -0.2401173864 | 0.8102396376 |
| PL.in.M.HDL | 129001 | PDI_H | -6.15914e-05 | 0.0002746486305 | -0.2242553136 | 0.8225590005 |
| Free.Chol.to.TLs.ratio.in.L.LDL | 129001 | PDI_H | 6.07603e-05 | 0.0002716354979 | 0.2236833118 | 0.8230040865 |
| TLs.in.M.LDL | 129001 | PDI_H | 6.04108e-05 | 0.0002972950876 | 0.2032015473 | 0.838977824 |
| Chol.in.M.HDL | 129001 | PDI_H | -5.15607e-05 | 0.0002656558924 | -0.1940883297 | 0.8461070251 |
| Chol.in.chylomicrons.and.extremely.L.VLDL | 129001 | PDI_H | 5.21475e-05 | 0.0002758951278 | 0.1890119569 | 0.8500837428 |
| PL.in.M.LDL | 129001 | PDI_H | -3.84678e-05 | 0.0002977772607 | -0.1291832124 | 0.8972128808 |
| Glycine | 129001 | PDI_H | -3.19512e-05 | 0.000277879149 | -0.1149824867 | 0.9084591825 |
| TLs.in.M.HDL | 129001 | PDI_H | -2.93994e-05 | 0.0002712036722 | -0.1084033662 | 0.9136758996 |
| Chol.to.TLs.ratio.in.M.HDL | 129001 | PDI_H | 2.69396e-05 | 0.0002693142016 | 0.1000301968 | 0.9203205075 |
| TG.in.IDL | 129001 | PDI_H | -1.69353e-05 | 0.0002898368014 | -0.05843038334 | 0.9534059042 |
| Chol.to.TLs.ratio.in.V..L.VLDL | 129001 | PDI_H | 6.32758e-08 | 0.0002738751449 | 0.000231038955 | 0.9998156579 |

**Supplementary Table 8. Associations between circulating plasma proteins and incident age-related macular degeneration (AMD) in the UK Biobank**

| Protein | beta | se | z | p_val | HR |
|---|---|---|---|---|---|
| pklr | 0.2511034939 | 0.05644058623 | 4.448988055 | 8.62758e-06 | 1.285443113 |
| blvrb | 0.2448707876 | 0.05702184621 | 4.294332855 | 1.75219e-05 | 1.277456239 |
| ubac1 | 0.2418563796 | 0.05750114321 | 4.206114281 | 2.59799e-05 | 1.273611263 |
| c9orf40 | 0.2418288248 | 0.05999890095 | 4.030554242 | 5.56455e-05 | 1.273576169 |
| ca2 | 0.2294033368 | 0.0575035634 | 3.989376019 | 6.62473e-05 | 1.257849274 |
| znf75d | -0.2403850308 | 0.0609515273 | -3.943872146 | 8.01764e-05 | 0.7863250434 |
| hagh | 0.2265583685 | 0.05745580506 | 3.943176295 | 8.04095e-05 | 1.254275818 |
| ca1 | 0.2220995646 | 0.05745801363 | 3.865423647 | 0.0001108965383 | 1.248695698 |
| sh3glb2 | 0.2340166686 | 0.06173769549 | 3.790498929 | 0.0001503449809 | 1.263665556 |
| dnaja4 | 0.2258161603 | 0.06122537519 | 3.68827728 | 0.000225777533 | 1.253345229 |
| aarsd1 | 0.2153633234 | 0.058595367 | 3.675432623 | 0.0002374467021 | 1.24031245 |
| psme2 | 0.2087731389 | 0.05806431742 | 3.595549696 | 0.0003237073227 | 1.232165436 |
| ampd3 | 0.2152399847 | 0.06053650591 | 3.555540272 | 0.0003772031583 | 1.240159481 |
| prdx2 | 0.2179204777 | 0.06129521927 | 3.555260594 | 0.0003776046415 | 1.243488179 |
| nap1l4 | 0.2181447062 | 0.06179122008 | 3.530351171 | 0.0004150084286 | 1.243767035 |
| arg1 | 0.2011320441 | 0.05719341754 | 3.516699172 | 0.0004369486969 | 1.222786223 |
| tbc1d17 | 0.1996391118 | 0.05810525017 | 3.435818815 | 0.0005907659165 | 1.220962048 |
| tgm2 | 0.1908950159 | 0.05606011021 | 3.405184456 | 0.0006611937652 | 1.21033238 |
| dnph1 | 0.193323619 | 0.05693165122 | 3.395714244 | 0.0006844978082 | 1.213275369 |
| cat | 0.210789011 | 0.06236222211 | 3.38007537 | 0.0007246595477 | 1.23465183 |
| psg1 | 0.1914199535 | 0.0568535903 | 3.36689297 | 0.0007602018647 | 1.210967896 |
| cbln4 | -0.1890015138 | 0.05659994652 | -3.339252516 | 0.0008400416533 | 0.8277852536 |
| psmg3 | 0.1925164873 | 0.05773027847 | 3.334757642 | 0.0008537377165 | 1.212296491 |
| ddi2 | 0.2023644142 | 0.0611678667 | 3.308345136 | 0.0009384907344 | 1.224294077 |
| ca12 | 0.1915210889 | 0.05835708426 | 3.281882419 | 0.001031165937 | 1.211090373 |
| ghr | -0.2259611105 | 0.06899875725 | -3.274857687 | 0.001057152147 | 0.7977491251 |
| cpm | -0.2151298708 | 0.06681027998 | -3.220011514 | 0.00128185448 | 0.8064367008 |
| lhpp | 0.1856115523 | 0.05790210645 | 3.205609669 | 0.001347766766 | 1.203954496 |
| psmd9 | 0.1840776196 | 0.05779658345 | 3.184922164 | 0.001447929469 | 1.202109127 |
| hmbs | 0.1784016786 | 0.05662065955 | 3.15082304 | 0.001628111011 | 1.195305353 |
| dsg2 | 0.1818625487 | 0.05785804323 | 3.143254395 | 0.001670805631 | 1.199449317 |
| ntf3 | -0.1783265649 | 0.0578579204 | -3.08214612 | 0.002055139282 | 0.8366691521 |
| psmg4 | 0.1910125867 | 0.06230101829 | 3.065962514 | 0.002169705152 | 1.210474688 |
| sod1 | 0.1782766156 | 0.05829027169 | 3.058428284 | 0.002225013287 | 1.195155874 |
| wfikkn1 | -0.1809511013 | 0.06005779321 | -3.012949555 | 0.002587219195 | 0.8344761625 |
| acyp1 | 0.1846558728 | 0.06204752175 | 2.976039454 | 0.00291997293 | 1.202804451 |
| ccs | 0.1705176134 | 0.05738569296 | 2.971430762 | 0.00296415702 | 1.18591854 |
| vsig4 | 0.1858443113 | 0.06310068606 | 2.945202706 | 0.003227431421 | 1.20423476 |
| zbtb17 | 0.174335744 | 0.05978514554 | 2.916037795 | 0.003545075533 | 1.190455187 |
| pnliprp2 | 0.1628071207 | 0.05583536055 | 2.915842561 | 0.003547294534 | 1.176809686 |
| tnfsf11 | -0.1693689325 | 0.05824842011 | -2.907700023 | 0.003640974138 | 0.8441973941 |
| cenpf | 0.1784482022 | 0.06145034768 | 2.903941295 | 0.003684972477 | 1.195360965 |
| st13 | 0.178474597 | 0.06182236407 | 2.886893758 | 0.003890655803 | 1.195392516 |
| hbq1 | 0.1622911995 | 0.05649883212 | 2.872469985 | 0.004072767653 | 1.176202701 |
| spink2 | -0.1955437881 | 0.06813372622 | -2.869999909 | 0.004104719169 | 0.822387332 |
| pebp1 | 0.1659034123 | 0.05820366945 | 2.850394381 | 0.004366504979 | 1.180459078 |
| itih3 | 0.1715320269 | 0.06041303506 | 2.83932146 | 0.004520958693 | 1.187122162 |
| ahsp | 0.1671436338 | 0.05903544934 | 2.831241833 | 0.004636764855 | 1.181924017 |
| clec5a | 0.16723201 | 0.05919467942 | 2.82511894 | 0.004726306811 | 1.182028476 |
| spink1 | 0.1662718887 | 0.0590349429 | 2.816499526 | 0.004855011462 | 1.18089413 |
| mmp12 | 0.1755149446 | 0.06237039005 | 2.814074827 | 0.004891784021 | 1.1918598 |
| aoc1 | -0.1601061465 | 0.05702679709 | -2.80755986 | 0.004991840141 | 0.8520533417 |
| nedd4l | 0.1750098482 | 0.0624889628 | 2.800652153 | 0.005099945907 | 1.191257948 |
| cx3cl1 | 0.1594441565 | 0.05713834523 | 2.790493071 | 0.005262782665 | 1.172858764 |
| fcamr | -0.1843741863 | 0.06618147274 | -2.785888235 | 0.005338127356 | 0.8316245631 |
| dcc | -0.1716106209 | 0.06163666887 | -2.784229325 | 0.005365508357 | 0.8423070861 |
| ca3 | 0.1635208277 | 0.05915390747 | 2.764328423 | 0.005704009626 | 1.177649883 |
| cyb5a | 0.1691679303 | 0.06137918212 | 2.756112488 | 0.0058492877 | 1.184319005 |
| ak1 | 0.15776836 | 0.05795422041 | 2.722292852 | 0.006483066336 | 1.170894937 |
| sumf1 | 0.1706861784 | 0.06283754599 | 2.716308789 | 0.006601431547 | 1.186118461 |
| oxt | -0.167692359 | 0.06184547742 | -2.711473272 | 0.00669849413 | 0.8456139402 |
| eif4ebp1 | 0.1566953997 | 0.05789586277 | 2.706504268 | 0.006799570833 | 1.169639287 |
| ppme1 | 0.1544731285 | 0.05708579774 | 2.705981779 | 0.006810278253 | 1.167042917 |
| tnfrsf10c | 0.1530310423 | 0.05665896874 | 2.700914713 | 0.006914906801 | 1.165361154 |
| ppy | -0.157599474 | 0.05852058931 | -2.693060269 | 0.007079946948 | 0.8541918395 |
| lpcat2 | 0.1575149123 | 0.05878394449 | 2.6795567 | 0.007371971696 | 1.170598214 |
| atp6v1d | 0.1536278582 | 0.05758424969 | 2.667879829 | 0.007633155457 | 1.166056868 |
| atg4a | 0.1519886157 | 0.05740549012 | 2.647632053 | 0.00810576953 | 1.164146983 |

| Protein | beta | se | z | p_val | HR |
|---|---|---|---|---|---|
| rilp | 0.1508442559 | 0.05703416136 | 2.644805365 | 0.008173791795 | 1.162815542 |
| yod1 | 0.1644601064 | 0.06232808234 | 2.638619708 | 0.008324430466 | 1.178756544 |
| sirpa | 0.1466377982 | 0.05557385845 | 2.638611071 | 0.008324642529 | 1.157934481 |
| tigit | 0.1573429321 | 0.05978804491 | 2.63167883 | 0.00849641393 | 1.170396912 |
| psme1 | 0.154013076 | 0.05856781835 | 2.629653628 | 0.008547190346 | 1.16650614 |
| creg1 | -0.1546913913 | 0.05895554143 | -2.623865163 | 0.008693819523 | 0.8566795154 |
| fap | -0.1511593552 | 0.05815640522 | -2.599186704 | 0.009344493378 | 0.8597106884 |
| glo1 | 0.1519059567 | 0.05847120725 | 2.597961695 | 0.009377895466 | 1.16405076 |
| azu1 | 0.1521859879 | 0.05865887843 | 2.594423759 | 0.00947496253 | 1.164376776 |
| vasn | 0.150889232 | 0.05821117324 | 2.592100857 | 0.009539180146 | 1.162867842 |
| timp4 | 0.1571011206 | 0.06071806336 | 2.587386881 | 0.00967069428 | 1.17011393 |
| dnajc9 | 0.1581021983 | 0.06117805223 | 2.584296043 | 0.009757799813 | 1.171285892 |
| igfbp2 | 0.1627161759 | 0.06339540898 | 2.56668706 | 0.01026752283 | 1.176702666 |
| ankrd54 | 0.1447428225 | 0.05644414926 | 2.564354754 | 0.01033678329 | 1.155742301 |
| apof | 0.1745182214 | 0.06814038135 | 2.561157099 | 0.01043241698 | 1.190672438 |
| dcdc2c | 0.1547161387 | 0.06079160613 | 2.545024692 | 0.01092700111 | 1.167326555 |
| dnajb2 | 0.1580066026 | 0.06217975884 | 2.541126013 | 0.01104960873 | 1.171173928 |
| ctso | -0.148962097 | 0.05875081819 | -2.535489745 | 0.01122902161 | 0.8616017715 |
| prcp | -0.1559032485 | 0.06159455263 | -2.531120721 | 0.01136987086 | 0.855641971 |
| gdf15 | 0.1865342441 | 0.07387716455 | 2.524924247 | 0.01157232314 | 1.205065888 |
| vcan | 0.1456432625 | 0.05830062957 | 2.49814219 | 0.01248461059 | 1.156783446 |
| sil1 | -0.1556252958 | 0.06261119722 | -2.485582494 | 0.01293396062 | 0.8558798321 |
| ddt | 0.1546094442 | 0.06223156857 | 2.484421456 | 0.01297621304 | 1.167202015 |
| sfrp4 | -0.1611369322 | 0.06491323744 | -2.48234318 | 0.01305215046 | 0.8511755097 |
| s100a3 | -0.1519249416 | 0.06126980186 | -2.479605564 | 0.01315277902 | 0.8590527574 |
| aldh1a1 | 0.1482094673 | 0.0599547191 | 2.472023379 | 0.01343507301 | 1.159755802 |
| pla2g7 | -0.1429769602 | 0.05817144352 | -2.457854775 | 0.01397696818 | 0.866774039 |
| ggct | 0.1484715913 | 0.0605346937 | 2.452669407 | 0.01418005922 | 1.160059842 |
| chac2 | 0.1409830144 | 0.05757632144 | 2.448628374 | 0.01434013184 | 1.15140509 |
| mdh1 | 0.1495111607 | 0.06115540843 | 2.444774135 | 0.01449428817 | 1.161266431 |
| pik3ap1 | 0.1397997186 | 0.05724561132 | 2.442103689 | 0.01460195202 | 1.150043443 |
| brd3 | -0.148832962 | 0.06096435624 | -2.441311139 | 0.01463404049 | 0.8617130417 |
| rbks | 0.1462005471 | 0.05991742622 | 2.440033833 | 0.01468588645 | 1.157428284 |
| hebp1 | 0.1385951835 | 0.05690646332 | 2.435491074 | 0.01487159151 | 1.14865901 |
| pzp | 0.2123216401 | 0.08731707468 | 2.431616507 | 0.01503161312 | 1.236545544 |
| klk4 | 0.1500430384 | 0.06175245346 | 2.429750236 | 0.01510923088 | 1.161884247 |
| ebi3_il27 | 0.1428971191 | 0.05883731675 | 2.428681779 | 0.01515382646 | 1.153611111 |
| cct5 | 0.138496002 | 0.05711768083 | 2.424748345 | 0.01531900168 | 1.14854509 |
| ret | -0.1456382883 | 0.0601768342 | -2.420171985 | 0.01551316806 | 0.8644703357 |
| ldlr | -0.14914423 | 0.06172821838 | -2.416143441 | 0.01568588063 | 0.8614448598 |
| enpp7 | -0.1446739312 | 0.05993197993 | -2.413968825 | 0.01577981245 | 0.8653043959 |
| serpina3 | 0.1475942824 | 0.06149075668 | 2.400267785 | 0.01638308185 | 1.159042557 |
| lxn | 0.136003012 | 0.05670057569 | 2.398617833 | 0.01645708057 | 1.145685344 |
| mansc1 | 0.1372876698 | 0.05743786305 | 2.390194595 | 0.01683944789 | 1.147158104 |
| lto1 | 0.1366471985 | 0.05765293208 | 2.370169106 | 0.01777995101 | 1.146423617 |
| entpd2 | -0.1375243082 | 0.05815672031 | -2.364719116 | 0.01804375006 | 0.8715131648 |
| grk5 | -0.1368442519 | 0.05809240751 | -2.355630586 | 0.01849129644 | 0.8721060444 |
| nell2 | -0.1386412543 | 0.05890910585 | -2.353477486 | 0.01859873411 | 0.870540275 |
| mamdc4 | -0.1487327412 | 0.06349416562 | -2.342463118 | 0.0191569273 | 0.8617994076 |
| tfrc | 0.1386187958 | 0.05936261909 | 2.335119271 | 0.01953719087 | 1.148686133 |
| shbg | 0.1622252251 | 0.06961152492 | 2.330436307 | 0.0197831027 | 1.176125104 |
| slitrk6 | 0.1375673301 | 0.05909049126 | 2.328078971 | 0.01990791072 | 1.147478963 |
| atxn3 | 0.1410980191 | 0.06072748025 | 2.323462434 | 0.02015432347 | 1.151537515 |
| erbb2 | -0.1465668376 | 0.06325880994 | -2.316939534 | 0.02050702583 | 0.8636680049 |
| crlf1 | -0.1422869632 | 0.06158876255 | -2.310274754 | 0.02087294772 | 0.8673723168 |
| lbp | 0.1334678993 | 0.05813607366 | 2.295784543 | 0.02168820366 | 1.142784581 |
| mstn | -0.146080103 | 0.06385933539 | -2.287529334 | 0.02216494525 | 0.8640884844 |
| nsfl1c | 0.1321690726 | 0.05779417023 | 2.286892815 | 0.02220208013 | 1.141301266 |
| fga | 0.1493619196 | 0.06596411058 | 2.264290661 | 0.02355624357 | 1.161093136 |
| cnpy2 | 0.1294061658 | 0.05729519741 | 2.258586612 | 0.0239091114 | 1.138152309 |
| ace2 | -0.1521056687 | 0.06738920402 | -2.257122204 | 0.0240004398 | 0.8588975174 |
| cd38 | -0.1585446045 | 0.07033519065 | -2.254129164 | 0.02418804346 | 0.8533848981 |
| abhd14b | 0.1312184112 | 0.0582779649 | 2.251595631 | 0.02434783738 | 1.14021679 |
| hla_a | -0.1371323939 | 0.06096291694 | -2.249439509 | 0.02448454733 | 0.8718547902 |
| gfap | 0.1383490643 | 0.06164969044 | 2.244116124 | 0.02482493081 | 1.148376338 |
| ssna1 | 0.13581217 | 0.06069966025 | 2.237445308 | 0.02525725009 | 1.14546672 |
| igsf9 | -0.157922891 | 0.07080665214 | -2.230339752 | 0.02572489602 | 0.853915624 |
| carhsp1 | 0.1297894203 | 0.05823577344 | 2.228688874 | 0.02583461306 | 1.138588594 |
| cstb | 0.1365705693 | 0.06143701567 | 2.222936251 | 0.0262200989 | 1.146335771 |
| serpina11 | 0.1377946896 | 0.06203397786 | 2.221277666 | 0.02633216058 | 1.147739883 |

| Protein | beta | se | z | p_val | HR |
|---|---|---|---|---|---|
| cdhr2 | -0.1666477231 | 0.07522134933 | -2.215431186 | 0.02673048279 | 0.8464977604 |
| sema4c | -0.1298628433 | 0.0586243807 | -2.215167849 | 0.02674854577 | 0.8782158758 |
| nadk | 0.1271620437 | 0.05794267859 | 2.194617971 | 0.02819101335 | 1.13560102 |
| s100a12 | 0.1259199169 | 0.05741837377 | 2.193024787 | 0.02830559326 | 1.134191335 |
| stambp | 0.1263772381 | 0.05778008804 | 2.187210896 | 0.02872713183 | 1.134710143 |
| spon2 | 0.1290841352 | 0.05932748737 | 2.175789687 | 0.02957099021 | 1.137785848 |
| dmp1 | -0.1385915552 | 0.0640301511 | -2.164473343 | 0.03042804026 | 0.8705835411 |
| nfasc | -0.1302115006 | 0.06032574606 | -2.158473108 | 0.030891067 | 0.8779097328 |
| gask1a | -0.137632714 | 0.0638552008 | -2.155387693 | 0.03113150859 | 0.8714186929 |
| mcee | -0.1307559986 | 0.06070087514 | -2.15410401 | 0.03123201614 | 0.8774318429 |
| erbb3 | -0.1337012471 | 0.06211907228 | -2.152338117 | 0.03137073379 | 0.87485139 |
| aprt | 0.1243669474 | 0.05788858506 | 2.148384648 | 0.03168321137 | 1.132431338 |
| cd300lf | 0.120502214 | 0.05614036709 | 2.146445067 | 0.031837487 | 1.128063239 |
| nptx1 | -0.1316298535 | 0.06171672666 | -2.132806787 | 0.03294057925 | 0.8766654297 |
| hddc2 | 0.1291947725 | 0.06071565238 | 2.127866002 | 0.03334819514 | 1.137911736 |
| cfi | -0.1447041412 | 0.068376915 | -2.116271862 | 0.03432169244 | 0.8652782554 |
| traf2 | 0.1235920426 | 0.05840712331 | 2.116043996 | 0.03434106588 | 1.131554151 |
| vsnl1 | -0.1429026832 | 0.06755646247 | -2.115307373 | 0.03440375848 | 0.8668384228 |
| tpk1 | 0.1304372333 | 0.06167335715 | 2.114968916 | 0.03443259671 | 1.139326426 |
| ece1 | -0.1217601124 | 0.05779164216 | -2.106880993 | 0.03512789653 | 0.8853607301 |
| dpp4 | -0.1270365389 | 0.06036959534 | -2.104313243 | 0.03535113162 | 0.8807014921 |
| fuom | -0.1411029178 | 0.06729949489 | -2.096641557 | 0.03602531763 | 0.8683999333 |
| spag1 | -0.1314907639 | 0.06277259658 | -2.094716023 | 0.03619624401 | 0.8767873732 |
| itm2a | -0.1186376407 | 0.05668196414 | -2.093040397 | 0.03634554859 | 0.8881295645 |
| eno3 | -0.1419868083 | 0.06784225381 | -2.092896392 | 0.03635840437 | 0.867632702 |
| coch | -0.1310336627 | 0.06283394646 | -2.085396033 | 0.03703336662 | 0.8771882454 |
| wasl | 0.1297082087 | 0.06225441882 | 2.083518104 | 0.03720402327 | 1.138496132 |
| gas6 | 0.1227395856 | 0.05898442751 | 2.080881188 | 0.03744478249 | 1.130589961 |
| cd163 | 0.1241096784 | 0.05966725976 | 2.080029799 | 0.0375227997 | 1.132140035 |
| selplg | -0.1196608368 | 0.05778903968 | -2.070649339 | 0.0383915778 | 0.8872212985 |
| klkb1 | -0.1333222567 | 0.06439157359 | -2.070492291 | 0.0384062672 | 0.875183013 |
| tp53i3 | 0.131996809 | 0.06381391121 | 2.068464485 | 0.03859636691 | 1.141104678 |
| omp | 0.1263964397 | 0.06117321651 | 2.066205554 | 0.03880907469 | 1.134731932 |
| lilrb2 | 0.1162963298 | 0.05633031422 | 2.064542536 | 0.03896630525 | 1.123328699 |
| ccl19 | 0.1184373501 | 0.05759202791 | 2.056488622 | 0.0397354419 | 1.125736345 |
| pcdh12 | -0.1232332751 | 0.06005339249 | -2.052061841 | 0.04016365282 | 0.8840574099 |
| s100a14 | -0.1195073669 | 0.05867764549 | -2.036676248 | 0.04168249626 | 0.8873574708 |
| was | -0.1161416343 | 0.05715907483 | -2.031901928 | 0.04216358533 | 0.8903491103 |
| il15ra | 0.1181974272 | 0.05818915666 | 2.031262077 | 0.04222841616 | 1.125466287 |
| gp2 | 0.1232418331 | 0.06074179554 | 2.028946164 | 0.04246377401 | 1.131157939 |
| il1rl1 | 0.1256656853 | 0.06198870292 | 2.027235276 | 0.04263835714 | 1.133903024 |
| bhlhe40 | -0.1245928326 | 0.06150486932 | -2.025739328 | 0.04279150426 | 0.8828562997 |
| csf2ra | -0.1158088533 | 0.05725304932 | -2.022754327 | 0.04309848266 | 0.890645451 |
| magea3 | -0.1224854405 | 0.06067021603 | -2.018872662 | 0.04350045634 | 0.8847187859 |
| mnda | 0.1135176622 | 0.0563049091 | 2.01612371 | 0.04378704137 | 1.120211674 |
| hspa2 | 0.1258040525 | 0.06242798297 | 2.015186884 | 0.04388507124 | 1.13405993 |
| fut3_fut5 | 0.1179718892 | 0.05854506992 | 2.015061035 | 0.04389825425 | 1.12521248 |
| cntn3 | -0.1306056673 | 0.06482551462 | -2.01472627 | 0.04393333808 | 0.8775637583 |
| gipc2 | 0.1301167597 | 0.06485824735 | 2.006171381 | 0.04483796611 | 1.13896136 |
| apod | 0.1300460261 | 0.06491790801 | 2.003238091 | 0.04515173896 | 1.1388808 |
| mln | -0.1212520603 | 0.06062741671 | -1.999954259 | 0.0455052033 | 0.8858106538 |
| il12rb1 | -0.1153946627 | 0.05776265787 | -1.997738106 | 0.04574506071 | 0.8910144243 |
| aif1 | 0.1163780639 | 0.05843476521 | 1.991589484 | 0.04641611815 | 1.123420517 |
| angptl7 | -0.1150263365 | 0.05785912259 | -1.988041494 | 0.04680710102 | 0.8913426687 |
| cep350 | -0.1222655653 | 0.06167892898 | -1.982290667 | 0.04744671801 | 0.8849133351 |
| fndc1 | -0.1229398442 | 0.06210721877 | -1.979477533 | 0.04776226669 | 0.8843168577 |
| add1 | 0.1212995653 | 0.06128977954 | 1.979115706 | 0.04780298056 | 1.12896306 |
| tef | -0.1211040763 | 0.06129757938 | -1.975674692 | 0.04819163303 | 0.8859417493 |
| csrp3 | -0.1256897053 | 0.06379470601 | -1.970221561 | 0.04881298355 | 0.8818884497 |
| cfhr5 | 0.1189325518 | 0.06052038801 | 1.965165057 | 0.04939513681 | 1.126293949 |
| rabgap1l | 0.1138950578 | 0.05800868319 | 1.96341395 | 0.04959809415 | 1.120634517 |
| clec4a | 0.1142057454 | 0.05817951925 | 1.962988812 | 0.04964747403 | 1.120982738 |
| gal | 0.1170894161 | 0.05968722739 | 1.961716454 | 0.04979550505 | 1.124219949 |
| gmpr2 | 0.1209320642 | 0.06173004027 | 1.959047226 | 0.05010725631 | 1.128548241 |
| cdh5 | -0.1114889904 | 0.05692499694 | -1.958524311 | 0.05016852118 | 0.8945012394 |
| anxa1 | 0.1254485074 | 0.06408679976 | 1.957478107 | 0.05029128335 | 1.133656793 |
| ptprm | -0.1136435143 | 0.05806787774 | -1.957080553 | 0.05033799842 | 0.8925760898 |
| cd55 | 0.1165224407 | 0.05959892952 | 1.955109624 | 0.05057013261 | 1.123582724 |
| rab44 | 0.1191019915 | 0.06092076033 | 1.955031271 | 0.0505793794 | 1.126484804 |
| col15a1 | -0.124451346 | 0.06368142135 | -1.954280281 | 0.05066807955 | 0.8829812209 |

| Protein | beta | se | z | p_val | HR |
|---|---|---|---|---|---|
| prkcq | -0.1115140706 | 0.0571398658 | -1.951598399 | 0.05098590344 | 0.8944788054 |
| mb | -0.1344030438 | 0.06889534159 | -1.950829196 | 0.05107736756 | 0.8742376375 |
| tdp1 | 0.1200704352 | 0.06174220086 | 1.944706109 | 0.05181035981 | 1.12757627 |
| cxcl17 | -0.1390959508 | 0.07162385407 | -1.942033874 | 0.05213299964 | 0.8701445334 |
| adamts8 | -0.110870234 | 0.05714126172 | -1.940283268 | 0.05234527373 | 0.8950548891 |
| dapk2 | 0.1193660273 | 0.06154198501 | 1.93958689 | 0.05242991542 | 1.126782276 |
| crygd | 0.1169999477 | 0.06036197965 | 1.938305343 | 0.05258598092 | 1.124119371 |
| pilra | 0.1124631691 | 0.0580991327 | 1.935711669 | 0.05290302442 | 1.119031041 |
| sgsh | -0.1212089911 | 0.06265896989 | -1.934423616 | 0.05306106518 | 0.8858488057 |
| nectin1 | 0.1184512227 | 0.06128999059 | 1.932635681 | 0.05328109365 | 1.125751962 |
| ssh3 | 0.1177840025 | 0.06098361384 | 1.931404112 | 0.05343309701 | 1.125001088 |
| sparcl1 | 0.1131480797 | 0.05864645099 | 1.929325267 | 0.05369049489 | 1.11979774 |
| nmnat1 | 0.1113794619 | 0.05786905121 | 1.924680976 | 0.05426928197 | 1.117818996 |
| nhlrc3 | -0.126782876 | 0.06591262117 | -1.923499229 | 0.05441738326 | 0.8809249218 |
| itprip | -0.1200226814 | 0.06244984256 | -1.921905268 | 0.05461767891 | 0.8869003204 |
| sema6c | -0.1182375482 | 0.06163049223 | -1.918491057 | 0.05504877498 | 0.8884849695 |
| fgr | 0.1107078309 | 0.05777852772 | 1.916072204 | 0.05535590581 | 1.117068486 |
| il5 | -0.1092066961 | 0.05704789314 | -1.914298497 | 0.05558202638 | 0.896545086 |
| ubxn1 | 0.1189269601 | 0.0622891888 | 1.909271294 | 0.05622710339 | 1.126287651 |
| otud6b | 0.1166590127 | 0.06112516582 | 1.908526728 | 0.056323172 | 1.123736185 |
| fam3b | 0.1092182145 | 0.05744171184 | 1.901374646 | 0.05725295803 | 1.115405722 |
| cd276 | 0.1081452566 | 0.05691875693 | 1.899993297 | 0.05743399932 | 1.11420958 |
| impact | 0.1150187718 | 0.06068197702 | 1.895435472 | 0.05803473296 | 1.121894497 |
| mylpf | -0.1217016059 | 0.06437550683 | -1.890495499 | 0.05869172195 | 0.8854125309 |
| glod4 | 0.1093400224 | 0.05786557515 | 1.889552158 | 0.0588178808 | 1.115541595 |
| arsa | -0.112636716 | 0.05967372319 | -1.887542958 | 0.05908733407 | 0.8934751864 |
| naaa | -0.1061194285 | 0.05622834833 | -1.887294072 | 0.05912078333 | 0.8993172376 |
| ntrk2 | -0.1108832224 | 0.0588899971 | -1.882887212 | 0.05971565468 | 0.8950432638 |
| areg | 0.1152171884 | 0.06122901663 | 1.881741611 | 0.05987110699 | 1.122117122 |
| pilrb | 0.1051254973 | 0.05609741828 | 1.873981023 | 0.06093304073 | 1.11085001 |
| sprr1b | -0.1125493838 | 0.06010503225 | -1.872545103 | 0.06113122808 | 0.893553219 |
| dtymk | 0.1158985886 | 0.06191302491 | 1.871958102 | 0.06121240024 | 1.122881993 |
| traf3 | 0.1176429994 | 0.06295813865 | 1.868590812 | 0.06167976508 | 1.12484247 |
| ca4 | 0.1055898954 | 0.05672539293 | 1.861422017 | 0.06268460373 | 1.111366007 |
| ddx1 | 0.1120529836 | 0.06021539867 | 1.860869248 | 0.06276264342 | 1.118572125 |
| il3 | -0.1141180688 | 0.06154774135 | -1.85413902 | 0.06371927353 | 0.8921526143 |
| ccl11 | 0.1124569164 | 0.06069395774 | 1.852851925 | 0.06390358536 | 1.119024044 |
| cinp | -0.1105897645 | 0.05978262122 | -1.849864764 | 0.06433304359 | 0.8953059599 |
| fam13a | 0.112318688 | 0.06082314753 | 1.846643796 | 0.0647987824 | 1.118869374 |
| bmper | -0.1128550322 | 0.06115900911 | -1.84527241 | 0.06499792175 | 0.8932801476 |
| prnd | -0.1096765298 | 0.05951714419 | -1.84277205 | 0.06536229922 | 0.8961239578 |
| prdx6 | 0.1035650253 | 0.05623088143 | 1.841782001 | 0.06550704391 | 1.109117912 |
| rgs8 | 0.1040277125 | 0.05649714408 | 1.841291523 | 0.06557884936 | 1.109631205 |
| efemp1 | 0.1179236668 | 0.06404916303 | 1.841142979 | 0.06560060878 | 1.125158221 |
| cfb | -0.1226277777 | 0.06668901162 | -1.838800347 | 0.06594455724 | 0.8845928665 |
| cxcl14 | -0.1223702642 | 0.06662600612 | -1.836674166 | 0.06625801128 | 0.8848206904 |
| igfbp1 | 0.1165298636 | 0.06362499365 | 1.831510809 | 0.06702433588 | 1.123591064 |
| cntf | -0.1113345452 | 0.06081557044 | -1.830691456 | 0.06714660909 | 0.8946394015 |
| lrrn1 | -0.1094174071 | 0.05979255154 | -1.829950458 | 0.06725734734 | 0.896356194 |
| cxcl12 | 0.1056099006 | 0.05773443576 | 1.829235867 | 0.06736428154 | 1.11138824 |
| ssb | 0.1054068362 | 0.05764235675 | 1.828635089 | 0.06745429251 | 1.11116258 |
| sugt1 | 0.1052055601 | 0.05753800003 | 1.828453545 | 0.06748151159 | 1.110938952 |
| nudt10 | -0.1129016769 | 0.06180420235 | -1.826763758 | 0.06773529724 | 0.8932384817 |
| prss8 | -0.1212218816 | 0.06651947639 | -1.822351711 | 0.06840163659 | 0.8858373867 |
| mcts1 | 0.1085580148 | 0.05958281586 | 1.821968519 | 0.06845976251 | 1.114669574 |
| cdk1 | 0.1124425511 | 0.06185853637 | 1.817737012 | 0.06910433787 | 1.119007969 |
| rnf43 | -0.1107991332 | 0.06101397673 | -1.815963146 | 0.06937602558 | 0.8951185305 |
| glrx | 0.104566591 | 0.0576784957 | 1.812921606 | 0.06984391335 | 1.110229323 |
| parp1 | 0.1060368651 | 0.05852279296 | 1.81189003 | 0.07000319018 | 1.111862865 |
| cd207 | 0.1041613811 | 0.05772139708 | 1.804554054 | 0.0711444933 | 1.109779538 |
| il18bp | 0.1082194207 | 0.05997982867 | 1.804263584 | 0.07118999562 | 1.114292217 |
| cntnap2 | -0.1073320057 | 0.05958958883 | -1.801187217 | 0.07167337648 | 0.8982274069 |
| scn3a | -0.1103153532 | 0.06131784215 | -1.799074289 | 0.07200693018 | 0.8955516756 |
| pdcd5 | 0.1042336564 | 0.05805571145 | 1.795407443 | 0.07258880749 | 1.10985975 |
| smpd3 | 0.1112996663 | 0.0620724746 | 1.79305992 | 0.07296334354 | 1.117729803 |
| itga11 | -0.1052287396 | 0.0587549232 | -1.7909774 | 0.0732969217 | 0.9001186063 |
| iglon5 | 0.1097066407 | 0.06126016918 | 1.7908315 | 0.07332033869 | 1.115950648 |
| gbp4 | 0.1040699112 | 0.05815132575 | 1.789639529 | 0.07351187957 | 1.109678031 |
| smoc2 | -0.1128831758 | 0.06311054929 | -1.788657793 | 0.07366994416 | 0.8932550078 |
| prtg | -0.1022348551 | 0.05720693532 | -1.787105962 | 0.07392036414 | 0.9028174954 |

| Protein | beta | se | z | p_val | HR |
|---|---|---|---|---|---|
| necap2 | 0.1060077246 | 0.05938765689 | 1.785012748 | 0.07425924919 | 1.111830465 |
| fes | -0.1044881519 | 0.05857228509 | -1.783917969 | 0.0744369956 | 0.90078547 |
| fam20a | -0.1187145281 | 0.06655454948 | -1.783717702 | 0.07446954815 | 0.8880612811 |
| septin8 | 0.1185221095 | 0.06653387349 | 1.781379969 | 0.07485039837 | 1.125831765 |
| igf2bp3 | 0.1085845242 | 0.06097279689 | 1.78086835 | 0.07493396014 | 1.114699124 |
| mep1a | 0.1150682077 | 0.06461559129 | 1.780811804 | 0.07494320039 | 1.121949961 |
| tbca | 0.1100573844 | 0.0620655889 | 1.773243215 | 0.076188414 | 1.116342129 |
| cfp | -0.1117201469 | 0.0630297673 | -1.772498165 | 0.07631189969 | 0.8942944936 |
| ngrn | -0.111375421 | 0.06293222671 | -1.769767682 | 0.07676584961 | 0.8946028332 |
| rp2 | 0.1022829413 | 0.05790372437 | 1.76643113 | 0.07732354632 | 1.107696841 |
| bcl2l15 | 0.1115030482 | 0.06316514223 | 1.765262362 | 0.07751968201 | 1.117957152 |
| acy1 | -0.115555746 | 0.0655140635 | -1.76383115 | 0.07776041137 | 0.8908709084 |
| igbp1 | 0.1080455282 | 0.06136941318 | 1.760576199 | 0.07831015993 | 1.114098467 |
| ctsc | -0.1019273523 | 0.05789445087 | -1.760572054 | 0.07831086212 | 0.9030951571 |
| septin9 | -0.1010670287 | 0.05743534152 | -1.759666192 | 0.07846442125 | 0.9038724455 |
| snx15 | 0.1094003816 | 0.06219708662 | 1.758930965 | 0.07858923491 | 1.11560893 |
| atrn | -0.1105295396 | 0.06288518964 | -1.757640237 | 0.07880874242 | 0.8953598812 |
| matn3 | 0.1026878229 | 0.05846337114 | 1.756447172 | 0.07901208429 | 1.108145417 |
| susd4 | 0.110850528 | 0.06323399873 | 1.753021005 | 0.07959840171 | 1.1172279 |
| ces3 | -0.102133783 | 0.05827518333 | -1.752611956 | 0.07966863768 | 0.9029087497 |
| ptk7 | 0.1006535338 | 0.05752045462 | 1.749873752 | 0.08014010081 | 1.105893421 |
| gh2 | 0.0990238829 | 0.05662220801 | 1.748852374 | 0.08031654087 | 1.104092668 |
| osbpl2 | -0.1072809976 | 0.06140857912 | -1.747003419 | 0.08063674511 | 0.8982732249 |
| adam12 | -0.1173278238 | 0.06719288512 | -1.746134632 | 0.08078756016 | 0.8892936138 |
| arntl | -0.1067739369 | 0.06126402545 | -1.742848861 | 0.08136001888 | 0.8987288194 |
| sit1 | -0.101534156 | 0.05827577683 | -1.742304634 | 0.08145515306 | 0.9034503206 |
| cd8a | -0.09847945934 | 0.05654394523 | -1.741644644 | 0.08157064442 | 0.9062143067 |
| nop56 | 0.1053635385 | 0.06054259214 | 1.7403209 | 0.08180268609 | 1.11111447 |
| clec4d | 0.1016398921 | 0.05854872067 | 1.735988266 | 0.0825659094 | 1.106984766 |
| adamts1 | 0.109200308 | 0.0630442953 | 1.732120368 | 0.0832521334 | 1.115385749 |
| mmp7 | 0.1029122019 | 0.05943830673 | 1.731412073 | 0.08337829483 | 1.10839409 |
| akap12 | -0.1071131617 | 0.06189699256 | -1.730506723 | 0.08353978119 | 0.898424 |
| dnajb1 | 0.0998332826 | 0.05777096421 | 1.728087526 | 0.08397253359 | 1.104986682 |
| csf1 | 0.1052403538 | 0.06094949192 | 1.726681396 | 0.08422489859 | 1.110977606 |
| dand5 | 0.1053556442 | 0.06113590568 | 1.723302257 | 0.08483387989 | 1.111105698 |
| gc | 0.1065517013 | 0.061949047 | 1.719989354 | 0.0854343767 | 1.112435439 |
| rpe | 0.104766353 | 0.06091309454 | 1.719931548 | 0.08544488515 | 1.110451126 |
| kdm3a | 0.1066794471 | 0.06208201358 | 1.718363193 | 0.08573038959 | 1.112577557 |
| hmgcs1 | -0.1039110131 | 0.06050834757 | -1.71730046 | 0.0859242881 | 0.9013054982 |
| scgb3a2 | 0.1025239928 | 0.05970246721 | 1.717248843 | 0.08593371468 | 1.107963885 |
| elob | 0.1055922475 | 0.06149445286 | 1.717101992 | 0.08596053824 | 1.111368621 |
| tctn3 | -0.1081435734 | 0.06303472477 | -1.71561903 | 0.08623179315 | 0.8974987303 |
| lyve1 | 0.1050560615 | 0.06124730799 | 1.715276393 | 0.08629456456 | 1.11077288 |
| pdgfc | -0.09759759126 | 0.05690136909 | -1.715206379 | 0.08630739571 | 0.9070138206 |
| cc2d1a | 0.1001307629 | 0.05845186293 | 1.713046563 | 0.08670397373 | 1.105315443 |
| nectin4 | 0.09671088322 | 0.05669442493 | 1.705827043 | 0.08804028655 | 1.101541853 |
| adamtsl4 | -0.1090486275 | 0.06396134638 | -1.70491451 | 0.08821036978 | 0.8966868128 |
| ids | -0.09601969839 | 0.05646616564 | -1.700482002 | 0.08904029901 | 0.908446121 |
| tff3 | 0.1054125715 | 0.06210678898 | 1.697279368 | 0.08964385601 | 1.111168952 |
| lpl | 0.1104542863 | 0.06511228339 | 1.696366347 | 0.08981652286 | 1.116785295 |
| serping1 | 0.1069207624 | 0.06306481804 | 1.695410623 | 0.0899975522 | 1.112846072 |
| trem2 | 0.1064981884 | 0.06288410091 | 1.693563029 | 0.09034834844 | 1.112375911 |
| ltbr | 0.09992240073 | 0.0592053135 | 1.687726909 | 0.09146366387 | 1.105085161 |
| psmd5 | 0.1024744796 | 0.06086609808 | 1.683605206 | 0.09225799201 | 1.107909027 |
| ace | -0.1002872542 | 0.05963544259 | -1.681672003 | 0.09263245989 | 0.904577537 |
| dpp6 | -0.1013237797 | 0.0603313274 | -1.679455501 | 0.09306330441 | 0.9036404051 |
| skap1 | -0.09507002579 | 0.05662881777 | -1.678827663 | 0.09318563565 | 0.9093092571 |
| cnp | 0.1035790604 | 0.06180706956 | 1.675844869 | 0.09376858286 | 1.109133478 |
| slc39a5 | -0.1024195706 | 0.06111571143 | -1.675830457 | 0.09377140658 | 0.9026507465 |
| treh | -0.1035410542 | 0.06180510752 | -1.675283133 | 0.09387869311 | 0.9016390059 |
| ccl26 | 0.09501409176 | 0.05675166744 | 1.674207932 | 0.0940897412 | 1.099674351 |
| tmem25 | -0.1034430589 | 0.06182706558 | -1.673103162 | 0.09430698913 | 0.9017273666 |
| lamb1 | -0.1034545758 | 0.06187880348 | -1.671890372 | 0.09454594163 | 0.9017169816 |
| gp1ba | 0.09386145217 | 0.05619219882 | 1.670364466 | 0.09484727561 | 1.098407553 |
| maea | 0.09598791959 | 0.05746963506 | 1.670237152 | 0.09487245216 | 1.100745766 |
| lama1 | -0.1003908173 | 0.06014796869 | -1.669064134 | 0.09510467035 | 0.9044838611 |
| nrp1 | 0.09571687158 | 0.05738106345 | 1.668091629 | 0.09529753875 | 1.100447452 |
| fkbpl | 0.1025775999 | 0.06160070878 | 1.665201618 | 0.09587253834 | 1.108023281 |
| park7 | 0.09600353692 | 0.05765401123 | 1.665166653 | 0.0958795119 | 1.100762957 |
| ing1 | -0.09685896999 | 0.0582600893 | -1.662526974 | 0.09640715949 | 0.9076840078 |

| Protein | beta | se | z | p_val | HR |
|---|---|---|---|---|---|
| hpgds | -0.09545849009 | 0.05746617624 | -1.661124793 | 0.09668838609 | 0.9089560916 |
| atox1 | 0.09590856254 | 0.05775972906 | 1.660474592 | 0.09681901522 | 1.100658418 |
| il18r1 | -0.1013642076 | 0.06121074063 | -1.655987275 | 0.09772439583 | 0.9036038736 |
| kcnh2 | -0.09982222758 | 0.06031521566 | -1.655009047 | 0.09792266278 | 0.9049982875 |
| psapl1 | -0.1010837338 | 0.0611217054 | -1.653810756 | 0.09816596976 | 0.9038573463 |
| lsm1 | 0.09272133519 | 0.05610820332 | 1.652545077 | 0.09842348381 | 1.097155954 |
| tcof1 | -0.1025511457 | 0.06218243368 | -1.649198007 | 0.09910707607 | 0.9025319879 |
| robo2 | -0.09882843645 | 0.05993481513 | -1.648932031 | 0.0991615601 | 0.9058981138 |
| dll1 | 0.0974935195 | 0.05919570346 | 1.646969523 | 0.09956431032 | 1.102404297 |
| dhps | -0.10043391 | 0.06099046115 | -1.646715045 | 0.09961663029 | 0.9044448852 |
| tyro3 | -0.09460791896 | 0.05761160913 | -1.642167619 | 0.1005552715 | 0.9097295523 |
| cemip2 | -0.102572617 | 0.06254227792 | -1.640052465 | 0.1009942589 | 0.9025126096 |
| pacs2 | 0.1003678714 | 0.06128205137 | 1.637802083 | 0.101462987 | 1.105577554 |
| hnrnpul1 | 0.1041419895 | 0.06359822766 | 1.637498298 | 0.1015263943 | 1.109758018 |
| cacna1c | -0.1008147415 | 0.06157966106 | -1.637143494 | 0.1016004905 | 0.9041005097 |
| cit | 0.09996391349 | 0.06108854144 | 1.63637748 | 0.1017606094 | 1.105131037 |
| adam15 | -0.09490282873 | 0.05802115751 | -1.635659005 | 0.1019109739 | 0.9094613037 |
| snapin | -0.1002685971 | 0.06130422949 | -1.635590201 | 0.1019253825 | 0.904594414 |
| caly | -0.09783363546 | 0.05987102162 | -1.63407326 | 0.1022434688 | 0.9067997505 |
| fgl1 | 0.1053351854 | 0.06450648024 | 1.63293959 | 0.1024817025 | 1.111082967 |
| itih4 | 0.1038138131 | 0.06365245994 | 1.630947385 | 0.1029014221 | 1.109393881 |
| sprr3 | -0.1040796862 | 0.06384558582 | -1.630178264 | 0.1030638265 | 0.901153485 |
| yars1 | 0.09864835087 | 0.06071606032 | 1.624748878 | 0.104216078 | 1.103678124 |
| tgfbr3 | 0.09111608424 | 0.05612172576 | 1.623543877 | 0.1044731917 | 1.095396156 |
| sugp1 | 0.09996314006 | 0.06171065546 | 1.619868389 | 0.1052605515 | 1.105130182 |
| hs6st1 | -0.09215870296 | 0.05689802635 | -1.619717043 | 0.1052930735 | 0.9119604072 |
| myl4 | 0.1027847421 | 0.06349009166 | 1.618909965 | 0.1054666365 | 1.108252823 |
| serpina7 | 0.098901872 | 0.06111475791 | 1.618297697 | 0.1055984567 | 1.103957965 |
| cxcl8 | 0.09156619475 | 0.056692964 | 1.615124493 | 0.1062837378 | 1.095889316 |
| cd2ap | 0.09246767374 | 0.05726508983 | 1.614730266 | 0.1063691202 | 1.096877683 |
| apoc1 | 0.105641986 | 0.06544398211 | 1.614235298 | 0.1064763982 | 1.1114239 |
| pon3 | 0.1046185602 | 0.06502276151 | 1.608952892 | 0.1076266428 | 1.110287022 |
| dlk1 | -0.09607650612 | 0.05987107014 | -1.604723381 | 0.108554694 | 0.9083945157 |
| ogt | 0.09631552309 | 0.06004099074 | 1.604162788 | 0.1086781748 | 1.101106434 |
| gid8 | 0.09737097244 | 0.06079905055 | 1.601521266 | 0.1092615136 | 1.102269209 |
| cfhr2 | 0.09551830254 | 0.0596960994 | 1.60007611 | 0.1095817 | 1.100228959 |
| ptgr1 | 0.09933817353 | 0.06212471892 | 1.59901204 | 0.1098179274 | 1.104439729 |
| agrp | -0.0941578953 | 0.05889875384 | -1.598639855 | 0.1099006487 | 0.9101390442 |
| slc4a1 | 0.09834238465 | 0.06153215483 | 1.598227543 | 0.109992346 | 1.103340487 |
| cyth3 | 0.09780008287 | 0.06123083162 | 1.597235907 | 0.1102131316 | 1.102742306 |
| taldo1 | 0.1002037646 | 0.06273875398 | 1.597158985 | 0.1102302727 | 1.105396136 |
| lgals9 | 0.1037539425 | 0.06496390249 | 1.597101444 | 0.1102430964 | 1.109327463 |
| fgf9 | 0.0984580958 | 0.06165810675 | 1.596839426 | 0.110301505 | 1.103468163 |
| tnfaip2 | 0.099867444 | 0.06260437985 | 1.595214971 | 0.110664172 | 1.105024431 |
| tnfrsf11b | 0.1031727432 | 0.06485964433 | 1.590707816 | 0.111675347 | 1.10868291 |
| s100a16 | -0.0922176346 | 0.05802810879 | -1.589189042 | 0.1120177197 | 0.9119066655 |
| aldh3a1 | 0.09312833485 | 0.05877008723 | 1.584621348 | 0.1130523916 | 1.097602587 |
| akt1s1 | 0.09294013209 | 0.05878172457 | 1.581105909 | 0.1138538238 | 1.097396034 |
| clec2l | 0.09667223055 | 0.06115910265 | 1.580667903 | 0.1139539909 | 1.101499277 |
| ciapin1 | 0.09071413476 | 0.05744589961 | 1.579122886 | 0.1143078733 | 1.094955951 |
| nagpa | -0.09953299943 | 0.06327179446 | -1.573102206 | 0.1156951543 | 0.9052600763 |
| ptprc | -0.09647664109 | 0.06152444363 | -1.568102617 | 0.1168571858 | 0.908031108 |
| gja8 | 0.09595893339 | 0.06157854224 | 1.558317718 | 0.119157951 | 1.10071386 |
| ltbp2 | 0.1039063785 | 0.06674194174 | 1.556837811 | 0.1195089975 | 1.109496577 |
| ptrhd1 | 0.09625853874 | 0.06184474458 | 1.556454625 | 0.1196000244 | 1.10104369 |
| pgd | 0.09661440827 | 0.06212835739 | 1.555077461 | 0.1199276222 | 1.101435587 |
| lmod1 | -0.1092415443 | 0.07032816306 | -1.553311498 | 0.1203487346 | 0.8965138435 |
| fam3d | 0.09517244941 | 0.06134157405 | 1.551516258 | 0.1207780143 | 1.099848507 |
| copb2 | -0.09306872284 | 0.06019391705 | -1.546148305 | 0.1220687548 | 0.9111308826 |
| cxcl16 | 0.09095829167 | 0.05886125601 | 1.545299877 | 0.1222737452 | 1.095223324 |
| eda2r | 0.1117223547 | 0.07230620304 | 1.545128218 | 0.1223152527 | 1.118202354 |
| megf9 | -0.09121193941 | 0.05905230627 | -1.544595718 | 0.1224440826 | 0.912824227 |
| thbs4 | -0.09327093526 | 0.06041909393 | -1.543732771 | 0.122653084 | 0.9109466593 |
| dnajc21 | 0.09205137475 | 0.05965742118 | 1.542999562 | 0.1228308822 | 1.096421149 |
| nudt16 | 0.09417785628 | 0.06115989081 | 1.539863055 | 0.1235937381 | 1.098755149 |
| sel1l | -0.100643752 | 0.06540286133 | -1.538827964 | 0.123846301 | 0.9042551146 |
| epha2 | 0.0950434689 | 0.06185307428 | 1.536600565 | 0.1243911541 | 1.099706657 |
| il11 | -0.08880509951 | 0.05784047468 | -1.535345275 | 0.1246990385 | 0.9150238948 |
| aspscr1 | 0.0943012749 | 0.06143266552 | 1.535034726 | 0.1247752982 | 1.098890764 |
| palld | 0.0958348609 | 0.06244152456 | 1.534793738 | 0.1248345014 | 1.100577301 |

| Protein | beta | se | z | p_val | HR |
|---|---|---|---|---|---|
| ctsf | -0.08994106939 | 0.05862635886 | -1.534140464 | 0.1249951004 | 0.9139850454 |
| dcun1d1 | 0.09363443092 | 0.06104465645 | 1.533867768 | 0.125062187 | 1.09815822 |
| gnas | -0.09258610691 | 0.06038538768 | -1.533253498 | 0.1252134073 | 0.911570715 |
| ccl21 | -0.08898551851 | 0.05805106952 | -1.53288336 | 0.1253045965 | 0.914858822 |
| ces1 | -0.09272509342 | 0.06051002995 | -1.532392126 | 0.1254256994 | 0.9114440278 |
| csnk2a1 | 0.09220563235 | 0.06019991904 | 1.531657082 | 0.1256070784 | 1.096590293 |
| smpd1 | -0.08911830146 | 0.05820091323 | -1.531218266 | 0.1257154578 | 0.9147373524 |
| il6st | -0.08881222008 | 0.05803888354 | -1.530219306 | 0.1259624542 | 0.9150173793 |
| pgm2 | 0.09354187715 | 0.06115160733 | 1.529671602 | 0.1260980363 | 1.098056586 |
| itgax | -0.09256520843 | 0.060517164 | -1.529569502 | 0.1261233232 | 0.9115897657 |
| cep20 | 0.08822736548 | 0.05770642596 | 1.528900188 | 0.1262891897 | 1.092236431 |
| lsm8 | 0.09152659992 | 0.06007841575 | 1.523452288 | 0.1276455889 | 1.095845926 |
| klk1 | 0.08723947959 | 0.05732680121 | 1.521792211 | 0.1280611536 | 1.091157958 |
| lyar | -0.0858536608 | 0.05648519498 | -1.519932096 | 0.1285280428 | 0.917728521 |
| pon2 | -0.08830718944 | 0.05810994322 | -1.519657128 | 0.1285971718 | 0.9154796079 |
| btd | -0.09472971891 | 0.06235635232 | -1.5191671 | 0.1287204403 | 0.909618754 |
| ist1 | 0.09317867801 | 0.06139264884 | 1.51774976 | 0.1290774948 | 1.097657845 |
| txn | 0.09262728094 | 0.06133086062 | 1.510288295 | 0.130969877 | 1.097052767 |
| ggt5 | 0.08656109726 | 0.05734880752 | 1.509379201 | 0.1312019052 | 1.090417987 |
| cdan1 | -0.09192233614 | 0.06095385128 | -1.508064449 | 0.1315380329 | 0.9121759899 |
| hrc | -0.09289900906 | 0.06162360024 | -1.507523233 | 0.1316765933 | 0.9112855272 |
| kirrel2 | 0.08978702026 | 0.05965463967 | 1.505113781 | 0.1322948266 | 1.093941272 |
| mmp1 | -0.08518214243 | 0.05666034596 | -1.503381968 | 0.1327405737 | 0.9183449996 |
| gfra3 | 0.08745786586 | 0.05831568153 | 1.499731523 | 0.1336839616 | 1.091396278 |
| ncan | -0.09502604602 | 0.06338586984 | -1.499167658 | 0.1338301431 | 0.9093492492 |
| kitlg | 0.08545623634 | 0.05703124861 | 1.498410756 | 0.1340265636 | 1.089213892 |
| mtss2 | 0.09068406452 | 0.06056418489 | 1.497321638 | 0.1343095873 | 1.094923026 |
| scg3 | 0.08705928942 | 0.05815331832 | 1.497064861 | 0.1343763818 | 1.09096136 |
| cda | 0.09187038944 | 0.06155607773 | 1.49246659 | 0.1355768694 | 1.096222731 |
| penk | -0.09262559252 | 0.06214975059 | -1.490361452 | 0.1361292225 | 0.9115347218 |
| metap2 | 0.08419425238 | 0.05654141419 | 1.489072277 | 0.1364683371 | 1.087840189 |
| atxn2 | -0.09033661281 | 0.06066890006 | -1.48901023 | 0.136484675 | 0.9136235961 |
| enpp5 | -0.09446743929 | 0.06354118059 | -1.486712057 | 0.1370908769 | 0.9098573597 |
| krt14 | -0.08566025646 | 0.0576629863 | -1.485532782 | 0.1374027461 | 0.9179060309 |
| cd34 | -0.08493593219 | 0.05717875549 | -1.485445625 | 0.1374258172 | 0.9185711334 |
| atp6v1f | 0.08611918078 | 0.05797796909 | 1.485377673 | 0.1374438068 | 1.08993622 |
| btnl10 | 0.0914024996 | 0.06155099046 | 1.484988282 | 0.1375469281 | 1.095709939 |
| atg16l1 | 0.09012543667 | 0.06069300741 | 1.484939378 | 0.1375598836 | 1.094311542 |
| ndrg1 | 0.08641468301 | 0.05820913716 | 1.484555299 | 0.1376616643 | 1.090258346 |
| gsap | 0.08276591864 | 0.05579606456 | 1.483364809 | 0.1379775119 | 1.086287499 |
| glp1r | -0.0906281429 | 0.0611062862 | -1.483123072 | 0.1380417151 | 0.9133572862 |
| lair1 | 0.09330840582 | 0.06297421553 | 1.48169223 | 0.1384222055 | 1.097800251 |
| mprip | -0.09116429004 | 0.06153395253 | -1.481528267 | 0.1384658582 | 0.9128677235 |
| evpl | -0.089117723 | 0.06018200362 | -1.480803523 | 0.1386589374 | 0.9147378816 |
| ercc1 | -0.09118886437 | 0.0616014427 | -1.480304038 | 0.1387921262 | 0.9128452907 |
| hdgf | 0.08228260704 | 0.05577804553 | 1.475179101 | 0.1401643986 | 1.085762611 |
| msr1 | 0.09157007932 | 0.06208932521 | 1.474811959 | 0.1402631052 | 1.095893573 |
| ccne1 | 0.09147750882 | 0.06204010778 | 1.474489844 | 0.1403497503 | 1.095792131 |
| dpp10 | -0.08640736921 | 0.05867272789 | -1.47270073 | 0.1408317496 | 0.9172205077 |
| dhodh | 0.08940484014 | 0.06076832152 | 1.471240902 | 0.1412259794 | 1.093523269 |
| prtn3 | 0.0862446227 | 0.05866238676 | 1.47018605 | 0.1415113717 | 1.090072952 |
| sat2 | 0.09101746178 | 0.06191637537 | 1.470006299 | 0.1415600481 | 1.095288131 |
| tp53bp1 | 0.09101142848 | 0.0619129114 | 1.469991096 | 0.1415641656 | 1.095281523 |
| fmr1 | -0.0837722928 | 0.05704049073 | -1.468646075 | 0.1419288109 | 0.919640641 |
| scn3b | -0.09045498828 | 0.06161185586 | -1.468142568 | 0.1420655008 | 0.9135154519 |
| omg | -0.08619520938 | 0.05875291956 | -1.467079594 | 0.1423544045 | 0.9174151257 |
| rbp7 | 0.09673453317 | 0.06596165153 | 1.466526852 | 0.1425048114 | 1.101567905 |
| rln1 | -0.09108760002 | 0.06226662518 | -1.46286393 | 0.1435046156 | 0.912937734 |
| heph | -0.09134666946 | 0.06248139841 | -1.461981834 | 0.1437461879 | 0.9127012504 |
| naprt | 0.08968307912 | 0.06135907504 | 1.461610676 | 0.1438479273 | 1.093827572 |
| bap18 | 0.09105539998 | 0.0623486315 | 1.460423393 | 0.1441737474 | 1.095329685 |
| cdh2 | -0.09309705832 | 0.06396694691 | -1.455393181 | 0.1455604432 | 0.9111050657 |
| furin | -0.09512577983 | 0.065374282 | -1.455094831 | 0.1456430099 | 0.9092585609 |
| pomc | -0.09434674221 | 0.06485224172 | -1.454795389 | 0.1457259151 | 0.9099671835 |
| sele | -0.08782372488 | 0.06050269991 | -1.451567038 | 0.1466220296 | 0.9159223168 |
| tfpi2 | 0.08843007503 | 0.06094065284 | 1.451085128 | 0.1467561572 | 1.09245786 |
| lcn2 | 0.08667595473 | 0.05977560543 | 1.450022197 | 0.1470523294 | 1.090543237 |
| islr2 | -0.08537548798 | 0.05891008997 | -1.44925068 | 0.1472675888 | 0.9181674588 |
| tjp3 | -0.08886598457 | 0.06138113301 | -1.447773611 | 0.1476803753 | 0.9149681852 |
| robo4 | -0.08879408422 | 0.06139036036 | -1.446384802 | 0.1480693022 | 0.9150339741 |

| Protein | beta | se | z | p_val | HR |
|---|---|---|---|---|---|
| ism1 | -0.0854929786 | 0.05933109188 | -1.440947333 | 0.1495995624 | 0.9180595891 |
| pi16 | 0.08664267258 | 0.06020685298 | 1.439083232 | 0.1501269422 | 1.090506942 |
| ranbp1 | 0.08813748686 | 0.06127515476 | 1.438388645 | 0.1503238125 | 1.092138266 |
| morn4 | 0.08856855574 | 0.06164205035 | 1.436820405 | 0.1507690309 | 1.092609155 |
| sirt2 | 0.08270643065 | 0.05759062777 | 1.436109205 | 0.1509712692 | 1.08622288 |
| mthfd2 | -0.08746128812 | 0.06090575899 | -1.43601015 | 0.150999453 | 0.9162543409 |
| zbtb16 | 0.08306362103 | 0.05786713387 | 1.43541965 | 0.15116755 | 1.086610938 |
| rabep1 | 0.0874337044 | 0.0610166342 | 1.432948663 | 0.1518725089 | 1.091369909 |
| rnase6 | -0.09400267716 | 0.06563821775 | -1.432133296 | 0.152105677 | 0.9102803253 |
| vstm1 | -0.08218876146 | 0.05740690611 | -1.431687702 | 0.1522332176 | 0.9210980745 |
| tpsg1 | -0.08721030923 | 0.0609409887 | -1.431061607 | 0.1524125594 | 0.9164843302 |
| mamdc2 | -0.08742014504 | 0.06121976698 | -1.427972522 | 0.1532997677 | 0.9162920392 |
| inhbc | -0.08903173138 | 0.06235921456 | -1.427723745 | 0.1533713887 | 0.9148165447 |
| il15 | 0.08108949214 | 0.05683207509 | 1.426826172 | 0.1536300054 | 1.084467944 |
| pecam1 | -0.08254855869 | 0.0578806244 | -1.426186389 | 0.1538145475 | 0.9207667255 |
| mri1 | 0.08704182111 | 0.06111871617 | 1.424143479 | 0.1544049423 | 1.090942303 |
| fen1 | 0.08124510744 | 0.05709002413 | 1.423105151 | 0.1547056753 | 1.084636717 |
| ccdc50 | -0.08635603571 | 0.06077668038 | -1.420874506 | 0.1553532457 | 0.9172675931 |
| rassf2 | 0.08262532513 | 0.05816656235 | 1.420495243 | 0.1554635524 | 1.086134785 |
| odam | 0.07989708351 | 0.05638296206 | 1.417043032 | 0.1564703466 | 1.083175585 |
| pcdh1 | -0.08350489782 | 0.0591024681 | -1.41288343 | 0.1576900021 | 0.9198865812 |
| nup50 | -0.08608352959 | 0.06092846084 | -1.41286237 | 0.1576961953 | 0.9175175881 |
| wfdc1 | -0.09010887961 | 0.06378984793 | -1.41258966 | 0.157776411 | 0.9138316822 |
| nrcam | -0.08094274495 | 0.05741861864 | -1.409695093 | 0.1586297343 | 0.9222464932 |
| smpdl3b | -0.08386446967 | 0.05949545229 | -1.409594624 | 0.1586594155 | 0.9195558754 |
| entr1 | 0.09116842231 | 0.06467793211 | 1.409575404 | 0.1586650938 | 1.095453489 |
| sh3bp1 | 0.08584808686 | 0.060906097 | 1.409515485 | 0.1586827979 | 1.089640785 |
| clstn3 | -0.08584519283 | 0.06099616587 | -1.407386704 | 0.1593127517 | 0.9177362924 |
| pdia2 | 0.0869250004 | 0.06178455169 | 1.406905092 | 0.1594555334 | 1.090814866 |
| nphs1 | 0.08752705451 | 0.06225723316 | 1.405893742 | 0.1597556798 | 1.091471793 |
| tcn2 | 0.07984034057 | 0.05683248184 | 1.404836424 | 0.1600699252 | 1.083114124 |
| acp1 | 0.08747440082 | 0.06227247591 | 1.404704077 | 0.1601092928 | 1.091414325 |
| sh3gl3 | 0.08408307178 | 0.05990057569 | 1.403710579 | 0.160405051 | 1.087719249 |
| igf2r | -0.08408643488 | 0.05990370675 | -1.403693351 | 0.1604101831 | 0.9193517886 |
| gh1 | 0.08941289346 | 0.0637104247 | 1.403426423 | 0.1604897182 | 1.093532075 |
| chi3l1 | -0.08621841476 | 0.06144272178 | -1.403232348 | 0.1605475644 | 0.917393837 |
| bsnd | -0.08622723004 | 0.0614812141 | -1.402497191 | 0.1607668289 | 0.9173857499 |
| bola2_bola2b | 0.08822454208 | 0.06301783874 | 1.399993142 | 0.1615153721 | 1.092233347 |
| clstn2 | 0.08103032073 | 0.05789888601 | 1.399514331 | 0.1616588038 | 1.084403776 |
| uso1 | 0.0800377746 | 0.0571899116 | 1.399508626 | 0.1616605135 | 1.083327989 |
| f13b | -0.08585144168 | 0.06137194279 | -1.398871174 | 0.161851618 | 0.9177305576 |
| snca | 0.08748866677 | 0.06260663699 | 1.397434377 | 0.1622829875 | 1.091429895 |
| nppc | -0.09160768392 | 0.06557485055 | -1.39699417 | 0.1624153243 | 0.9124630533 |
| erc2 | -0.08460874486 | 0.06059589219 | -1.396278556 | 0.1626306287 | 0.9188717274 |
| pbxip1 | -0.08650732609 | 0.0620769279 | -1.393550374 | 0.1634534233 | 0.9171288298 |
| mif | 0.07909927494 | 0.05687429586 | 1.390773701 | 0.1642940604 | 1.082311763 |
| hdac9 | -0.08529847686 | 0.06148281174 | -1.387354847 | 0.1653335864 | 0.9182381707 |
| plpbp | 0.07947261486 | 0.05732944224 | 1.386244341 | 0.1656723062 | 1.082715909 |
| dennd2b | -0.08412128656 | 0.06073331854 | -1.385092871 | 0.1660240714 | 0.9193197482 |
| anxa2 | 0.08742515847 | 0.06315176265 | 1.384366086 | 0.166246388 | 1.091360582 |
| ifngr2 | -0.08113428909 | 0.05864278938 | -1.383533934 | 0.1665012098 | 0.9220698592 |
| susd1 | -0.07947331081 | 0.05745750974 | -1.383166642 | 0.1666137755 | 0.9236026699 |
| gpr158 | -0.08743047112 | 0.06322637158 | -1.38281652 | 0.1667211323 | 0.9162825775 |
| map1lc3a | -0.08395925042 | 0.06074084231 | -1.382253641 | 0.1668938351 | 0.9194687233 |
| il13ra1 | -0.08355051614 | 0.06046974934 | -1.381691127 | 0.1670665599 | 0.9198446185 |
| ugdh | 0.08355605755 | 0.06063477372 | 1.378022089 | 0.1681964683 | 1.087146156 |
| ngf | -0.07833337318 | 0.05685197969 | -1.377847766 | 0.168250295 | 0.9246561196 |
| apoa4 | 0.08586468672 | 0.06233258871 | 1.377524799 | 0.1683500531 | 1.089658873 |
| nit1 | 0.08588283704 | 0.06239093915 | 1.376527397 | 0.1686584114 | 1.089678651 |
| upb1 | -0.09196061985 | 0.06682097722 | -1.37622381 | 0.1687523528 | 0.9121410691 |
| ggt1 | -0.08927904781 | 0.06489104915 | -1.375829933 | 0.1688742918 | 0.9145903235 |
| il1rn | 0.09225205085 | 0.0670617648 | 1.375628141 | 0.1689367896 | 1.096641197 |
| igfbp6 | 0.09330830603 | 0.06789040348 | 1.374396104 | 0.1693187451 | 1.097800142 |
| pinlyp | -0.08492804898 | 0.06179343122 | -1.37438636 | 0.1693217686 | 0.9185783747 |
| scrib | -0.08572643817 | 0.06257149336 | -1.370055812 | 0.1706694799 | 0.9178452843 |
| pag1 | -0.08137945514 | 0.05954150667 | -1.36676849 | 0.1716978825 | 0.9218438267 |
| igsf3 | -0.08667451644 | 0.06351317274 | -1.364669921 | 0.1723568175 | 0.9169755075 |
| atp1b2 | -0.08229489916 | 0.06031060713 | -1.364517837 | 0.1724046444 | 0.9210003164 |
| pydc1 | -0.08452853789 | 0.06201330933 | -1.363070908 | 0.1728601642 | 0.9189454302 |
| ctbs | -0.08560992219 | 0.06283545465 | -1.362446133 | 0.1730571332 | 0.9179522342 |

| Protein | beta | se | z | p_val | HR |
|---|---|---|---|---|---|
| nptxr | -0.08112077428 | 0.05966452218 | -1.359614915 | 0.1739518157 | 0.9220823209 |
| dcun1d2 | 0.08293937971 | 0.06101440488 | 1.35934096 | 0.1740385703 | 1.086475944 |
| usp25 | 0.08309203515 | 0.06114104197 | 1.359022229 | 0.1741395448 | 1.086641813 |
| rarres2 | -0.08686065103 | 0.06396215543 | -1.358000687 | 0.1744634661 | 0.9168048425 |
| nudt5 | 0.07841041517 | 0.05774174528 | 1.357950211 | 0.1744794834 | 1.081566459 |
| cradd | 0.0780925857 | 0.0575092192 | 1.357914205 | 0.1744909093 | 1.08122276 |
| hhex | -0.08491573137 | 0.062535189 | -1.357887179 | 0.1744994863 | 0.9185896894 |
| sez6 | -0.08526738772 | 0.0628133434 | -1.357472523 | 0.1746311184 | 0.9182667183 |
| htr1b | 0.0815667653 | 0.06017047313 | 1.355594548 | 0.1752282076 | 1.084985655 |
| becn1 | 0.08435409272 | 0.06222767539 | 1.355571973 | 0.1752353942 | 1.088014083 |
| scrg1 | -0.09415045895 | 0.06946980889 | -1.355271599 | 0.1753310403 | 0.9101458124 |
| adamtsl2 | -0.08771610313 | 0.06476047485 | -1.354469734 | 0.1755865631 | 0.9160208953 |
| cmc1 | 0.08179070226 | 0.06040109884 | 1.354126064 | 0.175696162 | 1.08522865 |
| sftpd | -0.07984256929 | 0.05901666326 | -1.352881794 | 0.1760933964 | 0.9232616847 |
| btla | -0.08472632857 | 0.06268012438 | -1.351725597 | 0.1764631133 | 0.9187636894 |
| tnfrsf21 | 0.07834057965 | 0.05799964265 | 1.350707971 | 0.1767889978 | 1.08149093 |
| cfhr4 | 0.08015042125 | 0.05935613868 | 1.350330784 | 0.1769099022 | 1.083450029 |
| spp1 | 0.07973245168 | 0.05911128904 | 1.348853205 | 0.1773841213 | 1.082997275 |
| tnfrsf4 | -0.07704773459 | 0.05720198338 | -1.346941662 | 0.1779990221 | 0.9258456577 |
| il2ra | 0.07740022722 | 0.05747157779 | 1.346756609 | 0.1780586337 | 1.080474425 |
| phldb2 | -0.08234704839 | 0.06115059523 | -1.346627095 | 0.1781003634 | 0.9209522882 |
| il21r | 0.08213630287 | 0.06100566697 | 1.346371689 | 0.1781826766 | 1.085603771 |
| eif5a | -0.0775411123 | 0.05761263198 | -1.345904702 | 0.1783332521 | 0.9253889787 |
| il9 | -0.07984747663 | 0.05940711916 | -1.344072525 | 0.1789249358 | 0.9232571539 |
| cndp1 | -0.07952908162 | 0.05918365373 | -1.343767689 | 0.1790235208 | 0.9235511612 |
| bmp6 | -0.07861144708 | 0.05854793837 | -1.342685144 | 0.1793739471 | 0.9243990326 |
| sod2 | -0.0771598681 | 0.05748155 | -1.342341466 | 0.1794853043 | 0.9257418452 |
| plb1 | 0.08770595289 | 0.06535977601 | 1.341894943 | 0.1796300617 | 1.091667073 |
| dxo | 0.08139434524 | 0.06075150693 | 1.339791379 | 0.1803131792 | 1.084798597 |
| dgkz | -0.07615155758 | 0.0569043244 | -1.338238497 | 0.1808187034 | 0.9266757512 |
| fgf5 | 0.08292127452 | 0.06197222733 | 1.338039281 | 0.1808836322 | 1.086456273 |
| tcn1 | -0.08161324851 | 0.06116400852 | -1.334334529 | 0.1820942462 | 0.9216283309 |
| fh | -0.08100956492 | 0.06079089487 | -1.332593723 | 0.1826651662 | 0.9221848708 |
| kdr | -0.07868930306 | 0.05907271963 | -1.33207517 | 0.1828354885 | 0.9243270654 |
| ahsg | -0.08016707187 | 0.0602127136 | -1.331397758 | 0.1830581668 | 0.9229621325 |
| rab2b | 0.08022830522 | 0.06028373803 | 1.330844899 | 0.183240051 | 1.083534416 |
| flt1 | 0.07768945936 | 0.05842664788 | 1.329692224 | 0.183619698 | 1.080786978 |
| c2 | -0.07846742667 | 0.05911484111 | -1.327372707 | 0.1843854234 | 0.9245321745 |
| timp2 | -0.08148422134 | 0.06140060582 | -1.327091488 | 0.1844784207 | 0.9217472537 |
| atp1b4 | 0.08197494003 | 0.06182949394 | 1.325822594 | 0.1848984673 | 1.085428609 |
| tsnax | 0.08249666985 | 0.06224274721 | 1.325402132 | 0.1850378103 | 1.085995057 |
| zbp1 | 0.0829353835 | 0.0626036523 | 1.32476909 | 0.1852477497 | 1.086471602 |
| icam1 | -0.07939979299 | 0.05993912384 | -1.324673901 | 0.1852793332 | 0.9236705736 |
| stx1b | -0.08098070504 | 0.06116497861 | -1.323971771 | 0.1855124201 | 0.9222114853 |
| ptx3 | 0.07550219552 | 0.05705877856 | 1.323235397 | 0.1857571079 | 1.078425595 |
| ceacam5 | 0.0781020706 | 0.05903227459 | 1.32304017 | 0.1858220196 | 1.081233015 |
| stip1 | 0.0754250813 | 0.05734084344 | 1.315381441 | 0.1883817395 | 1.078342437 |
| znrf4 | -0.079866811 | 0.06078300754 | -1.313966094 | 0.1888576147 | 0.9232393035 |
| bcat1 | -0.08277544883 | 0.06307870173 | -1.312256698 | 0.1894335373 | 0.9205578364 |
| cd7 | 0.08138617701 | 0.06203661217 | 1.311905569 | 0.1895519981 | 1.084789737 |
| clic5 | -0.08015217557 | 0.06112406996 | -1.311302988 | 0.1897554193 | 0.9229758813 |
| cd4 | -0.07815618186 | 0.05960805824 | -1.311168056 | 0.189800992 | 0.9248199752 |
| ccar2 | 0.07871393083 | 0.06005792825 | 1.310633469 | 0.1899816261 | 1.081894781 |
| polr2f | 0.08063431026 | 0.06158026004 | 1.309418151 | 0.1903927463 | 1.083974426 |
| ptprf | 0.07663045694 | 0.05854555672 | 1.308903036 | 0.1905671981 | 1.079643028 |
| palm3 | 0.07935513117 | 0.06066552991 | 1.308076123 | 0.1908474912 | 1.082588715 |
| itgb1bp1 | -0.07332185161 | 0.05611349592 | -1.306670533 | 0.1913246306 | 0.9293016846 |
| vgf | -0.08183294077 | 0.06282941662 | -1.302462209 | 0.1927584286 | 0.9214258785 |
| epha1 | -0.08289224373 | 0.06380325308 | -1.299185225 | 0.1938803706 | 0.9204503262 |
| tbr1 | -0.07886233311 | 0.06072130826 | -1.298758794 | 0.1940267198 | 0.9241671429 |
| cbx2 | -0.07918525512 | 0.06098308714 | -1.298478953 | 0.1941228042 | 0.9238687572 |
| tnfrsf1b | 0.07894642487 | 0.0608529477 | 1.297331154 | 0.1945172695 | 1.082146344 |
| ephx2 | -0.07631643204 | 0.05886618584 | -1.29643922 | 0.1948242074 | 0.9265229786 |
| slurp1 | -0.08093924308 | 0.06244546855 | -1.296158792 | 0.1949207833 | 0.9222497228 |
| maged1 | 0.07299224457 | 0.056343995 | 1.295475136 | 0.1951563733 | 1.075722194 |
| prg3 | 0.07805184396 | 0.0602705033 | 1.295025588 | 0.1953114027 | 1.08117871 |
| insr | 0.07994819977 | 0.06173803008 | 1.29495871 | 0.1953344737 | 1.083230955 |
| amn | -0.07521250159 | 0.05808258533 | -1.294923447 | 0.1953466392 | 0.9275463603 |
| urod | 0.07971241396 | 0.06163593871 | 1.293278169 | 0.1959148684 | 1.082975574 |
| vnn2 | 0.0740317229 | 0.05725165024 | 1.293093257 | 0.1959788069 | 1.076840965 |

| Protein | beta | se | z | p_val | HR |
|---|---|---|---|---|---|
| actn4 | 0.07407244613 | 0.05731465672 | 1.29238227 | 0.1962247938 | 1.076884819 |
| efhd1 | -0.0844384342 | 0.06544955603 | -1.290129977 | 0.1970055334 | 0.9190282343 |
| vat1 | 0.07429528944 | 0.05772642285 | 1.287023962 | 0.1980859364 | 1.077124822 |
| ctsl | 0.07470932061 | 0.05821962174 | 1.283232669 | 0.1994105737 | 1.077570878 |
| epcam | -0.07307871504 | 0.05700329191 | -1.28200868 | 0.1998396012 | 0.9295276593 |
| pqbp1 | -0.07403751437 | 0.05775553949 | -1.281911917 | 0.1998735469 | 0.928636856 |
| ogfr | 0.07587645495 | 0.05933931941 | 1.278687651 | 0.20100707 | 1.078829282 |
| ptn | -0.07901384451 | 0.06197093242 | -1.275014615 | 0.2023040692 | 0.9240271317 |
| cbs | -0.08164825519 | 0.06404129637 | -1.274931331 | 0.2023335485 | 0.9215960683 |
| krt6c | 0.07802438693 | 0.06121859477 | 1.274521038 | 0.2024788217 | 1.081149024 |
| calcb | 0.07970633809 | 0.0625566083 | 1.274147372 | 0.2026111924 | 1.082968994 |
| acvrl1 | 0.07902915469 | 0.06205302066 | 1.27357466 | 0.2028141975 | 1.082235874 |
| spink4 | 0.07445118481 | 0.05860644136 | 1.270358396 | 0.203956996 | 1.077292754 |
| cbln1 | -0.0765117621 | 0.06023559919 | -1.270208367 | 0.2040104179 | 0.9263420185 |
| bola1 | 0.07864616086 | 0.06207566178 | 1.266940353 | 0.2051766183 | 1.081821464 |
| mllt1 | -0.07944999948 | 0.06271409547 | -1.266860327 | 0.2052052367 | 0.9236242005 |
| gbp1 | -0.07801408663 | 0.06169524527 | -1.264507277 | 0.2060480103 | 0.924951397 |
| jpt2 | 0.07673057669 | 0.06071837295 | 1.263712662 | 0.2063331789 | 1.079751127 |
| cdh23 | -0.07731060584 | 0.06122047294 | -1.262822747 | 0.2066528878 | 0.9256023115 |
| il24 | -0.07166145521 | 0.05675054709 | -1.262744747 | 0.2066809274 | 0.9308459755 |
| ada | -0.07304666609 | 0.05790617115 | -1.261466 | 0.2071410024 | 0.9295574502 |
| adgre2 | 0.0749695534 | 0.05943916439 | 1.261282089 | 0.2072072321 | 1.077851333 |
| faslg | -0.07593265632 | 0.06031270694 | -1.258982728 | 0.2080365705 | 0.9268786239 |
| gkn1 | 0.07354293184 | 0.05866424504 | 1.253624449 | 0.2099785405 | 1.076314744 |
| scarb2 | 0.0784909272 | 0.06272011396 | 1.251447458 | 0.2107712717 | 1.081653541 |
| il10ra | -0.07193459837 | 0.05748355561 | -1.251394379 | 0.2107906271 | 0.930591756 |
| lrp1 | 0.07646359002 | 0.06116022746 | 1.250217555 | 0.2112200858 | 1.079462886 |
| c1qtnf6 | -0.07653970006 | 0.0612238252 | -1.250162005 | 0.2112403732 | 0.9263161388 |
| ncr3lg1 | 0.07955080764 | 0.06363859661 | 1.250040257 | 0.211284842 | 1.082800573 |
| tsc1 | 0.07764724355 | 0.06217812257 | 1.248787199 | 0.2117429171 | 1.080741353 |
| defb116 | -0.07636410636 | 0.06117960486 | -1.248195482 | 0.2119594782 | 0.9264788083 |
| ncf2 | 0.07150595219 | 0.05730651711 | 1.247780458 | 0.212111467 | 1.074124544 |
| gla | -0.08302207108 | 0.06654646042 | -1.247580571 | 0.2121846973 | 0.9203308343 |
| sdc4 | -0.07052288375 | 0.05655225496 | -1.247039288 | 0.2123830923 | 0.9319064138 |
| acrv1 | -0.1191460143 | 0.0955562548 | -1.246867769 | 0.2124459864 | 0.8876781776 |
| nptx2 | -0.09098159956 | 0.07300895035 | -1.246170492 | 0.2127018098 | 0.913034511 |
| cend1 | 0.0774135238 | 0.06216486913 | 1.245293763 | 0.2130237875 | 1.080488792 |
| rarres1 | 0.07219752348 | 0.05801934841 | 1.244369774 | 0.213363502 | 1.074867635 |
| qdpr | 0.07198450543 | 0.05796053591 | 1.241957209 | 0.2142523504 | 1.074638693 |
| cd82 | -0.07410006374 | 0.05966999203 | -1.241831299 | 0.2142988119 | 0.9285787721 |
| arhgef10 | 0.07470048735 | 0.06022401064 | 1.240377161 | 0.2148359245 | 1.077561359 |
| pafah1b3 | 0.07533029959 | 0.06080893827 | 1.238803073 | 0.2154184356 | 1.078240234 |
| gsta3 | -0.07686988772 | 0.06210770447 | -1.237686828 | 0.2158322052 | 0.9260103311 |
| il1rap | -0.07301665491 | 0.05900160304 | -1.237536798 | 0.2158878618 | 0.9295853477 |
| apex1 | 0.07066530908 | 0.05720313018 | 1.235339899 | 0.2167040303 | 1.073221968 |
| fam171a2 | -0.07389073673 | 0.05984780102 | -1.234644139 | 0.2169629743 | 0.9287731691 |
| tnfrsf10a | 0.07620277395 | 0.06178665459 | 1.233320924 | 0.2174560542 | 1.079181382 |
| pla2g15 | -0.07190557385 | 0.05831162304 | -1.233125921 | 0.2175287881 | 0.9306187664 |
| plxnb2 | -0.07272890097 | 0.05899924308 | -1.232709051 | 0.2176843336 | 0.9298528781 |
| stc2 | -0.07627358949 | 0.0619494748 | -1.231222536 | 0.2182396448 | 0.9265626741 |
| mmp10 | 0.07007288302 | 0.05695718353 | 1.230272964 | 0.2185949048 | 1.072586352 |
| col28a1 | -0.07778753011 | 0.06325769598 | -1.22969275 | 0.2188121829 | 0.9251609745 |
| hbegf | -0.06990688759 | 0.05689683561 | -1.228660379 | 0.2191991673 | 0.9324806414 |
| tprkb | 0.07526284187 | 0.06130440638 | 1.227690574 | 0.2195631461 | 1.078167501 |
| pla2g2a | 0.07439131539 | 0.06065479347 | 1.226470508 | 0.2200216668 | 1.077228259 |
| meltf | 0.07559975954 | 0.06169359411 | 1.225406959 | 0.2204219263 | 1.078530816 |
| c19orf12 | -0.07454317378 | 0.06085006525 | -1.225030302 | 0.2205638038 | 0.9281674007 |
| bglap | -0.07318230883 | 0.05976227593 | -1.224556925 | 0.2207422061 | 0.929431371 |
| fcrl2 | -0.06830489474 | 0.05580418332 | -1.224010293 | 0.2209483448 | 0.9339756659 |
| dnpep | 0.07609134259 | 0.06217796673 | 1.22376698 | 0.2210401439 | 1.079061134 |
| lrfn2 | -0.07477195147 | 0.06111301967 | -1.223502813 | 0.2211398424 | 0.927955081 |
| glb1 | -0.07356569384 | 0.06014622667 | -1.223114032 | 0.2212866293 | 0.9290751093 |
| csf3 | 0.07227226614 | 0.05911105001 | 1.222652383 | 0.2214610192 | 1.074947976 |
| dbh | -0.07532378555 | 0.06160872152 | -1.222615625 | 0.2214749088 | 0.927443145 |
| cenpj | 0.07423716769 | 0.06073737806 | 1.222264939 | 0.2216074534 | 1.07706222 |
| cspg5 | -0.07455999059 | 0.06101425719 | -1.222009314 | 0.2217041047 | 0.928151792 |
| mmp9 | 0.07287988308 | 0.05968439772 | 1.221087686 | 0.2220528217 | 1.075601331 |
| traf3ip2 | -0.07442887042 | 0.06098302677 | -1.220485016 | 0.2222810662 | 0.9282734994 |
| spesp1 | -0.07940804929 | 0.06512477788 | -1.219321614 | 0.2227221481 | 0.9236629475 |
| mslnl | -0.07380770639 | 0.06065417906 | -1.216861023 | 0.2236570975 | 0.9288502886 |

| Protein | beta | se | z | p_val | HR |
|---|---|---|---|---|---|
| rrp15 | 0.07632702039 | 0.06288702233 | 1.21371656 | 0.2248559786 | 1.079315475 |
| chmp1a | 0.06852844683 | 0.05650353128 | 1.212817062 | 0.2251997705 | 1.070931089 |
| nfx1 | 0.0737818956 | 0.06102109439 | 1.209121146 | 0.2266163063 | 1.076571975 |
| pdia3 | -0.07508554456 | 0.06211905608 | -1.208736084 | 0.2267642542 | 0.9276641263 |
| myom2 | 0.07521822484 | 0.06236518959 | 1.2060931 | 0.2277815951 | 1.078119398 |
| oga | 0.07373989668 | 0.06120106662 | 1.204879273 | 0.2282499111 | 1.076526761 |
| mia | -0.06921420622 | 0.05761675382 | -1.201286113 | 0.2296402356 | 0.9331267771 |
| cd300c | 0.06980271253 | 0.05813173688 | 1.200767709 | 0.2298413209 | 1.07229661 |
| tada3 | 0.07368643158 | 0.06137535302 | 1.200586684 | 0.2299115688 | 1.076469206 |
| stat5b | 0.06850343383 | 0.05714325457 | 1.198801754 | 0.2306050403 | 1.070904302 |
| grhpr | 0.07360232961 | 0.06140868628 | 1.198565448 | 0.2306969599 | 1.076378676 |
| fcrl5 | -0.07043908204 | 0.05882132725 | -1.197509225 | 0.2311081339 | 0.9319845124 |
| plat | -0.07888390213 | 0.06590643495 | -1.196907437 | 0.2313426352 | 0.9241472097 |
| icam3 | -0.06785697091 | 0.05671679532 | -1.196417578 | 0.231533645 | 0.9343941096 |
| bsg | -0.0695348253 | 0.0582373149 | -1.193990922 | 0.2324815192 | 0.9328276468 |
| gba | -0.07341237 | 0.06152165124 | -1.193276977 | 0.2327609161 | 0.9292175696 |
| podxl | 0.06781503633 | 0.05694342259 | 1.19091957 | 0.2336851611 | 1.070167348 |
| ruvbl1 | 0.06667668469 | 0.05602193207 | 1.190188953 | 0.2339721341 | 1.068949814 |
| cpq | -0.07308571982 | 0.06140706226 | -1.190184274 | 0.2339739727 | 0.9295211482 |
| met | -0.07128829433 | 0.05991469012 | -1.189829976 | 0.2341132251 | 0.9311933956 |
| sv2a | -0.07276210062 | 0.06115832574 | -1.189733364 | 0.2341512075 | 0.9298220078 |
| kel | 0.07001041844 | 0.05885541808 | 1.189532259 | 0.2342302845 | 1.072519355 |
| tnfrsf19 | 0.07114141217 | 0.05981634316 | 1.189330681 | 0.2343095665 | 1.073733054 |
| nampt | 0.06790215845 | 0.05709349365 | 1.189315176 | 0.2343156656 | 1.070260587 |
| crnn | -0.07206945519 | 0.06064976295 | -1.188289149 | 0.2347195113 | 0.9304662679 |
| lrtm2 | -0.07813468886 | 0.06576051704 | -1.188170233 | 0.2347663486 | 0.9248398525 |
| osm | 0.07085783094 | 0.05967334111 | 1.187428584 | 0.2350586107 | 1.073428607 |
| cd164 | -0.06899737522 | 0.05813705143 | -1.186805549 | 0.2353043297 | 0.9333291299 |
| ctse | 0.07406620842 | 0.06250646777 | 1.184936712 | 0.2360424706 | 1.076878102 |
| ror1 | 0.06858314237 | 0.05790903033 | 1.184325518 | 0.2362842311 | 1.070989666 |
| rpl14 | -0.0754698915 | 0.06377412122 | -1.183393672 | 0.236653164 | 0.92730765 |
| gmpr | 0.06746564954 | 0.05704438163 | 1.182687017 | 0.2369332116 | 1.069793511 |
| fgf21 | 0.0721381 | 0.06103318519 | 1.1819488 | 0.237226017 | 1.074803764 |
| casp8 | 0.06791398362 | 0.0575101817 | 1.180903652 | 0.2376410002 | 1.070273243 |
| spink6 | -0.07071957009 | 0.05990410985 | -1.180546214 | 0.2377830412 | 0.9317231385 |
| tff2 | 0.07326132779 | 0.0620631734 | 1.180431547 | 0.237828621 | 1.076011692 |
| sumf2 | -0.06773342957 | 0.05741574784 | -1.179701251 | 0.238119056 | 0.934509553 |
| prr5 | 0.07209094739 | 0.06112274279 | 1.179445557 | 0.2382208038 | 1.074753086 |
| adipoq | 0.08496858232 | 0.07210621306 | 1.178380873 | 0.2386447993 | 1.088682862 |
| prss53 | -0.07230230591 | 0.06136280437 | -1.178275776 | 0.2386866819 | 0.9302496333 |
| adamts16 | 0.07581614682 | 0.06441391204 | 1.177015095 | 0.2391894812 | 1.078764222 |
| casp4 | 0.070534593 | 0.05992972915 | 1.176954977 | 0.2392134769 | 1.07308169 |
| umod | 0.0707643142 | 0.06014071702 | 1.176645669 | 0.2393369617 | 1.073328228 |
| ckb | 0.08070368784 | 0.06866583704 | 1.175310625 | 0.2398704653 | 1.084049632 |
| acsl1 | 0.07092894567 | 0.06038184676 | 1.174673341 | 0.2401254289 | 1.073504946 |
| fcer2 | -0.0672899069 | 0.05738278981 | -1.172649624 | 0.2409363406 | 0.9349241211 |
| fgfr2 | -0.07273829668 | 0.06205396712 | -1.172178026 | 0.2411255888 | 0.9298441415 |
| lacrt | -0.07331655604 | 0.06255288546 | -1.172073127 | 0.2411676981 | 0.9293066058 |
| tgfbr1 | 0.07232798446 | 0.06171599656 | 1.171948741 | 0.2412176366 | 1.075007872 |
| casc3 | 0.0729199797 | 0.06228174766 | 1.170808181 | 0.2416758886 | 1.07564446 |
| asgr1 | -0.0749230937 | 0.06403007221 | -1.170123523 | 0.2419512633 | 0.9278148384 |
| slc27a4 | -0.0678786543 | 0.05803351091 | -1.169645834 | 0.2421435242 | 0.934373849 |
| serpini1 | 0.074172764 | 0.0635182498 | 1.167739417 | 0.242911892 | 1.076992855 |
| elavl4 | 0.07379875743 | 0.06320775843 | 1.167558529 | 0.2429848867 | 1.076590128 |
| mxra8 | 0.07982602656 | 0.06850651555 | 1.165232619 | 0.2439248452 | 1.083098621 |
| col3a1 | -0.07102273601 | 0.06108267201 | -1.162731323 | 0.244938529 | 0.9314407146 |
| klrb1 | -0.06622134242 | 0.05695605735 | -1.16267427 | 0.2449616847 | 0.9359236818 |
| rnf31 | 0.07196922527 | 0.06190902699 | 1.162499699 | 0.2450325467 | 1.074622272 |
| hao1 | -0.07097001666 | 0.06121008531 | -1.159449726 | 0.2462729176 | 0.9314898209 |
| rps10 | -0.07033506862 | 0.06072906573 | -1.158178012 | 0.2467913994 | 0.9320814563 |
| ocln | -0.07664199972 | 0.0662275325 | -1.157252835 | 0.2471690773 | 0.9262213818 |
| ermap | 0.07666467838 | 0.0663668203 | 1.15516576 | 0.2480225535 | 1.079679976 |
| ube2b | 0.07025539555 | 0.06084831605 | 1.154598847 | 0.248254739 | 1.07278213 |
| pigr | -0.07024749082 | 0.06085219728 | -1.154395305 | 0.2483381393 | 0.9321630896 |
| ppl | 0.0739550231 | 0.06413825283 | 1.153056403 | 0.2488872345 | 1.076758375 |
| slitrk2 | -0.06692273879 | 0.05813572622 | -1.151146518 | 0.2496719634 | 0.9352674584 |
| cep164 | -0.06524004936 | 0.05668616477 | -1.150898983 | 0.2497737964 | 0.9368425479 |
| tspan8 | -0.07046480614 | 0.06130453086 | -1.149422484 | 0.2503818133 | 0.9319605382 |
| igfbp3 | -0.06743259645 | 0.05871896229 | -1.148395575 | 0.2508052997 | 0.9347907267 |
| muc16 | -0.06521960901 | 0.05686331286 | -1.146954086 | 0.2514005975 | 0.9368616975 |

| Protein | beta | se | z | p_val | HR |
|---|---|---|---|---|---|
| pnma2 | -0.06876458446 | 0.05996763237 | -1.146695004 | 0.2515076959 | 0.9335464256 |
| ccl7 | 0.06968720896 | 0.06081526352 | 1.145883532 | 0.2518433461 | 1.072172763 |
| ppm1f | 0.06965108422 | 0.0608664002 | 1.144327313 | 0.25248792 | 1.072134032 |
| col4a1 | -0.06682324927 | 0.05845595913 | -1.14313836 | 0.2529811496 | 0.9353605124 |
| htr1a | -0.06868208311 | 0.06009586537 | -1.142875349 | 0.2530903487 | 0.9336234476 |
| rac3 | -0.06998915668 | 0.06126734053 | -1.142356696 | 0.2533057836 | 0.9324039302 |
| pglyrp1 | 0.06532915857 | 0.05722625028 | 1.141594255 | 0.253622713 | 1.067510347 |
| smad2 | 0.06941233217 | 0.06083845464 | 1.140928588 | 0.2538996415 | 1.071878088 |
| lilrb1 | 0.06615916509 | 0.05798718404 | 1.140927365 | 0.2539001506 | 1.068396755 |
| mmp3 | 0.08221218225 | 0.07221784962 | 1.138391446 | 0.2549570643 | 1.085686149 |
| cd5l | -0.0674674787 | 0.0592804056 | -1.138107576 | 0.2550755652 | 0.9347581197 |
| sf3b4 | 0.06550804565 | 0.05760861911 | 1.137122303 | 0.2554871615 | 1.067701328 |
| brsk2 | -0.06898152341 | 0.06070943097 | -1.136257124 | 0.2558489696 | 0.9333439249 |
| fhit | 0.06613307668 | 0.05825563181 | 1.135222031 | 0.2562823008 | 1.068368883 |
| ifi30 | 0.07829871481 | 0.06919285914 | 1.13160109 | 0.2578021817 | 1.081445654 |
| creb3 | -0.06958255976 | 0.06156394217 | -1.130248605 | 0.2583714842 | 0.9327831199 |
| mtus1 | 0.07096897895 | 0.0628794143 | 1.128652036 | 0.2590446504 | 1.073547923 |
| paxx | 0.0719178601 | 0.06376396321 | 1.127876256 | 0.2593721827 | 1.074567076 |
| abo | -0.06806942244 | 0.06051480767 | -1.124839111 | 0.2606572178 | 0.9341956172 |
| pla2g10 | 0.0675573388 | 0.06007813443 | 1.124491288 | 0.2608046641 | 1.069891604 |
| gzmb | -0.06575835772 | 0.05848254995 | -1.124409893 | 0.2608391771 | 0.9363571004 |
| pvr | 0.06445928955 | 0.05732784455 | 1.124397578 | 0.2608443991 | 1.066582156 |
| gadd45b | -0.06823657571 | 0.06075732099 | -1.123100469 | 0.2613948269 | 0.9340394764 |
| cd109 | -0.06366898677 | 0.05672200866 | -1.12247412 | 0.2616609053 | 0.938315543 |
| bcan | -0.06911564408 | 0.06164959238 | -1.12110464 | 0.2622433238 | 0.9332187526 |
| plxdc1 | -0.06500931848 | 0.05806671492 | -1.119562534 | 0.2629002289 | 0.9370587314 |
| fxyd5 | -0.06414667203 | 0.05741066657 | -1.117330208 | 0.2638531639 | 0.9378674305 |
| mgmt | 0.06387757806 | 0.05719420353 | 1.116854054 | 0.2640567327 | 1.065961894 |
| klk13 | 0.06390009054 | 0.05724246316 | 1.116305746 | 0.2642912829 | 1.065985891 |
| fabp3 | -0.08034459668 | 0.07198088384 | -1.116193528 | 0.2643393042 | 0.9227982984 |
| itih5 | -0.06686825814 | 0.0599963895 | -1.114538036 | 0.2650484362 | 0.9353184138 |
| lect2 | -0.07794100682 | 0.06994064858 | -1.114387819 | 0.2651128468 | 0.9250189947 |
| gpha2 | 0.07164328137 | 0.06433476781 | 1.113601305 | 0.2654502665 | 1.074272063 |
| hgs | 0.06332739681 | 0.0568909479 | 1.113136609 | 0.265649763 | 1.065375583 |
| cd300lg | 0.06529449981 | 0.05874658075 | 1.111460428 | 0.266370214 | 1.067473349 |
| epha4 | -0.06787601611 | 0.06110304449 | -1.110845076 | 0.2666350401 | 0.934376314 |
| dars1 | 0.06293872056 | 0.05676866362 | 1.108687726 | 0.2675649188 | 1.064961577 |
| robo1 | -0.0656255138 | 0.05921671708 | -1.10822614 | 0.2677641643 | 0.936481498 |
| chmp6 | 0.06726633615 | 0.06075854125 | 1.107109137 | 0.2682467458 | 1.069580308 |
| l3hypdh | 0.06655499897 | 0.06018196572 | 1.105896063 | 0.2687715089 | 1.068819746 |
| tnfsf8 | -0.06636088384 | 0.06000720452 | -1.105881942 | 0.268777622 | 0.9357930907 |
| mag | -0.06755038261 | 0.06124367345 | -1.102977317 | 0.2700370159 | 0.9346806278 |
| cd99l2 | -0.06657201325 | 0.06040906797 | -1.102020201 | 0.2704528892 | 0.9355955382 |
| pon1 | -0.0669447374 | 0.06074770657 | -1.102012589 | 0.2704561986 | 0.9352468841 |
| thtpa | 0.06666303526 | 0.06051540969 | 1.101587771 | 0.2706409273 | 1.068935224 |
| c4bpb | 0.06441469617 | 0.05849684576 | 1.101165291 | 0.270824725 | 1.066534595 |
| csf1r | 0.06829245133 | 0.06206091175 | 1.100410055 | 0.2711534993 | 1.070678384 |
| cdsn | -0.06273488126 | 0.05702538053 | -1.100122098 | 0.2712789269 | 0.9391924382 |
| anp32c | -0.06683541771 | 0.0607956959 | -1.099344562 | 0.2716178018 | 0.9353491306 |
| echdc3 | -0.06950695708 | 0.06322992318 | -1.099273154 | 0.2716489384 | 0.9328536434 |
| abl1 | 0.06373187702 | 0.05810619238 | 1.0968173 | 0.2727212662 | 1.065806593 |
| pcsk7 | 0.06648441787 | 0.06069186222 | 1.095442048 | 0.2733230212 | 1.068744311 |
| dsc2 | 0.06616768495 | 0.06042234361 | 1.09508637 | 0.2734787993 | 1.068405858 |
| pgf | -0.07327131868 | 0.06697728166 | -1.093972715 | 0.273966945 | 0.9293486462 |
| stxbp3 | 0.06264712347 | 0.05728186556 | 1.093664161 | 0.2741022979 | 1.064651083 |
| snx9 | 0.06268997733 | 0.05733517175 | 1.093394777 | 0.2742205058 | 1.064696708 |
| ccl2 | 0.06258402002 | 0.05726535516 | 1.092877532 | 0.2744475743 | 1.064583902 |
| tigar | 0.06325191131 | 0.05801513715 | 1.090265652 | 0.2755961411 | 1.065295165 |
| ephb6 | 0.0617321246 | 0.05676203875 | 1.087560031 | 0.2767893838 | 1.063677374 |
| ccdc28a | -0.06709859194 | 0.06170200213 | -1.087462151 | 0.276832617 | 0.9351030032 |
| cd3e | -0.0664148751 | 0.06112377304 | -1.086563734 | 0.2772296596 | 0.9357425675 |
| ccl15 | -0.0652927153 | 0.06010133544 | -1.086377113 | 0.2773121829 | 0.9367932095 |
| cebpb | 0.06423252377 | 0.05912676628 | 1.086352727 | 0.2773229679 | 1.066340319 |
| akr1b1 | 0.06255513969 | 0.05759905038 | 1.086044636 | 0.2774592449 | 1.064553156 |
| unc5d | 0.06754815188 | 0.06220690454 | 1.085862613 | 0.2775397801 | 1.069881775 |
| amot | 0.07567837985 | 0.06973929222 | 1.085161283 | 0.2778502285 | 1.078615614 |
| smad5 | -0.06500682361 | 0.05997436404 | -1.083910178 | 0.2784046264 | 0.9370610692 |
| mfap5 | 0.06790222565 | 0.06267449007 | 1.083410899 | 0.27862608 | 1.070260659 |
| spock1 | -0.06274263578 | 0.05794496569 | -1.082797013 | 0.2788985316 | 0.9391851552 |
| rnf168 | 0.06622358469 | 0.06117735516 | 1.082485252 | 0.2790369653 | 1.068465583 |

| Protein | beta | se | z | p_val | HR |
|---|---|---|---|---|---|
| slitrk1 | 0.07430651478 | 0.0686775649 | 1.08196199 | 0.2792694179 | 1.077136913 |
| gngt1 | 0.06617644718 | 0.06118060305 | 1.081657321 | 0.2794048241 | 1.068415219 |
| pi3 | 0.0658010581 | 0.0608527788 | 1.081315585 | 0.2795567571 | 1.068014223 |
| dctpp1 | 0.06273034725 | 0.05811143192 | 1.079483764 | 0.2803721279 | 1.064739691 |
| unc79 | 0.06598728249 | 0.06113727016 | 1.07932988 | 0.2804406971 | 1.068213132 |
| ren | 0.06246341403 | 0.05794418894 | 1.077992723 | 0.2810370036 | 1.064455514 |
| cd33 | 0.06046392233 | 0.05624497328 | 1.075010242 | 0.282370143 | 1.062329271 |
| mcemp1 | -0.06656710208 | 0.06192736085 | -1.074922315 | 0.2824095103 | 0.935600133 |
| fkbp4 | 0.06184354899 | 0.05766238061 | 1.0725112 | 0.2834904863 | 1.0637959 |
| med18 | 0.06152477314 | 0.05739198984 | 1.072009758 | 0.2837156493 | 1.063456841 |
| fabp4 | 0.08232327839 | 0.07679874696 | 1.071935177 | 0.283749149 | 1.085806771 |
| igdcc3 | -0.06499212013 | 0.06070839654 | -1.070562292 | 0.2843662862 | 0.9370748474 |
| sorbs1 | -0.07061400545 | 0.06603680777 | -1.069312825 | 0.2849287337 | 0.9318215007 |
| nxph3 | -0.06845562123 | 0.06416857663 | -1.066809096 | 0.2860580503 | 0.9338349016 |
| tk1 | 0.06401638555 | 0.06011754241 | 1.064853668 | 0.2869421544 | 1.066109867 |
| apom | 0.06305378159 | 0.05936882564 | 1.06206887 | 0.2882044209 | 1.06508412 |
| uhrf2 | -0.06299849046 | 0.05942681453 | -1.060102093 | 0.2890981557 | 0.9389448911 |
| notch3 | 0.06363656944 | 0.06006740033 | 1.059419404 | 0.2894088166 | 1.065705019 |
| crh | -0.06025391097 | 0.05687985874 | -1.059318928 | 0.2894545577 | 0.9415254396 |
| ltb | -0.06505776389 | 0.06142458883 | -1.059148545 | 0.2895321345 | 0.9370133363 |
| tdgf1 | 0.0602322831 | 0.05688349829 | 1.058870936 | 0.2896585621 | 1.062083222 |
| ndst1 | 0.06417978176 | 0.06063328968 | 1.058490841 | 0.2898317243 | 1.06628408 |
| cdhr5 | -0.06246920389 | 0.05909015794 | -1.057184581 | 0.2904273552 | 0.9394419935 |
| rab11fip3 | 0.06346733954 | 0.06018222574 | 1.054586113 | 0.2916146576 | 1.065524685 |
| itga2 | -0.06645459442 | 0.06308597711 | -1.053397244 | 0.2921589669 | 0.9357054011 |
| edar | -0.06063578395 | 0.05761081571 | -1.05250695 | 0.2925670234 | 0.9411659651 |
| ptpn9 | -0.06607200336 | 0.06280196636 | -1.052069022 | 0.2927678834 | 0.9360634622 |
| tgfbi | -0.06100651845 | 0.05811532112 | -1.049749313 | 0.2938333846 | 0.9408171071 |
| eef1d | 0.06343873062 | 0.06056349356 | 1.047474756 | 0.2948806693 | 1.065494202 |
| scly | -0.06486557804 | 0.06208370779 | -1.044808378 | 0.296111542 | 0.9371934343 |
| dscam | -0.06389901178 | 0.06119775446 | -1.044139811 | 0.2964207092 | 0.9380997318 |
| pyy | -0.06470702212 | 0.06197712401 | -1.044046867 | 0.2964637068 | 0.9373420436 |
| gnpda1 | 0.0650108493 | 0.06229518728 | 1.043593448 | 0.2966735254 | 1.067170602 |
| man2b2 | -0.0643699416 | 0.06176585839 | -1.042160561 | 0.2973372449 | 0.9376580566 |
| ppp3r1 | 0.06131312726 | 0.05886863193 | 1.041524582 | 0.2976321505 | 1.063231789 |
| cd40 | -0.06126065927 | 0.05884372732 | -1.04107374 | 0.2978413261 | 0.9405780374 |
| rnase3 | 0.05956362486 | 0.05726363399 | 1.040164948 | 0.2982632735 | 1.061373289 |
| lilra5 | 0.06147215865 | 0.05917952156 | 1.038740379 | 0.2989254964 | 1.06340089 |
| ckap4 | 0.06270373196 | 0.06038239238 | 1.038443982 | 0.2990634025 | 1.064711353 |
| serpinb9 | 0.05861587266 | 0.05647497679 | 1.037908752 | 0.2993125388 | 1.060367846 |
| cd177 | 0.05700478291 | 0.05505993683 | 1.035322345 | 0.3005183978 | 1.058660874 |
| cd59 | 0.06572567797 | 0.06353035635 | 1.034555475 | 0.3008765569 | 1.067933719 |
| eif4e | 0.06407275515 | 0.06206638975 | 1.032326117 | 0.30191937 | 1.066169965 |
| stc1 | -0.0629031176 | 0.0609413858 | -1.032190469 | 0.301982899 | 0.9390344452 |
| ctsb | 0.05927654065 | 0.05748978885 | 1.031079464 | 0.3025035573 | 1.061068629 |
| nme3 | -0.05873601032 | 0.05700739419 | -1.030322665 | 0.3028585627 | 0.9429556669 |
| yju2 | 0.06239557129 | 0.06058118415 | 1.029949681 | 0.3030336273 | 1.064383301 |
| scgb2a2 | -0.06166348912 | 0.05997437474 | -1.028163935 | 0.3038727201 | 0.9401992208 |
| nudt15 | 0.06235901571 | 0.06075482439 | 1.026404345 | 0.304701031 | 1.064344392 |
| cd2 | -0.06334178532 | 0.06174261077 | -1.025900663 | 0.3049384101 | 0.9386226114 |
| megf10 | -0.06121284602 | 0.05967318611 | -1.025801537 | 0.3049851415 | 0.9406230106 |
| vegfa | -0.06017710496 | 0.05871921888 | -1.02482809 | 0.3054443096 | 0.9415977572 |
| metap1 | -0.05857863705 | 0.05719866361 | -1.024125973 | 0.3057757779 | 0.9431040746 |
| comp | -0.06199417152 | 0.06054186902 | -1.023988399 | 0.3058407545 | 0.9398883649 |
| cdh22 | -0.06096884587 | 0.05956017433 | -1.023651233 | 0.3060000373 | 0.9408525508 |
| fgf2 | -0.05792427409 | 0.05668628066 | -1.021839384 | 0.3068569258 | 0.9437214089 |
| grik2 | -0.061376033 | 0.06013185006 | -1.020690914 | 0.3074009006 | 0.9404695257 |
| mdga1 | 0.05973148091 | 0.05855546474 | 1.020083799 | 0.3076887197 | 1.061551461 |
| idua | -0.05727891863 | 0.05616055123 | -1.019913754 | 0.3077693659 | 0.9443306412 |
| eng | -0.05772657562 | 0.05661973898 | -1.0195486 | 0.3079425927 | 0.9439079996 |
| gpa33 | -0.05754773496 | 0.05646797455 | -1.019121642 | 0.3081452207 | 0.9440768238 |
| eif4b | 0.05819692593 | 0.05711635773 | 1.018918717 | 0.3082415571 | 1.059923702 |
| eri1 | -0.06236067963 | 0.06123996204 | -1.018300429 | 0.3085352045 | 0.9395439513 |
| cxcl10 | 0.06219064076 | 0.06108496956 | 1.018100544 | 0.3086301767 | 1.064165199 |
| per3 | -0.06146178365 | 0.06037586903 | -1.017985905 | 0.3086846547 | 0.9403888833 |
| aamdc | 0.06319946944 | 0.06226420373 | 1.015020922 | 0.3100958547 | 1.065239301 |
| rab27b | 0.06056651535 | 0.05968803544 | 1.014717856 | 0.3102403399 | 1.062438264 |
| mfap3 | -0.05831829869 | 0.05748771086 | -1.014448094 | 0.3103689855 | 0.9433496327 |
| eif2s2 | -0.06313483264 | 0.06223794823 | -1.014410572 | 0.310386882 | 0.938816882 |
| myom3 | -0.06801082663 | 0.06709490286 | -1.013651168 | 0.3107492329 | 0.9342503587 |

| Protein | beta | se | z | p_val | HR |
|---|---|---|---|---|---|
| il2 | 0.05810640125 | 0.05732902435 | 1.013559919 | 0.3107927914 | 1.059827757 |
| tmed8 | 0.06124035513 | 0.06045541618 | 1.012983766 | 0.3110679154 | 1.063154418 |
| prg2 | 0.06094304727 | 0.06016770101 | 1.01288642 | 0.3111144156 | 1.062838381 |
| chga | 0.06090847074 | 0.06029064745 | 1.010247415 | 0.3123767677 | 1.062801632 |
| tshb | 0.05573143487 | 0.05517192671 | 1.010141175 | 0.3124276577 | 1.057313688 |
| pdp1 | -0.05793462369 | 0.05736714939 | -1.009891973 | 0.3125470488 | 0.9437116418 |
| kctd5 | 0.06370089355 | 0.0631088048 | 1.009382031 | 0.3127914528 | 1.065773571 |
| fgfbp2 | -0.0613460692 | 0.06089525329 | -1.007403137 | 0.3137410844 | 0.9404977062 |
| lelp1 | -0.06151396618 | 0.06121267854 | -1.004921981 | 0.3149344192 | 0.9403398127 |
| trim24 | 0.06079819684 | 0.06051769404 | 1.004635054 | 0.3150726113 | 1.062684439 |
| il12b | -0.05793734947 | 0.05771105799 | -1.003921111 | 0.3154166399 | 0.9437090695 |
| batf | -0.06147734189 | 0.06126840235 | -1.003410233 | 0.3156629687 | 0.9403742526 |
| mmp8 | 0.05793357073 | 0.05776653253 | 1.002891609 | 0.3159131618 | 1.059644602 |
| farsa | 0.06221212351 | 0.06210769076 | 1.001681479 | 0.3164974548 | 1.06418806 |
| usp28 | -0.06228104914 | 0.06220175358 | -1.001274812 | 0.3166939665 | 0.9396187706 |
| upk3bl1 | 0.06010596839 | 0.06005182091 | 1.000901679 | 0.3168743447 | 1.061949074 |
| prdx3 | -0.05746074172 | 0.05742024172 | -1.000705326 | 0.3169692917 | 0.9441589557 |
| vti1a | 0.06197471871 | 0.06197607953 | 0.9999780428 | 0.317321134 | 1.063935447 |
| ceacam8 | 0.06048471027 | 0.06051221058 | 0.9995455412 | 0.3175304893 | 1.062351354 |
| lrrc38 | -0.06271118973 | 0.06274299894 | -0.9994930237 | 0.3175559169 | 0.9392146894 |
| sema3f | -0.060990489 | 0.06102895646 | -0.9993696851 | 0.3176156395 | 0.940832188 |
| esr1 | 0.06096169881 | 0.06101344495 | 0.9991518895 | 0.3177211177 | 1.062858205 |
| gpc1 | -0.05783983156 | 0.0579466677 | -0.9981563023 | 0.3182035721 | 0.9438011025 |
| acy3 | -0.06269415217 | 0.06283473245 | -0.9977626979 | 0.3183944423 | 0.9392306914 |
| fus | 0.05862679935 | 0.05877509831 | 0.9974768403 | 0.3185331099 | 1.060379433 |
| erbb4 | 0.05953878559 | 0.05970269954 | 0.997254497 | 0.3186409945 | 1.061346925 |
| scn2a | -0.06086405966 | 0.06104041628 | -0.9971108221 | 0.3187107206 | 0.9409511443 |
| fam171b | 0.05989024497 | 0.06018503235 | 0.9951019819 | 0.3196866668 | 1.061720011 |
| ralb | 0.06094154918 | 0.06129563648 | 0.994223287 | 0.3201141733 | 1.062836789 |
| epgn | 0.05945846124 | 0.05988906672 | 0.9928099484 | 0.3208025809 | 1.061261677 |
| cd209 | 0.05645879588 | 0.05687085099 | 0.9927545464 | 0.3208295857 | 1.058083017 |
| icam4 | 0.0568392583 | 0.05725969739 | 0.9926573296 | 0.3208769761 | 1.058485654 |
| btc | 0.05692914287 | 0.05735681487 | 0.9925436585 | 0.3209323933 | 1.0585808 |
| gzma | -0.05639422772 | 0.05684723545 | -0.9920311388 | 0.3211823357 | 0.9451664516 |
| b4gat1 | -0.06031100258 | 0.06084085761 | -0.9912911315 | 0.3215434422 | 0.9414716879 |
| serpini2 | -0.05934321772 | 0.05988980186 | -0.9908735023 | 0.3217473526 | 0.942383271 |
| gucy2c | -0.06004048929 | 0.06071679571 | -0.988861296 | 0.3227310093 | 0.941726403 |
| thop1 | 0.05865544043 | 0.05934596147 | 0.9883644814 | 0.3229741761 | 1.060409803 |
| septin7 | -0.05926750315 | 0.05998526674 | -0.9880343352 | 0.3231358329 | 0.9424546259 |
| lamtor5 | 0.06003265164 | 0.06077055286 | 0.9878575858 | 0.3232224002 | 1.061871218 |
| nos2 | -0.0606333402 | 0.06141940794 | -0.9872016394 | 0.3235437983 | 0.9411682651 |
| nefl | 0.06838071044 | 0.06927450336 | 0.9870978083 | 0.3235946921 | 1.070772885 |
| hsbp1 | -0.06041590961 | 0.06137802711 | -0.9843247243 | 0.3249558767 | 0.9413729261 |
| f10 | 0.05929987866 | 0.06033265922 | 0.9828818988 | 0.3256655676 | 1.061093392 |
| apbb1ip | 0.06049542305 | 0.0615583642 | 0.9827327908 | 0.3257389676 | 1.062362735 |
| dpy30 | 0.0588708786 | 0.05993929486 | 0.9821750278 | 0.3260136279 | 1.060638281 |
| tnfrsf6b | -0.05946720895 | 0.06055912176 | -0.9819694743 | 0.326114887 | 0.942266431 |
| cxcl9 | 0.0616370639 | 0.06278976867 | 0.9816418376 | 0.3262763285 | 1.063576264 |
| tpd52l2 | 0.06042591994 | 0.06160636876 | 0.9808388509 | 0.3266722162 | 1.0622889 |
| ywhaq | 0.0602817597 | 0.06149272889 | 0.9803071157 | 0.3269345434 | 1.062135771 |
| obp2b | 0.07152797301 | 0.07299781604 | 0.9798645616 | 0.3271529781 | 1.074148197 |
| casq2 | -0.059312578 | 0.06072532614 | -0.9767354375 | 0.3287001463 | 0.9424121458 |
| sh3bgrl2 | -0.05911060122 | 0.06058048262 | -0.975736717 | 0.329194952 | 0.9426025104 |
| adgrg2 | -0.06425092859 | 0.06588348595 | -0.9752205376 | 0.3294508768 | 0.9377696568 |
| crtap | -0.06038440769 | 0.06191909975 | -0.9752145611 | 0.3294538408 | 0.9414025816 |
| lrrc25 | 0.05770945092 | 0.05918646038 | 0.9750448084 | 0.3295380338 | 1.059407141 |
| scpep1 | 0.06315392315 | 0.06484041788 | 0.9739900701 | 0.3300614697 | 1.065190784 |
| clps | 0.05508615189 | 0.05658436108 | 0.9735225571 | 0.3302936548 | 1.056631642 |
| flrt2 | 0.05478428955 | 0.05630773867 | 0.9729442319 | 0.3305810199 | 1.056312732 |
| rab3gap1 | -0.05861786637 | 0.0602786725 | -0.972447865 | 0.3308277895 | 0.943067078 |
| gsta1 | -0.06198661542 | 0.06378207326 | -0.9718501179 | 0.3311251186 | 0.9398954668 |
| il17ra | 0.05518998892 | 0.05682436671 | 0.9712380817 | 0.3314297343 | 1.056741365 |
| adgrg1 | -0.05881619925 | 0.06056517731 | -0.9711223819 | 0.3314873395 | 0.9428800553 |
| defb4a_defb4b | 0.05844766049 | 0.06028723333 | 0.9694865275 | 0.332302499 | 1.060189494 |
| qsox1 | 0.06089974851 | 0.06288406767 | 0.9684448027 | 0.3328222732 | 1.062792362 |
| mbl2 | 0.05965149395 | 0.06161987965 | 0.9680559956 | 0.333016405 | 1.061466555 |
| klb | 0.05638780964 | 0.05826471891 | 0.9677865217 | 0.3331509965 | 1.05800791 |
| proc | -0.05722751827 | 0.05918456558 | -0.9669331472 | 0.3335774548 | 0.9443791814 |
| khdc3l | 0.05910791123 | 0.06119712283 | 0.9658609506 | 0.3341137645 | 1.060889716 |
| scp2 | -0.05520451624 | 0.05729192512 | -0.9635653912 | 0.3352638651 | 0.9462915962 |

| Protein | beta | se | z | p_val | HR |
|---|---|---|---|---|---|
| myo9b | -0.05463953609 | 0.05672391953 | -0.9632538891 | 0.3354201274 | 0.9468263832 |
| reps1 | -0.05947035553 | 0.0617727408 | -0.9627281347 | 0.3356839738 | 0.9422634661 |
| adgrv1 | -0.05741895956 | 0.05968631323 | -0.9620121674 | 0.336043492 | 0.9441984055 |
| neb | -0.05954378652 | 0.06198652011 | -0.9605925033 | 0.3367570995 | 0.9421942773 |
| rbm25 | -0.05808762232 | 0.06063423214 | -0.9580004606 | 0.3380625263 | 0.9435672663 |
| cd99 | -0.06433388465 | 0.06723480144 | -0.9568539398 | 0.3386409823 | 0.9376918663 |
| lta | -0.05517210481 | 0.05773694397 | -0.9555771576 | 0.3392859065 | 0.9463222673 |
| clec11a | 0.05357654302 | 0.05614871291 | 0.9541900472 | 0.3399874517 | 1.055037744 |
| sct | -0.05671145622 | 0.05954207199 | -0.9524602407 | 0.3408636208 | 0.9448666654 |
| ceacam3 | -0.05426249384 | 0.0569869339 | -0.9521918469 | 0.3409996951 | 0.9471834441 |
| tnf | -0.05794240129 | 0.06086435053 | -0.9519924353 | 0.3411008184 | 0.943704302 |
| il12a_il12b | -0.05543832392 | 0.05824955101 | -0.9517382188 | 0.3412297615 | 0.9460703718 |
| amigo2 | -0.05412023926 | 0.05687552681 | -0.9515558325 | 0.3413222902 | 0.9473181948 |
| foxo3 | 0.05407149315 | 0.05683024168 | 0.9514563294 | 0.3413727772 | 1.055560065 |
| cxcl5 | -0.05506872177 | 0.05793784818 | -0.9504792376 | 0.3418687987 | 0.9464201061 |
| ncs1 | 0.05805172876 | 0.06125495386 | 0.9477066768 | 0.343278801 | 1.059769815 |
| gusb | -0.05999566734 | 0.06333317571 | -0.9473023683 | 0.3434847244 | 0.9417686139 |
| serpina1 | 0.05754599516 | 0.06092848482 | 0.9444842642 | 0.3449222393 | 1.059233989 |
| vps28 | 0.05802991088 | 0.06147903551 | 0.9438975482 | 0.345222005 | 1.059746693 |
| ddx53 | -0.05720534957 | 0.06062471441 | -0.9435978401 | 0.3453751964 | 0.9444001173 |
| gfra2 | 0.05349433396 | 0.05670160058 | 0.9434360479 | 0.3454579121 | 1.054951014 |
| ceacam21 | -0.05365336997 | 0.05689814929 | -0.9429721465 | 0.34569515 | 0.9477605718 |
| lcn15 | 0.05838998208 | 0.06199064165 | 0.9419160784 | 0.3462356076 | 1.060128346 |
| araf | 0.05739717608 | 0.06095993528 | 0.9415557253 | 0.3464201465 | 1.059076367 |
| coq7 | -0.05914120532 | 0.06282283416 | -0.9413966452 | 0.3465016322 | 0.9425736633 |
| eln | -0.07498406222 | 0.07974618102 | -0.9402840519 | 0.3470718778 | 0.9277582726 |
| vtcn1 | 0.05499040103 | 0.05850680533 | 0.9398975166 | 0.3472701313 | 1.056530473 |
| dtx3 | -0.05912107932 | 0.06295507046 | -0.9390995655 | 0.3476796273 | 0.9425926338 |
| klk14 | 0.05380793746 | 0.05739305456 | 0.9375339555 | 0.3484839659 | 1.055281903 |
| prok1 | 0.07381069954 | 0.07878927145 | 0.9368115504 | 0.3488555028 | 1.076602985 |
| plxnb3 | -0.05314501739 | 0.05681639308 | -0.9353817535 | 0.3495915969 | 0.9482424909 |
| abca2 | -0.05795284799 | 0.06196867003 | -0.9351959299 | 0.3496873357 | 0.9436944435 |
| afp | 0.05575670425 | 0.05962284305 | 0.9351567521 | 0.3497075227 | 1.057340406 |
| eloa | 0.0555734111 | 0.05945101368 | 0.9347765103 | 0.3499034875 | 1.057146621 |
| arfip1 | 0.05691314577 | 0.06093323595 | 0.9340246727 | 0.3502911661 | 1.058563866 |
| mertk | -0.05547821432 | 0.05942359294 | -0.9336058555 | 0.3505072437 | 0.9460326334 |
| pepd | -0.05563256179 | 0.05966858637 | -0.9323593062 | 0.3511508678 | 0.9458866269 |
| cntn1 | -0.05607611425 | 0.06018235631 | -0.9317700018 | 0.3514554008 | 0.9454671696 |
| gpc5 | -0.05397233527 | 0.0579477257 | -0.9313969551 | 0.3516482654 | 0.9474583173 |
| il6r | 0.05391693052 | 0.05790984683 | 0.931049441 | 0.3518279899 | 1.055396927 |
| acrbp | 0.05720921829 | 0.06144911733 | 0.9310014655 | 0.3518528061 | 1.058877324 |
| scgn | 0.05461268776 | 0.05871303518 | 0.9301629117 | 0.3522867415 | 1.056131483 |
| top1 | 0.05776927029 | 0.062144473 | 0.9295962698 | 0.3525801595 | 1.059470516 |
| sftpa2 | -0.05450834284 | 0.05864090113 | -0.9295277151 | 0.3526156689 | 0.9469506086 |
| fgf12 | 0.05697233369 | 0.06130988565 | 0.9292519972 | 0.3527585059 | 1.058626522 |
| srp14 | 0.05482013131 | 0.05903631933 | 0.928583149 | 0.3531051581 | 1.056350593 |
| denr | 0.05820678846 | 0.0626918624 | 0.9284584351 | 0.3531698189 | 1.059934155 |
| mapkapk2 | 0.05559321883 | 0.0599890249 | 0.9267231619 | 0.3540702885 | 1.05716756 |
| acan | 0.05673366439 | 0.06134233085 | 0.9248697206 | 0.3550336787 | 1.05837389 |
| ssc4d | -0.06165278514 | 0.06668707433 | -0.9245087711 | 0.3552214869 | 0.9402092848 |
| havcr2 | 0.05631782774 | 0.06093068403 | 0.9242933776 | 0.3553335898 | 1.057933871 |
| pcbp2 | 0.0562423668 | 0.06085236414 | 0.9242429212 | 0.3553598534 | 1.057854041 |
| ahcy | 0.0530005884 | 0.05736032428 | 0.9239938767 | 0.3554895039 | 1.054430266 |
| pdxdc1 | 0.05640919452 | 0.06115784164 | 0.9223542396 | 0.3563438306 | 1.058030535 |
| ung | 0.05653582563 | 0.0613234721 | 0.9219279943 | 0.3565661359 | 1.058164524 |
| itgb6 | -0.05608853238 | 0.06084233262 | -0.9218668971 | 0.3565980079 | 0.9454554288 |
| mrpl46 | -0.05261013253 | 0.05719998806 | -0.9197577537 | 0.3576993646 | 0.9487498271 |
| ttr | -0.0588699008 | 0.06405270529 | -0.9190853148 | 0.3580509498 | 0.9428294225 |
| tnfrsf13b | -0.05293207994 | 0.05764018841 | -0.9183189959 | 0.3584518851 | 0.9484444287 |
| aspn | 0.05699424673 | 0.06210305605 | 0.9177365875 | 0.3587567879 | 1.05864972 |
| afm | -0.05926028479 | 0.06475380949 | -0.915162911 | 0.3601061123 | 0.9424614289 |
| alms1 | -0.05510469108 | 0.06030888644 | -0.913707653 | 0.3608704816 | 0.9463860646 |
| iglc2 | 0.05579497884 | 0.06116397914 | 0.9122195716 | 0.3616531431 | 1.057380876 |
| lgals3 | 0.05503190614 | 0.06042292 | 0.9107786604 | 0.3624120084 | 1.056574325 |
| nectin2 | 0.05260630037 | 0.0577900985 | 0.9102995449 | 0.3626645585 | 1.054014598 |
| gast | -0.05675999764 | 0.06240929248 | -0.9094799089 | 0.3630968583 | 0.9448208014 |
| ntrk3 | -0.05556232711 | 0.06114624562 | -0.9086792908 | 0.3635194387 | 0.9459530633 |
| crip2 | -0.05711952373 | 0.06286048401 | -0.9086713956 | 0.3635236075 | 0.9444811747 |
| dnm3 | -0.05732052768 | 0.06309663895 | -0.9084561179 | 0.3636372882 | 0.9442913493 |
| clec10a | 0.05213339746 | 0.05739987989 | 0.908249243 | 0.3637465526 | 1.05351627 |

| Protein | beta | se | z | p_val | HR |
|---|---|---|---|---|---|
| lilra2 | 0.05305542212 | 0.05843304883 | 0.9079694313 | 0.3638943724 | 1.054488085 |
| rap1a | 0.05594661333 | 0.06180071074 | 0.9052745942 | 0.3653199323 | 1.057541224 |
| siae | -0.05265752348 | 0.05819165617 | -0.9048981752 | 0.3655193339 | 0.948704866 |
| ddr1 | 0.05251039553 | 0.05803928386 | 0.9047388602 | 0.3656037487 | 1.053913518 |
| c1qtnf9 | -0.05542416186 | 0.06128491813 | -0.9043687019 | 0.3657999283 | 0.9460837702 |
| mfap4 | -0.05733114576 | 0.06339928242 | -0.9042869821 | 0.3658432478 | 0.9442813228 |
| vwf | -0.05259768442 | 0.05819211868 | -0.9038626813 | 0.3660682197 | 0.9487616373 |
| duox2 | -0.05214687836 | 0.05771541699 | -0.903517311 | 0.366251405 | 0.9491894412 |
| pof1b | 0.05772546125 | 0.06389105098 | 0.9034983829 | 0.3662614461 | 1.059424103 |
| myh4 | -0.05547410514 | 0.06141424224 | -0.9032775316 | 0.3663786182 | 0.9460365208 |
| b3gnt7 | -0.05788493895 | 0.06409931956 | -0.9030507555 | 0.3664989579 | 0.943758531 |
| thsd1 | -0.05601851754 | 0.06205392833 | -0.9027392632 | 0.3666642929 | 0.945521627 |
| pvalb | 0.05092311383 | 0.05645133182 | 0.9020710795 | 0.3670191107 | 1.052241987 |
| plscr3 | 0.05540278336 | 0.06142637449 | 0.9019380327 | 0.3670897865 | 1.056966257 |
| serpind1 | 0.05855992264 | 0.06497850307 | 0.9012199399 | 0.3674713911 | 1.06030852 |
| s100a11 | 0.05350967794 | 0.05946154756 | 0.8999038898 | 0.3681713999 | 1.054967202 |
| cd6 | -0.05102025356 | 0.0567543936 | -0.8989657068 | 0.3686709272 | 0.9502594242 |
| creld1 | -0.05934785878 | 0.06601853027 | -0.8989575887 | 0.3686752514 | 0.9423788974 |
| smndc1 | 0.05583062488 | 0.06221415443 | 0.8973942569 | 0.369508576 | 1.057418568 |
| mpo | 0.05189826205 | 0.05784516002 | 0.8971928166 | 0.3696160376 | 1.05326858 |
| cela3a | 0.05016860004 | 0.05594431222 | 0.8967596177 | 0.3698472005 | 1.051448356 |
| stx5 | 0.055706842 | 0.0621422745 | 0.8964403451 | 0.3700176277 | 1.057287686 |
| shd | -0.05382487023 | 0.06005098315 | -0.8963195506 | 0.3700821203 | 0.9475980446 |
| fcrl1 | -0.05093194463 | 0.05684783446 | -0.8959346493 | 0.3702876671 | 0.9503433443 |
| wars | -0.05071560234 | 0.05661218299 | -0.8958425494 | 0.3703368612 | 0.950548966 |
| pmch | -0.05441277615 | 0.06076222465 | -0.8955033569 | 0.3705180721 | 0.9470411098 |
| ccnd2 | -0.05466213819 | 0.06106540468 | -0.895140849 | 0.3707117999 | 0.9468049832 |
| podxl2 | 0.05194762554 | 0.05805390844 | 0.8948170233 | 0.3708849087 | 1.053320574 |
| zcchc8 | 0.05483602801 | 0.06129572279 | 0.8946142652 | 0.3709933235 | 1.056367386 |
| mzt1 | 0.05253920017 | 0.05873768054 | 0.8944718227 | 0.3710694993 | 1.053943876 |
| fetub | -0.05464981566 | 0.06114955626 | -0.8937074772 | 0.3714784237 | 0.9468166503 |
| icoslg | 0.05113394031 | 0.05723186277 | 0.8934523155 | 0.3716149973 | 1.052463851 |
| adcyap1r1 | -0.05157188063 | 0.05772850872 | -0.8933520331 | 0.3716686813 | 0.9497353799 |
| olr1 | 0.05195963888 | 0.05817139477 | 0.893216315 | 0.3717413427 | 1.053333228 |
| chm | 0.05461582858 | 0.06121509849 | 0.8921953885 | 0.3722882135 | 1.0561348 |
| pcyt2 | 0.05384734122 | 0.06060067153 | 0.8885601407 | 0.3742395208 | 1.055323485 |
| mcfd2 | 0.05113950334 | 0.05755397058 | 0.8885486583 | 0.3742456942 | 1.052469706 |
| c2orf69 | 0.05478791411 | 0.06166475301 | 0.8884802329 | 0.3742824842 | 1.056316561 |
| ctrl | 0.05295805443 | 0.05966179493 | 0.887637633 | 0.3747357036 | 1.054385417 |
| bax | 0.05229718847 | 0.05898454685 | 0.886625248 | 0.375280696 | 1.05368884 |
| edf1 | -0.05434680329 | 0.06136750015 | -0.8855958472 | 0.3758353502 | 0.9471035909 |
| sort1 | -0.05078918664 | 0.05755765201 | -0.8824054642 | 0.3775575819 | 0.9504790231 |
| art3 | 0.05528427503 | 0.06268064992 | 0.8819990715 | 0.3777773094 | 1.056841005 |
| eci2 | -0.05321970578 | 0.0604058189 | -0.881036078 | 0.378298293 | 0.9481716708 |
| cntn2 | 0.05239218378 | 0.05949838352 | 0.8805648268 | 0.3785534031 | 1.05378894 |
| mrpl52 | 0.0538729445 | 0.06127195174 | 0.8792431605 | 0.3792694472 | 1.055350506 |
| fabp5 | 0.05167616795 | 0.05890720922 | 0.8772469217 | 0.3803525352 | 1.053034681 |
| zfyve19 | 0.05325828383 | 0.0609047905 | 0.8744514741 | 0.3818724358 | 1.054702022 |
| drg2 | 0.05005241253 | 0.05728341736 | 0.8737679217 | 0.3822446532 | 1.051326198 |
| b3gat3 | -0.05323481774 | 0.06112026496 | -0.8709847342 | 0.3837624885 | 0.9481573422 |
| gtf2ird1 | -0.05230689523 | 0.06009417845 | -0.8704153476 | 0.3840734624 | 0.949037567 |
| il5ra | -0.0493477196 | 0.05672421662 | -0.8699585916 | 0.3843230338 | 0.9518500952 |
| csde1 | 0.05304972954 | 0.06098592782 | 0.8698683685 | 0.3843723435 | 1.054482083 |
| clc | 0.05073383458 | 0.05834410354 | 0.8695623295 | 0.3845396318 | 1.052042839 |
| ep300 | 0.05456016603 | 0.06276730709 | 0.869244971 | 0.3847131546 | 1.056076014 |
| cirbp | 0.05267581655 | 0.06060096146 | 0.8692241061 | 0.3847245647 | 1.054087872 |
| folr3 | -0.05238446697 | 0.06029359716 | -0.8688230499 | 0.3849439236 | 0.9489639514 |
| vta1 | 0.04897784117 | 0.05644807758 | 0.86766181 | 0.3855794984 | 1.050197079 |
| lair2 | 0.04930496628 | 0.05688045628 | 0.8668173483 | 0.3860420952 | 1.050540681 |
| gimap8 | 0.0533791 | 0.06165781013 | 0.8657313629 | 0.3866374967 | 1.054829455 |
| treml2 | -0.04946670078 | 0.05721975241 | -0.8645039291 | 0.3873111228 | 0.9517368497 |
| nphs2 | 0.05245315017 | 0.0607585362 | 0.8633050342 | 0.387969777 | 1.053853188 |
| msmb | 0.05202894186 | 0.06028719926 | 0.8630180619 | 0.3881275363 | 1.05340623 |
| gpr101 | -0.05225001812 | 0.06059966886 | -0.8622162317 | 0.3885685392 | 0.9490915471 |
| sema4d | -0.04920308627 | 0.05708926172 | -0.8618623676 | 0.3887632598 | 0.9519877744 |
| lilra3 | 0.05221460383 | 0.06071930461 | 0.8599341538 | 0.3898253403 | 1.053601825 |
| rab10 | 0.05183526051 | 0.06029836884 | 0.8596461482 | 0.3899841282 | 1.053202224 |
| lilrb5 | -0.04834375088 | 0.05627556604 | -0.8590540138 | 0.3903107167 | 0.9528062028 |
| ahnak | -0.05827163855 | 0.06784908899 | -0.8588418713 | 0.3904277632 | 0.9433936505 |
| cryzl1 | 0.05317304092 | 0.06199961009 | 0.8576350859 | 0.3910939948 | 1.05461212 |

| Protein | beta | se | z | p_val | HR |
|---|---|---|---|---|---|
| cope | 0.04893344764 | 0.05722064897 | 0.8551711405 | 0.3924564104 | 1.050150458 |
| il6 | 0.0544195053 | 0.06373254411 | 0.8538731046 | 0.3931753032 | 1.055927476 |
| thbd | 0.05027486019 | 0.05890449142 | 0.8534979079 | 0.3933832473 | 1.051560089 |
| blmh | 0.04937745276 | 0.05785556303 | 0.8534607594 | 0.3934038397 | 1.050616834 |
| alpi | 0.05225207551 | 0.06128602876 | 0.8525935938 | 0.3938847164 | 1.053641306 |
| nacc1 | 0.05215306233 | 0.0611727801 | 0.852553411 | 0.3939070079 | 1.053536987 |
| slc51b | -0.05239432792 | 0.06145740412 | -0.8525307678 | 0.3939195696 | 0.9489545938 |
| gchfr | 0.05761589696 | 0.0675868732 | 0.852471704 | 0.3939523375 | 1.059308034 |
| pnpt1 | -0.04877492004 | 0.05727251856 | -0.8516286915 | 0.3944202099 | 0.9523954707 |
| pde4d | -0.05207350389 | 0.06117339622 | -0.8512442843 | 0.3946336677 | 0.94925909 |
| swap70 | -0.05353425477 | 0.06289832074 | -0.8511237525 | 0.3947006122 | 0.9478734713 |
| cd70 | -0.05004319777 | 0.05880887085 | -0.8509464142 | 0.3947991202 | 0.9511883344 |
| st3gal1 | 0.0517875944 | 0.06088723822 | 0.8505492434 | 0.3950197948 | 1.053152023 |
| timp1 | 0.05202061084 | 0.06128487193 | 0.8488328229 | 0.3959743233 | 1.053397454 |
| cela2a | 0.05092157549 | 0.06000683398 | 0.84859627 | 0.3961059832 | 1.052240369 |
| cd80 | -0.05340224441 | 0.06293364357 | -0.8485484294 | 0.3961326134 | 0.9479986086 |
| bpifb2 | 0.05552910116 | 0.06550120942 | 0.8477568835 | 0.3965733798 | 1.057099779 |
| cpa4 | 0.05322650764 | 0.06281336474 | 0.8473755204 | 0.3967858446 | 1.054668509 |
| gclm | 0.05091229002 | 0.06020086087 | 0.8457070095 | 0.3977162119 | 1.052230598 |
| scgb1a1 | -0.04916414098 | 0.05815905339 | -0.8453394289 | 0.3979213526 | 0.9520248506 |
| siglec10 | 0.04906475748 | 0.05808729869 | 0.8446727355 | 0.3982935861 | 1.050288363 |
| clns1a | 0.05187029801 | 0.06155279117 | 0.8426961154 | 0.3993984203 | 1.053239126 |
| adra2a | 0.05130775734 | 0.06100451075 | 0.8410485834 | 0.4003207178 | 1.052646803 |
| il20 | -0.04720042813 | 0.05613598702 | -0.8408229842 | 0.400447109 | 0.9538961908 |
| larp1 | 0.05083717316 | 0.0605474831 | 0.8396248787 | 0.4011187455 | 1.052151561 |
| gas2 | -0.05090832592 | 0.06067755369 | -0.8389976659 | 0.4014706194 | 0.9503657904 |
| ppm1b | 0.05192706398 | 0.06196603929 | 0.8379923031 | 0.4020350263 | 1.053298916 |
| tbcc | 0.04789577001 | 0.05721103436 | 0.837177138 | 0.4024930062 | 1.049061306 |
| nt5c1a | 0.05976816641 | 0.07140372571 | 0.8370454877 | 0.4025669999 | 1.061590406 |
| sncg | 0.0523330198 | 0.06260154557 | 0.8359700919 | 0.4031717287 | 1.053726596 |
| cpped1 | 0.04898157466 | 0.05864661408 | 0.8351986798 | 0.4036058529 | 1.050201 |
| slk | 0.05068182635 | 0.0606873834 | 0.8351295361 | 0.4036447783 | 1.051988125 |
| gigyf2 | -0.05136809838 | 0.06153270751 | -0.8348096559 | 0.4038248883 | 0.9499289389 |
| gdf2 | 0.05188594426 | 0.06222042954 | 0.8339052726 | 0.4043343658 | 1.053255606 |
| bcl2l1 | 0.0502795818 | 0.06033237038 | 0.8333765354 | 0.404632404 | 1.051565054 |
| cr1 | -0.05061043896 | 0.06075042226 | -0.8330878548 | 0.4047951827 | 0.9506489342 |
| serpina9 | -0.04942823929 | 0.05938335291 | -0.8323585123 | 0.4052066124 | 0.9517734556 |
| pfdn2 | -0.0484360509 | 0.05822872166 | -0.8318240469 | 0.4055082685 | 0.9527182628 |
| slc1a4 | 0.05212967684 | 0.0627115719 | 0.8312608864 | 0.4058262655 | 1.05351235 |
| lrrc37a2 | -0.04982697182 | 0.06001570464 | -0.830232222 | 0.4064075006 | 0.9513940282 |
| timm10 | 0.04973381601 | 0.05991652918 | 0.8300516851 | 0.4065095621 | 1.050991302 |
| apob | -0.04981280751 | 0.06003413245 | -0.8297414401 | 0.4066849862 | 0.9514075042 |
| nxph1 | -0.0486385991 | 0.058627317 | -0.8296234859 | 0.4067516938 | 0.952525311 |
| dtd1 | 0.05130353529 | 0.06185471028 | 0.8294200241 | 0.4068667746 | 1.052642359 |
| cd22 | -0.0480492765 | 0.05794978283 | -0.8291536941 | 0.4070174437 | 0.9530868211 |
| ctsd | -0.05323236555 | 0.0642178529 | -0.8289340604 | 0.4071417207 | 0.9481596672 |
| spink5 | 0.04724672625 | 0.0571750951 | 0.8263515113 | 0.4086047208 | 1.04838064 |
| fdx2 | 0.0502385802 | 0.06080108168 | 0.8262777373 | 0.4086465593 | 1.051521939 |
| ophn1 | 0.04977437442 | 0.06026663419 | 0.8259026754 | 0.4088593028 | 1.051033929 |
| rcor1 | 0.04811442145 | 0.05827883764 | 0.8255899294 | 0.4090367497 | 1.04929071 |
| dsg3 | 0.04965287896 | 0.06017959178 | 0.8250783611 | 0.4093271039 | 1.050906241 |
| baiap2 | 0.04893086918 | 0.05935937249 | 0.8243158095 | 0.4097601379 | 1.050147751 |
| vnn1 | -0.05268793851 | 0.06395105687 | -0.8238790895 | 0.4100082629 | 0.9486760115 |
| il1b | -0.04776514409 | 0.0580349136 | -0.8230415302 | 0.4104843769 | 0.9533576625 |
| kcnip4 | -0.04742507159 | 0.05772440963 | -0.821577421 | 0.4113174442 | 0.9536819283 |
| tia1 | -0.04792702432 | 0.0585092818 | -0.8191354063 | 0.4127091632 | 0.9532033452 |
| acadm | -0.04992496965 | 0.06102356754 | -0.8181260398 | 0.4132852213 | 0.9513007983 |
| c2cd2l | 0.04758027747 | 0.05821640724 | 0.8173001345 | 0.4137569299 | 1.048730387 |
| gbp6 | -0.04929542614 | 0.06036748947 | -0.8165889715 | 0.4141633597 | 0.9518998721 |
| layn | 0.04945501145 | 0.06065106451 | 0.8154022003 | 0.4148421253 | 1.050698322 |
| tdo2 | -0.0502580527 | 0.06171705378 | -0.8143300695 | 0.4154558881 | 0.9509839889 |
| c1qtnf1 | -0.04794483659 | 0.05888362155 | -0.8142304316 | 0.415512955 | 0.9531863666 |
| dkk1 | -0.04653745782 | 0.05716342659 | -0.8141124596 | 0.4155805287 | 0.9545288053 |
| cr2 | -0.04644236857 | 0.05707351247 | -0.8137289359 | 0.4158002537 | 0.9546195751 |
| cd86 | 0.04914649658 | 0.06040808095 | 0.8135748697 | 0.4158885393 | 1.050374216 |
| il4r | -0.04707940366 | 0.05800769932 | -0.8116061181 | 0.4170176796 | 0.9540116426 |
| ceacam19 | -0.05026469866 | 0.06201356608 | -0.810543593 | 0.4176278211 | 0.9509776687 |
| cgn | -0.04986525379 | 0.06156053241 | -0.8100198592 | 0.417928762 | 0.9513576077 |
| vipr1 | -0.05087006783 | 0.06282780818 | -0.8096743989 | 0.4181273357 | 0.9504021503 |
| bcl7b | 0.04883881772 | 0.06035488252 | 0.8091941477 | 0.4184034807 | 1.050051087 |

| Protein | beta | se | z | p_val | HR |
|---|---|---|---|---|---|
| ces2 | -0.04829316795 | 0.05970934873 | -0.8088041315 | 0.4186278194 | 0.9528543998 |
| ncr1 | 0.04660153644 | 0.05763520492 | 0.8085602629 | 0.4187681295 | 1.047704454 |
| tg | -0.04785324088 | 0.0592494562 | -0.8076570478 | 0.4192880362 | 0.9532736784 |
| vash1 | -0.0459879365 | 0.05699772954 | -0.806838042 | 0.4197597987 | 0.9550534834 |
| got1 | 0.0504053146 | 0.0624881305 | 0.8066382239 | 0.4198749449 | 1.051697278 |
| ap2b1 | 0.05060176645 | 0.06275575414 | 0.8063287126 | 0.4200533391 | 1.051903906 |
| dtx2 | -0.04972045266 | 0.06167252103 | -0.8062010734 | 0.4201269199 | 0.9514953753 |
| bche | -0.05141153263 | 0.06379752537 | -0.8058546524 | 0.4203266612 | 0.9498876803 |
| casp1 | 0.04713962154 | 0.05858346101 | 0.8046575046 | 0.4210173485 | 1.04826836 |
| actn2 | -0.05163718675 | 0.0642510924 | -0.8036779582 | 0.4215829871 | 0.9496733584 |
| cntn5 | -0.04884343693 | 0.06079474684 | -0.8034154179 | 0.4217346666 | 0.9523302178 |
| itgal | -0.05031082991 | 0.06263909854 | -0.8031857272 | 0.4218673938 | 0.9509337999 |
| il13ra2 | -0.0490296874 | 0.06107341666 | -0.8027991568 | 0.4220908296 | 0.9521528623 |
| bnip3l | 0.05050493181 | 0.06295706571 | 0.8022122892 | 0.4224301688 | 1.051802051 |
| hadh | -0.04844824486 | 0.06045701487 | -0.8013668051 | 0.4229193264 | 0.9527066455 |
| nlgn1 | 0.04852960409 | 0.06060118331 | 0.8008029123 | 0.4232457527 | 1.049726448 |
| prap1 | -0.05631115609 | 0.07034535111 | -0.8004957712 | 0.4234236127 | 0.9452449714 |
| hip1r | 0.05093002064 | 0.06369026431 | 0.7996515825 | 0.4239126925 | 1.052249255 |
| ache | -0.05175514363 | 0.06475232744 | -0.7992785075 | 0.4241289384 | 0.9495613445 |
| prkag3 | 0.04859341067 | 0.06080403885 | 0.7991806398 | 0.4241856762 | 1.049793429 |
| ntprobnp | -0.04763227724 | 0.05960803163 | -0.7990915978 | 0.4242373012 | 0.9534843405 |
| f2r | -0.04617800677 | 0.05782382043 | -0.7985983358 | 0.4245233529 | 0.9548719734 |
| folr1 | 0.04805350882 | 0.06023189894 | 0.7978082986 | 0.4249817448 | 1.049226797 |
| yap1 | -0.055841446 | 0.07006232324 | -0.7970253256 | 0.425436323 | 0.9456890668 |
| nmi | 0.0485917618 | 0.06107472879 | 0.7956115853 | 0.4262578307 | 1.049791698 |
| rwdd1 | 0.04524333078 | 0.05689450179 | 0.7952144646 | 0.4264887592 | 1.046282422 |
| asah1 | -0.04930535076 | 0.06200838354 | -0.7951400754 | 0.4265320251 | 0.9518904248 |
| guca2a | 0.04682629507 | 0.05897818699 | 0.7939595545 | 0.4272189775 | 1.047939961 |
| amotl2 | -0.04896599623 | 0.06171363899 | -0.7934388092 | 0.4275222071 | 0.952213508 |
| bcat2 | -0.04851137393 | 0.06120378705 | -0.7926204614 | 0.4279989837 | 0.9526465039 |
| adam9 | 0.04990172314 | 0.06298168347 | 0.7923212019 | 0.4281734123 | 1.051167786 |
| tmprss11d | -0.04801994638 | 0.06062305461 | -0.7921070077 | 0.4282982844 | 0.9531147757 |
| cst6 | 0.04642898016 | 0.05866213696 | 0.7914641805 | 0.4286731705 | 1.047523681 |
| pecr | -0.04878325051 | 0.06169059196 | -0.7907729357 | 0.4290765058 | 0.9523875368 |
| klk6 | -0.04526965455 | 0.05733698976 | -0.7895366452 | 0.4297984205 | 0.9557397275 |
| spry2 | -0.04531491729 | 0.0574161728 | -0.7892361174 | 0.4299740161 | 0.9556964691 |
| fbp1 | 0.0464191438 | 0.05882700738 | 0.7890787899 | 0.4300659577 | 1.047513378 |
| ikzf2 | -0.04473518613 | 0.05670910845 | -0.7888536313 | 0.4301975594 | 0.9562506767 |
| ak2 | 0.04766744188 | 0.06044257187 | 0.7886401985 | 0.4303223291 | 1.048821803 |
| tgfb1 | -0.04619507424 | 0.05858398685 | -0.7885273216 | 0.4303883238 | 0.9548556763 |
| hyal1 | 0.04577140301 | 0.05808511343 | 0.7880057437 | 0.4306933462 | 1.04683508 |
| angptl2 | -0.04738961953 | 0.06022172431 | -0.7869190077 | 0.4313292798 | 0.9537157389 |
| f11 | -0.05034379008 | 0.06398697873 | -0.7867817966 | 0.4314096114 | 0.9509024575 |
| cetn3 | 0.04833510244 | 0.06145878476 | 0.7864636866 | 0.4315958852 | 1.049522294 |
| capn3 | -0.04724128101 | 0.06011503977 | -0.785847954 | 0.4319565688 | 0.9538572222 |
| tlr2 | 0.0467452781 | 0.05950901581 | 0.7855158999 | 0.4321511518 | 1.047855063 |
| lamp2 | -0.04917304834 | 0.06275161709 | -0.7836140425 | 0.4332666135 | 0.9520163706 |
| nbl1 | 0.04613300673 | 0.05897989808 | 0.7821818659 | 0.4341077002 | 1.047213688 |
| gmfg | 0.04731696572 | 0.06050505402 | 0.7820332779 | 0.4341950167 | 1.04845428 |
| itgbl1 | -0.05010087057 | 0.06412667645 | -0.7812797005 | 0.4346380066 | 0.9511334783 |
| psap | -0.05019668709 | 0.06437531848 | -0.779750505 | 0.4355377448 | 0.9510423484 |
| reg4 | 0.04671307145 | 0.05998455708 | 0.7787516274 | 0.4361260375 | 1.047821316 |
| cd274 | -0.04552278455 | 0.0585266878 | -0.7778124179 | 0.4366796061 | 0.9554978317 |
| map1lc3b2 | 0.04751206833 | 0.06112951975 | 0.7772360805 | 0.4370194989 | 1.048658857 |
| bcl2 | 0.04912461505 | 0.06335744246 | 0.7753566613 | 0.4381289366 | 1.050351232 |
| elac1 | 0.04690020883 | 0.06054931169 | 0.774578728 | 0.438588631 | 1.048017421 |
| cd300e | 0.04729269511 | 0.06110737661 | 0.7739277602 | 0.4389735122 | 1.048428834 |
| espl1 | 0.04687571371 | 0.06057797162 | 0.7738079116 | 0.4390443932 | 1.04799175 |
| comt | 0.04580874919 | 0.0592062947 | 0.7737141704 | 0.4390998383 | 1.046874176 |
| cmip | 0.04643459359 | 0.06002316829 | 0.7736111724 | 0.4391607632 | 1.047529562 |
| plaur | 0.04939785908 | 0.06387481963 | 0.773354185 | 0.4393127962 | 1.050638274 |
| cdh3 | 0.04397012697 | 0.05686166335 | 0.773282461 | 0.4393552333 | 1.044951139 |
| sema3g | 0.04772543955 | 0.06173809771 | 0.7730306135 | 0.4395042632 | 1.048882634 |
| eddm3b | -0.07088856089 | 0.09179697066 | -0.7722320288 | 0.4399770148 | 0.9315656992 |
| ccl3 | 0.04862432435 | 0.06297561709 | 0.7721135035 | 0.4400472051 | 1.049825883 |
| nomo1 | -0.0472204617 | 0.06116981778 | -0.7719568802 | 0.4401399667 | 0.953877081 |
| igfbp4 | -0.04935053577 | 0.06397598114 | -0.7713916205 | 0.4404748404 | 0.9518474147 |
| cd14 | 0.04611777163 | 0.0597872197 | 0.7713650486 | 0.4404905858 | 1.047197734 |
| il2rb | -0.04366585017 | 0.05661745942 | -0.7712435461 | 0.4405625875 | 0.9572737769 |
| matn2 | 0.04573553225 | 0.05937490527 | 0.7702838774 | 0.441131518 | 1.04679753 |

| Protein | beta | se | z | p_val | HR |
|---|---|---|---|---|---|
| f11r | -0.04454194567 | 0.05782963717 | -0.7702269606 | 0.4411652738 | 0.9564354809 |
| enah | -0.04566072791 | 0.05934277369 | -0.7694404065 | 0.4416319092 | 0.9553660362 |
| s100a4 | 0.04400944847 | 0.05733677294 | 0.7675606111 | 0.442748271 | 1.044992228 |
| mdm1 | -0.047335097 | 0.06172121894 | -0.7669177281 | 0.4431304325 | 0.9537677393 |
| apol1 | 0.04736901683 | 0.06178003704 | 0.7667366208 | 0.4432381257 | 1.048508855 |
| asah2 | -0.04514841682 | 0.05889975295 | -0.7665298164 | 0.4433611177 | 0.9558556063 |
| chrm1 | 0.04727599118 | 0.06171345948 | 0.7660564093 | 0.4436427386 | 1.048411321 |
| ldlrap1 | 0.04627865604 | 0.06042028924 | 0.7659456222 | 0.4437086584 | 1.047366225 |
| itgb7 | -0.04371185836 | 0.05713626033 | -0.7650458414 | 0.4442442479 | 0.9572297355 |
| egfr | -0.04451356011 | 0.05819942834 | -0.7648453152 | 0.4443636603 | 0.9564626303 |
| caps | -0.04990418868 | 0.06537690019 | -0.7633306035 | 0.4452662551 | 0.9513205674 |
| pfdn4 | 0.04690528034 | 0.06148701991 | 0.7628484908 | 0.4455537583 | 1.048022736 |
| clec14a | -0.04494734571 | 0.05899772272 | -0.7618488246 | 0.4461502362 | 0.9560478205 |
| prkra | -0.04421650878 | 0.0581122438 | -0.7608811138 | 0.4467280801 | 0.956746791 |
| plin3 | 0.04413870243 | 0.05805134678 | 0.7603389908 | 0.447051981 | 1.045127307 |
| mvk | -0.04552566768 | 0.05988708771 | -0.760191711 | 0.447139999 | 0.9554950769 |
| nudc | -0.0435057358 | 0.05725183209 | -0.7599011982 | 0.4473136454 | 0.9574270625 |
| cd46 | 0.04537551688 | 0.05984107723 | 0.7582670464 | 0.4482911308 | 1.046420735 |
| lpo | -0.04353334732 | 0.05761059962 | -0.7556482246 | 0.4498601342 | 0.9574006268 |
| f9 | -0.04785778917 | 0.06336081389 | -0.755321566 | 0.4500560619 | 0.9532693427 |
| atp2b4 | -0.0458509759 | 0.06081731676 | -0.7539131672 | 0.4509013636 | 0.9551842971 |
| tagln3 | 0.04584504426 | 0.06088725152 | 0.7529498067 | 0.4514800767 | 1.046912173 |
| ppp2r5a | 0.04594877088 | 0.0610422699 | 0.7527369306 | 0.451608013 | 1.047020772 |
| tpsab1 | -0.04281159748 | 0.05688172334 | -0.7526424125 | 0.4516648239 | 0.95809188 |
| il17f | 0.04307753129 | 0.05723594467 | 0.7526307382 | 0.4516718411 | 1.044018836 |
| ap3b1 | 0.04550758287 | 0.06046496384 | 0.7526273064 | 0.4516739039 | 1.046558941 |
| efcab2 | 0.04596212836 | 0.06106995062 | 0.7526144673 | 0.4516816214 | 1.047034757 |
| siglec8 | 0.04845593319 | 0.06441406823 | 0.7522569917 | 0.4518965264 | 1.049649116 |
| sdhb | -0.04537622228 | 0.06042145179 | -0.7509952333 | 0.4526555245 | 0.9556378819 |
| rbp1 | -0.04543901507 | 0.06062095441 | -0.7495595461 | 0.4535200229 | 0.9555778766 |
| cox5b | 0.04324189214 | 0.05776832341 | 0.74853985 | 0.4541345978 | 1.044190446 |
| serpinf1 | -0.0517825932 | 0.06921835437 | -0.7481049452 | 0.4543968594 | 0.9495352798 |
| sirpb1 | 0.04335485111 | 0.05797585731 | 0.7478087108 | 0.4545755472 | 1.044308403 |
| ddx58 | -0.04349864375 | 0.05819554223 | -0.7474566279 | 0.4547879741 | 0.9574338526 |
| cspg4 | -0.04876760835 | 0.06532416575 | -0.7465477406 | 0.4553366038 | 0.9524024344 |
| pros1 | 0.04630420347 | 0.06208911399 | 0.7457700794 | 0.4558063173 | 1.047392983 |
| gp5 | -0.04557279328 | 0.0611545809 | -0.7452065341 | 0.4561468734 | 0.9554500497 |
| mepe | -0.04487561908 | 0.06022367489 | -0.7451491321 | 0.4561815701 | 0.9561163971 |
| hsp90b1 | -0.04245986004 | 0.05699579185 | -0.7449648239 | 0.4562929851 | 0.958428936 |
| palm2 | -0.05307369971 | 0.07134696387 | -0.7438816851 | 0.4569480564 | 0.9483101197 |
| rad23b | 0.04314802451 | 0.05801204552 | 0.7437769884 | 0.4570114039 | 1.044092435 |
| mfge8 | 0.04355097153 | 0.05857319895 | 0.7435306986 | 0.4571604428 | 1.044513233 |
| fcn2 | 0.04344212043 | 0.05843023161 | 0.7434870482 | 0.4571868601 | 1.044399543 |
| git1 | 0.0456806844 | 0.06144846425 | 0.7433983088 | 0.4572405679 | 1.046740117 |
| rpgr | -0.04532549483 | 0.06101199325 | -0.7428948378 | 0.4575453513 | 0.9556863602 |
| ceacam1 | -0.04252525824 | 0.05731369877 | -0.7419737193 | 0.4581032587 | 0.9583662586 |
| tpp1 | -0.04430326924 | 0.05971701182 | -0.7418869078 | 0.4581558588 | 0.9566637868 |
| phospho1 | 0.0460468508 | 0.06208230331 | 0.7417065467 | 0.4582651524 | 1.047123468 |
| tnfrsf1a | 0.04686762629 | 0.06322694114 | 0.741260378 | 0.4585355807 | 1.047983274 |
| optc | 0.04603599007 | 0.06218908844 | 0.740258319 | 0.4591432669 | 1.047112096 |
| inhbb | 0.0472869504 | 0.06389517366 | 0.7400707706 | 0.4592570534 | 1.048422811 |
| gpr15l | 0.04794603589 | 0.06478700492 | 0.7400563732 | 0.459265789 | 1.049114039 |
| tppp2 | -0.04437480453 | 0.05996237589 | -0.7400441339 | 0.4592732153 | 0.956595354 |
| sptlc1 | -0.04472652738 | 0.06044178847 | -0.7399934468 | 0.4593039707 | 0.9562589567 |
| rnf41 | 0.04327809289 | 0.05854304177 | 0.7392525496 | 0.4597536566 | 1.044228247 |
| ccl17 | 0.04216852595 | 0.05705090622 | 0.7391385824 | 0.4598228506 | 1.043070248 |
| ca11 | -0.04243835818 | 0.05741875981 | -0.7391026613 | 0.459844661 | 0.9584495443 |
| serpinh1 | 0.04456420319 | 0.06029535878 | 0.7390983998 | 0.4598472485 | 1.045572104 |
| tppp3 | 0.04340399589 | 0.05872666918 | 0.7390849252 | 0.4598554301 | 1.044359727 |
| btn3a2 | -0.04246375935 | 0.05745603704 | -0.7390652322 | 0.4598673875 | 0.9584251988 |
| krt17 | 0.04527806482 | 0.06149458471 | 0.7362935295 | 0.4615520831 | 1.046318764 |
| fkbp14 | 0.04472697928 | 0.06083820457 | 0.735179146 | 0.4622303978 | 1.045742312 |
| id4 | -0.04423687353 | 0.06018194643 | -0.7350522234 | 0.4623076896 | 0.9567273073 |
| rilpl2 | 0.04445048378 | 0.06048805564 | 0.7348638225 | 0.4624224331 | 1.045453209 |
| cd48 | -0.04169249564 | 0.05673849898 | -0.7348184458 | 0.4624500717 | 0.9591646826 |
| arnt | -0.04182709462 | 0.05693587917 | -0.7346350882 | 0.4625617626 | 0.9590355887 |
| igfbpl1 | -0.04478221809 | 0.06099054765 | -0.7342484993 | 0.4627972996 | 0.9562057035 |
| ttn | 0.04631268374 | 0.06309334712 | 0.7340343452 | 0.4629278061 | 1.047401865 |
| f3 | -0.04402676809 | 0.0600036497 | -0.7337348364 | 0.4631103625 | 0.956928342 |
| psma1 | -0.04200291648 | 0.05729175878 | -0.7331406361 | 0.4634726578 | 0.9588669841 |

| Protein | beta | se | z | p_val | HR |
|---|---|---|---|---|---|
| cracr2a | 0.04173838249 | 0.0569715892 | 0.7326174866 | 0.4637917627 | 1.042621675 |
| pla2g1b | 0.04117062801 | 0.05620203879 | 0.7325468773 | 0.4638348416 | 1.04202989 |
| cabp2 | 0.04504420894 | 0.06150431986 | 0.7323747185 | 0.4639398853 | 1.046074105 |
| ppp1r14a | 0.04398662546 | 0.06008486539 | 0.7320749605 | 0.464122816 | 1.044968379 |
| epb41l5 | -0.04439992561 | 0.06071278638 | -0.731310952 | 0.4645892424 | 0.9565713236 |
| arhgap1 | 0.04243406561 | 0.05806362094 | 0.7308201749 | 0.4648889989 | 1.043347262 |
| itgb2 | 0.04374086923 | 0.05987169124 | 0.7305768106 | 0.4650376806 | 1.044711603 |
| ptprr | -0.04510601067 | 0.06178230862 | -0.7300797215 | 0.4653414559 | 0.9558961413 |
| itgb5 | 0.04278012498 | 0.05864512079 | 0.7294745821 | 0.4657114104 | 1.043708384 |
| tnfsf14 | 0.04178127206 | 0.05729619014 | 0.729215537 | 0.4658698287 | 1.042666394 |
| snu13 | 0.04464767363 | 0.06123364692 | 0.7291362817 | 0.465918303 | 1.045659382 |
| lysmd3 | 0.04448906831 | 0.06110394277 | 0.7280883409 | 0.4665595104 | 1.045493548 |
| ehbp1 | 0.04428264877 | 0.06083334986 | 0.7279337547 | 0.4666541391 | 1.04527776 |
| cst7 | -0.04237908894 | 0.05822503324 | -0.7278499743 | 0.4667054291 | 0.9585063525 |
| vps53 | 0.04143795979 | 0.05693897863 | 0.7277608555 | 0.4667599906 | 1.042308495 |
| adamts13 | -0.04155436688 | 0.05714002826 | -0.7272374226 | 0.4670805251 | 0.9592971799 |
| cdh4 | -0.04382372045 | 0.06041927034 | -0.7253268734 | 0.4682515231 | 0.9571226638 |
| kit | -0.04275718674 | 0.0590156897 | -0.7245054148 | 0.4687555041 | 0.9581440119 |
| ighmbp2 | 0.04408776468 | 0.06094169408 | 0.7234417314 | 0.4694085405 | 1.045074071 |
| pbld | 0.04336085289 | 0.06006635399 | 0.7218825517 | 0.4703666896 | 1.044314671 |
| col4a4 | -0.043073187 | 0.05971835301 | -0.7212721857 | 0.4707420665 | 0.957841286 |
| siglec9 | -0.04208095819 | 0.05837183161 | -0.720912074 | 0.4709636137 | 0.9587921554 |
| megf11 | -0.04373797497 | 0.06071702138 | -0.7203577181 | 0.471304776 | 0.9572047362 |
| dbn1 | 0.04485988974 | 0.06231633923 | 0.7198736365 | 0.4716028014 | 1.045881311 |
| nfkb2 | -0.04464907924 | 0.06204995736 | -0.7195666385 | 0.471791859 | 0.9563330201 |
| st6gal1 | -0.04268533719 | 0.05935181115 | -0.7191918218 | 0.4720227377 | 0.9582128566 |
| arl13b | 0.0434133476 | 0.0603919357 | 0.7188600117 | 0.4722271772 | 1.044369493 |
| mars1 | 0.04416338869 | 0.06143885806 | 0.7188185146 | 0.4722527484 | 1.045153107 |
| pdlim5 | 0.04368819582 | 0.0608165907 | 0.718359831 | 0.4725354478 | 1.044656576 |
| rtbdn | -0.04274681855 | 0.0596769003 | -0.7163042707 | 0.4738034903 | 0.9581539462 |
| plcb1 | -0.04435044101 | 0.06198280759 | -0.7155281075 | 0.4742827792 | 0.9566186603 |
| ephb4 | 0.04048210111 | 0.0566536148 | 0.7145546008 | 0.4748843061 | 1.041312671 |
| hjv | -0.04541388735 | 0.063601034 | -0.7140432237 | 0.4752004522 | 0.9556018884 |
| gpkow | 0.04185984771 | 0.05883441637 | 0.7114857305 | 0.4767832902 | 1.042748325 |
| clec4c | 0.04040747611 | 0.05680323382 | 0.7113587273 | 0.476861968 | 1.041234966 |
| ifit3 | 0.04312239208 | 0.06079205017 | 0.7093426189 | 0.4781118868 | 1.044065672 |
| ilkap | -0.04085309515 | 0.05770188864 | -0.7080027381 | 0.4789435571 | 0.9599701439 |
| padi4 | 0.04075437469 | 0.05765014025 | 0.7069258551 | 0.4796125556 | 1.041596232 |
| eno2 | 0.04052988448 | 0.05733506866 | 0.706895194 | 0.4796316108 | 1.04136243 |
| syap1 | 0.04345568875 | 0.06151540964 | 0.7064195623 | 0.479927259 | 1.044413714 |
| ppp1r14d | 0.04310548006 | 0.06117734933 | 0.7045986878 | 0.4810600154 | 1.044048015 |
| nfe2 | 0.0433293746 | 0.0615423681 | 0.7040576425 | 0.4813968771 | 1.044281798 |
| chrdl1 | 0.04583194988 | 0.06512588015 | 0.703744038 | 0.48159219 | 1.046898465 |
| galnt5 | -0.04287361964 | 0.06093903926 | -0.70354932 | 0.4817134821 | 0.9580324589 |
| top1mt | -0.04221143157 | 0.06020394864 | -0.7011405817 | 0.4832152866 | 0.9586670667 |
| hk2 | 0.04119213936 | 0.05880773987 | 0.7004543866 | 0.4836435816 | 1.042052306 |
| npr1 | 0.04296914513 | 0.06144470793 | 0.6993140106 | 0.4843558131 | 1.043905685 |
| sema7a | -0.04026173212 | 0.0575791344 | -0.6992417052 | 0.4844009912 | 0.9605380026 |
| spart | 0.0433094508 | 0.06197659783 | 0.6988032954 | 0.4846749688 | 1.044260992 |
| ptprh | -0.04250494394 | 0.06094403767 | -0.6974422038 | 0.4855260974 | 0.9583857273 |
| neo1 | -0.04274324702 | 0.06141512875 | -0.695972603 | 0.4864459874 | 0.9581573682 |
| kir3dl1 | -0.03877996292 | 0.05581306048 | -0.6948187859 | 0.4871688737 | 0.9619623533 |
| gne | 0.03931509403 | 0.05667772838 | 0.6936603699 | 0.4878952246 | 1.040098161 |
| h2ap | 0.04226593555 | 0.06097483578 | 0.6931701415 | 0.4882027838 | 1.043171858 |
| nit2 | 0.04287870296 | 0.06192420429 | 0.6924384971 | 0.4886619969 | 1.043811276 |
| man1a2 | -0.0455646701 | 0.06584008741 | -0.6920505712 | 0.4889055711 | 0.955457811 |
| tnfsf10 | -0.03975575758 | 0.0574840799 | -0.6915959628 | 0.4891910977 | 0.9610241333 |
| tars1 | 0.04173814258 | 0.06040575087 | 0.6909630619 | 0.4895887543 | 1.042621425 |
| pten | 0.03974622287 | 0.05755595984 | 0.6905665892 | 0.4898379499 | 1.040546674 |
| ccl20 | 0.03957444235 | 0.05754704153 | 0.687688564 | 0.4916489231 | 1.040367943 |
| pglyrp2 | -0.04165489118 | 0.06079772682 | -0.6851389577 | 0.4932562388 | 0.9592007521 |
| ceacam6 | 0.04334625649 | 0.0632887919 | 0.684896254 | 0.49340939 | 1.044299428 |
| vcam1 | 0.04014427743 | 0.05865816142 | 0.6843766742 | 0.4937373414 | 1.040960951 |
| pcdh9 | -0.04249264852 | 0.06221820738 | -0.682961633 | 0.4946310867 | 0.9583975111 |
| cd84 | -0.03917070427 | 0.05738752863 | -0.6825647523 | 0.4948819132 | 0.9615865482 |
| fgf20 | -0.04175007949 | 0.06120046342 | -0.6821856758 | 0.495121551 | 0.9591094517 |
| cep43 | -0.03979076187 | 0.05836206378 | -0.6817915491 | 0.4953707687 | 0.960990494 |
| nt5e | -0.03915468017 | 0.05744740854 | -0.6815743506 | 0.4955081383 | 0.9616019569 |
| sox9 | -0.04115131171 | 0.06038116041 | -0.681525685 | 0.4955389201 | 0.9596839075 |
| klrf1 | 0.04352752984 | 0.06387301437 | 0.6814697923 | 0.4955742745 | 1.044488749 |

| Protein | beta | se | z | p_val | HR |
|---|---|---|---|---|---|
| cd302 | -0.04172876369 | 0.06133430596 | -0.6803494886 | 0.496283196 | 0.9591298961 |
| ghrl | -0.04086799956 | 0.06007077308 | -0.6803308408 | 0.4962950008 | 0.9599558362 |
| mdk | 0.03937976854 | 0.05793695502 | 0.6797003488 | 0.4966942151 | 1.040165431 |
| tax1bp1 | 0.04080601367 | 0.06003656727 | 0.6796859902 | 0.4967033087 | 1.04165002 |
| egfl7 | -0.0404312204 | 0.05949546553 | -0.6795680989 | 0.4967779747 | 0.9603752165 |
| il12rb2 | 0.04185279563 | 0.06167389618 | 0.6786144257 | 0.4973822001 | 1.042740971 |
| camkk1 | 0.03997386208 | 0.0589085022 | 0.6785754277 | 0.4974069166 | 1.04078357 |
| ptp4a3 | 0.04356546706 | 0.06420669498 | 0.6785190714 | 0.4974426358 | 1.044528374 |
| siglec6 | 0.0383059075 | 0.05645925107 | 0.67846999 | 0.4974737453 | 1.039049037 |
| cd5 | -0.03916972915 | 0.05779590876 | -0.6777249461 | 0.4979461064 | 0.9615874859 |
| egflam | -0.04120637844 | 0.06082550948 | -0.677452253 | 0.4981190546 | 0.9596310623 |
| lgals4 | 0.04046333541 | 0.06001538605 | 0.6742160314 | 0.5001739789 | 1.04129313 |
| washc3 | -0.04133346891 | 0.06137556623 | -0.6734515287 | 0.5006600759 | 0.9595091101 |
| bpifb1 | 0.04124697368 | 0.06125571162 | 0.6733571873 | 0.5007200788 | 1.042109447 |
| ube2l6 | 0.04108887802 | 0.06110234581 | 0.672459911 | 0.5012909533 | 1.041944707 |
| acox1 | 0.03794311652 | 0.05646608542 | 0.6719629356 | 0.5016072926 | 1.038672148 |
| naga | -0.04220888885 | 0.06290910441 | -0.6709504013 | 0.5022521268 | 0.9586695043 |
| clec1b | -0.03778074418 | 0.05631315191 | -0.6709044494 | 0.5022814017 | 0.9629240445 |
| gamt | -0.04080578116 | 0.06091897499 | -0.6698369624 | 0.5029617281 | 0.9600155649 |
| sap18 | -0.03997001062 | 0.0597330843 | -0.6691435926 | 0.5034038845 | 0.9608182531 |
| bgn | 0.03865477346 | 0.05781267969 | 0.6686210304 | 0.5037372538 | 1.039411589 |
| crisp2 | -0.04351191548 | 0.06507787453 | -0.6686130393 | 0.5037423526 | 0.9574211459 |
| jmjd1c | 0.04119602418 | 0.06165077112 | 0.6682158785 | 0.5039958012 | 1.042056354 |
| slc9a3r2 | 0.04126757348 | 0.06176311297 | 0.6681588978 | 0.504032169 | 1.042130915 |
| siglec1 | 0.03923564329 | 0.0587511774 | 0.667827353 | 0.5042438042 | 1.040015527 |
| mfap3l | -0.04066208474 | 0.06090056336 | -0.667679944 | 0.5043379149 | 0.9601535257 |
| dynlt3 | -0.04033843522 | 0.06045248944 | -0.6672750054 | 0.5045964887 | 0.9604643292 |
| muc2 | -0.03984300739 | 0.05979495855 | -0.6663272014 | 0.5052019826 | 0.9609402878 |
| dynlt1 | 0.04142211072 | 0.06220950365 | 0.6658485969 | 0.5055078791 | 1.042291975 |
| nt5c3a | 0.03771138907 | 0.05664910854 | 0.6657013683 | 0.5056019988 | 1.038431487 |
| eif4g1 | 0.03756662394 | 0.05652944888 | 0.664549623 | 0.5063385998 | 1.038281169 |
| ncam2 | -0.03944074427 | 0.05935529981 | -0.6644856382 | 0.5063795379 | 0.9613269164 |
| spint2 | -0.03867127862 | 0.05821091161 | -0.6643304073 | 0.5064788635 | 0.9620669091 |
| stk24 | -0.03856891785 | 0.05810938977 | -0.663729528 | 0.506863437 | 0.9621653921 |
| ccl24 | -0.03818516665 | 0.0576273133 | -0.6626227125 | 0.5075722201 | 0.9625346951 |
| cyb5r2 | 0.04123655178 | 0.06241719606 | 0.6606601126 | 0.5088303091 | 1.042098587 |
| ift20 | 0.04076974808 | 0.06171379881 | 0.6606261301 | 0.5088521074 | 1.041612245 |
| tomm20 | 0.04012301212 | 0.06074682313 | 0.6604956449 | 0.5089358124 | 1.040938814 |
| dclre1c | -0.03967284353 | 0.06012654522 | -0.6598224359 | 0.5093677838 | 0.9611038191 |
| nfya | 0.04072158237 | 0.06172844845 | 0.6596890638 | 0.5094533862 | 1.041562076 |
| adgre5 | 0.03911269856 | 0.05930392338 | 0.659529696 | 0.5095556832 | 1.039887671 |
| clmp | 0.04379281564 | 0.06640153817 | 0.6595150782 | 0.5095650669 | 1.044765873 |
| plxna4 | -0.03713448094 | 0.05638069466 | -0.6586382301 | 0.5101281073 | 0.963546548 |
| defa1_defa1b | 0.03861954529 | 0.05868507429 | 0.6580812201 | 0.5104859426 | 1.039374973 |
| ccl27 | -0.03960495071 | 0.0602531302 | -0.6573094306 | 0.5109819739 | 0.9611690733 |
| apoe | 0.03986953559 | 0.06068210934 | 0.6570229022 | 0.5111661905 | 1.040674994 |
| fhip2a | -0.03986317218 | 0.06071355025 | -0.6565778481 | 0.5114523963 | 0.9609209109 |
| itpr1 | -0.04123897066 | 0.06284698284 | -0.6561805961 | 0.5117079322 | 0.9595997864 |
| vav3 | 0.04032518776 | 0.06149603765 | 0.6557363579 | 0.5119937712 | 1.041149288 |
| calcoco1 | 0.03671653514 | 0.05604653228 | 0.655108062 | 0.5123981818 | 1.037398913 |
| cd164l2 | -0.04044136873 | 0.06173340833 | -0.6550969698 | 0.512405323 | 0.9603654703 |
| pdap1 | 0.03959753687 | 0.06046220389 | 0.6549138855 | 0.5125231997 | 1.040391971 |
| il7r | 0.03794981459 | 0.05799973841 | 0.6543100992 | 0.5129120407 | 1.038679105 |
| snx18 | 0.03958881189 | 0.06050491681 | 0.6543073517 | 0.5129138104 | 1.040382893 |
| ddhd2 | 0.03987559584 | 0.06094859071 | 0.654249678 | 0.5129509607 | 1.040681301 |
| flt3 | 0.03818194224 | 0.05843084236 | 0.6534552762 | 0.5134628131 | 1.038920239 |
| dkk4 | 0.03877078899 | 0.05941211088 | 0.6525738341 | 0.5140310588 | 1.039532184 |
| crhbp | -0.03860361693 | 0.05916133498 | -0.6525142973 | 0.5140694527 | 0.9621320064 |
| ceacam16 | 0.04121538334 | 0.06318222776 | 0.6523255795 | 0.514191162 | 1.042076527 |
| ment | 0.04279135406 | 0.06561560128 | 0.6521521287 | 0.5143030384 | 1.043720104 |
| tmem106a | -0.03963688184 | 0.06085288816 | -0.6513558031 | 0.5148168338 | 0.9611383826 |
| nlgn2 | -0.03975646971 | 0.06109192309 | -0.6507647443 | 0.5151983619 | 0.961023449 |
| insl5 | -0.04292080454 | 0.06596590797 | -0.6506513115 | 0.5152715994 | 0.9579872553 |
| lat | -0.03672390302 | 0.05645958835 | -0.6504458162 | 0.5154042907 | 0.9639422401 |
| serpina6 | 0.04608829304 | 0.07092770374 | 0.6497925438 | 0.5158262359 | 1.047166864 |
| lrg1 | 0.040206056 | 0.06190993577 | 0.6494281653 | 0.5160616639 | 1.041025262 |
| ca9 | 0.03705095852 | 0.05731037775 | 0.6464964981 | 0.5179578643 | 1.037745901 |
| pdcl2 | -0.03905634696 | 0.06041423363 | -0.6464759149 | 0.5179711902 | 0.9616965189 |
| cd1c | 0.03715884251 | 0.05767660321 | 0.6442619788 | 0.5194055663 | 1.037857864 |
| krt18 | -0.04161254674 | 0.0646521107 | -0.6436378687 | 0.519810288 | 0.9592413698 |

| Protein | beta | se | z | p_val | HR |
|---|---|---|---|---|---|
| fam3c | 0.03808128411 | 0.05921869052 | 0.643061908 | 0.5201839302 | 1.038815669 |
| ptprk | -0.03965210213 | 0.06172747321 | -0.6423736478 | 0.5206306057 | 0.9611237539 |
| hspb6 | -0.04534900668 | 0.07061068354 | -0.6422400181 | 0.5207173533 | 0.9556638905 |
| cxadr | -0.03705979174 | 0.05770936159 | -0.6421798946 | 0.5207563857 | 0.9636185172 |
| cblif | -0.03589178681 | 0.05590190802 | -0.6420494054 | 0.520841105 | 0.9647446859 |
| gipr | 0.04020845919 | 0.06266919454 | 0.6415984677 | 0.5211339281 | 1.041027763 |
| smad1 | -0.03724013614 | 0.05805861504 | -0.6414230879 | 0.5212478365 | 0.9634447497 |
| tgfbr2 | 0.04009861674 | 0.06259978367 | 0.6405551967 | 0.5218117165 | 1.040913421 |
| efna4 | 0.03800560689 | 0.05934372659 | 0.6404317537 | 0.5218919445 | 1.038737057 |
| wdr46 | -0.03860095751 | 0.06032969591 | -0.6398334507 | 0.522280883 | 0.9621345651 |
| gpd1 | -0.04393141975 | 0.06877428271 | -0.6387768511 | 0.5229681096 | 0.9570195879 |
| tnfrsf17 | -0.03760431337 | 0.05895864796 | -0.637808272 | 0.5235984942 | 0.9630939489 |
| xiap | 0.03979730037 | 0.06243902105 | 0.6373786728 | 0.5238782168 | 1.040599824 |
| tap1 | 0.03820668726 | 0.06001857513 | 0.6365810448 | 0.5243977753 | 1.038945948 |
| cdc26 | 0.03885582678 | 0.06109411175 | 0.635999537 | 0.5247767238 | 1.039620587 |
| gabarapl1 | 0.03844580115 | 0.0604826787 | 0.6356497757 | 0.5250047187 | 1.039194404 |
| siglec7 | -0.03775444699 | 0.05950810531 | -0.6344420947 | 0.5257923453 | 0.962949367 |
| rab33a | 0.03820436581 | 0.06024770081 | 0.6341215564 | 0.5260014957 | 1.038943536 |
| ckmt1a_ckmt1b | -0.03543123553 | 0.0559018448 | -0.6338115611 | 0.5262038073 | 0.9651891027 |
| fgf23 | -0.03892307189 | 0.06146385329 | -0.6332676818 | 0.5265588542 | 0.9618246977 |
| xrcc4 | -0.0364547106 | 0.05759700817 | -0.6329271565 | 0.5267812129 | 0.964201761 |
| appl2 | 0.03864008356 | 0.06114825293 | 0.6319082182 | 0.5274468529 | 1.03939632 |
| prkar2a | 0.03831627773 | 0.06065111911 | 0.6317488991 | 0.5275509698 | 1.039059812 |
| lmod2 | 0.03802245765 | 0.06021036184 | 0.6314935916 | 0.5277178381 | 1.038754561 |
| tmed1 | 0.03775341836 | 0.05990990269 | 0.6301699163 | 0.5285834197 | 1.038475132 |
| setmar | 0.03922496977 | 0.06226154257 | 0.6300031794 | 0.528692504 | 1.040004427 |
| folh1 | 0.03927967753 | 0.06238083932 | 0.6296753612 | 0.5289070061 | 1.040061325 |
| atp6v1g1 | -0.03944861659 | 0.06266161835 | -0.6295499163 | 0.5289891004 | 0.9613193486 |
| nrtn | -0.03628910023 | 0.05765656016 | -0.6294010625 | 0.5290865226 | 0.9643614561 |
| evi2b | 0.03906763771 | 0.06217988646 | 0.6283002421 | 0.5298072731 | 1.039840814 |
| gfer | 0.03723025701 | 0.05931438368 | 0.6276767067 | 0.5302157476 | 1.037931984 |
| casp9 | -0.03861964969 | 0.06154943017 | -0.6274574692 | 0.5303594067 | 0.9621165809 |
| dipk2b | 0.04067535142 | 0.06487244776 | 0.6270050356 | 0.5306559343 | 1.041513925 |
| bag6 | 0.03777338817 | 0.06028614674 | 0.626568295 | 0.5309422564 | 1.038495871 |
| cd58 | 0.03596344508 | 0.05751667843 | 0.6252698532 | 0.5317939625 | 1.036617952 |
| gipc3 | 0.03766001344 | 0.06023279436 | 0.6252410144 | 0.531812887 | 1.038378138 |
| mrc1 | -0.04022288002 | 0.06440650144 | -0.624515835 | 0.5322888743 | 0.9605753222 |
| spring1 | -0.03900971068 | 0.06249220595 | -0.6242332157 | 0.532474436 | 0.9617413699 |
| wfikkn2 | 0.03765781877 | 0.0603518121 | 0.6239716333 | 0.5326462145 | 1.038375859 |
| sptbn2 | 0.03792356584 | 0.06094808661 | 0.6222273405 | 0.5337923907 | 1.038651841 |
| tyrp1 | -0.03723394283 | 0.05988712267 | -0.6217353776 | 0.534115885 | 0.9634507166 |
| cdkl5 | -0.03745373637 | 0.06031409048 | -0.620978217 | 0.5346139556 | 0.9632389796 |
| ddah1 | 0.03666904794 | 0.05910257798 | 0.6204306003 | 0.5349743314 | 1.037349651 |
| kif20b | 0.03751346033 | 0.06046449669 | 0.6204212784 | 0.534980467 | 1.038225972 |
| grin2b | 0.03786850561 | 0.06109037661 | 0.6198767746 | 0.5353389177 | 1.038594655 |
| rictor | 0.03814435223 | 0.06156107072 | 0.6196180766 | 0.5355092628 | 1.038881187 |
| defb104a_defb104b | 0.03999609772 | 0.06459011304 | 0.619229412 | 0.5357652385 | 1.040806713 |
| dnajb14 | 0.03784426439 | 0.06112753617 | 0.6191033823 | 0.5358482553 | 1.038569478 |
| ttf2 | -0.03823178134 | 0.06185009969 | -0.6181361312 | 0.5364856069 | 0.9624898279 |
| ccl5 | -0.03528725548 | 0.05710446625 | -0.6179421295 | 0.5366134866 | 0.9653280806 |
| c1qtnf5 | 0.03847243197 | 0.06234843889 | 0.6170552568 | 0.5371982795 | 1.039222079 |
| stat2 | 0.03836657852 | 0.06232625194 | 0.61557654 | 0.5381740387 | 1.039112079 |
| cpb2 | -0.03798277751 | 0.06171439186 | -0.615460614 | 0.5382505723 | 0.9627295214 |
| csf2 | -0.03790818931 | 0.06165866392 | -0.6148071803 | 0.5386820668 | 0.9628013323 |
| ostn | -0.03801160689 | 0.06187316129 | -0.6143472564 | 0.5389858812 | 0.9627017669 |
| naa10 | -0.03703906985 | 0.06033208041 | -0.6139199842 | 0.5392682037 | 0.9636384854 |
| tslp | -0.03542573575 | 0.05776793174 | -0.613242238 | 0.5397161801 | 0.965194411 |
| xg | 0.04549207796 | 0.07418552286 | 0.6132204264 | 0.5397306002 | 1.046542714 |
| msra | 0.03505536197 | 0.05718562959 | 0.6130099856 | 0.5398697371 | 1.035677044 |
| ltbp3 | 0.03813024846 | 0.06221162163 | 0.6129119843 | 0.5399345386 | 1.038866535 |
| enpp2 | -0.04124968275 | 0.06736740642 | -0.6123092003 | 0.5403332037 | 0.9595895071 |
| btn1a1 | 0.03728973723 | 0.06098059038 | 0.6115017418 | 0.5408674656 | 1.037993723 |
| ctla4 | -0.03647852035 | 0.05966934241 | -0.6113444339 | 0.5409715804 | 0.9641788039 |
| nt5c | 0.0371927857 | 0.06093515817 | 0.6103666064 | 0.5416189839 | 1.037893093 |
| dlg4 | 0.03654912456 | 0.05995794749 | 0.609579315 | 0.5421405174 | 1.037225256 |
| ssbp1 | 0.03683261165 | 0.0604518431 | 0.6092884808 | 0.542333241 | 1.037519338 |
| stx3 | 0.03691547804 | 0.06059491078 | 0.6092174666 | 0.5423803044 | 1.037605317 |
| tnfrsf14 | 0.03616494839 | 0.0594934406 | 0.6078812727 | 0.5432662211 | 1.036826855 |
| ulbp2 | 0.03577577768 | 0.0589377738 | 0.6070093146 | 0.5438447306 | 1.036423431 |
| dgka | 0.03771973998 | 0.06220672354 | 0.6063611429 | 0.5442749653 | 1.038440159 |

| Protein | beta | se | z | p_val | HR |
|---|---|---|---|---|---|
| mybpc1 | -0.04113323754 | 0.06786813339 | -0.6060758633 | 0.5444643779 | 0.9597012532 |
| cadps | -0.03722955383 | 0.06144256629 | -0.6059244605 | 0.5445649158 | 0.9634549452 |
| tmem132a | 0.03755236399 | 0.06209405506 | 0.60476585 | 0.5453345874 | 1.038266363 |
| ndufa5 | -0.03822496519 | 0.06325105874 | -0.6043371597 | 0.5456195056 | 0.9624963884 |
| klk10 | 0.03462366343 | 0.05729720357 | 0.6042819068 | 0.5456562334 | 1.035230041 |
| cd160 | -0.03370059098 | 0.05582547203 | -0.6036776718 | 0.5460579618 | 0.9668609482 |
| ifnar1 | -0.03702401745 | 0.06150432631 | -0.6019741971 | 0.5471913132 | 0.9636529906 |
| vps37a | 0.0341187609 | 0.05669893343 | 0.6017531343 | 0.5473384754 | 1.034707482 |
| tmco5a | 0.03713523222 | 0.06173492835 | 0.6015270967 | 0.5474889696 | 1.03783336 |
| tst | -0.03473027757 | 0.05775413755 | -0.6013470038 | 0.5476088888 | 0.9658658968 |
| spint3 | -0.05791752592 | 0.09635034912 | -0.6011138148 | 0.5477641827 | 0.9437277773 |
| pdgfra | 0.03441504233 | 0.05728572905 | 0.6007611826 | 0.5479990619 | 1.035014092 |
| shisa5 | -0.0396505556 | 0.06601347279 | -0.6006433827 | 0.5480775364 | 0.9611252403 |
| c8b | 0.03683336185 | 0.06137297901 | 0.6001560042 | 0.5484022715 | 1.037520116 |
| irak4 | 0.03402295448 | 0.05677878223 | 0.5992195172 | 0.5490265094 | 1.034608355 |
| fkbp1b | -0.03370506412 | 0.05624935798 | -0.5992079792 | 0.5490342025 | 0.9668566233 |
| tpr | 0.03778796807 | 0.06308628054 | 0.5989886826 | 0.5491804316 | 1.038511012 |
| slamf7 | -0.03417853861 | 0.05710921518 | -0.5984767695 | 0.5495218549 | 0.9663989497 |
| adh4 | -0.03987776914 | 0.0667265762 | -0.5976294814 | 0.5500871883 | 0.9609068845 |
| apoa1 | 0.03934782134 | 0.0658448895 | 0.5975835275 | 0.5501178582 | 1.040132201 |
| psip1 | -0.03521428627 | 0.05894465742 | -0.5974126887 | 0.5502318842 | 0.9653985224 |
| rabepk | -0.03433525297 | 0.05748831866 | -0.5972561692 | 0.5503363631 | 0.966247513 |
| brd1 | 0.03605174006 | 0.06043727718 | 0.5965149614 | 0.550831262 | 1.036709485 |
| clec6a | 0.03488075141 | 0.05856538693 | 0.5955864588 | 0.551451525 | 1.03549622 |
| mrpl24 | -0.03684866456 | 0.06197823331 | -0.5945420286 | 0.5521496406 | 0.9638219847 |
| smpdl3a | -0.03375287656 | 0.05685551246 | -0.5936605809 | 0.5527391532 | 0.9668103966 |
| il1rl2 | -0.03640912587 | 0.06135494218 | -0.5934179802 | 0.5529014588 | 0.9642457149 |
| ctrc | 0.03317809727 | 0.05595845337 | 0.592905902 | 0.553244128 | 1.033734628 |
| slc39a14 | 0.03440372102 | 0.05805125658 | 0.5926438642 | 0.553419517 | 1.035002375 |
| il16 | -0.0352615458 | 0.05960237457 | -0.5916131036 | 0.5541096972 | 0.9653528992 |
| wnt9a | -0.03805875157 | 0.06441760088 | -0.5908129309 | 0.5546457699 | 0.9626563817 |
| efnb2 | -0.03648364358 | 0.06180433851 | -0.5903087787 | 0.554983655 | 0.9641738642 |
| brk1 | 0.03473851188 | 0.05886034755 | 0.5901853001 | 0.5550664262 | 1.035348942 |
| oxct1 | -0.03696974981 | 0.06269892532 | -0.5896392901 | 0.5554325047 | 0.9637052872 |
| palm | 0.03934150309 | 0.06672278548 | 0.5896262095 | 0.5554412761 | 1.040125629 |
| insl3 | -0.05760709934 | 0.09773823813 | -0.5894018599 | 0.5555917292 | 0.944020781 |
| lhb | 0.03788031473 | 0.06428410301 | 0.5892641096 | 0.5556841169 | 1.038606919 |
| prl | 0.03455851303 | 0.05866605292 | 0.5890717256 | 0.5558131595 | 1.035162597 |
| numb | 0.03678966292 | 0.06256271735 | 0.5880445171 | 0.5565024126 | 1.037474778 |
| sftpa1 | 0.03438095162 | 0.05851274203 | 0.5875805923 | 0.5568138409 | 1.034978808 |
| artn | -0.03339913002 | 0.05690002752 | -0.5869791541 | 0.5572177071 | 0.967152463 |
| retn | 0.03362649695 | 0.05730608462 | 0.5867875491 | 0.5573464 | 1.034198258 |
| rnase10 | -0.03634763803 | 0.06203392626 | -0.5859316058 | 0.557921477 | 0.9643050061 |
| mog | -0.03456791752 | 0.05904906453 | -0.5854100789 | 0.5582720134 | 0.9660227276 |
| tspan1 | 0.03758767481 | 0.06436793334 | 0.5839503128 | 0.5592537416 | 1.038303026 |
| epn1 | -0.03490898781 | 0.05984778762 | -0.5832962118 | 0.5596939122 | 0.9656933021 |
| mybpc2 | -0.037682333 | 0.06460799408 | -0.5832456733 | 0.5597279286 | 0.9630188116 |
| gart | 0.03545500422 | 0.06090869101 | 0.5821009059 | 0.560498715 | 1.036091027 |
| tnfrsf11a | 0.03529734158 | 0.06068737124 | 0.5816258122 | 0.5608187525 | 1.035927687 |
| klk8 | 0.03471240478 | 0.05969854027 | 0.5814615337 | 0.5609294361 | 1.035321912 |
| npy | -0.03327258386 | 0.05726764936 | -0.5810013897 | 0.5612395169 | 0.9672748601 |
| rbp2 | 0.03369894672 | 0.05805731467 | 0.5804427386 | 0.561616091 | 1.034273189 |
| entpd6 | -0.03277911871 | 0.05647324213 | -0.5804362822 | 0.5616204438 | 0.9677522944 |
| scarf1 | -0.03344001521 | 0.05765210445 | -0.5800311286 | 0.5618936261 | 0.9671129216 |
| c5 | 0.03601006845 | 0.06215164451 | 0.5793904366 | 0.5623257556 | 1.036666284 |
| cdh17 | -0.03306411664 | 0.05708014545 | -0.5792577503 | 0.5624152689 | 0.9674765263 |
| mthfsd | -0.03516440118 | 0.06071854238 | -0.5791377691 | 0.5624962171 | 0.9654466826 |
| fcer1a | 0.03508169356 | 0.06062168133 | 0.5786987887 | 0.5627924335 | 1.035704316 |
| col6a3 | 0.03653913799 | 0.06323809419 | 0.5778026434 | 0.5633973704 | 1.037214898 |
| prune2 | -0.03526081825 | 0.06113039183 | -0.5768132215 | 0.564065637 | 0.9653536016 |
| cd3d | -0.03475808907 | 0.06050883024 | -0.5744300284 | 0.5656768376 | 0.965839035 |
| hgf | -0.03757117949 | 0.06547101484 | -0.5738597391 | 0.5660627195 | 0.9631258605 |
| slc12a2 | 0.03414375073 | 0.0595627634 | 0.5732398697 | 0.5664822925 | 1.03473334 |
| reep4 | -0.03450070093 | 0.06019012055 | -0.5731954118 | 0.5665123906 | 0.9660876625 |
| cgref1 | 0.03307313769 | 0.05770963288 | 0.5730956175 | 0.5665799544 | 1.033626134 |
| gapdh | 0.03458570104 | 0.06039126477 | 0.5726937691 | 0.5668520571 | 1.035190741 |
| acadsb | 0.03403551587 | 0.05946067778 | 0.5724037656 | 0.5670484653 | 1.034621352 |
| gzmh | -0.03437902123 | 0.06009169366 | -0.5721093738 | 0.567247879 | 0.9662052229 |
| stoml2 | 0.03484303862 | 0.06090872296 | 0.5720533435 | 0.5672858363 | 1.035457169 |
| golm2 | -0.03401966743 | 0.05965088571 | -0.5703128634 | 0.5684655179 | 0.9665524948 |

| Protein | beta | se | z | p_val | HR |
|---|---|---|---|---|---|
| lrig1 | 0.03256849526 | 0.0571873394 | 0.5695053416 | 0.5690132468 | 1.033104653 |
| uxs1 | -0.03311879148 | 0.05827965303 | -0.5682736557 | 0.5698491645 | 0.9674236311 |
| spon1 | 0.03502592896 | 0.06168260458 | 0.5678412771 | 0.570142749 | 1.035646562 |
| sorcs2 | -0.03447944587 | 0.06072706663 | -0.5677772333 | 0.5701862408 | 0.966108197 |
| prdx1 | 0.03221072229 | 0.05677280862 | 0.5673617895 | 0.5704684047 | 1.032735103 |
| cd101 | 0.03461954369 | 0.06105605741 | 0.5670124335 | 0.5707057342 | 1.035225776 |
| ptges2 | 0.03584393729 | 0.06322507238 | 0.5669259986 | 0.5707644597 | 1.036494076 |
| qpct | 0.03262842373 | 0.05755775421 | 0.5668814598 | 0.5707947213 | 1.033166568 |
| itga6 | -0.03227171885 | 0.0569839682 | -0.5663297919 | 0.5711696117 | 0.9682434563 |
| bag3 | 0.03304354511 | 0.05837563669 | 0.5660502733 | 0.5713596056 | 1.033595546 |
| anpep | -0.0323126764 | 0.05712933517 | -0.5656056789 | 0.5716618665 | 0.9682038003 |
| l1cam | -0.03293604429 | 0.0582913982 | -0.5650240911 | 0.5720573781 | 0.9676004412 |
| sdc1 | 0.03298573785 | 0.05838202332 | 0.5649981959 | 0.5720749913 | 1.033535799 |
| eif1ax | 0.03410122431 | 0.06038382099 | 0.5647410805 | 0.5722498879 | 1.034689337 |
| crtam | 0.03221509189 | 0.05709524403 | 0.5642342447 | 0.5725947251 | 1.032739615 |
| axl | 0.03241133254 | 0.05745531371 | 0.5641137511 | 0.5726767202 | 1.032942301 |
| cd74 | 0.03483747774 | 0.06176652686 | 0.5640187253 | 0.5727413885 | 1.035451411 |
| tgoln2 | 0.03405410032 | 0.06038295853 | 0.5639687282 | 0.5727754147 | 1.03464058 |
| bhmt2 | -0.03418536229 | 0.06064990364 | -0.5636507272 | 0.5729918567 | 0.9663923553 |
| btn2a1 | 0.03300916372 | 0.05879294633 | 0.5614476869 | 0.5744923832 | 1.03356001 |
| pdgfa | -0.03144551472 | 0.05609521152 | -0.5605739576 | 0.575088009 | 0.9690437536 |
| ripk4 | -0.03430920341 | 0.06121078199 | -0.5605091504 | 0.5751322 | 0.9662726836 |
| tnfaip8l2 | 0.03480230393 | 0.06209647283 | 0.5604554065 | 0.5751688483 | 1.035414991 |
| c1s | -0.03478549749 | 0.06223811551 | -0.5589098771 | 0.5762232282 | 0.9658125633 |
| ftcd | -0.03815832187 | 0.06832148914 | -0.5585112729 | 0.5764953089 | 0.9625605344 |
| trim25 | 0.03369915498 | 0.06034046553 | 0.5584835099 | 0.5765142618 | 1.034273404 |
| rbpms | 0.03485578203 | 0.0625192457 | 0.5575208344 | 0.5771716283 | 1.035470365 |
| myo6 | -0.03391408892 | 0.06084397321 | -0.5573943832 | 0.5772580023 | 0.9666545474 |
| lta4h | -0.03252128516 | 0.05844821285 | -0.5564119684 | 0.577929259 | 0.9680018455 |
| ppp1r9b | -0.03133544601 | 0.05632124575 | -0.5563699027 | 0.5779580095 | 0.9691504209 |
| slc44a4 | -0.03394425774 | 0.06101967133 | -0.5562838508 | 0.5780168252 | 0.966625385 |
| scarb1 | 0.03212404094 | 0.05786142069 | 0.5551892877 | 0.5787651953 | 1.032645588 |
| esyt2 | -0.0337102398 | 0.06076793401 | -0.5547373026 | 0.5790743574 | 0.9668516192 |
| fbln2 | -0.03490263159 | 0.06300596028 | -0.5539576167 | 0.5796078521 | 0.9656994403 |
| s100p | 0.03343321086 | 0.06038421324 | 0.5536746951 | 0.5798014963 | 1.033998382 |
| scn2b | 0.03378062261 | 0.06102865973 | 0.5535206371 | 0.5799069533 | 1.034357667 |
| kazn | 0.03304340716 | 0.0599707231 | 0.5509923085 | 0.5816389472 | 1.033595404 |
| sod3 | 0.03676899841 | 0.06685845137 | 0.5499528879 | 0.5823516876 | 1.03745334 |
| tinagl1 | 0.0325034787 | 0.05916439995 | 0.5493756166 | 0.582747704 | 1.033037487 |
| pglyrp4 | -0.03333169371 | 0.0607559561 | -0.5486160674 | 0.583268957 | 0.9672176864 |
| syngap1 | -0.03340300379 | 0.06093769665 | -0.5481500881 | 0.5835888504 | 0.9671487164 |
| tnxb | -0.03188414065 | 0.05816994956 | -0.5481204796 | 0.5836091793 | 0.9686187991 |
| znf174 | 0.03422495348 | 0.06248997856 | 0.5476870736 | 0.5839067894 | 1.034817366 |
| scg2 | -0.03145860124 | 0.05749060749 | -0.5471954917 | 0.5842444331 | 0.9690310723 |
| dnajc6 | 0.03415281839 | 0.06249500261 | 0.5464887905 | 0.5847299909 | 1.034742722 |
| bach1 | -0.0311848926 | 0.05712239757 | -0.5459310871 | 0.5851133083 | 0.9692963408 |
| esm1 | 0.03232659552 | 0.05928225948 | 0.545299653 | 0.5855474427 | 1.032854776 |
| uros | 0.03335062924 | 0.06116715371 | 0.5452375534 | 0.5855901466 | 1.033912996 |
| slmap | 0.03352518384 | 0.06151928006 | 0.5449540991 | 0.5857850872 | 1.034093486 |
| amigo1 | -0.03298134641 | 0.06066259654 | -0.5436850431 | 0.5866582267 | 0.9675566078 |
| tubb3 | 0.03325928253 | 0.06133211785 | 0.5422816575 | 0.5876244896 | 1.033818556 |
| plxdc2 | -0.0331584029 | 0.06126166754 | -0.5412585754 | 0.5883293686 | 0.9673853108 |
| inppl1 | 0.03096995916 | 0.05726918793 | 0.5407787378 | 0.5886600998 | 1.031454518 |
| ca14 | 0.03365965839 | 0.06225112939 | 0.5407075939 | 0.5887091435 | 1.034232554 |
| draxin | 0.03108029002 | 0.05750337555 | 0.5404950532 | 0.5888556716 | 1.031568325 |
| clasp1 | 0.0324838223 | 0.0601236092 | 0.5402839705 | 0.5890012111 | 1.033017181 |
| mapk13 | -0.03337526715 | 0.06177514925 | -0.5402701176 | 0.5890107632 | 0.9671755423 |
| tnni3 | 0.03158841819 | 0.0585130872 | 0.5398521887 | 0.5892989725 | 1.032092627 |
| cdkn1a | -0.0310583859 | 0.05754846428 | -0.5396909595 | 0.5894101757 | 0.969418971 |
| pcna | 0.03323753495 | 0.06160457212 | 0.5395303271 | 0.5895209769 | 1.033796073 |
| cytl1 | -0.03414419801 | 0.06332262699 | -0.5392100681 | 0.5897419141 | 0.966432137 |
| kynu | -0.03253998971 | 0.06043806459 | -0.5384022459 | 0.5902993762 | 0.9679837397 |
| cd72 | -0.03337360433 | 0.06199173201 | -0.538355733 | 0.5903314812 | 0.9671771505 |
| rasgrf1 | 0.03291439767 | 0.0611908255 | 0.5378975917 | 0.5906477512 | 1.033462069 |
| thbs2 | -0.03118707155 | 0.05799337992 | -0.5377695107 | 0.5907361836 | 0.9692942287 |
| tnc | 0.03104666654 | 0.0577478359 | 0.537624762 | 0.5908361315 | 1.031533641 |
| lgals3bp | 0.0351288178 | 0.06541938454 | 0.5369787265 | 0.5912823088 | 1.035753124 |
| tspan15 | -0.03179925816 | 0.05929114503 | -0.5363239005 | 0.5917347152 | 0.9687010214 |
| thap12 | -0.03306450741 | 0.0616717982 | -0.5361365871 | 0.5918641556 | 0.9674761482 |
| havcr1 | 0.03459749069 | 0.06454031274 | 0.5360601649 | 0.5919169698 | 1.035202946 |

| Protein | beta | se | z | p_val | HR |
|---|---|---|---|---|---|
| epo | -0.03149615609 | 0.05877371535 | -0.5358884647 | 0.5920356372 | 0.9689946812 |
| fstl3 | 0.0355381415 | 0.06632041587 | 0.535855227 | 0.5920586101 | 1.036177169 |
| hdac8 | 0.03303103163 | 0.06164698327 | 0.5358093758 | 0.5920903017 | 1.033582612 |
| pdlim7 | -0.03052100857 | 0.05699656756 | -0.5354885369 | 0.5923120821 | 0.9699400548 |
| mink1 | 0.03236993329 | 0.06051566201 | 0.5349017463 | 0.5927178007 | 1.032899539 |
| timm8a | -0.032064712 | 0.05995261291 | -0.5348342706 | 0.5927644629 | 0.9684439101 |
| adamts4 | -0.03430444204 | 0.06415936758 | -0.5346755016 | 0.5928742648 | 0.9662772844 |
| ensa | -0.03278862116 | 0.06135611132 | -0.534398619 | 0.5930657742 | 0.9677430984 |
| erp29 | 0.03253507913 | 0.06108034427 | 0.5326603758 | 0.5942686999 | 1.033070132 |
| wasf1 | -0.03086029898 | 0.05794458183 | -0.5325829957 | 0.5943222756 | 0.9696110193 |
| gabra4 | 0.03240201703 | 0.06093346773 | 0.5317605946 | 0.5948918181 | 1.032932678 |
| znrd2 | 0.03280291501 | 0.06174286546 | 0.5312826795 | 0.5952229061 | 1.033346862 |
| tcl1b | 0.0302724846 | 0.05698960477 | 0.5311930961 | 0.5952849767 | 1.030735355 |
| pdrg1 | -0.03240474122 | 0.06112552054 | -0.5301344011 | 0.5960187491 | 0.9681146669 |
| lpa | 0.03187720675 | 0.06016619524 | 0.5298192219 | 0.5962372766 | 1.032390727 |
| kcnc4 | -0.03250732896 | 0.06135778039 | -0.5297996236 | 0.5962508662 | 0.9680153553 |
| nek7 | 0.03148245853 | 0.05942966078 | 0.5297431975 | 0.5962899932 | 1.031983273 |
| nedd9 | 0.0327267629 | 0.06187868661 | 0.5288858683 | 0.5968846257 | 1.033268173 |
| itga5 | -0.03248266762 | 0.06145849454 | -0.5285301546 | 0.5971314234 | 0.9680392281 |
| muc13 | 0.03093497645 | 0.05856710599 | 0.5281971156 | 0.5973625312 | 1.031418435 |
| vsir | -0.0299750426 | 0.0567922599 | -0.5278015463 | 0.5976370838 | 0.9704697537 |
| siglec15 | 0.03021154626 | 0.05725839797 | 0.5276351999 | 0.5977525569 | 1.030672546 |
| edil3 | -0.03361048035 | 0.06374070631 | -0.5273000928 | 0.5979852099 | 0.9669480766 |
| echs1 | 0.0328595543 | 0.06237508915 | 0.5268057288 | 0.5983285042 | 1.033405392 |
| polr2a | 0.03260272756 | 0.06192162877 | 0.5265159882 | 0.5985297464 | 1.03314002 |
| sh2d1a | -0.03048060774 | 0.05789503696 | -0.5264804954 | 0.5985544003 | 0.969979242 |
| klk12 | -0.02965805538 | 0.05634642748 | -0.5263520103 | 0.5986436524 | 0.9707774289 |
| tnfrsf13c | -0.03071607266 | 0.05836326184 | -0.5262912265 | 0.5986858779 | 0.9697508728 |
| ehd3 | 0.0323563486 | 0.06165922136 | 0.5247609017 | 0.5997494145 | 1.032885507 |
| vegfc | -0.02954517653 | 0.05636428266 | -0.5241826054 | 0.6001515382 | 0.9708870153 |
| klrd1 | -0.03026368483 | 0.05776076332 | -0.523948838 | 0.6003141251 | 0.9701896755 |
| postn | -0.03198245374 | 0.0610435547 | -0.5239284294 | 0.6003283204 | 0.9685235759 |
| txnrd1 | 0.0305239083 | 0.05829596166 | 0.5236024492 | 0.6005550776 | 1.030994539 |
| ca13 | 0.02966935234 | 0.05666829367 | 0.5235617736 | 0.600583375 | 1.030113873 |
| casp2 | -0.03024560011 | 0.0577775624 | -0.5234834918 | 0.6006378361 | 0.9702072213 |
| skiv2l | -0.03211933952 | 0.06138526032 | -0.5232418883 | 0.600805935 | 0.9683910079 |
| pafah2 | -0.0315227488 | 0.06025642626 | -0.5231433518 | 0.6008744991 | 0.9689689133 |
| gli2 | 0.03223883507 | 0.06165543374 | 0.5228871668 | 0.6010527756 | 1.032764136 |
| idi2 | -0.03141514126 | 0.06013101498 | -0.5224448859 | 0.6013606106 | 0.9690731873 |
| tank | -0.03029626384 | 0.05801326295 | -0.5222299574 | 0.6015102303 | 0.9701580682 |
| ptprz1 | 0.03596280363 | 0.0690005067 | 0.5211962252 | 0.6022300835 | 1.036617287 |
| rbp5 | -0.03350366394 | 0.06430364548 | -0.5210227771 | 0.6023509044 | 0.967051368 |
| spink8 | 0.0315317942 | 0.06064176809 | 0.5199682528 | 0.6030857026 | 1.032034188 |
| ca5a | -0.0326692157 | 0.06289786425 | -0.5194010336 | 0.6034811107 | 0.9678586591 |
| cert | 0.02998770168 | 0.05783234739 | 0.5185281773 | 0.6040898058 | 1.030441861 |
| klk7 | -0.03293768862 | 0.06354868218 | -0.5183063989 | 0.604244509 | 0.9675988501 |
| ednrb | 0.03233850379 | 0.06246827476 | 0.5176788363 | 0.6046823667 | 1.032867076 |
| fkbp7 | 0.03022425362 | 0.05844473387 | 0.517142463 | 0.6050567133 | 1.030685643 |
| creld2 | -0.02987213417 | 0.05781508382 | -0.5166840934 | 0.6053767018 | 0.9705696283 |
| rnase1 | 0.03392764117 | 0.06566647798 | 0.5166660709 | 0.6053892848 | 1.034509748 |
| mpi | 0.02991953407 | 0.05792193186 | 0.5165493123 | 0.6054708071 | 1.030371621 |
| dcbld2 | -0.03057336436 | 0.05925377054 | -0.5159733142 | 0.6058730475 | 0.9698892741 |
| arhgap45 | 0.03163523762 | 0.06144163755 | 0.5148827225 | 0.6066349749 | 1.03214095 |
| gstm4 | 0.03067676147 | 0.05979940502 | 0.5129944262 | 0.6079552196 | 1.031152142 |
| il17c | 0.02927119818 | 0.05729641719 | 0.5108730984 | 0.6094399195 | 1.02970381 |
| c1qbp | -0.03015392798 | 0.05906679843 | -0.5105055426 | 0.6096973326 | 0.9702961663 |
| tarm1 | -0.03119486529 | 0.06113538456 | -0.5102587563 | 0.6098701933 | 0.9692866743 |
| irag2 | -0.02907914915 | 0.05712039852 | -0.5090851937 | 0.6106925097 | 0.9713395807 |
| znf830 | 0.03066734765 | 0.06036088921 | 0.5080665319 | 0.6114066853 | 1.031142435 |
| gfra1 | 0.03114602435 | 0.06133655212 | 0.5077889656 | 0.611601349 | 1.031636137 |
| vwc2 | 0.03055335499 | 0.06017011498 | 0.5077828919 | 0.6116056089 | 1.031024899 |
| mucl3 | 0.0311153207 | 0.06127963053 | 0.5077596002 | 0.6116219452 | 1.031604462 |
| cpa2 | 0.02899808334 | 0.05723393409 | 0.5066589219 | 0.6123941579 | 1.029422621 |
| celsr2 | -0.03083671417 | 0.0609746745 | -0.5057298693 | 0.6130462967 | 0.9696338876 |
| kiaa2013 | 0.03053957703 | 0.06045838841 | 0.5051338256 | 0.6134648449 | 1.031010694 |
| dusp29 | 0.03213802752 | 0.0636381661 | 0.5050118425 | 0.6135505183 | 1.032660031 |
| cep152 | 0.03072959788 | 0.06100331942 | 0.5037364879 | 0.614446564 | 1.031206626 |
| csh1 | -0.03007473393 | 0.05973960616 | -0.5034304017 | 0.6146617015 | 0.9703730111 |
| dctn6 | -0.02909712312 | 0.05781023537 | -0.5033213052 | 0.6147383897 | 0.971322122 |
| adamtsl5 | 0.03109336538 | 0.0618234003 | 0.5029384542 | 0.615007544 | 1.031581813 |

| Protein | beta | se | z | p_val | HR |
|---|---|---|---|---|---|
| egln1 | 0.0299171756 | 0.05951184202 | 0.5027096219 | 0.6151684438 | 1.030369191 |
| mzb1 | -0.02888505071 | 0.05746780441 | -0.5026301424 | 0.6152243329 | 0.9715281345 |
| septin3 | 0.03115645098 | 0.06210416067 | 0.5016805741 | 0.6158922313 | 1.031646893 |
| dctn2 | -0.02821219484 | 0.05628396735 | -0.5012474452 | 0.6161969871 | 0.9721820529 |
| vwa5a | 0.03098782737 | 0.06192920774 | 0.5003750007 | 0.6168110527 | 1.031472948 |
| ccn1 | -0.02861164302 | 0.05720587337 | -0.5001521931 | 0.6169679177 | 0.9717937941 |
| glyr1 | 0.03067073199 | 0.0613548836 | 0.4998906394 | 0.6171520837 | 1.031145925 |
| phykpl | 0.03115619319 | 0.06239332877 | 0.4993513538 | 0.6175318831 | 1.031646627 |
| il34 | -0.02904020352 | 0.05819752789 | -0.4989937644 | 0.617783777 | 0.9713774109 |
| nmrk2 | -0.03032531795 | 0.06092202099 | -0.4977726848 | 0.6186442707 | 0.9701298815 |
| notch1 | 0.02882620628 | 0.05792491756 | 0.4976477739 | 0.6187323248 | 1.029245702 |
| hras | -0.02956899447 | 0.05952329385 | -0.4967634107 | 0.6193559002 | 0.9708638911 |
| shh | 0.03084698705 | 0.06211720573 | 0.4965932818 | 0.6194758917 | 1.031327685 |
| trim21 | 0.02847987572 | 0.05736010675 | 0.4965101589 | 0.6195345217 | 1.028889305 |
| c7orf50 | -0.03056318524 | 0.06156277898 | -0.4964555816 | 0.6195730187 | 0.9698991468 |
| ppp1r12b | 0.02992519292 | 0.06029001874 | 0.496354016 | 0.6196446623 | 1.030377452 |
| il36g | 0.03044578923 | 0.0613524482 | 0.4962440803 | 0.6197222143 | 1.030914002 |
| tsc22d1 | -0.03044030108 | 0.06135950025 | -0.4960976043 | 0.6198255494 | 0.9700183394 |
| akr1c4 | -0.02863256276 | 0.05778693224 | -0.4954850803 | 0.6202577511 | 0.9717734646 |
| nars1 | 0.02959161771 | 0.05974369092 | 0.4953095006 | 0.6203816656 | 1.0300338 |
| atxn10 | 0.028335166 | 0.05728429359 | 0.4946411001 | 0.6208534848 | 1.028740425 |
| map2k1 | 0.03017351439 | 0.06109815423 | 0.4938531248 | 0.6214099114 | 1.030633348 |
| tbcb | 0.02816209293 | 0.05717822943 | 0.4925317417 | 0.6223434885 | 1.028562394 |
| srpk2 | -0.02812505084 | 0.05721084316 | -0.4916035019 | 0.6229996674 | 0.9722667764 |
| adamts15 | -0.03120039135 | 0.06349486641 | -0.4913844712 | 0.6231545453 | 0.969281318 |
| tmprss11b | 0.02972914538 | 0.0605057646 | 0.4913440161 | 0.6231831532 | 1.030175468 |
| il17a | 0.0277029813 | 0.05642933965 | 0.4909322256 | 0.6234743836 | 1.028090277 |
| myh9 | -0.0292634032 | 0.05962893634 | -0.4907584304 | 0.6235973145 | 0.971160624 |
| cyp24a1 | -0.02938517695 | 0.06001281178 | -0.4896483946 | 0.6243827249 | 0.9710423693 |
| ceacam20 | -0.03024714348 | 0.06183458527 | -0.4891622278 | 0.6247268488 | 0.9702057239 |
| b4galt1 | 0.02869964283 | 0.05867645454 | 0.4891168537 | 0.6247589702 | 1.029115446 |
| sytl4 | 0.02953824376 | 0.06045508915 | 0.4885981341 | 0.6251262343 | 1.029978825 |
| arl2bp | 0.02964234657 | 0.06068588758 | 0.4884553518 | 0.6252273434 | 1.030086054 |
| mocs2 | -0.03079901288 | 0.06307417089 | -0.4882983389 | 0.6253385379 | 0.9696704448 |
| ddc | 0.02834408673 | 0.05817206137 | 0.4872456994 | 0.6260842234 | 1.028749603 |
| dffa | 0.02767509703 | 0.05683473034 | 0.4869398845 | 0.6263009332 | 1.02806161 |
| ppif | -0.02897157353 | 0.05954805362 | -0.4865242736 | 0.6265954995 | 0.9714440788 |
| tp53 | -0.02854742993 | 0.05871942731 | -0.4861666953 | 0.6268489825 | 0.971856198 |
| top2b | 0.03062091486 | 0.06299254926 | 0.4861037571 | 0.6268936033 | 1.031094557 |
| foxo1 | -0.02792224559 | 0.05752102576 | -0.4854267673 | 0.6273736488 | 0.9724639772 |
| fosb | -0.02784611362 | 0.05746472346 | -0.4845775277 | 0.6279760577 | 0.9725380156 |
| cd36 | 0.031145558 | 0.06428258093 | 0.4845100733 | 0.6280239171 | 1.031635656 |
| pdcd1 | -0.02711530087 | 0.0559654691 | -0.4845005555 | 0.6280306702 | 0.9732490186 |
| f2 | 0.02948283014 | 0.06105717994 | 0.4828724511 | 0.6291863036 | 1.029921752 |
| myh7b | -0.02974130976 | 0.06160065087 | -0.4828083688 | 0.6292318081 | 0.9706966108 |
| fcgr3b | 0.02813221757 | 0.05833281591 | 0.4822708648 | 0.6296135415 | 1.028531665 |
| fn1 | 0.03072086167 | 0.06370787293 | 0.4822145247 | 0.6296535598 | 1.031197617 |
| ccl14 | 0.028497933 | 0.05913495182 | 0.4819135237 | 0.6298673787 | 1.028907884 |
| sdccag8 | 0.02903740761 | 0.06025514995 | 0.4819074822 | 0.6298716706 | 1.029463104 |
| tgfa | 0.02878948463 | 0.05978513865 | 0.4815491822 | 0.6301262336 | 1.029207908 |
| cdkn2d | 0.02736365972 | 0.05689207705 | 0.4809748762 | 0.6305343549 | 1.027741483 |
| zp3 | 0.02907468688 | 0.06057476494 | 0.4799801849 | 0.6312414829 | 1.029501482 |
| clec1a | -0.02805048772 | 0.05844202341 | -0.4799711933 | 0.6312478766 | 0.9723392744 |
| frmd4b | -0.02909881353 | 0.06072110688 | -0.4792207359 | 0.6317816041 | 0.9713204801 |
| pm20d1 | 0.0280502403 | 0.0585965217 | 0.4787014567 | 0.6321510294 | 1.028447353 |
| dusp3 | -0.02748737434 | 0.057450672 | -0.4784517462 | 0.6323287109 | 0.9728869658 |
| mmp15 | -0.02858993053 | 0.05977897113 | -0.4782606657 | 0.6324646887 | 0.9718148944 |
| glt8d2 | 0.02723107797 | 0.05701065849 | 0.4776488939 | 0.6329001246 | 1.027605232 |
| eif4g3 | 0.02867723121 | 0.06004798061 | 0.4775719504 | 0.6329548991 | 1.029092382 |
| dkkl1 | -0.0264533505 | 0.05540053297 | -0.4774927077 | 0.6330113124 | 0.9738934744 |
| inpp5d | 0.03001201329 | 0.06312637465 | 0.4754274811 | 0.6344823108 | 1.030466913 |
| lztfl1 | 0.02940963421 | 0.06189126393 | 0.4751823173 | 0.6346570296 | 1.029846368 |
| ccn3 | -0.03013371583 | 0.06342806515 | -0.4750848975 | 0.6347264626 | 0.9703157783 |
| vasp | 0.02841866242 | 0.05993371676 | 0.4741681971 | 0.6353799702 | 1.028826325 |
| wwp2 | 0.02734076449 | 0.05771150688 | 0.4737489276 | 0.6356789584 | 1.027717953 |
| acaa1 | -0.02813878437 | 0.05948970467 | -0.4730025898 | 0.6362113315 | 0.9722534239 |
| acot13 | 0.02829224083 | 0.05988287723 | 0.4724596101 | 0.6365987646 | 1.028696268 |
| itgam | 0.02724549948 | 0.05768361893 | 0.4723264592 | 0.6366937871 | 1.027620052 |
| fos | 0.02924596907 | 0.06197733907 | 0.4718816508 | 0.637011266 | 1.029677832 |
| klk11 | 0.02680605172 | 0.05681640011 | 0.4718013051 | 0.6370686193 | 1.027168566 |

| Protein | beta | se | z | p_val | HR |
|---|---|---|---|---|---|
| tnr | -0.02819123396 | 0.05976652497 | -0.4716893608 | 0.6371485322 | 0.9722024309 |
| ddx25 | -0.02877190142 | 0.06102375954 | -0.4714868707 | 0.6372930933 | 0.9716380685 |
| il4 | -0.02644491351 | 0.05614048992 | -0.4710488553 | 0.637605847 | 0.9739016912 |
| nme1 | -0.02915724299 | 0.06190636163 | -0.4709894464 | 0.6376482714 | 0.971263728 |
| apoa2 | 0.02936879112 | 0.06248697382 | 0.4699986145 | 0.6383560075 | 1.029804307 |
| brd2 | -0.02872912743 | 0.06114756402 | -0.4698327381 | 0.6384745226 | 0.9716796302 |
| dpep2 | 0.02688505333 | 0.0572406324 | 0.4696847712 | 0.6385802497 | 1.027249717 |
| kifbp | 0.02660235243 | 0.05677019522 | 0.4685971631 | 0.6393576055 | 1.026959354 |
| blnk | -0.0296264791 | 0.06327914168 | -0.46818712 | 0.6396507821 | 0.9708080829 |
| eif2ak2 | 0.02846285421 | 0.0608472764 | 0.4677753204 | 0.6399452713 | 1.028871792 |
| arg2 | -0.0284115133 | 0.06074323706 | -0.4677313011 | 0.6399767541 | 0.9719882984 |
| ca7 | 0.02829111289 | 0.06050036648 | 0.4676188681 | 0.6400571695 | 1.028695107 |
| casp7 | 0.0281223012 | 0.06019196515 | 0.4672102187 | 0.6403494834 | 1.028521466 |
| il17d | -0.02977750284 | 0.0638110659 | -0.4666510803 | 0.6407495349 | 0.9706614789 |
| pear1 | 0.02655361204 | 0.0569224911 | 0.4664871746 | 0.6408668258 | 1.0269093 |
| mrps16 | 0.02862825978 | 0.06141345881 | 0.4661561216 | 0.6411037544 | 1.029041987 |
| ifnw1 | -0.02785636894 | 0.05977287427 | -0.4660369655 | 0.6411890413 | 0.972528042 |
| dock9 | -0.02876610958 | 0.06176848847 | -0.4657084914 | 0.6414241735 | 0.971643696 |
| sag | -0.02879933138 | 0.06188597268 | -0.4653612142 | 0.6416728047 | 0.9716114168 |
| gcg | -0.02689183867 | 0.05789161303 | -0.4645204592 | 0.6422749049 | 0.9734665273 |
| trim5 | -0.02673958825 | 0.05766346771 | -0.4637180057 | 0.6428497952 | 0.9736147492 |
| il33 | 0.02634143893 | 0.05681867222 | 0.4636053238 | 0.6429305394 | 1.026691441 |
| sh2b3 | 0.02654371952 | 0.0572704559 | 0.4634801504 | 0.6430202395 | 1.026899142 |
| eif5 | 0.02837859303 | 0.06126964185 | 0.4631754353 | 0.6432386224 | 1.028785102 |
| fmnl1 | -0.02743386941 | 0.05940720617 | -0.4617936305 | 0.644229319 | 0.9729390215 |
| rida | 0.03107628945 | 0.06743068744 | 0.4608627114 | 0.6448971059 | 1.031564198 |
| krt19 | 0.02709608425 | 0.0588300458 | 0.4605824096 | 0.6450982342 | 1.027466521 |
| ghrhr | 0.02627170459 | 0.05709100414 | 0.4601724035 | 0.6453924775 | 1.026619848 |
| aida | 0.02793506882 | 0.06070800224 | 0.4601546383 | 0.6454052281 | 1.028328912 |
| pkd1 | 0.0294893776 | 0.06411871324 | 0.4599184249 | 0.6455747746 | 1.029928495 |
| psmd1 | 0.02868629162 | 0.06237884194 | 0.4598721414 | 0.6456079976 | 1.029101706 |
| ereg | -0.0262035822 | 0.05707905688 | -0.4590752482 | 0.6461801305 | 0.9741367525 |
| thy1 | -0.02735793113 | 0.05965374632 | -0.458612121 | 0.6465127308 | 0.9730129076 |
| s100a13 | -0.02963948006 | 0.06466493851 | -0.4583547243 | 0.6466976139 | 0.9707954616 |
| fcgr2b | 0.02597245793 | 0.05673149491 | 0.4578137412 | 0.6470862627 | 1.026312681 |
| il25 | 0.0284066573 | 0.06211909377 | 0.4572934918 | 0.647460107 | 1.028813974 |
| rgs10 | 0.02734798763 | 0.05997820727 | 0.455965406 | 0.6484148549 | 1.027725376 |
| boc | 0.02779180006 | 0.06095684967 | 0.4559257936 | 0.6484433407 | 1.028181595 |
| serpinb8 | 0.02652909282 | 0.05823245835 | 0.4555722629 | 0.6486975926 | 1.026884122 |
| safb2 | -0.02719335128 | 0.0599048917 | -0.4539420823 | 0.6498705137 | 0.9731730591 |
| gp6 | 0.02565514562 | 0.05655982452 | 0.4535930908 | 0.6501217273 | 1.025987071 |
| prr4 | -0.02826343827 | 0.06234473409 | -0.4533412273 | 0.6503030503 | 0.9721322362 |
| asrgl1 | 0.02828105036 | 0.06240499507 | 0.4531856838 | 0.6504150404 | 1.028684756 |
| trpv3 | 0.02762141173 | 0.06113955503 | 0.4517764599 | 0.6514300297 | 1.02800642 |
| cd248 | 0.02808136827 | 0.06225780493 | 0.4510497649 | 0.6519536824 | 1.028479367 |
| tnfrsf12a | 0.02874109578 | 0.06385371189 | 0.4501084577 | 0.6526322386 | 1.029158107 |
| flt4 | 0.02658018974 | 0.05919276597 | 0.4490445632 | 0.6533995101 | 1.026936594 |
| naa80 | 0.02778494208 | 0.06189267881 | 0.4489213039 | 0.6534884273 | 1.028174544 |
| scin | -0.02745117894 | 0.06117245833 | -0.4487506256 | 0.65361156 | 0.9729221805 |
| ifnl1 | 0.02577498026 | 0.05750609119 | 0.4482130453 | 0.6539994488 | 1.026110027 |
| fzd10 | -0.02778657492 | 0.06202761124 | -0.4479710626 | 0.6541740809 | 0.972595921 |
| pam | 0.02535220561 | 0.05659572919 | 0.4479526278 | 0.6541873856 | 1.025676306 |
| brap | 0.02696114017 | 0.06029378008 | 0.447162877 | 0.6547574629 | 1.02732788 |
| rgl2 | -0.02734073393 | 0.06117648752 | -0.4469157194 | 0.6549359137 | 0.9730296408 |
| ryr1 | -0.0267874528 | 0.05997818733 | -0.4466199129 | 0.6551495155 | 0.9735681487 |
| lrp2 | 0.02764192799 | 0.06204580351 | 0.4455084216 | 0.6559523752 | 1.028027511 |
| ncam1 | 0.0274270907 | 0.06163532714 | 0.4449897806 | 0.6563271394 | 1.027806676 |
| sart1 | -0.02811013979 | 0.06324488121 | -0.4444650579 | 0.6567063862 | 0.972281274 |
| marco | 0.02592980803 | 0.05842798035 | 0.443790935 | 0.6571937429 | 1.02626891 |
| nell1 | 0.02480275239 | 0.05590913676 | 0.4436261017 | 0.6573129312 | 1.0251129 |
| lats1 | 0.02750244014 | 0.06209410928 | 0.4429154465 | 0.6578268943 | 1.027884123 |
| casp10 | 0.02580159604 | 0.05843307733 | 0.4415580561 | 0.6588090416 | 1.026137339 |
| nppb | -0.0251946695 | 0.05712125695 | -0.4410734435 | 0.6591598284 | 0.9751200674 |
| nfkb1 | 0.02700238183 | 0.06132181029 | 0.4403389546 | 0.6596916311 | 1.02737025 |
| apoh | 0.02459438953 | 0.05588983614 | 0.4400512012 | 0.6599000242 | 1.024899326 |
| txlna | -0.02530016973 | 0.05756428444 | -0.4395115822 | 0.6602908916 | 0.9750171974 |
| dusp13 | 0.02686952026 | 0.06115667603 | 0.4393554719 | 0.6604039857 | 1.027233761 |
| tpmt | 0.02501519609 | 0.05696240233 | 0.4391527581 | 0.6605508533 | 1.025330701 |
| shmt1 | -0.02546984859 | 0.05800094359 | -0.4391281765 | 0.6605686638 | 0.9748517717 |
| cxcl13 | -0.02553663394 | 0.05816072168 | -0.4390701009 | 0.6606107428 | 0.974786668 |

| Protein | beta | se | z | p_val | HR |
|---|---|---|---|---|---|
| lypd8 | -0.02496630076 | 0.05699520194 | -0.4380421493 | 0.6613557295 | 0.9753427798 |
| ptgds | -0.02632228683 | 0.06022539052 | -0.4370629498 | 0.662065696 | 0.9740211248 |
| bid | -0.02550568787 | 0.05838764016 | -0.4368336826 | 0.6622319697 | 0.9748168343 |
| ncln | -0.02493876408 | 0.05739485752 | -0.4345121698 | 0.6639165594 | 0.9753696379 |
| adgrb3 | 0.02484262736 | 0.05727513176 | 0.4337419505 | 0.6644758397 | 1.025153777 |
| cst5 | 0.0255304397 | 0.05893913625 | 0.4331661664 | 0.6648940566 | 1.025859133 |
| ppt1 | 0.0266786346 | 0.06176111131 | 0.4319649377 | 0.6657668966 | 1.027037695 |
| gad1 | 0.02690594281 | 0.06239144196 | 0.4312441253 | 0.6662908729 | 1.027271176 |
| dctd | 0.02646534793 | 0.06138643616 | 0.4311269652 | 0.6663760548 | 1.026818665 |
| pappa | 0.0261735796 | 0.06074424858 | 0.4308816096 | 0.666554456 | 1.026519116 |
| sirt1 | 0.02640638954 | 0.0615894363 | 0.4287486803 | 0.6681061298 | 1.026758127 |
| tacstd2 | 0.02642252441 | 0.06171137423 | 0.4281629561 | 0.6685324841 | 1.026774694 |
| clint1 | 0.02621985216 | 0.06124632286 | 0.4281049202 | 0.6685747348 | 1.026566617 |
| rras | 0.02633705546 | 0.06153498759 | 0.4280013125 | 0.6686501648 | 1.026686941 |
| egf | -0.02393818956 | 0.05594931278 | -0.4278549347 | 0.6687567387 | 0.9763460563 |
| cgb3_cgb5_cgb8 | -0.02749848701 | 0.06439971199 | -0.4269970496 | 0.6693814767 | 0.9728761545 |
| clec7a | 0.02373050936 | 0.05568664631 | 0.4261436256 | 0.670003193 | 1.024014318 |
| gtpbp2 | 0.02615575897 | 0.06141789175 | 0.4258654641 | 0.6702058816 | 1.026500823 |
| lmnb2 | 0.02794322557 | 0.06561615284 | 0.4258589442 | 0.6702106328 | 1.028337299 |
| myl6b | 0.02608603534 | 0.06130668328 | 0.4255006786 | 0.670471727 | 1.026429254 |
| agxt | -0.02538975108 | 0.05973733411 | -0.4250231695 | 0.6708197846 | 0.974929858 |
| sirt5 | -0.02418445449 | 0.05694754471 | -0.4246794943 | 0.6710703341 | 0.9761056461 |
| crybb1 | 0.02612072013 | 0.06154886866 | 0.4243899311 | 0.6712814627 | 1.026464856 |
| rpa2 | -0.02613477638 | 0.06165403923 | -0.423893985 | 0.671643131 | 0.9742037811 |
| ccl16 | -0.02596708933 | 0.06128233514 | -0.4237287837 | 0.6717636209 | 0.9743671562 |
| cdc42bpb | 0.02533579613 | 0.05987064661 | 0.4231755887 | 0.6721671559 | 1.025659475 |
| krt5 | -0.02574678142 | 0.06085482205 | -0.4230853128 | 0.6722330178 | 0.9745818406 |
| agr3 | -0.02464981669 | 0.05834767294 | -0.4224644352 | 0.6726860545 | 0.9756515091 |
| slamf1 | -0.02445241916 | 0.05789963574 | -0.4223242314 | 0.6727883738 | 0.9758441193 |
| il36a | 0.02585656861 | 0.06125109912 | 0.4221404837 | 0.67292248 | 1.02619375 |
| dnaja1 | -0.02599821781 | 0.06159665465 | -0.4220719122 | 0.6729725288 | 0.9743368261 |
| prkd2 | -0.02526974408 | 0.05990089111 | -0.4218592346 | 0.6731277666 | 0.9750468634 |
| calb1 | 0.02424023494 | 0.05759838776 | 0.4208491917 | 0.6738652083 | 1.024536418 |
| kiaa1549l | -0.02549390323 | 0.06075033951 | -0.4196503827 | 0.6747408767 | 0.9748283223 |
| sult2a1 | 0.02521564886 | 0.06010663776 | 0.4195152117 | 0.6748396399 | 1.025536252 |
| ccl25 | -0.0246871667 | 0.05888860524 | -0.4192180575 | 0.6750567762 | 0.9756150692 |
| ica1 | -0.02430574273 | 0.0580033596 | -0.4190402573 | 0.6751867113 | 0.9759872631 |
| cilp | -0.02619483607 | 0.0626102134 | -0.4183796006 | 0.6756695989 | 0.9741452725 |
| stk11 | -0.02459595929 | 0.05886566879 | -0.4178319858 | 0.6760699631 | 0.9757040566 |
| il22 | 0.02629582321 | 0.06295656963 | 0.4176819571 | 0.6761796659 | 1.026644609 |
| prss27 | 0.02318767552 | 0.05557865736 | 0.4172046722 | 0.676528708 | 1.0234586 |
| ppp1cc | 0.02516004462 | 0.06038378861 | 0.4166688642 | 0.6769206314 | 1.02547923 |
| cpb1 | 0.02330319071 | 0.05593097781 | 0.416641933 | 0.6769403329 | 1.023576831 |
| ccdc80 | 0.02574687658 | 0.06179661397 | 0.4166389536 | 0.6769425125 | 1.02608119 |
| serpina12 | 0.02509373411 | 0.06026089792 | 0.4164181912 | 0.6771040194 | 1.025411232 |
| ecm1 | -0.02522393133 | 0.06060955705 | -0.4161708575 | 0.6772849831 | 0.975091534 |
| ccl28 | -0.02486498693 | 0.05974790806 | -0.4161649794 | 0.677289284 | 0.9754416005 |
| cacybp | 0.02558742237 | 0.06157007486 | 0.4155821221 | 0.677715811 | 1.02591759 |
| slc28a1 | 0.02569941563 | 0.06184820784 | 0.4155240149 | 0.6777583387 | 1.026032493 |
| cd83 | -0.02468529054 | 0.05954478644 | -0.4145667827 | 0.6784590692 | 0.9756168996 |
| clul1 | 0.02485089299 | 0.05996464527 | 0.4144257483 | 0.6785623353 | 1.02516225 |
| tex101 | -0.0364398736 | 0.08820352507 | -0.4131339827 | 0.6795084529 | 0.964216067 |
| hrg | -0.02519381677 | 0.06102761872 | -0.4128264759 | 0.6797337522 | 0.9751208989 |
| ifngr1 | 0.02379330737 | 0.05766520308 | 0.412611178 | 0.6798915103 | 1.024078627 |
| hmox2 | -0.02443325999 | 0.05927148663 | -0.4122262049 | 0.6801736317 | 0.9758628158 |
| enox2 | 0.02504009378 | 0.06077277988 | 0.4120281124 | 0.6803188181 | 1.02535623 |
| dipk1c | -0.02530225765 | 0.06152253938 | -0.4112680963 | 0.6808759606 | 0.9750151617 |
| cep290 | 0.02480953148 | 0.06038380242 | 0.4108640146 | 0.6811722504 | 1.025119849 |
| atp5if1 | -0.0230459061 | 0.05618678498 | -0.4101659511 | 0.681684216 | 0.9772176225 |
| aldh2 | 0.02477984739 | 0.06046746747 | 0.4098046178 | 0.6819492785 | 1.02508942 |
| eif2ak3 | 0.02476166555 | 0.06045680402 | 0.4095761585 | 0.6821168891 | 1.025070782 |
| khk | -0.02664983011 | 0.06511006504 | -0.4093043079 | 0.6823163545 | 0.973702143 |
| app | -0.02327548266 | 0.05689506722 | -0.4090949146 | 0.6824700081 | 0.976993302 |
| grn | 0.02378137378 | 0.05813620933 | 0.4090630273 | 0.6824934083 | 1.024066406 |
| pdzd2 | -0.02510184195 | 0.06141186326 | -0.4087458126 | 0.6827262098 | 0.9752105896 |
| ezr | -0.02439925693 | 0.05979137256 | -0.4080732032 | 0.6832199325 | 0.9758959987 |
| ipcef1 | -0.02362372535 | 0.0579489205 | -0.4076646321 | 0.6835199067 | 0.9766531304 |
| amy2b | -0.0243627168 | 0.0597794417 | -0.407543398 | 0.6836089267 | 0.9759316587 |
| dok1 | 0.02456813946 | 0.0603125756 | 0.4073468793 | 0.6837532362 | 1.024872423 |
| sbsn | 0.02414461376 | 0.05930851223 | 0.4071019968 | 0.6839330769 | 1.024438455 |

| Protein | beta | se | z | p_val | HR |
|---|---|---|---|---|---|
| sult1a1 | 0.02281303 | 0.05604910508 | 0.4070186306 | 0.6839943047 | 1.023075237 |
| fgfr4 | -0.02472740822 | 0.06086194419 | -0.4062868603 | 0.6845318388 | 0.9755758097 |
| lama4 | -0.02340414818 | 0.05767247828 | -0.4058113832 | 0.6848811941 | 0.9768676047 |
| zhx2 | 0.02522769482 | 0.06238349199 | 0.4043969649 | 0.6859208317 | 1.025548606 |
| col9a2 | -0.02446453359 | 0.06051791655 | -0.4042527402 | 0.6860268744 | 0.9758322976 |
| hgfac | 0.02477658735 | 0.06140934763 | 0.4034660569 | 0.6866054002 | 1.025086078 |
| fcgr2a | 0.02254611579 | 0.05607963529 | 0.4020374897 | 0.687656436 | 1.0228022 |
| rhoc | 0.02294012725 | 0.05708876625 | 0.4018325978 | 0.6878072301 | 1.023205276 |
| arhgef1 | 0.02427761861 | 0.06048718975 | 0.4013679378 | 0.6881492514 | 1.024574719 |
| cxcl3 | -0.02280806242 | 0.05684290374 | -0.4012473136 | 0.6882380495 | 0.9774500752 |
| serpinc1 | 0.02545926648 | 0.06349241942 | 0.4009811992 | 0.6884339659 | 1.025786122 |
| sat1 | -0.02390707468 | 0.05963091822 | -0.4009174333 | 0.6884809143 | 0.9763764356 |
| otoa | -0.02422350581 | 0.06062602409 | -0.3995562331 | 0.689483398 | 0.9760675286 |
| plin1 | 0.02576818304 | 0.06450613384 | 0.3994687251 | 0.6895478637 | 1.026103053 |
| hspa1a | 0.02326382749 | 0.05828520095 | 0.3991378104 | 0.6897916636 | 1.023536541 |
| galnt3 | 0.02300290486 | 0.05773196397 | 0.3984431376 | 0.6903035653 | 1.023269512 |
| map2 | 0.02422245413 | 0.06081512948 | 0.398296515 | 0.6904116291 | 1.024518201 |
| c9 | 0.02420015635 | 0.06097186245 | 0.3969069564 | 0.6914360742 | 1.024495357 |
| nubp1 | 0.02375328444 | 0.06016647024 | 0.39479272 | 0.6929958681 | 1.024037641 |
| cd27 | 0.02304283967 | 0.0583766956 | 0.3947266873 | 0.6930446052 | 1.023310377 |
| klrc1 | -0.02415507607 | 0.06120121528 | -0.3946829481 | 0.6930768887 | 0.9761343229 |
| gbp2 | 0.02290535586 | 0.05810653428 | 0.3941958705 | 0.6934364343 | 1.023169698 |
| csf2rb | 0.02439484577 | 0.06195800635 | 0.3937319356 | 0.6937789608 | 1.024694834 |
| ambn | 0.02270362294 | 0.05772095146 | 0.393334177 | 0.6940726787 | 1.022963312 |
| ctsv | 0.02316209232 | 0.05889316936 | 0.3932899617 | 0.6941053315 | 1.023432417 |
| atraid | 0.02442804727 | 0.06223704577 | 0.3925001094 | 0.6946887305 | 1.024728856 |
| atp6v1g2 | 0.02414041026 | 0.06153073742 | 0.3923309109 | 0.6948137271 | 1.024434149 |
| metap1d | -0.02241975527 | 0.05716569185 | -0.3921889955 | 0.6949185745 | 0.9778296997 |
| cnpy4 | 0.02234409645 | 0.05703370462 | 0.3917700349 | 0.6952281374 | 1.022595595 |
| gpi | 0.02386663158 | 0.06094573196 | 0.3916046425 | 0.6953503571 | 1.024153719 |
| map4k5 | 0.02230851186 | 0.0569771853 | 0.3915341156 | 0.6954024766 | 1.022559207 |
| syt1 | -0.0239775524 | 0.06125110794 | -0.3914631621 | 0.6954549129 | 0.9763076253 |
| kir2ds4 | -0.02319926579 | 0.05928754766 | -0.3913008161 | 0.6955748957 | 0.9770677682 |
| msln | 0.02353668706 | 0.06019229633 | 0.3910249068 | 0.6957788257 | 1.023815861 |
| adam23 | -0.02177535707 | 0.05583732739 | -0.389978498 | 0.6965524468 | 0.9784600145 |
| bnip2 | -0.0239057914 | 0.06134316108 | -0.3897058935 | 0.696754038 | 0.9763776886 |
| dnajb6 | 0.02404262619 | 0.06174907757 | 0.3893600866 | 0.6970097934 | 1.02433398 |
| vim | 0.02254330232 | 0.05794544389 | 0.3890435694 | 0.6972439165 | 1.022799323 |
| fshb | -0.03679745021 | 0.09485600725 | -0.3879295712 | 0.6980681538 | 0.9638713475 |
| aif1l | -0.02551354264 | 0.06587313633 | -0.3873133126 | 0.6985242712 | 0.9748091774 |
| mansc4 | 0.02367088651 | 0.06124485496 | 0.3864959191 | 0.6991294243 | 1.023953266 |
| adam8 | 0.02250980851 | 0.0583141842 | 0.3860091472 | 0.6994898944 | 1.022765066 |
| csnk1d | 0.02378766438 | 0.06192117944 | 0.384160389 | 0.700859575 | 1.024072848 |
| gfral | -0.02498953912 | 0.06512722229 | -0.3837034383 | 0.701198264 | 0.9753201147 |
| pbk | 0.02317665737 | 0.06041671038 | 0.3836133617 | 0.7012650352 | 1.023447323 |
| nrn1 | -0.02312087558 | 0.06028204969 | -0.3835449475 | 0.7013157502 | 0.9771443637 |
| fzd8 | -0.02346395344 | 0.06118467834 | -0.3834939413 | 0.7013535617 | 0.9768091846 |
| tarbp2 | -0.02241784226 | 0.05859925053 | -0.382561928 | 0.7020446036 | 0.9778315703 |
| fabp6 | 0.02178987617 | 0.05709888086 | 0.3816165194 | 0.7027458293 | 1.022029009 |
| igsf21 | 0.02420039278 | 0.06350782517 | 0.3810615891 | 0.7031575484 | 1.024495599 |
| inpp5j | -0.02334973796 | 0.06128451309 | -0.381005523 | 0.7031991504 | 0.9769207577 |
| relt | -0.02431683771 | 0.06383462185 | -0.3809349379 | 0.7032515269 | 0.9759764346 |
| ly96 | -0.022383418 | 0.05879521122 | -0.3807013792 | 0.7034248453 | 0.977865232 |
| pamr1 | -0.02351971723 | 0.06181533369 | -0.380483544 | 0.7035865097 | 0.9767547156 |
| zp4 | -0.02314726742 | 0.06090716221 | -0.3800417977 | 0.7039143886 | 0.9771185754 |
| ager | -0.0222409748 | 0.05857088469 | -0.379727486 | 0.7041477149 | 0.9780045322 |
| prss22 | 0.02374698137 | 0.06265611246 | 0.3790050234 | 0.7046841335 | 1.024031186 |
| chp1 | 0.02309715606 | 0.06095889969 | 0.3788971943 | 0.7047642078 | 1.023365961 |
| il1r2 | -0.02205126106 | 0.05826547546 | -0.3784618745 | 0.7050875109 | 0.9781900907 |
| igdcc4 | -0.02391559302 | 0.06319333236 | -0.3784512088 | 0.7050954328 | 0.9763681186 |
| cpa1 | 0.0210421743 | 0.05582987986 | 0.376898076 | 0.7062493513 | 1.021265122 |
| saa4 | -0.02348736304 | 0.06232790425 | -0.3768354371 | 0.7062959038 | 0.9767863182 |
| reg3a | 0.02216929763 | 0.05892328386 | 0.3762400222 | 0.7067384642 | 1.022416863 |
| rfc4 | 0.0230840468 | 0.06162243111 | 0.3746046104 | 0.7079545437 | 1.023352545 |
| pdia4 | 0.02281702332 | 0.06093093459 | 0.374473549 | 0.7080520323 | 1.023079323 |
| cd28 | 0.02192657585 | 0.05860581852 | 0.3741365005 | 0.708302764 | 1.02216873 |
| ssc5d | -0.0228117221 | 0.06109450571 | -0.3733841829 | 0.7088625299 | 0.977446498 |
| myoc | 0.02197669654 | 0.05889861098 | 0.3731275862 | 0.7090534881 | 1.022219963 |
| dlgap5 | -0.02265347161 | 0.06074741364 | -0.3729125283 | 0.7092135473 | 0.9776011916 |
| enpp6 | 0.02460738453 | 0.06619451641 | 0.3717435502 | 0.7100837966 | 1.024912645 |

| Protein | beta | se | z | p_val | HR |
|---|---|---|---|---|---|
| axin1 | -0.02119940388 | 0.0571567828 | -0.3708991802 | 0.7107126258 | 0.979023724 |
| pdcd1lg2 | 0.02135134748 | 0.05781321329 | 0.3693160485 | 0.7118921648 | 1.021580918 |
| mmp13 | -0.02147901745 | 0.05826169109 | -0.368664504 | 0.7123778096 | 0.9787500139 |
| osmr | -0.02205546458 | 0.0599986815 | -0.3675991542 | 0.7131721456 | 0.9781859789 |
| ctsz | -0.0215810485 | 0.05890774485 | -0.3663533301 | 0.7141014397 | 0.9786501561 |
| rab6a | -0.02171194448 | 0.05928242322 | -0.3662459006 | 0.7141815942 | 0.9785220631 |
| clec4m | -0.02198296892 | 0.0600833962 | -0.3658742733 | 0.7144588943 | 0.9782568957 |
| rnf149 | 0.02323925905 | 0.0636179691 | 0.3652939472 | 0.7148919961 | 1.023511395 |
| ddx39a | -0.02251145284 | 0.0616315006 | -0.3652588793 | 0.7149181705 | 0.9777400392 |
| klf4 | 0.02308480168 | 0.0632374593 | 0.3650494807 | 0.7150744709 | 1.023353318 |
| hmgcl | 0.02271065748 | 0.06227182064 | 0.3647019992 | 0.7153338661 | 1.022970508 |
| plekho1 | 0.02197815756 | 0.06032583348 | 0.3643241426 | 0.7156159737 | 1.022221456 |
| col1a1 | 0.02043008631 | 0.05610747338 | 0.3641241546 | 0.7157653004 | 1.020640209 |
| adgrf5 | 0.02221213452 | 0.06115877133 | 0.3631880438 | 0.7164644184 | 1.022460661 |
| ifnl2 | -0.02202283551 | 0.06064921646 | -0.3631182197 | 0.7165165749 | 0.9782178967 |
| camlg | -0.02230255796 | 0.06144731504 | -0.3629541494 | 0.7166391355 | 0.9779443054 |
| insl4 | -0.02232723325 | 0.06161672164 | -0.3623567217 | 0.7170854761 | 0.9779201747 |
| fabp2 | 0.02056306027 | 0.05675258878 | 0.3623281459 | 0.7171068276 | 1.020775937 |
| smtn | 0.02219950083 | 0.06127223513 | 0.3623093034 | 0.7171209067 | 1.022447743 |
| apcs | 0.0255657311 | 0.07072891499 | 0.3614608128 | 0.7177549944 | 1.025895337 |
| tlr1 | 0.02248253666 | 0.0622011966 | 0.3614486197 | 0.7177641078 | 1.022737174 |
| crim1 | 0.02156315378 | 0.05969371155 | 0.3612299055 | 0.717927588 | 1.021797319 |
| gm2a | -0.02300259223 | 0.06376072317 | -0.3607642933 | 0.7182756575 | 0.9772599505 |
| clu | -0.02260435281 | 0.06283025804 | -0.3597685815 | 0.7190202004 | 0.9776492114 |
| ly9 | 0.02048919187 | 0.05695273133 | 0.3597578446 | 0.7190282304 | 1.020700536 |
| ccl13 | -0.02050708318 | 0.05701388639 | -0.3596857622 | 0.7190821406 | 0.979701757 |
| ccn5 | 0.02417941239 | 0.06722601416 | 0.3596734493 | 0.7190913495 | 1.024474105 |
| mtdh | 0.02233225823 | 0.06209475815 | 0.3596480427 | 0.7191103514 | 1.02258349 |
| rexo2 | -0.02149934118 | 0.05984532236 | -0.3592484814 | 0.7194092111 | 0.9787301223 |
| klhl41 | 0.02272254341 | 0.06333338675 | 0.3587766986 | 0.7197621456 | 1.022982667 |
| twf2 | 0.0216403713 | 0.06037672617 | 0.3584224033 | 0.7200272284 | 1.021876222 |
| mycbp2 | -0.02152401596 | 0.06006230778 | -0.3583614542 | 0.7200728338 | 0.9787059726 |
| micall2 | -0.02207653954 | 0.06162055877 | -0.3582658123 | 0.7201444002 | 0.9781653639 |
| cdon | -0.02112100181 | 0.05896402763 | -0.3582014773 | 0.7201925418 | 0.9791004845 |
| tet2 | -0.02128010027 | 0.05977416587 | -0.3560083183 | 0.7218343357 | 0.9789447235 |
| serpinb6 | -0.02059876226 | 0.0578681088 | -0.3559605228 | 0.7218701296 | 0.979611943 |
| fgf16 | 0.02203060084 | 0.06189785759 | 0.3559186326 | 0.7219015015 | 1.022275066 |
| cd226 | -0.02216273215 | 0.06230676813 | -0.3557034462 | 0.7220626639 | 0.9780810569 |
| med21 | 0.02146470042 | 0.06048341861 | 0.3548857011 | 0.722675221 | 1.021696724 |
| myl3 | -0.02446390366 | 0.06930552467 | -0.3529863424 | 0.7240986795 | 0.9758329123 |
| jam3 | -0.02208571156 | 0.06260427859 | -0.3527827819 | 0.7242512929 | 0.9781563921 |
| mnat1 | 0.0215099483 | 0.06101702953 | 0.3525236883 | 0.7244455566 | 1.021742955 |
| tpm3 | -0.02148118935 | 0.06112464707 | -0.3514325297 | 0.7252638816 | 0.9787478882 |
| kiaa0319 | -0.02182795664 | 0.06218014101 | -0.3510438589 | 0.725555445 | 0.9784085493 |
| rgmb | -0.02035968374 | 0.05804331768 | -0.3507670572 | 0.7257631133 | 0.9798461752 |
| cant1 | -0.02028350919 | 0.05783306992 | -0.3507250993 | 0.7257945938 | 0.9799208174 |
| hyou1 | 0.01997331672 | 0.05700610188 | 0.3503715579 | 0.7260598688 | 1.020174118 |
| nexn | -0.02085270752 | 0.05956545772 | -0.3500805386 | 0.7262782559 | 0.9793632068 |
| tek | -0.02011197621 | 0.05749674333 | -0.3497933108 | 0.7264938195 | 0.9800889205 |
| xpnpep2 | 0.0204530647 | 0.05854789682 | 0.3493390165 | 0.7268348103 | 1.020663662 |
| sez6l2 | -0.02270811566 | 0.06504519513 | -0.3491128839 | 0.7270045644 | 0.977547773 |
| icam5 | -0.02045888295 | 0.05870462742 | -0.348505456 | 0.727460617 | 0.97974898 |
| ca6 | 0.02191427679 | 0.0629835373 | 0.3479365835 | 0.7278878101 | 1.022156158 |
| serpina4 | 0.02140050839 | 0.06160949394 | 0.347357315 | 0.7283228969 | 1.021631142 |
| itih1 | -0.02091117592 | 0.06025030091 | -0.3470717258 | 0.7285374343 | 0.9793059467 |
| reck | -0.02289519413 | 0.06618158589 | -0.3459450816 | 0.7293839877 | 0.977364912 |
| frmd7 | -0.02096457616 | 0.06064975781 | -0.3456662799 | 0.7295935286 | 0.9792536529 |
| map3k5 | 0.01984238067 | 0.0574286079 | 0.3455138718 | 0.7297080834 | 1.020040549 |
| prkg1 | 0.02107355534 | 0.06120987245 | 0.3442836016 | 0.7306330154 | 1.021297171 |
| wfdc2 | 0.02352765788 | 0.06841596481 | 0.343891341 | 0.7309280041 | 1.023806617 |
| serpine2 | -0.02085306869 | 0.06066758611 | -0.3437266921 | 0.7310518356 | 0.9793628531 |
| b2m | 0.0224211179 | 0.06523819132 | 0.3436808631 | 0.7310863045 | 1.02267436 |
| tspan7 | -0.02086117761 | 0.06077198837 | -0.3432696242 | 0.7313956304 | 0.9793549115 |
| mad1l1 | 0.02127947272 | 0.06199735564 | 0.3432319412 | 0.731423977 | 1.021507495 |
| arhgap25 | -0.01980291247 | 0.05782690788 | -0.3424515194 | 0.7320111226 | 0.9803918773 |
| ddx4 | -0.0211863374 | 0.06192232439 | -0.3421437682 | 0.7322427006 | 0.9790365164 |
| cd3g | -0.02088403022 | 0.061048613 | -0.3420885291 | 0.7322842697 | 0.979332531 |
| ecscr | 0.02053654603 | 0.06008142739 | 0.3418118863 | 0.7324924638 | 1.020748872 |
| cd244 | -0.01932751823 | 0.05656692561 | -0.3416752461 | 0.7325953029 | 0.9808580607 |
| wfdc12 | 0.02111161085 | 0.06185511613 | 0.3413074322 | 0.7328721534 | 1.021336037 |

| Protein | beta | se | z | p_val | HR |
|---|---|---|---|---|---|
| nfkbie | 0.01972276543 | 0.05782658147 | 0.3410674629 | 0.7330527951 | 1.019918544 |
| sla2 | 0.0207451729 | 0.06083898434 | 0.3409848657 | 0.7331149751 | 1.02096185 |
| col2a1 | 0.0210803307 | 0.0618338375 | 0.3409190106 | 0.7331645528 | 1.02130409 |
| hsd11b1 | 0.02053093603 | 0.06036146622 | 0.3401331565 | 0.7337562535 | 1.020743145 |
| sarg | 0.02053152547 | 0.06054118584 | 0.3391331898 | 0.7345093965 | 1.020743747 |
| nos3 | -0.02059563756 | 0.06080720393 | -0.3387039073 | 0.7348327969 | 0.979615004 |
| cpxm2 | 0.02149147427 | 0.06353981005 | 0.3382363632 | 0.7351850751 | 1.021724079 |
| nfu1 | 0.02026445948 | 0.05997062921 | 0.3379064009 | 0.7354337237 | 1.020471178 |
| procr | -0.02039619165 | 0.0605219079 | -0.3370051004 | 0.7361130552 | 0.9798104037 |
| ern1 | -0.02098341932 | 0.06229000446 | -0.3368665567 | 0.7362174972 | 0.9792352008 |
| anxa4 | 0.0190723423 | 0.05664323952 | 0.3367099492 | 0.7363355625 | 1.019255381 |
| fgf6 | 0.02028498305 | 0.06036218621 | 0.3360544793 | 0.7368297844 | 1.020492122 |
| notch2 | 0.02133932818 | 0.0635055565 | 0.3360230089 | 0.7368535157 | 1.02156864 |
| defb103a_defb103b | -0.0210598513 | 0.06274669162 | -0.3356328558 | 0.7371477444 | 0.9791603588 |
| prame | 0.02047178486 | 0.06127533137 | 0.3340950494 | 0.7383078357 | 1.020682769 |
| gga1 | 0.01932297714 | 0.05793274194 | 0.3335415602 | 0.7387255231 | 1.019510874 |
| fut1 | -0.02036832097 | 0.06123866333 | -0.3326055773 | 0.7394320328 | 0.9798377121 |
| lrig3 | 0.02116742533 | 0.06368955983 | 0.3323531422 | 0.7396226165 | 1.021393044 |
| vegfd | -0.01968840755 | 0.0592512838 | -0.3322865984 | 0.7396728585 | 0.9805041434 |
| vit | 0.02256129418 | 0.0679182558 | 0.332183062 | 0.7397510329 | 1.022817725 |
| kif1c | 0.02127563743 | 0.06410965219 | 0.3318632484 | 0.7399925227 | 1.021503577 |
| pdia5 | -0.020641601 | 0.06222492541 | -0.3317256045 | 0.7400964648 | 0.9795699786 |
| nrgn | 0.01981121536 | 0.05978508345 | 0.3313738849 | 0.7403620884 | 1.02000876 |
| fgd3 | 0.02014357876 | 0.06078858513 | 0.3313710743 | 0.7403642112 | 1.02034783 |
| kir2dl2 | 0.01974378047 | 0.05966694814 | 0.3308997876 | 0.7407201824 | 1.019939978 |
| nptn | 0.01915838 | 0.05797294389 | 0.3304710563 | 0.741044059 | 1.019343079 |
| hif1a | -0.02053304544 | 0.06223611647 | -0.3299217014 | 0.7414591254 | 0.9796763221 |
| mapre3 | 0.02024784118 | 0.06146074574 | 0.3294434673 | 0.7418205176 | 1.020454219 |
| pdcd6 | 0.0187014942 | 0.0568825468 | 0.3287738551 | 0.7423266261 | 1.018877462 |
| gnly | -0.01853894015 | 0.05659848573 | -0.3275518754 | 0.7432505139 | 0.981631849 |
| lamp1 | 0.02024252907 | 0.06190365484 | 0.3270005482 | 0.7436674706 | 1.020448799 |
| shpk | -0.01967605514 | 0.06025794441 | -0.3265304738 | 0.7440230369 | 0.9805162551 |
| sost | 0.02002571148 | 0.06133206235 | 0.3265129317 | 0.7440363069 | 1.020227571 |
| cd300a | -0.02012644095 | 0.06167000551 | -0.3263570481 | 0.7441542307 | 0.9800747439 |
| s100g | -0.02013060802 | 0.06178655474 | -0.3258088771 | 0.7445689619 | 0.9800706599 |
| hs3st3b1 | 0.01865698595 | 0.05727537551 | 0.3257418355 | 0.7446196889 | 1.018832115 |
| mavs | 0.01870234444 | 0.05749625618 | 0.3252793431 | 0.7449696633 | 1.018878329 |
| pard3 | 0.01961187933 | 0.06030890304 | 0.3251904502 | 0.7450369359 | 1.019805456 |
| sord | 0.01967269743 | 0.06056717389 | 0.3248079144 | 0.7453264542 | 1.01986748 |
| akr7l | -0.020988293 | 0.06465320665 | -0.3246288017 | 0.7454620262 | 0.9792304283 |
| rapgef2 | -0.01987169667 | 0.06143076777 | -0.3234811706 | 0.7463308653 | 0.9803244441 |
| gcc1 | 0.01954970956 | 0.06056266681 | 0.3228013328 | 0.7468457036 | 1.019742057 |
| dab2 | -0.01829481481 | 0.05670613193 | -0.3226249822 | 0.7469792716 | 0.9818715194 |
| omd | 0.01939579339 | 0.06014308716 | 0.3224941436 | 0.7470783737 | 1.019585114 |
| nos1 | -0.02068305456 | 0.06426595831 | -0.3218353092 | 0.7475774634 | 0.9795293727 |
| masp1 | 0.01863980923 | 0.05794631167 | 0.321673782 | 0.7476998419 | 1.018814615 |
| gata3 | -0.01979688293 | 0.06181353054 | -0.3202677917 | 0.7487653368 | 0.9803977886 |
| ap1g2 | -0.01958016134 | 0.06120634949 | -0.3199040867 | 0.7490410398 | 0.980610285 |
| cdcp1 | 0.02107347977 | 0.06598902577 | 0.3193482481 | 0.7494624498 | 1.021297094 |
| selenop | 0.01921438863 | 0.0601848757 | 0.319256099 | 0.74953232 | 1.019400173 |
| lrp11 | -0.01857837394 | 0.05822710422 | -0.3190674547 | 0.7496753622 | 0.9815931403 |
| erbin | 0.01812541577 | 0.05695955451 | 0.3182155467 | 0.7503214407 | 1.018290678 |
| il32 | 0.01851029919 | 0.05825708662 | 0.3177347214 | 0.7506861711 | 1.018682677 |
| cxcl11 | 0.01835363008 | 0.05777100875 | 0.3176962023 | 0.7507153922 | 1.018523093 |
| kiaa1549 | 0.01883308955 | 0.05943514739 | 0.3168678867 | 0.7513438501 | 1.019011551 |
| fcrl3 | -0.01749910171 | 0.05527969795 | -0.3165556681 | 0.7515807787 | 0.9826531184 |
| tgfb2 | -0.01901027416 | 0.06009104147 | -0.3163578746 | 0.7517308874 | 0.9811692815 |
| afap1 | 0.02020831157 | 0.06392079656 | 0.3161461161 | 0.7518916048 | 1.020413882 |
| crybb2 | 0.01936437688 | 0.0614636387 | 0.3150541895 | 0.75272051 | 1.019553083 |
| trim26 | 0.01904344933 | 0.06053998041 | 0.3145598859 | 0.7530958404 | 1.019225932 |
| stab2 | -0.01985014537 | 0.0631972039 | -0.3140984751 | 0.7534462477 | 0.9803455716 |
| cep112 | -0.01949446506 | 0.06209246513 | -0.3139586263 | 0.7535524625 | 0.9806943233 |
| hexim1 | -0.01793901125 | 0.05717257206 | -0.3137695333 | 0.7536960856 | 0.982220935 |
| vsig2 | 0.01935513791 | 0.06171568234 | 0.3136178225 | 0.7538113216 | 1.019543663 |
| hepacam2 | -0.01900605593 | 0.06100520463 | -0.3115481056 | 0.7553839782 | 0.9811734203 |
| scgb3a1 | 0.0199686763 | 0.06417502571 | 0.3111596151 | 0.7556792826 | 1.020169384 |
| slc34a3 | -0.0189638612 | 0.06104581812 | -0.3106496363 | 0.7560669884 | 0.9812148215 |
| dnajb8 | 0.01773822626 | 0.05714158297 | 0.3104258815 | 0.756237115 | 1.017896483 |
| gadd45gip1 | -0.01934956999 | 0.06233673695 | -0.3104039599 | 0.7562537832 | 0.9808364313 |
| clpp | 0.01760439715 | 0.05681859797 | 0.3098351205 | 0.7566863426 | 1.017760268 |

| Protein | beta | se | z | p_val | HR |
|---|---|---|---|---|---|
| plg | 0.01982989358 | 0.06405789839 | 0.3095620381 | 0.7568940283 | 1.020027812 |
| hs1bp3 | 0.01951503634 | 0.06311710542 | 0.3091877583 | 0.7571787054 | 1.019706699 |
| angptl4 | -0.01841486943 | 0.05965164429 | -0.3087068203 | 0.7575445552 | 0.9817536483 |
| tpt1 | 0.0175685757 | 0.05699551075 | 0.3082449034 | 0.7578959868 | 1.017723811 |
| tie1 | 0.01729968879 | 0.05614564961 | 0.3081216249 | 0.7579897869 | 1.017450195 |
| ceacam18 | -0.01879557474 | 0.06101117272 | -0.3080677506 | 0.7580307799 | 0.9813799606 |
| enpep | 0.01842496435 | 0.05981113494 | 0.3080524115 | 0.7580424516 | 1.018595751 |
| hnmt | 0.01919903555 | 0.0623803104 | 0.3077739663 | 0.7582543325 | 1.019384522 |
| sfrp1 | 0.01937153954 | 0.06311616219 | 0.306918844 | 0.7589051454 | 1.019560385 |
| npc2 | -0.0201255056 | 0.06558182501 | -0.3068762664 | 0.7589375547 | 0.9800756606 |
| mrpl58 | -0.01823913706 | 0.05948572045 | -0.3066137037 | 0.7591374217 | 0.9819261893 |
| calb2 | 0.01922324578 | 0.0627222044 | 0.3064823051 | 0.7592374505 | 1.019409202 |
| ada2 | 0.01811192651 | 0.05910473097 | 0.3064378471 | 0.7592712957 | 1.018276942 |
| lgals7_lgals7b | 0.01829933985 | 0.05978296955 | 0.3060962007 | 0.7595314006 | 1.018467799 |
| dcxr | 0.01935887666 | 0.06337297747 | 0.3054752582 | 0.7600042111 | 1.019547475 |
| cdc25a | -0.01898926998 | 0.06225212817 | -0.3050380853 | 0.7603371459 | 0.9811898904 |
| rasa1 | -0.01742761258 | 0.0571458448 | -0.3049672753 | 0.7603910763 | 0.9827233699 |
| sowaha | 0.01820249954 | 0.05968686731 | 0.3049665758 | 0.7603916091 | 1.018369175 |
| cd79b | -0.01743247272 | 0.0571679097 | -0.3049345833 | 0.7604159757 | 0.9827185937 |
| cxcl1 | 0.01738149331 | 0.05708759358 | 0.3044705902 | 0.7607693954 | 1.01753343 |
| raly | 0.01836067937 | 0.06032422056 | 0.3043666242 | 0.7608485923 | 1.018530273 |
| esam | 0.01777875463 | 0.05841335407 | 0.3043611331 | 0.7608527753 | 1.017937737 |
| alcam | -0.01877264081 | 0.06177345241 | -0.3038949593 | 0.7612079182 | 0.9814024678 |
| crisp3 | 0.01845138977 | 0.06083509511 | 0.303301733 | 0.7616599257 | 1.018622668 |
| pxdnl | 0.01819368133 | 0.06017105761 | 0.3023659888 | 0.762373079 | 1.018360195 |
| lilrb4 | 0.01880470195 | 0.06229114335 | 0.301884039 | 0.7627404635 | 1.018982624 |
| ido1 | -0.01892101614 | 0.06269510526 | -0.3017941522 | 0.762808989 | 0.9812568626 |
| scn4b | 0.01876224402 | 0.06217114488 | 0.3017837947 | 0.7628168852 | 1.018939361 |
| skap2 | -0.01699285773 | 0.05636221338 | -0.3014937972 | 0.7630379792 | 0.9831507065 |
| steap4 | 0.01848012713 | 0.06138897342 | 0.3010333306 | 0.7633890785 | 1.018651941 |
| commd9 | 0.01844680726 | 0.0613491561 | 0.300685591 | 0.7636542573 | 1.018618001 |
| fgf19 | -0.01697377698 | 0.05666921918 | -0.2995237491 | 0.7645404542 | 0.983169466 |
| rab6b | 0.01715446207 | 0.05729151152 | 0.2994241487 | 0.7646164389 | 1.017302445 |
| lgmn | 0.01734421825 | 0.05793222504 | 0.2993880908 | 0.7646439478 | 1.017495503 |
| rgcc | 0.01784444607 | 0.05967798001 | 0.2990122332 | 0.7649307116 | 1.018004609 |
| mgll | 0.01692871093 | 0.05662618992 | 0.2989555002 | 0.7649739993 | 1.017072814 |
| fcar | 0.01707396807 | 0.05715477725 | 0.2987321252 | 0.7651444433 | 1.017220561 |
| nck2 | -0.01688582757 | 0.05652997059 | -0.2987057554 | 0.7651645653 | 0.983255939 |
| vegfb | 0.01970088885 | 0.06601828827 | 0.2984156265 | 0.7653859639 | 1.019896232 |
| bex3 | -0.01826662453 | 0.06121427683 | -0.2984046448 | 0.7653943444 | 0.981899199 |
| gorasp2 | 0.01807293198 | 0.06071303965 | 0.2976779302 | 0.7659489893 | 1.018237236 |
| mydgf | 0.01815187275 | 0.06108820682 | 0.297142013 | 0.7663580903 | 1.018317619 |
| mtss1 | -0.0174778452 | 0.05888244456 | -0.2968260801 | 0.7665992933 | 0.9826740064 |
| frzb | 0.01752816997 | 0.05905613372 | 0.2968052405 | 0.7666152043 | 1.01768269 |
| vps4b | 0.01776345836 | 0.06014002861 | 0.2953683058 | 0.7677125421 | 1.017922167 |
| ufd1 | 0.01788548363 | 0.06060634215 | 0.2951091089 | 0.7679105316 | 1.018046387 |
| ccl4 | 0.01703544399 | 0.05795811854 | 0.2939267944 | 0.768813843 | 1.017181375 |
| smoc1 | 0.01810690362 | 0.06161976845 | 0.2938489396 | 0.7688733366 | 1.018271828 |
| dnmbp | 0.01726922585 | 0.05889952703 | 0.2931980394 | 0.7693707826 | 1.017419201 |
| hcls1 | -0.01676813791 | 0.05719185343 | -0.2931910212 | 0.7693761468 | 0.9833716648 |
| ngfr | 0.01772918467 | 0.06048998347 | 0.2930928998 | 0.7694511437 | 1.01788728 |
| pcdh17 | -0.01679434325 | 0.05734629618 | -0.2928583774 | 0.7696304046 | 0.9833458956 |
| orm1 | 0.01837420275 | 0.06277876868 | 0.2926817957 | 0.7697653858 | 1.018544047 |
| fabp1 | 0.01782713189 | 0.06116142463 | 0.2914767274 | 0.7706867404 | 1.017986984 |
| mphosph8 | -0.01707890143 | 0.05861056334 | -0.2913963023 | 0.7707482422 | 0.9830661162 |
| casp3 | 0.01646099638 | 0.0565978324 | 0.2908414631 | 0.7711725724 | 1.016597225 |
| igfl4 | 0.01760420945 | 0.06083332858 | 0.2893842876 | 0.7722873176 | 1.017760077 |
| nudt2 | 0.01639931752 | 0.05675276955 | 0.288960656 | 0.7726114857 | 1.016534524 |
| slc16a1 | -0.01673591132 | 0.05800785018 | -0.2885111457 | 0.7729555 | 0.983403356 |
| itpa | 0.01780426023 | 0.06199703163 | 0.2871792369 | 0.7739750836 | 1.017963701 |
| pttg1 | -0.01734515796 | 0.06043492803 | -0.2870055202 | 0.7741080934 | 0.9828044033 |
| scara5 | -0.01753618218 | 0.06116794361 | -0.2866890914 | 0.7743503909 | 0.9826166818 |
| cd69 | -0.01600625753 | 0.05596975469 | -0.2859804838 | 0.7748930692 | 0.9841211619 |
| decr1 | -0.01622793386 | 0.05688251383 | -0.2852886198 | 0.7754230308 | 0.9839030297 |
| ankmy2 | 0.01752997602 | 0.06152817357 | 0.2849097414 | 0.7757132926 | 1.017684528 |
| gstt2b | -0.01733765456 | 0.06091969236 | -0.2845985245 | 0.7759517418 | 0.9828117777 |
| vwc2l | -0.01896567196 | 0.06687845382 | -0.2835841871 | 0.7767290567 | 0.9812130448 |
| hmox1 | -0.01649783335 | 0.05823129661 | -0.2833155761 | 0.7769349382 | 0.9836375106 |
| tmprss15 | 0.0163374019 | 0.05769796627 | 0.2831538606 | 0.7770588954 | 1.016471587 |
| ranbp2 | -0.01725128378 | 0.06114959891 | -0.2821160578 | 0.7778545208 | 0.9828966676 |

| Protein | beta | se | z | p_val | HR |
|---|---|---|---|---|---|
| il1r1 | 0.01625824358 | 0.05770574462 | 0.2817439354 | 0.778139863 | 1.016391128 |
| dok2 | -0.01596215058 | 0.05673297894 | -0.281355763 | 0.7784375442 | 0.9841645694 |
| col18a1 | -0.01705488711 | 0.06075035164 | -0.2807372574 | 0.7789119301 | 0.9830897242 |
| dync1h1 | 0.0171560269 | 0.06115435755 | 0.2805364587 | 0.779065958 | 1.017304037 |
| rab37 | -0.01588863441 | 0.0568041899 | -0.2797088461 | 0.7797008909 | 0.9842369241 |
| rnaset2 | -0.01635202943 | 0.05859417352 | -0.2790726184 | 0.780189096 | 0.9837809393 |
| tpbgl | -0.01687412292 | 0.06054425659 | -0.2787072444 | 0.7804695025 | 0.9832674477 |
| atp5po | -0.01601728771 | 0.05747951306 | -0.2786608107 | 0.7805051401 | 0.9841103069 |
| snap29 | -0.01568375763 | 0.05632678361 | -0.2784422725 | 0.7806728732 | 0.984438592 |
| dbnl | -0.01579979465 | 0.05676068481 | -0.2783580695 | 0.7807375036 | 0.9843243673 |
| susd5 | 0.01864947445 | 0.06716420809 | 0.2776698331 | 0.78126582 | 1.018824462 |
| evi5 | 0.01720098921 | 0.06203451548 | 0.2772809472 | 0.7815643881 | 1.017349778 |
| entpd5 | 0.01641042554 | 0.05929602609 | 0.2767542215 | 0.7819688345 | 1.016545816 |
| tp53inp1 | 0.01594197577 | 0.05766390361 | 0.2764636935 | 0.7821919418 | 1.016069727 |
| vcpkmt | 0.01673120774 | 0.06058681701 | 0.2761526116 | 0.7824308529 | 1.016871958 |
| pnma1 | -0.01695116347 | 0.06148375059 | -0.2757015196 | 0.7827773284 | 0.9831916991 |
| treml1 | -0.01652004449 | 0.05998133123 | -0.2754197706 | 0.7829937565 | 0.9836156631 |
| lepr | 0.01683672218 | 0.06119499515 | 0.2751323395 | 0.7832145667 | 1.016979259 |
| rbm17 | -0.01651505113 | 0.06005374624 | -0.2750045113 | 0.7833127724 | 0.9836205747 |
| hmcn2 | -0.01710792148 | 0.06232450331 | -0.2744975181 | 0.7837023107 | 0.983037588 |
| oscar | 0.01552490458 | 0.05661830671 | 0.274202912 | 0.7839286904 | 1.015646042 |
| dkk3 | 0.01636939519 | 0.05993448253 | 0.2731214904 | 0.7847598276 | 1.016504108 |
| a1bg | 0.01745873822 | 0.06393367197 | 0.2730757936 | 0.7847949537 | 1.017612033 |
| yy1 | 0.01692649855 | 0.06209348703 | 0.2725970042 | 0.785163015 | 1.017070563 |
| edn1 | -0.01709442938 | 0.0628291231 | -0.2720781149 | 0.7855619567 | 0.9830508514 |
| morc3 | -0.01656272531 | 0.06102385561 | -0.2714139437 | 0.7860726789 | 0.9835736825 |
| lefty2 | -0.01680616736 | 0.0619221354 | -0.2714080717 | 0.7860771946 | 0.9833342684 |
| ang | -0.01647558863 | 0.06076525198 | -0.2711350335 | 0.7862871776 | 0.9836593916 |
| lep | -0.02733701455 | 0.1008678685 | -0.2710180651 | 0.7863771381 | 0.9730332599 |
| lmnb1 | 0.01657989752 | 0.06118862712 | 0.2709637117 | 0.7864189423 | 1.016718107 |
| stx7 | 0.01712284375 | 0.06319436455 | 0.2709552328 | 0.7864254637 | 1.01727028 |
| foxj3 | -0.01704948667 | 0.06300043784 | -0.2706248918 | 0.7866795491 | 0.9830950333 |
| pithd1 | 0.01660499135 | 0.06136127786 | 0.2706102599 | 0.7866908039 | 1.01674362 |
| bdnf | 0.01605631906 | 0.0594854985 | 0.2699198874 | 0.7872218869 | 1.016185914 |
| impa1 | 0.01552346905 | 0.05774298433 | 0.2688373181 | 0.7880548744 | 1.015644584 |
| gad2 | 0.01657001435 | 0.06178856053 | 0.2681728496 | 0.7885662727 | 1.016708058 |
| vsig10l | 0.01615284996 | 0.06036933814 | 0.2675671202 | 0.7890325426 | 1.016284013 |
| chchd10 | 0.01868249518 | 0.06989195664 | 0.2673053679 | 0.7892340541 | 1.018858105 |
| fnta | -0.01685049837 | 0.06309572546 | -0.2670624397 | 0.7894210864 | 0.9832906772 |
| rad51 | -0.01617432229 | 0.0605644994 | -0.2670594565 | 0.7894233832 | 0.9839557797 |
| ndufb7 | 0.01596137225 | 0.05982380977 | 0.2668063487 | 0.7896182658 | 1.016089435 |
| il19 | -0.01541070391 | 0.0578212606 | -0.2665231396 | 0.7898363409 | 0.9847074334 |
| dut | 0.0159766601 | 0.06004617955 | 0.2660728829 | 0.7901830787 | 1.016104969 |
| ahnak2 | 0.01696710224 | 0.06381972104 | 0.2658598623 | 0.7903471382 | 1.017111861 |
| scarf2 | 0.01718420318 | 0.06467196373 | 0.2657133352 | 0.7904599924 | 1.017332701 |
| upk3a | -0.01625880212 | 0.06124908511 | -0.2654537957 | 0.7906598991 | 0.9838726588 |
| atp1b3 | 0.01622244578 | 0.06112583317 | 0.2653942684 | 0.790705751 | 1.016354744 |
| reg3g | 0.01583893557 | 0.05975782404 | 0.2650520802 | 0.7909693418 | 1.015965036 |
| fam172a | -0.01652070896 | 0.06243365874 | -0.2646122187 | 0.7913082064 | 0.9836150095 |
| snrpb2 | 0.01677102475 | 0.06355185678 | 0.263895118 | 0.7918607376 | 1.016912448 |
| alpp | -0.01650262781 | 0.06254391406 | -0.2638566527 | 0.7918903783 | 0.9836327946 |
| lpp | -0.01614825491 | 0.06132328288 | -0.2633299156 | 0.7922963038 | 0.9839814292 |
| atp6ap2 | -0.01510876923 | 0.05741286857 | -0.2631599779 | 0.7924272768 | 0.9850047956 |
| klk15 | -0.0157312143 | 0.05984344814 | -0.2628727921 | 0.7926486277 | 0.984391875 |
| birc2 | 0.01519588416 | 0.05791129624 | 0.2623993097 | 0.7930136048 | 1.015311929 |
| dsg4 | 0.01549205253 | 0.05905002375 | 0.2623547215 | 0.7930479774 | 1.015612676 |
| snx2 | 0.01557663897 | 0.05945314241 | 0.2619985813 | 0.7933225359 | 1.015698587 |
| lcp1 | 0.01595257376 | 0.06109666245 | 0.2611038495 | 0.7940124232 | 1.016080495 |
| cst3 | -0.01672412085 | 0.06425670891 | -0.2602704236 | 0.7946551851 | 0.9834149509 |
| vstm2b | -0.01577727715 | 0.0606351314 | -0.2602002632 | 0.7947093013 | 0.9843465321 |
| pcdhb15 | 0.0156339488 | 0.0601434742 | 0.2599442252 | 0.7949067968 | 1.015756798 |
| lap3 | -0.0151481863 | 0.05844027995 | -0.2592079695 | 0.7954747825 | 0.9849659703 |
| nenf | 0.01551848145 | 0.05994189492 | 0.2588920732 | 0.7957185144 | 1.015639518 |
| pltp | 0.01560614035 | 0.06037478748 | 0.2584877066 | 0.7960305354 | 1.015728552 |
| hla_e | 0.0154945895 | 0.060032679 | 0.2581025828 | 0.7963277383 | 1.015615253 |
| kif22 | -0.01544124042 | 0.05988103656 | -0.2578652827 | 0.7965108793 | 0.9846773643 |
| lilra4 | -0.01550060979 | 0.0603072112 | -0.2570274678 | 0.797157569 | 0.9846189063 |
| vamp5 | -0.01507732635 | 0.05867503907 | -0.2569632093 | 0.7972071743 | 0.9850357674 |
| thpo | 0.01493057119 | 0.05812271923 | 0.2568801217 | 0.7972713164 | 1.015042589 |
| ctag1a_ctag1b | -0.01546919199 | 0.06040631927 | -0.2560856576 | 0.7978846964 | 0.9846498414 |

| Protein | beta | se | z | p_val | HR |
|---|---|---|---|---|---|
| il2rg | 0.01555926339 | 0.06119689062 | 0.2542492475 | 0.7993030063 | 1.015680939 |
| mst1 | -0.01533523067 | 0.0604036962 | -0.2538790113 | 0.7995890302 | 0.9847817552 |
| tlr4 | -0.01561802932 | 0.06161384479 | -0.253482466 | 0.7998954089 | 0.9845032996 |
| kir3dl2 | 0.01526099438 | 0.06029480742 | 0.2531062795 | 0.8001860865 | 1.015378038 |
| p4hb | 0.01492444022 | 0.05903340564 | 0.2528134716 | 0.800412357 | 1.015036366 |
| tab2 | 0.01525311934 | 0.06036648381 | 0.252675299 | 0.8005191372 | 1.015370042 |
| il18 | 0.01461576856 | 0.05790558141 | 0.2524069045 | 0.8007265639 | 1.014723101 |
| tex33 | -0.01532173758 | 0.06076097346 | -0.252164123 | 0.8009142079 | 0.984795043 |
| fdx1 | -0.01565503311 | 0.06213835476 | -0.2519383266 | 0.8010887345 | 0.98446687 |
| rbm19 | -0.01529122457 | 0.06110444014 | -0.2502473557 | 0.8023960654 | 0.9848250926 |
| dpp7 | -0.01418803418 | 0.05672464368 | -0.2501211688 | 0.8024936458 | 0.9859121417 |
| folr2 | -0.01493067531 | 0.05975969324 | -0.2498452469 | 0.8027070271 | 0.9851802345 |
| irak1 | 0.01461524693 | 0.05851757763 | 0.2497582354 | 0.8027743196 | 1.014722572 |
| cdh6 | 0.01444732997 | 0.057898206 | 0.249529838 | 0.8029509633 | 1.014552197 |
| asgr2 | -0.01533594274 | 0.06146843064 | -0.2494929931 | 0.8029794602 | 0.984781054 |
| armcx2 | -0.01538395628 | 0.0617049429 | -0.2493148127 | 0.8031172741 | 0.9847337723 |
| smc3 | -0.01502725971 | 0.06028944559 | -0.2492519141 | 0.8031659246 | 0.9850850861 |
| max | 0.01434660064 | 0.05757868008 | 0.2491651532 | 0.8032330332 | 1.014450007 |
| pdgfb | -0.01394617802 | 0.05621200928 | -0.2480996179 | 0.8040573313 | 0.9861506194 |
| xcl1 | -0.01407015542 | 0.056746357 | -0.2479481707 | 0.8041745085 | 0.9860283666 |
| get3 | -0.01506035224 | 0.06080890901 | -0.2476668712 | 0.8043921663 | 0.9850524877 |
| lilra6 | 0.0147954653 | 0.05984748668 | 0.2472194927 | 0.8047383604 | 1.01490546 |
| fkbp5 | 0.01401967126 | 0.05695911235 | 0.2461357047 | 0.8055771848 | 1.014118408 |
| lonp1 | -0.01518966837 | 0.06174855102 | -0.2459923045 | 0.8056881896 | 0.9849251127 |
| hsdl2 | -0.01508145682 | 0.06130882128 | -0.2459916291 | 0.8056887125 | 0.9850316988 |
| reg1a | 0.01443776948 | 0.05897071835 | 0.244829466 | 0.8065884775 | 1.014542497 |
| hspb1 | -0.01402581833 | 0.05748623748 | -0.2439856729 | 0.8072419161 | 0.9860720852 |
| selp | 0.0139561317 | 0.05754058375 | 0.2425441451 | 0.8083585549 | 1.014053973 |
| anxa10 | -0.01430183005 | 0.05917552074 | -0.2416849041 | 0.8090243276 | 0.9857999553 |
| ptpn1 | -0.01381549178 | 0.05727288416 | -0.2412222116 | 0.8093828968 | 0.9862795042 |
| hs6st2 | -0.01571350639 | 0.06575056291 | -0.2389866443 | 0.811115939 | 0.9844093066 |
| tafa5 | -0.01388809446 | 0.05824909754 | -0.2384259164 | 0.8115507686 | 0.9862079002 |
| atxn2l | 0.01467608457 | 0.06155704936 | 0.2384143607 | 0.8115597303 | 1.014784307 |
| brme1 | -0.01443307579 | 0.06059266632 | -0.2381983938 | 0.8117272228 | 0.9856705817 |
| cwc15 | 0.01489548436 | 0.06256099539 | 0.2380953862 | 0.8118071132 | 1.015006975 |
| clta | 0.01416781208 | 0.05955094084 | 0.2379108017 | 0.8119502775 | 1.014268651 |
| pde5a | 0.01444722024 | 0.06101354691 | 0.2367870903 | 0.8128219673 | 1.014552086 |
| chgb | -0.01407423777 | 0.05947682253 | -0.2366339891 | 0.8129407495 | 0.9860243413 |
| grp | -0.01445494303 | 0.06111104391 | -0.2365356914 | 0.8130170151 | 0.9856490281 |
| chit1 | -0.01356057765 | 0.05733539405 | -0.2365132023 | 0.8130344639 | 0.9865309528 |
| tnpo1 | 0.01428209707 | 0.0604033667 | 0.2364453813 | 0.8130870851 | 1.014384573 |
| gdnf | 0.01408815591 | 0.05960370732 | 0.2363637522 | 0.8131504209 | 1.014187862 |
| commd1 | -0.01480985134 | 0.06287773408 | -0.2355341132 | 0.8137942049 | 0.9852992751 |
| bmp4 | -0.01369996334 | 0.05842324613 | -0.2344950726 | 0.8146006581 | 0.9863934541 |
| ly6d | -0.0136298749 | 0.05821737311 | -0.2341204038 | 0.8148915061 | 0.9864625913 |
| bloc1s3 | -0.01425337327 | 0.06093590033 | -0.2339076504 | 0.8150566738 | 0.9858477252 |
| pcbd1 | 0.01622482806 | 0.06936859069 | 0.233893004 | 0.8150680446 | 1.016357165 |
| slc9a3r1 | 0.01435699401 | 0.06141446874 | 0.2337721763 | 0.8151618512 | 1.014460551 |
| txk | 0.01387535497 | 0.05954102969 | 0.2330385457 | 0.8157314745 | 1.013972064 |
| lrtm1 | -0.01434741758 | 0.06166810651 | -0.2326553934 | 0.8160290096 | 0.9857550161 |
| hbz | -0.01398703788 | 0.06036172996 | -0.2317202951 | 0.8167552671 | 0.9861103263 |
| ppcdc | 0.01331960367 | 0.05757789537 | 0.2313318954 | 0.8170569697 | 1.013408705 |
| nmt1 | 0.01389454592 | 0.06006461441 | 0.2313266481 | 0.8170610459 | 1.013991524 |
| pxn | -0.01378356214 | 0.05963618521 | -0.2311274956 | 0.8172157549 | 0.9863109962 |
| tff1 | 0.01427167828 | 0.06187134885 | 0.2306669977 | 0.8175735142 | 1.014374005 |
| stau1 | 0.01403071997 | 0.0609754389 | 0.2301044523 | 0.8180106053 | 1.014129612 |
| abraxas2 | -0.01399389704 | 0.06096426411 | -0.2295426221 | 0.8184471972 | 0.9861035624 |
| triap1 | 0.01314727073 | 0.0575657879 | 0.2283868806 | 0.8193454877 | 1.013234076 |
| fyb1 | -0.01302175867 | 0.0570248865 | -0.2283522067 | 0.8193724414 | 0.9870626576 |
| sms | 0.01371501747 | 0.06036054068 | 0.2272182674 | 0.8202540238 | 1.0138095 |
| sned1 | 0.01427658404 | 0.0631319029 | 0.2261389786 | 0.8210933291 | 1.014378981 |
| milr1 | -0.01319509477 | 0.05836932671 | -0.2260621377 | 0.821153092 | 0.9868915788 |
| extl1 | 0.01375646543 | 0.06097098954 | 0.2256231289 | 0.8214945504 | 1.013851521 |
| nfat5 | 0.01370770167 | 0.06085665076 | 0.2252457455 | 0.8217881039 | 1.013802083 |
| tnip1 | 0.01351374807 | 0.06001636174 | 0.2251677323 | 0.8218487908 | 1.013605471 |
| usp8 | -0.01328528736 | 0.05905263775 | -0.2249736484 | 0.8219997743 | 0.9868025726 |
| ptprb | 0.01412774051 | 0.0628729722 | 0.2247029211 | 0.822210392 | 1.014228009 |
| lactb2 | 0.01280855357 | 0.05702288179 | 0.2246212953 | 0.822273897 | 1.012890934 |
| rspo3 | -0.01374527443 | 0.0612195601 | -0.2245242273 | 0.8223494175 | 0.9863487605 |
| akr1b10 | -0.01375138613 | 0.0612977792 | -0.224337428 | 0.8224947549 | 0.9863427323 |

| Protein | beta | se | z | p_val | HR |
|---|---|---|---|---|---|
| spaca5_spaca5b | -0.0139073882 | 0.06199953746 | -0.2243143864 | 0.8225126827 | 0.9861888728 |
| cacna1h | -0.01343410673 | 0.05998494495 | -0.2239579738 | 0.8227900047 | 0.9866557281 |
| shc1 | 0.01394226265 | 0.06236421472 | 0.223561905 | 0.8230982088 | 1.014039909 |
| pagr1 | 0.01402854777 | 0.06276058122 | 0.2235248224 | 0.8231270663 | 1.01412741 |
| coro6 | -0.0139493058 | 0.06247405395 | -0.2232815852 | 0.8233163584 | 0.986147535 |
| il20ra | -0.01286844811 | 0.05764535444 | -0.2232347816 | 0.823352783 | 0.9872139963 |
| stx4 | 0.01307349156 | 0.05869813196 | 0.2227241503 | 0.8237502042 | 1.013159323 |
| tjap1 | -0.01265418487 | 0.05687885547 | -0.2224760812 | 0.8239432911 | 0.9874255427 |
| nrp2 | -0.01317302201 | 0.05937480567 | -0.2218621495 | 0.8244211966 | 0.9869133625 |
| tnfrsf10b | -0.01447485761 | 0.06524358339 | -0.2218587156 | 0.8244238698 | 0.9856293995 |
| klk3 | -0.02011660073 | 0.09067600583 | -0.2218514209 | 0.8244295486 | 0.9800843881 |
| otud7b | 0.0137098377 | 0.0618351961 | 0.2217157632 | 0.8245351582 | 1.013804248 |
| rtn4r | 0.01383414326 | 0.06260044045 | 0.220991149 | 0.8250993247 | 1.013930278 |
| dpep1 | 0.01263702218 | 0.05731609346 | 0.2204794747 | 0.8254977559 | 1.012717207 |
| mme | 0.01315589946 | 0.05972446041 | 0.220276573 | 0.8256557642 | 1.013242819 |
| capg | -0.01293388658 | 0.05871893903 | -0.2202677159 | 0.8256626617 | 0.9871493967 |
| ndufs6 | -0.01289645802 | 0.05857150057 | -0.220183159 | 0.825728512 | 0.987186345 |
| agbl2 | 0.01358775143 | 0.06172487222 | 0.2201341363 | 0.8257666899 | 1.013680484 |
| fas | -0.01410438491 | 0.06414487216 | -0.2198832804 | 0.8259620579 | 0.9859946159 |
| cdhr1 | -0.01274845453 | 0.0579987742 | -0.2198055856 | 0.8260225693 | 0.9873324628 |
| ofd1 | -0.01335285056 | 0.06084992538 | -0.2194390622 | 0.8263080442 | 0.9867359033 |
| lypla2 | -0.01340250334 | 0.06116816316 | -0.2191091353 | 0.8265650348 | 0.9866869103 |
| rnf4 | -0.01325635478 | 0.06060809642 | -0.2187225067 | 0.8268662157 | 0.9868311237 |
| akt3 | 0.01267473066 | 0.05828724946 | 0.2174528868 | 0.8278554194 | 1.012755395 |
| gatd3 | 0.01349502604 | 0.06211488212 | 0.2172591428 | 0.828006396 | 1.013586495 |
| impg1 | 0.01318497593 | 0.06078361498 | 0.2169166137 | 0.82827333 | 1.013272281 |
| letm1 | -0.01357147468 | 0.06273154238 | -0.216342117 | 0.8287210817 | 0.9865202026 |
| crhr1 | -0.01256139843 | 0.05811764537 | -0.2161374287 | 0.8288806253 | 0.9875171666 |
| dmd | -0.01381021521 | 0.06418121715 | -0.2151753399 | 0.8296306166 | 0.9862847083 |
| prkar1a | 0.01208604939 | 0.05655221511 | 0.2137148716 | 0.8307694134 | 1.012159381 |
| agt | -0.01313100908 | 0.06168555246 | -0.2128700896 | 0.8314282927 | 0.9869548265 |
| gstp1 | -0.01249890941 | 0.05876663598 | -0.2126871686 | 0.8315709758 | 0.9875788775 |
| hars1 | 0.01207190406 | 0.05689381096 | 0.2121830802 | 0.8319642062 | 1.012145064 |
| clip2 | 0.01212457294 | 0.05719756329 | 0.2119770886 | 0.8321249088 | 1.012198374 |
| cplx2 | -0.01302138268 | 0.06149859396 | -0.2117346404 | 0.8323140616 | 0.9870630287 |
| calca | 0.0155378878 | 0.07345601226 | 0.2115264267 | 0.8324765132 | 1.015659228 |
| coro1a | -0.01207812433 | 0.05714682449 | -0.2113525019 | 0.8326122175 | 0.9879945234 |
| il3ra | -0.01244034978 | 0.05892052307 | -0.2111378028 | 0.8327797427 | 0.9876367115 |
| stxbp1 | 0.01281846822 | 0.06072073788 | 0.2111052774 | 0.8328051222 | 1.012900977 |
| ythdf3 | -0.01216728232 | 0.0577681375 | -0.2106227212 | 0.8331816811 | 0.9879064398 |
| manf | -0.01185971435 | 0.05652824721 | -0.2098015583 | 0.8338225568 | 0.9882103349 |
| clstn1 | -0.0120300581 | 0.05744967602 | -0.20940167 | 0.8341346892 | 0.9880420138 |
| akt2 | 0.01272497522 | 0.06085753515 | 0.2090944891 | 0.8343744767 | 1.012806282 |
| serpine1 | -0.01188420836 | 0.05687465363 | -0.2089543866 | 0.8344838468 | 0.9881861299 |
| bambi | 0.01218623464 | 0.05858700151 | 0.2080023611 | 0.8352271238 | 1.012260789 |
| bag4 | 0.01312271252 | 0.06320775512 | 0.2076123807 | 0.8355316366 | 1.013209193 |
| fgf3 | -0.01294542729 | 0.06240316749 | -0.2074482404 | 0.8356598115 | 0.9871380043 |
| kir2dl3 | 0.01186006059 | 0.05722619755 | 0.2072487968 | 0.8358155602 | 1.01193067 |
| tcp11 | 0.01231360669 | 0.05989940024 | 0.2055714522 | 0.8371256791 | 1.012389731 |
| grap2 | 0.01161178102 | 0.0565089559 | 0.2054856763 | 0.837192688 | 1.011679459 |
| pikfyve | 0.01288173472 | 0.06276138678 | 0.2052493639 | 0.8373773035 | 1.012965062 |
| slc13a1 | -0.01224712565 | 0.05982719201 | -0.2047083481 | 0.8377999977 | 0.9878275652 |
| ikbkg | -0.01169315786 | 0.05719411084 | -0.204446886 | 0.8380042941 | 0.9883749414 |
| ass1 | -0.01338571706 | 0.06564092964 | -0.2039233315 | 0.8384134123 | 0.9867034733 |
| pak4 | 0.01171934934 | 0.05755245947 | 0.2036289925 | 0.8386434351 | 1.01178829 |
| slamf8 | 0.01146289409 | 0.05644871938 | 0.2030673895 | 0.8390823601 | 1.011528845 |
| phldb1 | -0.01290056815 | 0.06382822711 | -0.202113841 | 0.8398277276 | 0.9871822875 |
| acp6 | -0.01134427839 | 0.0561944882 | -0.2018752863 | 0.840014223 | 0.9887198253 |
| prkab1 | 0.01184018924 | 0.05900478947 | 0.2006648841 | 0.8409606196 | 1.011910562 |
| art5 | -0.01206882339 | 0.06034498432 | -0.1999971253 | 0.8414828294 | 0.9880037128 |
| pspn | 0.01574219636 | 0.07899031204 | 0.1992927481 | 0.8420337516 | 1.015866757 |
| pde1c | 0.01194235722 | 0.05999555381 | 0.1990540376 | 0.8422204744 | 1.012013952 |
| nucb2 | -0.01171539144 | 0.05890681643 | -0.1988800643 | 0.8423565644 | 0.9883529666 |
| dcn | -0.01214422101 | 0.06108938749 | -0.1987942835 | 0.8424236679 | 0.9879292224 |
| dll4 | -0.01231012045 | 0.06226480365 | -0.1977059226 | 0.8432751557 | 0.9877653391 |
| lrpap1 | 0.01166591038 | 0.05912985379 | 0.1972930699 | 0.8435982022 | 1.011734222 |
| fxn | 0.0111132842 | 0.05642648816 | 0.1969515481 | 0.843865454 | 1.011175266 |
| slirp | -0.0118874415 | 0.06083813517 | -0.1953945739 | 0.8450840641 | 0.988182935 |
| ptprn2 | -0.01117132583 | 0.0573672016 | -0.1947336721 | 0.84560145 | 0.9888908417 |
| dbi | 0.01142191955 | 0.05872691619 | 0.194492071 | 0.8457906036 | 1.011487399 |

| Protein | beta | se | z | p_val | HR |
|---|---|---|---|---|---|
| tnfsf13b | -0.01131735564 | 0.05835548265 | -0.1939381722 | 0.846224294 | 0.9887464447 |
| rspo1 | -0.01164152922 | 0.0603427088 | -0.1929235438 | 0.8470188461 | 0.9884259712 |
| pibf1 | -0.01178352371 | 0.06114974284 | -0.192699481 | 0.84719433 | 0.9882856301 |
| csf3r | 0.01189098835 | 0.06196882652 | 0.1918866149 | 0.8478310224 | 1.011961967 |
| agrn | 0.01149655792 | 0.06044689949 | 0.1901926818 | 0.8491581455 | 1.011562897 |
| lcat | 0.01218528639 | 0.06422087906 | 0.1897402616 | 0.8495126696 | 1.012259829 |
| prss2 | -0.0105127107 | 0.05552980538 | -0.1893165415 | 0.8498447315 | 0.9895423547 |
| aoc3 | -0.01122209515 | 0.05951191538 | -0.1885688786 | 0.8504307265 | 0.9888406377 |
| itgb1 | -0.0114477588 | 0.06075444773 | -0.1884266788 | 0.8505421876 | 0.9886175175 |
| gcnt1 | -0.01102554515 | 0.05873641092 | -0.1877122721 | 0.8511022095 | 0.9890350134 |
| cntn4 | 0.01077710641 | 0.05784194795 | 0.1863199077 | 0.8521938967 | 1.010835389 |
| serpinb5 | -0.01076602372 | 0.05790248013 | -0.1859337234 | 0.8524967359 | 0.9892917225 |
| psrc1 | 0.01054638023 | 0.05699020993 | 0.1850559991 | 0.8531851131 | 1.010602189 |
| hmmr | -0.01123567958 | 0.06076642198 | -0.184899476 | 0.853307882 | 0.9888272049 |
| ogn | 0.01161408305 | 0.06287231953 | 0.1847249018 | 0.8534448135 | 1.011681788 |
| gpnmb | -0.01069387767 | 0.05794336929 | -0.1845574015 | 0.8535762006 | 0.9893630986 |
| mki67 | 0.01147955349 | 0.06227195267 | 0.1843454877 | 0.8537424313 | 1.011545696 |
| mn1 | -0.01129269394 | 0.06128026987 | -0.184279442 | 0.8537942406 | 0.9887708292 |
| agr2 | 0.0109971198 | 0.05981430407 | 0.1838543468 | 0.8541277202 | 1.01105781 |
| pth | -0.0119337896 | 0.0650983735 | -0.1833193206 | 0.8545474756 | 0.9881371356 |
| gip | 0.01154937666 | 0.06306357049 | 0.183138642 | 0.8546892365 | 1.011616328 |
| adh1b | 0.01236155526 | 0.06787031646 | 0.1821349288 | 0.8554768379 | 1.012438275 |
| efcab14 | -0.01145520934 | 0.0630028979 | -0.1818203562 | 0.8557237089 | 0.9886101518 |
| erp44 | -0.01056978199 | 0.05867113681 | -0.1801530116 | 0.8570324461 | 0.9894858819 |
| lypd3 | 0.01028988676 | 0.05721950922 | 0.1798317899 | 0.8572846257 | 1.01034301 |
| cacnb3 | 0.01094747292 | 0.06092497315 | 0.1796877759 | 0.8573976906 | 1.011007616 |
| slamf6 | -0.01033324898 | 0.05755539626 | -0.1795357108 | 0.8575170797 | 0.9897199556 |
| rangap1 | 0.01037131189 | 0.05779045608 | 0.1794640948 | 0.8575733078 | 1.01042528 |
| mmut | -0.01091229428 | 0.0608391638 | -0.1793629892 | 0.8576526904 | 0.9891470288 |
| hcg22 | -0.01090537767 | 0.06085636087 | -0.1791986493 | 0.8577817243 | 0.9891538704 |
| sdk2 | 0.01098986553 | 0.06148673432 | 0.1787355542 | 0.8581453505 | 1.011050476 |
| cthrc1 | -0.01245539211 | 0.06968778386 | -0.178731356 | 0.8581486471 | 0.9876218552 |
| tnn | -0.01067333037 | 0.05975917465 | -0.1786057192 | 0.8582473034 | 0.9893834275 |
| col24a1 | 0.01090936272 | 0.0611306042 | 0.1784599198 | 0.8583617953 | 1.010969087 |
| prdx5 | -0.01005263264 | 0.05654035741 | -0.1777957038 | 0.8588834215 | 0.9899977262 |
| ccn2 | -0.01028220628 | 0.05793674157 | -0.1774729818 | 0.8591368857 | 0.9897704749 |
| vsig10 | 0.0108192934 | 0.06113843435 | 0.176963861 | 0.8595367762 | 1.010878034 |
| gimap7 | 0.01087555998 | 0.06164539904 | 0.1764212763 | 0.8599629907 | 1.010934914 |
| atp1b1 | -0.01067498776 | 0.06055277182 | -0.1762923057 | 0.8600643065 | 0.9893817877 |
| tfap2a | -0.01072581483 | 0.06153105499 | -0.174315471 | 0.8616175413 | 0.9893315016 |
| col5a1 | -0.01072434303 | 0.06171071456 | -0.1737841331 | 0.8620351146 | 0.9893329577 |
| lrp2bp | 0.01060892533 | 0.06114578952 | 0.17350214 | 0.862256746 | 1.0106654 |
| defb118 | 0.0105539345 | 0.06093505966 | 0.1731997073 | 0.8624944537 | 1.010609824 |
| cd40lg | 0.009612911551 | 0.05599515203 | 0.1716739968 | 0.8636938298 | 1.009659264 |
| cdh1 | 0.01025139386 | 0.05975073048 | 0.1715693479 | 0.8637761069 | 1.010304119 |
| pkn3 | 0.01030729445 | 0.06047365152 | 0.1704427332 | 0.8646619676 | 1.010360598 |
| exosc10 | -0.01032056626 | 0.06064091926 | -0.1701914546 | 0.864859572 | 0.989732508 |
| nid1 | 0.009761855161 | 0.05737677657 | 0.1701359983 | 0.8649031837 | 1.009809657 |
| arid4b | -0.009803809691 | 0.05771989889 | -0.1698514703 | 0.8651269475 | 0.990244091 |
| eps8l2 | 0.01001612973 | 0.0590452541 | 0.1696347976 | 0.8652973546 | 1.010066459 |
| tpsd1 | 0.01038265468 | 0.06121052387 | 0.1696220523 | 0.8653073786 | 1.010436741 |
| mtif3 | 0.01012583236 | 0.0599039608 | 0.1690344382 | 0.8657695533 | 1.010177272 |
| f12 | 0.01039276635 | 0.06180068407 | 0.1681658788 | 0.8664527834 | 1.010446959 |
| ube2z | 0.01040204728 | 0.06192285439 | 0.1679839759 | 0.8665958853 | 1.010456337 |
| flt3lg | 0.009810138895 | 0.05860455746 | 0.1673954948 | 0.8670588698 | 1.009858416 |
| wif1 | -0.009761505549 | 0.05840919782 | -0.1671227463 | 0.8672734688 | 0.9902859833 |
| mindy1 | -0.009983095302 | 0.05982634546 | -0.1668678778 | 0.8674740085 | 0.9900665704 |
| tbc1d5 | 0.009405619414 | 0.0564154738 | 0.1667205605 | 0.867589927 | 1.009449991 |
| golga3 | -0.01002073428 | 0.06023194428 | -0.1663690986 | 0.8678664908 | 0.990029306 |
| tnfsf12 | 0.009541370365 | 0.05744673078 | 0.1660907459 | 0.8680855367 | 1.009587034 |
| nbn | -0.009563636871 | 0.05780761218 | -0.1654390574 | 0.8685984138 | 0.9904819493 |
| mecr | -0.010141741 | 0.06130274559 | -0.1654369785 | 0.8686000501 | 0.989909513 |
| doc2b | -0.01024324127 | 0.0619980476 | -0.1652187717 | 0.8687717906 | 0.9898090421 |
| rnase4 | 0.0109568484 | 0.06648171046 | 0.1648099654 | 0.8690935601 | 1.011017094 |
| txndc9 | -0.009951177794 | 0.06067830176 | -0.1639989503 | 0.8697319702 | 0.9900981713 |
| atf4 | 0.00994742761 | 0.06071027571 | 0.163850806 | 0.8698485947 | 1.009997068 |
| ccl8 | -0.009308538761 | 0.05699188526 | -0.16333095 | 0.8702578665 | 0.9907346516 |
| ppp1r12a | -0.009260994327 | 0.05682737309 | -0.1629671375 | 0.8705443092 | 0.9907817566 |
| col9a1 | -0.009285244067 | 0.05707889073 | -0.1626738703 | 0.8707752214 | 0.9907577307 |
| igsf8 | 0.00954130744 | 0.05869387161 | 0.1625605396 | 0.8708644585 | 1.009586971 |

| Protein | beta | se | z | p_val | HR |
| --- | --- | --- | --- | --- | --- |
| c7 | 0.01013274705 | 0.06234343978 | 0.1625310873 | 0.8708876496 | 1.010184257 |
| kyat1 | -0.009281182081 | 0.05710635269 | -0.1625245116 | 0.8708928274 | 0.9907617552 |
| cacnb1 | 0.009885564415 | 0.06092599065 | 0.1622552922 | 0.8711048197 | 1.009934588 |
| aldh5a1 | 0.009974719415 | 0.06160517848 | 0.1619136517 | 0.8713738521 | 1.010024633 |
| pkd2 | 0.01016091939 | 0.0628507627 | 0.1616673999 | 0.8715677779 | 1.010212717 |
| cdc27 | 0.009343015403 | 0.05780804551 | 0.1616213681 | 0.8716040292 | 1.009386798 |
| tnfsf13 | 0.009658911224 | 0.05977929205 | 0.1615762063 | 0.8716395957 | 1.009705709 |
| micb_mica | -0.009017878547 | 0.05581975947 | -0.1615535186 | 0.8716574632 | 0.9910226606 |
| rbpms2 | -0.00986055506 | 0.06131859664 | -0.1608085573 | 0.8722441852 | 0.9901879008 |
| bcr | -0.009187684808 | 0.05721153484 | -0.1605914757 | 0.8724151692 | 0.990854393 |
| suox | -0.01025028024 | 0.06423799563 | -0.1595672489 | 0.8732219799 | 0.9898020748 |
| ca8 | -0.009824103864 | 0.06164789868 | -0.1593582924 | 0.8733865968 | 0.990223995 |
| gpihbp1 | -0.009489930737 | 0.0598710292 | -0.1585062235 | 0.874057917 | 0.9905549566 |
| chl1 | 0.009339486 | 0.05900412299 | 0.1582853117 | 0.8742319818 | 1.009383235 |
| acta2 | 0.01075937772 | 0.06826379182 | 0.1576147095 | 0.8747604119 | 1.010817468 |
| zpr1 | -0.009507030818 | 0.06054836081 | -0.1570154946 | 0.8752326367 | 0.9905380181 |
| phactr2 | 0.009479008927 | 0.06052534536 | 0.1566122237 | 0.8755504683 | 1.009524077 |
| mapt | 0.008991186418 | 0.05770206104 | 0.1558208885 | 0.8761742051 | 1.009031729 |
| cpox | -0.009713315373 | 0.0623789264 | -0.155714693 | 0.8762579151 | 0.9903337065 |
| cdc37 | 0.008803559799 | 0.05685777119 | 0.1548347678 | 0.876951581 | 1.008842425 |
| sez6l | 0.009347278577 | 0.06099388657 | 0.1532494337 | 0.8782015756 | 1.009391101 |
| serpinf2 | 0.009788200928 | 0.06387528971 | 0.1532392412 | 0.8782096131 | 1.009836262 |
| rgma | -0.008750132315 | 0.0571058183 | -0.1532266339 | 0.8782195548 | 0.9912880387 |
| tmod4 | 0.009375210634 | 0.06120834996 | 0.1531688183 | 0.8782651469 | 1.009419296 |
| itgb1bp2 | -0.008892996169 | 0.05811868389 | -0.1530144108 | 0.878386911 | 0.9911464296 |
| rnf5 | 0.009474636544 | 0.06199544903 | 0.1528279364 | 0.8785339664 | 1.009519663 |
| yes1 | 0.008675919925 | 0.05688542824 | 0.1525156827 | 0.8787802221 | 1.008713665 |
| dapp1 | 0.008652554698 | 0.05683141611 | 0.1522495002 | 0.8789901535 | 1.008690096 |
| rrm2 | 0.008667107392 | 0.05715947062 | 0.151630295 | 0.8794785377 | 1.008704776 |
| lyn | 0.008512388151 | 0.05656448539 | 0.1504899778 | 0.8803780572 | 1.008548722 |
| anxa5 | -0.008865437886 | 0.0589437214 | -0.1504051267 | 0.8804449968 | 0.9911737442 |
| chrdl2 | 0.009310337983 | 0.06211032065 | 0.1499000148 | 0.8808435001 | 1.009353814 |
| angpt1 | -0.008300506271 | 0.05578660346 | -0.1487903144 | 0.8817190935 | 0.9917338478 |
| heg1 | -0.009674869286 | 0.06506786071 | -0.14868891 | 0.8817991125 | 0.9903717817 |
| c1r | -0.009141613208 | 0.06158301222 | -0.148443749 | 0.8819925758 | 0.9909000443 |
| scrn1 | -0.008638988399 | 0.05821614364 | -0.1483950646 | 0.8820309948 | 0.9913982204 |
| mtr | 0.008953601838 | 0.06125647069 | 0.146165813 | 0.883790493 | 1.008993805 |
| bin2 | 0.00817686723 | 0.05618277846 | 0.1455404566 | 0.884284176 | 1.008210389 |
| amy2a | 0.008548406657 | 0.05875151072 | 0.1455010527 | 0.8843152847 | 1.008585049 |
| ntf4 | 0.008195431354 | 0.05660990177 | 0.1447702804 | 0.8848922478 | 1.008229106 |
| cfc1 | -0.008519953063 | 0.05902399891 | -0.1443472692 | 0.8852262537 | 0.9915162389 |
| slit2 | 0.008306529018 | 0.05756040609 | 0.144309771 | 0.885255863 | 1.008341124 |
| mapk9 | -0.008404834212 | 0.05884379341 | -0.1428329774 | 0.8864220919 | 0.9916303877 |
| gpr37 | -0.008467040079 | 0.05928734797 | -0.1428136081 | 0.8864373895 | 0.9915687044 |
| terf1 | -0.008860573286 | 0.06208151954 | -0.1427248133 | 0.8865075191 | 0.9911785659 |
| rtkn2 | -0.008558572493 | 0.06013867612 | -0.1423139491 | 0.8868320289 | 0.9914779478 |
| plcb2 | -0.008656427011 | 0.06083766835 | -0.1422872908 | 0.8868530848 | 0.991380932 |
| prrt3 | -0.009000144184 | 0.06333951911 | -0.1420936614 | 0.8870060248 | 0.9910402359 |
| c1qa | -0.008438012623 | 0.05945675658 | -0.1419184817 | 0.8871443958 | 0.9915974875 |
| mcam | 0.008515042686 | 0.06019188165 | 0.1414649692 | 0.8875026322 | 1.008551399 |
| chad | -0.008784870968 | 0.06212924178 | -0.1413967194 | 0.8875565457 | 0.9912536033 |
| usp47 | -0.008640137695 | 0.06158713059 | -0.1402912851 | 0.8884298497 | 0.991397081 |
| cnst | -0.007880942149 | 0.05619124257 | -0.1402521423 | 0.8884607754 | 0.9921500311 |
| arhgef12 | 0.007993931301 | 0.05711042932 | 0.1399732307 | 0.8886811409 | 1.008025968 |
| nfatc1 | -0.008080972074 | 0.05774668979 | -0.1399382736 | 0.8887087609 | 0.9919515912 |
| plau | -0.008235479543 | 0.05891603795 | -0.1397833227 | 0.8888311905 | 0.9917983391 |
| daam1 | -0.008431392534 | 0.06038051672 | -0.1396376347 | 0.8889463038 | 0.991604052 |
| padi2 | 0.008062127232 | 0.05779903247 | 0.139485505 | 0.8890665093 | 1.008094714 |
| dhrs4l2 | 0.008540258016 | 0.06147933757 | 0.1389126551 | 0.8895191706 | 1.00857683 |
| babam1 | -0.008582762818 | 0.06179701958 | -0.1388863553 | 0.8895399534 | 0.9914539639 |
| ptpn6 | 0.007859316322 | 0.0566207529 | 0.1388062843 | 0.8896032279 | 1.007890282 |
| preb | 0.007851467342 | 0.05698654138 | 0.1377775726 | 0.8904162098 | 1.007882371 |
| dnlz | -0.008322377349 | 0.06085130859 | -0.1367657909 | 0.8912159244 | 0.9917121578 |
| ank2 | -0.008209310628 | 0.06008647686 | -0.1366249289 | 0.8913272709 | 0.9918242937 |
| snap25 | -0.008490319204 | 0.06232305923 | -0.1362307838 | 0.8916388401 | 0.9915456218 |
| snx5 | -0.008517674503 | 0.06261848787 | -0.1360249152 | 0.8918015845 | 0.9915184981 |
| lrch4 | 0.008482033705 | 0.06240310911 | 0.1359232549 | 0.8918819513 | 1.008518108 |
| siglec5 | -0.00765533529 | 0.05638883444 | -0.1357597717 | 0.8920111941 | 0.9923738922 |
| aifm1 | -0.007706498381 | 0.05752170585 | -0.133975484 | 0.8934219606 | 0.9923231205 |
| pga4 | -0.007957608595 | 0.05953793722 | -0.1336561018 | 0.8936745193 | 0.9920739694 |

| Protein | beta | se | z | p_val | HR |
|---|---|---|---|---|---|
| fut8 | -0.007535541579 | 0.05658929807 | -0.1331619553 | 0.8940652981 | 0.9924927794 |
| bcl2l11 | -0.007562073393 | 0.05721758076 | -0.1321634591 | 0.894855003 | 0.9924664471 |
| cxcl6 | 0.007446499559 | 0.05660127425 | 0.1315606346 | 0.8953318238 | 1.007474294 |
| ptprs | 0.007271904333 | 0.05611106301 | 0.1295984061 | 0.8968841643 | 1.007298409 |
| tmsb10 | 0.00770587374 | 0.059462355 | 0.1295924748 | 0.8968888573 | 1.00773564 |
| dner | 0.007904707522 | 0.06103595628 | 0.1295090305 | 0.8969548798 | 1.007936032 |
| stx8 | -0.007453597177 | 0.05766785004 | -0.1292504779 | 0.8971594556 | 0.992574112 |
| bank1 | 0.007318561292 | 0.05719068263 | 0.1279677205 | 0.8981745182 | 1.007345407 |
| rrm2b | -0.007425731649 | 0.05816744117 | -0.1276613085 | 0.8984170109 | 0.992601771 |
| fis1 | -0.00737419484 | 0.05793802184 | -0.1272772975 | 0.8987209282 | 0.9926529278 |
| psca | -0.007482314385 | 0.05882326108 | -0.1271999248 | 0.8987821649 | 0.9925456084 |
| htra2 | -0.007171849092 | 0.05647348623 | -0.1269949771 | 0.898944374 | 0.9928538072 |
| immt | -0.008098889386 | 0.06381384 | -0.1269143086 | 0.8990082216 | 0.9919338183 |
| arhgef5 | 0.00790997047 | 0.06266982292 | 0.126216576 | 0.8995604905 | 1.007941337 |
| cd200r1 | -0.007171758241 | 0.05707722194 | -0.1256500929 | 0.9000089087 | 0.9928538974 |
| tnfrsf9 | 0.007341134148 | 0.05876398438 | 0.1249257385 | 0.9005823416 | 1.007368146 |
| tbl1x | 0.007171560778 | 0.05754566304 | 0.1246238274 | 0.9008213639 | 1.007197338 |
| diablo | 0.006980383471 | 0.05609485128 | 0.1244389336 | 0.9009677484 | 1.007004803 |
| il17rb | 0.007390556616 | 0.05975380777 | 0.123683442 | 0.9015659224 | 1.007417934 |
| reg1b | 0.007239139881 | 0.05857211369 | 0.1235936254 | 0.9016370399 | 1.007265406 |
| hnf1a | 0.007551815294 | 0.06149500846 | 0.1228037118 | 0.9022625349 | 1.007580402 |
| colec12 | 0.008018054314 | 0.0654499627 | 0.1225066292 | 0.9024977962 | 1.008050285 |
| smad3 | -0.007409019777 | 0.06061965671 | -0.1222214077 | 0.9027236727 | 0.9926183594 |
| cpxm1 | 0.006773726866 | 0.05563218973 | 0.1217591272 | 0.9030897848 | 1.00679672 |
| angptl3 | -0.007498259444 | 0.06165499138 | -0.1216164219 | 0.9032028072 | 0.9925297824 |
| pnliprp1 | 0.007251826968 | 0.05991797469 | 0.1210292405 | 0.9036678749 | 1.007278185 |
| wasf3 | -0.006954219758 | 0.05751060559 | -0.1209206491 | 0.9037538866 | 0.9930699049 |
| pnlip | 0.007141256504 | 0.05909613711 | 0.1208413418 | 0.903816704 | 1.007166816 |
| hpse | -0.007326334133 | 0.0606763255 | -0.1207445255 | 0.9038933907 | 0.992700438 |
| lbr | 0.006908902044 | 0.05753549392 | 0.1200806941 | 0.9044192256 | 1.006932824 |
| cdc123 | -0.007287107846 | 0.06100443961 | -0.1194520906 | 0.9049171944 | 0.9927393787 |
| lrrfip1 | 0.007272543941 | 0.06092444264 | 0.1193698888 | 0.904982316 | 1.007299053 |
| clspn | -0.006851042408 | 0.05793446753 | -0.1182550337 | 0.9058655853 | 0.9931723725 |
| timp3 | -0.006727536093 | 0.05703313848 | -0.1179583707 | 0.9061006429 | 0.9932950431 |
| lipf | -0.007427737913 | 0.06329247706 | -0.1173557784 | 0.9065781256 | 0.9925997796 |
| tacc3 | 0.00664933068 | 0.05675884791 | 0.1171505576 | 0.9067407464 | 1.006671487 |
| rab39b | -0.007062051439 | 0.06052076413 | -0.1166880746 | 0.9071072408 | 0.9929628262 |
| gage2a | -0.007158796017 | 0.06156074615 | -0.1162883244 | 0.9074240386 | 0.9928667671 |
| tmed10 | -0.007170419982 | 0.06175122533 | -0.1161178575 | 0.9075591363 | 0.9928552261 |
| ppm1a | 0.006557799615 | 0.05655141822 | 0.1159617181 | 0.9076828816 | 1.006579349 |
| igf1r | 0.006862645352 | 0.05929413134 | 0.1157390318 | 0.9078593712 | 1.006886247 |
| cox6b1 | -0.006999397627 | 0.06064146706 | -0.1154226302 | 0.9081101425 | 0.9930250411 |
| dctn1 | 0.006461028494 | 0.05641733495 | 0.1145220436 | 0.9088239731 | 1.006481946 |
| tcl1a | -0.0064690383 | 0.05668535735 | -0.114121858 | 0.9091411953 | 0.9935518409 |
| bloc1s2 | -0.006878776329 | 0.06050629254 | -0.1136869578 | 0.9094859516 | 0.9931448283 |
| dnaja2 | -0.006501331423 | 0.05719100898 | -0.1136775087 | 0.9094934424 | 0.9935197565 |
| sell | 0.006914802159 | 0.0612819286 | 0.1128359097 | 0.9101606483 | 1.006938765 |
| pgr | -0.006877742903 | 0.06124695279 | -0.1122952668 | 0.9105892946 | 0.9931458546 |
| ppbp | 0.006787019381 | 0.06045049079 | 0.1122740162 | 0.9106061436 | 1.006810103 |
| gp1bb | -0.006782043854 | 0.06049623384 | -0.1121068771 | 0.9107386646 | 0.9932409023 |
| chek2 | 0.006623535234 | 0.0591260809 | 0.1120239179 | 0.9108044422 | 1.006645519 |
| cptp | -0.00680803368 | 0.06105981695 | -0.1114977741 | 0.9112216306 | 0.9932150885 |
| ccl22 | 0.006594847653 | 0.05925083437 | 0.1113038782 | 0.9113753802 | 1.006616642 |
| fabp9 | -0.006363422222 | 0.057413565 | -0.1108348214 | 0.911747332 | 0.9936567815 |
| ubqln3 | 0.006661714063 | 0.06033303423 | 0.1104156976 | 0.9120797044 | 1.006683953 |
| prtfdc1 | 0.006330898721 | 0.05756015716 | 0.1099875162 | 0.9124192754 | 1.006350981 |
| klrk1 | 0.006506919962 | 0.05922147081 | 0.109874339 | 0.9125090338 | 1.006528136 |
| adam22 | 0.006229647221 | 0.05693422724 | 0.1094183152 | 0.9128707075 | 1.006249092 |
| stx6 | 0.006276403446 | 0.0577457314 | 0.1086903446 | 0.9134481004 | 1.006296141 |
| rest | -0.006691560913 | 0.06165424191 | -0.1085336662 | 0.9135723764 | 0.9933307777 |
| cep85 | -0.00624343319 | 0.05753972086 | -0.1085064908 | 0.913593932 | 0.9937760165 |
| pik3ip1 | 0.006653281011 | 0.06134136811 | 0.1084631989 | 0.9136282713 | 1.006675463 |
| hspg2 | 0.006790574337 | 0.06278283942 | 0.1081597201 | 0.9138689962 | 1.006813683 |
| fcrlb | -0.00632473681 | 0.05868807242 | -0.1077686922 | 0.9141791782 | 0.9936952222 |
| hsd17b14 | -0.00655326918 | 0.06088627691 | -0.1076313007 | 0.9142881668 | 0.9934681567 |
| lrrc59 | -0.006470249664 | 0.06016322427 | -0.1075449287 | 0.914356684 | 0.9935506373 |
| c1galt1c1 | 0.006528439689 | 0.06082058034 | 0.1073393192 | 0.9145197925 | 1.006549796 |
| paep | -0.006994986611 | 0.06528083212 | -0.1071522281 | 0.9146682136 | 0.9930294214 |
| mep1b | -0.005995048572 | 0.05595723618 | -0.1071362523 | 0.9146808875 | 0.9940228859 |
| ifit1 | -0.006393794603 | 0.05973494206 | -0.1070360895 | 0.9147603489 | 0.9936266022 |

| Protein | beta | se | z | p_val | HR |
|---|---|---|---|---|---|
| cga | 0.009571791573 | 0.09035929274 | 0.1059303507 | 0.9156376131 | 1.009617748 |
| tor1aip1 | 0.006639981017 | 0.06288675959 | 0.105586312 | 0.9159105855 | 1.006662075 |
| vstm2l | 0.006094870037 | 0.05790080418 | 0.1052639963 | 0.916166331 | 1.006113482 |
| pcdh7 | 0.006611040207 | 0.06282085185 | 0.1052363986 | 0.9161882291 | 1.006632941 |
| amfr | -0.006055163944 | 0.05788036515 | -0.1046151649 | 0.9166811809 | 0.9939631316 |
| guk1 | -0.006401195967 | 0.06134297189 | -0.1043509268 | 0.9168908648 | 0.993619248 |
| pth1r | 0.005946485523 | 0.05737811247 | 0.1036368271 | 0.9174575612 | 1.005964201 |
| mesd | 0.005807529897 | 0.05661667291 | 0.1025763189 | 0.9182992384 | 1.005824426 |
| brdt | -0.006270515665 | 0.06115613408 | -0.1025328981 | 0.9183337015 | 0.993749103 |
| cuzd1 | 0.006331455284 | 0.06175729312 | 0.1025215803 | 0.9183426845 | 1.006351541 |
| nfic | 0.006111777602 | 0.0603630491 | 0.1012503128 | 0.9193517581 | 1.006130493 |
| dtnb | -0.00687005455 | 0.06798557251 | -0.101051654 | 0.919509456 | 0.9931534903 |
| adgrd1 | -0.007049308531 | 0.07018235199 | -0.1004427514 | 0.9199928307 | 0.9929754796 |
| mitd1 | 0.00572436131 | 0.05706359826 | 0.1003154635 | 0.9200938813 | 1.005740777 |
| cpvl | 0.00555575492 | 0.05578085671 | 0.09959966999 | 0.9206621558 | 1.005571217 |
| nxpe4 | -0.006008002498 | 0.0603947338 | -0.09947891348 | 0.9207580294 | 0.9940100095 |
| gsr | 0.006162452447 | 0.06214037825 | 0.09916985736 | 0.9210034072 | 1.006181479 |
| iqgap2 | -0.00573017331 | 0.05778691005 | -0.09916040336 | 0.9210109134 | 0.9942862128 |
| timd4 | 0.005709021886 | 0.05788609572 | 0.09862509839 | 0.9214359415 | 1.005725349 |
| jun | 0.005846138332 | 0.05927762077 | 0.09862302596 | 0.9214375871 | 1.00586326 |
| gprc5c | -0.005913898195 | 0.06014015267 | -0.09833527075 | 0.9216660719 | 0.9941035545 |
| cfd | 0.006299069296 | 0.06473265831 | 0.09730898531 | 0.9224810206 | 1.00631895 |
| ccn4 | 0.005755704463 | 0.05915890175 | 0.09729228049 | 0.9224942862 | 1.0057723 |
| fcrl6 | -0.005487865749 | 0.0564291142 | -0.09725238164 | 0.9225259706 | 0.9945271651 |
| cngb3 | -0.005953761485 | 0.06156687668 | -0.09670397145 | 0.9229614859 | 0.994063927 |
| ivd | 0.005585345447 | 0.0579403098 | 0.09639826687 | 0.9232042686 | 1.005600973 |
| pmvk | -0.005622180227 | 0.05847223987 | -0.09615127177 | 0.9234004309 | 0.9943935947 |
| trim58 | -0.005751399469 | 0.05983242498 | -0.09612512732 | 0.923421195 | 0.9942651082 |
| ly75 | -0.005427626193 | 0.0565230703 | -0.09602497111 | 0.9235007401 | 0.9945870768 |
| relb | 0.005913021166 | 0.06202439097 | 0.09533380455 | 0.9240496928 | 1.005930538 |
| srpx | 0.005990670096 | 0.06356557574 | 0.0942439367 | 0.9249153836 | 1.00600865 |
| bcam | -0.005432848893 | 0.05811530185 | -0.09348396583 | 0.9255190873 | 0.9945818823 |
| sox2 | 0.005693753359 | 0.06112654203 | 0.09314698934 | 0.9257867876 | 1.005709994 |
| morf4l2 | 0.005675984179 | 0.06100027283 | 0.09304850479 | 0.9258650271 | 1.005692123 |
| il20rb | -0.005702513791 | 0.06138143833 | -0.09290290268 | 0.9259806997 | 0.9943137147 |
| arhgap30 | 0.005657915006 | 0.06112153393 | 0.09256827574 | 0.9262465478 | 1.005673951 |
| bst2 | 0.005493114746 | 0.05957452531 | 0.09220576609 | 0.9265345569 | 1.00550823 |
| pcare | -0.00556885653 | 0.06065525143 | -0.09181161398 | 0.9268477164 | 0.9944466208 |
| anxa11 | 0.00519681364 | 0.057058295 | 0.09107902085 | 0.9274298023 | 1.00521034 |
| glrx5 | -0.005603583117 | 0.06182676524 | -0.09063361305 | 0.9277837225 | 0.9944120877 |
| c1ql2 | -0.005732473869 | 0.06424425135 | -0.08922936682 | 0.9288996271 | 0.9942839254 |
| il7 | 0.00514288896 | 0.05769342496 | 0.08914168233 | 0.9289693115 | 1.005156136 |
| pf4 | 0.00535480228 | 0.06017704267 | 0.08898413817 | 0.929094516 | 1.005369165 |
| myl1 | -0.005436682345 | 0.06121061431 | -0.08881927434 | 0.9292255395 | 0.9945780697 |
| ctrb1 | 0.004947074439 | 0.05590015271 | 0.08849840652 | 0.9294805508 | 1.004959331 |
| il10 | 0.005006695159 | 0.05659022331 | 0.08847279382 | 0.9295009069 | 1.00501925 |
| cntnap4 | -0.005465936132 | 0.06204174003 | -0.08810094832 | 0.9297964427 | 0.9945489749 |
| dag1 | 0.00508608867 | 0.05885293067 | 0.08642031268 | 0.9311323002 | 1.005099045 |
| lifr | 0.004993543339 | 0.05782879675 | 0.08635046238 | 0.9311878251 | 1.005006032 |
| vmo1 | -0.004965440783 | 0.05765761824 | -0.08611942245 | 0.9313714841 | 0.9950468666 |
| ggh | -0.005236351211 | 0.06115233899 | -0.0856279792 | 0.931762156 | 0.9947773346 |
| cd200 | 0.005072557749 | 0.05926052373 | 0.08559758555 | 0.9317863179 | 1.005085445 |
| mpig6b | -0.004734028122 | 0.05570963408 | -0.08497683031 | 0.9322798108 | 0.9952771597 |
| scamp3 | -0.004844421734 | 0.05713861139 | -0.08478367983 | 0.9324333684 | 0.9951672936 |
| hdgfl2 | 0.005246427777 | 0.06196697974 | 0.08466489409 | 0.9325278062 | 1.005260214 |
| dpt | 0.005307742421 | 0.06322394185 | 0.0839514631 | 0.9330950223 | 1.005321853 |
| ctf1 | 0.004853587419 | 0.05810388777 | 0.08353292017 | 0.9334278023 | 1.004865385 |
| samd9l | -0.004828941548 | 0.05785420387 | -0.08346742716 | 0.9334798764 | 0.995182699 |
| icam2 | 0.00462606295 | 0.05580657003 | 0.08289459373 | 0.9339353528 | 1.00463678 |
| ifng | -0.004644828142 | 0.05666637515 | -0.08196797713 | 0.9346721783 | 0.9953659424 |
| st8sia1 | 0.005035304742 | 0.06159983418 | 0.08174218014 | 0.9348517357 | 1.005048003 |
| f7 | 0.004768576563 | 0.05901818645 | 0.08079842586 | 0.93560226 | 1.004779964 |
| hnrnpk | 0.004705617591 | 0.05872481303 | 0.08012997144 | 0.9361338856 | 1.004716706 |
| tnfrsf8 | 0.004573050446 | 0.05710561807 | 0.08008057001 | 0.936173176 | 1.004583523 |
| crebzf | 0.004961769566 | 0.06208076539 | 0.07992442642 | 0.9362973625 | 1.0049741 |
| il10rb | 0.004775738833 | 0.05983351692 | 0.07981711721 | 0.9363827102 | 1.004787161 |
| izumo1 | 0.004867899862 | 0.0609942014 | 0.07980922367 | 0.9363889883 | 1.004879767 |
| adgre1 | 0.004853283963 | 0.06081504718 | 0.07980399898 | 0.9363931437 | 1.00486508 |
| maneal | -0.004888645101 | 0.06139573357 | -0.07962515986 | 0.9365353841 | 0.9951232849 |
| enoph1 | 0.004772615281 | 0.06034932766 | 0.07908315578 | 0.9369664813 | 1.004784022 |

| Protein | beta | se | z | p_val | HR |
|---|---|---|---|---|---|
| tp73 | -0.004698915409 | 0.05973436967 | -0.07866351372 | 0.9373002673 | 0.9953121072 |
| luzp2 | -0.004898112742 | 0.06250509274 | -0.07836341852 | 0.9375389718 | 0.9951138635 |
| ctnna1 | -0.004717129408 | 0.06083435095 | -0.07754055619 | 0.9381935292 | 0.9952939788 |
| spred2 | -0.004788453301 | 0.06283635963 | -0.07620513552 | 0.939255897 | 0.9952229931 |
| pfkfb2 | 0.004358481997 | 0.05733019864 | 0.07602419145 | 0.9393998519 | 1.004367994 |
| hip1 | -0.004699975135 | 0.06185675251 | -0.07598160175 | 0.9394337356 | 0.9953110525 |
| tmed4 | -0.004635879698 | 0.06110837088 | -0.07586325133 | 0.9395278938 | 0.9953748494 |
| jchain | -0.00426341017 | 0.05649570796 | -0.07546431976 | 0.9398452853 | 0.9957456653 |
| gys1 | -0.004387747172 | 0.05832993794 | -0.07522290143 | 0.9400373633 | 0.9956218649 |
| lyzl2 | -0.005166009066 | 0.06905130913 | -0.07481406408 | 0.9403626517 | 0.9948473118 |
| fuca1 | -0.004142926043 | 0.05544992714 | -0.07471472474 | 0.9404416918 | 0.995865644 |
| clgn | -0.00465428856 | 0.06234385489 | -0.07465512949 | 0.9404891095 | 0.9953565259 |
| arhgap5 | 0.004426611404 | 0.06013796593 | 0.07360760105 | 0.9413226228 | 1.004436423 |
| fstl1 | -0.004653747093 | 0.06336755093 | -0.07344053897 | 0.9414555592 | 0.9953570648 |
| stk4 | -0.004127193339 | 0.05668821687 | -0.072805136 | 0.9419611839 | 0.9958813118 |
| pcsk9 | -0.004280907516 | 0.05919151533 | -0.07232299244 | 0.9423448673 | 0.9957282425 |
| morf4l1 | 0.004376932881 | 0.0607269984 | 0.07207556765 | 0.9425417698 | 1.004386526 |
| eno1 | 0.004102427845 | 0.05692259538 | 0.07207028804 | 0.9425459714 | 1.004110854 |
| mrpl28 | 0.004323793524 | 0.06007757859 | 0.07197016966 | 0.9426256474 | 1.004333155 |
| ahsa1 | -0.004352898984 | 0.06068152062 | -0.07173351855 | 0.9428139809 | 0.9956565611 |
| spint1 | 0.004062443359 | 0.05706229923 | 0.07119312426 | 0.9432440536 | 1.004070706 |
| angptl1 | 0.004169162227 | 0.05947831998 | 0.0700954941 | 0.9441176531 | 1.004177865 |
| grpel1 | -0.004134843145 | 0.0591227944 | -0.06993653102 | 0.9442441767 | 0.9958736935 |
| ambp | -0.004122355733 | 0.0599638218 | -0.06874738148 | 0.9451907024 | 0.9958861295 |
| thrap3 | 0.00420947904 | 0.06125941208 | 0.0687156291 | 0.9452159774 | 1.004218351 |
| tf | 0.004202277668 | 0.0618713938 | 0.06791955716 | 0.9458496703 | 1.00421112 |
| arf6 | -0.004102386129 | 0.06066809455 | -0.06762015783 | 0.9460880085 | 0.9959060172 |
| ccl23 | -0.003884306917 | 0.0578981486 | -0.06708862048 | 0.946511153 | 0.9961232272 |
| ifnlr1 | 0.004019473172 | 0.06004710564 | 0.06693866639 | 0.9466305307 | 1.004027562 |
| nid2 | -0.003691052184 | 0.05600313104 | -0.06590796113 | 0.9474511023 | 0.9963157514 |
| cd93 | 0.003864589624 | 0.05889744363 | 0.0656155749 | 0.9476838889 | 1.003872067 |
| dgcr6 | -0.00401841552 | 0.06124417161 | -0.06561302756 | 0.947685917 | 0.9959896475 |
| nrxn3 | -0.003952720435 | 0.06192881884 | -0.06382683391 | 0.9491081114 | 0.9960550813 |
| tlr3 | 0.003628958509 | 0.05693215484 | 0.06374180846 | 0.9491758141 | 1.003635551 |
| stam | -0.003842140994 | 0.06070163646 | -0.06329550927 | 0.9495311917 | 0.9961652306 |
| cdh15 | -0.004308615358 | 0.06842552078 | -0.06296795857 | 0.949792019 | 0.9957006534 |
| lypd1 | 0.003586885127 | 0.0572706115 | 0.06263046671 | 0.9500607681 | 1.003593326 |
| tbc1d23 | -0.003487720396 | 0.05684616287 | -0.06135366436 | 0.9510775533 | 0.9965183546 |
| txnl1 | 0.00377391062 | 0.06186194455 | 0.06100536683 | 0.9513549349 | 1.003781041 |
| ccdc134 | -0.003668168885 | 0.06069431298 | -0.06043678073 | 0.9518077654 | 0.9963385506 |
| psmc3 | 0.003641327814 | 0.06031862581 | 0.06036821571 | 0.9518623727 | 1.003647966 |
| tfpi | -0.003635571975 | 0.06049238951 | -0.0600996589 | 0.9520762616 | 0.9963710287 |
| prelp | 0.003459861382 | 0.05759428918 | 0.0600729939 | 0.9520974989 | 1.003465854 |
| galnt10 | -0.003544684899 | 0.05923314636 | -0.05984292776 | 0.9522807354 | 0.9964615901 |
| serpinb1 | -0.003414179313 | 0.05744578312 | -0.0594330711 | 0.9526071727 | 0.9965916424 |
| mtpn | 0.003407210154 | 0.0582211742 | 0.05852183851 | 0.9533329677 | 1.003413021 |
| galnt2 | 0.00331031521 | 0.05685238784 | 0.05822649383 | 0.9535682175 | 1.0033158 |
| nub1 | 0.003337645591 | 0.05737565713 | 0.05817180593 | 0.9536117783 | 1.003343222 |
| fli1 | -0.003272760215 | 0.05761634873 | -0.05680263132 | 0.9547024178 | 0.9967325894 |
| paip2b | -0.00346403284 | 0.06123626716 | -0.05656832137 | 0.95488907 | 0.99654196 |
| amdhd2 | 0.003380640817 | 0.05982083132 | 0.05651276892 | 0.9549333237 | 1.003386362 |
| tymp | 0.003173028357 | 0.05715702851 | 0.05551422879 | 0.9557287945 | 1.003178068 |
| jam2 | 0.003264112399 | 0.0593426741 | 0.05500447103 | 0.9561349018 | 1.003269445 |
| lgals1 | -0.003452656362 | 0.06286288195 | -0.05492360921 | 0.9561993228 | 0.9965532972 |
| cpe | 0.00311453459 | 0.05701218648 | 0.05462927809 | 0.956433813 | 1.00311939 |
| arid3a | 0.003382826017 | 0.06196666349 | 0.05459106278 | 0.956464259 | 1.003388554 |
| bcl7a | -0.003267247399 | 0.06036160502 | -0.05412790792 | 0.9568332575 | 0.9967380842 |
| il18rap | 0.003023724023 | 0.05594865719 | 0.05404462189 | 0.956899613 | 1.0030283 |
| ppp1r2 | -0.003047884901 | 0.05687745966 | -0.05358686761 | 0.9572643196 | 0.9969567552 |
| cebpa | -0.003313807638 | 0.06247563254 | -0.05304160204 | 0.9576987607 | 0.996691677 |
| clec3b | 0.003242231279 | 0.0615272913 | 0.05269582343 | 0.9579742668 | 1.003247493 |
| angpt2 | 0.003148597967 | 0.060109915 | 0.05238067575 | 0.9582253715 | 1.00315356 |
| ccl18 | 0.003261607562 | 0.06272928047 | 0.05199497806 | 0.9585326949 | 1.003266932 |
| il22ra1 | 0.003019934092 | 0.05833723133 | 0.05176683951 | 0.9587144783 | 1.003024499 |
| crx | 0.002914491178 | 0.05717801387 | 0.05097223531 | 0.9593476448 | 1.002918742 |
| ppie | 0.003039264543 | 0.05969741121 | 0.05091116149 | 0.9593963115 | 1.003043888 |
| fbn2 | 0.003106459401 | 0.06128157994 | 0.05069156839 | 0.9595712955 | 1.003111289 |
| cep170 | 0.003083948824 | 0.06107145528 | 0.05049738556 | 0.9597260328 | 1.003088709 |
| vwa1 | 0.003004479996 | 0.05968141154 | 0.05034197279 | 0.9598498767 | 1.003008998 |
| hsd17b3 | 0.003051632575 | 0.06067223189 | 0.05029702188 | 0.959885697 | 1.003056294 |

| Protein | beta | se | z | p_val | HR |
|---|---|---|---|---|---|
| igfbp7 | 0.003045360387 | 0.06106377152 | 0.04987180306 | 0.9602245472 | 1.003050002 |
| anxa3 | 0.002803807067 | 0.05682828223 | 0.04933823366 | 0.9606497505 | 1.002807741 |
| hpcal1 | -0.002731673084 | 0.05670258397 | -0.04817546031 | 0.9615764073 | 0.9972720545 |
| npl | 0.003321196441 | 0.06926051935 | 0.04795223125 | 0.9617543127 | 1.003326718 |
| pdzk1 | -0.003103662003 | 0.0653081068 | -0.04752338041 | 0.9620960965 | 0.9969011494 |
| fgf7 | -0.002936159139 | 0.06237607112 | -0.04707188328 | 0.9624559364 | 0.9970681472 |
| vamp8 | 0.002865543151 | 0.06158211129 | 0.04653207062 | 0.9628861731 | 1.002869653 |
| clec4g | 0.002709890603 | 0.05875522636 | 0.04612169455 | 0.9632132547 | 1.002713566 |
| leo1 | -0.002765664894 | 0.06040614785 | -0.04578449367 | 0.9634820181 | 0.997238156 |
| crkl | 0.002604311344 | 0.05702814622 | 0.04566712259 | 0.9635755688 | 1.002607706 |
| cdk5rap3 | -0.002751173824 | 0.06075128233 | -0.04528585601 | 0.9638794611 | 0.9972526072 |
| fst | -0.002688212357 | 0.05975140612 | -0.04498994302 | 0.9641153251 | 0.9973153977 |
| acp5 | -0.002689743088 | 0.06000088209 | -0.04482839242 | 0.964244094 | 0.997313871 |
| lag3 | -0.002684916923 | 0.06011925916 | -0.04465984711 | 0.9643784391 | 0.9973186842 |
| fgfbp1 | 0.002576945865 | 0.05910984922 | 0.04359587953 | 0.9652265362 | 1.002580269 |
| ninj1 | 0.002532424291 | 0.05921087863 | 0.04276957798 | 0.965885215 | 1.002535634 |
| inpp1 | 0.002474653386 | 0.05874512736 | 0.04212525357 | 0.9663988486 | 1.002477718 |
| c1rl | -0.002666474293 | 0.06405934117 | -0.04162506582 | 0.9667975909 | 0.9973370776 |
| hla_dra | -0.002384776168 | 0.05736406225 | -0.0415726515 | 0.9668393753 | 0.9976180652 |
| azi2 | 0.002391541607 | 0.06021412767 | 0.03971728396 | 0.9683185219 | 1.002394404 |
| atp5f1d | 0.002412212342 | 0.06086696006 | 0.03963089892 | 0.968387393 | 1.002415124 |
| ggact | 0.002395124334 | 0.06059499415 | 0.03952676896 | 0.9684704116 | 1.002397995 |
| prc1 | -0.002378652801 | 0.06048010603 | -0.03932950778 | 0.968627681 | 0.997624174 |
| il1a | 0.002224710185 | 0.05691330808 | 0.03908945482 | 0.9688190684 | 1.002227187 |
| gsn | -0.002360675958 | 0.06056977474 | -0.03897448799 | 0.9689107288 | 0.9976421082 |
| dnm1 | -0.002343627905 | 0.06043677157 | -0.03877817832 | 0.9690672429 | 0.9976591162 |
| epha10 | -0.002239425463 | 0.05812426779 | -0.03852823524 | 0.9692665197 | 0.9977630802 |
| nagk | -0.002437708768 | 0.06424784869 | -0.03794226294 | 0.9697337163 | 0.99756526 |
| pla2g4a | -0.002092287457 | 0.05670579801 | -0.03689724032 | 0.9705669402 | 0.9979098999 |
| rcc1 | 0.002174943218 | 0.06257350795 | 0.0347582114 | 0.972272543 | 1.00217731 |
| kirrel1 | -0.002083570899 | 0.06055240977 | -0.03440938035 | 0.9725507035 | 0.9979185982 |
| lat2 | 0.001947860922 | 0.05706927084 | 0.03413151935 | 0.9727722743 | 1.001949759 |
| bst1 | 0.001946001448 | 0.05717049393 | 0.0340385628 | 0.9728463998 | 1.001947896 |
| sestd1 | 0.001954629675 | 0.05775423083 | 0.03384392186 | 0.9730016114 | 1.001956541 |
| satb1 | 0.0020117411 | 0.06102643314 | 0.03296507754 | 0.9737024366 | 1.002013766 |
| clec12a | 0.002023242692 | 0.06167360478 | 0.03280564999 | 0.9738295726 | 1.002025291 |
| il31 | -0.001961709089 | 0.06165255061 | -0.03181878235 | 0.9746165681 | 0.9980402138 |
| kazald1 | -0.001829823813 | 0.05751047194 | -0.03181722826 | 0.9746178074 | 0.9981718493 |
| txndc15 | 0.001759286788 | 0.05689032329 | 0.03092418334 | 0.9753300036 | 1.001760835 |
| myom1 | 0.001891086198 | 0.06185959935 | 0.03057061828 | 0.9756119744 | 1.001892875 |
| il31ra | 0.001845946559 | 0.06204365305 | 0.0297523835 | 0.9762645344 | 1.001847651 |
| trdmt1 | 0.001734858007 | 0.05884420529 | 0.02948222342 | 0.9764799964 | 1.001736364 |
| serpina5 | -0.001831525352 | 0.06342455545 | -0.02887722806 | 0.9769625074 | 0.9981701509 |
| pmm2 | 0.001736992967 | 0.06127832924 | 0.02834595832 | 0.9773862259 | 1.001738502 |
| tmprss5 | 0.001648339199 | 0.0588107899 | 0.02802783642 | 0.9776399496 | 1.001649698 |
| pfdn6 | -0.001728970701 | 0.06188008079 | -0.02794066651 | 0.9777094739 | 0.9982725231 |
| crtac1 | -0.001566129199 | 0.05751883716 | -0.02722810954 | 0.9782777958 | 0.9984350965 |
| susd2 | -0.001626558648 | 0.06025565318 | -0.02699429119 | 0.9784642873 | 0.9983747635 |
| galnt7 | 0.001486676664 | 0.05612386269 | 0.02648920784 | 0.9788671415 | 1.001487782 |
| rtn4ip1 | -0.001586489121 | 0.0601949068 | -0.02635586971 | 0.9789734928 | 0.9984147687 |
| pts | -0.001608380212 | 0.06131792514 | -0.02623017998 | 0.979073744 | 0.9983929125 |
| aplp1 | 0.001475055867 | 0.05893344688 | 0.02502918028 | 0.9800316884 | 1.001476144 |
| snap23 | -0.001374353511 | 0.05658428694 | -0.02428860706 | 0.9806224007 | 0.9986265905 |
| chchd6 | 0.001518134225 | 0.06377687449 | 0.02380383544 | 0.9810090807 | 1.001519287 |
| tnfaip8 | -0.001354455971 | 0.05720236185 | -0.02367832249 | 0.9811091973 | 0.9986464609 |
| lsp1 | -0.001382346942 | 0.05975266572 | -0.02313448154 | 0.9815430007 | 0.9986186081 |
| rbfox3 | -0.001603116694 | 0.07075115358 | -0.02265852375 | 0.9819226606 | 0.9983981676 |
| trim40 | -0.001326231996 | 0.0604894076 | -0.02192502867 | 0.9825077596 | 0.9986746471 |
| arsb | -0.001267093641 | 0.057917762 | -0.02187746207 | 0.9825457031 | 0.9987337088 |
| m6pr | 0.001344987553 | 0.06177086466 | 0.02177381782 | 0.9826283796 | 1.001345892 |
| nfatc3 | -0.0012365998 | 0.05796381453 | -0.02133399622 | 0.9829792249 | 0.9987641645 |
| il13 | 0.001192401166 | 0.05772180372 | 0.02065772531 | 0.9835186921 | 1.001193112 |
| bmp10 | -0.001347732429 | 0.065501969 | -0.0205754491 | 0.9835843251 | 0.9986531754 |
| fadd | -0.001166534155 | 0.05670612544 | -0.02057157223 | 0.9835874177 | 0.998834146 |
| gfod2 | -0.001143250317 | 0.05681344536 | -0.02012288305 | 0.9839453458 | 0.9988574029 |
| pdgfrb | 0.001090950974 | 0.05588715767 | 0.01952060222 | 0.984425802 | 1.001091546 |
| sec31a | 0.001199234776 | 0.06220716384 | 0.01927808153 | 0.9846192691 | 1.001199954 |
| ppib | 0.001076127122 | 0.05668694357 | 0.01898368573 | 0.98485412 | 1.001076706 |
| cdnf | -0.001107018693 | 0.05839774693 | -0.01895653088 | 0.9848757825 | 0.9988935938 |
| ctsh | -0.001088726989 | 0.05897459553 | -0.01846094882 | 0.9852711306 | 0.9989118655 |

| Protein | beta | se | z | p_val | HR |
|---|---|---|---|---|---|
| lif | -0.001063337664 | 0.05773724718 | -0.01841684036 | 0.9853063181 | 0.9989372275 |
| src | 0.001034735126 | 0.05672005484 | 0.01824284425 | 0.9854451235 | 1.001035271 |
| sparc | 0.0009386621135 | 0.05598441751 | 0.01676648888 | 0.9866229041 | 1.000939103 |
| ervv_1 | 0.0009566774032 | 0.06095415843 | 0.01569503095 | 0.9874776912 | 1.000957135 |
| ebag9 | -0.0008860089675 | 0.05675073922 | -0.01561228946 | 0.9875437013 | 0.9991143834 |
| npdc1 | 0.0009115582246 | 0.05901482344 | 0.01544625861 | 0.9876761588 | 1.000911974 |
| pms1 | -0.0009360939332 | 0.06079609888 | -0.01539726973 | 0.9877152416 | 0.9990643441 |
| tdrkh | 0.0008817763424 | 0.05838627145 | 0.01510246023 | 0.9879504382 | 1.000882165 |
| ap3s2 | 0.0008737139094 | 0.06105192657 | 0.01431099653 | 0.9885818666 | 1.000874096 |
| rln2 | 0.000962625732 | 0.06792782162 | 0.01417130285 | 0.9886933147 | 1.000963089 |
| txndc5 | 0.0007975331043 | 0.05739410146 | 0.01389573291 | 0.988913166 | 1.000797851 |
| ankra2 | 0.0008557016765 | 0.06181906323 | 0.01384203564 | 0.9889560061 | 1.000856068 |
| gnpda2 | 0.000805319869 | 0.06276556457 | 0.01283060026 | 0.989762943 | 1.000805644 |
| adm | -0.0008725682904 | 0.06924178113 | -0.01260175975 | 0.9899455166 | 0.9991278123 |
| lgals8 | 0.0007216370199 | 0.05772075362 | 0.01250221064 | 0.990024939 | 1.000721897 |
| fgfbp3 | -0.0007475266263 | 0.062520942 | -0.01195641976 | 0.9904603846 | 0.9992527527 |
| crym | -0.0007188562884 | 0.06135305737 | -0.01171671501 | 0.9906516279 | 0.999281402 |
| gopc | 0.0006358136697 | 0.0570236554 | 0.01114999846 | 0.9911037727 | 1.000636016 |
| smarca2 | 0.000597673852 | 0.0577500719 | 0.01034931789 | 0.9917425865 | 1.000597852 |
| fcn1 | 0.0006122656511 | 0.06297872704 | 0.009721785115 | 0.9922432599 | 1.000612453 |
| rnaseh2a | 0.0005959247255 | 0.06152966426 | 0.00968516134 | 0.9922724801 | 1.000596102 |
| map2k6 | -0.0005401630293 | 0.05714233647 | -0.009452939145 | 0.9924577581 | 0.9994599828 |
| cd63 | -0.0004952038609 | 0.05717804378 | -0.008660734579 | 0.99308982 | 0.9995049187 |
| krt8 | -0.0004893771736 | 0.06195428014 | -0.007899005081 | 0.9936975713 | 0.9995107426 |
| gabarap | 0.0004595460084 | 0.06103387652 | 0.00752935967 | 0.9939924969 | 1.000459652 |
| stx16 | -0.0003529340175 | 0.05654611482 | -0.006241525499 | 0.9950200155 | 0.9996471283 |
| atf2 | -0.0003484426675 | 0.05865347417 | -0.005940699549 | 0.9952600354 | 0.999651618 |
| apobr | 0.000347856049 | 0.06153925241 | 0.005652588151 | 0.9954899112 | 1.000347917 |
| tspyl1 | 0.0002974595689 | 0.06270394068 | 0.004743873602 | 0.9962149507 | 1.000297504 |
| ccer2 | -0.0002367692535 | 0.06202513273 | -0.003817311517 | 0.9969542335 | 0.9997632588 |
| eppk1 | 0.0001886417882 | 0.0629674819 | 0.002995860443 | 0.9976096528 | 1.00018866 |
| efna1 | -0.0001641420843 | 0.05797080618 | -0.002831461128 | 0.9977408239 | 0.9998358714 |
| dda1 | -0.0001282809737 | 0.06059388407 | -0.00211706141 | 0.9983108306 | 0.9998717273 |
| itgav | -0.0001173326816 | 0.06040336149 | -0.00194248596 | 0.9984501214 | 0.9998826742 |
| oplah | -0.0001059507406 | 0.06174664887 | -0.001715894587 | 0.9986309149 | 0.9998940549 |
| calcoco2 | 9.87661e-05 | 0.06213768389 | 0.001589472598 | 0.9987317849 | 1.000098771 |
| camsap1 | -8.60374e-05 | 0.05993652756 | -0.001435475726 | 0.9988546565 | 0.9999139663 |
| edem2 | 8.60775e-05 | 0.06012215024 | 0.001431710839 | 0.9988576604 | 1.000086081 |
| lamp3 | 6.44342e-05 | 0.06179070152 | 0.001042781897 | 0.9991679806 | 1.000064436 |
| cetn2 | 2.55103e-05 | 0.0583787189 | 0.0004369793721 | 0.9996513409 | 1.000025511 |
| ism2 | -1.57355e-05 | 0.06022935505 | -0.0002612604619 | 0.9997915443 | 0.9999842646 |

## Supplementary Table 9. Associations between circulating plasma proteins and incident cataract in the UK Biobank

| Protein | beta | se | z | p_val | HR |
|---|---|---|---|---|---|
| crybb2 | 0.3739456563 | 0.03409067181 | 10.96914893 | 5.37762e-28 | 1.453458162 |
| pklr | 0.1584171488 | 0.03104926398 | 5.102122513 | 3.35865e-07 | 1.171654847 |
| ampd3 | 0.1636536879 | 0.03317721181 | 4.93271372 | 8.1095e-07 | 1.177806356 |
| dnph1 | 0.1465841121 | 0.03140081062 | 4.668163312 | 3.03904e-06 | 1.157872318 |
| glrx | 0.1435508246 | 0.03162870756 | 4.538624423 | 5.66224e-06 | 1.15436548 |
| ca1 | 0.1432913267 | 0.03199164394 | 4.479023554 | 7.49853e-06 | 1.154065963 |
| psmg3 | 0.1416871918 | 0.03164553796 | 4.477319741 | 7.5586e-06 | 1.15221617 |
| ca2 | 0.1423622765 | 0.0318289225 | 4.472733141 | 7.72261e-06 | 1.152994276 |
| aldh1a1 | 0.1451250041 | 0.03292143096 | 4.408222846 | 1.04222e-05 | 1.156184089 |
| tgm2 | 0.1367665438 | 0.03123982408 | 4.377954992 | 1.19798e-05 | 1.146560446 |
| blvrb | 0.1345480344 | 0.03148014022 | 4.274060836 | 1.91945e-05 | 1.14401961 |
| ubac1 | 0.1346303184 | 0.03166111544 | 4.252229162 | 2.11653e-05 | 1.144113748 |
| hmbs | 0.1263380914 | 0.03133491894 | 4.031862716 | 5.53365e-05 | 1.134665724 |
| rnase3 | 0.1258184088 | 0.03140259991 | 4.006623947 | 6.15928e-05 | 1.134076211 |
| psmd9 | 0.1238800139 | 0.03168419597 | 3.909836122 | 9.23588e-05 | 1.131880053 |
| sod1 | 0.1245381386 | 0.03210482238 | 3.879110032 | 0.0001048393274 | 1.132625216 |
| cat | 0.1301607836 | 0.03437966575 | 3.785981648 | 0.0001531029742 | 1.139011503 |
| eif4ebp1 | 0.1177691655 | 0.03192955336 | 3.688406293 | 0.0002256631057 | 1.124984396 |
| ahsp | 0.1186504762 | 0.03227043518 | 3.676754762 | 0.0002362199334 | 1.125976294 |
| aarsd1 | 0.1171358583 | 0.03194325754 | 3.6669979 | 0.0002454148099 | 1.124272161 |
| pdcd5 | 0.1157501199 | 0.03168995632 | 3.652580608 | 0.0002596180605 | 1.122715293 |
| psmg4 | 0.1238663592 | 0.03397563053 | 3.645741295 | 0.0002666221029 | 1.131864598 |
| ankrd54 | 0.1140903772 | 0.03145943028 | 3.626587518 | 0.0002871915095 | 1.12085342 |
| ncf2 | 0.1126886037 | 0.03134029062 | 3.595646417 | 0.0003235870654 | 1.119283338 |
| rbks | 0.1184561159 | 0.03294595155 | 3.595468042 | 0.0003238088796 | 1.12575747 |
| zbtb17 | 0.1142365404 | 0.0326588283 | 3.497876266 | 0.0004689786576 | 1.121017259 |
| lhpp | 0.1106967395 | 0.03174285044 | 3.487296759 | 0.0004879296194 | 1.117056096 |
| ddi2 | 0.1159781055 | 0.03329902314 | 3.482928162 | 0.0004959614318 | 1.122971285 |
| c9orf40 | 0.1135277293 | 0.03290913547 | 3.449732961 | 0.0005611413328 | 1.120222951 |
| gclm | 0.1148564734 | 0.03349012177 | 3.42956273 | 0.000604554669 | 1.12171243 |
| mnda | 0.1066853024 | 0.03119384154 | 3.420075794 | 0.0006260369056 | 1.112584072 |
| bola2_bola2b | 0.1166427605 | 0.03414098842 | 3.416502156 | 0.0006343116151 | 1.123717922 |
| hagh | 0.1080459881 | 0.03176958689 | 3.400925182 | 0.0006715820601 | 1.114098979 |
| sh3glb2 | 0.1135805214 | 0.03373763788 | 3.366581912 | 0.0007610597595 | 1.120282092 |
| prdx2 | 0.1131912522 | 0.03376150319 | 3.352672173 | 0.0008003544421 | 1.119846086 |
| rln2 | -0.1213092086 | 0.03629361311 | -3.342439569 | 0.0008304543562 | 0.8857600326 |
| cd109 | 0.1044434162 | 0.03131250645 | 3.335517594 | 0.0008514076625 | 1.110092579 |
| anxa2 | 0.1143207968 | 0.0343996361 | 3.323314131 | 0.0008895468089 | 1.121111716 |
| yod1 | 0.1136392969 | 0.03443090759 | 3.30050251 | 0.0009651185037 | 1.120347939 |
| afm | 0.1176099721 | 0.0357924721 | 3.285885696 | 0.001016622548 | 1.12480532 |
| rabgap1l | 0.104714392 | 0.03187399657 | 3.285260816 | 0.001018880083 | 1.110393428 |
| psme2 | 0.1047781302 | 0.03192271092 | 3.282244121 | 0.001029844051 | 1.110464205 |
| cebpb | 0.105102109 | 0.03205898522 | 3.278397875 | 0.001043981323 | 1.11082403 |
| zp3 | -0.1078695756 | 0.03291232621 | -3.277482573 | 0.001047371956 | 0.8977446766 |
| ccl11 | 0.1096363905 | 0.03347663357 | 3.275012414 | 0.001056573305 | 1.115872255 |
| myom3 | -0.1186843232 | 0.03625912573 | -3.27322628 | 0.001063273189 | 0.8880881053 |
| thop1 | 0.1065747123 | 0.03264738294 | 3.264418236 | 0.001096891171 | 1.112461038 |
| atg4a | 0.1041193038 | 0.03189746167 | 3.264187756 | 0.001097783909 | 1.109732842 |
| slc4a1 | 0.1098755257 | 0.03378052028 | 3.252629764 | 0.001143423737 | 1.116139131 |
| ddt | 0.1115393523 | 0.03434196219 | 3.247902717 | 0.001162590127 | 1.117997739 |
| gsap | 0.1008633625 | 0.03111766409 | 3.24135392 | 0.001189633873 | 1.106125493 |
| pik3ap1 | 0.1007527789 | 0.0313267426 | 3.216190721 | 0.001299044696 | 1.106003181 |
| egln1 | 0.1046196969 | 0.03259569259 | 3.209617241 | 0.001329118305 | 1.110288284 |
| ccs | 0.1005684888 | 0.03139671053 | 3.203153678 | 0.001359314232 | 1.105799374 |
| pebp1 | 0.1017461579 | 0.03197072136 | 3.182479268 | 0.001460199692 | 1.107102407 |
| dnaja4 | 0.1062625819 | 0.03343581446 | 3.178106579 | 0.00148240248 | 1.112113859 |
| dnajb2 | 0.1069550783 | 0.03384195104 | 3.160428846 | 0.001575370781 | 1.112884261 |
| nadk | 0.100384599 | 0.03208680645 | 3.128531947 | 0.001756819119 | 1.105596047 |
| hbq1 | 0.09752679325 | 0.03126867791 | 3.118993183 | 0.001814701595 | 1.102440979 |
| ppcdc | 0.09768431616 | 0.03138406114 | 3.112545432 | 0.001854814627 | 1.102614653 |
| serpina11 | 0.1062288978 | 0.0342198101 | 3.104309972 | 0.001907233998 | 1.112076399 |
| txn | 0.1043305564 | 0.03383409966 | 3.083591921 | 0.002045179184 | 1.109967301 |
| tppp2 | -0.1020494271 | 0.03319952908 | -3.073821525 | 0.002113358211 | 0.9029849186 |
| ssc5d | 0.102431933 | 0.03336095668 | 3.070413536 | 0.002137625699 | 1.10786189 |
| rassf2 | 0.0995308678 | 0.03244586433 | 3.067597978 | 0.002157867079 | 1.104652568 |
| tbc1d17 | 0.09756612325 | 0.03180818834 | 3.067327262 | 0.002159822518 | 1.102484339 |
| arg1 | 0.09637363439 | 0.03142859635 | 3.06643139 | 0.002166305186 | 1.101170422 |
| eloa | 0.09981876028 | 0.03255497457 | 3.066159984 | 0.002168272642 | 1.104970635 |

| Protein | beta | se | z | p_val | HR |
|---|---|---|---|---|---|
| cdhr1 | 0.09776465316 | 0.03191958237 | 3.06284249 | 0.002192454277 | 1.102703237 |
| acyp1 | 0.1033246439 | 0.03374496856 | 3.061927401 | 0.002199167836 | 1.108851332 |
| apex1 | 0.09615790442 | 0.03162531086 | 3.040536261 | 0.002361572531 | 1.100932892 |
| ppme1 | 0.09515114651 | 0.03133671698 | 3.03641082 | 0.002394129189 | 1.099825077 |
| ak1 | 0.09686583263 | 0.03204693719 | 3.022623724 | 0.002505936208 | 1.10171255 |
| atxn3 | 0.1005685399 | 0.03332886463 | 3.017460722 | 0.002549020721 | 1.105799431 |
| psme1 | 0.09529756932 | 0.03189041383 | 2.988282618 | 0.002805499967 | 1.099986129 |
| gdf2 | 0.1023432379 | 0.03426324781 | 2.986968382 | 0.002817588612 | 1.107763633 |
| cpped1 | 0.09522279715 | 0.03188553768 | 2.986394587 | 0.002822881411 | 1.099903883 |
| pdcd6 | 0.09271049797 | 0.0313884612 | 2.95364903 | 0.003140409359 | 1.097144064 |
| dner | 0.09780942314 | 0.03313704727 | 2.951663808 | 0.003160668496 | 1.102752606 |
| nmi | 0.09900955406 | 0.03354371653 | 2.951657249 | 0.003160735628 | 1.104076848 |
| pten | 0.09252602577 | 0.03148530421 | 2.938705154 | 0.0032958645 | 1.09694169 |
| hyal1 | 0.09317723842 | 0.03186060525 | 2.924528197 | 0.003449788245 | 1.097656265 |
| ccl26 | 0.09051761737 | 0.03123768511 | 2.897705674 | 0.003759031751 | 1.094740794 |
| dcxr | 0.1000134567 | 0.03453686385 | 2.895846513 | 0.00378137299 | 1.10518579 |
| atp6v1d | 0.09052523892 | 0.03127799775 | 2.894214638 | 0.003801082348 | 1.094749138 |
| rilp | 0.09023717223 | 0.03125837519 | 2.886815828 | 0.003891619542 | 1.094433822 |
| pde4d | -0.09757597787 | 0.03386768591 | -2.88109374 | 0.003962977579 | 0.9070334245 |
| rab44 | 0.09650971066 | 0.03351421572 | 2.8796649 | 0.003980980518 | 1.101320276 |
| pcdh1 | -0.09292114241 | 0.03239173842 | -2.868667967 | 0.004122042664 | 0.9112653577 |
| rnf31 | 0.0969501452 | 0.03380293564 | 2.868098387 | 0.004129470968 | 1.101805442 |
| bola1 | 0.09774117436 | 0.03408899831 | 2.867235155 | 0.004140752151 | 1.102677347 |
| glo1 | 0.09150802432 | 0.03211182132 | 2.849667835 | 0.004376490863 | 1.09582557 |
| nsfl1c | 0.08984130282 | 0.03155002836 | 2.847582316 | 0.00440526999 | 1.094000655 |
| st13 | 0.09567171414 | 0.0336260204 | 2.845169098 | 0.004438785204 | 1.10039776 |
| fabp5 | 0.09134569591 | 0.03214860265 | 2.841358205 | 0.004492182366 | 1.095647701 |
| cd2ap | 0.08898364754 | 0.03151923456 | 2.823153823 | 0.004755374985 | 1.093062782 |
| septin8 | 0.1027195998 | 0.03642536567 | 2.820001885 | 0.004802336743 | 1.108180631 |
| glod4 | 0.08914838061 | 0.031802393 | 2.80319725 | 0.005059871271 | 1.09324286 |
| nap1l4 | 0.09450830642 | 0.03374328735 | 2.800803177 | 0.005097559926 | 1.099118293 |
| bst2 | 0.09026481074 | 0.03245341376 | 2.781365664 | 0.005413072819 | 1.094464071 |
| ccdc80 | 0.0950500808 | 0.0343934979 | 2.763606106 | 0.005716650245 | 1.099713928 |
| carhsp1 | 0.08829597999 | 0.03196451685 | 2.76231236 | 0.005739354087 | 1.092311376 |
| proc | 0.08852595519 | 0.03230015217 | 2.740728734 | 0.006130309596 | 1.09256261 |
| mphosph8 | 0.08641860616 | 0.03170173693 | 2.725989631 | 0.006410901235 | 1.090262623 |
| myl3 | -0.1014576749 | 0.03736190456 | -2.715538088 | 0.00661681649 | 0.9035194201 |
| traf3 | 0.09263467965 | 0.03435038814 | 2.696757873 | 0.007001816102 | 1.097060883 |
| polr2f | 0.09076751495 | 0.03371324804 | 2.692339665 | 0.007095264235 | 1.095014401 |
| stip1 | 0.08454734932 | 0.03145053101 | 2.688264605 | 0.007182445637 | 1.08822437 |
| sord | 0.08894204966 | 0.03317120227 | 2.681303166 | 0.007333604353 | 1.093017314 |
| ggct | 0.088987075 | 0.03322506451 | 2.678311579 | 0.007399435052 | 1.093066528 |
| cit | 0.08921819227 | 0.03342569854 | 2.669149671 | 0.007604355564 | 1.093319184 |
| bap18 | 0.09123298019 | 0.03444024497 | 2.649022394 | 0.008072498233 | 1.095524211 |
| igsf8 | 0.08540521026 | 0.03233346753 | 2.641387293 | 0.008256727385 | 1.089158315 |
| ccl14 | 0.08518837323 | 0.03226741965 | 2.640073924 | 0.008288794485 | 1.088922171 |
| ptp4a3 | 0.09216890941 | 0.03497022474 | 2.635639608 | 0.008397887203 | 1.096550024 |
| cenpf | 0.08854056268 | 0.03371195575 | 2.626384637 | 0.00862972394 | 1.09257857 |
| megf9 | 0.08512672293 | 0.03241368161 | 2.626258996 | 0.008632910233 | 1.08855041 |
| mxra8 | -0.09782650467 | 0.0372836915 | -2.623841705 | 0.008694418294 | 0.9068062167 |
| srp14 | 0.08480515338 | 0.03233120038 | 2.62301283 | 0.008715599053 | 1.088504955 |
| nfkb2 | 0.08904113002 | 0.03400313008 | 2.618615693 | 0.00882873482 | 1.093125616 |
| fgfr4 | -0.08676542886 | 0.03317294988 | -2.615547582 | 0.008908450794 | 0.9168921468 |
| nmnat1 | 0.08330560295 | 0.03186251951 | 2.614532819 | 0.008934957607 | 1.08687391 |
| prtn3 | 0.08289918939 | 0.03171254142 | 2.614082179 | 0.008946751421 | 1.086432279 |
| acvrl1 | 0.08863598321 | 0.03396201425 | 2.609856487 | 0.009058021386 | 1.092682829 |
| cxcl8 | 0.08146637602 | 0.03150256997 | 2.586023175 | 0.009709040232 | 1.084876739 |
| ggt1 | 0.09096198491 | 0.03528330003 | 2.578046408 | 0.009936065256 | 1.095227369 |
| nacc1 | 0.08661617577 | 0.03366094523 | 2.573194994 | 0.01007644183 | 1.090478047 |
| mdh1 | 0.08651168506 | 0.03374776866 | 2.563478668 | 0.01036290687 | 1.090364109 |
| c7 | 0.08753912807 | 0.03432793679 | 2.550084166 | 0.01076969157 | 1.091484971 |
| s100a4 | 0.07954428391 | 0.03130960461 | 2.540571333 | 0.01106715154 | 1.082793509 |
| tsnax | 0.08674014196 | 0.03420680816 | 2.535756671 | 0.01122046687 | 1.090613238 |
| lto1 | 0.08035438798 | 0.03176793092 | 2.529418368 | 0.0114251748 | 1.08367104 |
| park7 | 0.07978020431 | 0.03157426805 | 2.526747546 | 0.01151242242 | 1.083048992 |
| hdgfl2 | 0.08576871632 | 0.0339552912 | 2.525930814 | 0.01153922035 | 1.089554303 |
| parp1 | 0.08202991554 | 0.03248018154 | 2.525537471 | 0.01155214612 | 1.085488282 |
| lilra5 | 0.08235134923 | 0.03269039014 | 2.519130206 | 0.01176451435 | 1.085837251 |
| ang | 0.08379510344 | 0.0333779368 | 2.510493802 | 0.01205624386 | 1.087406065 |
| csf2rb | 0.0849119184 | 0.03391228622 | 2.503868888 | 0.01228435474 | 1.088621175 |

| Protein | beta | se | z | p_val | HR |
|---|---|---|---|---|---|
| heg1 | 0.08866246308 | 0.03548216589 | 2.498789486 | 0.01246183141 | 1.092711764 |
| padi4 | 0.07901469826 | 0.03166126616 | 2.495626607 | 0.01257348764 | 1.082220229 |
| fen1 | 0.07958018802 | 0.03202112941 | 2.485239887 | 0.01294641609 | 1.082832386 |
| slirp | 0.08300692278 | 0.03340862195 | 2.48459583 | 0.01296985945 | 1.08654933 |
| hspb6 | -0.09561718188 | 0.03872667521 | -2.469026359 | 0.01354812385 | 0.9088118591 |
| ren | 0.0793374024 | 0.03222438731 | 2.462029817 | 0.01381531808 | 1.082569522 |
| lgmn | 0.07842911484 | 0.03196257682 | 2.453779471 | 0.01413636452 | 1.081586684 |
| ranbp1 | 0.08165242759 | 0.03329643731 | 2.452287217 | 0.01419513067 | 1.085078601 |
| il1r2 | 0.07809911965 | 0.03186782437 | 2.45072016 | 0.01425707452 | 1.081229824 |
| add1 | 0.08113910215 | 0.03341651392 | 2.428113906 | 0.01517757563 | 1.084521745 |
| inpp5d | 0.08371878102 | 0.03451425357 | 2.425629193 | 0.01528187546 | 1.087323075 |
| ccl2 | 0.07611420544 | 0.03141553887 | 2.422820304 | 0.01540054251 | 1.079085805 |
| cxcl9 | 0.08317676794 | 0.03450537847 | 2.410545011 | 0.01592870569 | 1.086733891 |
| pecam1 | 0.07603416244 | 0.03155785421 | 2.409357808 | 0.01598062195 | 1.078999435 |
| foxo3 | 0.0759276473 | 0.03152571719 | 2.40843521 | 0.01602106969 | 1.078884511 |
| azu1 | 0.07728738164 | 0.0321716347 | 2.402345493 | 0.01629031463 | 1.080352505 |
| hsdl2 | 0.08091851713 | 0.03369896719 | 2.401216532 | 0.01634066401 | 1.084282543 |
| rnf41 | 0.07732135485 | 0.03220891878 | 2.400619387 | 0.01636735071 | 1.080389209 |
| taldo1 | 0.08287370373 | 0.03459071579 | 2.395836624 | 0.01658247975 | 1.086404591 |
| ptgr1 | 0.08207380983 | 0.03435576615 | 2.388938424 | 0.01689713413 | 1.08553593 |
| erbb4 | 0.07789586842 | 0.03265052226 | 2.385746476 | 0.01704449657 | 1.081010086 |
| plpbp | 0.07511552724 | 0.0315221409 | 2.382944974 | 0.01717476132 | 1.078008683 |
| rbpms | 0.08095898369 | 0.03400312888 | 2.380927472 | 0.01726911164 | 1.084326421 |
| cxcl14 | -0.08646013788 | 0.03633609973 | -2.379455652 | 0.01733822916 | 0.9171721085 |
| slc28a1 | 0.0802917194 | 0.03385146851 | 2.371882903 | 0.01769769852 | 1.08360313 |
| stambp | 0.07470585965 | 0.03149729273 | 2.371818438 | 0.0177007864 | 1.077567148 |
| prcp | 0.080533616 | 0.033958743 | 2.371513457 | 0.01771540159 | 1.083865281 |
| apol1 | 0.0803240743 | 0.0339372065 | 2.366844021 | 0.01794049204 | 1.08363819 |
| mb | -0.08874598304 | 0.03750140896 | -2.366470634 | 0.0179585989 | 0.9150779894 |
| becn1 | 0.08035216011 | 0.03406571519 | 2.358739855 | 0.0183371054 | 1.083668625 |
| alpp | -0.079065543 | 0.03368867485 | -2.346947256 | 0.01892793397 | 0.9239793621 |
| tlr1 | 0.0799823713 | 0.03410791576 | 2.344979736 | 0.01902811372 | 1.083267971 |
| aspscr1 | 0.07862520988 | 0.03353396738 | 2.344643835 | 0.01904526298 | 1.081798799 |
| dnajc9 | 0.07810735093 | 0.03347445727 | 2.333341816 | 0.01963021238 | 1.081238724 |
| atp5po | 0.07314157876 | 0.03144634713 | 2.325916535 | 0.02002300375 | 1.075882848 |
| nedd4l | 0.0790380761 | 0.0340213018 | 2.323193762 | 0.02016874576 | 1.082245529 |
| sumf1 | -0.07911711319 | 0.03406605367 | -2.322461943 | 0.02020807536 | 0.9239317135 |
| amigo2 | 0.07247115906 | 0.031209447 | 2.322090458 | 0.02022806539 | 1.075161797 |
| acp5 | 0.07567212681 | 0.03275012771 | 2.310590282 | 0.02085549654 | 1.078608869 |
| blmh | 0.07302001358 | 0.03162143049 | 2.309193874 | 0.02093282534 | 1.075752066 |
| nagk | 0.08029921109 | 0.03484848557 | 2.304238183 | 0.02120927688 | 1.083611248 |
| nudt5 | 0.07285325549 | 0.03164111233 | 2.30248718 | 0.02130771359 | 1.075572691 |
| anpep | 0.07212058987 | 0.03136895982 | 2.299106833 | 0.02149887358 | 1.074784944 |
| il1r1 | 0.07229221758 | 0.03152909953 | 2.29287289 | 0.02185532399 | 1.074969423 |
| nit1 | 0.07805278469 | 0.03404676344 | 2.292517021 | 0.02187582651 | 1.081179727 |
| mpo | 0.07252593633 | 0.03173637276 | 2.285262304 | 0.02229745188 | 1.075220693 |
| tpr | 0.07949053948 | 0.03481633995 | 2.283138882 | 0.02242218886 | 1.082735316 |
| nrp1 | 0.07165230332 | 0.03141160343 | 2.281077548 | 0.02254385846 | 1.074281755 |
| ca3 | 0.07442289585 | 0.03264961719 | 2.279441606 | 0.02264082744 | 1.077262279 |
| mybpc1 | -0.08444902796 | 0.03706538753 | -2.278379739 | 0.0227039625 | 0.9190184984 |
| impact | 0.07584858178 | 0.03336673116 | 2.273179875 | 0.02301534253 | 1.078799212 |
| txnrd1 | 0.07189637249 | 0.03179585251 | 2.26118713 | 0.02374767171 | 1.074543986 |
| lyar | 0.07148338203 | 0.03161371661 | 2.261150845 | 0.02374991778 | 1.074100301 |
| cxcl11 | 0.07134926255 | 0.03167264776 | 2.252709122 | 0.02427749539 | 1.073956253 |
| sugt1 | 0.07099499361 | 0.0317252849 | 2.237804762 | 0.02523378976 | 1.073575851 |
| btd | 0.07642989314 | 0.03418853973 | 2.235541317 | 0.02538183229 | 1.079426512 |
| tpsg1 | -0.07487671006 | 0.03354956595 | -2.231823511 | 0.02562662981 | 0.9278578748 |
| ccl28 | 0.07213250257 | 0.03234124904 | 2.230356115 | 0.02572381055 | 1.074797748 |
| ccl19 | 0.07021269513 | 0.03152193538 | 2.227423357 | 0.02591899282 | 1.072736323 |
| ceacam1 | 0.06841577702 | 0.03095684281 | 2.210037291 | 0.02710257431 | 1.070810434 |
| gapdh | 0.07358229363 | 0.03330776387 | 2.209163423 | 0.02716327601 | 1.07635711 |
| mmp12 | 0.07527089807 | 0.03416680086 | 2.203042023 | 0.02759178694 | 1.078176187 |
| hgs | 0.06931653722 | 0.03153473006 | 2.198101493 | 0.02794187416 | 1.071775412 |
| myl4 | 0.07611286804 | 0.03464494821 | 2.196939871 | 0.02802474083 | 1.079084361 |
| fus | 0.07202265294 | 0.03281450682 | 2.194841852 | 0.02817494412 | 1.074679688 |
| mif | 0.06853986506 | 0.03124251988 | 2.193800798 | 0.02824973341 | 1.070943317 |
| susd2 | 0.0719296305 | 0.03284088303 | 2.190246543 | 0.02850636146 | 1.074579724 |
| cd164l2 | -0.07359823453 | 0.03369681814 | -2.184130093 | 0.02895269 | 0.929044877 |
| pts | 0.07353070414 | 0.03369539158 | 2.182218419 | 0.02909341656 | 1.076301583 |
| ada | 0.06928628149 | 0.03178339059 | 2.179952491 | 0.0292609834 | 1.071742985 |

| Protein | beta | se | z | p_val | HR |
|---|---|---|---|---|---|
| trim24 | 0.07342349825 | 0.03369392809 | 2.179131446 | 0.0293219048 | 1.076186203 |
| arl2bp | 0.07389289636 | 0.03394581565 | 2.176789538 | 0.02949627402 | 1.076691482 |
| cstb | 0.07310451873 | 0.03358407596 | 2.176761356 | 0.02949837773 | 1.075842977 |
| crlf1 | -0.07341246457 | 0.03380174562 | -2.171854241 | 0.02986665892 | 0.9292174817 |
| chit1 | -0.0680269265 | 0.03135785552 | -2.16937432 | 0.03005427697 | 0.9342353175 |
| ptgds | 0.07219853653 | 0.03333867601 | 2.165608991 | 0.03034107873 | 1.074868724 |
| clc | 0.06933138032 | 0.03209205186 | 2.160391009 | 0.03074241287 | 1.071791321 |
| gnpda1 | 0.07335287066 | 0.03397164979 | 2.159237809 | 0.03083172218 | 1.076110198 |
| cc2d1a | 0.06884470308 | 0.03191590215 | 2.157065865 | 0.03100053283 | 1.071269831 |
| ica1 | 0.06842272458 | 0.03178724217 | 2.152521575 | 0.03135629787 | 1.070817874 |
| gp1ba | 0.06645399396 | 0.03088087013 | 2.151946939 | 0.03140153368 | 1.068711796 |
| traf2 | 0.06811682106 | 0.03167565909 | 2.150446842 | 0.03151988651 | 1.070490357 |
| cd99l2 | 0.0704943791 | 0.0327893421 | 2.149917461 | 0.03156174424 | 1.073038538 |
| rarres2 | 0.074484269 | 0.0347475227 | 2.143585016 | 0.03206615246 | 1.077328396 |
| serpina12 | 0.0713230951 | 0.03327566936 | 2.143400763 | 0.03208093187 | 1.073928151 |
| csnk2a1 | 0.0707107398 | 0.0331747667 | 2.131461554 | 0.03305113649 | 1.073270726 |
| cdh5 | 0.06591670174 | 0.03098409976 | 2.127436403 | 0.03338384023 | 1.068137739 |
| pamr1 | 0.07227151691 | 0.03399852965 | 2.125724779 | 0.03352618248 | 1.074947171 |
| il19 | 0.06885265538 | 0.03243432434 | 2.122833041 | 0.03376784504 | 1.07127835 |
| afap1 | -0.07304521937 | 0.03441680779 | -2.122370553 | 0.03380663303 | 0.929558795 |
| fgf5 | 0.07232641786 | 0.03410131914 | 2.120927274 | 0.03392792301 | 1.075006188 |
| chchd6 | 0.07362680311 | 0.03476506691 | 2.117838671 | 0.03418873299 | 1.07640502 |
| ngfr | -0.07058833258 | 0.03333639451 | -2.11745552 | 0.0342212065 | 0.9318454236 |
| sbsn | 0.06938588207 | 0.03277616418 | 2.116961634 | 0.03426310404 | 1.071849737 |
| tmprss5 | 0.06858336829 | 0.03243860253 | 2.11425163 | 0.03449378122 | 1.070989908 |
| hbz | 0.06925406715 | 0.03290107544 | 2.104918038 | 0.03529844328 | 1.07170846 |
| dbi | 0.06730618935 | 0.03198832694 | 2.104085952 | 0.03537095005 | 1.069622935 |
| fcgr3b | 0.0671466612 | 0.03199083173 | 2.098934525 | 0.03582267289 | 1.069452314 |
| hnrnpk | 0.06680017695 | 0.03184726942 | 2.097516621 | 0.03594786737 | 1.06908183 |
| wwp2 | 0.06591311945 | 0.03149098343 | 2.093079106 | 0.0363420935 | 1.068133913 |
| nbn | 0.06737819782 | 0.0322216846 | 2.091082408 | 0.03652067744 | 1.06969996 |
| rbp2 | 0.06587340028 | 0.03151759458 | 2.090051641 | 0.03661316107 | 1.068091489 |
| il18r1 | 0.0701932715 | 0.03359952436 | 2.089115035 | 0.03669736929 | 1.072715487 |
| tfpi | 0.06890675452 | 0.03301467781 | 2.087155141 | 0.03687411299 | 1.071336307 |
| ccl13 | 0.06516056347 | 0.03133996071 | 2.079152686 | 0.03760331887 | 1.067330385 |
| atrn | 0.0716453261 | 0.03456332011 | 2.072871642 | 0.03818422682 | 1.074274259 |
| cnp | 0.06989362252 | 0.03372867332 | 2.072231595 | 0.03824384821 | 1.072394097 |
| apbb1ip | 0.06942085121 | 0.03350893759 | 2.071711496 | 0.03829235464 | 1.071887219 |
| mad1l1 | 0.0702146917 | 0.0338972256 | 2.071399368 | 0.03832148987 | 1.072738465 |
| il6st | 0.06604388752 | 0.03192417942 | 2.068773222 | 0.03856737239 | 1.0682736 |
| dag1 | 0.06638270452 | 0.03215285069 | 2.064597791 | 0.03896107242 | 1.068635611 |
| cnst | 0.06405899927 | 0.0310437378 | 2.063507934 | 0.03906439473 | 1.066155299 |
| lap3 | 0.06574260293 | 0.0319065536 | 2.060473336 | 0.03935331236 | 1.067951794 |
| dtymk | 0.06985367894 | 0.03391003971 | 2.059970426 | 0.0394013682 | 1.072351262 |
| pdzd2 | 0.07005209912 | 0.03417151221 | 2.050014605 | 0.04036300559 | 1.072564059 |
| ptk7 | 0.06443057065 | 0.03145724577 | 2.04819491 | 0.04054090504 | 1.066551526 |
| rac3 | 0.06836752923 | 0.03342066247 | 2.045666488 | 0.04078919501 | 1.070758771 |
| adamts1 | 0.07031481287 | 0.03440392794 | 2.043801888 | 0.04097312261 | 1.072845874 |
| vta1 | 0.06304177276 | 0.03084859007 | 2.043586842 | 0.04099438027 | 1.065071329 |
| dxo | 0.06807080068 | 0.03332862898 | 2.042412267 | 0.04111065393 | 1.070441094 |
| snca | 0.06947473491 | 0.03406238763 | 2.039631974 | 0.04138699498 | 1.071944978 |
| rbm25 | 0.06808595965 | 0.03344161265 | 2.035965202 | 0.04175384962 | 1.070457321 |
| lamp2 | 0.06924225333 | 0.0340158645 | 2.035587052 | 0.0417918391 | 1.0716958 |
| oxct1 | 0.06884823137 | 0.03387257396 | 2.032565681 | 0.04209642168 | 1.071273611 |
| chac2 | 0.06446014935 | 0.03172603995 | 2.031774197 | 0.04217652055 | 1.066583073 |
| got1 | 0.06949851431 | 0.03422032306 | 2.030913448 | 0.04226377533 | 1.071970469 |
| notch1 | 0.06401041308 | 0.0316349463 | 2.023408305 | 0.0430310686 | 1.0661035 |
| stx7 | 0.0699179286 | 0.03462216132 | 2.019455919 | 0.04343985456 | 1.072420163 |
| lbr | 0.0638172286 | 0.03160235806 | 2.01938186 | 0.0434475455 | 1.065897565 |
| ubxn1 | 0.06825988627 | 0.03382743161 | 2.017885574 | 0.04360317976 | 1.070643518 |
| bag6 | 0.06539755436 | 0.03244175429 | 2.015845191 | 0.04381616638 | 1.067583362 |
| setmar | 0.06852447675 | 0.03401764588 | 2.01438033 | 0.04396961785 | 1.070926837 |
| mycbp2 | 0.06685022829 | 0.0332183344 | 2.012449736 | 0.04417255017 | 1.06913534 |
| atp6v1f | 0.06353458571 | 0.03160067141 | 2.010545437 | 0.04437349231 | 1.06559634 |
| ciapin1 | 0.06324635407 | 0.03146512084 | 2.010046438 | 0.04442627415 | 1.065289245 |
| dgka | 0.06819798383 | 0.03397818882 | 2.007110626 | 0.04473788504 | 1.070577244 |
| rrm2b | 0.06416471563 | 0.03198308547 | 2.006207803 | 0.04483408173 | 1.066268015 |
| tagln3 | -0.06746472292 | 0.03364901285 | -2.004954 | 0.04496796492 | 0.9347606957 |
| ceacam20 | -0.06825733987 | 0.03407331102 | -2.0032494 | 0.04515052564 | 0.934020082 |
| casp10 | 0.06402900513 | 0.03198545536 | 2.001816276 | 0.04530449482 | 1.066123321 |

| Protein | beta | se | z | p_val | HR |
| --- | --- | --- | --- | --- | --- |
| bcl2l15 | 0.06859033813 | 0.03436829363 | 1.99574465 | 0.04596172433 | 1.070997372 |
| krt18 | 0.07058243581 | 0.03540142986 | 1.993773587 | 0.04617680329 | 1.07313303 |
| ube2z | 0.06699746526 | 0.03365324848 | 1.990817181 | 0.04650098985 | 1.069292768 |
| fgr | 0.06329953238 | 0.03181805442 | 1.98942184 | 0.04665466096 | 1.065345897 |
| ptprm | 0.06336986578 | 0.03185393371 | 1.989389014 | 0.04665828129 | 1.065420829 |
| kirrel2 | 0.06449932713 | 0.03242803153 | 1.988999149 | 0.04670129685 | 1.066624861 |
| ret | 0.06575007821 | 0.03310113735 | 1.986338944 | 0.04699570113 | 1.067959777 |
| ccdc50 | 0.06596459894 | 0.03321664034 | 1.985890153 | 0.0470455224 | 1.068188901 |
| ppy | -0.06334947185 | 0.03194623278 | -1.983002888 | 0.04736710697 | 0.9386153967 |
| sf3b4 | 0.06239237304 | 0.03146644815 | 1.982822235 | 0.04738728949 | 1.064379897 |
| gfra1 | 0.06666962597 | 0.03375364035 | 1.975183277 | 0.04824735297 | 1.068942269 |
| bmper | -0.066264475 | 0.03354996431 | -1.975098226 | 0.04825700209 | 0.9358833138 |
| snx9 | 0.06203453361 | 0.03142844685 | 1.973833893 | 0.04840063357 | 1.063999088 |
| gpkow | 0.06361788297 | 0.03224885579 | 1.972717525 | 0.0485277542 | 1.065685105 |
| cmip | 0.06529811571 | 0.03310242737 | 1.97260808 | 0.04854023167 | 1.067477209 |
| kitlg | 0.06211709633 | 0.03149641544 | 1.972195739 | 0.04858726589 | 1.064086938 |
| snx15 | 0.06679544627 | 0.03387918032 | 1.971577991 | 0.04865780174 | 1.069076772 |
| tspyl1 | 0.0672133097 | 0.03416834317 | 1.967122297 | 0.0491691133 | 1.069523594 |
| dapk2 | 0.06628370542 | 0.03370786684 | 1.966416496 | 0.04925051943 | 1.068529822 |
| gh1 | 0.06871906321 | 0.03496006107 | 1.965644827 | 0.04933965213 | 1.071135246 |
| insr | 0.06637708288 | 0.03376922803 | 1.965608537 | 0.04934384727 | 1.068629603 |
| stx3 | 0.06510534117 | 0.03317342651 | 1.96257511 | 0.04969556522 | 1.067271446 |
| pmm2 | 0.06694597726 | 0.03417367106 | 1.95899285 | 0.05011362412 | 1.069237713 |
| klk12 | 0.06155154026 | 0.03151920518 | 1.952826536 | 0.0508401531 | 1.063485307 |
| tp53i3 | 0.06753883668 | 0.0346129067 | 1.951261628 | 0.05102593116 | 1.069871809 |
| entr1 | 0.06864739717 | 0.0351813899 | 1.95124176 | 0.05102829343 | 1.071058484 |
| pear1 | 0.06037534676 | 0.03097021471 | 1.949464908 | 0.05123992968 | 1.062235178 |
| plekho1 | 0.06471484908 | 0.03321843021 | 1.948160966 | 0.0513957058 | 1.066854766 |
| kif22 | 0.06515929557 | 0.03347580353 | 1.946459493 | 0.05159956916 | 1.067329032 |
| nfe2 | 0.06512317859 | 0.0334874065 | 1.944706545 | 0.0518103074 | 1.067290484 |
| edf1 | 0.06528006629 | 0.03356881947 | 1.944663748 | 0.05181546137 | 1.067457942 |
| cntn4 | 0.06192176987 | 0.03188405569 | 1.942092012 | 0.05212596231 | 1.063879114 |
| usp25 | 0.0649537059 | 0.03348762597 | 1.939633044 | 0.05242430205 | 1.067109622 |
| rpe | 0.06471390015 | 0.03337017794 | 1.939273451 | 0.05246804982 | 1.066853754 |
| hip1r | 0.0684383166 | 0.03530629007 | 1.938417105 | 0.05257235521 | 1.07083457 |
| sptlc1 | 0.06460729778 | 0.03333941672 | 1.93786527 | 0.05263966208 | 1.066740031 |
| ifng | 0.06080561107 | 0.03146613473 | 1.932414375 | 0.05330838123 | 1.062692318 |
| ptrhd1 | 0.06491733726 | 0.03360828581 | 1.931587277 | 0.0534104675 | 1.067070814 |
| stx16 | 0.06058265459 | 0.03137347064 | 1.931015388 | 0.05348114944 | 1.062455411 |
| flt1 | 0.06153643793 | 0.03189080366 | 1.929598219 | 0.05365663968 | 1.063469246 |
| ass1 | 0.06978655625 | 0.03616637339 | 1.929597848 | 0.05365668572 | 1.072279286 |
| fcrl2 | -0.05924445921 | 0.03071588514 | -1.928788929 | 0.05375707081 | 0.942476344 |
| zcchc8 | 0.06510412718 | 0.03380698946 | 1.925759383 | 0.05413442519 | 1.06727015 |
| tgfb2 | -0.06375844633 | 0.03320708643 | -1.920025307 | 0.05485470282 | 0.9382316055 |
| lypd8 | 0.06040773518 | 0.03146563262 | 1.919800435 | 0.05488311197 | 1.062269583 |
| atf2 | 0.06129108654 | 0.03194938956 | 1.918380519 | 0.05506277934 | 1.063208355 |
| eif2ak3 | 0.06443516036 | 0.0336085353 | 1.917226079 | 0.0552092163 | 1.066556421 |
| npy | -0.06061672055 | 0.03162152039 | -1.916945162 | 0.05524489869 | 0.9411839071 |
| ccl27 | 0.06304829566 | 0.03290291941 | 1.916191535 | 0.05534072042 | 1.065078277 |
| wfdc12 | 0.06540381919 | 0.03421090996 | 1.911782506 | 0.0559040965 | 1.067590051 |
| igfbp3 | 0.06126428697 | 0.03205718687 | 1.911093672 | 0.0559925442 | 1.063179862 |
| dars1 | 0.05959451333 | 0.03118778858 | 1.910828438 | 0.0560266319 | 1.061406073 |
| fkbp4 | 0.06031469547 | 0.03161065337 | 1.90804963 | 0.05638480198 | 1.062170754 |
| gask1a | -0.06682546379 | 0.03507570629 | -1.905177995 | 0.05675693755 | 0.935358441 |
| rida | 0.06991004963 | 0.03673231749 | 1.903230027 | 0.05701053664 | 1.072411713 |
| hebp1 | 0.05908822652 | 0.03108805522 | 1.900672979 | 0.05734485997 | 1.060868833 |
| tcl1b | 0.05926196972 | 0.03119964612 | 1.899443651 | 0.05750616883 | 1.061053168 |
| timp4 | 0.06298674182 | 0.03317576276 | 1.898577051 | 0.05762010817 | 1.065012719 |
| akt1s1 | 0.06077553619 | 0.03201325925 | 1.898448881 | 0.05763697572 | 1.062660359 |
| mvk | 0.06241588403 | 0.03288046054 | 1.898266721 | 0.05766095547 | 1.064404922 |
| spp1 | 0.06105036677 | 0.03217572923 | 1.897404293 | 0.05777459946 | 1.06295245 |
| snrpb2 | 0.06599050169 | 0.03481868087 | 1.895261395 | 0.05805777991 | 1.068216571 |
| plxnb2 | 0.06162061306 | 0.03251822948 | 1.894955969 | 0.05809823524 | 1.063558768 |
| gadd45b | -0.06310100159 | 0.03330043857 | -1.894900016 | 0.05810564904 | 0.9388486437 |
| timp1 | 0.06325532014 | 0.03346826318 | 1.890009045 | 0.05875675038 | 1.065298797 |
| cep43 | 0.05976899596 | 0.03167420509 | 1.886992768 | 0.05916129832 | 1.061591286 |
| siglec7 | 0.06187191787 | 0.03282976403 | 1.884628772 | 0.05947997494 | 1.063826079 |
| asgr1 | 0.06558615456 | 0.03481293038 | 1.883959605 | 0.05957043975 | 1.067784728 |
| trim25 | 0.06248015903 | 0.03318050356 | 1.8830383 | 0.0596951777 | 1.064473339 |
| il18 | 0.0606052617 | 0.0321959639 | 1.882386932 | 0.05978349894 | 1.06247943 |

| Protein | beta | se | z | p_val | HR |
|---|---|---|---|---|---|
| eif4e | 0.06360619489 | 0.03379774644 | 1.881965562 | 0.05984069157 | 1.065672649 |
| prss27 | 0.05756341258 | 0.03075562805 | 1.871638338 | 0.06125665563 | 1.059252438 |
| ung | 0.06262682554 | 0.03346683912 | 1.871309845 | 0.06130214671 | 1.064629473 |
| adam8 | 0.05938767464 | 0.03175528492 | 1.870166645 | 0.06146068008 | 1.061186556 |
| bach1 | 0.05835216472 | 0.03122622415 | 1.868691022 | 0.06166581382 | 1.060088256 |
| itga5 | 0.06296009771 | 0.03373387446 | 1.866376119 | 0.0619887618 | 1.064984343 |
| echs1 | 0.06293531414 | 0.03376540472 | 1.863899297 | 0.06233584742 | 1.064957949 |
| lta4h | -0.05987501562 | 0.03214788478 | -1.862486942 | 0.06253448433 | 0.9418822468 |
| baiap2 | 0.06142569261 | 0.03303290096 | 1.859530675 | 0.06295195546 | 1.063351479 |
| dsg3 | 0.0613899938 | 0.03304263496 | 1.857902491 | 0.06318286262 | 1.063313519 |
| nt5c | 0.06214131277 | 0.03345552711 | 1.85743039 | 0.06324994612 | 1.064112707 |
| dcc | -0.06260151577 | 0.03370894922 | -1.857118576 | 0.06329428589 | 0.9393177024 |
| zbp1 | 0.06378017885 | 0.03439692205 | 1.854240875 | 0.06370470663 | 1.065858075 |
| trem2 | -0.06351792369 | 0.0342971081 | -1.851990655 | 0.06402716486 | 0.9384572985 |
| ggh | 0.06222440138 | 0.03360411271 | 1.851690057 | 0.06407034261 | 1.064201126 |
| mmp1 | -0.0587779511 | 0.03176397501 | -1.850459556 | 0.06424734202 | 0.9429161194 |
| igbp1 | 0.06200087201 | 0.03354635664 | 1.848214775 | 0.06457127878 | 1.063963273 |
| lep | 0.103128829 | 0.0558230112 | 1.847425044 | 0.06468556224 | 1.108634224 |
| snap25 | 0.06303806646 | 0.03417025395 | 1.844822885 | 0.06506330733 | 1.065067382 |
| plxnb3 | 0.05785357864 | 0.03136479045 | 1.844538982 | 0.06510463022 | 1.059559842 |
| yju2 | 0.06145371994 | 0.03334387912 | 1.843028512 | 0.06532484764 | 1.063381282 |
| lifr | 0.05862056656 | 0.03182079516 | 1.842209356 | 0.06544453243 | 1.060372823 |
| ccnd2 | 0.06185913122 | 0.03358371402 | 1.841938363 | 0.06548416627 | 1.063812476 |
| denr | 0.06269605064 | 0.0340409101 | 1.841785383 | 0.065506549 | 1.064703174 |
| ermap | 0.06657296566 | 0.03615471075 | 1.841335867 | 0.06557235481 | 1.06883895 |
| klk14 | 0.05812035804 | 0.0315869985 | 1.84000889 | 0.06576693216 | 1.059842549 |
| thtpa | 0.06111846649 | 0.03325785217 | 1.837715382 | 0.06610435609 | 1.06302484 |
| pappa | 0.06107092556 | 0.03329119686 | 1.83444668 | 0.06658771576 | 1.062974304 |
| scn2a | 0.06186522521 | 0.03374235185 | 1.833459194 | 0.06673431169 | 1.063818959 |
| apcs | 0.0713440495 | 0.03892207186 | 1.83299722 | 0.06680298456 | 1.073950654 |
| psma1 | 0.05831089072 | 0.03182058598 | 1.832489532 | 0.0668785199 | 1.060044502 |
| oga | 0.06142345092 | 0.03352680654 | 1.832069835 | 0.06694101685 | 1.063349095 |
| pikfyve | 0.06272569463 | 0.03423865771 | 1.832013835 | 0.06694935936 | 1.064734737 |
| gpihbp1 | -0.06072891871 | 0.03315690532 | -1.831561725 | 0.0670167436 | 0.9410783139 |
| fabp9 | 0.05734566634 | 0.03135217124 | 1.829081179 | 0.067387448 | 1.059021815 |
| actn2 | -0.06484387079 | 0.03545903393 | -1.828698179 | 0.06744483537 | 0.9372137784 |
| nrp2 | 0.05946775492 | 0.03251924643 | 1.828694126 | 0.06744544297 | 1.06127154 |
| lgals3bp | 0.06486044112 | 0.03552243639 | 1.82590069 | 0.0678652222 | 1.067010103 |
| sirt2 | 0.05726439772 | 0.03139292786 | 1.824117775 | 0.0681342688 | 1.058935754 |
| milr1 | -0.05848351449 | 0.03208866954 | -1.822559655 | 0.06837011082 | 0.9431937893 |
| clec5a | 0.05910060931 | 0.0325256334 | 1.817047145 | 0.06920989485 | 1.06088197 |
| casp7 | 0.06015358908 | 0.03311768646 | 1.816358433 | 0.06931540712 | 1.061999646 |
| hgf | 0.06507258767 | 0.03582910847 | 1.816193326 | 0.06934072155 | 1.06723649 |
| sdc4 | 0.05680013638 | 0.03130212563 | 1.814577612 | 0.06958884562 | 1.058444245 |
| ssc4d | 0.06691399304 | 0.03688975561 | 1.813890928 | 0.06969451967 | 1.069203515 |
| nudt16 | 0.06092299294 | 0.03362039622 | 1.812084323 | 0.06997316829 | 1.062817067 |
| dusp29 | -0.06293079169 | 0.03481456497 | -1.807599542 | 0.0706688496 | 0.9390084586 |
| thpo | 0.05708607166 | 0.03160442832 | 1.806268131 | 0.07087646734 | 1.058746935 |
| rangap1 | 0.05751439136 | 0.03185678808 | 1.805404588 | 0.0710113937 | 1.059200514 |
| cr1 | -0.06068260508 | 0.03368656223 | -1.801389072 | 0.07164157732 | 0.9411218997 |
| ifi30 | 0.06801690778 | 0.03779372451 | 1.799687876 | 0.07190993658 | 1.070383406 |
| maged1 | 0.05632385872 | 0.03131265319 | 1.798757147 | 0.07205710469 | 1.057940251 |
| itpa | 0.06185371303 | 0.03440202916 | 1.797966996 | 0.07218223803 | 1.063806712 |
| clec2l | -0.06019220765 | 0.03349326735 | -1.797143498 | 0.07231284174 | 0.9415835366 |
| klrf1 | 0.06272082537 | 0.03490483721 | 1.796909265 | 0.07235002554 | 1.064729552 |
| mri1 | 0.06007847952 | 0.03344877739 | 1.796133796 | 0.07247324061 | 1.061919882 |
| asah2 | 0.05824206664 | 0.0324402047 | 1.795366804 | 0.07259527782 | 1.059971548 |
| pi16 | -0.05955640512 | 0.03328131488 | -1.789484741 | 0.07353678281 | 0.9421823882 |
| acox1 | 0.05549621489 | 0.03101885015 | 1.789112576 | 0.07359668728 | 1.057065016 |
| il5 | 0.05656444125 | 0.03162389158 | 1.788661623 | 0.07366932699 | 1.058194804 |
| relb | 0.06068271672 | 0.0339432679 | 1.787768841 | 0.07381330992 | 1.062561728 |
| cetn2 | 0.05671293948 | 0.03177416278 | 1.784875966 | 0.07428143787 | 1.058351956 |
| s100g | 0.06011371149 | 0.03367964648 | 1.784867651 | 0.07428278692 | 1.061957296 |
| lcn2 | 0.05812145174 | 0.03258136483 | 1.783886342 | 0.0744421356 | 1.059843708 |
| klk4 | 0.06037976868 | 0.03385474469 | 1.783495023 | 0.07450575737 | 1.062239875 |
| cradd | 0.05592006791 | 0.03138563027 | 1.781709255 | 0.07479665689 | 1.057513151 |
| upk3bl1 | -0.0594677008 | 0.03339548327 | -1.780710892 | 0.07495969286 | 0.9422659675 |
| penk | -0.06017656497 | 0.03380529148 | -1.780093066 | 0.07506073111 | 0.9415982656 |
| pglyrp1 | 0.05580103617 | 0.03137039345 | 1.778780246 | 0.07527579709 | 1.057387281 |
| cct5 | 0.05611807026 | 0.03155102234 | 1.778645068 | 0.07529797041 | 1.057722562 |

| Protein | beta | se | z | p_val | HR |
| --- | --- | --- | --- | --- | --- |
| clec11a | 0.05503375973 | 0.03096265572 | 1.777423753 | 0.07549854557 | 1.056576284 |
| muc16 | -0.05560669868 | 0.03131188833 | -1.775897323 | 0.07574984221 | 0.9459110908 |
| chl1 | 0.05727291594 | 0.03225635116 | 1.775554701 | 0.07580634197 | 1.058944774 |
| mocs2 | 0.06072405859 | 0.03424239569 | 1.773358942 | 0.07616924785 | 1.062605657 |
| lrpap1 | 0.05699553338 | 0.03220380387 | 1.769838545 | 0.07675404079 | 1.058651082 |
| ddx58 | 0.05683298715 | 0.03212979906 | 1.768855978 | 0.07691791218 | 1.058479016 |
| smndc1 | 0.06054656655 | 0.03423038079 | 1.768796174 | 0.07692789557 | 1.06241707 |
| cxcl16 | 0.05719624776 | 0.03234388583 | 1.768378977 | 0.07699756865 | 1.05886359 |
| ppp3r1 | 0.05665964727 | 0.03207753012 | 1.766334473 | 0.0773397513 | 1.058295555 |
| cope | -0.05532150082 | 0.03132493522 | -1.76605316 | 0.07738693074 | 0.9461809011 |
| mmp9 | 0.05755244625 | 0.0326367456 | 1.76342479 | 0.07782887178 | 1.059240822 |
| hmgcl | 0.05959784698 | 0.03379764182 | 1.763372939 | 0.07783761083 | 1.061409612 |
| tmsb10 | 0.05717784789 | 0.03245264962 | 1.761885348 | 0.07808867058 | 1.058844107 |
| eda2r | 0.06931202286 | 0.03937386109 | 1.76035626 | 0.07834742063 | 1.071770574 |
| il1rl1 | 0.0597705434 | 0.03398540386 | 1.758712171 | 0.07862640905 | 1.061592929 |
| otud6b | 0.05882620023 | 0.03345251035 | 1.758498828 | 0.07866267087 | 1.060590894 |
| pcbd1 | 0.06591147971 | 0.03749006891 | 1.758105056 | 0.0787296356 | 1.068132162 |
| txndc15 | 0.05462849438 | 0.03108630694 | 1.757316959 | 0.0788637987 | 1.056148177 |
| lcn15 | 0.05941889586 | 0.03385539804 | 1.755078933 | 0.07924580732 | 1.061219688 |
| st6gal1 | 0.05701471969 | 0.03248724359 | 1.754987909 | 0.07926137598 | 1.058671394 |
| cst6 | 0.05662942532 | 0.03233829256 | 1.751156936 | 0.07991888097 | 1.058263572 |
| tbc1d5 | 0.05430085636 | 0.03102561055 | 1.75019461 | 0.08008473859 | 1.055802199 |
| il2 | 0.05492306953 | 0.03150637289 | 1.743236828 | 0.08129225495 | 1.056459338 |
| itih5 | -0.05748754154 | 0.03300849067 | -1.741598612 | 0.08157870438 | 0.9441336528 |
| ntf3 | -0.05499929375 | 0.03158116643 | -1.741521925 | 0.08159213357 | 0.9464858164 |
| otoa | 0.05846783971 | 0.03365208743 | 1.737420891 | 0.08231290649 | 1.060210888 |
| defb118 | 0.05860328174 | 0.03373186715 | 1.737326946 | 0.08232947794 | 1.060354495 |
| tpmt | 0.0548673108 | 0.0315894354 | 1.736887985 | 0.08240694458 | 1.056400432 |
| grhpr | 0.05839899739 | 0.03367942115 | 1.733966778 | 0.0829239779 | 1.060137904 |
| vasn | 0.05525349661 | 0.03188754204 | 1.732761231 | 0.08313811626 | 1.056808478 |
| irak1 | 0.05527595431 | 0.03192676401 | 1.731335951 | 0.08339186297 | 1.056832212 |
| dnajb1 | 0.05469551752 | 0.03161461887 | 1.730070438 | 0.08361769113 | 1.056218966 |
| c2orf69 | 0.05798587468 | 0.03352721718 | 1.729516481 | 0.08371669932 | 1.059700027 |
| tnfrsf14 | 0.05659412671 | 0.03274945543 | 1.728093673 | 0.08397143174 | 1.058226217 |
| wfdc1 | -0.05963303821 | 0.03462175291 | -1.722415337 | 0.08499430737 | 0.9421101886 |
| ccl20 | 0.05480579739 | 0.03183071419 | 1.721789749 | 0.08510761226 | 1.056335452 |
| serpind1 | 0.06119339421 | 0.03560837864 | 1.718511107 | 0.08570343027 | 1.063104493 |
| dync1h1 | 0.05762908075 | 0.03360778398 | 1.714753962 | 0.0863903454 | 1.059322 |
| podxl | 0.05345474129 | 0.03121207636 | 1.712630094 | 0.08678061318 | 1.054909247 |
| cntn2 | -0.05590493851 | 0.03269101582 | -1.71010099 | 0.08724719985 | 0.9456290245 |
| ikbkg | 0.05327816152 | 0.03119386182 | 1.707969402 | 0.08764202013 | 1.054722988 |
| nt5c1a | 0.06657893972 | 0.03901090965 | 1.706674885 | 0.08788249788 | 1.068845335 |
| hs6st1 | -0.05357947436 | 0.03140898693 | -1.705864455 | 0.08803331911 | 0.9478306098 |
| hk2 | 0.05423203497 | 0.03180318266 | 1.705239238 | 0.08814981467 | 1.05572954 |
| plscr3 | 0.05723963467 | 0.0336261786 | 1.702234302 | 0.08871145485 | 1.058909531 |
| maea | 0.05422921053 | 0.03187084878 | 1.701530163 | 0.08884347882 | 1.055726558 |
| arhgef5 | 0.0587301898 | 0.03452026396 | 1.70132505 | 0.08888196663 | 1.060489071 |
| abhd14b | 0.05420413686 | 0.03186869955 | 1.700858135 | 0.08896962967 | 1.055700087 |
| ccl18 | 0.05800375992 | 0.03413547956 | 1.699222061 | 0.08927735125 | 1.05971898 |
| klhl41 | -0.05887204252 | 0.03469538227 | -1.696826455 | 0.08972947553 | 0.9428274033 |
| pla2g4a | 0.05288087276 | 0.0311729066 | 1.696372861 | 0.08981528991 | 1.054304041 |
| pacs2 | 0.05805602278 | 0.03422362321 | 1.696372778 | 0.08981530564 | 1.059774366 |
| adgre2 | 0.05530550099 | 0.03262281992 | 1.695301054 | 0.09001832499 | 1.056863438 |
| mtpn | 0.05308345628 | 0.0313297379 | 1.694347283 | 0.09019931071 | 1.054517648 |
| tnfsf13b | 0.05456848394 | 0.03221775353 | 1.693739568 | 0.09031478215 | 1.056084799 |
| klk1 | 0.05316823172 | 0.03142329926 | 1.692000298 | 0.09064591678 | 1.054607049 |
| trim58 | 0.05565165862 | 0.03290337004 | 1.691366524 | 0.09076682168 | 1.057229343 |
| enah | 0.05540873971 | 0.03276772876 | 1.69095454 | 0.09084548543 | 1.056972553 |
| clint1 | -0.05687236571 | 0.03366001253 | -1.689612137 | 0.09110218242 | 0.9447146397 |
| sema4c | -0.05413035691 | 0.03204094089 | -1.689412214 | 0.09114046187 | 0.9473086102 |
| ceacam19 | 0.05690030514 | 0.03378263743 | 1.684306184 | 0.09212251093 | 1.058550273 |
| gmpr2 | 0.05631908032 | 0.03348177313 | 1.682081773 | 0.09255298419 | 1.057935196 |
| stx5 | 0.05678402215 | 0.03376140415 | 1.681921223 | 0.09258411673 | 1.058427189 |
| il18rap | -0.05185128904 | 0.03086113763 | -1.680148336 | 0.09292845835 | 0.9494700529 |
| mmp8 | 0.05336153637 | 0.03177479808 | 1.679366655 | 0.09308060772 | 1.054810929 |
| ccl7 | 0.05617823719 | 0.03350957498 | 1.676483131 | 0.09364359763 | 1.057786204 |
| cryzl1 | 0.056237827 | 0.03358986905 | 1.674249665 | 0.09408154241 | 1.057849239 |
| letm1 | 0.05723274217 | 0.03419700992 | 1.673618316 | 0.09420563641 | 1.058902233 |
| qdpr | 0.05333805095 | 0.03190714359 | 1.671664867 | 0.09459042573 | 1.054786156 |
| trim26 | -0.05607121582 | 0.03360145945 | -1.668713703 | 0.09517413213 | 0.9454718009 |

| Protein | beta | se | z | p_val | HR |
|---|---|---|---|---|---|
| sost | 0.05634945874 | 0.03376834042 | 1.668706784 | 0.09517550404 | 1.057967335 |
| gne | 0.0525361959 | 0.03152032808 | 1.666740136 | 0.09556608848 | 1.05394071 |
| vsig10 | 0.05582897726 | 0.03360218315 | 1.661468751 | 0.09661933997 | 1.057416826 |
| lonp1 | 0.0557920408 | 0.03359270799 | 1.660837846 | 0.09674601784 | 1.057377769 |
| lxn | 0.05184408598 | 0.03122479591 | 1.660349875 | 0.09684408768 | 1.053211519 |
| map2k1 | 0.0558627581 | 0.03364722913 | 1.660248393 | 0.09686449288 | 1.057452547 |
| agrn | 0.05511879887 | 0.03331501545 | 1.654473159 | 0.09803141248 | 1.056666138 |
| met | 0.05427603163 | 0.03282253807 | 1.653620799 | 0.09820458393 | 1.05577599 |
| adam15 | 0.05219148771 | 0.03157829098 | 1.652764798 | 0.09837874095 | 1.05357747 |
| apobr | 0.05582471515 | 0.03379051008 | 1.652082642 | 0.09851770505 | 1.057412319 |
| fabp1 | 0.05551368104 | 0.03362696114 | 1.650868207 | 0.09876548904 | 1.057083479 |
| mln | 0.05458050676 | 0.03313601906 | 1.64716548 | 0.099524037 | 1.056097496 |
| gba | -0.05530050914 | 0.03357424053 | -1.647111245 | 0.09953518214 | 0.9462007633 |
| cacna1h | -0.0552511157 | 0.03358260917 | -1.645229988 | 0.09992239146 | 0.9462475005 |
| rp2 | 0.05185867616 | 0.03154621165 | 1.643895525 | 0.1001977847 | 1.053226886 |
| crtac1 | -0.05217227285 | 0.03175946089 | -1.64273169 | 0.1004384591 | 0.9491653373 |
| tspan1 | -0.05625083873 | 0.03426493524 | -1.641644391 | 0.1006637224 | 0.9453019878 |
| pcdh7 | -0.05614189545 | 0.03426572215 | -1.638427324 | 0.1013325831 | 0.9454049777 |
| tnfrsf10c | 0.05140443407 | 0.0313826189 | 1.637990578 | 0.1014236593 | 1.052748575 |
| comt | 0.0525527144 | 0.03210820848 | 1.636737672 | 0.101685294 | 1.053958119 |
| kdr | 0.05290328075 | 0.03232350117 | 1.636681635 | 0.1016970082 | 1.054327666 |
| tax1bp1 | 0.05421449789 | 0.03322049044 | 1.631959588 | 0.1026879997 | 1.055711026 |
| bin2 | 0.05043024516 | 0.03091116422 | 1.631457321 | 0.1027938583 | 1.051723498 |
| pi3 | 0.05521282814 | 0.03386784533 | 1.630243306 | 0.1030500845 | 1.0567655 |
| tnfrsf12a | -0.05670932268 | 0.03487012608 | -1.626301051 | 0.1038856284 | 0.9448686813 |
| med18 | 0.05160676803 | 0.03176559593 | 1.62461199 | 0.1042452607 | 1.052961603 |
| tbl1x | 0.0516073544 | 0.0317885313 | 1.623458281 | 0.1044914746 | 1.05296222 |
| susd1 | 0.05094780379 | 0.03140980801 | 1.622034868 | 0.1047958825 | 1.052267967 |
| igf1r | 0.05233276415 | 0.0323002966 | 1.620194539 | 0.1051904942 | 1.053726327 |
| casq2 | -0.05450494143 | 0.03372825401 | -1.616002459 | 0.1060937815 | 0.9469538295 |
| cdon | 0.05231832988 | 0.03239045449 | 1.615239141 | 0.1062589173 | 1.053711117 |
| s100a11 | 0.0529349674 | 0.0327859561 | 1.614562261 | 0.1064055237 | 1.054361075 |
| bid | 0.05150826504 | 0.03192282945 | 1.613524425 | 0.1066306211 | 1.052857888 |
| fhit | 0.05123697952 | 0.03177667491 | 1.612408462 | 0.1068730847 | 1.052572302 |
| msra | 0.05052339034 | 0.03135423244 | 1.611373853 | 0.1070982626 | 1.051821466 |
| ggact | 0.0532210806 | 0.0330352862 | 1.61103737 | 0.1071715776 | 1.054662785 |
| cst3 | 0.05627629365 | 0.03498250626 | 1.608698166 | 0.1076823571 | 1.057889932 |
| usp8 | 0.05141745797 | 0.03196583023 | 1.60851314 | 0.1077228406 | 1.052762286 |
| tdo2 | 0.05412945242 | 0.03365216443 | 1.608498393 | 0.1077260678 | 1.055621246 |
| cd63 | 0.05056666492 | 0.03144915573 | 1.607886245 | 0.1078600949 | 1.051866984 |
| vnn2 | 0.05056324467 | 0.03152324367 | 1.603998789 | 0.1087143196 | 1.051863386 |
| hs3st3b1 | 0.05019604683 | 0.03138661369 | 1.599282016 | 0.1097579536 | 1.051477215 |
| slamf7 | -0.05019666314 | 0.03139281368 | -1.598985795 | 0.1098237589 | 0.9510423711 |
| sdc1 | 0.05147642622 | 0.03222108161 | 1.597600814 | 0.1101318451 | 1.052824367 |
| stat2 | 0.05440409057 | 0.0340633476 | 1.597144567 | 0.1102334857 | 1.0559112 |
| fcgr2a | 0.04944982345 | 0.03097388372 | 1.596500584 | 0.1103770754 | 1.050692871 |
| fgd3 | 0.05315572315 | 0.03330083603 | 1.596227888 | 0.1104379234 | 1.054593857 |
| sestd1 | 0.05026101539 | 0.03149940842 | 1.595617756 | 0.1105741608 | 1.05154553 |
| cfhr5 | 0.05365564959 | 0.03364791312 | 1.594620427 | 0.1107971419 | 1.055121208 |
| cemip2 | 0.05427983005 | 0.03404545924 | 1.594333907 | 0.1108612671 | 1.05578 |
| rwdd1 | 0.04960713617 | 0.03111732537 | 1.59419666 | 0.1108919944 | 1.050858171 |
| cxcl10 | 0.05361864982 | 0.03373472657 | 1.589420021 | 0.1119655975 | 1.05508217 |
| ogn | 0.05466027656 | 0.03440683112 | 1.588646056 | 0.112140324 | 1.056181744 |
| cd164 | 0.05017896554 | 0.0316564801 | 1.585108811 | 0.112941614 | 1.051459255 |
| sv2a | 0.05278634706 | 0.03332444263 | 1.584012902 | 0.1131907832 | 1.054204387 |
| hs1bp3 | 0.05432798919 | 0.03431021199 | 1.583434961 | 0.1133223597 | 1.055830846 |
| itpr1 | 0.05415512439 | 0.03422747604 | 1.582212032 | 0.1136011746 | 1.055648346 |
| arfip1 | 0.05263881555 | 0.03328454568 | 1.581479166 | 0.1137685188 | 1.05404887 |
| spint1 | 0.04947884165 | 0.03128842727 | 1.581378355 | 0.1137915535 | 1.05072336 |
| klkb1 | 0.05616283807 | 0.03551867608 | 1.581219918 | 0.1138277627 | 1.057769915 |
| jpt2 | 0.05263146575 | 0.033285894 | 1.581194297 | 0.1138336191 | 1.054041123 |
| gmfg | 0.0525864234 | 0.03327527474 | 1.58034528 | 0.1140278155 | 1.053993648 |
| scgn | -0.05085224967 | 0.03221467182 | -1.578543154 | 0.1144408823 | 0.9504190849 |
| cd46 | 0.05137926129 | 0.03258977231 | 1.576545574 | 0.1149001244 | 1.052722074 |
| vstm2b | -0.05263530549 | 0.03338849742 | -1.576450261 | 0.1149220729 | 0.9487259445 |
| gpha2 | -0.05505150952 | 0.03495936258 | -1.574728641 | 0.1153190939 | 0.9464363962 |
| top2b | 0.0542884887 | 0.03448819944 | 1.574117802 | 0.1154602177 | 1.055789141 |
| rictor | 0.05289045744 | 0.03360523041 | 1.573875757 | 0.1155161757 | 1.054314147 |
| mapk9 | 0.05029928205 | 0.03196813896 | 1.573419151 | 0.1156217955 | 1.05158577 |
| csf2ra | -0.04925175377 | 0.03135517927 | -1.570769325 | 0.1162362397 | 0.9519414447 |

| Protein | beta | se | z | p_val | HR |
|---|---|---|---|---|---|
| nme3 | 0.04906676993 | 0.03125118919 | 1.57007689 | 0.1163972243 | 1.050290476 |
| vav3 | 0.05329900628 | 0.03394954128 | 1.56994776 | 0.1164272652 | 1.054744973 |
| mavs | 0.04980086937 | 0.03180334735 | 1.565900244 | 0.1173719728 | 1.051061777 |
| smad2 | 0.05221600664 | 0.03336552768 | 1.564968705 | 0.1175902474 | 1.053603303 |
| enpp7 | 0.05182685526 | 0.03316612335 | 1.56264435 | 0.1181362706 | 1.053193372 |
| tigar | 0.04958133267 | 0.03173021643 | 1.562590434 | 0.1181489598 | 1.050831056 |
| calcoco2 | 0.05274774037 | 0.0337724646 | 1.561856412 | 0.1183218189 | 1.054163689 |
| prkcq | 0.0491817679 | 0.03151013912 | 1.560823572 | 0.1185653841 | 1.050411264 |
| bst1 | 0.04894211976 | 0.03136160354 | 1.560574532 | 0.1186241718 | 1.050159565 |
| foxj3 | 0.05380279291 | 0.0345372315 | 1.557820085 | 0.1192759035 | 1.055276474 |
| nit2 | 0.05279440705 | 0.03400124444 | 1.552719847 | 0.1204900788 | 1.054212884 |
| prkar2a | 0.05180236234 | 0.03338325117 | 1.551747075 | 0.1207227541 | 1.053167576 |
| paxx | 0.05354723623 | 0.03460203563 | 1.547516938 | 0.1217386433 | 1.055006825 |
| fmnl1 | 0.05014442074 | 0.03248898965 | 1.543428136 | 0.1227269315 | 1.051422933 |
| izumo1 | 0.05214494405 | 0.03378797257 | 1.543298993 | 0.1227582477 | 1.053528434 |
| dnm1 | 0.05136099148 | 0.03329074472 | 1.542800917 | 0.1228790869 | 1.052702841 |
| adam23 | 0.04770686232 | 0.03092420793 | 1.542702805 | 0.122902901 | 1.048863149 |
| edar | 0.04851547388 | 0.03145070398 | 1.542587852 | 0.1229308075 | 1.049711615 |
| gys1 | 0.04874379653 | 0.03160939049 | 1.542066955 | 0.1230573241 | 1.049951315 |
| dffa | 0.04809334666 | 0.03120998737 | 1.540960145 | 0.1233264862 | 1.049268596 |
| vgf | -0.05264556239 | 0.03417741825 | -1.540361007 | 0.1234723806 | 0.9487162136 |
| mthfsd | 0.05170062683 | 0.033603363 | 1.538555139 | 0.1239129377 | 1.053060437 |
| cd48 | -0.04814381092 | 0.03136270837 | -1.535065478 | 0.124767745 | 0.9529967259 |
| lbp | 0.04890457769 | 0.03187184816 | 1.534412985 | 0.1249280851 | 1.050120141 |
| ap1g2 | 0.05152519773 | 0.0335903479 | 1.533928672 | 0.1250472014 | 1.052875716 |
| zbtb16 | 0.04883630704 | 0.031876861 | 1.53202999 | 0.1255150343 | 1.050048451 |
| shc1 | 0.05231405638 | 0.03415269481 | 1.531769504 | 0.1255793241 | 1.053706614 |
| nfatc1 | 0.04810606682 | 0.03144970624 | 1.529618956 | 0.1261110745 | 1.049281943 |
| vamp5 | 0.04856063191 | 0.03176215046 | 1.528883631 | 0.126293295 | 1.049759019 |
| babam1 | 0.05116160996 | 0.03349015694 | 1.527661099 | 0.1265967033 | 1.052492973 |
| arhgef1 | 0.05097885252 | 0.03337160126 | 1.52761182 | 0.1266089454 | 1.05230064 |
| irag2 | 0.04771556583 | 0.03125259004 | 1.526771566 | 0.1268178245 | 1.048872278 |
| tarbp2 | 0.04856386176 | 0.03184976602 | 1.524779232 | 0.1273141717 | 1.049762409 |
| fcrl1 | -0.04802888449 | 0.031565927 | -1.521542025 | 0.1281238736 | 0.9531062567 |
| scin | -0.05147002157 | 0.03384335107 | -1.520831122 | 0.1283022224 | 0.949832124 |
| dok1 | 0.05027665564 | 0.03307603493 | 1.520032729 | 0.1285027501 | 1.051561977 |
| cdh2 | 0.05343552196 | 0.03516499933 | 1.519565562 | 0.1286201988 | 1.054888972 |
| fli1 | 0.04831187202 | 0.03182525486 | 1.518035668 | 0.1290054073 | 1.049497913 |
| edem2 | 0.05092612886 | 0.03356431995 | 1.517269795 | 0.1291985809 | 1.05224516 |
| mansc1 | 0.0474440012 | 0.03131473016 | 1.515069776 | 0.1297547348 | 1.04858748 |
| ift20 | 0.05084544178 | 0.03358906308 | 1.513749927 | 0.1300892769 | 1.052160261 |
| ythdf3 | 0.04814318074 | 0.03181354254 | 1.513292042 | 0.1302054933 | 1.049320887 |
| fabp3 | -0.0593941739 | 0.03925033412 | -1.513214479 | 0.1302251876 | 0.942335252 |
| aifm1 | 0.04754354662 | 0.03146512219 | 1.510991959 | 0.1307904996 | 1.048691867 |
| dscam | 0.05046922049 | 0.03340216689 | 1.510956479 | 0.1307995394 | 1.05176449 |
| galnt2 | -0.04732914193 | 0.03135079283 | -1.509663318 | 0.1311293557 | 0.9537734191 |
| prkab1 | 0.04846769265 | 0.03214513908 | 1.507776729 | 0.1316116801 | 1.049661459 |
| egfr | 0.04792115224 | 0.03179412385 | 1.507232986 | 0.1317509479 | 1.049087934 |
| anxa4 | 0.04706547406 | 0.03123556027 | 1.506791415 | 0.1318641307 | 1.048190636 |
| cst5 | 0.04845871053 | 0.03216300148 | 1.506660085 | 0.1318978078 | 1.049652031 |
| rab33a | 0.05045548103 | 0.03352406428 | 1.505052627 | 0.132310547 | 1.051750039 |
| tnfaip8 | 0.04750361522 | 0.03156534105 | 1.504929573 | 0.1323421843 | 1.048649992 |
| itgax | -0.05025059564 | 0.03340493166 | -1.504286737 | 0.1325075526 | 0.9509910804 |
| ctsh | 0.04809685457 | 0.03199613409 | 1.503208307 | 0.132785336 | 1.049272277 |
| icam1 | 0.04946194994 | 0.03299024374 | 1.499290224 | 0.1337983573 | 1.050705612 |
| fetub | 0.05011936086 | 0.03344329804 | 1.498636911 | 0.1339678518 | 1.051396584 |
| nucb2 | 0.04843228718 | 0.03232941495 | 1.498087338 | 0.1341105604 | 1.049624296 |
| rnf5 | 0.05070102622 | 0.03386827476 | 1.497006463 | 0.1343915763 | 1.052008323 |
| mylpf | -0.05307084708 | 0.03545199271 | -1.496977829 | 0.134399027 | 0.9483128249 |
| nfkb1 | 0.0511786115 | 0.03419522591 | 1.496659552 | 0.1344818659 | 1.052510867 |
| nectin4 | 0.0465527287 | 0.0311335618 | 1.495258686 | 0.1348469428 | 1.047653319 |
| actn4 | 0.04709556802 | 0.03151687782 | 1.494296747 | 0.1350980749 | 1.048222181 |
| tyro3 | 0.04746562747 | 0.03179291175 | 1.492962578 | 0.135446983 | 1.048610157 |
| cdc37 | 0.04629501652 | 0.03108551578 | 1.489279343 | 0.136413825 | 1.047383361 |
| dtx2 | 0.05051469501 | 0.03394618229 | 1.488081769 | 0.1367293306 | 1.05181232 |
| ada2 | 0.04817491139 | 0.03241068002 | 1.486390022 | 0.1371759875 | 1.049354183 |
| fgf21 | 0.05032408178 | 0.03388060542 | 1.485335966 | 0.137454849 | 1.051611849 |
| gsn | -0.04942159274 | 0.03329267487 | -1.484458456 | 0.1376873366 | 0.9517797817 |
| lrp1 | 0.04928347766 | 0.03326782527 | 1.48141567 | 0.1384958416 | 1.050518107 |
| ces3 | -0.04699123149 | 0.03175743395 | -1.479692332 | 0.1389553729 | 0.9540957635 |

| Protein | beta | se | z | p_val | HR |
|---|---|---|---|---|---|
| rbp7 | 0.05297656242 | 0.03583234475 | 1.478456484 | 0.1392856365 | 1.054404932 |
| calcb | 0.05032770267 | 0.03406913757 | 1.477222679 | 0.1396159565 | 1.051615657 |
| pbld | 0.04809618486 | 0.03259714757 | 1.475472194 | 0.1400856384 | 1.049271575 |
| stx8 | 0.04652954795 | 0.03153935124 | 1.475285512 | 0.1401357998 | 1.047629034 |
| wars | 0.04622301889 | 0.03134745066 | 1.474538373 | 0.1403366938 | 1.047307954 |
| il4 | -0.04590076134 | 0.03114907467 | -1.473583464 | 0.1405937764 | 0.955136744 |
| pfkfb2 | 0.04643090116 | 0.03152106965 | 1.473011597 | 0.1407479087 | 1.047525694 |
| lactb2 | 0.04621548971 | 0.03137562846 | 1.472974151 | 0.1407580058 | 1.047300069 |
| gabarap | -0.04959109248 | 0.03367897191 | -1.472464558 | 0.1408954711 | 0.9516184689 |
| plg | 0.05201387087 | 0.03535097659 | 1.471355982 | 0.141194871 | 1.053390354 |
| khk | 0.05172244732 | 0.03523661461 | 1.467860857 | 0.1421420226 | 1.053083416 |
| pcdh12 | 0.04851424747 | 0.03306042331 | 1.467441811 | 0.1422559075 | 1.049710327 |
| sdccag8 | 0.04854429556 | 0.03308298098 | 1.467349499 | 0.1422810049 | 1.04974187 |
| umod | 0.04848589972 | 0.03306025183 | 1.466591966 | 0.1424870869 | 1.049680571 |
| fam172a | 0.04974835158 | 0.03394827225 | 1.465416302 | 0.1428073724 | 1.051006579 |
| pttg1 | 0.04897133901 | 0.03342637345 | 1.465050915 | 0.1429070271 | 1.050190251 |
| dnajc21 | 0.04820544116 | 0.03290802462 | 1.464853686 | 0.142960841 | 1.04938622 |
| scly | 0.0495368487 | 0.03383570536 | 1.464040669 | 0.1431828368 | 1.050784312 |
| serpina6 | 0.05665622013 | 0.03874131162 | 1.462423903 | 0.1436250833 | 1.058291928 |
| polr2a | 0.04910004728 | 0.03358725124 | 1.461865603 | 0.1437780425 | 1.050325428 |
| cd40 | 0.04701066061 | 0.03215996765 | 1.461775743 | 0.1438026733 | 1.048133183 |
| muc2 | 0.04831372194 | 0.03305283874 | 1.461711725 | 0.1438202228 | 1.049499855 |
| acadm | 0.04904496105 | 0.03356533922 | 1.461178769 | 0.1439663878 | 1.050267571 |
| odam | 0.04507272793 | 0.03085029433 | 1.461014519 | 0.144011457 | 1.046103938 |
| il20 | 0.04555392733 | 0.03118137499 | 1.460933886 | 0.1440335862 | 1.046607444 |
| htra2 | 0.04518231932 | 0.03093270806 | 1.460664848 | 0.1441074404 | 1.046218588 |
| pagr1 | 0.04950237662 | 0.03392095744 | 1.459344911 | 0.1444702001 | 1.050748089 |
| brd3 | -0.04931633164 | 0.0338322317 | -1.457673029 | 0.1449306896 | 0.9518799723 |
| apoa2 | -0.04988244779 | 0.03427809349 | -1.455228186 | 0.1456061003 | 0.9513412502 |
| fdx2 | 0.04905313687 | 0.03373318364 | 1.454150827 | 0.1459044947 | 1.050276158 |
| klb | 0.04664141058 | 0.03208826413 | 1.453534862 | 0.1460753075 | 1.047746231 |
| lilrb4 | 0.04924106833 | 0.03390635734 | 1.452266542 | 0.1464275067 | 1.050473556 |
| sfrp4 | -0.05163540415 | 0.03560250159 | -1.450330787 | 0.1469662977 | 0.9496750513 |
| c2cd2l | 0.04611689236 | 0.03181021441 | 1.44975107 | 0.1471279488 | 1.047196813 |
| trim21 | 0.04549220051 | 0.03139738575 | 1.448916826 | 0.1473608117 | 1.046542842 |
| mslnl | -0.04815809587 | 0.0332462957 | -1.448525162 | 0.1474702341 | 0.9529831125 |
| pkd2 | 0.05046446407 | 0.03485169382 | 1.447977374 | 0.1476233787 | 1.051759487 |
| upb1 | 0.05283920036 | 0.03650081881 | 1.44761685 | 0.147724236 | 1.054260107 |
| enpp5 | 0.05109239388 | 0.03532333644 | 1.446420384 | 0.1480593278 | 1.052420126 |
| sod2 | 0.04534078096 | 0.03141323562 | 1.443365514 | 0.1489175335 | 1.046384387 |
| bcam | 0.04574098177 | 0.03171243592 | 1.442367338 | 0.149198773 | 1.046803235 |
| raly | 0.04773354054 | 0.03311570417 | 1.441417048 | 0.149466897 | 1.048891131 |
| erbb2 | 0.0494482147 | 0.03432461694 | 1.440604998 | 0.1496963077 | 1.05069118 |
| stoml2 | -0.04780351983 | 0.03339473574 | -1.431468726 | 0.1522959238 | 0.9533210774 |
| csh1 | 0.04705445487 | 0.03290263767 | 1.430111936 | 0.1526848949 | 1.048179086 |
| gdnf | -0.04709985473 | 0.0329490027 | -1.429477401 | 0.1528670659 | 0.9539921322 |
| abraxas2 | 0.04823528216 | 0.03382400207 | 1.426066675 | 0.1538490971 | 1.049417536 |
| arsb | 0.04510380643 | 0.03168788078 | 1.423377181 | 0.1546268437 | 1.04613645 |
| ensa | 0.04785022327 | 0.03362295504 | 1.423141518 | 0.1546951349 | 1.049013526 |
| prune2 | 0.04782568127 | 0.03360703249 | 1.423085519 | 0.1547113657 | 1.048987781 |
| eppk1 | 0.04855191953 | 0.03412653933 | 1.422702697 | 0.1548223586 | 1.049749873 |
| git1 | 0.04774030755 | 0.0335895854 | 1.421283025 | 0.1552344963 | 1.048898229 |
| fabp2 | 0.04431548389 | 0.03118195035 | 1.421190252 | 0.1552614578 | 1.045312082 |
| cd69 | 0.04409339537 | 0.03103923615 | 1.420569603 | 0.1554419206 | 1.045079956 |
| ntprobnp | -0.04682159951 | 0.03297008896 | -1.420123542 | 0.1555717176 | 0.9542576224 |
| crym | 0.04777898828 | 0.03373182816 | 1.416436371 | 0.1566477813 | 1.048938802 |
| itga6 | 0.04405445159 | 0.03114450755 | 1.41451752 | 0.1572100068 | 1.045039257 |
| znrd2 | 0.04786963145 | 0.03384866674 | 1.414225022 | 0.1572958435 | 1.049033885 |
| crybb1 | 0.04765869745 | 0.03372685458 | 1.41307863 | 0.157632606 | 1.048812632 |
| atox1 | 0.04477913863 | 0.03169739954 | 1.412707013 | 0.1577418887 | 1.045796858 |
| vegfd | -0.04614606541 | 0.03266520241 | -1.412697978 | 0.1577445462 | 0.9549024738 |
| cx3cl1 | 0.04414682136 | 0.03128392269 | 1.411166426 | 0.1581955474 | 1.045135792 |
| ipcef1 | 0.04471855723 | 0.03170594046 | 1.410415732 | 0.1584169627 | 1.045733504 |
| serpina7 | 0.04721218543 | 0.03352535316 | 1.408253187 | 0.1590561122 | 1.048344429 |
| itgb6 | 0.04723769347 | 0.03355659363 | 1.407702283 | 0.1592192454 | 1.04837117 |
| serpinc1 | 0.04906069515 | 0.034851992 | 1.407686974 | 0.1592237807 | 1.050284096 |
| rfc4 | 0.04732688057 | 0.03364120118 | 1.406813042 | 0.1594828341 | 1.048464676 |
| ptprb | 0.04884535075 | 0.03472367365 | 1.4066873 | 0.1595201332 | 1.050057948 |
| cpxm1 | 0.04319008653 | 0.03070673031 | 1.406534857 | 0.1595653615 | 1.044136352 |
| ccdc28a | -0.04733144148 | 0.03370206713 | -1.404407667 | 0.160197489 | 0.9537712258 |

| Protein | beta | se | z | p_val | HR |
|---|---|---|---|---|---|
| snx18 | 0.04622288646 | 0.03298668647 | 1.401258853 | 0.1611366807 | 1.047307816 |
| ist1 | 0.04689278401 | 0.0335189808 | 1.398991941 | 0.1618153997 | 1.04800964 |
| f11r | 0.04422882494 | 0.03162442047 | 1.398565548 | 0.1619433037 | 1.0452215 |
| lyzl2 | 0.05305523719 | 0.03793613441 | 1.398540943 | 0.1619506868 | 1.05448789 |
| cd5l | 0.04610992548 | 0.03297922481 | 1.398150676 | 0.1620678248 | 1.047189517 |
| wasf1 | 0.04405019012 | 0.03150641559 | 1.398133977 | 0.1620728385 | 1.045034804 |
| ceacam8 | 0.04552822184 | 0.03259281608 | 1.396879046 | 0.1624499468 | 1.046580541 |
| efhd1 | -0.0498012402 | 0.03578263216 | -1.391771292 | 0.1639916658 | 0.9514185095 |
| cntnap4 | -0.04707129896 | 0.03382608545 | -1.391568026 | 0.1640532467 | 0.9540193746 |
| ppp1r12b | 0.04651355969 | 0.0334553528 | 1.390317417 | 0.1644325112 | 1.047612284 |
| clec12a | 0.04667736337 | 0.03360996581 | 1.388795324 | 0.1648949975 | 1.047783901 |
| ddx4 | -0.04665886823 | 0.03367326365 | -1.385635462 | 0.1658582441 | 0.9544129226 |
| serpinb1 | 0.04367184348 | 0.03152572962 | 1.385276217 | 0.165968023 | 1.044639493 |
| inppl1 | 0.04315346809 | 0.03119773569 | 1.383224363 | 0.1665960815 | 1.044098118 |
| il1rap | -0.04484079292 | 0.03242257791 | -1.38301134 | 0.1666613889 | 0.9561496955 |
| selplg | -0.04364231843 | 0.03160652202 | -1.380801039 | 0.167340144 | 0.9572963035 |
| mindy1 | 0.04559489601 | 0.03302892156 | 1.380453671 | 0.1674470049 | 1.046650323 |
| tbcc | 0.04309274681 | 0.03125728672 | 1.378646432 | 0.1680037931 | 1.044034721 |
| eif2ak2 | 0.04605498806 | 0.03340883276 | 1.378527301 | 0.1680405446 | 1.047131989 |
| ddah1 | 0.04448176213 | 0.03228432971 | 1.377812782 | 0.1682610986 | 1.045485909 |
| arg2 | 0.04562461632 | 0.03313457273 | 1.376948986 | 0.1685280205 | 1.04668143 |
| gip | -0.04727645976 | 0.03435489147 | -1.376120189 | 0.1687844259 | 0.9538236673 |
| ism2 | -0.04561472856 | 0.03316924276 | -1.375211635 | 0.1690658423 | 0.9554099835 |
| cd36 | 0.04781801485 | 0.03477360428 | 1.375123915 | 0.1690930314 | 1.048979739 |
| ly6d | 0.04416136171 | 0.03212197021 | 1.374802399 | 0.1691927142 | 1.045150989 |
| hnrnpul1 | 0.0477192314 | 0.03483816462 | 1.369740109 | 0.1707680435 | 1.048876123 |
| scgb1a1 | -0.04392133463 | 0.03207845858 | -1.369184698 | 0.170941548 | 0.9570292396 |
| ndufa5 | 0.04711430143 | 0.03442725769 | 1.368517407 | 0.1711501771 | 1.048241818 |
| ly96 | 0.04436553417 | 0.03244103566 | 1.367574532 | 0.1714452927 | 1.045364401 |
| idi2 | -0.04472501681 | 0.03271163938 | -1.367250852 | 0.1715466911 | 0.9562604012 |
| lmnb2 | 0.04930999451 | 0.03609258566 | 1.366208422 | 0.1718735547 | 1.050545964 |
| f11 | 0.04765699089 | 0.03489609344 | 1.365682694 | 0.1720385785 | 1.048810842 |
| gsta1 | 0.04795153746 | 0.03511434034 | 1.36558275 | 0.1720699638 | 1.049119811 |
| hla_e | -0.04458910269 | 0.03265558753 | -1.365435629 | 0.1721161721 | 0.9563903793 |
| b3gnt7 | 0.04806855694 | 0.03524276359 | 1.363927004 | 0.172590541 | 1.049242586 |
| mmp10 | 0.0429671272 | 0.03150826423 | 1.363678014 | 0.1726689266 | 1.043903578 |
| eif4b | 0.04262806174 | 0.03127531359 | 1.362993903 | 0.1728844322 | 1.043549687 |
| pyy | -0.04647179532 | 0.03409695905 | -1.36293079 | 0.1729043239 | 0.9545914841 |
| bag4 | 0.0470500466 | 0.03452536796 | 1.36276742 | 0.1729558222 | 1.048174465 |
| gtpbp2 | 0.04561452387 | 0.0335211541 | 1.3607683 | 0.1735869227 | 1.046670867 |
| tnfsf14 | 0.04300902495 | 0.03162835419 | 1.359824944 | 0.173885327 | 1.043947316 |
| slc12a2 | 0.04514373109 | 0.03320361202 | 1.359603017 | 0.1739555828 | 1.046178217 |
| eif4g1 | 0.04237621207 | 0.03117518945 | 1.359292849 | 0.1740538092 | 1.043286902 |
| pecr | 0.04563914653 | 0.03357706684 | 1.359235658 | 0.1740719254 | 1.046696639 |
| fga | 0.04949226506 | 0.03641184902 | 1.35923515 | 0.1740720863 | 1.050737465 |
| evi5 | 0.04602748262 | 0.03394840813 | 1.355806801 | 0.1751606469 | 1.047103188 |
| tjap1 | 0.0421883485 | 0.03111930842 | 1.355696853 | 0.1751956411 | 1.043090925 |
| gimap8 | 0.04647968704 | 0.03428777275 | 1.355576152 | 0.1752340639 | 1.047576799 |
| spon2 | 0.04445183538 | 0.03281043107 | 1.354808027 | 0.1754787285 | 1.045454622 |
| xiap | 0.04612596831 | 0.03404886385 | 1.354699191 | 0.1755134158 | 1.047206317 |
| crygd | 0.04500047216 | 0.03323212808 | 1.354125503 | 0.175696341 | 1.046028354 |
| mtss2 | 0.04521746701 | 0.03342045617 | 1.352987727 | 0.1760595514 | 1.046255361 |
| dpep2 | 0.04225684298 | 0.03123720766 | 1.352772739 | 0.1761282442 | 1.043162373 |
| bnip3l | 0.04672578054 | 0.0345597319 | 1.352029601 | 0.176365846 | 1.047834633 |
| mcee | 0.04473161792 | 0.03309624968 | 1.351561532 | 0.1765156233 | 1.045747162 |
| gp2 | 0.04516282566 | 0.03342249271 | 1.351270417 | 0.1766088249 | 1.046198194 |
| st3gal1 | 0.04487338343 | 0.03329414814 | 1.3477859 | 0.1777272541 | 1.045895424 |
| brdt | 0.04584194173 | 0.03403506079 | 1.346903477 | 0.1780113215 | 1.046908925 |
| arhgap45 | 0.04490695455 | 0.03334359531 | 1.346794013 | 0.1780465833 | 1.045930536 |
| adcyap1r1 | -0.04301479539 | 0.03194232045 | -1.346639655 | 0.1780963161 | 0.9578972175 |
| tppp3 | 0.04293921869 | 0.03188824941 | 1.346553024 | 0.1781242321 | 1.043874445 |
| gc | 0.04553911587 | 0.03388237328 | 1.344035599 | 0.1789368756 | 1.046591942 |
| apom | 0.0437507802 | 0.03255560473 | 1.34387859 | 0.1789876503 | 1.044721957 |
| rad51 | 0.04483546166 | 0.03338518929 | 1.342974613 | 0.1792801945 | 1.045855762 |
| c1rl | 0.04706838065 | 0.03505272697 | 1.342787986 | 0.1793406347 | 1.048193683 |
| cpe | -0.0417809101 | 0.03116959416 | -1.340438053 | 0.1801029714 | 0.9590798823 |
| zhx2 | 0.04610197532 | 0.03442360954 | 1.339254539 | 0.1804878229 | 1.047181192 |
| tymp | 0.04219470822 | 0.03151634046 | 1.338820041 | 0.1806292649 | 1.043097559 |
| ophn1 | 0.04423501013 | 0.03306936977 | 1.337642974 | 0.1810128485 | 1.045227965 |
| lif | 0.0423182815 | 0.03163824323 | 1.337567361 | 0.1810375101 | 1.043226466 |

| Protein | beta | se | z | p_val | HR |
|---|---|---|---|---|---|
| glyr1 | 0.0450495947 | 0.03370299503 | 1.336664432 | 0.181332197 | 1.046079739 |
| plxna4 | 0.04137982596 | 0.03097772378 | 1.33579298 | 0.1816169481 | 1.042247903 |
| ceacam5 | 0.04372534272 | 0.03273982125 | 1.335540057 | 0.1816996542 | 1.044695382 |
| chga | 0.0441935843 | 0.03311307148 | 1.334626549 | 0.1819986044 | 1.045184667 |
| anxa3 | 0.04154594596 | 0.03113023561 | 1.334585015 | 0.1820122052 | 1.042421056 |
| rpa2 | 0.04488073659 | 0.03363010364 | 1.334540538 | 0.1820267707 | 1.045903115 |
| ostn | -0.04512746673 | 0.03382199399 | -1.334263933 | 0.1821173733 | 0.9558756317 |
| nphs2 | -0.0443051709 | 0.03321886852 | -1.333735099 | 0.1822906875 | 0.9566619675 |
| washc3 | 0.0449698374 | 0.03372538136 | 1.333412273 | 0.1823965472 | 1.045996309 |
| trim40 | -0.04443854011 | 0.03332767687 | -1.33338247 | 0.1824063221 | 0.9565343868 |
| stx4 | 0.04279522369 | 0.0320999853 | 1.333185149 | 0.1824710517 | 1.043724143 |
| ppm1f | 0.04446816406 | 0.03336048834 | 1.332959026 | 0.1825452503 | 1.045471693 |
| fam171b | 0.04434485814 | 0.03332386697 | 1.330723658 | 0.1832799557 | 1.045342788 |
| aprt | 0.04194093946 | 0.03152396686 | 1.330446122 | 0.1833713268 | 1.042832887 |
| mmp3 | 0.05349168629 | 0.04029638671 | 1.327456148 | 0.1843578366 | 1.054948221 |
| cracr2a | 0.04189928761 | 0.03160270732 | 1.325813235 | 0.1849015681 | 1.042789452 |
| satb1 | 0.04412553133 | 0.03333118851 | 1.323851123 | 0.1855524936 | 1.045113541 |
| tbca | 0.04464677543 | 0.03372844683 | 1.323712759 | 0.1855984592 | 1.045658442 |
| ldlr | 0.04432963426 | 0.03349615827 | 1.323424433 | 0.185694271 | 1.045326874 |
| mapkapk2 | 0.0439858946 | 0.03328953333 | 1.321313044 | 0.1863970066 | 1.044967615 |
| tpt1 | 0.04132893843 | 0.031279038 | 1.321298258 | 0.1864019346 | 1.042194867 |
| asrgl1 | 0.04474978656 | 0.0338774388 | 1.32093181 | 0.186524102 | 1.045766162 |
| sfrp1 | 0.04541772849 | 0.03439111346 | 1.320623961 | 0.1866267793 | 1.046464907 |
| c1r | 0.04458265271 | 0.03379107463 | 1.31936179 | 0.1870481896 | 1.045591394 |
| col18a1 | 0.04387527101 | 0.03327682921 | 1.318493139 | 0.1873386202 | 1.044852023 |
| tnni3 | -0.04202127123 | 0.03189219384 | -1.317603657 | 0.1876363608 | 0.9588493845 |
| osmr | 0.04318804579 | 0.03279442942 | 1.316932374 | 0.1878612937 | 1.044134221 |
| pqbp1 | 0.04223632627 | 0.03213442701 | 1.314363759 | 0.1887238203 | 1.043140971 |
| angptl2 | 0.04369740124 | 0.03327053925 | 1.313396243 | 0.189049463 | 1.044666192 |
| myo9b | 0.04108405621 | 0.03129283887 | 1.312890031 | 0.1892200065 | 1.041939683 |
| elavl4 | -0.0453244454 | 0.03462077054 | -1.309169169 | 0.1904770533 | 0.9556873632 |
| tnfaip2 | 0.04492702532 | 0.03431774513 | 1.309148522 | 0.1904840457 | 1.045951529 |
| eno1 | 0.04089364373 | 0.03124257058 | 1.308907781 | 0.1905655906 | 1.041741304 |
| ezr | 0.0426702269 | 0.03261706836 | 1.308217723 | 0.1907994725 | 1.043593689 |
| esyt2 | 0.04378437613 | 0.03349202709 | 1.307307438 | 0.1911083191 | 1.044757056 |
| usp28 | -0.04480014478 | 0.034273817 | -1.307124467 | 0.1911704429 | 0.956188562 |
| tcn2 | 0.04091788864 | 0.03131650806 | 1.306591672 | 0.1913514264 | 1.041766561 |
| atp5if1 | 0.04053682175 | 0.03105251379 | 1.305428025 | 0.1917471406 | 1.041369654 |
| cfb | 0.04773296081 | 0.03665589726 | 1.30219049 | 0.1928512754 | 1.048890523 |
| il13ra1 | 0.04289160242 | 0.03294674636 | 1.301846378 | 0.192968906 | 1.043824741 |
| rnase4 | 0.04777076964 | 0.03670001384 | 1.301655358 | 0.1930342269 | 1.048930181 |
| ptn | 0.04385476873 | 0.03370943938 | 1.300964049 | 0.1932707609 | 1.044830602 |
| cfp | 0.04498222593 | 0.03458869981 | 1.300489067 | 0.1934334011 | 1.046009268 |
| skap2 | 0.0404781114 | 0.0311282912 | 1.300364068 | 0.1934762189 | 1.041308517 |
| icam4 | 0.04081918778 | 0.03139432318 | 1.300209198 | 0.1935292788 | 1.041663743 |
| itga11 | -0.04176207411 | 0.03217475522 | -1.297976436 | 0.1942954324 | 0.9590979477 |
| scamp3 | 0.04057766347 | 0.03127981701 | 1.29724747 | 0.1945460523 | 1.041412186 |
| lsp1 | 0.04260050499 | 0.03284724299 | 1.296927873 | 0.194656005 | 1.04352093 |
| cep170 | 0.04322909356 | 0.03335446262 | 1.296051268 | 0.1949578227 | 1.044177082 |
| urod | 0.04358165732 | 0.03362983853 | 1.295922289 | 0.1950022594 | 1.044545286 |
| vps53 | 0.04036353727 | 0.03114700175 | 1.295904421 | 0.1950084159 | 1.041189216 |
| tie1 | 0.0400799718 | 0.03096818236 | 1.294230683 | 0.1955857515 | 1.040894013 |
| fstl1 | 0.04450666401 | 0.03440425996 | 1.293638173 | 0.1957904304 | 1.045511944 |
| fbp1 | 0.04217644047 | 0.03261202178 | 1.293278925 | 0.1959146069 | 1.043078504 |
| gusb | 0.04501454398 | 0.03482993902 | 1.292409498 | 0.1962153694 | 1.046043073 |
| fn1 | 0.04496186642 | 0.03481986622 | 1.29127051 | 0.1966098926 | 1.045987972 |
| defb4a_defb4b | 0.04316242163 | 0.0334279512 | 1.29120751 | 0.1966317318 | 1.044107467 |
| omg | -0.04100614644 | 0.03180261718 | -1.289395342 | 0.1972606801 | 0.9598232304 |
| eng | 0.03991983784 | 0.0309823543 | 1.288470122 | 0.1975823628 | 1.040727344 |
| rgs10 | 0.04242302847 | 0.03292777503 | 1.288366081 | 0.1976185603 | 1.043335746 |
| camsap1 | 0.0424417175 | 0.03296270289 | 1.287567881 | 0.1978964262 | 1.043355245 |
| rtn4r | 0.04450831933 | 0.0346047184 | 1.286192213 | 0.198375988 | 1.045513675 |
| clu | 0.04395441967 | 0.0342078627 | 1.284921542 | 0.1988197025 | 1.044934725 |
| c8b | 0.04334495923 | 0.03374331927 | 1.284549362 | 0.1989498037 | 1.044298073 |
| gnly | 0.04014446337 | 0.03125287188 | 1.284504781 | 0.1989653917 | 1.040961144 |
| acp6 | -0.03984518479 | 0.0310435134 | -1.283526909 | 0.1993075385 | 0.9609381955 |
| casp3 | 0.03970013654 | 0.03100435498 | 1.280469681 | 0.200380001 | 1.04049872 |
| hspb1 | 0.04083027524 | 0.03190788223 | 1.279629746 | 0.2006753831 | 1.041675292 |
| tor1aip1 | 0.04376381297 | 0.03423314801 | 1.278404573 | 0.2011068124 | 1.044735573 |
| gcg | -0.04077346211 | 0.03192085813 | -1.277329762 | 0.2014858507 | 0.9600465922 |

| Protein | beta | se | z | p_val | HR |
|---|---|---|---|---|---|
| frmd7 | -0.04250114488 | 0.03328309705 | -1.276958837 | 0.2016167802 | 0.9583893683 |
| ogfr | 0.0412768309 | 0.03235481594 | 1.275755392 | 0.2020420008 | 1.042140562 |
| ptpn1 | 0.03999208239 | 0.0313497066 | 1.275676449 | 0.2020699171 | 1.040802533 |
| grik2 | -0.0420333659 | 0.03295975002 | -1.275293832 | 0.2022052601 | 0.9588377876 |
| tars1 | 0.04225764453 | 0.0331458957 | 1.274898253 | 0.2023452574 | 1.043163209 |
| il5ra | 0.03978815222 | 0.03121706051 | 1.274564343 | 0.2024634849 | 1.040590304 |
| map3k5 | 0.04044313407 | 0.03175241471 | 1.273702628 | 0.2027688246 | 1.041272095 |
| gh2 | 0.04008864706 | 0.031489644 | 1.273074001 | 0.2029917839 | 1.040903043 |
| nfya | 0.04348318505 | 0.03416320601 | 1.272807506 | 0.2030863569 | 1.044442432 |
| afp | 0.04129816691 | 0.03252730861 | 1.269645989 | 0.2042107599 | 1.042162798 |
| nppb | -0.03973928942 | 0.03132292074 | -1.268696804 | 0.204549223 | 0.9610399598 |
| rabep1 | 0.04203323592 | 0.03313981173 | 1.268360734 | 0.2046691574 | 1.042929141 |
| eef1d | 0.04214174188 | 0.03322770708 | 1.268271138 | 0.2047011403 | 1.043042311 |
| akr1b1 | 0.04026464066 | 0.03174943528 | 1.268200215 | 0.2047264601 | 1.041086251 |
| sart1 | 0.04446227205 | 0.03510699902 | 1.266478859 | 0.2053416937 | 1.045465533 |
| serpini1 | -0.04394385774 | 0.03470063248 | -1.266370513 | 0.2053804628 | 0.9570076845 |
| il11 | -0.04057712885 | 0.03204829077 | -1.266124585 | 0.2054684819 | 0.9602350998 |
| gbp2 | 0.04031287942 | 0.03185593672 | 1.26547462 | 0.2057012408 | 1.041136473 |
| coro6 | -0.04333572928 | 0.03428359448 | -1.264036923 | 0.2062167747 | 0.9575898451 |
| cr2 | -0.03959476663 | 0.03132939407 | -1.263821654 | 0.2062940471 | 0.961178862 |
| mdk | 0.04025019593 | 0.03185256205 | 1.263640767 | 0.2063589943 | 1.041071213 |
| prkra | 0.04068916664 | 0.03221700342 | 1.262971795 | 0.2065993162 | 1.041528313 |
| dusp13 | 0.04243357377 | 0.03366219466 | 1.260570625 | 0.2074635875 | 1.043346748 |
| folr3 | 0.04123025457 | 0.03271272757 | 1.260373489 | 0.2075346608 | 1.042092024 |
| phactr2 | 0.0418381428 | 0.03322761417 | 1.259137734 | 0.2079805872 | 1.042725692 |
| fuom | 0.04608578809 | 0.03662094155 | 1.258454484 | 0.2082274384 | 1.047164241 |
| lrch4 | 0.04365498866 | 0.03473325869 | 1.256864179 | 0.2088028215 | 1.044621886 |
| tfrc | 0.04071197315 | 0.0324095123 | 1.256173582 | 0.2090530427 | 1.041552067 |
| mpig6b | 0.03872134919 | 0.03084542256 | 1.255335346 | 0.2093570492 | 1.039480791 |
| ivd | 0.03954633466 | 0.03153153313 | 1.254183693 | 0.2097752458 | 1.040338702 |
| erbb3 | 0.04251649642 | 0.03390445281 | 1.25400922 | 0.2098386543 | 1.043433269 |
| cd99 | 0.0458967294 | 0.03660682688 | 1.253775137 | 0.2099237486 | 1.046966285 |
| cd59 | 0.04426273667 | 0.03535523494 | 1.251942937 | 0.2105906577 | 1.045256946 |
| adm | -0.04748360849 | 0.03796509888 | -1.250717367 | 0.2110376121 | 0.9536261044 |
| gpr158 | -0.04311608884 | 0.03447615884 | -1.250605934 | 0.2110782844 | 0.9578001937 |
| ppm1a | 0.03924099774 | 0.03138593676 | 1.250273269 | 0.2111997399 | 1.040021096 |
| hras | -0.04124094684 | 0.03305718888 | -1.247563639 | 0.2121909014 | 0.95959789 |
| tnip1 | 0.04128324154 | 0.03314513436 | 1.245529467 | 0.2129371908 | 1.042147243 |
| sat2 | 0.04231029059 | 0.03404856611 | 1.242645298 | 0.2139985697 | 1.043218129 |
| pag1 | 0.04032453076 | 0.03246049765 | 1.242264712 | 0.2141389106 | 1.041148604 |
| map2k6 | 0.03879513892 | 0.03124901543 | 1.24148356 | 0.2144271677 | 1.039557497 |
| csrp3 | -0.04365769104 | 0.03521150929 | -1.239869915 | 0.2150235131 | 0.9572815875 |
| ngf | 0.03849642685 | 0.03109347159 | 1.238087125 | 0.2156837572 | 1.039247015 |
| diablo | 0.03837247033 | 0.03104755641 | 1.235925618 | 0.2164862136 | 1.039118202 |
| mecr | 0.04132596545 | 0.03351706035 | 1.232982995 | 0.2175821086 | 1.042191769 |
| pdia3 | -0.04163520356 | 0.03379579752 | -1.231963931 | 0.2179625581 | 0.9592196367 |
| lcp1 | 0.04154402709 | 0.03373057093 | 1.231643164 | 0.2180824096 | 1.042419056 |
| rnf43 | -0.04150640272 | 0.03370218258 | -1.231564235 | 0.2181119078 | 0.9593431929 |
| tmed1 | 0.04059642428 | 0.03297695605 | 1.231054322 | 0.2183025479 | 1.041431724 |
| clstn2 | 0.03918890386 | 0.03187832403 | 1.229327609 | 0.2189489999 | 1.039966919 |
| impa1 | 0.03866359686 | 0.03146247866 | 1.228879558 | 0.219116967 | 1.03942076 |
| ifnar1 | 0.04111758393 | 0.03350152339 | 1.227334753 | 0.2196967987 | 1.041974618 |
| stx6 | 0.03923399801 | 0.03196887945 | 1.227255965 | 0.2197264006 | 1.040013816 |
| scpep1 | 0.0429778605 | 0.03502048728 | 1.227220517 | 0.2197397201 | 1.043914783 |
| cdc27 | 0.03913930196 | 0.03192032408 | 1.226156159 | 0.2201399159 | 1.039915336 |
| il1rl2 | 0.04129191675 | 0.03368939989 | 1.225664953 | 0.2203247839 | 1.042156284 |
| ckb | -0.04611862425 | 0.03774774298 | -1.221758458 | 0.2217989825 | 0.9549286778 |
| osm | 0.03978845554 | 0.03260001632 | 1.22050416 | 0.2222738135 | 1.04059062 |
| myo6 | 0.04039592466 | 0.03311948461 | 1.219702696 | 0.2225775993 | 1.041222938 |
| gmpr | 0.03833312992 | 0.03143282899 | 1.219525291 | 0.2226448827 | 1.039077323 |
| serpinb9 | 0.03788671018 | 0.03107473824 | 1.219212528 | 0.2227635381 | 1.038613562 |
| ghrhr | -0.03806488254 | 0.03124372907 | -1.218320721 | 0.2231021188 | 0.9626504797 |
| cd1c | 0.03855573023 | 0.03164774384 | 1.218277373 | 0.2231185855 | 1.039308648 |
| zpr1 | 0.0408179526 | 0.03351610727 | 1.217860781 | 0.2232768819 | 1.041662456 |
| clec4d | 0.03893246274 | 0.03197246365 | 1.217687294 | 0.2233428273 | 1.039700263 |
| eno2 | 0.03800973825 | 0.03124057166 | 1.216678704 | 0.2237264846 | 1.038741348 |
| ager | -0.0388917542 | 0.03197156871 | -1.216448106 | 0.2238142681 | 0.9618548203 |
| alcam | 0.04095645787 | 0.03368288206 | 1.215942798 | 0.224006713 | 1.041806742 |
| txlna | 0.03825348625 | 0.0314811635 | 1.215123013 | 0.2243191775 | 1.03899457 |
| sort1 | 0.03884877412 | 0.03199113685 | 1.214360537 | 0.224610078 | 1.039613255 |

| Protein | beta | se | z | p_val | HR |
|---|---|---|---|---|---|
| reep4 | 0.04002558676 | 0.03300772183 | 1.212612823 | 0.2252778838 | 1.040837406 |
| gatd3 | 0.04099242991 | 0.03387891464 | 1.209968806 | 0.2262908628 | 1.041844219 |
| npl | 0.04596917141 | 0.03800256463 | 1.209633398 | 0.2264195966 | 1.047042132 |
| ldlrap1 | 0.04013023607 | 0.03318569328 | 1.209263152 | 0.2265617623 | 1.040946334 |
| atg16l1 | 0.04034607623 | 0.03336509206 | 1.209230179 | 0.2265744266 | 1.041171036 |
| birc2 | 0.03848370825 | 0.03182865336 | 1.209090056 | 0.2266282493 | 1.039233797 |
| arhgef12 | 0.03764480835 | 0.03115040758 | 1.208485258 | 0.2268606628 | 1.03836235 |
| amotl2 | -0.04080915848 | 0.03378480066 | -1.207914733 | 0.2270800618 | 0.9600123227 |
| bmp4 | -0.0384362622 | 0.03183553129 | -1.207338487 | 0.2273018143 | 0.9622930372 |
| fut1 | -0.04066398969 | 0.03370145118 | -1.20659462 | 0.2275882995 | 0.9601516966 |
| casp8 | 0.0381488685 | 0.03166761516 | 1.204665028 | 0.2283326419 | 1.038885879 |
| rcor1 | 0.03833265322 | 0.03182980707 | 1.204300521 | 0.2284734456 | 1.039076828 |
| ctsl | 0.0386628745 | 0.03216066968 | 1.202178776 | 0.2292942706 | 1.03942001 |
| sned1 | 0.04115080517 | 0.03424297116 | 1.201729983 | 0.2294681604 | 1.042009234 |
| bcr | 0.03757866364 | 0.03131208279 | 1.200132993 | 0.2300876937 | 1.03829367 |
| man2b2 | 0.04085461219 | 0.0340521059 | 1.199767565 | 0.2302296241 | 1.041700644 |
| rln1 | -0.04101194917 | 0.03420648197 | -1.198952561 | 0.2305463922 | 0.9598176609 |
| coro1a | 0.03746655368 | 0.03125181945 | 1.198859917 | 0.2305824197 | 1.038177273 |
| srpk2 | 0.03753609188 | 0.03134655179 | 1.197455214 | 0.2311291738 | 1.038249469 |
| wfdc2 | 0.04459990262 | 0.03724865134 | 1.197356173 | 0.2311677584 | 1.045609431 |
| dgkz | 0.03779148482 | 0.03156354722 | 1.197314249 | 0.2311840928 | 1.038514664 |
| nudc | 0.03756598204 | 0.03137662257 | 1.19726022 | 0.2312051443 | 1.038280503 |
| stat5b | 0.0373051326 | 0.03116751068 | 1.196923713 | 0.2313362907 | 1.038009703 |
| calcoco1 | 0.03692543055 | 0.03089626415 | 1.195142246 | 0.2320314585 | 1.037615644 |
| s100a12 | 0.03770501856 | 0.03156686913 | 1.194449105 | 0.2323023383 | 1.038424872 |
| crtap | -0.04016900232 | 0.03365092235 | -1.193696918 | 0.2325965464 | 0.9606270772 |
| adamtsl5 | -0.04034088763 | 0.03379824158 | -1.193579481 | 0.2326425045 | 0.9604619737 |
| acot13 | 0.03941075191 | 0.03302567107 | 1.193336899 | 0.2327374568 | 1.040197659 |
| adgre5 | 0.03873060482 | 0.03247311243 | 1.192697648 | 0.2329878071 | 1.039490412 |
| arhgap1 | 0.03827880308 | 0.03210329906 | 1.192363533 | 0.2331187329 | 1.039020875 |
| smtn | 0.04011648436 | 0.03368883359 | 1.190794696 | 0.2337341915 | 1.040932019 |
| itgb1 | 0.0393362141 | 0.03303569995 | 1.190718349 | 0.2337641722 | 1.040120128 |
| snap29 | 0.0368081278 | 0.03092050148 | 1.190411734 | 0.2338846032 | 1.037493935 |
| vti1a | 0.0401507629 | 0.03374339643 | 1.189885049 | 0.2340915758 | 1.040967702 |
| adgrb3 | -0.03722641486 | 0.0313039245 | -1.189193223 | 0.2343636407 | 0.9634579694 |
| tab2 | 0.03966422837 | 0.03336838898 | 1.188676756 | 0.2345668907 | 1.040461358 |
| mrpl58 | 0.03902688021 | 0.03283581744 | 1.188546022 | 0.2346183593 | 1.039798433 |
| rab3gap1 | -0.03961159977 | 0.0333345585 | -1.188304317 | 0.2347135374 | 0.9611626825 |
| gchfr | 0.04364984559 | 0.03673342363 | 1.188286886 | 0.2347204025 | 1.044616514 |
| il2ra | 0.03753729366 | 0.03160368866 | 1.187750394 | 0.2349317633 | 1.038250717 |
| ceacam16 | 0.04166920509 | 0.03509709771 | 1.18725501 | 0.2351270483 | 1.042549552 |
| serpina5 | -0.04156708203 | 0.03501155522 | -1.187238949 | 0.2351333815 | 0.9592849824 |
| selp | 0.03765898769 | 0.03173257759 | 1.186761068 | 0.2353218792 | 1.038377073 |
| sh2b3 | 0.03774249912 | 0.03180497636 | 1.186685338 | 0.2353517606 | 1.038463793 |
| reck | 0.04270862209 | 0.03600251502 | 1.186267739 | 0.2355165826 | 1.043633759 |
| nfatc3 | 0.03808059149 | 0.03213445125 | 1.185039421 | 0.236001861 | 1.038814949 |
| glb1 | 0.03913776577 | 0.03302826595 | 1.184977916 | 0.2360261785 | 1.039913738 |
| cox5b | 0.03732939129 | 0.03151509734 | 1.184492337 | 0.236218228 | 1.038034884 |
| cwc15 | 0.04073811241 | 0.03439436291 | 1.184441547 | 0.236238322 | 1.041579293 |
| thap12 | -0.04029401794 | 0.03403375233 | -1.183942856 | 0.2364356839 | 0.9605069913 |
| golga3 | 0.03930587789 | 0.03319975578 | 1.183920694 | 0.2364444578 | 1.040088575 |
| ppp2r5a | 0.03967783004 | 0.03351788763 | 1.183780747 | 0.2364998656 | 1.04047551 |
| azi2 | 0.03914657591 | 0.03308173274 | 1.183329066 | 0.2366787577 | 1.0399229 |
| fkbp7 | 0.03754022951 | 0.03172510488 | 1.183297255 | 0.2366913605 | 1.038253765 |
| cep164 | 0.03706247077 | 0.03132442892 | 1.183181052 | 0.2367374006 | 1.037757848 |
| hmcn2 | 0.0401424528 | 0.03392944738 | 1.183115432 | 0.2367634026 | 1.040959051 |
| smpd3 | 0.04006916948 | 0.03387554856 | 1.182834557 | 0.236874722 | 1.040882769 |
| psmd1 | 0.04005652897 | 0.03387118197 | 1.182613852 | 0.2369622203 | 1.040869612 |
| bax | 0.03766646936 | 0.03185996654 | 1.182250751 | 0.2371062207 | 1.038384842 |
| brme1 | -0.03976885645 | 0.03363943339 | -1.182209462 | 0.2371225991 | 0.9610115451 |
| tnfrsf17 | -0.03836387498 | 0.03245163178 | -1.182186315 | 0.2371317815 | 0.9623626975 |
| nfkbie | 0.03793545094 | 0.0320958708 | 1.181941788 | 0.2372287993 | 1.038664186 |
| nell1 | 0.03613808631 | 0.03058270101 | 1.181651232 | 0.2373441165 | 1.036799004 |
| reg3a | 0.03812608223 | 0.03228246258 | 1.1810153 | 0.2375966453 | 1.038862207 |
| il1rn | 0.04351650014 | 0.03688422379 | 1.179813364 | 0.2380744532 | 1.044477228 |
| dut | 0.03915557468 | 0.03320603814 | 1.179170322 | 0.2383303612 | 1.039932258 |
| hddc2 | 0.03923735785 | 0.03327814603 | 1.179072831 | 0.2383691761 | 1.040017311 |
| src | 0.03660137694 | 0.0310734403 | 1.177899086 | 0.2388368395 | 1.037279455 |
| tnpo1 | 0.03930275089 | 0.03338682964 | 1.177193262 | 0.2391183775 | 1.040085323 |
| btc | 0.0372140591 | 0.03163608722 | 1.176316744 | 0.2394683273 | 1.037915172 |

| Protein | beta | se | z | p_val | HR |
|---|---|---|---|---|---|
| plin1 | 0.04121330814 | 0.03513316871 | 1.173059808 | 0.2407718225 | 1.042074365 |
| znf75d | -0.03917903934 | 0.03340661398 | -1.17279289 | 0.2408788702 | 0.9615785333 |
| dnajc6 | 0.03968202238 | 0.03386333533 | 1.171828528 | 0.2412659068 | 1.040479872 |
| mtdh | 0.03997696887 | 0.03411885931 | 1.171697111 | 0.2413186835 | 1.040786803 |
| glt8d2 | -0.03683420927 | 0.03145071154 | -1.171172526 | 0.2415294363 | 0.9638359172 |
| ep300 | 0.03993137256 | 0.03409536251 | 1.171167268 | 0.2415315493 | 1.040739348 |
| trpv3 | 0.0396891555 | 0.03389958361 | 1.170785929 | 0.2416848349 | 1.040487294 |
| chrdl2 | -0.03972996489 | 0.03397043788 | -1.169545269 | 0.2421840138 | 0.9610489211 |
| tprkb | 0.03924783008 | 0.03357584571 | 1.168930499 | 0.2424316344 | 1.040028202 |
| sparcl1 | 0.03721254319 | 0.03185044868 | 1.168352244 | 0.2426647097 | 1.037913599 |
| fxn | 0.03635703716 | 0.03114089474 | 1.167501367 | 0.2430079568 | 1.037026037 |
| bpifb2 | 0.04226686118 | 0.03622610017 | 1.166751623 | 0.2433106886 | 1.043172824 |
| gbp6 | -0.03898675012 | 0.03344726524 | -1.165618469 | 0.243768737 | 0.9617634523 |
| dsg4 | 0.037413287 | 0.03216604502 | 1.163129846 | 0.2447768244 | 1.038121975 |
| tpm3 | -0.03935965728 | 0.03384808704 | -1.162832548 | 0.2448974489 | 0.9614048707 |
| sema4d | 0.03614740837 | 0.03108570193 | 1.162830695 | 0.2448982008 | 1.03680867 |
| il9 | 0.03825337309 | 0.03289988772 | 1.162720475 | 0.2449429314 | 1.038994453 |
| cetn3 | 0.03897612208 | 0.03359875067 | 1.160046767 | 0.2460297659 | 1.039745656 |
| yap1 | -0.04479846243 | 0.03862831451 | -1.159731223 | 0.2461582538 | 0.9561901707 |
| cacnb3 | 0.03875299276 | 0.03345846258 | 1.158241885 | 0.24676534 | 1.039513685 |
| cfhr2 | 0.03830593397 | 0.0330921356 | 1.157554001 | 0.24704609 | 1.039049065 |
| csf3 | 0.03760171431 | 0.03248871402 | 1.157377737 | 0.2471180657 | 1.038317603 |
| gabarapl1 | -0.03845773644 | 0.03324914183 | -1.156653505 | 0.2474139528 | 0.9622723729 |
| vnn1 | 0.04049734052 | 0.0350459774 | 1.155548897 | 0.2478657212 | 1.04132854 |
| uso1 | 0.03605244088 | 0.03120348625 | 1.155397849 | 0.2479275426 | 1.036710211 |
| mtr | 0.0390090883 | 0.03379543866 | 1.154270808 | 0.2483891605 | 1.039779933 |
| jam2 | 0.03742236738 | 0.03242482893 | 1.154126903 | 0.2484481454 | 1.038131401 |
| fabp6 | 0.03578847531 | 0.03102089997 | 1.153689137 | 0.2486276392 | 1.036436591 |
| fis1 | 0.03658267855 | 0.03174552542 | 1.152372754 | 0.2491679322 | 1.03726006 |
| amfr | -0.03656458132 | 0.03174407503 | -1.151855308 | 0.2493805359 | 0.9640958293 |
| cxcl17 | -0.04490446795 | 0.03898492238 | -1.151841923 | 0.2493860372 | 0.9560888146 |
| larp1 | 0.03827297445 | 0.03324365418 | 1.151286626 | 0.2496143375 | 1.039014819 |
| mzt1 | 0.03704189497 | 0.03218277318 | 1.150985179 | 0.2497383327 | 1.037736496 |
| cyth3 | 0.03882542969 | 0.03378234778 | 1.14928157 | 0.2504398951 | 1.039588986 |
| commd1 | 0.03916300738 | 0.03410494652 | 1.148308717 | 0.2508411418 | 1.039939988 |
| tnn | 0.03802396271 | 0.03313782813 | 1.147448848 | 0.2511961624 | 1.038756124 |
| hars1 | 0.03571446799 | 0.03117222548 | 1.145714412 | 0.2519133387 | 1.03635989 |
| naga | -0.03933236805 | 0.03441298549 | -1.142951345 | 0.2530587928 | 0.9614311071 |
| hcls1 | 0.035850267 | 0.03141066238 | 1.141340688 | 0.2537281766 | 1.036500637 |
| c5 | -0.03876858577 | 0.03402838787 | -1.139301278 | 0.2545775157 | 0.9619732977 |
| enpp6 | -0.04124105377 | 0.03621572993 | -1.138760805 | 0.2548029337 | 0.9595977874 |
| kir3dl2 | -0.03800074823 | 0.03342463942 | -1.136908248 | 0.2555766439 | 0.9627122206 |
| gm2a | 0.04041228957 | 0.03557694729 | 1.135912231 | 0.2559932988 | 1.041239978 |
| scarf1 | 0.03583180128 | 0.03154490155 | 1.135898339 | 0.2559991135 | 1.036481497 |
| zfyve19 | 0.03784969781 | 0.03333919847 | 1.135291175 | 0.2562533386 | 1.038575121 |
| max | 0.03570538609 | 0.03148273842 | 1.134125806 | 0.2567417802 | 1.036350478 |
| itm2a | -0.03526752853 | 0.03109719549 | -1.134106403 | 0.2567499179 | 0.9653471238 |
| cd300a | -0.03836581229 | 0.03384001029 | -1.133741153 | 0.2569031418 | 0.9623608331 |
| rtn4ip1 | 0.03750691464 | 0.03312468597 | 1.13229495 | 0.2575104526 | 1.038219176 |
| retn | 0.03547477064 | 0.03133506911 | 1.132110815 | 0.2575878485 | 1.036111507 |
| dpp10 | -0.03624136796 | 0.03202448057 | -1.131676996 | 0.2577702564 | 0.9644074883 |
| trim5 | 0.03551578643 | 0.03138938732 | 1.131458415 | 0.2578621967 | 1.036154005 |
| enox2 | 0.03829946998 | 0.03386753732 | 1.130860789 | 0.2581136893 | 1.039042348 |
| dnmbp | 0.03637584022 | 0.03223446734 | 1.128476541 | 0.2591187186 | 1.037045537 |
| sncg | -0.03831227398 | 0.03397265407 | -1.127738619 | 0.2594303228 | 0.9624123576 |
| nectin2 | 0.03598664682 | 0.03194025347 | 1.126686326 | 0.2598751259 | 1.036642004 |
| rab2b | 0.03726515829 | 0.03307625868 | 1.126643695 | 0.259893157 | 1.03796821 |
| gart | 0.03755482913 | 0.03337737506 | 1.125158257 | 0.2605219785 | 1.038268923 |
| mcam | 0.0367902706 | 0.03270515029 | 1.124907554 | 0.2606282108 | 1.037475409 |
| gfra2 | -0.03496813624 | 0.03108735727 | -1.124834637 | 0.2606591137 | 0.9656361846 |
| unc79 | 0.03773946598 | 0.03359237416 | 1.12345337 | 0.2612449939 | 1.038460643 |
| serpinf2 | 0.03930624475 | 0.0350036029 | 1.122919971 | 0.2614714849 | 1.040088957 |
| sec31a | 0.03808599908 | 0.03394257165 | 1.1220717 | 0.2618319553 | 1.038820567 |
| l1cam | 0.03556599383 | 0.03170267421 | 1.121861001 | 0.2619215445 | 1.036206029 |
| ppif | 0.03682412558 | 0.03286002476 | 1.120635966 | 0.2624428492 | 1.037510533 |
| lpp | 0.03792904339 | 0.03386940876 | 1.119861396 | 0.262772831 | 1.038657531 |
| vcpkmt | 0.03746082052 | 0.03346153716 | 1.119518818 | 0.2629188676 | 1.038171321 |
| lrig3 | 0.03898382952 | 0.03498743063 | 1.114223846 | 0.2651831678 | 1.03975367 |
| amy2a | 0.03567278016 | 0.0320421473 | 1.113308038 | 0.2655761553 | 1.036316688 |
| cd207 | -0.03485037107 | 0.0313429561 | -1.111904409 | 0.266179253 | 0.9657499096 |

| Protein | beta | se | z | p_val | HR |
|---|---|---|---|---|---|
| hdgf | 0.03444968836 | 0.0310194717 | 1.110582691 | 0.2667480166 | 1.035049952 |
| ccl24 | 0.03516617849 | 0.03172164135 | 1.108586346 | 0.2676086709 | 1.035791821 |
| m6pr | -0.03724177763 | 0.03360695708 | -1.108156788 | 0.2677941091 | 0.9634431682 |
| bank1 | 0.03469670795 | 0.03133162909 | 1.107401975 | 0.2681201722 | 1.035305661 |
| hspa1a | 0.0353945929 | 0.03200359224 | 1.105956876 | 0.2687451851 | 1.036028438 |
| spink5 | 0.03475573438 | 0.03144726869 | 1.105206774 | 0.2690700024 | 1.035366773 |
| pzp | 0.05221768312 | 0.04727428528 | 1.104568431 | 0.2693466364 | 1.05360507 |
| gp6 | 0.03433836837 | 0.03112731013 | 1.103158873 | 0.2699581778 | 1.034934737 |
| clec1b | 0.03403114573 | 0.03086366998 | 1.102627969 | 0.2701887593 | 1.03461683 |
| pla2g2a | 0.03667747917 | 0.0332794495 | 1.102105946 | 0.2704156147 | 1.037358397 |
| fcgr2b | -0.03491199606 | 0.03168156563 | -1.101965619 | 0.2704766191 | 0.9656903971 |
| atraid | 0.03767897571 | 0.03423516296 | 1.100592854 | 0.271073897 | 1.038397828 |
| cadps | -0.03681784414 | 0.0334954581 | -1.099189151 | 0.2716855699 | 0.9638516906 |
| hpcal1 | 0.03420923377 | 0.03117212662 | 1.09743022 | 0.2724533691 | 1.034801099 |
| eps8l2 | -0.03594390248 | 0.03275766909 | -1.097266792 | 0.272524783 | 0.9646944089 |
| dpy30 | 0.03619853395 | 0.03301549856 | 1.096410338 | 0.2728992416 | 1.036861678 |
| snap23 | 0.03425338524 | 0.03124942029 | 1.096128661 | 0.2730224733 | 1.034846788 |
| fadd | 0.03428481891 | 0.03129208748 | 1.0956386 | 0.2732369625 | 1.034879318 |
| pcyt2 | 0.03665828472 | 0.03345931182 | 1.095607851 | 0.2732504245 | 1.037338486 |
| tf | 0.03704980701 | 0.033824666 | 1.095348791 | 0.2733638597 | 1.037744707 |
| podxl2 | 0.03466498829 | 0.0316799027 | 1.094226476 | 0.2738556624 | 1.035272822 |
| spon1 | 0.03679038623 | 0.03363607574 | 1.093777601 | 0.2740525302 | 1.037475529 |
| pydc1 | -0.03701971788 | 0.03384644282 | -1.093755054 | 0.2740624215 | 0.9636571339 |
| rab6b | 0.03412904382 | 0.03124508155 | 1.09230132 | 0.2747006804 | 1.034718122 |
| axin1 | 0.03405150086 | 0.03122540747 | 1.090506213 | 0.2754902179 | 1.03463789 |
| ing1 | 0.03459713123 | 0.03173097886 | 1.090326629 | 0.2755692889 | 1.035202574 |
| akap12 | -0.03658840232 | 0.03355818741 | -1.090297335 | 0.2755821886 | 0.9640728639 |
| rrm2 | 0.03477975606 | 0.03191405295 | 1.089794396 | 0.2758037231 | 1.035391645 |
| c1qbp | 0.03567386084 | 0.03275575728 | 1.089086738 | 0.2761156376 | 1.036317808 |
| clta | 0.03486227268 | 0.03201587567 | 1.088905799 | 0.2761954287 | 1.035477085 |
| tbcb | 0.03396152663 | 0.03122408946 | 1.087670681 | 0.2767405155 | 1.034544804 |
| tnfsf13 | 0.03574840219 | 0.0328750843 | 1.08740108 | 0.2768595939 | 1.036395059 |
| map2 | 0.03626789847 | 0.03335283206 | 1.087400866 | 0.2768596887 | 1.036933602 |
| gfod2 | -0.03440329585 | 0.0316424513 | -1.087251285 | 0.2769257712 | 0.966181769 |
| tgfbi | 0.03468007455 | 0.03190575599 | 1.086953544 | 0.277057341 | 1.035288441 |
| vegfb | -0.03863216943 | 0.03557290272 | -1.085999918 | 0.2774790286 | 0.9621045355 |
| igf2bp3 | 0.03679614601 | 0.03389736083 | 1.085516545 | 0.2776929399 | 1.037481505 |
| dpp4 | 0.03581474501 | 0.03305431506 | 1.083511939 | 0.2785812544 | 1.036463819 |
| agbl2 | 0.03659279461 | 0.03383029751 | 1.081657488 | 0.2794047499 | 1.037270553 |
| acy1 | 0.03883582708 | 0.03593737149 | 1.080652966 | 0.2798515125 | 1.039599796 |
| sirpa | 0.03307516382 | 0.03061494071 | 1.080360212 | 0.2799818066 | 1.033628228 |
| pcsk7 | 0.0359646444 | 0.03329729778 | 1.080107 | 0.2800945352 | 1.036619196 |
| ptprc | 0.0363296769 | 0.03368875875 | 1.078391672 | 0.2808590017 | 1.036997664 |
| stc2 | -0.03645481479 | 0.03382236601 | -1.077831597 | 0.2811089158 | 0.9642016606 |
| spring1 | -0.03684996371 | 0.034199449 | -1.077501679 | 0.2812562012 | 0.9638207326 |
| nme1 | 0.03625597188 | 0.03366820737 | 1.076860775 | 0.2815424691 | 1.036921235 |
| mog | -0.03470769676 | 0.03224471938 | -1.076383899 | 0.2817556003 | 0.9658877071 |
| foxo1 | 0.03379146251 | 0.03140248616 | 1.076076026 | 0.2818932566 | 1.03436888 |
| akt2 | 0.03581586548 | 0.03329918034 | 1.075577991 | 0.2821160351 | 1.03646498 |
| myoc | 0.03481571978 | 0.03240682251 | 1.074333029 | 0.2826734467 | 1.035428882 |
| bsg | -0.03464138622 | 0.03224614933 | -1.07427978 | 0.2826973046 | 0.9659517578 |
| cdhr5 | 0.03480538407 | 0.03240763197 | 1.073987267 | 0.2828283886 | 1.03541818 |
| dnaja2 | 0.0336702347 | 0.03135118249 | 1.073970167 | 0.2828360526 | 1.034243493 |
| c4bpb | 0.03460389015 | 0.03225314721 | 1.072884141 | 0.2833231023 | 1.035209571 |
| gfer | 0.0350789898 | 0.03271136296 | 1.072379339 | 0.2835496841 | 1.035701515 |
| pros1 | 0.03653521908 | 0.03408960818 | 1.071740657 | 0.2838365339 | 1.037210833 |
| tspan8 | -0.03613756743 | 0.03371878121 | -1.071734094 | 0.2838394827 | 0.9645075995 |
| tnfsf10 | 0.03387232102 | 0.0316233497 | 1.071117429 | 0.2841166324 | 1.03445252 |
| arhgap30 | -0.03586264786 | 0.03348576529 | -1.07098188 | 0.2841775772 | 0.964772798 |
| reg1a | 0.03462592107 | 0.0323344276 | 1.070868534 | 0.2842285462 | 1.035232378 |
| kiaa0319 | 0.03670049612 | 0.03427835747 | 1.070660873 | 0.2843219419 | 1.037382274 |
| nudt15 | 0.03593480616 | 0.03360857804 | 1.069215309 | 0.284972662 | 1.036588265 |
| cd33 | -0.03309278703 | 0.03099608985 | -1.067643925 | 0.2856811617 | 0.9674487887 |
| cdc42bpb | 0.03517367431 | 0.03297063661 | 1.066818173 | 0.2860539507 | 1.035799585 |
| epha4 | -0.03545427206 | 0.03329312905 | -1.064912583 | 0.2869154902 | 0.9651668683 |
| dbnl | 0.03320394789 | 0.03120582617 | 1.064030406 | 0.2873149255 | 1.033761351 |
| lpcat2 | 0.03396478443 | 0.03195865355 | 1.062772697 | 0.2878850439 | 1.034548174 |
| klk7 | 0.03683045602 | 0.03471052871 | 1.061074474 | 0.2886560571 | 1.037517101 |
| cacybp | 0.03558452594 | 0.03354604776 | 1.060766568 | 0.2887959993 | 1.036225232 |
| olr1 | 0.03399135861 | 0.03206578677 | 1.060050666 | 0.28912155 | 1.034575667 |

| Protein | beta | se | z | p_val | HR |
|---|---|---|---|---|---|
| anxa5 | 0.03389616606 | 0.0319812543 | 1.059876068 | 0.2892009846 | 1.034477187 |
| cd22 | -0.03399835529 | 0.0321235098 | -1.058363657 | 0.2898896819 | 0.9665730944 |
| il7r | -0.033684421 | 0.03183864654 | -1.057972768 | 0.2900678579 | 0.9668765824 |
| mamdc4 | -0.03703906188 | 0.03502413671 | -1.057529617 | 0.2902699451 | 0.9636384931 |
| stk11 | 0.03365379576 | 0.03183814915 | 1.057027392 | 0.2904990863 | 1.034226491 |
| mgll | 0.03291333285 | 0.03118707509 | 1.055351704 | 0.2912645022 | 1.033460968 |
| ddhd2 | 0.03515918342 | 0.03332886068 | 1.054917051 | 0.2914632632 | 1.035784575 |
| pcbp2 | 0.0349970163 | 0.03332130824 | 1.050289384 | 0.2935850847 | 1.035616619 |
| tfap2a | 0.03541722903 | 0.03375434478 | 1.049264302 | 0.294056491 | 1.03605189 |
| mrpl52 | 0.03521975546 | 0.03367453341 | 1.045886962 | 0.2956132245 | 1.035847317 |
| gpi | 0.03497000241 | 0.03344270723 | 1.045669005 | 0.2957138776 | 1.035588643 |
| map4k5 | 0.03255876564 | 0.03115762114 | 1.04496956 | 0.2960370387 | 1.033094602 |
| gstt2b | -0.03490585135 | 0.03340995225 | -1.044774057 | 0.296127408 | 0.965696331 |
| plin3 | 0.03333170434 | 0.03195589238 | 1.043053467 | 0.2969235304 | 1.033893429 |
| dnajb8 | -0.03261633522 | 0.03130507434 | -1.041886528 | 0.2974642908 | 0.9679098413 |
| b3gat3 | 0.03521158032 | 0.03380616648 | 1.041572707 | 0.2976098281 | 1.035838849 |
| elac1 | 0.03465618176 | 0.03328512701 | 1.041191213 | 0.2977868131 | 1.035263705 |
| gfra3 | 0.03332472038 | 0.03208325593 | 1.038695089 | 0.2989465658 | 1.033886209 |
| ndufb7 | 0.03430946675 | 0.03306666824 | 1.03758463 | 0.2994634764 | 1.034904826 |
| plb1 | 0.0370953989 | 0.03575736925 | 1.03741969 | 0.2995403061 | 1.03779202 |
| oplah | 0.03491308643 | 0.03365381854 | 1.037418277 | 0.2995409642 | 1.035529703 |
| cd55 | 0.03372776208 | 0.03252624097 | 1.036940055 | 0.2997637952 | 1.034302992 |
| lrp11 | 0.03337881164 | 0.03223227926 | 1.035570937 | 0.3004023565 | 1.033942134 |
| tinagl1 | 0.03363961894 | 0.03254498948 | 1.033634347 | 0.301307136 | 1.034211829 |
| ca7 | -0.03425036956 | 0.03314246717 | -1.033428483 | 0.3014034229 | 0.9663295349 |
| rhoc | 0.03229286206 | 0.03131433537 | 1.031248522 | 0.3024242919 | 1.032819935 |
| b4galt1 | 0.03314894081 | 0.03220990776 | 1.029153547 | 0.3034075273 | 1.033704489 |
| ahsa1 | 0.03405332637 | 0.03320378665 | 1.025585628 | 0.3050869449 | 1.034639779 |
| eln | -0.04479928769 | 0.04368635277 | -1.025475574 | 0.305138845 | 0.9561893816 |
| gipc3 | 0.03402353786 | 0.03322008311 | 1.024185814 | 0.305747518 | 1.034608959 |
| cilp | -0.03543770841 | 0.03463273647 | -1.023243094 | 0.3061929223 | 0.9651828551 |
| nos2 | 0.03487054349 | 0.03408944117 | 1.022913321 | 0.3063488305 | 1.03548565 |
| il17d | 0.03584336461 | 0.03505049029 | 1.022620919 | 0.3064871147 | 1.036493482 |
| tnfrsf9 | 0.03301919831 | 0.03230862728 | 1.021993229 | 0.3067841052 | 1.033570382 |
| psapl1 | -0.03429494253 | 0.03356834915 | -1.021645193 | 0.3069488599 | 0.9662864636 |
| col2a1 | 0.03450209967 | 0.03378285021 | 1.021290372 | 0.3071168865 | 1.035104202 |
| pik3ip1 | 0.03415410328 | 0.03348524812 | 1.019974622 | 0.3077404969 | 1.034744052 |
| spink1 | 0.03274848198 | 0.03211387075 | 1.019761281 | 0.3078416903 | 1.033290615 |
| skap1 | 0.03207943507 | 0.03146103774 | 1.019655974 | 0.3078916486 | 1.032599527 |
| bcl2l1 | 0.03374701336 | 0.03312046377 | 1.018917295 | 0.3082422321 | 1.034322904 |
| myl6b | -0.03413398307 | 0.03350417126 | -1.018798012 | 0.3082988698 | 0.9664420091 |
| eif5 | 0.0343667833 | 0.03377595375 | 1.017492609 | 0.3089191465 | 1.034964145 |
| fkbp5 | 0.03178433546 | 0.0312395249 | 1.017439784 | 0.3089442644 | 1.032294852 |
| c1qtnf9 | -0.03399470788 | 0.03344248818 | -1.016512519 | 0.3093853892 | 0.9665766199 |
| inpp5j | 0.03413282885 | 0.03359966398 | 1.015868161 | 0.3096921725 | 1.034722039 |
| psip1 | 0.03267273488 | 0.03216301635 | 1.01584797 | 0.3097017885 | 1.03321235 |
| tarm1 | -0.03396809108 | 0.03344228521 | -1.015722785 | 0.3097614145 | 0.9666023474 |
| tff3 | 0.03429140315 | 0.03377232282 | 1.015369992 | 0.3099294918 | 1.034886132 |
| igfbp7 | 0.03385732864 | 0.03336004225 | 1.014906647 | 0.3101503292 | 1.034437012 |
| pnpt1 | 0.03207845096 | 0.0316084991 | 1.014867896 | 0.3101688036 | 1.03259851 |
| tada3 | 0.03399222014 | 0.03351376685 | 1.014276321 | 0.3104509197 | 1.034576558 |
| tek | 0.0319373437 | 0.03149688443 | 1.013984217 | 0.3105902834 | 1.032452814 |
| pdgfc | -0.03237806099 | 0.03195408591 | -1.013268259 | 0.3109320444 | 0.9681404967 |
| mfap3 | 0.03183025565 | 0.03142623238 | 1.012856243 | 0.3111288317 | 1.032342256 |
| ccar2 | 0.03354188483 | 0.03314277956 | 1.012041998 | 0.3115179734 | 1.034110756 |
| pibf1 | 0.03386591249 | 0.03347211095 | 1.011765064 | 0.3116503977 | 1.034445891 |
| dlg4 | 0.03336595075 | 0.03298703271 | 1.011486878 | 0.3117834582 | 1.033928837 |
| bpifb1 | 0.0335620937 | 0.03318139569 | 1.011473237 | 0.3117899842 | 1.034131655 |
| abl1 | 0.03203512684 | 0.0316771672 | 1.011300241 | 0.3118727506 | 1.032553775 |
| pcdh9 | -0.03430423732 | 0.03392549564 | -1.011163925 | 0.3119379789 | 0.9662774822 |
| gipc2 | 0.03582915976 | 0.03544619375 | 1.010804151 | 0.3121101762 | 1.036478759 |
| dcun1d1 | -0.03392194543 | 0.03356016232 | -1.010780136 | 0.3121216725 | 0.9666469529 |
| treh | -0.03443722612 | 0.03408089296 | -1.010455511 | 0.3122771038 | 0.9661489867 |
| hspg2 | 0.03483118372 | 0.03449014154 | 1.009888106 | 0.3125489016 | 1.035444894 |
| gpc5 | -0.03174041545 | 0.03145603789 | -1.009040476 | 0.3129552228 | 0.9687580241 |
| chp1 | -0.03385722068 | 0.0335696881 | -1.008565244 | 0.3131831827 | 0.9667095209 |
| bche | 0.03539741265 | 0.03512022068 | 1.00789266 | 0.3135059956 | 1.036031359 |
| dhodh | -0.0340898103 | 0.03386379671 | -1.006674195 | 0.3140913666 | 0.9664847005 |
| nxpe4 | -0.03352795222 | 0.0333223268 | -1.0061708 | 0.3143334158 | 0.9670278803 |
| ebag9 | 0.03120913225 | 0.03105299531 | 1.00502808 | 0.3148833291 | 1.031701243 |

| Protein | beta | se | z | p_val | HR |
|---|---|---|---|---|---|
| tpk1 | -0.03385298154 | 0.03369224377 | -1.004770765 | 0.3150072444 | 0.9667136189 |
| dctn6 | 0.03164304189 | 0.03149359207 | 1.004745404 | 0.3150194591 | 1.032149006 |
| kiaa1549l | -0.03347445574 | 0.0333383668 | -1.004082052 | 0.3153390659 | 0.9670796143 |
| il21r | 0.03350238729 | 0.03336796899 | 1.004028363 | 0.3153649425 | 1.034069912 |
| metap2 | 0.03127927543 | 0.03120500972 | 1.002379929 | 0.3161601321 | 1.031773613 |
| procr | -0.03376947606 | 0.03370893243 | -1.001796071 | 0.3164420953 | 0.9667943482 |
| nt5e | 0.03156690851 | 0.03151913027 | 1.001515849 | 0.3165774817 | 1.032070428 |
| ppp1r12a | 0.0311398591 | 0.03109981147 | 1.001287713 | 0.3166877316 | 1.031629777 |
| gcc1 | 0.03334388981 | 0.03331120801 | 1.000981105 | 0.3168359434 | 1.033906028 |
| msln | -0.03256241511 | 0.0325545405 | -1.00024189 | 0.3171934615 | 0.9679620325 |
| scarb2 | 0.03459722476 | 0.03459323291 | 1.000115394 | 0.3172546671 | 1.035202671 |
| fyb1 | 0.03124856405 | 0.03124891463 | 0.999988781 | 0.3173159372 | 1.031741926 |
| kctd5 | 0.03522276278 | 0.03525134841 | 0.9991890911 | 0.3177030994 | 1.035850432 |
| znf174 | 0.03375614331 | 0.03380856474 | 0.9984494633 | 0.3180614586 | 1.034332347 |
| col24a1 | -0.03349834024 | 0.03355090825 | -0.9984331867 | 0.3180693478 | 0.9670565163 |
| atp6v1g1 | 0.0339477002 | 0.03400765235 | 0.9982370982 | 0.3181644011 | 1.0345305 |
| arsa | 0.0323026685 | 0.03236463727 | 0.9980852938 | 0.3182380007 | 1.032830063 |
| atxn2 | -0.03347988783 | 0.03356183858 | -0.9975582162 | 0.318493631 | 0.967074361 |
| extl1 | -0.03384696346 | 0.03396577283 | -0.9965020853 | 0.3190062544 | 0.9667194367 |
| il17a | 0.03104442692 | 0.03119002559 | 0.9953318836 | 0.3195748757 | 1.031531331 |
| eif4g3 | 0.0330004138 | 0.03316711486 | 0.9949739052 | 0.319748956 | 1.033550967 |
| slmap | 0.03354729837 | 0.03373998446 | 0.9942890878 | 0.3200821467 | 1.034116355 |
| hgfac | -0.03363292098 | 0.03385058829 | -0.9935697629 | 0.3204323711 | 0.9669263779 |
| slc39a5 | -0.03331788269 | 0.03355278615 | -0.9929989879 | 0.3207104477 | 0.9672310447 |
| fuca1 | 0.03051125708 | 0.03073671197 | 0.9926649637 | 0.3208732545 | 1.030981496 |
| reps1 | 0.03333257818 | 0.03359260146 | 0.9922595074 | 0.3210709507 | 1.033894333 |
| vsig4 | 0.03425080806 | 0.03454932195 | 0.9913597758 | 0.3215099342 | 1.034844121 |
| bcan | -0.03307661905 | 0.03338009395 | -0.9909085067 | 0.3217302582 | 0.9674644305 |
| pak4 | 0.03112829983 | 0.03146158911 | 0.9894064702 | 0.3224643102 | 1.031617852 |
| adh1b | 0.03629138527 | 0.03670040136 | 0.9888552693 | 0.3227339583 | 1.036957957 |
| gngt1 | 0.03314954958 | 0.03352674936 | 0.9887492888 | 0.322785821 | 1.033705118 |
| znf830 | 0.0327528083 | 0.03312775589 | 0.9886817692 | 0.3228188652 | 1.033295086 |
| slit2 | 0.0312263926 | 0.03163003401 | 0.9872386667 | 0.3235256503 | 1.031719051 |
| rnase1 | -0.03570065157 | 0.03617900115 | -0.9867782534 | 0.3237513578 | 0.9649291003 |
| aamdc | 0.03326605863 | 0.0337244989 | 0.9864063134 | 0.3239337682 | 1.033825561 |
| cep85 | 0.03142647301 | 0.03186716956 | 0.9861708286 | 0.3240492916 | 1.031925498 |
| cert | 0.03106018721 | 0.03151105449 | 0.9856917744 | 0.3242843871 | 1.031547588 |
| s100a16 | -0.03126136684 | 0.03173673091 | -0.9850216436 | 0.3246134397 | 0.9692222174 |
| crkl | 0.0308422614 | 0.03132605922 | 0.9845560589 | 0.3248421825 | 1.031322812 |
| b2m | -0.03512066485 | 0.03568644378 | -0.9841458304 | 0.3250438156 | 0.9654889087 |
| mgmt | 0.03079699582 | 0.03129939854 | 0.9839484866 | 0.3251408419 | 1.031276129 |
| sult1a1 | 0.03023379561 | 0.03073300712 | 0.9837565032 | 0.3252352507 | 1.030695478 |
| ereg | 0.03087602751 | 0.03141637672 | 0.9828003971 | 0.3257056864 | 1.031357636 |
| tmed8 | 0.03260626814 | 0.03318819961 | 0.9824657114 | 0.3258704673 | 1.033143678 |
| numb | 0.03350855713 | 0.03421255886 | 0.9794227102 | 0.3273711604 | 1.034076292 |
| plcb2 | 0.03279490033 | 0.03349389177 | 0.9791307787 | 0.3275153656 | 1.03333858 |
| prkd2 | 0.03265043764 | 0.03335059052 | 0.9790062823 | 0.3275768754 | 1.033189312 |
| calca | 0.03913009433 | 0.04000489527 | 0.9781326527 | 0.3280087201 | 1.039905761 |
| pof1b | 0.03399263824 | 0.03479826182 | 0.9768487408 | 0.3286440418 | 1.03457699 |
| zp4 | 0.03262743709 | 0.03346018986 | 0.9751121323 | 0.3295046412 | 1.033165548 |
| ncam1 | 0.03254095359 | 0.03349412314 | 0.9715421853 | 0.3312783567 | 1.0330762 |
| cyb5a | 0.03275465904 | 0.03371863394 | 0.9714112115 | 0.3313435477 | 1.033296998 |
| lrrfip1 | 0.03254054535 | 0.03350157576 | 0.9713138743 | 0.3313920019 | 1.033075779 |
| hhex | 0.03329725426 | 0.03428746548 | 0.9711203145 | 0.3314883689 | 1.033857812 |
| sod3 | -0.0354205943 | 0.03650012258 | -0.970423982 | 0.3318351995 | 0.9651993735 |
| ecm1 | -0.03251640232 | 0.03352526514 | -0.9699073872 | 0.3320926575 | 0.9680065721 |
| lrrn1 | 0.03152682457 | 0.03257074549 | 0.967949124 | 0.3330697789 | 1.032029059 |
| hexim1 | 0.03015832695 | 0.03119566672 | 0.966747312 | 0.3336703693 | 1.030617696 |
| leo1 | 0.03204157971 | 0.03317445195 | 0.9658510637 | 0.3341187125 | 1.032560438 |
| apoa4 | -0.03264911883 | 0.03384566854 | -0.9646468879 | 0.334721706 | 0.9678781102 |
| cfc1 | 0.03150556359 | 0.03270935378 | 0.9631973716 | 0.3354484839 | 1.032007117 |
| relt | 0.033735889 | 0.03503775044 | 0.9628440346 | 0.3356257987 | 1.034311398 |
| gpnmb | 0.03083618433 | 0.03203582306 | 0.9625532104 | 0.3357717879 | 1.031316544 |
| flt4 | 0.03106667309 | 0.03228187186 | 0.96235662 | 0.3358704965 | 1.031554279 |
| gad2 | 0.03231140945 | 0.03360087677 | 0.9616239978 | 0.3362385124 | 1.032839091 |
| ccn3 | 0.03345081127 | 0.03478700436 | 0.961589303 | 0.3362559469 | 1.034016581 |
| ccl16 | 0.03220106527 | 0.03351266631 | 0.9608625277 | 0.3366212942 | 1.03272513 |
| pithd1 | 0.03227821344 | 0.03360560566 | 0.9605008689 | 0.3368031939 | 1.032804806 |
| cyb5r2 | 0.03267992092 | 0.03402413478 | 0.9604923426 | 0.336807483 | 1.033219774 |
| gja8 | -0.03240357189 | 0.03376600895 | -0.9596506338 | 0.3372310765 | 0.9681157989 |

| Protein | beta | se | z | p_val | HR |
|---|---|---|---|---|---|
| serpini2 | 0.03169151172 | 0.03310926063 | 0.9571796867 | 0.3384765681 | 1.032199035 |
| prtfdc1 | 0.03007678549 | 0.03142519826 | 0.9570913521 | 0.3385211481 | 1.030533661 |
| krt8 | 0.03251198943 | 0.03402897433 | 0.9554207868 | 0.3393649462 | 1.033046279 |
| ccl23 | 0.03043125357 | 0.03186562636 | 0.9549868319 | 0.3395843561 | 1.030899017 |
| cd200r1 | 0.0297042681 | 0.03110678593 | 0.9549128015 | 0.3396217954 | 1.030149841 |
| crip2 | 0.03287843831 | 0.03444262786 | 0.9545856503 | 0.3397872767 | 1.033424907 |
| acp1 | 0.03263671288 | 0.03419943764 | 0.954305542 | 0.3399290036 | 1.033175132 |
| appl2 | 0.03177941135 | 0.03334768849 | 0.9529719385 | 0.3406042888 | 1.032289769 |
| grpel1 | 0.03067852598 | 0.03220211976 | 0.9526865375 | 0.3407489164 | 1.031153961 |
| cd14 | 0.03121851752 | 0.03277014143 | 0.9526512905 | 0.3407667806 | 1.031710926 |
| adam22 | -0.0296409186 | 0.03116245345 | -0.9511740998 | 0.341516004 | 0.9707940651 |
| mitd1 | 0.02960657493 | 0.03121200447 | 0.9485637157 | 0.3428425526 | 1.030049207 |
| yars1 | 0.03168793086 | 0.03342817562 | 0.9479407796 | 0.3431596032 | 1.032195339 |
| sel1l | 0.03374270735 | 0.03563920761 | 0.9467861271 | 0.3437477723 | 1.03431845 |
| tcof1 | 0.03249117128 | 0.03435281382 | 0.9458081496 | 0.3442464481 | 1.033024773 |
| samd9l | 0.02987813266 | 0.03159747677 | 0.9455860313 | 0.3443597717 | 1.030328963 |
| cirbp | 0.03146642072 | 0.03328548015 | 0.9453497615 | 0.3444803414 | 1.031966722 |
| bloc1s3 | 0.03147651515 | 0.03333600993 | 0.9442196358 | 0.3450574231 | 1.031977139 |
| crhr1 | 0.02980533851 | 0.03158458187 | 0.943667345 | 0.3453396662 | 1.030253964 |
| adra2a | 0.03190964037 | 0.03381641469 | 0.9436139419 | 0.3453669651 | 1.032424212 |
| ifit3 | 0.03148990392 | 0.03340683576 | 0.9426185752 | 0.3458760352 | 1.031990956 |
| vim | 0.02986670569 | 0.03170459579 | 0.9420307985 | 0.346176872 | 1.030317189 |
| lepr | -0.03165775914 | 0.03361711945 | -0.9417154015 | 0.3463383676 | 0.9688381013 |
| egf | 0.02920363992 | 0.0310681521 | 0.939986383 | 0.3472245454 | 1.029634248 |
| suox | 0.03276373669 | 0.0348864708 | 0.9391530854 | 0.3476521521 | 1.033306378 |
| f3 | -0.03095990548 | 0.03300341452 | -0.9380818904 | 0.3482023277 | 0.9695144445 |
| ywhaq | 0.03128736944 | 0.03336909422 | 0.9376151845 | 0.3484422051 | 1.031781964 |
| pdrg1 | 0.03138072077 | 0.03349874219 | 0.9367731061 | 0.348875282 | 1.031878287 |
| pigr | 0.0312411671 | 0.03335962721 | 0.9364962894 | 0.3490177222 | 1.031734294 |
| serpina9 | -0.03000081048 | 0.03210857407 | -0.9343551168 | 0.3501207419 | 0.970444747 |
| il36a | -0.031170229 | 0.03338400726 | -0.9336874615 | 0.3504651346 | 0.9693105543 |
| per3 | 0.03096863794 | 0.03319289609 | 0.9329899344 | 0.3508251654 | 1.031453155 |
| fcrlb | -0.03044937347 | 0.03265418078 | -0.9324800912 | 0.3510884708 | 0.970009539 |
| nfat5 | 0.03111218499 | 0.03337497838 | 0.9322009034 | 0.3512327087 | 1.031601228 |
| bsnd | 0.03163430551 | 0.03393538175 | 0.9321924163 | 0.351237094 | 1.032139988 |
| tp53bp1 | 0.03183043006 | 0.03415824508 | 0.9318520312 | 0.3514130007 | 1.032342436 |
| ptprz1 | 0.03487039137 | 0.03745009142 | 0.9311163219 | 0.3517933965 | 1.035485492 |
| kiaa1549 | 0.03044486976 | 0.03269863402 | 0.9310746662 | 0.3518149422 | 1.030913054 |
| dusp3 | 0.02919465973 | 0.03137069911 | 0.9306346548 | 0.3520425819 | 1.029625001 |
| fzd8 | 0.03127189441 | 0.03362933014 | 0.9298994146 | 0.3524231661 | 1.031765997 |
| vamp8 | 0.03122616365 | 0.03361972359 | 0.9288048894 | 0.3529902101 | 1.031718815 |
| gsr | 0.0317856548 | 0.03423843366 | 0.9283618261 | 0.3532199133 | 1.032296214 |
| pcdh17 | 0.02940781848 | 0.03170938646 | 0.9274168238 | 0.353710159 | 1.029844498 |
| ambn | 0.02942574789 | 0.03175227275 | 0.9267288714 | 0.3540673234 | 1.029862963 |
| ggt5 | 0.02901880055 | 0.03134419783 | 0.9258109175 | 0.3545442525 | 1.029443948 |
| bglap | -0.03050321138 | 0.03294810513 | -0.9257956188 | 0.3545522045 | 0.9699573172 |
| matn2 | -0.03010796797 | 0.03252355312 | -0.9257281289 | 0.3545872858 | 0.9703407622 |
| dok2 | 0.02886675736 | 0.03132334292 | 0.9215733275 | 0.3567511765 | 1.02928744 |
| epo | -0.02976583128 | 0.03233234638 | -0.9206208214 | 0.3572484276 | 0.9706728081 |
| ttn | -0.03206012815 | 0.03483176935 | -0.9204277805 | 0.3573492568 | 0.9684483493 |
| sema6c | 0.03123659475 | 0.03395055234 | 0.9200614599 | 0.3575406429 | 1.031729577 |
| mprip | 0.03089127692 | 0.03357642327 | 0.9200288154 | 0.3575577013 | 1.031373364 |
| ephx2 | -0.02936468275 | 0.03194621293 | -0.9191913549 | 0.3579954922 | 0.9710622702 |
| pga4 | -0.03003839949 | 0.03269888815 | -0.9186367239 | 0.3582856167 | 0.9704082697 |
| f2r | 0.02908247904 | 0.03174730589 | 0.9160613232 | 0.3596347321 | 1.029509504 |
| nfx1 | 0.03087716053 | 0.03371753735 | 0.9157596598 | 0.359792966 | 1.031358805 |
| prkar1a | 0.02856054912 | 0.03124188901 | 0.9141748476 | 0.3606249784 | 1.028972312 |
| twf2 | 0.03039146364 | 0.03328047283 | 0.9131920629 | 0.3611415376 | 1.030857998 |
| cntn1 | 0.03021544681 | 0.03310722473 | 0.9126541731 | 0.3614244531 | 1.030676566 |
| man1a2 | -0.0327587287 | 0.03590681162 | -0.9123263029 | 0.361596972 | 0.967772027 |
| serpinb5 | 0.02867783489 | 0.03147304075 | 0.9111872957 | 0.3621966967 | 1.029093003 |
| nfu1 | 0.03015281027 | 0.03310645854 | 0.9107833213 | 0.3624095521 | 1.03061201 |
| btn1a1 | 0.03028981625 | 0.03333899784 | 0.9085400946 | 0.3635929403 | 1.03075322 |
| hnf1a | 0.03065380195 | 0.03374522647 | 0.9083892794 | 0.3636725878 | 1.031128467 |
| brap | 0.03011808266 | 0.03317563354 | 0.9078374529 | 0.3639641074 | 1.03057622 |
| pdia5 | 0.03076503808 | 0.0339030512 | 0.907441572 | 0.3641733339 | 1.031243173 |
| ube2b | 0.03001327424 | 0.03307465838 | 0.9074401885 | 0.3641740653 | 1.030468213 |
| igfl4 | 0.03018900354 | 0.03331022422 | 0.906298419 | 0.3647779221 | 1.030649312 |
| stk4 | 0.02836350027 | 0.03129963415 | 0.906192709 | 0.3648338614 | 1.028769574 |
| sap18 | -0.0299331059 | 0.03303594537 | -0.9060768676 | 0.3648951682 | 0.9705104528 |

| Protein | beta | se | z | p_val | HR |
|---|---|---|---|---|---|
| akt3 | 0.0290786977 | 0.0321355297 | 0.9048768753 | 0.3655306192 | 1.029505611 |
| apoh | 0.02804215414 | 0.03099473643 | 0.9047392355 | 0.3656035499 | 1.028439036 |
| pltp | -0.02986433424 | 0.03301088192 | -0.9046815022 | 0.3656341435 | 0.9705771987 |
| ido1 | 0.03108005103 | 0.03437577512 | 0.904126552 | 0.3659283005 | 1.031568079 |
| sema3g | -0.03029506608 | 0.03353143235 | -0.9034826119 | 0.3662698126 | 0.9701592302 |
| serpinb6 | 0.02846971537 | 0.03151115255 | 0.9034806112 | 0.366270874 | 1.028878851 |
| pon1 | -0.03025652207 | 0.03359876845 | -0.9005247353 | 0.367841068 | 0.9701966248 |
| ppp1r14d | 0.03051097307 | 0.03389212275 | 0.9002378899 | 0.3679936662 | 1.030981203 |
| fam3c | 0.02945227177 | 0.03272038876 | 0.9001198606 | 0.3680564678 | 1.029890279 |
| snx2 | 0.02938191685 | 0.03265292441 | 0.8998249736 | 0.3682134019 | 1.029817824 |
| cst7 | 0.02890637123 | 0.03213079428 | 0.899646955 | 0.3683081607 | 1.029328215 |
| dctn1 | 0.02801114081 | 0.03114461148 | 0.8993896366 | 0.3684451573 | 1.028407142 |
| pnma1 | 0.03010679808 | 0.03353342851 | 0.8978144919 | 0.3692844573 | 1.03056459 |
| mydgf | 0.03004008168 | 0.03346655215 | 0.8976150739 | 0.3693907999 | 1.030495837 |
| ocln | 0.03232448752 | 0.03605755386 | 0.8964692292 | 0.3700022074 | 1.032852599 |
| inhbc | 0.03085708318 | 0.03443232252 | 0.8961661871 | 0.3701640117 | 1.031338098 |
| mars1 | 0.03003270885 | 0.03351795564 | 0.8960185154 | 0.3702428745 | 1.030488239 |
| sema3f | 0.02996043762 | 0.03347780594 | 0.8949343236 | 0.3708221972 | 1.030413768 |
| mn1 | 0.02985129657 | 0.03348123016 | 0.891583028 | 0.3726164704 | 1.030301313 |
| lmod1 | -0.03406479692 | 0.03824356629 | -0.8907327485 | 0.3730725616 | 0.9665088758 |
| celsr2 | 0.02963739517 | 0.03330380735 | 0.8899101194 | 0.3735141498 | 1.030080954 |
| epgn | 0.02928636384 | 0.03293470705 | 0.8892249686 | 0.3738821865 | 1.029719427 |
| tcn1 | 0.02992943582 | 0.03366933085 | 0.8889227991 | 0.3740445715 | 1.030381823 |
| nub1 | 0.02791070907 | 0.03143392454 | 0.8879167804 | 0.3745855176 | 1.028303862 |
| fgf23 | -0.02980267959 | 0.03356523928 | -0.8879030875 | 0.3745928838 | 0.9706370412 |
| ccn4 | 0.0287832608 | 0.03246307286 | 0.8866462187 | 0.375269402 | 1.029201502 |
| cfi | 0.03326764211 | 0.03754643788 | 0.8860399012 | 0.3755960262 | 1.033827198 |
| ccl22 | 0.02881639658 | 0.03254128834 | 0.8855333654 | 0.3758690324 | 1.029235606 |
| dctpp1 | 0.02848557491 | 0.03224025479 | 0.8835406262 | 0.3769442424 | 1.028895169 |
| clic5 | 0.02941317421 | 0.03330237726 | 0.8832154526 | 0.3771198742 | 1.029850014 |
| pde1c | 0.02935002147 | 0.0332353996 | 0.8830951883 | 0.3771848437 | 1.029784978 |
| kazn | 0.02906733323 | 0.03294661805 | 0.8822554469 | 0.3776386837 | 1.029493911 |
| il10 | -0.02753874713 | 0.03123035579 | -0.8817942171 | 0.3778880995 | 0.9728369872 |
| timm10 | 0.02900233523 | 0.03290477894 | 0.8814019166 | 0.3781003207 | 1.029426998 |
| mapt | -0.02786139001 | 0.03161056643 | -0.8813948357 | 0.3781041519 | 0.9725231589 |
| itprip | 0.03024958098 | 0.03433856814 | 0.8809214424 | 0.3783603409 | 1.030711748 |
| ecscr | -0.02932789588 | 0.03329851572 | -0.8807568519 | 0.3784494383 | 0.9710979932 |
| med21 | -0.02969135282 | 0.03371734911 | -0.8805957051 | 0.3785366841 | 0.9707451051 |
| lat2 | 0.02743926615 | 0.03116231556 | 0.8805271899 | 0.3785737823 | 1.02781919 |
| ddc | -0.028206027 | 0.03207222723 | -0.8794533289 | 0.3791555278 | 0.9721880492 |
| rab27b | 0.02881743089 | 0.03283096057 | 0.877751683 | 0.3800784904 | 1.029236671 |
| arf6 | 0.02924144427 | 0.03331422748 | 0.8777464308 | 0.3800813412 | 1.029673173 |
| psrc1 | -0.02778014121 | 0.03166276564 | -0.8773757014 | 0.3802826066 | 0.9726021784 |
| il36g | 0.02969001543 | 0.03384377525 | 0.8772666528 | 0.3803418205 | 1.030135158 |
| stx1b | 0.02949446027 | 0.03365629604 | 0.8763430247 | 0.3808435819 | 1.02993373 |
| pnma2 | -0.02910256801 | 0.0332276776 | -0.8758532079 | 0.3811098401 | 0.9713168333 |
| tex101 | 0.04256147305 | 0.04861035614 | 0.8755639011 | 0.3812671572 | 1.0434802 |
| reg1b | 0.02804271827 | 0.032037387 | 0.8753122803 | 0.3814040141 | 1.028439617 |
| fcn2 | 0.02812388007 | 0.03214278633 | 0.8749670853 | 0.3815918152 | 1.02852309 |
| lmod2 | 0.02911171742 | 0.03336016805 | 0.8726490039 | 0.3828544216 | 1.029539606 |
| serpina4 | 0.02952562715 | 0.03384950636 | 0.8722616759 | 0.3830656398 | 1.02996583 |
| crhbp | 0.02852783402 | 0.03271967889 | 0.8718861243 | 0.3832705041 | 1.02893865 |
| ftcd | 0.03244779335 | 0.03722106518 | 0.8717588599 | 0.3833399424 | 1.032979963 |
| palm3 | 0.02930188074 | 0.03362329219 | 0.8714756597 | 0.3834944904 | 1.029735405 |
| ddx53 | -0.02890104358 | 0.03317320484 | -0.8712165052 | 0.3836359495 | 0.9715125971 |
| ahnak2 | -0.03039123565 | 0.03491058782 | -0.870544942 | 0.3840026701 | 0.9700659349 |
| efnb2 | -0.02938080833 | 0.03375457977 | -0.8704243553 | 0.3840685415 | 0.9710466114 |
| mki67 | 0.02946284329 | 0.03385559578 | 0.8702503268 | 0.3841636183 | 1.029901167 |
| mink1 | 0.02893408589 | 0.03327610242 | 0.8695154717 | 0.3845652493 | 1.029356743 |
| irak4 | 0.02699802338 | 0.03108374615 | 0.8685575814 | 0.3850891644 | 1.027365772 |
| ccl17 | 0.02733855159 | 0.03148460645 | 0.8683148582 | 0.3852219904 | 1.027715679 |
| klf4 | 0.03002565549 | 0.03458566417 | 0.8681532134 | 0.3853104631 | 1.030480971 |
| lgals3 | 0.02872614054 | 0.03309889653 | 0.8678881639 | 0.3854555591 | 1.029142715 |
| shh | 0.02935513795 | 0.0338270451 | 0.8676897262 | 0.3855642116 | 1.029786377 |
| ltbp3 | 0.02977798377 | 0.0343194584 | 0.8676705624 | 0.3855747056 | 1.030225782 |
| serpina1 | 0.02894449593 | 0.03337389731 | 0.8672794687 | 0.3857889033 | 1.029367459 |
| gstm4 | 0.02885470188 | 0.03330265717 | 0.8664384265 | 0.3862497789 | 1.029275032 |
| ptprh | 0.02909171015 | 0.03359350031 | 0.8659922272 | 0.3864944243 | 1.029519007 |
| galnt5 | -0.0290495576 | 0.03355996429 | -0.8656015649 | 0.3867086971 | 0.9713683246 |
| gzma | 0.02727008902 | 0.03151410599 | 0.8653296092 | 0.3868579038 | 1.027645321 |

| Protein | beta | se | z | p_val | HR |
|---|---|---|---|---|---|
| txndc9 | 0.02881784074 | 0.03332911101 | 0.8646447465 | 0.3872338047 | 1.029237092 |
| smad5 | 0.0282818624 | 0.03270967763 | 0.8646328685 | 0.3872403262 | 1.028685591 |
| erbin | 0.02696315462 | 0.03121302728 | 0.8638429837 | 0.3876741516 | 1.02732995 |
| nos1 | -0.03008134985 | 0.03483254418 | -0.8635989865 | 0.3878082211 | 0.9703665912 |
| susd5 | -0.03185593391 | 0.0368979521 | -0.8633523568 | 0.3879437658 | 0.9686461211 |
| doc2b | 0.02918899779 | 0.03384054613 | 0.862545116 | 0.3883876175 | 1.029619172 |
| sftpa2 | -0.02792304146 | 0.03238740495 | -0.862157419 | 0.3886008979 | 0.9724632033 |
| ca6 | 0.02951872323 | 0.03433686321 | 0.8596802523 | 0.3899653233 | 1.029958719 |
| dcbld2 | 0.02763858654 | 0.03218472349 | 0.8587486096 | 0.3904792257 | 1.028024076 |
| fam171a2 | 0.02810284219 | 0.03274441166 | 0.8582484998 | 0.3907552604 | 1.028501452 |
| smoc1 | 0.02899980365 | 0.03379847782 | 0.8580209974 | 0.3908808691 | 1.029424392 |
| fcer1a | -0.02854496382 | 0.03328233227 | -0.8576611636 | 0.3910795908 | 0.9718585947 |
| tcl1a | 0.02679429172 | 0.03125708407 | 0.8572230109 | 0.3913216476 | 1.027156486 |
| mesd | 0.02665823456 | 0.03110399294 | 0.8570679206 | 0.3914073488 | 1.027016744 |
| pomc | 0.03021048199 | 0.03525271528 | 0.8569689384 | 0.3914620513 | 1.030671449 |
| itgav | -0.02844691606 | 0.03320556965 | -0.8566911019 | 0.3916156222 | 0.9719538879 |
| lztfl1 | 0.0288308724 | 0.03368223895 | 0.8559666252 | 0.3920162403 | 1.029250505 |
| lilrb1 | 0.02713993426 | 0.03175507059 | 0.8546645858 | 0.3927368613 | 1.027511577 |
| yes1 | 0.02651989101 | 0.03103219445 | 0.8545928341 | 0.392776596 | 1.026874673 |
| mertk | 0.02805357083 | 0.03287617686 | 0.8533100107 | 0.3934874101 | 1.028450778 |
| pdgfb | 0.02660760119 | 0.03119710183 | 0.8528869551 | 0.3937219964 | 1.026964744 |
| atp2b4 | -0.02875000509 | 0.03372348334 | -0.852521811 | 0.3939245386 | 0.971659344 |
| stxbp1 | 0.0285295343 | 0.03347226533 | 0.8523335372 | 0.3940289971 | 1.028940399 |
| cend1 | -0.0290558935 | 0.03412990645 | -0.851332351 | 0.3945847588 | 0.9713621701 |
| lsm1 | -0.02675448439 | 0.03143413457 | -0.8511283913 | 0.3946980357 | 0.9736002462 |
| plaur | 0.02951856894 | 0.03468295785 | 0.8510972182 | 0.3947153506 | 1.029958561 |
| fgf2 | 0.02632020538 | 0.03098340357 | 0.8494936757 | 0.3956066478 | 1.026669641 |
| triap1 | 0.02688263177 | 0.03168617546 | 0.8484025408 | 0.3962138282 | 1.027247229 |
| ca14 | 0.02914032737 | 0.03436277443 | 0.8480202154 | 0.3964267126 | 1.029569061 |
| insl4 | -0.02862519227 | 0.0337764673 | -0.8474892299 | 0.3967224876 | 0.9717806271 |
| asgr2 | -0.02868370334 | 0.03387062919 | -0.8468606584 | 0.3970727932 | 0.9717237688 |
| ssna1 | 0.02820939813 | 0.03332369952 | 0.8465266021 | 0.3972590401 | 1.028611051 |
| iqgap2 | 0.0266842181 | 0.03155902247 | 0.8455337337 | 0.3978129063 | 1.02704343 |
| prr5 | -0.02833697101 | 0.03355072177 | -0.844600936 | 0.3983336863 | 0.9720607553 |
| sugp1 | -0.02846497958 | 0.03370786544 | -0.8444610541 | 0.3984118176 | 0.9719363312 |
| pla2g15 | 0.02708497092 | 0.03208898773 | 0.8440581282 | 0.3986369242 | 1.027455103 |
| chek2 | 0.02687316854 | 0.03190163858 | 0.8423758069 | 0.3995776306 | 1.027237508 |
| plat | 0.03043114747 | 0.03615432535 | 0.8417014335 | 0.3999550956 | 1.030898908 |
| klk8 | 0.02767283546 | 0.03291636315 | 0.8407014874 | 0.4005151871 | 1.028059285 |
| dnajb14 | 0.02829312214 | 0.03366319104 | 0.8404765343 | 0.4006412531 | 1.028697174 |
| lat | 0.02612418722 | 0.03108392459 | 0.8404404385 | 0.4006614838 | 1.026468415 |
| ehd3 | 0.02814891565 | 0.03350572731 | 0.8401225076 | 0.4008397016 | 1.02854884 |
| cspg4 | -0.029973973 | 0.03570186296 | -0.8395632752 | 0.4011532976 | 0.9704707917 |
| fxyd5 | 0.02646455686 | 0.03154552327 | 0.8389322515 | 0.4015073283 | 1.026817853 |
| tpp1 | 0.02773926005 | 0.03307143665 | 0.8387679177 | 0.4015995573 | 1.028127576 |
| tpsd1 | 0.02826179326 | 0.0337359291 | 0.8377357318 | 0.4021791407 | 1.028664947 |
| glrx5 | 0.02817443414 | 0.03363433101 | 0.8376689319 | 0.4022166668 | 1.028575087 |
| lrfn2 | -0.02784549978 | 0.03325878531 | -0.8372374253 | 0.4024591246 | 0.9725386126 |
| tfpi2 | 0.02802537191 | 0.03350955928 | 0.8363396151 | 0.4029638729 | 1.028421777 |
| ube2l6 | 0.02802188187 | 0.03352738791 | 0.8357907852 | 0.4032726113 | 1.028418188 |
| nid1 | 0.02664199693 | 0.03196893715 | 0.8333713694 | 0.4046353166 | 1.027000068 |
| furin | 0.02969762864 | 0.03566122428 | 0.8327708665 | 0.4049739684 | 1.030143001 |
| dipk2b | -0.02942930834 | 0.03534139155 | -0.8327150418 | 0.4050054592 | 0.9709995168 |
| safb2 | 0.02733450203 | 0.03284313541 | 0.8322744371 | 0.4052540561 | 1.027711517 |
| cd248 | -0.02848567579 | 0.03428648817 | -0.8308134576 | 0.406079019 | 0.971916216 |
| scrib | 0.02828084167 | 0.03409306451 | 0.8295189087 | 0.4068108417 | 1.028684541 |
| myl1 | -0.02819965754 | 0.03400405177 | -0.8293028646 | 0.4069330503 | 0.9721942415 |
| rgs8 | 0.02587265672 | 0.03120406632 | 0.829143755 | 0.4070230671 | 1.026210259 |
| edn1 | 0.02851076493 | 0.03441312766 | 0.8284851411 | 0.4073958065 | 1.028921087 |
| atp1b1 | 0.02780916727 | 0.03356916553 | 0.8284140172 | 0.4074360709 | 1.028199452 |
| f9 | 0.02871678802 | 0.03468998927 | 0.8278119603 | 0.4077770002 | 1.02913309 |
| il6 | 0.02901832991 | 0.03508472912 | 0.8270928874 | 0.4081844153 | 1.029443464 |
| terf1 | 0.02803218784 | 0.03390506397 | 0.8267846911 | 0.4083591085 | 1.028428787 |
| stk24 | 0.02609999554 | 0.03157074626 | 0.8267145579 | 0.408398868 | 1.026443583 |
| gnas | -0.027342645 | 0.03308027372 | -0.8265543758 | 0.4084896861 | 0.9730277813 |
| krt5 | -0.02744980296 | 0.03321497763 | -0.8264284645 | 0.4085610821 | 0.9729235192 |
| mep1b | 0.02576932946 | 0.03122008853 | 0.8254085968 | 0.4091396558 | 1.026104229 |
| kcnh2 | -0.02721575593 | 0.03298410306 | -0.8251173565 | 0.4093049668 | 0.9731512557 |
| spart | 0.02776648083 | 0.03367832507 | 0.8244614531 | 0.4096774095 | 1.028155562 |
| sorbs1 | 0.02963361086 | 0.03594545815 | 0.8244048731 | 0.4097095469 | 1.030077056 |

| Protein | beta | se | z | p_val | HR |
| --- | --- | --- | --- | --- | --- |
| rbp1 | -0.02750664654 | 0.03336600461 | -0.8243913786 | 0.409717212 | 0.9728682163 |
| nop56 | -0.0276053212 | 0.03349457316 | -0.8241729509 | 0.409841294 | 0.9727722236 |
| edil3 | -0.02826135283 | 0.03430096681 | -0.8239229228 | 0.4099833547 | 0.9721342636 |
| areg | 0.02736910228 | 0.03322726901 | 0.8236940049 | 0.4101134467 | 1.027747077 |
| sult2a1 | 0.02715280631 | 0.03301891102 | 0.8223410607 | 0.4108828137 | 1.027524803 |
| myom1 | -0.02790834626 | 0.03394635057 | -0.822130974 | 0.4110023587 | 0.9724774939 |
| chmp1a | 0.02542487922 | 0.03100786261 | 0.8199494281 | 0.4122449374 | 1.025750848 |
| siglec15 | 0.02579280115 | 0.03146850946 | 0.8196384763 | 0.4124222325 | 1.026128314 |
| dipk1c | -0.02762408058 | 0.03372054428 | -0.8192062484 | 0.4126687505 | 0.9727539752 |
| spry2 | 0.02591683665 | 0.03167548167 | 0.8181986597 | 0.4132437604 | 1.026255598 |
| adh4 | 0.02943935554 | 0.03601147843 | 0.8174992204 | 0.4136431946 | 1.029876977 |
| nagpa | -0.02792221069 | 0.03416050394 | -0.8173828682 | 0.413709663 | 0.9724640112 |
| app | 0.02559589365 | 0.03133258483 | 0.8169097375 | 0.4139800127 | 1.025926281 |
| nrgn | 0.02691602697 | 0.03296037171 | 0.8166178223 | 0.4141468669 | 1.027281535 |
| rab37 | 0.02570098829 | 0.03147592496 | 0.8165284523 | 0.4141979573 | 1.026034106 |
| epha2 | -0.02739439485 | 0.03357504182 | -0.8159154348 | 0.4145485037 | 0.9729774286 |
| fgf6 | 0.02711844551 | 0.03325282123 | 0.8155231496 | 0.4147729191 | 1.027489497 |
| casp1 | 0.02599227158 | 0.03189707219 | 0.8148795423 | 0.4151412644 | 1.026333017 |
| kel | 0.02641962658 | 0.03242201199 | 0.8148669673 | 0.4151484632 | 1.026771719 |
| cd200 | -0.02640704596 | 0.03242741304 | -0.8143432821 | 0.415448321 | 0.9739385711 |
| pdgfra | 0.02597756281 | 0.03191449947 | 0.8139736873 | 0.415660025 | 1.026317921 |
| fmr1 | -0.02571777437 | 0.03160100595 | -0.8138277121 | 0.4157436572 | 0.9746101108 |
| nrxn3 | -0.02742746571 | 0.0337514023 | -0.8126318863 | 0.4164291442 | 0.9729452519 |
| decr1 | 0.02534728818 | 0.03119191712 | 0.8126236065 | 0.4164338927 | 1.025671262 |
| ppp1r2 | 0.02547044562 | 0.03136810637 | 0.811985439 | 0.4167999867 | 1.025797589 |
| camlg | 0.02727449559 | 0.0335944746 | 0.81187445 | 0.4168636766 | 1.027649849 |
| clgn | -0.02771028729 | 0.03414106306 | -0.8116410212 | 0.4169976458 | 0.9726701209 |
| grap2 | 0.02529203072 | 0.03120763162 | 0.8104437731 | 0.4176851684 | 1.025614588 |
| wif1 | 0.02584477723 | 0.03194023762 | 0.8091604558 | 0.4184228576 | 1.026181649 |
| tbc1d23 | 0.0250343286 | 0.03108698012 | 0.8052994695 | 0.4206468878 | 1.025350319 |
| cep112 | -0.02767379512 | 0.03437126467 | -0.8051433482 | 0.4207369636 | 0.9727056164 |
| adamtsl2 | -0.02876334166 | 0.03572862209 | -0.8050504043 | 0.4207905939 | 0.9716463855 |
| shmt1 | 0.02549873313 | 0.03170803755 | 0.8041725412 | 0.4212973346 | 1.025826607 |
| cep350 | -0.02730279289 | 0.03395836965 | -0.8040077653 | 0.4213924904 | 0.9730665593 |
| amigo1 | 0.02659495623 | 0.03312286604 | 0.8029183284 | 0.4220219416 | 1.026951758 |
| il17ra | 0.02476077433 | 0.03089730121 | 0.8013895503 | 0.4229061628 | 1.025069868 |
| kcnip4 | 0.02535024469 | 0.03164078747 | 0.8011888046 | 0.4230223515 | 1.025674295 |
| ntrk3 | 0.02657737401 | 0.03322068108 | 0.8000249589 | 0.4236963366 | 1.026933702 |
| defb104a_defb104b | 0.02853643137 | 0.03570856237 | 0.7991481446 | 0.4242045159 | 1.028947496 |
| arhgap25 | 0.025581215 | 0.03206500536 | 0.7977923193 | 0.4249910192 | 1.025911222 |
| rbm19 | 0.02708003845 | 0.03399414948 | 0.7966087948 | 0.425678268 | 1.027450035 |
| itgb7 | -0.02518612534 | 0.03162521696 | -0.7963937569 | 0.4258032058 | 0.975128399 |
| espl1 | 0.02665690834 | 0.03347256925 | 0.7963807063 | 0.4258107889 | 1.027015382 |
| reg3g | -0.02632962197 | 0.03311537777 | -0.7950874714 | 0.426562622 | 0.9740139803 |
| id4 | 0.0260835174 | 0.03287198574 | 0.7934877317 | 0.4274937143 | 1.026426669 |
| thsd1 | 0.0268833401 | 0.03388234067 | 0.7934322003 | 0.4275260563 | 1.027247957 |
| lrig1 | 0.02486080701 | 0.03145641718 | 0.7903254484 | 0.4293377285 | 1.025172414 |
| tnfrsf1b | 0.02634327574 | 0.03335529191 | 0.7897780001 | 0.4296574293 | 1.026693327 |
| cdhr2 | 0.03207694567 | 0.04062906275 | 0.7895073993 | 0.4298155068 | 1.032596956 |
| klk6 | -0.02460897539 | 0.03119543364 | -0.7888646675 | 0.4301911084 | 0.9756913568 |
| pdia4 | 0.02645802927 | 0.03354227692 | 0.7887964593 | 0.4302309793 | 1.02681115 |
| f7 | 0.02559445121 | 0.03250631267 | 0.7873686402 | 0.431066099 | 1.025924802 |
| arl13b | 0.02653020295 | 0.03373442675 | 0.7864429756 | 0.4316080145 | 1.026885262 |
| col9a1 | -0.02464325201 | 0.03133694058 | -0.7863962325 | 0.43163539 | 0.975657914 |
| tspan7 | -0.02653163628 | 0.03373944664 | -0.7863684476 | 0.4316516629 | 0.9738172354 |
| hadh | 0.0262658153 | 0.0334212159 | 0.7859024452 | 0.4319246419 | 1.026613802 |
| erp29 | 0.02619022038 | 0.03335585431 | 0.7851761232 | 0.4323503126 | 1.026536198 |
| robo1 | 0.02533208956 | 0.03226769318 | 0.7850604447 | 0.43241813 | 1.025655674 |
| lefty2 | 0.02669978085 | 0.03401045026 | 0.7850463797 | 0.4324263762 | 1.027059414 |
| tpd52l2 | 0.02621355847 | 0.03345181685 | 0.7836213677 | 0.433262314 | 1.026560156 |
| smad1 | 0.02513594251 | 0.03208553073 | 0.7834042927 | 0.4333897362 | 1.025454514 |
| kifbp | 0.02436272761 | 0.03112664435 | 0.7826968861 | 0.4338051313 | 1.024661924 |
| il17rb | 0.02549227656 | 0.03260217255 | 0.7819195644 | 0.4342618464 | 1.025819983 |
| b4gat1 | 0.02584962546 | 0.03308735527 | 0.7812539036 | 0.4346531759 | 1.026186625 |
| vsnl1 | 0.02883885431 | 0.03692715807 | 0.7809659832 | 0.434822502 | 1.029258721 |
| pcdhb15 | 0.02584668944 | 0.03311560408 | 0.7804988056 | 0.4350973303 | 1.026183612 |
| lats1 | 0.02643999274 | 0.03391491802 | 0.7795977193 | 0.4356276988 | 1.02679263 |
| prss22 | 0.02676949107 | 0.03436501659 | 0.778975066 | 0.4359944027 | 1.027131013 |
| nptn | 0.02504687337 | 0.03217756738 | 0.7783954913 | 0.4363358959 | 1.025363182 |
| lrrc37a2 | 0.02565561948 | 0.03298790513 | 0.7777280606 | 0.436729346 | 1.025987557 |

| Protein | beta | se | z | p_val | HR |
|---|---|---|---|---|---|
| gfap | -0.02640416761 | 0.03396400857 | -0.7774161154 | 0.4369133076 | 0.9739413745 |
| tnfrsf1a | 0.02701113929 | 0.03477792332 | 0.7766748761 | 0.4373506132 | 1.027379247 |
| ccer2 | 0.02634828034 | 0.03395229706 | 0.7760382249 | 0.4377264165 | 1.026698465 |
| serpina3 | 0.02609352045 | 0.03362741755 | 0.7759596885 | 0.4377727879 | 1.026436937 |
| commd9 | 0.02618374897 | 0.03374545323 | 0.7759193154 | 0.4377966271 | 1.026529555 |
| apoe | -0.02587207042 | 0.03339874201 | -0.7746420632 | 0.4385511948 | 0.9744597439 |
| itgb1bp1 | 0.02419065092 | 0.03124784782 | 0.7741541452 | 0.4388396413 | 1.024485618 |
| casp9 | 0.02600420401 | 0.03362412186 | 0.7733794244 | 0.4392978633 | 1.026345263 |
| csde1 | 0.02612660353 | 0.03381266537 | 0.7726868983 | 0.4397077024 | 1.026470895 |
| gas2 | -0.02582267312 | 0.03342982699 | -0.772444115 | 0.4398514341 | 0.9745078807 |
| gpr15l | 0.02757379567 | 0.03569954059 | 0.7723851684 | 0.4398863356 | 1.027957471 |
| cxcl12 | 0.02451155916 | 0.03174840318 | 0.7720564409 | 0.4400809997 | 1.024814437 |
| tnfrsf8 | 0.0241829895 | 0.03133306592 | 0.7718041242 | 0.4402304487 | 1.024477769 |
| f10 | -0.02577602611 | 0.03341147272 | -0.7714723123 | 0.4404270277 | 0.9745533397 |
| cebpa | -0.02641214253 | 0.03427223939 | -0.7706570392 | 0.4409102425 | 0.9739336074 |
| immt | 0.02696408444 | 0.03499117194 | 0.7705967803 | 0.4409459702 | 1.027330905 |
| kynu | 0.02550460246 | 0.03309723438 | 0.7705961824 | 0.4409463247 | 1.025832628 |
| chmp6 | 0.02571525329 | 0.03339323094 | 0.7700738313 | 0.441256098 | 1.026048743 |
| htr1a | 0.02558162796 | 0.03326070802 | 0.7691245761 | 0.4418193599 | 1.025911646 |
| brd1 | -0.0255568437 | 0.03322912153 | -0.7691098208 | 0.4418281186 | 0.974766968 |
| rilpl2 | 0.02549798694 | 0.03315415346 | 0.769073684 | 0.4418495696 | 1.025825841 |
| boc | 0.02531199015 | 0.03300217303 | 0.7669794993 | 0.4430937045 | 1.025635059 |
| prdx3 | 0.02427017967 | 0.03167189362 | 0.7663002397 | 0.4434976757 | 1.024567098 |
| tacstd2 | -0.02578057729 | 0.0336629369 | -0.7658445657 | 0.4437687934 | 0.9745489043 |
| s100a14 | -0.02482019827 | 0.03246731353 | -0.7644672617 | 0.4445888391 | 0.9754852902 |
| itgb5 | 0.0244092042 | 0.03193986862 | 0.7642236883 | 0.4447339524 | 1.024709548 |
| ppib | 0.02377975599 | 0.03117738717 | 0.7627244663 | 0.4456277362 | 1.024064749 |
| cd226 | 0.02574692109 | 0.03377628084 | 0.7622781566 | 0.445894008 | 1.026081236 |
| cpm | 0.02727448234 | 0.03595019353 | 0.7586741448 | 0.4480475071 | 1.027649836 |
| lacrt | -0.02599477145 | 0.03428019204 | -0.7583029704 | 0.4482696294 | 0.974340184 |
| cngb3 | 0.02575186232 | 0.03396210425 | 0.7582528492 | 0.4482996283 | 1.026086306 |
| stab2 | 0.02647104292 | 0.03493709227 | 0.7576773336 | 0.4486441715 | 1.026824513 |
| ism1 | -0.02468475615 | 0.03264685955 | -0.7561142633 | 0.449580691 | 0.9756174209 |
| megf11 | -0.02548991224 | 0.03371256653 | -0.7560952745 | 0.449592075 | 0.9748322128 |
| mapre3 | -0.02552770066 | 0.0337660776 | -0.75601617 | 0.4496395009 | 0.9747953761 |
| ca9 | -0.02375741661 | 0.03148898047 | -0.7544676346 | 0.4505684726 | 0.9765225692 |
| msmb | -0.02495629978 | 0.03310784292 | -0.7537881535 | 0.4509764385 | 0.9753525342 |
| mtif3 | 0.0249907282 | 0.03317730962 | 0.7532475805 | 0.4513011522 | 1.025305614 |
| fnta | 0.0258805221 | 0.03436942821 | 0.7530099697 | 0.451443923 | 1.026218331 |
| csf1 | 0.02515368576 | 0.03341820557 | 0.7526940878 | 0.4516337635 | 1.025472709 |
| tdrkh | 0.02384477055 | 0.03180305333 | 0.7497635619 | 0.4533971181 | 1.02413133 |
| optc | 0.02530032892 | 0.03376327384 | 0.7493446589 | 0.4536494972 | 1.025623099 |
| ccn2 | 0.02397304166 | 0.03200532903 | 0.7490328139 | 0.4538374279 | 1.024262705 |
| micb_mica | -0.02284354362 | 0.03050348512 | -0.7488830712 | 0.4539276846 | 0.9774153947 |
| col5a1 | 0.02498513999 | 0.03339435591 | 0.7481845154 | 0.4543488696 | 1.025299884 |
| lgals1 | 0.02580079142 | 0.03450068766 | 0.7478341207 | 0.4545602184 | 1.026136513 |
| pdcd1lg2 | 0.02356716601 | 0.03153163412 | 0.7474134046 | 0.4548140565 | 1.023847066 |
| art5 | 0.02494275733 | 0.03338406811 | 0.7471455318 | 0.4549757184 | 1.02525643 |
| cd209 | 0.02330065385 | 0.03119842039 | 0.7468536407 | 0.4551519123 | 1.023574235 |
| col1a1 | 0.02315987008 | 0.0310186121 | 0.7466443054 | 0.4552782969 | 1.023430142 |
| vash1 | 0.02326714039 | 0.03116846097 | 0.7464962872 | 0.4553676736 | 1.023539932 |
| tmod4 | 0.02530286606 | 0.03390735001 | 0.7462354344 | 0.4555252064 | 1.025625701 |
| plcb1 | 0.02513246968 | 0.03369211225 | 0.745945208 | 0.4557005144 | 1.025450953 |
| metap1d | 0.02330405353 | 0.03125866199 | 0.7455230661 | 0.4559555723 | 1.023577715 |
| otud7b | 0.02520481312 | 0.03383555231 | 0.7449209898 | 0.4563194853 | 1.02552514 |
| ankra2 | -0.02526939033 | 0.03398662577 | -0.7435098294 | 0.4571730727 | 0.9750472084 |
| gipr | -0.02517327157 | 0.0338885702 | -0.742824835 | 0.4575877375 | 0.9751409332 |
| ntrk2 | 0.02413296778 | 0.03249064127 | 0.7427667426 | 0.4576229138 | 1.024426525 |
| jun | 0.02418691072 | 0.03258581844 | 0.7422526693 | 0.4579342627 | 1.024481787 |
| dpt | 0.02592866014 | 0.03495467126 | 0.7417795449 | 0.4582209158 | 1.026267732 |
| clns1a | 0.02492970299 | 0.03361672393 | 0.7415863318 | 0.4583380073 | 1.025243046 |
| fgf19 | -0.02326005159 | 0.03137200706 | -0.7414269527 | 0.4584346073 | 0.9770083782 |
| dctd | 0.02470334848 | 0.03345105522 | 0.7384923531 | 0.4602153123 | 1.025011004 |
| mrc1 | 0.02642194102 | 0.03580015723 | 0.7380398038 | 0.4604902617 | 1.026774095 |
| insl5 | -0.02655064677 | 0.03601720171 | -0.7371657296 | 0.4610215713 | 0.9737987228 |
| hsp90b1 | -0.02300958564 | 0.03127048014 | -0.7358245071 | 0.4618375048 | 0.9772531161 |
| lrg1 | -0.02500171282 | 0.0339814285 | -0.7357463745 | 0.4618850617 | 0.9753082415 |
| txndc5 | 0.02299576805 | 0.03139804944 | 0.7323947973 | 0.4639276334 | 1.023262209 |
| tshb | 0.02238305019 | 0.03058719496 | 0.7317784522 | 0.464303803 | 1.02263543 |
| pon3 | 0.02624813658 | 0.03587621332 | 0.7316306309 | 0.4643940471 | 1.026595653 |

| Protein | beta | se | z | p_val | HR |
|---|---|---|---|---|---|
| ca4 | 0.02285833523 | 0.03125845637 | 0.7312688433 | 0.4646149573 | 1.023121589 |
| kyat1 | 0.02298817293 | 0.03144350827 | 0.7310944038 | 0.4647214923 | 1.023254437 |
| mme | 0.02387705905 | 0.03274975267 | 0.7290760113 | 0.4659551676 | 1.024164398 |
| siglec10 | -0.02327632026 | 0.03192717734 | -0.7290440996 | 0.4659746871 | 0.9769924837 |
| krt14 | 0.02326314626 | 0.03194052716 | 0.7283269355 | 0.4664134777 | 1.023535844 |
| gla | 0.02623025663 | 0.03602098256 | 0.7281938127 | 0.4664949528 | 1.026577297 |
| spred2 | 0.02479171079 | 0.03409215072 | 0.7271970311 | 0.4671052647 | 1.025101581 |
| ndrg1 | 0.02304212074 | 0.03172827054 | 0.7262331147 | 0.4676958746 | 1.023309641 |
| ahsg | -0.02413017973 | 0.03324777076 | -0.7257683502 | 0.4679807925 | 0.9761586254 |
| cmc1 | 0.02405997932 | 0.03315223906 | 0.7257422124 | 0.4679968188 | 1.024351756 |
| dmp1 | 0.02543886393 | 0.03507786631 | 0.7252112688 | 0.4683224307 | 1.025765193 |
| h2ap | 0.0243829334 | 0.03364908379 | 0.7246239912 | 0.4686827366 | 1.024682628 |
| cd4 | -0.023599512 | 0.03258149792 | -0.7243224991 | 0.4688677673 | 0.9766767788 |
| folh1 | 0.02460073123 | 0.0340004141 | 0.7235421062 | 0.469346895 | 1.024905826 |
| cd80 | -0.02516022254 | 0.03480672527 | -0.7228552055 | 0.4697688465 | 0.9751536579 |
| ahnak | 0.02651396615 | 0.03669334332 | 0.7225824562 | 0.4699364498 | 1.026868589 |
| ccdc134 | 0.02408691294 | 0.03337366493 | 0.7217341275 | 0.4704579557 | 1.024379346 |
| kir2ds4 | -0.02355665858 | 0.03275110912 | -0.719262926 | 0.4719789343 | 0.9767186336 |
| eci2 | 0.02418727623 | 0.03363637028 | 0.7190810432 | 0.4720909867 | 1.024482161 |
| adamts13 | 0.02243579844 | 0.0313003816 | 0.7167899333 | 0.4735037241 | 1.022689374 |
| tff2 | 0.02433943693 | 0.03398563261 | 0.7161684234 | 0.4738873582 | 1.024638059 |
| frzb | 0.02300875961 | 0.0321357797 | 0.7159857275 | 0.4740001619 | 1.023275503 |
| mrpl28 | -0.02363397763 | 0.03301156286 | -0.7159302857 | 0.4740343967 | 0.9766431176 |
| pbxip1 | 0.02444519117 | 0.03418391621 | 0.7151079771 | 0.4745423252 | 1.024746424 |
| fgf20 | 0.0236667827 | 0.03309874438 | 0.715035665 | 0.4745870056 | 1.023949063 |
| hdac9 | -0.02402305343 | 0.03363226354 | -0.7142859534 | 0.4750503762 | 0.9762632033 |
| ccne1 | 0.0242210342 | 0.0339130127 | 0.7142106311 | 0.475096944 | 1.024516746 |
| acadsb | 0.02338453023 | 0.03276616632 | 0.7136791654 | 0.4754255925 | 1.023660092 |
| lpo | 0.02259745479 | 0.03166995109 | 0.7135298294 | 0.4755179615 | 1.022854711 |
| nubp1 | -0.02364894197 | 0.03317700735 | -0.7128111864 | 0.4759626026 | 0.9766285029 |
| lipf | -0.02439854439 | 0.03423435295 | -0.7126918515 | 0.4760364598 | 0.9758966941 |
| rrp15 | -0.02435845886 | 0.03430980252 | -0.7099562537 | 0.4777312646 | 0.9759358142 |
| frmd4b | 0.0239467792 | 0.03373406391 | 0.709869385 | 0.477785137 | 1.024235806 |
| il31ra | 0.02404693537 | 0.03390837811 | 0.7091738593 | 0.4782165932 | 1.024338394 |
| cela3a | 0.02179348932 | 0.03073564481 | 0.7090623755 | 0.4782857699 | 1.022032702 |
| pilra | 0.02258251324 | 0.03185877774 | 0.7088317517 | 0.4784288914 | 1.022839428 |
| jmjd1c | 0.02406984166 | 0.03397536288 | 0.7084498773 | 0.4786659279 | 1.024361859 |
| fas | 0.02463816419 | 0.03497473518 | 0.7044560613 | 0.481148804 | 1.024944192 |
| agr2 | 0.02298245578 | 0.03265194927 | 0.7038616773 | 0.4815189192 | 1.023248587 |
| nup50 | -0.02364584342 | 0.03359727315 | -0.7038024578 | 0.4815558029 | 0.976631529 |
| cd93 | 0.02252935988 | 0.03211505893 | 0.7015201165 | 0.482978485 | 1.022785063 |
| adamts16 | -0.02438953573 | 0.03480675899 | -0.7007126328 | 0.4834823707 | 0.9759054857 |
| agrp | -0.02265604575 | 0.03233497193 | -0.7006669373 | 0.4835108941 | 0.9775986752 |
| pde5a | 0.02322945757 | 0.03328512825 | 0.6978929869 | 0.4852441206 | 1.023501363 |
| pmvk | 0.02220565993 | 0.03196932718 | 0.6945926576 | 0.4873106149 | 1.022454041 |
| crh | -0.02197378276 | 0.03166778498 | -0.6938844245 | 0.4877546921 | 0.9782658821 |
| gzmb | 0.0223117223 | 0.03215780388 | 0.6938198387 | 0.4877951996 | 1.02256249 |
| c1s | 0.02361060933 | 0.03404820794 | 0.6934464621 | 0.4880294131 | 1.023891546 |
| naa10 | 0.0233022825 | 0.03362975507 | 0.6929066968 | 0.4883681067 | 1.023575902 |
| sdhb | 0.02297513491 | 0.03318720239 | 0.6922889926 | 0.4887558613 | 1.023241096 |
| bmp10 | 0.02475635112 | 0.03576294158 | 0.6922347554 | 0.4887899158 | 1.025065334 |
| defb103a_defb103b | 0.02360059791 | 0.03410139314 | 0.6920713712 | 0.4888925093 | 1.023881296 |
| mansc4 | -0.02338317529 | 0.03385422085 | -0.6907019185 | 0.4897528835 | 0.9768880927 |
| ppl | -0.02417648763 | 0.03500699949 | -0.6906186758 | 0.4898052079 | 0.9761134226 |
| ppie | 0.02270623175 | 0.03291665214 | 0.6898098764 | 0.4903137569 | 1.02296598 |
| fzd10 | -0.02306307708 | 0.03356251252 | -0.6871677759 | 0.4919770082 | 0.9772008428 |
| dock9 | -0.02320255897 | 0.03378624043 | -0.6867458076 | 0.4922429252 | 0.9770645505 |
| cdkn1a | 0.02152742988 | 0.03136634211 | 0.6863226131 | 0.4925096923 | 1.021760817 |
| itgb1bp2 | 0.0216058437 | 0.03155732318 | 0.6846538781 | 0.4935623597 | 1.02184094 |
| mybpc2 | -0.02418993321 | 0.03534356217 | -0.684422614 | 0.4937083402 | 0.9761002983 |
| gigyf2 | 0.02300595018 | 0.03361415131 | 0.6844126442 | 0.4937146339 | 1.023272628 |
| rbpms2 | 0.02290891817 | 0.03352171844 | 0.6834052441 | 0.4943508072 | 1.023173343 |
| angpt1 | -0.02117947508 | 0.03107696815 | -0.6815167739 | 0.4955445567 | 0.9790432349 |
| klk13 | 0.02139041561 | 0.03146560072 | 0.6798031857 | 0.4966290893 | 1.021620831 |
| tcp11 | -0.0224968556 | 0.03312075563 | -0.6792373897 | 0.4969874601 | 0.9777543116 |
| cd3e | -0.02270612629 | 0.03344509375 | -0.6789075391 | 0.4971964485 | 0.9775497177 |
| tlr4 | 0.02282827758 | 0.0336703172 | 0.6779941347 | 0.4977754121 | 1.023090837 |
| icam5 | -0.02169832522 | 0.03212124609 | -0.6755131839 | 0.499349778 | 0.97853539 |
| brsk2 | 0.02235297152 | 0.03310119729 | 0.6752919337 | 0.4994903077 | 1.022604671 |
| prss53 | -0.02254125246 | 0.03338893261 | -0.6751115026 | 0.4996049262 | 0.9777109034 |

| Protein | beta | se | z | p_val | HR |
|---|---|---|---|---|---|
| vps4b | 0.02231022274 | 0.03305284604 | 0.6749864357 | 0.4996843829 | 1.022560957 |
| osbpl2 | -0.02260863085 | 0.03352545668 | -0.674372047 | 0.5000748096 | 0.977645029 |
| tnxb | 0.02153644052 | 0.03194106094 | 0.674255641 | 0.5001488006 | 1.021770023 |
| slitrk2 | 0.02172802568 | 0.03222685919 | 0.674221014 | 0.5001708116 | 1.021965798 |
| fkbp14 | 0.02241465606 | 0.03329242819 | 0.673265883 | 0.5007781536 | 1.022667752 |
| rab39b | 0.02264067759 | 0.03373006468 | 0.6712313719 | 0.5020731462 | 1.022898923 |
| hrc | -0.0224698263 | 0.03348328394 | -0.6710759416 | 0.5021721524 | 0.97778074 |
| nt5c3a | 0.02082643195 | 0.03103888418 | 0.6709787577 | 0.502234062 | 1.021044815 |
| scg3 | 0.02148210794 | 0.03201949315 | 0.6709071827 | 0.5022796603 | 1.02171451 |
| rap1a | 0.02259533018 | 0.03370621256 | 0.6703609948 | 0.5026276931 | 1.022852538 |
| pgd | 0.02278725042 | 0.03400472209 | 0.670120178 | 0.5027811829 | 1.023048863 |
| tgfbr1 | -0.02254489141 | 0.03368873687 | -0.6692115378 | 0.5033605473 | 0.9777073456 |
| cd82 | -0.02200355717 | 0.03291608295 | -0.6684743505 | 0.5038308493 | 0.9782367553 |
| defa1_defa1b | 0.02121202684 | 0.03176265491 | 0.6678291502 | 0.5042426569 | 1.021438601 |
| ghrl | 0.02212690929 | 0.03314746745 | 0.667529407 | 0.5044340323 | 1.022373525 |
| pspn | 0.02881659135 | 0.04319384628 | 0.6671457587 | 0.504679034 | 1.029235806 |
| itgb2 | 0.02180115077 | 0.03272820911 | 0.6661272145 | 0.5053297909 | 1.022040532 |
| scg2 | -0.02088101528 | 0.03136107873 | -0.6658257983 | 0.5055224531 | 0.9793354836 |
| yy1 | 0.02238846503 | 0.03373005566 | 0.663754168 | 0.5068476639 | 1.022640968 |
| fam13a | 0.02219480224 | 0.03350648091 | 0.6624032617 | 0.5077128139 | 1.022442939 |
| rarres1 | -0.0210796554 | 0.03182559932 | -0.6623490475 | 0.50774755 | 0.9791409676 |
| cdan1 | 0.02205850855 | 0.03331451662 | 0.6621290292 | 0.507888533 | 1.022303596 |
| rgcc | 0.02175671094 | 0.03292345672 | 0.660827055 | 0.5087232302 | 1.021995114 |
| thy1 | 0.02155551624 | 0.03263320661 | 0.660539324 | 0.5089077918 | 1.021789515 |
| dnpep | 0.02250677931 | 0.03413300224 | 0.6593846963 | 0.5096487669 | 1.022761968 |
| magea3 | -0.02204411369 | 0.03346681078 | -0.6586858195 | 0.5100975409 | 0.9781970822 |
| prelp | 0.02103385305 | 0.03193856251 | 0.6585723151 | 0.5101704457 | 1.021256624 |
| tafa5 | 0.02088808055 | 0.0317722314 | 0.6574319659 | 0.5109032033 | 1.021107763 |
| adamtsl4 | -0.02298042613 | 0.03499258186 | -0.6567227942 | 0.5113591751 | 0.9772816128 |
| tgfa | 0.02150988747 | 0.03277650161 | 0.6562594057 | 0.5116572319 | 1.021742893 |
| pgm2 | 0.02200379396 | 0.03355821832 | 0.6556901726 | 0.5120234933 | 1.022247663 |
| spock1 | 0.02080177718 | 0.03174595852 | 0.6552574927 | 0.5123019837 | 1.021019642 |
| efna1 | 0.02083487034 | 0.03181449103 | 0.6548861752 | 0.5125410418 | 1.021053432 |
| pdcl2 | 0.02185737582 | 0.03339716488 | 0.6544680034 | 0.5128103348 | 1.022097998 |
| rasa1 | -0.02050399681 | 0.03143422957 | -0.6522824671 | 0.5142189685 | 0.9797047808 |
| ctsz | 0.02121048336 | 0.03252874161 | 0.652053609 | 0.5143665896 | 1.021437025 |
| amn | 0.02099572387 | 0.03220101554 | 0.6520205502 | 0.5143879155 | 1.021217685 |
| preb | 0.02049636955 | 0.03146274148 | 0.6514489389 | 0.5147567281 | 1.020707863 |
| tp53inp1 | 0.02038894743 | 0.03133185408 | 0.65074181 | 0.5152131689 | 1.020598222 |
| adgrv1 | -0.02157174965 | 0.03318623178 | -0.6500210627 | 0.5156786164 | 0.9786592565 |
| serpinb8 | 0.02083581303 | 0.03207265308 | 0.6496441994 | 0.5159220757 | 1.021054394 |
| igfbp6 | -0.02446695763 | 0.03766350973 | -0.6496196931 | 0.5159379092 | 0.9758299321 |
| fut3_fut5 | 0.0206689093 | 0.03182366197 | 0.6494824297 | 0.5160265997 | 1.02088399 |
| cep290 | -0.02171971324 | 0.03344654934 | -0.6493857712 | 0.5160890586 | 0.9785144613 |
| aldh3a1 | 0.02090708408 | 0.03221567725 | 0.6489723595 | 0.5163562422 | 1.021127168 |
| tnfrsf4 | 0.02058888998 | 0.03176364981 | 0.6481903088 | 0.5168618692 | 1.020802303 |
| esam | 0.02063649887 | 0.03188299605 | 0.6472572039 | 0.5174654943 | 1.020850904 |
| col9a2 | -0.02153167138 | 0.03327572764 | -0.6470683861 | 0.5175876848 | 0.9786984802 |
| tacc3 | 0.02011556418 | 0.0311467261 | 0.6458323779 | 0.5183879169 | 1.020319246 |
| btnl10 | 0.02178868575 | 0.03375486955 | 0.6454975545 | 0.5186048026 | 1.022027793 |
| mmut | 0.02183157918 | 0.03387199463 | 0.6445318447 | 0.5192306146 | 1.022071632 |
| cd72 | -0.02204886237 | 0.03424147923 | -0.6439226012 | 0.519625625 | 0.9781924371 |
| inpp1 | -0.02062974932 | 0.03204620831 | -0.6437500848 | 0.5197375063 | 0.9795815882 |
| tmprss15 | -0.02033419544 | 0.03159113579 | -0.6436677546 | 0.5197909039 | 0.9798711501 |
| acrv1 | 0.03406222039 | 0.05297774953 | 0.6429533283 | 0.5202543844 | 1.034648981 |
| lcat | 0.02272752126 | 0.03535573056 | 0.6428242578 | 0.520338141 | 1.022987759 |
| scn4b | -0.02184709764 | 0.03403337163 | -0.6419316275 | 0.5209175777 | 0.9783898217 |
| smpd1 | 0.0205401117 | 0.03200240598 | 0.6418302336 | 0.5209834169 | 1.020752512 |
| siae | 0.02042676247 | 0.03183099121 | 0.6417256168 | 0.5210513534 | 1.020636817 |
| tmed4 | -0.02177760761 | 0.03398315719 | -0.6408353257 | 0.5216296788 | 0.9784578124 |
| tgfb1 | 0.02062901777 | 0.03221268899 | 0.6404003646 | 0.5219123459 | 1.020843267 |
| lamp1 | 0.02197534605 | 0.03437098944 | 0.6393573888 | 0.5225904626 | 1.022218582 |
| cntf | 0.02140091069 | 0.03349241623 | 0.6389778075 | 0.5228373691 | 1.021631553 |
| ckap4 | -0.02106028589 | 0.03296828384 | -0.6388044337 | 0.5229501636 | 0.9791599333 |
| prss2 | 0.01951817377 | 0.03061099133 | 0.6376197869 | 0.5237212121 | 1.019709899 |
| echdc3 | 0.02191934457 | 0.03437703264 | 0.6376159571 | 0.5237237057 | 1.022161338 |
| vegfa | -0.02058906378 | 0.03229498218 | -0.6375313559 | 0.5237787923 | 0.9796214438 |
| cspg5 | -0.02142914622 | 0.03362690432 | -0.637261938 | 0.523954239 | 0.9787988266 |
| spesp1 | -0.02269366919 | 0.03564547514 | -0.636649367 | 0.5243532613 | 0.9775618952 |
| shd | 0.02128012626 | 0.0334526346 | 0.6361270648 | 0.5246936063 | 1.021508163 |

| Protein | beta | se | z | p_val | HR |
|---|---|---|---|---|---|
| clstn3 | 0.02150125106 | 0.03382239288 | 0.6357105227 | 0.5249651166 | 1.021734069 |
| camkk1 | 0.02062893234 | 0.03246170739 | 0.6354851298 | 0.525112062 | 1.020843179 |
| hnmt | 0.02171082843 | 0.03417914828 | 0.6352068301 | 0.5252935293 | 1.021948223 |
| dsc2 | 0.02083632915 | 0.0328908176 | 0.6334998846 | 0.5264072562 | 1.021054921 |
| siglec5 | -0.01957791508 | 0.03092664715 | -0.6330435687 | 0.5267051918 | 0.9806124877 |
| metap1 | 0.01975751675 | 0.03122070892 | 0.6328337003 | 0.526842247 | 1.019953988 |
| ilkap | 0.0198838474 | 0.0314748587 | 0.6317374635 | 0.5275584435 | 1.020082848 |
| il10ra | -0.01992548685 | 0.03158137675 | -0.630925213 | 0.5280894253 | 0.9802717137 |
| sit1 | 0.02041048291 | 0.0323552138 | 0.6308251595 | 0.5281548509 | 1.020620201 |
| serpinh1 | 0.02091272257 | 0.03318731611 | 0.6301420249 | 0.5286016663 | 1.021132926 |
| ervv_1 | 0.02123534137 | 0.03370798168 | 0.6299796165 | 0.5287079205 | 1.021462416 |
| casc3 | 0.02137837523 | 0.03394156394 | 0.6298582844 | 0.5287873081 | 1.02160853 |
| il22ra1 | 0.02024949514 | 0.03216879674 | 0.6294762998 | 0.52903728 | 1.020455907 |
| masp1 | 0.01996077179 | 0.03173092232 | 0.6290637123 | 0.529307346 | 1.02016132 |
| klk11 | -0.01964527541 | 0.03127489857 | -0.6281483333 | 0.529906773 | 0.9805464356 |
| il12rb1 | -0.02004962266 | 0.03192532518 | -0.6280162394 | 0.5299933017 | 0.9801500344 |
| sat1 | 0.02099009973 | 0.0334524309 | 0.6274611191 | 0.5303570149 | 1.021211941 |
| phldb2 | -0.02104939284 | 0.0337292628 | -0.6240691641 | 0.5325821638 | 0.9791705994 |
| meltf | 0.02104293842 | 0.03375770897 | 0.6233520894 | 0.5330531747 | 1.021265902 |
| kif1c | -0.02216109923 | 0.03556851394 | -0.6230538411 | 0.5332491412 | 0.978082654 |
| srpx | -0.02163354285 | 0.03483407229 | -0.6210454713 | 0.5345697053 | 0.9785987839 |
| il24 | -0.01936225172 | 0.03120717367 | -0.6204423358 | 0.5349666072 | 0.9808239927 |
| gdf15 | 0.02511543601 | 0.04052710766 | 0.6197194287 | 0.5354425221 | 1.025433486 |
| cntn5 | -0.02067723618 | 0.03340466489 | -0.6189924744 | 0.5359213166 | 0.979535072 |
| calb2 | -0.02112327891 | 0.03417030858 | -0.6181764166 | 0.5364590541 | 0.979098255 |
| cbln1 | -0.02058960969 | 0.03330758777 | -0.6181657415 | 0.5364660902 | 0.979620909 |
| sez6l | 0.02060866478 | 0.03334532097 | 0.6180376792 | 0.5365505014 | 1.02082249 |
| ccl3 | 0.02121065997 | 0.03433438692 | 0.6177672552 | 0.5367287712 | 1.021437205 |
| il15ra | 0.0197861195 | 0.03205015276 | 0.61734868 | 0.5370047644 | 1.019983162 |
| clip2 | 0.01929323831 | 0.03129042578 | 0.6165859949 | 0.5375078345 | 1.019480556 |
| pnliprp2 | 0.01905670316 | 0.03091673166 | 0.6163880248 | 0.537638455 | 1.019239441 |
| clec3b | -0.02077754104 | 0.033774072 | -0.6151920632 | 0.5384278887 | 0.9794368248 |
| ctrb1 | 0.01883786112 | 0.03066119293 | 0.6143877429 | 0.5389591335 | 1.019016413 |
| pard3 | 0.02038844762 | 0.03319945292 | 0.6141199878 | 0.539136041 | 1.020597712 |
| mcemp1 | 0.02070966853 | 0.03380994861 | 0.6125317956 | 0.540185968 | 1.020925602 |
| p4hb | 0.01978470062 | 0.03233825237 | 0.6118048804 | 0.5406668603 | 1.019981715 |
| ctsf | -0.01979774939 | 0.03236353692 | -0.6117300911 | 0.5407163496 | 0.9803969391 |
| timp2 | -0.0205964682 | 0.03368722833 | -0.6114028734 | 0.5409329008 | 0.9796141903 |
| pdap1 | 0.02027899802 | 0.03318314499 | 0.6111234491 | 0.541117857 | 1.020486014 |
| pafah1b3 | 0.02025249259 | 0.03314262486 | 0.6110708696 | 0.5411526639 | 1.020458966 |
| icam2 | 0.01867720231 | 0.03057481823 | 0.6108687932 | 0.541286446 | 1.018852712 |
| aspn | 0.02091845696 | 0.03426303458 | 0.6105255188 | 0.5415137443 | 1.021138781 |
| impg1 | 0.02057505943 | 0.03372133988 | 0.6101495226 | 0.5417627639 | 1.020788185 |
| c2 | 0.01979949648 | 0.03255010243 | 0.6082775475 | 0.5430034097 | 1.019996807 |
| serping1 | 0.02108851221 | 0.03469557432 | 0.6078156256 | 0.5433097646 | 1.021312446 |
| kir3dl1 | -0.01882053818 | 0.03100406239 | -0.6070345859 | 0.5438279598 | 0.9813554623 |
| ceacam6 | 0.02100999287 | 0.03465326131 | 0.6062919357 | 0.5443209127 | 1.021232257 |
| ncr3lg1 | -0.02116928152 | 0.03494821485 | -0.6057328424 | 0.5446921715 | 0.9790532149 |
| il20rb | -0.02033892131 | 0.03359488697 | -0.6054171675 | 0.544901847 | 0.9798665194 |
| sla2 | 0.02019906526 | 0.0334195784 | 0.6044081411 | 0.5455723245 | 1.020404447 |
| qsox1 | 0.02075207174 | 0.03438871921 | 0.6034557906 | 0.5462055172 | 1.020968893 |
| vps28 | 0.02031668133 | 0.03367207418 | 0.6033688696 | 0.5462633269 | 1.02052447 |
| casp2 | 0.01895989632 | 0.03145907313 | 0.602684518 | 0.5467185829 | 1.019140777 |
| chm | -0.02018315118 | 0.03360972325 | -0.6005152446 | 0.5481629042 | 0.9800191652 |
| ceacam3 | 0.01900673175 | 0.03165275089 | 0.6004764583 | 0.5481887456 | 1.01918851 |
| cfd | -0.02122254933 | 0.03542922766 | -0.5990124745 | 0.549164566 | 0.9790010643 |
| uxs1 | 0.01934487394 | 0.0323033029 | 0.5988512692 | 0.54927207 | 1.019533198 |
| alms1 | -0.01981958697 | 0.03312275824 | -0.5983676488 | 0.5495946471 | 0.9803755299 |
| capn3 | -0.01964605583 | 0.03285442612 | -0.5979728806 | 0.5498580286 | 0.9805456703 |
| krt19 | 0.01930601963 | 0.03228655586 | 0.5979584726 | 0.5498676425 | 1.019493586 |
| sdk2 | 0.01993856877 | 0.03335480299 | 0.5977720443 | 0.5499920464 | 1.02013867 |
| cntnap2 | -0.01928532998 | 0.03231689116 | -0.5967569679 | 0.550669652 | 0.9808994423 |
| epha1 | -0.02101726987 | 0.03525680916 | -0.5961194554 | 0.551095428 | 0.9792020537 |
| hao1 | 0.01995377186 | 0.033539206 | 0.5949387072 | 0.5518844425 | 1.020154179 |
| wfikkn2 | -0.01976841679 | 0.03325965349 | -0.594366288 | 0.5522671513 | 0.9804256972 |
| stc1 | 0.01967216253 | 0.033110211 | 0.5941418656 | 0.5524172315 | 1.019866935 |
| ckmt1a_ckmt1b | -0.01842167357 | 0.03102100007 | -0.5938452508 | 0.5526156203 | 0.9817469683 |
| adamts8 | -0.01872251931 | 0.03153920285 | -0.593626903 | 0.5527616831 | 0.9814516583 |
| ddr1 | 0.01878856157 | 0.03166675727 | 0.5933212995 | 0.552966147 | 1.018966177 |
| clec4g | 0.01908330993 | 0.03220960257 | 0.5924726915 | 0.5535341022 | 1.01926656 |

| Protein | beta | se | z | p_val | HR |
|---|---|---|---|---|---|
| dynlt1 | 0.0202004873 | 0.03413374675 | 0.5918039836 | 0.5539818553 | 1.020405898 |
| rnase6 | -0.02147868288 | 0.03630033739 | -0.5916937535 | 0.5540556802 | 0.9787503414 |
| matn3 | -0.01887347941 | 0.03193696263 | -0.590960375 | 0.5545469713 | 0.9813035095 |
| shisa5 | -0.02149308227 | 0.03637719722 | -0.5908394245 | 0.5546280166 | 0.9787362481 |
| slc9a3r1 | 0.01977515612 | 0.03349812347 | 0.5903362361 | 0.5549652503 | 1.01997198 |
| insl3 | -0.03189856539 | 0.05414664187 | -0.5891143807 | 0.5557845472 | 0.9686048272 |
| tyrp1 | -0.01929009491 | 0.03279644549 | -0.5881763899 | 0.5564139032 | 0.9808947684 |
| pdxdc1 | 0.01964846259 | 0.03341297438 | 0.5880488928 | 0.5564994756 | 1.019842764 |
| slc34a3 | 0.019804363 | 0.03367949344 | 0.5880243726 | 0.5565159337 | 1.02000177 |
| ruvbl1 | 0.0182464763 | 0.03111414445 | 0.5864367033 | 0.5575820855 | 1.01841396 |
| smpdl3a | -0.01847810192 | 0.03151695816 | -0.586290778 | 0.5576801272 | 0.9816915715 |
| txk | -0.01928587399 | 0.0329051002 | -0.586105919 | 0.557804339 | 0.9808989087 |
| sh3gl3 | -0.01934793959 | 0.03306378815 | -0.5851700809 | 0.5584333603 | 0.9808380305 |
| slamf8 | -0.01842732261 | 0.03150921851 | -0.5848232195 | 0.5586665899 | 0.9817414224 |
| ankmy2 | 0.0196723378 | 0.03366588993 | 0.5843403469 | 0.5589913522 | 1.019867113 |
| ap3s2 | 0.0194623587 | 0.03330736633 | 0.5843259568 | 0.5590010318 | 1.019652985 |
| crisp2 | -0.02094135237 | 0.03587321714 | -0.5837600873 | 0.5593817347 | 0.9792763951 |
| cptp | -0.01971460507 | 0.03378279727 | -0.5835693509 | 0.5595100857 | 0.980478457 |
| cdh22 | -0.01921026767 | 0.03295444742 | -0.5829339945 | 0.559937735 | 0.9809730736 |
| fgfbp3 | 0.01997006823 | 0.03429817499 | 0.5822487125 | 0.5603991661 | 1.020170804 |
| pdlim5 | 0.01931260207 | 0.03321833938 | 0.5813837304 | 0.5609818602 | 1.019500297 |
| cep20 | 0.01849697734 | 0.03200061583 | 0.5780194182 | 0.5632510093 | 1.018669106 |
| arhgap5 | 0.01915150584 | 0.03324347944 | 0.576098115 | 0.5645488655 | 1.019336072 |
| thrap3 | 0.0195737611 | 0.03402101571 | 0.5753432309 | 0.5650591894 | 1.019766583 |
| morn4 | 0.01945869447 | 0.03383078317 | 0.5751771803 | 0.5651714743 | 1.019649249 |
| get3 | 0.01912946122 | 0.03342085276 | 0.5723810028 | 0.5670638831 | 1.019313602 |
| ppp1r9b | 0.01775360958 | 0.03102454693 | 0.5722439597 | 0.5671567097 | 1.017912142 |
| itga2 | -0.01982580424 | 0.03465544761 | -0.5720833408 | 0.5672655148 | 0.9803694346 |
| ctf1 | 0.01802692969 | 0.03155487554 | 0.5712882521 | 0.5678042639 | 1.018190396 |
| cxcl1 | 0.01783678851 | 0.03123304955 | 0.5710869982 | 0.5679406716 | 1.017996814 |
| npdc1 | 0.0185267074 | 0.03247814851 | 0.5704360703 | 0.5683819708 | 1.018699392 |
| pfdn4 | 0.0192834223 | 0.03380668745 | 0.5704025964 | 0.568404669 | 1.019470548 |
| fos | 0.01944611775 | 0.03411403888 | 0.5700327017 | 0.5686555184 | 1.019636425 |
| creb3 | -0.01915602579 | 0.03368700384 | -0.5686473596 | 0.5695954781 | 0.9810262849 |
| spink6 | -0.01875273133 | 0.03298601414 | -0.5685055265 | 0.5696917542 | 0.9814220072 |
| klk10 | 0.01782015648 | 0.0314437097 | 0.5667319998 | 0.5708962765 | 1.017979883 |
| dbh | -0.01920333888 | 0.03390302439 | -0.5664196404 | 0.5711085465 | 0.9809798706 |
| tet2 | -0.01870547322 | 0.03303370572 | -0.566254158 | 0.5712210185 | 0.9814683884 |
| gadd45gip1 | 0.01922650669 | 0.03395578623 | 0.5662218087 | 0.5712430063 | 1.019412526 |
| traf3ip2 | -0.01899355877 | 0.0336608199 | -0.5642631054 | 0.5725750864 | 0.9811856823 |
| znrf4 | -0.01880100704 | 0.03332788228 | -0.564122463 | 0.5726707916 | 0.9813746295 |
| tst | 0.01777186919 | 0.03150405468 | 0.5641137109 | 0.5726767476 | 1.017930729 |
| gopc | 0.017572959 | 0.03118494452 | 0.5635077846 | 0.5730891607 | 1.017728272 |
| ccl21 | -0.01797147584 | 0.03189967596 | -0.5633748713 | 0.5731796447 | 0.9821890481 |
| cd3g | 0.01878782376 | 0.03342988976 | 0.5620067519 | 0.5741114189 | 1.018965425 |
| brd2 | 0.01893021966 | 0.03368584257 | 0.5619636683 | 0.5741407731 | 1.019110532 |
| asah1 | -0.01914790149 | 0.03410944857 | -0.561366492 | 0.5745477218 | 0.9810342551 |
| stam | 0.01858936846 | 0.03329774614 | 0.5582770793 | 0.5766551939 | 1.018763226 |
| ceacam21 | 0.01746412975 | 0.03128887668 | 0.5581577737 | 0.5767366524 | 1.017617519 |
| cdcp1 | 0.02006597983 | 0.03601110187 | 0.5572164912 | 0.5773795236 | 1.020268655 |
| hip1 | 0.01907870516 | 0.03425071784 | 0.5570308118 | 0.5775063777 | 1.019261867 |
| lgals7_lgals7b | -0.01828352938 | 0.03291103564 | -0.5555440302 | 0.5785226024 | 0.9818826003 |
| rcc1 | -0.01904206185 | 0.03431108941 | -0.5549827237 | 0.5789064774 | 0.9811380929 |
| sarg | 0.01846464298 | 0.03329921725 | 0.5545068175 | 0.5792320413 | 1.018636169 |
| nptxr | 0.01799828649 | 0.03250617764 | 0.5536881846 | 0.5797922628 | 1.018161232 |
| vwa1 | 0.01812381073 | 0.03278942276 | 0.552733449 | 0.5804459455 | 1.018289044 |
| ctag1a_ctag1b | 0.01844187057 | 0.03339466265 | 0.5522400618 | 0.5807838901 | 1.018612972 |
| slc16a1 | 0.01747875988 | 0.03181542009 | 0.5493801378 | 0.5827446019 | 1.017632407 |
| sele | 0.01820726129 | 0.03316493787 | 0.5489912677 | 0.5830114425 | 1.018374024 |
| ptpn6 | 0.01716400244 | 0.03126906101 | 0.5489132672 | 0.5830649729 | 1.01731215 |
| cd5 | -0.01744243596 | 0.03181131302 | -0.5483092114 | 0.5834796035 | 0.9827088027 |
| folr2 | 0.01777169208 | 0.03241839984 | 0.5481976953 | 0.5835561644 | 1.017930548 |
| cfhr4 | 0.01787559957 | 0.03265727762 | 0.5473695566 | 0.5841248661 | 1.018036324 |
| dsg2 | 0.0175920539 | 0.03214201693 | 0.5473226505 | 0.5841570852 | 1.017747705 |
| gcnt1 | -0.01753228993 | 0.03205558623 | -0.5469339977 | 0.5844240777 | 0.9826205064 |
| khdc3l | 0.01834477302 | 0.03356162825 | 0.5465996133 | 0.5846538348 | 1.018514072 |
| akr1b10 | -0.01835164117 | 0.0335744146 | -0.5465960132 | 0.5846563087 | 0.9818157248 |
| wnt9a | 0.01927482048 | 0.03537808901 | 0.5448236754 | 0.5858747938 | 1.019461779 |
| ncan | -0.01871998396 | 0.03438714626 | -0.544388994 | 0.5861738179 | 0.9814541467 |
| eif1ax | 0.01791773737 | 0.03301831371 | 0.5426605831 | 0.5873635182 | 1.018079223 |

| Protein | beta | se | z | p_val | HR |
|---|---|---|---|---|---|
| mzb1 | 0.01735003524 | 0.03198991695 | 0.5423594962 | 0.5875708766 | 1.017501421 |
| ifnl1 | 0.0170622291 | 0.03148480308 | 0.5419195114 | 0.5878739549 | 1.01720862 |
| epn1 | -0.01787751173 | 0.03299680607 | -0.5417952178 | 0.5879595862 | 0.9822813429 |
| icam3 | 0.01680977712 | 0.03103387918 | 0.5416589084 | 0.5880535022 | 1.016951856 |
| guca2a | 0.0174605856 | 0.03223586381 | 0.5416509296 | 0.5880589998 | 1.017613913 |
| calb1 | 0.0170651581 | 0.03154690152 | 0.5409456167 | 0.5885450677 | 1.0172116 |
| tgoln2 | -0.01799125575 | 0.03328994314 | -0.5404411678 | 0.5888928235 | 0.9821696207 |
| mag | -0.01804315975 | 0.03343800016 | -0.5396004444 | 0.5894726101 | 0.9821186434 |
| lamb1 | 0.01834398957 | 0.03403155217 | 0.5390288833 | 0.589866925 | 1.018513274 |
| ment | 0.01937222025 | 0.03598149697 | 0.5383939491 | 0.5903051029 | 1.019561079 |
| nrtn | 0.01704020946 | 0.03173123688 | 0.5370168685 | 0.5912559622 | 1.017186222 |
| tlr2 | -0.0176641843 | 0.03291764472 | -0.5366175026 | 0.5915318516 | 0.9824909128 |
| pdia2 | 0.01815726419 | 0.03386286509 | 0.53619988 | 0.5918204165 | 1.01832311 |
| pbk | 0.01786266512 | 0.03332485802 | 0.5360162407 | 0.5919473262 | 1.018023157 |
| npc2 | 0.01950724171 | 0.0364138904 | 0.535708805 | 0.5921598173 | 1.019698751 |
| igsf21 | 0.01848475365 | 0.03452714202 | 0.5353687728 | 0.5923948791 | 1.018656654 |
| mrpl46 | 0.01667658645 | 0.03127426603 | 0.533236701 | 0.5938697389 | 1.016816417 |
| amy2b | 0.01730927379 | 0.03254506127 | 0.5318556215 | 0.5948259958 | 1.017459947 |
| cinp | -0.01757337764 | 0.03308346088 | -0.5311831704 | 0.5952918542 | 0.9825801336 |
| mnat1 | -0.0177614596 | 0.03345008114 | -0.5309840513 | 0.5954298311 | 0.9823953454 |
| bcat2 | -0.01788422279 | 0.03372447824 | -0.5303039136 | 0.5959012337 | 0.9822747508 |
| adamts15 | -0.01842304427 | 0.03474998107 | -0.530159836 | 0.5960011155 | 0.9817456226 |
| ank2 | 0.01773247668 | 0.03350896262 | 0.5291860833 | 0.5966763698 | 1.01789063 |
| nrn1 | -0.01749152331 | 0.03307466822 | -0.5288495472 | 0.5969098236 | 0.9826605653 |
| agxt | 0.01733602842 | 0.03293016289 | 0.5264483044 | 0.5985767612 | 1.017487169 |
| kir2dl2 | 0.01736190414 | 0.03301219089 | 0.5259240198 | 0.5989409992 | 1.017513498 |
| cpxm2 | 0.01826591367 | 0.03478114371 | 0.5251671374 | 0.5994670077 | 1.018433756 |
| hsbp1 | -0.0175345593 | 0.03345246374 | -0.5241634648 | 0.6001648499 | 0.9826182765 |
| tia1 | 0.01665412434 | 0.03182991176 | 0.5232224478 | 0.6008194618 | 1.016793577 |
| pdzk1 | -0.01862650669 | 0.03563020036 | -0.5227729989 | 0.6011322316 | 0.9815458946 |
| cbln4 | -0.01630394674 | 0.03119721531 | -0.52260904 | 0.6012463483 | 0.9838282432 |
| vwc2 | -0.01713529827 | 0.03279095481 | -0.5225617361 | 0.601279274 | 0.983010676 |
| cdc26 | 0.01748221157 | 0.03345630085 | 0.5225386886 | 0.6012953165 | 1.01763592 |
| mllt1 | -0.0179097191 | 0.03433997787 | -0.5215413698 | 0.6019896943 | 0.9822497067 |
| dynlt3 | 0.01730366989 | 0.03324706687 | 0.5204570363 | 0.6027450655 | 1.017454246 |
| comp | -0.01727475143 | 0.03322467412 | -0.5199374227 | 0.6031071912 | 0.9828736016 |
| vcam1 | -0.01661462175 | 0.03196957004 | -0.5197011325 | 0.6032718974 | 0.9835226398 |
| cndp1 | 0.01678368131 | 0.03231006914 | 0.5194566818 | 0.6034423132 | 1.016925319 |
| lyve1 | 0.01743759211 | 0.03357057928 | 0.519430778 | 0.603460373 | 1.017590514 |
| stau1 | -0.01756787361 | 0.03388148597 | -0.5185095372 | 0.6041028076 | 0.9825855418 |
| morf4l1 | 0.01742852454 | 0.03361910535 | 0.5184113129 | 0.6041713232 | 1.017581287 |
| cdk5rap3 | 0.01721827431 | 0.03328731153 | 0.5172623899 | 0.6049730047 | 1.017367363 |
| nexn | -0.01700253885 | 0.03288045534 | -0.5171016847 | 0.6050851776 | 0.9831411886 |
| prl | 0.01650590921 | 0.03195620548 | 0.5165165563 | 0.6054936785 | 1.016642884 |
| vmo1 | -0.01620507436 | 0.03137979373 | -0.5164174914 | 0.6055628518 | 0.9839255215 |
| spink2 | -0.01865157861 | 0.03612730832 | -0.5162736854 | 0.6056632725 | 0.9815212857 |
| scp2 | 0.01640376209 | 0.03186992888 | 0.5147097176 | 0.6067558818 | 1.016539042 |
| fh | 0.01726526734 | 0.03365567544 | 0.5129972024 | 0.6079532777 | 1.017415174 |
| rab10 | 0.01695094584 | 0.03310433883 | 0.5120460473 | 0.6086187829 | 1.017095428 |
| ifngr1 | -0.01612225405 | 0.03154812467 | -0.5110368435 | 0.6093252583 | 0.9840070139 |
| top1mt | -0.01700804507 | 0.03331764505 | -0.5104816093 | 0.6097140956 | 0.9831357752 |
| ctrl | 0.01677769488 | 0.03305033347 | 0.507640714 | 0.6117053325 | 1.016919231 |
| ctla4 | 0.01655602343 | 0.03266190729 | 0.5068908953 | 0.6122313744 | 1.016693834 |
| cytl1 | -0.01753037705 | 0.03466082358 | -0.505769201 | 0.6130186821 | 0.982622386 |
| aif1l | 0.0179505397 | 0.03552584097 | 0.505281204 | 0.6133613425 | 1.018112619 |
| sorcs2 | -0.01680217477 | 0.03327091896 | -0.5050108412 | 0.6135512216 | 0.9833381945 |
| pglyrp2 | 0.0169017827 | 0.03349913045 | 0.5045439233 | 0.6138792046 | 1.017045426 |
| ccl8 | 0.01577079369 | 0.03127716248 | 0.5042271242 | 0.6141017818 | 1.015895809 |
| slitrk1 | 0.01875097834 | 0.03726371864 | 0.5031966488 | 0.6148260207 | 1.018927882 |
| megf10 | 0.01634208255 | 0.03250578911 | 0.5027437572 | 0.6151444409 | 1.016476345 |
| prr4 | -0.01694305289 | 0.03376352025 | -0.5018153546 | 0.6157974113 | 0.9831996734 |
| gp1bb | 0.01679763332 | 0.03356250932 | 0.5004880047 | 0.6167315003 | 1.016939507 |
| chchd10 | -0.01920048376 | 0.03836514857 | -0.5004668162 | 0.6167464162 | 0.9809826714 |
| gnpda2 | 0.01697683173 | 0.03393291193 | 0.5003057729 | 0.6168597899 | 1.017121757 |
| il10rb | 0.01650485046 | 0.03299378765 | 0.5002411555 | 0.6169052827 | 1.016641808 |
| fst | -0.01651231613 | 0.03303513813 | -0.4998409896 | 0.617187046 | 0.9836232649 |
| notch2 | 0.01724193688 | 0.03449782092 | 0.4997978546 | 0.6172174214 | 1.017391437 |
| flt3 | -0.01620655402 | 0.03243174639 | -0.4997126526 | 0.6172774221 | 0.9839240656 |
| bambi | 0.0161766537 | 0.03242102423 | 0.4989556649 | 0.6178106177 | 1.016308204 |
| cblif | -0.01534913863 | 0.0307726707 | -0.4987912418 | 0.6179264582 | 0.984768059 |

| Protein | beta | se | z | p_val | HR |
|---|---|---|---|---|---|
| sptbn2 | 0.01676503589 | 0.03362357193 | 0.4986096043 | 0.6180544378 | 1.016906358 |
| cd34 | -0.01557260017 | 0.03125817955 | -0.4981928055 | 0.6183481529 | 0.9845480258 |
| pafah2 | 0.01659765037 | 0.0333198814 | 0.4981305357 | 0.6183920392 | 1.016736157 |
| kirrel1 | 0.0166123225 | 0.03342996356 | 0.4969291238 | 0.619239033 | 1.016751074 |
| ifngr2 | 0.01580963114 | 0.03181483483 | 0.4969263936 | 0.6192409583 | 1.015935265 |
| cd38 | -0.01898589346 | 0.03821006154 | -0.4968820436 | 0.6192722348 | 0.9811932034 |
| rtkn2 | -0.01664069907 | 0.03349087611 | -0.4968726113 | 0.6192788867 | 0.9834969925 |
| tnfrsf19 | 0.01631441669 | 0.03283949519 | 0.4967925542 | 0.6193353465 | 1.016448223 |
| lgals8 | 0.0156084698 | 0.03143072958 | 0.4965990293 | 0.6194718378 | 1.015730918 |
| galnt7 | 0.01531000603 | 0.03096910073 | 0.49436392 | 0.6210491901 | 1.015427805 |
| vegfc | 0.01536533395 | 0.03109606189 | 0.4941247546 | 0.6212180763 | 1.015483988 |
| padi2 | 0.01577603998 | 0.03202056415 | 0.4926846358 | 0.6222354354 | 1.015901139 |
| erp44 | 0.01582556553 | 0.03213337302 | 0.4924962443 | 0.6223685763 | 1.015951453 |
| robo4 | 0.01654522415 | 0.03360761345 | 0.4923058335 | 0.6225031569 | 1.016682854 |
| grin2b | -0.01651772269 | 0.03359034497 | -0.4917401922 | 0.6229030212 | 0.9836179469 |
| syngap1 | 0.01646553714 | 0.03348468061 | 0.4917334386 | 0.6229077962 | 1.016601841 |
| wdr46 | -0.01640867473 | 0.0334268674 | -0.490882814 | 0.623509333 | 0.9837252143 |
| vasp | 0.01618155424 | 0.03305817099 | 0.4894872814 | 0.6244967568 | 1.016313185 |
| nomo1 | 0.01635995361 | 0.03343414971 | 0.4893186682 | 0.6246161065 | 1.01649451 |
| ppbp | 0.01623024855 | 0.03318284462 | 0.4891156481 | 0.6247598236 | 1.016362675 |
| angptl3 | 0.01658949459 | 0.03394499575 | 0.488716944 | 0.6250421062 | 1.016727864 |
| lama1 | -0.01632339846 | 0.03345539715 | -0.4879152497 | 0.6256098724 | 0.9838091063 |
| cd28 | 0.01581401186 | 0.03241918275 | 0.48779798 | 0.6256929423 | 1.015939715 |
| ccl25 | -0.01576383766 | 0.03238960366 | -0.4866943675 | 0.6264749374 | 0.9843597613 |
| efcab14 | -0.01684803344 | 0.03462091836 | -0.4866431694 | 0.6265112254 | 0.983293101 |
| ednrb | 0.01654132648 | 0.03402437237 | 0.4861611055 | 0.6268529454 | 1.016678892 |
| sct | -0.01598136392 | 0.03300144114 | -0.4842626069 | 0.62819951 | 0.9841456605 |
| tomm20 | 0.0161510549 | 0.03337954736 | 0.4838608122 | 0.6284846534 | 1.016282188 |
| treml2 | -0.01513882832 | 0.03131750182 | -0.4833983375 | 0.6288129284 | 0.9849751877 |
| swap70 | 0.01666071032 | 0.03447341112 | 0.4832916088 | 0.6288886972 | 1.016800274 |
| fabp4 | 0.02029911772 | 0.04204452972 | 0.4828004465 | 0.6292374337 | 1.020506546 |
| il13ra2 | -0.0161758948 | 0.03358996102 | -0.4815693233 | 0.6301119227 | 0.9839542324 |
| f2 | 0.01605715872 | 0.03343709979 | 0.4802198402 | 0.6310710806 | 1.016186768 |
| dcun1d2 | 0.01619887901 | 0.03375009707 | 0.4799654049 | 0.6312519926 | 1.016330792 |
| ifnw1 | -0.01596524493 | 0.03326970128 | -0.4798734078 | 0.631317411 | 0.9841615241 |
| ntf4 | -0.01514706187 | 0.03157620556 | -0.4796986086 | 0.6314417174 | 0.9849670778 |
| hsd17b3 | 0.01616287068 | 0.0337163939 | 0.4793772054 | 0.6316703067 | 1.016294196 |
| drg2 | 0.01491671284 | 0.03122420519 | 0.4777291447 | 0.6328429978 | 1.015028522 |
| unc5d | 0.01625569623 | 0.03403745237 | 0.4775826361 | 0.632947292 | 1.016388539 |
| scn3a | -0.01592689473 | 0.03336035887 | -0.4774197661 | 0.6330632418 | 0.9841992676 |
| nck2 | 0.01486654916 | 0.03115577561 | 0.4771683218 | 0.6332422668 | 1.014977606 |
| naa80 | 0.01611660075 | 0.03379317384 | 0.4769188247 | 0.6334199266 | 1.016247174 |
| ctsb | 0.01507200828 | 0.03160871427 | 0.4768307926 | 0.6334826168 | 1.015186164 |
| cnpy4 | 0.01489783777 | 0.031254044 | 0.476669124 | 0.6335977526 | 1.015009364 |
| ppp1r14a | 0.01570475471 | 0.03298595441 | 0.4761042994 | 0.6340000742 | 1.015828722 |
| ak2 | 0.01579936653 | 0.03319630578 | 0.4759374923 | 0.6341189107 | 1.015924836 |
| rpl14 | 0.01641681881 | 0.03462371861 | 0.4741494984 | 0.6353933032 | 1.016552315 |
| evi2b | 0.01616694088 | 0.03414422845 | 0.4734897115 | 0.6358638395 | 1.016298333 |
| adam9 | -0.01623716985 | 0.03432994504 | -0.472973954 | 0.6362317616 | 0.9838939424 |
| lrrc25 | -0.01529563215 | 0.03237773807 | -0.4724120048 | 0.6366327373 | 0.9848207519 |
| gbp4 | 0.01486914347 | 0.03147915595 | 0.4723488613 | 0.6366777996 | 1.014980239 |
| sag | 0.01615664061 | 0.03423757658 | 0.4718979034 | 0.6369996646 | 1.016287865 |
| art3 | 0.01613972638 | 0.03424295098 | 0.4713298918 | 0.6374051727 | 1.016270675 |
| exosc10 | -0.01581041102 | 0.03359970701 | -0.4705520503 | 0.637960656 | 0.9843139174 |
| ifnl2 | 0.01571387015 | 0.03350231917 | 0.4690382796 | 0.6390422745 | 1.015837982 |
| cd244 | 0.01450031133 | 0.03101187242 | 0.4675729068 | 0.6400900436 | 1.014605951 |
| tnfrsf10b | 0.01666175696 | 0.03577154083 | 0.465782479 | 0.6413712077 | 1.016801338 |
| mmp15 | -0.01532815565 | 0.03296365312 | -0.4650017278 | 0.6419302193 | 0.9847887226 |
| qpct | 0.01472384671 | 0.03171429254 | 0.464265337 | 0.6424576552 | 1.014832777 |
| gpa33 | -0.01435693543 | 0.03107396757 | -0.4620245355 | 0.6440637257 | 0.9857456339 |
| c1qa | 0.01496261925 | 0.03249925917 | 0.4603987793 | 0.6452300108 | 1.01507512 |
| myom2 | -0.01588817605 | 0.03457154871 | -0.4595737431 | 0.6458222094 | 0.9842373752 |
| cd163 | 0.01503021256 | 0.03275553004 | 0.4588603067 | 0.6463344845 | 1.015143734 |
| ttf2 | -0.01544067725 | 0.03366258121 | -0.4586896399 | 0.6464570548 | 0.9846779188 |
| dlk1 | -0.0149353247 | 0.03260599691 | -0.4580545335 | 0.6469132627 | 0.9851756541 |
| ofd1 | -0.01536423848 | 0.03357317371 | -0.4576343784 | 0.6472151404 | 0.9847531893 |
| mfge8 | 0.01459172518 | 0.03197192012 | 0.456391894 | 0.6481081937 | 1.014698704 |
| pmch | 0.01536840661 | 0.033694758 | 0.4561067515 | 0.6483132154 | 1.015487108 |
| pdlim7 | 0.01425508267 | 0.03129934672 | 0.455443457 | 0.6487902373 | 1.014357171 |
| dbn1 | 0.0154423591 | 0.03393474534 | 0.4550604091 | 0.6490657796 | 1.015562208 |

| Protein | beta | se | z | p_val | HR |
|---|---|---|---|---|---|
| uros | 0.01509909482 | 0.03334796123 | 0.4527741508 | 0.6507113789 | 1.015213662 |
| rnase10 | 0.01538266682 | 0.03400749063 | 0.4523317227 | 0.6510300261 | 1.015501589 |
| slc44a4 | 0.01514132076 | 0.03350794957 | 0.4518724946 | 0.6513608405 | 1.015256531 |
| col3a1 | -0.01512455513 | 0.03356473448 | -0.4506085143 | 0.6522717291 | 0.9849892465 |
| bloc1s2 | -0.0151375672 | 0.03360386959 | -0.4504709543 | 0.6523708932 | 0.9849764298 |
| cnpy2 | -0.01413401821 | 0.03139947752 | -0.4501354586 | 0.6526127706 | 0.9859653981 |
| ogt | -0.01490828654 | 0.03315630753 | -0.4496365142 | 0.6529725548 | 0.9852022918 |
| clul1 | 0.01485606627 | 0.03305718724 | 0.4494050315 | 0.6531395024 | 1.014966966 |
| wfikkn1 | -0.014713936 | 0.03280358898 | -0.4485465296 | 0.6537588135 | 0.985393785 |
| efna4 | 0.01462689644 | 0.03261279078 | 0.4485018329 | 0.6537910637 | 1.014734393 |
| ids | -0.01395574116 | 0.03113082842 | -0.4482932794 | 0.6539415503 | 0.9861411888 |
| xpnpep2 | 0.01431625574 | 0.03194811209 | 0.4481096004 | 0.6540740998 | 1.014419224 |
| cyp24a1 | -0.01499289782 | 0.03347918469 | -0.4478274474 | 0.6542777329 | 0.9851189361 |
| spink8 | -0.01505376982 | 0.03364627015 | -0.447412737 | 0.6545770811 | 0.9850589717 |
| crim1 | 0.01465184923 | 0.03277428203 | 0.447053248 | 0.6548366139 | 1.014759714 |
| pla2g1b | 0.01387924722 | 0.03123923436 | 0.4442889688 | 0.6568336756 | 1.013976011 |
| mapk13 | 0.01499009253 | 0.03375158033 | 0.4441300934 | 0.6569485303 | 1.015103007 |
| spink4 | 0.01426268037 | 0.03211715603 | 0.444082918 | 0.6569826361 | 1.014364878 |
| s100a3 | -0.01483059041 | 0.03344066589 | -0.4434896858 | 0.657411578 | 0.9852788412 |
| vipr1 | -0.01508360785 | 0.03402547236 | -0.4433034079 | 0.6575462911 | 0.9850295799 |
| golm2 | 0.01439526001 | 0.03256215028 | 0.4420856695 | 0.658427214 | 1.014499371 |
| sms | -0.01468388396 | 0.0332574517 | -0.4415216202 | 0.6588354131 | 0.9854233985 |
| tnfaip8l2 | 0.01486883563 | 0.03378196676 | 0.4401412072 | 0.6598348384 | 1.014979927 |
| snapin | -0.01471048448 | 0.03343164334 | -0.440016793 | 0.6599249447 | 0.9853971861 |
| acaa1 | 0.01440081221 | 0.03273150876 | 0.4399678708 | 0.6599603777 | 1.014505003 |
| vsig2 | 0.01492780262 | 0.03411463513 | 0.4375776721 | 0.6616924598 | 1.015039779 |
| ehbp1 | 0.01448519849 | 0.03325877559 | 0.435530119 | 0.6631776819 | 1.014590617 |
| itih1 | 0.01441316349 | 0.03309633449 | 0.4354912323 | 0.6632059017 | 1.014517534 |
| enoph1 | -0.01447408899 | 0.03326233277 | -0.4351495457 | 0.6634538824 | 0.9856301571 |
| fdx1 | 0.01491086034 | 0.03427879209 | 0.4349879163 | 0.6635711985 | 1.015022582 |
| cdkn2d | 0.01354587457 | 0.03116987395 | 0.4345822696 | 0.663865667 | 1.013638036 |
| robo2 | -0.01428439585 | 0.03288562902 | -0.4343659001 | 0.664022756 | 0.9858171421 |
| fap | -0.01384244553 | 0.03195241799 | -0.4332205948 | 0.6648545184 | 0.9862529206 |
| lta | -0.01381744869 | 0.03192451915 | -0.4328161883 | 0.6651483118 | 0.9862775741 |
| mpi | 0.01370146159 | 0.03166794346 | 0.4326602895 | 0.6652615829 | 1.013795757 |
| kazald1 | 0.0135603984 | 0.03138641617 | 0.4320467277 | 0.6657074518 | 1.013652758 |
| oscar | -0.01350947971 | 0.03128135646 | -0.4318700093 | 0.6658358931 | 0.9865813638 |
| gas6 | 0.01385804065 | 0.03210972418 | 0.4315839204 | 0.6660438472 | 1.013954508 |
| nphs1 | -0.01455797437 | 0.03374711781 | -0.4313842281 | 0.666189016 | 0.9855474806 |
| iglc2 | -0.01450964029 | 0.03364580372 | -0.4312466545 | 0.666289034 | 0.9855951173 |
| hjv | -0.01500506562 | 0.03480316666 | -0.4311408146 | 0.6663659853 | 0.9851069494 |
| inhbb | -0.01503599814 | 0.03488962215 | -0.430959042 | 0.6664981519 | 0.985076478 |
| itgam | 0.013595884 | 0.03159127469 | 0.4303683257 | 0.6669277322 | 1.013688728 |
| vsir | 0.01353005721 | 0.03147761336 | 0.4298311009 | 0.6673185074 | 1.013622003 |
| prkag3 | 0.01429844803 | 0.03326753813 | 0.4298018077 | 0.6673398178 | 1.01440116 |
| anxa1 | 0.01488099544 | 0.03471467057 | 0.4286658982 | 0.6681663813 | 1.014992269 |
| evpl | 0.01412306844 | 0.03296159404 | 0.428470432 | 0.6683086561 | 1.01422327 |
| pvr | 0.01337401313 | 0.03127955594 | 0.4275640342 | 0.6689685556 | 1.013463845 |
| syt1 | -0.01434397779 | 0.03360018098 | -0.4269018016 | 0.6694508533 | 0.9857584069 |
| gage2a | 0.01437720113 | 0.03370790986 | 0.4265230679 | 0.6697267427 | 1.01448105 |
| slitrk6 | 0.01356742644 | 0.03184663493 | 0.426023863 | 0.6700904578 | 1.013659882 |
| lilra4 | 0.01433971695 | 0.03366147071 | 0.4259979333 | 0.670109352 | 1.014443024 |
| ghr | 0.01576255377 | 0.03700913976 | 0.4259097582 | 0.6701736041 | 1.015887438 |
| marco | -0.01374499499 | 0.03230901243 | -0.4254229379 | 0.6705283876 | 0.9863490361 |
| rnf168 | -0.01423282012 | 0.03346840946 | -0.4252613239 | 0.6706461844 | 0.9858679876 |
| tmprss11b | 0.01419019391 | 0.03339246458 | 0.424951979 | 0.6708716816 | 1.014291353 |
| dlgap5 | -0.01409306431 | 0.0331751441 | -0.4248079305 | 0.670976696 | 0.986005778 |
| cd101 | 0.01419713231 | 0.03343741541 | 0.4245882086 | 0.6711368903 | 1.01429839 |
| arnt | -0.01341250525 | 0.03161440677 | -0.4242529473 | 0.6713813504 | 0.9866770416 |
| postn | -0.01429976661 | 0.03373930211 | -0.423831132 | 0.671688972 | 0.9858019894 |
| npr1 | -0.01433796876 | 0.03388850987 | -0.4230923347 | 0.6722278948 | 0.9857643304 |
| ptpn9 | 0.01438649578 | 0.03407426905 | 0.4222099601 | 0.6728717722 | 1.014490479 |
| mthfd2 | 0.01406514803 | 0.03333580993 | 0.4219230927 | 0.6730811538 | 1.014164528 |
| txnl1 | -0.01436455468 | 0.03406282668 | -0.4217076527 | 0.673238418 | 0.9857381233 |
| nars1 | 0.01385524842 | 0.03288842759 | 0.4212803542 | 0.6735503742 | 1.013951677 |
| psca | -0.01363432089 | 0.03238944035 | -0.4209495671 | 0.6737919093 | 0.9864582055 |
| mdga1 | -0.01351730708 | 0.03211415584 | -0.4209142892 | 0.6738176706 | 0.9865736415 |
| orm1 | -0.01443097499 | 0.03429534992 | -0.4207851799 | 0.6739119545 | 0.9856726525 |
| fgf12 | 0.01416114265 | 0.03368013668 | 0.4204597739 | 0.6741496096 | 1.014261887 |
| ctrc | 0.01299813296 | 0.03098096349 | 0.4195522506 | 0.6748125768 | 1.013082976 |

| Protein | beta | se | z | p_val | HR |
|---|---|---|---|---|---|
| cd276 | 0.01323080666 | 0.03154377123 | 0.4194427661 | 0.6748925748 | 1.013318721 |
| slamf6 | -0.01340295581 | 0.03199381288 | -0.4189233668 | 0.6752721392 | 0.9866864639 |
| slc39a14 | -0.01329710331 | 0.03174362964 | -0.41889045 | 0.6752961968 | 0.9867909126 |
| tsc1 | 0.01411908638 | 0.03372091631 | 0.4187041138 | 0.6754323888 | 1.014219231 |
| dcn | 0.01394748181 | 0.03331534479 | 0.4186503816 | 0.6754716633 | 1.014045202 |
| hepacam2 | -0.01407991952 | 0.03365449211 | -0.4183667214 | 0.6756790139 | 0.986018739 |
| gid8 | -0.01388029865 | 0.03321444268 | -0.4178994897 | 0.6760206057 | 0.9862155885 |
| adgrg2 | -0.01502984019 | 0.03600031357 | -0.4174919242 | 0.67631863 | 0.9850825441 |
| chad | 0.01414412493 | 0.03390138243 | 0.41721381 | 0.6765220249 | 1.014244626 |
| hrg | -0.0140469439 | 0.03367191642 | -0.4171709067 | 0.6765534036 | 0.9860512541 |
| gucy2c | -0.01393786714 | 0.03342088471 | -0.4170406397 | 0.6766486824 | 0.9861588152 |
| ctsc | -0.0132866003 | 0.03193209132 | -0.4160892616 | 0.6773446875 | 0.9868012769 |
| pilrb | 0.01288018805 | 0.03101824375 | 0.4152455617 | 0.6779621483 | 1.012963495 |
| bcat1 | -0.01431172952 | 0.03446802355 | -0.4152175857 | 0.6779826263 | 0.9857901965 |
| phykpl | 0.01396464163 | 0.0336430627 | 0.415082353 | 0.6780816175 | 1.014062603 |
| dapp1 | 0.01293748602 | 0.03118783234 | 0.4148247906 | 0.6782701701 | 1.013021537 |
| cbs | -0.01434919589 | 0.03465603615 | -0.4140460791 | 0.6788403609 | 0.9857532632 |
| angpt2 | 0.01375489996 | 0.03322756867 | 0.4139604704 | 0.6789030568 | 1.013849934 |
| pam | -0.01277956493 | 0.03100898228 | -0.4121246166 | 0.6802480865 | 0.987301747 |
| vstm2l | 0.01293752808 | 0.03152472655 | 0.410393031 | 0.6815176577 | 1.01302158 |
| sytl4 | -0.01361821106 | 0.03319862643 | -0.4102040512 | 0.6816562693 | 0.9864740973 |
| crx | 0.01277601651 | 0.03116941103 | 0.4098895707 | 0.6818869562 | 1.012857978 |
| omd | 0.01346406698 | 0.03291040388 | 0.4091127847 | 0.6824568944 | 1.013555116 |
| smad3 | 0.01357812407 | 0.03322182801 | 0.4087109254 | 0.6827518151 | 1.013670725 |
| lrrc38 | 0.01409039269 | 0.03455091363 | 0.407815343 | 0.6834092485 | 1.01419013 |
| mfap5 | 0.01398122045 | 0.03431542816 | 0.407432493 | 0.6836903661 | 1.014079415 |
| folr1 | -0.01343884719 | 0.03301443338 | -0.4070597559 | 0.6839641002 | 0.986651051 |
| ctsv | 0.01325105583 | 0.03258076049 | 0.4067141352 | 0.6842179575 | 1.01333924 |
| gp5 | 0.0135359681 | 0.03333410174 | 0.4060696821 | 0.6846914014 | 1.013627994 |
| wasf3 | -0.01273613557 | 0.03141143924 | -0.4054617004 | 0.6851381654 | 0.9873446258 |
| clps | 0.01252623657 | 0.03100444222 | 0.4040142533 | 0.686202238 | 1.012605018 |
| rspo1 | 0.01323903202 | 0.0327720096 | 0.4039737625 | 0.6862320132 | 1.013327056 |
| cdc25a | -0.01371408044 | 0.03400607388 | -0.4032832632 | 0.6867398527 | 0.9863795291 |
| rras | 0.01353447983 | 0.03356992082 | 0.4031728254 | 0.6868210893 | 1.013626486 |
| erc2 | -0.01339556079 | 0.03323748463 | -0.4030257083 | 0.6869293122 | 0.9866937605 |
| lpl | -0.01450803869 | 0.03600909236 | -0.4028993163 | 0.6870222943 | 0.9855966958 |
| adamts4 | 0.01406148991 | 0.03491142448 | 0.4027761719 | 0.6871128919 | 1.014160818 |
| capg | -0.01297692066 | 0.03225305945 | -0.4023469673 | 0.6874286936 | 0.9871069165 |
| idua | -0.01258362817 | 0.03128179055 | -0.4022668762 | 0.6874876293 | 0.9874952146 |
| dnajb6 | 0.01356407293 | 0.03375284929 | 0.4018645304 | 0.6877837278 | 1.013656482 |
| pcna | 0.01359426293 | 0.03386112087 | 0.401471144 | 0.6880732791 | 1.013687085 |
| syap1 | 0.01342192667 | 0.03348628591 | 0.400818613 | 0.6885536741 | 1.013512405 |
| scn2b | -0.01341730531 | 0.03359056754 | -0.3994366958 | 0.6895714597 | 0.9866723055 |
| slurp1 | -0.01366246229 | 0.03421061771 | -0.3993632155 | 0.6896255939 | 0.9864304455 |
| entpd2 | 0.01283774694 | 0.0322353444 | 0.3982506526 | 0.6904454318 | 1.012920505 |
| sez6l2 | 0.01400580084 | 0.03518357167 | 0.3980778577 | 0.6905727955 | 1.014104342 |
| cenpj | 0.01325678007 | 0.03333838854 | 0.397643097 | 0.6908932877 | 1.013345041 |
| cdh23 | -0.01328142503 | 0.03342883338 | -0.3973044729 | 0.6911429494 | 0.9868063839 |
| akr1c4 | 0.01242455841 | 0.03130811895 | 0.3968478089 | 0.6914796929 | 1.012502064 |
| trdmt1 | -0.01294182222 | 0.03263714229 | -0.396536624 | 0.6917091953 | 0.9871415631 |
| nedd9 | 0.01345798976 | 0.03395310052 | 0.3963699795 | 0.6918321093 | 1.013548956 |
| iglon5 | -0.01334702157 | 0.03370895442 | -0.395948845 | 0.6921427664 | 0.986741655 |
| nid2 | -0.01225668699 | 0.03098546186 | -0.3955625075 | 0.6924278004 | 0.9878181203 |
| tmco5a | 0.01333720446 | 0.0337260654 | 0.395456876 | 0.6925057414 | 1.013426542 |
| lypd3 | -0.01239095919 | 0.03139728465 | -0.3946506624 | 0.6931007188 | 0.9876854927 |
| eif5a | 0.01245561385 | 0.03158298429 | 0.3943773564 | 0.6933024589 | 1.012533508 |
| cxcl5 | 0.01250963641 | 0.03186105853 | 0.3926309103 | 0.6945921062 | 1.012588209 |
| tpsab1 | -0.01229802053 | 0.03133937415 | -0.3924143625 | 0.6947520756 | 0.9877772911 |
| mstn | 0.01365995469 | 0.03483318308 | 0.392153501 | 0.6949447988 | 1.013753678 |
| hdac8 | -0.01320739907 | 0.03374582708 | -0.3913787336 | 0.6955173093 | 0.9868794359 |
| smarca2 | 0.01237791054 | 0.03162946727 | 0.3913411009 | 0.6955451223 | 1.012454834 |
| l3hypdh | 0.01291140096 | 0.03305224451 | 0.3906361322 | 0.6960662139 | 1.012995113 |
| blnk | 0.01351675201 | 0.03461829968 | 0.3904510659 | 0.6962030332 | 1.013608516 |
| myh4 | -0.01330619794 | 0.03408741667 | -0.3903551293 | 0.6962739629 | 0.9867819382 |
| klk15 | 0.01292261804 | 0.03312488391 | 0.3901181381 | 0.6964491911 | 1.013006476 |
| gpd1 | -0.01455729367 | 0.0373895598 | -0.3893411356 | 0.6970238103 | 0.9855481514 |
| cpa1 | 0.01193294356 | 0.03065967933 | 0.3892064048 | 0.6971234659 | 1.012004425 |
| ca8 | 0.01298206957 | 0.03342077095 | 0.3884431507 | 0.6976881166 | 1.013066702 |
| itgal | -0.01323250211 | 0.0340960971 | -0.3880943343 | 0.6979462246 | 0.9868546626 |
| adam12 | -0.01412606016 | 0.03655961024 | -0.3863843205 | 0.6992120607 | 0.9859732445 |

| Protein | beta | se | z | p_val | HR |
|---|---|---|---|---|---|
| flrt2 | -0.0120179355 | 0.03111957175 | -0.3861857612 | 0.6993590983 | 0.9880539915 |
| hmox1 | 0.01233428279 | 0.03204619105 | 0.3848907587 | 0.7003183529 | 1.012410664 |
| igfbp2 | -0.01355818705 | 0.03526116904 | -0.3845075877 | 0.7006022732 | 0.9865333112 |
| scgb3a1 | -0.01338471251 | 0.03484225779 | -0.3841516985 | 0.7008660157 | 0.9867044644 |
| sprr3 | -0.0135939795 | 0.03539091284 | -0.3841093209 | 0.7008974233 | 0.9864980014 |
| psmd5 | -0.01280763576 | 0.03339530741 | -0.3835160314 | 0.701337186 | 0.987274033 |
| cpvl | 0.01189426132 | 0.03103284979 | 0.3832796989 | 0.70151239 | 1.011965279 |
| vstm1 | -0.01194826018 | 0.03120837628 | -0.382854272 | 0.7018278184 | 0.9881228368 |
| ces1 | 0.01273436992 | 0.03328517005 | 0.3825838924 | 0.7020283152 | 1.012815797 |
| itgbl1 | -0.01344535987 | 0.03521370704 | -0.3818217676 | 0.7025935716 | 0.9866446252 |
| s100a13 | -0.01346907798 | 0.03533032462 | -0.381232783 | 0.7030305253 | 0.9866212242 |
| cd300c | -0.01206422619 | 0.03170796812 | -0.3804793214 | 0.7035896435 | 0.9880082548 |
| rab11fip3 | 0.01256678005 | 0.0330792162 | 0.3798995712 | 0.7040199657 | 1.012646074 |
| faslg | -0.01265320856 | 0.03331218155 | -0.3798372838 | 0.7040662044 | 0.9874265067 |
| skiv2l | 0.01291216467 | 0.03406916169 | 0.3789986024 | 0.7046889016 | 1.012995887 |
| omp | 0.01280476401 | 0.0338195529 | 0.3786201445 | 0.7049699609 | 1.012887096 |
| necap2 | 0.01256965031 | 0.03325226658 | 0.3780088277 | 0.7054240362 | 1.01264898 |
| s100p | -0.01233700343 | 0.0326996056 | -0.3772829429 | 0.7059633468 | 0.9877387854 |
| il7 | 0.01186588145 | 0.03146937971 | 0.3770611799 | 0.7061281395 | 1.01193656 |
| c19orf12 | 0.01257334374 | 0.03344312931 | 0.375961939 | 0.706945192 | 1.012652721 |
| aoc3 | 0.01223119118 | 0.03253423666 | 0.3759483068 | 0.7069553267 | 1.012306298 |
| apoc1 | -0.01349065067 | 0.03589393994 | -0.3758475857 | 0.7070302088 | 0.9865999403 |
| mep1a | 0.01319180695 | 0.03512663462 | 0.3755499806 | 0.7072514827 | 1.013279203 |
| ikzf2 | -0.01192144219 | 0.03174897144 | -0.3754906585 | 0.7072955925 | 0.9881493367 |
| pfdn2 | 0.01189645612 | 0.03170800156 | 0.3751878244 | 0.7075207846 | 1.0119675 |
| cpb2 | 0.01274790431 | 0.03400084654 | 0.3749290269 | 0.7077132508 | 1.012829505 |
| il3ra | 0.01209685453 | 0.03238014971 | 0.3735885916 | 0.708710423 | 1.012170317 |
| abca2 | -0.01294162599 | 0.03465860451 | -0.3734029739 | 0.7088485465 | 0.9871417568 |
| ctsd | 0.01319045117 | 0.0353861951 | 0.3727569785 | 0.709329325 | 1.013277829 |
| septin3 | -0.01267666243 | 0.03402640167 | -0.3725537172 | 0.709480625 | 0.987403348 |
| prnd | 0.01232261363 | 0.03311624739 | 0.3721017505 | 0.709817093 | 1.01239885 |
| ppp1cc | 0.01233611246 | 0.03319506141 | 0.3716249327 | 0.7101721229 | 1.012412516 |
| pinlyp | -0.0125114647 | 0.03372257592 | -0.3710115364 | 0.7106289392 | 0.9875664783 |
| cpb1 | -0.0113566583 | 0.03061453091 | -0.3709564696 | 0.7106699543 | 0.9887075851 |
| reg4 | 0.01210656535 | 0.03270063009 | 0.3702242225 | 0.7112154299 | 1.012180146 |
| nenf | -0.01222332674 | 0.03305674295 | -0.3697680306 | 0.7115553375 | 0.9878510747 |
| mrps16 | 0.01243148356 | 0.03363955341 | 0.3695496015 | 0.7117181088 | 1.012509076 |
| manf | 0.01153181058 | 0.0312069138 | 0.3695274278 | 0.7117346331 | 1.011598558 |
| nrcam | 0.01170416486 | 0.03169508994 | 0.3692737543 | 0.7119236863 | 1.011772927 |
| tnfrsf11b | -0.01311447097 | 0.03558473486 | -0.3685420453 | 0.7124691002 | 0.986971149 |
| serpine2 | 0.01231829222 | 0.03345024106 | 0.3682572033 | 0.7126814604 | 1.012394475 |
| tmprss11d | -0.01232763687 | 0.03352889227 | -0.3676720594 | 0.7131177768 | 0.9877480372 |
| lrp2 | 0.01247438852 | 0.03398903317 | 0.3670121611 | 0.7136099468 | 1.012552518 |
| hpse | 0.01233141617 | 0.03362541102 | 0.3667290837 | 0.7138211101 | 1.012407762 |
| fbn2 | -0.0122688948 | 0.03347239856 | -0.3665376646 | 0.7139639128 | 0.9878060612 |
| ulbp2 | 0.01166340657 | 0.03195698625 | 0.3649720433 | 0.7151322751 | 1.011731689 |
| hmgcs1 | 0.01225229659 | 0.03366777553 | 0.3639176156 | 0.71591953 | 1.012327663 |
| dnaja1 | -0.0122923757 | 0.03379158554 | -0.3637703146 | 0.7160295316 | 0.9877828669 |
| pms1 | 0.01220409312 | 0.03359109249 | 0.363313373 | 0.7163708047 | 1.012278867 |
| rbfox3 | 0.01393267081 | 0.03842603865 | 0.3625841043 | 0.7169155863 | 1.014030183 |
| cd302 | -0.01216383237 | 0.0335778991 | -0.3622571006 | 0.7171599129 | 0.987909848 |
| bgn | -0.01165134785 | 0.03217906577 | -0.3620784996 | 0.71729337 | 0.9884162662 |
| ufd1 | 0.01205056526 | 0.03334976905 | 0.3613387921 | 0.717846198 | 1.012123466 |
| tff1 | -0.01218280162 | 0.03373905115 | -0.3610890409 | 0.7180328854 | 0.9878911083 |
| igf2r | 0.01176995102 | 0.03261455246 | 0.3608803474 | 0.7181888955 | 1.011839489 |
| btla | -0.01232993575 | 0.03424400962 | -0.3600610994 | 0.7188014427 | 0.9877457665 |
| tnfsf12 | -0.0114279426 | 0.0317480822 | -0.3599569424 | 0.7188793332 | 0.9886371083 |
| map1lc3b2 | 0.01215880465 | 0.03380989596 | 0.3596226578 | 0.7191293372 | 1.012233023 |
| tslp | -0.0113538638 | 0.03163004175 | -0.3589582301 | 0.7196263372 | 0.9887103481 |
| aplp1 | -0.01160769947 | 0.03246961342 | -0.3574942308 | 0.7207218444 | 0.98845941 |
| aif1 | 0.01151754202 | 0.03228142205 | 0.3567854601 | 0.721252422 | 1.011584124 |
| btn3a2 | -0.01125934065 | 0.03158022986 | -0.3565313076 | 0.7214427104 | 0.9888038085 |
| gkn1 | 0.01135927592 | 0.03186917856 | 0.3564345375 | 0.7215151683 | 1.011424037 |
| mtss1 | 0.01155410569 | 0.03260951019 | 0.3543170574 | 0.7231012859 | 1.011621112 |
| paep | 0.01254849857 | 0.03547448276 | 0.3537330947 | 0.7235389181 | 1.012627561 |
| itih3 | 0.0117091803 | 0.03311430903 | 0.3535988109 | 0.7236395656 | 1.011778001 |
| c7orf50 | 0.01192156195 | 0.0337307134 | 0.3534334363 | 0.7237635227 | 1.011992907 |
| vcan | 0.01116194828 | 0.03169547965 | 0.3521621508 | 0.7247166603 | 1.011224475 |
| lyn | 0.01093450652 | 0.03106065979 | 0.3520371621 | 0.7248103928 | 1.010994507 |
| galnt3 | 0.01112403247 | 0.03176027529 | 0.3502498756 | 0.7261511791 | 1.011186135 |

| Protein | beta | se | z | p_val | HR |
|---|---|---|---|---|---|
| cda | 0.01195719652 | 0.03416263539 | 0.3500080245 | 0.7263326754 | 1.01202897 |
| cpa2 | -0.01096738866 | 0.0313682828 | -0.3496330585 | 0.7266140978 | 0.9890925339 |
| ly75 | 0.01084199963 | 0.03108998496 | 0.3487296517 | 0.7272922813 | 1.010900987 |
| vit | -0.01293022292 | 0.03708557363 | -0.3486591052 | 0.7273452492 | 0.9871530133 |
| il18bp | 0.01139000311 | 0.03277613572 | 0.3475090292 | 0.7282089363 | 1.011455116 |
| cd79b | -0.01099727931 | 0.0316821751 | -0.3471125097 | 0.7285067958 | 0.9890629697 |
| col28a1 | 0.0118558138 | 0.0341654792 | 0.3470114886 | 0.7285826878 | 1.011926373 |
| ninj1 | -0.01136473227 | 0.03280716686 | -0.3464100485 | 0.7290345744 | 0.9886996024 |
| lama4 | 0.01101803978 | 0.0318712255 | 0.3457049299 | 0.7295644789 | 1.011078962 |
| dnlz | -0.01161770591 | 0.03361599985 | -0.3456004867 | 0.7296429801 | 0.988449519 |
| plxdc1 | -0.01114317712 | 0.03224454999 | -0.3455832728 | 0.7296559186 | 0.9889186781 |
| klk3 | 0.01740016005 | 0.05042445652 | 0.3450738243 | 0.7300388714 | 1.017552425 |
| ncln | -0.01090257497 | 0.03166185455 | -0.3443441683 | 0.7305874714 | 0.9891566427 |
| pon2 | -0.01090998851 | 0.0316840806 | -0.3443365975 | 0.7305931644 | 0.9891493096 |
| lypd1 | -0.01075871727 | 0.03124969235 | -0.3442823419 | 0.7306339627 | 0.9892989507 |
| cd8a | 0.01060509457 | 0.03086364864 | 0.3436111749 | 0.7311387195 | 1.010661528 |
| lpa | -0.01146276434 | 0.03336119922 | -0.3435956921 | 0.7311503649 | 0.9886026828 |
| tnfrsf13b | 0.01077083055 | 0.03172125967 | 0.339546117 | 0.7341983619 | 1.010829045 |
| mfap3l | -0.01124798341 | 0.03320696478 | -0.3387236228 | 0.7348179431 | 0.9888150386 |
| ap2b1 | 0.01156135763 | 0.0341737802 | 0.3383107623 | 0.7351290142 | 1.011628448 |
| f12 | 0.01141350551 | 0.03375736106 | 0.3381041986 | 0.7352846667 | 1.011478888 |
| sh3bp1 | -0.01138658748 | 0.03373159355 | -0.3375644694 | 0.7356914213 | 0.9886779944 |
| crisp3 | 0.01125718459 | 0.03343879194 | 0.3366504569 | 0.736380415 | 1.011320785 |
| hyou1 | -0.01044674339 | 0.03107911293 | -0.3361338985 | 0.7367698968 | 0.9896076343 |
| rtbdn | -0.01086287673 | 0.03232101398 | -0.3360933148 | 0.7368004995 | 0.9891959113 |
| fam3d | 0.01135204011 | 0.03378557546 | 0.3360025678 | 0.7368689301 | 1.011416719 |
| lelp1 | 0.01131658838 | 0.03384200161 | 0.334394771 | 0.7380816847 | 1.011380863 |
| ptges2 | 0.01158444931 | 0.03469813833 | 0.3338637135 | 0.7384824026 | 1.011651809 |
| mmp13 | -0.01060701776 | 0.0318477494 | -0.3330539193 | 0.7390935825 | 0.9894490383 |
| tank | 0.01058719596 | 0.03182815082 | 0.3326362257 | 0.7394088949 | 1.010643439 |
| pth | -0.01176227028 | 0.03536824903 | -0.3325658069 | 0.7394620576 | 0.9883066348 |
| lrrc59 | 0.0110225204 | 0.03314709228 | 0.3325335541 | 0.7394864073 | 1.011083492 |
| bcl2l11 | -0.01040738395 | 0.03131766711 | -0.3323167052 | 0.7396501271 | 0.9896465855 |
| batf | -0.01111652176 | 0.03361809963 | -0.3306707364 | 0.7408932089 | 0.9889450384 |
| sirpb1 | 0.01052985303 | 0.03195967739 | 0.3294730701 | 0.7417981456 | 1.010585487 |
| ddx1 | -0.01091003835 | 0.03313556578 | -0.3292546268 | 0.7419632363 | 0.9891492603 |
| rbp5 | 0.01152111279 | 0.03510470063 | 0.3281928796 | 0.7427658312 | 1.011587736 |
| atf4 | -0.01101314291 | 0.033598071 | -0.3277909292 | 0.7430697462 | 0.9890472797 |
| ca11 | -0.01041513889 | 0.03179290337 | -0.327593198 | 0.7432192656 | 0.9896389109 |
| duox2 | -0.01033479273 | 0.03155601297 | -0.3275062899 | 0.7432849864 | 0.9897184277 |
| dtx3 | -0.01117976271 | 0.03433331285 | -0.3256243508 | 0.7447085862 | 0.9888824986 |
| gfral | 0.01154499285 | 0.03546302058 | 0.3255501832 | 0.7447647085 | 1.011611893 |
| tctn3 | 0.01124145192 | 0.03460492008 | 0.3248512607 | 0.7452936462 | 1.011304874 |
| nlgn1 | 0.01080849849 | 0.03327564234 | 0.3248171253 | 0.7453194826 | 1.010867121 |
| cpox | 0.01105722089 | 0.03405412782 | 0.3246954657 | 0.7454115667 | 1.011118578 |
| galnt10 | -0.01043237976 | 0.03220595845 | -0.3239270079 | 0.7459932961 | 0.9896218488 |
| siglec6 | 0.0101256431 | 0.0312621451 | 0.3238946997 | 0.7460177569 | 1.010177081 |
| ubqln3 | 0.01080248707 | 0.0333693621 | 0.3237247102 | 0.7461464614 | 1.010861045 |
| chgb | -0.01042434579 | 0.03225931617 | -0.3231421811 | 0.7465875665 | 0.9896297994 |
| pm20d1 | 0.01039841747 | 0.03218201736 | 0.3231126674 | 0.7466099172 | 1.010452669 |
| muc13 | -0.01033979473 | 0.03201587635 | -0.322958354 | 0.746726782 | 0.9897134772 |
| tnr | -0.01055558644 | 0.03269574381 | -0.3228428293 | 0.7468142752 | 0.9894999283 |
| creld1 | 0.01155068792 | 0.03582833927 | 0.3223897103 | 0.7471574785 | 1.011617655 |
| pcare | -0.01077742845 | 0.03360229465 | -0.3207348952 | 0.7484113007 | 0.98928044 |
| ddx25 | -0.01075245395 | 0.03353451918 | -0.3206383815 | 0.7484844479 | 0.989305147 |
| eif2s2 | 0.01083459248 | 0.03386930405 | 0.3198941574 | 0.749048567 | 1.010893499 |
| fkbpl | 0.010772422 | 0.03373395522 | 0.319334686 | 0.7494727329 | 1.010830653 |
| cd40lg | 0.009878946693 | 0.03096541643 | 0.3190316111 | 0.7497025421 | 1.009927905 |
| scgb2a2 | 0.01066389696 | 0.03347367551 | 0.318575621 | 0.7500483426 | 1.010720959 |
| clec4m | 0.01058628758 | 0.03324136615 | 0.3184672835 | 0.7501305078 | 1.010642521 |
| ctse | -0.0108149978 | 0.03400594189 | -0.3180325908 | 0.7504602155 | 0.989243274 |
| ror1 | 0.01012352552 | 0.0318786113 | 0.3175648221 | 0.7508150617 | 1.010174942 |
| atp1b3 | -0.01075383362 | 0.03390240621 | -0.3171997159 | 0.7510920655 | 0.9893037821 |
| hpgds | -0.01005329453 | 0.03172352869 | -0.3169034136 | 0.7513168918 | 0.9899970709 |
| ltbr | -0.01026677509 | 0.03244783107 | -0.3164086704 | 0.7516923367 | 0.9897857483 |
| brk1 | -0.0101248892 | 0.03203554206 | -0.3160517522 | 0.7519632272 | 0.9899261949 |
| smoc2 | 0.01100846742 | 0.0348435356 | 0.3159400226 | 0.752048033 | 1.011069284 |
| tspan15 | -0.01032514031 | 0.03299797966 | -0.3129021963 | 0.7543549689 | 0.989727981 |
| vwf | 0.009971185132 | 0.03187589521 | 0.312812709 | 0.7544229592 | 1.010021063 |
| islr2 | 0.009985103802 | 0.03202997889 | 0.3117424409 | 0.7552362709 | 1.010035121 |

| Protein | beta | se | z | p_val | HR |
| --- | --- | --- | --- | --- | --- |
| prkg1 | 0.01040134054 | 0.03337856481 | 0.3116173688 | 0.7553313327 | 1.010455623 |
| sez6 | -0.01069316726 | 0.0343375203 | -0.3114134967 | 0.7554862948 | 0.9893638014 |
| lag3 | -0.0102348242 | 0.03287973112 | -0.3112806539 | 0.7555872732 | 0.9898173734 |
| hif1a | -0.01063806524 | 0.03429149413 | -0.3102246055 | 0.7563901604 | 0.9894183189 |
| krt6c | 0.01060379479 | 0.03421771182 | 0.3098919893 | 0.7566430947 | 1.010660214 |
| pvalb | 0.009625687676 | 0.0310754259 | 0.3097523975 | 0.7567492536 | 1.009672164 |
| nhlrc3 | -0.01124247648 | 0.03644678362 | -0.3084627876 | 0.7577302118 | 0.988820484 |
| bmp6 | -0.009746692353 | 0.0316504265 | -0.3079482152 | 0.7581217369 | 0.9903006527 |
| farsa | -0.01036727138 | 0.03370401062 | -0.3075975586 | 0.7583885781 | 0.9896862835 |
| cd3d | 0.01033346109 | 0.03360179864 | 0.3075270227 | 0.7584422577 | 1.010387036 |
| gad1 | 0.01049369728 | 0.03423641371 | 0.3065069073 | 0.7592187215 | 1.010548949 |
| mepe | -0.01008169078 | 0.03291776029 | -0.3062690381 | 0.7593998113 | 0.9899689591 |
| gabra4 | 0.01035735295 | 0.03383069708 | 0.3061525136 | 0.7594885261 | 1.010411176 |
| krt17 | 0.01036252664 | 0.03394242374 | 0.3052971914 | 0.7601398148 | 1.010416404 |
| gbp1 | 0.01037443656 | 0.0339889487 | 0.3052296985 | 0.7601912149 | 1.010428438 |
| cpq | 0.01019539963 | 0.03351006381 | 0.3042488874 | 0.7609382823 | 1.01024755 |
| vtcn1 | -0.009840215667 | 0.03235008586 | -0.304178966 | 0.7609915488 | 0.9902080408 |
| ace2 | -0.01109444209 | 0.03648999526 | -0.3040406557 | 0.7610969176 | 0.9889668743 |
| col4a4 | 0.01001493121 | 0.03298127177 | 0.3036550948 | 0.7613906726 | 1.010065248 |
| ca5a | 0.01035087858 | 0.03412115791 | 0.3033566038 | 0.7616181135 | 1.010404634 |
| ap3b1 | 0.0100894448 | 0.03326329653 | 0.3033206524 | 0.7616455088 | 1.010140515 |
| tnfrsf6b | 0.01004363644 | 0.03314999639 | 0.3029754912 | 0.7619085395 | 1.010094243 |
| atp5f1d | 0.0102495209 | 0.03388585669 | 0.3024719426 | 0.7622923189 | 1.010302227 |
| cgref1 | -0.009569946901 | 0.03170438236 | -0.3018493404 | 0.7627669159 | 0.9904756993 |
| nmt1 | 0.0099334462 | 0.03294032832 | 0.3015588098 | 0.762988412 | 1.009982947 |
| lrtm2 | -0.01082983477 | 0.0359184956 | -0.3015113685 | 0.7630245823 | 0.9892285968 |
| pdgfrb | 0.009247378828 | 0.03070562045 | 0.3011624157 | 0.7632906481 | 1.009290268 |
| ephb6 | 0.009320212955 | 0.03097565632 | 0.3008883124 | 0.763499663 | 1.009363781 |
| siglec9 | 0.009536176881 | 0.03179943452 | 0.2998851088 | 0.7642647933 | 1.009581791 |
| gpr37 | 0.009669303589 | 0.0322630954 | 0.2997016706 | 0.7644047242 | 1.009716202 |
| myh7b | 0.01010101667 | 0.03371079387 | 0.2996374607 | 0.7644537068 | 1.010152204 |
| acta2 | -0.01099963434 | 0.03673720621 | -0.2994140131 | 0.7646241714 | 0.9890606404 |
| fshb | -0.01534515599 | 0.05130243515 | -0.2991116493 | 0.7648548581 | 0.984771981 |
| snu13 | -0.01018724458 | 0.0341145938 | -0.2986183753 | 0.7652312434 | 0.9898644696 |
| lamtor5 | -0.009932668891 | 0.03332047498 | -0.2980950571 | 0.7656306141 | 0.9901164971 |
| psg1 | 0.0092079833 | 0.03089151927 | 0.2980747959 | 0.7656460777 | 1.009250507 |
| efcab2 | -0.01000464852 | 0.03357571992 | -0.297972718 | 0.7657239862 | 0.9900452315 |
| arid3a | 0.0100533076 | 0.03375250482 | 0.2978536748 | 0.7658148461 | 1.010104012 |
| lrp2bp | -0.009961823386 | 0.03349301936 | -0.2974298399 | 0.7661383649 | 0.9900876312 |
| clpp | 0.009267275633 | 0.03118035328 | 0.2972152224 | 0.7663022009 | 1.00931035 |
| scrn1 | -0.009408771853 | 0.03170291519 | -0.2967793907 | 0.7666349408 | 0.9906353521 |
| cd74 | 0.01003880904 | 0.03385871975 | 0.2964910994 | 0.7668550633 | 1.010089367 |
| pepd | -0.009671540832 | 0.03289222145 | -0.2940373257 | 0.7687293816 | 0.9903750781 |
| thbd | 0.009537569728 | 0.03248154991 | 0.2936303764 | 0.7690403614 | 1.009583197 |
| dab2 | 0.009158294076 | 0.0311982787 | 0.2935512618 | 0.7691008231 | 1.00920036 |
| tmed10 | 0.01002449267 | 0.03417154669 | 0.2933578852 | 0.7692486128 | 1.010074906 |
| igsf9 | -0.01144189618 | 0.039054527 | -0.2929723406 | 0.7695432935 | 0.9886233134 |
| micall2 | 0.009811245937 | 0.03365145148 | 0.2915549108 | 0.7706269542 | 1.009859534 |
| ifnlr1 | -0.009572068312 | 0.03283408043 | -0.291528442 | 0.7706471945 | 0.9904735981 |
| bcl7b | -0.009663280035 | 0.03321590414 | -0.2909232876 | 0.7711099903 | 0.9903832594 |
| epha10 | -0.009199222566 | 0.03176873672 | -0.2895684096 | 0.7721464375 | 0.9908429608 |
| rgmb | -0.009166298467 | 0.03169035651 | -0.289245672 | 0.7723933835 | 0.990875584 |
| nbl1 | -0.009321687163 | 0.03225871044 | -0.2889665159 | 0.7726070014 | 0.9907216251 |
| treml1 | 0.009535319058 | 0.03307760897 | 0.2882711101 | 0.7731392197 | 1.009580925 |
| layn | 0.009529371527 | 0.03309297927 | 0.2879574984 | 0.7733792726 | 1.009574921 |
| a1bg | 0.0102008898 | 0.03550889314 | 0.2872770424 | 0.7739001997 | 1.010253096 |
| lilra6 | 0.00940920056 | 0.03278886569 | 0.2869632835 | 0.7741404339 | 1.009453606 |
| dhrs4l2 | -0.009606470561 | 0.03353279758 | -0.2864798423 | 0.7745106303 | 0.9904395242 |
| vsig10l | -0.009563311556 | 0.03342552064 | -0.2861080807 | 0.7747953427 | 0.9904822715 |
| il20ra | 0.009057471411 | 0.03169681739 | 0.2857533392 | 0.7750670484 | 1.009098614 |
| scgb3a2 | 0.009402793591 | 0.03301606775 | 0.2847944723 | 0.7758016074 | 1.009447139 |
| fgf7 | 0.009739596791 | 0.03426596852 | 0.2842352693 | 0.7762300886 | 1.009787181 |
| fgfbp1 | 0.009140730218 | 0.03216338465 | 0.2841967758 | 0.7762595862 | 1.009182634 |
| neo1 | -0.00954942619 | 0.03361635907 | -0.2840708053 | 0.7763561197 | 0.9904960248 |
| il4r | -0.008993279654 | 0.0317477036 | -0.283273391 | 0.7769672732 | 0.9910470389 |
| naprt | 0.009585632888 | 0.03387049231 | 0.283008372 | 0.7771704193 | 1.009631722 |
| anxa10 | -0.009110872981 | 0.03234064447 | -0.2817158758 | 0.7781613802 | 0.9909305053 |
| caly | 0.009352948129 | 0.03339313236 | 0.2800859778 | 0.7794115424 | 1.009396824 |
| il6r | 0.008875911457 | 0.03169976912 | 0.2799992462 | 0.7794780832 | 1.008915419 |
| il2rg | -0.009362088208 | 0.0334778675 | -0.2796500765 | 0.7797459837 | 0.9906815997 |

| Protein | beta | se | z | p_val | HR |
|---|---|---|---|---|---|
| rabepk | 0.008863560571 | 0.03179283178 | 0.2787911637 | 0.780405096 | 1.008902958 |
| dll4 | 0.009452185967 | 0.03395714207 | 0.2783563454 | 0.780738827 | 1.009496999 |
| tnfrsf11a | 0.009296323305 | 0.03349615439 | 0.2775340475 | 0.7813700661 | 1.009339668 |
| dpp6 | 0.00906245061 | 0.03268687852 | 0.2772504143 | 0.7815878313 | 1.009103639 |
| ncr1 | 0.008708030523 | 0.03143804934 | 0.2769901665 | 0.7817876568 | 1.008746056 |
| il12a_il12b | -0.008804861159 | 0.0319224667 | -0.2758202003 | 0.7826861677 | 0.9912337881 |
| rspo3 | 0.009275391201 | 0.03365810095 | 0.2755767836 | 0.7828731434 | 1.009318541 |
| ssbp1 | -0.009171085539 | 0.03328643671 | -0.2755201951 | 0.7829166125 | 0.9908708406 |
| tk1 | -0.009149534962 | 0.03336166576 | -0.2742529413 | 0.7838902458 | 0.9908921947 |
| rbm17 | 0.009042152078 | 0.03307894108 | 0.2733507114 | 0.784583637 | 1.009083156 |
| igfbp1 | -0.009556243632 | 0.0350117911 | -0.2729435808 | 0.7848965853 | 0.9904892722 |
| acan | -0.009245063125 | 0.03398104494 | -0.2720652982 | 0.7855718114 | 0.9907975411 |
| lilrb5 | -0.008348691093 | 0.03078662692 | -0.2711791426 | 0.7862532539 | 0.9916860624 |
| clec7a | -0.008366433163 | 0.03089627678 | -0.2707909831 | 0.7865517953 | 0.991668468 |
| sox9 | -0.009016799505 | 0.03333130254 | -0.2705204662 | 0.7867598739 | 0.9910237299 |
| spint3 | 0.01456332913 | 0.0539168574 | 0.2701071582 | 0.7870778153 | 1.014669891 |
| artn | -0.008438917009 | 0.03125082511 | -0.2700382143 | 0.7871308545 | 0.9915965907 |
| timm8a | 0.008893557246 | 0.0331067845 | 0.2686324686 | 0.7882125241 | 1.008933222 |
| plau | 0.008562708553 | 0.0320225806 | 0.267395956 | 0.7891643128 | 1.008599473 |
| dtd1 | 0.008998794066 | 0.0337160339 | 0.2668995438 | 0.7895465079 | 1.009039405 |
| gstp1 | -0.008520648742 | 0.03199146676 | -0.2663412968 | 0.7899763711 | 0.9915155491 |
| axl | 0.008350028816 | 0.03149313538 | 0.2651380599 | 0.7909031086 | 1.008384988 |
| cdh3 | 0.008214461359 | 0.03098608435 | 0.2651016265 | 0.7909311744 | 1.008248293 |
| nudt2 | -0.008212403502 | 0.03111131612 | -0.2639683731 | 0.7918042892 | 0.9918212262 |
| scrg1 | -0.01000800785 | 0.03805028299 | -0.2630205892 | 0.7925347098 | 0.9900419056 |
| bag3 | -0.008395977666 | 0.03210051089 | -0.2615527739 | 0.7936662577 | 0.9916391701 |
| tmem25 | 0.008794263656 | 0.03365597109 | 0.2612987643 | 0.7938621195 | 1.008833047 |
| akr7l | -0.00925204494 | 0.03554238441 | -0.2603101928 | 0.7946245108 | 0.9907906235 |
| lect2 | 0.009953708118 | 0.03836977607 | 0.2594153299 | 0.7953148029 | 1.010003411 |
| caps | -0.00928085595 | 0.03584834317 | -0.2588921866 | 0.7957184269 | 0.9907620783 |
| csnk1d | 0.008758931449 | 0.0340369306 | 0.2573361139 | 0.7969193161 | 1.008797403 |
| acsl1 | 0.008613539912 | 0.03351288336 | 0.2570217495 | 0.7971619833 | 1.008650743 |
| ncam2 | 0.00834623225 | 0.03257122352 | 0.2562455858 | 0.797761211 | 1.008381159 |
| cgb3_cgb5_cgb8 | -0.009065054187 | 0.03546079683 | -0.2556359416 | 0.7982319629 | 0.9909759095 |
| csf3r | 0.008688825332 | 0.0340012788 | 0.2555440748 | 0.7983029065 | 1.008726683 |
| ppt1 | -0.008633964605 | 0.03382256572 | -0.2552723136 | 0.7985127822 | 0.991403201 |
| mtus1 | 0.008776413186 | 0.03446170091 | 0.2546715035 | 0.7989768276 | 1.008815039 |
| rgma | 0.007989972091 | 0.03144499948 | 0.2540935672 | 0.7994232729 | 1.008021977 |
| phospho1 | -0.008588311548 | 0.03382201491 | -0.2539266679 | 0.7995522119 | 0.9914484626 |
| nxph3 | -0.008921604574 | 0.035254489 | -0.2530629383 | 0.8002195778 | 0.9911180749 |
| cd300lg | 0.008175784248 | 0.03236421257 | 0.2526180493 | 0.800563381 | 1.008209297 |
| cd274 | -0.008039284858 | 0.03204298447 | -0.2508906393 | 0.8018986618 | 0.9919929438 |
| il17c | 0.007940872892 | 0.03166814069 | 0.2507527351 | 0.8020052861 | 1.007972485 |
| igfbpl1 | -0.008488540687 | 0.03390224532 | -0.2503828465 | 0.8022912937 | 0.9915473853 |
| grp | -0.008366461177 | 0.03341621149 | -0.2503713259 | 0.8023002022 | 0.9916684403 |
| thbs2 | 0.007955963365 | 0.03180464405 | 0.2501509954 | 0.8024705807 | 1.007987696 |
| ndufs6 | -0.008144298121 | 0.03256496016 | -0.2500939071 | 0.8025147277 | 0.9918887768 |
| amdhd2 | 0.008205755071 | 0.03285184384 | 0.2497806549 | 0.8027569808 | 1.008239515 |
| casp4 | 0.008201901154 | 0.03292451863 | 0.2491122572 | 0.8032739484 | 1.008235629 |
| arntl | -0.008467675189 | 0.03404255208 | -0.2487379668 | 0.8035634784 | 0.9915680746 |
| guk1 | 0.008442592331 | 0.03400018269 | 0.2483102049 | 0.8038944038 | 1.008478332 |
| cgn | 0.008368705406 | 0.03371619402 | 0.2482102636 | 0.8039717256 | 1.008403821 |
| fes | 0.008018145879 | 0.0323478608 | 0.2478725233 | 0.8042330398 | 1.008050377 |
| xcl1 | 0.007780698191 | 0.03141195267 | 0.2476986475 | 0.8043675784 | 1.007811046 |
| hbegf | -0.007776121679 | 0.03148567109 | -0.2469733504 | 0.8049288486 | 0.9922540341 |
| il12b | 0.007867481838 | 0.03186506385 | 0.2468999239 | 0.8049856752 | 1.007898512 |
| clec14a | -0.007961291891 | 0.03232618626 | -0.246279961 | 0.8054655211 | 0.9920703153 |
| tgfbr3 | -0.007568567426 | 0.03083357166 | -0.2454651543 | 0.8060962858 | 0.9924600021 |
| pdcd1 | 0.007604720776 | 0.03109451068 | 0.2445679513 | 0.8067909817 | 1.00763371 |
| lilrb2 | 0.007544868462 | 0.03100881956 | 0.2433136304 | 0.8077624461 | 1.007573403 |
| il1b | 0.007721696176 | 0.03187345673 | 0.2422610212 | 0.8085779148 | 1.007751585 |
| dtnb | 0.00899209632 | 0.03712972198 | 0.2421805454 | 0.8086402689 | 1.009032647 |
| atp1b2 | 0.008156183999 | 0.03370455646 | 0.2419905454 | 0.8087874893 | 1.008189536 |
| igdcc4 | -0.008439894975 | 0.03488790029 | -0.2419146726 | 0.8088462807 | 0.991595621 |
| sumf2 | 0.007618976029 | 0.03150656718 | 0.2418218395 | 0.8089182156 | 1.007648074 |
| lrtm1 | -0.008117221888 | 0.03381017832 | -0.2400821969 | 0.8102665355 | 0.9919156338 |
| tlr3 | 0.007521013253 | 0.03135353925 | 0.239877648 | 0.8104251093 | 1.007549367 |
| il22 | -0.008269463929 | 0.0345321051 | -0.2394717584 | 0.810739793 | 0.991764634 |
| kit | -0.007710299196 | 0.03221772291 | -0.2393185644 | 0.8108585713 | 0.9923193489 |
| cdk1 | -0.008114242878 | 0.03394416195 | -0.2390467878 | 0.8110693028 | 0.9919185887 |

| Protein | beta | se | z | p_val | HR |
|---|---|---|---|---|---|
| klrb1 | -0.007491355156 | 0.031431685 | -0.2383376887 | 0.811619192 | 0.9925366351 |
| pgr | -0.008002281631 | 0.03361244123 | -0.2380749907 | 0.8118229317 | 0.9920296514 |
| rab6a | 0.007665361617 | 0.03232702263 | 0.2371193198 | 0.8125642247 | 1.007694816 |
| ptprs | 0.007282125157 | 0.03090082039 | 0.2356612241 | 0.8136955612 | 1.007308704 |
| abo | 0.007810617929 | 0.03316645712 | 0.2354975058 | 0.8138226145 | 1.0078412 |
| rasgrf1 | -0.007875292518 | 0.03357330023 | -0.2345701037 | 0.8145424159 | 0.9921556364 |
| dclre1c | 0.007812989714 | 0.03330877588 | 0.2345624992 | 0.8145483188 | 1.007843591 |
| clmp | -0.008559210872 | 0.03652053034 | -0.2343671023 | 0.814699996 | 0.9914773149 |
| eri1 | 0.007895790554 | 0.03379446498 | 0.2336415315 | 0.8152632825 | 1.007927045 |
| clstn1 | -0.007372599695 | 0.03155657454 | -0.2336311784 | 0.8152713207 | 0.9926545113 |
| armcx2 | -0.00789568487 | 0.03383202802 | -0.2333790001 | 0.8154671185 | 0.9921354042 |
| anxa11 | 0.007296157448 | 0.03128888217 | 0.2331869003 | 0.8156162775 | 1.007322839 |
| atxn10 | 0.007315197007 | 0.03141183951 | 0.232880249 | 0.8158543958 | 1.007342018 |
| hsd11b1 | -0.007663277737 | 0.03296659922 | -0.2324558165 | 0.8161840007 | 0.9923660103 |
| prrt3 | -0.007970810765 | 0.03430267029 | -0.2323670635 | 0.8162529284 | 0.9920608719 |
| gga1 | 0.007347794169 | 0.03164599945 | 0.232187142 | 0.8163926641 | 1.007374855 |
| ccn1 | 0.007309171371 | 0.03149155976 | 0.2320993761 | 0.8164608295 | 1.007335949 |
| ptprn2 | 0.007220342756 | 0.03112177337 | 0.2320029347 | 0.8165357344 | 1.007246472 |
| pxdnl | 0.007708617127 | 0.03323345281 | 0.231953543 | 0.816574097 | 1.007738405 |
| il32 | -0.007389937934 | 0.03190256377 | -0.2316408797 | 0.8168169536 | 0.9926373005 |
| vwc2l | -0.008325142054 | 0.03598998325 | -0.2313183087 | 0.8170675241 | 0.991709416 |
| cdnf | -0.007337486198 | 0.03175720389 | -0.2310495037 | 0.8172763439 | 0.9926893674 |
| rpgr | 0.007737910038 | 0.03359759387 | 0.2303114344 | 0.817849776 | 1.007767925 |
| cd70 | 0.007447443133 | 0.03235503905 | 0.2301787713 | 0.8179528571 | 1.007475244 |
| cga | 0.0111095718 | 0.04841273778 | 0.2294762146 | 0.8184988054 | 1.011171512 |
| ngrn | 0.007852853598 | 0.03426037598 | 0.2292109579 | 0.8187049561 | 1.007883768 |
| fgfr2 | 0.007765095024 | 0.0339639335 | 0.2286276713 | 0.8191583156 | 1.007795322 |
| il2rb | -0.007150315426 | 0.03133108966 | -0.228217898 | 0.8194768479 | 0.9928751873 |
| sftpa1 | -0.00736118837 | 0.03231024058 | -0.2278283367 | 0.8197796964 | 0.9926658388 |
| rad23b | 0.007254710393 | 0.03190427168 | 0.2273899391 | 0.8201205429 | 1.00728109 |
| hs6st2 | 0.008109030876 | 0.03569609991 | 0.2271685394 | 0.8202926902 | 1.008141998 |
| dnm3 | 0.007842957405 | 0.03465402784 | 0.2263216686 | 0.8209512465 | 1.007873794 |
| msr1 | -0.007661335112 | 0.03400406138 | -0.2253064722 | 0.821740865 | 0.9923679381 |
| pdgfa | 0.006979504955 | 0.03104759223 | 0.2248002004 | 0.8221347102 | 1.007003918 |
| il34 | 0.007105896803 | 0.03161702917 | 0.2247490352 | 0.8221745157 | 1.007131204 |
| ctso | -0.007264842004 | 0.03234502043 | -0.2246046504 | 0.8222868469 | 0.9927614832 |
| nampt | 0.00709219597 | 0.03172818149 | 0.2235298601 | 0.823123146 | 1.007117405 |
| cdh17 | -0.007050211163 | 0.03162859642 | -0.2229062292 | 0.8236084882 | 0.9929745833 |
| havcr2 | -0.007382432163 | 0.03322096383 | -0.2222220945 | 0.8241409952 | 0.9926447511 |
| dkk3 | -0.007264392718 | 0.0329503458 | -0.2204648401 | 0.8255091523 | 0.9927619292 |
| il15 | 0.006853153787 | 0.03111718239 | 0.2202369643 | 0.82568661 | 1.00687669 |
| ace | -0.007202541548 | 0.03272577125 | -0.2200877557 | 0.8258028106 | 0.9928233346 |
| aoc1 | 0.006932494682 | 0.03154055021 | 0.2197962507 | 0.8260298397 | 1.00695658 |
| ptprk | -0.00743627404 | 0.03391896706 | -0.2192364534 | 0.8264658605 | 0.9925913066 |
| fcrl5 | -0.007117943576 | 0.03247303706 | -0.2191954994 | 0.8264977612 | 0.992907329 |
| cabp2 | -0.007317422773 | 0.03353716256 | -0.2181884875 | 0.8272822547 | 0.9927092844 |
| prtg | 0.006832439807 | 0.03136832638 | 0.2178133357 | 0.8275745536 | 1.006855834 |
| cd160 | 0.006677435887 | 0.03085381313 | 0.2164217388 | 0.8286590226 | 1.00669978 |
| bex3 | 0.007313506887 | 0.03383201264 | 0.2161712034 | 0.8288542992 | 1.007340316 |
| coch | -0.007438172283 | 0.03445951325 | -0.2158525058 | 0.8291027192 | 0.9925894225 |
| egfl7 | -0.007050734295 | 0.03286645724 | -0.2145267512 | 0.8301363081 | 0.9929740638 |
| arid4b | 0.006765366271 | 0.03155153442 | 0.2144227339 | 0.8302174148 | 1.006788303 |
| slk | -0.007133991022 | 0.03334629804 | -0.2139365219 | 0.8305965591 | 0.9928913955 |
| sell | -0.007213196694 | 0.03375903832 | -0.213667126 | 0.830806649 | 0.992812756 |
| lhb | 0.007494322871 | 0.03517635907 | 0.2130499878 | 0.831287973 | 1.007522476 |
| gsta3 | -0.007281098729 | 0.03454248596 | -0.2107867609 | 0.8330536697 | 0.9927453443 |
| cdh4 | 0.006984386593 | 0.03338032257 | 0.2092366417 | 0.8342635095 | 1.007008834 |
| spaca5_spaca5b | 0.007050153175 | 0.03375904864 | 0.2088374364 | 0.8345751456 | 1.007075064 |
| dand5 | 0.006983902546 | 0.0334670284 | 0.2086800914 | 0.8346979827 | 1.007008347 |
| dpp7 | -0.00651033693 | 0.03127527559 | -0.2081624161 | 0.8351021534 | 0.9935108094 |
| obp2b | 0.008265107733 | 0.03973040084 | 0.2080298099 | 0.8352056916 | 1.008299358 |
| ltb | -0.006971560903 | 0.03351607658 | -0.2080064737 | 0.8352239126 | 0.9930526841 |
| prg3 | 0.006858301482 | 0.03302760243 | 0.2076536284 | 0.8354994275 | 1.006881873 |
| tsc22d1 | 0.006958399533 | 0.03357157689 | 0.2072705597 | 0.8357985648 | 1.006982665 |
| igsf3 | 0.007130245766 | 0.03468761813 | 0.2055559346 | 0.8371378014 | 1.007155726 |
| prok1 | -0.008880407801 | 0.04323475414 | -0.2053997525 | 0.8372598136 | 0.9911589066 |
| heph | -0.007064296806 | 0.03440729916 | -0.2053139007 | 0.8373268842 | 0.9929605967 |
| col15a1 | 0.007236671906 | 0.03525316415 | 0.2052772306 | 0.8373555326 | 1.00726292 |
| anp32c | -0.006806934624 | 0.03316625438 | -0.205236761 | 0.8373871496 | 0.9932161801 |
| il25 | 0.006985936082 | 0.03407575352 | 0.2050119325 | 0.8375628022 | 1.007010395 |

| Protein | beta | se | z | p_val | HR |
|---|---|---|---|---|---|
| adgrd1 | 0.007793869683 | 0.03821965574 | 0.2039230739 | 0.8384136136 | 1.007824321 |
| clasp1 | 0.006781498725 | 0.03326944191 | 0.2038356623 | 0.8384819234 | 1.006804545 |
| fcrl3 | -0.0062553431 | 0.03083065153 | -0.2028936396 | 0.8392181658 | 0.9937641808 |
| pgf | -0.007453012683 | 0.03677540419 | -0.2026629713 | 0.8393984672 | 0.9925746921 |
| xrcc4 | 0.006408348011 | 0.03164263706 | 0.2025225647 | 0.8395082198 | 1.006428925 |
| eno3 | -0.007478268912 | 0.03692580586 | -0.2025214816 | 0.8395090664 | 0.9925496238 |
| septin7 | -0.006747430244 | 0.03335930144 | -0.2022653339 | 0.8397092994 | 0.9932752826 |
| tmem106a | 0.006726065214 | 0.03328802083 | 0.2020566271 | 0.839872455 | 1.006748736 |
| daam1 | 0.006664688449 | 0.03302513009 | 0.2018065767 | 0.8400679399 | 1.006686947 |
| fcamr | 0.007358012407 | 0.03655181464 | 0.2013036146 | 0.8404611764 | 1.007385149 |
| acrbp | -0.006733025962 | 0.03364394224 | -0.2001259517 | 0.8413820773 | 0.9932895901 |
| cd177 | 0.005994451334 | 0.03047088242 | 0.1967271985 | 0.8440410247 | 1.006012454 |
| spint2 | 0.006250514373 | 0.0319900654 | 0.195389234 | 0.8450882442 | 1.00627009 |
| thbs4 | 0.00647914892 | 0.03322791192 | 0.1949911549 | 0.8453998721 | 1.006500184 |
| kir2dl3 | 0.006122647524 | 0.03151253733 | 0.1942924323 | 0.8459469109 | 1.006141429 |
| fkbp1b | 0.006004197997 | 0.03098540419 | 0.193775042 | 0.8463520302 | 1.006022259 |
| tnf | 0.006394867192 | 0.03302755839 | 0.1936221599 | 0.8464717454 | 1.006415358 |
| havcr1 | 0.006832579202 | 0.03539705479 | 0.1930267714 | 0.8469380018 | 1.006855975 |
| nefl | 0.007225468776 | 0.03754830404 | 0.1924312951 | 0.8474043805 | 1.007251635 |
| apob | -0.006327578387 | 0.03309833023 | -0.1911751542 | 0.8483883686 | 0.9936923986 |
| fhip2a | -0.006430735835 | 0.03366078434 | -0.1910453354 | 0.8484900747 | 0.9935898971 |
| coq7 | 0.006529577664 | 0.03427612227 | 0.1904993106 | 0.8489178833 | 1.006550942 |
| dmd | -0.006630602339 | 0.03487028404 | -0.1901505113 | 0.8491911898 | 0.9933913316 |
| cacna1c | -0.006436232395 | 0.0339033756 | -0.1898404593 | 0.8494341504 | 0.9935844358 |
| palm | 0.00689605393 | 0.03647254383 | 0.1890752112 | 0.8500338696 | 1.006919886 |
| ern1 | 0.006453353061 | 0.03435514816 | 0.1878423877 | 0.8510002065 | 1.006474221 |
| cd83 | 0.006135431124 | 0.03268781465 | 0.187697807 | 0.8511135495 | 1.006154291 |
| prdx5 | -0.005847656229 | 0.03117925054 | -0.1875496084 | 0.8512297318 | 0.994169408 |
| tnc | 0.005881610611 | 0.03142405574 | 0.1871690484 | 0.8515280918 | 1.005898941 |
| igfbp4 | 0.006533452966 | 0.03503922397 | 0.1864611206 | 0.8520831654 | 1.006554843 |
| pdp1 | 0.005875861707 | 0.03175012674 | 0.1850657717 | 0.853177448 | 1.005893158 |
| efemp1 | 0.006406720872 | 0.03480824185 | 0.1840575833 | 0.8539682816 | 1.006427288 |
| bnip2 | 0.006130741068 | 0.03347385064 | 0.1831501591 | 0.8546802001 | 1.006149573 |
| fgf3 | 0.00620138177 | 0.0340161117 | 0.1823071909 | 0.8553416557 | 1.00622065 |
| sox2 | -0.006104733527 | 0.03356822271 | -0.1818604929 | 0.8556922095 | 0.9939138625 |
| slc13a1 | 0.005979627841 | 0.03308963797 | 0.1807099807 | 0.8565952243 | 1.005997542 |
| morf4l2 | -0.006040289505 | 0.03346205638 | -0.180511605 | 0.8567509446 | 0.9939779164 |
| tap1 | 0.005922878204 | 0.03294406119 | 0.1797859156 | 0.8573206412 | 1.005940453 |
| naaa | -0.00557277676 | 0.03109164987 | -0.1792370872 | 0.8577515438 | 0.9944427224 |
| cant1 | -0.005707583332 | 0.0319516232 | -0.1786320305 | 0.8582266423 | 0.994308674 |
| luzp2 | -0.006068289785 | 0.03410608217 | -0.1779239772 | 0.8587826802 | 0.9939500851 |
| smpdl3b | -0.005849323455 | 0.03292047104 | -0.1776804301 | 0.8589739552 | 0.9941677505 |
| rnf4 | -0.005911841187 | 0.03337792541 | -0.1771182934 | 0.859415473 | 0.9941055994 |
| prame | 0.005918509496 | 0.03347404451 | 0.1768089152 | 0.8596584861 | 1.005936058 |
| tdp1 | 0.006007377268 | 0.03406271582 | 0.1763622519 | 0.8600093584 | 1.006025458 |
| gata3 | 0.005974669867 | 0.0339401868 | 0.1760352676 | 0.8602662355 | 1.005992554 |
| ambp | -0.00580516302 | 0.03317713766 | -0.1749747998 | 0.8610994349 | 0.9942116544 |
| tp73 | -0.005735502115 | 0.03299174133 | -0.1738466017 | 0.8619860191 | 0.9942809145 |
| c1qtnf5 | -0.005863345065 | 0.03388397836 | -0.1730418136 | 0.8626185609 | 0.9941538108 |
| slc27a4 | -0.005470494194 | 0.03177633581 | -0.1721562305 | 0.863314707 | 0.9945444417 |
| crtam | -0.005401628811 | 0.03140438101 | -0.172002397 | 0.8634356446 | 0.9946129338 |
| was | 0.005413808961 | 0.03152994882 | 0.1717037028 | 0.8636704747 | 1.00542849 |
| hcg22 | 0.005794436411 | 0.03374746874 | 0.1716998823 | 0.8636734783 | 1.005811257 |
| ttr | 0.006011742528 | 0.03518749159 | 0.1708488516 | 0.8643426157 | 1.006029849 |
| cd300lf | 0.005236319565 | 0.03079276316 | 0.1700503309 | 0.8649705547 | 1.005250053 |
| pnliprp1 | 0.005580364522 | 0.03292993022 | 0.1694617779 | 0.8654334343 | 1.005595964 |
| cela2a | -0.005594915268 | 0.03313958213 | -0.1688287814 | 0.865931319 | 0.9944207071 |
| kcnc4 | -0.00568952745 | 0.03372247167 | -0.1687162052 | 0.8660198716 | 0.9943266273 |
| ly9 | -0.005289704412 | 0.03139748684 | -0.1684754082 | 0.8662092886 | 0.9947242614 |
| icoslg | -0.005230730063 | 0.03130744862 | -0.1670762165 | 0.8673100794 | 0.9947829264 |
| prdx6 | 0.005189206464 | 0.03106747427 | 0.1670302007 | 0.867346286 | 1.005202694 |
| sowaha | 0.005496733955 | 0.03302275459 | 0.1664529208 | 0.86780053 | 1.005511869 |
| tnfrsf21 | 0.005230936371 | 0.03151519091 | 0.1659814274 | 0.8681715662 | 1.005244642 |
| aldh2 | -0.005455859797 | 0.03308405898 | -0.1649090216 | 0.8690155914 | 0.9945589964 |
| serpine1 | -0.005162152958 | 0.03151827445 | -0.1637828545 | 0.8699020895 | 0.9948511481 |
| il3 | -0.005538422253 | 0.03382204463 | -0.1637518463 | 0.8699265009 | 0.9944768865 |
| xg | -0.006711467522 | 0.04107553594 | -0.1633933038 | 0.8702087748 | 0.9933110041 |
| slc9a3r2 | -0.005568958696 | 0.03418005626 | -0.1629300623 | 0.8705735008 | 0.9944465192 |
| draxin | 0.005127505488 | 0.03151130031 | 0.162719578 | 0.8707392315 | 1.005140674 |
| klrk1 | 0.005349815149 | 0.03288237926 | 0.1626955004 | 0.8707581899 | 1.005364151 |

| Protein | beta | se | z | p_val | HR |
|---|---|---|---|---|---|
| ltbp2 | -0.005988995083 | 0.03692251879 | -0.1622044021 | 0.8711448934 | 0.9940289032 |
| itih4 | -0.005684955997 | 0.03505126548 | -0.1621897503 | 0.871156431 | 0.9943311728 |
| maneal | -0.005432985653 | 0.03386434479 | -0.160433804 | 0.8725393628 | 0.9945817463 |
| hsd17b14 | -0.005332510158 | 0.03344544124 | -0.1594390733 | 0.8733229565 | 0.9946816824 |
| fcar | 0.004988295733 | 0.03134156664 | 0.1591591062 | 0.8735435216 | 1.005000758 |
| fam20a | -0.005782591334 | 0.0363825812 | -0.1589384574 | 0.8737173611 | 0.9942340957 |
| pf4 | 0.005241196763 | 0.03299707817 | 0.1588382079 | 0.8737963454 | 1.005254956 |
| csf2 | -0.005408085704 | 0.03434868396 | -0.157446664 | 0.8748928395 | 0.9946065117 |
| gamt | 0.005318171198 | 0.03378610504 | 0.1574070522 | 0.8749240559 | 1.005332338 |
| vat1 | -0.004967709581 | 0.03158021934 | -0.1573044673 | 0.8750048997 | 0.9950446091 |
| flt3lg | -0.00508776257 | 0.03239325592 | -0.1570624016 | 0.8751956689 | 0.9949251582 |
| btn2a1 | -0.005070643567 | 0.03233842961 | -0.1567993137 | 0.8754030138 | 0.9949421904 |
| st8sia1 | -0.005278230789 | 0.03372974226 | -0.1564859508 | 0.8756499924 | 0.9947356746 |
| apof | -0.005824119647 | 0.03739977251 | -0.1557260715 | 0.8762489458 | 0.9941928077 |
| notch3 | 0.005132767424 | 0.03310727689 | 0.1550344186 | 0.8767941832 | 1.005145963 |
| il31 | 0.005220280677 | 0.03372530171 | 0.1547882573 | 0.876988249 | 1.00523393 |
| agr3 | -0.005021256701 | 0.03245019482 | -0.1547373361 | 0.8770283946 | 0.9949913287 |
| chi3l1 | -0.005251998555 | 0.03394578938 | -0.1547172315 | 0.8770442448 | 0.9947617691 |
| glp1r | 0.00517226631 | 0.03360542743 | 0.1539116358 | 0.8776794093 | 1.005185666 |
| col4a1 | 0.004980591052 | 0.0324438604 | 0.1535141315 | 0.8779928471 | 1.004993015 |
| cdc123 | -0.005168272712 | 0.03374821617 | -0.1531421005 | 0.878286216 | 0.9948450598 |
| il16 | 0.005030845595 | 0.03285171586 | 0.1531379857 | 0.8782894609 | 1.005043522 |
| adgrf5 | 0.005185871051 | 0.03393048206 | 0.1528381189 | 0.8785259363 | 1.005199341 |
| aldh5a1 | 0.005104363772 | 0.03345951902 | 0.1525534115 | 0.8787504671 | 1.005117413 |
| shbg | 0.005810463255 | 0.03809095609 | 0.1525418066 | 0.8787596194 | 1.005827377 |
| cacnb1 | 0.005135302384 | 0.03371381685 | 0.1523204094 | 0.8789342284 | 1.005148511 |
| ndst1 | 0.004986823215 | 0.03306033136 | 0.1508400857 | 0.8801018642 | 1.004999278 |
| creg1 | -0.004899343554 | 0.03270551579 | -0.1498017517 | 0.8809210273 | 0.9951126387 |
| dll1 | 0.004834868856 | 0.03237123829 | 0.1493569326 | 0.8812719933 | 1.004846576 |
| dctn2 | -0.004604583932 | 0.03102759972 | -0.1484028405 | 0.8820248585 | 0.9954060009 |
| nek7 | -0.004895155291 | 0.03302310111 | -0.1482342701 | 0.8821578869 | 0.9951168065 |
| fndc1 | 0.005054079425 | 0.03422148773 | 0.1476873088 | 0.8825895479 | 1.005066873 |
| copb2 | -0.004946975924 | 0.03351544686 | -0.1476028634 | 0.8826561952 | 0.9950652402 |
| ephb4 | 0.004558393872 | 0.03105089697 | 0.1468039353 | 0.8832867784 | 1.004568799 |
| septin9 | 0.004585747786 | 0.03127508151 | 0.1466262457 | 0.8834270365 | 1.004596278 |
| map1lc3a | 0.004915918501 | 0.03354570949 | 0.1465438822 | 0.8834920508 | 1.004928021 |
| ighmbp2 | 0.004915357657 | 0.03372121406 | 0.1457645519 | 0.8841072604 | 1.004927458 |
| grk5 | 0.004591967195 | 0.03150303142 | 0.1457627088 | 0.8841087155 | 1.004602526 |
| htr1b | -0.004841964794 | 0.03323800194 | -0.1456755675 | 0.8841775099 | 0.9951697386 |
| gast | -0.004974616302 | 0.03416971677 | -0.1455855293 | 0.8842485922 | 0.9950377366 |
| lgals4 | 0.004795399064 | 0.03308425924 | 0.1449450335 | 0.8847542702 | 1.004806915 |
| ercc1 | -0.004904608143 | 0.0339702001 | -0.1443797248 | 0.8852006263 | 0.9951073998 |
| tp53 | 0.004657227856 | 0.03228656032 | 0.1442466404 | 0.8853057123 | 1.00466809 |
| col6a3 | 0.005002183767 | 0.03469540392 | 0.1441742479 | 0.8853628756 | 1.005014716 |
| tbr1 | -0.004817204805 | 0.0334310978 | -0.1440935273 | 0.8854266158 | 0.9951943793 |
| nfasc | 0.004794515827 | 0.03348800213 | 0.1431711515 | 0.8861550128 | 1.004806028 |
| ncs1 | 0.004789430936 | 0.03384978487 | 0.1414907349 | 0.8874822789 | 1.004800919 |
| adgrg1 | -0.004698092648 | 0.03329048004 | -0.1411242085 | 0.8877718194 | 0.9953129261 |
| cbx2 | 0.004748237581 | 0.03375424434 | 0.1406708304 | 0.8881299899 | 1.004759528 |
| sftpd | 0.004533363429 | 0.03231892775 | 0.1402696112 | 0.8884469737 | 1.004543655 |
| mdm1 | 0.0047310851 | 0.03375532732 | 0.1401581758 | 0.8885350166 | 1.004742294 |
| tgfbr2 | 0.004771451379 | 0.03422881144 | 0.1393986872 | 0.8891351097 | 1.004782853 |
| sprr1b | 0.004613660455 | 0.03315883045 | 0.1391382143 | 0.8893409313 | 1.00462432 |
| cd86 | 0.004628190603 | 0.033313055 | 0.1389302363 | 0.8895052775 | 1.004638917 |
| hla_dra | 0.004322665814 | 0.0314330683 | 0.1375196902 | 0.8906200301 | 1.004332022 |
| dennd2b | -0.004648135133 | 0.03388536918 | -0.1371723326 | 0.8908945795 | 0.9953626507 |
| ahcy | 0.004326366115 | 0.03164545905 | 0.1367136469 | 0.8912571421 | 1.004335738 |
| nfic | -0.004517571522 | 0.03316322473 | -0.1362223233 | 0.8916455282 | 0.9954926174 |
| bhmt2 | -0.004569842133 | 0.03356924109 | -0.1361318274 | 0.8917170671 | 0.9954405837 |
| ache | -0.004757841166 | 0.03512694162 | -0.1354470656 | 0.892258414 | 0.9952534594 |
| sparc | -0.004198305187 | 0.03102601978 | -0.1353156227 | 0.8923623336 | 0.9958104954 |
| cdh6 | -0.004261597288 | 0.03173707014 | -0.1342782201 | 0.8931825751 | 0.9957474704 |
| nptx2 | 0.005329716598 | 0.04003556 | 0.1331245672 | 0.8940948662 | 1.005343945 |
| mfap4 | -0.004590528541 | 0.03452567477 | -0.1329598501 | 0.8942251335 | 0.9954199918 |
| grn | 0.004242211647 | 0.03196351876 | 0.1327204204 | 0.8944144926 | 1.004251223 |
| lgals9 | 0.004715013483 | 0.03579934364 | 0.1317067019 | 0.8952162843 | 1.004726147 |
| morc3 | 0.00438279691 | 0.03339396257 | 0.1312451884 | 0.8955813499 | 1.004392415 |
| mucl3 | 0.004400896507 | 0.03354263434 | 0.1312030672 | 0.8956146697 | 1.004410595 |
| timp3 | 0.004085650483 | 0.03140308625 | 0.1301034698 | 0.896484565 | 1.004094008 |
| pla2g7 | 0.004090479323 | 0.03169741194 | 0.1290477384 | 0.8973198749 | 1.004098857 |

| Protein | beta | se | z | p_val | HR |
|---|---|---|---|---|---|
| atxn2l | 0.004325179812 | 0.03368132887 | 0.1284147614 | 0.8978207499 | 1.004334547 |
| ceacam18 | -0.004306510017 | 0.03356382982 | -0.1283080638 | 0.8979051839 | 0.9957027497 |
| ralb | -0.004291565394 | 0.03350159496 | -0.1281003307 | 0.8980695743 | 0.9957176302 |
| fgl1 | 0.004485451157 | 0.03518939277 | 0.1274660005 | 0.8985715813 | 1.004495526 |
| gzmh | -0.00414165417 | 0.03261314487 | -0.1269934006 | 0.8989456218 | 0.9958669107 |
| adipoq | 0.004962403366 | 0.0393548641 | 0.1260937747 | 0.8996576952 | 1.004974736 |
| lmnb1 | 0.004205639911 | 0.03356271513 | 0.1253069037 | 0.9002805869 | 1.004214496 |
| ddx39a | -0.004184676424 | 0.03370483243 | -0.1241565711 | 0.9011913074 | 0.9958240671 |
| clec10a | 0.00390122977 | 0.03143215347 | 0.1241158922 | 0.9012235153 | 1.003908849 |
| tnfsf8 | -0.004035700661 | 0.03285741997 | -0.1228246364 | 0.9022459649 | 0.9959724318 |
| nudt10 | -0.004069384916 | 0.03366975238 | -0.1208617417 | 0.9038005457 | 0.9959388838 |
| paip2b | 0.004040073687 | 0.03355632673 | 0.12039678 | 0.904168842 | 1.004048246 |
| clspn | 0.003755826705 | 0.03156492493 | 0.1189873479 | 0.9052853795 | 1.003762889 |
| tubb3 | -0.004000905496 | 0.03370503232 | -0.1187035057 | 0.9055102591 | 0.9960070875 |
| slc1a4 | -0.003998573003 | 0.03418857906 | -0.1169563963 | 0.906894607 | 0.9960094106 |
| mia | -0.003699225269 | 0.03163229215 | -0.1169445847 | 0.906903967 | 0.9963076084 |
| mrpl24 | -0.004009168764 | 0.03428928988 | -0.1169218954 | 0.9069219472 | 0.9959988572 |
| saa4 | -0.003948226902 | 0.03393863392 | -0.1163342907 | 0.9073876101 | 0.9960595571 |
| bdnf | 0.003830797546 | 0.03295779138 | 0.1162334424 | 0.9074675332 | 1.003838144 |
| ranbp2 | -0.003848201825 | 0.03355504201 | -0.1146832665 | 0.9086961778 | 0.996159193 |
| mmp7 | 0.003680351073 | 0.03236347596 | 0.1137192765 | 0.9094603312 | 1.003687132 |
| palld | -0.003858212968 | 0.03410257063 | -0.1131355466 | 0.9099230938 | 0.9961492204 |
| gpr101 | 0.003794334187 | 0.03360592563 | 0.1129067007 | 0.9101045239 | 1.003801542 |
| psmc3 | -0.003748503496 | 0.03359287276 | -0.1115862738 | 0.911151456 | 0.9962585134 |
| cox6b1 | -0.003690483476 | 0.03331091823 | -0.1107890047 | 0.9117836646 | 0.996316318 |
| fstl3 | -0.004012424165 | 0.0362372552 | -0.1107264925 | 0.9118332372 | 0.9959956149 |
| il33 | 0.003465348203 | 0.03134320589 | 0.1105613834 | 0.9119641713 | 1.003471359 |
| ssb | -0.003484616806 | 0.03152061685 | -0.1105504002 | 0.9119728812 | 0.9965214474 |
| dpep1 | -0.003423064887 | 0.03142978768 | -0.1089114862 | 0.9132726962 | 0.9965827871 |
| sirt5 | 0.003395547474 | 0.03126217981 | 0.108615186 | 0.9135077154 | 1.003401319 |
| ca13 | 0.003383531235 | 0.03122998854 | 0.1083423784 | 0.9137241074 | 1.003389262 |
| creld2 | -0.003432736447 | 0.0317711349 | -0.108045761 | 0.9139593927 | 0.9965731487 |
| mamdc2 | 0.003659694484 | 0.03400649497 | 0.1076175151 | 0.9142991027 | 1.003666399 |
| prss8 | -0.003934875536 | 0.03657286744 | -0.1075900199 | 0.914320914 | 0.9960728559 |
| ccl15 | -0.003508144743 | 0.03271247128 | -0.1072418135 | 0.9145971443 | 0.9964980016 |
| siglec8 | 0.003740110548 | 0.03494307898 | 0.107034373 | 0.9147617107 | 1.003747113 |
| tigit | 0.003485872757 | 0.03297242404 | 0.1057208518 | 0.9158038357 | 1.003491955 |
| tdgf1 | -0.003288358343 | 0.0311563535 | -0.1055437487 | 0.9159443574 | 0.9967170424 |
| ssh3 | 0.003540354151 | 0.03365988865 | 0.1051802098 | 0.9162328139 | 1.003546629 |
| chrm1 | -0.003550029566 | 0.03386237289 | -0.1048369994 | 0.9165051505 | 0.9964562643 |
| lamp3 | 0.003554339254 | 0.03392614709 | 0.1047669588 | 0.9165607288 | 1.003560663 |
| il1a | 0.00325243102 | 0.0312104642 | 0.104209633 | 0.9170029896 | 1.003257726 |
| lsm8 | 0.003437524604 | 0.03313193848 | 0.1037525953 | 0.9173656868 | 1.00344344 |
| hla_a | 0.003413347077 | 0.03331769792 | 0.102448467 | 0.9184007149 | 1.003419179 |
| ece1 | -0.003231440744 | 0.03167901052 | -0.1020057347 | 0.9187521232 | 0.9967737747 |
| pkn3 | -0.003373213617 | 0.0333335353 | -0.1011957954 | 0.9193950344 | 0.9966324693 |
| defb116 | 0.003376097237 | 0.03362926399 | 0.1003916481 | 0.9200334001 | 1.003381803 |
| c1qtnf1 | 0.003193888396 | 0.03242419289 | 0.09850325053 | 0.921532691 | 1.003198994 |
| enpp2 | 0.003686812813 | 0.03744008409 | 0.0984723433 | 0.9215572321 | 1.003693617 |
| klrc1 | -0.003274706685 | 0.03359263625 | -0.09748287275 | 0.922342935 | 0.9967306493 |
| cd27 | 0.00308748879 | 0.03205334364 | 0.09632345457 | 0.9232636836 | 1.00309226 |
| tmem132a | 0.00324007517 | 0.03374935367 | 0.09600406579 | 0.9235173435 | 1.00324533 |
| fcn1 | -0.003300606199 | 0.03441169428 | -0.09591524825 | 0.9235878841 | 0.9967048348 |
| il17f | 0.00301496378 | 0.03158273331 | 0.09546240822 | 0.9239475478 | 1.003019513 |
| slc51b | -0.003243939732 | 0.03406927947 | -0.09521597706 | 0.9241432798 | 0.9967613162 |
| rgl2 | -0.003163166665 | 0.03353783519 | -0.09431636381 | 0.9248578514 | 0.9968418309 |
| gpc1 | -0.002971394275 | 0.03184572352 | -0.09330591194 | 0.9256605355 | 0.9970330159 |
| tnfrsf13c | 0.002976594877 | 0.03192819793 | 0.09322777575 | 0.9257226086 | 1.002981029 |
| araf | 0.00309840108 | 0.03339571207 | 0.0927784104 | 0.926079603 | 1.003103206 |
| gli2 | -0.003119696857 | 0.03365808303 | -0.09268789473 | 0.9261515142 | 0.9968851643 |
| dkkl1 | -0.00283129481 | 0.03070933404 | -0.09219655517 | 0.926541875 | 0.9971727095 |
| tnfsf11 | -0.002891613488 | 0.03179586593 | -0.09094306456 | 0.9275378314 | 0.9971125632 |
| cuzd1 | -0.003054493957 | 0.03360449563 | -0.09089539658 | 0.9275757081 | 0.9969501663 |
| psap | -0.003227688466 | 0.03553397309 | -0.09083387491 | 0.927624593 | 0.9967775149 |
| gorasp2 | 0.002934488002 | 0.033311753 | 0.08809167148 | 0.9298038158 | 1.002938798 |
| cntn3 | -0.003051678017 | 0.0351510822 | -0.08681604735 | 0.9308177318 | 0.9969529736 |
| scarb1 | -0.002690185217 | 0.03164209392 | -0.0850191907 | 0.932246134 | 0.9973134301 |
| colec12 | 0.003054398269 | 0.03596309498 | 0.08493146298 | 0.9323158783 | 1.003059068 |
| nectin1 | 0.002830020461 | 0.03357136523 | 0.08429864086 | 0.932818993 | 1.002834029 |
| scara5 | -0.002829277225 | 0.03356550135 | -0.08429122497 | 0.932824889 | 0.9971747214 |

| Protein | beta | se | z | p_val | HR |
|---|---|---|---|---|---|
| chrdl1 | 0.002980659208 | 0.03553576805 | 0.08387772014 | 0.9331536539 | 1.002985106 |
| ryr1 | 0.002778418947 | 0.03322318229 | 0.08362892281 | 0.9333514704 | 1.002782282 |
| ctbs | -0.002890771888 | 0.03462152453 | -0.0834963777 | 0.9334568575 | 0.9971134024 |
| epcam | -0.002615743095 | 0.03136974256 | -0.08338427037 | 0.9335459954 | 0.997387675 |
| smc3 | -0.002799339819 | 0.03358906517 | -0.08334080763 | 0.9335805533 | 0.9972045747 |
| pth1r | -0.002598724671 | 0.03144170056 | -0.08265216656 | 0.9341281202 | 0.9974046491 |
| rnaseh2a | 0.002767385308 | 0.03362981761 | 0.08228963179 | 0.9344163991 | 1.002771218 |
| gal | -0.002697564471 | 0.03291828484 | -0.08194729721 | 0.9346886232 | 0.9973060707 |
| ebi3_il27 | -0.002639542402 | 0.03248885256 | -0.08124455601 | 0.935247466 | 0.9973639381 |
| hmmr | 0.002651210391 | 0.03341457882 | 0.0793429241 | 0.9367598654 | 1.002654728 |
| amot | -0.002981666112 | 0.03791209814 | -0.07864682406 | 0.9373135426 | 0.9970227746 |
| vps37a | 0.002420738286 | 0.03095357676 | 0.07820544631 | 0.9376646298 | 1.002423671 |
| egflam | -0.002597497088 | 0.03339980196 | -0.07776983502 | 0.9380111419 | 0.9974058735 |
| ppm1b | -0.002625296346 | 0.03376682388 | -0.07774780228 | 0.9380286684 | 0.9973781467 |
| apoa1 | -0.002783631875 | 0.03594199635 | -0.07744789266 | 0.9382672423 | 0.9972202388 |
| ces2 | 0.002505428015 | 0.03254516301 | 0.07698311465 | 0.9386369777 | 1.002508569 |
| hspa2 | 0.002625081967 | 0.03416658731 | 0.07683184579 | 0.9387573163 | 1.002628531 |
| apod | -0.002752514669 | 0.03610111167 | -0.07624459584 | 0.9392245036 | 0.99725127 |
| steap4 | -0.002532348899 | 0.03385386451 | -0.07480235818 | 0.9403719655 | 0.9974708548 |
| c1ql2 | -0.002556042431 | 0.03500351002 | -0.07302246059 | 0.9417882443 | 0.9974472215 |
| hmox2 | 0.002366469797 | 0.03243527958 | 0.07295974715 | 0.9418381492 | 1.002369272 |
| tef | -0.002460215642 | 0.03412532524 | -0.07209354415 | 0.9425274639 | 0.9975428082 |
| ca12 | 0.002270290441 | 0.03157389566 | 0.07190403318 | 0.9426782803 | 1.00227287 |
| atp6v1g2 | -0.002438009757 | 0.033985983 | -0.07173574345 | 0.9428122103 | 0.9975649598 |
| c1galt1c1 | 0.002423616894 | 0.03387271196 | 0.07155071897 | 0.9429594601 | 1.002426556 |
| lair1 | 0.00244087435 | 0.03465580753 | 0.07043189942 | 0.9438499022 | 1.002443856 |
| lysmd3 | 0.002349137459 | 0.03347854769 | 0.07016843983 | 0.9440595938 | 1.002351899 |
| nppc | -0.002504688373 | 0.03575249209 | -0.07005632967 | 0.9441488251 | 0.9974984457 |
| igdcc3 | -0.002334758065 | 0.03343836201 | -0.06982274027 | 0.9443347472 | 0.9976679654 |
| pcsk9 | 0.002246334043 | 0.03238788553 | 0.06935723054 | 0.944705272 | 1.002248859 |
| angptl1 | 0.002243608857 | 0.03255532135 | 0.06891680881 | 0.9450558388 | 1.002246128 |
| fgf16 | 0.002314753885 | 0.03380003548 | 0.06848377085 | 0.9454005387 | 1.002317435 |
| nptx1 | 0.002299296846 | 0.03360182034 | 0.06842774656 | 0.945445135 | 1.002301942 |
| palm2 | -0.002668917803 | 0.03903701921 | -0.06836889335 | 0.9454919834 | 0.9973346406 |
| elob | -0.002317488039 | 0.03403912437 | -0.06808306857 | 0.9457195084 | 0.9976851953 |
| cdh15 | -0.002497524553 | 0.03708989545 | -0.06733706103 | 0.9463133734 | 0.9975055917 |
| jchain | 0.002109904276 | 0.03149067374 | 0.0670009252 | 0.9465809667 | 1.002112132 |
| kif20b | -0.002253561749 | 0.0336814945 | -0.06690800933 | 0.9466549368 | 0.9977489756 |
| angptl4 | -0.002162841779 | 0.03277091852 | -0.0659988147 | 0.9473787691 | 0.9978394955 |
| gtf2ird1 | 0.002170778954 | 0.03318300863 | 0.06541838863 | 0.9478408834 | 1.002173137 |
| bcl2 | 0.002260838193 | 0.03465052363 | 0.06524686951 | 0.9479774441 | 1.002263396 |
| ccl4 | -0.002089132659 | 0.03208472772 | -0.06511299323 | 0.9480840353 | 0.9979130481 |
| tpbgl | -0.00215565275 | 0.03320336117 | -0.06492272691 | 0.9482355253 | 0.997846669 |
| sgsh | 0.002208729842 | 0.0342782434 | 0.06443532758 | 0.9486236011 | 1.002211171 |
| ripk4 | 0.002161535001 | 0.0337338583 | 0.06407612737 | 0.9489096104 | 1.002163873 |
| tg | 0.002092403037 | 0.03274408414 | 0.06390171208 | 0.949048489 | 1.002094594 |
| enpep | 0.002113719806 | 0.0333026807 | 0.06346995984 | 0.9493922796 | 1.002115955 |
| f13b | 0.002120986346 | 0.03379251265 | 0.062764979 | 0.9499536536 | 1.002123237 |
| dgcr6 | -0.002127254954 | 0.03401755268 | -0.06253403865 | 0.9501375561 | 0.997875006 |
| pfdn6 | -0.00209146179 | 0.03391019057 | -0.06167649767 | 0.9508204565 | 0.9979107238 |
| cpa4 | -0.002086538079 | 0.03399335993 | -0.06138075444 | 0.9510559792 | 0.9979156372 |
| neb | -0.00208770549 | 0.03433963145 | -0.0607958036 | 0.9515218324 | 0.9979144723 |
| prap1 | -0.002348634696 | 0.03887618699 | -0.06041319578 | 0.9518265491 | 0.9976541212 |
| clec4a | -0.001927381439 | 0.03190420387 | -0.06041151964 | 0.9518278841 | 0.9980744748 |
| kiaa2013 | -0.001995194117 | 0.03313381965 | -0.06021624245 | 0.9519834096 | 0.998006795 |
| kdm3a | -0.002029570852 | 0.03392125229 | -0.05983183741 | 0.9522895684 | 0.9979724873 |
| cplx2 | 0.00199350164 | 0.03367894303 | 0.05919133619 | 0.9527997103 | 1.00199549 |
| ptprf | 0.001870736526 | 0.03216895947 | 0.0581534671 | 0.9536263858 | 1.001872487 |
| prc1 | 0.00193097539 | 0.03323253409 | 0.05810496982 | 0.9536650157 | 1.001932841 |
| cep152 | -0.001931314183 | 0.03362019451 | -0.05744506273 | 0.9541906674 | 0.9980705496 |
| clec4c | -0.001784579761 | 0.03114330535 | -0.05730219514 | 0.9543044718 | 0.9982170117 |
| cthrc1 | 0.0021942356 | 0.03851002205 | 0.05697830028 | 0.9545624809 | 1.002196645 |
| rapgef2 | -0.001900271238 | 0.03364502417 | -0.05648000812 | 0.9549594213 | 0.9981015331 |
| ptprr | -0.001896116848 | 0.03382798334 | -0.05605172585 | 0.9553006006 | 0.9981056796 |
| scarf2 | 0.001925836976 | 0.03528327026 | 0.05458215641 | 0.9564713547 | 1.001927693 |
| rnaset2 | 0.001741251134 | 0.03213516256 | 0.05418522875 | 0.9567875891 | 1.001742768 |
| tnfrsf10a | 0.00181714578 | 0.03360911772 | 0.05406704796 | 0.9568817458 | 1.001818798 |
| pla2g10 | 0.001764995554 | 0.03275041933 | 0.05389230398 | 0.9570209683 | 1.001766554 |
| ifit1 | -0.001753711922 | 0.03287546773 | -0.05334409039 | 0.9574577511 | 0.9982478249 |
| nxph1 | 0.001638753121 | 0.03191001106 | 0.05135545449 | 0.9590422801 | 1.001640097 |

| Protein | beta | se | z | p_val | HR |
|---|---|---|---|---|---|
| susd4 | -0.001739068272 | 0.03430341841 | -0.05069664636 | 0.959567249 | 0.998262443 |
| plxdc2 | 0.001706474309 | 0.03372021018 | 0.05060687047 | 0.959638788 | 1.001707931 |
| nlgn2 | -0.001707643735 | 0.03381286796 | -0.05050277714 | 0.9597217364 | 0.9982938135 |
| clec6a | 0.001607216478 | 0.03246709264 | 0.04950293813 | 0.9605184958 | 1.001608509 |
| pxn | 0.001606078372 | 0.0327863282 | 0.04898622261 | 0.9609302755 | 1.001607369 |
| csf1r | -0.001665391419 | 0.03426124958 | -0.04860860124 | 0.9612312153 | 0.9983359946 |
| tex33 | 0.001615335935 | 0.0333442988 | 0.04844414168 | 0.9613622806 | 1.001616641 |
| usp47 | 0.001647774537 | 0.03413367343 | 0.04827416366 | 0.961497745 | 1.001649133 |
| mst1 | 0.001605752421 | 0.03330890963 | 0.0482078951 | 0.9615505581 | 1.001607042 |
| nmrk2 | -0.00158540737 | 0.03367080023 | -0.0470855269 | 0.9624450624 | 0.9984158487 |
| stxbp3 | -0.001460840751 | 0.03170650937 | -0.04607384351 | 0.9632513937 | 0.9985402258 |
| fcer2 | -0.001451523904 | 0.03151358332 | -0.04606026198 | 0.9632622187 | 0.998549529 |
| entpd5 | 0.001482786103 | 0.03224119511 | 0.04599041995 | 0.9633178856 | 1.001483886 |
| agt | -0.00155659001 | 0.03385702162 | -0.045975397 | 0.9633298595 | 0.9984446208 |
| clec1a | -0.001443385457 | 0.03169904919 | -0.04553403002 | 0.963681651 | 0.9985576557 |
| il13 | -0.001434088318 | 0.03152619726 | -0.04548878211 | 0.9637177162 | 0.9985669395 |
| rest | 0.001548672778 | 0.03406213812 | 0.04546610587 | 0.9637357905 | 1.001549873 |
| cdkl5 | 0.001453878404 | 0.03320697189 | 0.04378232405 | 0.9650779169 | 1.001454936 |
| entpd6 | -0.001336102744 | 0.03100265744 | -0.04309639412 | 0.9656246937 | 0.9986647894 |
| shpk | 0.001428661754 | 0.03344922396 | 0.04271135723 | 0.9659316261 | 1.001429683 |
| crnn | 0.001430451986 | 0.03350503944 | 0.04269363685 | 0.965945752 | 1.001431476 |
| sirt1 | -0.001442607945 | 0.03380271437 | -0.04267728116 | 0.9659587901 | 0.9985584321 |
| cd58 | 0.001335548828 | 0.03160655945 | 0.04225543215 | 0.9662950735 | 1.001336441 |
| eddm3b | 0.002125764004 | 0.05060027963 | 0.04201091416 | 0.9664899976 | 1.002128025 |
| bcl7a | 0.001398157239 | 0.03332507191 | 0.04195511542 | 0.9665344793 | 1.001399135 |
| rnf149 | -0.001426579227 | 0.03452030793 | -0.04132579669 | 0.9670361678 | 0.9985744379 |
| esm1 | -0.001341202144 | 0.03255450933 | -0.04119865946 | 0.9671375224 | 0.9986596969 |
| epb41l5 | -0.001373185314 | 0.03344021038 | -0.04106389576 | 0.9672449573 | 0.9986277571 |
| jam3 | -0.001390908155 | 0.03393518034 | -0.04098720387 | 0.9673060971 | 0.9986100587 |
| mcts1 | 0.001322019989 | 0.03287099618 | 0.04021843397 | 0.9679189813 | 1.001322894 |
| fcrl6 | 0.001232459106 | 0.03089848973 | 0.03988735751 | 0.9681829303 | 1.001233219 |
| sh3bgrl2 | 0.001318643846 | 0.03338923344 | 0.03949308536 | 0.9684972663 | 1.001319514 |
| gprc5c | 0.00122988548 | 0.03307007955 | 0.03719027885 | 0.9703332896 | 1.001230642 |
| ptx3 | -0.001154076718 | 0.03120362394 | -0.03698534247 | 0.9704966928 | 0.998846589 |
| dkk1 | 0.00107109756 | 0.03138506748 | 0.03412761691 | 0.9727753862 | 1.001071671 |
| scn3b | 0.001144193298 | 0.0336156752 | 0.03403749267 | 0.9728472532 | 1.001144848 |
| pkd1 | 0.001203485579 | 0.03547082894 | 0.03392888226 | 0.9729338617 | 1.00120421 |
| top1 | 0.001132740924 | 0.03415944225 | 0.03316040455 | 0.9735466733 | 1.001133383 |
| lypla2 | 0.001082642434 | 0.03356846529 | 0.03225177036 | 0.9742712709 | 1.001083229 |
| siglec1 | 0.00104876976 | 0.03252359639 | 0.03224642649 | 0.9742755324 | 1.00104932 |
| ccl5 | 0.001002815937 | 0.03139677088 | 0.03194009795 | 0.9745198214 | 1.001003319 |
| sema7a | 0.001004624205 | 0.03156276029 | 0.03182941529 | 0.9746080885 | 1.001005129 |
| pglyrp4 | -0.001073940749 | 0.03396079939 | -0.03162295259 | 0.974772739 | 0.9989266357 |
| vwa5a | 0.001072342663 | 0.03394332964 | 0.03159214709 | 0.974797306 | 1.001072918 |
| arhgef10 | -0.001039377955 | 0.03290431081 | -0.03158789623 | 0.974800696 | 0.998961162 |
| fosb | 0.0009655480214 | 0.0315642068 | 0.03058996627 | 0.9755965442 | 1.000966014 |
| selenop | -0.00101802775 | 0.03331703935 | -0.03055576875 | 0.9756238171 | 0.9989824903 |
| alpi | 0.001018483824 | 0.03350823668 | 0.03039502896 | 0.9757520093 | 1.001019003 |
| uhrf2 | 0.001011558295 | 0.03330884719 | 0.0303690575 | 0.975772722 | 1.00101207 |
| spag1 | 0.001020426401 | 0.03408448039 | 0.02993815335 | 0.9761163775 | 1.001020947 |
| cdsn | -0.0009346021163 | 0.03127314571 | -0.02988513292 | 0.9761586628 | 0.9990658345 |
| dda1 | -0.001007894677 | 0.03376033448 | -0.02985440436 | 0.9761831697 | 0.9989926131 |
| crebzf | 0.0009986986812 | 0.03387071425 | 0.02948561031 | 0.9764772953 | 1.000999198 |
| snx5 | 0.0009846532039 | 0.03399021008 | 0.02896872957 | 0.9768895303 | 1.000985138 |
| angptl7 | -0.0009049136547 | 0.03210123795 | -0.02818936939 | 0.9775111159 | 0.9990954957 |
| oxt | -0.0009456169665 | 0.03397760707 | -0.02783059338 | 0.9777972654 | 0.99905483 |
| lilra2 | -0.0008820288769 | 0.03214060627 | -0.02744282013 | 0.9781065456 | 0.99911836 |
| mbl2 | 0.0009183327435 | 0.03365221115 | 0.0272889273 | 0.9782292883 | 1.000918755 |
| atp6ap2 | -0.0008731952546 | 0.03205472932 | -0.02724076207 | 0.9782677043 | 0.9991271859 |
| phldb1 | 0.000939924783 | 0.03491039742 | 0.02692392102 | 0.9785204142 | 1.000940367 |
| dhps | -0.0008964512419 | 0.03361825287 | -0.02666561066 | 0.9787264421 | 0.9991039505 |
| aida | 0.0008827159033 | 0.03337727806 | 0.02644661143 | 0.9789011166 | 1.000883106 |
| sil1 | -0.0008807089082 | 0.03392009506 | -0.02596422288 | 0.9792858748 | 0.9991196788 |
| cd7 | -0.0008668613327 | 0.03398408579 | -0.02550786089 | 0.9796498784 | 0.9991335143 |
| lair2 | 0.0007625374367 | 0.03121503256 | 0.02442853248 | 0.9805107895 | 1.000762828 |
| nell2 | -0.0007671192914 | 0.03212181921 | -0.02388156432 | 0.9809470796 | 0.9992331749 |
| c1qtnf6 | 0.0007768600549 | 0.03353071486 | 0.02316860998 | 0.9815157775 | 1.000777162 |
| dcdc2c | -0.0007246234631 | 0.03315096702 | -0.02185829037 | 0.9825609963 | 0.999275639 |
| rexo2 | 0.0006970479311 | 0.03329874065 | 0.02093316196 | 0.983298973 | 1.000697291 |
| nos3 | -0.0006981620031 | 0.0334012679 | -0.02090226051 | 0.9833236234 | 0.9993020817 |

| Protein | beta | se | z | p_val | HR |
|---|---|---|---|---|---|
| fgf9 | 0.0006942662792 | 0.03380472524 | 0.02053755131 | 0.9836145568 | 1.000694507 |
| cdh1 | 0.0006038004884 | 0.03283952269 | 0.0183863966 | 0.9853306045 | 1.000603983 |
| ctnna1 | 0.0005193713392 | 0.03360350915 | 0.01545586614 | 0.987668494 | 1.000519506 |
| mcfd2 | -0.0004774244105 | 0.03171697032 | -0.01505264865 | 0.9879901776 | 0.9995226895 |
| acy3 | 0.0005138868108 | 0.03467095778 | 0.01482182333 | 0.988174329 | 1.000514019 |
| dkk4 | -0.0004692419872 | 0.03241892481 | -0.01447432294 | 0.9884515644 | 0.9995308681 |
| esr1 | -0.0004703995692 | 0.03368673108 | -0.0139639423 | 0.9888587481 | 0.9995297111 |
| fbln2 | 0.0004772714681 | 0.03453134238 | 0.0138213992 | 0.9889724701 | 1.000477385 |
| cxadr | 0.0004320843744 | 0.03169365866 | 0.01363314911 | 0.9891226578 | 1.000432178 |
| ccn5 | 0.0004702342919 | 0.03685373656 | 0.01275947396 | 0.989819689 | 1.000470345 |
| sh2d1a | 0.0003910256882 | 0.0317163259 | 0.0123288457 | 0.9901632536 | 1.000391102 |
| il12rb2 | 0.0004039541285 | 0.03392247728 | 0.01190815533 | 0.9904988913 | 1.000404036 |
| fut8 | 0.0003620465354 | 0.03108555466 | 0.0116467774 | 0.9907074262 | 1.000362112 |
| upk3a | 0.0003123411947 | 0.03358254573 | 0.009300700347 | 0.9925792218 | 1.00031239 |
| serpinf1 | -0.0003289974646 | 0.03814491858 | -0.008624935556 | 0.9931183824 | 0.9996710566 |
| prg2 | -0.0002717407284 | 0.03322122607 | -0.008179732073 | 0.9934735908 | 0.9997282962 |
| bhlhe40 | 0.0002570561921 | 0.03399295429 | 0.007562043296 | 0.9939664199 | 1.000257089 |
| myh9 | -0.0002308573346 | 0.03299177152 | -0.006997421598 | 0.9944169109 | 0.9997691693 |
| cd2 | 0.0002383350732 | 0.03416345637 | 0.006976316173 | 0.9944337502 | 1.000238363 |
| atp1b4 | -0.0002253547424 | 0.03377176484 | -0.00667287432 | 0.9946758561 | 0.9997746706 |
| prdx1 | -0.0002077932721 | 0.03121264505 | -0.006657342618 | 0.9946882483 | 0.9997922283 |
| klrd1 | -0.000204517348 | 0.03198454292 | -0.006394255769 | 0.9948981568 | 0.9997955036 |
| fam3b | -0.0001977788292 | 0.03114706138 | -0.006349839131 | 0.9949335954 | 0.9998022407 |
| wasl | -0.0002049910012 | 0.03400095209 | -0.006028978268 | 0.9951896005 | 0.99979503 |
| rps10 | 0.0001591451872 | 0.03371339318 | 0.004720533065 | 0.9962335735 | 1.000159158 |
| pnlip | -0.000146049164 | 0.0327117564 | -0.004464730116 | 0.9964376726 | 0.9998539615 |
| c9 | 0.0001488796559 | 0.03361771253 | 0.004428607561 | 0.996466494 | 1.000148891 |
| fgfbp2 | 0.0001416094043 | 0.03365253729 | 0.004207985955 | 0.9966425229 | 1.000141619 |
| slamf1 | 0.0001226745062 | 0.03197483013 | 0.003836596026 | 0.9969388468 | 1.000122682 |
| cd6 | -0.0001172134623 | 0.03163669457 | -0.003704984476 | 0.9970438569 | 0.9998827934 |
| ugdh | -0.0001149389796 | 0.03369061513 | -0.003411602285 | 0.9972779405 | 0.9998850676 |
| cxcl6 | -0.0001019127437 | 0.03103654317 | -0.003283637072 | 0.9973800414 | 0.9998980924 |
| cd84 | 7.62551e-05 | 0.03133159576 | 0.002433807763 | 0.9980581043 | 1.000076258 |
| adgre1 | -8.12792e-05 | 0.03366584345 | -0.002414292671 | 0.998073675 | 0.9999187241 |
| timd4 | 7.36958e-05 | 0.03170085907 | 0.00232472435 | 0.99814514 | 1.000073698 |
| tjp3 | -6.83476e-05 | 0.03384677421 | -0.002019325024 | 0.9983888128 | 0.9999316547 |
| gimap7 | -6.247e-05 | 0.03351391226 | -0.001864000846 | 0.9985127434 | 0.999937532 |
| cd300e | -6.24254e-05 | 0.03379498432 | -0.001847180492 | 0.998526164 | 0.9999375765 |
| lilra3 | 3.82515e-05 | 0.03333461723 | 0.001147500599 | 0.9990844272 | 1.000038252 |
| cxcl13 | 1.24601e-05 | 0.03188000888 | 0.0003908441559 | 0.9996881515 | 1.00001246 |
| cxcl3 | -1.19194e-05 | 0.0312454208 | -0.0003814782126 | 0.9996956244 | 0.9999880806 |

## Supplementary Table 10. Associations between circulating plasma proteins and incident diabetic retinopathy (DR) in the UK Biobank

| Protein | beta | se | z | p_val | HR |
|---|---|---|---|---|---|
| gdf15 | 1.006358861 | 0.1202617667 | 8.368069826 | 5.85697e-17 | 2.735622078 |
| lgals4 | 0.7338664438 | 0.1057323548 | 6.940793527 | 3.89904e-12 | 2.083119321 |
| pla2g7 | -0.6672838664 | 0.09805423496 | -6.805252895 | 1.00872e-11 | 0.5131003359 |
| reg4 | 0.6432017666 | 0.09471464624 | 6.790943028 | 1.11403e-11 | 1.902562699 |
| spink4 | 0.6218935823 | 0.09765734752 | 6.368118714 | 1.91361e-10 | 1.86245141 |
| muc13 | 0.6023398902 | 0.09815937793 | 6.136345838 | 8.44412e-10 | 1.82638735 |
| lifr | 0.6094603502 | 0.1000319647 | 6.092656004 | 1.11053e-09 | 1.839438478 |
| gfra1 | 0.6054931766 | 0.09961643029 | 6.078246077 | 1.21504e-09 | 1.832155562 |
| reg1a | 0.5993995655 | 0.1015462102 | 5.902727085 | 3.57541e-09 | 1.821025066 |
| tff1 | 0.5814776245 | 0.0988727736 | 5.881069209 | 4.07625e-09 | 1.788679476 |
| cd276 | 0.5541359544 | 0.0952617157 | 5.816984823 | 5.99186e-09 | 1.740436518 |
| scara5 | 0.5791557319 | 0.09963718987 | 5.812646188 | 6.14929e-09 | 1.784531172 |
| havcr1 | 0.5974727121 | 0.1068381983 | 5.592313626 | 2.24064e-08 | 1.817519596 |
| ren | 0.6135307089 | 0.1100425922 | 5.575393098 | 2.46972e-08 | 1.846940911 |
| b3gnt7 | 0.5975952461 | 0.1088845955 | 5.48833601 | 4.05738e-08 | 1.817742317 |
| krt19 | 0.5440783762 | 0.1003656949 | 5.420959592 | 5.928e-08 | 1.723019675 |
| ctse | 0.5854909154 | 0.1102307393 | 5.311503115 | 1.08725e-07 | 1.795872391 |
| cdh17 | 0.5164529438 | 0.09747752819 | 5.298174394 | 1.16966e-07 | 1.676071972 |
| nfasc | 0.5544097975 | 0.1057474892 | 5.242770316 | 1.58183e-07 | 1.74091319 |
| cdh1 | 0.5583020438 | 0.106779647 | 5.228543634 | 1.70851e-07 | 1.747702457 |
| fam3b | 0.4690420272 | 0.08991860922 | 5.216295395 | 1.82537e-07 | 1.598462176 |
| heph | 0.5845821601 | 0.1121132879 | 5.214209405 | 1.84603e-07 | 1.794241123 |
| galnt7 | 0.486690308 | 0.09444124111 | 5.153366287 | 2.55852e-07 | 1.626922686 |
| efna4 | 0.5202770352 | 0.1023326925 | 5.084172247 | 3.69233e-07 | 1.682493695 |
| agr2 | 0.4953390923 | 0.09907848097 | 4.999461916 | 5.74905e-07 | 1.641054614 |
| fam3c | 0.4696796198 | 0.0953649613 | 4.925075346 | 8.43279e-07 | 1.599481669 |
| tnfrsf10a | 0.5022441793 | 0.1021315257 | 4.917621429 | 8.76021e-07 | 1.652425452 |
| dcn | 0.4848624612 | 0.09978241155 | 4.859197665 | 1.17862e-06 | 1.623951637 |
| spink1 | 0.4597787832 | 0.09541525768 | 4.818713425 | 1.44487e-06 | 1.5837236 |
| ncs1 | 0.4679802302 | 0.09799412024 | 4.775594996 | 1.79177e-06 | 1.596765835 |
| adgrg1 | 0.5340986547 | 0.1132623881 | 4.715587085 | 2.41015e-06 | 1.705909935 |
| pdcd1lg2 | 0.4499406139 | 0.09647402527 | 4.663852396 | 3.10344e-06 | 1.568219052 |
| tcn2 | 0.4459669508 | 0.09570046031 | 4.660029318 | 3.16164e-06 | 1.561999843 |
| tff3 | 0.4972072437 | 0.1066996492 | 4.659877023 | 3.16398e-06 | 1.644123218 |
| areg | 0.5085103529 | 0.1092707484 | 4.653673196 | 3.26073e-06 | 1.662812345 |
| cd27 | 0.4539068499 | 0.09789801241 | 4.636527736 | 3.54311e-06 | 1.57445133 |
| prss8 | 0.5174587585 | 0.1120997803 | 4.616055065 | 3.91103e-06 | 1.677758638 |
| layn | 0.4384948536 | 0.09547819564 | 4.592617725 | 4.3772e-06 | 1.550371925 |
| klk4 | 0.426452251 | 0.09310168421 | 4.580499855 | 4.63866e-06 | 1.531813383 |
| ca12 | 0.4235275301 | 0.0926712622 | 4.570214325 | 4.87226e-06 | 1.527339801 |
| fabp4 | 0.5331797655 | 0.1171824756 | 4.549995746 | 5.3647e-06 | 1.704343113 |
| cpa2 | 0.4171935744 | 0.09218846032 | 4.525442479 | 6.02693e-06 | 1.517696272 |
| lgals3 | 0.4330440511 | 0.0963278636 | 4.495522219 | 6.93994e-06 | 1.541944144 |
| fabp1 | 0.4510861569 | 0.1003459649 | 4.495309376 | 6.94689e-06 | 1.570016544 |
| c7 | 0.4562406319 | 0.1018827784 | 4.478093739 | 7.53125e-06 | 1.578130047 |
| pigr | 0.4715381062 | 0.106299348 | 4.435945422 | 9.16691e-06 | 1.602457048 |
| plxnb2 | 0.4330386507 | 0.09792163052 | 4.422298203 | 9.76565e-06 | 1.541935816 |
| hspb6 | 0.4884138594 | 0.1104989756 | 4.420075903 | 9.86662e-06 | 1.629729189 |
| tnfrsf12a | 0.4625572032 | 0.1046585013 | 4.419681129 | 9.88466e-06 | 1.588129968 |
| creg1 | -0.4351509989 | 0.09876610065 | -4.40587404 | 1.05358e-05 | 0.6471669382 |
| reg1b | 0.4248831698 | 0.09673008325 | 4.392461533 | 1.12074e-05 | 1.529411728 |
| apoa4 | 0.4757767645 | 0.1085485647 | 4.383077435 | 1.17014e-05 | 1.609263731 |
| nefl | 0.4630074957 | 0.1063741367 | 4.352632229 | 1.34513e-05 | 1.588845252 |
| mmp12 | 0.428559813 | 0.09868342539 | 4.34277399 | 1.40695e-05 | 1.535045179 |
| wfdc2 | 0.4673433715 | 0.1080117992 | 4.32678073 | 1.51304e-05 | 1.595749244 |
| npy | 0.4081310242 | 0.09615376323 | 4.244566312 | 2.19017e-05 | 1.504004209 |
| xg | 0.4317057929 | 0.1018109865 | 4.240267262 | 2.23254e-05 | 1.539882004 |
| ccl15 | 0.417264389 | 0.09842707767 | 4.239325183 | 2.24193e-05 | 1.517803751 |
| tff2 | 0.4256110904 | 0.1006004019 | 4.230709642 | 2.32955e-05 | 1.530525423 |
| rtn4r | 0.4632334364 | 0.1096598327 | 4.224276336 | 2.3971e-05 | 1.589204277 |
| fgfbp1 | -0.4351015948 | 0.1032598491 | -4.213657084 | 2.51269e-05 | 0.6471989117 |
| cd74 | 0.4349746704 | 0.1039468683 | 4.184586583 | 2.85686e-05 | 1.544923926 |
| col6a3 | 0.4189966964 | 0.1022298264 | 4.098575836 | 4.157e-05 | 1.520435332 |
| pla2g10 | 0.4191968204 | 0.1034195492 | 4.053361514 | 5.04869e-05 | 1.520739638 |
| fabp3 | 0.4775989797 | 0.1183944461 | 4.033964392 | 5.48436e-05 | 1.612198829 |
| tnfrsf13b | 0.3862801652 | 0.09626835408 | 4.01253526 | 6.00701e-05 | 1.471496875 |
| tmprss15 | 0.3955492914 | 0.09865652743 | 4.009357533 | 6.08842e-05 | 1.485199774 |
| tnfsf12 | -0.3907828502 | 0.09881725402 | -3.954601391 | 7.66624e-05 | 0.6765270478 |

| Protein | beta | se | z | p_val | HR |
|---|---|---|---|---|---|
| umod | -0.4019817215 | 0.1017123617 | -3.952142245 | 7.74547e-05 | 0.6689929738 |
| dkk4 | 0.3923984142 | 0.09945696601 | 3.945409055 | 7.96639e-05 | 1.480527457 |
| ces2 | 0.3941570464 | 0.1003733523 | 3.926909258 | 8.60444e-05 | 1.483133451 |
| fgf23 | 0.4105675047 | 0.104852221 | 3.915677709 | 9.01506e-05 | 1.507673154 |
| fam3d | 0.4145289821 | 0.1064113345 | 3.895534099 | 9.79826e-05 | 1.513657613 |
| mmp7 | 0.3587942882 | 0.09249003796 | 3.879274958 | 0.0001047682766 | 1.431602274 |
| eda2r | 0.438454399 | 0.1132154724 | 3.87274274 | 0.0001076174049 | 1.550309206 |
| smoc2 | 0.382577052 | 0.09912684894 | 3.859469519 | 0.0001136334291 | 1.466057833 |
| scg2 | 0.3444254377 | 0.08942134662 | 3.851713833 | 0.0001172940447 | 1.411178877 |
| tnfrsf11a | 0.3928303078 | 0.1025235319 | 3.831611149 | 0.0001273068373 | 1.481167025 |
| nbl1 | 0.3724463624 | 0.09772312446 | 3.811240834 | 0.0001382709725 | 1.451280634 |
| sncg | 0.3894873105 | 0.1022571471 | 3.808900615 | 0.0001395860454 | 1.476223755 |
| prap1 | 0.4482438874 | 0.1185359421 | 3.781501877 | 0.0001558850463 | 1.56556047 |
| pyy | 0.4003953657 | 0.1064083887 | 3.762817673 | 0.0001680096912 | 1.492414631 |
| selplg | 0.3604931189 | 0.09581918927 | 3.762222594 | 0.0001684100571 | 1.434036391 |
| timd4 | -0.3611763802 | 0.09639385357 | -3.746881848 | 0.0001790463667 | 0.696856076 |
| pcsk9 | 0.3654842549 | 0.09767212059 | 3.741950648 | 0.0001825973801 | 1.441211753 |
| pof1b | 0.4228649743 | 0.1130453995 | 3.740665046 | 0.0001835339789 | 1.526328189 |
| fgf5 | 0.3856126283 | 0.1032797761 | 3.733670258 | 0.0001887095239 | 1.470514925 |
| edil3 | 0.3758825378 | 0.1011786479 | 3.715038157 | 0.0002031730334 | 1.456276066 |
| scgn | 0.3435379987 | 0.09437213872 | 3.6402481 | 0.000272375464 | 1.409927097 |
| tnfrsf4 | 0.3486993547 | 0.09580068963 | 3.639841801 | 0.0002728055953 | 1.417223045 |
| cd207 | 0.3412149542 | 0.09404482844 | 3.628216031 | 0.0002853864195 | 1.406655575 |
| bcan | -0.3752198865 | 0.1037608824 | -3.616197913 | 0.0002989618353 | 0.6871381697 |
| dmp1 | -0.4288442943 | 0.1187128718 | -3.612449838 | 0.0003033177948 | 0.6512613264 |
| nrp1 | 0.3449159308 | 0.09600299983 | 3.592762012 | 0.0003271913917 | 1.41187122 |
| cgref1 | 0.3218329442 | 0.08959408372 | 3.592122726 | 0.0003279953078 | 1.379654278 |
| tnfrsf10b | 0.4069620598 | 0.1134714252 | 3.586471741 | 0.0003351823692 | 1.502247109 |
| eif5a | -0.3473286372 | 0.09697729375 | -3.581545986 | 0.000341567 | 0.7065730839 |
| igfbp3 | -0.3368918823 | 0.09436046079 | -3.570265337 | 0.0003566198078 | 0.7139860302 |
| spon2 | 0.3449033892 | 0.09668078677 | 3.567444998 | 0.0003604790129 | 1.411853513 |
| micb_mica | 0.3127195941 | 0.08797221 | 3.554754326 | 0.0003783324157 | 1.367138124 |
| aoc3 | 0.3439300494 | 0.09683699192 | 3.551639127 | 0.0003828395357 | 1.410479968 |
| dll1 | 0.3414313441 | 0.09679990776 | 3.527186668 | 0.000420000543 | 1.406959994 |
| cst3 | 0.3659986555 | 0.1037809336 | 3.526646398 | 0.0004208584233 | 1.441953304 |
| adgrg2 | -0.3881970853 | 0.1103952276 | -3.516429954 | 0.0004373920524 | 0.6782786514 |
| mamdc2 | 0.3514784458 | 0.09999333397 | 3.515018771 | 0.0004397229069 | 1.421167115 |
| ccl11 | 0.3626518628 | 0.1032076765 | 3.513807065 | 0.0004417335302 | 1.437135452 |
| ambp | 0.3445513581 | 0.09832928107 | 3.504056517 | 0.0004582280975 | 1.411356584 |
| klrb1 | 0.3360562852 | 0.09603982039 | 3.499134878 | 0.0004667704002 | 1.399417789 |
| dsc2 | 0.3369356957 | 0.09635626988 | 3.496769812 | 0.000470927997 | 1.400648993 |
| boc | 0.3474019826 | 0.0994352524 | 3.493750699 | 0.0004762855593 | 1.415385571 |
| gpnmb | 0.343598152 | 0.09836037254 | 3.493257936 | 0.0004771653693 | 1.410011911 |
| il1r1 | 0.3184010919 | 0.09122018163 | 3.490467638 | 0.0004821760069 | 1.374927623 |
| apoc1 | -0.3946019508 | 0.1134538155 | -3.478084444 | 0.0005050108041 | 0.6739482505 |
| epha2 | 0.3488897744 | 0.100647004 | 3.466469546 | 0.0005273414702 | 1.417492938 |
| mb | 0.374940503 | 0.1084308767 | 3.457875787 | 0.0005444523438 | 1.45490485 |
| tnfrsf1a | 0.3650263029 | 0.1063242188 | 3.43314352 | 0.0005966261903 | 1.440551898 |
| pik3ip1 | 0.3391475411 | 0.09892533359 | 3.428318397 | 0.0006073327137 | 1.403750441 |
| calb1 | 0.3246718331 | 0.09501513134 | 3.417053984 | 0.0006330272574 | 1.383576527 |
| reg3a | 0.314738143 | 0.09213772951 | 3.415952886 | 0.0006355924261 | 1.369900546 |
| scarf2 | 0.3435262887 | 0.1007457962 | 3.409832486 | 0.0006500279175 | 1.409910587 |
| wnt9a | 0.3440173325 | 0.1009329293 | 3.408375592 | 0.0006535087676 | 1.410603085 |
| igfbp4 | 0.3512500958 | 0.1032071519 | 3.403350343 | 0.0006656486825 | 1.420842628 |
| igfbp2 | 0.334999578 | 0.09848803563 | 3.401424101 | 0.0006703574065 | 1.397939795 |
| adamtsl2 | 0.3809811446 | 0.1120288114 | 3.40074254 | 0.0006720308956 | 1.463720006 |
| tfpi | -0.3358395432 | 0.0988386539 | -3.397856304 | 0.0006791608334 | 0.7147377811 |
| gcg | 0.3276515164 | 0.09692300136 | 3.380534154 | 0.0007234508325 | 1.387705296 |
| rnase1 | 0.3945997786 | 0.116897972 | 3.37559131 | 0.0007365724552 | 1.483790227 |
| ccl21 | 0.337895074 | 0.1003619556 | 3.366764548 | 0.0007605559423 | 1.40199339 |
| pcdh7 | 0.3692347697 | 0.1101562675 | 3.351917945 | 0.0008025380152 | 1.446627188 |
| ca3 | 0.3250706474 | 0.0978884472 | 3.3208275 | 0.000897509907 | 1.384128428 |
| slitrk6 | 0.3051628645 | 0.09213340402 | 3.312184845 | 0.0009257036006 | 1.356845967 |
| adam9 | 0.3734771357 | 0.1128814395 | 3.308578784 | 0.0009377079786 | 1.452777346 |
| lpa | 0.3484245329 | 0.1053485684 | 3.30734948 | 0.0009418331148 | 1.416833615 |
| palm2 | 0.4064770218 | 0.1232006459 | 3.299309179 | 0.0009692309742 | 1.501518639 |
| pdgfc | -0.3283458373 | 0.1000827575 | -3.280743311 | 0.00103533922 | 0.7201139344 |
| pon3 | -0.3618331539 | 0.1110585681 | -3.25803907 | 0.001121849597 | 0.6963985495 |
| xcl1 | 0.3151929633 | 0.09713098344 | 3.245030084 | 0.001174382162 | 1.370523746 |
| rbfox3 | 0.3878515228 | 0.1196258785 | 3.242204175 | 0.001186090145 | 1.473810941 |

| Protein | beta | se | z | p_val | HR |
|---|---|---|---|---|---|
| hsd11b1 | -0.3156221085 | 0.09756750155 | -3.234910226 | 0.001216809831 | 0.7293350076 |
| spon1 | 0.3080802412 | 0.09534183369 | 3.231322802 | 0.001232186976 | 1.360810178 |
| ccl25 | 0.309856095 | 0.09596041748 | 3.228999031 | 0.001242243159 | 1.363228925 |
| mybpc1 | 0.3723075303 | 0.1155529624 | 3.221964393 | 0.001273149562 | 1.451079164 |
| itih3 | 0.3218158794 | 0.09998691275 | 3.218580018 | 0.001288270209 | 1.379630734 |
| xpnpep2 | -0.3073763927 | 0.09562578976 | -3.214367102 | 0.001307324164 | 0.7353737595 |
| ccn4 | 0.3325130115 | 0.1036981075 | 3.206548504 | 0.001343376557 | 1.394468043 |
| ccn5 | 0.3536200634 | 0.1104092783 | 3.202811112 | 0.001360932123 | 1.424213973 |
| vmo1 | 0.3030061711 | 0.09482594067 | 3.195393254 | 0.00139640428 | 1.35392282 |
| dcbld2 | 0.3094216143 | 0.0972670897 | 3.181154235 | 0.001466895108 | 1.362636757 |
| chad | -0.3526232898 | 0.1113363785 | -3.167188431 | 0.001539205484 | 0.7028419112 |
| klk11 | 0.2920142533 | 0.09237865159 | 3.161057758 | 0.001571973244 | 1.339122104 |
| tgfbi | -0.302419009 | 0.09571886032 | -3.159450582 | 0.001580669043 | 0.7390283405 |
| fcn2 | -0.309824033 | 0.09822320494 | -3.15428552 | 0.001608915928 | 0.73357603 |
| slitrk2 | 0.3069071062 | 0.09746131085 | 3.149014758 | 0.001638219219 | 1.3592147 |
| sned1 | 0.3382140061 | 0.1074368039 | 3.148027435 | 0.001643762649 | 1.402440602 |
| afp | -0.3193339524 | 0.1018460541 | -3.135457287 | 0.001715864131 | 0.726632848 |
| cd4 | 0.3131555172 | 0.09994438802 | 3.133297661 | 0.001728540502 | 1.367734221 |
| cd5 | 0.2995868754 | 0.09581835756 | 3.12661251 | 0.001768328279 | 1.349301263 |
| vsig4 | 0.3214306289 | 0.1028128467 | 3.126366395 | 0.001769809011 | 1.379099333 |
| cthrc1 | 0.3635472074 | 0.116364744 | 3.124204076 | 0.001782867563 | 1.438422759 |
| rbp2 | 0.2740271764 | 0.08783271468 | 3.119875976 | 0.001809272009 | 1.315250546 |
| rabepk | -0.2944303782 | 0.09442766434 | -3.118052112 | 0.001820506114 | 0.7449558097 |
| col18a1 | 0.2903386136 | 0.093906748 | 3.091775829 | 0.001989630396 | 1.336880097 |
| ggt5 | 0.2754372019 | 0.08930887611 | 3.084096608 | 0.002041712838 | 1.31710639 |
| fstl1 | 0.3436066464 | 0.1123148317 | 3.059316755 | 0.002218424561 | 1.410023888 |
| il15 | 0.2925810269 | 0.09577938044 | 3.054739189 | 0.002252563159 | 1.339881299 |
| gpc5 | -0.2912127182 | 0.09563965895 | -3.044894988 | 0.002327615659 | 0.7473566848 |
| folr2 | 0.2940153852 | 0.09657814885 | 3.044326162 | 0.002332021609 | 1.341804547 |
| btn2a1 | 0.2875597385 | 0.09456040603 | 3.041016325 | 0.002357810462 | 1.333170231 |
| crisp3 | -0.3207614123 | 0.1055744621 | -3.038248133 | 0.002379579289 | 0.7255963487 |
| clec7a | 0.2759241079 | 0.09096023142 | 3.033458728 | 0.002417677792 | 1.317747854 |
| pard3 | -0.3062540818 | 0.1013486865 | -3.021786394 | 0.002512878038 | 0.7361995408 |
| scarb2 | 0.3137604737 | 0.1038885444 | 3.020164307 | 0.002526375913 | 1.368561891 |
| nell1 | -0.2725748138 | 0.0902593079 | -3.019908087 | 0.002528514054 | 0.7614164626 |
| tafa5 | 0.2767567516 | 0.09170275174 | 3.017976521 | 0.002544686193 | 1.318845525 |
| spock1 | -0.2859686321 | 0.0949301047 | -3.01241248 | 0.002591801604 | 0.7512861819 |
| bmper | 0.3263284004 | 0.108749518 | 3.000734224 | 0.002693295287 | 1.385870415 |
| il17c | 0.2891562051 | 0.0966690505 | 2.991197323 | 0.002778858661 | 1.335300293 |
| shbg | -0.3259397913 | 0.1089983338 | -2.990319026 | 0.002786862138 | 0.7218486477 |
| tpk1 | -0.3012732437 | 0.1009435877 | -2.984570397 | 0.002839768481 | 0.7398755788 |
| vasn | 0.278422728 | 0.09347541835 | 2.978566268 | 0.002896003989 | 1.321044522 |
| igdcc4 | 0.3047673142 | 0.1023525653 | 2.977622627 | 0.002904934122 | 1.356309372 |
| lcn15 | 0.317266374 | 0.1066166725 | 2.975766985 | 0.002922568319 | 1.373368353 |
| tnfrsf14 | 0.2888754677 | 0.09721140546 | 2.971621142 | 0.002962319816 | 1.334925477 |
| tgfbr2 | 0.3015049421 | 0.1015571728 | 2.968819768 | 0.002989458809 | 1.351891796 |
| ada2 | 0.2902740941 | 0.09805065354 | 2.960450376 | 0.003071896057 | 1.336793845 |
| hyal1 | 0.2857546218 | 0.09679199323 | 2.95225475 | 0.003154625532 | 1.330765874 |
| vsig2 | 0.3304846834 | 0.1121806254 | 2.946005 | 0.003219071962 | 1.391642471 |
| ogn | 0.2830597379 | 0.09612893814 | 2.944584049 | 0.003233890974 | 1.327184443 |
| chchd10 | 0.3587907302 | 0.1222989826 | 2.933718028 | 0.00334928328 | 1.43159718 |
| nectin2 | 0.2844347284 | 0.09714835402 | 2.927838884 | 0.003413268681 | 1.329010564 |
| nos1 | 0.2985954194 | 0.102287765 | 2.919170434 | 0.003509642806 | 1.347964153 |
| phospho1 | 0.2939882363 | 0.100881337 | 2.914198453 | 0.003566031349 | 1.341768119 |
| tfrc | 0.2752688757 | 0.09468744183 | 2.907131826 | 0.003647594452 | 1.316884706 |
| yap1 | 0.3380083141 | 0.1167260734 | 2.895739609 | 0.003782661293 | 1.402152161 |
| nectin4 | 0.2591972529 | 0.08951414309 | 2.895601119 | 0.003784330853 | 1.295889398 |
| ca9 | 0.2702888727 | 0.09346554594 | 2.891855709 | 0.003829737889 | 1.310342918 |
| b2m | 0.335385578 | 0.1162184338 | 2.885820837 | 0.003903943251 | 1.398479504 |
| cd160 | 0.264764213 | 0.09218934947 | 2.871960965 | 0.004079333531 | 1.30312368 |
| art3 | 0.2795525056 | 0.09740979893 | 2.869860206 | 0.004106533068 | 1.322537852 |
| dtx3 | 0.2849424046 | 0.09934031536 | 2.868346085 | 0.004126239072 | 1.329685442 |
| ckmt1a_ckmt1b | 0.2660942573 | 0.09289857543 | 2.864352398 | 0.004178628494 | 1.304858045 |
| s100p | 0.2931791466 | 0.1025387575 | 2.859203228 | 0.004247066008 | 1.340682948 |
| gpr37 | 0.2819900798 | 0.09865614033 | 2.858312508 | 0.004259007172 | 1.325765568 |
| mrc1 | 0.3239880391 | 0.1139556848 | 2.843105543 | 0.004467627319 | 1.382630769 |
| insl4 | 0.3152052063 | 0.1110487963 | 2.838438749 | 0.004533481942 | 1.370540526 |
| sftpa2 | -0.2652260406 | 0.09347729796 | -2.837331057 | 0.004549241504 | 0.7670325499 |
| igfbp7 | 0.2799769303 | 0.0989576798 | 2.829259243 | 0.004665588896 | 1.323099288 |
| gp1ba | -0.2582260944 | 0.09135055461 | -2.826760007 | 0.004702155365 | 0.7724205724 |

| Protein | beta | se | z | p_val | HR |
|---|---|---|---|---|---|
| il18bp | 0.2791936838 | 0.09877442587 | 2.826578654 | 0.00470481882 | 1.322063381 |
| mfap4 | 0.3025325191 | 0.1071072422 | 2.824575753 | 0.004734325559 | 1.353281683 |
| efna1 | 0.2623214844 | 0.09287944907 | 2.82432214 | 0.004738073703 | 1.299944387 |
| gart | -0.300898031 | 0.1068422644 | -2.816282794 | 0.004858288168 | 0.7401532416 |
| ddr1 | 0.2696399088 | 0.09577993384 | 2.815202496 | 0.004874650774 | 1.309492829 |
| fas | 0.2975324107 | 0.1060802701 | 2.804785569 | 0.005035006389 | 1.346532017 |
| cilp | 0.3122116018 | 0.1113150694 | 2.804755937 | 0.005035469249 | 1.366443804 |
| acta2 | 0.2934695941 | 0.1047418502 | 2.801837027 | 0.00508125355 | 1.341072402 |
| b4galt1 | 0.2707675792 | 0.09670499437 | 2.799933767 | 0.005111309285 | 1.310970338 |
| nagk | -0.3020236305 | 0.1079115508 | -2.798807247 | 0.005129174574 | 0.7393205942 |
| vstm2l | 0.2806913098 | 0.1005637641 | 2.791177442 | 0.005251667309 | 1.324044821 |
| pbxip1 | 0.3073781196 | 0.1101445124 | 2.790680288 | 0.005259739829 | 1.359855059 |
| plau | -0.2616570992 | 0.09402949309 | -2.782713068 | 0.005390645661 | 0.7697749349 |
| hmcn2 | 0.3033079252 | 0.1093202856 | 2.774488958 | 0.005528851084 | 1.354331433 |
| clmp | 0.2789678347 | 0.100590453 | 2.773303294 | 0.005549037658 | 1.321764828 |
| myl3 | 0.3107693194 | 0.1121837276 | 2.770181789 | 0.005602501387 | 1.364474427 |
| ahnak2 | 0.3040903886 | 0.1097761766 | 2.770094551 | 0.00560400222 | 1.355391562 |
| cd79b | 0.2754050058 | 0.09947030101 | 2.768715918 | 0.00562776817 | 1.317063985 |
| jam2 | 0.2659885724 | 0.09614567239 | 2.766516326 | 0.005665874775 | 1.304720149 |
| igsf3 | 0.296768321 | 0.1076281944 | 2.75734739 | 0.005827240701 | 1.345503538 |
| hmox1 | -0.2705280309 | 0.09859571151 | -2.743811336 | 0.00607304297 | 0.7629765127 |
| colec12 | 0.2880005721 | 0.105126194 | 2.739570046 | 0.006151960422 | 1.333758067 |
| vegfd | 0.2467895251 | 0.09033671287 | 2.731885158 | 0.00629730872 | 1.279909696 |
| clec3b | -0.291517992 | 0.1068131073 | -2.729234261 | 0.006348159011 | 0.7471285711 |
| il6st | 0.2604102944 | 0.09623500161 | 2.705983167 | 0.006810249789 | 1.297462319 |
| capg | 0.2718991892 | 0.1005604229 | 2.703838958 | 0.006854349985 | 1.312454685 |
| rnase6 | 0.2952254175 | 0.1092360018 | 2.70263844 | 0.006879153055 | 1.343429157 |
| ulbp2 | 0.2517411613 | 0.09355246407 | 2.690908934 | 0.007125764327 | 1.28626306 |
| plat | -0.2778928843 | 0.1034392588 | -2.686532052 | 0.007219802085 | 0.7573779442 |
| scgb1a1 | 0.2594810019 | 0.09659557505 | 2.686261786 | 0.007225645134 | 1.296257157 |
| phykpl | -0.2719498056 | 0.10129826 | -2.684644392 | 0.007260701311 | 0.7618925029 |
| guca2a | 0.2313832873 | 0.08624377256 | 2.682898491 | 0.007298714025 | 1.26034222 |
| cpm | 0.3007533016 | 0.1121465541 | 2.681788166 | 0.007322981427 | 1.350876041 |
| sorcs2 | 0.2558679124 | 0.09543627401 | 2.681034177 | 0.007339501946 | 1.291582115 |
| igsf8 | 0.2537055564 | 0.09478902216 | 2.676528891 | 0.007438915184 | 1.288792272 |
| tnfsf11 | -0.2693764053 | 0.1009046591 | -2.669613155 | 0.007593868103 | 0.7638556822 |
| fstl3 | 0.2748779298 | 0.1031039302 | 2.66602766 | 0.007675337762 | 1.316369976 |
| mybpc2 | 0.2981034532 | 0.1121795935 | 2.657376835 | 0.007875136022 | 1.347301163 |
| hla_e | 0.2589448761 | 0.09779335781 | 2.647877954 | 0.008099876134 | 1.295562386 |
| ncr1 | 0.2393469152 | 0.09077948234 | 2.636575017 | 0.008374768036 | 1.270419188 |
| adm | 0.2893765469 | 0.1099857751 | 2.631036119 | 0.008512498842 | 1.335594548 |
| prss53 | -0.2623994818 | 0.09977388747 | -2.629941445 | 0.008539957606 | 0.7692036794 |
| apoe | -0.2794159851 | 0.1062687053 | -2.629334613 | 0.008555213446 | 0.7562252593 |
| tnfsf13 | 0.252261727 | 0.09643136726 | 2.615971692 | 0.008897393373 | 1.286932818 |
| apom | -0.2624471787 | 0.1003520799 | -2.615263969 | 0.008915852023 | 0.7691669917 |
| pth1r | 0.2506708989 | 0.0960337163 | 2.610238451 | 0.009047913049 | 1.284887157 |
| crisp2 | -0.2731102796 | 0.1050440457 | -2.599959642 | 0.009323472467 | 0.7610088593 |
| lmod1 | 0.2860715827 | 0.110224117 | 2.59536289 | 0.009449109488 | 1.331187742 |
| hjv | 0.2966063071 | 0.1144300557 | 2.592031483 | 0.009541103973 | 1.345285566 |
| cd300e | 0.2583669833 | 0.09984411548 | 2.587703663 | 0.009661806059 | 1.294813907 |
| sel1l | 0.303070593 | 0.1172260457 | 2.585352011 | 0.009727962352 | 1.354010045 |
| pdgfra | 0.2527182922 | 0.09782631092 | 2.583336628 | 0.009784979738 | 1.287520521 |
| psg1 | 0.2497443143 | 0.09674663266 | 2.581426427 | 0.009839296148 | 1.283697152 |
| ctrl | -0.2434020237 | 0.09442290371 | -2.577785835 | 0.009943560489 | 0.7839562814 |
| snapin | -0.2548243768 | 0.0989683641 | -2.574806396 | 0.01002962097 | 0.7750526032 |
| angpt2 | 0.2700161286 | 0.1050633189 | 2.570032353 | 0.01016890176 | 1.309985579 |
| apof | -0.3230051249 | 0.1257348868 | -2.568937971 | 0.01020107154 | 0.7239701441 |
| klk12 | 0.2551705204 | 0.09945884488 | 2.565589021 | 0.01030007873 | 1.29068169 |
| cxcl12 | -0.2496611773 | 0.09736957869 | -2.564057282 | 0.01034564687 | 0.7790647032 |
| eps8l2 | 0.2467774337 | 0.09699915393 | 2.544119446 | 0.01095536148 | 1.27989422 |
| rnaset2 | 0.2544961643 | 0.1005955947 | 2.529893731 | 0.01140970783 | 1.289811604 |
| pkd1 | 0.2773815483 | 0.1101917738 | 2.517261849 | 0.01182708952 | 1.319669793 |
| fst | -0.2610855811 | 0.103749419 | -2.51650162 | 0.01185263552 | 0.770215001 |
| krt17 | 0.2611498637 | 0.1037983321 | 2.515935067 | 0.01187170526 | 1.298422237 |
| anxa5 | 0.2468847133 | 0.09867813137 | 2.501919218 | 0.01235221057 | 1.280031534 |
| faslg | -0.2505331959 | 0.1005720299 | -2.491082225 | 0.01273546429 | 0.7783856404 |
| ctrc | -0.2183105041 | 0.08765941572 | -2.490439873 | 0.01275850816 | 0.8038757961 |
| col4a1 | 0.2317404093 | 0.09391424971 | 2.467574516 | 0.01360319052 | 1.260792396 |
| enpep | 0.2455220644 | 0.09969481547 | 2.462736535 | 0.01378811923 | 1.278288488 |
| ncan | -0.2684758977 | 0.1090515805 | -2.461916612 | 0.0138196793 | 0.7645438499 |

| Protein | beta | se | z | p_val | HR |
|---|---|---|---|---|---|
| fabp2 | 0.2133108427 | 0.08693130726 | 2.453786207 | 0.01413609975 | 1.237769343 |
| myom3 | 0.2723081708 | 0.1111066969 | 2.450870906 | 0.01425110537 | 1.312991565 |
| acy3 | 0.2657753256 | 0.1084816636 | 2.449956212 | 0.01428735885 | 1.304441951 |
| il18r1 | 0.2464736511 | 0.1009655977 | 2.441164682 | 0.01463997701 | 1.279505469 |
| lair1 | 0.2525962688 | 0.1034958939 | 2.440640486 | 0.01466124218 | 1.287363423 |
| scgb3a2 | 0.2220950516 | 0.0914078275 | 2.429715897 | 0.0151106623 | 1.248690062 |
| sirpa | 0.2244486636 | 0.09243276102 | 2.428237144 | 0.01517241887 | 1.251632455 |
| adam23 | 0.2200346205 | 0.09086384321 | 2.421586109 | 0.0154529395 | 1.246119871 |
| fshb | 0.3806914198 | 0.1575464725 | 2.416375396 | 0.01567589053 | 1.463295992 |
| cd80 | 0.2594394128 | 0.1078202915 | 2.406220659 | 0.01611852565 | 1.296203248 |
| rgmb | 0.2198722213 | 0.09157826289 | 2.400921511 | 0.01635384386 | 1.245917519 |
| tpsab1 | 0.238970921 | 0.09953759865 | 2.400810591 | 0.01635880156 | 1.269941607 |
| crip2 | 0.2572015691 | 0.107186826 | 2.399563255 | 0.01641464348 | 1.293305791 |
| tnxb | -0.2362263672 | 0.09849227194 | -2.398425404 | 0.0164657299 | 0.7896019138 |
| ctsz | 0.2448429023 | 0.1022247192 | 2.395143799 | 0.0166138481 | 1.277420618 |
| tnfrsf19 | 0.2259927977 | 0.09449372264 | 2.391617045 | 0.01677433453 | 1.253566637 |
| cdon | 0.2258644873 | 0.09478973943 | 2.382794685 | 0.01718177409 | 1.253405801 |
| apcs | -0.2913161535 | 0.1222890317 | -2.382193639 | 0.01720984519 | 0.7472793857 |
| ntf3 | 0.2231178759 | 0.09405641549 | 2.372170732 | 0.01768391714 | 1.249967906 |
| sestd1 | -0.2110498512 | 0.08907748126 | -2.369284001 | 0.01782256205 | 0.8097336997 |
| myl4 | 0.2673074084 | 0.1134284699 | 2.356616542 | 0.01844227973 | 1.306441996 |
| krt5 | -0.2256201295 | 0.09605191874 | -2.348939329 | 0.01882697427 | 0.7980211888 |
| fgfr2 | 0.2378183625 | 0.1014141964 | 2.345020431 | 0.01902603697 | 1.268478769 |
| gc | -0.2389328189 | 0.1021112933 | -2.339925498 | 0.01928758702 | 0.7874677836 |
| slamf8 | 0.2164851233 | 0.092906297 | 2.330144783 | 0.01979850019 | 1.241704613 |
| eln | 0.2825852345 | 0.1219302812 | 2.317596842 | 0.02047124201 | 1.326554839 |
| ptgds | 0.2285748293 | 0.0986701468 | 2.316555075 | 0.02052798101 | 1.256807568 |
| itgbl1 | 0.2375714411 | 0.1025804458 | 2.315952511 | 0.02056086172 | 1.268165593 |
| lypla2 | -0.240920417 | 0.1041975732 | -2.312149983 | 0.02076941949 | 0.7859041685 |
| muc2 | 0.2360728126 | 0.1021227044 | 2.311658452 | 0.02079651274 | 1.266266507 |
| sez6l | 0.2271322371 | 0.09839335383 | 2.308410358 | 0.02097632345 | 1.254995814 |
| nlgn1 | 0.2361844829 | 0.1026337639 | 2.301235714 | 0.02137831122 | 1.266407919 |
| fn1 | -0.2511865002 | 0.1092660612 | -2.298851972 | 0.02151334646 | 0.7778772838 |
| cspg4 | 0.2366073449 | 0.1029999256 | 2.297160347 | 0.02160962427 | 1.266943548 |
| rgma | 0.2089897631 | 0.09112253881 | 2.293502418 | 0.02181909631 | 1.232432382 |
| tnfrsf1b | 0.2296843955 | 0.1003139796 | 2.289654907 | 0.02204132937 | 1.258202853 |
| ifi30 | 0.2827243604 | 0.1237344788 | 2.284927881 | 0.02231705689 | 1.32673941 |
| smoc1 | 0.2306251584 | 0.1009722853 | 2.284044158 | 0.02236893592 | 1.25938708 |
| ctsb | -0.2152578944 | 0.09429105833 | -2.282908881 | 0.02243573627 | 0.8063334645 |
| il19 | 0.225245949 | 0.09890802798 | 2.277327267 | 0.02276668986 | 1.252630762 |
| grn | 0.2220314011 | 0.09761046611 | 2.274667973 | 0.02292585515 | 1.248610585 |
| cd28 | 0.217683414 | 0.09575328252 | 2.273378085 | 0.02300340565 | 1.243193428 |
| mrpl46 | -0.2102068036 | 0.09249776352 | -2.272560931 | 0.02305265224 | 0.8104166315 |
| cd48 | 0.2147231556 | 0.0945906428 | 2.270025335 | 0.02320604589 | 1.239518696 |
| ctsc | -0.2157045597 | 0.09510449842 | -2.268079463 | 0.02332436383 | 0.8059733837 |
| lypd1 | -0.2093014688 | 0.09229613847 | -2.267716421 | 0.02334649634 | 0.8111506622 |
| agr3 | 0.2216626326 | 0.09779040365 | 2.266711501 | 0.02340785538 | 1.248150222 |
| ebi3_il27 | 0.2187981159 | 0.09675731033 | 2.261308372 | 0.02374016818 | 1.24457999 |
| sct | 0.2198216858 | 0.09732909131 | 2.25854041 | 0.02391198819 | 1.245854557 |
| ces1 | -0.2012094097 | 0.08924892075 | -2.25447443 | 0.02416633754 | 0.8177411707 |
| ccs | -0.2225377697 | 0.09871313948 | -2.254388533 | 0.02417173605 | 0.8004847721 |
| prdx1 | -0.2133211142 | 0.09467985594 | -2.253078146 | 0.02425422217 | 0.8078966684 |
| ace | 0.2262322276 | 0.1004199775 | 2.252860767 | 0.02426792929 | 1.253866814 |
| gpc1 | 0.213262204 | 0.09482610163 | 2.248982088 | 0.0245136357 | 1.237709141 |
| fcrl2 | 0.2158328907 | 0.09602725196 | 2.247621236 | 0.02460035227 | 1.240894997 |
| cntn1 | 0.222930114 | 0.09919886176 | 2.24730516 | 0.02462053131 | 1.249733232 |
| il7 | -0.2174223374 | 0.0968161739 | -2.245723299 | 0.02472173687 | 0.804590089 |
| mdk | 0.2261177654 | 0.1008104821 | 2.242998553 | 0.02489690782 | 1.253723302 |
| cd14 | 0.2210014701 | 0.0988565202 | 2.235578085 | 0.02537942143 | 1.247325264 |
| ckap4 | 0.2186808397 | 0.09794956575 | 2.232586107 | 0.0255762511 | 1.244434039 |
| bgn | 0.2099967357 | 0.09416481008 | 2.2300978 | 0.02574095089 | 1.233674033 |
| rnf149 | 0.2484970324 | 0.1114523817 | 2.229625143 | 0.02577233925 | 1.282097018 |
| hpgds | -0.2165095939 | 0.09716825867 | -2.228192589 | 0.02586767505 | 0.8053248087 |
| ceacam16 | 0.2385661348 | 0.1070900772 | 2.227714658 | 0.02589954894 | 1.269427657 |
| slamf1 | 0.2148415366 | 0.09652745607 | 2.225703912 | 0.02603402058 | 1.23966544 |
| sdc4 | -0.2062115184 | 0.09272575926 | -2.223886005 | 0.02615611487 | 0.8136609538 |
| pnpt1 | -0.2051783154 | 0.09229116039 | -2.223163243 | 0.02620479438 | 0.8145020652 |
| dsg2 | 0.2100806511 | 0.09450657932 | 2.222920908 | 0.02622113368 | 1.233777561 |
| cd38 | 0.2518154512 | 0.1134260062 | 2.220085672 | 0.02641295262 | 1.286358619 |
| mmp10 | 0.2100600648 | 0.09480076531 | 2.21580558 | 0.02670482014 | 1.233752163 |

| Protein | beta | se | z | p_val | HR |
|---|---|---|---|---|---|
| cga | 0.3099615711 | 0.1401096025 | 2.212279285 | 0.02694737353 | 1.36337272 |
| angptl7 | 0.2055005595 | 0.09327724681 | 2.203115621 | 0.02758660047 | 1.228139668 |
| spint3 | -0.3787292459 | 0.1719327469 | -2.20277552 | 0.02761057441 | 0.6847309813 |
| gzmb | -0.2116870603 | 0.09623452189 | -2.199699818 | 0.02782819972 | 0.8092178944 |
| anpep | 0.2117913254 | 0.09637654299 | 2.197540178 | 0.02798189029 | 1.235889959 |
| atp1b3 | 0.2397856851 | 0.1091889361 | 2.196062107 | 0.0280874983 | 1.270976732 |
| btn3a2 | 0.213110801 | 0.09708855072 | 2.195014752 | 0.02816253958 | 1.237521762 |
| mcam | 0.2121759033 | 0.0967080145 | 2.193984691 | 0.02823651016 | 1.236365347 |
| nomo1 | -0.217537354 | 0.09920843928 | -2.192730332 | 0.02832681402 | 0.8044975532 |
| nt5c1a | 0.2705401057 | 0.1234573132 | 2.191365572 | 0.02842534842 | 1.310672161 |
| hcg22 | 0.2416979083 | 0.1103034696 | 2.191208573 | 0.02843670248 | 1.273409448 |
| ptgr1 | 0.2296342079 | 0.1049404495 | 2.188233507 | 0.02865259759 | 1.258139708 |
| dipk1c | 0.2295684661 | 0.1049146167 | 2.188145687 | 0.02865899188 | 1.258056999 |
| idi2 | 0.2103927524 | 0.09619513838 | 2.187145379 | 0.02873191277 | 1.234162685 |
| itgb7 | 0.2028844283 | 0.09291961055 | 2.183440364 | 0.02900339615 | 1.224930893 |
| dsg4 | -0.2113380555 | 0.09701499743 | -2.178406031 | 0.02937582129 | 0.8095003646 |
| mln | 0.2082940681 | 0.09573594984 | 2.175714227 | 0.02957663574 | 1.231575283 |
| il4r | 0.2077213114 | 0.09578816724 | 2.168548761 | 0.03011695884 | 1.230870092 |
| cblif | 0.1986702142 | 0.091754315 | 2.165241103 | 0.03036922612 | 1.219779634 |
| fmr1 | -0.2108815241 | 0.09742921276 | -2.164458874 | 0.03042914964 | 0.8098700112 |
| clc | 0.2074980311 | 0.09593595466 | 2.162880766 | 0.0305503515 | 1.230595294 |
| tnfrsf9 | 0.2139469671 | 0.09892993924 | 2.162610922 | 0.03057111751 | 1.238556969 |
| tfpi2 | 0.2119510949 | 0.09814820661 | 2.159500435 | 0.03081136363 | 1.236087433 |
| csf1r | 0.2293345713 | 0.106432279 | 2.154746413 | 0.03118168369 | 1.25776278 |
| cd302 | 0.211651991 | 0.09824037447 | 2.154429807 | 0.03120648118 | 1.235717769 |
| nudt10 | 0.2344132863 | 0.108935877 | 2.151846506 | 0.03140944561 | 1.264166847 |
| tcl1a | -0.1979717605 | 0.09209825945 | -2.14957114 | 0.03158915333 | 0.8203930203 |
| pcdh12 | 0.2206939517 | 0.1032599974 | 2.137264741 | 0.03257646494 | 1.246941748 |
| maneal | -0.2338779289 | 0.1096044956 | -2.133835183 | 0.03285627474 | 0.7914584242 |
| asah2 | -0.2053614682 | 0.09628254194 | -2.132904513 | 0.03293256008 | 0.8143529005 |
| meltf | 0.2242760191 | 0.1053241953 | 2.129387444 | 0.03322221844 | 1.251416387 |
| cpox | -0.2248820804 | 0.1056147196 | -2.129268357 | 0.03323206424 | 0.798610385 |
| mfap3l | -0.2234703218 | 0.1051895316 | -2.124454005 | 0.03363219824 | 0.7997386263 |
| ccl28 | 0.2029970722 | 0.09563421852 | 2.122640571 | 0.0337839825 | 1.225068881 |
| sbsn | -0.2062370604 | 0.09722131314 | -2.121315314 | 0.03389527647 | 0.8136401715 |
| fgf16 | -0.2181999462 | 0.1028673127 | -2.121178638 | 0.03390677227 | 0.8039646759 |
| fabp9 | -0.1954851996 | 0.09216225865 | -2.121098186 | 0.03391354061 | 0.8224355158 |
| clec11a | -0.1939534198 | 0.0914492287 | -2.120886339 | 0.03393136859 | 0.8236962713 |
| coq7 | -0.2288731924 | 0.1079483352 | -2.120210487 | 0.03398829844 | 0.7954293936 |
| cd3e | 0.2201812924 | 0.1039247115 | 2.11866157 | 0.03411907834 | 1.246302655 |
| rad51 | 0.2296481151 | 0.1084134607 | 2.118262009 | 0.03415288421 | 1.258157206 |
| tnfsf10 | -0.2027898979 | 0.09574477757 | -2.118025683 | 0.0341728926 | 0.8164497612 |
| lyve1 | 0.2238816784 | 0.1057530511 | 2.117023349 | 0.03425786623 | 1.250922999 |
| lilrb4 | 0.2237674259 | 0.1057632264 | 2.115739407 | 0.03436697708 | 1.250780087 |
| timp4 | 0.1924089158 | 0.09105311523 | 2.113150278 | 0.03458790746 | 1.212166089 |
| notch2 | 0.2165022458 | 0.1026351789 | 2.109435071 | 0.03490704497 | 1.241725874 |
| epo | 0.2087930286 | 0.0990177541 | 2.108642339 | 0.03497546538 | 1.232189944 |
| hepacam2 | -0.2197861657 | 0.1046726423 | -2.099747947 | 0.03575101935 | 0.8026904223 |
| dand5 | 0.2158729859 | 0.1030840633 | 2.094145098 | 0.03624705676 | 1.240944752 |
| gata3 | -0.2248013196 | 0.1074916893 | -2.091336744 | 0.03649788819 | 0.7986748841 |
| omd | -0.2032957515 | 0.09721345525 | -2.091230591 | 0.03650739833 | 0.8160368616 |
| slc4a1 | 0.2318792226 | 0.1109878022 | 2.089231592 | 0.03668688086 | 1.260967423 |
| gdnf | 0.2121891171 | 0.1016346374 | 2.087763804 | 0.03681914619 | 1.236381684 |
| erbb4 | 0.1901100274 | 0.09107744545 | 2.087344747 | 0.03685698261 | 1.209382656 |
| angptl3 | -0.2012155692 | 0.09666003372 | -2.08168321 | 0.03737141528 | 0.8177361338 |
| pros1 | -0.2160525839 | 0.1037881133 | -2.081669827 | 0.03737263846 | 0.8056929343 |
| atraid | 0.2174026285 | 0.1044898582 | 2.080609854 | 0.03746963126 | 1.242844406 |
| bmp4 | 0.1993428042 | 0.09595075987 | 2.077553158 | 0.03775053436 | 1.220600321 |
| efemp1 | 0.2149727025 | 0.1035040139 | 2.076950394 | 0.0378061379 | 1.239828052 |
| plaur | 0.2170122568 | 0.1047905182 | 2.070915008 | 0.03836673943 | 1.242359329 |
| dpp6 | 0.1853362756 | 0.08954842729 | 2.0696765 | 0.03848264883 | 1.203623121 |
| slc9a3r2 | 0.2348148462 | 0.1138047679 | 2.063312905 | 0.03908290879 | 1.264674588 |
| fgf3 | -0.2211242915 | 0.1072402313 | -2.061952766 | 0.03921223312 | 0.8016170399 |
| ptprc | 0.2202823283 | 0.1068695615 | 2.06122609 | 0.03928147559 | 1.246428583 |
| serpine1 | -0.2012709929 | 0.09769328151 | -2.060233721 | 0.03937620267 | 0.8176908131 |
| cr2 | 0.191557603 | 0.09326137069 | 2.053986572 | 0.03997699142 | 1.211134596 |
| ccl7 | 0.2195371052 | 0.1069220537 | 2.053244374 | 0.04004888275 | 1.245500062 |
| rbp7 | 0.2318792863 | 0.1129967212 | 2.052088625 | 0.04016105018 | 1.260967504 |
| gp2 | 0.1794489848 | 0.08753525779 | 2.050019493 | 0.04036252867 | 1.19655786 |
| clul1 | -0.2097962134 | 0.1023572415 | -2.049647004 | 0.04039889006 | 0.810749449 |

| Protein | beta | se | z | p_val | HR |
|---|---|---|---|---|---|
| megf9 | -0.2007419194 | 0.09806572199 | -2.047014138 | 0.04065669641 | 0.8181235462 |
| vcam1 | 0.1919547 | 0.09388310733 | 2.044613834 | 0.04089294458 | 1.21161563 |
| hsd17b14 | 0.2140754963 | 0.1049882536 | 2.039042359 | 0.04144580019 | 1.23871617 |
| otoa | 0.2113869171 | 0.1037305854 | 2.037845599 | 0.04156537646 | 1.235390256 |
| sirpb1 | 0.1892501928 | 0.09299388575 | 2.035082105 | 0.04184261228 | 1.208343233 |
| gsr | -0.2113906844 | 0.1041361913 | -2.029944458 | 0.04236218536 | 0.8094577626 |
| klrd1 | 0.2035487502 | 0.1005218597 | 2.024920258 | 0.04287555303 | 1.225744911 |
| ghr | -0.22856407 | 0.1130755179 | -2.021340024 | 0.04324457869 | 0.7956753167 |
| ceacam20 | 0.2211029141 | 0.1094104863 | 2.020856698 | 0.04329460155 | 1.247451804 |
| tnfrsf6b | 0.2201294435 | 0.1089330787 | 2.020776848 | 0.04330287049 | 1.246238038 |
| ndufs6 | 0.2089590819 | 0.1035014912 | 2.018899239 | 0.04349769332 | 1.23239457 |
| kitlg | 0.1777407826 | 0.08818226514 | 2.015606906 | 0.04384109705 | 1.194515642 |
| ptprb | 0.2200119355 | 0.1091543389 | 2.015604122 | 0.04384138839 | 1.246091603 |
| neb | 0.2249154001 | 0.1116064579 | 2.015254352 | 0.04387800516 | 1.252216774 |
| ngfr | 0.2188940231 | 0.1086934763 | 2.013865327 | 0.04402367472 | 1.24469936 |
| ism1 | -0.1920464428 | 0.09556224697 | -2.009647627 | 0.04446849672 | 0.8252685398 |
| adamts16 | 0.1980650801 | 0.09867012533 | 2.007345987 | 0.04471283573 | 1.219041727 |
| siglec1 | 0.2010955206 | 0.1004068445 | 2.002806895 | 0.04519801949 | 1.222741563 |
| ccl5 | -0.1871657751 | 0.0934697414 | -2.002421022 | 0.04523946946 | 0.8293062467 |
| lcp1 | -0.209847638 | 0.1051076221 | -1.996502573 | 0.04587924594 | 0.8107077576 |
| omg | -0.1819683985 | 0.0913478436 | -1.99203825 | 0.04636686126 | 0.8336276839 |
| thy1 | 0.1927015399 | 0.09688603122 | 1.988950703 | 0.04670664453 | 1.21252085 |
| plb1 | -0.2231166617 | 0.1122994333 | -1.986801315 | 0.04694441889 | 0.800021512 |
| cntn4 | 0.1864646484 | 0.09390962523 | 1.985575472 | 0.04708048225 | 1.204982023 |
| pnliprp2 | 0.1820743959 | 0.09169953916 | 1.985554099 | 0.04708285742 | 1.199703444 |
| pon1 | -0.2041889082 | 0.1029955015 | -1.982503171 | 0.04742295288 | 0.8153083382 |
| lpcat2 | 0.1984331028 | 0.1001612223 | 1.981136993 | 0.04757591271 | 1.219490444 |
| ampd3 | 0.2127161122 | 0.1074885136 | 1.978965985 | 0.04781983606 | 1.237033423 |
| cd59 | 0.2009875522 | 0.1016271475 | 1.977695499 | 0.04796306837 | 1.222609553 |
| flrt2 | 0.177822133 | 0.08993861353 | 1.977150037 | 0.04802467329 | 1.19461282 |
| c2 | 0.197407386 | 0.1001540264 | 1.971037941 | 0.04871953622 | 1.218240234 |
| scp2 | -0.1897808802 | 0.09644404047 | -1.967782346 | 0.04909308635 | 0.8271403569 |
| vnn2 | -0.1808619817 | 0.0919929953 | -1.966040796 | 0.04929389833 | 0.834550534 |
| siglec6 | 0.1868625852 | 0.09513027636 | 1.964280903 | 0.04949752505 | 1.205461625 |
| ece1 | -0.1919063187 | 0.09788078075 | -1.960612872 | 0.04992419968 | 0.825384188 |
| pilra | 0.1871509015 | 0.09559777797 | 1.957690915 | 0.05026629189 | 1.20580923 |
| s100a14 | -0.1890828682 | 0.09668185048 | -1.955722478 | 0.05049785535 | 0.8277179124 |
| il16 | 0.1928514032 | 0.09878664646 | 1.952201133 | 0.05091432969 | 1.212702576 |
| insl3 | -0.3297370526 | 0.1689882067 | -1.951242983 | 0.05102814807 | 0.7191127974 |
| usp47 | -0.2095064457 | 0.1074590618 | -1.949639631 | 0.05121908629 | 0.8109844121 |
| ifnar1 | -0.2066913668 | 0.106185941 | -1.946504075 | 0.05159421894 | 0.8132706136 |
| kynu | -0.1867689661 | 0.09608808527 | -1.943726587 | 0.05192843071 | 0.8296353882 |
| hnf1a | -0.2101859023 | 0.1081400431 | -1.943645446 | 0.05193822148 | 0.8104335705 |
| akr1b1 | -0.1792809281 | 0.09240281936 | -1.940210584 | 0.05235410274 | 0.8358710467 |
| fcamr | 0.2244801996 | 0.1157699876 | 1.939018949 | 0.05249903087 | 1.251671928 |
| gp5 | -0.2070043777 | 0.106766722 | -1.938847365 | 0.0525199267 | 0.8130160909 |
| siglec10 | 0.1864900509 | 0.09622324285 | 1.938097755 | 0.05261129731 | 1.205012633 |
| hrc | 0.2027364703 | 0.1049733497 | 1.931313718 | 0.05344426794 | 1.224749668 |
| ubqln3 | -0.2014605248 | 0.1043655612 | -1.930335281 | 0.05356530827 | 0.8175358493 |
| sort1 | -0.1825262419 | 0.09460892204 | -1.929270918 | 0.05369723811 | 0.8331627799 |
| rilpl2 | -0.1969720593 | 0.1022021232 | -1.927279524 | 0.05394480399 | 0.8212135783 |
| ptpn9 | 0.195594076 | 0.1016599531 | 1.924003208 | 0.0543541813 | 1.216033188 |
| ptprn2 | 0.1678914688 | 0.08732755171 | 1.922548675 | 0.0545367551 | 1.182808232 |
| clec14a | 0.184711267 | 0.09617430221 | 1.920588585 | 0.05478359545 | 1.202871081 |
| mylpf | 0.2112859661 | 0.1102041855 | 1.9172227 | 0.05520964536 | 1.235265549 |
| ddx4 | -0.194712304 | 0.1015678033 | -1.917067198 | 0.05522939524 | 0.8230714184 |
| npc2 | 0.2290553569 | 0.1194833264 | 1.917048712 | 0.05523174352 | 1.257411643 |
| rln2 | -0.2274160519 | 0.1187468242 | -1.91513376 | 0.05547544739 | 0.7965892909 |
| ckb | 0.2157866826 | 0.112731839 | 1.914159164 | 0.05559982183 | 1.240837658 |
| shisa5 | 0.2169448411 | 0.1133835147 | 1.913371989 | 0.05570044772 | 1.242275578 |
| mog | 0.175577665 | 0.09181279659 | 1.912344156 | 0.05583206574 | 1.191934557 |
| ly9 | 0.183845727 | 0.09631172536 | 1.908861318 | 0.05627998419 | 1.201830399 |
| gnpda2 | -0.2016386367 | 0.1058104573 | -1.905658872 | 0.05669447862 | 0.8173902494 |
| ppp1cc | -0.1932745195 | 0.1015672797 | -1.902921099 | 0.05705084141 | 0.8242556688 |
| rbks | -0.181896652 | 0.09561167266 | -1.902452357 | 0.05711204175 | 0.8336874959 |
| slamf7 | 0.1800641357 | 0.09473807427 | 1.900652268 | 0.05734757445 | 1.19729415 |
| ceacam19 | -0.2022412204 | 0.1064811091 | -1.899315495 | 0.05752300681 | 0.8168978517 |
| adam15 | 0.1790105237 | 0.09436094758 | 1.897082727 | 0.05781702056 | 1.196033331 |
| pepd | -0.1923468609 | 0.1014124225 | -1.896679481 | 0.05787025341 | 0.8250206515 |
| il17f | 0.1914705016 | 0.1010348933 | 1.895092827 | 0.05808010478 | 1.211029109 |

| Protein | beta | se | z | p_val | HR |
|---|---|---|---|---|---|
| myom2 | 0.1981857589 | 0.1046891354 | 1.893088124 | 0.0583461513 | 1.219188848 |
| sost | 0.2020327616 | 0.1070614164 | 1.887073499 | 0.05915044062 | 1.223888104 |
| fosb | -0.1746252256 | 0.09288926189 | -1.87992909 | 0.06011774271 | 0.8397716868 |
| il5ra | 0.1745602866 | 0.09290182097 | 1.878975942 | 0.06024777821 | 1.190722525 |
| ca4 | 0.1680184519 | 0.08946574154 | 1.87802 | 0.06037842919 | 1.182958438 |
| fam171b | 0.1998934773 | 0.1065348678 | 1.876319758 | 0.06061138563 | 1.221272658 |
| lrp11 | 0.1771638779 | 0.09510946139 | 1.862736633 | 0.06249932914 | 1.193826719 |
| palm | 0.206680522 | 0.1109856413 | 1.862227578 | 0.0625710187 | 1.229589682 |
| srpx | 0.193142851 | 0.1037820533 | 1.861042877 | 0.06273812192 | 1.213056067 |
| klk6 | 0.1676684174 | 0.09016553119 | 1.859562243 | 0.0629474854 | 1.182544434 |
| fut8 | -0.1813976722 | 0.09764392835 | -1.857746562 | 0.06320501297 | 0.8341035929 |
| lsm8 | 0.1948785045 | 0.1049594644 | 1.856702543 | 0.06335348549 | 1.215163341 |
| bcat1 | -0.1904545821 | 0.1025769933 | -1.856698818 | 0.06335401582 | 0.8265832985 |
| prtn3 | -0.1730569164 | 0.09322835132 | -1.856269193 | 0.06341519793 | 0.8410897416 |
| creld1 | 0.2145226452 | 0.1158869838 | 1.851136669 | 0.06414989389 | 1.239270184 |
| impa1 | -0.174697008 | 0.09442965792 | -1.850022671 | 0.06431028206 | 0.8397114081 |
| mmp15 | -0.1911944504 | 0.1034049719 | -1.848987015 | 0.06445968768 | 0.8259719619 |
| sox9 | 0.1993842108 | 0.1078674257 | 1.848419109 | 0.06454173648 | 1.220650863 |
| idua | 0.1686416055 | 0.09123987824 | 1.848332207 | 0.06455429935 | 1.183695833 |
| lamb1 | 0.1947087791 | 0.1053533506 | 1.84814985 | 0.06458066792 | 1.214957114 |
| treml2 | -0.1691336934 | 0.09158088433 | -1.846823108 | 0.06477278168 | 0.8443960056 |
| edn1 | 0.1971837469 | 0.1069017827 | 1.844531886 | 0.06510566331 | 1.217967818 |
| tnn | 0.1927094352 | 0.1044760854 | 1.844531544 | 0.06510571309 | 1.212530424 |
| bst2 | 0.1846609088 | 0.1001424266 | 1.843982766 | 0.06518565187 | 1.202810509 |
| chgb | 0.166573843 | 0.09039991164 | 1.842632807 | 0.0653826405 | 1.18125076 |
| il17d | 0.1853856506 | 0.1006190653 | 1.842450535 | 0.06540927559 | 1.203682552 |
| vasp | -0.1847113862 | 0.100398594 | -1.839780608 | 0.0658004536 | 0.8313441866 |
| apoa1 | -0.2085174765 | 0.1133578746 | -1.839461769 | 0.06584729611 | 0.8117868474 |
| gpa33 | -0.1858071918 | 0.1012006157 | -1.836028275 | 0.06635347501 | 0.830433694 |
| col28a1 | 0.187665658 | 0.1022329809 | 1.835666499 | 0.06640699561 | 1.206430088 |
| il2rg | 0.1996325322 | 0.1088311896 | 1.834331987 | 0.06660472872 | 1.220954014 |
| cpxm2 | 0.1987077741 | 0.1083716219 | 1.833577561 | 0.06671672568 | 1.219825449 |
| myoc | 0.1783522288 | 0.09737316098 | 1.831636428 | 0.06700560573 | 1.195246247 |
| lrrn1 | -0.179181901 | 0.09786331578 | -1.830940425 | 0.06710943566 | 0.8359538247 |
| bmp6 | -0.1629508713 | 0.0890461386 | -1.829959994 | 0.06725592118 | 0.8496329287 |
| mxra8 | -0.2193885569 | 0.1199473593 | -1.829040324 | 0.06739356768 | 0.8030096426 |
| lypd8 | 0.1698233663 | 0.09297104409 | 1.82662643 | 0.06775595675 | 1.185095505 |
| cnpy2 | -0.1720365333 | 0.09418274974 | -1.826624661 | 0.06775622282 | 0.8419484134 |
| foxo3 | -0.17182678 | 0.09411172702 | -1.82577438 | 0.06788425398 | 0.8421250334 |
| cdsn | 0.17108418 | 0.09383698326 | 1.82320631 | 0.06827214979 | 1.186590632 |
| cntn2 | 0.177602612 | 0.09743901957 | 1.822705244 | 0.06834804558 | 1.194350606 |
| eif2ak3 | -0.1907846116 | 0.1046879239 | -1.822412791 | 0.0683923752 | 0.8263105467 |
| dcc | -0.1912658243 | 0.1051250398 | -1.819412622 | 0.06884850241 | 0.8259130112 |
| mstn | 0.1884342704 | 0.1039348153 | 1.813004333 | 0.06983115306 | 1.207357721 |
| galnt3 | 0.1772066546 | 0.0977637514 | 1.812600807 | 0.06989341333 | 1.193877788 |
| cst5 | 0.1786431065 | 0.09856917524 | 1.812362801 | 0.06993015677 | 1.195593968 |
| lypd3 | 0.167808169 | 0.09267655042 | 1.810686395 | 0.07018941008 | 1.182709708 |
| krt6c | 0.1951606864 | 0.1078132862 | 1.810172876 | 0.07026898256 | 1.215506286 |
| lrig3 | 0.2079126168 | 0.1149044473 | 1.809439248 | 0.07038279036 | 1.231105587 |
| ccn3 | 0.1792680904 | 0.09922069739 | 1.80676104 | 0.07079954582 | 1.196341429 |
| ncam1 | 0.1726437921 | 0.09563199532 | 1.805293213 | 0.07102881111 | 1.188442697 |
| atp5po | 0.1734934429 | 0.09611278514 | 1.805102647 | 0.07105862088 | 1.189452887 |
| ephb4 | 0.1618286655 | 0.0896706253 | 1.804700982 | 0.0711214861 | 1.175658793 |
| syap1 | -0.1919691639 | 0.1063830441 | -1.804509031 | 0.07115154469 | 0.8253323181 |
| anp32c | -0.1919918838 | 0.1064277864 | -1.803963893 | 0.07123696755 | 0.8253135669 |
| krt8 | 0.1797129033 | 0.09964749052 | 1.803486494 | 0.07131184465 | 1.196873695 |
| gip | 0.2099412763 | 0.1164160986 | 1.803369798 | 0.0713301575 | 1.233605616 |
| ostn | 0.1903276445 | 0.1056785501 | 1.801005448 | 0.07170202126 | 1.209645867 |
| adam12 | 0.2186526706 | 0.1216583914 | 1.797267481 | 0.0722931661 | 1.244398985 |
| mfap5 | 0.1758527544 | 0.09786641424 | 1.796865204 | 0.07235702187 | 1.19226249 |
| vwa1 | 0.1924352096 | 0.1073714235 | 1.792238599 | 0.07309475418 | 1.212197962 |
| cdnf | 0.1611055647 | 0.09012072164 | 1.787663944 | 0.07383024223 | 1.174808981 |
| gne | -0.1661469226 | 0.0929988637 | -1.786547878 | 0.0740105923 | 0.8469217931 |
| ngf | 0.1742841834 | 0.09763328483 | 1.785089825 | 0.07424674808 | 1.190393808 |
| itgb6 | 0.1789515304 | 0.1004271368 | 1.781904135 | 0.0747648661 | 1.195962775 |
| cxcl16 | 0.1722407061 | 0.09666388129 | 1.781851751 | 0.07477341046 | 1.187963749 |
| csf1 | 0.1770867016 | 0.09938593231 | 1.781808526 | 0.07478046136 | 1.193734587 |
| septin8 | 0.2022790806 | 0.1135368982 | 1.781615351 | 0.07481197942 | 1.224189608 |
| crhbp | 0.1704334637 | 0.09582866813 | 1.778522722 | 0.07531804344 | 1.185818749 |
| amot | -0.212274771 | 0.1194712415 | -1.776785512 | 0.07560353656 | 0.8087424481 |

| Protein | beta | se | z | p_val | HR |
|---|---|---|---|---|---|
| rab33a | -0.1776129658 | 0.09998672939 | -1.776365393 | 0.07567271135 | 0.8372664115 |
| agrn | 0.1788402605 | 0.1008516674 | 1.773299987 | 0.07617901119 | 1.195829708 |
| ctso | 0.1782439791 | 0.1006463677 | 1.770992667 | 0.07656192107 | 1.195116869 |
| nfat5 | -0.179904102 | 0.1016310416 | -1.770168829 | 0.07669902028 | 0.835350316 |
| sema3f | 0.1794836044 | 0.1014154199 | 1.769786138 | 0.07676277395 | 1.196599285 |
| ccl14 | 0.1716523901 | 0.09707346599 | 1.768273012 | 0.0770152734 | 1.187265056 |
| cd6 | -0.1711726304 | 0.09681996309 | -1.767947693 | 0.07706964858 | 0.8426760894 |
| pdcd1 | 0.167907767 | 0.09508968879 | 1.765783116 | 0.0774322422 | 1.18282751 |
| cdh15 | 0.1944109187 | 0.1102087768 | 1.764023923 | 0.07772795163 | 1.21459528 |
| havcr2 | 0.1747444177 | 0.09912802281 | 1.762815526 | 0.07793160797 | 1.190941794 |
| ntprobnp | 0.167718037 | 0.09519532304 | 1.761830641 | 0.07809791614 | 1.182603113 |
| nfatc1 | -0.1612287473 | 0.09169277757 | -1.7583582 | 0.07868658071 | 0.8510973626 |
| actn2 | 0.1962515648 | 0.1119465411 | 1.75308288 | 0.07958778165 | 1.216832979 |
| mdm1 | -0.1879036245 | 0.1073210924 | -1.750854565 | 0.07997096484 | 0.8286945693 |
| ret | 0.1737976706 | 0.099274326 | 1.750680943 | 0.08000088392 | 1.189814807 |
| dnaja4 | 0.1882617068 | 0.1076082893 | 1.749509337 | 0.08020301622 | 1.207149393 |
| dpt | 0.1831980209 | 0.1051663203 | 1.741983749 | 0.08151128792 | 1.201052218 |
| il1rl1 | 0.1815968045 | 0.1043204262 | 1.740759803 | 0.08172569074 | 1.199130612 |
| cngb3 | 0.1801533957 | 0.1036027338 | 1.738886505 | 0.0820547284 | 1.197401025 |
| ddx53 | -0.1877994023 | 0.1080409934 | -1.73822358 | 0.08217142576 | 0.8287809421 |
| mfge8 | 0.1654148516 | 0.0951762218 | 1.73798506 | 0.08221344626 | 1.179882493 |
| cdhr2 | -0.210273297 | 0.1211316127 | -1.735907681 | 0.08258015938 | 0.810362746 |
| vwc2 | 0.1612367468 | 0.09291409307 | 1.735331438 | 0.08268211634 | 1.174963105 |
| rab37 | -0.1656721633 | 0.09553870218 | -1.734084298 | 0.0829031272 | 0.8473239726 |
| gfap | -0.1785011822 | 0.1030616502 | -1.731984515 | 0.08327631958 | 0.8365230679 |
| adgrb3 | 0.1591146972 | 0.09190210954 | 1.731349781 | 0.08338939776 | 1.17247241 |
| fgf12 | -0.178007313 | 0.1028560813 | -1.730644516 | 0.08351518672 | 0.8369363029 |
| eddm3b | -0.2908064037 | 0.1688909293 | -1.721859219 | 0.08509502397 | 0.7476604083 |
| robo4 | 0.1848831144 | 0.1073879464 | 1.721637489 | 0.08513520772 | 1.203077809 |
| masp1 | -0.1632009281 | 0.09483652109 | -1.720865824 | 0.08527517439 | 0.8494204989 |
| ppy | 0.1655431804 | 0.09622811548 | 1.720320299 | 0.08537423566 | 1.180033916 |
| rnase4 | 0.1974220513 | 0.1148179326 | 1.719435691 | 0.08553506811 | 1.2182581 |
| ltbp3 | 0.1791875618 | 0.104216717 | 1.719374464 | 0.08554620895 | 1.196245093 |
| lilra3 | 0.1744342697 | 0.1014541774 | 1.719340437 | 0.08555240092 | 1.190572483 |
| ccl16 | 0.1680273096 | 0.09782188652 | 1.717686251 | 0.08585385845 | 1.182968917 |
| glyr1 | -0.1852137146 | 0.1080371127 | -1.714352688 | 0.08646397204 | 0.8309266837 |
| tdgf1 | 0.164343137 | 0.09601702091 | 1.711604207 | 0.08696963247 | 1.178618673 |
| lilra6 | 0.1774901268 | 0.1037043593 | 1.711501117 | 0.08698864507 | 1.194216267 |
| fap | 0.1581138262 | 0.09259878516 | 1.707515125 | 0.08772634893 | 1.171299512 |
| insr | 0.1729871626 | 0.1014384351 | 1.705341396 | 0.08813077131 | 1.188850843 |
| dtx2 | -0.178349053 | 0.1046227687 | -1.704686802 | 0.08825285261 | 0.8366503372 |
| gipc2 | 0.181082881 | 0.1063341606 | 1.702960554 | 0.08857545066 | 1.198514509 |
| gabarap | -0.1804410803 | 0.1060246694 | -1.701878263 | 0.08877819139 | 0.8349018715 |
| tgfb2 | 0.1848238533 | 0.108843429 | 1.698070843 | 0.08949439184 | 1.203006516 |
| ndst1 | -0.1791544401 | 0.1055120441 | -1.69795251 | 0.08951672544 | 0.8359767811 |
| hpse | -0.1755230726 | 0.1034578278 | -1.696566382 | 0.0897786701 | 0.8390180386 |
| wif1 | -0.1641016063 | 0.09674024209 | -1.696311718 | 0.08982686243 | 0.8486557887 |
| hla_dra | 0.15573135 | 0.09200943804 | 1.69255843 | 0.09053954931 | 1.16851224 |
| fmnl1 | 0.1727754369 | 0.1021291741 | 1.691734397 | 0.09069662684 | 1.18859916 |
| vwf | -0.1643039903 | 0.09714529702 | -1.691322126 | 0.09077529637 | 0.8484840517 |
| bcl7b | -0.1816867224 | 0.107516696 | -1.689846592 | 0.0910573075 | 0.83386253 |
| rrm2b | -0.1627222027 | 0.09640765155 | -1.687855685 | 0.09143893515 | 0.8498272353 |
| eif5 | -0.1786140528 | 0.1058535239 | -1.687369927 | 0.09153224272 | 0.8364286544 |
| zp4 | 0.1799646904 | 0.1067356165 | 1.686079083 | 0.09178056831 | 1.197175091 |
| nenf | -0.1712435324 | 0.1015754577 | -1.685875075 | 0.09181986362 | 0.8426163441 |
| rspo1 | -0.1682306685 | 0.09992980344 | -1.683488436 | 0.09228057613 | 0.8451588607 |
| klhl41 | 0.1791040515 | 0.1064003811 | 1.683302725 | 0.09231650308 | 1.196145198 |
| pfdn4 | 0.1833257624 | 0.1090736712 | 1.680751738 | 0.09281114655 | 1.201205652 |
| folr1 | 0.1577114448 | 0.09418142316 | 1.674549391 | 0.09402267591 | 1.170828297 |
| cd70 | 0.1641360366 | 0.0980400175 | 1.674173881 | 0.09409643116 | 1.178374606 |
| git1 | -0.1700008114 | 0.1015958941 | -1.673303955 | 0.09426747429 | 0.843664132 |
| atp1b4 | 0.1838594854 | 0.1100251204 | 1.671068249 | 0.0947081979 | 1.201846934 |
| cd300c | 0.1594857638 | 0.09557813028 | 1.668642851 | 0.09518818134 | 1.172907564 |
| tst | -0.1578061401 | 0.09460035662 | -1.668134727 | 0.09528898474 | 0.8540153253 |
| psap | 0.1893330317 | 0.1136365745 | 1.666127587 | 0.09568800486 | 1.208443335 |
| ereg | -0.1595632307 | 0.09592070183 | -1.663491068 | 0.09621417736 | 0.8525160605 |
| arnt | -0.1635860914 | 0.09836335266 | -1.663079663 | 0.09629649026 | 0.8490933962 |
| ltbp2 | 0.1708136255 | 0.102738615 | 1.662603934 | 0.09639174308 | 1.186269638 |
| cytl1 | -0.1744165372 | 0.1049737374 | -1.661525458 | 0.09660796024 | 0.8399469556 |
| tp53 | -0.1596886245 | 0.09615320514 | -1.660772767 | 0.09675909261 | 0.852409167 |

| Protein | beta | se | z | p_val | HR |
| --- | --- | --- | --- | --- | --- |
| espl1 | 0.1798512157 | 0.1083623192 | 1.65972099 | 0.0969705947 | 1.197039249 |
| efnb2 | 0.1746551866 | 0.1052330398 | 1.659699148 | 0.09697499081 | 1.19083553 |
| bambi | 0.1537846197 | 0.09266998408 | 1.659486847 | 0.09701772885 | 1.166239675 |
| chchd6 | 0.1897356935 | 0.1143770614 | 1.658861411 | 0.09714372222 | 1.208930027 |
| gprc5c | 0.1769932095 | 0.1067653058 | 1.657778323 | 0.09736221823 | 1.193622988 |
| tbc1d17 | 0.1613418623 | 0.09751180985 | 1.654587916 | 0.09800811629 | 1.175086618 |
| tgfa | 0.172051076 | 0.1041655623 | 1.651707841 | 0.09859412346 | 1.187738497 |
| cacna1h | 0.1748945511 | 0.1060327539 | 1.649438921 | 0.0990577467 | 1.191120608 |
| treml1 | -0.1692967689 | 0.1027255252 | -1.648049679 | 0.09934247715 | 0.8442583166 |
| cend1 | 0.1683219924 | 0.1023070334 | 1.645263153 | 0.09991555498 | 1.183317569 |
| siglec7 | 0.16469267 | 0.1003949956 | 1.640447007 | 0.1009122584 | 1.179030712 |
| defb103a_defb103b | 0.1760500523 | 0.107331522 | 1.640245559 | 0.10095412 | 1.192497744 |
| calb2 | 0.1664184607 | 0.101473136 | 1.640024812 | 0.1010000081 | 1.181067229 |
| apol1 | -0.1809160284 | 0.1103457744 | -1.639537439 | 0.1011013801 | 0.8345054305 |
| nid2 | -0.1571096168 | 0.09582582354 | -1.639533176 | 0.1011022672 | 0.854610374 |
| lilra5 | 0.1632365711 | 0.09958446322 | 1.639177095 | 0.1011763827 | 1.177315175 |
| lpo | 0.156680226 | 0.09562586276 | 1.638471241 | 0.1013234286 | 1.16962154 |
| timp3 | -0.1521650245 | 0.0930333177 | -1.635597098 | 0.1019239381 | 0.8588465384 |
| cd163 | 0.1664121225 | 0.1019279468 | 1.632644704 | 0.1025437433 | 1.181059743 |
| nmnat1 | 0.1620683641 | 0.09931823006 | 1.631808823 | 0.1027197661 | 1.175940631 |
| zbtb16 | -0.1513888723 | 0.09279578435 | -1.631419718 | 0.1028017871 | 0.8595133927 |
| acvrl1 | 0.1645746983 | 0.1008940521 | 1.631163532 | 0.102855818 | 1.178891627 |
| ctsf | 0.1599359659 | 0.09805348483 | 1.631109452 | 0.1028672267 | 1.173435729 |
| asgr1 | 0.1737010522 | 0.1065541853 | 1.630166396 | 0.1030663341 | 1.189699854 |
| pglyrp4 | 0.1735377584 | 0.1065173371 | 1.629197304 | 0.1032712576 | 1.1895056 |
| tmprss11d | 0.1662973329 | 0.102080036 | 1.629087718 | 0.1032944509 | 1.180924177 |
| ppp1r14d | 0.1663751561 | 0.1021304653 | 1.629045315 | 0.1033034263 | 1.181016084 |
| shc1 | -0.1708157012 | 0.104999512 | -1.626823763 | 0.1037745331 | 0.8429769188 |
| inhbb | 0.1812740569 | 0.1116479426 | 1.62362201 | 0.1044565051 | 1.198743658 |
| prdx6 | -0.1553637846 | 0.09590562604 | -1.619965283 | 0.1052397348 | 0.8561036835 |
| sftpd | -0.159227415 | 0.09832275999 | -1.619435978 | 0.105353491 | 0.8528023969 |
| nacc1 | -0.1755107334 | 0.1084395456 | -1.618512254 | 0.1055522481 | 0.8390283915 |
| trim5 | -0.1473433876 | 0.09105940267 | -1.618101846 | 0.1056406507 | 0.8629975839 |
| tpm3 | 0.1688254823 | 0.1043747258 | 1.6174939 | 0.1057717114 | 1.183913507 |
| adam22 | 0.1474066154 | 0.09126988711 | 1.615062975 | 0.1062970578 | 1.158825063 |
| serpinf1 | 0.1972179068 | 0.1221165281 | 1.614997658 | 0.1063112021 | 1.218009424 |
| ang | 0.1602889471 | 0.0993182289 | 1.613892524 | 0.1065507405 | 1.173850003 |
| dars1 | -0.1542375007 | 0.09577019534 | -1.610495835 | 0.1072896541 | 0.8570684425 |
| amotl2 | -0.171082426 | 0.1062938546 | -1.609523209 | 0.1075019844 | 0.842752106 |
| dgkz | -0.1461566247 | 0.0908252648 | -1.609206701 | 0.1075711518 | 0.8640223653 |
| chi3l1 | 0.1655158067 | 0.1029204254 | 1.608192018 | 0.1077931307 | 1.180001614 |
| fcer1a | 0.1611265454 | 0.1003054599 | 1.606358672 | 0.1081951258 | 1.174833629 |
| ccnd2 | 0.1710984143 | 0.1065459842 | 1.605864506 | 0.1083036837 | 1.186607522 |
| tspan7 | -0.1718444406 | 0.1071440883 | -1.603863016 | 0.1087442506 | 0.8421101612 |
| tacc3 | -0.1464442311 | 0.09134766733 | -1.603152389 | 0.1089010141 | 0.8637739027 |
| lif | -0.1558209189 | 0.09738253311 | -1.600091042 | 0.109578388 | 0.8557124186 |
| tmem106a | -0.165830427 | 0.1038674501 | -1.596558179 | 0.1103642274 | 0.8471898826 |
| gfer | -0.1531829655 | 0.09594831403 | -1.596515448 | 0.1103737596 | 0.857972728 |
| rras | -0.1626345561 | 0.1018829542 | -1.596288186 | 0.1104244666 | 0.849901723 |
| gys1 | -0.1503094703 | 0.09422413769 | -1.595233175 | 0.1106601027 | 0.8604416541 |
| kirrel2 | -0.1538467893 | 0.09648546351 | -1.594507438 | 0.110822426 | 0.8574033743 |
| egfr | -0.1529072936 | 0.09628112396 | -1.588133658 | 0.1122561189 | 0.8582092797 |
| bnip2 | -0.1660723134 | 0.1045954034 | -1.587759193 | 0.1123408022 | 0.8469849836 |
| serpine2 | -0.1711279388 | 0.1079979185 | -1.584548491 | 0.1130689559 | 0.8427137508 |
| pdlim5 | -0.1610895206 | 0.1017079976 | -1.583843202 | 0.1132294053 | 0.8512158663 |
| bcl2l11 | 0.1535005949 | 0.09700845948 | 1.582342362 | 0.1135714351 | 1.165908481 |
| ptprk | 0.173617047 | 0.1098449667 | 1.580564428 | 0.1139776646 | 1.189599918 |
| prkd2 | -0.1719918706 | 0.1088603001 | -1.57993199 | 0.1141224422 | 0.841986018 |
| il2ra | 0.1455848978 | 0.09256501431 | 1.572785344 | 0.1157685305 | 1.156715933 |
| wfdc1 | 0.1776776463 | 0.113266194 | 1.56867323 | 0.1167240995 | 1.194440227 |
| pbld | -0.1482294866 | 0.09449982994 | -1.568568818 | 0.1167484428 | 0.8622332213 |
| trim25 | -0.1598531579 | 0.1020331341 | -1.566678896 | 0.1171897661 | 0.8522689288 |
| chm | -0.15975597 | 0.102046806 | -1.565516612 | 0.1174618254 | 0.852351763 |
| thpo | -0.1455210797 | 0.09301501272 | -1.564490241 | 0.1177024831 | 0.864571665 |
| tspan1 | 0.1661401433 | 0.1062133238 | 1.564211884 | 0.1177678172 | 1.180738563 |
| gtpbp2 | -0.1603107804 | 0.1024940826 | -1.56409791 | 0.1177945768 | 0.8518790005 |
| adgre2 | 0.1512076697 | 0.0966793791 | 1.564011593 | 0.1178148459 | 1.163238202 |
| clec4m | -0.1698519604 | 0.1086315574 | -1.563560023 | 0.1179209294 | 0.8437897216 |
| dnajc21 | -0.1585873369 | 0.1014871377 | -1.562634837 | 0.1181385094 | 0.8533484318 |
| prkcq | -0.1450531796 | 0.09290752585 | -1.561264045 | 0.1184614633 | 0.8649762928 |

| Protein | beta | se | z | p_val | HR |
|---|---|---|---|---|---|
| ptprf | -0.1480159268 | 0.09484817407 | -1.560556418 | 0.1186284485 | 0.8624173793 |
| heg1 | 0.1697279117 | 0.1088434015 | 1.559377136 | 0.1189071445 | 1.184982388 |
| rbp5 | 0.1656698525 | 0.1062611127 | 1.559082606 | 0.11897683 | 1.180183403 |
| sdhb | -0.1611660464 | 0.1033891088 | -1.558830018 | 0.1190366175 | 0.8511507289 |
| thap12 | 0.1696493728 | 0.1089145642 | 1.557637163 | 0.1193192839 | 1.184889324 |
| il33 | 0.1469769336 | 0.09450344971 | 1.555254692 | 0.1198854233 | 1.158327244 |
| appl2 | -0.1603204017 | 0.1031429922 | -1.55435089 | 0.1201007408 | 0.8518708043 |
| vegfc | -0.1522693509 | 0.098188527 | -1.55078557 | 0.1209530797 | 0.8587569427 |
| sit1 | 0.1560598123 | 0.1006688956 | 1.55022871 | 0.1210866311 | 1.168896115 |
| tbl1x | -0.1422792646 | 0.09194587826 | -1.547424063 | 0.1217610224 | 0.8673789944 |
| fyb1 | -0.141296711 | 0.09135845719 | -1.546618839 | 0.1219551841 | 0.8682316596 |
| pilrb | 0.1428001381 | 0.09245547396 | 1.544528755 | 0.1224602907 | 1.153499238 |
| cd300lf | 0.1352141171 | 0.08763594596 | 1.54290703 | 0.1228533348 | 1.144781876 |
| igfbp6 | 0.1664787995 | 0.1081330348 | 1.539573914 | 0.1236642482 | 1.181138495 |
| hip1r | 0.1730834171 | 0.112450749 | 1.539193102 | 0.1237571612 | 1.188965281 |
| bmp10 | 0.163055382 | 0.1060397531 | 1.537681646 | 0.1241264736 | 1.177101878 |
| trim40 | -0.1503140323 | 0.09776710802 | -1.537470376 | 0.1241781642 | 0.8604377288 |
| rgl2 | -0.1615138226 | 0.1051446259 | -1.536111058 | 0.1245111446 | 0.8508547704 |
| stam | -0.1646218563 | 0.1072341038 | -1.535163259 | 0.1247437306 | 0.8482143904 |
| pf4 | -0.1581586533 | 0.1031774064 | -1.532880684 | 0.1253052559 | 0.8537143266 |
| prkar1a | -0.1454949155 | 0.09491807767 | -1.532847263 | 0.1253134924 | 0.8645942861 |
| nrcam | 0.1441546781 | 0.09416302083 | 1.530905411 | 0.1257927717 | 1.155062758 |
| clstn2 | 0.1404439609 | 0.09174452049 | 1.530815793 | 0.1258149253 | 1.150784589 |
| ppm1f | -0.1542921986 | 0.101104573 | -1.526065479 | 0.1269935583 | 0.857021564 |
| usp28 | 0.1770238759 | 0.1160272692 | 1.525709233 | 0.127082294 | 1.193659593 |
| dnlz | -0.1610014983 | 0.1055966301 | -1.524684057 | 0.1273379203 | 0.8512907956 |
| angptl4 | 0.1534891715 | 0.1007332078 | 1.523719683 | 0.1275787503 | 1.165895162 |
| dkk1 | -0.1467505374 | 0.09634370823 | -1.523197935 | 0.1277091927 | 0.8635093638 |
| gfral | -0.152675961 | 0.1002609638 | -1.52278569 | 0.1278123312 | 0.8584078344 |
| angptl1 | 0.1455245997 | 0.09563392619 | 1.521683836 | 0.1280883196 | 1.156646187 |
| ido1 | 0.1657104445 | 0.1089499625 | 1.520977527 | 0.128265477 | 1.18023131 |
| hs6st2 | 0.1576289403 | 0.1037922068 | 1.518697262 | 0.1288387162 | 1.170731703 |
| nppb | 0.1398200751 | 0.09208450443 | 1.518388744 | 0.1289164275 | 1.150066855 |
| mindy1 | -0.1523664106 | 0.100418799 | -1.517309629 | 0.1291885283 | 0.858673596 |
| frmd4b | -0.1547234968 | 0.1019855448 | -1.517112029 | 0.1292384017 | 0.8566520117 |
| il12rb1 | 0.1523322023 | 0.1005004621 | 1.515736337 | 0.1295860355 | 1.164547037 |
| atxn10 | -0.1365678285 | 0.09013524053 | -1.515143552 | 0.1297360544 | 0.8723471483 |
| lpp | -0.1570088471 | 0.1036765748 | -1.514410052 | 0.1299218713 | 0.8546964972 |
| bloc1s3 | -0.1516968327 | 0.100243767 | -1.513279451 | 0.13020869 | 0.8592487374 |
| psrc1 | -0.1382336779 | 0.09148630503 | -1.510976728 | 0.1307943802 | 0.8708951591 |
| timp1 | 0.1533014478 | 0.1015058434 | 1.510272144 | 0.1309739965 | 1.165676317 |
| ambn | -0.1456608665 | 0.09649040591 | -1.509589116 | 0.1311483004 | 0.8644508177 |
| nid1 | -0.154723748 | 0.1027525524 | -1.505789826 | 0.1321211359 | 0.8566517966 |
| gkn1 | 0.1518921049 | 0.1009295704 | 1.50493165 | 0.1323416502 | 1.164034636 |
| sumf2 | -0.1420273334 | 0.09438987727 | -1.504688188 | 0.1324042613 | 0.8675975418 |
| msmb | 0.1485061691 | 0.09869654266 | 1.504674481 | 0.132407787 | 1.160099955 |
| mep1a | 0.1652173882 | 0.1098100438 | 1.504574467 | 0.1324335149 | 1.179649533 |
| prss27 | 0.1431633565 | 0.09524797146 | 1.503059375 | 0.1328237335 | 1.153918286 |
| vsig10l | 0.1604681391 | 0.1068530018 | 1.501765383 | 0.1331577114 | 1.174060366 |
| duox2 | -0.143104707 | 0.09532242617 | -1.501270087 | 0.1332857187 | 0.8666633184 |
| cd226 | -0.1542320391 | 0.1028457069 | -1.499644893 | 0.1337064124 | 0.8570731235 |
| ctsh | 0.1475569111 | 0.09839482936 | 1.499640907 | 0.1337074454 | 1.158999243 |
| fgfbp3 | -0.1601797468 | 0.1069268031 | -1.498031758 | 0.1341249994 | 0.8519906326 |
| fus | -0.1497987626 | 0.1002869819 | -1.493700974 | 0.1352537938 | 0.8608812005 |
| lmnb2 | 0.166400876 | 0.1114872718 | 1.492554919 | 0.1355537313 | 1.18104646 |
| casp2 | -0.1367432083 | 0.09167796319 | -1.491560278 | 0.1358144579 | 0.8721941696 |
| acadsb | -0.1491124436 | 0.09999063082 | -1.491264155 | 0.1358921559 | 0.8614722424 |
| m6pr | -0.1632185522 | 0.1094577247 | -1.491156085 | 0.1359205204 | 0.8494055287 |
| ly6d | 0.1385081287 | 0.09291639183 | 1.490674852 | 0.1360468815 | 1.148559018 |
| nme3 | -0.1396989347 | 0.09377886967 | -1.489663238 | 0.1363128053 | 0.8696200084 |
| clec6a | 0.1410959086 | 0.09472589891 | 1.489517758 | 0.1363510807 | 1.151535085 |
| hspg2 | 0.1500613421 | 0.100770288 | 1.489142733 | 0.1364497871 | 1.161905514 |
| cdc25a | 0.1628630031 | 0.1094635232 | 1.48782899 | 0.1367959983 | 1.17687545 |
| krt18 | 0.1592024073 | 0.1071510992 | 1.48577484 | 0.1373386873 | 1.17257526 |
| ttn | 0.1692470642 | 0.1139754722 | 1.484942866 | 0.1375589597 | 1.184412729 |
| brsk2 | -0.1472004789 | 0.09919875494 | -1.48389442 | 0.1378369325 | 0.8631209225 |
| pcdh1 | 0.149883188 | 0.1010159196 | 1.483758092 | 0.1378731087 | 1.161698535 |
| naga | -0.1594239783 | 0.1075353773 | -1.482525866 | 0.1382004263 | 0.8526347836 |
| pdgfa | -0.1384457258 | 0.09339513564 | -1.482365487 | 0.1382430719 | 0.8707105071 |
| nub1 | -0.1399952528 | 0.09445089393 | -1.482201459 | 0.1382866985 | 0.8693623624 |

| Protein | beta | se | z | p_val | HR |
|---|---|---|---|---|---|
| tmem132a | 0.152218468 | 0.1027408026 | 1.481577565 | 0.1384527323 | 1.164414596 |
| dtnb | 0.1726385263 | 0.1166267614 | 1.480265115 | 0.1388025092 | 1.188436439 |
| dctd | -0.1517822441 | 0.1025679439 | -1.479821456 | 0.1389209011 | 0.8591753509 |
| prelp | 0.1398755588 | 0.09462351876 | 1.47823248 | 0.1393455629 | 1.150130666 |
| nxph1 | -0.1446822717 | 0.09788730679 | -1.478049365 | 0.1393945656 | 0.8652971789 |
| traf3 | 0.16861299 | 0.1140910671 | 1.477880734 | 0.139439704 | 1.183661961 |
| gabra4 | 0.1577335947 | 0.1067750492 | 1.477251436 | 0.1396082507 | 1.170854231 |
| c1qa | 0.1407889833 | 0.09546128109 | 1.474828136 | 0.1402587547 | 1.151181704 |
| cd83 | 0.1430266704 | 0.09698740103 | 1.474693299 | 0.1402950185 | 1.153760572 |
| chp1 | -0.1584501865 | 0.1075544969 | -1.473208384 | 0.1406948551 | 0.8534654768 |
| sh2b3 | -0.1361193036 | 0.09242029779 | -1.472829096 | 0.1407971247 | 0.8727385054 |
| enpp6 | 0.1688045169 | 0.1146786602 | 1.471978454 | 0.141026696 | 1.183888686 |
| slitrk1 | -0.1700386639 | 0.1155278166 | -1.471841751 | 0.1410636161 | 0.8436321978 |
| kyat1 | -0.1398468342 | 0.09502425854 | -1.471696137 | 0.1411029512 | 0.8694914015 |
| ctsv | -0.1464582347 | 0.09967851877 | -1.46930589 | 0.14174984 | 0.8637618068 |
| pcna | 0.1616508161 | 0.1100229553 | 1.469246264 | 0.1417660061 | 1.175449721 |
| sod3 | 0.1566703692 | 0.1067370095 | 1.467816739 | 0.1421540093 | 1.169610011 |
| ung | -0.1533022655 | 0.1044713099 | -1.467410198 | 0.142264502 | 0.857870378 |
| gas6 | 0.1421347412 | 0.09688088973 | 1.467108132 | 0.1423466422 | 1.152731959 |
| csnk1d | -0.1477442751 | 0.1008021378 | -1.46568593 | 0.1427338691 | 0.8626516883 |
| f3 | 0.1456157964 | 0.09944604758 | 1.46426932 | 0.1431203765 | 1.156751675 |
| coro6 | 0.1560433157 | 0.106684089 | 1.46266718 | 0.1435584708 | 1.168876833 |
| pga4 | 0.1455952614 | 0.09959332111 | 1.461897844 | 0.1437692058 | 1.156727921 |
| cd109 | 0.1336968252 | 0.09160997744 | 1.459413362 | 0.1444513704 | 1.143046224 |
| robo1 | 0.1339202597 | 0.09184020406 | 1.458187741 | 0.1447888019 | 1.143301649 |
| agrp | 0.1416384933 | 0.09713512577 | 1.458159365 | 0.1447966214 | 1.15216006 |
| dock9 | -0.1505800684 | 0.1035624992 | -1.454001879 | 0.1459457851 | 0.8602088517 |
| dapk2 | -0.1528991342 | 0.1051756612 | -1.453750159 | 0.1460155862 | 0.8582162821 |
| lelp1 | -0.1579786591 | 0.1088267953 | -1.45165222 | 0.1465983308 | 0.8538680041 |
| setmar | -0.1516714449 | 0.1046839821 | -1.448850549 | 0.1473793235 | 0.8592705521 |
| rab11fip3 | -0.1440758729 | 0.0995619837 | -1.447097251 | 0.1478696879 | 0.8658220531 |
| folr3 | 0.141124908 | 0.09809770968 | 1.438615727 | 0.1502594278 | 1.151568479 |
| matn3 | -0.1352164642 | 0.09404323969 | -1.437811635 | 0.1504875073 | 0.8735268039 |
| polr2f | 0.1487617204 | 0.1034841256 | 1.437531791 | 0.1505669463 | 1.160396457 |
| pi3 | 0.1465570167 | 0.1019931915 | 1.436929412 | 0.1507380517 | 1.157840945 |
| pnliprp1 | -0.1386320863 | 0.09651744004 | -1.436342347 | 0.1509049498 | 0.8705482562 |
| pxdnl | 0.1486475166 | 0.1036035227 | 1.434772803 | 0.1513518504 | 1.160263943 |
| milr1 | 0.1356535213 | 0.09454820775 | 1.434755079 | 0.1513569027 | 1.145285008 |
| cetn3 | -0.1475979809 | 0.1029363865 | -1.433875676 | 0.1516077442 | 0.8627778985 |
| ralb | -0.1514083334 | 0.1057063524 | -1.432348482 | 0.1520441146 | 0.8594966658 |
| btc | -0.1346789878 | 0.0940802163 | -1.431533569 | 0.1522773533 | 0.8739964302 |
| zpr1 | -0.148959848 | 0.1041275009 | -1.430552415 | 0.1525585335 | 0.8616037093 |
| map3k5 | -0.1382303104 | 0.09664235927 | -1.430328393 | 0.1526227894 | 0.8708980917 |
| cxcl8 | 0.1344105551 | 0.09433472074 | 1.424825918 | 0.1542075284 | 1.143862342 |
| tank | -0.1332175358 | 0.09354039393 | -1.424171208 | 0.1543969174 | 0.8752746678 |
| rgcc | -0.1438514349 | 0.1012283618 | -1.421058607 | 0.1552997222 | 0.8660163983 |
| ceacam6 | -0.1456823573 | 0.1025288841 | -1.420890888 | 0.1553484823 | 0.8644322402 |
| gcnt1 | 0.1366247621 | 0.0962017896 | 1.420189403 | 0.1555525479 | 1.146397896 |
| cd40lg | -0.1318485089 | 0.09297009781 | -1.418181889 | 0.1561376682 | 0.876473763 |
| icam2 | 0.1281617132 | 0.09039080501 | 1.417862283 | 0.1562309759 | 1.136736813 |
| cbln1 | -0.148444453 | 0.1048240709 | -1.416129442 | 0.1567376095 | 0.86204789 |
| il10rb | 0.1389415202 | 0.09817538989 | 1.415237774 | 0.1569987927 | 1.149056901 |
| cdh22 | 0.1456792027 | 0.1030170442 | 1.414127185 | 0.1573245627 | 1.156825022 |
| ptprh | 0.1534917372 | 0.1086243131 | 1.413051395 | 0.1576406131 | 1.165898153 |
| glrx5 | -0.1486695656 | 0.1054899596 | -1.40932432 | 0.1587392908 | 0.8618538541 |
| arhgap45 | -0.1444479225 | 0.1026344631 | -1.407401745 | 0.1593082941 | 0.8654999843 |
| fgf6 | 0.1459168351 | 0.1037625735 | 1.406256902 | 0.1596478528 | 1.157099954 |
| dpep1 | -0.1389622311 | 0.09881906084 | -1.40622902 | 0.1596561295 | 0.8702608967 |
| mydgf | -0.1477408082 | 0.1050810082 | -1.405970601 | 0.1597328549 | 0.862654679 |
| ophn1 | -0.1418041949 | 0.1008679259 | -1.405840297 | 0.1597715531 | 0.8677911578 |
| anxa4 | -0.1318725268 | 0.09391870884 | -1.404113498 | 0.1602850549 | 0.8764527122 |
| fcer2 | 0.1358944223 | 0.09701170789 | 1.400804349 | 0.1612725877 | 1.145560942 |
| kazn | -0.1412199083 | 0.1009629509 | -1.39873 | 0.1618939645 | 0.8682983447 |
| wwp2 | -0.1354149536 | 0.09688716878 | -1.397656215 | 0.1622163285 | 0.8733534353 |
| pkn3 | -0.1473587667 | 0.1054706782 | -1.397153874 | 0.1623673041 | 0.8629843119 |
| ttr | -0.1456425315 | 0.1043637077 | -1.395528529 | 0.1628565179 | 0.8644666676 |
| tgfbr1 | 0.1405209389 | 0.100834105 | 1.393585424 | 0.1634428325 | 1.150873177 |
| orm1 | -0.147692667 | 0.1060945925 | -1.392084776 | 0.1638967277 | 0.8626962092 |
| wfdc12 | 0.1445204626 | 0.1039465167 | 1.390334829 | 0.1644272262 | 1.155485339 |
| egf | -0.1261348435 | 0.09088402501 | -1.387865947 | 0.1651778688 | 0.8814959748 |

| Protein | beta | se | z | p_val | HR |
| --- | --- | --- | --- | --- | --- |
| timp2 | 0.1405358754 | 0.1013492904 | 1.386648834 | 0.1655488697 | 1.150890367 |
| get3 | -0.1470998567 | 0.1062033349 | -1.385077566 | 0.1660287508 | 0.863207776 |
| tpmt | -0.1314428616 | 0.09505322159 | -1.382834368 | 0.1667156583 | 0.8768293743 |
| arsa | -0.1415364062 | 0.1025271257 | -1.380477656 | 0.1674396246 | 0.8680235736 |
| brd3 | -0.1488558998 | 0.107882983 | -1.379790359 | 0.167651202 | 0.8616932761 |
| arg1 | 0.1291284926 | 0.09358889177 | 1.379741657 | 0.167666202 | 1.137836318 |
| mprip | -0.1507621949 | 0.1093150122 | -1.379153621 | 0.1678473942 | 0.8600521992 |
| pdgfb | -0.1272005108 | 0.09224276271 | -1.378975511 | 0.1679023046 | 0.8805570937 |
| ifng | 0.13371526 | 0.09706026086 | 1.377651974 | 0.1683107658 | 1.143067296 |
| prtfdc1 | -0.1251974747 | 0.09095089244 | -1.376539266 | 0.1686547392 | 0.8823226489 |
| kiaa2013 | -0.1450373382 | 0.1053665326 | -1.376502905 | 0.1686659886 | 0.8649899953 |
| serpini2 | -0.1287719303 | 0.09363756008 | -1.375216635 | 0.1690642925 | 0.8791744557 |
| xiap | -0.1384208722 | 0.1007188441 | -1.374329436 | 0.169339432 | 0.8707321477 |
| eno1 | -0.1256076712 | 0.0914725222 | -1.373173803 | 0.1696983216 | 0.8819607975 |
| camlg | -0.1424352035 | 0.1039425055 | -1.370326824 | 0.1705849032 | 0.8672437468 |
| chrm1 | 0.1446591002 | 0.1055937044 | 1.369959516 | 0.1706995393 | 1.155645544 |
| ceacam18 | -0.1422102295 | 0.1038691098 | -1.369129183 | 0.1709588976 | 0.8674388761 |
| pdia4 | -0.1361335748 | 0.09947434488 | -1.368529493 | 0.1711463966 | 0.8727260505 |
| tacstd2 | 0.1396244383 | 0.1020465627 | 1.368242443 | 0.1712362002 | 1.149841881 |
| ddhd2 | -0.1404352457 | 0.1026392151 | -1.368241617 | 0.1712364588 | 0.8689799333 |
| sele | 0.1334841635 | 0.09764358379 | 1.367055144 | 0.1716080218 | 1.142803168 |
| amfr | -0.1340758495 | 0.09809883077 | -1.366742585 | 0.1717060049 | 0.8745237299 |
| chmp6 | -0.1351909924 | 0.09893576474 | -1.36645219 | 0.1717970772 | 0.8735490545 |
| fli1 | -0.1265432686 | 0.09273074835 | -1.364631159 | 0.1723690063 | 0.8811360232 |
| tymp | -0.130329317 | 0.09552669082 | -1.364323582 | 0.172465747 | 0.8778063067 |
| vav3 | -0.1360253008 | 0.1000158499 | -1.360037444 | 0.1738180752 | 0.8728205492 |
| crtam | 0.1339517922 | 0.09853751859 | 1.359398878 | 0.1740202264 | 1.143337701 |
| ehd3 | -0.1389248976 | 0.102260793 | -1.358535305 | 0.1742938879 | 0.8702933871 |
| enoph1 | -0.1419442195 | 0.1047953959 | -1.354489081 | 0.1755803946 | 0.8676696541 |
| pappa | -0.1410295153 | 0.1041693608 | -1.353848332 | 0.1757847702 | 0.8684636783 |
| ceacam21 | 0.1332816562 | 0.09845146368 | 1.353780342 | 0.175806467 | 1.142571765 |
| cgb3_cgb5_cgb8 | 0.1503347628 | 0.1111755526 | 1.352228608 | 0.1763021945 | 1.162223247 |
| pon2 | -0.1217068381 | 0.09050616139 | -1.344735388 | 0.1787107022 | 0.8854078983 |
| pde1c | 0.1448317566 | 0.1079838783 | 1.341234996 | 0.1798441675 | 1.155845091 |
| rab44 | 0.143765735 | 0.1072415922 | 1.340578147 | 0.1800574564 | 1.154613591 |
| sorbs1 | 0.1518074769 | 0.1135522941 | 1.336894847 | 0.1812569631 | 1.16393613 |
| il1rn | 0.1592472265 | 0.1192319434 | 1.335608747 | 0.1816771898 | 1.172627816 |
| msr1 | 0.1398184563 | 0.1047021645 | 1.335392224 | 0.1817480084 | 1.150064993 |
| serpina12 | -0.1430289465 | 0.1071180571 | -1.335245899 | 0.1817958788 | 0.8667289798 |
| muc16 | -0.1349872432 | 0.1011334012 | -1.334744423 | 0.181960009 | 0.8737270576 |
| lrtm2 | -0.1423570759 | 0.106657705 | -1.334709723 | 0.1819713701 | 0.8673115052 |
| scn2b | 0.1365052744 | 0.1022924838 | 1.334460455 | 0.1820529987 | 1.146260924 |
| cxadr | 0.1339441232 | 0.1003767028 | 1.334414455 | 0.1820680652 | 1.143328932 |
| stat2 | -0.1363584099 | 0.1022053503 | -1.334161172 | 0.1821510417 | 0.8725298531 |
| ccdc28a | 0.1384454254 | 0.1038843124 | 1.33268847 | 0.1826340584 | 1.148487002 |
| dbn1 | -0.1322141924 | 0.09921699542 | -1.332576056 | 0.1826709672 | 0.8761533096 |
| stx5 | -0.1369536144 | 0.1027892922 | -1.332372386 | 0.1827378515 | 0.8720106739 |
| prcp | 0.1336060142 | 0.1002933561 | 1.332152192 | 0.1828101828 | 1.142942428 |
| procr | 0.1388096772 | 0.1042402573 | 1.331632143 | 0.1829810971 | 1.148905416 |
| farsa | -0.1354466338 | 0.1018072943 | -1.330421702 | 0.183379368 | 0.8733257678 |
| tf | 0.1373604939 | 0.1033671098 | 1.32886074 | 0.1838939186 | 1.147241648 |
| chrdl2 | -0.1345167258 | 0.1012451376 | -1.32862406 | 0.1839720301 | 0.8741382581 |
| pikfyve | -0.1364026226 | 0.1027266722 | -1.327820903 | 0.1842372797 | 0.872491277 |
| ppt1 | -0.1423069107 | 0.1074923405 | -1.323879544 | 0.1855430529 | 0.8673550151 |
| ada | 0.1300143674 | 0.09822177235 | 1.323681749 | 0.1856087623 | 1.138844745 |
| foxo1 | -0.1232111236 | 0.09312931677 | -1.323011141 | 0.1858316729 | 0.8840769933 |
| cct5 | -0.1257742523 | 0.09509952422 | -1.32255396 | 0.1859837536 | 0.8818138917 |
| sart1 | 0.1533005287 | 0.116015814 | 1.321376142 | 0.1863759771 | 1.165675245 |
| dok1 | -0.1342539277 | 0.1016579464 | -1.320643712 | 0.1866201905 | 0.8743680101 |
| cxcl9 | 0.1372464842 | 0.1040250791 | 1.319359575 | 0.1870489297 | 1.147110858 |
| ccl4 | -0.1307717538 | 0.09914761631 | -1.318960139 | 0.1871824388 | 0.8774180188 |
| itgal | 0.1344201112 | 0.1020755714 | 1.316868565 | 0.187882685 | 1.143873273 |
| adamts8 | 0.1216385188 | 0.09256037671 | 1.314153238 | 0.1887946412 | 1.129345791 |
| ifit1 | -0.1399758788 | 0.1066633246 | -1.31231498 | 0.1894138797 | 0.8693792056 |
| cdc42bpb | -0.132221214 | 0.1007560609 | -1.312290425 | 0.1894221615 | 0.8761471576 |
| selp | -0.1259077673 | 0.09606742183 | -1.310618781 | 0.1899865907 | 0.8816961642 |
| tax1bp1 | -0.1355112736 | 0.1034993438 | -1.309295969 | 0.1904341146 | 0.873269318 |
| hbegf | -0.120790679 | 0.09225725594 | -1.309281073 | 0.1904391586 | 0.8862194445 |
| dynlt3 | -0.1334550426 | 0.1019486529 | -1.309041746 | 0.1905202102 | 0.8750668088 |
| aspn | 0.1366403572 | 0.1044166999 | 1.308606357 | 0.1906677266 | 1.146415774 |

| Protein | beta | se | z | p_val | HR |
|---|---|---|---|---|---|
| brd2 | -0.1322991898 | 0.1011244412 | -1.308281047 | 0.190778001 | 0.876078842 |
| ptx3 | 0.1291129396 | 0.09873988316 | 1.307606769 | 0.1910067198 | 1.137818622 |
| rspo3 | 0.1269564112 | 0.09713256406 | 1.307042725 | 0.1911982015 | 1.135367527 |
| cbln4 | -0.1202376838 | 0.09206045286 | -1.30607313 | 0.1915276896 | 0.8867096551 |
| dlg4 | -0.1319092225 | 0.1010140622 | -1.305850093 | 0.191603541 | 0.8764205507 |
| fam171a2 | -0.1313683936 | 0.1006446282 | -1.305269799 | 0.191800994 | 0.8768946725 |
| erbb3 | -0.1320602068 | 0.1012446525 | -1.304367229 | 0.1921084031 | 0.876288235 |
| daam1 | -0.1316782713 | 0.1010811215 | -1.302698954 | 0.1926775594 | 0.8766229845 |
| sh3bp1 | -0.1352945511 | 0.1039105513 | -1.302028999 | 0.1929064727 | 0.8734585956 |
| twf2 | -0.130161819 | 0.09999384705 | -1.301698283 | 0.1930195469 | 0.8779533499 |
| ift20 | -0.1385906054 | 0.1065344498 | -1.300899433 | 0.1932928803 | 0.8705843681 |
| anxa10 | 0.125375875 | 0.09641018234 | 1.300442256 | 0.1934494352 | 1.133574455 |
| cd101 | 0.1403961858 | 0.1080267837 | 1.299642375 | 0.1937235691 | 1.150729611 |
| cfhr2 | 0.1332192573 | 0.1027038979 | 1.297119778 | 0.1945899774 | 1.142500472 |
| zbtb17 | 0.130979138 | 0.1010408213 | 1.296299222 | 0.1948724165 | 1.139944 |
| cd69 | -0.1179380406 | 0.09107807497 | -1.294911434 | 0.1953507838 | 0.8887511174 |
| lilrb2 | 0.1200259766 | 0.09272277769 | 1.294460537 | 0.1955063921 | 1.12752614 |
| sell | -0.1380725691 | 0.1066718347 | -1.294367623 | 0.1955384686 | 0.8710354791 |
| lrig1 | -0.1205437162 | 0.09317760236 | -1.293698412 | 0.1957696141 | 0.8864383348 |
| nit1 | -0.1402092509 | 0.1083819655 | -1.2936585 | 0.1957834059 | 0.8691763404 |
| lect2 | 0.1505832698 | 0.1165360757 | 1.292160122 | 0.1963016986 | 1.162512103 |
| sh3bgrl2 | -0.1329208849 | 0.102875502 | -1.29205576 | 0.1963378352 | 0.8755343573 |
| cndp1 | -0.1252742894 | 0.0970276906 | -1.291118944 | 0.196662436 | 0.8822548762 |
| pecr | -0.1353157394 | 0.1049407225 | -1.289449284 | 0.1972419372 | 0.8734400887 |
| baiap2 | 0.1246125656 | 0.09694872236 | 1.285345104 | 0.1986717153 | 1.132709517 |
| ccdc134 | -0.1353505226 | 0.1053176528 | -1.285164633 | 0.1987347597 | 0.8734097082 |
| sugp1 | -0.131031774 | 0.1022422611 | -1.28158134 | 0.1999895495 | 0.8771899021 |
| per3 | -0.1283953683 | 0.1003584009 | -1.279368416 | 0.2007673505 | 0.8795055817 |
| irag2 | -0.1232735223 | 0.09639584639 | -1.278826079 | 0.2009583082 | 0.8840218298 |
| ddx39a | -0.1358487955 | 0.1063000164 | -1.277975302 | 0.2012581349 | 0.8729746202 |
| polr2a | 0.1370285225 | 0.1072789215 | 1.277310777 | 0.2014925505 | 1.146860859 |
| lrrc37a2 | 0.1319803412 | 0.1033499829 | 1.277023348 | 0.2015940044 | 1.141085887 |
| angpt1 | -0.1203578691 | 0.09425535903 | -1.276933963 | 0.2016255624 | 0.8866030921 |
| eno3 | 0.1541910942 | 0.1208274127 | 1.276126757 | 0.2019107146 | 1.166713818 |
| mki67 | -0.1312725058 | 0.1029212538 | -1.275465474 | 0.2021445369 | 0.87697876 |
| asah1 | -0.1367282163 | 0.1072900031 | -1.274379834 | 0.2025288356 | 0.8722072456 |
| stk4 | -0.118026135 | 0.09268866205 | -1.273361082 | 0.2028899406 | 0.8886728268 |
| pde5a | -0.1293986194 | 0.1016459881 | -1.273032235 | 0.2030066034 | 0.8786236593 |
| cela2a | -0.1208242012 | 0.09492396164 | -1.272852493 | 0.20307039 | 0.886189737 |
| bank1 | -0.1177888449 | 0.09255192026 | -1.272678564 | 0.2031321273 | 0.8888837251 |
| tpr | -0.1452173582 | 0.1141419968 | -1.272251777 | 0.2032836763 | 0.8648342938 |
| scrg1 | 0.1448466087 | 0.1138971014 | 1.271732177 | 0.203468294 | 1.155862257 |
| myo6 | -0.1308946339 | 0.1029686358 | -1.271208781 | 0.2036543836 | 0.8773102082 |
| il22ra1 | 0.1258426194 | 0.0989947171 | 1.271205405 | 0.2036555842 | 1.134103668 |
| sarg | -0.1297529518 | 0.1021291957 | -1.270478544 | 0.2039142208 | 0.8783123896 |
| obp2b | -0.145923694 | 0.1148670681 | -1.270370145 | 0.2039528126 | 0.8642236462 |
| mars1 | -0.1328468027 | 0.1046357103 | -1.26961247 | 0.2042227052 | 0.8755992213 |
| tppp3 | -0.1183814007 | 0.09325677987 | -1.269413343 | 0.2042936798 | 0.888357168 |
| evi2b | 0.1401797406 | 0.1105094138 | 1.268486871 | 0.2046241362 | 1.150480568 |
| cmip | -0.1274550986 | 0.1005128581 | -1.268047701 | 0.2047809165 | 0.8803329431 |
| mapk9 | -0.1224587913 | 0.09674210633 | -1.265827218 | 0.2055749482 | 0.8847423632 |
| kit | 0.1225193181 | 0.09688996599 | 1.264520189 | 0.206043379 | 1.130340956 |
| stk24 | -0.1176420452 | 0.09304713377 | -1.264327448 | 0.2061125217 | 0.8890142226 |
| mepe | -0.121606064 | 0.09627080492 | -1.263166587 | 0.2065293182 | 0.885497129 |
| il12b | 0.1190109503 | 0.09437105274 | 1.261095928 | 0.2072742876 | 1.126382252 |
| lag3 | 0.1225440142 | 0.0972046024 | 1.260681194 | 0.2074237322 | 1.130368871 |
| irak1 | -0.1200596499 | 0.09531347904 | -1.259629289 | 0.2078031245 | 0.8868675336 |
| ctrb1 | -0.1119284443 | 0.0888585065 | -1.259625541 | 0.2078044773 | 0.8941082337 |
| c1ql2 | 0.1354862934 | 0.1076033816 | 1.259126725 | 0.2079845628 | 1.1450935 |
| cd36 | -0.1306782567 | 0.1038352382 | -1.258515499 | 0.2082053854 | 0.8775000588 |
| erp44 | -0.123795031 | 0.0984240651 | -1.257771978 | 0.2084742327 | 0.8835609249 |
| nfkb1 | -0.1303179497 | 0.1037146325 | -1.256504955 | 0.2089329504 | 0.8778162851 |
| rab6a | -0.1207079597 | 0.09615580624 | -1.255337191 | 0.2093563795 | 0.886292755 |
| coch | -0.1379712513 | 0.1099477627 | -1.254880026 | 0.209522316 | 0.8711237351 |
| klk13 | -0.112714106 | 0.08982316171 | -1.254844562 | 0.2095351921 | 0.893406043 |
| pgm2 | -0.1312505273 | 0.1047141152 | -1.253417718 | 0.2100537268 | 0.8769980349 |
| sult1a1 | -0.1130217863 | 0.09026311276 | -1.252137034 | 0.2105199351 | 0.8931312019 |
| ccl20 | 0.1259725613 | 0.1007253685 | 1.250653764 | 0.2110608261 | 1.134251045 |
| ednrb | 0.1333046186 | 0.1066791251 | 1.249584851 | 0.21145124 | 1.142598002 |
| pdzk1 | 0.1450433385 | 0.1161225607 | 1.249053911 | 0.2116453565 | 1.156089672 |

| Protein | beta | se | z | p_val | HR |
|---|---|---|---|---|---|
| klk10 | 0.1187100495 | 0.09505646792 | 1.248837161 | 0.2117246392 | 1.126043374 |
| rarres1 | 0.1204065385 | 0.09643599748 | 1.248564247 | 0.2118244962 | 1.127955316 |
| ankmy2 | -0.1236547605 | 0.09907882568 | -1.248044269 | 0.2120148459 | 0.8836848711 |
| syt1 | 0.1228975587 | 0.09859848356 | 1.246444714 | 0.2126011741 | 1.130768578 |
| znf75d | -0.1301950241 | 0.1044793253 | -1.246131937 | 0.2127159615 | 0.8779241979 |
| reep4 | -0.1267074496 | 0.1019240974 | -1.243154984 | 0.2138107276 | 0.8809913693 |
| gpd1 | 0.1396946638 | 0.1123855452 | 1.242994938 | 0.2138696987 | 1.149922632 |
| cuzd1 | 0.1234585958 | 0.09951190442 | 1.240641474 | 0.2147382236 | 1.131403159 |
| kiaa1549 | 0.1299021718 | 0.1047629338 | 1.239963097 | 0.214989044 | 1.138716979 |
| art5 | 0.1359180094 | 0.1096779028 | 1.239246976 | 0.2152540487 | 1.145587962 |
| arl2bp | -0.1260496031 | 0.1017579314 | -1.238720181 | 0.2154491424 | 0.881571117 |
| serpina4 | 0.1285951007 | 0.1038264251 | 1.238558494 | 0.2155090473 | 1.137229567 |
| itga11 | -0.1222200085 | 0.09869471451 | -1.238364274 | 0.2155810213 | 0.8849536498 |
| frzb | -0.1216955531 | 0.09832468716 | -1.237690722 | 0.2158307606 | 0.8854178902 |
| csf3r | 0.1274511478 | 0.1032097534 | 1.234875035 | 0.216877016 | 1.135929374 |
| relt | 0.1275280771 | 0.1032834906 | 1.234738257 | 0.2169279328 | 1.136016764 |
| btn1a1 | 0.132880973 | 0.1076460724 | 1.234424722 | 0.2170446816 | 1.142114048 |
| ocln | 0.1341789663 | 0.1087045105 | 1.234345895 | 0.2170740407 | 1.143597467 |
| ntrk3 | 0.1173821901 | 0.09514980861 | 1.233656608 | 0.2173308897 | 1.124549139 |
| gfra3 | 0.1164836224 | 0.09453921529 | 1.232119624 | 0.2179044018 | 1.123539109 |
| itgb2 | 0.11538875 | 0.09375286455 | 1.230775727 | 0.2184067564 | 1.122309651 |
| serpinb9 | -0.1170413492 | 0.09510553421 | -1.230647093 | 0.2184548838 | 0.8895484103 |
| myh4 | 0.1311513516 | 0.1066104534 | 1.230192232 | 0.218625128 | 1.14014033 |
| gpr15l | 0.1391052117 | 0.1132136684 | 1.228696267 | 0.2191857064 | 1.149245008 |
| thop1 | -0.1167662393 | 0.09521232274 | -1.226377384 | 0.2200566929 | 0.8897931676 |
| epn1 | 0.1270728789 | 0.1036959036 | 1.225437789 | 0.2204103161 | 1.135499769 |
| ankra2 | -0.1325384555 | 0.1084183482 | -1.222472558 | 0.2215289751 | 0.8758692515 |
| evi5 | -0.1238660681 | 0.1014190903 | -1.22132892 | 0.2219615077 | 0.8834981616 |
| stxbp3 | -0.1124603859 | 0.0924261873 | -1.216758899 | 0.223695962 | 0.8936327468 |
| aprt | 0.1145748482 | 0.09425838355 | 1.215540134 | 0.2241601514 | 1.121396572 |
| batf | -0.1314091357 | 0.108172621 | -1.214809575 | 0.224438728 | 0.8768589467 |
| xrcc4 | -0.1159099995 | 0.09542721545 | -1.214643003 | 0.2245022799 | 0.8905553701 |
| mitd1 | -0.113490156 | 0.09349419603 | -1.213873811 | 0.224795915 | 0.8927129842 |
| gpi | -0.1280092472 | 0.1055243423 | -1.213077896 | 0.2251000401 | 0.879845243 |
| chrdl1 | 0.1198584489 | 0.09888670763 | 1.212078466 | 0.225482346 | 1.127337264 |
| cd7 | 0.1253715196 | 0.1035150227 | 1.211143237 | 0.2258405132 | 1.133569518 |
| siglec15 | 0.1082616168 | 0.08942733397 | 1.210609911 | 0.2260449439 | 1.114339237 |
| reck | 0.1387106104 | 0.1145934031 | 1.21045895 | 0.2261028332 | 1.148791604 |
| eif4e | -0.1241780364 | 0.1025943129 | -1.210379336 | 0.2261333672 | 0.8832225811 |
| flt1 | 0.1159025293 | 0.095846697 | 1.209249071 | 0.2265671704 | 1.122886418 |
| phldb2 | 0.1332107863 | 0.1101833471 | 1.208992009 | 0.2266659152 | 1.142490794 |
| ube2l6 | -0.1287768057 | 0.1067620534 | -1.206203904 | 0.2277388789 | 0.8791701694 |
| htra2 | -0.1147230695 | 0.09511835106 | -1.206108687 | 0.2277755858 | 0.8916130246 |
| snu13 | -0.1307754675 | 0.1084382623 | -1.20599007 | 0.2278213194 | 0.8774147604 |
| rnf43 | 0.1353832562 | 0.1123996617 | 1.204480993 | 0.2284037241 | 1.144975519 |
| gp6 | -0.1112995619 | 0.09251512849 | -1.203041748 | 0.2289601655 | 0.8946706995 |
| cd200r1 | 0.1161560533 | 0.09659592071 | 1.202494396 | 0.2291720361 | 1.123171133 |
| csf2 | 0.1275153578 | 0.1061114021 | 1.201712118 | 0.2294750844 | 1.136002315 |
| dcxr | -0.1280854932 | 0.1067821336 | -1.199503034 | 0.2303324056 | 0.8797781608 |
| mapk13 | 0.1298551841 | 0.1082633078 | 1.199438542 | 0.2303574684 | 1.138663475 |
| f11 | 0.1226457787 | 0.1022528842 | 1.199435885 | 0.230358501 | 1.130483909 |
| pgf | 0.1334060107 | 0.1112675698 | 1.198965799 | 0.2305412446 | 1.142713858 |
| nfatc3 | -0.1136060845 | 0.0948160493 | -1.198173573 | 0.2308494514 | 0.8926094993 |
| hadh | 0.1237874634 | 0.103409839 | 1.19705692 | 0.2312843696 | 1.131775302 |
| cemip2 | 0.1296288095 | 0.1083296732 | 1.196614055 | 0.2314570196 | 1.138405739 |
| bhlhe40 | -0.12114339 | 0.1012671616 | -1.19627516 | 0.2315891988 | 0.8859069203 |
| c9 | -0.117091654 | 0.09797415305 | -1.195128004 | 0.2320370223 | 0.8895036629 |
| creld2 | -0.1145420914 | 0.09598001538 | -1.193395218 | 0.232714627 | 0.8917744016 |
| slc51b | 0.1233274556 | 0.1033790304 | 1.192963942 | 0.2328834952 | 1.131254796 |
| c1qtnf9 | -0.1285700709 | 0.1080541119 | -1.189867453 | 0.2340984926 | 0.8793519433 |
| bglap | -0.1228671789 | 0.1032720297 | -1.189743043 | 0.2341474021 | 0.8843811193 |
| tcof1 | 0.1294190886 | 0.1088966631 | 1.1884578 | 0.234653096 | 1.138167017 |
| snap25 | -0.1187229291 | 0.1000693969 | -1.186405963 | 0.2354620181 | 0.8880538205 |
| mucl3 | 0.1256988994 | 0.105980526 | 1.186056572 | 0.2355999591 | 1.133940687 |
| fgd3 | -0.120270662 | 0.1014713637 | -1.185267032 | 0.2359118842 | 0.8866804135 |
| il1rap | -0.1060342369 | 0.08952786322 | -1.184371358 | 0.236266093 | 0.8993938551 |
| il17rb | 0.1141473893 | 0.09654391488 | 1.182336447 | 0.2370722293 | 1.120917324 |
| ca6 | -0.1208786731 | 0.1023903603 | -1.180566928 | 0.2377748084 | 0.8861414659 |
| gzma | 0.115707482 | 0.09803740977 | 1.180238057 | 0.2379055467 | 1.122667424 |
| lats1 | -0.1163036532 | 0.09879518898 | -1.177219806 | 0.2391077855 | 0.8902048687 |

| Protein | beta | se | z | p_val | HR |
|---|---|---|---|---|---|
| dlgap5 | -0.1244010015 | 0.1056923117 | -1.177010887 | 0.2391911609 | 0.8830256752 |
| ervv_1 | -0.1256635591 | 0.1069480917 | -1.174995805 | 0.2399963941 | 0.881911508 |
| prg3 | -0.1202620326 | 0.1023821029 | -1.174639211 | 0.2401390888 | 0.8866880651 |
| stoml2 | -0.1264413302 | 0.107728163 | -1.173707289 | 0.2405122895 | 0.8812258494 |
| celsr2 | -0.1292603637 | 0.1103164254 | -1.171723642 | 0.2413080278 | 0.8787451424 |
| skap2 | -0.1084543855 | 0.09258895632 | -1.171353365 | 0.2414567694 | 0.8972198201 |
| dpp7 | -0.1043177851 | 0.08911459388 | -1.17060271 | 0.2417585073 | 0.9009389469 |
| sdccag8 | -0.1192862005 | 0.1019292859 | -1.170283883 | 0.2418867455 | 0.887553746 |
| fgl1 | 0.1282851263 | 0.1096943687 | 1.169477776 | 0.2422111902 | 1.13687711 |
| marco | 0.1091994652 | 0.09337745139 | 1.169441483 | 0.2422258048 | 1.115384809 |
| nxpe4 | 0.1225660975 | 0.1048578061 | 1.168879096 | 0.242452347 | 1.130393834 |
| azi2 | -0.1155158002 | 0.0988340437 | -1.168785531 | 0.2424900514 | 0.8909064956 |
| ssh3 | -0.1229586631 | 0.1053827738 | -1.166781426 | 0.2432986497 | 0.884300216 |
| itgav | -0.1182407036 | 0.1014358008 | -1.165670332 | 0.2437477596 | 0.888482166 |
| tada3 | -0.1166829157 | 0.1001136794 | -1.16550422 | 0.2438149531 | 0.8898673113 |
| slirp | -0.1205807254 | 0.1035409288 | -1.164570637 | 0.244192835 | 0.886405529 |
| mn1 | -0.1235171167 | 0.1060643292 | -1.164549077 | 0.244201567 | 0.8838065133 |
| ppm1a | -0.1091117008 | 0.09372915267 | -1.164117009 | 0.2443765967 | 0.8966302576 |
| c2cd2l | -0.1111058781 | 0.09550569398 | -1.163342974 | 0.2446903764 | 0.8948439995 |
| rasgrf1 | 0.1267055926 | 0.1089576284 | 1.16288868 | 0.2448746708 | 1.135082792 |
| asrgl1 | -0.1228045996 | 0.1056923969 | -1.161905712 | 0.2452737668 | 0.8844364649 |
| serpina3 | -0.1223374243 | 0.105303112 | -1.161764567 | 0.2453311106 | 0.8848497483 |
| cd84 | -0.107663911 | 0.09270183175 | -1.161400039 | 0.2454792531 | 0.8979293299 |
| naaa | -0.1096342425 | 0.09441700539 | -1.161170513 | 0.2455725639 | 0.8961618532 |
| serpini1 | -0.1220683823 | 0.1052032108 | -1.160310426 | 0.2459224411 | 0.8850878421 |
| lta | -0.112488543 | 0.0970016727 | -1.159655703 | 0.246189012 | 0.8936075851 |
| blmh | -0.1080213611 | 0.09329603486 | -1.157834427 | 0.2469316112 | 0.8976084223 |
| nlgn2 | 0.119629981 | 0.103350564 | 1.15751648 | 0.2470614102 | 1.127079734 |
| c1rl | 0.1261854999 | 0.1091003687 | 1.156600124 | 0.2474357717 | 1.134492597 |
| iglc2 | 0.1262150975 | 0.1091369398 | 1.156483752 | 0.2474833419 | 1.134526176 |
| ncr3lg1 | 0.1233910687 | 0.1068911136 | 1.154362271 | 0.2483516765 | 1.131326761 |
| pltp | -0.1112068293 | 0.09634526099 | -1.154253237 | 0.2483963624 | 0.8947536685 |
| mmp3 | 0.1389578305 | 0.1204110101 | 1.154029273 | 0.2484881679 | 1.149075643 |
| vipr1 | 0.1227403224 | 0.1064302222 | 1.153246887 | 0.2488090635 | 1.130590794 |
| ddc | -0.1088207074 | 0.09436753277 | -1.15315834 | 0.2488453996 | 0.896891209 |
| mycbp2 | -0.1190125138 | 0.1032293754 | -1.152893866 | 0.24895395 | 0.887796691 |
| bpifb2 | -0.1312608291 | 0.1138711682 | -1.152713467 | 0.2490280119 | 0.8769890003 |
| crybb1 | -0.1177974975 | 0.102346483 | -1.150967713 | 0.2497455187 | 0.888876034 |
| tars1 | -0.1170247918 | 0.1017457232 | -1.150169148 | 0.2500742104 | 0.889563139 |
| clasp1 | -0.1189982971 | 0.1035167509 | -1.149555952 | 0.250326809 | 0.8878093126 |
| f9 | -0.1224899043 | 0.1066913519 | -1.14807716 | 0.250936712 | 0.8847148367 |
| foxj3 | -0.1201216119 | 0.1046714234 | -1.147606557 | 0.2511310216 | 0.8868125832 |
| itga2 | -0.1256910987 | 0.1096284608 | -1.146518867 | 0.2515805251 | 0.8818872209 |
| copb2 | -0.1179519546 | 0.1030107137 | -1.145045505 | 0.2521903078 | 0.8887387514 |
| hspa2 | 0.1215486673 | 0.1061613103 | 1.144943171 | 0.2522326993 | 1.129244322 |
| csf2ra | -0.1123423096 | 0.09818229392 | -1.144221683 | 0.2525317128 | 0.8937382699 |
| agxt | 0.1169056411 | 0.1021787047 | 1.144129214 | 0.2525700538 | 1.124013364 |
| scgb2a2 | 0.1198581548 | 0.1048293055 | 1.143364961 | 0.2528870941 | 1.127336933 |
| defb116 | 0.1222579478 | 0.1069455988 | 1.14317886 | 0.2529643374 | 1.130045557 |
| dennd2b | 0.1202722251 | 0.1052188166 | 1.143067646 | 0.253010506 | 1.127803826 |
| ca14 | 0.1140641156 | 0.09980418887 | 1.14287904 | 0.253088816 | 1.120823985 |
| gga1 | -0.1052496508 | 0.09214462433 | -1.142222366 | 0.2533616015 | 0.9000997839 |
| slc27a4 | -0.1080867623 | 0.09470773904 | -1.14126642 | 0.2537590715 | 0.8975497196 |
| lgals7_lgals7b | 0.1094830869 | 0.09597252154 | 1.14077535 | 0.253963421 | 1.115701201 |
| dok2 | -0.1052073895 | 0.0922532926 | -1.140418803 | 0.254111863 | 0.9001378241 |
| stambp | -0.1104523313 | 0.0968530977 | -1.140410931 | 0.2541151409 | 0.8954290131 |
| axl | 0.107756118 | 0.09450637619 | 1.140199448 | 0.2542032174 | 1.113776082 |
| ak2 | -0.1146149413 | 0.1005445454 | -1.139941912 | 0.2543105024 | 0.8917094383 |
| trim24 | -0.1236285113 | 0.1087071172 | -1.137262347 | 0.2554286302 | 0.8837080675 |
| washc3 | -0.1167776272 | 0.102776954 | -1.136223859 | 0.2558628876 | 0.8897830347 |
| mansc4 | -0.1204511625 | 0.1061709168 | -1.134502424 | 0.2565838574 | 0.8865203817 |
| thbs2 | 0.1020831411 | 0.08998241054 | 1.134478844 | 0.2565937431 | 1.107475545 |
| eif2ak2 | -0.1160696174 | 0.1023675344 | -1.133851841 | 0.2568567009 | 0.8904132329 |
| mrps16 | -0.1164110234 | 0.1026854945 | -1.133665704 | 0.2569348009 | 0.8901092924 |
| gusb | -0.1101477583 | 0.09721533453 | -1.13302864 | 0.2572022263 | 0.8957017782 |
| cacybp | -0.1164672079 | 0.102795839 | -1.132995353 | 0.2572162046 | 0.8900592834 |
| klk8 | 0.1161250944 | 0.1025359591 | 1.132530434 | 0.2574114966 | 1.123136361 |
| smc3 | -0.1210817756 | 0.1071398273 | -1.130128531 | 0.2584220692 | 0.8859615066 |
| chek2 | -0.1095812431 | 0.09701076645 | -1.129578161 | 0.2586540177 | 0.8962093505 |
| oscar | 0.1052225568 | 0.09317661139 | 1.129280785 | 0.2587794043 | 1.110957834 |

| Protein | beta | se | z | p_val | HR |
|---|---|---|---|---|---|
| hrg | 0.1175149775 | 0.1041545794 | 1.128274707 | 0.2592039218 | 1.124698475 |
| tjap1 | -0.1065156635 | 0.09443722454 | -1.127899131 | 0.2593625208 | 0.8989609672 |
| gmfg | -0.1137101822 | 0.1008917204 | -1.127051673 | 0.2597206343 | 0.8925165856 |
| klkb1 | -0.124944814 | 0.110960321 | -1.126031476 | 0.2601521963 | 0.8825456054 |
| mllt1 | 0.1225261473 | 0.108816462 | 1.125989074 | 0.2601701439 | 1.130348675 |
| snx18 | -0.1146699676 | 0.1019430668 | -1.124843221 | 0.2606554758 | 0.8916603722 |
| tnip1 | -0.1147359817 | 0.1021837114 | -1.122840227 | 0.2615053574 | 0.891601512 |
| lama1 | -0.1225918613 | 0.109245214 | -1.12217146 | 0.2617895447 | 0.8846246385 |
| fabp5 | 0.1130399659 | 0.1007358983 | 1.122141836 | 0.2618021383 | 1.119676681 |
| tnfrsf21 | 0.1049704882 | 0.09362194043 | 1.121216755 | 0.2621956096 | 1.110677832 |
| pdcd5 | 0.1118026773 | 0.09972003164 | 1.121165682 | 0.2622173444 | 1.118292174 |
| s100a11 | 0.1158421432 | 0.1034404111 | 1.119892525 | 0.2627595639 | 1.122818614 |
| lrrc25 | 0.1063861076 | 0.09504159367 | 1.119363675 | 0.2629850212 | 1.112251242 |
| cradd | -0.1051362388 | 0.09394798487 | -1.119089877 | 0.263101798 | 0.9002018718 |
| tnf | 0.1131107389 | 0.1011985183 | 1.117711413 | 0.2636902666 | 1.119755927 |
| sh3glb2 | 0.1205668303 | 0.1078805554 | 1.117595565 | 0.2637397637 | 1.128136132 |
| ikbkg | -0.1032532622 | 0.09271067942 | -1.113714869 | 0.2654015282 | 0.9018985277 |
| gh1 | 0.1129494871 | 0.1015518246 | 1.112234935 | 0.2660371507 | 1.119575379 |
| tmprss11b | -0.1206003765 | 0.1087751156 | -1.108712924 | 0.2675540446 | 0.8863881104 |
| lxn | -0.1056374005 | 0.09542658441 | -1.107001798 | 0.268293151 | 0.8997508381 |
| prr5 | 0.1128778975 | 0.1019729792 | 1.106939293 | 0.268320176 | 1.119495231 |
| ehbp1 | -0.1137669686 | 0.1028936881 | -1.105674903 | 0.2688672564 | 0.8924659042 |
| nxph3 | 0.1235576184 | 0.1118150322 | 1.105017957 | 0.2691518085 | 1.131515199 |
| tap1 | 0.1169825592 | 0.1059248523 | 1.104391998 | 0.2694231305 | 1.124099824 |
| ubac1 | 0.1081460475 | 0.09796640073 | 1.103909572 | 0.2696323662 | 1.114210461 |
| fhit | -0.1046870668 | 0.09485976795 | -1.103598175 | 0.2697674832 | 0.9006063081 |
| bsnd | 0.1190130901 | 0.1078800364 | 1.103198461 | 0.2699409897 | 1.126384663 |
| clec1b | -0.1037627092 | 0.09405634023 | -1.103197391 | 0.269941454 | 0.9014391753 |
| cep170 | -0.1140486558 | 0.1035236348 | -1.101667808 | 0.2706061174 | 0.8922145434 |
| tcl1b | -0.1061141249 | 0.09640170733 | -1.100749436 | 0.2710057242 | 0.8993220072 |
| map4k5 | -0.1031863751 | 0.09387086517 | -1.0992375 | 0.2716644858 | 0.9019588551 |
| numb | -0.1110062859 | 0.1013902934 | -1.094841352 | 0.2735861464 | 0.8949331234 |
| upk3bl1 | -0.1155584861 | 0.1055943708 | -1.094362183 | 0.2737961628 | 0.8908684673 |
| pklr | 0.1034862476 | 0.09457875702 | 1.094180669 | 0.2738757481 | 1.109030541 |
| clec4g | 0.1034514451 | 0.09460863972 | 1.093467207 | 0.2741887193 | 1.108991945 |
| hagh | 0.1100570135 | 0.1007361826 | 1.09252714 | 0.274601468 | 1.116341715 |
| dynlt1 | -0.1166324887 | 0.1068669426 | -1.09138042 | 0.2751055243 | 0.8899121858 |
| tgfb1 | -0.1064986485 | 0.09763063864 | -1.090832243 | 0.2753467062 | 0.8989762631 |
| efhd1 | 0.118770789 | 0.1088938872 | 1.090702078 | 0.2754039961 | 1.126111772 |
| txnrd1 | -0.1024047076 | 0.09394666232 | -1.09003029 | 0.2756998013 | 0.9026641627 |
| eif4b | -0.1065328772 | 0.0978101425 | -1.089180268 | 0.2760743986 | 0.8989454928 |
| ifit3 | -0.1087147578 | 0.09990908051 | -1.088136906 | 0.2765346731 | 0.8969862394 |
| lhpp | -0.1024506583 | 0.09420465699 | -1.087532841 | 0.2768013927 | 0.9026226856 |
| lrrc59 | -0.1091681271 | 0.1005251761 | -1.085977973 | 0.2774887374 | 0.8965796655 |
| rtkn2 | 0.1128916915 | 0.1039804587 | 1.085701034 | 0.2776112831 | 1.119510674 |
| psma1 | -0.1009354426 | 0.09335701613 | -1.081176828 | 0.2796184635 | 0.9039913904 |
| vps4b | -0.1104367417 | 0.1022635935 | -1.079922365 | 0.2801767532 | 0.8954429726 |
| naa80 | -0.1077687359 | 0.09985020263 | -1.079304128 | 0.2804521731 | 0.8978352095 |
| pik3ap1 | -0.1038016557 | 0.0961798808 | -1.079245003 | 0.2804785228 | 0.901404068 |
| pak4 | -0.1009291296 | 0.09353014178 | -1.079108057 | 0.2805395598 | 0.9039970973 |
| tnfrsf8 | 0.1037023473 | 0.09622603855 | 1.077695278 | 0.2811697667 | 1.109270229 |
| tmco5a | -0.1122633018 | 0.1041961443 | -1.077422802 | 0.2812914217 | 0.8938088851 |
| bag4 | -0.1136620751 | 0.1055564436 | -1.076789548 | 0.281574296 | 0.892559523 |
| cebpa | 0.119891012 | 0.1115779576 | 1.07450445 | 0.2825966514 | 1.127373975 |
| mme | 0.1087868678 | 0.1012642078 | 1.074287452 | 0.2826938672 | 1.114924699 |
| crtap | -0.114726107 | 0.1068379733 | -1.073832678 | 0.2828976809 | 0.8916103163 |
| bin2 | -0.09824813824 | 0.09154859587 | -1.073180176 | 0.2831902834 | 0.9064239574 |
| nt5c3a | -0.1013163811 | 0.09443550742 | -1.072863205 | 0.2833324973 | 0.9036470908 |
| nexn | -0.1074580409 | 0.1001774962 | -1.072676449 | 0.2834163106 | 0.8981142058 |
| dtd1 | -0.1103317939 | 0.1029514175 | -1.071687954 | 0.2838602132 | 0.8955369523 |
| spink8 | -0.1110552881 | 0.1036606806 | -1.071334738 | 0.2840189455 | 0.8948892708 |
| rnf5 | -0.1090796592 | 0.1020800261 | -1.068570056 | 0.2852634473 | 0.8966589875 |
| esr1 | -0.1118969616 | 0.1047437518 | -1.068292473 | 0.2853886028 | 0.8941363831 |
| crym | -0.1085062286 | 0.1017358453 | -1.066548651 | 0.2861756985 | 0.8971733067 |
| postn | -0.1066617544 | 0.1000583403 | -1.065995638 | 0.2864256136 | 0.8988296468 |
| tppp2 | 0.1155901473 | 0.1085054128 | 1.065293834 | 0.286742982 | 1.122535704 |
| metap1d | -0.1002142133 | 0.0940765481 | -1.065241182 | 0.2867668022 | 0.9046436106 |
| cstb | 0.1103557699 | 0.1037797991 | 1.063364651 | 0.2876166166 | 1.116675279 |
| cep290 | -0.1150502665 | 0.1082172705 | -1.063141456 | 0.2877178069 | 0.8913213392 |
| serpinc1 | 0.1102151773 | 0.1036833053 | 1.0629983 | 0.2877827221 | 1.116518294 |

| Protein | beta | se | z | p_val | HR |
|---|---|---|---|---|---|
| pla2g4a | -0.09692357415 | 0.09124632924 | -1.062218885 | 0.2881363282 | 0.9076253695 |
| phactr2 | -0.1062461961 | 0.1000260024 | -1.062185768 | 0.2881513597 | 0.8992032405 |
| adgrd1 | 0.1260753538 | 0.1187703817 | 1.061504998 | 0.2884604628 | 1.134367644 |
| tmem25 | 0.1072528426 | 0.1012606799 | 1.059175613 | 0.2895198094 | 1.113215687 |
| calcb | 0.106509263 | 0.1005850665 | 1.058897376 | 0.2896465195 | 1.11238823 |
| ofd1 | -0.1128234468 | 0.1066135671 | -1.058246618 | 0.2899430231 | 0.8933083627 |
| rabep1 | -0.1109752642 | 0.1050112408 | -1.056794143 | 0.290605548 | 0.8949608862 |
| pag1 | 0.1042811912 | 0.09880388798 | 1.05543611 | 0.2912259153 | 1.109912509 |
| zfyve19 | -0.1086954092 | 0.103162932 | -1.053628537 | 0.2920530186 | 0.897003595 |
| rida | 0.1215409558 | 0.1156339493 | 1.05108367 | 0.2932201634 | 1.129235614 |
| mzt1 | -0.09559909637 | 0.09103436915 | -1.0501429 | 0.2936524176 | 0.9088282956 |
| tnfrsf13c | 0.1041701146 | 0.09924092833 | 1.049668885 | 0.2938703741 | 1.10978923 |
| mink1 | -0.1083088959 | 0.1031921123 | -1.049585027 | 0.2939089443 | 0.8973503658 |
| ufd1 | -0.1057405585 | 0.1007495427 | -1.049538843 | 0.2939301876 | 0.8996580264 |
| dpep2 | 0.09453281266 | 0.09011859605 | 1.048982306 | 0.2941862623 | 1.099145228 |
| khk | -0.1161624078 | 0.1110198129 | -1.046321416 | 0.2954126609 | 0.8903306149 |
| lrg1 | 0.1166809581 | 0.11152504 | 1.046231036 | 0.2954543769 | 1.123760846 |
| clip2 | -0.09602581227 | 0.09182046218 | -1.045799705 | 0.2956535174 | 0.9084405669 |
| eno2 | -0.09718629713 | 0.09302795581 | -1.044699911 | 0.2961616861 | 0.9073869468 |
| tmed8 | -0.1049420468 | 0.1004873917 | -1.044330488 | 0.2963325118 | 0.9003767008 |
| ism2 | 0.1129261422 | 0.1081326724 | 1.044329523 | 0.2963329581 | 1.119549242 |
| nap1l4 | 0.110148403 | 0.1056044403 | 1.04302814 | 0.29693526 | 1.116443742 |
| cryzl1 | -0.107717662 | 0.103358518 | -1.042174985 | 0.2973305583 | 0.8978810666 |
| rcor1 | -0.1014313415 | 0.09739762976 | -1.041414886 | 0.2976830368 | 0.9035432132 |
| pecam1 | -0.09831020863 | 0.09443798854 | -1.04100278 | 0.2978742579 | 0.9063676971 |
| got1 | -0.1034607884 | 0.09940605608 | -1.04078959 | 0.2979732124 | 0.9017113796 |
| lrrc38 | 0.1124711341 | 0.108150354 | 1.039951604 | 0.2983623859 | 1.119039954 |
| nrgn | -0.1033025728 | 0.09940701669 | -1.039187939 | 0.2987173384 | 0.9018540556 |
| rarres2 | 0.1079628198 | 0.1039677093 | 1.038426455 | 0.2990715586 | 1.114006326 |
| lcn2 | 0.1014510935 | 0.09770628582 | 1.038327193 | 0.2991177529 | 1.106775789 |
| sytl4 | -0.1060118616 | 0.1021302585 | -1.038006397 | 0.2992670769 | 0.8994139795 |
| tnfaip8l2 | -0.1059670246 | 0.1023935268 | -1.034899645 | 0.3007157804 | 0.8994543075 |
| entpd2 | -0.09328417978 | 0.09014954375 | -1.034771513 | 0.3007756296 | 0.9109345943 |
| vegfa | -0.1085408934 | 0.1050017013 | -1.033706046 | 0.3012736058 | 0.8971422068 |
| fcrl3 | -0.09616620329 | 0.09307378213 | -1.033225481 | 0.3014983911 | 0.9083130389 |
| znrf4 | -0.1125305164 | 0.1089299712 | -1.03305376 | 0.301578741 | 0.8935700782 |
| spink2 | 0.118766029 | 0.1150406169 | 1.032383451 | 0.3018925216 | 1.126106411 |
| thrap3 | -0.1122551011 | 0.1087648858 | -1.032089541 | 0.3020301731 | 0.8938162149 |
| s100a13 | 0.111014209 | 0.1076244978 | 1.031495721 | 0.3023084139 | 1.117410784 |
| atrn | 0.1063054383 | 0.1032338824 | 1.029753369 | 0.3031257955 | 1.112161521 |
| cd40 | 0.09682656478 | 0.09404621273 | 1.029563679 | 0.3032148727 | 1.101669289 |
| artn | -0.1010141223 | 0.09826594866 | -1.027966693 | 0.3039654952 | 0.9039202673 |
| apoa2 | 0.1062763093 | 0.1034756149 | 1.027066226 | 0.3043892806 | 1.112129126 |
| rab27b | -0.09950655091 | 0.09693683325 | -1.026509198 | 0.3046516305 | 0.9052840194 |
| lilra4 | 0.1101320757 | 0.10739195 | 1.025515187 | 0.3051201632 | 1.116425513 |
| naprt | -0.112987635 | 0.1103946671 | -1.023488163 | 0.3060770936 | 0.893161704 |
| tab2 | -0.1052924407 | 0.1029188513 | -1.023062727 | 0.3062781886 | 0.9000612696 |
| med18 | -0.09456269715 | 0.09245071307 | -1.022844433 | 0.3063814059 | 0.9097706928 |
| txk | -0.1075256246 | 0.1051393314 | -1.022696484 | 0.3064513741 | 0.8980535099 |
| calcoco1 | -0.09546505199 | 0.09336370011 | -1.022507162 | 0.3065409249 | 0.9089501271 |
| pcare | -0.1117987092 | 0.1093652047 | -1.022251177 | 0.3066620347 | 0.8942242385 |
| col1a1 | -0.1017002741 | 0.09965605984 | -1.020512694 | 0.3074853722 | 0.9033002536 |
| ldlrap1 | -0.103695038 | 0.1016330247 | -1.020288812 | 0.3075915078 | 0.9015001788 |
| fgf2 | -0.09529750805 | 0.09354686639 | -1.01871406 | 0.3083387357 | 0.9091024289 |
| sec31a | -0.1038431201 | 0.1019831438 | -1.018238077 | 0.3085648283 | 0.9013666926 |
| c1qtnf5 | -0.1083574367 | 0.1064291453 | -1.018118077 | 0.3086218454 | 0.8973068087 |
| ptprs | 0.09077703719 | 0.08929433545 | 1.016604656 | 0.3093415383 | 1.095024828 |
| snx15 | -0.1130905781 | 0.1113744107 | -1.015408992 | 0.3099109085 | 0.8930697639 |
| doc2b | -0.1085258175 | 0.1069435741 | -1.014795124 | 0.3102034987 | 0.8971557322 |
| exosc10 | -0.1076453705 | 0.1060997695 | -1.01456743 | 0.3103120714 | 0.8979459781 |
| diablo | -0.09269692066 | 0.09151306692 | -1.012936445 | 0.3110905191 | 0.9114697061 |
| pear1 | 0.09285988265 | 0.09174703482 | 1.012129524 | 0.3114761278 | 1.097307973 |
| crx | -0.09677729821 | 0.09576503216 | -1.01057031 | 0.3122221321 | 0.907758143 |
| mavs | -0.0945801603 | 0.09364416216 | -1.009995264 | 0.312497559 | 0.9097548055 |
| mcemp1 | -0.1040238656 | 0.1030558304 | -1.009393308 | 0.3127860466 | 0.9012037894 |
| lgals1 | 0.1016640711 | 0.1008364302 | 1.008207757 | 0.3133547347 | 1.107011532 |
| fadd | -0.0943972569 | 0.09380274384 | -1.006337907 | 0.3142530517 | 0.909921218 |
| serpinf2 | 0.1079076343 | 0.107232749 | 1.006293649 | 0.3142743348 | 1.11394485 |
| hmox2 | -0.1032237731 | 0.102663614 | -1.005456257 | 0.3146772026 | 0.9019251243 |
| commd1 | -0.1055382322 | 0.1050433662 | -1.004711064 | 0.315035999 | 0.8998400693 |

| Protein | beta | se | z | p_val | HR |
|---|---|---|---|---|---|
| ap1g2 | -0.1043494724 | 0.1039149586 | -1.004181436 | 0.3152911683 | 0.9009103991 |
| crkl | -0.09273873908 | 0.0924217298 | -1.00343003 | 0.3156534211 | 0.9114315906 |
| axin1 | -0.09238218862 | 0.09209675766 | -1.003099251 | 0.3158129761 | 0.9117566199 |
| clint1 | 0.1070460963 | 0.1067629953 | 1.002651677 | 0.3160289526 | 1.112985558 |
| rrp15 | 0.1072816856 | 0.1070605187 | 1.002065812 | 0.3163118084 | 1.113247796 |
| casp3 | -0.09137305815 | 0.09118721906 | -1.002037995 | 0.3163252427 | 0.9126771657 |
| plxdc2 | 0.1043839435 | 0.1041949575 | 1.001813772 | 0.3164335442 | 1.110026561 |
| ahcy | -0.09964362302 | 0.09950273138 | -1.001415958 | 0.3166257525 | 0.9051599387 |
| oga | -0.1037236335 | 0.1036170656 | -1.001028478 | 0.3168130408 | 0.9014744004 |
| tnfaip8 | -0.09070901992 | 0.09070177014 | -1.00007993 | 0.317271828 | 0.9132834195 |
| dscam | 0.1033337144 | 0.1035257397 | 0.9981451444 | 0.3182089819 | 1.10886139 |
| cacnb1 | 0.1038300852 | 0.1041636693 | 0.996797501 | 0.3188628115 | 1.109411933 |
| slc34a3 | -0.1096204286 | 0.1099831054 | -0.9967024319 | 0.3189089689 | 0.8961742329 |
| ebag9 | -0.09198521658 | 0.09232801848 | -0.9962871303 | 0.319110655 | 0.9121186337 |
| lap3 | -0.100073505 | 0.1005282885 | -0.9954760653 | 0.3195047795 | 0.9047709104 |
| tpsd1 | 0.1039223064 | 0.1044112994 | 0.9953166656 | 0.3195822748 | 1.109514249 |
| casp7 | -0.09929855153 | 0.1001174076 | -0.9918210416 | 0.3212848314 | 0.9054723375 |
| fcrl1 | -0.09474708873 | 0.09554505361 | -0.9916482868 | 0.3213691256 | 0.9096029542 |
| hsd17b3 | 0.1023848356 | 0.1033136082 | 0.9910101619 | 0.3216806183 | 1.107809714 |
| epgn | -0.1054652868 | 0.1064344162 | -0.9908945862 | 0.3217370562 | 0.8999057109 |
| eif4g3 | -0.09938296358 | 0.1003822832 | -0.990044861 | 0.3221521923 | 0.905395908 |
| calcoco2 | -0.1059083842 | 0.1070509336 | -0.9893270478 | 0.3225031546 | 0.8995070534 |
| clstn3 | 0.1056676827 | 0.106860769 | 0.988835133 | 0.3227438119 | 1.11145246 |
| st8sia1 | 0.1056083704 | 0.1068111043 | 0.9887396177 | 0.3227905539 | 1.111386539 |
| arhgef5 | 0.1046223651 | 0.1058621048 | 0.9882891075 | 0.3230110785 | 1.110291246 |
| atg16l1 | -0.1027376635 | 0.1039789083 | -0.9880625337 | 0.3231220234 | 0.9023636653 |
| enpp7 | -0.09899468646 | 0.1002314229 | -0.9876611908 | 0.3233186073 | 0.9057475207 |
| rtbdn | 0.09292238287 | 0.09423156944 | 0.9861067094 | 0.3240807516 | 1.097376557 |
| ap3b1 | -0.1023853251 | 0.1039053343 | -0.9853712115 | 0.3244417652 | 0.9026816587 |
| col2a1 | -0.1024949196 | 0.1040467865 | -0.9850849127 | 0.3245823636 | 0.9025827352 |
| col4a4 | 0.101582038 | 0.1031913864 | 0.9844042375 | 0.3249167954 | 1.106920724 |
| plekho1 | -0.09887138024 | 0.100457589 | -0.9842101647 | 0.325012189 | 0.9058592119 |
| cntnap2 | 0.09019931403 | 0.09165005466 | 0.98417087 | 0.325031506 | 1.09439239 |
| morf4l1 | 0.1088347228 | 0.1106057993 | 0.9839874898 | 0.325121664 | 1.114978055 |
| upb1 | 0.1100614265 | 0.1119533896 | 0.9831004393 | 0.3255580081 | 1.116346642 |
| fcgr2a | 0.09241904924 | 0.09402974544 | 0.9828703546 | 0.32567125 | 1.096824349 |
| mep1b | -0.09722520634 | 0.09892214918 | -0.9828456736 | 0.3256833989 | 0.9073516418 |
| c1qbp | -0.1003354796 | 0.1021618703 | -0.9821225796 | 0.3260394628 | 0.9045339145 |
| bst1 | 0.09084589855 | 0.09258261441 | 0.9812414472 | 0.3264736895 | 1.095100236 |
| rpl14 | 0.1150846841 | 0.1173280665 | 0.9808794056 | 0.3266522144 | 1.121968446 |
| cetn2 | -0.09474304526 | 0.09671585332 | -0.9796020198 | 0.3272826075 | 0.9096066322 |
| igfbp1 | -0.1006606379 | 0.1028164176 | -0.9790327289 | 0.3275638083 | 0.9042398455 |
| mvk | 0.09490661061 | 0.09699460815 | 0.9784730556 | 0.3278404113 | 1.099556163 |
| sod2 | -0.08484395834 | 0.08673451559 | -0.9782029422 | 0.3279739616 | 0.9186556218 |
| samd9l | -0.09406471576 | 0.09616901977 | -0.9781186913 | 0.3280156244 | 0.9102238545 |
| arsb | -0.09033006044 | 0.09252194696 | -0.9763095504 | 0.3289110886 | 0.9136295825 |
| luzp2 | 0.102102838 | 0.104593594 | 0.9761863432 | 0.3289721297 | 1.107497359 |
| ror1 | 0.09029220128 | 0.09259860697 | 0.9750924364 | 0.3295144101 | 1.09449405 |
| serpinb1 | -0.09136266122 | 0.09373115728 | -0.9747309632 | 0.3296937298 | 0.9126866548 |
| letm1 | 0.1034499538 | 0.1061343511 | 0.9747075539 | 0.3297053449 | 1.108990291 |
| prkg1 | -0.1002112526 | 0.1028757142 | -0.9741001883 | 0.3300067961 | 0.904646289 |
| enox2 | -0.1020873147 | 0.1049131544 | -0.9730649622 | 0.3305210166 | 0.9029507074 |
| gapdh | 0.1065627928 | 0.1096224256 | 0.9720893529 | 0.3310060985 | 1.112447778 |
| prok1 | 0.1243056008 | 0.1278960428 | 0.9719268718 | 0.3310869303 | 1.132361869 |
| gja8 | 0.1015447924 | 0.1044792583 | 0.9719134122 | 0.3310936268 | 1.106879497 |
| dbnl | -0.08864579196 | 0.09132316941 | -0.9706823858 | 0.3317064659 | 0.9151696766 |
| ppbp | -0.09971211455 | 0.1027361277 | -0.9705652411 | 0.3317648219 | 0.9050979451 |
| plxnb3 | -0.09345148723 | 0.09636631583 | -0.9697526197 | 0.3321698149 | 0.9107822009 |
| qdpr | -0.09581779414 | 0.09884677393 | -0.9693568169 | 0.3323671902 | 0.9086295586 |
| grik2 | -0.0987714751 | 0.102226845 | -0.966198997 | 0.3339446147 | 0.9059497164 |
| padi2 | 0.1000226887 | 0.1035717111 | 0.9657336707 | 0.3341774665 | 1.105195993 |
| gchfr | 0.1157297103 | 0.1198720782 | 0.9654434296 | 0.3343227577 | 1.122692379 |
| eef1d | -0.1013805224 | 0.1050165364 | -0.9653767486 | 0.3343561431 | 0.9035891315 |
| fcgr2b | -0.09367405996 | 0.09712366914 | -0.9644823017 | 0.3348041773 | 0.9105795082 |
| msln | 0.08800924339 | 0.09125614267 | 0.9644199373 | 0.3348354305 | 1.091998216 |
| rbpms2 | -0.09941402206 | 0.1030891852 | -0.9643496733 | 0.3348706447 | 0.9053677882 |
| amy2b | -0.09527241858 | 0.09882442236 | -0.9640574294 | 0.3350171341 | 0.9091252381 |
| ctsl | 0.0953648424 | 0.09895760782 | 0.9636938937 | 0.3351994166 | 1.10006013 |
| tarbp2 | -0.08958403057 | 0.09302303649 | -0.9630305992 | 0.3355321676 | 0.9143114318 |
| s100a16 | -0.09468885105 | 0.09834983126 | -0.962775938 | 0.3356599784 | 0.9096559289 |

| Protein | beta | se | z | p_val | HR |
|---|---|---|---|---|---|
| vim | -0.09357679877 | 0.09730326817 | -0.9617025258 | 0.3361990532 | 0.9106680765 |
| bcat2 | -0.1027388699 | 0.1068567935 | -0.9614631558 | 0.3363193424 | 0.9023625768 |
| sap18 | -0.1037244468 | 0.1078994106 | -0.9613068903 | 0.3363978845 | 0.9014736671 |
| bcl2 | -0.1019386714 | 0.1060612862 | -0.9611298812 | 0.3364868669 | 0.9030849349 |
| fen1 | 0.09291244368 | 0.09676322176 | 0.9602041146 | 0.3369524964 | 1.09736565 |
| lgals9 | 0.1054119059 | 0.1098013569 | 0.9600237084 | 0.3370432829 | 1.111168213 |
| nbn | -0.09362335115 | 0.09753493845 | -0.9598955271 | 0.3371077976 | 0.9106256838 |
| tshb | -0.09113186196 | 0.09502769854 | -0.9590031471 | 0.3375571598 | 0.9128973265 |
| grin2b | 0.09953323428 | 0.103809255 | 0.9588088684 | 0.3376550408 | 1.104655183 |
| gpr158 | 0.09951428806 | 0.1038160426 | 0.9585636822 | 0.3377785958 | 1.104634254 |
| lepr | 0.09260332087 | 0.09686326486 | 0.9560210571 | 0.3390615961 | 1.097026481 |
| anxa11 | -0.08884036257 | 0.09299535581 | -0.9553204221 | 0.339415683 | 0.9149916288 |
| spink6 | 0.09489441736 | 0.09940113037 | 0.9546613505 | 0.3397489811 | 1.099542756 |
| ezr | 0.09705410164 | 0.1016685917 | 0.9546124327 | 0.3397737276 | 1.101919988 |
| tigit | 0.09930673049 | 0.1040611247 | 0.9543115235 | 0.3399259767 | 1.104405002 |
| serpinb8 | 0.09412663049 | 0.09884183567 | 0.9522954512 | 0.3409471641 | 1.098698866 |
| nrn1 | -0.09704858335 | 0.1019467571 | -0.9519536092 | 0.3411205096 | 0.9075119151 |
| wasf3 | -0.08956961428 | 0.09409483969 | -0.9519078259 | 0.3411437303 | 0.9143246129 |
| vps53 | -0.08737917219 | 0.09184168354 | -0.951410828 | 0.3413958658 | 0.9163295831 |
| top2b | -0.09819075692 | 0.1032443862 | -0.951051777 | 0.3415780927 | 0.9064759707 |
| unc5d | -0.09674887068 | 0.1017739926 | -0.950624695 | 0.3417949279 | 0.9077839487 |
| lilrb1 | 0.09212147864 | 0.09695024432 | 0.9501933624 | 0.3420140107 | 1.096498015 |
| nfkbie | -0.08927017677 | 0.09395862495 | -0.9501009281 | 0.3420609717 | 0.9145984369 |
| ssc5d | 0.09866525371 | 0.103878184 | 0.9498168905 | 0.342205302 | 1.103696779 |
| mthfsd | -0.0952751263 | 0.1004084245 | -0.9488758218 | 0.3426837729 | 0.9091227765 |
| lpl | -0.1052843263 | 0.110959223 | -0.948856017 | 0.342693847 | 0.900068573 |
| sms | -0.100330125 | 0.1057462901 | -0.9487815115 | 0.342731747 | 0.9045387579 |
| bpifb1 | 0.08981938007 | 0.09472681405 | 0.9481938242 | 0.3430307906 | 1.093976672 |
| rnf41 | -0.091400753 | 0.09649707647 | -0.9471867579 | 0.3435436219 | 0.9126518896 |
| golga3 | -0.09822732469 | 0.1037588875 | -0.9466882991 | 0.3437976346 | 0.9064428235 |
| palm3 | -0.1012545 | 0.106973651 | -0.9465368251 | 0.3438748489 | 0.9037030112 |
| abca2 | 0.104453896 | 0.1103778542 | 0.946330192 | 0.3439801986 | 1.110104212 |
| fam172a | -0.0952745117 | 0.1006927653 | -0.9461902391 | 0.3440515638 | 0.9091233352 |
| ifnl2 | 0.1025186729 | 0.1085503542 | 0.9444342551 | 0.3449477836 | 1.10795799 |
| optc | -0.09353363754 | 0.09904027619 | -0.9444000072 | 0.3449652778 | 0.910707383 |
| anxa2 | 0.1033341046 | 0.109419733 | 0.9443827155 | 0.3449741108 | 1.108861823 |
| podxl2 | -0.0883798598 | 0.09376444813 | -0.9425732414 | 0.3458992321 | 0.915413082 |
| gpkow | 0.09073238315 | 0.09633297821 | 0.9418621207 | 0.3462632357 | 1.094975932 |
| itpr1 | -0.1005529803 | 0.1067770855 | -0.9417093548 | 0.3463414643 | 0.9043371991 |
| erp29 | -0.09674337105 | 0.102777415 | -0.9412901755 | 0.346556176 | 0.9077889412 |
| dclre1c | -0.09556676097 | 0.101585857 | -0.940748681 | 0.3468336654 | 0.9088576834 |
| tnfrsf11b | 0.09243864734 | 0.09831952278 | 0.9401860864 | 0.3471221174 | 1.096845845 |
| dhodh | 0.09952999835 | 0.1061444186 | 0.9376847101 | 0.3484064637 | 1.104651608 |
| tsnax | 0.1029953306 | 0.1098409371 | 0.9376770929 | 0.3484103794 | 1.108486233 |
| cdan1 | -0.09531042969 | 0.1016555296 | -0.937582344 | 0.3484590884 | 0.9090906819 |
| lmod2 | 0.1020352133 | 0.1089070538 | 0.9369017867 | 0.3488090801 | 1.107422467 |
| hla_a | 0.1038443082 | 0.1109797801 | 0.9357047571 | 0.3494252207 | 1.109427713 |
| csde1 | -0.09761052563 | 0.1045647729 | -0.9334934023 | 0.3505652754 | 0.907002089 |
| igf1r | 0.08339282911 | 0.08935481422 | 0.9332774046 | 0.3506767584 | 1.086968718 |
| arfip1 | -0.09609619262 | 0.1030739168 | -0.9323036866 | 0.351179603 | 0.9083766327 |
| leo1 | -0.09363542899 | 0.1004525542 | -0.9321358697 | 0.3512663127 | 0.9106146854 |
| tlr4 | -0.09820550066 | 0.1054137972 | -0.931619041 | 0.3515334392 | 0.906462606 |
| pdxdc1 | 0.09930295236 | 0.1066220921 | 0.9313543785 | 0.3516702816 | 1.10440083 |
| cep350 | 0.09684410678 | 0.1039957702 | 0.9312312091 | 0.3517339773 | 1.101688614 |
| evpl | 0.09901179676 | 0.1064544848 | 0.9300857261 | 0.3523267006 | 1.104079324 |
| tnfaip2 | -0.09620690088 | 0.1034939792 | -0.9295893504 | 0.3525837435 | 0.9082760735 |
| cd22 | -0.08929599438 | 0.09609463791 | -0.9292505422 | 0.3527592598 | 0.9145748245 |
| lhb | -0.09424242072 | 0.101446216 | -0.9289890191 | 0.3528947772 | 0.9100621176 |
| fdx2 | 0.1022207051 | 0.1102159677 | 0.9274582189 | 0.3536886751 | 1.107627904 |
| il12a_il12b | 0.08788495628 | 0.09480845558 | 0.9269738204 | 0.353940127 | 1.091862503 |
| btd | 0.09713194295 | 0.1047846295 | 0.9269674707 | 0.3539434239 | 1.102005766 |
| clpp | 0.08356805399 | 0.09023553052 | 0.9261102972 | 0.3543886632 | 1.087159198 |
| phldb1 | 0.09804061266 | 0.1058875159 | 0.9258939719 | 0.3545010843 | 1.10300758 |
| bap18 | -0.1027979066 | 0.1113567326 | -0.9231404719 | 0.3559340059 | 0.9023093058 |
| il7r | 0.09161484327 | 0.09947386693 | 0.9209940872 | 0.3570535141 | 1.095942631 |
| ceacam3 | -0.08591435946 | 0.09344985419 | -0.9193632265 | 0.3579056171 | 0.9176728178 |
| brme1 | -0.09894138662 | 0.1076248548 | -0.9193172601 | 0.3579296525 | 0.9057957982 |
| fgf21 | 0.09489397155 | 0.1032580397 | 0.9189983835 | 0.3580964177 | 1.099542266 |
| ccl27 | 0.09210616524 | 0.1002603723 | 0.918669691 | 0.3582683676 | 1.096481224 |
| rassf2 | 0.09217355329 | 0.1003676836 | 0.9183588776 | 0.3584310122 | 1.096555116 |

| Protein | beta | se | z | p_val | HR |
|---|---|---|---|---|---|
| ap3s2 | -0.09159239576 | 0.09996500749 | -0.9162445746 | 0.3595386311 | 0.9124770032 |
| qpct | 0.08688265794 | 0.0948511184 | 0.9159898102 | 0.3596722394 | 1.090768679 |
| gclm | -0.09801640997 | 0.1071317845 | -0.914914378 | 0.3602365813 | 0.9066340258 |
| p4hb | 0.09155480796 | 0.1001808148 | 0.9138956212 | 0.3607716948 | 1.095876838 |
| aldh1a1 | -0.08921033411 | 0.09771589898 | -0.9129561826 | 0.3612655872 | 0.9146531706 |
| txlna | -0.08633527699 | 0.09461227792 | -0.9125166298 | 0.3614968194 | 0.9172866346 |
| nfkb2 | -0.09887411926 | 0.1085135256 | -0.9111686193 | 0.3622065356 | 0.9058567308 |
| ank2 | 0.09921120646 | 0.1090229636 | 0.9100028395 | 0.3628210123 | 1.10429951 |
| spart | -0.09636196973 | 0.1059027266 | -0.9099101867 | 0.3628698771 | 0.9081352391 |
| nagpa | 0.09394268716 | 0.1033182965 | 0.9092550919 | 0.3632154896 | 1.098496786 |
| cd34 | -0.08718182181 | 0.09589267389 | -0.9091604006 | 0.3632654636 | 0.9165104389 |
| met | -0.08852502965 | 0.0974320517 | -0.9085822181 | 0.3635706964 | 0.9152802013 |
| spry2 | -0.083631422 | 0.0920981252 | -0.9080686694 | 0.3638419423 | 0.9197702007 |
| spint2 | 0.08723696254 | 0.09610456516 | 0.9077296422 | 0.3640210789 | 1.091155212 |
| rbp1 | 0.09390569445 | 0.1035771342 | 0.9066257252 | 0.3646047531 | 1.098456151 |
| ceacam5 | 0.08849954666 | 0.09769788875 | 0.9058491211 | 0.3650157173 | 1.092533757 |
| mphosph8 | -0.08656712492 | 0.09558636494 | -0.9056430273 | 0.3651248268 | 0.9170739882 |
| itpa | -0.09276261673 | 0.102491014 | -0.9050804854 | 0.3654227497 | 0.9114098281 |
| app | -0.08615438384 | 0.09524394322 | -0.9045654866 | 0.3656956266 | 0.9174525804 |
| snap23 | -0.08054414161 | 0.08914954164 | -0.903472302 | 0.3662752821 | 0.922614177 |
| amy2a | -0.08681895695 | 0.09610161074 | -0.9034079272 | 0.3663094343 | 0.9168430687 |
| aida | -0.09447065673 | 0.1046911153 | -0.9023751104 | 0.3668576382 | 0.9098544323 |
| comp | -0.08905200872 | 0.09869020177 | -0.9023389062 | 0.3668768641 | 0.9147979949 |
| ggact | -0.09188516596 | 0.1020511095 | -0.9003838019 | 0.3679160379 | 0.9122098963 |
| slc39a14 | 0.08709368811 | 0.09688283464 | 0.8989589171 | 0.3686745438 | 1.090998889 |
| rpa2 | -0.09410085708 | 0.1047108031 | -0.8986738167 | 0.3688264275 | 0.9101909584 |
| susd4 | 0.09581249197 | 0.1067040529 | 0.8979273925 | 0.3692242598 | 1.100552682 |
| hgfac | -0.09531870225 | 0.1062519465 | -0.8971007626 | 0.3696651519 | 0.9090831614 |
| akt3 | -0.08424700597 | 0.09394542946 | -0.8967653505 | 0.3698441408 | 0.9192041791 |
| spaca5_spaca5b | 0.09356120941 | 0.1044884195 | 0.8954218068 | 0.3705616478 | 1.098077814 |
| pdcd6 | 0.08434751529 | 0.09420224372 | 0.8953875403 | 0.3705799588 | 1.088006927 |
| mtss1 | -0.08802201791 | 0.09845588519 | -0.8940249508 | 0.3713085416 | 0.9157407138 |
| nit2 | -0.09622106841 | 0.1076554009 | -0.8937876562 | 0.3714355149 | 0.9082632056 |
| ubxn1 | -0.0973989866 | 0.1090422205 | -0.8932227002 | 0.371737924 | 0.9071939757 |
| s100a12 | 0.09083084946 | 0.1018661611 | 0.8916685238 | 0.3725706295 | 1.095083756 |
| mnat1 | -0.0929310095 | 0.1043591369 | -0.8904923157 | 0.3732015927 | 0.9112563662 |
| sv2a | 0.08584234561 | 0.09642838176 | 0.8902186684 | 0.3733484825 | 1.089634529 |
| camsap1 | -0.08800971037 | 0.09891836093 | -0.88972067 | 0.3736158923 | 0.9157519844 |
| pagr1 | -0.09753415554 | 0.1096341472 | -0.8896330021 | 0.3736629795 | 0.9070713595 |
| ing1 | -0.08344478787 | 0.09396708371 | -0.8880214706 | 0.3745292021 | 0.9199418772 |
| fcn1 | -0.0964813499 | 0.1087474664 | -0.8872054966 | 0.3749682737 | 0.9080268322 |
| nt5e | -0.08763926324 | 0.09879115012 | -0.8871165397 | 0.3750161602 | 0.9160912849 |
| ly75 | 0.0842529279 | 0.09503805019 | 0.8865178497 | 0.3753385399 | 1.08790402 |
| erbin | -0.08123258326 | 0.09181276956 | -0.8847634556 | 0.3762842232 | 0.9219792296 |
| nos3 | -0.09317770252 | 0.1054774634 | -0.883389679 | 0.3770257653 | 0.9110315933 |
| ndrg1 | -0.08791791638 | 0.09960363066 | -0.8826778281 | 0.3774103648 | 0.9158360488 |
| ddx25 | 0.09389878378 | 0.1064191079 | 0.8823489089 | 0.3775881553 | 1.098448559 |
| ldlr | -0.09086093565 | 0.1030417081 | -0.8817879417 | 0.3778914937 | 0.9131446879 |
| gstt2b | -0.08922552577 | 0.1012034046 | -0.8816454954 | 0.3779685442 | 0.9146392756 |
| cdk5rap3 | -0.08909752874 | 0.1011845418 | -0.8805448655 | 0.3785642115 | 0.9147563542 |
| cdh3 | 0.07964351867 | 0.09048531892 | 0.880181665 | 0.3787609044 | 1.082900965 |
| rbm25 | 0.09702079575 | 0.1102621091 | 0.8799105741 | 0.3789077559 | 1.101883288 |
| mpig6b | -0.08048908889 | 0.09149962404 | -0.8796657881 | 0.3790403881 | 0.9226649708 |
| dhps | -0.09477680838 | 0.1077914923 | -0.8792605643 | 0.3792600128 | 0.9095759215 |
| spred2 | -0.08803806594 | 0.100133263 | -0.8792089993 | 0.3792879658 | 0.9157260181 |
| eif2s2 | 0.0925121347 | 0.1052893636 | 0.8786465369 | 0.3795929549 | 1.096926452 |
| cfd | 0.09644983438 | 0.1098496643 | 0.8780166512 | 0.3799346825 | 1.101254335 |
| trim21 | -0.08533155049 | 0.09735509617 | -0.876498035 | 0.3807593442 | 0.9182078017 |
| cd177 | 0.07978917929 | 0.09110710844 | 0.875773369 | 0.3811532503 | 1.083058712 |
| tnfrsf17 | 0.09091432202 | 0.1038414431 | 0.875510965 | 0.3812959467 | 1.095175169 |
| adgre5 | 0.08406038429 | 0.09611704824 | 0.8745626903 | 0.3818118958 | 1.087694571 |
| edf1 | -0.09013061069 | 0.103297473 | -0.8725345171 | 0.3829168463 | 0.9138118239 |
| psmg4 | 0.09641291949 | 0.110615464 | 0.871604349 | 0.3834242573 | 1.101213683 |
| sirt2 | -0.08305427848 | 0.09532243672 | -0.8712983148 | 0.3835912904 | 0.9203011933 |
| bag6 | -0.08189152341 | 0.0940659874 | -0.8705752809 | 0.3839860983 | 0.9213719006 |
| cd99 | 0.09544752235 | 0.1096600682 | 0.8703945191 | 0.3840848409 | 1.100151087 |
| timm8a | -0.08696744481 | 0.09993051446 | -0.8702791663 | 0.3841478615 | 0.9167069387 |
| gucy2c | 0.09463626389 | 0.1089370252 | 0.8687245101 | 0.3849978319 | 1.099258942 |
| gsn | -0.09209509226 | 0.1061248764 | -0.8677992892 | 0.3855042192 | 0.9120184195 |
| kif22 | 0.08847184122 | 0.1020764019 | 0.866721785 | 0.3860944661 | 1.092503489 |

| Protein | beta | se | z | p_val | HR |
|---|---|---|---|---|---|
| ssbp1 | 0.09265422618 | 0.1069401699 | 0.8664118105 | 0.3862643695 | 1.097082327 |
| cit | 0.0959681876 | 0.1107690627 | 0.866380786 | 0.3862813771 | 1.100724047 |
| cxcl11 | -0.08574789884 | 0.09905749844 | -0.8656376366 | 0.3866889093 | 0.917825587 |
| inppl1 | -0.07961048612 | 0.0919829259 | -0.8654919958 | 0.3867688073 | 0.9234759831 |
| nfx1 | -0.08887272553 | 0.1029805 | -0.86300538 | 0.388134509 | 0.9149620175 |
| satb1 | 0.09095528436 | 0.1055994043 | 0.8613238396 | 0.3890597092 | 1.095220031 |
| ccl17 | -0.08105917989 | 0.09421981311 | -0.8603198968 | 0.389612728 | 0.9221391177 |
| fnta | -0.09429435905 | 0.109882354 | -0.8581392335 | 0.3908155855 | 0.9100148517 |
| nck2 | -0.07851129208 | 0.09160056344 | -0.8571049034 | 0.3913869115 | 0.9244916204 |
| hsbp1 | 0.09440311393 | 0.1101576997 | 0.8569815293 | 0.3914550927 | 1.09900268 |
| snx5 | -0.09024007839 | 0.1054114434 | -0.8560747821 | 0.3919564165 | 0.9137117965 |
| rad23b | -0.07975034311 | 0.09319559603 | -0.8557308124 | 0.3921466927 | 0.9233468375 |
| klk15 | 0.09108989569 | 0.1065487287 | 0.8549130222 | 0.3925993009 | 1.09536747 |
| azu1 | 0.08557739989 | 0.100125921 | 0.8546977552 | 0.3927184936 | 1.089345873 |
| saa4 | -0.08805156789 | 0.1032370607 | -0.8529065757 | 0.3937111148 | 0.9157136541 |
| islr2 | 0.08017353409 | 0.09405152549 | 0.8524426762 | 0.3939684423 | 1.083475071 |
| rab10 | -0.08534931718 | 0.1001447467 | -0.8522595543 | 0.3940700491 | 0.9181914883 |
| cert | -0.07935863905 | 0.09314690544 | -0.8519728989 | 0.394229134 | 0.9237085871 |
| akap12 | -0.09130318639 | 0.1071977769 | -0.8517264912 | 0.3943659138 | 0.9127409383 |
| map2 | -0.08803383832 | 0.103505782 | -0.8505209723 | 0.3950355055 | 0.9157298894 |
| terf1 | -0.08866594459 | 0.104374611 | -0.8494972457 | 0.3956046622 | 0.9151512337 |
| tpd52l2 | -0.08814278682 | 0.1038158399 | -0.849030234 | 0.395864469 | 0.9156301275 |
| ddx1 | -0.08947835741 | 0.1054577055 | -0.8484762396 | 0.3961727995 | 0.9144080551 |
| tlr3 | -0.07629216925 | 0.09012998521 | -0.8464682322 | 0.3972915885 | 0.9265454589 |
| ccl8 | -0.08090527927 | 0.095707854 | -0.8453358412 | 0.3979233552 | 0.9222810464 |
| arhgef12 | -0.07945800573 | 0.09415151918 | -0.8439375851 | 0.3987042841 | 0.9236168058 |
| lbr | 0.08399641667 | 0.09962558303 | 0.8431209546 | 0.3991608005 | 1.087624996 |
| il13 | 0.07969790753 | 0.09456366662 | 0.8427962913 | 0.3993423825 | 1.082959864 |
| cd244 | 0.07603516748 | 0.09022712785 | 0.842708499 | 0.3993914928 | 1.079000519 |
| fgfbp2 | -0.08761900424 | 0.104100702 | -0.8416754407 | 0.3999696488 | 0.9161098442 |
| preb | 0.08521301318 | 0.1012711045 | 0.8414346189 | 0.4001044982 | 1.088949002 |
| plxna4 | -0.07670730516 | 0.09132101399 | -0.839974304 | 0.4009227941 | 0.9261608964 |
| klf4 | 0.08711734594 | 0.103787643 | 0.8393807142 | 0.4012557027 | 1.0910247 |
| cntn5 | -0.0836787992 | 0.09977239675 | -0.8386968934 | 0.4016394221 | 0.9197266256 |
| pspn | 0.106133849 | 0.1266586587 | 0.8379517838 | 0.4020577837 | 1.111970703 |
| cc2d1a | -0.0843671106 | 0.1009384422 | -0.8358273492 | 0.4032520383 | 0.9190937851 |
| f13b | -0.08932485159 | 0.1068826087 | -0.835728587 | 0.4033076092 | 0.9145484328 |
| pcsk7 | -0.08883518217 | 0.1065233576 | -0.8339502636 | 0.4043090114 | 0.9149963689 |
| slc9a3r1 | -0.08576624526 | 0.1028437839 | -0.8339468077 | 0.4043109589 | 0.9178087483 |
| cadps | 0.08637688359 | 0.1036401905 | 0.833430382 | 0.4046020458 | 1.090217136 |
| grap2 | -0.07653360118 | 0.0919054367 | -0.8327429141 | 0.4049897362 | 0.9263217883 |
| akt2 | -0.08402904154 | 0.1009414029 | -0.8324536724 | 0.4051529174 | 0.9194045548 |
| c9orf40 | 0.09012206712 | 0.1086534736 | 0.8294448774 | 0.4068527162 | 1.094307855 |
| pfdn2 | -0.07718030035 | 0.09317312393 | -0.8283536828 | 0.4074702291 | 0.9257229304 |
| mbl2 | -0.08228343507 | 0.09936173597 | -0.8281199424 | 0.4076025766 | 0.9210108749 |
| metap1 | -0.0787134829 | 0.09511361662 | -0.8275732298 | 0.4079122342 | 0.9243047156 |
| lactb2 | -0.07863857069 | 0.0951662922 | -0.8263279873 | 0.4086180614 | 0.9243739599 |
| fkbp4 | -0.08073194736 | 0.09770146925 | -0.826312521 | 0.4086268326 | 0.922440921 |
| dkk3 | 0.07952471567 | 0.09626430052 | 0.8261080717 | 0.4087427893 | 1.082772321 |
| ids | -0.08171781409 | 0.09892256629 | -0.8260785901 | 0.4087595119 | 0.9215319653 |
| c1s | -0.08735797308 | 0.1061012975 | -0.8233450028 | 0.4103118287 | 0.9163490086 |
| akr1c4 | 0.07830234525 | 0.09516862581 | 0.8227747808 | 0.4106360806 | 1.08144958 |
| icoslg | 0.07673360949 | 0.09350094051 | 0.8206720603 | 0.4118330895 | 1.079754402 |
| cacnb3 | -0.08306660617 | 0.1014076566 | -0.8191354477 | 0.4127091395 | 0.9202898482 |
| tspan15 | 0.08876461823 | 0.1085810764 | 0.8174962081 | 0.4136449154 | 1.092823395 |
| cd55 | 0.08139670427 | 0.09963415416 | 0.8169558417 | 0.4139536638 | 1.084801156 |
| ptrhd1 | -0.08680720883 | 0.1063687412 | -0.8160969835 | 0.4144446691 | 0.9168538399 |
| mocs2 | -0.08645473856 | 0.1059486237 | -0.8160062448 | 0.4144965641 | 0.9171770606 |
| vamp8 | -0.08265577422 | 0.1013078866 | -0.8158868673 | 0.4145648439 | 0.9206680103 |
| smad3 | -0.08421113248 | 0.1032985652 | -0.8152207374 | 0.4149459697 | 0.9192371548 |
| chit1 | 0.07778394347 | 0.09543145084 | 0.8150766103 | 0.4150284591 | 1.0808891 |
| prl | 0.07429017862 | 0.09134074341 | 0.813330129 | 0.4160288073 | 1.077119317 |
| lair2 | 0.07494569578 | 0.09228738765 | 0.8120903375 | 0.4167397972 | 1.077825619 |
| gorasp2 | -0.08143104445 | 0.1002926811 | -0.8119340668 | 0.4168294654 | 0.9217962706 |
| hs1bp3 | -0.08429188359 | 0.1038375414 | -0.8117669435 | 0.4169253734 | 0.9191629284 |
| psmg3 | 0.081058437 | 0.09995845356 | 0.8109212789 | 0.4174108795 | 1.084434266 |
| dgka | -0.08269894445 | 0.1020492189 | -0.8103829243 | 0.4177201288 | 0.9206282658 |
| rnaseh2a | -0.08605451888 | 0.1064282354 | -0.8085685018 | 0.4187633887 | 0.9175442064 |
| ppp1r9b | -0.07305017569 | 0.09050115441 | -0.8071739655 | 0.4195662629 | 0.9295541878 |
| man1a2 | 0.08919578103 | 0.1105114835 | 0.80711776 | 0.4195986409 | 1.093294682 |

| Protein | beta | se | z | p_val | HR |
|---|---|---|---|---|---|
| atxn2l | -0.08526993918 | 0.1056981989 | -0.8067302951 | 0.4198218861 | 0.9182643754 |
| acox1 | -0.07820149759 | 0.09693648013 | -0.8067292878 | 0.4198224665 | 0.9247780672 |
| mesd | -0.07336132714 | 0.0909736076 | -0.8064023081 | 0.4200109165 | 0.9292650007 |
| ctsd | 0.09046491287 | 0.1122209445 | 0.8061321646 | 0.4201666472 | 1.094683098 |
| cmc1 | -0.08075087824 | 0.1002853688 | -0.8052109612 | 0.4206979522 | 0.9224234586 |
| dll4 | 0.08722250573 | 0.1083677243 | 0.80487531 | 0.4208916374 | 1.091139437 |
| smpdl3a | -0.07341883348 | 0.09142339463 | -0.8030639616 | 0.4219377662 | 0.9292115636 |
| nfya | 0.08721407189 | 0.1086065718 | 0.8030275742 | 0.4219587971 | 1.091130235 |
| serping1 | -0.0870226231 | 0.1085548573 | -0.8016465155 | 0.4227574624 | 0.9166563578 |
| alcam | 0.07498163355 | 0.09354489322 | 0.8015577438 | 0.4228088292 | 1.077864354 |
| bag3 | -0.0782469539 | 0.09762462453 | -0.8015083722 | 0.4228373992 | 0.9247360312 |
| kcnh2 | -0.08191164271 | 0.1023912237 | -0.7999869495 | 0.4237183585 | 0.9213533634 |
| igf2bp3 | -0.08808703535 | 0.1101266363 | -0.7998703881 | 0.423785896 | 0.9156811766 |
| cd99l2 | -0.07758980313 | 0.09704957355 | -0.7994862862 | 0.4240084954 | 0.9253439219 |
| psca | -0.08117704532 | 0.1015382462 | -0.799472596 | 0.4240164306 | 0.9220304358 |
| fcrl6 | 0.07472082263 | 0.09360944219 | 0.7982188642 | 0.4247434922 | 1.077583272 |
| prnd | 0.08402294047 | 0.1053427632 | 0.797614738 | 0.4250940957 | 1.087653845 |
| glt8d2 | 0.07821615813 | 0.09806518131 | 0.7975935708 | 0.4251063832 | 1.081356377 |
| dda1 | -0.08662886904 | 0.1087529016 | -0.7965660482 | 0.4257031023 | 0.917017366 |
| pacs2 | -0.08319507056 | 0.1045429895 | -0.7957976996 | 0.4261496291 | 0.9201716313 |
| slit2 | -0.07614192969 | 0.0957697617 | -0.7950518863 | 0.4265833206 | 0.9266846732 |
| brk1 | 0.07352369422 | 0.09251748583 | 0.7947005213 | 0.4267877291 | 1.076294038 |
| smad2 | -0.08213863203 | 0.1034189259 | -0.794232113 | 0.4270603168 | 0.9211442497 |
| cntn3 | 0.08281878371 | 0.1043359499 | 0.7937703526 | 0.4273291351 | 1.086344927 |
| relb | -0.08329842795 | 0.104992964 | -0.7933715246 | 0.4275613961 | 0.9200765297 |
| egflam | 0.08350019608 | 0.1054484867 | 0.7918576995 | 0.4284436541 | 1.087085428 |
| cpe | -0.07591407058 | 0.09599087194 | -0.7908467654 | 0.4290334163 | 0.9268958508 |
| ptpn6 | -0.07168478517 | 0.09080422876 | -0.7894432468 | 0.4298529879 | 0.9308242592 |
| cdhr5 | -0.07110243895 | 0.09013348356 | -0.7888571055 | 0.4301955286 | 0.931366479 |
| jmjd1c | 0.0825534592 | 0.1046523345 | 0.7888353339 | 0.430208255 | 1.086056732 |
| gage2a | -0.08375707687 | 0.1062165036 | -0.7885504987 | 0.4303747726 | 0.9196546343 |
| sftpa1 | 0.07269342075 | 0.09223647829 | 0.788120081 | 0.4306264702 | 1.075400791 |
| mamdc4 | 0.08955771338 | 0.113677912 | 0.787819831 | 0.4308020996 | 1.093690452 |
| lat2 | -0.07230682391 | 0.09183134654 | -0.7873871683 | 0.431055256 | 0.9302454305 |
| uxs1 | -0.07403607048 | 0.09405976886 | -0.7871172912 | 0.4312132081 | 0.9286381968 |
| cd248 | -0.07963830759 | 0.1012501074 | -0.7865503522 | 0.4315451324 | 0.9234502909 |
| il6 | -0.08683946374 | 0.1104816036 | -0.7860083574 | 0.431862591 | 0.9168242674 |
| vegfb | -0.08583313808 | 0.1097147484 | -0.7823299907 | 0.4340206659 | 0.9177473555 |
| oplah | -0.08330426882 | 0.1066657222 | -0.7809844355 | 0.434811649 | 0.9200711557 |
| dctpp1 | -0.07443809795 | 0.09535964369 | -0.7806037761 | 0.4350355702 | 0.9282649338 |
| nadk | 0.07936281983 | 0.10167638 | 0.780543326 | 0.4350711358 | 1.082597039 |
| fkbp5 | -0.07447924204 | 0.09542975167 | -0.7804614467 | 0.4351193119 | 0.9282267419 |
| magea3 | 0.08276667562 | 0.1063887833 | 0.7779643029 | 0.4365900579 | 1.086288321 |
| nudt15 | -0.08221411909 | 0.1057221443 | -0.7776433179 | 0.4367793164 | 0.9210747179 |
| f2r | 0.07262591668 | 0.09341326253 | 0.7774690094 | 0.4368821116 | 1.075328199 |
| atp6v1f | -0.07602266925 | 0.09785389756 | -0.7768997571 | 0.4372179146 | 0.9267951966 |
| snap29 | -0.07061536613 | 0.09132259201 | -0.7732518819 | 0.4393733269 | 0.9318202328 |
| cxcl17 | 0.08461684709 | 0.109501643 | 0.7727449999 | 0.4396733092 | 1.088300001 |
| mmp8 | 0.07295368889 | 0.09447390916 | 0.77220991 | 0.439990113 | 1.07568072 |
| bach1 | -0.07182498616 | 0.09314589963 | -0.7711019642 | 0.4406464965 | 0.9306937659 |
| elac1 | -0.07848507001 | 0.1018316628 | -0.7707334621 | 0.4408649336 | 0.9245158628 |
| max | -0.06909857023 | 0.08968714988 | -0.7704400276 | 0.4410389171 | 0.9332346864 |
| psme1 | -0.07804966612 | 0.1013663872 | -0.7699758106 | 0.4413142417 | 0.9249184883 |
| il31 | 0.08294557319 | 0.1077286379 | 0.7699491499 | 0.4413300571 | 1.086482673 |
| dsg3 | -0.07491891657 | 0.09738573773 | -0.7693007037 | 0.4417148196 | 0.927818714 |
| ssb | 0.07415191297 | 0.09648581702 | 0.7685265593 | 0.442174418 | 1.076970399 |
| pomc | -0.08001208417 | 0.1041270286 | -0.7684084069 | 0.4422445874 | 0.9231051914 |
| lipf | 0.07883759817 | 0.1026699567 | 0.7678740766 | 0.4425619999 | 1.082028584 |
| scly | -0.07689918565 | 0.1002616065 | -0.7669853733 | 0.443090212 | 0.9259832013 |
| icam3 | 0.06921404453 | 0.0907002573 | 0.7631074771 | 0.4453993012 | 1.071665569 |
| smad1 | -0.06936669486 | 0.09103645716 | -0.761966107 | 0.446080233 | 0.9329844967 |
| matn2 | 0.0727163226 | 0.09551273517 | 0.761325937 | 0.4464624123 | 1.07542542 |
| hexim1 | -0.0712254718 | 0.09374383038 | -0.759788367 | 0.4473810976 | 0.9312518974 |
| bid | -0.07553221821 | 0.09946141644 | -0.7594122517 | 0.4476059869 | 0.9272498557 |
| hcls1 | -0.07360003523 | 0.09695521515 | -0.7591137322 | 0.4477845252 | 0.9290432041 |
| acp5 | 0.07488469053 | 0.09877285272 | 0.7581505288 | 0.4483608734 | 1.077759868 |
| myom1 | 0.07990455708 | 0.1057288009 | 0.7557501497 | 0.4497990101 | 1.083183681 |
| cd58 | -0.07121468958 | 0.09427343894 | -0.7554056623 | 0.4500056168 | 0.9312619384 |
| wasf1 | -0.07027965583 | 0.09310760083 | -0.7548218964 | 0.4503558539 | 0.932133107 |
| glo1 | 0.07422662939 | 0.09839245699 | 0.7543934938 | 0.4506129772 | 1.077050869 |

| Protein | beta | se | z | p_val | HR |
|---|---|---|---|---|---|
| hnmt | -0.07570333292 | 0.1004336576 | -0.753764572 | 0.4509906008 | 0.9270912032 |
| nt5c | -0.07749686543 | 0.1029385652 | -0.75284579 | 0.451542587 | 0.9254299252 |
| cdkn2d | -0.06883648269 | 0.09158897301 | -0.7515804624 | 0.4523033963 | 0.9334793076 |
| mapre3 | -0.08035217559 | 0.1069414676 | -0.7513659334 | 0.4524324589 | 0.9227913046 |
| yju2 | -0.0776595573 | 0.1033771647 | -0.7512254519 | 0.4525169851 | 0.9252793775 |
| ppcdc | -0.07198437329 | 0.09585189736 | -0.7509958099 | 0.4526551775 | 0.9305454371 |
| manf | -0.06974244057 | 0.09315651185 | -0.7486587806 | 0.4540628936 | 0.9326339977 |
| dpy30 | 0.07574553335 | 0.1012391566 | 0.7481841603 | 0.4543490838 | 1.078688049 |
| cnp | -0.07838067288 | 0.1048534699 | -0.7475257896 | 0.4547462414 | 0.9246123847 |
| il36a | 0.07800455594 | 0.1045629105 | 0.7460059744 | 0.455663806 | 1.081127584 |
| c2orf69 | -0.07758671859 | 0.1041228797 | -0.7451457243 | 0.4561836299 | 0.9253467762 |
| epha4 | 0.08049294773 | 0.1080775279 | 0.7447704375 | 0.4564105092 | 1.083821203 |
| prr4 | -0.07802435107 | 0.1048290076 | -0.7443011517 | 0.4566943047 | 0.9249419029 |
| rnf31 | 0.07691359868 | 0.1033389139 | 0.7442849527 | 0.4567041026 | 1.079948763 |
| npdc1 | 0.06762545218 | 0.09086027059 | 0.7442796697 | 0.456707298 | 1.069964481 |
| cspg5 | -0.07869127039 | 0.1057447294 | -0.7441625773 | 0.456778125 | 0.924325247 |
| hras | 0.08040494913 | 0.1080649513 | 0.7440428014 | 0.4568505815 | 1.083725833 |
| ube2b | -0.07636706565 | 0.1027676236 | -0.7431043258 | 0.4574185209 | 0.9264760666 |
| fam13a | -0.07784861367 | 0.1047866651 | -0.7429248138 | 0.4575272017 | 0.9251044641 |
| uros | 0.08075773664 | 0.1087318406 | 0.7427238995 | 0.4576488572 | 1.084108225 |
| mrpl52 | 0.08090367506 | 0.1090042074 | 0.7422069019 | 0.4579619875 | 1.08426645 |
| stx1b | 0.07916669165 | 0.1072111461 | 0.7384184807 | 0.4602601877 | 1.082384732 |
| ccl22 | -0.07144627356 | 0.09675734227 | -0.7384067389 | 0.4602673208 | 0.9310462981 |
| podxl | -0.07038245779 | 0.0953210149 | -0.7383729376 | 0.4602878551 | 0.9320372868 |
| il22 | -0.08351288014 | 0.1132309227 | -0.7375448166 | 0.4607910996 | 0.9198792384 |
| upk3a | -0.07704817591 | 0.1044950537 | -0.7373380194 | 0.4609168171 | 0.9258452491 |
| pbk | 0.0779283271 | 0.1058010605 | 0.7365552548 | 0.4613928541 | 1.081045174 |
| ica1 | -0.07045667943 | 0.09566961039 | -0.7364583084 | 0.461451831 | 0.931968112 |
| ptp4a3 | -0.08412338383 | 0.1142683623 | -0.7361913843 | 0.4616142348 | 0.9193178201 |
| il17ra | 0.06769324572 | 0.09195632181 | 0.7361456438 | 0.4616420678 | 1.07003702 |
| pla2g1b | -0.06541808917 | 0.08893104313 | -0.7356046536 | 0.4619713297 | 0.9366757675 |
| decr1 | -0.06767429928 | 0.09204415079 | -0.7352373693 | 0.4621949441 | 0.9345648124 |
| pam | 0.06717345953 | 0.09142552358 | 0.7347342066 | 0.4625013835 | 1.069480974 |
| ppie | -0.07559734493 | 0.1029000058 | -0.7346680339 | 0.4625416928 | 0.9271894689 |
| hhex | -0.07776410713 | 0.105956286 | -0.7339263205 | 0.4629936446 | 0.9251826448 |
| cd93 | 0.06848891248 | 0.09332056411 | 0.7339101851 | 0.4630034791 | 1.070888751 |
| rabgap1l | -0.07119082711 | 0.09706311123 | -0.733448848 | 0.4632847154 | 0.9312841609 |
| ccer2 | 0.07840230743 | 0.1070079779 | 0.7326772171 | 0.4637553228 | 1.08155769 |
| cdh6 | 0.06515509954 | 0.0889371049 | 0.7325974869 | 0.4638039643 | 1.067324553 |
| tnfsf14 | -0.07556704305 | 0.1032962137 | -0.7315567562 | 0.4644391507 | 0.927217565 |
| ceacam1 | -0.06309726305 | 0.08641586373 | -0.7301583335 | 0.4652934081 | 0.9388521536 |
| dbh | -0.08173731192 | 0.1119720753 | -0.7299794322 | 0.4654027569 | 0.9215139976 |
| fkbp1b | -0.06720113077 | 0.09206290619 | -0.7299479623 | 0.4654219935 | 0.9350071237 |
| fut1 | -0.07542927476 | 0.1033364907 | -0.7299384202 | 0.4654278264 | 0.9273453149 |
| acyp1 | 0.07757478906 | 0.1063364454 | 0.72952212 | 0.465682342 | 1.080663051 |
| gnpda1 | 0.07981667093 | 0.1098449701 | 0.7266301845 | 0.4674525323 | 1.083088488 |
| srp14 | 0.07179573924 | 0.09883155374 | 0.7264455179 | 0.4675656956 | 1.074435857 |
| gipr | -0.07878505333 | 0.1084663118 | -0.7263550498 | 0.4676211398 | 0.9242385651 |
| nedd9 | 0.07619916389 | 0.1050154881 | 0.7255992925 | 0.4680844549 | 1.079177486 |
| klrk1 | 0.07665337999 | 0.1057066405 | 0.7251519832 | 0.4683587966 | 1.079667777 |
| slc16a1 | 0.07138929754 | 0.09859081329 | 0.724096852 | 0.4690062771 | 1.07399925 |
| fabp6 | 0.06628689736 | 0.09156046521 | 0.7239685513 | 0.4690850425 | 1.068533233 |
| atp1b1 | -0.07483985602 | 0.1033754926 | -0.7239613001 | 0.4690894943 | 0.9278920708 |
| dnajc6 | -0.07747953665 | 0.10704266 | -0.7238192385 | 0.4691767167 | 0.9254459619 |
| stc2 | 0.07111256548 | 0.09830689252 | 0.7233731396 | 0.4694506689 | 1.073702081 |
| arhgef10 | -0.07316033015 | 0.1011450272 | -0.7233210788 | 0.4694826455 | 0.9294517989 |
| hars1 | -0.06539546644 | 0.09043285566 | -0.7231383546 | 0.4695948874 | 0.9366969579 |
| neo1 | 0.07489725363 | 0.1036059518 | 0.7229049328 | 0.4697382928 | 1.077773408 |
| tnfsf13b | 0.06906097232 | 0.0955721396 | 0.7226056946 | 0.4699221686 | 1.071501539 |
| pibf1 | -0.07277413787 | 0.1008320588 | -0.7217361101 | 0.4704567365 | 0.9298108154 |
| ifnl1 | -0.07013652535 | 0.09721099417 | -0.7214875842 | 0.4706095769 | 0.9322665332 |
| odam | 0.0655292959 | 0.09084453268 | 0.7213345038 | 0.470703733 | 1.067724017 |
| ercc1 | 0.08018462936 | 0.1112540193 | 0.7207346744 | 0.4710727744 | 1.083487093 |
| mad1l1 | 0.07731580693 | 0.1073357989 | 0.7203170585 | 0.4713298041 | 1.080383215 |
| gpr101 | -0.07368755923 | 0.1024675976 | -0.719130349 | 0.4720606095 | 0.9289618941 |
| prtg | 0.0662014847 | 0.09208710795 | 0.7189006819 | 0.4722021163 | 1.06844197 |
| galnt2 | -0.06669861563 | 0.09280965005 | -0.7186603504 | 0.4723502189 | 0.935477097 |
| gopc | -0.06659098446 | 0.09301436149 | -0.715921535 | 0.4740398003 | 0.9355777889 |
| vta1 | -0.06818520723 | 0.09530416172 | -0.7154483708 | 0.4743320325 | 0.9340874578 |
| mif | -0.06692051976 | 0.09357695561 | -0.7151388856 | 0.474523228 | 0.9352695338 |

| Protein | beta | se | z | p_val | HR |
|---|---|---|---|---|---|
| gzmh | -0.07304940042 | 0.1022496886 | -0.7144217399 | 0.4749664329 | 0.9295549085 |
| rnase10 | 0.07681343433 | 0.107557477 | 0.7141617345 | 0.4751271756 | 1.079840596 |
| itgb1bp2 | -0.06538765977 | 0.09176248267 | -0.7125750946 | 0.4761087277 | 0.9367042704 |
| klb | 0.07111027368 | 0.09988121031 | 0.7119484581 | 0.4764966934 | 1.07369962 |
| tk1 | -0.07709191548 | 0.1082914914 | -0.7118926377 | 0.4765312615 | 0.9258047539 |
| dab2 | -0.06617519511 | 0.09302340947 | -0.7113821723 | 0.4768474434 | 0.9359668731 |
| fga | 0.0789941794 | 0.1111425833 | 0.7107462957 | 0.4772414655 | 1.082198023 |
| brdt | -0.07246947385 | 0.1020728293 | -0.7099781045 | 0.4777177141 | 0.9300941384 |
| prss22 | 0.0716599901 | 0.101340428 | 0.7071214471 | 0.4794910089 | 1.074290013 |
| fis1 | -0.06651059132 | 0.0940950542 | -0.7068447102 | 0.4796629864 | 0.935653006 |
| retn | 0.06618907201 | 0.09365826545 | 0.7067082835 | 0.4797477808 | 1.068428708 |
| cpa4 | -0.07108277564 | 0.1006200601 | -0.7064473584 | 0.4799099785 | 0.931384793 |
| c8b | -0.0726304181 | 0.1028562371 | -0.7061352831 | 0.4801040117 | 0.9299444572 |
| gla | 0.08556437393 | 0.1214322985 | 0.7046261578 | 0.4810429156 | 1.089331684 |
| adam8 | -0.06685824128 | 0.09490858523 | -0.7044488243 | 0.4811533094 | 0.9353277828 |
| atf2 | -0.06777073652 | 0.09629135001 | -0.7038091845 | 0.4815516132 | 0.9344746899 |
| jpt2 | -0.07313863656 | 0.1040367984 | -0.703007375 | 0.4820511535 | 0.9294719623 |
| gtf2ird1 | 0.07593197435 | 0.108051465 | 0.7027389618 | 0.4822184421 | 1.07888918 |
| wfikkn1 | -0.06885373324 | 0.09810266879 | -0.7018538241 | 0.4827703282 | 0.9334632047 |
| smarca2 | -0.06573922538 | 0.0938734165 | -0.7002965038 | 0.4837421547 | 0.9363750153 |
| prkra | -0.06738835625 | 0.0962722442 | -0.6999769955 | 0.483941671 | 0.9348320829 |
| bloc1s2 | -0.07136878932 | 0.1020957039 | -0.699038124 | 0.4845282058 | 0.9311184423 |
| s100a3 | -0.07441100802 | 0.1064871702 | -0.6987790911 | 0.4846900974 | 0.9282900808 |
| ngrn | 0.07381954025 | 0.1056629596 | 0.6986321462 | 0.4847819487 | 1.076612503 |
| megf10 | 0.0660129588 | 0.09453481872 | 0.6982925412 | 0.4849942629 | 1.06824056 |
| chl1 | 0.06655028811 | 0.09536685446 | 0.6978345725 | 0.4852806554 | 1.068814711 |
| yars1 | -0.07113086935 | 0.1020973392 | -0.6966966028 | 0.4859926853 | 0.9313400003 |
| pdia5 | 0.07485100591 | 0.1076791039 | 0.6951302824 | 0.4869736587 | 1.077723564 |
| nucb2 | 0.06693222705 | 0.09632571346 | 0.6948531669 | 0.4871473251 | 1.069223011 |
| pdgfrb | -0.0660380514 | 0.09503932771 | -0.6948497321 | 0.4871494778 | 0.9360952439 |
| c4bpb | 0.06799746909 | 0.09787481048 | 0.6947392159 | 0.4872187471 | 1.070362599 |
| iqgap2 | -0.06812658329 | 0.0980719622 | -0.6946591233 | 0.4872689507 | 0.9341422193 |
| clic5 | 0.06849600308 | 0.09872021031 | 0.69383972 | 0.4877827301 | 1.070896345 |
| sparc | -0.06648635043 | 0.09601439007 | -0.6924623525 | 0.4886470204 | 0.9356756873 |
| glb1 | -0.07132317336 | 0.1030561287 | -0.6920808519 | 0.4888865558 | 0.9311609171 |
| c7orf50 | -0.07467594431 | 0.108021175 | -0.6913083873 | 0.4893717621 | 0.9280441756 |
| hpcal1 | -0.0630578125 | 0.09125912279 | -0.6909754398 | 0.4895809755 | 0.9388891926 |
| dffa | -0.06676345765 | 0.09671078384 | -0.6903413973 | 0.4899795204 | 0.9354164408 |
| lrch4 | -0.07476596322 | 0.1083984117 | -0.6897330143 | 0.4903621003 | 0.9279606378 |
| spink5 | 0.064757243 | 0.09392507939 | 0.6894563562 | 0.4905361291 | 1.066899995 |
| cd82 | -0.07380140848 | 0.107119523 | -0.6889631921 | 0.490846431 | 0.9288561385 |
| sema4d | -0.06464957934 | 0.09397510511 | -0.6879436768 | 0.4914882505 | 0.9373958887 |
| top1mt | -0.07482038775 | 0.1087853291 | -0.6877801298 | 0.4915912508 | 0.9279101354 |
| furin | -0.07564868836 | 0.1100663613 | -0.6873007106 | 0.4918932512 | 0.9271418651 |
| pmvk | -0.06350855499 | 0.09246270895 | -0.6868558765 | 0.4921735543 | 0.9384660907 |
| ctnna1 | 0.07131674243 | 0.104115376 | 0.6849780039 | 0.4933578012 | 1.073921328 |
| ndufa5 | 0.0707268333 | 0.1032655101 | 0.6849027638 | 0.4934052819 | 1.073287999 |
| tjp3 | 0.06998184147 | 0.102310148 | 0.6840166185 | 0.4939646719 | 1.072488706 |
| megf11 | 0.07010643986 | 0.1028190025 | 0.6818432215 | 0.495338091 | 1.072622345 |
| ggh | 0.07422715291 | 0.1089161661 | 0.6815072141 | 0.4955506035 | 1.077051433 |
| amn | 0.06569500445 | 0.09643927108 | 0.6812059414 | 0.4957411891 | 1.067900962 |
| itgam | 0.06322954652 | 0.0930044775 | 0.6798548653 | 0.4965963627 | 1.065271341 |
| prame | 0.06895923502 | 0.1015970113 | 0.6787525949 | 0.497294635 | 1.071392533 |
| dxo | 0.07102847667 | 0.1047549448 | 0.6780441419 | 0.4977437056 | 1.073611798 |
| il10ra | -0.06525019631 | 0.09632931242 | -0.6773659509 | 0.4981737961 | 0.9368330419 |
| tbcb | -0.06360793305 | 0.09392221633 | -0.6772405458 | 0.4982533463 | 0.9383728324 |
| kiaa0319 | 0.06962565644 | 0.1028372691 | 0.677046921 | 0.4983761846 | 1.07210677 |
| osm | -0.06917292355 | 0.1022848686 | -0.6762771902 | 0.498864672 | 0.9331652999 |
| frmd7 | -0.0685162373 | 0.101346991 | -0.67605596 | 0.4990051164 | 0.9337782979 |
| grhpr | -0.07072951756 | 0.1049580618 | -0.6738836098 | 0.5003853132 | 0.9317138703 |
| dut | 0.06941596228 | 0.1030170704 | 0.6738297059 | 0.5004195866 | 1.071881979 |
| prc1 | -0.0687478469 | 0.1020515978 | -0.6736577223 | 0.5005289463 | 0.933562051 |
| ahsa1 | -0.07187828407 | 0.1067784964 | -0.6731531769 | 0.5008498461 | 0.9306441631 |
| cfb | 0.07756319978 | 0.1155683375 | 0.6711457605 | 0.5021276777 | 1.080650527 |
| cpxm1 | -0.06353447419 | 0.09476477776 | -0.6704439739 | 0.5025748103 | 0.9384417667 |
| atg4a | -0.06339606528 | 0.09458784889 | -0.6702347715 | 0.5027081412 | 0.9385716644 |
| clu | 0.07122284799 | 0.1063172583 | 0.6699086222 | 0.502916043 | 1.073820498 |
| ppl | 0.07014210317 | 0.1047325939 | 0.6697256371 | 0.5030327055 | 1.072660599 |
| tubb3 | 0.06877581601 | 0.1026996054 | 0.6696794574 | 0.5030621498 | 1.071196037 |
| il24 | -0.06461169015 | 0.09653434634 | -0.669312971 | 0.5032958543 | 0.9374314066 |

| Protein | beta | se | z | p_val | HR |
|---|---|---|---|---|---|
| prdx5 | -0.06413311838 | 0.09598280704 | -0.6681729818 | 0.5040231798 | 0.9378801421 |
| arid3a | -0.0698278143 | 0.1046422445 | -0.6673004258 | 0.5045802545 | 0.9325543786 |
| clec5a | 0.06171867315 | 0.09254335804 | 0.6669162915 | 0.504825604 | 1.063663066 |
| nfe2 | -0.06814060646 | 0.1026423001 | -0.6638647655 | 0.5067768692 | 0.9341291198 |
| trdmt1 | -0.06622371633 | 0.09977161384 | -0.6637530835 | 0.5068483581 | 0.93592146 |
| col9a1 | 0.06205751492 | 0.09354305374 | 0.6634112576 | 0.5070671979 | 1.06402354 |
| igsf9 | 0.08185612782 | 0.1234074554 | 0.6632996972 | 0.5071386305 | 1.085299654 |
| arid4b | -0.06339269546 | 0.09564975447 | -0.6627585801 | 0.5074851851 | 0.9385748272 |
| ppp1r14a | -0.0653049271 | 0.0985915653 | -0.6623784388 | 0.5077287183 | 0.9367817697 |
| safb2 | 0.06911929404 | 0.1043576173 | 0.6623310862 | 0.5077590585 | 1.071564033 |
| defb104a_defb104b | 0.07426776608 | 0.1122378852 | 0.6616996206 | 0.5081637479 | 1.077095176 |
| c1r | -0.06955910127 | 0.1051775869 | -0.6613490888 | 0.5083884674 | 0.9328050018 |
| rwdd1 | -0.06340226078 | 0.09589308936 | -0.6611765374 | 0.5084991061 | 0.9385658495 |
| otud6b | -0.06731039548 | 0.1018502238 | -0.6608762649 | 0.5086916686 | 0.934904966 |
| pzp | -0.09373385341 | 0.1423591714 | -0.6584321367 | 0.5102604909 | 0.9105250631 |
| cxcl3 | -0.06223804575 | 0.09453220636 | -0.6583792777 | 0.5102944476 | 0.9396591783 |
| ggt1 | -0.07453071128 | 0.1133009107 | -0.6578121115 | 0.5106588709 | 0.9281789681 |
| sat1 | -0.06954230695 | 0.1058335829 | -0.6570911146 | 0.5111223318 | 0.9328206678 |
| gsta3 | -0.06520250151 | 0.09930783739 | -0.6565695439 | 0.5114577374 | 0.936877725 |
| timm10 | -0.06679487584 | 0.1019100266 | -0.6554298737 | 0.5121910228 | 0.9353870521 |
| fxyd5 | -0.06024036654 | 0.09199058339 | -0.65485362 | 0.512562004 | 0.9415381921 |
| insl5 | 0.07869446082 | 0.1202611372 | 0.6543631852 | 0.5128778469 | 1.081873717 |
| bsg | 0.06202165403 | 0.09481362877 | 0.654142815 | 0.5130197996 | 1.063985384 |
| itgb1bp1 | -0.06184500989 | 0.09456756018 | -0.6539770061 | 0.5131266198 | 0.9400285706 |
| vamp5 | -0.06579701358 | 0.1006491763 | -0.6537262998 | 0.5132881561 | 0.9363209054 |
| gast | 0.06951052927 | 0.1065008609 | 0.6526757499 | 0.5139653393 | 1.071983348 |
| stx3 | -0.06790930601 | 0.1040790115 | -0.6524783916 | 0.5140926081 | 0.9343452092 |
| serpinh1 | -0.06590141386 | 0.1010464642 | -0.652189212 | 0.5142791185 | 0.9362231584 |
| icam1 | 0.06272173854 | 0.0963906352 | 0.650703654 | 0.515237804 | 1.064730525 |
| yes1 | -0.05929841622 | 0.09124737272 | -0.6498643682 | 0.5157798361 | 0.9424254921 |
| nudc | -0.05985840955 | 0.09229818172 | -0.6485329228 | 0.5166403241 | 0.9418978879 |
| rab2b | -0.0663448904 | 0.1024159929 | -0.6477981467 | 0.5171155142 | 0.9358080574 |
| clspn | -0.05970148911 | 0.09222449401 | -0.6473495978 | 0.5174057084 | 0.9420457025 |
| scn4b | 0.06705783826 | 0.1036478712 | 0.6469774776 | 0.5176465202 | 1.069357326 |
| cat | 0.07113937135 | 0.1100363967 | 0.646507642 | 0.5179506496 | 1.073730863 |
| acot13 | -0.06537500963 | 0.1012149706 | -0.6459025702 | 0.518342455 | 0.9367161199 |
| thbs4 | -0.06469539967 | 0.1002908674 | -0.6450776759 | 0.5188768499 | 0.9373529379 |
| msra | -0.05991464769 | 0.09293918863 | -0.6446650609 | 0.5191442629 | 0.9418449188 |
| lilrb5 | 0.05981933315 | 0.09289693942 | 0.6439322277 | 0.5196193824 | 1.061644725 |
| nectin1 | -0.0650355498 | 0.1011579944 | -0.642910629 | 0.5202820921 | 0.9370341514 |
| gadd45b | -0.06923356658 | 0.1078011061 | -0.6422342879 | 0.5207210733 | 0.9331087116 |
| vps37a | -0.0576763857 | 0.08985209275 | -0.6419036433 | 0.5209357485 | 0.9439553755 |
| tdrkh | -0.06062214802 | 0.09446698285 | -0.6417284239 | 0.5210495304 | 0.9411787989 |
| atp6v1d | 0.06065882426 | 0.09477728608 | 0.6400143617 | 0.5221632625 | 1.062536341 |
| plxdc1 | 0.05891449339 | 0.09212626807 | 0.6394972316 | 0.522499514 | 1.060684541 |
| rangap1 | 0.05978217504 | 0.09353761175 | 0.639124454 | 0.5227419727 | 1.061605277 |
| cdc123 | -0.06674753929 | 0.1044530659 | -0.6390194363 | 0.5228102879 | 0.9354313312 |
| trim58 | -0.06450111383 | 0.1010175258 | -0.6385140929 | 0.5231390831 | 0.93753507 |
| ptges2 | 0.06367955159 | 0.09976345544 | 0.6383053925 | 0.5232749024 | 1.065750826 |
| kir3dl2 | 0.06733917367 | 0.1055381029 | 0.6380555633 | 0.5234375115 | 1.069658217 |
| fut3_fut5 | -0.06176227754 | 0.09689304781 | -0.637427338 | 0.5238465259 | 0.9401063446 |
| bcr | -0.05934726169 | 0.09310605666 | -0.6374156936 | 0.5238541087 | 0.94237946 |
| plin3 | 0.06272103243 | 0.0986169071 | 0.6360068904 | 0.524771931 | 1.064729773 |
| ppm1b | 0.06485271747 | 0.1021437246 | 0.6349163172 | 0.5254829944 | 1.067001862 |
| rbm19 | -0.07067654987 | 0.1116296688 | -0.6331340997 | 0.5266460759 | 0.9317632223 |
| ephb6 | 0.05782658005 | 0.09139336548 | 0.6327218584 | 0.5269152931 | 1.059531236 |
| dnm3 | -0.06626879452 | 0.1048516859 | -0.6320241199 | 0.5273711164 | 0.9358792713 |
| commd9 | -0.06353564888 | 0.1005322502 | -0.6319927063 | 0.5273916432 | 0.9384406643 |
| fdx1 | 0.07182122655 | 0.1137040801 | 0.6316503899 | 0.527615352 | 1.074463241 |
| smndc1 | 0.07010492832 | 0.1111114971 | 0.6309421628 | 0.5280783422 | 1.072620724 |
| rasa1 | 0.06143344907 | 0.09754753599 | 0.629779609 | 0.5288387886 | 1.063359727 |
| arg2 | 0.0632326104 | 0.1005494984 | 0.6288704708 | 0.5294338596 | 1.065274604 |
| tlr1 | 0.06396868685 | 0.1017294185 | 0.6288120763 | 0.529472093 | 1.066059017 |
| itm2a | 0.05843655026 | 0.09329020906 | 0.626395319 | 0.5310556791 | 1.060177716 |
| arhgap30 | 0.06601284207 | 0.1056875645 | 0.6246036834 | 0.5322312016 | 1.068240435 |
| ache | -0.07159244669 | 0.1146551869 | -0.6244152457 | 0.5323549153 | 0.930910214 |
| snx2 | -0.06414980663 | 0.1028299163 | -0.6238438089 | 0.5327301658 | 0.9378644907 |
| aldh3a1 | 0.05835594262 | 0.09367496336 | 0.622962001 | 0.5333094929 | 1.060092261 |
| plcb2 | -0.06262337046 | 0.1005829275 | -0.6226043727 | 0.533544537 | 0.9392971741 |
| pmch | -0.06513308019 | 0.1046715246 | -0.6222616935 | 0.5337698053 | 0.9369427666 |

| Protein | beta | se | z | p_val | HR |
|---|---|---|---|---|---|
| vat1 | -0.05966716307 | 0.09589744196 | -0.6221976504 | 0.5338119108 | 0.9420780398 |
| clps | 0.05374033492 | 0.08640594297 | 0.6219518366 | 0.5339735384 | 1.055210565 |
| tie1 | 0.05643057033 | 0.09094984415 | 0.6204581312 | 0.5349562109 | 1.058053152 |
| dcun1d2 | -0.06556878322 | 0.1057306018 | -0.6201495317 | 0.5351593446 | 0.9365346267 |
| clstn1 | 0.05909601057 | 0.09539605866 | 0.6194806305 | 0.5355997782 | 1.060877091 |
| edar | 0.06152773762 | 0.09942798782 | 0.6188170853 | 0.5360368655 | 1.063459994 |
| ntrk2 | 0.05867058147 | 0.09500808979 | 0.6175324816 | 0.5368835635 | 1.060425859 |
| snx9 | -0.05980477056 | 0.09706755409 | -0.616114943 | 0.5378186604 | 0.9419484117 |
| tlr2 | 0.0656432738 | 0.1066310558 | 0.6156112149 | 0.5381511477 | 1.067845721 |
| il15ra | 0.06110529496 | 0.09940101302 | 0.6147351331 | 0.5387296538 | 1.063010838 |
| cirbp | -0.06211136283 | 0.1010994461 | -0.6143590814 | 0.5389780689 | 0.9397782246 |
| brap | -0.06302021331 | 0.1027458917 | -0.6133599336 | 0.5396383724 | 0.9389244947 |
| prkar2a | -0.06667523349 | 0.1088340297 | -0.6126322223 | 0.5401195473 | 0.9354989707 |
| dhrs4l2 | -0.06497924945 | 0.1061111955 | -0.6123694028 | 0.5402933808 | 0.9370869082 |
| rbm17 | -0.06479399582 | 0.1062622327 | -0.609755641 | 0.5420236902 | 0.9372605231 |
| mzb1 | 0.06014766678 | 0.09871124417 | 0.6093294364 | 0.5423060994 | 1.061993356 |
| aldh5a1 | -0.06338981207 | 0.1040435991 | -0.6092620077 | 0.5423507853 | 0.9385775335 |
| scarb1 | 0.05977887109 | 0.09822558057 | 0.6085876076 | 0.5427978206 | 1.06160177 |
| hbz | -0.06442291592 | 0.1059229849 | -0.6082052536 | 0.5430513506 | 0.9376083862 |
| epha10 | 0.05801794828 | 0.09542849271 | 0.6079730134 | 0.5432053725 | 1.059734016 |
| fh | 0.06308733514 | 0.1037781006 | 0.6079060492 | 0.5432497873 | 1.065119858 |
| rnf4 | -0.06637402722 | 0.1093172967 | -0.6071685744 | 0.5437390452 | 0.9357807913 |
| sema7a | 0.05752700479 | 0.09489589285 | 0.6062117449 | 0.5443741547 | 1.059213874 |
| pglyrp2 | -0.06471518826 | 0.1070657818 | -0.6044432423 | 0.5455489936 | 0.9373343892 |
| pvr | 0.05615552326 | 0.09302014907 | 0.6036920368 | 0.5460484094 | 1.057762178 |
| aoc1 | 0.06109355106 | 0.1013119303 | 0.603024253 | 0.5464925551 | 1.062998354 |
| was | -0.05841425766 | 0.09687476014 | -0.6029873785 | 0.5465170856 | 0.9432591142 |
| stat5b | -0.05568013569 | 0.09243310873 | -0.6023830255 | 0.5469192064 | 0.9458416285 |
| cep85 | -0.05568031629 | 0.09256404082 | -0.601532904 | 0.5474851029 | 0.9458414577 |
| agt | 0.06342326734 | 0.1054971585 | 0.6011846027 | 0.5477170387 | 1.065477726 |
| map2k6 | -0.05699967234 | 0.09481327869 | -0.6011781591 | 0.54772133 | 0.9445943789 |
| ly96 | -0.06006144207 | 0.09993735318 | -0.6009909224 | 0.5478460324 | 0.9417066714 |
| gbp2 | 0.05720982967 | 0.09527217755 | 0.6004883182 | 0.5481808439 | 1.058877971 |
| slmap | 0.0609481447 | 0.1018228899 | 0.5985701715 | 0.5494595521 | 1.062843799 |
| gas2 | 0.06333418226 | 0.1058858221 | 0.598136568 | 0.5497488121 | 1.065382812 |
| inhbc | 0.06259245295 | 0.1046518037 | 0.5981019986 | 0.5497718768 | 1.064592879 |
| vstm1 | -0.05684998418 | 0.09523464852 | -0.5969464377 | 0.5505431419 | 0.9447357841 |
| aplp1 | -0.0580923218 | 0.09772168523 | -0.5944670485 | 0.5521997753 | 0.943562832 |
| cpa1 | -0.05135409764 | 0.08649301397 | -0.5937369423 | 0.5526880704 | 0.9499422387 |
| pmm2 | -0.06337487655 | 0.1068461861 | -0.5931412141 | 0.5530866504 | 0.9385915518 |
| kir2ds4 | 0.06287606066 | 0.1062572412 | 0.5917343603 | 0.5540284839 | 1.064894849 |
| bola2_bola2b | -0.06598066048 | 0.1115271726 | -0.5916106268 | 0.5541113561 | 0.9361489688 |
| ankrd54 | -0.05623189521 | 0.09522240631 | -0.5905321802 | 0.5548339179 | 0.9453198953 |
| scn2a | 0.0578643802 | 0.09799718732 | 0.5904698061 | 0.5548757228 | 1.059571287 |
| zp3 | 0.06057127155 | 0.1027235811 | 0.5896530371 | 0.5554232864 | 1.062443317 |
| paep | -0.06377474407 | 0.1081877593 | -0.589482068 | 0.5555379378 | 0.9382163145 |
| nars1 | -0.05972269673 | 0.1014129101 | -0.5889062515 | 0.5559241639 | 0.9420257242 |
| atp1b2 | -0.06496535619 | 0.1105774627 | -0.5875099195 | 0.5568612904 | 0.9370999275 |
| echs1 | -0.0632395708 | 0.1076410556 | -0.5875041867 | 0.5568651395 | 0.9387185572 |
| gh2 | -0.0534695592 | 0.09111037052 | -0.5868657858 | 0.55729385 | 0.9479347965 |
| npl | -0.07067321339 | 0.1204645169 | -0.586672451 | 0.5574237134 | 0.9317663311 |
| comt | -0.05613552012 | 0.09577838129 | -0.586098025 | 0.5578096434 | 0.945411005 |
| omp | 0.06294495421 | 0.1074303603 | 0.5859140196 | 0.5579332955 | 1.064968216 |
| glod4 | -0.05655924679 | 0.09653863408 | -0.5858716288 | 0.5579617842 | 0.945010494 |
| tnni3 | 0.05547062864 | 0.09469394896 | 0.5857885245 | 0.5580176363 | 1.05703797 |
| cd46 | 0.05650442987 | 0.09646593264 | 0.5857449187 | 0.5580469436 | 1.058131302 |
| lsp1 | 0.05940334286 | 0.1015333043 | 0.5850626383 | 0.5585055997 | 1.061203183 |
| cplx2 | 0.06186221361 | 0.1058393791 | 0.5844914637 | 0.5588897068 | 1.063815755 |
| ment | -0.06881496438 | 0.1178022143 | -0.5841567989 | 0.5591148239 | 0.9334993947 |
| hgf | 0.06604763378 | 0.113160465 | 0.5836635063 | 0.5594467245 | 1.068277602 |
| cfhr5 | -0.05940478977 | 0.1019168263 | -0.5828751927 | 0.5599773216 | 0.9423252483 |
| ncf2 | 0.05671298226 | 0.09731557148 | 0.5827739734 | 0.5600454679 | 1.058352001 |
| atp6v1g1 | -0.06001396753 | 0.1030108622 | -0.5825984393 | 0.5601636563 | 0.9417513796 |
| stxbp1 | -0.05976334172 | 0.1027791606 | -0.5814733394 | 0.5609214816 | 0.9419874363 |
| ywhaq | -0.06050182124 | 0.1040535959 | -0.5814486343 | 0.5609381276 | 0.9412920547 |
| vcpkmt | -0.0630504794 | 0.1084406693 | -0.5814283497 | 0.5609517952 | 0.9388960776 |
| cdc26 | -0.05963209034 | 0.1026646355 | -0.5808435401 | 0.5613459074 | 0.9421110816 |
| ptpn1 | -0.05507262827 | 0.09516368223 | -0.5787147679 | 0.5627816497 | 0.9464164089 |
| arhgef1 | -0.05876130851 | 0.1017330529 | -0.577602921 | 0.5635322344 | 0.9429318121 |
| c19orf12 | -0.05548998253 | 0.09623233176 | -0.5766251479 | 0.5641927072 | 0.9460215004 |

| Protein | beta | se | z | p_val | HR |
|---|---|---|---|---|---|
| cfp | -0.06115089029 | 0.1061243002 | -0.5762194915 | 0.5644668321 | 0.9406812894 |
| cyb5r2 | -0.05885387185 | 0.1021918556 | -0.5759154827 | 0.56467231 | 0.9428445352 |
| rtn4ip1 | -0.05655207568 | 0.09819689469 | -0.5759049292 | 0.5646794437 | 0.9450172708 |
| acadm | 0.06270675894 | 0.1088928918 | 0.5758572293 | 0.5647116872 | 1.064714575 |
| gcc1 | -0.05764970028 | 0.1001968587 | -0.5753643481 | 0.5650449106 | 0.9439805657 |
| tpt1 | -0.0563832018 | 0.09810268766 | -0.5747365658 | 0.5654694737 | 0.945176873 |
| ensa | -0.06012198075 | 0.1046587384 | -0.5744573426 | 0.5656583588 | 0.9416496634 |
| tek | 0.05785236608 | 0.1009387454 | 0.5731433043 | 0.5665476685 | 1.059558557 |
| sptlc1 | -0.05789662181 | 0.1010706834 | -0.5728329903 | 0.5667577795 | 0.9437475053 |
| scn3b | -0.05861316533 | 0.1023543946 | -0.5726492306 | 0.5668822191 | 0.9430715114 |
| arhgap1 | -0.05403539053 | 0.09445020039 | -0.572104562 | 0.5672511387 | 0.947398577 |
| ppib | -0.05290451699 | 0.09249067154 | -0.5719984092 | 0.5673230524 | 0.948470571 |
| fgf20 | -0.0577912216 | 0.101218567 | -0.5709547497 | 0.5680303167 | 0.9438469817 |
| tcp11 | 0.06268044218 | 0.1097964708 | 0.5708784782 | 0.5680820207 | 1.064686556 |
| adgrv1 | -0.05988413763 | 0.1053997707 | -0.5681619348 | 0.5699250158 | 0.941873655 |
| ssc4d | -0.06172545138 | 0.1086736461 | -0.5679891453 | 0.5700423385 | 0.9401409658 |
| htr1b | -0.05943456862 | 0.1049810171 | -0.5661458637 | 0.5712946277 | 0.9422971874 |
| sugt1 | -0.0534116525 | 0.09470197326 | -0.5639972501 | 0.5727560036 | 0.9479896898 |
| maged1 | -0.05102106357 | 0.0905225876 | -0.5636279842 | 0.5730073378 | 0.9502586545 |
| tmed4 | 0.06028075316 | 0.1070155421 | 0.5632897054 | 0.573237627 | 1.062134702 |
| ceacam8 | -0.05587687821 | 0.09924068671 | -0.5630440504 | 0.5734048885 | 0.9456555595 |
| akt1s1 | -0.05406518112 | 0.09614786074 | -0.5623128867 | 0.5739028598 | 0.9473703538 |
| ivd | -0.05274535168 | 0.09384851344 | -0.5620265015 | 0.5740979631 | 0.9486215466 |
| lrpap1 | -0.05838767395 | 0.1038920058 | -0.5620035295 | 0.5741136144 | 0.9432841898 |
| cnst | -0.05068535195 | 0.09037564408 | -0.5608297729 | 0.5749135881 | 0.9505777209 |
| tdo2 | -0.06056365815 | 0.1079901446 | -0.56082579 | 0.5749163036 | 0.9412338499 |
| dnajb14 | -0.05667749963 | 0.1012566341 | -0.5597410986 | 0.575656045 | 0.9448987504 |
| mgll | -0.05059753085 | 0.09056773612 | -0.5586705931 | 0.5763865523 | 0.9506612054 |
| pdlim7 | -0.05139076902 | 0.09216753032 | -0.5575799725 | 0.5771312355 | 0.9499074036 |
| mthfd2 | 0.05748018968 | 0.1031196096 | 0.557412794 | 0.5772454262 | 1.059164288 |
| c1qtnf6 | 0.05763883567 | 0.1034269743 | 0.5572901657 | 0.5773291937 | 1.059332334 |
| il25 | 0.06076142048 | 0.1091260389 | 0.5568003852 | 0.5776638207 | 1.062645359 |
| gamt | 0.05886573323 | 0.1057341912 | 0.5567331871 | 0.5777097388 | 1.060632823 |
| eif4g1 | -0.0532357961 | 0.09564767437 | -0.5565822321 | 0.5778128962 | 0.9481564145 |
| casp10 | -0.05172156597 | 0.09295014456 | -0.5564441692 | 0.5779072513 | 0.9495932291 |
| crh | -0.05200337665 | 0.09352138699 | -0.5560586549 | 0.578170758 | 0.9493256613 |
| ass1 | 0.06006537759 | 0.1080689142 | 0.5558062466 | 0.5783433148 | 1.061905969 |
| ppp2r5a | -0.05677031782 | 0.1022133865 | -0.5554098126 | 0.5786143823 | 0.9448110507 |
| ermap | -0.06254283855 | 0.1126086572 | -0.5553999142 | 0.5786211513 | 0.9393728206 |
| triap1 | 0.05337791198 | 0.09635299842 | 0.5539828843 | 0.5795905593 | 1.054828202 |
| shmt1 | -0.05341481582 | 0.09642199674 | -0.553969194 | 0.5795999288 | 0.947986691 |
| itga5 | 0.05546279908 | 0.100160675 | 0.5537382716 | 0.5797579791 | 1.057029694 |
| ncam2 | -0.0536810304 | 0.09698031253 | -0.5535250299 | 0.5799039461 | 0.9477343567 |
| rilp | -0.05467623769 | 0.09886532673 | -0.5530375461 | 0.5802377006 | 0.9467916338 |
| cbs | -0.06123682801 | 0.1108829827 | -0.5522653387 | 0.5807665745 | 0.9406004529 |
| tnfrsf10c | -0.05221522187 | 0.0946052778 | -0.5519271555 | 0.5809982624 | 0.9491245725 |
| casp9 | -0.05537511421 | 0.1004613029 | -0.5512084019 | 0.5814908212 | 0.9461301745 |
| hgs | -0.0533861701 | 0.09691072207 | -0.5508799125 | 0.5817159985 | 0.9480138472 |
| reps1 | -0.05648891135 | 0.1025656709 | -0.5507584638 | 0.5817992611 | 0.9450769641 |
| ctla4 | -0.05708277019 | 0.1037351575 | -0.5502740976 | 0.5821313876 | 0.9445158884 |
| blnk | -0.05904519643 | 0.1074158441 | -0.5496879622 | 0.5825334148 | 0.9426641631 |
| tagln3 | 0.05744685184 | 0.1045636735 | 0.549395884 | 0.5827337981 | 1.059128978 |
| ryr1 | -0.05808171123 | 0.1058027354 | -0.5489622836 | 0.5830313335 | 0.9435728438 |
| pgd | 0.05873971533 | 0.1070054045 | 0.5489415754 | 0.5830455452 | 1.060499173 |
| fcrl5 | 0.05369917797 | 0.09785727476 | 0.5487499841 | 0.5831770387 | 1.055167137 |
| mfap3 | 0.05357438316 | 0.09775344617 | 0.5480562094 | 0.5836533078 | 1.055035466 |
| mrpl24 | -0.05586175911 | 0.1020018968 | -0.5476541206 | 0.5839294204 | 0.9456698571 |
| cd2 | -0.05918034614 | 0.1081492766 | -0.5472098194 | 0.5842345909 | 0.942536771 |
| zbp1 | 0.06030876019 | 0.1102114842 | 0.5472094004 | 0.5842348787 | 1.06216445 |
| il6r | 0.05267625523 | 0.09630418339 | 0.5469778505 | 0.5843939493 | 1.054088334 |
| birc2 | -0.05268639911 | 0.0964184649 | -0.5464347432 | 0.5847671333 | 0.9486774719 |
| lrrfip1 | -0.05708675659 | 0.1044904744 | -0.5463345527 | 0.5848359891 | 0.9445121232 |
| hs6st1 | 0.05162071634 | 0.09465847411 | 0.5453364511 | 0.5855221386 | 1.05297629 |
| nedd4l | -0.05761783398 | 0.1057074809 | -0.5450686509 | 0.5857063026 | 0.9440106473 |
| tigar | -0.05399859958 | 0.09928765712 | -0.5438601448 | 0.5865377168 | 0.9474334333 |
| fkbp14 | -0.05454062405 | 0.1008982775 | -0.540550596 | 0.5888173781 | 0.9469200403 |
| pinlyp | -0.05990599326 | 0.1109063348 | -0.5401494274 | 0.5890939861 | 0.94185307 |
| il1r2 | 0.0513800382 | 0.09523029967 | 0.5395345639 | 0.5895180543 | 1.052722892 |
| irak4 | -0.04977478333 | 0.09270281193 | -0.5369285169 | 0.591316992 | 0.9514436814 |
| hs3st3b1 | 0.047551934 | 0.08859675519 | 0.5367232005 | 0.5914588278 | 1.048700663 |

| Protein | beta | se | z | p_val | HR |
|---|---|---|---|---|---|
| bdnf | -0.05582319571 | 0.1040581898 | -0.5364613379 | 0.5916397492 | 0.945706326 |
| pcbd1 | 0.06168748711 | 0.1151339018 | 0.535789078 | 0.5921043314 | 1.063629895 |
| tia1 | -0.0492818091 | 0.09198296673 | -0.535771033 | 0.5921168043 | 0.9519128342 |
| spag1 | -0.05673061904 | 0.1059474421 | -0.5354600162 | 0.592331799 | 0.9448485593 |
| cdh2 | 0.05674602617 | 0.1060483212 | 0.5350959402 | 0.5925835171 | 1.058386974 |
| adgre1 | 0.0574421659 | 0.1074311542 | 0.5346881576 | 0.5928655117 | 1.059124015 |
| psmd9 | 0.05345738561 | 0.1001109493 | 0.5339814075 | 0.5933543976 | 1.054912036 |
| ccl2 | -0.05241192699 | 0.09818836562 | -0.5337895855 | 0.5934871199 | 0.9489378932 |
| susd5 | -0.06183499091 | 0.1158734169 | -0.5336425952 | 0.5935888323 | 0.9400379888 |
| kdm3a | 0.05457395379 | 0.1022750654 | 0.5335997936 | 0.5936184511 | 1.056090575 |
| bola1 | -0.0567893403 | 0.1066210577 | -0.5326278083 | 0.5942912484 | 0.9447930782 |
| septin3 | -0.05593014437 | 0.105060753 | -0.5323600182 | 0.5944766709 | 0.9456051894 |
| gal | -0.05323946622 | 0.1002236399 | -0.5312066723 | 0.5952755698 | 0.9481529347 |
| rab39b | 0.05775938621 | 0.1088652397 | 0.530558573 | 0.5957247097 | 1.059460044 |
| il18 | -0.05288148212 | 0.09972891503 | -0.5302522554 | 0.5959370449 | 0.9484924191 |
| elavl4 | 0.05923910126 | 0.1119561588 | 0.5291276686 | 0.5967168888 | 1.061028904 |
| ddx58 | -0.0525942445 | 0.0994626638 | -0.5287837917 | 0.5969554427 | 0.948764901 |
| tor1aip1 | 0.05542092339 | 0.1049798421 | 0.5279196681 | 0.5975550931 | 1.056985431 |
| scin | -0.05645415868 | 0.1070036696 | -0.5275908657 | 0.5977833342 | 0.9451098086 |
| nptxr | -0.04717893617 | 0.08985349383 | -0.5250651273 | 0.5995379174 | 0.9539166921 |
| tp53i3 | 0.05574106522 | 0.1061866673 | 0.5249346892 | 0.5996285936 | 1.05732387 |
| vit | 0.05921964844 | 0.1129241506 | 0.5244196934 | 0.5999866621 | 1.061008264 |
| mtpn | 0.05050893703 | 0.09633022996 | 0.5243311165 | 0.600048258 | 1.051806263 |
| sla2 | -0.05334998763 | 0.1017821623 | -0.5241585207 | 0.6001682884 | 0.9480481493 |
| sdc1 | -0.0516485553 | 0.09862843736 | -0.5236679874 | 0.6005094851 | 0.9496625621 |
| il4 | -0.04784578191 | 0.09138120812 | -0.5235844754 | 0.6005675817 | 0.9532807889 |
| mtus1 | 0.0561737992 | 0.1073016571 | 0.5235128769 | 0.6006173925 | 1.057781509 |
| hdgfl2 | -0.0549322269 | 0.1049401785 | -0.5234622971 | 0.6006525817 | 0.9465492964 |
| pcbp2 | -0.0537725696 | 0.102826782 | -0.522943231 | 0.6010137592 | 0.9476476059 |
| sod1 | -0.05335228693 | 0.1020936277 | -0.5225819489 | 0.6012652049 | 0.9480459694 |
| galnt10 | 0.05080116828 | 0.09726186427 | 0.5223133307 | 0.6014521891 | 1.052113679 |
| kifbp | -0.04789697059 | 0.091709155 | -0.5222703293 | 0.6014821246 | 0.953231993 |
| hmgcl | -0.05420969736 | 0.1038052454 | -0.5222250295 | 0.6015136609 | 0.9472334533 |
| eci2 | 0.05571813463 | 0.1067099765 | 0.5221455055 | 0.6015690249 | 1.057299626 |
| prdx2 | 0.05774166038 | 0.1107645048 | 0.5213011199 | 0.602157021 | 1.059441265 |
| gmpr2 | -0.05492370095 | 0.1054479453 | -0.520860798 | 0.6024637461 | 0.9465573666 |
| pnlip | -0.0492345465 | 0.09453073543 | -0.5208310956 | 0.6024844391 | 0.9519578251 |
| cabp2 | 0.05374456602 | 0.1032065711 | 0.5207475209 | 0.6025426655 | 1.05521503 |
| casp1 | -0.04726671286 | 0.09091247361 | -0.5199144956 | 0.6031231717 | 0.9538329641 |
| il1rl2 | -0.0530884356 | 0.1022305788 | -0.5193009392 | 0.6035508986 | 0.9482961456 |
| krt14 | -0.0496215738 | 0.09558108246 | -0.5191568512 | 0.6036513659 | 0.9515894628 |
| ifnlr1 | 0.05284176661 | 0.1018188802 | 0.5189780766 | 0.6037760295 | 1.054262812 |
| clns1a | -0.05418546551 | 0.1044504837 | -0.5187670136 | 0.6039232235 | 0.9472564068 |
| cd300lg | 0.04873345069 | 0.09407713329 | 0.5180158981 | 0.6044471772 | 1.049940453 |
| sirt5 | 0.04937949207 | 0.09533345561 | 0.5179660357 | 0.6044819668 | 1.050618977 |
| rfc4 | -0.05585352588 | 0.1078747259 | -0.5177628531 | 0.604623739 | 0.945677643 |
| kif20b | -0.05306882802 | 0.1025743149 | -0.5173695586 | 0.6048982057 | 0.9483147396 |
| pafah2 | -0.0534998745 | 0.1035596581 | -0.5166092228 | 0.6054289762 | 0.94790606 |
| anxa3 | -0.04792726476 | 0.09281525876 | -0.5163726891 | 0.6055941367 | 0.953203116 |
| plscr3 | -0.05470006973 | 0.1059590108 | -0.5162380182 | 0.6056881802 | 0.9467690701 |
| nubp1 | 0.05259505434 | 0.1020722795 | 0.5152726536 | 0.6063625056 | 1.054002745 |
| ddi2 | 0.05627655301 | 0.1092584803 | 0.5150772081 | 0.6064990687 | 1.057890206 |
| crybb2 | 0.05115694504 | 0.09943958995 | 0.5144524939 | 0.6069356662 | 1.052488063 |
| tpbgl | -0.05347188701 | 0.1040778373 | -0.5137682373 | 0.6074140373 | 0.9479325898 |
| f10 | -0.05238188805 | 0.102093529 | -0.513077455 | 0.6078971412 | 0.9489663987 |
| aifm1 | -0.04836315051 | 0.09455089633 | -0.5115038819 | 0.6089982709 | 0.9527877189 |
| spint1 | 0.04603917167 | 0.09018239911 | 0.5105117198 | 0.609693006 | 1.047115427 |
| dnajb6 | -0.05237434186 | 0.1025924147 | -0.5105089106 | 0.6096949736 | 0.9489735598 |
| mertk | 0.05177829855 | 0.10145785 | 0.5103429507 | 0.6098112171 | 1.053142233 |
| pglyrp1 | -0.04785439891 | 0.09392171457 | -0.5095136852 | 0.6103922083 | 0.9532725745 |
| immt | 0.05849963513 | 0.1148368448 | 0.5094152077 | 0.6104612188 | 1.060244599 |
| wasl | 0.05428157944 | 0.106646721 | 0.5089849828 | 0.6107627503 | 1.055781847 |
| col24a1 | 0.052704823 | 0.1035915284 | 0.5087754167 | 0.6109096527 | 1.054118448 |
| il34 | -0.04555745556 | 0.08959845841 | -0.5084624933 | 0.611129036 | 0.9554647042 |
| tsc1 | -0.05121955814 | 0.1008630183 | -0.507813062 | 0.6115844485 | 0.950070052 |
| src | -0.04731626159 | 0.09324692805 | -0.5074297092 | 0.6118533445 | 0.9537857041 |
| sema4c | -0.04859846331 | 0.09577687919 | -0.5074133102 | 0.6118648485 | 0.9525635421 |
| ccn2 | -0.05035346909 | 0.09939226085 | -0.5066135799 | 0.612425978 | 0.9508932537 |
| adipoq | -0.06132928169 | 0.1214572793 | -0.5049452949 | 0.6135972594 | 0.9405134949 |
| rictor | -0.05180067236 | 0.1026359225 | -0.5047031398 | 0.6137673554 | 0.9495181132 |

| Protein | beta | se | z | p_val | HR |
|---|---|---|---|---|---|
| afm | -0.05387015572 | 0.1071823491 | -0.5026028646 | 0.6152435148 | 0.9475551331 |
| draxin | 0.04715165109 | 0.0938752994 | 0.5022796347 | 0.6154708326 | 1.04828097 |
| gmpr | 0.04981361109 | 0.0992772632 | 0.5017625334 | 0.615834571 | 1.051075169 |
| mcts1 | -0.05489570571 | 0.1094127769 | -0.5017303032 | 0.6158572454 | 0.9465838661 |
| cenpj | 0.05397423761 | 0.1075906228 | 0.5016630277 | 0.6159045759 | 1.055457411 |
| babam1 | -0.05165231911 | 0.1030699944 | -0.5011382739 | 0.6162738121 | 0.9496589877 |
| ccl18 | 0.0526225463 | 0.1050075268 | 0.5011311844 | 0.6162788012 | 1.054031722 |
| kcnip4 | -0.04781489495 | 0.09550209452 | -0.5006685475 | 0.6166044114 | 0.9533102333 |
| atxn2 | 0.05190018344 | 0.1036662649 | 0.5006467968 | 0.6166197216 | 1.053270603 |
| mmp1 | 0.04734454861 | 0.0946950812 | 0.499968404 | 0.6170973253 | 1.0484832 |
| crim1 | 0.04775508497 | 0.0955627466 | 0.4997249103 | 0.6172687899 | 1.048913729 |
| crhr1 | -0.0476255786 | 0.09541952929 | -0.4991177273 | 0.6176964497 | 0.9534907276 |
| penk | 0.05254131565 | 0.1055407021 | 0.4978298854 | 0.6186039498 | 1.053946106 |
| pcdh17 | -0.04586144054 | 0.0921506468 | -0.4976789869 | 0.6187103211 | 0.9551743015 |
| srpk2 | -0.04661448151 | 0.09382966924 | -0.4967989538 | 0.6193308331 | 0.9544552868 |
| ppp1r2 | -0.04673326817 | 0.09419723454 | -0.4961214455 | 0.6198087295 | 0.954341917 |
| il13ra2 | -0.05058706502 | 0.1022097944 | -0.4949336347 | 0.6206469675 | 0.9506711549 |
| btla | -0.0538430274 | 0.1088836395 | -0.4945006216 | 0.6209526674 | 0.9475808391 |
| gsap | 0.04501041534 | 0.09104138996 | 0.4943950808 | 0.6210271874 | 1.046038755 |
| sprr3 | 0.05468668633 | 0.1107946802 | 0.4935858495 | 0.6215986964 | 1.056209638 |
| npr1 | 0.05172105141 | 0.1048044959 | 0.4935003117 | 0.6216591196 | 1.053081946 |
| dpp10 | 0.04257943806 | 0.08631682705 | 0.4932924381 | 0.6218059707 | 1.043498947 |
| caps | 0.05410368706 | 0.110283574 | 0.4905869942 | 0.623718587 | 1.055594048 |
| sema6c | 0.05113796841 | 0.1043987637 | 0.4898330843 | 0.6242520173 | 1.052468091 |
| atp6ap2 | 0.04840013618 | 0.09887428549 | 0.4895118679 | 0.6244793545 | 1.04959055 |
| itgb1 | -0.04504051769 | 0.09212801321 | -0.4888905786 | 0.6249191661 | 0.9559587478 |
| cep43 | -0.04551499085 | 0.09319270118 | -0.488396519 | 0.6252690071 | 0.9555052786 |
| defa1_defa1b | -0.04581557987 | 0.093955085 | -0.4876327861 | 0.6258099683 | 0.9552181074 |
| ddt | -0.05389031372 | 0.1106959099 | -0.4868320227 | 0.626377375 | 0.9475360325 |
| spesp1 | 0.05315529074 | 0.1092668146 | 0.4864724111 | 0.6266322614 | 1.054593401 |
| efcab14 | 0.05300765444 | 0.10926084 | 0.4851477844 | 0.6275715182 | 1.054437716 |
| tinagl1 | 0.04639375304 | 0.09593698793 | 0.4835856747 | 0.6286799434 | 1.047486781 |
| stc1 | 0.0495000909 | 0.1024983706 | 0.4829353933 | 0.6291416102 | 1.050745688 |
| cxcl13 | 0.0486881953 | 0.1008184262 | 0.4829295312 | 0.6291457727 | 1.049892938 |
| mdga1 | 0.04609976188 | 0.09548084925 | 0.4828168396 | 0.6292257929 | 1.047178874 |
| glrx | -0.04685547312 | 0.09720531564 | -0.4820258317 | 0.629787596 | 0.9542252988 |
| becn1 | -0.05228520604 | 0.1084706061 | -0.482021885 | 0.6297903997 | 0.9490581511 |
| igfl4 | 0.04916102596 | 0.1020009055 | 0.4819665642 | 0.6298296986 | 1.050389477 |
| ilkap | -0.04461581772 | 0.09260401469 | -0.4817913982 | 0.6299541405 | 0.9563648297 |
| cacna1c | 0.0513294599 | 0.106610751 | 0.4814660755 | 0.630185285 | 1.052669649 |
| atox1 | 0.04580934228 | 0.09540967881 | 0.480133073 | 0.6311327725 | 1.046874797 |
| nmt1 | -0.04862319349 | 0.1012838057 | -0.4800687843 | 0.6311784837 | 0.9525399854 |
| spring1 | -0.05344281558 | 0.1115525693 | -0.4790818887 | 0.6318803737 | 0.947960148 |
| rps10 | 0.05260363511 | 0.1098533664 | 0.4788531916 | 0.6320430727 | 1.054011789 |
| ep300 | -0.04812769625 | 0.1005192595 | -0.4787907956 | 0.6320874654 | 0.9530120832 |
| spp1 | -0.04437278389 | 0.09269681143 | -0.4786872732 | 0.6321611211 | 0.9565972869 |
| vstm2b | -0.04993205465 | 0.1043424697 | -0.4785400883 | 0.6322658486 | 0.9512940583 |
| micall2 | 0.04956467644 | 0.1041222606 | 0.4760238217 | 0.6340574068 | 1.050813553 |
| gpha2 | 0.05119624018 | 0.1076610741 | 0.4755315755 | 0.6344081329 | 1.052529422 |
| b3gat3 | -0.05060856021 | 0.1066260653 | -0.4746359164 | 0.6350465017 | 0.9506507202 |
| hnrnpk | -0.04467936572 | 0.09456788616 | -0.4724581201 | 0.6365998279 | 0.9563040565 |
| fcgr3b | -0.04850792669 | 0.1027044738 | -0.4723058781 | 0.6367084753 | 0.9526497879 |
| ap2b1 | -0.04870250765 | 0.1032303638 | -0.4717847138 | 0.637080463 | 0.9524644384 |
| tnpo1 | 0.05210392863 | 0.110449042 | 0.4717463158 | 0.6371078736 | 1.053485224 |
| acaa1 | -0.04449422772 | 0.09434379813 | -0.4716179399 | 0.6371995192 | 0.9564811212 |
| pten | -0.04346010566 | 0.09216924245 | -0.4715250392 | 0.6372658431 | 0.957470751 |
| osbpl2 | -0.05107537933 | 0.1085419318 | -0.4705589671 | 0.6379557156 | 0.9502070419 |
| ccl13 | -0.04594009649 | 0.09766022843 | -0.4704074241 | 0.638063961 | 0.9550991743 |
| f7 | -0.04538082293 | 0.09671583262 | -0.4692181383 | 0.6389137217 | 0.9556334854 |
| usp25 | -0.04988190853 | 0.1063239892 | -0.4691500848 | 0.6389623612 | 0.9513417632 |
| dync1h1 | 0.0511465468 | 0.1094109509 | 0.4674719158 | 0.6401622805 | 1.052477119 |
| treh | -0.04825022612 | 0.103254634 | -0.4672935661 | 0.640289859 | 0.9528953179 |
| pla2g2a | -0.04508009512 | 0.09657832745 | -0.4667723734 | 0.6406627435 | 0.9559209142 |
| cpb2 | 0.04973651474 | 0.1066134226 | 0.4665126914 | 0.6408485653 | 1.050994138 |
| crebzf | 0.04922804973 | 0.1055685241 | 0.466313706 | 0.6409909696 | 1.050459881 |
| fcar | -0.04298424741 | 0.09227213964 | -0.4658421011 | 0.6413285271 | 0.9579264798 |
| cela3a | -0.04063440424 | 0.08729036754 | -0.4655084563 | 0.6415673826 | 0.9601801036 |
| psip1 | 0.04414096966 | 0.0948429081 | 0.4654113897 | 0.6416368793 | 1.045129676 |
| tgfbr3 | 0.0415634562 | 0.08953814049 | 0.4641983402 | 0.6425056503 | 1.042439309 |
| mcfd2 | -0.04323601048 | 0.09330173508 | -0.4633998547 | 0.6430777828 | 0.9576853396 |

| Protein | beta | se | z | p_val | HR |
|---|---|---|---|---|---|
| sord | -0.04517575772 | 0.09748766241 | -0.4633997431 | 0.6430778628 | 0.9558294727 |
| wfikkn2 | 0.04499227304 | 0.09725771998 | 0.462608758 | 0.6436448299 | 1.046019777 |
| eif1ax | -0.04861084688 | 0.1051275182 | -0.4623988818 | 0.643795301 | 0.9525517461 |
| ager | -0.04189447759 | 0.09061442403 | -0.4623378457 | 0.6438390638 | 0.9589709682 |
| tnc | 0.04319352429 | 0.09343088661 | 0.4623045532 | 0.6438629349 | 1.044139942 |
| ccl19 | 0.04685021789 | 0.1014954319 | 0.461599276 | 0.6443687139 | 1.047965031 |
| otud7b | -0.0473457321 | 0.1026370126 | -0.4612929674 | 0.6445884298 | 0.953757596 |
| vsig10 | 0.04946148666 | 0.1073357826 | 0.4608107891 | 0.6449343604 | 1.050705125 |
| ppif | -0.04491007529 | 0.09759680312 | -0.4601592865 | 0.6454018919 | 0.9560834535 |
| ca11 | 0.04348375555 | 0.09454882104 | 0.4599079616 | 0.6455822853 | 1.044443028 |
| siglec9 | -0.04223274941 | 0.09202042637 | -0.4589497254 | 0.6462702692 | 0.9586466302 |
| clec4c | -0.04284548347 | 0.09353274727 | -0.4580800278 | 0.6468949471 | 0.9580594146 |
| cd8a | 0.04285298908 | 0.09363775534 | 0.4576464795 | 0.647206445 | 1.043784436 |
| clec1a | 0.0425364827 | 0.09296567385 | 0.4575504155 | 0.6472754739 | 1.043454124 |
| pdcl2 | -0.0483217016 | 0.105653079 | -0.4573619818 | 0.6474108859 | 0.9528272117 |
| klrf1 | 0.04917549556 | 0.1076619806 | 0.4567582286 | 0.6478448328 | 1.050404676 |
| hnrnpul1 | 0.05117641205 | 0.1120778622 | 0.4566148125 | 0.6479479305 | 1.052508552 |
| dnaja1 | 0.05078347745 | 0.1113620538 | 0.4560213801 | 0.648374604 | 1.052095066 |
| ythdf3 | -0.04263993193 | 0.09377443839 | -0.4547074092 | 0.6493197498 | 0.9582563655 |
| ghrhr | 0.04149497081 | 0.09129630878 | 0.4545087459 | 0.6494626985 | 1.04236792 |
| sprr1b | 0.04846015095 | 0.1066234195 | 0.454498188 | 0.6494702959 | 1.049653543 |
| stab2 | 0.04847340301 | 0.1072246581 | 0.4520732812 | 0.6512161913 | 1.049667453 |
| cdc37 | -0.04270161638 | 0.09447470601 | -0.4519899366 | 0.6512762322 | 0.9581972578 |
| igfbpl1 | 0.04301143849 | 0.09519832264 | 0.451808785 | 0.6514067404 | 1.043949836 |
| st13 | -0.04857457841 | 0.1076062998 | -0.4514101729 | 0.6516939527 | 0.9525862943 |
| jchain | 0.04351637258 | 0.09653654036 | 0.4507761767 | 0.6521508731 | 1.044477095 |
| csrp3 | 0.05126682154 | 0.1139810184 | 0.4497838522 | 0.6528663024 | 1.052603713 |
| kazald1 | 0.04169655107 | 0.09289900125 | 0.4488374526 | 0.6535489191 | 1.042578062 |
| ikzf2 | 0.04271819318 | 0.09530713672 | 0.4482161005 | 0.653997244 | 1.043643747 |
| tarm1 | 0.04664337006 | 0.1045727383 | 0.4460375699 | 0.6555701076 | 1.047748284 |
| col15a1 | -0.04554263212 | 0.1021061422 | -0.4460322478 | 0.655573952 | 0.9554788676 |
| gimap7 | -0.04685209564 | 0.1051197491 | -0.4457021259 | 0.6558124287 | 0.9542285217 |
| denr | -0.04681184464 | 0.1051850361 | -0.4450428157 | 0.6562888129 | 0.9542669311 |
| elob | -0.04650896388 | 0.1045786946 | -0.4447269499 | 0.6565170909 | 0.954556004 |
| cebpb | -0.04359736542 | 0.09804781609 | -0.4446541204 | 0.6565697299 | 0.9573393378 |
| kel | -0.04310427096 | 0.09707519554 | -0.4440297104 | 0.6570211037 | 0.9578115129 |
| tmed1 | 0.04572883159 | 0.1030120991 | 0.4439170932 | 0.6571025258 | 1.046790516 |
| parp1 | 0.04454515647 | 0.1003875173 | 0.4437320261 | 0.657236338 | 1.045552189 |
| tnfsf8 | 0.04391159034 | 0.0991519624 | 0.4428716213 | 0.6578585951 | 1.044889972 |
| pde4d | -0.04763862266 | 0.1079632953 | -0.4412483198 | 0.6590332355 | 0.9534782903 |
| fkbp7 | -0.04299282031 | 0.09750114044 | -0.4409468455 | 0.6592514787 | 0.9579182676 |
| vsir | -0.04242505205 | 0.09631305446 | -0.4404911908 | 0.6595813911 | 0.9584622976 |
| rest | 0.04715643837 | 0.1071174749 | 0.4402310493 | 0.6597697738 | 1.048285988 |
| stk11 | -0.04111936159 | 0.09370528344 | -0.4388158285 | 0.6607949902 | 0.9597145701 |
| lysmd3 | -0.04594910755 | 0.1047513843 | -0.438649168 | 0.6609157646 | 0.9550905679 |
| folh1 | -0.04728421395 | 0.1082462213 | -0.4368209198 | 0.6622412262 | 0.9538162712 |
| epha1 | 0.04487151716 | 0.102932767 | 0.4359303503 | 0.6628872639 | 1.045893472 |
| syngap1 | 0.04561592184 | 0.1047233711 | 0.435584926 | 0.6631379095 | 1.04667233 |
| scg3 | 0.04014869328 | 0.09221570936 | 0.4353780236 | 0.6632880594 | 1.040965547 |
| dusp3 | -0.03972682084 | 0.09157297079 | -0.4338269306 | 0.6644141238 | 0.9610519427 |
| adh1b | 0.04978343208 | 0.1148039917 | 0.4336385116 | 0.6645509641 | 1.051043449 |
| pcdh9 | -0.04385229849 | 0.1011681381 | -0.4334595783 | 0.6646809257 | 0.9570953115 |
| hspa1a | -0.04268744473 | 0.09854636476 | -0.4331711762 | 0.6648904173 | 0.9582108371 |
| dner | 0.04138360796 | 0.09579597145 | 0.4319973725 | 0.6657433228 | 1.042251845 |
| pdp1 | -0.04002237385 | 0.09264843765 | -0.4319810982 | 0.665755151 | 0.9607679428 |
| pkd2 | -0.04729432644 | 0.1095383402 | -0.4317604809 | 0.6659155048 | 0.9538066258 |
| tbca | -0.04622107699 | 0.1071849839 | -0.4312271674 | 0.6663032019 | 0.9548308477 |
| gask1a | 0.04968899759 | 0.1154200067 | 0.4305059324 | 0.6668276523 | 1.050944199 |
| slc44a4 | -0.04441252475 | 0.1032194344 | -0.4302728941 | 0.6669971422 | 0.9565592717 |
| ighmbp2 | -0.0453082437 | 0.1058063194 | -0.4282186922 | 0.6684919086 | 0.955702847 |
| chga | 0.04295128948 | 0.1006555566 | 0.4267155328 | 0.6695865356 | 1.043887045 |
| mtss2 | -0.04455615089 | 0.1047184997 | -0.4254850004 | 0.6704831538 | 0.9564218946 |
| iglon5 | 0.04453880426 | 0.1047758397 | 0.4250865887 | 0.6707735541 | 1.045545548 |
| maea | -0.04005261528 | 0.09424510908 | -0.4249834891 | 0.670848711 | 0.9607388883 |
| alpi | 0.04395750813 | 0.1034463155 | 0.4249306311 | 0.6708872443 | 1.044937953 |
| flt3lg | 0.04161908611 | 0.09801785703 | 0.4246071825 | 0.6711230562 | 1.042497301 |
| tbc1d23 | -0.03898855782 | 0.09196981597 | -0.4239277572 | 0.6716185003 | 0.9617617137 |
| smad5 | -0.04003631025 | 0.09464231728 | -0.4230275779 | 0.6722751403 | 0.9607545533 |
| abl1 | 0.0400043317 | 0.09474232692 | 0.4222434998 | 0.6728472935 | 1.040815283 |
| pnma2 | -0.04517427389 | 0.1071112755 | -0.4217508724 | 0.6732068679 | 0.9558308909 |

| Protein | beta | se | z | p_val | HR |
|---|---|---|---|---|---|
| ca13 | -0.03935992626 | 0.09333744305 | -0.4216949273 | 0.6732477076 | 0.9614046121 |
| col9a2 | -0.04480431784 | 0.1062486785 | -0.4216929423 | 0.6732491566 | 0.9561845718 |
| stx4 | 0.04185482641 | 0.09932699635 | 0.4213841952 | 0.6734745582 | 1.042743089 |
| ifngr2 | 0.04167743551 | 0.09941118063 | 0.4192429387 | 0.675038594 | 1.042558132 |
| il32 | -0.03636011607 | 0.08676400117 | -0.4190691483 | 0.6751655973 | 0.9642929736 |
| impact | -0.04407016246 | 0.1054779814 | -0.4178138589 | 0.6760832173 | 0.9568868176 |
| mapkapk2 | -0.04273606013 | 0.1024595787 | -0.4171016578 | 0.6766040524 | 0.9581642544 |
| hddc2 | -0.04389533646 | 0.1053196934 | -0.4167818483 | 0.6768379806 | 0.9570541209 |
| armcx2 | -0.0427618277 | 0.1026377727 | -0.4166285626 | 0.6769501141 | 0.9581395652 |
| necap2 | 0.04404265527 | 0.1057545899 | 0.4164609339 | 0.6770727482 | 1.04502693 |
| tsc22d1 | -0.04175314033 | 0.1004247469 | -0.4157654525 | 0.6775816412 | 0.9591065161 |
| grk5 | -0.03875931895 | 0.09329173782 | -0.4154635754 | 0.6778025745 | 0.9619822122 |
| stip1 | -0.04018490735 | 0.09708305362 | -0.413922985 | 0.6789305101 | 0.9606117985 |
| map1lc3b2 | -0.04216885747 | 0.1018788885 | -0.4139116368 | 0.6789388213 | 0.9587078819 |
| scgb3a1 | -0.04468387971 | 0.108360147 | -0.4123645175 | 0.6800722664 | 0.9562997398 |
| defb4a_defb4b | -0.04356677248 | 0.1056875359 | -0.4122224265 | 0.6801764009 | 0.9573686261 |
| ctf1 | -0.03865494896 | 0.0938001332 | -0.4120990839 | 0.6802668001 | 0.9620826195 |
| kir2dl3 | 0.03612796212 | 0.08786721199 | 0.4111654542 | 0.6809512175 | 1.036788508 |
| col5a1 | -0.0426516433 | 0.1038624728 | -0.4106549954 | 0.6813255315 | 0.9582451431 |
| rap1a | -0.04215132968 | 0.1026630144 | -0.4105795056 | 0.681380894 | 0.9587246861 |
| echdc3 | 0.0443361422 | 0.1084035898 | 0.4089914577 | 0.6825459301 | 1.045333677 |
| cfi | 0.04851893374 | 0.118643294 | 0.4089479657 | 0.6825778476 | 1.049715247 |
| cd5l | -0.04266046699 | 0.1043580293 | -0.4087895037 | 0.6826941434 | 0.9582366878 |
| sdk2 | 0.04464496546 | 0.109358373 | 0.4082446019 | 0.683094106 | 1.04565655 |
| nphs2 | -0.04227109647 | 0.1035715566 | -0.4081342201 | 0.6831751381 | 0.9586098696 |
| fam20a | 0.04819817883 | 0.1181954813 | 0.4077835995 | 0.6834325553 | 1.049378599 |
| mmut | 0.04258114758 | 0.104490429 | 0.4075124201 | 0.6836316739 | 1.04350073 |
| cyb5a | -0.0453897997 | 0.1115042809 | -0.4070677764 | 0.6839582096 | 0.9556249069 |
| oxt | 0.04223428544 | 0.1038343759 | 0.4067466584 | 0.6841940678 | 1.043138842 |
| f2 | -0.04375307564 | 0.1078809965 | -0.4055679595 | 0.6850600749 | 0.9571902819 |
| gpihbp1 | 0.04123929591 | 0.1017101162 | 0.4054591368 | 0.6851400495 | 1.042101446 |
| nhlrc3 | 0.0474658778 | 0.1170703769 | 0.4054473819 | 0.6851486884 | 1.04861042 |
| atp5if1 | -0.03769318027 | 0.09323359773 | -0.4042875228 | 0.6860012996 | 0.9630083655 |
| il36g | -0.0430724293 | 0.1066393993 | -0.403907276 | 0.6862809057 | 0.9578420117 |
| bax | -0.03654781017 | 0.09103185719 | -0.4014837365 | 0.6880640097 | 0.9641119984 |
| il20ra | -0.03952347544 | 0.09855013263 | -0.4010494394 | 0.6883837248 | 0.961247388 |
| abraxas2 | -0.04197366378 | 0.1051123314 | -0.3993219749 | 0.6896559772 | 0.9588950339 |
| hdac8 | -0.04213780795 | 0.1056024494 | -0.399023017 | 0.6898762446 | 0.9587376498 |
| ptprz1 | 0.04684133357 | 0.1174784057 | 0.3987229253 | 0.6900973738 | 1.04795572 |
| lgals8 | -0.03782652323 | 0.09489713627 | -0.3986055293 | 0.6901838868 | 0.9628799637 |
| carhsp1 | -0.0399828211 | 0.1004188676 | -0.398160446 | 0.6905119203 | 0.9608059446 |
| cd3d | -0.04102497599 | 0.1031318548 | -0.3977915075 | 0.6907838778 | 0.9598051576 |
| suox | -0.04478081248 | 0.1127004863 | -0.3973435603 | 0.6911141292 | 0.9562070475 |
| igf2r | 0.03908619127 | 0.09863000035 | 0.3962910994 | 0.6918902926 | 1.039860107 |
| atp6v1g2 | -0.04120793847 | 0.1040813938 | -0.3959203173 | 0.6921638122 | 0.9596295653 |
| cfhr4 | 0.04355278294 | 0.1101213978 | 0.3954979124 | 0.692475462 | 1.044515125 |
| kirrel1 | 0.04207232374 | 0.1064580176 | 0.395201082 | 0.6926944942 | 1.042969908 |
| atp5f1d | 0.04237187923 | 0.1072193499 | 0.3951887349 | 0.6927036058 | 1.043282382 |
| stau1 | 0.0431934003 | 0.1094735465 | 0.3945555951 | 0.69317089 | 1.044139812 |
| slc28a1 | -0.04269850456 | 0.1084974527 | -0.3935438437 | 0.6939178482 | 0.9582002395 |
| galnt5 | 0.03875774783 | 0.0984861954 | 0.3935348266 | 0.6939245068 | 1.039518627 |
| ak1 | -0.03925339164 | 0.09980763685 | -0.3932904624 | 0.6941049618 | 0.9615070404 |
| pdia2 | 0.04270252602 | 0.1086167487 | 0.3931486306 | 0.6942097079 | 1.043627397 |
| snca | -0.04302822454 | 0.1094983021 | -0.3929579156 | 0.6943505646 | 0.9578843538 |
| prkab1 | -0.03678998159 | 0.09364025051 | -0.3928864071 | 0.6944033815 | 0.9638785463 |
| esam | 0.03547999779 | 0.09038336597 | 0.3925500828 | 0.6946518139 | 1.036116923 |
| prune2 | -0.04119453798 | 0.1049769891 | -0.3924149314 | 0.6947516553 | 0.9596424249 |
| tp53inp1 | 0.03730404093 | 0.09513084562 | 0.3921340201 | 0.694959192 | 1.03800857 |
| fbn2 | 0.04217464507 | 0.1076397132 | 0.3918130568 | 0.695196347 | 1.043076631 |
| ddah1 | 0.03719027807 | 0.09506712486 | 0.3912001979 | 0.695649262 | 1.03789049 |
| acrbp | -0.04145834758 | 0.106096 | -0.3907625883 | 0.6959727308 | 0.9593892954 |
| slc12a2 | 0.04158222121 | 0.1065270511 | 0.3903442439 | 0.696282011 | 1.042458871 |
| gatd3 | -0.0400183815 | 0.1027819011 | -0.3893524158 | 0.697015467 | 0.9607717786 |
| znf830 | -0.04042847082 | 0.103987183 | -0.3887832104 | 0.6974365219 | 0.9603778571 |
| rnf168 | -0.04119728171 | 0.1059971647 | -0.388663997 | 0.6975247187 | 0.9596397919 |
| tmod4 | 0.04271282829 | 0.1100451759 | 0.3881390343 | 0.6979131467 | 1.043638148 |
| larp1 | -0.04139820523 | 0.1066879228 | -0.3880308486 | 0.6979932049 | 0.9594469971 |
| tcn1 | -0.04150372541 | 0.1071456867 | -0.387357874 | 0.6984912858 | 0.9593457614 |
| st3gal1 | 0.03826474458 | 0.09881884971 | 0.387221109 | 0.698592524 | 1.039006268 |
| cd164 | -0.03783057673 | 0.0980511213 | -0.3858250291 | 0.6996262573 | 0.9628760607 |

| Protein | beta | se | z | p_val | HR |
|---|---|---|---|---|---|
| epcam | -0.04019426163 | 0.1043422018 | -0.3852157701 | 0.7000775604 | 0.9606028128 |
| tbcc | -0.03636388192 | 0.09445663079 | -0.3849796633 | 0.7002524828 | 0.9642893422 |
| vtcn1 | -0.0356991002 | 0.09312243453 | -0.3833566033 | 0.7014553755 | 0.9649305972 |
| lsm1 | -0.03618295553 | 0.09463089029 | -0.3823588198 | 0.702195231 | 0.9644638233 |
| lto1 | 0.03734194985 | 0.09795911767 | 0.3811993282 | 0.7030553476 | 1.03804792 |
| camkk1 | -0.03637024714 | 0.09585033394 | -0.3794483091 | 0.7043549825 | 0.9642832043 |
| sult2a1 | 0.03661013669 | 0.09671436795 | 0.3785387577 | 0.7050304075 | 1.037288541 |
| igsf21 | 0.04199088147 | 0.1111066683 | 0.3779330451 | 0.7054803334 | 1.042884969 |
| efcab2 | 0.04027942364 | 0.1067545628 | 0.3773086845 | 0.7059442191 | 1.041101642 |
| usp8 | -0.03642519487 | 0.09663329158 | -0.3769425037 | 0.7062163338 | 0.9642302206 |
| ranbp2 | -0.03829455323 | 0.1019746091 | -0.3755302774 | 0.7072661332 | 0.9624294124 |
| l3hypdh | -0.03804455419 | 0.1016619426 | -0.3742261187 | 0.7082360935 | 0.962670049 |
| acsl1 | -0.03969997835 | 0.1062665169 | -0.3735887796 | 0.708710283 | 0.96107774 |
| plpbp | -0.03561895824 | 0.09546956343 | -0.373092292 | 0.7090797553 | 0.9650079318 |
| cwc15 | -0.04179877003 | 0.1120459879 | -0.3730501273 | 0.7091111362 | 0.9590627533 |
| metap2 | -0.03547907852 | 0.09513862694 | -0.3729198084 | 0.7092081288 | 0.9651429262 |
| ssna1 | -0.03861476124 | 0.1036936841 | -0.3723926059 | 0.7096005584 | 0.9621212842 |
| bnip3l | -0.03826376614 | 0.1029316435 | -0.3717395822 | 0.7100867513 | 0.9624590433 |
| scarf1 | 0.03498847796 | 0.09415644888 | 0.3715993793 | 0.7101911513 | 1.035607776 |
| fgf9 | 0.03980922868 | 0.1072000526 | 0.371354563 | 0.7103734632 | 1.040612236 |
| pms1 | -0.03895277554 | 0.1052540355 | -0.3700834399 | 0.7113203206 | 0.9617961284 |
| lyzl2 | -0.04375571613 | 0.118260733 | -0.3699936152 | 0.7113872477 | 0.9571877544 |
| akr7l | 0.0416684319 | 0.1126621182 | 0.3698530842 | 0.7114919598 | 1.042548746 |
| aspscr1 | -0.03752704011 | 0.1015985584 | -0.3693658721 | 0.7118550324 | 0.9631683732 |
| reg3g | 0.03850848873 | 0.1044591987 | 0.3686462197 | 0.7123914399 | 1.03925955 |
| gbp4 | -0.0351970224 | 0.095520928 | -0.3684744603 | 0.7125194853 | 0.9654151891 |
| aif1l | -0.0398687186 | 0.1082326245 | -0.3683613772 | 0.7126037924 | 0.9609155812 |
| izumo1 | 0.03886329527 | 0.105810198 | 0.3672925294 | 0.713400826 | 1.039628352 |
| dapp1 | -0.03364398222 | 0.09244874826 | -0.3639203651 | 0.7159174768 | 0.9669156825 |
| cpvl | -0.0350147824 | 0.09655983846 | -0.3626226282 | 0.7168868044 | 0.9655911424 |
| lamp3 | -0.0363439253 | 0.100296866 | -0.3623635189 | 0.7170803974 | 0.9643085863 |
| gipc3 | -0.03645408694 | 0.1010674232 | -0.3606907725 | 0.7183306235 | 0.9642023624 |
| flt4 | 0.03447095447 | 0.09575111334 | 0.3600057823 | 0.7188428094 | 1.035071964 |
| pfdn6 | 0.03790115744 | 0.105364144 | 0.3597158956 | 0.7190596037 | 1.038628567 |
| cst6 | -0.03399626348 | 0.09461965639 | -0.3592938801 | 0.7193752521 | 0.9665751163 |
| gabarapl1 | -0.03762079726 | 0.1048411232 | -0.3588362667 | 0.7197175801 | 0.9630780735 |
| cxcl10 | -0.03747778931 | 0.1044539636 | -0.3587971966 | 0.71974681 | 0.9632158112 |
| mri1 | 0.03653157601 | 0.1019124984 | 0.3584602142 | 0.7199989368 | 1.037207054 |
| znf174 | -0.03675174733 | 0.1028124884 | -0.3574638441 | 0.7207445887 | 0.9639154002 |
| dgcr6 | -0.03910109009 | 0.1094606257 | -0.3572160296 | 0.7209300866 | 0.9616534906 |
| pcdhb15 | 0.03850180762 | 0.1079573918 | 0.35663892 | 0.721362137 | 1.039252607 |
| casp8 | -0.034421005 | 0.09676836345 | -0.3557051476 | 0.7220613896 | 0.9661646588 |
| cd300a | 0.03933100055 | 0.1105786585 | 0.3556834662 | 0.7220776284 | 1.040114705 |
| gm2a | 0.03893788567 | 0.109626477 | 0.3551868738 | 0.7224495976 | 1.039705901 |
| sf3b4 | 0.03468298798 | 0.09773652602 | 0.3548620909 | 0.7226929097 | 1.035291457 |
| itih5 | -0.03729567807 | 0.105395208 | -0.3538650264 | 0.7234400382 | 0.9633912396 |
| extl1 | 0.03693173646 | 0.104536189 | 0.3532913988 | 0.7238699931 | 1.037622187 |
| mgmt | -0.0337900849 | 0.0956543399 | -0.3532519793 | 0.7238995426 | 0.9667744239 |
| ppp3r1 | -0.03326377088 | 0.09423998046 | -0.3529687794 | 0.7241118464 | 0.9672833848 |
| lefty2 | -0.03395851483 | 0.09650797361 | -0.3518726335 | 0.7249337836 | 0.9666116038 |
| fos | -0.03613558393 | 0.1028147124 | -0.3514631621 | 0.7252409042 | 0.9645095126 |
| lta4h | -0.03345629049 | 0.09532059249 | -0.3509870178 | 0.7255980879 | 0.9670971817 |
| nek7 | -0.0362542583 | 0.1034400549 | -0.3504856831 | 0.7259742329 | 0.9643950569 |
| dnph1 | -0.03418082368 | 0.09771194271 | -0.349812139 | 0.7264796883 | 0.9663967414 |
| rgs8 | 0.03341913149 | 0.09554492522 | 0.3497740086 | 0.7265083066 | 1.033983824 |
| kir3dl1 | -0.03032739774 | 0.08693698011 | -0.3488434691 | 0.7272068272 | 0.9701278639 |
| cdcp1 | -0.03724416263 | 0.1070254839 | -0.3479934057 | 0.7278451358 | 0.9634408704 |
| proc | -0.03365610124 | 0.09699589988 | -0.3469847827 | 0.728602751 | 0.9669039645 |
| cd33 | 0.03096496751 | 0.08940683091 | 0.3463378267 | 0.729088844 | 1.031449369 |
| il18rap | -0.03352770186 | 0.09708398358 | -0.3453474057 | 0.7298332117 | 0.9670281224 |
| tgoln2 | -0.03477470883 | 0.1011978736 | -0.3436308253 | 0.7311239397 | 0.9658229831 |
| kiaa1549l | 0.03662209424 | 0.1068239912 | 0.3428264928 | 0.7317289941 | 1.037300945 |
| cox5b | 0.03339043831 | 0.09747925598 | 0.3425389122 | 0.7319453654 | 1.033954156 |
| sgsh | -0.0368659898 | 0.1076854021 | -0.3423490008 | 0.7320882636 | 0.9638052864 |
| osmr | 0.03408862895 | 0.09983791375 | 0.3414397164 | 0.7327725802 | 1.034676305 |
| steap4 | 0.03581931512 | 0.1049378467 | 0.3413383852 | 0.732848854 | 1.036468555 |
| amdhd2 | -0.03421386975 | 0.1003476464 | -0.3409533854 | 0.7331386743 | 0.9663648063 |
| prrt3 | -0.03386986615 | 0.09937591002 | -0.3408257206 | 0.7332347863 | 0.9666972965 |
| siglec8 | 0.03750192984 | 0.110075703 | 0.3406921675 | 0.7333353358 | 1.038214001 |
| gnly | -0.03223161454 | 0.09496407039 | -0.3394085195 | 0.7343020015 | 0.9682822879 |

| Protein | beta | se | z | p_val | HR |
| --- | --- | --- | --- | --- | --- |
| angptl2 | -0.03446141281 | 0.1015689404 | -0.3392908568 | 0.7343906298 | 0.966125619 |
| gp1bb | 0.03539990404 | 0.1044611909 | 0.3388809158 | 0.7346994417 | 1.03603394 |
| ghrl | 0.03328336511 | 0.09848344739 | 0.3379589768 | 0.7353941025 | 1.033843453 |
| calca | 0.04007002678 | 0.1187869756 | 0.3373267698 | 0.7358705817 | 1.040883661 |
| ntf4 | -0.03101981641 | 0.09210670822 | -0.3367812944 | 0.7362817751 | 0.9694563617 |
| enpp5 | -0.03741471114 | 0.1111697879 | -0.3365546688 | 0.7364526335 | 0.963276571 |
| siglec5 | -0.03089192369 | 0.09184996706 | -0.3363302642 | 0.7366218303 | 0.9695803561 |
| myl1 | 0.03682909339 | 0.1100660486 | 0.3346090266 | 0.7379200348 | 1.037515687 |
| vgf | -0.03417333768 | 0.1022959086 | -0.3340635824 | 0.73833158 | 0.9664039759 |
| ifngr1 | 0.03015573815 | 0.09056832703 | 0.3329611922 | 0.7391635774 | 1.030615028 |
| fetub | 0.03301807293 | 0.09996334161 | 0.3303018126 | 0.7411719235 | 1.033569219 |
| mrpl58 | 0.0350125824 | 0.1062152892 | 0.3296378767 | 0.7416735993 | 1.035632739 |
| ahnak | 0.03803318112 | 0.1156403667 | 0.3288919103 | 0.7422373892 | 1.0387657 |
| kctd5 | -0.03506554034 | 0.1070320191 | -0.3276172926 | 0.7432010453 | 0.9655421322 |
| btnl10 | 0.03487709399 | 0.1067783715 | 0.3266306976 | 0.7439472226 | 1.035492433 |
| entpd6 | -0.03104473916 | 0.09560735639 | -0.3247107789 | 0.745399976 | 0.9694322005 |
| txndc15 | -0.02859827464 | 0.08807796437 | -0.3246927293 | 0.745413638 | 0.9718067855 |
| atp2b4 | -0.03520590886 | 0.1084909232 | -0.3245055698 | 0.7455553061 | 0.96540661 |
| cinp | 0.03460384874 | 0.1067009666 | 0.3243067971 | 0.7457057742 | 1.035209528 |
| mapt | 0.03205017343 | 0.09886950458 | 0.3241664209 | 0.7458120428 | 1.032569312 |
| amigo2 | 0.02963688014 | 0.09150292483 | 0.3238899761 | 0.7460213332 | 1.030080423 |
| ca8 | -0.03311284197 | 0.1025592307 | -0.3228655454 | 0.7467970708 | 0.9674293868 |
| ogt | 0.03502478022 | 0.1090269973 | 0.3212486913 | 0.7480219356 | 1.035645372 |
| taldo1 | -0.03551629721 | 0.1105728505 | -0.3212026916 | 0.7480567925 | 0.9651070056 |
| ca5a | 0.0332339627 | 0.1034950853 | 0.3211163371 | 0.7481222302 | 1.03379238 |
| swap70 | 0.03357147223 | 0.1046357618 | 0.3208412846 | 0.7483306714 | 1.034141353 |
| scrib | -0.03648022092 | 0.1138903234 | -0.3203101004 | 0.7487332672 | 0.9641771642 |
| sh3gl3 | 0.03352108204 | 0.1046622232 | 0.320278712 | 0.7487570593 | 1.034089244 |
| ncln | -0.02989256721 | 0.09352819143 | -0.3196102347 | 0.7492638149 | 0.9705497968 |
| cdhr1 | -0.03080525826 | 0.09652396284 | -0.3191462239 | 0.7496156333 | 0.9696643888 |
| shd | 0.0330221922 | 0.1035672525 | 0.3188478153 | 0.7498419178 | 1.033573476 |
| lrp2 | -0.03353472244 | 0.1052371348 | -0.3186586418 | 0.7499853799 | 0.9670213333 |
| paip2b | 0.03363170061 | 0.1056546279 | 0.3183173447 | 0.7502442289 | 1.03420364 |
| ltb | -0.03362234037 | 0.1059598676 | -0.317312027 | 0.7510068524 | 0.9669366086 |
| cpped1 | 0.03167410941 | 0.09986978265 | 0.3171540838 | 0.7511266885 | 1.032181072 |
| skiv2l | -0.03476694414 | 0.1096797277 | -0.3169860545 | 0.7512541839 | 0.9658304825 |
| susd1 | -0.02939634168 | 0.09278377421 | -0.3168263194 | 0.7513753922 | 0.9710315279 |
| psapl1 | 0.033150569 | 0.1046836003 | 0.3166739479 | 0.7514910187 | 1.033706172 |
| atxn3 | 0.03456065458 | 0.1092937799 | 0.316217946 | 0.7518370872 | 1.035164814 |
| col3a1 | 0.0326437955 | 0.1033222748 | 0.3159415098 | 0.7520469041 | 1.033182449 |
| itprip | 0.03223068759 | 0.1020830355 | 0.3157301058 | 0.7522073736 | 1.032755722 |
| nptx2 | -0.037418318 | 0.118606895 | -0.3154818107 | 0.7523958595 | 0.9632730966 |
| amigo1 | -0.03265935282 | 0.104015025 | -0.3139868767 | 0.753531006 | 0.967868205 |
| plin1 | 0.03392340453 | 0.1080713987 | 0.3138980799 | 0.7535984487 | 1.034505365 |
| hk2 | -0.03006968573 | 0.09589078011 | -0.3135826583 | 0.7538380323 | 0.9703779097 |
| pqbp1 | -0.03034072701 | 0.09676475565 | -0.3135514249 | 0.7538617575 | 0.9701149329 |
| pnma1 | 0.03301098257 | 0.1055590923 | 0.312725146 | 0.7544894893 | 1.03356189 |
| ftcd | 0.0335765696 | 0.1079639364 | 0.3109980121 | 0.7558021329 | 1.034146625 |
| crygd | -0.03308014888 | 0.106428024 | -0.3108217895 | 0.755936104 | 0.9674610156 |
| tp73 | 0.03327245978 | 0.1072765614 | 0.3101559124 | 0.7564423952 | 1.033832179 |
| nudt2 | 0.02880780143 | 0.09347423462 | 0.3081897546 | 0.757937948 | 1.02922676 |
| clec4a | -0.03057881117 | 0.09938290434 | -0.3076868338 | 0.7583206392 | 0.9698839914 |
| smtn | -0.03200869129 | 0.1043542757 | -0.3067309995 | 0.7590481323 | 0.9684981645 |
| morf4l2 | -0.03269150912 | 0.1068319042 | -0.3060088591 | 0.7595979005 | 0.9678370825 |
| casc3 | -0.03241983927 | 0.1060829433 | -0.3056084065 | 0.7599028191 | 0.9681000503 |
| fgf19 | 0.02851624447 | 0.09356119319 | 0.3047870971 | 0.7605283093 | 1.028926725 |
| actn4 | -0.02940991186 | 0.096567144 | -0.3045540195 | 0.7607058443 | 0.9710183509 |
| ninj1 | -0.02969055784 | 0.09753676137 | -0.3044037697 | 0.7608202962 | 0.9707458768 |
| guk1 | 0.03218778881 | 0.1057543169 | 0.3043638289 | 0.7608507217 | 1.032711419 |
| pdia3 | 0.03367334116 | 0.1110900673 | 0.3031174792 | 0.761800334 | 1.034246706 |
| atf4 | -0.03134707851 | 0.1035732663 | -0.3026560775 | 0.7621519738 | 0.9691391473 |
| sag | -0.03220727486 | 0.1064177245 | -0.3026495352 | 0.7621569601 | 0.9683058558 |
| il2 | 0.02902168327 | 0.09598525673 | 0.3023556352 | 0.7623809709 | 1.029446916 |
| mcee | -0.03268310661 | 0.1081041083 | -0.3023299219 | 0.7624005705 | 0.9678452147 |
| nudt16 | 0.031304179 | 0.1036487916 | 0.3020216494 | 0.7626355593 | 1.031799308 |
| znrd2 | -0.03099362121 | 0.1026744752 | -0.3018629621 | 0.7627565314 | 0.9694817572 |
| ccl24 | -0.02917852946 | 0.09675063475 | -0.3015848892 | 0.7629685286 | 0.9712430535 |
| mrpl28 | -0.03126485454 | 0.1038172194 | -0.3011528792 | 0.7632979197 | 0.9692188371 |
| ccl26 | -0.02763291535 | 0.09186212849 | -0.3008085683 | 0.7635604743 | 0.9727453812 |
| paxx | -0.03217639018 | 0.1070211618 | -0.3006544655 | 0.7636779943 | 0.9683357621 |

| Protein | beta | se | z | p_val | HR |
|---|---|---|---|---|---|
| ndufb7 | -0.02989193416 | 0.09952130025 | -0.3003571505 | 0.7639047445 | 0.9705504112 |
| dag1 | 0.02927337798 | 0.09751811227 | 0.3001840099 | 0.7640368012 | 1.029706055 |
| ctbs | -0.03231498929 | 0.1078280121 | -0.2996901143 | 0.7644135398 | 0.9682015609 |
| mtif3 | 0.0295647642 | 0.09866773029 | 0.2996396503 | 0.7644520364 | 1.030006141 |
| gadd45gip1 | 0.03076496185 | 0.1027802581 | 0.2993275403 | 0.7646901432 | 1.031243094 |
| skap1 | -0.02851853348 | 0.09561605487 | -0.2982609303 | 0.765504021 | 0.9718842816 |
| inpp1 | 0.02834780061 | 0.09506264059 | 0.2982012748 | 0.7655495489 | 1.028753423 |
| cd63 | -0.02944242662 | 0.09916237782 | -0.2969112608 | 0.7665342588 | 0.970986779 |
| acrv1 | -0.04519596077 | 0.1524377254 | -0.2964880291 | 0.7668574076 | 0.9558101622 |
| bcl7a | 0.03156524174 | 0.106587108 | 0.2961450247 | 0.7671193305 | 1.032068707 |
| c1qtnf1 | 0.02723202127 | 0.09225255537 | 0.2951898857 | 0.767848828 | 1.027606202 |
| vcan | 0.02859445287 | 0.09705932178 | 0.294608002 | 0.7682933486 | 1.029007199 |
| fuca1 | -0.02612156338 | 0.08924814862 | -0.2926846526 | 0.7697632019 | 0.9742166533 |
| nmrk2 | -0.03086094317 | 0.1054784594 | -0.2925805263 | 0.7698428005 | 0.9696103946 |
| gad2 | 0.03067175948 | 0.1051558551 | 0.2916790459 | 0.7705320318 | 1.031146984 |
| nop56 | -0.0307709502 | 0.1055432561 | -0.2915482365 | 0.7706320579 | 0.9696976567 |
| blvrb | 0.02835546864 | 0.09748924546 | 0.2908574018 | 0.7711603818 | 1.028761312 |
| fes | -0.02732050953 | 0.09413850635 | -0.2902160931 | 0.7716509253 | 0.97304932 |
| myh7b | 0.03121474148 | 0.1078238325 | 0.2894976069 | 0.7722006109 | 1.03170703 |
| fhip2a | 0.03040438949 | 0.1052268205 | 0.2889414442 | 0.7726261878 | 1.030871323 |
| snrpb2 | 0.03226735917 | 0.1126150241 | 0.2865280136 | 0.7744737407 | 1.032793595 |
| sil1 | 0.03164830697 | 0.1104865614 | 0.2864448542 | 0.7745374245 | 1.03215444 |
| cxcl14 | -0.03208656645 | 0.1120983887 | -0.2862357507 | 0.7746975636 | 0.9684227455 |
| lamp1 | -0.03047231288 | 0.1064933822 | -0.2861427841 | 0.7747687639 | 0.9699872879 |
| klk1 | 0.02723874267 | 0.09546470156 | 0.2853278984 | 0.7753929409 | 1.027613109 |
| ccar2 | 0.03017083779 | 0.1062003173 | 0.2840936689 | 0.7763385987 | 1.03063059 |
| mansc1 | -0.02515596255 | 0.08896331142 | -0.2827678304 | 0.7773548159 | 0.9751578121 |
| bcam | 0.02641276412 | 0.09343926248 | 0.28267308 | 0.777427454 | 1.026764673 |
| hao1 | 0.02901378969 | 0.1026673834 | 0.2825998748 | 0.7774835763 | 1.02943879 |
| ephx2 | 0.02777888136 | 0.09847832121 | 0.2820811832 | 0.7778812613 | 1.028168312 |
| glp1r | 0.02931642736 | 0.1039838102 | 0.2819326133 | 0.7779951817 | 1.029750384 |
| cox6b1 | -0.0283050236 | 0.1005367252 | -0.2815391445 | 0.7782969087 | 0.9720918106 |
| tctn3 | -0.02970047748 | 0.1058699639 | -0.2805373346 | 0.779065286 | 0.9707362474 |
| adh4 | -0.03021465054 | 0.1080189085 | -0.2797163104 | 0.7796951638 | 0.9702372492 |
| pafah1b3 | 0.02895914274 | 0.1036229793 | 0.2794664169 | 0.7798869071 | 1.029382536 |
| nppc | 0.02726035323 | 0.09760620577 | 0.2792891396 | 0.7800229402 | 1.027635316 |
| fgr | 0.02732643008 | 0.0987604349 | 0.2766941044 | 0.7820149992 | 1.027703221 |
| lrp2bp | 0.02914034828 | 0.1054533517 | 0.2763340169 | 0.782291531 | 1.029569083 |
| klk14 | 0.0256557701 | 0.09287866511 | 0.276228885 | 0.782372273 | 1.025987712 |
| cr1 | -0.03028611036 | 0.1098848547 | -0.2756167848 | 0.7828424164 | 0.9701679187 |
| eif4ebp1 | -0.02725552981 | 0.09896312145 | -0.2754109754 | 0.7830005128 | 0.9731125505 |
| chmp1a | -0.02612584349 | 0.09492648548 | -0.2752218556 | 0.7831457967 | 0.9742124836 |
| septin9 | -0.02586152216 | 0.09412187663 | -0.2747663252 | 0.7834957713 | 0.9744700228 |
| adamts1 | -0.02901975719 | 0.1056304948 | -0.2747289714 | 0.7835244713 | 0.9713972722 |
| ppp1r12a | -0.02537975211 | 0.09255183864 | -0.2742220196 | 0.7839140073 | 0.9749396063 |
| crtac1 | -0.02677000359 | 0.09831573798 | -0.2722860464 | 0.7854020844 | 0.9735851369 |
| nmi | -0.02874434869 | 0.1056940976 | -0.2719579364 | 0.7856543626 | 0.9716648401 |
| lmnb1 | 0.02809047815 | 0.1034864529 | 0.2714411148 | 0.7860517835 | 1.028488736 |
| adgrf5 | -0.03073789011 | 0.1133467519 | -0.2711845695 | 0.7862490802 | 0.9697297155 |
| txndc9 | -0.02752896581 | 0.1015226401 | -0.2711608542 | 0.7862673192 | 0.9728465029 |
| pdap1 | -0.02779737473 | 0.1028440181 | -0.2702867433 | 0.7869396632 | 0.9725854172 |
| tet2 | -0.02796956459 | 0.1038679177 | -0.2692801128 | 0.7877141349 | 0.9724179623 |
| acp1 | 0.02780954054 | 0.103486115 | 0.268727264 | 0.7881395696 | 1.028199835 |
| kir2dl2 | 0.02667565375 | 0.0998908367 | 0.2670480559 | 0.7894321609 | 1.027034634 |
| nrtn | -0.02565941198 | 0.09631115504 | -0.2664220148 | 0.7899142123 | 0.974666993 |
| inpp5d | 0.02907561464 | 0.1094627241 | 0.2656211498 | 0.7905309953 | 1.029502437 |
| casp4 | 0.02870714656 | 0.1086997712 | 0.2640957404 | 0.7917061461 | 1.029123168 |
| myh9 | 0.02579254595 | 0.09780994737 | 0.2637006423 | 0.7920106005 | 1.026128052 |
| cdh4 | -0.02792079792 | 0.1060921461 | -0.2631749752 | 0.792415718 | 0.972465385 |
| jun | 0.02547566486 | 0.09709636956 | 0.2623750504 | 0.793032306 | 1.025802943 |
| rab6b | -0.02489130926 | 0.09500911864 | -0.2619886345 | 0.7933302046 | 0.9754159249 |
| ptn | -0.02702943383 | 0.103358983 | -0.2615102533 | 0.7936990436 | 0.9733325922 |
| tmprss5 | 0.02694356093 | 0.1031751213 | 0.2611439713 | 0.7939814836 | 1.027309821 |
| il20 | 0.02590262441 | 0.09925942015 | 0.2609588528 | 0.7941242387 | 1.026241013 |
| gnas | -0.02749184853 | 0.105741599 | -0.259990853 | 0.7948708293 | 0.9728826129 |
| notch1 | 0.0240502095 | 0.09267575136 | 0.2595091936 | 0.7952423895 | 1.024341748 |
| crnn | -0.02592466602 | 0.0999807019 | -0.2592966996 | 0.7954063257 | 0.9744084929 |
| erc2 | -0.0267021249 | 0.1030827972 | -0.2590357037 | 0.7956076928 | 0.9736512248 |
| tomm20 | -0.02686909604 | 0.1038477422 | -0.258735486 | 0.7958393378 | 0.9734886667 |
| enpp2 | 0.02815844891 | 0.1089089368 | 0.2585503975 | 0.7959821591 | 1.028558646 |

| Protein | beta | se | z | p_val | HR |
|---|---|---|---|---|---|
| clgn | 0.02727984481 | 0.1056130421 | 0.2582999626 | 0.7961754151 | 1.027655347 |
| traf3ip2 | 0.02707216613 | 0.1051292748 | 0.2575131063 | 0.796782699 | 1.027441947 |
| dnajb1 | 0.02500903057 | 0.09714364155 | 0.2574438241 | 0.7968361759 | 1.02532438 |
| clec4d | -0.02504582143 | 0.09770845849 | -0.2563321724 | 0.7976943569 | 0.975265223 |
| lgals3bp | -0.02859585769 | 0.1118561145 | -0.2556485876 | 0.7982221972 | 0.9718091343 |
| il31ra | 0.02799520231 | 0.1095654325 | 0.2555112654 | 0.7983282437 | 1.028390751 |
| icam4 | -0.02465077762 | 0.09675465624 | -0.2547761377 | 0.7988960066 | 0.9756505716 |
| dpp4 | -0.02564107345 | 0.1009364947 | -0.2540317406 | 0.7994710368 | 0.9746848671 |
| psmd5 | -0.02589612486 | 0.1028184696 | -0.2518625784 | 0.8011472854 | 0.9744363041 |
| pth | 0.02678341567 | 0.1067243149 | 0.2509588906 | 0.8018458928 | 1.027145315 |
| vash1 | -0.0232237483 | 0.09264327333 | -0.2506792718 | 0.8020620878 | 0.9770438474 |
| ahsg | -0.02640705337 | 0.1053983882 | -0.2505451348 | 0.8021658049 | 0.9739385639 |
| tnr | 0.02473362667 | 0.09927197533 | 0.2491501412 | 0.8032446449 | 1.02504204 |
| dctn1 | -0.02374786292 | 0.09531807601 | -0.2491433306 | 0.803249913 | 0.9765318986 |
| dnajb8 | 0.02329556567 | 0.09351775189 | 0.2491031403 | 0.8032810004 | 1.023569027 |
| sfrp1 | 0.02653209737 | 0.1068787613 | 0.248244806 | 0.8039450009 | 1.026887207 |
| agbl2 | 0.02607156147 | 0.1050397346 | 0.2482066579 | 0.8039745153 | 1.026414398 |
| clta | 0.02461433657 | 0.09948425202 | 0.2474194264 | 0.8045836413 | 1.02491977 |
| mpo | -0.02373742234 | 0.09633423463 | -0.2464069231 | 0.8053672476 | 0.9765420942 |
| cda | 0.02639207979 | 0.1072705906 | 0.2460327629 | 0.8056568707 | 1.026743435 |
| mmp13 | -0.02440427848 | 0.09942569332 | -0.2454524345 | 0.8061061336 | 0.9758910982 |
| notch3 | 0.02322030643 | 0.09483731448 | 0.2448435677 | 0.8065775582 | 1.023491997 |
| cxcl6 | -0.02388969257 | 0.09776888938 | -0.2443486136 | 0.8069608361 | 0.9763934073 |
| cxcl5 | -0.02354629812 | 0.09648636999 | -0.2440375581 | 0.807201732 | 0.9767287529 |
| serpind1 | -0.02626894032 | 0.1077240246 | -0.243854056 | 0.8073438531 | 0.9740730869 |
| il11 | 0.02412907157 | 0.098988825 | 0.2437555105 | 0.8074201786 | 1.024422533 |
| tdp1 | 0.02683880513 | 0.110142997 | 0.24367237 | 0.807484574 | 1.02720221 |
| bcl2l1 | -0.02492046672 | 0.102293668 | -0.2436169043 | 0.8075275349 | 0.9753874847 |
| cd1c | 0.02274516854 | 0.09362759393 | 0.2429323192 | 0.8080578283 | 1.023005812 |
| adcyap1r1 | -0.02401039092 | 0.09939268945 | -0.2415709953 | 0.8091125989 | 0.9762755653 |
| ctag1a_ctag1b | 0.02595879456 | 0.1077903832 | 0.2408266284 | 0.8096894904 | 1.026298659 |
| apbb1ip | 0.02501046591 | 0.1039868968 | 0.2405155522 | 0.8099306082 | 1.025325851 |
| apoh | -0.0224338699 | 0.09342213619 | -0.2401344136 | 0.8102260564 | 0.9778158981 |
| mmp9 | -0.02424482765 | 0.101280892 | -0.2393820509 | 0.8108093468 | 0.9760467173 |
| ecm1 | 0.02433200876 | 0.1017312278 | 0.2391793483 | 0.8109665157 | 1.024630448 |
| adamts15 | 0.02454891996 | 0.1026997407 | 0.2390358514 | 0.811077783 | 1.024852726 |
| cep164 | 0.02307440893 | 0.09710207656 | 0.2376304374 | 0.812167741 | 1.023342683 |
| nfic | -0.02519020364 | 0.1062607735 | -0.2370602321 | 0.8126100632 | 0.9751244222 |
| vti1a | -0.02493240885 | 0.1054547996 | -0.2364274452 | 0.8131010016 | 0.9753758366 |
| rcc1 | 0.02704608167 | 0.1149625599 | 0.2352599115 | 0.814007008 | 1.027415147 |
| txndc5 | 0.0225896557 | 0.09622083566 | 0.234768858 | 0.81438814 | 1.022846734 |
| nme1 | 0.02376154782 | 0.1013224864 | 0.2345140617 | 0.8145859179 | 1.024046103 |
| entpd5 | 0.02270887681 | 0.09692096764 | 0.2343030343 | 0.8147497304 | 1.022968686 |
| smpdl3b | -0.02423319886 | 0.1035164678 | -0.2340999396 | 0.8149073928 | 0.9760580676 |
| chac2 | -0.02262095525 | 0.09672429158 | -0.2338704671 | 0.8150855413 | 0.9776329802 |
| arntl | -0.0258497714 | 0.1106863091 | -0.23354082 | 0.8153414759 | 0.9744814736 |
| ca7 | -0.02448799024 | 0.1049537229 | -0.2333217875 | 0.8155115414 | 0.9758094081 |
| gdf2 | 0.02302651405 | 0.09894962785 | 0.2327094558 | 0.8159870261 | 1.023293671 |
| lyar | -0.02244546844 | 0.09653330981 | -0.2325152684 | 0.8161378296 | 0.9778045569 |
| myo9b | 0.02144921762 | 0.09228006668 | 0.2324360871 | 0.8161993228 | 1.021680906 |
| gigyf2 | 0.02361769903 | 0.1019534952 | 0.2316516857 | 0.8168085598 | 1.023898806 |
| pebp1 | 0.02266849997 | 0.09793738847 | 0.2314591018 | 0.8169581548 | 1.022927383 |
| scpep1 | -0.0263144858 | 0.1142863236 | -0.2302505232 | 0.8178971045 | 0.9740287232 |
| tpp1 | -0.02422199851 | 0.1052166834 | -0.2302106255 | 0.8179281056 | 0.9760689998 |
| slc1a4 | -0.02446272368 | 0.1068009726 | -0.229049634 | 0.818830339 | 0.9758340637 |
| gbp1 | 0.02445143603 | 0.1071960163 | 0.2281002305 | 0.8195683209 | 1.024752824 |
| cntf | 0.02424410435 | 0.1064638979 | 0.2277213668 | 0.8198628605 | 1.024540382 |
| aarsd1 | 0.02254158038 | 0.09900499957 | 0.227681233 | 0.8198940632 | 1.022797562 |
| psmc3 | 0.02488275865 | 0.109436667 | 0.2273713128 | 0.8201350252 | 1.025194918 |
| arhgap5 | 0.02434492563 | 0.1073713027 | 0.2267358692 | 0.8206291338 | 1.024643683 |
| nampt | 0.0205438165 | 0.09090588976 | 0.2259899392 | 0.8212092453 | 1.020756293 |
| csf3 | -0.02238274104 | 0.09928466265 | -0.2254400674 | 0.8216369445 | 0.977865894 |
| slc39a5 | -0.02271928958 | 0.1012209682 | -0.2244524034 | 0.8224052986 | 0.97753685 |
| add1 | 0.02431393146 | 0.1083305275 | 0.2244421035 | 0.8224133124 | 1.024611925 |
| pfkfb2 | 0.02088594867 | 0.09360376325 | 0.2231315061 | 0.8234331581 | 1.021105587 |
| olr1 | -0.02257455735 | 0.1012760979 | -0.2229011367 | 0.8236124517 | 0.9776783414 |
| pvalb | -0.02082118019 | 0.09398928618 | -0.2215271659 | 0.8246819867 | 0.979394084 |
| igbp1 | 0.02342364046 | 0.106431306 | 0.2200822421 | 0.8258071045 | 1.023700128 |
| gstm4 | 0.02359116884 | 0.1072710553 | 0.2199211035 | 0.8259326005 | 1.023871642 |
| arf6 | -0.0228442848 | 0.1039197765 | -0.219826154 | 0.8260065499 | 0.9774146702 |

| Protein | beta | se | z | p_val | HR |
|---|---|---|---|---|---|
| susd2 | -0.02109982273 | 0.0961088268 | -0.2195409457 | 0.8262286877 | 0.9791212211 |
| dnajc9 | -0.02373395542 | 0.108210446 | -0.2193314628 | 0.8263918547 | 0.9765454798 |
| klk7 | -0.02393078941 | 0.1095465376 | -0.2184531792 | 0.8270760349 | 0.9763532814 |
| drg2 | 0.02022046136 | 0.09256986194 | 0.2184346064 | 0.8270905044 | 1.02042628 |
| clec12a | -0.02290573541 | 0.1053236752 | -0.2174794543 | 0.8278347169 | 0.9773546094 |
| tef | 0.02327405747 | 0.107109123 | 0.2172929516 | 0.8279800497 | 1.023547012 |
| id4 | -0.02265026684 | 0.104335072 | -0.2170915916 | 0.8281369666 | 0.9776043246 |
| st6gal1 | 0.0205916164 | 0.09513151897 | 0.2164541954 | 0.8286337255 | 1.020805086 |
| slc13a1 | 0.02258920566 | 0.1047103374 | 0.2157304256 | 0.8291978834 | 1.022846274 |
| pgr | 0.02194332012 | 0.1027649776 | 0.2135291675 | 0.8309142413 | 1.022185845 |
| apex1 | 0.0204700359 | 0.09632812839 | 0.2125032038 | 0.8317144786 | 1.020680984 |
| il13ra1 | -0.02077730577 | 0.09802228324 | -0.2119651275 | 0.8321342403 | 0.9794370553 |
| pts | 0.02067596418 | 0.09773467229 | 0.2115519876 | 0.8324565698 | 1.020891193 |
| nptn | -0.02061116532 | 0.09764343007 | -0.2110860434 | 0.8328201306 | 0.9795997929 |
| arhgap25 | -0.02037026423 | 0.09661550652 | -0.2108384561 | 0.8330133294 | 0.979835808 |
| ggct | -0.02200692446 | 0.1044575339 | -0.2106781928 | 0.8331383923 | 0.9782334613 |
| serpina11 | -0.02084581033 | 0.09897835667 | -0.2106097842 | 0.8331917769 | 0.9793699617 |
| mdh1 | 0.02302884734 | 0.1096660817 | 0.2099906094 | 0.8336750022 | 1.023296058 |
| dbi | 0.02070709925 | 0.09877010438 | 0.209649462 | 0.8339412723 | 1.020922979 |
| ltbr | -0.01925520784 | 0.09190645231 | -0.2095087707 | 0.8340510893 | 0.9809289895 |
| adra2a | -0.0229242013 | 0.109952916 | -0.2084910717 | 0.8348455532 | 0.9773365618 |
| f11r | -0.01983571884 | 0.09526032694 | -0.208226441 | 0.8350521641 | 0.9803597147 |
| lgmn | -0.02044654501 | 0.09827678332 | -0.2080506129 | 0.8351894485 | 0.9797610682 |
| rapgef2 | -0.02158972728 | 0.1039717917 | -0.2076498531 | 0.8355023755 | 0.9786416627 |
| dtymk | -0.02156976913 | 0.1043303542 | -0.2067449046 | 0.8362090862 | 0.9786611948 |
| cd209 | -0.02077158927 | 0.1006837741 | -0.2063052311 | 0.8365524926 | 0.9794426542 |
| tbc1d5 | -0.01960649152 | 0.09581445074 | -0.2046297962 | 0.837861374 | 0.9805844657 |
| padi4 | -0.02005079182 | 0.09829332794 | -0.2039893474 | 0.8383618234 | 0.9801488885 |
| defb118 | 0.02173339842 | 0.1065518501 | 0.2039701647 | 0.8383768139 | 1.021971289 |
| ccdc80 | 0.02236776763 | 0.1099910392 | 0.2033599081 | 0.8388537337 | 1.022619802 |
| rhoc | -0.01835179075 | 0.09102663643 | -0.2016090177 | 0.8402223949 | 0.981815578 |
| htr1a | 0.02122786939 | 0.1053063885 | 0.2015819714 | 0.8402435406 | 1.021454783 |
| smpd3 | 0.02002574747 | 0.09941810439 | 0.2014295846 | 0.8403626841 | 1.020227608 |
| rln1 | 0.02169192157 | 0.1076922173 | 0.2014251551 | 0.8403661474 | 1.021928902 |
| hip1 | 0.02163504456 | 0.1075506496 | 0.2011614495 | 0.8405723342 | 1.021870779 |
| mtr | 0.02090041129 | 0.1041844922 | 0.200609619 | 0.8410038361 | 1.021120355 |
| lztfl1 | -0.02137311767 | 0.1068570954 | -0.200015896 | 0.8414681491 | 0.9788536688 |
| ogfr | -0.01941249944 | 0.09712424119 | -0.1998728557 | 0.8415800201 | 0.9807747098 |
| cdh5 | 0.01844088242 | 0.09233039621 | 0.1997271015 | 0.8416940169 | 1.018611966 |
| dusp29 | 0.02232158866 | 0.1125854626 | 0.198263507 | 0.8428389021 | 1.022572579 |
| thtpa | -0.02047811773 | 0.1036526209 | -0.1975648812 | 0.8433855139 | 0.979730135 |
| cst7 | 0.02034328972 | 0.1031993364 | 0.1971261679 | 0.8437288061 | 1.020551625 |
| lama4 | 0.01813307643 | 0.09204292782 | 0.1970067322 | 0.8438222695 | 1.018298479 |
| plcb1 | 0.02155807723 | 0.1094549323 | 0.1969584811 | 0.8438600286 | 1.021792131 |
| sirt1 | -0.02049677422 | 0.1041962069 | -0.1967132473 | 0.8440519428 | 0.9797118568 |
| tex33 | 0.02005254465 | 0.1025122006 | 0.1956112983 | 0.8449144164 | 1.020254948 |
| cdkl5 | -0.02047812186 | 0.1053920996 | -0.1943041455 | 0.8459377398 | 0.9797301309 |
| capn3 | 0.02001655078 | 0.1031000331 | 0.19414689 | 0.8460608672 | 1.020218225 |
| fcrlb | 0.01879313527 | 0.09702216774 | 0.1936993958 | 0.8464112649 | 1.018970838 |
| rab3gap1 | -0.02020234941 | 0.1048443692 | -0.1926889307 | 0.847202593 | 0.9800003508 |
| hspb1 | -0.01875022755 | 0.09733087322 | -0.1926441933 | 0.8472376319 | 0.9814244644 |
| dipk2b | -0.02107144615 | 0.1094274508 | -0.1925608793 | 0.8473028853 | 0.9791490056 |
| itga6 | -0.01790979107 | 0.0936670642 | -0.1912069223 | 0.8483634805 | 0.9822496361 |
| anxa1 | 0.02126639474 | 0.1112798532 | 0.1911073221 | 0.8484415112 | 1.021494136 |
| csnk2a1 | 0.01884335204 | 0.09887892602 | 0.1905699505 | 0.8488625347 | 1.019022008 |
| mst1 | -0.01905660676 | 0.1003063588 | -0.1899840347 | 0.8493216412 | 0.9811238224 |
| lonp1 | -0.01967485776 | 0.1036895118 | -0.1897478098 | 0.8495067545 | 0.9805174291 |
| trem2 | -0.01939803296 | 0.1023337268 | -0.1895565966 | 0.8496566013 | 0.9807888982 |
| dcdc2c | -0.02019839065 | 0.1066603085 | -0.1893712004 | 0.8498018948 | 0.9800042303 |
| pdzd2 | -0.01995199351 | 0.1054169439 | -0.1892674249 | 0.849883225 | 0.9802457303 |
| brd1 | 0.01953694396 | 0.1033867352 | 0.1889695415 | 0.850116689 | 1.019729039 |
| tmed10 | 0.0196029874 | 0.1040092037 | 0.1884735842 | 0.8505054212 | 1.019796388 |
| serpina6 | 0.02294732606 | 0.1219327721 | 0.1881965419 | 0.8507225832 | 1.023212641 |
| il17a | 0.01784370675 | 0.09508842162 | 0.1876538326 | 0.8511480235 | 1.018003857 |
| jam3 | 0.01848529961 | 0.09855604746 | 0.1875612922 | 0.851220572 | 1.01865721 |
| eri1 | -0.02035982109 | 0.1092319354 | -0.1863907383 | 0.8521383549 | 0.9798460406 |
| ern1 | 0.02055066381 | 0.1112655419 | 0.184699265 | 0.8534649227 | 1.020763283 |
| nrxn3 | -0.01912513195 | 0.1036345323 | -0.184544008 | 0.8535867066 | 0.981056593 |
| kdr | -0.01789190001 | 0.09706047506 | -0.1843376513 | 0.8537485785 | 0.9822672097 |
| ugdh | 0.02034918225 | 0.1108250524 | 0.1836153632 | 0.8543152099 | 1.020557638 |

| Protein | beta | se | z | p_val | HR |
|---|---|---|---|---|---|
| abhd14b | -0.01792699068 | 0.09877413158 | -0.1814947942 | 0.8559792189 | 0.9822327419 |
| yy1 | -0.01844532229 | 0.1020666963 | -0.1807183239 | 0.8565886752 | 0.9817237515 |
| sez6 | 0.01895337051 | 0.1054360028 | 0.1797618461 | 0.857339538 | 1.019134126 |
| robo2 | -0.01798780349 | 0.10026269 | -0.1794067512 | 0.8576183307 | 0.9821730114 |
| gbp6 | 0.01976155474 | 0.1103595233 | 0.1790652419 | 0.8578864738 | 1.019958107 |
| dlk1 | 0.01688288275 | 0.09433104911 | 0.1789748223 | 0.8579574714 | 1.017026204 |
| ptprm | 0.01663998395 | 0.09298893589 | 0.1789458476 | 0.8579802226 | 1.0167792 |
| apob | -0.01836241197 | 0.1028823767 | -0.1784796635 | 0.858346291 | 0.9818051499 |
| mecr | -0.01775918859 | 0.09996025017 | -0.1776625065 | 0.8589880322 | 0.9823975764 |
| casq2 | 0.01928538938 | 0.1085751148 | 0.1776225557 | 0.8590194094 | 1.019472554 |
| acp6 | 0.01679590098 | 0.0946027779 | 0.1775413086 | 0.859083221 | 1.016937745 |
| alpp | 0.01799387098 | 0.1017272107 | 0.176883558 | 0.8595998538 | 1.018156736 |
| pttg1 | -0.0187480247 | 0.1073312481 | -0.1746744311 | 0.8613354599 | 0.9814266264 |
| clec10a | -0.01679005104 | 0.09623342113 | -0.1744721412 | 0.861494423 | 0.9833501163 |
| il20rb | 0.01775056371 | 0.1026336298 | 0.1729507545 | 0.8626901364 | 1.017909041 |
| alms1 | -0.0187048792 | 0.1084194122 | -0.1725233408 | 0.8630261132 | 0.9814689714 |
| kif1c | 0.01974471462 | 0.1145918493 | 0.1723047035 | 0.8631979868 | 1.019940931 |
| qsox1 | 0.0184175213 | 0.1073420403 | 0.1715778949 | 0.8637693871 | 1.01858817 |
| ccn1 | -0.01651734851 | 0.09639375294 | -0.1713528938 | 0.8639462922 | 0.9836183149 |
| cep20 | -0.01590865448 | 0.09307307728 | -0.1709264908 | 0.8642815666 | 0.9842172198 |
| rrm2 | -0.01615225465 | 0.09506656076 | -0.1699046912 | 0.8650850917 | 0.9839774935 |
| klk3 | -0.02726601535 | 0.160982901 | -0.1693721208 | 0.8655039511 | 0.9731023469 |
| cdc27 | -0.01588137174 | 0.09377621164 | -0.1693539487 | 0.8655182438 | 0.9842440723 |
| park7 | -0.01648124389 | 0.09736373262 | -0.1692749799 | 0.8655803551 | 0.9836538287 |
| lamtor5 | -0.01807951689 | 0.1070442199 | -0.1688976473 | 0.8658771498 | 0.9820829371 |
| serpina7 | 0.01821450743 | 0.1081955592 | 0.1683480132 | 0.8663095038 | 1.018381403 |
| map2k1 | -0.01736958668 | 0.1034551122 | -0.1678949092 | 0.8666659551 | 0.982780395 |
| tyro3 | 0.01621646699 | 0.09667779357 | 0.1677372476 | 0.866789992 | 1.016348668 |
| cenpf | 0.01797744609 | 0.1077420685 | 0.1668563295 | 0.8674830953 | 1.018140013 |
| cd2ap | 0.01620427926 | 0.09772303599 | 0.1658184183 | 0.8682998511 | 1.016336281 |
| ciapin1 | 0.01610433244 | 0.09739331512 | 0.165353571 | 0.8686656956 | 1.016234706 |
| cnpy4 | -0.01565616151 | 0.09542630373 | -0.1640654715 | 0.8696796032 | 0.9844657591 |
| il1b | 0.01607134522 | 0.09808198109 | 0.163856246 | 0.8698443121 | 1.016201184 |
| ifnw1 | -0.01738500936 | 0.106435604 | -0.1633382882 | 0.8702520891 | 0.982765238 |
| pi16 | 0.01696634104 | 0.1051710878 | 0.1613213421 | 0.8718403149 | 1.017111087 |
| smpd1 | 0.01553894847 | 0.09697455095 | 0.1602373852 | 0.8726940808 | 1.015660306 |
| itih1 | 0.01683974565 | 0.1052813632 | 0.1599499203 | 0.872920524 | 1.016982333 |
| nfu1 | -0.01597570912 | 0.1000109181 | -0.1597396506 | 0.8730861653 | 0.9841512257 |
| hdac9 | 0.01683588168 | 0.1054133745 | 0.1597129563 | 0.8731071942 | 1.016978404 |
| hmmr | -0.01636615416 | 0.102610216 | -0.1594982918 | 0.8732763041 | 0.9837670437 |
| prss2 | 0.01420325271 | 0.08917290637 | 0.1592776696 | 0.8734501133 | 1.014304598 |
| csh1 | 0.01620460413 | 0.1020838585 | 0.1587381626 | 0.8738751699 | 1.016336611 |
| pamr1 | 0.01586063438 | 0.1000644431 | 0.1585041987 | 0.8740595124 | 1.015987082 |
| c5 | -0.01743002605 | 0.11001836 | -0.1584283391 | 0.8741192844 | 0.9827209981 |
| adamts4 | 0.01694641719 | 0.1077229385 | 0.1573148433 | 0.8749967227 | 1.017090822 |
| cgn | -0.01633321998 | 0.1046489524 | -0.1560762875 | 0.8759728891 | 0.9837994438 |
| rp2 | -0.01428742716 | 0.09160451626 | -0.1559685891 | 0.8760577805 | 0.9858141538 |
| h2ap | 0.01673858707 | 0.1075023012 | 0.1557044536 | 0.8762659865 | 1.016879462 |
| txn | -0.01723448624 | 0.1111658034 | -0.1550340636 | 0.8767944631 | 0.982913178 |
| tg | 0.01644807835 | 0.1067122494 | 0.1541348668 | 0.8775033971 | 1.016584093 |
| ipcef1 | -0.01458120066 | 0.09463063834 | -0.1540854095 | 0.8775423924 | 0.9855245902 |
| flt3 | 0.01471454388 | 0.09571833239 | 0.1537275411 | 0.8778245683 | 1.014823336 |
| esyt2 | 0.0151686927 | 0.09888273482 | 0.1534008209 | 0.8780821975 | 1.015284321 |
| tp53bp1 | -0.01641009982 | 0.1073796507 | -0.1528231813 | 0.8785377164 | 0.9837238124 |
| dmd | 0.01689458839 | 0.1112436022 | 0.1518702025 | 0.8792893106 | 1.017038109 |
| nphs1 | -0.01572875928 | 0.1037505752 | -0.1516016586 | 0.8795011252 | 0.9843942917 |
| vnn1 | 0.01516085136 | 0.1008977393 | 0.1502595743 | 0.8805598258 | 1.01527636 |
| ruvbl1 | -0.01382629187 | 0.09230386813 | -0.1497910342 | 0.8809294832 | 0.9862688523 |
| sowaha | -0.01477336342 | 0.09900045786 | -0.149225203 | 0.8813759336 | 0.9853352273 |
| cope | 0.01402969562 | 0.09417388309 | 0.1489765013 | 0.8815721752 | 1.014128574 |
| dctn2 | -0.01338271766 | 0.08988595691 | -0.1488855225 | 0.8816439653 | 0.9867064328 |
| ptk7 | -0.01408052284 | 0.09469022774 | -0.1487009079 | 0.8817896448 | 0.9860181441 |
| inpp5j | 0.01588523323 | 0.1071578354 | 0.1482414532 | 0.8821522183 | 1.016012074 |
| aamdc | 0.01542955721 | 0.1041385836 | 0.1481636938 | 0.8822135836 | 1.015549207 |
| bex3 | -0.01628987381 | 0.1100831993 | -0.1479778378 | 0.8823602586 | 0.9838420887 |
| bhmt2 | -0.01581429728 | 0.1069372854 | -0.1478838481 | 0.8824344354 | 0.9843100921 |
| gngt1 | -0.01529514331 | 0.1040334868 | -0.1470213464 | 0.8831151714 | 0.9848212333 |
| fzd8 | 0.01541120936 | 0.1050741719 | 0.146669815 | 0.8833926451 | 1.015530574 |
| hyou1 | 0.0129113308 | 0.08813170759 | 0.1465004044 | 0.8835263707 | 1.012995042 |
| igdcc3 | 0.01514988869 | 0.103444081 | 0.1464548628 | 0.8835623199 | 1.01526523 |

| Protein | beta | se | z | p_val | HR |
|---|---|---|---|---|---|
| lat | -0.01363772061 | 0.09361309866 | -0.1456817561 | 0.8841726242 | 0.9864548518 |
| bcl2l15 | 0.01537133245 | 0.1056688817 | 0.1454669738 | 0.8843421895 | 1.015490079 |
| serpina9 | -0.013482234 | 0.09279868358 | -0.1452847549 | 0.8844860508 | 0.9866082442 |
| thbd | 0.01373088271 | 0.09534820654 | 0.1440077711 | 0.8854943331 | 1.013825584 |
| stx8 | 0.01366149929 | 0.09499100539 | 0.1438188724 | 0.8856434998 | 1.013755244 |
| tex101 | 0.0211773686 | 0.1473386499 | 0.1437326092 | 0.8857116201 | 1.0214032 |
| rexo2 | 0.01584831823 | 0.1106217943 | 0.1432657853 | 0.8860802764 | 1.015974569 |
| prdx3 | -0.01332167453 | 0.09302466176 | -0.1432058368 | 0.8861276202 | 0.9867666663 |
| dusp13 | -0.01494896721 | 0.1044427656 | -0.1431307102 | 0.8861869514 | 0.9851622139 |
| stx16 | -0.01365103549 | 0.09572517905 | -0.1426065287 | 0.8866009411 | 0.9864417174 |
| dnmbp | -0.01419596085 | 0.09954947969 | -0.1426020597 | 0.8866044707 | 0.9859043267 |
| lep | 0.02436743504 | 0.1710308746 | 0.142473896 | 0.886705697 | 1.024666747 |
| lacrt | 0.01553142343 | 0.1092375499 | 0.1421802617 | 0.8869376223 | 1.015652663 |
| fgfr4 | 0.01553654867 | 0.1094212073 | 0.1419884596 | 0.8870891212 | 1.015657868 |
| ccl3 | -0.01531271251 | 0.1081311884 | -0.1416123576 | 0.8873862054 | 0.9848039309 |
| acy1 | 0.01518101874 | 0.1078285604 | 0.1407884765 | 0.8880370468 | 1.015296836 |
| gfod2 | -0.013569677 | 0.09639944426 | -0.1407650957 | 0.888055518 | 0.986521976 |
| mag | -0.01496714814 | 0.1070059129 | -0.1398721597 | 0.8887609983 | 0.9851443029 |
| ripk4 | 0.01515767116 | 0.1085065011 | 0.1396936682 | 0.8889020294 | 1.015273131 |
| cbx2 | 0.01531310424 | 0.1101560211 | 0.1390128664 | 0.8894399818 | 1.015430951 |
| il2rb | -0.01316318012 | 0.09474283976 | -0.1389358832 | 0.8895008152 | 0.9869230757 |
| naa10 | 0.01421495127 | 0.1024108269 | 0.1388032076 | 0.8896056592 | 1.014316464 |
| ahsp | -0.01353289722 | 0.09763718281 | -0.1386039297 | 0.8897631378 | 0.9865582608 |
| shh | 0.01527922537 | 0.1108110327 | 0.1378854163 | 0.8903309764 | 1.01539655 |
| dcun1d1 | 0.01457425106 | 0.105896017 | 0.1376279436 | 0.8905344699 | 1.014680973 |
| wdr46 | 0.01488627628 | 0.1084464654 | 0.1372684322 | 0.8908186217 | 1.014997629 |
| clec2l | -0.0140945183 | 0.1033311045 | -0.1364015063 | 0.891503883 | 0.9860043444 |
| septin7 | -0.01368288208 | 0.1005656777 | -0.1360591645 | 0.8917745093 | 0.9864103031 |
| s100a4 | -0.01322953974 | 0.09733272473 | -0.1359207787 | 0.8918839089 | 0.986857586 |
| cracr2a | -0.01257792217 | 0.09260381549 | -0.1358250965 | 0.8919595508 | 0.9875008493 |
| fbln2 | -0.01389893677 | 0.1035463562 | -0.1342291247 | 0.8932213961 | 0.9861972075 |
| pcyt2 | -0.0140970182 | 0.1055590912 | -0.1335462255 | 0.893761409 | 0.9860018795 |
| rgs10 | 0.0131774968 | 0.09944467577 | 0.1325108328 | 0.8945802553 | 1.013264703 |
| tbr1 | 0.01379937756 | 0.1042473156 | 0.1323715386 | 0.8946904255 | 1.013895028 |
| gfra2 | 0.01252340568 | 0.09466460806 | 0.1322923735 | 0.8947530395 | 1.012602152 |
| nudt5 | -0.01294495618 | 0.09818204083 | -0.1318464769 | 0.8951057241 | 0.9871384694 |
| ranbp1 | -0.01413124508 | 0.1073276904 | -0.1316644849 | 0.8952496778 | 0.9859681323 |
| gli2 | -0.0136386639 | 0.1043669214 | -0.1306799485 | 0.8960284951 | 0.9864539213 |
| man2b2 | 0.01393686354 | 0.1069468264 | 0.1303158216 | 0.896316563 | 1.014034434 |
| cd164l2 | 0.01376390292 | 0.1058302449 | 0.1300564213 | 0.896521788 | 1.013859062 |
| hsp90b1 | -0.01215320205 | 0.09402564408 | -0.1292541218 | 0.8971565723 | 0.9879203498 |
| hsdl2 | -0.01364247429 | 0.1056592329 | -0.1291176731 | 0.8972645379 | 0.9864501625 |
| psme2 | 0.01278138113 | 0.09912978479 | 0.1289358305 | 0.8974084246 | 1.012863412 |
| l1cam | 0.01188995132 | 0.09237404733 | 0.1287152795 | 0.8975829447 | 1.011960918 |
| trpv3 | -0.01340980766 | 0.1042906538 | -0.1285811064 | 0.8976891171 | 0.9866797033 |
| ist1 | -0.01338171445 | 0.1043099205 | -0.1282880323 | 0.8979210357 | 0.9867074227 |
| grp | -0.01295966454 | 0.1011717046 | -0.1280957417 | 0.8980732059 | 0.9871239503 |
| shpk | -0.01432850498 | 0.1122809594 | -0.1276129546 | 0.8984552787 | 0.9857736595 |
| plg | -0.01431533351 | 0.112313965 | -0.1274581795 | 0.8985777711 | 0.9857866437 |
| tfap2a | -0.01307005812 | 0.1027753716 | -0.1271711103 | 0.8988049704 | 0.9870149842 |
| golm2 | -0.01257288173 | 0.1001928482 | -0.1254868182 | 0.9001381601 | 0.9875058267 |
| ttf2 | -0.01309694116 | 0.1053577282 | -0.1243092594 | 0.9010704163 | 0.9869884506 |
| serpina5 | -0.01364595358 | 0.1121391575 | -0.1216876771 | 0.903146373 | 0.9864467304 |
| fgf7 | 0.01299758205 | 0.1077564152 | 0.1206200302 | 0.9039920029 | 1.013082418 |
| stx6 | 0.01124667763 | 0.09416591311 | 0.1194347005 | 0.904930971 | 1.011310159 |
| lilra2 | 0.01133420447 | 0.09612335693 | 0.1179131153 | 0.9061365012 | 1.01139868 |
| creb3 | 0.01250029982 | 0.1063532099 | 0.117535708 | 0.9064355493 | 1.012578755 |
| hmgcs1 | 0.01248524537 | 0.1071133565 | 0.1165610507 | 0.9072079043 | 1.012563511 |
| morc3 | -0.01241221813 | 0.1068844623 | -0.116127432 | 0.9075515483 | 0.9876644957 |
| itih4 | 0.01224463124 | 0.1058304462 | 0.1157004594 | 0.9078899422 | 1.012319904 |
| gad1 | -0.01303249666 | 0.1133824814 | -0.1149427715 | 0.908490483 | 0.9870520586 |
| gba | -0.01157716628 | 0.1016319619 | -0.1139126517 | 0.9093070362 | 0.9884895912 |
| dnajb2 | 0.01243409232 | 0.1095189985 | 0.1135336561 | 0.9096074819 | 1.012511717 |
| rnase3 | 0.01106188842 | 0.09753744089 | 0.1134117147 | 0.9097041527 | 1.011123297 |
| asgr2 | 0.0122013081 | 0.1083972489 | 0.1125610496 | 0.9103785667 | 1.012276048 |
| cdh23 | -0.01150362823 | 0.1039364365 | -0.1106794558 | 0.9118705378 | 0.9885622855 |
| vsnl1 | 0.01281121601 | 0.1167461203 | 0.1097356895 | 0.9126189952 | 1.012893631 |
| cd200 | -0.01025849533 | 0.09348368929 | -0.1097356705 | 0.9126190102 | 0.9897939436 |
| ube2z | -0.01114150681 | 0.1034747711 | -0.1076736551 | 0.9142545682 | 0.9889203299 |
| zhx2 | -0.01212048448 | 0.1140088906 | -0.1063117483 | 0.9153350106 | 0.9879526727 |

| Protein | beta | se | z | p_val | HR |
|---|---|---|---|---|---|
| lcat | -0.01153883537 | 0.1086724764 | -0.1061799248 | 0.9154395986 | 0.9885274817 |
| serpinb6 | -0.009921738588 | 0.09405618211 | -0.1054873626 | 0.9159890972 | 0.9901273195 |
| slurp1 | -0.01124661982 | 0.107781302 | -0.1043466688 | 0.9168942437 | 0.988816387 |
| scamp3 | 0.009725644641 | 0.09332818922 | 0.104209079 | 0.9170034292 | 1.009773092 |
| lrp1 | 0.01013006801 | 0.0975770891 | 0.1038160505 | 0.9173153288 | 1.010181551 |
| cyp24a1 | -0.0105677818 | 0.1030629719 | -0.1025371344 | 0.9183303391 | 0.989487861 |
| hebp1 | 0.009754800099 | 0.09599663996 | 0.1016160576 | 0.9190614333 | 1.009802533 |
| esm1 | 0.009626736374 | 0.09476100473 | 0.1015896402 | 0.9190824028 | 1.009673222 |
| hbq1 | -0.01012163978 | 0.09976432127 | -0.1014555068 | 0.9191888757 | 0.9899294116 |
| ccdc50 | -0.01059878445 | 0.105142277 | -0.1008042126 | 0.9197058827 | 0.9894571848 |
| map1lc3a | -0.01045825578 | 0.1045336761 | -0.1000467617 | 0.9203072012 | 0.9895962416 |
| dnpep | 0.01049379018 | 0.1059282863 | 0.09906504246 | 0.9210866276 | 1.010549043 |
| egln1 | 0.009945991977 | 0.1018798826 | 0.09762469023 | 0.9222303182 | 1.009995618 |
| tspyl1 | -0.00985724468 | 0.1022469382 | -0.09640625774 | 0.9231979223 | 0.9901911787 |
| top1 | 0.009438992159 | 0.09871451708 | 0.09561908864 | 0.9238231041 | 1.00948368 |
| pithd1 | -0.01057247684 | 0.1108801293 | -0.09535050968 | 0.9240364245 | 0.9894832154 |
| cep152 | -0.01001209537 | 0.1067584454 | -0.09378270107 | 0.9252817737 | 0.9900378588 |
| f12 | 0.01012345652 | 0.1085957466 | 0.09322148273 | 0.925727608 | 1.010174872 |
| siae | -0.009191953079 | 0.0989950655 | -0.09285263899 | 0.9260206317 | 0.9908501638 |
| wars | -0.008830391404 | 0.09534985579 | -0.09261043272 | 0.9262130553 | 0.991208482 |
| lyn | -0.008524603241 | 0.09211950976 | -0.09253852158 | 0.9262701867 | 0.9915116282 |
| med21 | 0.009986746711 | 0.1080233618 | 0.09244987887 | 0.9263406115 | 1.010036781 |
| nos2 | 0.009954033898 | 0.1082845824 | 0.09192475676 | 0.9267578217 | 1.01000374 |
| cep112 | -0.01011054359 | 0.1102142382 | -0.09173536697 | 0.9269082971 | 0.9899403961 |
| oxct1 | 0.009235845244 | 0.1007842999 | 0.09163972221 | 0.9269842905 | 1.009278627 |
| uso1 | -0.008369824453 | 0.09232401378 | -0.09065706862 | 0.9277650844 | 0.991665105 |
| tgm2 | 0.008905895453 | 0.09825838942 | 0.09063750694 | 0.9277806283 | 1.008945671 |
| hdgf | 0.008802481495 | 0.09762954754 | 0.09016206381 | 0.9281584302 | 1.008841337 |
| vwa5a | -0.009093112233 | 0.1015082154 | -0.08958006204 | 0.9286209289 | 0.9909481051 |
| fkbpl | -0.009443868948 | 0.1059195915 | -0.08916073798 | 0.9289541676 | 0.9906005843 |
| lbp | 0.008767843959 | 0.09895634841 | 0.08860314775 | 0.9293973064 | 1.008806394 |
| pydc1 | -0.009678872887 | 0.1094580046 | -0.08842544608 | 0.9295385375 | 0.9903678166 |
| adamtsl5 | -0.009071569036 | 0.1027372188 | -0.08829876009 | 0.9296392244 | 0.9909694535 |
| mia | -0.008098310904 | 0.09182774566 | -0.08819023973 | 0.9297254747 | 0.9919343921 |
| prg2 | 0.008978743373 | 0.1028354774 | 0.08731172939 | 0.9304237309 | 1.009019173 |
| yod1 | 0.009683027439 | 0.1116120145 | 0.08675613894 | 0.9308653522 | 1.00973006 |
| mslnl | -0.008540177889 | 0.09926442177 | -0.08603463091 | 0.9314388878 | 0.9914961858 |
| scn3a | -0.008563164817 | 0.1012588879 | -0.08456704392 | 0.9326056003 | 0.9914733946 |
| sparcl1 | 0.008046414565 | 0.09519275721 | 0.08452759223 | 0.9326369659 | 1.008078874 |
| ces3 | 0.008396373664 | 0.09992283197 | 0.08402857983 | 0.9330337087 | 1.008431722 |
| il21r | 0.008799421406 | 0.1047796625 | 0.08398024193 | 0.9330721409 | 1.00883825 |
| edem2 | 0.00915402735 | 0.1091762484 | 0.08384632636 | 0.9331786146 | 1.009196054 |
| vps28 | -0.008718841623 | 0.1040333657 | -0.08380812797 | 0.9332089856 | 0.9913190573 |
| bche | 0.00912038086 | 0.1091054219 | 0.08359237057 | 0.9333805331 | 1.009162098 |
| araf | 0.008443707111 | 0.102879368 | 0.0820738626 | 0.9345879776 | 1.008479456 |
| psmd1 | 0.008755768145 | 0.1067598435 | 0.08201368469 | 0.9346358313 | 1.008794212 |
| caly | 0.008642088126 | 0.1060128489 | 0.08151925184 | 0.9350290151 | 1.008679539 |
| il1a | -0.007402322278 | 0.0908964073 | -0.08143690711 | 0.9350944989 | 0.9926250074 |
| trim26 | -0.008776099956 | 0.1077952119 | -0.08141456194 | 0.9351122688 | 0.9912622976 |
| tslp | -0.007981390145 | 0.09944955536 | -0.08025566446 | 0.9360339191 | 0.9920503766 |
| gid8 | 0.008277030199 | 0.1031405101 | 0.08025004137 | 0.9360383912 | 1.00831138 |
| sumf1 | 0.008322642516 | 0.1043308056 | 0.07977166922 | 0.9364188572 | 1.008357372 |
| akr1b10 | -0.007637312575 | 0.09760352609 | -0.07824832648 | 0.9376305209 | 0.9923917776 |
| cyth3 | 0.008442065344 | 0.1084248075 | 0.07786101301 | 0.9379386123 | 1.0084778 |
| cd3g | 0.007865871013 | 0.1015645285 | 0.07744702926 | 0.9382679292 | 1.007896888 |
| il12rb2 | 0.007983895331 | 0.1055263272 | 0.07565785282 | 0.9396913084 | 1.008015852 |
| rpe | 0.007859105441 | 0.1067304625 | 0.07363507343 | 0.9413007623 | 1.007890069 |
| scrn1 | -0.007028312036 | 0.09862517693 | -0.07126285858 | 0.9431885546 | 0.9929963288 |
| a1bg | -0.007740006708 | 0.111895756 | -0.0691715842 | 0.9448530414 | 0.99228987 |
| traf2 | 0.006624995523 | 0.09621974894 | 0.06885276251 | 0.9451068193 | 1.006646989 |
| rac3 | -0.007237078348 | 0.105278448 | -0.06874225905 | 0.9451947799 | 0.9927890462 |
| epb41l5 | -0.007275818578 | 0.1068547702 | -0.06809072316 | 0.9457134151 | 0.9927505861 |
| nell2 | 0.006366162163 | 0.09392643937 | 0.06777816988 | 0.9459622217 | 1.006386469 |
| abo | -0.006847502991 | 0.103140007 | -0.06639036775 | 0.9470670387 | 0.9931758877 |
| nup50 | 0.006876637586 | 0.1039536773 | 0.06615097961 | 0.9472576238 | 1.006900336 |
| sh2d1a | -0.006497560858 | 0.09872873524 | -0.0658122566 | 0.947527298 | 0.9935235026 |
| il3ra | 0.006546628694 | 0.09954008979 | 0.0657687642 | 0.9475619249 | 1.006568105 |
| cx3cl1 | -0.006200159935 | 0.09465206688 | -0.06550474955 | 0.9477721249 | 0.9938190214 |
| entr1 | 0.006839750513 | 0.1056592605 | 0.06473403734 | 0.9483857618 | 1.006863195 |
| aldh2 | -0.006815242057 | 0.1076094323 | -0.063333129 | 0.9495012356 | 0.993207929 |

| Protein | beta | se | z | p_val | HR |
|---|---|---|---|---|---|
| fzd10 | 0.006486665584 | 0.1054731177 | 0.06150065274 | 0.9509604946 | 1.00650775 |
| impg1 | -0.00660387144 | 0.1075294324 | -0.06141454756 | 0.9510290669 | 0.9934178862 |
| klrc1 | 0.00609311333 | 0.09936765104 | 0.06131888262 | 0.9511052529 | 1.006111714 |
| serpinb5 | 0.005856107769 | 0.09604707533 | 0.06097122425 | 0.9513821261 | 1.005873288 |
| cd86 | -0.006166596121 | 0.1013742874 | -0.06082998244 | 0.951494612 | 0.9938523783 |
| apod | 0.006691101125 | 0.1102597086 | 0.06068491571 | 0.9516101451 | 1.006713537 |
| ccne1 | -0.00644881913 | 0.1064806544 | -0.06056329354 | 0.9517070073 | 0.9935719299 |
| vwc2l | 0.006449568878 | 0.1074793676 | 0.06000750676 | 0.9521496559 | 1.006470412 |
| b4gat1 | 0.005737887199 | 0.09682568165 | 0.05925997216 | 0.9527450426 | 1.00575438 |
| aif1 | -0.005648430111 | 0.09632252634 | -0.05864080112 | 0.953238212 | 0.9943674923 |
| nsfl1c | 0.005828319562 | 0.09987287785 | 0.05835738078 | 0.9534639621 | 1.005845337 |
| hif1a | -0.006294946389 | 0.108407191 | -0.05806760909 | 0.953694775 | 0.9937248253 |
| gstp1 | 0.005342557128 | 0.09556922918 | 0.05590248215 | 0.9554194935 | 1.005356854 |
| nptx1 | -0.005490522294 | 0.09890194994 | -0.05551480327 | 0.9557283368 | 0.9945245231 |
| fbp1 | -0.005344493317 | 0.09640619165 | -0.05543724138 | 0.9557901271 | 0.9946697631 |
| adamtsl4 | -0.005945201647 | 0.1074113108 | -0.0553498659 | 0.9558597358 | 0.9940724361 |
| eloa | 0.005651617349 | 0.1026628091 | 0.05505028938 | 0.9560983994 | 1.005667618 |
| ccl23 | 0.004928854957 | 0.09236303972 | 0.05336393185 | 0.9574419425 | 1.004941022 |
| zcchc8 | 0.005779892747 | 0.1085229238 | 0.05325964821 | 0.9575250306 | 1.005796629 |
| dnaja2 | -0.004990612114 | 0.09422581372 | -0.0529643833 | 0.9577602859 | 0.9950218203 |
| mnda | 0.005047940254 | 0.09888841759 | 0.05104683013 | 0.9592882041 | 1.005060703 |
| cdkn1a | -0.004711958846 | 0.09231329236 | -0.05104312418 | 0.9592911572 | 0.995299125 |
| hmbs | -0.004915469495 | 0.09741628698 | -0.05045839508 | 0.9597571031 | 0.9950965917 |
| ace2 | 0.005331813911 | 0.1063888175 | 0.05011630016 | 0.9600297104 | 1.005346053 |
| pm20d1 | -0.004526759507 | 0.09036993012 | -0.05009143529 | 0.9600495248 | 0.9954834708 |
| prkag3 | 0.005125972952 | 0.102959389 | 0.04978635755 | 0.9602926383 | 1.005139133 |
| dctn6 | -0.004746292184 | 0.09619652597 | -0.04933953836 | 0.9606487108 | 0.9952649537 |
| arl13b | -0.004857521344 | 0.1003787316 | -0.0483919379 | 0.9614038844 | 0.9951542573 |
| eng | 0.004264937975 | 0.08827407753 | 0.0483147272 | 0.9614654177 | 1.004274046 |
| khdc3l | 0.005282859159 | 0.1093677399 | 0.0483036329 | 0.9614742593 | 1.005296838 |
| slamf6 | 0.004624152689 | 0.09620077374 | 0.04806772866 | 0.9616622653 | 1.004634861 |
| palld | 0.004991078029 | 0.1056182724 | 0.04725581961 | 0.9623093396 | 1.005003554 |
| sptbn2 | 0.005183461961 | 0.1100637771 | 0.04709507611 | 0.9624374517 | 1.005196919 |
| ppme1 | 0.004560796025 | 0.09743923151 | 0.0468065681 | 0.9626673942 | 1.004571212 |
| morn4 | 0.004722387394 | 0.1024468429 | 0.0460959778 | 0.9632337519 | 1.004733555 |
| il5 | 0.004358684939 | 0.09481982802 | 0.04596807471 | 0.9633356957 | 1.004368198 |
| cd72 | 0.005037867511 | 0.1110109434 | 0.04538171963 | 0.9638030516 | 1.005050579 |
| icam5 | -0.004187740225 | 0.09280820478 | -0.04512252161 | 0.9640096501 | 0.9958210161 |
| afap1 | 0.005047530061 | 0.111906951 | 0.04510470542 | 0.9640238509 | 1.00506029 |
| c1galt1c1 | 0.004981609443 | 0.1109897288 | 0.04488351756 | 0.9642001547 | 1.004994038 |
| pxn | 0.004460661698 | 0.1004074111 | 0.04442562206 | 0.9645651384 | 1.004470625 |
| fndc1 | 0.004472034359 | 0.1044879657 | 0.04279951599 | 0.9658613498 | 1.004482049 |
| slk | 0.004811161155 | 0.1126308468 | 0.04271619448 | 0.96592777 | 1.004822753 |
| lrtm1 | 0.004526211209 | 0.1081537892 | 0.04184977007 | 0.966618459 | 1.00453647 |
| lrfn2 | 0.004252692773 | 0.1030375216 | 0.04127324402 | 0.9670780631 | 1.004261748 |
| cxcl1 | 0.004080887967 | 0.1000006388 | 0.04080861899 | 0.9674484681 | 1.004089226 |
| nrp2 | 0.003711980136 | 0.09563156392 | 0.03881542854 | 0.9690375439 | 1.003718878 |
| s100g | 0.003821510288 | 0.09896607237 | 0.03861434728 | 0.9691978633 | 1.003828822 |
| sat2 | -0.004156608983 | 0.1081526892 | -0.03843278436 | 0.9693426221 | 0.9958520178 |
| pdrg1 | -0.004153726541 | 0.1098359603 | -0.03781754653 | 0.9698331543 | 0.9958548882 |
| raly | -0.004186045211 | 0.1111260484 | -0.0376693428 | 0.9699513195 | 0.9958227041 |
| sema3g | 0.004031925891 | 0.1071647091 | 0.03762363491 | 0.9699877633 | 1.004040065 |
| fxn | -0.003443783074 | 0.09431463512 | -0.03651377191 | 0.9708726976 | 0.9965621399 |
| crlf1 | -0.003462263725 | 0.09686614675 | -0.03574276299 | 0.9714874724 | 0.996543723 |
| tmsb10 | 0.00341741055 | 0.09809502804 | 0.03483775496 | 0.9722091148 | 1.003423257 |
| cptp | -0.003471415298 | 0.09974757712 | -0.03480200119 | 0.9722376249 | 0.9965346031 |
| enah | 0.003388021729 | 0.09854563599 | 0.03438023099 | 0.9725739475 | 1.003393768 |
| ca2 | -0.003172685515 | 0.09764446245 | -0.03249222164 | 0.974079519 | 0.9968323421 |
| myl6b | 0.003293066712 | 0.1029190933 | 0.03199665492 | 0.9744747185 | 1.003298495 |
| acan | 0.00321888663 | 0.1027744547 | 0.03131990961 | 0.9750144126 | 1.003224073 |
| unc79 | 0.003195090788 | 0.106183818 | 0.03009018555 | 0.975995128 | 1.003200201 |
| uhrf2 | 0.00316563352 | 0.1079403814 | 0.02932761103 | 0.976603306 | 1.003170649 |
| sez6l2 | -0.00292121977 | 0.100003453 | -0.02921118904 | 0.9766961575 | 0.9970830428 |
| eppk1 | -0.003055373714 | 0.1050016874 | -0.02909832964 | 0.976786168 | 0.9969492892 |
| apobr | -0.00304763983 | 0.1056706884 | -0.0288409196 | 0.9769914653 | 0.9969569995 |
| sfrp4 | 0.003137580902 | 0.1125003337 | 0.0278895253 | 0.9777502628 | 1.003142508 |
| dnm1 | -0.002759678029 | 0.1016251622 | -0.02715546002 | 0.9783357403 | 0.9972441264 |
| ca1 | 0.002737690744 | 0.1011244174 | 0.02707249955 | 0.9784019089 | 1.002741442 |
| tyrp1 | -0.002779852407 | 0.1040092574 | -0.026726971 | 0.9786775011 | 0.9972240078 |
| cpq | -0.002618507783 | 0.1023898255 | -0.02557390611 | 0.9795971992 | 0.9973849175 |

| Protein | beta | se | z | p_val | HR |
|---|---|---|---|---|---|
| csf2rb | -0.002789675063 | 0.1091391758 | -0.0255607122 | 0.9796077229 | 0.9972142125 |
| coro1a | -0.002409244891 | 0.09505131721 | -0.02534678069 | 0.9797783603 | 0.997593655 |
| itgb5 | 0.002309751292 | 0.09186926983 | 0.02514171819 | 0.9799419244 | 1.002312421 |
| cfc1 | 0.0023225268 | 0.09306214981 | 0.02495672843 | 0.9800894786 | 1.002325226 |
| gsta1 | -0.002634988239 | 0.1056862315 | -0.02493218087 | 0.9801090586 | 0.9973684803 |
| tspan8 | 0.002637869048 | 0.1075850227 | 0.0245189245 | 0.9804386887 | 1.002641351 |
| mpi | 0.002310757271 | 0.09538829619 | 0.02422474625 | 0.9806733393 | 1.002313429 |
| pla2g15 | -0.002402564292 | 0.09990764746 | -0.02404785172 | 0.9808144396 | 0.9976003196 |
| egfl7 | -0.002346962429 | 0.09775908636 | -0.0240076142 | 0.9808465352 | 0.9976557895 |
| sox2 | 0.002503069989 | 0.1050215585 | 0.02383386826 | 0.9809851248 | 1.002506205 |
| adamts13 | 0.0020765517 | 0.08916436979 | 0.02328903019 | 0.981419722 | 1.002078709 |
| kcnc4 | -0.002487436384 | 0.1090673931 | -0.02280641642 | 0.9818046898 | 0.9975156547 |
| rpgr | 0.002316341031 | 0.1032533948 | 0.02243355811 | 0.9821021116 | 1.002319026 |
| itgax | 0.002360460862 | 0.1065960834 | 0.02214397365 | 0.9823331092 | 1.002363249 |
| il3 | 0.002319397533 | 0.1048489825 | 0.02212131656 | 0.9823511825 | 1.002322089 |
| serpina1 | 0.002309171791 | 0.1052075937 | 0.02194871786 | 0.9824888629 | 1.00231184 |
| lamp2 | -0.002075775303 | 0.09918190201 | -0.02092897253 | 0.9833023149 | 0.9979263776 |
| erbb2 | 0.002092157756 | 0.1025193089 | 0.02040745083 | 0.9837183402 | 1.002094348 |
| rbpms | 0.002084951706 | 0.108635383 | 0.01919219731 | 0.9846877821 | 1.002087127 |
| il9 | -0.002011217727 | 0.1074841525 | -0.01871176056 | 0.9850710463 | 0.9979908034 |
| il10 | 0.001759480979 | 0.09632516097 | 0.01826605802 | 0.9854266047 | 1.00176103 |
| cd274 | 0.001805392236 | 0.09955837714 | 0.01813400628 | 0.9855319493 | 1.001807023 |
| grpel1 | -0.001573103584 | 0.09917210271 | -0.01586235989 | 0.9873441987 | 0.9984281331 |
| tprkb | -0.00159025203 | 0.1115903427 | -0.01425080335 | 0.9886298889 | 0.9984110118 |
| selenop | 0.001443397647 | 0.1043357802 | 0.0138341578 | 0.9889622912 | 1.00144444 |
| gimap8 | 0.001414114768 | 0.1078272786 | 0.01311462913 | 0.9895363398 | 1.001415115 |
| txnl1 | -0.001199597609 | 0.1052311644 | -0.01139964207 | 0.9909045986 | 0.9988011216 |
| urod | 0.001016564555 | 0.1084274955 | 0.009375523711 | 0.992519524 | 1.001017081 |
| ptprr | -0.000841370619 | 0.102090354 | -0.008241431107 | 0.9934243638 | 0.9991589832 |
| dkkl1 | 0.0007460016988 | 0.09811577565 | 0.007603279839 | 0.9939335189 | 1.00074628 |
| tpsg1 | -0.0007583290216 | 0.1021719875 | -0.007422083489 | 0.9940780885 | 0.9992419584 |
| fuom | 0.0008210144865 | 0.1134564837 | 0.007236382266 | 0.9942262527 | 1.000821352 |
| ppp1r12b | -0.0006220489484 | 0.1048638546 | -0.005931967225 | 0.9952670027 | 0.9993781445 |
| thsd1 | 0.0004698886994 | 0.09842762627 | 0.004773951351 | 0.9961909524 | 1.000469999 |
| cpb1 | 0.0004074989184 | 0.08735147606 | 0.004665049027 | 0.9962778429 | 1.000407582 |
| cant1 | -0.0002142255181 | 0.09557602591 | -0.00224141479 | 0.9982116112 | 0.9997857974 |
| mtdh | 0.0001939946758 | 0.09861032818 | 0.001967285571 | 0.9984303342 | 1.000194013 |
| cdk1 | 0.0001446623006 | 0.1037341868 | 0.00139454798 | 0.9988873121 | 1.000144673 |
| ecscr | 0.0001353336846 | 0.1069539508 | 0.001265345352 | 0.9989904007 | 1.000135343 |
| stx7 | 6.74728e-05 | 0.1070770828 | 0.000630133101 | 0.9994972266 | 1.000067475 |
| cntnap4 | 6.03016e-06 | 0.103891232 | 5.8043e-05 | 0.9999536884 | 1.00000603 |

**Supplementary Table 11. Associations between circulating plasma proteins and incident glaucoma in the UK Biobank**

| Protein | beta | se | z | p_val | HR |
|---|---|---|---|---|---|
| psmd9 | 0.2205222622 | 0.05909219686 | 3.731833878 | 0.0001900908482 | 1.246727679 |
| cxcl10 | -0.2196852924 | 0.06387907848 | -3.439080489 | 0.0005836936638 | 0.8027713964 |
| dpep1 | 0.1887471261 | 0.05940608023 | 3.177235822 | 0.001486860814 | 1.207735509 |
| pla2g7 | -0.1827723767 | 0.05911528925 | -3.091795354 | 0.001989499538 | 0.8329577348 |
| klk13 | 0.1784701215 | 0.05900421692 | 3.024701129 | 0.002488789301 | 1.195387166 |
| ubac1 | 0.1730430352 | 0.05916722681 | 2.924643329 | 0.003448512293 | 1.188917269 |
| serpind1 | 0.1929285201 | 0.06761315773 | 2.8534168 | 0.004325185201 | 1.2127961 |
| pth1r | 0.1633421562 | 0.05871483083 | 2.781957367 | 0.005403213741 | 1.177439489 |
| col24a1 | 0.173482607 | 0.06345843178 | 2.733799151 | 0.006260822175 | 1.189439999 |
| nt5c1a | 0.1965771601 | 0.07289097205 | 2.696865669 | 0.006999550012 | 1.217229239 |
| aarsd1 | 0.1610634028 | 0.05986770036 | 2.690322191 | 0.007138306392 | 1.17475945 |
| ssc5d | 0.165520501 | 0.06191567012 | 2.673321643 | 0.007510420179 | 1.180007154 |
| tgm2 | 0.1571102749 | 0.05893170321 | 2.665972072 | 0.007676606993 | 1.170124642 |
| eif5 | 0.1701559137 | 0.06414646145 | 2.652615746 | 0.007987073836 | 1.185489671 |
| cdan1 | 0.1649479935 | 0.06290207046 | 2.622298316 | 0.008733894441 | 1.179331784 |
| tmprss5 | 0.1600297554 | 0.06105196461 | 2.621205663 | 0.008761938639 | 1.17354579 |
| dtx3 | 0.1671524781 | 0.06429864802 | 2.599626637 | 0.009332523732 | 1.181934471 |
| fabp9 | 0.1511646517 | 0.05831375872 | 2.592263902 | 0.00953466007 | 1.163188163 |
| plb1 | 0.1750929331 | 0.06780225114 | 2.582405896 | 0.009811411572 | 1.191356928 |
| blvrb | 0.1521131112 | 0.05898327468 | 2.578919397 | 0.009910990941 | 1.164291923 |
| fcrl5 | -0.1553167506 | 0.06043121629 | -2.570141064 | 0.01016571109 | 0.8561439505 |
| creld1 | 0.1740466733 | 0.06772370802 | 2.569951918 | 0.01017126311 | 1.190111111 |
| nell2 | -0.1539015434 | 0.05996013051 | -2.566731294 | 0.01026621326 | 0.8573564293 |
| sf3b4 | 0.1505908289 | 0.05873865469 | 2.563743241 | 0.01035501152 | 1.162520891 |
| ifngr2 | -0.1550224212 | 0.06047669241 | -2.563341596 | 0.01036699948 | 0.8563959758 |
| fcrl3 | 0.1458547599 | 0.05736803288 | 2.542439624 | 0.01100816178 | 1.157028129 |
| morc3 | 0.15985103 | 0.06314499141 | 2.531491832 | 0.01135784628 | 1.173336066 |
| serpina6 | 0.1857035891 | 0.07378998428 | 2.516650341 | 0.01184763421 | 1.204065309 |
| tp53inp1 | -0.1471854751 | 0.05857146369 | -2.512921238 | 0.01197360636 | 0.8631338728 |
| angptl7 | 0.1494423356 | 0.05964992385 | 2.505323157 | 0.01223395593 | 1.16118651 |
| fst | -0.1542427278 | 0.06188487984 | -2.492413788 | 0.01268781279 | 0.8570639625 |
| ca2 | 0.1476455522 | 0.05941071946 | 2.485166878 | 0.0129490717 | 1.159101982 |
| afp | 0.1533620947 | 0.06173014062 | 2.484395681 | 0.01297715241 | 1.165747013 |
| scgb1a1 | -0.1459946963 | 0.05958290454 | -2.45027827 | 0.01427458495 | 0.8641622865 |
| ctrl | 0.1477930151 | 0.06137256678 | 2.408128303 | 0.01603454477 | 1.15927292 |
| palm2 | 0.1780220709 | 0.0740031582 | 2.405600993 | 0.0161458885 | 1.194851692 |
| kcnh2 | 0.1490515085 | 0.06207261201 | 2.401244345 | 0.01633942197 | 1.160732775 |
| col2a1 | 0.1532712286 | 0.06390421506 | 2.398452566 | 0.01646450879 | 1.165641091 |
| creg1 | -0.1454573517 | 0.06074876127 | -2.394408523 | 0.01664719546 | 0.8646267641 |
| ca14 | -0.1532711575 | 0.06404869948 | -2.393040901 | 0.01670937827 | 0.857897065 |
| mzb1 | -0.1418975281 | 0.05935334385 | -2.390725087 | 0.01681513847 | 0.8677101678 |
| xcl1 | -0.1388067276 | 0.05816048816 | -2.386615587 | 0.01700426106 | 0.8703962358 |
| mme | 0.1452183373 | 0.06117639712 | 2.373764133 | 0.0176077943 | 1.156292004 |
| cd14 | 0.1445364409 | 0.06107021033 | 2.366725775 | 0.01794622447 | 1.155503802 |
| ada2 | -0.1410513387 | 0.05992145292 | -2.353937226 | 0.01857574774 | 0.8684447257 |
| cep164 | 0.1369655943 | 0.05825643966 | 2.351080758 | 0.01871897063 | 1.146788692 |
| serpinc1 | 0.1539037208 | 0.0657344878 | 2.341293375 | 0.01921705985 | 1.166378583 |
| stx5 | 0.1514380358 | 0.0647224183 | 2.339808058 | 0.0192936527 | 1.163506204 |
| fos | 0.1496277869 | 0.06411075308 | 2.333895326 | 0.01960120357 | 1.161401873 |
| psmg3 | 0.1365655784 | 0.05869157743 | 2.326834349 | 0.01997408345 | 1.14633005 |
| crisp2 | -0.154205714 | 0.06642893208 | -2.321363737 | 0.02026722096 | 0.8570956863 |
| glrx | 0.1364708169 | 0.059310799 | 2.300943828 | 0.02139480636 | 1.146221427 |
| vcpkmt | 0.1443229605 | 0.06286406651 | 2.295794219 | 0.02168765012 | 1.155257151 |
| ddr1 | 0.1346316795 | 0.0592292114 | 2.273062164 | 0.02302243406 | 1.144115306 |
| fga | 0.1567547487 | 0.06922837687 | 2.264313505 | 0.02355483955 | 1.169708706 |
| crtap | -0.1450762118 | 0.06414113617 | -2.261827907 | 0.02370803797 | 0.8649563708 |
| nxpe4 | -0.1407495449 | 0.06312513856 | -2.229690866 | 0.02576797269 | 0.8687068565 |
| tnfrsf17 | -0.1346728751 | 0.06087935907 | -2.212127019 | 0.02695788972 | 0.8740017727 |
| ahsp | 0.1334242518 | 0.06040774858 | 2.208727439 | 0.02719360469 | 1.142734703 |
| hagh | 0.1295775374 | 0.0593109244 | 2.1847162 | 0.02890966162 | 1.138347373 |
| ppme1 | 0.1276529724 | 0.05849579989 | 2.182258772 | 0.02909043992 | 1.136158656 |
| ckmt1a_ckmt1b | 0.1263971033 | 0.05794929928 | 2.18116707 | 0.02917106153 | 1.134732685 |
| actn2 | -0.1454488962 | 0.06672205752 | -2.179922226 | 0.02926322708 | 0.864634075 |
| cebpa | 0.1425698704 | 0.06545097906 | 2.178269485 | 0.02938597963 | 1.153233655 |
| pvr | 0.1271846167 | 0.0586131948 | 2.169897361 | 0.0300146223 | 1.135626654 |
| ranbp1 | 0.1364531773 | 0.06307799792 | 2.163245217 | 0.03052232413 | 1.146201208 |
| cfhr2 | 0.1353318875 | 0.06261722409 | 2.161256579 | 0.03067552504 | 1.144916705 |
| eif1ax | 0.1351887992 | 0.06260684858 | 2.159329247 | 0.03082463269 | 1.144752892 |

| Protein | beta | se | z | p_val | HR |
|---|---|---|---|---|---|
| itgbl1 | 0.1428437447 | 0.06630070689 | 2.154482981 | 0.03120231519 | 1.153549539 |
| cpped1 | 0.1272056062 | 0.05950761432 | 2.137635792 | 0.03254631447 | 1.135650491 |
| il17rb | 0.1291330114 | 0.06061576039 | 2.130353733 | 0.03314242053 | 1.13784146 |
| igfbp3 | 0.1259029606 | 0.05915083437 | 2.128506925 | 0.03329507643 | 1.134172104 |
| ivd | 0.1229547778 | 0.05809029503 | 2.116614793 | 0.0342925536 | 1.130833281 |
| tagln3 | -0.1345249168 | 0.06383597608 | -2.107352703 | 0.0350870181 | 0.8741310981 |
| pklr | 0.1222270503 | 0.05816250709 | 2.101474926 | 0.0355992968 | 1.130010642 |
| ddt | 0.1357098601 | 0.06473735762 | 2.09631448 | 0.03605430312 | 1.145349534 |
| ca1 | 0.1250859546 | 0.05974902799 | 2.093522837 | 0.03630250761 | 1.133245857 |
| nppb | -0.1215895697 | 0.05824164491 | -2.087674032 | 0.03682724884 | 0.8855117348 |
| wfikkn1 | -0.1292724863 | 0.06202274807 | -2.084275373 | 0.03713512614 | 0.8787344898 |
| il2 | -0.1208609242 | 0.05834905949 | -2.071343142 | 0.0383267403 | 0.886157194 |
| cd3g | 0.1319919168 | 0.06377840879 | 2.069539195 | 0.03849551724 | 1.141099095 |
| c4bpb | 0.1236333163 | 0.06024250167 | 2.052260661 | 0.04014433699 | 1.131600855 |
| slc34a3 | -0.1303844647 | 0.06367923042 | -2.04751948 | 0.04060710625 | 0.8777578991 |
| rbks | 0.1266066658 | 0.06187106788 | 2.046298377 | 0.04072702333 | 1.134970507 |
| megf10 | -0.1237270656 | 0.06057913707 | -2.042403896 | 0.04111148361 | 0.8836209786 |
| fut3_fut5 | 0.1221289461 | 0.05982533441 | 2.041425214 | 0.04120858041 | 1.129899789 |
| tspan7 | -0.129622013 | 0.0635507843 | -2.039660319 | 0.0413841697 | 0.8784274023 |
| mfge8 | 0.1215751298 | 0.05998752213 | 2.02667364 | 0.04269580002 | 1.129274205 |
| pglyrp2 | 0.1292098328 | 0.06395961387 | 2.020178437 | 0.04336488209 | 1.137928874 |
| slitrk1 | 0.1428223272 | 0.07082615719 | 2.016519501 | 0.04374568109 | 1.153524833 |
| cdh15 | 0.1403446184 | 0.06967991452 | 2.014133045 | 0.04399556691 | 1.150670273 |
| slirp | 0.1276498428 | 0.06340788587 | 2.01315406 | 0.04409842453 | 1.1361551 |
| tcp11 | -0.1266175192 | 0.0630488784 | -2.008243802 | 0.04461739054 | 0.8810706007 |
| aldh1a1 | 0.1231485738 | 0.06171843258 | 1.995328926 | 0.04600701705 | 1.131052453 |
| ifng | -0.1159586334 | 0.05820545426 | -1.992229678 | 0.04634586342 | 0.89051206 |
| cfhr5 | 0.1259507004 | 0.06340891148 | 1.98632491 | 0.04699725847 | 1.13422625 |
| pdcd1lg2 | 0.1166336004 | 0.0588621078 | 1.981471693 | 0.04753840077 | 1.123707628 |
| icoslg | 0.1165360637 | 0.05886017665 | 1.979879611 | 0.04771705795 | 1.123598031 |
| ctla4 | 0.1223524012 | 0.06195393386 | 1.974893176 | 0.04828027195 | 1.130152299 |
| tnfrsf10c | 0.1155743522 | 0.05859291377 | 1.972497095 | 0.04855288766 | 1.122517973 |
| c9orf40 | 0.1233401839 | 0.06260749953 | 1.970054464 | 0.04883212889 | 1.131269195 |
| hmbs | 0.1148854343 | 0.05855905852 | 1.961872973 | 0.04977727511 | 1.121744917 |
| impact | 0.1230703184 | 0.06309550078 | 1.950540322 | 0.05111175224 | 1.130963946 |
| csf2 | -0.125247244 | 0.06449307056 | -1.942026374 | 0.0521339075 | 0.8822787375 |
| scarb2 | -0.125481551 | 0.06464248487 | -1.941162244 | 0.05223860119 | 0.8820720377 |
| ambn | -0.1143725396 | 0.05911614343 | -1.934709083 | 0.05302600516 | 0.8919256163 |
| tdgf1 | -0.1108881976 | 0.05739906801 | -1.931881499 | 0.05337413375 | 0.8950388108 |
| raly | 0.1223307712 | 0.06341422003 | 1.929074759 | 0.05372158204 | 1.130127854 |
| ptgr1 | 0.1259452908 | 0.06531024064 | 1.928415659 | 0.05380344561 | 1.134220114 |
| prdx6 | 0.1108528953 | 0.05758608102 | 1.924994606 | 0.05423003307 | 1.117230545 |
| calb1 | 0.1140156237 | 0.05924038923 | 1.924626512 | 0.05427610011 | 1.120769635 |
| ece1 | -0.111651596 | 0.05829600072 | -1.915253098 | 0.05546023385 | 0.8943558004 |
| adam9 | 0.1256487167 | 0.06561548793 | 1.914924671 | 0.05550211098 | 1.133883784 |
| npy | -0.1128834321 | 0.05911546724 | -1.9095414 | 0.05619228638 | 0.8932547789 |
| f9 | 0.1227269405 | 0.06430931114 | 1.908385245 | 0.05634144253 | 1.130575664 |
| galnt10 | 0.1151293733 | 0.06036224874 | 1.907307558 | 0.05648077203 | 1.122018587 |
| ggt1 | 0.1258744817 | 0.06616381863 | 1.902467003 | 0.05711012873 | 1.134139804 |
| fn1 | 0.1252749086 | 0.06600750206 | 1.897888948 | 0.05771071254 | 1.133460008 |
| gnpda1 | 0.1215080259 | 0.06445512835 | 1.885156837 | 0.05940866631 | 1.129198429 |
| mepe | -0.1155500133 | 0.06168887191 | -1.873109521 | 0.06105326293 | 0.8908760155 |
| igdcc4 | 0.1225626562 | 0.06544926374 | 1.872636134 | 0.061118648 | 1.130389944 |
| enpp2 | -0.1307411915 | 0.0698986725 | -1.870438835 | 0.06142290337 | 0.8774448352 |
| pttg1 | -0.118563775 | 0.06339206376 | -1.870325211 | 0.06143867063 | 0.8881951692 |
| zp3 | -0.1168595903 | 0.06258984622 | -1.867069459 | 0.06189188878 | 0.8897101083 |
| klk4 | 0.1161739479 | 0.06240459988 | 1.861624754 | 0.06265600163 | 1.123191232 |
| tff3 | -0.1147546397 | 0.06223079786 | -1.844016847 | 0.065180685 | 0.8915848766 |
| clns1a | 0.1170407382 | 0.06350634299 | 1.842977137 | 0.06533234873 | 1.124165225 |
| crlf1 | -0.1149740266 | 0.0624623335 | -1.840693746 | 0.06566645099 | 0.891389296 |
| acp5 | 0.1120289581 | 0.06094864433 | 1.838087775 | 0.06604947229 | 1.118545251 |
| itgb7 | 0.1070669066 | 0.05828465244 | 1.836965688 | 0.06621496093 | 1.11300872 |
| kiaa2013 | -0.1163320534 | 0.0633747198 | -1.835622371 | 0.06641352618 | 0.890179587 |
| plxdc2 | 0.1162953447 | 0.06361068147 | 1.828236107 | 0.06751412422 | 1.123327592 |
| gpr37 | -0.1107149495 | 0.06055989517 | -1.828189252 | 0.06752115346 | 0.895193888 |
| atp5po | 0.1061688822 | 0.05807989126 | 1.827980045 | 0.06755254658 | 1.112009659 |
| col9a1 | 0.1076405765 | 0.05899853983 | 1.824461704 | 0.06808230093 | 1.113647402 |
| gad1 | -0.1170857309 | 0.0647332639 | -1.808741347 | 0.07049119629 | 0.8895089315 |
| mfap3 | 0.1058509178 | 0.05855664242 | 1.80766713 | 0.07065832333 | 1.111656136 |
| aldh3a1 | 0.109072365 | 0.06052146376 | 1.802209634 | 0.0715124296 | 1.115243052 |

| Protein | beta | se | z | p_val | HR |
|---|---|---|---|---|---|
| hpgds | -0.1054567125 | 0.05857983399 | -1.800222112 | 0.07182557367 | 0.899913427 |
| scn3a | 0.1139676142 | 0.06336049367 | 1.79871727 | 0.07206341566 | 1.120715829 |
| pdzd2 | 0.1165067832 | 0.06478580549 | 1.798338114 | 0.07212344338 | 1.123565132 |
| gchfr | 0.122406311 | 0.06833654522 | 1.791227674 | 0.07325676697 | 1.130213227 |
| bambi | -0.1070763064 | 0.0598627868 | -1.788695651 | 0.07366384377 | 0.8984571123 |
| brd3 | -0.1144914019 | 0.06401270516 | -1.78857309 | 0.07368359493 | 0.8918196064 |
| myh7b | 0.1138169757 | 0.06375598536 | 1.785196716 | 0.07422941443 | 1.120547019 |
| anpep | 0.1039903723 | 0.0584651079 | 1.778674085 | 0.0752932102 | 1.109589772 |
| psme2 | 0.1068182845 | 0.06007849165 | 1.7779788 | 0.07540733686 | 1.112732035 |
| atp1b1 | 0.1125194813 | 0.06333597197 | 1.776549373 | 0.07564241171 | 1.119094058 |
| cd302 | 0.1122858848 | 0.0632109617 | 1.776367292 | 0.07567239856 | 1.118832672 |
| lrp1 | 0.1094562162 | 0.0616697539 | 1.774876811 | 0.07591822993 | 1.115671221 |
| blnk | 0.1156386436 | 0.06540463513 | 1.76804967 | 0.07705260039 | 1.122590144 |
| brd2 | -0.1121252545 | 0.06365097109 | -1.761563926 | 0.07814300336 | 0.8939322814 |
| cat | 0.1148675655 | 0.06556157651 | 1.752056184 | 0.0797641475 | 1.121724873 |
| gstt2b | 0.1106314993 | 0.06317813146 | 1.751104325 | 0.07992794134 | 1.116983222 |
| gfra3 | 0.1054458964 | 0.06040933355 | 1.745523254 | 0.08089382832 | 1.111205983 |
| myom2 | -0.1133894211 | 0.06502990395 | -1.743650447 | 0.08122006095 | 0.8928029161 |
| aprt | 0.1032622798 | 0.05923369079 | 1.743303151 | 0.0812806752 | 1.108782182 |
| ceacam6 | -0.1125176795 | 0.0645608566 | -1.742815778 | 0.08136579935 | 0.8935815489 |
| optc | 0.1097743008 | 0.0630532652 | 1.740977258 | 0.08168756492 | 1.116026156 |
| atg4a | 0.1018129596 | 0.05854465678 | 1.739064933 | 0.08202334197 | 1.107176366 |
| adra2a | -0.11078294 | 0.06380479249 | -1.736279294 | 0.0825144626 | 0.8951330254 |
| fgfr4 | 0.1077415828 | 0.06208762249 | 1.735315003 | 0.08268502583 | 1.113759893 |
| fgf6 | 0.1096207392 | 0.06318379781 | 1.734950145 | 0.08274963616 | 1.11585479 |
| myh4 | -0.110785728 | 0.0640512075 | -1.729643083 | 0.08369406358 | 0.8951305298 |
| nudt15 | 0.1106557992 | 0.06402393703 | 1.728350432 | 0.08392541652 | 1.117010365 |
| ccl25 | 0.10445647 | 0.06053726049 | 1.725490534 | 0.08443910816 | 1.11010707 |
| slc39a14 | 0.1004935835 | 0.05831174381 | 1.723384981 | 0.0848189291 | 1.105716547 |
| skiv2l | -0.11087796 | 0.0643587462 | -1.722811064 | 0.08492269727 | 0.8950479139 |
| agbl2 | -0.1096297713 | 0.06370172717 | -1.720985854 | 0.08525339084 | 0.8961658602 |
| serpina1 | 0.1068520481 | 0.06218932642 | 1.718173427 | 0.08576498684 | 1.112769606 |
| epcam | -0.1014102816 | 0.05924720539 | -1.711646666 | 0.08696180291 | 0.9035622419 |
| fgfbp2 | 0.108318749 | 0.06334590889 | 1.709956505 | 0.08727391641 | 1.114402904 |
| stx3 | 0.107066705 | 0.06265897877 | 1.708720875 | 0.08750266552 | 1.113008495 |
| batf | -0.1085649985 | 0.0635844412 | -1.707414525 | 0.08774503258 | 0.8971205815 |
| tspan8 | -0.1107168863 | 0.06486897601 | -1.706777154 | 0.08786348024 | 0.8951921542 |
| scarf2 | 0.1124890319 | 0.06593537826 | 1.706049693 | 0.08799882778 | 1.119059983 |
| cpq | 0.1080888175 | 0.06335928223 | 1.705966571 | 0.08801430379 | 1.114146697 |
| sh3glb2 | 0.1082551458 | 0.06349242114 | 1.705008942 | 0.08819275671 | 1.114332026 |
| ntprobnp | -0.1048427448 | 0.06149453978 | -1.704911448 | 0.08821094101 | 0.9004661144 |
| cdhr1 | 0.09985468163 | 0.058707229 | 1.700892434 | 0.0889631876 | 1.105010328 |
| serping1 | 0.111230711 | 0.06559177954 | 1.69580261 | 0.08992326799 | 1.117652732 |
| scg2 | -0.09891205816 | 0.05834454469 | -1.695309453 | 0.09001673247 | 0.9058223642 |
| snap25 | 0.1085838532 | 0.06409890124 | 1.694004906 | 0.09026435083 | 1.114698376 |
| tnfsf11 | -0.1000637302 | 0.05920010294 | -1.690262773 | 0.0909776937 | 0.9047797544 |
| afm | 0.1145137655 | 0.06775638159 | 1.690080886 | 0.09101248105 | 1.121328077 |
| igbp1 | 0.106993626 | 0.06336004267 | 1.688660889 | 0.09128443481 | 1.112927161 |
| edn1 | 0.1109464387 | 0.0657198662 | 1.688172011 | 0.09137821427 | 1.117335059 |
| msln | -0.1015534862 | 0.06028078703 | -1.684674192 | 0.09205144846 | 0.9034328569 |
| timd4 | 0.1000180191 | 0.05941584439 | 1.683356016 | 0.0923061926 | 1.105190832 |
| clu | 0.1079194917 | 0.06441229364 | 1.675448669 | 0.09384623435 | 1.113958059 |
| gusb | -0.1098734873 | 0.06560833748 | -1.674687876 | 0.09399548742 | 0.8959474768 |
| cela3a | 0.09539900321 | 0.05699053355 | 1.673944729 | 0.09414146246 | 1.10009771 |
| vmo1 | -0.09777665647 | 0.05847161161 | -1.672207312 | 0.09448344913 | 0.9068514205 |
| skap1 | -0.09861635356 | 0.05898254392 | -1.671958295 | 0.09453254624 | 0.9060902597 |
| hbq1 | 0.09766326271 | 0.05842130196 | 1.671706371 | 0.09458223717 | 1.102591439 |
| mcee | 0.1046415098 | 0.06278405555 | 1.666689239 | 0.09557621375 | 1.110312503 |
| lgals7_lgals7b | 0.1020043676 | 0.06120854347 | 1.66650539 | 0.09561279549 | 1.107388308 |
| il17ra | 0.09624557068 | 0.05778804886 | 1.665492651 | 0.09581450866 | 1.101029411 |
| kitlg | 0.09786618587 | 0.05888631266 | 1.661951334 | 0.09652253274 | 1.102815203 |
| lilra3 | 0.1047428 | 0.06305147884 | 1.661226698 | 0.0966679254 | 1.110424972 |
| dnph1 | 0.09714631962 | 0.05858373279 | 1.65824735 | 0.09726755125 | 1.102021609 |
| elavl4 | 0.1082677441 | 0.06531285369 | 1.65767897 | 0.09738228065 | 1.114346065 |
| dbh | 0.1064757292 | 0.06433909966 | 1.654914815 | 0.09794177864 | 1.112350928 |
| prkd2 | 0.1043994762 | 0.06323631392 | 1.650941836 | 0.09875045205 | 1.110043802 |
| itgax | -0.1049203724 | 0.06355506757 | -1.650857696 | 0.09876763567 | 0.9003962161 |
| timp4 | 0.1014302409 | 0.06144712824 | 1.650691315 | 0.09880162205 | 1.10675271 |
| hyal1 | 0.09713603946 | 0.05889402934 | 1.649335944 | 0.09907882984 | 1.10201028 |
| s100p | -0.1004001166 | 0.06105310056 | -1.644472036 | 0.100078736 | 0.90447545 |

| Protein | beta | se | z | p_val | HR |
|---|---|---|---|---|---|
| prtg | 0.09622101089 | 0.05854847348 | 1.643441838 | 0.1002915498 | 1.101002371 |
| pcbd1 | 0.1151694319 | 0.07008085339 | 1.643379416 | 0.1003044562 | 1.122063535 |
| lmnb2 | 0.1123120733 | 0.0684402441 | 1.641023857 | 0.1007924631 | 1.118861973 |
| polr2f | 0.1031495428 | 0.06288149778 | 1.640379864 | 0.1009262094 | 1.108657188 |
| klk7 | 0.1071829275 | 0.06541642523 | 1.638471182 | 0.1013234409 | 1.113137859 |
| plcb1 | -0.10605708 | 0.06473284155 | -1.638381344 | 0.1013421683 | 0.8993733104 |
| fnta | 0.1077976069 | 0.06579752172 | 1.638323208 | 0.1013542888 | 1.113822293 |
| scgn | 0.09783548502 | 0.05983609759 | 1.635057916 | 0.1020369072 | 1.102781346 |
| sox9 | 0.1029825892 | 0.06307258867 | 1.632763001 | 0.1025188514 | 1.108472109 |
| ccar2 | 0.1020464547 | 0.06250000612 | 1.632743115 | 0.1025230355 | 1.107434916 |
| matn2 | 0.0988915829 | 0.06072204052 | 1.628594528 | 0.1033988835 | 1.103946606 |
| ptprb | 0.1077126328 | 0.06616282945 | 1.627993145 | 0.1035263393 | 1.113727651 |
| cyp24a1 | -0.1030722471 | 0.06342085238 | -1.625210688 | 0.1041176738 | 0.9020617997 |
| f12 | 0.1041086557 | 0.06420149522 | 1.621592384 | 0.1048906545 | 1.109721026 |
| chac2 | 0.09506011003 | 0.05872078065 | 1.618849562 | 0.1054796353 | 1.099724958 |
| f13b | 0.1032889876 | 0.06385087885 | 1.617659608 | 0.1057359754 | 1.108811796 |
| ccdc80 | 0.1043997988 | 0.06456875432 | 1.616878006 | 0.1059046168 | 1.11004416 |
| flt1 | 0.0961335751 | 0.05949425363 | 1.615846392 | 0.1061275284 | 1.100906108 |
| vit | 0.1137251817 | 0.0705009994 | 1.613100278 | 0.1067227236 | 1.120444164 |
| exosc10 | -0.1021868599 | 0.063356401 | -1.612889278 | 0.1067685651 | 0.9028608275 |
| il1r1 | 0.09493678364 | 0.0589003556 | 1.611820212 | 0.1070010686 | 1.099589341 |
| heg1 | 0.1067417543 | 0.06623416661 | 1.611581451 | 0.1070530498 | 1.112646881 |
| tprkb | 0.10306336 | 0.06397057847 | 1.611105643 | 0.1071566986 | 1.108561645 |
| septin8 | 0.1099442938 | 0.06864515462 | 1.601632256 | 0.1092369537 | 1.116215889 |
| fcn1 | 0.104182779 | 0.06519940895 | 1.597909868 | 0.1100630376 | 1.109803285 |
| h2ap | 0.1012374943 | 0.06339444126 | 1.59694592 | 0.1102777627 | 1.106539407 |
| podxl | 0.09346079042 | 0.05852611938 | 1.596907354 | 0.1102863604 | 1.097967552 |
| vta1 | 0.09184174791 | 0.05752792304 | 1.596472514 | 0.1103833377 | 1.096191334 |
| adgrd1 | 0.1129855208 | 0.07094425909 | 1.592595684 | 0.1112509224 | 1.119615722 |
| kctd5 | 0.1055427555 | 0.06629951582 | 1.591908391 | 0.11140529 | 1.111313618 |
| s100a14 | -0.09772433477 | 0.06150351561 | -1.588922744 | 0.1120778357 | 0.9068988698 |
| il12b | -0.09392628818 | 0.05918190726 | -1.58707775 | 0.1124950366 | 0.9103498633 |
| tmprss11b | -0.1001520924 | 0.06314000643 | -1.586190722 | 0.1126960517 | 0.9046998096 |
| kiaa1549 | -0.09892270977 | 0.06263329352 | -1.579394986 | 0.1142454866 | 0.9058127158 |
| mcts1 | 0.0982567075 | 0.06230864127 | 1.576935486 | 0.1148103703 | 1.10324596 |
| pcdh9 | 0.1012998657 | 0.06424621339 | 1.576744532 | 0.1148543192 | 1.106608426 |
| il1rl2 | 0.09824278039 | 0.06249749277 | 1.571947546 | 0.1159627166 | 1.103230595 |
| mmp8 | 0.09291372943 | 0.05923350205 | 1.568600981 | 0.1167409437 | 1.097367061 |
| chga | 0.09808108766 | 0.06257272836 | 1.567473406 | 0.1170040776 | 1.103052225 |
| megf11 | 0.09970521424 | 0.0636089321 | 1.567471909 | 0.1170044272 | 1.104845177 |
| plxdc1 | -0.09298088153 | 0.05933534378 | -1.567040411 | 0.117105246 | 0.9112109211 |
| qsox1 | 0.1015341394 | 0.06488824134 | 1.564754064 | 0.1176405863 | 1.106867706 |
| eif4ebp1 | 0.09355036195 | 0.05979170182 | 1.564604437 | 0.117675688 | 1.098065903 |
| slc9a3r2 | 0.1015496033 | 0.06495204707 | 1.563455008 | 0.1179456103 | 1.106884822 |
| acyp1 | 0.09877060026 | 0.06322043307 | 1.562320843 | 0.1182124244 | 1.103813056 |
| gstm4 | -0.09857385907 | 0.0631037767 | -1.562091276 | 0.1182664878 | 0.9061287643 |
| chl1 | -0.09358267292 | 0.05993480154 | -1.561407905 | 0.1184275378 | 0.9106627272 |
| b3gat3 | 0.09987309084 | 0.06399262796 | 1.560696818 | 0.1185953024 | 1.105030671 |
| ccl21 | -0.09321361254 | 0.05980880788 | -1.558526509 | 0.119108489 | 0.9109988787 |
| clec4a | 0.09205577852 | 0.05917970353 | 1.555529566 | 0.1198199988 | 1.096425977 |
| ddx39a | 0.1004524071 | 0.06457853631 | 1.555507647 | 0.1198252147 | 1.105671018 |
| ak1 | 0.09311910781 | 0.06012039911 | 1.548877073 | 0.1214112733 | 1.097592459 |
| lgals9 | -0.104288521 | 0.06735263997 | -1.548395446 | 0.121527117 | 0.9009653125 |
| pqbp1 | 0.09180681415 | 0.05929381292 | 1.548337164 | 0.1215411411 | 1.09615304 |
| rgs8 | 0.09006504726 | 0.05819480129 | 1.547647647 | 0.1217071532 | 1.094245459 |
| pros1 | 0.09971355035 | 0.06444843772 | 1.547183359 | 0.1218190376 | 1.104854388 |
| erbb2 | 0.0992891673 | 0.06426947684 | 1.54488837 | 0.1223732671 | 1.104385606 |
| ube2b | 0.09793532699 | 0.06341387586 | 1.544383239 | 0.1224955181 | 1.102891455 |
| gm2a | 0.1036914144 | 0.06716500704 | 1.543830917 | 0.1226292993 | 1.109258101 |
| slk | -0.09836307731 | 0.06372869243 | -1.543466115 | 0.1227177229 | 0.9063197799 |
| ncam2 | -0.09291812538 | 0.06054433172 | -1.534712214 | 0.1248545343 | 0.911268107 |
| nos2 | -0.09828089191 | 0.06417405882 | -1.531473834 | 0.1256523285 | 0.9063942692 |
| atxn3 | 0.0964981617 | 0.06302965765 | 1.530996126 | 0.12577035 | 1.101307557 |
| col15a1 | 0.101729961 | 0.06647716644 | 1.530299296 | 0.1259426624 | 1.107084475 |
| rbfox3 | 0.1111935455 | 0.07271504363 | 1.52916838 | 0.1262227072 | 1.117611194 |
| prkab1 | 0.0913993628 | 0.05977826323 | 1.528973206 | 0.1262710863 | 1.095706502 |
| mtr | 0.09797353016 | 0.06411637229 | 1.528057915 | 0.1264981593 | 1.10293359 |
| fcer1a | -0.0956744817 | 0.06270008095 | -1.525906829 | 0.1270330698 | 0.9087597859 |
| sod1 | 0.09114038979 | 0.05976096954 | 1.525082182 | 0.1272386009 | 1.095422781 |
| cd8a | -0.08793411 | 0.05770714578 | -1.523799329 | 0.1275588473 | 0.9158212182 |

| Protein | beta | se | z | p_val | HR |
|---|---|---|---|---|---|
| casp9 | 0.0981602324 | 0.06443966454 | 1.523289004 | 0.127686417 | 1.10313953 |
| gas6 | 0.09178968087 | 0.06032890248 | 1.521487663 | 0.1281375049 | 1.09613426 |
| cd244 | 0.08729932198 | 0.05740451333 | 1.520774534 | 0.1283164272 | 1.091223258 |
| sprr3 | 0.1031568632 | 0.06793099272 | 1.518553742 | 0.1288748623 | 1.108665304 |
| acan | 0.09657940449 | 0.06361937608 | 1.518081604 | 0.128993828 | 1.101397034 |
| tpm3 | -0.09655435332 | 0.0636258478 | -1.517533466 | 0.1291320508 | 0.9079605456 |
| clic5 | 0.09562510895 | 0.06310896653 | 1.515238075 | 0.1297121241 | 1.100346477 |
| psap | 0.1015287332 | 0.06703444326 | 1.514575616 | 0.1298799111 | 1.106861722 |
| park7 | 0.08949116815 | 0.05910381765 | 1.514135156 | 0.1299915637 | 1.093617675 |
| usp28 | 0.09814927635 | 0.06482778953 | 1.514000046 | 0.1300258277 | 1.103127444 |
| hsd11b1 | -0.09281904442 | 0.06136664409 | -1.512532513 | 0.1303984478 | 0.9113584008 |
| nrn1 | 0.09483233518 | 0.06284357086 | 1.509022067 | 0.1312931437 | 1.099474496 |
| cxcl6 | -0.08781103624 | 0.05819208482 | -1.508985913 | 0.1313023828 | 0.9159339387 |
| scn2a | 0.09509616079 | 0.0631179479 | 1.506642151 | 0.1319024069 | 1.099764604 |
| ppl | 0.1004687396 | 0.06671741001 | 1.505884889 | 0.1320967263 | 1.105689077 |
| kif1c | 0.1016556646 | 0.06753590921 | 1.505209092 | 0.1322703284 | 1.107002226 |
| wfdc2 | -0.1041382948 | 0.06925009679 | -1.503799989 | 0.1326328741 | 0.9011006713 |
| gage2a | -0.09612747878 | 0.06404016862 | -1.501049745 | 0.1333426956 | 0.9083482136 |
| ddc | 0.09019140674 | 0.06010893701 | 1.500465841 | 0.1334937754 | 1.094383736 |
| kir2dl2 | 0.09401537904 | 0.06267411714 | 1.500067066 | 0.1335970308 | 1.098576641 |
| s100a13 | 0.1011875307 | 0.0674568695 | 1.500033006 | 0.1336058531 | 1.106484122 |
| chek2 | 0.08943732905 | 0.05970529659 | 1.497979813 | 0.1341384953 | 1.093558797 |
| sftpd | 0.09086029059 | 0.06066846533 | 1.497652695 | 0.1342235083 | 1.095115997 |
| cptp | 0.09546906399 | 0.06376081763 | 1.497299871 | 0.1343152486 | 1.100174786 |
| clps | 0.085761643 | 0.05733815185 | 1.495716905 | 0.1347274432 | 1.089546596 |
| ctrc | 0.08574610007 | 0.05734272738 | 1.495326504 | 0.1348292513 | 1.089529662 |
| arid3a | -0.09478589233 | 0.06365038427 | -1.489164495 | 0.1364440578 | 0.909567659 |
| gfra1 | 0.09275778407 | 0.06231425442 | 1.488548406 | 0.1366063265 | 1.097195945 |
| nudt2 | 0.08696029539 | 0.05845206677 | 1.487719771 | 0.1368248113 | 1.090853367 |
| flt3 | -0.08911137004 | 0.05990041488 | -1.487658645 | 0.136840939 | 0.9147436929 |
| taldo1 | 0.09726129314 | 0.06540103636 | 1.48715217 | 0.1369746253 | 1.10214832 |
| ggct | 0.09466602608 | 0.06371095616 | 1.485867295 | 0.1373142259 | 1.099291659 |
| chrdl2 | -0.09467872658 | 0.06373472591 | -1.485512415 | 0.1374081373 | 0.9096651388 |
| myo9b | 0.08651964246 | 0.0582757462 | 1.484659539 | 0.137634035 | 1.090372785 |
| clstn3 | 0.09499985656 | 0.0640439566 | 1.483353959 | 0.1379803931 | 1.099658697 |
| ctrb1 | 0.08447880218 | 0.05697902879 | 1.482629732 | 0.1381728132 | 1.088149778 |
| preb | 0.08678637193 | 0.05861384785 | 1.480646214 | 0.1387008735 | 1.090663658 |
| serpinf1 | 0.1073540732 | 0.07256284164 | 1.479463466 | 0.1390164886 | 1.113328384 |
| dtx2 | 0.0949125779 | 0.06429208879 | 1.476271493 | 0.1398710238 | 1.099562725 |
| enox2 | 0.09451605729 | 0.06403791848 | 1.475938936 | 0.1399602857 | 1.099126812 |
| rnf168 | 0.09449461552 | 0.06406704023 | 1.47493337 | 0.1402304578 | 1.099103245 |
| psca | 0.09058305374 | 0.06141968999 | 1.474821084 | 0.1402606513 | 1.094812432 |
| ccn4 | 0.08925474661 | 0.06052929346 | 1.474571096 | 0.1403278904 | 1.09335915 |
| thy1 | 0.08950691014 | 0.06084004137 | 1.471184242 | 0.1412412976 | 1.09363489 |
| fcamr | 0.102431435 | 0.0697677096 | 1.468178267 | 0.142055806 | 1.107861339 |
| nid2 | 0.08463435709 | 0.05782684319 | 1.463582524 | 0.1433080505 | 1.088319058 |
| il10 | 0.08489598529 | 0.05801618306 | 1.463315593 | 0.1433810431 | 1.08860383 |
| snrpb2 | 0.09596086705 | 0.06561061681 | 1.462581389 | 0.1435819589 | 1.100715989 |
| ddah1 | 0.08796840711 | 0.06025723888 | 1.459881149 | 0.1443227411 | 1.091953624 |
| prss8 | 0.09980375479 | 0.06842216664 | 1.458646513 | 0.1446624246 | 1.104954055 |
| adam8 | 0.08648368772 | 0.05930090065 | 1.458387424 | 0.1447337852 | 1.090333582 |
| vegfd | -0.08812480588 | 0.0605353317 | -1.455758206 | 0.1454594731 | 0.9156465915 |
| fh | -0.09215105457 | 0.06331302432 | -1.455483379 | 0.1455354884 | 0.9119673823 |
| lyzl2 | 0.1042434097 | 0.07176872193 | 1.45249082 | 0.1463651799 | 1.109870575 |
| ca3 | 0.08842955807 | 0.06098391606 | 1.450047222 | 0.1470453513 | 1.092457295 |
| ccl23 | -0.08642976621 | 0.05960941355 | -1.449934852 | 0.1470766874 | 0.917199965 |
| ca6 | -0.0924329812 | 0.06375390182 | -1.449840379 | 0.1471030363 | 0.9117103106 |
| palm | 0.1001789444 | 0.06911499197 | 1.449453173 | 0.1472110684 | 1.1053687 |
| bcam | 0.08580042787 | 0.05923949304 | 1.44836533 | 0.1475149057 | 1.089588855 |
| kir3dl1 | -0.08227424081 | 0.05682718118 | -1.447797323 | 0.1476737416 | 0.921019343 |
| ssb | -0.08373068108 | 0.05784665961 | -1.447459225 | 0.1477683486 | 0.9196789097 |
| abraxas2 | 0.09278782825 | 0.06419152697 | 1.445484048 | 0.1483219705 | 1.09722891 |
| psmg4 | 0.09432532801 | 0.06525646521 | 1.445455675 | 0.1483299346 | 1.098917196 |
| ube2z | 0.09264601187 | 0.06423839983 | 1.442221664 | 0.1492398511 | 1.097073316 |
| nop56 | -0.09162261733 | 0.0635348918 | -1.442083472 | 0.1492788272 | 0.9124494272 |
| urod | 0.09092437657 | 0.06306379123 | 1.441784181 | 0.1493632669 | 1.09518618 |
| btc | 0.08369528216 | 0.05810289368 | 1.440466677 | 0.1497354113 | 1.087297524 |
| gpa33 | -0.08426143601 | 0.058804047 | -1.432918996 | 0.1518809878 | 0.9191909151 |
| asah2 | 0.08796172151 | 0.06141980754 | 1.432139322 | 0.152103953 | 1.091946323 |
| thop1 | 0.08782591229 | 0.06142838211 | 1.42972856 | 0.15279494 | 1.091798037 |

| Protein | beta | se | z | p_val | HR |
|---|---|---|---|---|---|
| nmrk2 | 0.09084945985 | 0.06374182912 | 1.425272244 | 0.15407852 | 1.095104136 |
| gcg | -0.08385287177 | 0.05888525339 | -1.424004601 | 0.1544451402 | 0.9195665403 |
| pfdn4 | -0.09132859723 | 0.06414083996 | -1.423875916 | 0.1544823948 | 0.9127177451 |
| dsg4 | 0.08568919308 | 0.06018456206 | 1.423773641 | 0.1545120082 | 1.089467662 |
| cryzl1 | 0.09101287961 | 0.06408600596 | 1.420167761 | 0.1555588468 | 1.095283112 |
| spag1 | 0.09121217446 | 0.06425081596 | 1.41962671 | 0.1557163852 | 1.095501418 |
| sord | 0.08744411735 | 0.06166152327 | 1.418130995 | 0.1561525236 | 1.091381273 |
| fcrl6 | 0.08230716061 | 0.05804242479 | 1.418051725 | 0.1561756638 | 1.08578927 |
| plg | 0.09481601905 | 0.06690917967 | 1.417085361 | 0.1564579719 | 1.099456557 |
| nid1 | 0.08465332587 | 0.05977844697 | 1.416117851 | 0.1567410024 | 1.088339702 |
| ces1 | -0.08760830824 | 0.06191103954 | -1.415067634 | 0.1570486668 | 0.916119643 |
| padi2 | 0.08385189042 | 0.05933885068 | 1.413102705 | 0.1576255279 | 1.087467817 |
| cdh23 | 0.08943606296 | 0.06330239779 | 1.412838472 | 0.1577032235 | 1.093557412 |
| smtn | 0.09016889648 | 0.06382129499 | 1.412834016 | 0.1577045341 | 1.094359101 |
| prr5 | -0.09072350196 | 0.06437504131 | -1.409296214 | 0.158747598 | 0.9132701934 |
| mep1a | 0.09398077653 | 0.06676061424 | 1.407727859 | 0.1592116693 | 1.098538628 |
| ctsv | 0.08464211902 | 0.06022769062 | 1.405368829 | 0.159911631 | 1.088327505 |
| sel1l | -0.0945571638 | 0.06730975464 | -1.404806247 | 0.160078901 | 0.9097757269 |
| dnaja4 | 0.08936530086 | 0.06362312726 | 1.404604029 | 0.160139058 | 1.093480033 |
| rictor | -0.08988529727 | 0.06403658787 | -1.40365532 | 0.1604215132 | 0.9140360217 |
| serpina3 | 0.08924697803 | 0.0636313595 | 1.402562804 | 0.16074725 | 1.093350657 |
| mmut | -0.08956807347 | 0.06396342267 | -1.400301449 | 0.161423067 | 0.9143260217 |
| psip1 | 0.08360777231 | 0.05973209699 | 1.399712659 | 0.1615993815 | 1.087202379 |
| tfpi2 | 0.08662663384 | 0.06195845431 | 1.398140654 | 0.1620708338 | 1.090489452 |
| psme1 | 0.08396826504 | 0.06009914399 | 1.397162413 | 0.1623647368 | 1.087594378 |
| tdp1 | 0.09077265998 | 0.06506136711 | 1.395185254 | 0.1629599826 | 1.095020035 |
| mycbp2 | 0.0877092383 | 0.06292456133 | 1.393879217 | 0.1633540811 | 1.09167066 |
| fshb | -0.132466555 | 0.09508880611 | -1.393082534 | 0.1635948341 | 0.8759322292 |
| klf4 | 0.09038408766 | 0.06495845681 | 1.391413714 | 0.1641000083 | 1.094594623 |
| nfasc | 0.087014524 | 0.06257470043 | 1.390570365 | 0.1643557481 | 1.090912524 |
| itga6 | 0.08037644241 | 0.05794074405 | 1.387217988 | 0.1653753019 | 1.08369494 |
| rexo2 | -0.08781361767 | 0.06345263319 | -1.383923933 | 0.1663817477 | 0.9159315743 |
| ift20 | 0.08828546567 | 0.06379767624 | 1.383835131 | 0.1664089432 | 1.092299892 |
| dnajb2 | 0.087785749 | 0.06346924305 | 1.383122672 | 0.1666272552 | 1.091754188 |
| ifi30 | 0.09854876211 | 0.07144432057 | 1.379378533 | 0.1677780744 | 1.103568215 |
| cela2a | 0.08481759886 | 0.06150414917 | 1.37905491 | 0.1678778247 | 1.088518502 |
| acta2 | 0.09428528692 | 0.06840896646 | 1.378259193 | 0.1681232773 | 1.098873195 |
| ptges2 | 0.09074428921 | 0.06587810583 | 1.377457473 | 0.1683708542 | 1.094988969 |
| defb103a_defb103b | -0.08912546827 | 0.06472350828 | -1.3770185 | 0.1685065283 | 0.9147307967 |
| lypd1 | 0.07964630537 | 0.05784727621 | 1.376837608 | 0.1685624607 | 1.082903983 |
| tnfaip2 | 0.08879759844 | 0.06468958487 | 1.372672257 | 0.1698542577 | 1.092859437 |
| adgrb3 | 0.08078150432 | 0.05885346167 | 1.372587135 | 0.1698807337 | 1.084133992 |
| cit | 0.08731788656 | 0.0636252514 | 1.372377863 | 0.1699458377 | 1.091243516 |
| rilp | 0.07960396495 | 0.05804837105 | 1.37133848 | 0.1702694654 | 1.082858133 |
| nap1l4 | 0.0872590052 | 0.0636819842 | 1.370230628 | 0.1706149204 | 1.091179264 |
| mybpc2 | 0.09176328044 | 0.06701069537 | 1.369382603 | 0.1708797095 | 1.096105322 |
| b4gat1 | 0.08418849829 | 0.06155506829 | 1.367694012 | 0.1714078752 | 1.087833929 |
| tap1 | 0.08627474623 | 0.06308766792 | 1.367537414 | 0.1714569185 | 1.09010579 |
| galnt3 | -0.0804137486 | 0.05889186833 | -1.365447402 | 0.1721124741 | 0.9227344873 |
| adgrf5 | 0.08844911904 | 0.0648326768 | 1.364267579 | 0.1724833657 | 1.092478665 |
| c2cd2l | -0.07995819024 | 0.05862809741 | -1.363820314 | 0.1726241251 | 0.9231549425 |
| echdc3 | 0.08939078081 | 0.06555469919 | 1.363605995 | 0.1726916043 | 1.093507895 |
| vcam1 | -0.08103308729 | 0.05956712322 | -1.360365969 | 0.1737141421 | 0.9221631791 |
| il1r2 | 0.08058203643 | 0.05925715524 | 1.359870147 | 0.1738710193 | 1.083917764 |
| cd200 | 0.08203845011 | 0.06036590714 | 1.359019586 | 0.1741403824 | 1.085497547 |
| penk | -0.08611028975 | 0.06337960978 | -1.35864342 | 0.1742596093 | 0.9174930356 |
| crhr1 | 0.08017981575 | 0.05901839129 | 1.358556443 | 0.1742871854 | 1.083481877 |
| il12a_il12b | -0.08065661569 | 0.05943766909 | -1.356994931 | 0.1747828211 | 0.9225104127 |
| tgfb2 | -0.08562729857 | 0.06311438708 | -1.356700152 | 0.1748765041 | 0.9179362836 |
| dtymk | 0.08762327025 | 0.06461027912 | 1.356181578 | 0.1750414021 | 1.091576815 |
| lair1 | -0.08746492755 | 0.06453824279 | -1.35524185 | 0.1753405151 | 0.9162510063 |
| spaca5_spaca5b | -0.08653476287 | 0.06389446631 | -1.354338926 | 0.1756282728 | 0.9171036671 |
| bdnf | 0.08472816301 | 0.06258489459 | 1.353811708 | 0.1757964576 | 1.088421153 |
| fuca1 | -0.07734266965 | 0.05713812644 | -1.353608781 | 0.1758612241 | 0.9255726336 |
| umod | -0.08346990348 | 0.06177688825 | -1.351150986 | 0.1766470717 | 0.9199187726 |
| dscam | 0.08539819876 | 0.06323655578 | 1.350456199 | 0.1768696945 | 1.089150679 |
| dtnb | -0.09613292568 | 0.07125774737 | -1.349087352 | 0.1773089104 | 0.9083432659 |
| rabgap1l | 0.07949225736 | 0.05899189635 | 1.347511477 | 0.1778155597 | 1.082737176 |
| vash1 | 0.07820362421 | 0.0580480006 | 1.347223391 | 0.1779082967 | 1.081342824 |
| adam12 | 0.0950512867 | 0.07059542552 | 1.346422746 | 0.1781662193 | 1.099715254 |

| Protein | beta | se | z | p_val | HR |
|---|---|---|---|---|---|
| nfic | -0.08495573978 | 0.06310184147 | -1.346327426 | 0.1781969448 | 0.9185529389 |
| pak4 | -0.07787676322 | 0.05786215667 | -1.345901496 | 0.1783342864 | 0.9250784232 |
| ptprc | 0.08524942089 | 0.0633652426 | 1.345365651 | 0.1785071819 | 1.088988649 |
| pdgfc | -0.0795175984 | 0.05915339012 | -1.344261051 | 0.1788639856 | 0.9235617666 |
| bcat2 | 0.08497671659 | 0.06330026295 | 1.342438604 | 0.179453825 | 1.088691718 |
| fam3b | 0.0782632657 | 0.0583135462 | 1.342111238 | 0.1795599305 | 1.081407319 |
| lrtm1 | 0.08577115433 | 0.06394636944 | 1.341298264 | 0.1798236332 | 1.089556959 |
| cfi | 0.0956892522 | 0.0714424651 | 1.339389004 | 0.1804440671 | 1.100417059 |
| ngfr | -0.08519667783 | 0.06366977847 | -1.338102313 | 0.1808630869 | 0.9183316512 |
| nudt16 | 0.08537184971 | 0.06389779502 | 1.336068791 | 0.1815267898 | 1.089121981 |
| orm1 | 0.08646047067 | 0.06487272298 | 1.332770796 | 0.1826070321 | 1.090308268 |
| nrp1 | 0.07816743097 | 0.05866378986 | 1.332464731 | 0.1827075235 | 1.081303687 |
| dsg3 | 0.0816748526 | 0.061327666 | 1.331778265 | 0.1829330623 | 1.085102934 |
| dnajc9 | 0.08469212407 | 0.06365066405 | 1.330577227 | 0.1833281599 | 1.088381929 |
| cox5b | 0.0781326112 | 0.05884474061 | 1.327775607 | 0.1842522475 | 1.081266037 |
| oscar | -0.07731659446 | 0.05829265367 | -1.326352286 | 0.1847230354 | 0.9255967684 |
| hla_a | -0.08400641846 | 0.06333900917 | -1.326298273 | 0.1847409187 | 0.9194253548 |
| atp1b3 | 0.08465520881 | 0.06407084288 | 1.321275092 | 0.1864096561 | 1.088341751 |
| vat1 | 0.07808777458 | 0.05923431979 | 1.318286001 | 0.1874079253 | 1.081217558 |
| il12rb2 | -0.08532064839 | 0.06476846912 | -1.317317663 | 0.1877321669 | 0.9182178121 |
| pmch | 0.08391286409 | 0.06372801581 | 1.316734297 | 0.1879277031 | 1.087534126 |
| reg4 | -0.07965559139 | 0.06061513536 | -1.31412049 | 0.1888056598 | 0.9234343304 |
| clec4d | 0.07833484786 | 0.05963494588 | 1.313572884 | 0.1889899788 | 1.081484731 |
| hadh | -0.08328044769 | 0.06340997579 | -1.313365076 | 0.1890599597 | 0.9200930731 |
| mrpl52 | 0.08380510718 | 0.06385493876 | 1.312429529 | 0.1893752491 | 1.087416943 |
| mdh1 | 0.08352106593 | 0.06375137207 | 1.310106171 | 0.1901599213 | 1.087108116 |
| ung | 0.08396940029 | 0.06411959188 | 1.309574778 | 0.1903397256 | 1.087595613 |
| apoa2 | 0.08469485235 | 0.06467929793 | 1.309458437 | 0.190379108 | 1.088384898 |
| csnk2a1 | 0.08200029007 | 0.06273704257 | 1.307047427 | 0.1911966046 | 1.085456125 |
| casp7 | 0.08202074444 | 0.06276116393 | 1.30687099 | 0.1912565301 | 1.085478327 |
| tgfa | 0.07918572765 | 0.0606439061 | 1.30574913 | 0.1916378844 | 1.082405336 |
| snx2 | 0.08183642194 | 0.06272609094 | 1.304663191 | 0.1920075607 | 1.085278268 |
| cpox | 0.08467455323 | 0.065017785 | 1.302329097 | 0.1928039091 | 1.088362805 |
| colec12 | -0.08677268051 | 0.06666002666 | -1.301719859 | 0.1930121683 | 0.9168854979 |
| bola2_bola2b | 0.08445020517 | 0.06496565954 | 1.299920693 | 0.193628152 | 1.08811866 |
| sele | -0.08060316172 | 0.06205322394 | -1.298935923 | 0.1939659202 | 0.9225597258 |
| il19 | -0.07905945982 | 0.06088039407 | -1.298602958 | 0.1940802223 | 0.9239849828 |
| ampd3 | 0.08181254627 | 0.0630446643 | 1.297691838 | 0.1943932496 | 1.085252356 |
| robo4 | -0.08258578652 | 0.06364337427 | -1.297633689 | 0.1944132399 | 0.920732448 |
| serpini2 | 0.08044523912 | 0.06206179477 | 1.296211936 | 0.1949024786 | 1.083769497 |
| eif4e | 0.08363335226 | 0.06454664614 | 1.295704072 | 0.1950774578 | 1.08723019 |
| erbb4 | 0.07843191013 | 0.06070627763 | 1.291990107 | 0.1963605707 | 1.081589707 |
| prap1 | 0.09527974593 | 0.07376312969 | 1.291698798 | 0.196461474 | 1.099966523 |
| pten | 0.07490149555 | 0.05801965348 | 1.290967647 | 0.1967148963 | 1.07777798 |
| ccl16 | -0.08058560595 | 0.06245509636 | -1.290296719 | 0.1969476555 | 0.9225759222 |
| nectin4 | 0.07499380814 | 0.05815386736 | 1.289575596 | 0.1971980536 | 1.077877477 |
| rgmb | -0.07611206994 | 0.05906992271 | -1.288508033 | 0.1975691743 | 0.9267123441 |
| ercc1 | 0.08226726818 | 0.0638771216 | 1.287898799 | 0.1977811937 | 1.085745956 |
| crym | 0.08215953451 | 0.06380949615 | 1.287575353 | 0.1978938237 | 1.085628991 |
| sptlc1 | 0.08110248125 | 0.06301036351 | 1.287129239 | 0.1980492456 | 1.08448203 |
| bhmt2 | -0.08144726487 | 0.0634475361 | -1.283694685 | 0.1992488048 | 0.9217813188 |
| nudt5 | 0.07580517096 | 0.05920521968 | 1.280379861 | 0.2004115731 | 1.078752381 |
| crygd | 0.0803382641 | 0.06277434104 | 1.279794623 | 0.2006173755 | 1.083653567 |
| sit1 | -0.07664670416 | 0.05991038745 | -1.279355842 | 0.2007717765 | 0.9262170244 |
| nsfl1c | 0.07517344853 | 0.05876322692 | 1.279260049 | 0.2008054963 | 1.078071125 |
| smarca2 | 0.0743265777 | 0.0582035699 | 1.277010634 | 0.201598493 | 1.077158524 |
| c5 | 0.08249966605 | 0.06470484534 | 1.275015273 | 0.2023038362 | 1.085998311 |
| gipc2 | 0.08543116797 | 0.0670175438 | 1.274758267 | 0.2023948163 | 1.089186588 |
| scrg1 | 0.09185084647 | 0.07212218119 | 1.273545045 | 0.202824699 | 1.096201308 |
| ncan | -0.0820330191 | 0.06442450436 | -1.273320143 | 0.2029044618 | 0.9212415396 |
| cyb5a | -0.08114880748 | 0.06374577261 | -1.273006886 | 0.2030155984 | 0.9220564723 |
| pepd | -0.07905719634 | 0.06211975539 | -1.272657882 | 0.2031394693 | 0.9239870743 |
| unc5d | 0.08214977274 | 0.06460649184 | 1.271540528 | 0.2035364189 | 1.085618394 |
| nedd9 | 0.08210625623 | 0.06460835232 | 1.270830369 | 0.2037890022 | 1.085571152 |
| ceacam16 | 0.08304120816 | 0.06536725066 | 1.270379392 | 0.2039495203 | 1.086586584 |
| eif4g3 | 0.07963209858 | 0.06270448986 | 1.269958479 | 0.2040994206 | 1.082888598 |
| ctse | 0.08070320418 | 0.06370165038 | 1.266893459 | 0.205193388 | 1.084049108 |
| rasa1 | 0.07341388951 | 0.05795491347 | 1.266741422 | 0.2052477636 | 1.076175863 |
| arg2 | 0.08020582774 | 0.06335579523 | 1.26595882 | 0.205527826 | 1.083510061 |
| lhpp | 0.07483363499 | 0.05920156401 | 1.264048277 | 0.2062126996 | 1.077704844 |

| Protein | beta | se | z | p_val | HR |
|---|---|---|---|---|---|
| amigo2 | 0.07351814679 | 0.05820562178 | 1.263076393 | 0.2065617271 | 1.076288068 |
| lgals3 | -0.07834910483 | 0.0620614226 | -1.262444552 | 0.2067888666 | 0.9246415733 |
| tmem132a | -0.08055863689 | 0.06385396061 | -1.26160752 | 0.2070900489 | 0.9226008036 |
| hcg22 | -0.08082829629 | 0.06412273625 | -1.260524753 | 0.2074801242 | 0.9223520491 |
| serpinf2 | 0.08295287346 | 0.06600642612 | 1.256739356 | 0.208848032 | 1.086490605 |
| stc2 | -0.07901055516 | 0.06290722075 | -1.255985469 | 0.2091212385 | 0.9240301711 |
| pebp1 | 0.07482879627 | 0.05965618916 | 1.254334166 | 0.2097205705 | 1.077699629 |
| tbc1d17 | 0.07356118861 | 0.05869650751 | 1.253246432 | 0.2101160368 | 1.076334394 |
| cend1 | -0.08021419704 | 0.06406467959 | -1.252081452 | 0.2105401856 | 0.9229186388 |
| ggt5 | 0.07414772644 | 0.05926578959 | 1.251105013 | 0.210896167 | 1.07696589 |
| spink4 | 0.07455318881 | 0.05965209739 | 1.249799958 | 0.2113726315 | 1.077402648 |
| tnni3 | 0.07376162795 | 0.05904098525 | 1.249329218 | 0.2115446854 | 1.076550155 |
| lcn15 | 0.08053424006 | 0.06449842007 | 1.248623454 | 0.2118028297 | 1.083865958 |
| kiaa0319 | 0.08058644928 | 0.06455105282 | 1.248414174 | 0.211879421 | 1.083922547 |
| pagr1 | 0.08051819514 | 0.06451380998 | 1.248076887 | 0.2120029015 | 1.083848567 |
| mprip | -0.08013575954 | 0.06423288462 | -1.247581516 | 0.2121843512 | 0.922991033 |
| mtpn | 0.07328199219 | 0.05878210767 | 1.246671736 | 0.2125178866 | 1.076033927 |
| mgmt | 0.0726560947 | 0.05828535968 | 1.246558228 | 0.2125595261 | 1.075360651 |
| maneal | 0.07970843208 | 0.06395498024 | 1.246320955 | 0.2126465876 | 1.082971262 |
| fgf5 | 0.07888129881 | 0.06330727572 | 1.246006844 | 0.2127618827 | 1.082075871 |
| tp53bp1 | -0.08137173215 | 0.06531686013 | -1.245799813 | 0.2128378981 | 0.9218509461 |
| tmed10 | 0.08020283122 | 0.06439378511 | 1.245505775 | 0.2129458941 | 1.083506814 |
| nme3 | -0.07270474534 | 0.05840084309 | -1.244926297 | 0.2131588434 | 0.9298753395 |
| cpvl | -0.07167972662 | 0.05760950266 | -1.244234429 | 0.2134132961 | 0.9308289678 |
| efcab2 | 0.07969515092 | 0.06416732714 | 1.241989568 | 0.214240411 | 1.082956879 |
| hnf1a | 0.07899691253 | 0.06370843628 | 1.239975695 | 0.2149843842 | 1.082200981 |
| cfb | 0.08577234924 | 0.06929700085 | 1.237749804 | 0.2158088459 | 1.089558261 |
| smpdl3a | -0.07182553261 | 0.05808965288 | -1.236460007 | 0.2162876228 | 0.9306932573 |
| kit | -0.07414600276 | 0.06004540763 | -1.234832199 | 0.2168929611 | 0.9285361151 |
| crisp3 | 0.07802174642 | 0.06319639731 | 1.234591681 | 0.2169825065 | 1.081146169 |
| lepr | -0.0772450541 | 0.06259168407 | -1.234110493 | 0.217161734 | 0.9256629883 |
| prkcq | 0.07210678741 | 0.05846437468 | 1.233345739 | 0.2174467999 | 1.07477011 |
| bid | 0.07318424546 | 0.05935341965 | 1.233024919 | 0.2175664673 | 1.075928754 |
| lgals3bp | 0.08227604472 | 0.06700489344 | 1.227910985 | 0.2194803853 | 1.085755486 |
| crtam | -0.07246937943 | 0.05905429627 | -1.227165236 | 0.2197604927 | 0.9300942262 |
| arhgef1 | 0.07742154991 | 0.06317475777 | 1.225513997 | 0.2203816196 | 1.080497464 |
| atraid | 0.07908346088 | 0.06461718451 | 1.223876612 | 0.2209987775 | 1.082294648 |
| il20 | 0.07125968096 | 0.05823553033 | 1.223646124 | 0.221085752 | 1.073860051 |
| fgf9 | 0.07814307065 | 0.06386196608 | 1.223624568 | 0.2210938873 | 1.081277347 |
| mri1 | 0.07727186024 | 0.06317221757 | 1.223193727 | 0.2212565344 | 1.080335737 |
| itih1 | 0.07655781913 | 0.06262940735 | 1.222394118 | 0.2215586227 | 1.079564608 |
| il6 | 0.07936452493 | 0.06497859504 | 1.221394905 | 0.2219365353 | 1.082598884 |
| ftcd | 0.08538505588 | 0.0699636285 | 1.220420634 | 0.2223054591 | 1.089136364 |
| spon1 | 0.07569701309 | 0.06208383409 | 1.219270913 | 0.2227413846 | 1.078635712 |
| dda1 | -0.07773133035 | 0.06379137294 | -1.218524179 | 0.2230248421 | 0.9252129698 |
| ccl2 | -0.0725562882 | 0.05959186329 | -1.217553609 | 0.2233936527 | 0.9300133964 |
| cdc123 | -0.07760807175 | 0.06378272044 | -1.216757003 | 0.2236966838 | 0.9253270173 |
| siglec5 | -0.07047004269 | 0.05791819803 | -1.216716768 | 0.2237119972 | 0.931955658 |
| cfhr4 | -0.07511774504 | 0.06197516711 | -1.212062001 | 0.2254886481 | 0.9276342556 |
| mansc4 | 0.07801117749 | 0.06439875238 | 1.211377156 | 0.2257508905 | 1.081134743 |
| acrv1 | -0.120530041 | 0.09958930196 | -1.210270969 | 0.2261749337 | 0.8864504571 |
| c1r | 0.07720030163 | 0.06401846482 | 1.205906793 | 0.227853431 | 1.080258432 |
| atrn | 0.07874758664 | 0.06533882259 | 1.205218942 | 0.2281187918 | 1.081931194 |
| gpc1 | 0.07088164082 | 0.05887078461 | 1.204020658 | 0.2285815941 | 1.073454165 |
| yy1 | 0.07762296218 | 0.06454776626 | 1.202566203 | 0.2291442329 | 1.080715111 |
| mapk13 | -0.07635873895 | 0.06361547778 | -1.200316992 | 0.2300162529 | 0.9264837811 |
| hao1 | 0.0744716275 | 0.06211945562 | 1.198845462 | 0.2305880413 | 1.077314777 |
| lacrt | -0.07861400132 | 0.06561784396 | -1.198058281 | 0.2308943288 | 0.9243966715 |
| aida | -0.07567085356 | 0.06320588732 | -1.197212107 | 0.2312238919 | 0.927121315 |
| adamtsl2 | 0.08079160181 | 0.06749841914 | 1.196940652 | 0.2313296877 | 1.084144939 |
| relt | -0.07850955569 | 0.06563786726 | -1.196101564 | 0.2316569273 | 0.9244932257 |
| enoph1 | -0.07614322596 | 0.06370205176 | -1.195302567 | 0.2319688374 | 0.9266834719 |
| reg3g | 0.07563392232 | 0.06328732333 | 1.195088026 | 0.2320526397 | 1.078567662 |
| apobr | 0.07658759187 | 0.06415025487 | 1.193878216 | 0.2325256098 | 1.07959675 |
| hgfac | -0.0765907607 | 0.06436419301 | -1.189959155 | 0.2340624463 | 0.9262688417 |
| pla2g2a | 0.07364698985 | 0.06189531851 | 1.189863654 | 0.2340999859 | 1.076426749 |
| dpep2 | 0.0692381607 | 0.05833405442 | 1.186925225 | 0.2352571162 | 1.071691414 |
| clec4g | 0.07071033064 | 0.05965359535 | 1.185349018 | 0.2358794803 | 1.073270287 |
| cep290 | 0.07518623334 | 0.06343344341 | 1.185277502 | 0.2359077458 | 1.078084908 |
| smpdl3b | -0.07479325149 | 0.0631043169 | -1.185231933 | 0.2359257575 | 0.9279353157 |

| Protein | beta | se | z | p_val | HR |
|---|---|---|---|---|---|
| ptx3 | 0.06924142644 | 0.05845682899 | 1.184488239 | 0.2362198491 | 1.071694913 |
| igsf3 | 0.07734593475 | 0.06538130422 | 1.182997734 | 0.2368100454 | 1.080415765 |
| cgb3_cgb5_cgb8 | 0.08009150231 | 0.06771046803 | 1.182852587 | 0.2368675749 | 1.083386195 |
| maged1 | 0.06856111702 | 0.05813744091 | 1.179293687 | 0.2382812513 | 1.070966077 |
| shbg | -0.08451655436 | 0.07174819401 | -1.177960721 | 0.2388122657 | 0.9189564425 |
| sirt2 | 0.06917863878 | 0.05873912322 | 1.177726786 | 0.2389055448 | 1.071627626 |
| cspg4 | 0.07849173885 | 0.0667723639 | 1.175512357 | 0.2397897966 | 1.081654419 |
| ccl3 | -0.07558909746 | 0.06436267087 | -1.174424498 | 0.2402250373 | 0.9271971159 |
| fbn2 | 0.07438578191 | 0.06334881897 | 1.174225236 | 0.2403048204 | 1.077222298 |
| lbr | 0.06897335897 | 0.05881969914 | 1.172623457 | 0.2409468384 | 1.071407665 |
| pnlip | 0.07189487265 | 0.06132195508 | 1.172416511 | 0.2410298736 | 1.074542374 |
| prss53 | 0.07363130087 | 0.06293313052 | 1.169992662 | 0.2420039221 | 1.076409861 |
| cfc1 | 0.070301663 | 0.06012666284 | 1.169226092 | 0.2423125515 | 1.072831766 |
| il36a | -0.07442172029 | 0.0637053175 | -1.16821834 | 0.2427187044 | 0.9282801367 |
| dnm1 | 0.07378023949 | 0.06323052092 | 1.166845353 | 0.243272828 | 1.076570192 |
| gja8 | 0.07453750658 | 0.06390537352 | 1.166373068 | 0.2434636426 | 1.077385752 |
| amigo1 | 0.0734412959 | 0.06298005116 | 1.1661041 | 0.243572359 | 1.076205357 |
| sct | 0.07195393447 | 0.0617428578 | 1.165380694 | 0.2438649285 | 1.074605841 |
| pfdn2 | -0.0677996377 | 0.05822500867 | -1.164441865 | 0.2442449898 | 0.9344476829 |
| ca11 | -0.06846614972 | 0.05883210656 | -1.163754856 | 0.2445233715 | 0.9338250698 |
| tpk1 | 0.07464994928 | 0.0641524307 | 1.163633996 | 0.2445723683 | 1.077506903 |
| bcl7a | 0.0731580482 | 0.06309276935 | 1.159531416 | 0.2462396384 | 1.075900568 |
| frmd7 | 0.07298176223 | 0.06300075782 | 1.158426736 | 0.2466899339 | 1.075710918 |
| lrp11 | 0.06971998198 | 0.06019538954 | 1.158227939 | 0.2467710295 | 1.072207902 |
| cd300a | -0.07334157091 | 0.06340528691 | -1.156710654 | 0.2473905953 | 0.9292833596 |
| cfp | 0.07595373889 | 0.06570996412 | 1.155893781 | 0.2477246067 | 1.078912661 |
| evi5 | 0.07506812392 | 0.06494438899 | 1.155883135 | 0.247728962 | 1.077957583 |
| cd300lf | -0.06632542215 | 0.05740560581 | -1.155382322 | 0.2479338978 | 0.9358262761 |
| spint1 | 0.06709597839 | 0.05809987615 | 1.154838579 | 0.2481565357 | 1.069398112 |
| dpp7 | -0.06725758217 | 0.05829109449 | -1.153822599 | 0.2485729069 | 0.9349543427 |
| cd209 | -0.06705564156 | 0.05819621926 | -1.15223364 | 0.2492250776 | 0.9351431671 |
| atp6v1d | 0.06732262072 | 0.05856990339 | 1.149440529 | 0.2503743763 | 1.069640511 |
| slc44a4 | 0.07259380909 | 0.06316171339 | 1.149332486 | 0.2504189074 | 1.075293674 |
| cpm | 0.07768052213 | 0.06759543817 | 1.149197701 | 0.2504744687 | 1.080777319 |
| lefty2 | -0.07202799212 | 0.06270324378 | -1.148712376 | 0.2506746011 | 0.9305048486 |
| thsd1 | 0.07421046181 | 0.06469275234 | 1.147121727 | 0.2513313153 | 1.077033456 |
| zhx2 | 0.07442197347 | 0.06501484961 | 1.144691927 | 0.2523367968 | 1.077261285 |
| cgref1 | 0.06774088986 | 0.05924728486 | 1.143358552 | 0.2528897537 | 1.070088002 |
| muc2 | 0.07118557595 | 0.06227412969 | 1.143100294 | 0.2529969523 | 1.073780475 |
| cst6 | 0.06879439747 | 0.06018286276 | 1.143089483 | 0.2530014404 | 1.071215942 |
| hsdl2 | 0.07283748317 | 0.06377572954 | 1.142087808 | 0.2534175226 | 1.075555727 |
| akt1s1 | 0.06698109715 | 0.0586879023 | 1.141310126 | 0.2537408899 | 1.069275266 |
| ssh3 | 0.07260848213 | 0.06371694971 | 1.139547365 | 0.2544749247 | 1.075309452 |
| stab2 | 0.07500019959 | 0.065857471 | 1.13882599 | 0.2547757395 | 1.077884366 |
| man1a2 | 0.07690140459 | 0.06757882769 | 1.137951149 | 0.2551408816 | 1.079935594 |
| dapk2 | 0.07292502365 | 0.06415374327 | 1.136722815 | 0.2556541785 | 1.075649886 |
| guk1 | -0.07283242843 | 0.06412568813 | -1.135776169 | 0.256050253 | 0.9297566177 |
| shh | 0.07327293723 | 0.06459536442 | 1.134337392 | 0.25665305 | 1.076024184 |
| swap70 | 0.07356553772 | 0.06486525042 | 1.134128632 | 0.2567405951 | 1.076339075 |
| il33 | 0.06628282561 | 0.05851371928 | 1.132774099 | 0.2573091312 | 1.068528882 |
| ruvbl1 | -0.06523723945 | 0.05767738283 | -1.131071423 | 0.2580250312 | 0.9368451804 |
| tlr4 | 0.07292886162 | 0.0645241163 | 1.130257426 | 0.2583677682 | 1.075654014 |
| adh1b | 0.07784304638 | 0.06895392882 | 1.128913866 | 0.2589341707 | 1.080952986 |
| hmgcl | 0.07241236732 | 0.06415113009 | 1.128777735 | 0.2589916074 | 1.075098588 |
| st13 | 0.0710934748 | 0.06300657112 | 1.128350163 | 0.2591720662 | 1.073681583 |
| lxn | 0.0664211495 | 0.05893580866 | 1.127008367 | 0.2597389433 | 1.068676695 |
| thbs4 | 0.06942759325 | 0.0617666472 | 1.124030466 | 0.2610001014 | 1.071894446 |
| klk10 | 0.06572793017 | 0.05850444996 | 1.123468902 | 0.2612384009 | 1.067936124 |
| c1qbp | 0.06964975712 | 0.06202919256 | 1.122854486 | 0.2614993003 | 1.072132609 |
| olr1 | 0.06715514934 | 0.0598329432 | 1.122377502 | 0.261701966 | 1.069461392 |
| larp1 | 0.07159524293 | 0.06380721254 | 1.122055644 | 0.2618387819 | 1.074220458 |
| top1 | 0.07193046436 | 0.06412108225 | 1.121791178 | 0.2619512383 | 1.07458062 |
| rasgrf1 | -0.07136906059 | 0.06362992594 | -1.121627277 | 0.2620209487 | 0.9311181897 |
| zcchc8 | -0.0717777468 | 0.06405047137 | -1.120643537 | 0.2624396251 | 0.9307377323 |
| gclm | 0.07120585933 | 0.06358846666 | 1.119792048 | 0.2628023886 | 1.073802255 |
| ly96 | -0.06791920457 | 0.06068199459 | -1.119264537 | 0.2630273002 | 0.9343359606 |
| lrrc25 | 0.06754970789 | 0.06035820025 | 1.119147152 | 0.263077367 | 1.06988344 |
| col9a2 | 0.07135539942 | 0.06381481903 | 1.118163469 | 0.2634971833 | 1.073962844 |
| slitrk6 | 0.06647296838 | 0.0594497828 | 1.118136438 | 0.2635087261 | 1.068732074 |
| mag | -0.07072300055 | 0.06325085907 | -1.11813502 | 0.2635093318 | 0.9317199423 |

| Protein | beta | se | z | p_val | HR |
|---|---|---|---|---|---|
| vav3 | 0.07150725351 | 0.06399379091 | 1.117409244 | 0.2638193846 | 1.074125942 |
| pdia2 | 0.07096371474 | 0.06368273661 | 1.114332055 | 0.2651367602 | 1.073542271 |
| galnt7 | 0.06444421054 | 0.05789509881 | 1.113120314 | 0.2656567605 | 1.066566073 |
| dcc | 0.0706136079 | 0.06357724583 | 1.11067422 | 0.266708603 | 1.073166483 |
| scn2b | 0.07048624155 | 0.06357451134 | 1.108718574 | 0.2675516067 | 1.073029806 |
| cblif | 0.06449009243 | 0.0581768124 | 1.108518837 | 0.2676378084 | 1.066615011 |
| s100a12 | 0.06558748944 | 0.05931311104 | 1.105784004 | 0.26882002 | 1.067786153 |
| pnma2 | 0.0695216569 | 0.06288352386 | 1.105562358 | 0.2689159901 | 1.071995277 |
| tgfbr1 | 0.07048768423 | 0.06390393835 | 1.103025667 | 0.2700160189 | 1.073031354 |
| ngf | -0.0647158469 | 0.05869572506 | -1.102564911 | 0.2702161551 | 0.9373337718 |
| stx8 | 0.06489418928 | 0.05898658719 | 1.100151617 | 0.2712660672 | 1.067046113 |
| sh2d1a | -0.06469628983 | 0.05888426095 | -1.098702587 | 0.2718978139 | 0.9373521035 |
| lmod1 | 0.07939949126 | 0.07236601783 | 1.097193042 | 0.272557014 | 1.08263674 |
| cdk1 | -0.06981475461 | 0.06364838051 | -1.09688187 | 0.2726930353 | 0.9325665576 |
| btd | 0.07082995469 | 0.06470386816 | 1.09467883 | 0.2736573664 | 1.073398684 |
| fgf19 | 0.06397615396 | 0.05849508011 | 1.09370145 | 0.2740859379 | 1.066066977 |
| egfr | -0.06531286419 | 0.05974766 | -1.093145141 | 0.2743300792 | 0.9367743344 |
| clec12a | -0.07030015823 | 0.06435078119 | -1.092452289 | 0.2746343503 | 0.9321139962 |
| hmox1 | 0.0653560788 | 0.0598316302 | 1.092333245 | 0.2746866527 | 1.067539085 |
| paxx | 0.07107028449 | 0.06512167625 | 1.091346055 | 0.2751206396 | 1.073656685 |
| fam20a | 0.07539962523 | 0.06909024615 | 1.091320837 | 0.2751317323 | 1.078314987 |
| mtss1 | 0.0673977135 | 0.06187733858 | 1.089214809 | 0.2760591701 | 1.069720836 |
| spink6 | -0.06648355432 | 0.06114353098 | -1.087335868 | 0.2768884026 | 0.9356783036 |
| dnpep | 0.06995001202 | 0.06441422846 | 1.085940384 | 0.2775053684 | 1.07245457 |
| was | 0.06346756161 | 0.05846273717 | 1.085607084 | 0.2776528644 | 1.065524921 |
| arg1 | 0.06312632549 | 0.05823207756 | 1.084047284 | 0.2783438346 | 1.065161388 |
| fgfr2 | 0.06919919948 | 0.0638718103 | 1.083407518 | 0.2786275801 | 1.07164966 |
| pm20d1 | 0.06464095235 | 0.05968616298 | 1.083014038 | 0.2788021923 | 1.066775932 |
| adam15 | -0.06347665684 | 0.05872899347 | -1.080840197 | 0.2797682045 | 0.9384960265 |
| igfl4 | -0.06868331116 | 0.06369108044 | -1.078381944 | 0.2808633413 | 0.933622301 |
| xrcc4 | -0.0622524437 | 0.05790010069 | -1.075169869 | 0.2822986826 | 0.9396456492 |
| nomo1 | -0.06684105035 | 0.06231059933 | -1.07270755 | 0.2834023518 | 0.9353438621 |
| mrps16 | 0.06904222317 | 0.0644099416 | 1.071918736 | 0.2837565341 | 1.07148145 |
| pag1 | -0.06457540297 | 0.06026098729 | -1.071595503 | 0.2839017542 | 0.9374654239 |
| sbsn | 0.06634859541 | 0.06191899414 | 1.07153865 | 0.2839273017 | 1.068599161 |
| txn | 0.0685834488 | 0.0640424189 | 1.070906596 | 0.28421143 | 1.070989994 |
| fgr | 0.06241836518 | 0.05834159752 | 1.069877546 | 0.2846744313 | 1.064407563 |
| echs1 | 0.06872721851 | 0.06431188393 | 1.068655034 | 0.2852251403 | 1.071143981 |
| tmprss11d | 0.06781772725 | 0.0635155018 | 1.067735046 | 0.2856400449 | 1.070170228 |
| trpv3 | -0.06808878805 | 0.06380201607 | -1.06718866 | 0.2858866521 | 0.9341775261 |
| cd2ap | 0.06319508591 | 0.05925181471 | 1.066551062 | 0.2861746092 | 1.065234631 |
| siglec1 | -0.06492914371 | 0.06090514559 | -1.06606992 | 0.2863920358 | 0.9371338629 |
| sez6l2 | 0.06996210386 | 0.06565803303 | 1.065552844 | 0.2866258254 | 1.072467538 |
| ptprk | 0.06851544747 | 0.06440509604 | 1.063820283 | 0.2874101213 | 1.070917168 |
| vtcn1 | -0.06330796233 | 0.0595564757 | -1.062990407 | 0.2877863018 | 0.938654359 |
| vsig4 | -0.06790363821 | 0.06393448259 | -1.06208161 | 0.2881986378 | 0.9343505049 |
| dock9 | -0.06759837197 | 0.06367274744 | -1.061653136 | 0.288393182 | 0.9346357741 |
| maea | -0.0624472738 | 0.05883220641 | -1.061447082 | 0.2884867699 | 0.9394625958 |
| eif5a | 0.06217403774 | 0.05863663662 | 1.06032749 | 0.2889956371 | 1.06414753 |
| epha10 | 0.06235523893 | 0.05883523102 | 1.059828233 | 0.28922275 | 1.064340373 |
| pibf1 | 0.06730803461 | 0.06353488658 | 1.059387027 | 0.2894235554 | 1.069624909 |
| eda2r | 0.07714374766 | 0.07283691834 | 1.059129758 | 0.2895406894 | 1.080197341 |
| milr1 | 0.06291270345 | 0.05943981084 | 1.058427047 | 0.2898607941 | 1.06493387 |
| cnp | 0.06769031819 | 0.06395821859 | 1.058352151 | 0.2898949255 | 1.070033887 |
| bnip3l | 0.07019739438 | 0.06640424188 | 1.057122142 | 0.2904558473 | 1.072719909 |
| acp1 | 0.06909280031 | 0.06537894811 | 1.056805016 | 0.2906005846 | 1.071535643 |
| mln | -0.06517875886 | 0.06169732679 | -1.0564276 | 0.290772902 | 0.9368999692 |
| fcar | 0.06158171936 | 0.05830438182 | 1.056210827 | 0.2908719053 | 1.063517403 |
| il21r | 0.06732404718 | 0.06381572019 | 1.054975905 | 0.2914363446 | 1.069642037 |
| kdm3a | -0.06832711326 | 0.06478890007 | -1.05461141 | 0.2916030833 | 0.9339549146 |
| oplah | 0.06773015456 | 0.06422401903 | 1.054592278 | 0.2916118368 | 1.070076514 |
| serpine2 | 0.06720469421 | 0.06374997786 | 1.054191648 | 0.2917951832 | 1.069514379 |
| lilrb1 | 0.06301990958 | 0.05981088384 | 1.053652873 | 0.2920418727 | 1.065048044 |
| cntf | -0.06633976284 | 0.06302599762 | -1.052577751 | 0.2925345586 | 0.9358128558 |
| itpr1 | 0.0681725467 | 0.06483091315 | 1.05154383 | 0.2930088903 | 1.070550012 |
| lmnb1 | -0.06645984923 | 0.06337581419 | -1.04866265 | 0.2943334104 | 0.9357004842 |
| brme1 | -0.06613773121 | 0.06315308776 | -1.047260452 | 0.294979471 | 0.9360019387 |
| cdk5rap3 | -0.06583290694 | 0.06289150334 | -1.046769491 | 0.2952059047 | 0.9362872983 |
| ncr3lg1 | 0.06938505169 | 0.06633636833 | 1.045957948 | 0.2955804481 | 1.071848847 |
| phldb2 | -0.06662015788 | 0.06370209338 | -1.045807984 | 0.2956496941 | 0.9355504953 |

| Protein | beta | se | z | p_val | HR |
|---|---|---|---|---|---|
| c8b | 0.06684498662 | 0.06394925076 | 1.045281779 | 0.2958927563 | 1.069129736 |
| kynu | 0.06490317782 | 0.06211688462 | 1.04485565 | 0.2960896903 | 1.067055705 |
| lcat | 0.06965293093 | 0.06673957424 | 1.043652611 | 0.2966461423 | 1.072136011 |
| ckb | -0.0743624051 | 0.0712543951 | -1.043618502 | 0.2966619291 | 0.9283351995 |
| ccl28 | 0.06286158517 | 0.0603418515 | 1.041757646 | 0.2975240552 | 1.064879434 |
| cntnap4 | -0.06675106894 | 0.06422157066 | -1.039387051 | 0.2986247639 | 0.9354280294 |
| mrpl46 | -0.06020520609 | 0.05793551958 | -1.039176079 | 0.2987228533 | 0.9415712976 |
| upk3bl1 | -0.06569499813 | 0.06324451939 | -1.038746104 | 0.298922833 | 0.9364164295 |
| dkkl1 | 0.06050365845 | 0.05831524497 | 1.037527296 | 0.2994901812 | 1.062371484 |
| rcc1 | -0.06840063733 | 0.06596107107 | -1.03698494 | 0.2997428758 | 0.9338862489 |
| dnaja1 | -0.06644042207 | 0.06408381078 | -1.036773894 | 0.2998412447 | 0.9357186624 |
| ceacam18 | -0.06598128392 | 0.06371580101 | -1.035556061 | 0.3004092997 | 0.9361483852 |
| arsa | -0.06339992366 | 0.06126756624 | -1.034804017 | 0.3007604465 | 0.9385680431 |
| cdhr5 | 0.06258629682 | 0.06057189582 | 1.033256364 | 0.3014839421 | 1.064586325 |
| eloa | 0.06289651018 | 0.06091660641 | 1.032501872 | 0.301837071 | 1.064916626 |
| ifnl2 | 0.06506105944 | 0.06301990667 | 1.032389016 | 0.3018899154 | 1.067224187 |
| siglec10 | 0.06127162307 | 0.0594120722 | 1.031299209 | 0.3024005291 | 1.063187661 |
| get3 | 0.06588308415 | 0.06389489674 | 1.031116529 | 0.3024861775 | 1.068101832 |
| sncg | 0.06654000054 | 0.06457300491 | 1.030461578 | 0.3027933796 | 1.068803716 |
| il10ra | -0.06052962986 | 0.05880908597 | -1.02925643 | 0.3033591916 | 0.941265879 |
| pts | 0.06366216736 | 0.06192550383 | 1.028044399 | 0.3039289432 | 1.065732299 |
| ophn1 | 0.06439002289 | 0.06265050974 | 1.027765347 | 0.3040602204 | 1.06650828 |
| eppk1 | -0.06654929026 | 0.06477864657 | -1.027333756 | 0.3042633328 | 0.9356167979 |
| angptl3 | -0.06455574996 | 0.06286138643 | -1.026953964 | 0.3044421421 | 0.9374838481 |
| prnd | 0.06456817706 | 0.06289187474 | 1.026653718 | 0.3045835495 | 1.0666983 |
| ltbp3 | -0.06473047087 | 0.06305225659 | -1.026616245 | 0.3046012018 | 0.9373200644 |
| paip2b | 0.06546906968 | 0.06378662718 | 1.026376101 | 0.3047143388 | 1.067659714 |
| defb4a_defb4b | -0.06437162612 | 0.06272480866 | -1.026254643 | 0.3047715707 | 0.9376564771 |
| scgb3a1 | -0.06679295524 | 0.06523608491 | -1.023865171 | 0.3058989629 | 0.9353888486 |
| tnfrsf11a | 0.06394002482 | 0.06248294261 | 1.02331968 | 0.3061567219 | 1.066028462 |
| cxcl14 | -0.06902909288 | 0.06752662433 | -1.022250017 | 0.3066625833 | 0.9332995273 |
| fgfbp1 | -0.06148480295 | 0.06022690891 | -1.020885914 | 0.3073084936 | 0.9403672364 |
| nedd4l | 0.06616809136 | 0.06484831299 | 1.020351776 | 0.3075616558 | 1.068406292 |
| enah | 0.06201039692 | 0.06078728865 | 1.020121119 | 0.3076710219 | 1.063973407 |
| septin3 | -0.06539266944 | 0.06416489236 | -1.019134717 | 0.3081390141 | 0.9366995779 |
| aifm1 | 0.06002301513 | 0.05890061006 | 1.019055916 | 0.3081764214 | 1.061860985 |
| cd248 | 0.06558386962 | 0.06445812886 | 1.017464683 | 0.3089324251 | 1.067782288 |
| itgb6 | -0.06315575184 | 0.06212824197 | -1.016538531 | 0.3093730086 | 0.9387972429 |
| hspb6 | 0.0720179974 | 0.07086477324 | 1.016273589 | 0.309499122 | 1.074674685 |
| cpa4 | 0.06482452684 | 0.06381337422 | 1.015845466 | 0.3097029815 | 1.066971783 |
| apoa1 | 0.06880170192 | 0.06778382194 | 1.015016562 | 0.3100979327 | 1.071223766 |
| scly | 0.06415097265 | 0.06324658508 | 1.01429939 | 0.3104399152 | 1.066253362 |
| araf | 0.06465429313 | 0.06374367386 | 1.014285642 | 0.3104464734 | 1.066790164 |
| insl3 | -0.1032635815 | 0.101895285 | -1.013428457 | 0.3108555527 | 0.9018892209 |
| oxt | -0.06392411445 | 0.06309841013 | -1.013085977 | 0.3110190959 | 0.9380761833 |
| rnf5 | 0.06499290424 | 0.06420628325 | 1.012251464 | 0.311417835 | 1.067151452 |
| cd46 | 0.06108645761 | 0.06039361071 | 1.011472189 | 0.3117904856 | 1.062990814 |
| yap1 | 0.07425270421 | 0.0734152289 | 1.011407379 | 0.3118214911 | 1.077078953 |
| sema3f | -0.06368784034 | 0.06302644287 | -1.010493968 | 0.312258688 | 0.9382978526 |
| chit1 | -0.05926093711 | 0.05864706103 | -1.010467295 | 0.3122714609 | 0.9424608141 |
| siglec15 | 0.05846731644 | 0.05787350927 | 1.010260431 | 0.3123705332 | 1.060210334 |
| fetub | 0.06285386577 | 0.06222985354 | 1.010027538 | 0.3124820966 | 1.064871214 |
| qpct | 0.05973923747 | 0.05917874638 | 1.009471155 | 0.3127487284 | 1.061559695 |
| f10 | 0.06350322173 | 0.06291590159 | 1.009335003 | 0.3128139985 | 1.065562919 |
| dcn | -0.06266905735 | 0.06209224062 | -1.009289675 | 0.3128357305 | 0.9392542616 |
| csh1 | 0.06283355562 | 0.06225623592 | 1.009273283 | 0.3128435895 | 1.064849586 |
| btn3a2 | -0.0594043284 | 0.05887607385 | -1.008972313 | 0.3129879128 | 0.9423256831 |
| atp1b4 | 0.06440240263 | 0.06387850229 | 1.008201513 | 0.3133577319 | 1.066521483 |
| cdh5 | -0.05877651386 | 0.05832339496 | -1.007769076 | 0.3135653347 | 0.9429174746 |
| adam22 | 0.05919809258 | 0.05875098157 | 1.007610273 | 0.3136415952 | 1.060985393 |
| tex101 | -0.09063305223 | 0.09014244421 | -1.005442586 | 0.3146837824 | 0.9133528022 |
| abca2 | -0.06573929324 | 0.06543297152 | -1.004681458 | 0.3150502592 | 0.9363749518 |
| pikfyve | 0.06538619989 | 0.06509856132 | 1.004418509 | 0.3151769324 | 1.067571241 |
| plpbp | 0.05931628539 | 0.05909462001 | 1.003751025 | 0.3154986361 | 1.061110802 |
| efhd1 | -0.06830129838 | 0.06806804747 | -1.003426731 | 0.3156550119 | 0.9339790248 |
| hspb1 | 0.05962776907 | 0.05942426444 | 1.003424605 | 0.3156560374 | 1.061441372 |
| lect2 | -0.07295141066 | 0.0727226069 | -1.003146254 | 0.3157903006 | 0.9296459998 |
| adamts15 | 0.06589723605 | 0.06577734752 | 1.001822642 | 0.3164292599 | 1.068116948 |
| c19orf12 | -0.0615492212 | 0.0614684709 | -1.001313686 | 0.3166751781 | 0.9403066616 |
| crnn | 0.06254147753 | 0.06253265016 | 1.000141164 | 0.3172421975 | 1.064538612 |

| Protein | beta | se | z | p_val | HR |
|---|---|---|---|---|---|
| nefl | 0.06871588841 | 0.06876449729 | 0.9992931109 | 0.3176527217 | 1.071131845 |
| dnajb8 | 0.05820910587 | 0.05828110208 | 0.9987646731 | 0.317908703 | 1.059936612 |
| crx | -0.05794853379 | 0.05806176539 | -0.9980498079 | 0.3182552069 | 0.9436985148 |
| chm | 0.0631721823 | 0.06340242492 | 0.9963685518 | 0.3190711071 | 1.065210234 |
| capn3 | 0.06255875189 | 0.06289775924 | 0.9946101841 | 0.3199258924 | 1.064557002 |
| nub1 | 0.0579787793 | 0.05834127423 | 0.9937866471 | 0.3203267483 | 1.059692508 |
| oxct1 | 0.06370460767 | 0.06417458246 | 0.992676621 | 0.3208675718 | 1.06577753 |
| fam171a2 | -0.06167063667 | 0.0621593346 | -0.9921379801 | 0.3211302216 | 0.9401925007 |
| cd99 | -0.06809741446 | 0.06863763738 | -0.9921293486 | 0.3211344316 | 0.9341694675 |
| lta | -0.05884213836 | 0.05945189725 | -0.9897436597 | 0.3222994291 | 0.9428555981 |
| hebp1 | 0.05793564245 | 0.05855370055 | 0.9894445937 | 0.3224456655 | 1.059646797 |
| ubxn1 | 0.06298552508 | 0.0637605061 | 0.987845438 | 0.3232283505 | 1.065011423 |
| rcor1 | 0.0586439467 | 0.05947399138 | 0.9860435685 | 0.3241117335 | 1.060397615 |
| folr1 | 0.06052564481 | 0.0614890586 | 0.9843319477 | 0.3249523262 | 1.062394842 |
| tsnax | 0.06394463769 | 0.0649836103 | 0.9840117746 | 0.3251097235 | 1.066033379 |
| col28a1 | 0.06352545606 | 0.0645850873 | 0.9835932522 | 0.3253155442 | 1.065586611 |
| pacs2 | 0.06348404865 | 0.06454996719 | 0.9834869235 | 0.325367848 | 1.065542489 |
| susd2 | 0.06047768333 | 0.06150986888 | 0.983219188 | 0.325499573 | 1.062343889 |
| jpt2 | 0.06210336811 | 0.06321759898 | 0.9823746728 | 0.3259152991 | 1.06407233 |
| slc51b | -0.06359833271 | 0.06476141137 | -0.9820405603 | 0.3260798665 | 0.9383818411 |
| cetn2 | 0.05747349563 | 0.05859547919 | 0.9808520457 | 0.3266657083 | 1.059157198 |
| cthrc1 | 0.07128833071 | 0.07268318133 | 0.9808091694 | 0.3266868557 | 1.073890817 |
| agxt | 0.06050510911 | 0.06178208809 | 0.9793309189 | 0.3274164981 | 1.062373025 |
| rida | 0.06722836539 | 0.06868651081 | 0.9787710075 | 0.327693138 | 1.069539696 |
| ceacam3 | 0.05717484253 | 0.05844158735 | 0.9783245992 | 0.3279138072 | 1.058840925 |
| krt19 | -0.05872494999 | 0.06004065736 | -0.978086393 | 0.3280315971 | 0.9429660963 |
| lhb | 0.06199124201 | 0.06338194011 | 0.9780584486 | 0.328045417 | 1.063953027 |
| c1qtnf5 | 0.06215055441 | 0.06359090597 | 0.9773497241 | 0.328396044 | 1.064122541 |
| muc16 | 0.05727389689 | 0.05869928137 | 0.9757171733 | 0.3292046395 | 1.058945813 |
| tpp1 | -0.05996931518 | 0.06153868803 | -0.9744977851 | 0.3298094384 | 0.9417934319 |
| atxn2 | -0.06123017298 | 0.06289318044 | -0.9735582228 | 0.3302759381 | 0.9406067126 |
| pdcd5 | 0.05746282766 | 0.05903157014 | 0.9734253641 | 0.3303419379 | 1.059145899 |
| foxj3 | 0.06376499771 | 0.06558581688 | 0.9722376078 | 0.3309323551 | 1.065841894 |
| cd6 | -0.05742044282 | 0.05909878287 | -0.9716011062 | 0.331249032 | 0.9441970051 |
| rbp7 | 0.06548389533 | 0.06753426226 | 0.9696396042 | 0.3322261647 | 1.067675543 |
| prtn3 | -0.0571709228 | 0.05907677064 | -0.9677394716 | 0.3331744998 | 0.9444326305 |
| rabepk | -0.05662373452 | 0.05856025803 | -0.9669310967 | 0.33357848 | 0.9449495544 |
| xg | -0.07426427412 | 0.07683872659 | -0.9664953783 | 0.3337963585 | 0.9284263024 |
| klk12 | 0.05639314802 | 0.05840572863 | 0.9655413834 | 0.3342737186 | 1.058013558 |
| cxcl9 | -0.0627146109 | 0.06496411733 | -0.9653730934 | 0.3343579732 | 0.9392114762 |
| dctn6 | 0.05604930337 | 0.05808713539 | 0.9649176706 | 0.33458605 | 1.057649828 |
| pkn3 | -0.06121033277 | 0.06344746813 | -0.9647403524 | 0.3346748784 | 0.9406253746 |
| lgals4 | 0.05936104741 | 0.06155290085 | 0.9643907369 | 0.3348500646 | 1.0611583 |
| fzd8 | 0.06119055351 | 0.0634638539 | 0.9641796038 | 0.3349558883 | 1.063101473 |
| flrt2 | 0.05674156249 | 0.05888369258 | 0.963620996 | 0.3352359764 | 1.058382249 |
| bcl2 | 0.06320255002 | 0.06571955637 | 0.9617008014 | 0.3361999197 | 1.065242582 |
| ggh | 0.06020656639 | 0.06262991517 | 0.9613068487 | 0.3363979053 | 1.062055909 |
| naprt | 0.06120257421 | 0.06375205056 | 0.9600095004 | 0.3370504335 | 1.063114252 |
| cd226 | 0.06180862366 | 0.0643868823 | 0.9599567715 | 0.3370769718 | 1.063758747 |
| itgb2 | -0.05829159085 | 0.06073384964 | -0.9597875188 | 0.3371621652 | 0.9433748279 |
| thap12 | 0.06189406716 | 0.064534399 | 0.9590864425 | 0.3375151997 | 1.063849642 |
| siglec9 | 0.05669518919 | 0.059146194 | 0.9585602277 | 0.3377803368 | 1.05833317 |
| abhd14b | 0.05738352346 | 0.05989809525 | 0.9580191693 | 0.3380530924 | 1.059061908 |
| cpb2 | 0.06173443808 | 0.06447614408 | 0.9574772028 | 0.3383264476 | 1.063679834 |
| notch3 | -0.05931048895 | 0.06198991543 | -0.9567764133 | 0.3386801198 | 0.9424141146 |
| st3gal1 | 0.05912086533 | 0.06179917521 | 0.956661074 | 0.3387383515 | 1.060903459 |
| vps28 | -0.06145142358 | 0.06427798786 | -0.9560259371 | 0.3390591307 | 0.9403986258 |
| cd48 | -0.05573916376 | 0.05839252282 | -0.9545599517 | 0.3398002779 | 0.9457857989 |
| mmp12 | 0.06043745425 | 0.06331659354 | 0.9545278871 | 0.3398165001 | 1.062301153 |
| afap1 | 0.06200823627 | 0.06496680039 | 0.9544603689 | 0.3398506608 | 1.063971108 |
| pcsk7 | -0.06074393511 | 0.06367591195 | -0.9539546942 | 0.3401065761 | 0.9410641824 |
| hif1a | 0.06212405883 | 0.06525765308 | 0.9519811991 | 0.3411065169 | 1.064094347 |
| ror1 | 0.05679541276 | 0.05967489684 | 0.9517471461 | 0.3412252328 | 1.058439245 |
| dcun1d2 | 0.06048729252 | 0.06357767627 | 0.951391999 | 0.3414054204 | 1.062354098 |
| ncs1 | 0.05982233743 | 0.0628916654 | 0.9511965862 | 0.3415045911 | 1.061647915 |
| dgkz | 0.05565281622 | 0.05859422146 | 0.9498004213 | 0.3422136718 | 1.057230567 |
| cbln4 | 0.05487873217 | 0.05778814722 | 0.9496537752 | 0.3422882046 | 1.056412498 |
| zpr1 | 0.05973597422 | 0.0631019586 | 0.9466580047 | 0.3438130763 | 1.061556231 |
| tff2 | 0.05967267514 | 0.06308625736 | 0.9458902404 | 0.3442045719 | 1.061489038 |
| cyb5r2 | 0.06127214771 | 0.06484144227 | 0.9449534983 | 0.3446826171 | 1.063188219 |

| Protein | beta | se | z | p_val | HR |
|---|---|---|---|---|---|
| necap2 | -0.05954289712 | 0.06301366726 | -0.9449203595 | 0.3446995365 | 0.9421951153 |
| tpsd1 | -0.05997227583 | 0.06352846732 | -0.9440220794 | 0.3451583655 | 0.9417906436 |
| ret | 0.0588301195 | 0.06238193724 | 0.9430633626 | 0.3456484942 | 1.060595051 |
| pnliprp2 | 0.0545206358 | 0.05782650048 | 0.9428313205 | 0.3457671887 | 1.056034268 |
| mbl2 | 0.06005622797 | 0.06370517565 | 0.9427213309 | 0.3458234597 | 1.061896253 |
| mmp1 | -0.05574504862 | 0.05915212232 | -0.9424014969 | 0.3459871211 | 0.9457802331 |
| gmfg | 0.05970903528 | 0.06365689174 | 0.9379822616 | 0.3482535261 | 1.061527635 |
| cwc15 | 0.06134181953 | 0.06549215941 | 0.9366284466 | 0.3489497141 | 1.063262296 |
| tmod4 | 0.06025904788 | 0.06434268171 | 0.9365330491 | 0.3489988048 | 1.062111649 |
| ccs | 0.05566715673 | 0.05951395082 | 0.9353631537 | 0.349601179 | 1.057245728 |
| enpep | -0.05899040726 | 0.06307770582 | -0.9352021684 | 0.3496841212 | 0.9427158123 |
| angptl4 | -0.057206875 | 0.06123502091 | -0.9342182651 | 0.3501913158 | 0.9443986767 |
| trim40 | -0.05841826712 | 0.06256625156 | -0.9337025259 | 0.3504573617 | 0.9432553322 |
| fgfbp3 | 0.06029974778 | 0.06466534021 | 0.932489454 | 0.3510836343 | 1.062154877 |
| amfr | -0.05454948942 | 0.05850155839 | -0.932445065 | 0.3511065645 | 0.9469116456 |
| cd28 | 0.05582412479 | 0.05989964762 | 0.9319608214 | 0.3513567732 | 1.057411695 |
| ostn | -0.05960764256 | 0.06397542337 | -0.9317272076 | 0.3514775219 | 0.9421341144 |
| anxa11 | 0.05407315916 | 0.05812049093 | 0.9303630835 | 0.3521831257 | 1.055561823 |
| anxa1 | 0.0604040845 | 0.06508611837 | 0.9280640175 | 0.3533743632 | 1.062265705 |
| tnfrsf11b | 0.06084388522 | 0.06556234189 | 0.9280309926 | 0.3533914933 | 1.062732993 |
| slc28a1 | 0.05947974046 | 0.06413504476 | 0.9274140321 | 0.3537116079 | 1.06128426 |
| pde1c | 0.05850209615 | 0.06317000889 | 0.9261055551 | 0.3543911273 | 1.060247208 |
| plat | 0.06220490515 | 0.06721758449 | 0.9254260715 | 0.3547443224 | 1.064180378 |
| nacc1 | 0.05941197672 | 0.06434518783 | 0.9233320894 | 0.35583417 | 1.061212345 |
| apcs | 0.06769714237 | 0.07339729659 | 0.9223383628 | 0.3563521095 | 1.070041189 |
| omp | 0.05883991137 | 0.06380679548 | 0.9221574431 | 0.3564464576 | 1.060605436 |
| lrg1 | 0.05970391408 | 0.06474449196 | 0.9221466146 | 0.356452105 | 1.061522198 |
| ubqln3 | -0.05815192227 | 0.0630789502 | -0.9218910918 | 0.3565853862 | 0.9435065969 |
| adamts4 | 0.06161328851 | 0.06686198982 | 0.9214994749 | 0.3567897153 | 1.063550978 |
| entpd6 | -0.05331277688 | 0.05791449274 | -0.9205429309 | 0.3572891093 | 0.9480834275 |
| cst5 | 0.05598929088 | 0.06084275758 | 0.9202293439 | 0.357452923 | 1.057586358 |
| lrfn2 | 0.05817954828 | 0.06329642604 | 0.9191600841 | 0.3580118459 | 1.059905283 |
| ppp1r14d | -0.05812030642 | 0.06324396234 | -0.918985849 | 0.358102974 | 0.9435364271 |
| marco | -0.05538100761 | 0.06031529738 | -0.918191736 | 0.3585184944 | 0.9461245986 |
| c1qtnf9 | -0.05740494869 | 0.06252360793 | -0.9181323756 | 0.3585495669 | 0.9442116347 |
| ccl4 | -0.05478689103 | 0.05968264916 | -0.9179701605 | 0.3586344881 | 0.9466868739 |
| a1bg | 0.06212290134 | 0.06783789546 | 0.9157551383 | 0.3597953381 | 1.064093115 |
| ppp1r12b | 0.05805632822 | 0.06347357875 | 0.9146534569 | 0.3603735856 | 1.059774689 |
| f2 | 0.0577556012 | 0.06318028426 | 0.9141396225 | 0.3606434849 | 1.059456034 |
| cxcl17 | -0.06561263472 | 0.07177591187 | -0.9141316775 | 0.3606476591 | 0.9364935591 |
| sez6l | -0.05712752568 | 0.06255575263 | -0.9132257752 | 0.3611238105 | 0.944473617 |
| spon2 | 0.05640194935 | 0.06177959811 | 0.9129542937 | 0.3612665806 | 1.05802287 |
| c1qtnf1 | 0.05518561388 | 0.06049462433 | 0.9122399634 | 0.3616424108 | 1.056736741 |
| pi16 | 0.05764950352 | 0.06327854874 | 0.9110433895 | 0.3622725124 | 1.059343634 |
| ccl24 | -0.05427955041 | 0.05960262893 | -0.9106905413 | 0.3624584492 | 0.9471672885 |
| nit1 | 0.0591242914 | 0.06499149552 | 0.9097235097 | 0.3629683425 | 1.060907094 |
| ngrn | 0.05914466847 | 0.065091658 | 0.9086366869 | 0.3635419345 | 1.060928713 |
| tigit | 0.05731985749 | 0.06315471477 | 0.9076101079 | 0.364084252 | 1.058994484 |
| ifnlr1 | 0.05560848899 | 0.06128667943 | 0.907350333 | 0.3642215652 | 1.057183704 |
| pdrg1 | -0.05757860104 | 0.06362245905 | -0.9050043318 | 0.3654630923 | 0.9440476843 |
| ifngr1 | 0.05305855683 | 0.05865370907 | 0.9046070175 | 0.3656736163 | 1.054491391 |
| itga11 | -0.05404090387 | 0.05981339174 | -0.9034917148 | 0.3662649835 | 0.9473933537 |
| msmb | -0.05583979313 | 0.06181868781 | -0.9032833777 | 0.3663755162 | 0.9456906299 |
| csnk1d | -0.0584712683 | 0.06478554801 | -0.9025356749 | 0.3667723793 | 0.9432053399 |
| c2orf69 | 0.05742654278 | 0.06364271924 | 0.9023269821 | 0.3668831964 | 1.059107469 |
| igfbp6 | -0.06314734524 | 0.07006429625 | -0.9012770929 | 0.3674410101 | 0.938805135 |
| fxn | 0.05193007974 | 0.05768177901 | 0.9002856818 | 0.3679682388 | 1.053302093 |
| lpa | -0.05680312485 | 0.06316358655 | -0.8993017647 | 0.3684919477 | 0.9447800548 |
| dxo | 0.05686187333 | 0.06327792529 | 0.8986052099 | 0.3688629827 | 1.058509592 |
| pde4d | -0.05759628842 | 0.0641388549 | -0.8979937124 | 0.3691889016 | 0.9440309868 |
| fam3d | 0.05716662078 | 0.06369419932 | 0.8975169073 | 0.3694431557 | 1.058832219 |
| prss27 | 0.05191663795 | 0.05786014733 | 0.8972780117 | 0.3695705866 | 1.053287935 |
| dsg2 | 0.05381858084 | 0.06001801382 | 0.8967071286 | 0.3698752158 | 1.055293134 |
| cdcp1 | 0.06042893883 | 0.06754913535 | 0.8945923366 | 0.3710050499 | 1.062292107 |
| ecm1 | 0.05671444191 | 0.06355799511 | 0.8923258484 | 0.3722183034 | 1.058353546 |
| igsf21 | 0.0584512305 | 0.06553044974 | 0.8919705379 | 0.3724087242 | 1.060193279 |
| tef | -0.05736837725 | 0.06442361109 | -0.8904868305 | 0.3732045367 | 0.9442461665 |
| timp3 | 0.0524528912 | 0.05891298959 | 0.8903450931 | 0.3732806151 | 1.053852915 |
| hyou1 | 0.05181982291 | 0.05820416741 | 0.8903112134 | 0.3732988017 | 1.053185965 |
| klrf1 | 0.0591970968 | 0.06656119003 | 0.8893635581 | 0.3738077234 | 1.060984337 |

| Protein | beta | se | z | p_val | HR |
|---|---|---|---|---|---|
| pam | 0.05194768055 | 0.05844438021 | 0.8888396175 | 0.3740892807 | 1.053320632 |
| apoc1 | -0.06030921658 | 0.06786201456 | -0.888703599 | 0.3741623963 | 0.9414733694 |
| esr1 | 0.0571923066 | 0.06435696222 | 0.8886731851 | 0.3741787463 | 1.058859416 |
| matn3 | -0.05304164823 | 0.059708278 | -0.8883466415 | 0.3743543179 | 0.948340515 |
| ccn3 | 0.0576702579 | 0.06504226919 | 0.8866581474 | 0.3752629777 | 1.059365621 |
| mapkapk2 | 0.05588031613 | 0.06313341605 | 0.8851147241 | 0.3760947589 | 1.057471114 |
| atg16l1 | 0.05598924218 | 0.06326260851 | 0.8850289847 | 0.3761409989 | 1.057586306 |
| adgre1 | -0.05616029971 | 0.0635884801 | -0.8831835518 | 0.377137107 | 0.9453875784 |
| mif | 0.0514726631 | 0.05828500933 | 0.8831200971 | 0.3771713868 | 1.052820405 |
| hip1r | 0.05865013434 | 0.06648582847 | 0.8821448974 | 0.3776984555 | 1.060404177 |
| nrgn | 0.05507338172 | 0.06252860752 | 0.8807709607 | 0.3784418003 | 1.056618148 |
| mcam | 0.05358096897 | 0.06091396379 | 0.8796171786 | 0.3790667296 | 1.055042414 |
| pla2g15 | -0.05301552988 | 0.06027952056 | -0.8794948828 | 0.3791330065 | 0.9483652844 |
| sms | -0.05535991006 | 0.06300773134 | -0.878620907 | 0.379606856 | 0.9461445597 |
| magea3 | 0.05580926175 | 0.06355063762 | 0.8781857088 | 0.3798429463 | 1.057395979 |
| procr | 0.05594008042 | 0.06372061056 | 0.8778961772 | 0.380000064 | 1.057534315 |
| prss2 | 0.04981333071 | 0.0567503879 | 0.8777619424 | 0.3800729216 | 1.051074875 |
| spink2 | -0.06069192761 | 0.06918687977 | -0.8772172963 | 0.3803686232 | 0.9411131261 |
| lilra2 | 0.05279816752 | 0.06021773615 | 0.8767876526 | 0.3806019869 | 1.054216848 |
| ctsf | 0.05316492323 | 0.06063840167 | 0.8767533735 | 0.3806206096 | 1.054603559 |
| sdk2 | 0.05482885177 | 0.06255585151 | 0.8764783861 | 0.3807700214 | 1.056359805 |
| rab37 | 0.05113276875 | 0.0583557049 | 0.8762257064 | 0.3809073443 | 1.052462618 |
| ccne1 | 0.05674627696 | 0.06478787766 | 0.875878004 | 0.3810963585 | 1.058387239 |
| aoc3 | 0.05319145023 | 0.06074208036 | 0.875693587 | 0.3811966326 | 1.054631535 |
| galnt5 | 0.05568012747 | 0.06365444434 | 0.8747249001 | 0.3817236083 | 1.057259441 |
| agrp | -0.05282150471 | 0.06045136875 | -0.8737850904 | 0.3822353015 | 0.948549309 |
| asrgl1 | 0.05626627432 | 0.06439741051 | 0.8737350442 | 0.3822625616 | 1.057879332 |
| pithd1 | -0.05613126256 | 0.06425016367 | -0.8736361022 | 0.3823164589 | 0.9454150302 |
| fus | 0.05266931595 | 0.06037098069 | 0.872427702 | 0.3829750935 | 1.05408102 |
| shpk | -0.05566522247 | 0.06390947299 | -0.8710011187 | 0.3837535423 | 0.9458557341 |
| duox2 | 0.05120284371 | 0.05878738251 | 0.8709835602 | 0.3837631295 | 1.052536372 |
| gabra4 | 0.05531504312 | 0.06357200109 | 0.8701164376 | 0.3842367756 | 1.056873523 |
| adam23 | 0.05003138276 | 0.05756641716 | 0.8691071154 | 0.3847885452 | 1.051304089 |
| pfdn6 | 0.05548947576 | 0.06389600251 | 0.8684342303 | 0.3851566627 | 1.057057892 |
| fkbp1b | 0.0502018575 | 0.05787519804 | 0.8674157358 | 0.385714263 | 1.051483325 |
| lair2 | -0.05091150755 | 0.05876747132 | -0.8663212217 | 0.3863140317 | 0.9503627667 |
| mki67 | -0.05607896708 | 0.06474273654 | -0.8661815993 | 0.3863905825 | 0.9454644724 |
| grhpr | 0.05562668446 | 0.06426466777 | 0.8655873654 | 0.3867164867 | 1.05720294 |
| plau | 0.05174568432 | 0.05985189086 | 0.8645622316 | 0.3872791098 | 1.053107887 |
| ptprs | -0.04995051377 | 0.05788973541 | -0.8628561421 | 0.3882165669 | 0.9512764984 |
| map1lc3a | -0.05420850806 | 0.06286816708 | -0.8622568555 | 0.388546189 | 0.9472345799 |
| lilra5 | 0.05284703219 | 0.06137665496 | 0.8610282236 | 0.3892224988 | 1.054268364 |
| clec1a | -0.05101985141 | 0.05930010156 | -0.8603670157 | 0.389586762 | 0.9502598063 |
| golm2 | 0.05225852433 | 0.0608760903 | 0.8584408768 | 0.3906490642 | 1.053648101 |
| ccdc50 | 0.05403638728 | 0.06300016595 | 0.8577181737 | 0.3910481023 | 1.055523009 |
| cd83 | -0.05181401004 | 0.06043878299 | -0.8572973755 | 0.3912805586 | 0.9495054489 |
| dhps | -0.05506614032 | 0.06423371937 | -0.8572777796 | 0.3912913858 | 0.9464225492 |
| cstb | 0.05357401904 | 0.06251689705 | 0.856952625 | 0.3914710673 | 1.055035082 |
| nfya | 0.0550558733 | 0.0644569855 | 0.8541490557 | 0.3930224061 | 1.056599649 |
| lilra4 | -0.05437337917 | 0.06375436642 | -0.852857337 | 0.393738423 | 0.9470784211 |
| ndrg1 | -0.04996776932 | 0.05868050217 | -0.8515225241 | 0.3944791567 | 0.9512600838 |
| susd4 | 0.05594296745 | 0.06571212152 | 0.8513340637 | 0.3945838077 | 1.057537368 |
| mzt1 | 0.05053980111 | 0.05939542851 | 0.850903889 | 0.3948227443 | 1.051838727 |
| nt5c | 0.05427555982 | 0.06386156189 | 0.8498940241 | 0.3953840083 | 1.055775491 |
| chchd10 | 0.06166983456 | 0.07256503116 | 0.8498561025 | 0.3954050938 | 1.063611119 |
| il18bp | -0.05186355196 | 0.06110219073 | -0.848800204 | 0.3959924766 | 0.9494584097 |
| cytl1 | -0.0553204137 | 0.0652461783 | -0.847872092 | 0.3965092084 | 0.9461819297 |
| tet2 | 0.05302899581 | 0.06255663875 | 0.8476957341 | 0.3966074427 | 1.05446022 |
| tbc1d5 | 0.0489301907 | 0.05774135012 | 0.8474029547 | 0.3967705582 | 1.050147038 |
| wfikkn2 | -0.05253027501 | 0.06203739368 | -0.8467518039 | 0.3971334772 | 0.9488255949 |
| ddi2 | 0.05303004804 | 0.0627034306 | 0.8457280174 | 0.3977044896 | 1.054461329 |
| prune2 | 0.05398563081 | 0.06409433733 | 0.8422839374 | 0.3996290398 | 1.055469436 |
| morf4l2 | 0.0536376857 | 0.06368124186 | 0.8422839149 | 0.3996290524 | 1.055102254 |
| sowaha | 0.05215395891 | 0.06195936389 | 0.8417445829 | 0.3999309374 | 1.053537932 |
| tlr2 | 0.05277378551 | 0.0628344334 | 0.8398863912 | 0.4009720886 | 1.054191145 |
| scamp3 | 0.04918711687 | 0.05857455334 | 0.8397352445 | 0.4010568482 | 1.050416883 |
| esm1 | -0.05109987378 | 0.06086754428 | -0.8395257995 | 0.4011743178 | 0.9501837674 |
| aplp1 | -0.05105533759 | 0.06087473514 | -0.8386950264 | 0.40164047 | 0.9502260859 |
| mfap5 | -0.05402422075 | 0.06451979647 | -0.8373278235 | 0.4024083238 | 0.9474091593 |
| tppp2 | 0.05291285014 | 0.06320711505 | 0.837134397 | 0.4025170277 | 1.054337756 |

| Protein | beta | se | z | p_val | HR |
|---|---|---|---|---|---|
| ace | -0.05160726583 | 0.06167993455 | -0.8366945621 | 0.4027642765 | 0.9497017739 |
| dok1 | 0.05238444995 | 0.06261590843 | 0.8365996958 | 0.4028176166 | 1.053780791 |
| anxa5 | 0.04942567311 | 0.05908803705 | 0.8364751239 | 0.4028876656 | 1.050667496 |
| insl5 | -0.05717952126 | 0.06841649404 | -0.835756378 | 0.4032919715 | 0.9444245099 |
| fabp3 | 0.06213016959 | 0.07441759592 | 0.8348854706 | 0.4037821961 | 1.064100849 |
| clint1 | 0.05296200773 | 0.0635078401 | 0.8339444018 | 0.4043123147 | 1.054389586 |
| tnfsf10 | -0.04923784073 | 0.05908079592 | -0.833398399 | 0.4046200774 | 0.9519546892 |
| rtbdn | 0.05034088615 | 0.06046770233 | 0.8325252029 | 0.4051125585 | 1.051629521 |
| rbpms2 | 0.05295616264 | 0.06363920059 | 0.832131173 | 0.4053349079 | 1.054383423 |
| mad1l1 | -0.05334362027 | 0.06411916206 | -0.8319450623 | 0.4054399548 | 0.9480541859 |
| fgf21 | 0.05249974442 | 0.06313511639 | 0.8315458563 | 0.4056653344 | 1.053902293 |
| tie1 | 0.04840445848 | 0.05821463205 | 0.8314826836 | 0.4057010066 | 1.049595087 |
| stip1 | 0.04877384233 | 0.05875659728 | 0.8300998456 | 0.4064823344 | 1.049982862 |
| rnaset2 | -0.05011133343 | 0.06042813366 | -0.8292715725 | 0.406950753 | 0.9511235268 |
| cd7 | -0.05362867554 | 0.06467413206 | -0.8292136876 | 0.4069835011 | 0.9477839765 |
| stx7 | 0.05443847366 | 0.06587155484 | 0.8264337132 | 0.4085581057 | 1.055947506 |
| shisa5 | -0.05679806189 | 0.06873503722 | -0.826333471 | 0.4086149515 | 0.9447848382 |
| ntrk3 | 0.05113864653 | 0.06192980761 | 0.8257517422 | 0.4089449341 | 1.052468804 |
| prdx2 | 0.0527302871 | 0.06389951125 | 0.8252064228 | 0.4092544076 | 1.05414529 |
| mecr | 0.05241594425 | 0.06353362893 | 0.8250110238 | 0.4093653322 | 1.053813979 |
| klkb1 | 0.05544572359 | 0.06734782543 | 0.8232741477 | 0.4103521116 | 1.057011645 |
| elac1 | 0.05174194272 | 0.06289720536 | 0.8226429525 | 0.4107110653 | 1.053103946 |
| gp1bb | -0.05238727994 | 0.06372697447 | -0.8220581688 | 0.4110437916 | 0.948961282 |
| sema7a | -0.04836686939 | 0.05891427017 | -0.8209703566 | 0.4116631534 | 0.9527841756 |
| psmd5 | -0.05156464938 | 0.06286967952 | -0.8201831118 | 0.412111728 | 0.9497422477 |
| f11 | 0.05415624315 | 0.06605409095 | 0.8198772002 | 0.4122861155 | 1.055649527 |
| safb2 | 0.0511394863 | 0.06245201589 | 0.8188604574 | 0.4128660328 | 1.052469688 |
| prg3 | -0.05111930919 | 0.06243246787 | -0.8187936651 | 0.4129041459 | 0.9501653003 |
| ca12 | 0.04823424661 | 0.05891672993 | 0.8186850605 | 0.4129661223 | 1.049416449 |
| septin7 | -0.05172069612 | 0.06327989632 | -0.817332188 | 0.4137386169 | 0.9495940551 |
| bsg | 0.04934277663 | 0.06041307866 | 0.8167565322 | 0.4140675775 | 1.050580403 |
| bche | 0.05387565667 | 0.06596541585 | 0.8167257944 | 0.414085147 | 1.055353368 |
| snu13 | -0.05255957656 | 0.06448341896 | -0.8150866907 | 0.4150226894 | 0.9487977933 |
| b2m | -0.05534953579 | 0.06793022531 | -0.8147998264 | 0.4151869003 | 0.9461543753 |
| neb | -0.05282993621 | 0.06484283331 | -0.8147382449 | 0.4152221567 | 0.9485413113 |
| serpine1 | -0.04804378574 | 0.05903021143 | -0.8138846969 | 0.4157110082 | 0.9530920543 |
| zbtb17 | 0.04946386421 | 0.06078501967 | 0.8137508959 | 0.4157876707 | 1.050707623 |
| ada | 0.04838602378 | 0.05948973073 | 0.8133508623 | 0.4160169234 | 1.049575738 |
| igf2r | 0.04948169658 | 0.06092116962 | 0.8122249932 | 0.4166625408 | 1.05072636 |
| ids | -0.04733425573 | 0.05829836245 | -0.8119311374 | 0.4168311464 | 0.9537685417 |
| sprr1b | -0.05149019389 | 0.06346512689 | -0.8113147552 | 0.4171849387 | 0.9498129639 |
| clec14a | 0.04896187864 | 0.06039187749 | 0.8107361565 | 0.417517205 | 1.050180316 |
| phldb1 | 0.0536193705 | 0.06618098562 | 0.8101929882 | 0.4178292668 | 1.05508293 |
| akt2 | 0.05096869966 | 0.06293552765 | 0.8098557614 | 0.41802308 | 1.052289956 |
| reg1a | 0.04860384574 | 0.06002796462 | 0.8096867193 | 0.4181202529 | 1.049804384 |
| rtn4ip1 | 0.05063431148 | 0.06254818312 | 0.8095248968 | 0.418213288 | 1.051938141 |
| ptprr | 0.05164213224 | 0.06380479774 | 0.8093769445 | 0.4182983596 | 1.052998841 |
| brk1 | -0.04763406595 | 0.05887179507 | -0.8091152291 | 0.4184488692 | 0.953482635 |
| anxa3 | 0.0469596318 | 0.058101462 | 0.8082349426 | 0.418955346 | 1.048079699 |
| atf4 | -0.05126516487 | 0.06346516219 | -0.8077685947 | 0.4192238073 | 0.9500267234 |
| ehd3 | 0.05124327686 | 0.06346259734 | 0.8074563444 | 0.4194036163 | 1.05257893 |
| pcsk9 | 0.04907451737 | 0.06078921623 | 0.8072898519 | 0.4194995093 | 1.050298613 |
| myl6b | -0.05099993886 | 0.06328269003 | -0.8059066206 | 0.4202966935 | 0.9502787286 |
| clul1 | 0.05018624605 | 0.06228902902 | 0.8056996045 | 0.4204160776 | 1.05146691 |
| stxbp3 | 0.04697873862 | 0.05831134125 | 0.8056535422 | 0.420442644 | 1.048099725 |
| bap18 | 0.05229948465 | 0.06494234196 | 0.8053218143 | 0.4206339967 | 1.05369126 |
| met | -0.0493258522 | 0.0612786075 | -0.8049440778 | 0.4208519512 | 0.9518709099 |
| ptprz1 | -0.05735817822 | 0.07126849309 | -0.8048181705 | 0.4209246145 | 0.9442557969 |
| ccn5 | -0.0553818528 | 0.0688982765 | -0.8038205832 | 0.4215006006 | 0.9461237989 |
| cd80 | 0.05344729628 | 0.06650801821 | 0.8036218446 | 0.4216154033 | 1.054901393 |
| snca | 0.05102779851 | 0.06353085478 | 0.803197103 | 0.4218608197 | 1.052352147 |
| siae | -0.04802185092 | 0.05988997354 | -0.8018345655 | 0.4226486611 | 0.9531129605 |
| thbd | 0.04870514633 | 0.06082429053 | 0.8007515733 | 0.4232754792 | 1.049910735 |
| grp | -0.05085690425 | 0.063514217 | -0.8007168576 | 0.4232955811 | 0.9504146611 |
| dclre1c | 0.05022146654 | 0.06274722005 | 0.8003775546 | 0.4234920815 | 1.051503944 |
| tbc1d23 | 0.04645662509 | 0.05814582014 | 0.798967578 | 0.424309212 | 1.047552641 |
| ddx53 | 0.05044980016 | 0.0631514746 | 0.7988697094 | 0.4243659645 | 1.051744065 |
| caly | 0.05048938969 | 0.06320174987 | 0.7988606295 | 0.42437123 | 1.051785704 |
| ccl20 | -0.04759342702 | 0.05959312451 | -0.7986395647 | 0.4244994393 | 0.9535213843 |
| luzp2 | -0.05215804167 | 0.0653128541 | -0.7985876959 | 0.4245295245 | 0.9491788452 |

| Protein | beta | se | z | p_val | HR |
|---|---|---|---|---|---|
| txnrd1 | 0.04754563423 | 0.05957189039 | 0.7981219651 | 0.424799716 | 1.048694056 |
| gimap8 | 0.05160900107 | 0.06466472869 | 0.7981012542 | 0.4248117337 | 1.052963954 |
| s100a16 | -0.0465634364 | 0.05838959495 | -0.7974611991 | 0.4251832287 | 0.9545040084 |
| bag3 | 0.0482686143 | 0.06054154527 | 0.7972808437 | 0.4252879432 | 1.049452515 |
| ahnak | -0.05568742947 | 0.06987586999 | -0.7969479232 | 0.4254812768 | 0.9458347298 |
| cd164 | -0.04728120775 | 0.05940217725 | -0.7959507536 | 0.4260606597 | 0.9538191385 |
| glod4 | 0.04706230886 | 0.05915138965 | 0.7956247375 | 0.4262501839 | 1.048187318 |
| m6pr | 0.05099799394 | 0.06415868989 | 0.7948727449 | 0.4266875299 | 1.052320782 |
| hmgcs1 | -0.05073669821 | 0.06386211738 | -0.7944725338 | 0.4269203925 | 0.9505289136 |
| gapdh | 0.04957357786 | 0.06250316046 | 0.793137139 | 0.4276979269 | 1.050822907 |
| dynlt1 | -0.05115599949 | 0.06452625863 | -0.7927935166 | 0.4278981345 | 0.9501304391 |
| ceacam21 | 0.04625883992 | 0.05839582017 | 0.7921601201 | 0.4282673186 | 1.047345471 |
| cilp | 0.05219763823 | 0.06590364931 | 0.7920295578 | 0.4283434417 | 1.05358395 |
| bst2 | 0.04819585824 | 0.060899231 | 0.7914033962 | 0.4287086288 | 1.049376164 |
| cyth3 | 0.05075032351 | 0.06415131529 | 0.7911033979 | 0.4288836565 | 1.052060186 |
| cd34 | 0.04624570978 | 0.05848897675 | 0.7906739415 | 0.4291342862 | 1.047331719 |
| blmh | 0.04675143228 | 0.05915804069 | 0.7902802684 | 0.4293641076 | 1.047861512 |
| bola1 | 0.05072092815 | 0.06419134988 | 0.7901520726 | 0.4294389621 | 1.052029261 |
| lats1 | 0.05103335084 | 0.06459905969 | 0.7900014503 | 0.4295269214 | 1.05235799 |
| tpsg1 | 0.0504360326 | 0.06385437731 | 0.7898602215 | 0.4296094045 | 1.051729585 |
| vipr1 | -0.05086002539 | 0.06445679426 | -0.7890560797 | 0.4300792304 | 0.9504116947 |
| bmper | 0.04997134508 | 0.06336420903 | 0.788636769 | 0.4303243341 | 1.051240973 |
| gmpr | 0.04589570609 | 0.05839774472 | 0.7859157286 | 0.4319168593 | 1.046965213 |
| itgb5 | 0.04680917267 | 0.05959722371 | 0.78542539 | 0.4322041991 | 1.047922018 |
| rln2 | -0.05365148672 | 0.06848354969 | -0.7834215218 | 0.4333796219 | 0.9477623567 |
| tlr1 | 0.05047066769 | 0.06442870426 | 0.7833568635 | 0.4334175798 | 1.051766012 |
| pla2g10 | 0.04847811374 | 0.0620699049 | 0.7810244566 | 0.4347881107 | 1.049672398 |
| klrd1 | -0.04652465577 | 0.05957490645 | -0.7809438326 | 0.4348355304 | 0.9545410253 |
| tyrp1 | 0.04903608521 | 0.06280615857 | 0.7807528167 | 0.4349478898 | 1.050258249 |
| tnfrsf10a | 0.0492100621 | 0.06303014983 | 0.7807384598 | 0.4349563355 | 1.050440985 |
| arhgap1 | -0.04606895769 | 0.05907280608 | -0.7798674339 | 0.4354689089 | 0.954976107 |
| tnfsf8 | 0.04811047572 | 0.06176157383 | 0.7789710128 | 0.4359967904 | 1.04928657 |
| wfdc1 | 0.0518044872 | 0.0665771318 | 0.7781123308 | 0.436502794 | 1.053169814 |
| fgd3 | 0.04907789489 | 0.06311593961 | 0.7775832094 | 0.4368147627 | 1.050302161 |
| mb | -0.05485010067 | 0.07054418938 | -0.7775282578 | 0.4368471694 | 0.9466270361 |
| ppcdc | 0.04556076833 | 0.05860169032 | 0.7774650882 | 0.4368844242 | 1.046614604 |
| smpd3 | 0.05028335073 | 0.06469473702 | 0.777240206 | 0.4370170653 | 1.051569017 |
| adamts13 | -0.04562880216 | 0.05881468461 | -0.7758062881 | 0.4378633706 | 0.9553965375 |
| ogfr | 0.04712566071 | 0.0607453714 | 0.7757901488 | 0.4378729015 | 1.048253725 |
| ifnw1 | -0.04905642924 | 0.06327233551 | -0.7753219293 | 0.4381494544 | 0.9521274004 |
| acadsb | 0.04858904148 | 0.06272229964 | 0.7746693243 | 0.4385350818 | 1.049788842 |
| traf2 | 0.0456422116 | 0.05898671478 | 0.7737710392 | 0.4390662017 | 1.046699847 |
| fcgr2b | 0.04534177262 | 0.05863915873 | 0.7732336821 | 0.4393840958 | 1.046385425 |
| prrt3 | -0.05039352373 | 0.06528130639 | -0.7719441677 | 0.4401474963 | 0.9508551668 |
| ervv_1 | 0.04907686042 | 0.06362749714 | 0.7713152745 | 0.4405200808 | 1.050301074 |
| pf4 | 0.04819439407 | 0.06272529557 | 0.7683406452 | 0.4422848332 | 1.049374628 |
| rarres2 | -0.04979571614 | 0.06484204203 | -0.7679541634 | 0.442514417 | 0.9514237652 |
| ndufs6 | -0.04625854134 | 0.06024928461 | -0.7677857362 | 0.44261449 | 0.9547950763 |
| cbln1 | -0.04814618968 | 0.06280619309 | -0.7665834739 | 0.4433292043 | 0.9529944589 |
| clstn2 | 0.04550428313 | 0.05937432903 | 0.7663965871 | 0.4434403629 | 1.046555487 |
| vegfb | 0.05225510089 | 0.06819727834 | 0.7662344034 | 0.4435368412 | 1.053644494 |
| lrrn1 | 0.04598517176 | 0.06017446462 | 0.7641974391 | 0.4447495925 | 1.047058885 |
| hmmr | 0.04827857029 | 0.0632462644 | 0.7633426377 | 0.44525908 | 1.049462964 |
| il34 | -0.04441134792 | 0.05822018634 | -0.7628170006 | 0.4455725408 | 0.9565603974 |
| rnf43 | -0.04931945847 | 0.06468697429 | -0.7624326074 | 0.4458018511 | 0.951876996 |
| rab3gap1 | -0.04784645449 | 0.06281247716 | -0.7617348758 | 0.4462182557 | 0.9532801477 |
| fgf20 | 0.04826810566 | 0.06342924194 | 0.7609756035 | 0.4466716392 | 1.049451982 |
| ctf1 | 0.04461188764 | 0.05865124908 | 0.7606297963 | 0.4468782175 | 1.045621962 |
| creb3 | -0.04834674931 | 0.06356936845 | -0.7605353096 | 0.4469346715 | 0.9528033459 |
| tmprss15 | 0.04515890324 | 0.05939797548 | 0.7602768087 | 0.4470891413 | 1.04619409 |
| atp6v1f | 0.04417396068 | 0.05810952324 | 0.7601845312 | 0.4471442901 | 1.045164157 |
| cgn | 0.04859288005 | 0.06394717538 | 0.7598909531 | 0.4473197698 | 1.049792872 |
| gp1ba | 0.04361159719 | 0.05741866545 | 0.7595369355 | 0.447531428 | 1.04457656 |
| areg | 0.04750203763 | 0.06256859745 | 0.7591993359 | 0.4477333233 | 1.048648338 |
| sirpb1 | -0.04489053417 | 0.05916070537 | -0.7587897049 | 0.4479783651 | 0.9561021366 |
| baiap2 | 0.04631296536 | 0.06107882187 | 0.7582491598 | 0.4483018366 | 1.04740216 |
| cxcl11 | -0.04476752569 | 0.059055264 | -0.7580615622 | 0.4484141292 | 0.9562197525 |
| trim21 | 0.04444541562 | 0.05867472532 | 0.7574882605 | 0.4487573965 | 1.04544791 |
| suox | 0.05062373206 | 0.06683389761 | 0.7574559298 | 0.4487767591 | 1.051927012 |
| siglec6 | 0.04388042204 | 0.0579705634 | 0.7569431701 | 0.4490839104 | 1.044857405 |

| Protein | beta | se | z | p_val | HR |
|---|---|---|---|---|---|
| rab44 | -0.04817874644 | 0.06365218369 | -0.7569064194 | 0.4491059292 | 0.952963433 |
| ofd1 | -0.04831272505 | 0.06384471244 | -0.756722416 | 0.4492161823 | 0.9528357649 |
| ednrb | 0.04916506971 | 0.06501340336 | 0.7562297491 | 0.4495114593 | 1.050393725 |
| fcgr3b | 0.04531328737 | 0.05999586154 | 0.7552735507 | 0.4500848652 | 1.046355619 |
| clec2l | -0.04799305188 | 0.06354512628 | -0.7552593675 | 0.4500933736 | 0.9531404096 |
| cndp1 | -0.04528242654 | 0.06001339094 | -0.7545387093 | 0.4505258108 | 0.9557275209 |
| cxcl12 | 0.04460610246 | 0.05912627352 | 0.754421001 | 0.4505964651 | 1.045615913 |
| ist1 | 0.04776540352 | 0.0633405814 | 0.7541042798 | 0.4507866077 | 1.048924552 |
| gh1 | 0.04912076777 | 0.06518499919 | 0.7535593829 | 0.4511138412 | 1.050347191 |
| palld | -0.04886347207 | 0.06485197491 | -0.7534615891 | 0.4511725848 | 0.9523111379 |
| pspn | -0.06079185526 | 0.08071641568 | -0.753153553 | 0.4513576465 | 0.9410190876 |
| adh4 | 0.05080493612 | 0.06750902748 | 0.7525650719 | 0.4517113132 | 1.052117643 |
| pcbp2 | 0.04750795925 | 0.06316922779 | 0.7520744026 | 0.4520063165 | 1.048654548 |
| robo1 | 0.04515431413 | 0.0600804498 | 0.7515641824 | 0.4523131898 | 1.046189289 |
| casp1 | 0.0438836297 | 0.05854478463 | 0.7495736806 | 0.4535115073 | 1.044860757 |
| il2ra | 0.04478471992 | 0.05975540149 | 0.7494673085 | 0.4535755956 | 1.045802695 |
| fabp5 | 0.04477925986 | 0.05977115008 | 0.7491784882 | 0.4537496331 | 1.045796985 |
| ptpn9 | 0.04851919242 | 0.06481271367 | 0.748606094 | 0.454094658 | 1.049715518 |
| parp1 | 0.04507059507 | 0.06031696574 | 0.7472291505 | 0.4549252508 | 1.046101707 |
| cd3e | 0.04738937406 | 0.06355848823 | 0.7456025997 | 0.455907512 | 1.0485302 |
| serpina9 | -0.04488638206 | 0.06030155127 | -0.744365296 | 0.4566555082 | 0.9561061065 |
| pltp | 0.04551905585 | 0.06127163293 | 0.7429058714 | 0.4575386707 | 1.046570948 |
| cst3 | -0.04848182128 | 0.06530060431 | -0.7424406219 | 0.4578204152 | 0.9526746576 |
| ankmy2 | 0.04730547178 | 0.06383915426 | 0.7410103145 | 0.4586871864 | 1.04844223 |
| ep300 | 0.04758762783 | 0.06453303539 | 0.7374149927 | 0.4608700208 | 1.048738096 |
| stoml2 | 0.04644250877 | 0.06304071365 | 0.7367065835 | 0.4613008024 | 1.047537853 |
| ankra2 | 0.04716900435 | 0.06409242802 | 0.7359528388 | 0.4617593993 | 1.048299161 |
| prkar2a | 0.04674130139 | 0.06351836877 | 0.7358706197 | 0.4618094388 | 1.047850896 |
| tyro3 | 0.04380933269 | 0.05954931464 | 0.73568156 | 0.4619245143 | 1.04478313 |
| rnf41 | 0.04374034282 | 0.05955832065 | 0.7344119569 | 0.4626977016 | 1.044711053 |
| rac3 | -0.04635879525 | 0.06312445087 | -0.734403145 | 0.4627030706 | 0.9546993591 |
| ptrhd1 | 0.04640428128 | 0.06320897041 | 0.7341407554 | 0.4628629567 | 1.047497809 |
| capg | 0.04384455829 | 0.05980610897 | 0.7331117012 | 0.463490304 | 1.044819934 |
| faslg | -0.045360359 | 0.06188093708 | -0.7330263753 | 0.4635423429 | 0.9556530416 |
| gucy2c | 0.04670040076 | 0.06374806799 | 0.7325775076 | 0.4638161537 | 1.04780804 |
| slurp1 | 0.04768385503 | 0.06511066124 | 0.7323509563 | 0.4639543849 | 1.048839018 |
| cdh17 | -0.04325067932 | 0.0590687195 | -0.7322095297 | 0.4640406887 | 0.9576712916 |
| dnm3 | 0.04793715922 | 0.06548902713 | 0.7319876523 | 0.4641761044 | 1.049104727 |
| anxa2 | 0.04799037234 | 0.06558499087 | 0.7317279717 | 0.46433462 | 1.049160554 |
| slc1a4 | 0.04723803703 | 0.06462740683 | 0.7309288636 | 0.4648226048 | 1.048371531 |
| gstp1 | -0.04321276349 | 0.05912565118 | -0.7308632147 | 0.4648627067 | 0.9577076032 |
| sec31a | 0.04684346524 | 0.06410180844 | 0.7307666722 | 0.4649216837 | 1.047957954 |
| il31ra | 0.04724245677 | 0.06483205745 | 0.7286897661 | 0.4661914543 | 1.048376164 |
| epha1 | -0.04762534305 | 0.06538872648 | -0.7283418046 | 0.4664043777 | 0.9534909522 |
| tnfrsf13b | -0.04306250416 | 0.05921525871 | -0.7272197251 | 0.4670913646 | 0.9578515185 |
| il20rb | 0.04685572764 | 0.06443771602 | 0.7271475547 | 0.4671355697 | 1.047970805 |
| gzma | -0.04283691185 | 0.05895235429 | -0.7266361516 | 0.4674488759 | 0.9580676268 |
| ceacam8 | -0.04446681869 | 0.06119999838 | -0.7265820239 | 0.4674820435 | 0.9565073377 |
| eno1 | 0.04222436821 | 0.05814261416 | 0.72622067 | 0.4677035024 | 1.043128497 |
| pnma1 | -0.0462263617 | 0.06376220319 | -0.7249806216 | 0.4684639188 | 0.9548258017 |
| dut | 0.04531113352 | 0.06283453134 | 0.7211183493 | 0.4708367024 | 1.046353365 |
| pon3 | 0.04823810461 | 0.06690122231 | 0.7210347277 | 0.4708881485 | 1.049420497 |
| gngt1 | -0.04545237294 | 0.06315085187 | -0.7197428315 | 0.4716833495 | 0.9555651122 |
| clec3b | 0.04625065628 | 0.06427513179 | 0.7195731069 | 0.4717878751 | 1.0473369 |
| fas | 0.0473667296 | 0.06591480716 | 0.7186052974 | 0.4723841485 | 1.048506457 |
| mapre3 | -0.04626081451 | 0.06438909635 | -0.718457272 | 0.4724753844 | 0.9547929059 |
| pkd1 | 0.04853020751 | 0.06762824553 | 0.7176026396 | 0.4730023296 | 1.049727081 |
| cd274 | -0.04347663445 | 0.06071177563 | -0.7161153499 | 0.4739201264 | 0.9574549253 |
| s100a4 | 0.04196483803 | 0.05862217853 | 0.7158525848 | 0.4740823787 | 1.042857809 |
| tp53i3 | 0.04642315048 | 0.06490649944 | 0.7152311537 | 0.4744662217 | 1.047517575 |
| artn | 0.04178851972 | 0.05845670349 | 0.7148627485 | 0.4746938573 | 1.04267395 |
| cd164l2 | -0.04568030549 | 0.0639009386 | -0.7148612601 | 0.4746947771 | 0.9553473327 |
| il36g | 0.046284816 | 0.06478745134 | 0.7144101989 | 0.4749735673 | 1.047372677 |
| ctag1a_ctag1b | -0.04542781493 | 0.06364503186 | -0.7137684373 | 0.4753703797 | 0.9555885793 |
| pmm2 | 0.04593803311 | 0.0644626371 | 0.7126303728 | 0.476074512 | 1.047009529 |
| bsnd | 0.04546356073 | 0.06381762977 | 0.7123981397 | 0.4762182672 | 1.04651287 |
| bloc1s3 | 0.04494962742 | 0.06320103645 | 0.7112166183 | 0.4769500121 | 1.04597517 |
| mtss2 | 0.04535545991 | 0.06377195382 | 0.7111997734 | 0.476960449 | 1.046398846 |
| hmox2 | -0.042540825 | 0.05987633442 | -0.7104781116 | 0.4774076997 | 0.95835134 |
| lilrb5 | -0.04113498477 | 0.05799939377 | -0.7092312884 | 0.4781809601 | 0.9596995764 |

| Protein | beta | se | z | p_val | HR |
|---|---|---|---|---|---|
| ufd1 | -0.04468059066 | 0.06303112237 | -0.708865541 | 0.4784079208 | 0.9563028851 |
| cxcl16 | -0.04281125319 | 0.06050794411 | -0.7075311156 | 0.4792364833 | 0.9580922098 |
| nectin2 | 0.04237094375 | 0.05988840184 | 0.7074983211 | 0.4792568557 | 1.043281406 |
| ctsb | -0.04189455266 | 0.05926518773 | -0.7068998558 | 0.4796287136 | 0.9589708962 |
| casp4 | -0.04457067121 | 0.06307112943 | -0.7066731104 | 0.4797696435 | 0.9564080072 |
| pnliprp1 | 0.04348060037 | 0.06185695651 | 0.7029217541 | 0.4821045132 | 1.044439732 |
| krt17 | 0.04507727278 | 0.06414333788 | 0.7027584512 | 0.4822062943 | 1.046108693 |
| tnpo1 | 0.04465239762 | 0.06364321542 | 0.7016049917 | 0.4829255378 | 1.045664321 |
| defa1_defa1b | -0.04181064994 | 0.05960995507 | -0.7014038157 | 0.4830510414 | 0.9590513598 |
| rspo3 | -0.04362992309 | 0.06225037656 | -0.7008780589 | 0.4833791183 | 0.9573081696 |
| anxa10 | -0.04164828109 | 0.05949013637 | -0.7000871679 | 0.4838728691 | 0.9592070925 |
| tank | -0.04128066991 | 0.05901207606 | -0.6995291925 | 0.4842213759 | 0.9595597726 |
| nup50 | 0.04443389856 | 0.06375429857 | 0.6969553357 | 0.485830746 | 1.04543587 |
| srp14 | 0.04213343982 | 0.06045434804 | 0.6969463931 | 0.4858363426 | 1.043033652 |
| il1rl1 | 0.04395359298 | 0.06307452436 | 0.6968517547 | 0.4858955732 | 1.044933861 |
| ap2b1 | 0.04508944278 | 0.0647263908 | 0.6966160513 | 0.4860431079 | 1.046121424 |
| gadd45gip1 | 0.04471978045 | 0.06423526948 | 0.6961873253 | 0.4863115242 | 1.045734784 |
| angptl1 | 0.04267035813 | 0.06129221687 | 0.6961790633 | 0.4863166976 | 1.043593826 |
| yod1 | 0.04522209595 | 0.06499062414 | 0.6958249216 | 0.4865384799 | 1.046260204 |
| cd160 | 0.0397953 | 0.05722261197 | 0.6954471078 | 0.4867751473 | 1.040597742 |
| bmp6 | -0.04096545971 | 0.05892714652 | -0.6951882472 | 0.4869373367 | 0.9598622833 |
| il13 | -0.04075223516 | 0.05866160282 | -0.6947003354 | 0.4872431178 | 0.9600669713 |
| anp32c | 0.04394149634 | 0.06326342872 | 0.6945797474 | 0.487318708 | 1.044921221 |
| sptbn2 | 0.04410270173 | 0.06354797465 | 0.6940064097 | 0.4876781893 | 1.045089682 |
| mybpc1 | -0.04901938682 | 0.07064204266 | -0.693912364 | 0.4877371693 | 0.9521626701 |
| ceacam1 | 0.04030816321 | 0.05809325702 | 0.6938526996 | 0.4877745894 | 1.041131563 |
| cntn4 | 0.04139838928 | 0.05966988798 | 0.6937902966 | 0.4878137288 | 1.042267251 |
| fes | -0.04114430467 | 0.05931568497 | -0.6936496593 | 0.4879019431 | 0.9596906321 |
| coq7 | 0.04495908482 | 0.06487234337 | 0.6930393213 | 0.4882848754 | 1.045985062 |
| gla | 0.04698432889 | 0.06787057829 | 0.6922635709 | 0.4887718229 | 1.048105584 |
| crhbp | 0.04224324833 | 0.06104852901 | 0.6919617724 | 0.4889613361 | 1.043148192 |
| nrxn3 | -0.04405341083 | 0.06369889815 | -0.6915882709 | 0.4891959296 | 0.9569028471 |
| enpp7 | -0.04270630545 | 0.06175682492 | -0.6915236577 | 0.4892365188 | 0.9581927647 |
| ppbp | 0.04357118784 | 0.06304290874 | 0.6911354299 | 0.4894804369 | 1.04453435 |
| gabarap | 0.04459971851 | 0.06453646205 | 0.6910778355 | 0.4895166283 | 1.045609238 |
| susd5 | 0.04751730556 | 0.06875887454 | 0.6910716018 | 0.4895205455 | 1.048664349 |
| boc | 0.04229828229 | 0.06123160056 | 0.6907917138 | 0.4896964436 | 1.043205602 |
| ddhd2 | 0.04378932033 | 0.06341600498 | 0.6905089708 | 0.4898741705 | 1.044762222 |
| ang | -0.04304824283 | 0.06234491427 | -0.6904852358 | 0.4898890915 | 0.9578651788 |
| bag4 | 0.04552957231 | 0.06598896739 | 0.6899573385 | 0.490221016 | 1.046581954 |
| hdgfl2 | 0.04434044337 | 0.06430016513 | 0.6895852177 | 0.4904550661 | 1.045338173 |
| smad3 | -0.04380082272 | 0.06356661 | -0.6890539345 | 0.4907893274 | 0.9571445799 |
| clec7a | -0.03962172146 | 0.05751637014 | -0.6888772947 | 0.4909004889 | 0.9611529539 |
| efemp1 | -0.04422242952 | 0.06429915227 | -0.6877606929 | 0.4916034928 | 0.9567411263 |
| ezr | 0.04149561723 | 0.06041156968 | 0.6868819574 | 0.4921571176 | 1.042368593 |
| rilpl2 | 0.04341220185 | 0.06325394902 | 0.6863160722 | 0.492513816 | 1.044368297 |
| mapk9 | -0.04083931692 | 0.0595397244 | -0.6859171306 | 0.492765367 | 0.9599833706 |
| fcrl2 | 0.03930576955 | 0.05738036078 | 0.6850038762 | 0.493341475 | 1.040088462 |
| prelp | 0.0409669685 | 0.05984719385 | 0.6845261384 | 0.4936429898 | 1.041817692 |
| plin1 | 0.04401648961 | 0.06434314678 | 0.6840897876 | 0.4939184702 | 1.044999586 |
| il1a | 0.03953467514 | 0.05788552478 | 0.6829803355 | 0.4946192685 | 1.040326572 |
| gpr101 | 0.04300293656 | 0.06301425134 | 0.6824319206 | 0.4949658773 | 1.04394096 |
| pdgfrb | 0.03947407396 | 0.05784674327 | 0.6823906019 | 0.4949919968 | 1.040263529 |
| hsd17b14 | 0.04295543793 | 0.06307020393 | 0.6810733951 | 0.4958250505 | 1.043891376 |
| sema6c | 0.04405781555 | 0.06477492158 | 0.6801677945 | 0.4963982219 | 1.045042773 |
| trim25 | 0.04271025948 | 0.06288517488 | 0.6791785117 | 0.4970247609 | 1.043635468 |
| ripk4 | 0.04327259697 | 0.06373115956 | 0.6789865 | 0.4971464158 | 1.044222508 |
| itgam | -0.03999028031 | 0.05891175649 | -0.6788166352 | 0.4972540521 | 0.9607987778 |
| ccl7 | 0.04217814675 | 0.06224300661 | 0.6776367186 | 0.4980020588 | 1.043080284 |
| igfbp2 | -0.04507342724 | 0.06655131348 | -0.6772732931 | 0.4982325725 | 0.9559272882 |
| ppt1 | -0.04331910809 | 0.06400121565 | -0.6768482075 | 0.498502268 | 0.9576057616 |
| adamts8 | -0.03986687159 | 0.05890633198 | -0.6767841461 | 0.4985429185 | 0.960917356 |
| robo2 | 0.04159712861 | 0.06146809453 | 0.6767271529 | 0.4985790853 | 1.042474411 |
| pyy | -0.04362164809 | 0.06452560572 | -0.6760362434 | 0.4990176342 | 0.9573160913 |
| uso1 | 0.03944394811 | 0.05836898617 | 0.6757689435 | 0.4991873555 | 1.04023219 |
| txnl1 | 0.04325670855 | 0.06407716213 | 0.6750721647 | 0.4996299173 | 1.044205917 |
| morf4l1 | -0.04333801538 | 0.06430200244 | -0.6739761397 | 0.5003264833 | 0.957587656 |
| nfe2 | 0.04278868333 | 0.06370563146 | 0.6716624942 | 0.5017985834 | 1.043717317 |
| gmpr2 | 0.04225522082 | 0.06291626512 | 0.6716104452 | 0.5018317269 | 1.043160681 |
| morn4 | -0.04292564917 | 0.06392953364 | -0.6714525623 | 0.5019322698 | 0.9579826142 |

| Protein | beta | se | z | p_val | HR |
|---|---|---|---|---|---|
| nme1 | -0.04308262316 | 0.06416646918 | -0.6714195703 | 0.5019532812 | 0.9578322477 |
| cirbp | 0.04236641048 | 0.06313243695 | 0.671071996 | 0.5021746658 | 1.043276676 |
| tgfbr2 | 0.04277898734 | 0.06375928275 | 0.6709452411 | 0.5022554142 | 1.043707197 |
| rtkn2 | -0.04238070265 | 0.06320520747 | -0.670525489 | 0.5025228634 | 0.9585048058 |
| nlgn1 | 0.04220156008 | 0.06300887162 | 0.6697717163 | 0.5030033263 | 1.043104706 |
| leo1 | -0.04192549131 | 0.06267728641 | -0.6689104413 | 0.5035526093 | 0.9589412274 |
| il22ra1 | 0.03995057866 | 0.05978501891 | 0.6682372839 | 0.5039821396 | 1.040759337 |
| sftpa1 | -0.03987011805 | 0.05974378716 | -0.6673517022 | 0.5045475086 | 0.9609142365 |
| pilrb | 0.03899401795 | 0.05844006671 | 0.6672480053 | 0.5046137321 | 1.039764264 |
| tpsab1 | -0.03882731834 | 0.0582849371 | -0.6661638542 | 0.5053063738 | 0.9619168002 |
| lag3 | -0.04075322927 | 0.06125722855 | -0.6652803307 | 0.5058712088 | 0.9600660169 |
| egflam | 0.04189407563 | 0.06298935608 | 0.6650976965 | 0.5059880079 | 1.042784017 |
| bgn | -0.03918246049 | 0.05894504504 | -0.6647286547 | 0.5062240626 | 0.9615752436 |
| lamp1 | 0.04312577609 | 0.06497649463 | 0.663713491 | 0.506873703 | 1.044069206 |
| glyr1 | 0.04254464394 | 0.06415522193 | 0.6631516916 | 0.5072334073 | 1.04346264 |
| wnt9a | 0.04345747643 | 0.06555115393 | 0.6629551706 | 0.5073592658 | 1.044415581 |
| ccl8 | -0.03839178532 | 0.05802022562 | -0.6616965878 | 0.5081656919 | 0.962335838 |
| wars | -0.03881773914 | 0.05881297598 | -0.6600199784 | 0.5092410087 | 0.9619260146 |
| proc | 0.03948490954 | 0.05984270642 | 0.659811561 | 0.5093747633 | 1.040274801 |
| agr3 | 0.03944439171 | 0.05983102915 | 0.6592631326 | 0.5097268124 | 1.040232652 |
| cntn3 | -0.0439757693 | 0.0667299023 | -0.6590114444 | 0.5098884197 | 0.9569771454 |
| tax1bp1 | 0.04155597122 | 0.06311840761 | 0.6583811728 | 0.5102932302 | 1.042431506 |
| ssc4d | -0.04541142012 | 0.06898006893 | -0.6583266851 | 0.5103282344 | 0.9556042461 |
| appl2 | 0.04117577074 | 0.06256420824 | 0.6581362075 | 0.5104506117 | 1.042035249 |
| chgb | -0.0395720889 | 0.06020641067 | -0.6572736766 | 0.5110049592 | 0.9612006596 |
| nptx2 | 0.04924184731 | 0.07502255265 | 0.6563605952 | 0.5115921379 | 1.050474374 |
| cxcl5 | -0.03925592391 | 0.05985503443 | -0.6558499929 | 0.5119206464 | 0.9615046056 |
| fen1 | 0.03863873411 | 0.05893053782 | 0.6556657302 | 0.5120392233 | 1.039394918 |
| ephb6 | 0.03814705892 | 0.05818185896 | 0.6556521156 | 0.5120479852 | 1.038883999 |
| akr1b1 | 0.03845755224 | 0.05869293187 | 0.6552331092 | 0.5123176803 | 1.039206615 |
| tmem106a | -0.04159271879 | 0.0636536281 | -0.6534225941 | 0.5134838767 | 0.9592603898 |
| dctd | 0.04109661547 | 0.06291157904 | 0.6532440625 | 0.513598948 | 1.041952769 |
| dtd1 | 0.04137077134 | 0.06337942888 | 0.6527476197 | 0.5139189973 | 1.042238466 |
| aoc1 | -0.03822072246 | 0.05856918176 | -0.6525739529 | 0.5140309822 | 0.962500472 |
| klrc1 | -0.04101608238 | 0.06297938647 | -0.651262019 | 0.5148773616 | 0.9598136937 |
| fut1 | -0.04147158518 | 0.06368862679 | -0.6511615538 | 0.5149422054 | 0.9593765955 |
| snx18 | 0.04062008128 | 0.06241884565 | 0.6507663008 | 0.515197357 | 1.041456362 |
| vwf | 0.0386215043 | 0.0594606344 | 0.6495306464 | 0.5159954443 | 1.039377009 |
| commd9 | -0.04161750185 | 0.06408575976 | -0.6494032684 | 0.5160777519 | 0.9592366166 |
| nmnat1 | 0.03893960974 | 0.06003561303 | 0.6486085138 | 0.5165914511 | 1.039707694 |
| dner | 0.03979242386 | 0.06141595053 | 0.6479167629 | 0.5170387881 | 1.040594749 |
| hdgf | 0.03724467917 | 0.05748638102 | 0.647887004 | 0.5170580369 | 1.037946954 |
| sestd1 | 0.03741848024 | 0.05776828466 | 0.6477339679 | 0.5171570303 | 1.038127366 |
| enpp5 | -0.04186615375 | 0.0647000642 | -0.6470805596 | 0.5175798064 | 0.9589981303 |
| cd38 | -0.04646902133 | 0.07182398123 | -0.646984761 | 0.5176418062 | 0.9545941322 |
| pglyrp1 | -0.03803541781 | 0.05884359823 | -0.6463815768 | 0.5180322687 | 0.9626788443 |
| folr3 | -0.03898865477 | 0.060406727 | -0.6454356444 | 0.5186449106 | 0.9617616205 |
| smoc2 | -0.04167550168 | 0.06466241476 | -0.6445088981 | 0.5192454895 | 0.9591809827 |
| clc | -0.0386651211 | 0.06001255738 | -0.6442838431 | 0.5193913908 | 0.9620728331 |
| pcdh1 | 0.03891080906 | 0.06041248197 | 0.6440855894 | 0.5195199345 | 1.03967775 |
| fgl1 | 0.04278682607 | 0.06657228419 | 0.6427123027 | 0.5204107966 | 1.043715378 |
| casp8 | 0.03769995597 | 0.05873814984 | 0.6418308386 | 0.520983024 | 1.038419615 |
| fcrlb | -0.03879570497 | 0.06044715665 | -0.6418119085 | 0.5209953166 | 0.9619472101 |
| nlgn2 | -0.04081812851 | 0.06362619942 | -0.6415302012 | 0.5211782654 | 0.9600037114 |
| ensa | 0.04111598446 | 0.06419678613 | 0.6404679569 | 0.5218684147 | 1.041972951 |
| gfral | 0.04256088653 | 0.06657314422 | 0.6393101457 | 0.5226211895 | 1.043479588 |
| pon1 | -0.04021719861 | 0.06302056583 | -0.6381599099 | 0.5233695911 | 0.9605807797 |
| myoc | -0.03876103228 | 0.06075159451 | -0.6380249374 | 0.5234574471 | 0.961980564 |
| cdc25a | -0.04118229899 | 0.06457258982 | -0.6377674971 | 0.5236250404 | 0.95965417 |
| acy1 | -0.04322148282 | 0.06783195865 | -0.6371846498 | 0.5240045751 | 0.9576992526 |
| tafa5 | 0.03770464268 | 0.05926944286 | 0.6361565228 | 0.5246744078 | 1.038424481 |
| myom3 | -0.04325079741 | 0.06811009185 | -0.6350130537 | 0.5254199014 | 0.9576711785 |
| adamts1 | 0.04154597932 | 0.06557323425 | 0.6335813659 | 0.5263540649 | 1.042421091 |
| impa1 | 0.03754154266 | 0.05931280083 | 0.6329416608 | 0.5267717408 | 1.038255128 |
| hgs | 0.03682513213 | 0.05818773862 | 0.6328675595 | 0.5268201339 | 1.037511578 |
| vsnl1 | 0.04451724086 | 0.07039353174 | 0.6324052759 | 0.5271220874 | 1.045523002 |
| lamp3 | -0.03990009387 | 0.06311867057 | -0.632144079 | 0.5272927344 | 0.9608854327 |
| cntn1 | -0.03909664163 | 0.06188867048 | -0.6317253438 | 0.5275663644 | 0.9616577685 |
| vegfc | -0.03681113566 | 0.05829529784 | -0.631459775 | 0.5277399425 | 0.9638581566 |
| celsr2 | -0.03960902087 | 0.06280117182 | -0.6307051242 | 0.528233348 | 0.9611651612 |

| Protein | beta | se | z | p_val | HR |
|---|---|---|---|---|---|
| kcnc4 | 0.04002661454 | 0.06360936075 | 0.6292566702 | 0.5291810335 | 1.040838475 |
| tnfsf13 | 0.03842910157 | 0.06115094508 | 0.628430215 | 0.5297221487 | 1.03917705 |
| lamp2 | 0.03954201104 | 0.06295192325 | 0.6281303096 | 0.529918579 | 1.040334203 |
| amot | -0.04444630936 | 0.07085249971 | -0.6273075691 | 0.5304576428 | 0.9565269553 |
| ahsa1 | 0.03990582123 | 0.06367329015 | 0.6267278027 | 0.5308376759 | 1.040712757 |
| ogn | 0.03975199677 | 0.06369197171 | 0.6241288454 | 0.5325429716 | 1.040552682 |
| apoa4 | 0.04008582301 | 0.06425636933 | 0.6238420164 | 0.5327313431 | 1.040900104 |
| fis1 | 0.03680995478 | 0.05902083182 | 0.6236773296 | 0.5328395143 | 1.037495831 |
| cdhr2 | 0.04741919915 | 0.07604302616 | 0.6235837991 | 0.532900953 | 1.048561473 |
| nfkbie | 0.03708330133 | 0.05950659813 | 0.6231796557 | 0.5331664692 | 1.037779466 |
| hk2 | 0.03704012819 | 0.05943857409 | 0.6231665002 | 0.5331751133 | 1.037734662 |
| pbk | -0.03927104354 | 0.06303491983 | -0.6230045766 | 0.5332815144 | 0.9614900682 |
| znf830 | -0.03940875745 | 0.06330552604 | -0.6225168625 | 0.5336020593 | 0.9613576667 |
| itm2a | -0.03603606295 | 0.05791642407 | -0.6222080097 | 0.5338051 | 0.9646055063 |
| tp73 | 0.03880558569 | 0.06238573018 | 0.6220266329 | 0.5339243557 | 1.039568357 |
| ppm1b | 0.04028740328 | 0.06478202653 | 0.6218916795 | 0.5340130967 | 1.04110995 |
| tnn | 0.03900173879 | 0.06274615924 | 0.6215796992 | 0.5342182732 | 1.039772292 |
| postn | 0.03928036608 | 0.06319827465 | 0.6215417477 | 0.5342432351 | 1.040062041 |
| eno3 | 0.04365247759 | 0.07025156749 | 0.6213737166 | 0.5343537613 | 1.044619263 |
| trem2 | 0.03986583676 | 0.06426126535 | 0.6203711759 | 0.5350134447 | 1.040671145 |
| rab6b | 0.03581608755 | 0.05775633769 | 0.6201239376 | 0.5351761936 | 1.03646521 |
| becn1 | 0.04027225657 | 0.06497716869 | 0.6197908802 | 0.5353954736 | 1.04109418 |
| nagk | 0.04091498582 | 0.06603756865 | 0.619571354 | 0.5355400312 | 1.041763537 |
| cspg5 | -0.03919856405 | 0.06327824921 | -0.6194634734 | 0.5356110776 | 0.961559759 |
| hs1bp3 | 0.04024853939 | 0.0650306678 | 0.6189162859 | 0.5359715091 | 1.041069489 |
| ocln | -0.04250373148 | 0.06867986955 | -0.6188673881 | 0.536003724 | 0.9583868893 |
| dkk3 | -0.03809480516 | 0.06155932828 | -0.6188307478 | 0.536027864 | 0.9626216751 |
| eln | -0.0515385232 | 0.08331141004 | -0.618625026 | 0.5361634114 | 0.9497670612 |
| arl13b | -0.03952036602 | 0.06389061896 | -0.6185628917 | 0.5362043542 | 0.9612503769 |
| scgb3a2 | 0.03782057742 | 0.06134493628 | 0.6165232164 | 0.537549254 | 1.038544878 |
| dcdc2c | 0.03887457633 | 0.06310756519 | 0.6160050101 | 0.5378912132 | 1.03964008 |
| reck | 0.04203220051 | 0.06827540448 | 0.6156272647 | 0.5381405524 | 1.042928061 |
| c1galt1c1 | 0.03930841655 | 0.06390993886 | 0.6150595237 | 0.5385154115 | 1.040091216 |
| tcl1b | -0.03563267841 | 0.05798341175 | -0.6145322832 | 0.5388636468 | 0.9649946918 |
| dennd2b | 0.03916039764 | 0.06375313677 | 0.6142505236 | 0.5390497915 | 1.039937274 |
| arhgap25 | -0.03636083907 | 0.05923285516 | -0.6138626776 | 0.539306075 | 0.9642922764 |
| egln1 | 0.03720879496 | 0.06063707057 | 0.6136311435 | 0.5394590987 | 1.037909709 |
| prcp | 0.03889738932 | 0.06340540854 | 0.6134711567 | 0.5395648487 | 1.039663798 |
| sirpa | 0.03513419304 | 0.0573196059 | 0.6129524529 | 0.539907779 | 1.035758691 |
| traf3 | -0.04034679385 | 0.06585930005 | -0.6126210545 | 0.5401269334 | 0.9604563011 |
| per3 | 0.03885327904 | 0.06367470015 | 0.610183934 | 0.5417399711 | 1.039617939 |
| eddm3b | -0.05971293705 | 0.09809754721 | -0.6087097868 | 0.5427168188 | 0.9420349181 |
| kir2ds4 | 0.03776257897 | 0.06204101571 | 0.6086711918 | 0.5427424057 | 1.038484646 |
| rgs10 | 0.0379412063 | 0.06245061001 | 0.6075394028 | 0.5434930016 | 1.038670164 |
| tlr3 | 0.03557479799 | 0.05860703253 | 0.6070056178 | 0.543847184 | 1.036215152 |
| bmp10 | -0.04048233848 | 0.06676105334 | -0.6063765691 | 0.5442647239 | 0.9603261252 |
| rbm25 | -0.03848538299 | 0.06346804578 | -0.6063741606 | 0.5442663229 | 0.9622457698 |
| icam2 | 0.03478175946 | 0.05748231207 | 0.6050862989 | 0.5451216574 | 1.035393719 |
| adamtsl5 | -0.03876499168 | 0.06409625475 | -0.6047933975 | 0.5453162812 | 0.9619767551 |
| apof | -0.04286456446 | 0.0710199901 | -0.6035563282 | 0.5461386552 | 0.9580411341 |
| tbca | 0.03836746868 | 0.063571374 | 0.6035337332 | 0.5461536816 | 1.039113004 |
| pcyt2 | 0.03817562701 | 0.06335001866 | 0.6026142978 | 0.5467653066 | 1.038913678 |
| bcl2l1 | 0.03777116183 | 0.06270092865 | 0.6024019522 | 0.5469066108 | 1.038493559 |
| cxcl3 | -0.03516319861 | 0.05837452539 | -0.6023723255 | 0.5469263272 | 0.9654478437 |
| pdp1 | 0.03521997539 | 0.05850193819 | 0.6020309153 | 0.5471535589 | 1.035847545 |
| prdx5 | 0.03500313085 | 0.05821972147 | 0.6012246359 | 0.5476903778 | 1.035622951 |
| apom | -0.03630833342 | 0.06039951879 | -0.6011361372 | 0.5477493159 | 0.9643429085 |
| tnxb | 0.03591212319 | 0.05978001189 | 0.6007379733 | 0.5480145227 | 1.036564752 |
| mllt1 | -0.03879362535 | 0.06474355726 | -0.5991889694 | 0.5490468777 | 0.9619492106 |
| gsta1 | 0.03959204705 | 0.06610814393 | 0.5988981794 | 0.5492407856 | 1.040386259 |
| coro1a | 0.03482887769 | 0.05815795317 | 0.5988669785 | 0.5492615934 | 1.035442506 |
| dsc2 | 0.03670579616 | 0.06132765162 | 0.5985195126 | 0.5494933432 | 1.037387772 |
| shd | -0.0378062418 | 0.06320292197 | -0.5981723728 | 0.5497249236 | 0.9628994925 |
| dusp3 | -0.0343760344 | 0.05754200083 | -0.5974077005 | 0.5502352138 | 0.9662081088 |
| hla_e | -0.03635506773 | 0.06097389513 | -0.5962398769 | 0.5510149896 | 0.9642978416 |
| sema3g | 0.03737901869 | 0.06269587175 | 0.5961958523 | 0.5510443962 | 1.0380864 |
| otud6b | 0.03775385879 | 0.06344244182 | 0.5950883621 | 0.5517844075 | 1.03847559 |
| mrpl58 | 0.03706870625 | 0.06229787461 | 0.59502361 | 0.5518276891 | 1.037764319 |
| b3gnt7 | 0.03952159177 | 0.06649129702 | 0.5943874392 | 0.5522530077 | 1.040312961 |
| nptx1 | 0.03684618853 | 0.06199515364 | 0.5943398212 | 0.5522848497 | 1.037533424 |

| Protein | beta | se | z | p_val | HR |
|---|---|---|---|---|---|
| slamf1 | -0.03500624354 | 0.05897635219 | -0.5935640684 | 0.5528037195 | 0.9655993875 |
| c7orf50 | 0.03773860195 | 0.06368036133 | 0.5926254369 | 0.5534318519 | 1.038459746 |
| bcan | -0.0372705994 | 0.06290904494 | -0.5924521575 | 0.5535478487 | 0.9634154004 |
| adgrg1 | -0.03656041769 | 0.06190357626 | -0.5906026743 | 0.5547866726 | 0.9640998434 |
| fkbp4 | 0.03517851244 | 0.05958699449 | 0.5903723243 | 0.5549410609 | 1.035804596 |
| sned1 | 0.03848209978 | 0.06520256873 | 0.590193002 | 0.5550612633 | 1.039232126 |
| gorasp2 | -0.03740803696 | 0.06338897994 | -0.5901347046 | 0.5551003436 | 0.9632830001 |
| esyt2 | 0.03729912537 | 0.06321071658 | 0.5900759775 | 0.5551397133 | 1.038003468 |
| pgf | 0.0402155423 | 0.06853196228 | 0.5868143996 | 0.5573283648 | 1.041035137 |
| dnajc6 | 0.03769974419 | 0.06428226177 | 0.5864719622 | 0.5575583977 | 1.038419395 |
| ctnna1 | -0.03699285408 | 0.06308587391 | -0.5863888662 | 0.5576142245 | 0.9636830217 |
| pxn | -0.03531090822 | 0.06023237876 | -0.5862446237 | 0.5577111383 | 0.9653052483 |
| clmp | -0.0395286176 | 0.06747797288 | -0.5858003125 | 0.5580097137 | 0.9612424451 |
| ghr | 0.04065828453 | 0.06954937407 | 0.5845959806 | 0.558819411 | 1.041496149 |
| fdx2 | 0.03738383884 | 0.06397762234 | 0.5843267923 | 0.5590004698 | 1.038091404 |
| angpt2 | -0.03580082521 | 0.06137997985 | -0.5832655093 | 0.5597145773 | 0.9648324447 |
| prl | -0.03475444849 | 0.05959064047 | -0.5832199188 | 0.5597452638 | 0.9658425512 |
| htr1a | 0.0365825518 | 0.06276119543 | 0.582884879 | 0.5599708005 | 1.037259928 |
| cd59 | -0.03868091881 | 0.06642583233 | -0.5823174126 | 0.5603528989 | 0.9620576347 |
| actn4 | -0.03407125585 | 0.05854959943 | -0.581921246 | 0.5606197289 | 0.9665026332 |
| alms1 | -0.03691479467 | 0.06348228491 | -0.581497574 | 0.5609051528 | 0.9637582492 |
| lgals1 | -0.03734165315 | 0.06425370997 | -0.5811594874 | 0.561132969 | 0.9633469486 |
| ahsg | -0.03664072097 | 0.06310504754 | -0.5806305898 | 0.5614894511 | 0.9640224262 |
| shmt1 | 0.03430693664 | 0.05920665592 | 0.5794439174 | 0.562289678 | 1.034902207 |
| asgr2 | -0.03698649781 | 0.0638365752 | -0.5793935169 | 0.5623236776 | 0.9636891472 |
| galnt2 | -0.03372557794 | 0.05822565061 | -0.5792220024 | 0.5624393865 | 0.9668307896 |
| gipc3 | 0.03651041254 | 0.06304664994 | 0.5791015475 | 0.5625206559 | 1.037185104 |
| crh | -0.03389239699 | 0.05852710907 | -0.5790888621 | 0.562529215 | 0.9666755162 |
| lypd8 | 0.03372183559 | 0.05823638478 | 0.5790509785 | 0.5625547759 | 1.034296862 |
| fadd | 0.03400698372 | 0.05875039489 | 0.5788383853 | 0.5626982279 | 1.034591832 |
| pecr | 0.03665522741 | 0.06348187858 | 0.5774124558 | 0.5636608619 | 1.037335314 |
| erbb3 | 0.03606062242 | 0.06246828711 | 0.5772628655 | 0.5637618951 | 1.036718693 |
| ppm1f | 0.03678913026 | 0.06376313888 | 0.5769654837 | 0.5639627726 | 1.037474226 |
| bag6 | -0.03470701608 | 0.06033049052 | -0.5752815164 | 0.56510092 | 0.9658883646 |
| gdf2 | 0.03664550147 | 0.06373056219 | 0.575006719 | 0.5652867529 | 1.037325225 |
| il18rap | -0.03321982901 | 0.05779663957 | -0.5747709427 | 0.5654462209 | 0.9673258899 |
| ceacam19 | 0.03692127167 | 0.06425907289 | 0.5745690064 | 0.5655828183 | 1.037611328 |
| lrig3 | -0.03844721526 | 0.06696653195 | -0.5741258228 | 0.5658826602 | 0.9622824972 |
| csf3r | 0.03709353948 | 0.0647469951 | 0.5728997835 | 0.5667125512 | 1.037790091 |
| spint3 | -0.0584511907 | 0.1020594592 | -0.5727170337 | 0.5668363023 | 0.9432242774 |
| inpp5d | 0.03732651045 | 0.06524769491 | 0.5720740097 | 0.567271836 | 1.038031894 |
| icam1 | 0.03490232832 | 0.06103924577 | 0.571801435 | 0.567456505 | 1.035518563 |
| palm3 | -0.03646659203 | 0.06381092204 | -0.5714788451 | 0.5676750964 | 0.964190305 |
| vasp | 0.03578649936 | 0.06265939232 | 0.5711274565 | 0.5679132481 | 1.036434543 |
| ulbp2 | 0.03431306605 | 0.06008116877 | 0.5711118268 | 0.5679238422 | 1.034908551 |
| jam2 | 0.03445147838 | 0.06036433074 | 0.5707257574 | 0.5681855559 | 1.035051805 |
| gpr15l | 0.03849238104 | 0.06744703367 | 0.5707053216 | 0.5681994109 | 1.03924281 |
| ereg | -0.03375809764 | 0.05920385152 | -0.5702010388 | 0.5685413516 | 0.9668053488 |
| ddx25 | 0.0360342804 | 0.06323380023 | 0.5698578968 | 0.5687740833 | 1.036691384 |
| pde5a | 0.03566538925 | 0.06260362039 | 0.5697017046 | 0.5688800337 | 1.036309028 |
| stx4 | 0.03415355749 | 0.0599555403 | 0.5696480646 | 0.5689164218 | 1.034743487 |
| hspa2 | 0.03709334652 | 0.06514947774 | 0.5693575422 | 0.569113524 | 1.03778989 |
| mog | -0.034101393 | 0.05995485511 | -0.5687845119 | 0.5695023867 | 0.966473506 |
| qdpr | 0.0339727989 | 0.05977262301 | 0.5683672088 | 0.5697856515 | 1.034556465 |
| arhgef5 | 0.0369595375 | 0.06503399994 | 0.5683109994 | 0.5698238115 | 1.037651034 |
| ifnar1 | -0.03605283249 | 0.06344310981 | -0.5682702598 | 0.56985147 | 0.9645893305 |
| arsb | 0.03374396734 | 0.05938749277 | 0.5681998982 | 0.5698992406 | 1.034319753 |
| cdh1 | 0.03507131531 | 0.0617305646 | 0.5681353401 | 0.5699430727 | 1.035693567 |
| pof1b | 0.03773782758 | 0.06643396404 | 0.5680502154 | 0.5700008711 | 1.038458942 |
| gbp2 | 0.03366541535 | 0.05932423331 | 0.5674816761 | 0.5703869725 | 1.034238509 |
| mstn | 0.03681871128 | 0.06492445095 | 0.5671008494 | 0.5706456659 | 1.037504916 |
| slit2 | -0.03309040121 | 0.05835962412 | -0.5670084705 | 0.5707084267 | 0.9674510969 |
| tnc | 0.03318887382 | 0.0585729632 | 0.566624463 | 0.570969351 | 1.033745768 |
| reg3a | 0.03418752009 | 0.06034170438 | 0.5665653704 | 0.5710095081 | 1.03477863 |
| hrg | 0.03624103011 | 0.06397607458 | 0.5664778647 | 0.5710689762 | 1.036905742 |
| ace2 | -0.03884834772 | 0.06862906642 | -0.5660625992 | 0.5713512269 | 0.9618965719 |
| snx15 | 0.03595506817 | 0.06367227752 | 0.5646895252 | 0.5722849601 | 1.036609269 |
| il3ra | -0.03401835752 | 0.06033628961 | -0.5638125536 | 0.5728817074 | 0.9665537609 |
| s100a3 | 0.03597012532 | 0.06382316484 | 0.5635904363 | 0.573032897 | 1.036624877 |
| tmem25 | -0.03579497006 | 0.06353460664 | -0.5633932741 | 0.5731671161 | 0.9648380939 |

| Protein | beta | se | z | p_val | HR |
|---|---|---|---|---|---|
| tor1aip1 | 0.03631956452 | 0.06453677474 | 0.5627731579 | 0.5735893605 | 1.036987178 |
| stc1 | -0.03466833113 | 0.06161968686 | -0.5626177751 | 0.5736951855 | 0.9659257306 |
| mtus1 | -0.03707279976 | 0.06591368942 | -0.5624446165 | 0.5738131279 | 0.9636059825 |
| xpnpep2 | 0.03319381542 | 0.05908169079 | 0.5618291383 | 0.5742324373 | 1.033750877 |
| app | 0.0328991095 | 0.05857990385 | 0.5616108484 | 0.5743811875 | 1.033446269 |
| upb1 | 0.03918431241 | 0.06984948232 | 0.5609821449 | 0.5748097092 | 1.039962144 |
| pomc | 0.03750734769 | 0.06689259623 | 0.5607100008 | 0.5749952484 | 1.038219626 |
| c1s | 0.03618758482 | 0.06454195494 | 0.5606831223 | 0.5750135749 | 1.036850326 |
| ttf2 | -0.03583338171 | 0.06393527607 | -0.5604633923 | 0.5751634027 | 0.9648010336 |
| fhip2a | 0.03589897091 | 0.06410393032 | 0.5600120106 | 0.5754712454 | 1.036551119 |
| slamf8 | 0.03270044779 | 0.05840950302 | 0.5598480744 | 0.5755830693 | 1.033240983 |
| ptprh | -0.03550935002 | 0.06359611998 | -0.558357177 | 0.5766005085 | 0.9651137104 |
| itga2 | 0.03606584363 | 0.06464440569 | 0.5579112878 | 0.5769049632 | 1.036724106 |
| gtpbp2 | 0.03551401977 | 0.06368481199 | 0.557652895 | 0.5770814293 | 1.036152175 |
| asah1 | 0.0361908687 | 0.06491732568 | 0.557491676 | 0.5771915448 | 1.036853731 |
| myom1 | 0.03553454854 | 0.06379148549 | 0.5570421861 | 0.5774986065 | 1.036173446 |
| flt4 | 0.03359931097 | 0.06038329121 | 0.5564339124 | 0.5779142612 | 1.034170143 |
| nenf | -0.03517666608 | 0.06322240215 | -0.5563955952 | 0.5779404494 | 0.9654348416 |
| dlg4 | 0.03476093466 | 0.06254044791 | 0.5558152495 | 0.5783371596 | 1.035372158 |
| lcp1 | 0.03526558292 | 0.0634620132 | 0.5556959375 | 0.5784187343 | 1.035894788 |
| st6gal1 | -0.03380803111 | 0.06084168895 | -0.5556721336 | 0.5784350098 | 0.9667570741 |
| vnn2 | 0.03287261107 | 0.05915903128 | 0.5556651344 | 0.5784397955 | 1.033418885 |
| gpha2 | 0.03643167268 | 0.06565493601 | 0.5548961723 | 0.5789656802 | 1.037103439 |
| aamdc | 0.03559043079 | 0.06418259976 | 0.554518373 | 0.5792241352 | 1.036231351 |
| epgn | -0.03494749681 | 0.06303943968 | -0.554375118 | 0.5793221511 | 0.9656561149 |
| ndst1 | 0.03526991629 | 0.06362156478 | 0.5543704625 | 0.5793253365 | 1.035899277 |
| apod | 0.03756391474 | 0.06787807149 | 0.5534027989 | 0.5799876228 | 1.038278356 |
| zp4 | -0.03520185105 | 0.06362991637 | -0.553227995 | 0.5801072995 | 0.9654105275 |
| gamt | -0.03535501656 | 0.06403287267 | -0.5521385357 | 0.5808534416 | 0.9652626712 |
| lmod2 | -0.03494786108 | 0.06347166474 | -0.5506057108 | 0.5819039932 | 0.9656557632 |
| top2b | 0.03622436124 | 0.06586274964 | 0.5499977065 | 0.5823209467 | 1.036888458 |
| trim24 | 0.03533847993 | 0.06425880161 | 0.5499399155 | 0.5823605854 | 1.035970305 |
| mertk | -0.03370878215 | 0.06131442525 | -0.5497691939 | 0.5824776907 | 0.9668530285 |
| osbpl2 | 0.03489261569 | 0.06354604983 | 0.5490918128 | 0.5829424436 | 1.035508505 |
| sat2 | -0.03562250419 | 0.06491358698 | -0.5487680753 | 0.5831646217 | 0.9650045099 |
| tpmt | -0.03203955375 | 0.05838511058 | -0.5487624058 | 0.583168513 | 0.9684682748 |
| npl | 0.0395422782 | 0.07212203834 | 0.5482690051 | 0.5835072065 | 1.040334481 |
| smpd1 | -0.03246187718 | 0.05928753371 | -0.5475329323 | 0.5840126518 | 0.9680593543 |
| gkn1 | -0.03205839032 | 0.05858793716 | -0.5471841453 | 0.5842522275 | 0.9684500323 |
| lamb1 | -0.03512866447 | 0.06422136828 | -0.5469933981 | 0.5843832677 | 0.9654811851 |
| dnaja2 | 0.03187302534 | 0.05833452366 | 0.5463835707 | 0.5848023011 | 1.03238641 |
| carhsp1 | 0.03232069976 | 0.05917288744 | 0.54620792 | 0.5849230226 | 1.032848687 |
| bmp4 | 0.0326740636 | 0.05995186345 | 0.545004971 | 0.5857500987 | 1.033213722 |
| lto1 | 0.03195921101 | 0.05875617714 | 0.5439293802 | 0.5864900703 | 1.032475391 |
| acp6 | -0.03126068692 | 0.05775145135 | -0.5412969923 | 0.5883028933 | 0.9692228764 |
| ddx58 | 0.0319889192 | 0.05928766455 | 0.5395543819 | 0.5895043837 | 1.032506064 |
| eno2 | 0.03139843492 | 0.05824210617 | 0.5391019828 | 0.5898164875 | 1.031896566 |
| mucl3 | 0.03455678187 | 0.06411160211 | 0.5390098006 | 0.5898800921 | 1.035160805 |
| dctn2 | 0.03126593164 | 0.0580307698 | 0.5387819556 | 0.5900373158 | 1.031759845 |
| cnpy4 | 0.03152055285 | 0.0585140692 | 0.5386833163 | 0.5901053875 | 1.032022586 |
| insl4 | -0.03498289315 | 0.06495470958 | -0.5385736212 | 0.5901810932 | 0.9656219349 |
| megf9 | -0.03246782103 | 0.06043734256 | -0.5372145705 | 0.5911194078 | 0.9680536003 |
| sugt1 | 0.03147771508 | 0.05862438267 | 0.5369389603 | 0.591309778 | 1.031978378 |
| micall2 | 0.03427957042 | 0.06385551433 | 0.5368302297 | 0.5913848884 | 1.034873886 |
| drg2 | 0.03128970781 | 0.05831094096 | 0.5366009757 | 0.59154327 | 1.031784377 |
| ccdc134 | 0.03412511178 | 0.06361360134 | 0.5364436388 | 0.5916519785 | 1.034714054 |
| ment | 0.03611732605 | 0.06738269156 | 0.5360030182 | 0.5919564645 | 1.03677748 |
| syngap1 | -0.03383753236 | 0.06323618837 | -0.5350975957 | 0.5925823724 | 0.966728554 |
| rp2 | 0.03144446223 | 0.05884907288 | 0.5343238337 | 0.5931175053 | 1.031944062 |
| reg1b | 0.03211638747 | 0.06019887947 | 0.5335047388 | 0.5936842316 | 1.032637684 |
| mmp13 | -0.03122697653 | 0.0585566349 | -0.5332781944 | 0.5938410198 | 0.9692555498 |
| itgb1bp1 | 0.03084908963 | 0.05801680141 | 0.5317268253 | 0.5949152099 | 1.031329854 |
| thrap3 | 0.03405312254 | 0.06421459232 | 0.5303019347 | 0.5959026055 | 1.034639568 |
| aif1l | -0.03570811001 | 0.06733618464 | -0.5302960095 | 0.595906713 | 0.9649219034 |
| cst7 | 0.03157791199 | 0.05955288425 | 0.530249918 | 0.5959386653 | 1.032081784 |
| crim1 | -0.0323959106 | 0.06114022402 | -0.529862478 | 0.5962072832 | 0.9681232159 |
| hs3st3b1 | 0.03118353713 | 0.0589229095 | 0.5292260242 | 0.5966486657 | 1.031674837 |
| spock1 | -0.03144179676 | 0.05945789932 | -0.5288077299 | 0.596938835 | 0.9690473565 |
| cd84 | 0.03062397115 | 0.05801316688 | 0.527879666 | 0.5975828588 | 1.031097708 |
| pxdnl | 0.03347562001 | 0.06346677715 | 0.5274510778 | 0.5978803812 | 1.034042233 |

| Protein | beta | se | z | p_val | HR |
|---|---|---|---|---|---|
| muc13 | -0.0309570394 | 0.0586958216 | -0.5274147044 | 0.5979056345 | 0.9695172232 |
| cetn3 | 0.03356108479 | 0.06366485268 | 0.527152477 | 0.5980877073 | 1.034130611 |
| alpp | -0.03305790396 | 0.06272597412 | -0.5270209738 | 0.5981790236 | 0.9674825369 |
| setmar | 0.03380228386 | 0.0642289362 | 0.5262781211 | 0.5986949822 | 1.034380073 |
| irag2 | 0.03077129353 | 0.05847653062 | 0.5262161282 | 0.5987380494 | 1.031249623 |
| naaa | 0.03061134688 | 0.05820394166 | 0.5259325401 | 0.598935079 | 1.031084692 |
| ebi3_il27 | -0.0317420105 | 0.06046232649 | -0.5249882421 | 0.5995913646 | 0.9687564788 |
| sdc4 | 0.03073579567 | 0.05854917466 | 0.5249569418 | 0.5996131239 | 1.031213017 |
| amn | 0.03123719004 | 0.05953839529 | 0.5246562304 | 0.5998221896 | 1.031730191 |
| pamr1 | 0.0332485498 | 0.06337845129 | 0.5246033805 | 0.5998589362 | 1.03380746 |
| gip | -0.03442594255 | 0.06565599509 | -0.5243381431 | 0.6000433716 | 0.9661598884 |
| gimap7 | -0.03305587364 | 0.06307997213 | -0.5240312024 | 0.6002568378 | 0.9674845012 |
| efnb2 | -0.03364532154 | 0.06424195059 | -0.5237282062 | 0.6004675944 | 0.9669143875 |
| il11 | 0.03103012097 | 0.05924930117 | 0.5237212989 | 0.6004723994 | 1.031516574 |
| serpini1 | 0.0339751451 | 0.06487611374 | 0.5236926681 | 0.600492316 | 1.034558893 |
| inhbc | -0.03383715604 | 0.06461699197 | -0.5236572457 | 0.6005169576 | 0.9667289178 |
| git1 | 0.03357234267 | 0.06413132643 | 0.5234936581 | 0.6006307632 | 1.034142254 |
| art5 | 0.03315640472 | 0.0633878611 | 0.5230718334 | 0.6009242657 | 1.033712204 |
| spart | 0.03307960747 | 0.06324293732 | 0.5230561526 | 0.6009351775 | 1.033632821 |
| dcxr | 0.03398289315 | 0.06501466633 | 0.5226958018 | 0.6011859602 | 1.034566908 |
| lilrb2 | 0.03049128765 | 0.05840831511 | 0.5220367613 | 0.6016447354 | 1.030960908 |
| ppy | -0.03127659398 | 0.05994077815 | -0.5217915908 | 0.6018154453 | 0.969207459 |
| furin | -0.03486228497 | 0.0668826096 | -0.5212458841 | 0.602195494 | 0.9657384038 |
| scpep1 | 0.03467162065 | 0.0665499666 | 0.5209862968 | 0.6023763174 | 1.035279689 |
| pon2 | 0.03098023847 | 0.05946886062 | 0.5209489159 | 0.6024023582 | 1.03146512 |
| glo1 | 0.03115339213 | 0.05981833509 | 0.5208000538 | 0.6025060656 | 1.031643738 |
| ifit3 | -0.03305879159 | 0.0635019557 | -0.5205948577 | 0.6026490323 | 0.9674816781 |
| mfap3l | 0.0327469775 | 0.06292484453 | 0.5204141185 | 0.6027749718 | 1.033289061 |
| map2k1 | 0.03317068628 | 0.06375996465 | 0.5202431724 | 0.6028940983 | 1.033726967 |
| casc3 | 0.03332625023 | 0.06409476788 | 0.5199527407 | 0.6030965145 | 1.03388779 |
| reps1 | -0.0332833941 | 0.06401484033 | -0.5199324708 | 0.6031106428 | 0.9672644037 |
| bcl2l15 | 0.03426270515 | 0.06592491829 | 0.5197231342 | 0.6032565603 | 1.034856433 |
| smoc1 | 0.03262508638 | 0.06278523916 | 0.5196298814 | 0.603321567 | 1.03316312 |
| bnip2 | 0.03311913419 | 0.06380702434 | 0.5190515391 | 0.6037248011 | 1.033673678 |
| dcun1d1 | 0.03274094323 | 0.06333382669 | 0.5169582344 | 0.6051853144 | 1.033282826 |
| rbm19 | -0.03308062495 | 0.0641086006 | -0.516009157 | 0.6058480137 | 0.967460555 |
| glrx5 | 0.03255161894 | 0.06320127425 | 0.5150468772 | 0.6065202631 | 1.033087219 |
| lif | 0.03004543386 | 0.05837167704 | 0.5147262403 | 0.6067443343 | 1.030501353 |
| icam5 | 0.03067296998 | 0.05983130959 | 0.5126575064 | 0.6081909192 | 1.031148232 |
| spink8 | 0.03253210762 | 0.06346258864 | 0.5126186674 | 0.6082180926 | 1.033067062 |
| hras | 0.03220297736 | 0.06283068432 | 0.5125358368 | 0.6082760459 | 1.032727104 |
| pappa | 0.03154067796 | 0.06159942938 | 0.512028736 | 0.6086308983 | 1.032043356 |
| arntl | 0.03305067898 | 0.06456320907 | 0.5119119612 | 0.6087126265 | 1.03360292 |
| gadd45b | 0.03245321099 | 0.06347357461 | 0.5112869598 | 0.6091501348 | 1.03298556 |
| snx9 | 0.03015101358 | 0.05899639535 | 0.5110653523 | 0.6093052961 | 1.030610158 |
| phykpl | -0.03230582626 | 0.06325109289 | -0.5107552262 | 0.6095224645 | 0.9682104326 |
| otoa | -0.0320758827 | 0.06294997734 | -0.5095455798 | 0.6103698581 | 0.968433092 |
| fabp2 | 0.02985252623 | 0.05861223938 | 0.5093223966 | 0.6105262617 | 1.03030258 |
| creld2 | -0.03029552335 | 0.05957912688 | -0.5084922343 | 0.6111081838 | 0.9701587866 |
| krt8 | -0.032242804 | 0.06343023544 | -0.50831916 | 0.6112295353 | 0.9682714534 |
| dhrs4l2 | 0.03223921117 | 0.06343281113 | 0.5082418798 | 0.611283724 | 1.032764525 |
| nbl1 | -0.03060821018 | 0.06023538944 | -0.5081433101 | 0.6113528439 | 0.9698554782 |
| il16 | -0.03114409684 | 0.06129476236 | -0.5081037211 | 0.6113806058 | 0.9693358848 |
| cntn2 | 0.03078414684 | 0.06060000994 | 0.5079891384 | 0.6114609604 | 1.031262879 |
| kif22 | 0.03211718062 | 0.06324286723 | 0.5078387813 | 0.6115664101 | 1.032638503 |
| calb2 | -0.03210472616 | 0.06333088817 | -0.5069363006 | 0.6121995142 | 0.9684051594 |
| coro6 | -0.03251426864 | 0.0641777328 | -0.5066285021 | 0.6124155058 | 0.9680086376 |
| il7 | -0.0296992108 | 0.05862652899 | -0.5065831341 | 0.6124473448 | 0.970737477 |
| crebzf | 0.03242129748 | 0.06400965289 | 0.5065063786 | 0.612501213 | 1.032952594 |
| cdh6 | 0.0300505792 | 0.05936636241 | 0.5061886559 | 0.6127242179 | 1.030506655 |
| klrk1 | 0.03141396396 | 0.0620986606 | 0.5058718442 | 0.6129466191 | 1.03191259 |
| lztfl1 | 0.03202541507 | 0.06331073175 | 0.5058449679 | 0.6129654879 | 1.032543747 |
| gdf15 | 0.03811870806 | 0.07538920234 | 0.5056255654 | 0.613119531 | 1.038854546 |
| tpd52l2 | 0.0317419204 | 0.06283531048 | 0.5051605564 | 0.6134460716 | 1.032251068 |
| vsir | -0.02972157348 | 0.05886984532 | -0.50486923 | 0.6136506871 | 0.9707157689 |
| ache | 0.03317800662 | 0.06572105696 | 0.5048306914 | 0.6136777573 | 1.033734534 |
| bax | 0.02959156926 | 0.05873201761 | 0.5038405024 | 0.6143734634 | 1.030033751 |
| calcoco2 | 0.03236531709 | 0.06428375405 | 0.5034758403 | 0.6146297621 | 1.032894771 |
| ltb | 0.03237300896 | 0.06431350511 | 0.5033625349 | 0.6147094072 | 1.032902715 |
| prr4 | 0.03183592372 | 0.06330917164 | 0.5028643227 | 0.6150596663 | 1.032348108 |

| Protein | beta | se | z | p_val | HR |
|---|---|---|---|---|---|
| lipf | -0.03245930635 | 0.06457259557 | -0.5026792877 | 0.6151897742 | 0.968061843 |
| cd109 | 0.02934769965 | 0.05851670007 | 0.501526908 | 0.6160003455 | 1.029782587 |
| defb104a_defb104b | -0.03398173181 | 0.06775707027 | -0.5015230391 | 0.6160030675 | 0.9665891623 |
| sumf2 | -0.0294193038 | 0.05873637798 | -0.5008702411 | 0.6164624474 | 0.9710092312 |
| tmed8 | 0.03154338501 | 0.06297968196 | 0.5008501793 | 0.6164765674 | 1.03204615 |
| grik2 | 0.03121222407 | 0.06255395521 | 0.4989648372 | 0.6178041559 | 1.031704433 |
| jchain | 0.02908462468 | 0.05831338123 | 0.4987641612 | 0.6179455381 | 1.029511713 |
| cep170 | 0.03160290195 | 0.06336983633 | 0.4987057531 | 0.617986691 | 1.032107576 |
| pglyrp4 | 0.03199582433 | 0.06432411801 | 0.4974156711 | 0.6188959568 | 1.032513194 |
| tpbgl | -0.03149296933 | 0.06334081729 | -0.4971986577 | 0.6190489679 | 0.9689977691 |
| myl3 | -0.03520461819 | 0.07088183274 | -0.4966663083 | 0.6194243851 | 0.965407856 |
| hbz | -0.03120626611 | 0.06304278477 | -0.4950013903 | 0.6205991392 | 0.9692756237 |
| immt | -0.03326192533 | 0.06724418016 | -0.4946439267 | 0.6208514892 | 0.9672851699 |
| f3 | 0.03036652161 | 0.06141226102 | 0.4944700147 | 0.6209742779 | 1.030832287 |
| lrrc37a2 | 0.03077429752 | 0.06227534766 | 0.4941650054 | 0.6211896519 | 1.031252721 |
| ak2 | 0.03106265148 | 0.06286606507 | 0.4941084104 | 0.6212296184 | 1.03155013 |
| ythdf3 | -0.02925352402 | 0.05922366785 | -0.4939498867 | 0.6213415716 | 0.9711702183 |
| enpp6 | 0.0342643879 | 0.06947086665 | 0.4932195257 | 0.6218574828 | 1.034858175 |
| casp10 | -0.02888941134 | 0.05857681286 | -0.4931885149 | 0.6218793923 | 0.971523898 |
| pnpt1 | 0.02892388877 | 0.05865353897 | 0.493131178 | 0.6219199023 | 1.029346247 |
| plcb2 | 0.0311169662 | 0.06321612357 | 0.4922314822 | 0.6225557111 | 1.03160616 |
| ryr1 | 0.03097110691 | 0.06297546997 | 0.4917963601 | 0.6228633099 | 1.031455702 |
| tnfrsf19 | 0.02987910617 | 0.06083164492 | 0.4911770215 | 0.6233012496 | 1.030329966 |
| ahnak2 | -0.0322594748 | 0.06571809081 | -0.4908766278 | 0.6235137086 | 0.9682553116 |
| itih5 | -0.03089422145 | 0.06294613082 | -0.4908041376 | 0.6235649833 | 0.9695781282 |
| cntnap2 | -0.02930939626 | 0.05976145659 | -0.4904397907 | 0.6238227256 | 0.9711159583 |
| nrtn | 0.02889407749 | 0.05905646971 | 0.4892618476 | 0.6246563281 | 1.029315561 |
| ciapin1 | 0.02874245967 | 0.05879028518 | 0.4888981161 | 0.6249138295 | 1.02915951 |
| ccer2 | 0.03140486399 | 0.0643010095 | 0.4884039028 | 0.625263778 | 1.0319032 |
| mvk | 0.02989490931 | 0.06124281648 | 0.4881374016 | 0.6254525204 | 1.030346248 |
| naa80 | 0.03122852892 | 0.06400263919 | 0.4879256435 | 0.62560251 | 1.031721255 |
| hspa1a | 0.02901918775 | 0.05949483865 | 0.487759752 | 0.6257200228 | 1.029444347 |
| tg | -0.03082876909 | 0.06322442244 | -0.4876085522 | 0.6258271367 | 0.9696415915 |
| cadps | 0.03107848768 | 0.0637695689 | 0.4873560887 | 0.6260060061 | 1.031566466 |
| cngb3 | -0.03106596129 | 0.06375349146 | -0.4872825091 | 0.6260581411 | 0.9694116273 |
| twf2 | 0.03059924778 | 0.06282218665 | 0.4870770886 | 0.6262037022 | 1.031072217 |
| cd40lg | -0.02802876898 | 0.05773714025 | -0.4854547499 | 0.6273538035 | 0.9723603926 |
| vti1a | 0.0308759605 | 0.0636500104 | 0.4850896379 | 0.6276127621 | 1.031357567 |
| washc3 | 0.03097260922 | 0.06393370856 | 0.484448813 | 0.6280673832 | 1.031457251 |
| c7 | 0.03140395444 | 0.06488734534 | 0.4839765639 | 0.6284025017 | 1.031902261 |
| il7r | 0.0287454797 | 0.05948796041 | 0.4832150825 | 0.6289430273 | 1.029162618 |
| sult2a1 | 0.0296706902 | 0.06153109287 | 0.4822064557 | 0.6296592913 | 1.030115251 |
| pdia5 | 0.03087273584 | 0.06409429563 | 0.4816768097 | 0.6300355525 | 1.031354241 |
| dll4 | -0.03107004685 | 0.0645460167 | -0.4813627306 | 0.6302587201 | 0.9694076667 |
| yars1 | 0.0304556029 | 0.06334439528 | 0.480793964 | 0.6306629406 | 1.030924119 |
| cuzd1 | 0.03043210796 | 0.06333440078 | 0.4804988692 | 0.6308727071 | 1.030899898 |
| lsm8 | -0.03018940743 | 0.06283040653 | -0.4804904043 | 0.6308787247 | 0.9702617414 |
| kifbp | 0.02792540539 | 0.05816898122 | 0.4800738263 | 0.6311748987 | 1.028318975 |
| selenop | -0.030212104 | 0.0629453715 | -0.4799734005 | 0.6312463071 | 0.97023972 |
| mars1 | 0.03029614235 | 0.06315620369 | 0.4797017646 | 0.631439473 | 1.03075974 |
| sod2 | 0.02832035841 | 0.0590718134 | 0.479422533 | 0.6316380665 | 1.028725192 |
| pydc1 | 0.03092072952 | 0.06459640281 | 0.4786757184 | 0.6321693425 | 1.031403741 |
| sh2b3 | 0.02804383903 | 0.05859155455 | 0.4786327866 | 0.6321998895 | 1.028440769 |
| mpi | 0.0282745863 | 0.05911081381 | 0.4783318732 | 0.6324140141 | 1.028678107 |
| brsk2 | -0.02999439758 | 0.06271877704 | -0.4782363272 | 0.6324820095 | 0.9704509704 |
| tnr | 0.02922294808 | 0.06114113973 | 0.4779588378 | 0.6326795024 | 1.029654128 |
| nfkb1 | -0.03086831025 | 0.06470460573 | -0.4770651162 | 0.633315754 | 0.9696032515 |
| tfap2a | -0.03017859458 | 0.06334331319 | -0.4764290508 | 0.6337687422 | 0.9702722327 |
| cd79b | -0.02803058267 | 0.05884601217 | -0.4763378457 | 0.6338337072 | 0.972358629 |
| cdh2 | 0.03148842588 | 0.06611449418 | 0.4762711455 | 0.6338812193 | 1.031989431 |
| meltf | 0.03058713091 | 0.06430014568 | 0.4756930266 | 0.6342930897 | 1.031059723 |
| inhbb | 0.03131219337 | 0.06591150664 | 0.4750641423 | 0.6347412556 | 1.031807577 |
| glb1 | -0.02961279874 | 0.06233834289 | -0.4750334604 | 0.634763124 | 0.970821364 |
| arfip1 | 0.029944652 | 0.06312875953 | 0.4743424744 | 0.6352557075 | 1.030397502 |
| ppib | -0.02760571083 | 0.05821042228 | -0.4742400029 | 0.6353287702 | 0.9727718446 |
| lep | 0.04970046802 | 0.1048773034 | 0.4738915515 | 0.6355772442 | 1.050956254 |
| prkra | -0.02824065104 | 0.05966627422 | -0.4733101137 | 0.6359919476 | 0.9721543887 |
| art3 | -0.03013744477 | 0.0637788688 | -0.4725302492 | 0.6365483558 | 0.9703121601 |
| lilra6 | 0.02959385671 | 0.06263455881 | 0.4724844762 | 0.6365810197 | 1.030036107 |
| sdc1 | 0.02863542727 | 0.06060793255 | 0.4724699567 | 0.6365913811 | 1.029049363 |

| Protein | beta | se | z | p_val | HR |
|---|---|---|---|---|---|
| eif4b | 0.02784097279 | 0.05893139903 | 0.4724302027 | 0.6366197506 | 1.028232155 |
| vamp5 | -0.02838277766 | 0.06010820307 | -0.4721947457 | 0.6367877898 | 0.9720162295 |
| triap1 | 0.02775986952 | 0.0588060363 | 0.4720581639 | 0.636885273 | 1.028148765 |
| psmd1 | -0.03023246122 | 0.06407631017 | -0.4718196342 | 0.6370555352 | 0.9702199688 |
| lcn2 | 0.02862266485 | 0.06076541771 | 0.4710354332 | 0.6376154318 | 1.02903623 |
| cntn5 | -0.02921723498 | 0.06210568861 | -0.4704437812 | 0.6380379909 | 0.9712054617 |
| gcc1 | 0.0295865819 | 0.06289889126 | 0.4703832024 | 0.638081263 | 1.030028613 |
| masp1 | 0.02800418145 | 0.05955106491 | 0.4702549232 | 0.6381728984 | 1.028399985 |
| itih4 | 0.0311710502 | 0.06641234968 | 0.469356232 | 0.6388150276 | 1.031661955 |
| ighmbp2 | 0.02993643532 | 0.06380170706 | 0.4692105698 | 0.6389191311 | 1.030389036 |
| serpinh1 | 0.02974372896 | 0.06340989779 | 0.4690707602 | 0.6390190585 | 1.030190492 |
| chi3l1 | -0.02943013684 | 0.06278115211 | -0.4687734431 | 0.6392315844 | 0.9709987123 |
| dusp13 | 0.0299235518 | 0.06383558738 | 0.4687597159 | 0.6392413975 | 1.030375761 |
| cacnb3 | 0.0296247384 | 0.0632673389 | 0.4682469488 | 0.6396080016 | 1.030067916 |
| ptprm | 0.02769807241 | 0.0592907963 | 0.4671563572 | 0.6403880155 | 1.02808523 |
| apoe | -0.02952036094 | 0.06321672871 | -0.4669707139 | 0.6405208311 | 0.9709111088 |
| wasf3 | 0.02695239546 | 0.05775254865 | 0.4666875505 | 0.6407234381 | 1.027318897 |
| tjp3 | 0.02957940104 | 0.06342936678 | 0.4663360607 | 0.6409749707 | 1.030021217 |
| neo1 | -0.02966342616 | 0.06364041364 | -0.4661098894 | 0.6411368449 | 0.9707722151 |
| satb1 | -0.02933628048 | 0.06296809609 | -0.4658911783 | 0.641293396 | 0.971089851 |
| lrig1 | 0.02718588312 | 0.05838839434 | 0.4656042254 | 0.6414988179 | 1.027558791 |
| tnfrsf8 | -0.0273113538 | 0.05866173253 | -0.4655735967 | 0.6415207458 | 0.973058229 |
| kirrel1 | 0.02944295308 | 0.0632700533 | 0.4653536949 | 0.6416781885 | 1.029880682 |
| cacybp | 0.02934328897 | 0.06307106688 | 0.4652416777 | 0.6417583955 | 1.029778045 |
| slamf6 | -0.02756798216 | 0.05926952768 | -0.4651291016 | 0.6418390068 | 0.9728085467 |
| scrib | 0.03011051783 | 0.06479194615 | 0.4647262449 | 0.6421275113 | 1.030568424 |
| igsf8 | -0.02800480619 | 0.06035696687 | -0.4639863075 | 0.6426575557 | 0.9723836933 |
| vwa5a | -0.02974064218 | 0.06411464725 | -0.4638665805 | 0.6427433377 | 0.9706972588 |
| slc27a4 | -0.02699561184 | 0.05840626646 | -0.4622040319 | 0.6439350122 | 0.9733655128 |
| stx1b | 0.02946543844 | 0.06376917811 | 0.4620639518 | 0.6440354601 | 1.02990384 |
| slc13a1 | -0.02907377529 | 0.06296352494 | -0.4617558391 | 0.6442564228 | 0.9713448006 |
| rln1 | -0.02996347799 | 0.06495015781 | -0.4613303339 | 0.6445616251 | 0.9704809768 |
| med21 | -0.02941715731 | 0.06393820349 | -0.4600873297 | 0.6454535382 | 0.9710113155 |
| pcna | 0.02945070395 | 0.06402633638 | 0.4599779655 | 0.6455320365 | 1.029888665 |
| ifit1 | 0.02902981798 | 0.06313425805 | 0.4598108677 | 0.6456519819 | 1.02945529 |
| il10rb | -0.02823658291 | 0.06142508752 | -0.4596913746 | 0.6457377615 | 0.9721583435 |
| chchd6 | 0.03053354474 | 0.06645241031 | 0.4594798683 | 0.6458896056 | 1.031004474 |
| atp6v1g1 | -0.02962789376 | 0.06448500698 | -0.4594539901 | 0.645908185 | 0.9708067096 |
| insr | -0.02954826902 | 0.0643240111 | -0.4593660829 | 0.6459713002 | 0.9708840129 |
| cep152 | 0.02900735967 | 0.06321784685 | 0.4588476375 | 0.646343583 | 1.029432171 |
| siglec7 | 0.02835123567 | 0.06180204161 | 0.4587427038 | 0.6464189441 | 1.028756957 |
| edar | 0.02679251319 | 0.05844987425 | 0.4583844454 | 0.6466762647 | 1.02715466 |
| tmsb10 | 0.02778594242 | 0.06076268312 | 0.4572862981 | 0.6474652769 | 1.028175572 |
| il1rn | -0.03144541655 | 0.06887912311 | -0.4565304425 | 0.6480085849 | 0.9690438488 |
| irak4 | 0.02634029314 | 0.05775439405 | 0.4560742705 | 0.6483365714 | 1.026690265 |
| cd99l2 | -0.02784256051 | 0.06106260268 | -0.4559674708 | 0.64841337 | 0.9725414712 |
| mmp9 | 0.02757724036 | 0.06063371555 | 0.4548169299 | 0.6492409494 | 1.027961012 |
| dlgap5 | -0.02854424645 | 0.06281660279 | -0.4544060835 | 0.6495365746 | 0.9718592919 |
| rpe | 0.02908271375 | 0.06413113202 | 0.4534882332 | 0.6501972145 | 1.029509746 |
| rad23b | 0.02672618829 | 0.05898261293 | 0.4531197749 | 0.6504624966 | 1.027086536 |
| mylpf | 0.03010413479 | 0.06645756231 | 0.4529828321 | 0.6505611038 | 1.030561846 |
| jmjd1c | 0.02890883822 | 0.06387955492 | 0.4525522799 | 0.6508711676 | 1.029330755 |
| b4galt1 | 0.02724115949 | 0.06021562809 | 0.4523935123 | 0.6509855199 | 1.027615592 |
| tnfrsf1a | 0.02926836098 | 0.06485652609 | 0.4512785798 | 0.651788781 | 1.029700889 |
| csf3 | -0.02714752064 | 0.0602085508 | -0.4508914477 | 0.6520677877 | 0.9732176612 |
| rnf149 | 0.02956068527 | 0.06577617602 | 0.4494132535 | 0.6531335723 | 1.03000194 |
| plekho1 | 0.02828703205 | 0.0629473547 | 0.4493760251 | 0.6531604232 | 1.028690909 |
| gli2 | 0.02890732935 | 0.06433342193 | 0.4493361068 | 0.6531892149 | 1.029329201 |
| gnly | -0.02622981227 | 0.0583954536 | -0.449175589 | 0.6533049955 | 0.9741112012 |
| impg1 | -0.02847251739 | 0.06341856671 | -0.4489618556 | 0.6534591734 | 0.9719290049 |
| atp5if1 | 0.02588585057 | 0.05772451582 | 0.4484377253 | 0.6538373205 | 1.026223799 |
| vwc2l | -0.03038705308 | 0.06789467269 | -0.4475616698 | 0.6544695714 | 0.9700699923 |
| lsm1 | -0.02591400942 | 0.05794921583 | -0.447184816 | 0.6547416236 | 0.9744188768 |
| hsbp1 | 0.02854623211 | 0.06391114722 | 0.4466549788 | 0.6551241929 | 1.028957581 |
| clpp | -0.02621126628 | 0.05869245242 | -0.4465866597 | 0.6551735294 | 0.9741292672 |
| mcfd2 | -0.02643280784 | 0.05935734304 | -0.4453165604 | 0.6560910018 | 0.973913481 |
| fcer2 | -0.02624838067 | 0.05896865722 | -0.4451242729 | 0.6562299484 | 0.9740931137 |
| omd | 0.02737719405 | 0.06150626379 | 0.4451122919 | 0.6562386062 | 1.027755393 |
| lpo | 0.02663984838 | 0.05985279113 | 0.4450894917 | 0.6562550825 | 1.026997861 |
| clasp1 | 0.02803714146 | 0.06300753408 | 0.4449807768 | 0.6563336462 | 1.028433881 |

| Protein | beta | se | z | p_val | HR |
|---|---|---|---|---|---|
| selp | -0.026303545 | 0.05912637418 | -0.4448699141 | 0.6564137659 | 0.9740393799 |
| prok1 | 0.03596166715 | 0.08096535471 | 0.4441611758 | 0.6569260595 | 1.036616109 |
| birc2 | 0.02610954912 | 0.05878423058 | 0.4441590689 | 0.6569275827 | 1.026453389 |
| dnlz | 0.02801807305 | 0.06309550876 | 0.4440581209 | 0.6570005636 | 1.028414271 |
| ccdc28a | 0.02816010918 | 0.06348351829 | 0.4435814199 | 0.6573452414 | 1.028560353 |
| cxcl13 | -0.02643058166 | 0.05962997603 | -0.4432432045 | 0.6575898317 | 0.9739156491 |
| nfkb2 | -0.02862094681 | 0.06472197579 | -0.4422137375 | 0.6583345461 | 0.9717847528 |
| draxin | -0.02626161342 | 0.05939063807 | -0.442184396 | 0.6583557767 | 0.9740802238 |
| wasf1 | -0.02595798418 | 0.05872049061 | -0.4420600699 | 0.6584457381 | 0.974376028 |
| clta | -0.02646427847 | 0.05989718732 | -0.4418284006 | 0.6586133853 | 0.9738828318 |
| col4a4 | 0.02776179101 | 0.06284394716 | 0.4417575959 | 0.6586646265 | 1.02815074 |
| spring1 | 0.02875116568 | 0.06508707651 | 0.441733862 | 0.658681803 | 1.02916847 |
| cd36 | 0.02933578716 | 0.06642227781 | 0.4416558439 | 0.658738267 | 1.02977032 |
| stat5b | 0.02571395209 | 0.05824327152 | 0.4414922346 | 0.6588566821 | 1.026047408 |
| crtac1 | -0.02649019115 | 0.06002155666 | -0.4413446206 | 0.6589635276 | 0.9738575962 |
| hgf | 0.02948664858 | 0.06682055772 | 0.4412810906 | 0.6590095139 | 1.029925684 |
| il6st | 0.02632641856 | 0.05974477018 | 0.4406480849 | 0.659467786 | 1.02667602 |
| apol1 | 0.028478102 | 0.06466461281 | 0.4403970078 | 0.6596495917 | 1.02888748 |
| inpp5j | 0.0281085686 | 0.06397062013 | 0.4393980946 | 0.6603731069 | 1.028507342 |
| tmed1 | 0.02772837177 | 0.06310564083 | 0.4393960889 | 0.66037456 | 1.028116381 |
| cradd | 0.02592116499 | 0.05902375341 | 0.4391649717 | 0.6605420041 | 1.02626004 |
| rrm2 | 0.02599430544 | 0.05921110856 | 0.4390106194 | 0.6606538417 | 1.026335104 |
| tnip1 | 0.02750970867 | 0.06275266757 | 0.4383830956 | 0.6611085984 | 1.027891595 |
| mthfd2 | 0.02748791419 | 0.06281639253 | 0.4375914165 | 0.6616824945 | 1.027869192 |
| tfpi | -0.0268273409 | 0.06131136646 | -0.4375590114 | 0.6617059896 | 0.9735293157 |
| vstm2b | -0.02792863506 | 0.06385488378 | -0.4373766485 | 0.6618382163 | 0.9724577637 |
| psrc1 | -0.0255708111 | 0.05846619147 | -0.437360643 | 0.661849822 | 0.9747533532 |
| med18 | 0.02556365729 | 0.05850991723 | 0.4369115272 | 0.6621755118 | 1.02589321 |
| ccl27 | -0.02711718422 | 0.0621742542 | -0.4361481223 | 0.6627292644 | 0.9732471856 |
| gprc5c | 0.02743030814 | 0.06297775735 | 0.4355554928 | 0.6631592685 | 1.027809983 |
| dpt | 0.02868733581 | 0.06587530401 | 0.4354793688 | 0.6632145111 | 1.029102781 |
| rbp2 | 0.0257522719 | 0.05919577112 | 0.4350356691 | 0.663536537 | 1.026086726 |
| kazald1 | -0.02559063831 | 0.05883648103 | -0.4349450862 | 0.6636022874 | 0.9747340267 |
| vstm2l | 0.02564198086 | 0.05903428115 | 0.4343574676 | 0.6640288785 | 1.025973565 |
| rgma | 0.02530099258 | 0.05846582348 | 0.4327484173 | 0.6651975511 | 1.025623779 |
| phactr2 | 0.02721673079 | 0.06290208621 | 0.4326840718 | 0.665244303 | 1.027590489 |
| adgrg2 | 0.02915285955 | 0.06744219794 | 0.432264375 | 0.6655492762 | 1.029581964 |
| tdrkh | 0.0252214994 | 0.05841336066 | 0.4317762087 | 0.6659040726 | 1.025542252 |
| gsap | -0.02490566452 | 0.05778551942 | -0.4310018283 | 0.6664670411 | 0.9754019227 |
| hdac9 | -0.02740638321 | 0.06371728129 | -0.4301248053 | 0.6671048568 | 0.9729657642 |
| cd200r1 | 0.02487114646 | 0.05782862275 | 0.4300836727 | 0.6671347765 | 1.025183014 |
| lamtor5 | -0.02746812833 | 0.06392798574 | -0.429672983 | 0.667433539 | 0.9729056902 |
| ppm1a | 0.02488576865 | 0.05792724689 | 0.4296038563 | 0.6674838314 | 1.025198004 |
| zbp1 | 0.02829143362 | 0.06588385443 | 0.4294137595 | 0.6676221422 | 1.028695437 |
| idua | -0.02479644617 | 0.05778981142 | -0.4290798942 | 0.6678650835 | 0.9755084603 |
| glt8d2 | -0.02487434994 | 0.05798863479 | -0.4289521564 | 0.6679580427 | 0.9754324675 |
| dok2 | -0.02508937539 | 0.05855656408 | -0.4284639268 | 0.6683133913 | 0.9752227472 |
| tspyl1 | 0.02786370332 | 0.06512224231 | 0.4278676891 | 0.6687474523 | 1.028255527 |
| rhoc | 0.02511071655 | 0.0587082275 | 0.4277205704 | 0.6688545717 | 1.025428646 |
| osm | 0.02583119434 | 0.06042792282 | 0.4274711612 | 0.6690361858 | 1.026167711 |
| il32 | 0.02533282083 | 0.05930843772 | 0.4271368763 | 0.669279635 | 1.025656424 |
| uhrf2 | -0.02694962034 | 0.06312903621 | -0.4268973829 | 0.6694540718 | 0.9734102804 |
| serpina5 | 0.02834549871 | 0.06640092431 | 0.4268840984 | 0.6694637482 | 1.028751055 |
| stam | -0.02723622857 | 0.06382058569 | -0.4267624353 | 0.6695523698 | 0.9731313329 |
| brap | 0.02677388073 | 0.06280560753 | 0.4262976156 | 0.6698909951 | 1.027135521 |
| itga5 | 0.02680701173 | 0.06297463351 | 0.4256795195 | 0.6703413876 | 1.027169552 |
| tnf | 0.02636911796 | 0.06195220053 | 0.4256365026 | 0.6703727375 | 1.026719859 |
| manf | -0.02470422911 | 0.05808446447 | -0.4253156043 | 0.6706066197 | 0.975598423 |
| ywhaq | 0.02671384367 | 0.06283279217 | 0.4251576724 | 0.670721738 | 1.027073857 |
| itgal | -0.02763187116 | 0.06499583425 | -0.4251329563 | 0.6707397545 | 0.9727463969 |
| metap1 | -0.02447436729 | 0.05775667348 | -0.4237496001 | 0.671748438 | 0.9758227016 |
| oga | 0.02687271907 | 0.06351793108 | 0.4230729593 | 0.6722420306 | 1.027237047 |
| serpina11 | 0.02710627577 | 0.06408080886 | 0.4230014609 | 0.6722941952 | 1.027476993 |
| casp2 | 0.02470993393 | 0.05843040386 | 0.4228951418 | 0.6723717675 | 1.025017755 |
| lelp1 | -0.02681763331 | 0.06342299866 | -0.4228376752 | 0.6724136976 | 0.9735387664 |
| mapt | -0.02456695255 | 0.05814940712 | -0.4224798458 | 0.6726748085 | 0.975732359 |
| nfatc1 | -0.02473392338 | 0.05854956552 | -0.4224441831 | 0.6727008339 | 0.9755694537 |
| yju2 | -0.02685452047 | 0.06361983623 | -0.422109236 | 0.6729452867 | 0.973502856 |
| ccl19 | -0.02495603992 | 0.05913972212 | -0.421984396 | 0.673036407 | 0.9753527877 |
| kdr | 0.02534442903 | 0.06010028709 | 0.4217022955 | 0.6732423287 | 1.02566833 |

| Protein | beta | se | z | p_val | HR |
|---|---|---|---|---|---|
| mtif3 | -0.02625167124 | 0.06233397933 | -0.4211454414 | 0.6736488812 | 0.9740899083 |
| mrc1 | 0.02828196418 | 0.06716369835 | 0.4210900364 | 0.6736893369 | 1.028685696 |
| nptn | 0.02494468713 | 0.05931130251 | 0.4205722362 | 0.6740674709 | 1.025258409 |
| dpy30 | 0.02574595242 | 0.06127644966 | 0.4201606418 | 0.6743681048 | 1.026080242 |
| col3a1 | 0.02652646684 | 0.06318466156 | 0.4198244666 | 0.67461369 | 1.026881425 |
| wif1 | -0.02500887444 | 0.05958106186 | -0.419745363 | 0.6746714824 | 0.9753012567 |
| trim58 | 0.0263395992 | 0.06290049503 | 0.4187502688 | 0.6753986534 | 1.026689552 |
| amy2a | 0.02503668424 | 0.05981613681 | 0.4185607024 | 0.6755372145 | 1.025352734 |
| mrpl28 | -0.02614612063 | 0.06246677139 | -0.4185604609 | 0.6755373911 | 0.9741927296 |
| ppif | -0.02592765434 | 0.06195293279 | -0.4185056811 | 0.6755774337 | 0.9744055811 |
| extl1 | 0.02675968042 | 0.06396730802 | 0.4183336966 | 0.675703156 | 1.027120936 |
| myo6 | 0.02624809725 | 0.06276635304 | 0.4181873883 | 0.6758101159 | 1.026595612 |
| sema4c | 0.02506028971 | 0.05997754705 | 0.417827853 | 0.6760729849 | 1.025376938 |
| hnrnpk | 0.0248269239 | 0.05947256474 | 0.4174517109 | 0.676348038 | 1.025137678 |
| erp29 | 0.0261444102 | 0.0626879011 | 0.4170567166 | 0.6766369232 | 1.026489173 |
| vasn | 0.02477574291 | 0.05946148654 | 0.416668744 | 0.6769207194 | 1.025085212 |
| htr1b | -0.0261477368 | 0.06275855352 | -0.4166402081 | 0.6769415947 | 0.9741911551 |
| plaur | 0.02659229758 | 0.06396653319 | 0.4157220386 | 0.6776134126 | 1.026949028 |
| slc39a5 | -0.02593893871 | 0.0624081533 | -0.415633813 | 0.6776779802 | 0.9743945856 |
| pms1 | -0.02639884686 | 0.06352836009 | -0.4155442832 | 0.6777435046 | 0.9739465566 |
| scarf1 | 0.024448482 | 0.05894426136 | 0.4147728962 | 0.6783081628 | 1.024749797 |
| evpl | -0.02614068519 | 0.06305991809 | -0.4145372525 | 0.6784806908 | 0.9741980247 |
| apbb1ip | 0.02589981137 | 0.06255737589 | 0.4140169084 | 0.6788617239 | 1.026238126 |
| pik3ip1 | -0.02579800226 | 0.0623494322 | -0.4137648307 | 0.6790463427 | 0.974531923 |
| pga4 | 0.02568858287 | 0.06216532341 | 0.4132301011 | 0.6794380363 | 1.026021378 |
| pfkfb2 | 0.0242505264 | 0.05878487126 | 0.4125300588 | 0.6799509534 | 1.024546962 |
| nampt | -0.02408490141 | 0.05838436603 | -0.41252313 | 0.6799560309 | 0.9762028252 |
| fam13a | 0.02619444869 | 0.06351362766 | 0.4124224935 | 0.6800297791 | 1.026540539 |
| ube2l6 | 0.02641143356 | 0.06409988372 | 0.4120355924 | 0.6803133356 | 1.026763306 |
| cabp2 | 0.02620980943 | 0.0637443286 | 0.4111708446 | 0.6809472652 | 1.026556307 |
| cbs | 0.02706319308 | 0.06589199313 | 0.4107205109 | 0.6812774853 | 1.027432727 |
| rab6a | 0.02471454079 | 0.06019814326 | 0.4105532073 | 0.681400181 | 1.025022477 |
| evi2b | 0.02643176448 | 0.06438281133 | 0.4105407007 | 0.6814093533 | 1.026784182 |
| cacna1h | -0.02598822879 | 0.06337195398 | -0.4100903816 | 0.6817396481 | 0.9743465588 |
| col4a1 | 0.0248551261 | 0.06064365595 | 0.4098553379 | 0.6819120695 | 1.02516659 |
| slc16a1 | 0.02413605967 | 0.05899816022 | 0.409098514 | 0.6824673668 | 1.024429692 |
| il15ra | 0.02440007201 | 0.05964532255 | 0.4090860938 | 0.6824764811 | 1.02470019 |
| tmed4 | 0.02624105051 | 0.06422038354 | 0.4086093708 | 0.6828263527 | 1.026588378 |
| clec11a | -0.02364051815 | 0.05789420279 | -0.4083399892 | 0.6830240845 | 0.9766367298 |
| ssbp1 | 0.02585256701 | 0.06334409131 | 0.4081291005 | 0.6831788965 | 1.026189643 |
| tctn3 | -0.026778951 | 0.06562144575 | -0.4080823075 | 0.6832132488 | 0.9735764258 |
| top1mt | -0.0258510618 | 0.06346812045 | -0.4073078203 | 0.6837819198 | 0.9744802161 |
| camkk1 | 0.02432242423 | 0.0598955749 | 0.4060804871 | 0.6846834626 | 1.024620627 |
| tjap1 | -0.02364614621 | 0.05824089571 | -0.4060058817 | 0.6847382788 | 0.9766312333 |
| stxbp1 | 0.0256204195 | 0.06317054243 | 0.4055754234 | 0.6850545898 | 1.025951443 |
| entpd5 | -0.02423537399 | 0.05983451678 | -0.4050400219 | 0.6854480927 | 0.9760559445 |
| nos3 | 0.02501640782 | 0.06188682256 | 0.4042283443 | 0.6860448124 | 1.025331944 |
| sorbs1 | 0.02752659132 | 0.06810957008 | 0.4041515941 | 0.6861012468 | 1.027908948 |
| igfbp4 | -0.0263179376 | 0.06512570187 | -0.4041098498 | 0.6861319421 | 0.9740253611 |
| col6a3 | -0.02631874429 | 0.06516229206 | -0.4038953121 | 0.6862897038 | 0.9740245754 |
| pdxdc1 | 0.02554598636 | 0.06330049496 | 0.4035669291 | 0.6865312087 | 1.025875081 |
| podxl2 | -0.02401658974 | 0.05953619584 | -0.4033947651 | 0.6866578372 | 0.9762695136 |
| mamdc2 | -0.02586814432 | 0.06424451207 | -0.4026514248 | 0.6872046731 | 0.9744635697 |
| tgfb1 | -0.02418650273 | 0.06017268624 | -0.4019515205 | 0.6877197051 | 0.9761036468 |
| il4r | 0.02380632625 | 0.05932661459 | 0.4012756571 | 0.6882171839 | 1.024091959 |
| lta4h | -0.02382790139 | 0.05973045268 | -0.3989238374 | 0.6899493243 | 0.9764537416 |
| ankrd54 | 0.0232251434 | 0.05823575761 | 0.3988124195 | 0.6900314252 | 1.023496947 |
| mansc1 | -0.0233215429 | 0.05848502663 | -0.3987609178 | 0.6900693767 | 0.9769483025 |
| tpr | -0.02632838153 | 0.06604612497 | -0.3986362795 | 0.6901612255 | 0.9740151885 |
| egfl7 | -0.02425695727 | 0.06098602683 | -0.3977461483 | 0.6908173164 | 0.9760348783 |
| sh3bp1 | -0.0253020501 | 0.06364789321 | -0.3975316199 | 0.6909754741 | 0.9750153641 |
| gh2 | 0.02294431583 | 0.0577398148 | 0.3973742539 | 0.6910914983 | 1.023209561 |
| pdia3 | -0.025139128 | 0.06334310566 | -0.3968723627 | 0.6914615855 | 0.9751742285 |
| adipoq | -0.02964087128 | 0.0747036955 | -0.3967791831 | 0.6915303028 | 0.970794111 |
| klk6 | 0.02306915266 | 0.05823955674 | 0.3961079711 | 0.6920253782 | 1.023337304 |
| bloc1s2 | 0.02495445406 | 0.06305206705 | 0.3957753525 | 0.6922707612 | 1.025268423 |
| fam172a | 0.02545139137 | 0.0643434939 | 0.39555501 | 0.6924333324 | 1.025778043 |
| calcb | 0.02530862991 | 0.06401646703 | 0.3953456209 | 0.6925878352 | 1.025631612 |
| tnfaip8l2 | 0.02504347778 | 0.06336163043 | 0.3952467386 | 0.6926608024 | 1.0253597 |
| cd70 | 0.02384081298 | 0.06031979455 | 0.3952402881 | 0.6926655624 | 1.024127277 |

| Protein | beta | se | z | p_val | HR |
|---|---|---|---|---|---|
| ntrk2 | 0.0240330023 | 0.06086173771 | 0.3948786742 | 0.6929324292 | 1.024324122 |
| grpel1 | -0.02388168648 | 0.06048117136 | -0.3948615072 | 0.6929450992 | 0.9764012244 |
| klhl41 | -0.02589522711 | 0.0656588806 | -0.3943903228 | 0.6932928874 | 0.9744371789 |
| tspan15 | -0.02463912562 | 0.06248280492 | -0.3943344998 | 0.6933340954 | 0.9756619399 |
| krt14 | -0.0233860459 | 0.05931306816 | -0.394281507 | 0.693373215 | 0.9768852884 |
| adamts16 | -0.02565499759 | 0.06510702602 | -0.3940434567 | 0.6935489556 | 0.9746712956 |
| kel | 0.02360431822 | 0.05993885295 | 0.393806639 | 0.6937238025 | 1.023885105 |
| mdga1 | 0.02392786248 | 0.06076961596 | 0.3937471399 | 0.6937677343 | 1.024216431 |
| angptl2 | 0.02434711648 | 0.06189005549 | 0.3933930304 | 0.6940292165 | 1.024645928 |
| slmap | 0.02502444046 | 0.06368106697 | 0.3929651567 | 0.6943452164 | 1.02534018 |
| igfbp7 | 0.02459114375 | 0.06259206112 | 0.3928795971 | 0.6944084116 | 1.024896 |
| steap4 | 0.02502206001 | 0.06377660031 | 0.3923391948 | 0.6948076071 | 1.025337739 |
| rnf31 | -0.025412906 | 0.06477673671 | -0.3923153171 | 0.6948252476 | 0.9749072838 |
| mindy1 | 0.02468854339 | 0.06301438563 | 0.391792178 | 0.6952117751 | 1.024995829 |
| ap1g2 | -0.02512513518 | 0.06420541227 | -0.3913242559 | 0.6955575719 | 0.9751878741 |
| map3k5 | 0.02292861649 | 0.05879481557 | 0.3899768417 | 0.6965536715 | 1.023193498 |
| scrn1 | 0.02296239912 | 0.05888817605 | 0.3899322522 | 0.696586644 | 1.023228065 |
| nphs1 | 0.02496935677 | 0.06403925709 | 0.3899070337 | 0.6966052926 | 1.025283702 |
| rest | -0.02493381722 | 0.06402191624 | -0.3894575277 | 0.6969377234 | 0.9753744629 |
| cd300e | -0.02436773341 | 0.06271114215 | -0.388571035 | 0.6975934969 | 0.9759267629 |
| vegfa | -0.0234434031 | 0.06035938673 | -0.388396973 | 0.697722284 | 0.9768292586 |
| dcbld2 | 0.02342735655 | 0.06033694051 | 0.3882755134 | 0.6978121561 | 1.023703933 |
| adgrv1 | -0.02431466117 | 0.06271766124 | -0.3876844367 | 0.6982495745 | 0.9759785589 |
| pi3 | 0.02464177636 | 0.06363654341 | 0.3872268203 | 0.6985882962 | 1.024947894 |
| rnaseh2a | -0.02467551591 | 0.06372775051 | -0.3872020542 | 0.6986066295 | 0.9756264359 |
| anxa4 | 0.02263580621 | 0.05850899841 | 0.3868773492 | 0.6988470106 | 1.02289394 |
| ncam1 | 0.02378424575 | 0.0615532234 | 0.3864013034 | 0.699199485 | 1.024069347 |
| erp44 | -0.02321372441 | 0.06007829862 | -0.3863911752 | 0.6992069848 | 0.9770536412 |
| fuom | 0.02662334058 | 0.06897930975 | 0.3859612495 | 0.6995253678 | 1.026980908 |
| lap3 | 0.02287879208 | 0.05936162377 | 0.3854138521 | 0.6999308213 | 1.023142519 |
| srpk2 | 0.02251749227 | 0.05842610917 | 0.3854011947 | 0.6999401976 | 1.022772925 |
| fgf12 | -0.02470528981 | 0.0641216343 | -0.3852878997 | 0.7000241255 | 0.9755973882 |
| adgre5 | -0.02338068717 | 0.0607363865 | -0.3849535429 | 0.7002718354 | 0.9768905233 |
| khk | -0.02576808674 | 0.06694234403 | -0.3849295557 | 0.7002896077 | 0.974561077 |
| cnpy2 | 0.02256780652 | 0.05869518544 | 0.384491613 | 0.700614111 | 1.022824386 |
| rassf2 | 0.02292722577 | 0.05972452839 | 0.3838829103 | 0.7010652334 | 1.023192075 |
| ddx1 | 0.02411617207 | 0.06283001223 | 0.3838320448 | 0.7011029356 | 1.024409319 |
| prtfdc1 | 0.02215113291 | 0.0577174254 | 0.3837858802 | 0.7011371541 | 1.022398291 |
| zbtb16 | 0.02255266049 | 0.05881045005 | 0.3834804949 | 0.7013635298 | 1.022808894 |
| ren | 0.02339419374 | 0.06104635709 | 0.3832201438 | 0.7015565434 | 1.023669984 |
| iglc2 | -0.02442089401 | 0.06396364897 | -0.3817933217 | 0.7026146727 | 0.9758748834 |
| cd300lg | -0.02292394942 | 0.06017054951 | -0.380982883 | 0.7032159498 | 0.977336808 |
| slc4a1 | 0.02418975976 | 0.06372607632 | 0.3795896618 | 0.7042500362 | 1.024484705 |
| folr2 | -0.02295175319 | 0.06051069129 | -0.3793007931 | 0.7044645108 | 0.9773096347 |
| pdia4 | 0.0240337671 | 0.06337692707 | 0.379219508 | 0.7045248663 | 1.024324906 |
| plscr3 | 0.02418100674 | 0.06379997199 | 0.3790128112 | 0.7046783504 | 1.024475738 |
| prdx1 | 0.02238315579 | 0.05918273058 | 0.3782041749 | 0.7052789243 | 1.022635538 |
| foxo1 | 0.02203857538 | 0.05840223834 | 0.3773584028 | 0.7059072754 | 1.022283219 |
| cd58 | 0.02219973069 | 0.05884293863 | 0.3772709387 | 0.7059722668 | 1.022447978 |
| folh1 | -0.02462409847 | 0.06529650653 | -0.3771120351 | 0.7060903476 | 0.9756766014 |
| il1b | -0.02227108538 | 0.05914923538 | -0.3765236395 | 0.7065276445 | 0.9779750844 |
| cd300c | -0.02225827352 | 0.05918864157 | -0.3760565022 | 0.706874891 | 0.9779876141 |
| calca | -0.02841755281 | 0.07578782012 | -0.3749620026 | 0.7076887259 | 0.9719824281 |
| osmr | 0.02290953543 | 0.06117232281 | 0.3745081824 | 0.7080262702 | 1.023173974 |
| tshb | 0.02173247597 | 0.05805105815 | 0.3743683003 | 0.7081303238 | 1.021970346 |
| sema4d | 0.02164569776 | 0.0578554272 | 0.3741342654 | 0.7083044267 | 1.021881665 |
| cinp | -0.02360989678 | 0.06314235578 | -0.3739153613 | 0.7084672874 | 0.9766666363 |
| cpa1 | 0.02133519245 | 0.05711715797 | 0.3735338593 | 0.7087511499 | 1.021564415 |
| rbp1 | -0.02357302311 | 0.06323114767 | -0.3728071367 | 0.7092919909 | 0.9767026502 |
| tsc1 | 0.02404258876 | 0.06465923495 | 0.3718353424 | 0.710015448 | 1.024333942 |
| dmp1 | 0.02491527784 | 0.06718731956 | 0.3708330382 | 0.7107618921 | 1.025228257 |
| tymp | 0.02184493839 | 0.05897039095 | 0.3704390973 | 0.7110553471 | 1.022085286 |
| chrm1 | -0.02363115564 | 0.06385852757 | -0.3700548155 | 0.711341648 | 0.9766458737 |
| pdcl2 | 0.02320372312 | 0.0628130067 | 0.3694095274 | 0.7118224976 | 1.023475024 |
| lama4 | -0.02175004651 | 0.05890072949 | -0.3692661653 | 0.7119293424 | 0.9784847802 |
| gbp1 | 0.02364756569 | 0.06413978166 | 0.3686879669 | 0.7123603189 | 1.023929386 |
| krt18 | -0.02457806055 | 0.06666635089 | -0.3686726544 | 0.7123717337 | 0.9757215206 |
| fgf3 | 0.02379715638 | 0.06469684538 | 0.3678256064 | 0.7130032744 | 1.024082568 |
| ggact | 0.02321311664 | 0.06313823537 | 0.3676554547 | 0.7131301595 | 1.023484638 |
| znf174 | -0.02359067235 | 0.0643622529 | -0.3665296239 | 0.7139699116 | 0.9766854123 |

| Protein | beta | se | z | p_val | HR |
|---|---|---|---|---|---|
| mamdc4 | 0.02425311396 | 0.06627642684 | 0.3659387676 | 0.7144107673 | 1.024549613 |
| hnrnpul1 | 0.02409445919 | 0.06589413792 | 0.3656540619 | 0.7146232289 | 1.024387076 |
| cd207 | -0.02149326002 | 0.05885879194 | -0.3651665166 | 0.7149871108 | 0.9787360741 |
| reep4 | 0.02280119258 | 0.06249876124 | 0.3648263122 | 0.7152410625 | 1.023063127 |
| gp5 | 0.02283823765 | 0.06260329469 | 0.3648088774 | 0.715254078 | 1.023101027 |
| fbln2 | 0.02371656334 | 0.06501508611 | 0.3647855407 | 0.7152714993 | 1.024000038 |
| fxyd5 | 0.02134151781 | 0.05852874123 | 0.3646331249 | 0.7153852847 | 1.021570877 |
| il9 | 0.02296047025 | 0.06303710162 | 0.364237404 | 0.7156807382 | 1.023226091 |
| bach1 | 0.02115992981 | 0.05811142923 | 0.3641268179 | 0.7157633116 | 1.021385389 |
| comp | 0.02231841517 | 0.06136787919 | 0.3636823606 | 0.7160952169 | 1.022569334 |
| acvrl1 | -0.02324208706 | 0.06408848136 | -0.3626562303 | 0.7168617 | 0.9770259298 |
| gfap | 0.02275124612 | 0.06278729434 | 0.3623543005 | 0.7170872852 | 1.02301203 |
| il24 | 0.0210728253 | 0.05815744793 | 0.3623409564 | 0.7170972558 | 1.021296425 |
| nxph1 | -0.02122992542 | 0.05862870257 | -0.3621080544 | 0.7172712849 | 0.9789938431 |
| ctsc | -0.02153314523 | 0.05948964817 | -0.3619645752 | 0.7173785029 | 0.9786970378 |
| crip2 | 0.02339992562 | 0.06479972509 | 0.3611114952 | 0.7180161003 | 1.023675852 |
| s100g | 0.02282328887 | 0.06321289337 | 0.3610543301 | 0.7180588329 | 1.023085733 |
| sult1a1 | 0.02073185738 | 0.05742510178 | 0.3610243035 | 0.718081279 | 1.020948255 |
| relb | 0.02335311255 | 0.06477209283 | 0.360542813 | 0.7184412462 | 1.023627932 |
| usp47 | -0.02325026275 | 0.06462677403 | -0.3597620816 | 0.7190250616 | 0.977017942 |
| ass1 | -0.02454612859 | 0.06830165515 | -0.3593782395 | 0.7193121512 | 0.9757526778 |
| gpnmb | 0.02157395848 | 0.06011075774 | 0.3589034524 | 0.7196673168 | 1.021808359 |
| il13ra1 | 0.02190109384 | 0.06104911539 | 0.358745474 | 0.7197855064 | 1.022142683 |
| metap2 | 0.0207219558 | 0.05789975593 | 0.3578936642 | 0.7204228924 | 1.020938146 |
| erbin | 0.02086302915 | 0.05830912372 | 0.3578004233 | 0.7204926738 | 1.021082184 |
| ctbs | -0.02343606725 | 0.06564491457 | -0.3570126856 | 0.721082309 | 0.9768364245 |
| jun | 0.02148260739 | 0.06034444227 | 0.355999767 | 0.7218407396 | 1.02171502 |
| ca5a | -0.02269230264 | 0.06413285468 | -0.3538327235 | 0.7234642481 | 0.9775632311 |
| pdap1 | 0.02236521849 | 0.06323973579 | 0.3536576839 | 0.7235954389 | 1.022617195 |
| spry2 | -0.02074889991 | 0.05867232355 | -0.3536403308 | 0.7236084454 | 0.9794648774 |
| pcdh12 | -0.02209771906 | 0.06249751945 | -0.3535775381 | 0.7236555103 | 0.978144647 |
| ccl15 | 0.0217174361 | 0.0615581896 | 0.3527952371 | 0.7242419547 | 1.021954976 |
| camsap1 | 0.02196214084 | 0.06226048489 | 0.3527460617 | 0.724278824 | 1.022205084 |
| il18r1 | 0.02208661379 | 0.06266586051 | 0.3524504988 | 0.7245004359 | 1.022332329 |
| btn2a1 | -0.02111242295 | 0.05998671718 | -0.351951631 | 0.7248745373 | 0.9791088841 |
| cd55 | 0.02137329969 | 0.06082660626 | 0.3513807691 | 0.7253027078 | 1.021603345 |
| gabarapl1 | -0.0221432202 | 0.06312552771 | -0.3507807539 | 0.725752837 | 0.9781001413 |
| erc2 | 0.02198260721 | 0.06274968181 | 0.35032221 | 0.7260968989 | 1.022226005 |
| fmr1 | 0.02043919328 | 0.05843796341 | 0.3497588226 | 0.7265197042 | 1.020649504 |
| ddx4 | -0.0224366277 | 0.06427619582 | -0.349065893 | 0.7270398414 | 0.9778132015 |
| cd1c | -0.02065619558 | 0.05932753204 | -0.3481721702 | 0.7277108867 | 0.9795556823 |
| klk1 | -0.02046950068 | 0.05884235871 | -0.3478701589 | 0.727937697 | 0.9797385774 |
| rab39b | -0.0222162924 | 0.06406911702 | -0.3467550894 | 0.7287753195 | 0.978028672 |
| lifr | -0.02051958603 | 0.05920906986 | -0.3465615332 | 0.7289207489 | 0.9796895081 |
| sla2 | 0.02186913215 | 0.0631934648 | 0.3460663571 | 0.7292928462 | 1.022110014 |
| fosb | 0.02005092236 | 0.05794721742 | 0.346020452 | 0.7293273446 | 1.020253292 |
| arl2bp | 0.02213297314 | 0.06401411053 | 0.345751475 | 0.7295294958 | 1.022379724 |
| iqgap2 | 0.02049300591 | 0.05933721211 | 0.3453651627 | 0.7298198639 | 1.020704429 |
| slitrk2 | 0.02059168867 | 0.05964264623 | 0.3452510908 | 0.7299056124 | 1.02080516 |
| rad51 | 0.02198327765 | 0.0637419441 | 0.3448793091 | 0.7301851063 | 1.02222669 |
| cmip | 0.02176569021 | 0.06316830314 | 0.3445666438 | 0.7304201861 | 1.022004291 |
| l1cam | 0.02037599878 | 0.05917251164 | 0.3443490604 | 0.7305837928 | 1.020585007 |
| scin | -0.02189144322 | 0.06364242996 | -0.3439756029 | 0.7308646339 | 0.9783464354 |
| brd1 | 0.021833285 | 0.06348101391 | 0.3439340939 | 0.730895851 | 1.022073375 |
| pdgfa | -0.01995694122 | 0.05808705157 | -0.3435695336 | 0.73117004 | 0.9802408804 |
| rnase10 | 0.02212400887 | 0.06440015136 | 0.3435397031 | 0.7311924774 | 1.02237056 |
| man2b2 | 0.02228594248 | 0.06488137079 | 0.3434875406 | 0.7312317125 | 1.022536129 |
| pcdh7 | 0.02222326523 | 0.06474796982 | 0.3432272129 | 0.7314275338 | 1.022472041 |
| treh | -0.02195882433 | 0.06398792126 | -0.3431713969 | 0.7314695215 | 0.9782805156 |
| cenpf | 0.02208406311 | 0.06436457746 | 0.3431089581 | 0.7315164922 | 1.022329721 |
| dapp1 | 0.0198271115 | 0.05781889171 | 0.3429175295 | 0.7316605039 | 1.020024974 |
| npr1 | -0.02191340367 | 0.06399762591 | -0.3424096341 | 0.7320426393 | 0.9783249507 |
| mxra8 | -0.02374479776 | 0.06970159328 | -0.3406636296 | 0.7333568219 | 0.9765348919 |
| ncf2 | 0.01989324834 | 0.05846867895 | 0.3402376914 | 0.7336775359 | 1.020092438 |
| ahcy | 0.02024693451 | 0.05951913541 | 0.3401752121 | 0.7337245842 | 1.020453294 |
| lyn | 0.0196751905 | 0.05806963507 | 0.3388206327 | 0.734744857 | 1.019870023 |
| prc1 | 0.02142457323 | 0.06333688349 | 0.3382637738 | 0.7351644206 | 1.021655727 |
| nt5e | 0.0199887084 | 0.05910701996 | 0.338178247 | 0.7352288675 | 1.02018982 |
| sirt1 | 0.02177059077 | 0.06457307256 | 0.3371465831 | 0.7360064027 | 1.022009299 |
| dbn1 | 0.02179923942 | 0.06468755405 | 0.3369927915 | 0.7361223341 | 1.022038579 |

| Protein | beta | se | z | p_val | HR |
| --- | --- | --- | --- | --- | --- |
| cemip2 | 0.02186273338 | 0.06509971977 | 0.3358345237 | 0.7369956545 | 1.022103474 |
| agr2 | 0.02013498979 | 0.0600981771 | 0.3350349505 | 0.7375987216 | 1.020339066 |
| ndufa5 | -0.02185177431 | 0.06522994077 | -0.3349960777 | 0.737628045 | 0.9783852461 |
| ltbp2 | -0.02324667624 | 0.06943181112 | -0.3348130469 | 0.737766118 | 0.9770214461 |
| stat2 | 0.02174712027 | 0.06504988081 | 0.3343145291 | 0.7381422279 | 1.021985312 |
| c1rl | 0.02216058652 | 0.06630602908 | 0.3342167647 | 0.7382159942 | 1.022407956 |
| cep350 | 0.02136116741 | 0.06418492399 | 0.3328066169 | 0.7392802632 | 1.02159095 |
| lpl | -0.02247520986 | 0.06754926431 | -0.3327232368 | 0.7393432076 | 0.9777754761 |
| eif4g1 | 0.0193030743 | 0.05804707538 | 0.3325417202 | 0.7394802421 | 1.019490583 |
| nucb2 | -0.01993087181 | 0.05999571786 | -0.3322049059 | 0.7397345395 | 0.980266435 |
| bglap | 0.02081446004 | 0.06274480655 | 0.331731998 | 0.7400916367 | 1.021032592 |
| nexn | 0.02056667307 | 0.06210091749 | 0.3311814688 | 0.7405074169 | 1.020779624 |
| grk5 | -0.01942565295 | 0.05867584304 | -0.3310673003 | 0.7405936507 | 0.9807618092 |
| hjv | -0.02202646348 | 0.06653525245 | -0.3310495214 | 0.7406070798 | 0.9782143478 |
| ces2 | -0.02023681709 | 0.0612793469 | -0.3302387854 | 0.7412195427 | 0.979966573 |
| arhgef12 | -0.01913735317 | 0.0580248947 | -0.329812803 | 0.7415414128 | 0.9810446034 |
| rnase1 | -0.02254034017 | 0.06866838071 | -0.3282491874 | 0.74272326 | 0.9777117953 |
| gpihbp1 | -0.02049931496 | 0.06268631194 | -0.3270142129 | 0.7436571354 | 0.9797093676 |
| upk3a | 0.0205920592 | 0.06298321015 | 0.3269452152 | 0.7437093219 | 1.020805538 |
| igsf9 | 0.02423523591 | 0.07415922591 | 0.3268000119 | 0.7438191504 | 1.024531296 |
| fap | 0.01959456764 | 0.05996865756 | 0.3267468113 | 0.7438593915 | 1.019787801 |
| spesp1 | 0.02218496262 | 0.06795157733 | 0.3264819375 | 0.7440597531 | 1.022432879 |
| ambp | 0.02006549464 | 0.06146184452 | 0.3264707527 | 0.7440682141 | 1.02026816 |
| itgb1 | 0.01983131691 | 0.06076052286 | 0.3263848956 | 0.7441331639 | 1.020029264 |
| stambp | 0.01934144688 | 0.05932388472 | 0.326031361 | 0.7444006277 | 1.019529704 |
| dnajb1 | 0.01924399067 | 0.05909331966 | 0.3256542496 | 0.7446859623 | 1.01943035 |
| rrp15 | 0.02102971753 | 0.06462508875 | 0.325411043 | 0.7448699989 | 1.0212524 |
| ldlrap1 | 0.02040961956 | 0.06274593115 | 0.3252739928 | 0.7449737123 | 1.02061932 |
| asgr1 | 0.02124884323 | 0.06543145461 | 0.3247496691 | 0.7453705398 | 1.021476207 |
| cr1 | -0.02069586376 | 0.06373961173 | -0.3246939102 | 0.7454127441 | 0.9795168258 |
| aspscr1 | 0.02061178108 | 0.06364855204 | 0.3238373917 | 0.7460611458 | 1.020825671 |
| thtpa | 0.02055225401 | 0.06352090939 | 0.323551004 | 0.7462779872 | 1.020764906 |
| edf1 | 0.02064157417 | 0.06389358987 | 0.3230617377 | 0.7466484867 | 1.020856085 |
| il18 | 0.01954194343 | 0.06051434006 | 0.3229307866 | 0.7467476601 | 1.019734137 |
| heph | 0.02110089607 | 0.06537263193 | 0.3227787447 | 0.7468628114 | 1.021325094 |
| f2r | -0.0190710211 | 0.05909784944 | -0.3227024551 | 0.7469205927 | 0.9811096803 |
| cep112 | -0.02081021378 | 0.06506176844 | -0.3198531838 | 0.7490796288 | 0.9794048245 |
| il22 | -0.02121211784 | 0.06642979165 | -0.3193163385 | 0.7494866443 | 0.9790112768 |
| ccnd2 | 0.02025974993 | 0.06352699124 | 0.3189156221 | 0.7497904977 | 1.020466372 |
| ccl18 | -0.02027899226 | 0.06359220866 | -0.3188911455 | 0.7498090589 | 0.9799252436 |
| ttn | -0.02105453545 | 0.06607941968 | -0.3186247027 | 0.750011119 | 0.9791655639 |
| kirrel2 | 0.0189816406 | 0.05963289895 | 0.3183081979 | 0.7502511664 | 1.019162937 |
| dctpp1 | 0.01925741536 | 0.06050571532 | 0.3182743193 | 0.7502768625 | 1.019444035 |
| lrp2 | -0.02028199115 | 0.06403474072 | -0.3167341809 | 0.7514453105 | 0.9799223049 |
| usp25 | 0.02022346251 | 0.06390314699 | 0.3164705255 | 0.7516453936 | 1.020429342 |
| csf1r | -0.02042428193 | 0.06455780601 | -0.3163719957 | 0.7517201703 | 0.9797828809 |
| znrf4 | 0.02006742573 | 0.06349688594 | 0.316037951 | 0.7519737025 | 1.02027013 |
| ccl13 | -0.01856330621 | 0.05877265154 | -0.3158493912 | 0.7521168268 | 0.9816079307 |
| hrc | 0.02015041044 | 0.06381127793 | 0.3157813335 | 0.7521684874 | 1.0203548 |
| col18a1 | -0.01955683263 | 0.06199030955 | -0.3154820934 | 0.7523956448 | 0.9806331616 |
| aspn | -0.02045194525 | 0.06488620657 | -0.3151971172 | 0.7526119943 | 0.9797557773 |
| nck2 | -0.01824173429 | 0.0579060316 | -0.3150230432 | 0.7527441581 | 0.9819236391 |
| scara5 | 0.01964376644 | 0.06236341869 | 0.314988608 | 0.7527703034 | 1.019837975 |
| gdnf | -0.01904135944 | 0.06057969508 | -0.3143191694 | 0.7532786404 | 0.9811387821 |
| fstl1 | 0.0203892052 | 0.0650023617 | 0.3136686832 | 0.7537726884 | 1.020598485 |
| vim | 0.01845870783 | 0.05887488462 | 0.3135243143 | 0.7538823511 | 1.018630123 |
| lrpap1 | 0.01900393231 | 0.06062698838 | 0.3134566439 | 0.7539337552 | 1.019185656 |
| tff1 | -0.01969054523 | 0.06290879353 | -0.3130014761 | 0.7542795407 | 0.9805020474 |
| cdh22 | 0.01967013016 | 0.06286104937 | 0.3129144417 | 0.7543456653 | 1.019864862 |
| akap12 | -0.0196730032 | 0.06305809715 | -0.3119821893 | 0.7550540591 | 0.9805192475 |
| ccl17 | -0.01829061751 | 0.05866771524 | -0.3117663171 | 0.7552181241 | 0.9818756406 |
| txlna | -0.01849005121 | 0.05933252135 | -0.3116343413 | 0.7553184325 | 0.9816798411 |
| foxo3 | 0.01811469318 | 0.05813393692 | 0.3116027253 | 0.7553424628 | 1.018279759 |
| alcam | 0.01956935894 | 0.06284622031 | 0.3113848191 | 0.7555080933 | 1.019762094 |
| tars1 | 0.01967776316 | 0.06323856848 | 0.3111671189 | 0.7556735784 | 1.019872647 |
| prkag3 | 0.0196191275 | 0.06306178036 | 0.3111096355 | 0.7557172763 | 1.019812847 |
| sfrp1 | 0.02014203548 | 0.06480717926 | 0.310799447 | 0.7559530901 | 1.020346255 |
| fkbp5 | -0.01825346037 | 0.05878518292 | -0.3105112455 | 0.7561722091 | 0.981912125 |
| lyve1 | -0.01975826896 | 0.06372067151 | -0.3100762828 | 0.7565029476 | 0.9804356464 |
| prss22 | 0.02035102436 | 0.06563290138 | 0.310073514 | 0.7565050531 | 1.020559518 |

| Protein | beta | se | z | p_val | HR |
|---|---|---|---|---|---|
| il2rg | -0.01997866934 | 0.06447217139 | -0.3098805098 | 0.7566518247 | 0.9802195818 |
| cd177 | -0.01775910346 | 0.05739865241 | -0.3093993102 | 0.7570177951 | 0.9823976601 |
| gzmh | 0.01884295966 | 0.06097423032 | 0.3090315296 | 0.7572975428 | 1.019021609 |
| thbs2 | -0.01826192454 | 0.05917235267 | -0.3086225867 | 0.7576086374 | 0.981903814 |
| dync1h1 | 0.01977528396 | 0.06415425308 | 0.3082458764 | 0.7578952465 | 1.01997211 |
| tex33 | -0.01945907195 | 0.06314771865 | -0.3081516224 | 0.7579669621 | 0.9807290337 |
| gfod2 | -0.01794312012 | 0.05834295406 | -0.3075456225 | 0.7584281028 | 0.9822168991 |
| csf2ra | 0.01815976726 | 0.05905507409 | 0.3075056215 | 0.7584585448 | 1.018325658 |
| tnfsf14 | 0.01829606241 | 0.05950984769 | 0.3074459627 | 0.7585039479 | 1.018464461 |
| smad2 | 0.01953808348 | 0.06357130823 | 0.3073412208 | 0.7585836633 | 1.019730201 |
| ptprn2 | 0.01794659216 | 0.05844072289 | 0.3070905231 | 0.7587744709 | 1.0181086 |
| guca2a | 0.01830889369 | 0.05965761156 | 0.3068995424 | 0.7589198374 | 1.018477529 |
| ilkap | -0.01782102102 | 0.05809992938 | -0.3067305109 | 0.7590485042 | 0.9823368343 |
| crkl | -0.017890647 | 0.05836384329 | -0.3065364786 | 0.7591962098 | 0.9822684405 |
| cfd | 0.02072597069 | 0.06770144911 | 0.3061377705 | 0.7594997509 | 1.020942245 |
| pmvk | -0.01787323024 | 0.05849276529 | -0.3055630923 | 0.7599373252 | 0.9822855486 |
| pdgfra | -0.0182998977 | 0.0599509403 | -0.3052478846 | 0.7601773649 | 0.9818665287 |
| eif2s2 | 0.01963078727 | 0.06431923342 | 0.3052086634 | 0.7602072346 | 1.019824738 |
| tbr1 | 0.01921233613 | 0.0630534501 | 0.3046992052 | 0.760595255 | 1.019398081 |
| ctso | 0.01840691975 | 0.06055601469 | 0.3039651775 | 0.761154421 | 1.018577371 |
| mitd1 | -0.01781063732 | 0.0587793082 | -0.3030086243 | 0.7618832891 | 0.9823470346 |
| il13ra2 | -0.01910230811 | 0.06306237191 | -0.3029113485 | 0.7619574226 | 0.9810789848 |
| bpifb2 | 0.02094981077 | 0.06921967743 | 0.3026568679 | 0.7621513714 | 1.021170799 |
| fam171b | -0.01924227303 | 0.06361652814 | -0.3024728571 | 0.7622916219 | 0.9809416777 |
| klb | 0.01785198057 | 0.05916671268 | 0.3017233806 | 0.7628629433 | 1.01801228 |
| btnl10 | 0.01919531993 | 0.06365814192 | 0.3015375465 | 0.7630046235 | 1.019380735 |
| daam1 | -0.01867469473 | 0.06200141035 | -0.3011979022 | 0.7632635894 | 0.981498597 |
| sumf1 | 0.01954640149 | 0.06494837755 | 0.3009528833 | 0.7634504234 | 1.019738683 |
| rps10 | -0.0191650323 | 0.06378017384 | -0.3004857332 | 0.7638066772 | 0.9810174493 |
| treml2 | -0.01756832132 | 0.05853975619 | -0.3001092328 | 0.7640938369 | 0.9825851019 |
| dand5 | 0.01903036804 | 0.06342250389 | 0.3000570283 | 0.7641336562 | 1.0192126 |
| vgf | -0.01936592413 | 0.06454795077 | -0.3000238411 | 0.7641589703 | 0.9808203907 |
| sirt5 | -0.01725864199 | 0.05761001315 | -0.2995771229 | 0.7644997364 | 0.9828894353 |
| dipk2b | -0.01975648667 | 0.06598540037 | -0.2994069379 | 0.7646295692 | 0.9804373938 |
| apoh | 0.01728790695 | 0.05790941001 | 0.2985336399 | 0.765295905 | 1.017438208 |
| ninj1 | -0.01815569907 | 0.06090063331 | -0.2981200372 | 0.7656115491 | 0.9820081227 |
| ecscr | -0.01878070602 | 0.06300293872 | -0.2980925398 | 0.7656325352 | 0.9813945526 |
| cox6b1 | -0.018815983 | 0.06341356747 | -0.296718569 | 0.7666813791 | 0.9813599325 |
| gga1 | -0.01723741261 | 0.05821773896 | -0.2960852297 | 0.7671649935 | 0.9829103016 |
| siglec8 | 0.01953246033 | 0.06620206169 | 0.2950430822 | 0.7679609689 | 1.019724467 |
| sorcs2 | 0.01813976819 | 0.06153160699 | 0.2948040703 | 0.7681435569 | 1.018305293 |
| espl1 | 0.01868940225 | 0.06343336239 | 0.2946304839 | 0.7682761726 | 1.018865142 |
| igfbpl1 | 0.0186612228 | 0.0634067573 | 0.2943096855 | 0.7685212723 | 1.018836432 |
| rrm2b | 0.01736704785 | 0.05901342146 | 0.2942897975 | 0.7685364681 | 1.017518732 |
| sh3bgrl2 | 0.01848257942 | 0.06299142744 | 0.293414202 | 0.7692055713 | 1.018654439 |
| f7 | 0.01765966159 | 0.06018700725 | 0.2934131867 | 0.7692063473 | 1.017816515 |
| gsta3 | 0.01876273801 | 0.06397810812 | 0.2932680969 | 0.7693172371 | 1.018939864 |
| prdx3 | -0.01723867695 | 0.05878446084 | -0.2932522762 | 0.7693293289 | 0.9829090589 |
| cacnb1 | 0.01858243866 | 0.06347543005 | 0.2927501025 | 0.7697131704 | 1.018756167 |
| mmp3 | 0.02206098027 | 0.07541957271 | 0.292510014 | 0.7698967045 | 1.022306123 |
| ceacam20 | -0.0188085678 | 0.0646408511 | -0.2909702994 | 0.7710740348 | 0.9813672095 |
| igf2bp3 | -0.01861213848 | 0.06398441768 | -0.2908854867 | 0.7711389015 | 0.9815599978 |
| il6r | 0.0170583885 | 0.05880006072 | 0.2901083483 | 0.7717333493 | 1.017204714 |
| txndc5 | -0.01695213327 | 0.05855152255 | -0.2895250632 | 0.7721796031 | 0.9831907456 |
| gys1 | 0.01708177961 | 0.059083792 | 0.2891110917 | 0.7724963659 | 1.017228507 |
| myl4 | -0.01909073588 | 0.06605424869 | -0.2890160172 | 0.7725691204 | 0.9810903381 |
| ctsh | 0.01731527461 | 0.05996629242 | 0.2887501281 | 0.7727725991 | 1.017466053 |
| pdcd6 | -0.01686573228 | 0.05851820938 | -0.2882134034 | 0.7731833895 | 0.983275698 |
| ccl5 | -0.01679721179 | 0.05829530036 | -0.2881400678 | 0.773239523 | 0.9833430748 |
| aldh5a1 | -0.01830669279 | 0.06361572405 | -0.2877699353 | 0.7735228525 | 0.9818598568 |
| itgb1bp2 | -0.01686288375 | 0.05891012759 | -0.2862476189 | 0.7746884743 | 0.9832784989 |
| treml1 | 0.01798160599 | 0.06284025849 | 0.286147868 | 0.7747648702 | 1.018144248 |
| dffa | 0.01667954664 | 0.05835427354 | 0.2858324786 | 0.7750064311 | 1.016819427 |
| havcr2 | 0.01772940041 | 0.06205053764 | 0.2857251699 | 0.7750886252 | 1.017887499 |
| thpo | -0.01684425277 | 0.05897434516 | -0.2856200052 | 0.7751691796 | 0.9832968185 |
| nadk | 0.01720955141 | 0.06029609632 | 0.2854173398 | 0.7753244246 | 1.017358489 |
| cx3cl1 | -0.01685383584 | 0.05921701786 | -0.2846113575 | 0.7759419089 | 0.9832873955 |
| hars1 | 0.01657329571 | 0.05831389551 | 0.2842083446 | 0.776250721 | 1.016711395 |
| il12rb1 | 0.01674897146 | 0.05913590601 | 0.2832284578 | 0.7770017149 | 1.016890022 |
| slc9a3r1 | 0.01778300361 | 0.06287649251 | 0.2828243577 | 0.7773114816 | 1.017942063 |

| Protein | beta | se | z | p_val | HR |
|---|---|---|---|---|---|
| gba | 0.01786994254 | 0.06323789814 | 0.2825828034 | 0.7774966641 | 1.018030565 |
| plxnb2 | -0.01707029099 | 0.06059590271 | -0.281707017 | 0.7781681735 | 0.9830745809 |
| rwdd1 | 0.01632407336 | 0.05802914544 | 0.2813081811 | 0.778474036 | 1.016458039 |
| plxna4 | -0.01621545888 | 0.05766193984 | -0.2812159792 | 0.7785447493 | 0.9839153039 |
| defb118 | 0.01786600283 | 0.06357058508 | 0.2810419757 | 0.7786782048 | 1.018026555 |
| epha4 | -0.01752726589 | 0.06241987687 | -0.2807962266 | 0.778866698 | 0.9826254431 |
| ppp1r12a | 0.01632518805 | 0.0581528015 | 0.280729176 | 0.7789181291 | 1.016459172 |
| epo | 0.01679782861 | 0.05986617511 | 0.2805896414 | 0.7790251619 | 1.016939705 |
| atxn2l | -0.01784387568 | 0.06383661935 | -0.2795241331 | 0.7798426203 | 0.9823143835 |
| adcyap1r1 | -0.01636539165 | 0.05876501719 | -0.2784886729 | 0.780637259 | 0.9837677938 |
| denr | 0.01803295076 | 0.06485650552 | 0.2780438233 | 0.7809787184 | 1.018196526 |
| btla | 0.01796180941 | 0.06462961494 | 0.2779191772 | 0.7810744024 | 1.018124093 |
| dgka | 0.01799717006 | 0.064881038 | 0.2773872092 | 0.781482802 | 1.018160095 |
| clec4m | 0.01730099339 | 0.06267351374 | 0.27604952 | 0.7825100318 | 1.017451522 |
| egf | -0.01593995853 | 0.05789745046 | -0.2753136521 | 0.7830752768 | 0.9841864103 |
| sort1 | -0.01637670011 | 0.05968921649 | -0.274366143 | 0.783803259 | 0.983756669 |
| tek | 0.01617274651 | 0.0589682546 | 0.2742619164 | 0.7838833491 | 1.016304233 |
| azu1 | -0.01641614986 | 0.05996775509 | -0.2737496149 | 0.7842770464 | 0.9837178608 |
| babam1 | -0.01750803125 | 0.06416290894 | -0.2728684147 | 0.7849543669 | 0.9826443438 |
| pigr | 0.016817116 | 0.06165809406 | 0.2727479053 | 0.7850470072 | 1.01695932 |
| gnpda2 | -0.01731937979 | 0.06352811847 | -0.2726254171 | 0.7851411718 | 0.9828297385 |
| nagpa | 0.01744812699 | 0.06410813807 | 0.2721671151 | 0.7854935261 | 1.017601235 |
| pard3 | 0.01723222638 | 0.0633809501 | 0.2718833711 | 0.7857116977 | 1.017381558 |
| znf75d | 0.01729710521 | 0.06364041748 | 0.2717943392 | 0.7857801581 | 1.017447566 |
| pik3ap1 | 0.01582646337 | 0.05827615666 | 0.2715769926 | 0.785947292 | 1.015952365 |
| acy3 | 0.01777257975 | 0.06564387914 | 0.2707423751 | 0.786589183 | 1.017931452 |
| cacna1c | 0.01720808041 | 0.06372337514 | 0.2700434554 | 0.7871268224 | 1.017356992 |
| mthfsd | 0.01734773872 | 0.06428704104 | 0.2698481442 | 0.7872770825 | 1.017499085 |
| sparcl1 | 0.01600684493 | 0.05941823803 | 0.2693927902 | 0.7876274337 | 1.016135641 |
| snapin | -0.01707509514 | 0.06339395002 | -0.2693489701 | 0.7876611514 | 0.9830698581 |
| cpa2 | 0.01562207242 | 0.05814775527 | 0.2686616595 | 0.7881900586 | 1.015744735 |
| kazn | 0.01679976634 | 0.06253894665 | 0.2686288663 | 0.7882152964 | 1.016941676 |
| nppc | 0.01791069385 | 0.06668710216 | 0.2685780798 | 0.7882543824 | 1.018072052 |
| ephb4 | -0.01563017402 | 0.05826012044 | -0.268282556 | 0.7884818325 | 0.9844913432 |
| saa4 | 0.01716102544 | 0.06404547867 | 0.2679506157 | 0.7887373321 | 1.017309122 |
| ghrl | 0.01668197565 | 0.06228509306 | 0.2678325556 | 0.7888282102 | 1.016821897 |
| wfdc12 | -0.01720247414 | 0.0642632679 | -0.2676875095 | 0.788939865 | 0.9829446436 |
| cpe | -0.01545266105 | 0.05775958 | -0.2675341657 | 0.7890579121 | 0.9846661187 |
| casq2 | -0.01709884097 | 0.06398631515 | -0.2672265301 | 0.7892947506 | 0.9830465146 |
| mslnl | -0.01682023545 | 0.06296562851 | -0.2671336068 | 0.789366293 | 0.9833204349 |
| tigar | 0.01560675223 | 0.05862630317 | 0.2662073401 | 0.7900795304 | 1.015729174 |
| kir3dl2 | 0.01680336831 | 0.06329312345 | 0.2654848963 | 0.7906359436 | 1.016945339 |
| srpx | -0.01753000007 | 0.06605419567 | -0.2653881391 | 0.7907104723 | 0.9826227565 |
| igfbp1 | -0.01730879889 | 0.06542546999 | -0.2645575018 | 0.7913503625 | 0.9828401378 |
| acox1 | -0.01528678615 | 0.05807467362 | -0.2632263807 | 0.7923760987 | 0.9848294637 |
| fmnl1 | 0.01562968803 | 0.05946600089 | 0.2628340194 | 0.7926785134 | 1.01575247 |
| lrrfip1 | 0.01669693433 | 0.06385365671 | 0.26148752 | 0.7937165724 | 1.016837107 |
| omg | -0.01541693776 | 0.05903968721 | -0.2611283781 | 0.7939935081 | 0.9847012948 |
| lrtm2 | 0.01758562294 | 0.067549657 | 0.2603362285 | 0.7946044295 | 1.01774116 |
| eps8l2 | -0.01586045132 | 0.06092962218 | -0.2603077248 | 0.7946264144 | 0.9842646633 |
| p4hb | -0.01571405853 | 0.06039386206 | -0.2601929731 | 0.7947149243 | 0.9844087631 |
| phospho1 | 0.01653089299 | 0.06354687799 | 0.2601369809 | 0.794758113 | 1.016668284 |
| pbld | -0.01560196184 | 0.06017910194 | -0.2592588015 | 0.7954355645 | 0.9845191183 |
| map2 | -0.01636085185 | 0.06316277951 | -0.2590267873 | 0.7956145724 | 0.98377226 |
| syap1 | -0.01629670182 | 0.06293763623 | -0.2589341258 | 0.7956860672 | 0.983835371 |
| nxph3 | 0.01736266516 | 0.06711560591 | 0.2586978829 | 0.7958683531 | 1.017514272 |
| cdkl5 | 0.0161305781 | 0.06245463097 | 0.2582767338 | 0.796193341 | 1.016261378 |
| smc3 | -0.01630010539 | 0.06329514335 | -0.2575253728 | 0.796773231 | 0.9838320225 |
| itgav | -0.01589334098 | 0.06172909583 | -0.2574692009 | 0.7968165881 | 0.9842322917 |
| lactb2 | 0.01512845213 | 0.05877994886 | 0.2573743671 | 0.7968897886 | 1.015243466 |
| kir2dl3 | 0.01493081471 | 0.05801698881 | 0.2573524586 | 0.7969066996 | 1.015042836 |
| icam4 | -0.01497275896 | 0.058235686 | -0.257106252 | 0.7970967513 | 0.9851387754 |
| gfer | 0.01556648559 | 0.06069070432 | 0.2564887946 | 0.7975734318 | 1.015688274 |
| sh3gl3 | 0.01624543825 | 0.06366802492 | 0.2551585081 | 0.7986006763 | 1.016378113 |
| hexim1 | 0.01482322071 | 0.05818744654 | 0.2547494622 | 0.7989166109 | 1.01493363 |
| ap3b1 | -0.01607357665 | 0.0631184839 | -0.2546572043 | 0.7989878726 | 0.9840549139 |
| ssna1 | -0.01606616889 | 0.06314530293 | -0.2544317336 | 0.7991620375 | 0.9840622036 |
| lgals8 | -0.01482495088 | 0.05842748774 | -0.2537324718 | 0.7997022459 | 0.9852843977 |
| fcgr2a | -0.01464276006 | 0.05788944359 | -0.2529435274 | 0.8003118529 | 0.9854639238 |
| cd276 | 0.01480056051 | 0.05856801407 | 0.2527072285 | 0.8004944617 | 1.014910631 |

| Protein | beta | se | z | p_val | HR |
|---|---|---|---|---|---|
| edil3 | 0.01597850259 | 0.06325616135 | 0.2525999404 | 0.8005773761 | 1.016106842 |
| mnda | 0.01474623933 | 0.05870696432 | 0.2511838161 | 0.8016719964 | 1.014855502 |
| tnfaip8 | -0.01469826543 | 0.05852852985 | -0.2511299271 | 0.8017136587 | 0.9854092268 |
| lrrc59 | -0.01578505828 | 0.06324194643 | -0.2495979199 | 0.8028983073 | 0.9843388728 |
| tnfrsf13c | -0.01478309492 | 0.05938865474 | -0.2489211953 | 0.8034217398 | 0.9853256386 |
| efna4 | 0.01528655955 | 0.06141555412 | 0.2489037145 | 0.803435262 | 1.015403997 |
| rgl2 | -0.01591205626 | 0.06405505527 | -0.2484121853 | 0.8038155065 | 0.9842138717 |
| igf1r | 0.01497056839 | 0.06040698058 | 0.2478284503 | 0.8042671413 | 1.015083189 |
| skap2 | 0.0143335268 | 0.057951118 | 0.2473382273 | 0.8046464765 | 1.014436744 |
| sap18 | 0.01561085901 | 0.06320570268 | 0.2469849769 | 0.8049198507 | 1.015733345 |
| azi2 | -0.01550930305 | 0.06285338481 | -0.2467536648 | 0.8050988719 | 0.9846103468 |
| clec10a | -0.01463904087 | 0.05938524912 | -0.2465097156 | 0.8052876845 | 0.9854675889 |
| bin2 | 0.01421767831 | 0.0577427133 | 0.2462246316 | 0.8055083492 | 1.01431923 |
| cda | 0.01606483011 | 0.06531024201 | 0.2459771946 | 0.8056998863 | 1.016194563 |
| cdkn2d | 0.01428952025 | 0.05818325433 | 0.2455950671 | 0.8059957082 | 1.014392103 |
| angpt1 | -0.01426849488 | 0.05811170879 | -0.2455356275 | 0.8060417255 | 0.9858328177 |
| gart | 0.01554792624 | 0.06346549921 | 0.2449823359 | 0.8064701086 | 1.015669424 |
| agrn | -0.01514328389 | 0.06191974036 | -0.2445631038 | 0.8067947355 | 0.984970799 |
| ntf3 | 0.01434585987 | 0.05871955461 | 0.2443114558 | 0.8069896118 | 1.014449256 |
| rap1a | 0.01567948177 | 0.06424245032 | 0.2440673059 | 0.8071786931 | 1.01580305 |
| ebag9 | -0.01425497735 | 0.05848491896 | -0.243737661 | 0.8074340036 | 0.9858461438 |
| camlg | 0.01544142457 | 0.06338875079 | 0.2435988148 | 0.8075415463 | 1.015561259 |
| rgcc | -0.01518379544 | 0.06233347195 | -0.2435897595 | 0.8075485602 | 0.9849308972 |
| rapgef2 | 0.01564957894 | 0.06438135342 | 0.2430762653 | 0.8079463174 | 1.015772675 |
| tk1 | 0.01546646303 | 0.0636844687 | 0.2428608315 | 0.8081132092 | 1.015586688 |
| lrch4 | -0.0159054444 | 0.06559924961 | -0.2424638162 | 0.8084207909 | 0.9842203792 |
| nptxr | -0.01464400353 | 0.0605806521 | -0.2417274001 | 0.8089913968 | 0.9854626984 |
| paep | -0.01606594457 | 0.0664881581 | -0.2416361805 | 0.8090620847 | 0.9840624243 |
| mnat1 | 0.0153803788 | 0.06377607422 | 0.2411622068 | 0.8094294011 | 1.015499266 |
| fabp6 | 0.01397082149 | 0.0579374563 | 0.2411362593 | 0.8094495109 | 1.014068869 |
| rab2b | 0.01522678561 | 0.063155083 | 0.2411015058 | 0.8094764457 | 1.015343304 |
| nt5c3a | 0.0138940253 | 0.05786694261 | 0.240102979 | 0.8102504249 | 1.013990996 |
| rangap1 | -0.01415106606 | 0.05911738154 | -0.2393723417 | 0.8108168748 | 0.9859485896 |
| ntf4 | 0.01393821141 | 0.05830706277 | 0.2390484231 | 0.8110680348 | 1.014035801 |
| klk11 | 0.01397371371 | 0.05847955021 | 0.2389504307 | 0.81114402 | 1.014071802 |
| ccl22 | -0.01441641932 | 0.06053645538 | -0.2381444243 | 0.81176908 | 0.9856869997 |
| gbp6 | 0.01508114404 | 0.06333199318 | 0.2381283659 | 0.8117815346 | 1.015195438 |
| fstl3 | -0.01602567302 | 0.06736069906 | -0.2379083538 | 0.8119521761 | 0.9841020549 |
| krt6c | -0.01541941943 | 0.06486188909 | -0.2377269557 | 0.8120928753 | 0.9846988511 |
| rpa2 | 0.0152509938 | 0.06417617179 | 0.237642623 | 0.812158289 | 1.015367884 |
| plin3 | 0.01422440816 | 0.05986128485 | 0.2376228341 | 0.8121736386 | 1.014326056 |
| prkg1 | 0.0148180308 | 0.06243979342 | 0.2373171016 | 0.8124107959 | 1.014928362 |
| clec5a | 0.01438472614 | 0.0606712 | 0.2370931536 | 0.8125845236 | 1.014488684 |
| dkk1 | 0.01387552362 | 0.05858478492 | 0.2368451747 | 0.8127769041 | 1.013972235 |
| ogt | 0.01496407928 | 0.06324597982 | 0.236601272 | 0.8129661333 | 1.015076602 |
| arf6 | 0.01483372052 | 0.0629567381 | 0.2356176792 | 0.8137293536 | 1.014944286 |
| serpinb8 | -0.01411188993 | 0.05990242891 | -0.2355812642 | 0.8137576133 | 0.985987216 |
| coch | 0.01540842731 | 0.06549555949 | 0.2352591141 | 0.8140076268 | 1.015527749 |
| alpi | 0.01507929205 | 0.06411223294 | 0.2352014796 | 0.8140523577 | 1.015193558 |
| lilrb4 | 0.01488490625 | 0.06330308525 | 0.2351371373 | 0.8141022952 | 1.014996238 |
| rpgr | 0.01488720915 | 0.06341616499 | 0.2347541695 | 0.8143995411 | 1.014998576 |
| clec4c | 0.01354887098 | 0.05781108878 | 0.2343645702 | 0.8147019616 | 1.013641073 |
| ndufb7 | -0.01466050652 | 0.06257317012 | -0.2342938115 | 0.81475689 | 0.9854464355 |
| rpl14 | 0.01541002906 | 0.06583177343 | 0.2340819373 | 0.8149213683 | 1.015529376 |
| htra2 | -0.01353652194 | 0.0579124434 | -0.2337411642 | 0.8151859284 | 0.9865546848 |
| gopc | 0.01354873768 | 0.05804552986 | 0.2334156948 | 0.815438627 | 1.013640938 |
| cep43 | 0.01383003439 | 0.0593501553 | 0.233024401 | 0.815742458 | 1.013926112 |
| mfap4 | -0.01507911821 | 0.06476789876 | -0.232817777 | 0.8159029082 | 0.9850340024 |
| snx5 | 0.01499588431 | 0.06446481431 | 0.2326212286 | 0.8160555414 | 1.015108887 |
| c9 | 0.01462443391 | 0.06297434853 | 0.2322284272 | 0.8163605995 | 1.014731894 |
| tfrc | 0.01394789168 | 0.0600990905 | 0.2320815766 | 0.816474654 | 1.014045617 |
| sell | 0.01483752574 | 0.06397594734 | 0.2319235019 | 0.8165974303 | 1.014948148 |
| serpina7 | 0.0147321637 | 0.06359645954 | 0.2316506895 | 0.8168093336 | 1.014841217 |
| snap29 | 0.01337136441 | 0.05772391006 | 0.2316434282 | 0.816814974 | 1.013461161 |
| ca13 | -0.01355781635 | 0.05853583699 | -0.2316156572 | 0.8168365455 | 0.9865336769 |
| trim5 | 0.01349122627 | 0.05825682617 | 0.2315818962 | 0.81686277 | 1.013582644 |
| gask1a | -0.01543973169 | 0.06668298105 | -0.2315393141 | 0.8168958469 | 0.9846788499 |
| acsl1 | -0.01471874235 | 0.06357202609 | -0.2315286024 | 0.8169041675 | 0.9853890488 |
| irak1 | -0.01374680824 | 0.05939055928 | -0.2314645359 | 0.8169539337 | 0.9863472476 |
| eng | -0.01342345612 | 0.0581677325 | -0.2307715213 | 0.8174923068 | 0.9866662367 |

| Protein | beta | se | z | p_val | HR |
|---|---|---|---|---|---|
| ppie | 0.01452295792 | 0.0629466281 | 0.2307186001 | 0.8175334225 | 1.014628928 |
| atp6ap2 | -0.01366830112 | 0.05946339629 | -0.2298607543 | 0.8181999733 | 0.986424686 |
| cd22 | 0.01365392907 | 0.05952077027 | 0.2293977213 | 0.8185598071 | 1.01374757 |
| znrd2 | 0.0147403144 | 0.06459149674 | 0.2282082804 | 0.8194843245 | 1.014849489 |
| pkd2 | 0.01503930661 | 0.0659095485 | 0.2281809989 | 0.8195055325 | 1.015152966 |
| lpcat2 | 0.01355789113 | 0.05955524793 | 0.2276523329 | 0.8199165322 | 1.013650216 |
| il4 | -0.01316602074 | 0.05787683558 | -0.2274834242 | 0.820047857 | 0.9869202722 |
| fbp1 | 0.01398939501 | 0.06155448522 | 0.2272684917 | 0.8202149721 | 1.014087704 |
| rnase4 | 0.01578704892 | 0.06951974535 | 0.2270872662 | 0.8203558855 | 1.015912323 |
| ptp4a3 | -0.0153115966 | 0.0676000196 | -0.2265028426 | 0.8208103486 | 0.9848050299 |
| hpse | -0.01435072472 | 0.06361510729 | -0.2255867408 | 0.8215228543 | 0.9857517561 |
| kcnip4 | -0.01297181062 | 0.05801727164 | -0.2235853265 | 0.8230799824 | 0.9871119607 |
| rbm17 | 0.01404513114 | 0.06284117397 | 0.2235020489 | 0.8231447887 | 1.014144227 |
| padi4 | 0.0130116496 | 0.05825791583 | 0.2233456075 | 0.8232665339 | 1.013096669 |
| mcemp1 | 0.0145005027 | 0.06492748343 | 0.2233338169 | 0.8232757098 | 1.014606145 |
| prkar1a | -0.01307408748 | 0.05854622296 | -0.2233122278 | 0.8232925112 | 0.9870110072 |
| hs6st2 | 0.01498786409 | 0.06721463944 | 0.2229851147 | 0.8235470916 | 1.015100745 |
| epha2 | 0.01394297217 | 0.06253996513 | 0.2229449943 | 0.8235783171 | 1.014040629 |
| spink5 | 0.01301399456 | 0.05849887614 | 0.2224657194 | 0.8239513566 | 1.013099045 |
| uros | 0.01409327126 | 0.06336028791 | 0.2224306694 | 0.823978639 | 1.01419305 |
| gbp4 | -0.01290150101 | 0.0580841962 | -0.2221172342 | 0.824222622 | 0.9871813666 |
| cracr2a | 0.01298950577 | 0.05848834555 | 0.2220870783 | 0.8242460968 | 1.013074236 |
| samd9l | 0.01301305427 | 0.05879661309 | 0.221323195 | 0.8248407908 | 1.013098093 |
| sil1 | -0.01427613899 | 0.06456612474 | -0.2211088097 | 0.8250077109 | 0.9858252819 |
| fcrl1 | 0.01300134698 | 0.0590150718 | 0.2203055353 | 0.8256332096 | 1.013086232 |
| il5 | 0.01283684647 | 0.05842849556 | 0.2197018141 | 0.8261033918 | 1.012919592 |
| ccl26 | 0.01281432207 | 0.05836804435 | 0.2195434543 | 0.8262267338 | 1.012896777 |
| pecam1 | 0.01289689081 | 0.05883435172 | 0.2192068143 | 0.8264889475 | 1.012980414 |
| ern1 | 0.01437878626 | 0.06563858878 | 0.2190599543 | 0.8266033449 | 1.014482658 |
| il3 | 0.0139313828 | 0.063624996 | 0.2189608436 | 0.8266805499 | 1.014028877 |
| metap1d | 0.01270678394 | 0.05806894911 | 0.2188223505 | 0.8267884356 | 1.012787858 |
| max | 0.01264717003 | 0.05790653903 | 0.2184065953 | 0.8271123273 | 1.012727484 |
| ccn1 | -0.01296555923 | 0.05945350683 | -0.218078965 | 0.8273675865 | 0.9871181315 |
| cxadr | -0.01293716063 | 0.05932660099 | -0.2180667764 | 0.8273770831 | 0.9871461647 |
| vsig2 | 0.01412975003 | 0.06482496491 | 0.2179677236 | 0.8274542596 | 1.014230047 |
| mtdh | -0.01406560153 | 0.0645926083 | -0.2177586864 | 0.8276171356 | 0.9860328569 |
| sat1 | 0.01371103716 | 0.06307782189 | 0.217367004 | 0.8279223434 | 1.013805465 |
| copb2 | -0.01372512827 | 0.06322741467 | -0.2170755889 | 0.8281494377 | 0.9863686319 |
| scn4b | -0.01399671232 | 0.06467289764 | -0.2164231514 | 0.8286579216 | 0.9861007862 |
| tsc22d1 | -0.01370371916 | 0.06372273279 | -0.2150522828 | 0.8297265562 | 0.9863897494 |
| stx6 | 0.01265483108 | 0.05902135639 | 0.2144110515 | 0.8302265242 | 1.012735242 |
| hmcn2 | 0.01364998357 | 0.06370641952 | 0.2142638634 | 0.8303412964 | 1.01374357 |
| hnmt | 0.01374982889 | 0.06426565907 | 0.2139529741 | 0.8305837292 | 1.013844793 |
| clip2 | 0.01237942318 | 0.05796909885 | 0.2135521066 | 0.8308963512 | 1.012456365 |
| eif2ak2 | 0.01344713271 | 0.06301299978 | 0.2134025163 | 0.831013018 | 1.013537952 |
| notch2 | -0.01366940347 | 0.06451237147 | -0.2118880946 | 0.8321943389 | 0.9864235986 |
| gne | 0.01227467191 | 0.05799321881 | 0.2116570205 | 0.832374621 | 1.012350315 |
| adm | -0.01492075647 | 0.07055880675 | -0.2114655442 | 0.832524016 | 0.9851900064 |
| fabp1 | 0.01332669196 | 0.06303172779 | 0.2114283143 | 0.8325530645 | 1.013415888 |
| doc2b | -0.01346406239 | 0.06372951038 | -0.2112688818 | 0.8326774636 | 0.9866261727 |
| rarres1 | 0.01247296113 | 0.05924432013 | 0.2105342942 | 0.8332506885 | 1.012551073 |
| dars1 | 0.01230838162 | 0.05848811539 | 0.2104424383 | 0.8333223732 | 1.012384441 |
| rbp5 | 0.01384025983 | 0.06588138824 | 0.2100784486 | 0.8336064457 | 1.01393648 |
| clec1b | -0.01210083538 | 0.05769258238 | -0.2097468145 | 0.8338652854 | 0.9879720853 |
| kiaa1549l | 0.01335155165 | 0.06367060409 | 0.2096972668 | 0.8339039589 | 1.013441082 |
| serpinb5 | -0.01231583635 | 0.05884277416 | -0.209300743 | 0.8342134718 | 0.9877596932 |
| ralb | 0.01339747252 | 0.06430115893 | 0.2083550708 | 0.8349517347 | 1.013487621 |
| ica1 | -0.01228946337 | 0.0590015423 | -0.2082905444 | 0.8350021142 | 0.9877857437 |
| cd40 | -0.01236461868 | 0.05977628081 | -0.2068482433 | 0.8361283781 | 0.9877115091 |
| dlk1 | 0.01257453504 | 0.0608150934 | 0.2067666814 | 0.8361920783 | 1.012653927 |
| c1ql2 | -0.0137761553 | 0.06663228312 | -0.2067489609 | 0.8362059181 | 0.9863183017 |
| msra | 0.01204801455 | 0.05856909116 | 0.2057060185 | 0.8370205571 | 1.012120884 |
| polr2a | 0.01323663046 | 0.06447949843 | 0.2052843273 | 0.8373499883 | 1.013324622 |
| ism2 | 0.01282085027 | 0.06259308005 | 0.204828557 | 0.8377060747 | 1.01290339 |
| timm8a | 0.01281976954 | 0.06261164559 | 0.2047505607 | 0.8377670154 | 1.012902295 |
| stk24 | -0.01197222094 | 0.05847626416 | -0.2047364194 | 0.8377780645 | 0.9880991609 |
| mst1 | 0.0127466324 | 0.0622597372 | 0.2047331545 | 0.8377806155 | 1.012828217 |
| pdlim5 | 0.01293431986 | 0.06322024009 | 0.2045914385 | 0.837891345 | 1.01301833 |
| serpina12 | -0.01260474731 | 0.06173131294 | -0.2041872545 | 0.838207171 | 0.9874743598 |
| unc79 | 0.01296769428 | 0.0636794068 | 0.2036403121 | 0.8386345887 | 1.013052139 |

| Protein | beta | se | z | p_val | HR |
|---|---|---|---|---|---|
| tbl1x | 0.01190605517 | 0.05848939304 | 0.2035592191 | 0.8386979643 | 1.011977214 |
| mpig6b | -0.01172096583 | 0.05759128096 | -0.2035197973 | 0.8387287735 | 0.9883474571 |
| casp3 | -0.01181210476 | 0.05808901778 | -0.203344887 | 0.8388654736 | 0.9882573843 |
| tgfbi | 0.01205459755 | 0.05936921576 | 0.2030445812 | 0.8391001871 | 1.012127547 |
| pcare | -0.01281936926 | 0.06317368755 | -0.2029226052 | 0.8391955254 | 0.9872624489 |
| sdhb | 0.01277913672 | 0.06308411547 | 0.2025729714 | 0.8394688176 | 1.012861139 |
| ncln | -0.01187934516 | 0.05865629892 | -0.2025246287 | 0.8395066064 | 0.9881909357 |
| acaa1 | -0.01211181328 | 0.06035619693 | -0.2006722407 | 0.8409548669 | 0.9879612395 |
| cebpb | 0.01201810418 | 0.05990428578 | 0.2006217755 | 0.8409943298 | 1.012090612 |
| pla2g4a | -0.01163583255 | 0.05801085692 | -0.2005802563 | 0.8410267975 | 0.9884316019 |
| retn | 0.01173692786 | 0.05885374889 | 0.1994253227 | 0.8419300537 | 1.011806076 |
| spred2 | -0.01284977724 | 0.06472351218 | -0.1985333739 | 0.8426277758 | 0.9872324287 |
| ppp1r2 | -0.0116692809 | 0.05883194986 | -0.198349382 | 0.8427717178 | 0.9883985411 |
| sugp1 | -0.01262898976 | 0.06369452976 | -0.198274323 | 0.8428304402 | 0.9874504213 |
| acrbp | 0.0127166844 | 0.06420003151 | 0.1980790991 | 0.8429831774 | 1.012797885 |
| agt | 0.01271516493 | 0.06431457657 | 0.1977026921 | 0.8432776833 | 1.012796346 |
| mesd | -0.01145854093 | 0.05801126131 | -0.1975226994 | 0.8434185197 | 0.9886068581 |
| vps37a | 0.0114536095 | 0.05806952118 | 0.1972396064 | 0.8436400379 | 1.011519453 |
| idi2 | -0.01190750378 | 0.06037384669 | -0.197229503 | 0.843647944 | 0.98816311 |
| mydgf | 0.01262191104 | 0.06414130373 | 0.1967828889 | 0.843997442 | 1.012701904 |
| aldh2 | -0.01249035178 | 0.06356064223 | -0.1965107863 | 0.844210391 | 0.9875873289 |
| dctn1 | 0.01132110461 | 0.05767651967 | 0.1962861954 | 0.8443861657 | 1.011385431 |
| atp5f1d | -0.01266621917 | 0.06454466561 | -0.196239597 | 0.8444226366 | 0.9874136598 |
| amdhd2 | 0.01211588837 | 0.06178508122 | 0.1960973123 | 0.8445339999 | 1.012189583 |
| dusp29 | -0.01290929426 | 0.06597257016 | -0.1956766915 | 0.8448632292 | 0.9871736733 |
| col1a1 | 0.0113986716 | 0.05831162915 | 0.1954785309 | 0.8450183434 | 1.011463884 |
| dbnl | 0.01134263737 | 0.05805577763 | 0.1953748246 | 0.8450995238 | 1.011407209 |
| ly75 | 0.01130323023 | 0.05789463824 | 0.1952379456 | 0.8452066743 | 1.011367353 |
| smndc1 | 0.01265115194 | 0.06491756186 | 0.1948802693 | 0.8454866809 | 1.012731516 |
| ranbp2 | -0.01234448366 | 0.06338932907 | -0.1947407212 | 0.8455959313 | 0.9877313969 |
| usp8 | 0.01167562901 | 0.05998526591 | 0.1946416147 | 0.8456735223 | 1.011744055 |
| lrrc38 | -0.01263469896 | 0.06505190649 | -0.1942248834 | 0.8459997997 | 0.9874447838 |
| itpa | -0.01241193613 | 0.06460528359 | -0.192119521 | 0.8476485842 | 0.9876647742 |
| sost | -0.01221433928 | 0.0636743305 | -0.1918251701 | 0.8478791542 | 0.987859953 |
| sag | -0.01232535433 | 0.06440231586 | -0.1913806075 | 0.8482274123 | 0.9877502917 |
| tgoln2 | 0.01204027082 | 0.06300254263 | 0.1911076969 | 0.8484412175 | 1.012113047 |
| atox1 | 0.011272265 | 0.05899240865 | 0.1910799246 | 0.8484629757 | 1.011336036 |
| trim26 | 0.01211734795 | 0.06363518175 | 0.1904190044 | 0.8489808067 | 1.01219106 |
| gad2 | -0.01213688998 | 0.06420074756 | -0.1890459293 | 0.8500568194 | 0.987936465 |
| cc2d1a | 0.01133251533 | 0.05998637206 | 0.1889181649 | 0.8501569564 | 1.011396972 |
| fkbpl | 0.01206257417 | 0.06394726365 | 0.1886331562 | 0.8503803444 | 1.01213562 |
| tacc3 | -0.01094530603 | 0.05812279674 | -0.1883134784 | 0.8506309201 | 0.9891143759 |
| gatd3 | 0.01208952972 | 0.06420584665 | 0.188293284 | 0.8506467498 | 1.012162903 |
| ppp1r9b | -0.01082677148 | 0.05771870166 | -0.1875782228 | 0.8512072989 | 0.9892316271 |
| ctsz | 0.0113907147 | 0.06077045497 | 0.1874383646 | 0.851316945 | 1.011455836 |
| chrdl1 | 0.01231854657 | 0.06574804209 | 0.1873598997 | 0.8513784612 | 1.012394732 |
| clstn1 | 0.01086347927 | 0.05804701368 | 0.1871496667 | 0.8515432876 | 1.010922701 |
| sez6 | 0.012004732 | 0.06504953657 | 0.1845475407 | 0.8535839354 | 1.012077078 |
| ppp1r14a | 0.01152178076 | 0.06243344666 | 0.1845450055 | 0.8535859241 | 1.011588412 |
| cd5 | -0.01083080197 | 0.05873693564 | -0.1843950804 | 0.8537035289 | 0.98922764 |
| ccl14 | 0.01104146991 | 0.05999004591 | 0.1840550336 | 0.8539702817 | 1.011102652 |
| shc1 | -0.01184199485 | 0.06443172532 | -0.1837913666 | 0.8541771292 | 0.9882278456 |
| cplx2 | 0.01172215463 | 0.06379688465 | 0.1837418033 | 0.8542160129 | 1.011791128 |
| gnas | -0.01155625905 | 0.0628971856 | -0.1837325301 | 0.8542232879 | 0.988510258 |
| nek7 | 0.01151160614 | 0.06280748276 | 0.1832839915 | 0.8545751946 | 1.01157812 |
| brdt | 0.01165009958 | 0.06376139223 | 0.1827140088 | 0.8550224234 | 1.011718226 |
| cope | 0.0104974921 | 0.05767638928 | 0.1820067489 | 0.8555774295 | 1.010552784 |
| arid4b | -0.01055791414 | 0.05801284468 | -0.1819926983 | 0.8555884562 | 0.989497625 |
| khdc3l | -0.01153502094 | 0.06339070731 | -0.1819670648 | 0.8556085728 | 0.9885312523 |
| hla_dra | 0.01065288935 | 0.05854933257 | 0.181947238 | 0.8556241326 | 1.010709833 |
| ca8 | 0.01162364531 | 0.06401817081 | 0.1815679074 | 0.8559218363 | 1.011691462 |
| scgb2a2 | -0.01145574937 | 0.06318678887 | -0.1812997555 | 0.8561322979 | 0.9886096179 |
| fzd10 | -0.01157040553 | 0.06395883514 | -0.180903944 | 0.8564429731 | 0.9884962742 |
| ager | 0.01075486614 | 0.05961897071 | 0.1803933549 | 0.8568437708 | 1.010812908 |
| mep1b | -0.01062213886 | 0.05894992318 | -0.1801891893 | 0.8570040452 | 0.9894340768 |
| ugdh | -0.01149981796 | 0.06401063551 | -0.1796548006 | 0.8574235799 | 0.9885660522 |
| atp2b4 | 0.01149312277 | 0.06402393179 | 0.1795129172 | 0.8575349756 | 1.011559422 |
| pdcd1 | 0.0103619187 | 0.0579227684 | 0.1788919796 | 0.8580225205 | 1.010415789 |
| inpp1 | 0.01067980206 | 0.059747128 | 0.178750049 | 0.8581339685 | 1.010737035 |
| nudc | 0.01041661906 | 0.05838527352 | 0.1784117541 | 0.8583996189 | 1.010471061 |

| Protein | beta | se | z | p_val | HR |
|---|---|---|---|---|---|
| cnst | -0.01034900914 | 0.05808785766 | -0.1781613156 | 0.8585962893 | 0.9897043576 |
| ermap | -0.01225128863 | 0.06888132036 | -0.1778608273 | 0.8588322755 | 0.9878234529 |
| ckap4 | -0.01089622355 | 0.06134762704 | -0.1776144258 | 0.8590257945 | 0.9891629253 |
| scp2 | -0.01045953045 | 0.05894615238 | -0.1774421236 | 0.8591611224 | 0.9895949802 |
| pcdhb15 | -0.01119804057 | 0.06330605279 | -0.1768873604 | 0.859596867 | 0.9888644241 |
| npc2 | -0.01214299033 | 0.06915240526 | -0.1755975122 | 0.8606101569 | 0.9879304383 |
| ctsd | 0.0116692736 | 0.06648244124 | 0.1755241441 | 0.860667801 | 1.011737625 |
| cdh4 | -0.0109487151 | 0.06278881447 | -0.1743736555 | 0.861571817 | 0.9891110039 |
| serpinb6 | 0.01024091761 | 0.05889553318 | 0.1738827558 | 0.8619576051 | 1.010293535 |
| col5a1 | -0.01088120253 | 0.06274567626 | -0.1734175673 | 0.8623232176 | 0.9891777836 |
| fhit | 0.01010262387 | 0.05837443506 | 0.1730658955 | 0.8625996319 | 1.010153828 |
| slamf7 | 0.01004821162 | 0.05807366575 | 0.1730252687 | 0.8626315656 | 1.010098864 |
| clec6a | -0.0103576157 | 0.05986461319 | -0.1730173327 | 0.8626378036 | 0.9896958397 |
| chp1 | 0.01095219462 | 0.06338851584 | 0.172778846 | 0.8628252654 | 1.011012389 |
| tcn2 | -0.01008102479 | 0.05865574723 | -0.171867639 | 0.8635415883 | 0.9899696184 |
| cd27 | -0.01025411437 | 0.05971838751 | -0.171707824 | 0.8636672345 | 0.9897982798 |
| acot13 | 0.01067793906 | 0.06245911931 | 0.1709588476 | 0.864256124 | 1.010735152 |
| cenpj | -0.01084902732 | 0.06369439669 | -0.1703293835 | 0.8647511042 | 0.9892096111 |
| tnfrsf10b | 0.01135125357 | 0.06694571638 | 0.1695590724 | 0.8653569119 | 1.011415924 |
| tia1 | -0.01005816169 | 0.05932555492 | -0.1695418054 | 0.8653704923 | 0.9899922525 |
| cdh3 | 0.009769518108 | 0.05797356484 | 0.1685167737 | 0.8661767489 | 1.009817396 |
| txndc9 | -0.01060540017 | 0.06312405415 | -0.1680088567 | 0.8665763115 | 0.9894506388 |
| ptn | -0.01055634432 | 0.06300002183 | -0.1675609628 | 0.8669286841 | 0.9894991783 |
| tomm20 | 0.0107084977 | 0.0639999432 | 0.1673204251 | 0.8671179338 | 1.010766039 |
| ank2 | -0.0105653138 | 0.06324084194 | -0.167064724 | 0.8673191221 | 0.9894903031 |
| fgf2 | 0.009645964169 | 0.05782690298 | 0.1668075527 | 0.8675214757 | 1.009692636 |
| nell1 | 0.009559907871 | 0.05733534443 | 0.1667367305 | 0.8675772033 | 1.00960575 |
| lgmn | -0.00984770902 | 0.05951105067 | -0.1654769813 | 0.8685685663 | 0.9902006209 |
| tnfsf13b | 0.009930764757 | 0.0602287301 | 0.1648841797 | 0.8690351447 | 1.009980238 |
| izumo1 | 0.01052036526 | 0.06396215397 | 0.1644779703 | 0.8693548877 | 1.010575899 |
| ldlr | -0.01032749622 | 0.06316917008 | -0.163489503 | 0.8701330373 | 0.9897256493 |
| snap23 | -0.009549481876 | 0.05851699483 | -0.1631915977 | 0.8703675816 | 0.9904959696 |
| prg2 | -0.0102375395 | 0.06277644674 | -0.1630793081 | 0.8704559914 | 0.9898146857 |
| id4 | -0.01021996744 | 0.0626699566 | -0.1630760254 | 0.870458576 | 0.989832079 |
| lypd3 | 0.009604371297 | 0.05890936921 | 0.1630363969 | 0.8704897775 | 1.009650641 |
| src | 0.009464718127 | 0.05814322396 | 0.1627828229 | 0.8706894332 | 1.00950965 |
| rspo1 | -0.01004390397 | 0.06177311326 | -0.1625934559 | 0.8708385398 | 0.9900063676 |
| pla2g1b | 0.009501084142 | 0.05843916946 | 0.1625807524 | 0.8708485427 | 1.009546363 |
| cdc26 | 0.01026553247 | 0.06318251001 | 0.1624742744 | 0.8709323851 | 1.010318404 |
| apob | -0.01007300887 | 0.06230465846 | -0.1616734466 | 0.8715630159 | 0.989977554 |
| cdc42bpb | 0.01006251335 | 0.06249182818 | 0.161021267 | 0.8720766506 | 1.010113311 |
| ces3 | -0.009571830777 | 0.05949900008 | -0.160873809 | 0.872192791 | 0.9904738334 |
| elob | 0.01026876397 | 0.06431183877 | 0.159671441 | 0.873139899 | 1.010321669 |
| arhgap45 | 0.0100305673 | 0.06283221937 | 0.1596405061 | 0.8731642689 | 1.010081042 |
| micb_mica | -0.009059163942 | 0.05694899983 | -0.1590750315 | 0.8736097597 | 0.9909817467 |
| l3hypdh | 0.01002661318 | 0.06316862193 | 0.1587277491 | 0.8738833747 | 1.010077048 |
| psmc3 | -0.009979174689 | 0.06322244596 | -0.1578422748 | 0.8745810857 | 0.9900704521 |
| atxn10 | 0.009112330645 | 0.05783723426 | 0.1575512862 | 0.874810392 | 1.009153974 |
| gas2 | 0.01009257586 | 0.06421868952 | 0.1571594801 | 0.8751191616 | 1.010143678 |
| nfat5 | -0.01001908673 | 0.06385740367 | -0.1568978091 | 0.8753253866 | 0.9900309371 |
| cd163 | -0.009487410625 | 0.06050696808 | -0.1567986453 | 0.8754035406 | 0.9905574529 |
| cd4 | 0.009529560228 | 0.06102034306 | 0.1561702172 | 0.8758988519 | 1.009575111 |
| nhlrc3 | -0.01064901501 | 0.06864824005 | -0.1551243703 | 0.87672327 | 0.989407485 |
| calcoco1 | -0.008899933305 | 0.05745208442 | -0.154910538 | 0.8768918458 | 0.9911395539 |
| nfx1 | -0.009874691871 | 0.06377282449 | -0.1548416892 | 0.8769461243 | 0.9901739028 |
| epb41l5 | 0.009774000554 | 0.06325515411 | 0.1545170618 | 0.8772020594 | 1.009821922 |
| atf2 | 0.009061559258 | 0.0586539749 | 0.1544918187 | 0.8772219615 | 1.009102739 |
| rab11fip3 | 0.009719249356 | 0.06304247343 | 0.1541698608 | 0.8774758056 | 1.009766635 |
| cpxm1 | -0.008845556765 | 0.05758381808 | -0.153611849 | 0.8779157937 | 0.9911934501 |
| tp53 | 0.009131213111 | 0.0594882905 | 0.1534959743 | 0.8780071647 | 1.00917303 |
| gigyf2 | 0.009726488376 | 0.06350116788 | 0.1531702282 | 0.878264035 | 1.009773944 |
| ehbp1 | 0.009718825108 | 0.0635143807 | 0.1530177103 | 0.8783843089 | 1.009766206 |
| serpinb9 | 0.008911979896 | 0.0583824623 | 0.1526482362 | 0.8786756837 | 1.00895181 |
| efna1 | -0.009045152538 | 0.05942414507 | -0.1522134231 | 0.8790186072 | 0.9909956318 |
| bcl2l11 | 0.008890878899 | 0.05860874312 | 0.1516988494 | 0.8794244647 | 1.00893052 |
| map4k5 | -0.008868137454 | 0.05854441198 | -0.151477095 | 0.8795993779 | 0.9911710685 |
| frzb | -0.009179763348 | 0.06065012355 | -0.1513560536 | 0.8796948542 | 0.990862242 |
| bcl7b | -0.009585073811 | 0.06369054477 | -0.1504944548 | 0.8803745254 | 0.9904607166 |
| ptgds | 0.009268713576 | 0.06168032538 | 0.1502701797 | 0.880551459 | 1.009311801 |
| kyat1 | -0.00882381133 | 0.0587863041 | -0.1500997803 | 0.8806858933 | 0.9912150042 |

| Protein | beta | se | z | p_val | HR |
|---|---|---|---|---|---|
| hspg2 | -0.009578594811 | 0.06395347534 | -0.1497744221 | 0.8809425899 | 0.9904671338 |
| spp1 | -0.009037932424 | 0.06038812993 | -0.1496640554 | 0.8810296683 | 0.9910027869 |
| lypla2 | -0.009449737279 | 0.06318159489 | -0.1495647157 | 0.8811080478 | 0.9905947712 |
| esam | 0.008914260981 | 0.05984926276 | 0.1489452095 | 0.881596867 | 1.008954111 |
| gpi | 0.009412355184 | 0.06343932431 | 0.1483678347 | 0.8820524833 | 1.009456791 |
| clgn | -0.009492386349 | 0.06410860964 | -0.1480672628 | 0.8822896852 | 0.9905525241 |
| tnfrsf14 | 0.00895424503 | 0.06094763426 | 0.1469170237 | 0.8831975147 | 1.008994454 |
| krt5 | 0.009037877013 | 0.06152697849 | 0.1468929116 | 0.883216547 | 1.009078842 |
| pdzk1 | -0.009962848434 | 0.06802716138 | -0.1464539786 | 0.8835630179 | 0.9900866163 |
| hddc2 | 0.00915135552 | 0.06279925563 | 0.1457239489 | 0.8841393147 | 1.009193357 |
| fcn2 | 0.008665932123 | 0.05973430638 | 0.1450746254 | 0.8846519523 | 1.00870359 |
| ccl11 | 0.009044230153 | 0.06253982974 | 0.1446155225 | 0.8850144409 | 1.009085253 |
| il17d | 0.009297212678 | 0.06464228986 | 0.1438255467 | 0.8856382293 | 1.009340566 |
| serpina4 | -0.009224664577 | 0.06415346983 | -0.1437905791 | 0.8856658424 | 0.9908177521 |
| ido1 | -0.009369112175 | 0.06523848103 | -0.143613279 | 0.8858058543 | 0.9906746412 |
| nfu1 | 0.008967688757 | 0.06248235396 | 0.1435235421 | 0.8858767198 | 1.009008019 |
| ppp3r1 | -0.008652652369 | 0.06047289765 | -0.1430831448 | 0.8862245165 | 0.9913846741 |
| comt | -0.008604038352 | 0.06018396929 | -0.1429622947 | 0.8863199597 | 0.9914328705 |
| odam | -0.008132642266 | 0.05707598167 | -0.1424879963 | 0.8866945602 | 0.9919003382 |
| ceacam5 | 0.008627137732 | 0.06071508802 | 0.1420921556 | 0.8870072143 | 1.008664459 |
| psma1 | -0.008316993345 | 0.05877159922 | -0.1415138171 | 0.8874640454 | 0.9917174972 |
| cdc27 | 0.008314724574 | 0.05879130741 | 0.1414277882 | 0.8875320031 | 1.008349388 |
| tnfrsf21 | 0.008239916819 | 0.05858108459 | 0.1406583179 | 0.8881398751 | 1.008273958 |
| ltbr | -0.008468771122 | 0.06023293544 | -0.1406003387 | 0.8881856807 | 0.9915669879 |
| grn | -0.008344821807 | 0.05967145594 | -0.1398461237 | 0.8887815699 | 0.9916898996 |
| lsp1 | 0.008478377587 | 0.06069828665 | 0.139680674 | 0.8889122966 | 1.008514421 |
| arnt | -0.00819795976 | 0.05892479872 | -0.1391258 | 0.8893507411 | 0.9918355519 |
| npdc1 | 0.0084834734 | 0.06100068362 | 0.1390717759 | 0.8893934311 | 1.00851956 |
| scarb1 | 0.008072722968 | 0.05823390066 | 0.1386258327 | 0.8897458289 | 1.008105395 |
| rbpms | 0.009016215139 | 0.06523734413 | 0.1382063488 | 0.8900773376 | 1.009056984 |
| sytl4 | -0.008751264113 | 0.06335196324 | -0.1381372205 | 0.8901319701 | 0.9912869167 |
| gfra2 | -0.008007000981 | 0.05821850677 | -0.1375336027 | 0.890609034 | 0.9920249697 |
| vsig10 | 0.008820060373 | 0.06417302847 | 0.1374418597 | 0.8906815458 | 1.008859072 |
| cbx2 | -0.008692374842 | 0.06372748792 | -0.1363991446 | 0.8915057498 | 0.9913452946 |
| cdc37 | 0.007886014443 | 0.05794514611 | 0.1360944785 | 0.8917465924 | 1.007917191 |
| atp6v1g2 | 0.008718702959 | 0.06411183801 | 0.1359920918 | 0.8918275327 | 1.008756822 |
| pbxip1 | 0.008815025925 | 0.06511548443 | 0.1353752645 | 0.8923151802 | 1.008853993 |
| tspan1 | 0.008612876197 | 0.0636624169 | 0.1352898086 | 0.8923827426 | 1.008650074 |
| lonp1 | 0.008565586311 | 0.06374150559 | 0.1343800438 | 0.8931020613 | 1.008602376 |
| plxnb3 | 0.007912143195 | 0.05898015887 | 0.134149235 | 0.8932845675 | 1.007943527 |
| ctsl | 0.008099516557 | 0.06043393236 | 0.1340226631 | 0.8933846535 | 1.008132406 |
| sarg | 0.008519749704 | 0.06363339101 | 0.1338880353 | 0.8934911115 | 1.008556146 |
| gsr | 0.008569473889 | 0.06426861088 | 0.1333384022 | 0.8939257581 | 1.008606297 |
| letm1 | 0.008594294919 | 0.06476010365 | 0.1327097153 | 0.8944229591 | 1.008631332 |
| spint2 | -0.007919611643 | 0.05968855477 | -0.1326822483 | 0.8944446825 | 0.9921116659 |
| ptk7 | -0.007609743686 | 0.05769960215 | -0.1318855486 | 0.8950748193 | 0.9924191371 |
| bst1 | 0.007724164575 | 0.05857710212 | 0.1318632075 | 0.8950924906 | 1.007754073 |
| ly9 | 0.007726773936 | 0.05871783034 | 0.1315916118 | 0.8953073206 | 1.007756702 |
| armcx2 | 0.008361311162 | 0.06371019329 | 0.1312397708 | 0.8955856355 | 1.008396365 |
| susd1 | -0.007673554056 | 0.0585362085 | -0.1310907258 | 0.8957035376 | 0.9923558125 |
| tab2 | -0.008320215097 | 0.06353833472 | -0.1309479566 | 0.8958164774 | 0.9917143021 |
| xiap | 0.008483132569 | 0.06493147217 | 0.1306474701 | 0.8960541888 | 1.008519216 |
| il1rap | -0.007765730373 | 0.05990184127 | -0.1296409294 | 0.8968505195 | 0.992264345 |
| mavs | 0.007585928057 | 0.05865860687 | 0.1293233587 | 0.8971017891 | 1.007614774 |
| chmp1a | -0.007539125711 | 0.05832731884 | -0.129255482 | 0.8971554961 | 0.9924892222 |
| cdsn | 0.00749628015 | 0.05806479996 | 0.1291019715 | 0.897276962 | 1.007524448 |
| ttr | 0.008576027521 | 0.06648249762 | 0.128996771 | 0.8973602038 | 1.008612907 |
| cep20 | -0.007545184333 | 0.05881489209 | -0.1282869706 | 0.8979218758 | 0.9924832091 |
| csf1 | 0.007951708621 | 0.06205580109 | 0.1281380384 | 0.8980397339 | 1.007983407 |
| sv2a | 0.0080336383 | 0.06274342115 | 0.1280395323 | 0.8981176882 | 1.008065995 |
| tnfrsf6b | -0.007972873083 | 0.06236798412 | -0.1278359914 | 0.8982787667 | 0.992058826 |
| icam3 | -0.007391476455 | 0.05788590315 | -0.1276904402 | 0.8983939558 | 0.9926357733 |
| lyar | -0.007427862589 | 0.05823411485 | -0.1275517385 | 0.8985037261 | 0.9925996558 |
| nmi | -0.008171844145 | 0.06415014058 | -0.1273862235 | 0.8986347195 | 0.9918614546 |
| map1lc3b2 | -0.008101941514 | 0.06360677819 | -0.1273754425 | 0.898643252 | 0.9919307908 |
| rtn4r | -0.008122485985 | 0.06420949991 | -0.1264997546 | 0.8993363433 | 0.9919104123 |
| mink1 | -0.00794833195 | 0.06287382935 | -0.126417176 | 0.8994017068 | 0.9920831725 |
| mdm1 | 0.008063426244 | 0.0638048373 | 0.1263764095 | 0.8994339749 | 1.008096023 |
| dpp6 | 0.007700956837 | 0.06094983346 | 0.1263491038 | 0.8994555884 | 1.007730685 |
| cd2 | -0.008122952077 | 0.06430306327 | -0.1263229411 | 0.8994762973 | 0.99190995 |

| Protein | beta | se | z | p_val | HR |
|---|---|---|---|---|---|
| nphs2 | -0.00799389245 | 0.06334574315 | -0.1261946273 | 0.8995778641 | 0.9920379737 |
| gzmb | -0.007501006932 | 0.0597419302 | -0.1255568226 | 0.900082743 | 0.9925270554 |
| got1 | 0.008059575072 | 0.06420972612 | 0.1255195367 | 0.9001122593 | 1.008092141 |
| c1qtnf6 | -0.007953640534 | 0.0635386304 | -0.1251780292 | 0.9003826105 | 0.992077906 |
| sftpa2 | -0.00741044333 | 0.05970174922 | -0.1241243921 | 0.9012167854 | 0.9926169463 |
| pgm2 | 0.007848626758 | 0.0634131531 | 0.1237696972 | 0.9014976254 | 1.007879508 |
| rnase3 | 0.007266488218 | 0.05894275397 | 0.1232804328 | 0.901885035 | 1.007292953 |
| fgf16 | 0.007946606695 | 0.06451076297 | 0.1231826494 | 0.9019624647 | 1.007978265 |
| dynlt3 | 0.007816866675 | 0.06357805463 | 0.1229491327 | 0.9021473785 | 1.007847498 |
| mdk | 0.007366544403 | 0.06009744159 | 0.1225766723 | 0.9024423279 | 1.007393744 |
| tacstd2 | 0.007797332034 | 0.06367997706 | 0.1224455849 | 0.9025461386 | 1.00782781 |
| hcls1 | 0.007162829442 | 0.05868631989 | 0.1220527962 | 0.9028572055 | 1.007188544 |
| trdmt1 | -0.007597591113 | 0.06242683252 | -0.1217039341 | 0.9031334974 | 0.9924311976 |
| bex3 | 0.007793635539 | 0.06417324206 | 0.12144681 | 0.9033371422 | 1.007824085 |
| itprip | -0.007816123934 | 0.06440610782 | -0.1213568743 | 0.9034083736 | 0.9922143425 |
| cdon | 0.0073230715 | 0.06037146394 | 0.1213002141 | 0.9034532505 | 1.007349951 |
| ptprf | 0.007240095245 | 0.06025509556 | 0.1201573938 | 0.904358468 | 1.007266368 |
| vcan | -0.007105934581 | 0.0592931255 | -0.119844156 | 0.9046066026 | 0.9929192529 |
| dnajb6 | 0.007557008476 | 0.06315313795 | 0.1196616466 | 0.9047511836 | 1.007585635 |
| clspn | 0.00695338636 | 0.0582226205 | 0.1194275747 | 0.9049366162 | 1.006977617 |
| sparc | 0.006919036667 | 0.05794711367 | 0.1194026109 | 0.9049563929 | 1.006943029 |
| uxs1 | -0.00708511291 | 0.05977742388 | -0.1185248953 | 0.9056517705 | 0.9929399273 |
| ly6d | 0.007064681944 | 0.05967243734 | 0.1183910405 | 0.9057578245 | 1.007089696 |
| tslp | 0.006946825992 | 0.05911694312 | 0.1175098986 | 0.9064560004 | 1.006971011 |
| kif20b | -0.007391225444 | 0.06319294313 | -0.1169628297 | 0.9068895089 | 0.9926360225 |
| il17a | -0.006782584284 | 0.05800177974 | -0.1169375201 | 0.9069095654 | 0.9932403655 |
| terf1 | 0.007518964313 | 0.06454821365 | 0.116486017 | 0.9072673675 | 1.007547303 |
| ca4 | -0.006777647629 | 0.05834040063 | -0.1161741701 | 0.9075145074 | 0.9932452688 |
| epn1 | 0.007292994633 | 0.06280887238 | 0.1161140832 | 0.9075621275 | 1.007319653 |
| mmp10 | -0.006811545638 | 0.05866562528 | -0.1161079526 | 0.9075669862 | 0.9932116004 |
| scg3 | -0.006899587381 | 0.05996212338 | -0.1150657614 | 0.9083929981 | 0.9931241601 |
| sgsh | -0.007522529875 | 0.06540394627 | -0.1150164524 | 0.9084320814 | 0.9925056935 |
| vps53 | 0.006685935911 | 0.05828924901 | 0.1147027286 | 0.9086807511 | 1.006708337 |
| klk8 | 0.007089783713 | 0.06210797492 | 0.11415255 | 0.9091168655 | 1.007114976 |
| hs6st1 | -0.006672937572 | 0.05846115643 | -0.1141430991 | 0.9091243573 | 0.993349277 |
| pear1 | 0.006610848584 | 0.05799084056 | 0.1139981507 | 0.9092392594 | 1.006632748 |
| cd101 | 0.007133179564 | 0.06320057241 | 0.112865743 | 0.9101369959 | 1.007158681 |
| mmp7 | -0.006760986031 | 0.0599883266 | -0.112705028 | 0.9102644149 | 0.993261818 |
| gpkow | 0.006642652656 | 0.05920355919 | 0.1122002249 | 0.9106646508 | 1.006664764 |
| yes1 | -0.00650800686 | 0.05814948048 | -0.1119185727 | 0.9108879702 | 0.9935131244 |
| amotl2 | -0.007135213826 | 0.06438174023 | -0.1108266692 | 0.9117537967 | 0.9928901814 |
| pgr | 0.007092669392 | 0.06407547024 | 0.1106924283 | 0.9118602505 | 1.007117882 |
| tnfsf12 | 0.00653285601 | 0.05913178357 | 0.1104796036 | 0.9120290248 | 1.006554242 |
| layn | 0.006833292523 | 0.06209546389 | 0.1100449549 | 0.9123737225 | 1.006856693 |
| commd1 | 0.007127233174 | 0.06484466708 | 0.1099124029 | 0.912478846 | 1.007152692 |
| inppl1 | 0.006422113205 | 0.05845172261 | 0.1098703839 | 0.9125121705 | 1.006442779 |
| cd3d | 0.006944827145 | 0.06321207157 | 0.1098655205 | 0.9125160276 | 1.006968998 |
| csrp3 | -0.007273473581 | 0.06660586199 | -0.1092017033 | 0.9130425093 | 0.9927529141 |
| gpc5 | 0.006464941304 | 0.05926972648 | 0.1090766178 | 0.9131417205 | 1.006485884 |
| tbcb | -0.006393116264 | 0.05875636794 | -0.1088072066 | 0.9133554077 | 0.9936272762 |
| c1qa | 0.006483190059 | 0.06054777543 | 0.1070756112 | 0.9147289954 | 1.006504251 |
| cmc1 | 0.006749223422 | 0.06324159274 | 0.1067212752 | 0.9150101038 | 1.006772051 |
| islr2 | -0.006254259346 | 0.05875901168 | -0.1064391515 | 0.9152339311 | 0.9937652578 |
| grin2b | -0.006714435901 | 0.06339014871 | -0.1059223876 | 0.9156439313 | 0.9933080556 |
| pzp | 0.009299872214 | 0.08938074468 | 0.1040478265 | 0.9171313945 | 1.00934325 |
| ppp2r5a | -0.006571324515 | 0.06326886815 | -0.1038634751 | 0.9172776929 | 0.9934502194 |
| grap2 | -0.006021158184 | 0.05836687235 | -0.103160542 | 0.9178355557 | 0.9939969327 |
| gipr | 0.006676193898 | 0.06480101561 | 0.1030260689 | 0.9179422811 | 1.006698529 |
| edem2 | -0.006504197238 | 0.06327325528 | -0.1027953629 | 0.9181253856 | 0.9935169093 |
| ikzf2 | 0.006010092685 | 0.05859874372 | 0.1025635074 | 0.9183094069 | 1.00602819 |
| klk14 | 0.006028681723 | 0.05885202528 | 0.1024379653 | 0.9184090502 | 1.006046891 |
| cd33 | -0.005940218834 | 0.05801946074 | -0.1023832135 | 0.9184525074 | 0.9940773894 |
| hepacam2 | 0.006476274513 | 0.06343837986 | 0.1020876404 | 0.9186871113 | 1.006497291 |
| cd86 | 0.006423771471 | 0.06298604298 | 0.1019872208 | 0.9187668185 | 1.006444448 |
| ghrhr | -0.005872646555 | 0.05765737775 | -0.1018542082 | 0.9188723975 | 0.9941445637 |
| stk4 | 0.005932443362 | 0.05849136515 | 0.1014242589 | 0.91921368 | 1.005950075 |
| crybb1 | 0.006476664444 | 0.0641569185 | 0.1009503666 | 0.9195898605 | 1.006497683 |
| nubp1 | 0.006416160792 | 0.06364567972 | 0.1008106256 | 0.9197007918 | 1.006436788 |
| mn1 | -0.006417222177 | 0.06366357172 | -0.1007989656 | 0.919710048 | 0.9936033242 |
| nmt1 | 0.006259128115 | 0.06250354449 | 0.1001403707 | 0.9202328852 | 1.006278757 |

| Protein | beta | se | z | p_val | HR |
|---|---|---|---|---|---|
| pcdh17 | 0.005919884455 | 0.05914771112 | 0.1000864504 | 0.9202756923 | 1.005937442 |
| tgfbr3 | -0.005745719499 | 0.0576598706 | -0.09964849797 | 0.9206233896 | 0.9942707556 |
| vwc2 | 0.006108209994 | 0.06150752895 | 0.0993083302 | 0.9208934646 | 1.006126903 |
| nbn | -0.005880740155 | 0.0596886175 | -0.09852364491 | 0.9215164974 | 0.9941365176 |
| csf2rb | 0.006356730267 | 0.06507562468 | 0.09768220126 | 0.9221846493 | 1.006376977 |
| caps | -0.006511681243 | 0.06728984196 | -0.09677064254 | 0.9229085383 | 0.9935094738 |
| myh9 | -0.005998889207 | 0.06202648599 | -0.09671496154 | 0.9229527579 | 0.9940190682 |
| rab10 | -0.006103464758 | 0.06326665518 | -0.09647206322 | 0.9231456607 | 0.9939151235 |
| bpifb1 | 0.005990713576 | 0.06219175884 | 0.09632648582 | 0.9232612762 | 1.006008694 |
| rab27b | 0.006010524769 | 0.06256096271 | 0.09607468473 | 0.9234612569 | 1.006028624 |
| cxcl1 | -0.005631510952 | 0.05865343754 | -0.09601331461 | 0.9235099979 | 0.9943843163 |
| s100a11 | -0.005852718115 | 0.06153123959 | -0.09511783209 | 0.9242212343 | 0.9941643757 |
| ppp1cc | 0.006049288438 | 0.0636017475 | 0.09511198475 | 0.9242258788 | 1.006067622 |
| dbi | 0.005624825953 | 0.05998918533 | 0.09376399967 | 0.9252966298 | 1.005640675 |
| ncr1 | 0.005460208259 | 0.05830072464 | 0.09365592439 | 0.9253824836 | 1.005475142 |
| pgd | 0.006004750163 | 0.06496309659 | 0.09243325024 | 0.9263538226 | 1.006022815 |
| il17f | 0.005410138928 | 0.05878685779 | 0.09202973474 | 0.9266744149 | 1.0054248 |
| vwa1 | 0.005589255729 | 0.06112275798 | 0.0914431206 | 0.9271405 | 1.005604905 |
| lbp | -0.00542569512 | 0.05955110212 | -0.09110990269 | 0.9274052642 | 0.9945889974 |
| traf3ip2 | -0.005756421095 | 0.06357753769 | -0.09054174327 | 0.9278567238 | 0.9942601154 |
| wdr46 | -0.005772070426 | 0.06377599359 | -0.0905053783 | 0.9278856202 | 0.994244556 |
| fkbp7 | 0.005302853376 | 0.05866480761 | 0.09039241056 | 0.9279753875 | 1.005316938 |
| nrp2 | 0.005506609836 | 0.06092951005 | 0.0903767293 | 0.9279878483 | 1.005521799 |
| tst | -0.00521109726 | 0.05805434494 | -0.08976239875 | 0.928476029 | 0.994802457 |
| dnajc21 | 0.005529968046 | 0.06246617764 | 0.08852739603 | 0.9294575109 | 1.005545287 |
| tpt1 | 0.005033594149 | 0.05855307375 | 0.085966352 | 0.9314931654 | 1.005046284 |
| ipcef1 | -0.005063169114 | 0.05953981471 | -0.0850383754 | 0.932230882 | 0.9949496271 |
| add1 | 0.005319236696 | 0.06271129867 | 0.08482102603 | 0.9324036774 | 1.005333409 |
| klk15 | 0.005313295352 | 0.06304328781 | 0.0842801119 | 0.9328337245 | 1.005327436 |
| frmd4b | 0.005332991581 | 0.06359653961 | 0.08385663142 | 0.9331704212 | 1.005347237 |
| scn3b | -0.005302476904 | 0.06328375835 | -0.08378890639 | 0.9332242684 | 0.9947115564 |
| naa10 | -0.005245988081 | 0.06374253778 | -0.08229964265 | 0.9344084386 | 0.9947677481 |
| golga3 | 0.005164905482 | 0.06301941387 | 0.08195737099 | 0.9346806124 | 1.005178267 |
| mmp15 | 0.005141173699 | 0.06278617194 | 0.08188385341 | 0.9347390744 | 1.005154412 |
| stau1 | 0.005275275386 | 0.06445977974 | 0.08183824716 | 0.9347753412 | 1.005289214 |
| tarm1 | 0.005152631144 | 0.06324228575 | 0.08147446101 | 0.9350646345 | 1.005165929 |
| vsig10l | 0.00516136714 | 0.06335950905 | 0.08146160249 | 0.9350748601 | 1.00517471 |
| smad1 | -0.00474114114 | 0.05905010393 | -0.08029014049 | 0.9360064996 | 0.9952700803 |
| tmco5a | 0.005125232203 | 0.06392721489 | 0.08017293122 | 0.9360997186 | 1.005138389 |
| bank1 | 0.004660532495 | 0.05818445435 | 0.08009927302 | 0.9361583009 | 1.00467141 |
| adamtsl4 | 0.005287442179 | 0.06617014204 | 0.07990676786 | 0.9363114071 | 1.005301445 |
| gata3 | -0.005086383765 | 0.06422633922 | -0.0791946704 | 0.9368777837 | 0.99492653 |
| rabep1 | 0.004949060761 | 0.06251969224 | 0.07916003077 | 0.9369053356 | 1.004961328 |
| hbegf | 0.004610223494 | 0.05827034124 | 0.07911783929 | 0.9369388943 | 1.004620867 |
| sfrp4 | 0.00527001424 | 0.06666175051 | 0.07905604338 | 0.9369880463 | 1.005283925 |
| ap3s2 | 0.004988175248 | 0.06312929052 | 0.07901522743 | 0.9370205112 | 1.005000637 |
| otud7b | 0.005047775082 | 0.06391136368 | 0.07898086963 | 0.9370478394 | 1.005060537 |
| tnfrsf9 | 0.004818700124 | 0.06101973774 | 0.07896953187 | 0.9370568574 | 1.004830329 |
| il15 | 0.0045257207 | 0.05754111699 | 0.07865194379 | 0.9373094703 | 1.004535977 |
| fam3c | -0.004751912931 | 0.06115269221 | -0.07770570287 | 0.9380621576 | 0.9952593595 |
| arhgap5 | -0.004913084737 | 0.06326366925 | -0.07766044549 | 0.938098159 | 0.9950989647 |
| iglon5 | -0.004854005807 | 0.06334322622 | -0.07663022704 | 0.938917712 | 0.9951577558 |
| tinagl1 | -0.004659156768 | 0.0610313204 | -0.07634042223 | 0.9391482674 | 0.9953516803 |
| txndc15 | -0.004375206627 | 0.05811862194 | -0.07528063263 | 0.9399914307 | 0.9956343506 |
| gid8 | -0.004694193112 | 0.06260501383 | -0.07498110495 | 0.9402297456 | 0.9953168074 |
| dgcr6 | -0.004755888859 | 0.06402257282 | -0.07428456323 | 0.9407839599 | 0.9952554025 |
| tada3 | 0.004739926755 | 0.06382008034 | 0.0742701471 | 0.9407954306 | 1.004751178 |
| timm10 | 0.00462795416 | 0.06296768724 | 0.07349728667 | 0.9414104032 | 1.00463868 |
| acadm | -0.004697799298 | 0.06422738005 | -0.07314324971 | 0.9416921255 | 0.9953132181 |
| lama1 | -0.004619126349 | 0.0635532358 | -0.07268121427 | 0.9420597978 | 0.9953915254 |
| timp1 | 0.00450440076 | 0.06250372577 | 0.07206611613 | 0.9425492915 | 1.004514561 |
| vamp8 | -0.004625304571 | 0.06429527591 | -0.07193848234 | 0.9426508649 | 0.9953853757 |
| pth | -0.004779052057 | 0.06673428038 | -0.07161315039 | 0.9429097745 | 0.9952323494 |
| hsp90b1 | 0.004114936326 | 0.05752085509 | 0.07153816331 | 0.9429694524 | 1.004123414 |
| txk | -0.004477096034 | 0.06260641384 | -0.07151177905 | 0.9429904502 | 0.9955329112 |
| myl1 | -0.004604184209 | 0.06466533055 | -0.07120019599 | 0.9432384254 | 0.9954063988 |
| dipk1c | 0.004528974791 | 0.0636694028 | 0.0711326727 | 0.943292165 | 1.004539246 |
| c2 | -0.004315585184 | 0.06068048228 | -0.0711198234 | 0.9433023913 | 0.9956937136 |
| lat | -0.004115007754 | 0.05800549906 | -0.07094168346 | 0.9434441683 | 0.9958934473 |
| havcr1 | -0.00465192056 | 0.06565425031 | -0.07085482718 | 0.9435132957 | 0.9953588829 |

| Protein | beta | se | z | p_val | HR |
|---|---|---|---|---|---|
| il31 | -0.00455782608 | 0.06446482166 | -0.07070253144 | 0.9436345061 | 0.995452545 |
| cxcl8 | -0.004130933882 | 0.05882178553 | -0.07022795796 | 0.944012222 | 0.9958775867 |
| il5ra | -0.004044995717 | 0.05784080384 | -0.06993325556 | 0.9442467838 | 0.9959631743 |
| chmp6 | 0.004420642117 | 0.0638070745 | 0.06928137909 | 0.9447656474 | 1.004430428 |
| arhgap30 | -0.004386185847 | 0.06385312864 | -0.06869179225 | 0.9452349516 | 0.9956234194 |
| gal | -0.004198696231 | 0.06113273295 | -0.06868163794 | 0.9452430345 | 0.995810106 |
| flt3lg | -0.004095712034 | 0.06020190321 | -0.06803293278 | 0.9457594184 | 0.995912664 |
| cd69 | -0.003930513236 | 0.057900165 | -0.06788431841 | 0.9458777221 | 0.9960772011 |
| st8sia1 | -0.004337808123 | 0.06423006158 | -0.06753548131 | 0.9461554165 | 0.9956715866 |
| il17c | 0.003992895663 | 0.05918522196 | 0.06746440295 | 0.9462119998 | 1.004000878 |
| sox2 | -0.004220665718 | 0.06308891929 | -0.06690026974 | 0.9466610983 | 0.9957882288 |
| cd74 | -0.004190007012 | 0.06330920558 | -0.06618321891 | 0.9472319569 | 0.9958187588 |
| rras | -0.004206475315 | 0.06372429273 | -0.06601054535 | 0.9473694298 | 0.9958023595 |
| tcof1 | 0.004276907273 | 0.06513500226 | 0.06566219581 | 0.9476467708 | 1.004286066 |
| nars1 | 0.004066891645 | 0.0622261574 | 0.06535662518 | 0.9478900583 | 1.004075173 |
| cpxm2 | -0.004332330912 | 0.06643995769 | -0.06520670787 | 0.9480094204 | 0.9956770401 |
| dmd | -0.004312011495 | 0.06619285592 | -0.06514315533 | 0.9480600204 | 0.9956972719 |
| rnf4 | 0.0041381659 | 0.06356337646 | 0.0651029906 | 0.9480919993 | 1.00414674 |
| decr1 | 0.003759982412 | 0.05787563747 | 0.06496658311 | 0.9482006068 | 1.00376706 |
| nfatc3 | -0.003874067761 | 0.05975354597 | -0.06483410646 | 0.9483060855 | 0.9961334268 |
| dll1 | -0.003895046994 | 0.06043651794 | -0.0644485673 | 0.9486130593 | 0.9961125289 |
| tbcc | -0.003787985723 | 0.05885260206 | -0.06436394638 | 0.9486804371 | 0.9962191796 |
| naga | -0.004207554114 | 0.06581358919 | -0.06393138811 | 0.9490248593 | 0.9958012852 |
| akr7l | 0.004338795453 | 0.06793123284 | 0.06387040647 | 0.9490734164 | 1.004348222 |
| entpd2 | -0.003776794183 | 0.05924722969 | -0.06374634227 | 0.949172204 | 0.9962303289 |
| cdkn1a | 0.003739251396 | 0.05874063141 | 0.06365698336 | 0.9492433575 | 1.003746251 |
| apex1 | 0.003748968209 | 0.05902168412 | 0.06351848926 | 0.9493536367 | 1.003756004 |
| nos1 | -0.004000832277 | 0.06410441958 | -0.062411177 | 0.9502353944 | 0.9960071604 |
| dab2 | -0.003569500043 | 0.05775271183 | -0.06180662224 | 0.9507168298 | 0.996436863 |
| axl | -0.00359639851 | 0.05829038283 | -0.06169797376 | 0.9508033536 | 0.9964100608 |
| lpp | 0.00395164511 | 0.06443649325 | 0.06132619748 | 0.9510994275 | 1.003959463 |
| efcab14 | -0.003986240244 | 0.06574725339 | -0.06062976077 | 0.9516540715 | 0.9960216943 |
| lrp2bp | 0.003778788913 | 0.06324656463 | 0.05974694334 | 0.9523571831 | 1.003785938 |
| pdgfb | -0.003440365406 | 0.05810220231 | -0.0592123064 | 0.9527830077 | 0.9965655459 |
| prame | -0.003703881037 | 0.06357454404 | -0.05826044202 | 0.9535411767 | 0.9963029699 |
| cga | -0.0052273028 | 0.08989889664 | -0.05814646226 | 0.9536319654 | 0.9947863358 |
| pdlim7 | -0.003328545041 | 0.05825154883 | -0.0571408848 | 0.9544329683 | 0.9966769884 |
| diablo | -0.003271285039 | 0.05795786893 | -0.05644246588 | 0.954989328 | 0.9967340598 |
| mpo | 0.003353264445 | 0.0596033146 | 0.05625969743 | 0.9551349248 | 1.003358893 |
| ca7 | -0.00355813921 | 0.06332627048 | -0.05618741137 | 0.9551925096 | 0.9964481835 |
| mia | -0.003303837485 | 0.05895944859 | -0.05603575956 | 0.9553133199 | 0.9967016142 |
| ism1 | -0.003388728012 | 0.06098925797 | -0.05556270276 | 0.9556901775 | 0.9966170072 |
| cant1 | 0.003283211521 | 0.06005941255 | 0.05466606119 | 0.9564045082 | 1.003288607 |
| cpb1 | 0.00311574832 | 0.05710338411 | 0.05456328672 | 0.9564863881 | 1.003120607 |
| chad | 0.003474686951 | 0.06395115832 | 0.05433344825 | 0.9566695011 | 1.003480731 |
| abl1 | -0.003204800247 | 0.0594435602 | -0.05391332949 | 0.9570042167 | 0.9968003296 |
| aif1 | -0.003101656365 | 0.0592344474 | -0.0523623753 | 0.9582399531 | 0.9969031488 |
| smad5 | -0.003212442641 | 0.06150368977 | -0.05223170599 | 0.9583440697 | 0.9967927117 |
| dnmbp | -0.003144654118 | 0.06079761135 | -0.05172331689 | 0.9587491579 | 0.9968602851 |
| eri1 | -0.003334254711 | 0.06449553757 | -0.05169744818 | 0.9587697706 | 0.9966712977 |
| axin1 | 0.002985855971 | 0.05807404217 | 0.05141463999 | 0.9589951192 | 1.002990318 |
| tnfrsf4 | -0.00305565372 | 0.05947195044 | -0.05139460162 | 0.9590110864 | 0.9969481293 |
| il25 | 0.003234050561 | 0.06430194952 | 0.05029475132 | 0.9598875063 | 1.003239286 |
| map2k6 | -0.002878263862 | 0.05812480771 | -0.04951868188 | 0.9605059495 | 0.9971258744 |
| wasl | 0.003190502654 | 0.06482963354 | 0.04921364629 | 0.9607490363 | 1.003195598 |
| hsd17b3 | 0.003120784042 | 0.06349839719 | 0.04914744592 | 0.9608017927 | 1.003125659 |
| dkk4 | -0.002988764731 | 0.06097684882 | -0.04901474558 | 0.9609075448 | 0.9970156972 |
| hdac8 | 0.003109409249 | 0.06443451441 | 0.04825688961 | 0.9615115116 | 1.003114248 |
| cd93 | 0.002880818931 | 0.0598228903 | 0.04815579649 | 0.9615920786 | 1.002884972 |
| gcnt1 | 0.00288956135 | 0.0602329495 | 0.04797310067 | 0.9617376805 | 1.00289374 |
| tnfrsf1b | -0.002987140684 | 0.0625756269 | -0.04773648834 | 0.9619262537 | 0.9970173164 |
| gp2 | -0.002973941638 | 0.06304798617 | -0.04716949451 | 0.9623781403 | 0.9970304761 |
| cd63 | 0.002781975018 | 0.05932367777 | 0.0468948508 | 0.962597032 | 1.002785848 |
| gsn | -0.002944967412 | 0.06300577406 | -0.04674123056 | 0.962719469 | 0.9970593648 |
| itih3 | -0.002702346134 | 0.06179592437 | -0.04373016767 | 0.9651194919 | 0.9973013019 |
| ing1 | -0.002549510299 | 0.05860908037 | -0.0435002611 | 0.9653027564 | 0.9974537369 |
| gast | -0.002782272784 | 0.0644632299 | -0.04316061713 | 0.9655734988 | 0.9972215941 |
| cep85 | -0.002544105235 | 0.05894867959 | -0.04315796814 | 0.9655756104 | 0.9974591283 |
| rab33a | 0.002734339582 | 0.06344745659 | 0.04309612598 | 0.9656249075 | 1.002738081 |
| hip1 | 0.00277388953 | 0.06480572964 | 0.04280315252 | 0.9658584509 | 1.00277774 |

| Protein | beta | se | z | p_val | HR |
|---|---|---|---|---|---|
| wwp2 | -0.002524693058 | 0.05937748303 | -0.0425193681 | 0.9660846722 | 0.9974784913 |
| f11r | 0.002471266472 | 0.05910541755 | 0.04181116681 | 0.966649233 | 1.002474323 |
| sart1 | -0.002709518194 | 0.06592434771 | -0.0411004172 | 0.967215842 | 0.9972941492 |
| ptpn1 | -0.002426359576 | 0.05911211398 | -0.04104674004 | 0.9672586341 | 0.9975765817 |
| vps4b | 0.002540279064 | 0.06335740836 | 0.04009442825 | 0.9680178438 | 1.002543508 |
| ifnl1 | 0.00231705074 | 0.05783901569 | 0.04006034184 | 0.968045019 | 1.002319737 |
| fgf23 | 0.002483589898 | 0.06215326622 | 0.03995912119 | 0.9681257168 | 1.002486677 |
| nrcam | 0.002309097255 | 0.05914596581 | 0.03904065515 | 0.9688579752 | 1.002311765 |
| bhlhe40 | -0.002474794178 | 0.06376444617 | -0.03881150589 | 0.9690406714 | 0.9975282656 |
| eci2 | -0.002421430278 | 0.06336618638 | -0.03821328719 | 0.969517627 | 0.997581499 |
| akr1c4 | 0.002251462647 | 0.05892510152 | 0.03820888872 | 0.9695211339 | 1.002253999 |
| tppp3 | 0.002227448672 | 0.05861137353 | 0.03800369344 | 0.9696847372 | 1.002229931 |
| tf | -0.002419411001 | 0.06398983673 | -0.03780930104 | 0.9698397285 | 0.9975835134 |
| glp1r | 0.002358637871 | 0.06343220176 | 0.03718360399 | 0.9703386117 | 1.002361422 |
| msr1 | 0.002339148801 | 0.06312231089 | 0.03705740122 | 0.9704392376 | 1.002341887 |
| fdx1 | 0.002416862688 | 0.06536870074 | 0.03697278148 | 0.9705067081 | 1.002419786 |
| fndc1 | 0.00231200017 | 0.06454772751 | 0.0358184596 | 0.9714271139 | 1.002314675 |
| cd82 | -0.002215894276 | 0.06257021924 | -0.03541451992 | 0.9717492068 | 0.997786559 |
| tnfrsf12a | -0.002233307412 | 0.06487447828 | -0.03442505391 | 0.9725382052 | 0.9977691846 |
| mgll | 0.001982388057 | 0.05802686255 | 0.03416328179 | 0.9727469463 | 1.001984354 |
| psg1 | 0.001945591633 | 0.05865344012 | 0.03317097221 | 0.9735382462 | 1.001947486 |
| fut8 | -0.001920236585 | 0.05856647121 | -0.03278730211 | 0.9738442042 | 0.9980816059 |
| ca9 | 0.001919817442 | 0.05883663036 | 0.03262962938 | 0.9739699416 | 1.001921661 |
| septin9 | -0.001891066935 | 0.05854341573 | -0.03230195764 | 0.974231248 | 0.99811072 |
| zfyve19 | 0.002032231008 | 0.06321450017 | 0.03214817806 | 0.9743538827 | 1.002034297 |
| eif2ak3 | 0.002027908476 | 0.06352066857 | 0.03192517525 | 0.9745317219 | 1.002029966 |
| cd5l | -0.001967366299 | 0.06233563612 | -0.03156086023 | 0.9748222568 | 0.9980345677 |
| gp6 | -0.001830401875 | 0.05810866495 | -0.03149963738 | 0.9748710813 | 0.9981712723 |
| fyb1 | -0.001835943073 | 0.05842374608 | -0.0314246038 | 0.9749309198 | 0.9981657412 |
| akt3 | -0.001841550342 | 0.05939510504 | -0.03100508603 | 0.9752654836 | 0.9981601443 |
| pafah1b3 | 0.001951029607 | 0.06321013672 | 0.03086577103 | 0.9753765877 | 1.001952934 |
| adgre2 | -0.001873510761 | 0.06075258469 | -0.03083837126 | 0.9753984391 | 0.9981282432 |
| notch1 | -0.001822771663 | 0.05926901847 | -0.03075420701 | 0.9754655606 | 0.9981788886 |
| lysmd3 | -0.001927727868 | 0.06329967009 | -0.03045399549 | 0.9757049826 | 0.998074129 |
| pafah2 | 0.001929285668 | 0.06342328649 | 0.0304192005 | 0.9757327322 | 1.001931148 |
| slc12a2 | -0.001904523058 | 0.06286319896 | -0.03029631151 | 0.9758307382 | 0.9980972894 |
| akr1b10 | 0.001919827237 | 0.06337545491 | 0.0302929145 | 0.9758334474 | 1.001921671 |
| stk11 | -0.001770375583 | 0.05937994941 | -0.02981436664 | 0.9762111009 | 0.9982311906 |
| atp1b2 | 0.001860105378 | 0.06373014494 | 0.02918721399 | 0.9767152787 | 1.001861836 |
| spink1 | 0.001741445711 | 0.05980355187 | 0.02911943616 | 0.9767693346 | 1.001742963 |
| klrb1 | 0.0016782109 | 0.05854847658 | 0.02866361344 | 0.9771328767 | 1.00167962 |
| cert | -0.001652066305 | 0.05789899746 | -0.02853359087 | 0.9772365773 | 0.9983492976 |
| hpcal1 | 0.00163962344 | 0.05808035329 | 0.02823025941 | 0.9774785033 | 1.001640968 |
| cr2 | -0.001644448656 | 0.05849198905 | -0.02811408335 | 0.9775711616 | 0.9983569027 |
| bcat1 | -0.001813330965 | 0.06570534717 | -0.02759792077 | 0.97798284 | 0.9981883121 |
| abo | -0.001734811549 | 0.06286769357 | -0.02759464282 | 0.9779854544 | 0.9982666924 |
| gpr158 | 0.00177525752 | 0.06481444451 | 0.02738984395 | 0.9781487986 | 1.001776834 |
| farsa | -0.001742024247 | 0.06427383632 | -0.02710316275 | 0.9783774522 | 0.9982594922 |
| lat2 | 0.00157615173 | 0.05821200306 | 0.02707606072 | 0.9783990685 | 1.001577395 |
| nudt10 | -0.001718823787 | 0.0640959077 | -0.02681643569 | 0.9786061441 | 0.9982826525 |
| dnajb14 | 0.001681386814 | 0.06379585022 | 0.02635573958 | 0.9789735966 | 1.001682801 |
| tarbp2 | -0.00146219497 | 0.05857258337 | -0.02496381217 | 0.9800838283 | 0.9985388735 |
| mphosph8 | -0.001476976213 | 0.05922950212 | -0.02493649549 | 0.9801056171 | 0.998524114 |
| ephx2 | -0.001440118109 | 0.05987893661 | -0.02405049572 | 0.9808123306 | 0.9985609184 |
| defb116 | -0.001508800949 | 0.06328977814 | -0.02383956768 | 0.9809805786 | 0.9984923367 |
| cct5 | 0.001369586875 | 0.05802776216 | 0.02360226939 | 0.9811698619 | 1.001370525 |
| numb | 0.001520007243 | 0.06512842292 | 0.02333861585 | 0.9813801691 | 1.001521163 |
| dhodh | -0.001492311437 | 0.0640155757 | -0.02331169283 | 0.9814016447 | 0.9985088015 |
| tcl1a | 0.001345536435 | 0.05834586342 | 0.02306138527 | 0.9816013076 | 1.001346442 |
| cdnf | 0.001314659475 | 0.05969168468 | 0.02202416437 | 0.9824286798 | 1.001315524 |
| fgf7 | 0.001410851613 | 0.06466783346 | 0.02181689934 | 0.9825940137 | 1.001411847 |
| bcr | 0.001264689538 | 0.05817063528 | 0.0217410302 | 0.9826545341 | 1.00126549 |
| mocs2 | -0.001352258582 | 0.06438847272 | -0.02100156324 | 0.9832444087 | 0.9986486553 |
| pilra | 0.001240477493 | 0.05966618603 | 0.02079029305 | 0.9834129411 | 1.001241247 |
| vstm1 | 0.001187724685 | 0.05760379083 | 0.02061886324 | 0.983549693 | 1.00118843 |
| selplg | 0.001214323817 | 0.05904126308 | 0.02056737531 | 0.9835907657 | 1.001215061 |
| tdo2 | 0.001173068543 | 0.0637976503 | 0.01838733146 | 0.9853298588 | 1.001173757 |
| gtf2ird1 | -0.001154005153 | 0.06310585571 | -0.0182868157 | 0.9854100453 | 0.9988466605 |
| timp2 | -0.001167698933 | 0.06395790707 | -0.01825730369 | 0.9854335885 | 0.9988329826 |
| entr1 | 0.001184383403 | 0.06638465468 | 0.01784122263 | 0.9857655191 | 1.001185085 |

| Protein | beta | se | z | p_val | HR |
|---|---|---|---|---|---|
| amy2b | -0.001073449407 | 0.06083880623 | -0.01764415631 | 0.9859227305 | 0.9989271265 |
| hhex | -0.001137553995 | 0.06490709383 | -0.01752588089 | 0.9860170861 | 0.9988630928 |
| arhgef10 | -0.001091657717 | 0.06239496217 | -0.01749592721 | 0.986040982 | 0.9989089379 |
| ptpn6 | -0.0009257408409 | 0.05816332058 | -0.01591623091 | 0.9873012212 | 0.9990746875 |
| il2rb | 0.0009036195841 | 0.05823352717 | 0.01551717074 | 0.9876195859 | 1.000904028 |
| vnn1 | -0.001020764513 | 0.06603138901 | -0.01545877693 | 0.9876661718 | 0.9989797563 |
| rnase6 | 0.001046378866 | 0.06866683021 | 0.01523849088 | 0.9878419139 | 1.001046927 |
| btn1a1 | 0.0009504007295 | 0.06391577158 | 0.01486958079 | 0.9881362282 | 1.000950853 |
| eef1d | -0.0009415054264 | 0.06348934895 | -0.01482934448 | 0.9881683286 | 0.9990589377 |
| stx16 | 0.0008221094566 | 0.05815255689 | 0.01413711624 | 0.9887205889 | 1.000822447 |
| fkbp14 | -0.0008789887632 | 0.0631572737 | -0.01391745893 | 0.9888958329 | 0.9991213974 |
| klk3 | 0.001293270431 | 0.09572598412 | 0.01351012938 | 0.9892208043 | 1.001294107 |
| cd72 | 0.0008333491368 | 0.06425365284 | 0.01296967721 | 0.9896519849 | 1.000833696 |
| gpd1 | 0.0009206395028 | 0.07117835107 | 0.01293426286 | 0.9896802391 | 1.000921063 |
| fli1 | 0.0007452655389 | 0.05868870942 | 0.01269861863 | 0.9898682405 | 1.000745543 |
| fabp4 | 0.0009954751578 | 0.07864320447 | 0.01265812049 | 0.9899005508 | 1.000995971 |
| dag1 | -0.0007583675736 | 0.06002950112 | -0.01263324798 | 0.9899203946 | 0.9992419199 |
| ccn2 | 0.0007181161484 | 0.05974547009 | 0.01201959157 | 0.9904099844 | 1.000718374 |
| csde1 | 0.0006935941395 | 0.06441054898 | 0.01076833144 | 0.9914082806 | 1.000693835 |
| gc | -0.0006507846493 | 0.06352932789 | -0.01024384597 | 0.9918267364 | 0.9993494271 |
| pinlyp | -0.000639409596 | 0.06318270829 | -0.01012000931 | 0.9919255386 | 0.9993607948 |
| mrpl24 | -0.0006405353584 | 0.06461816879 | -0.009912620094 | 0.992091003 | 0.9993596697 |
| syt1 | 0.0005877655009 | 0.06303894301 | 0.009323847655 | 0.9925607537 | 1.000587938 |
| dpp4 | -0.0005729181229 | 0.06183566274 | -0.009265173163 | 0.9926075671 | 0.999427246 |
| rfc4 | -0.0005459404581 | 0.06459035116 | -0.008452353151 | 0.9932560782 | 0.9994542085 |
| igdcc3 | -0.0005139606352 | 0.0629688728 | -0.008162138091 | 0.9934876283 | 0.9994861714 |
| tubb3 | 0.0004527074849 | 0.06337406444 | 0.007143418825 | 0.9943004249 | 1.00045281 |
| obp2b | -0.0005038104598 | 0.07484261287 | -0.006731599024 | 0.9946290016 | 0.9994963164 |
| il20ra | -0.0003412631488 | 0.05876025383 | -0.005807720806 | 0.9953661353 | 0.9996587951 |
| tcn1 | 0.0003012414664 | 0.06359361815 | 0.004736976369 | 0.9962204538 | 1.000301287 |
| pvalb | 0.0002661593048 | 0.05755898864 | 0.004624113645 | 0.9963105043 | 1.000266195 |
| psapl1 | -0.0002922865337 | 0.06372992882 | -0.004586330773 | 0.9963406503 | 0.9997077562 |
| nectin1 | 0.0002647281965 | 0.06401147131 | 0.004135636802 | 0.9967002487 | 1.000264763 |
| nit2 | 0.0002654388621 | 0.06496559933 | 0.004085837195 | 0.9967399827 | 1.000265474 |
| sdccag8 | 0.0002291225038 | 0.06317407674 | 0.003626843725 | 0.9971062037 | 1.000229149 |
| serpinb1 | -0.000190922544 | 0.05882219364 | -0.003245756953 | 0.9974102652 | 0.9998090957 |
| jam3 | -0.0001974895808 | 0.06366293264 | -0.003102112527 | 0.9975248763 | 0.9998025299 |
| ikbkg | -0.0001290207806 | 0.05824986461 | -0.002214954171 | 0.9982327237 | 0.9998709875 |
| sod3 | -0.0001423652677 | 0.06899211403 | -0.002063500586 | 0.9983535659 | 0.9998576449 |
| dpp10 | -0.0001138464811 | 0.05910393453 | -0.001926208162 | 0.9984631092 | 0.99988616 |
| crybb2 | 4.80025e-05 | 0.06296455097 | 0.0007623734464 | 0.9993917141 | 1.000048004 |

## Supplementary Table 12. Associations between circulating plasma proteins and incident retinal vein occlusion (RVO) in the UK Biobank

| Protein | beta | se | z | p_val | HR |
|---|---|---|---|---|---|
| mrpl52 | 0.6093129832 | 0.1642831479 | 3.708919576 | 0.0002081455226 | 1.839167426 |
| pla2g7 | -0.512946072 | 0.1451467129 | -3.533983386 | 0.000409346808 | 0.5987290791 |
| yy1 | 0.5288876468 | 0.1559656239 | 3.391052679 | 0.0006962471716 | 1.697043546 |
| hif1a | 0.5028490917 | 0.1600470707 | 3.141882507 | 0.001678653832 | 1.653425327 |
| csf2 | 0.5068845354 | 0.1633154347 | 3.103714822 | 0.001911074396 | 1.660111113 |
| st8sia1 | 0.4758437511 | 0.1550082556 | 3.069796181 | 0.002142048977 | 1.609371534 |
| ifnl2 | 0.4623107711 | 0.1536788701 | 3.008291061 | 0.002627213865 | 1.58773865 |
| c1qtnf9 | -0.470480821 | 0.1586523331 | -2.965483155 | 0.003022078948 | 0.6247018262 |
| nedd9 | 0.4603865037 | 0.1576879458 | 2.919604929 | 0.003504753845 | 1.584686354 |
| cep152 | 0.4564579037 | 0.1602460179 | 2.848482039 | 0.004392833288 | 1.578472968 |
| rab44 | 0.4393412562 | 0.1543700422 | 2.846026664 | 0.004426848796 | 1.551684719 |
| c7 | 0.4437194878 | 0.1607059842 | 2.761063877 | 0.005761340683 | 1.558493248 |
| slc9a3r2 | 0.4357759294 | 0.1587618963 | 2.744839534 | 0.006054049197 | 1.546162306 |
| mdm1 | 0.4349248456 | 0.1611404095 | 2.699042699 | 0.006953925356 | 1.544846953 |
| gzmb | -0.410175281 | 0.1542580235 | -2.659020721 | 0.007836814293 | 0.6635339352 |
| gask1a | 0.4382208516 | 0.1667285617 | 2.628349019 | 0.008580043364 | 1.549947178 |
| plcb1 | 0.4060964449 | 0.1549796432 | 2.620321201 | 0.008784698254 | 1.500947304 |
| nxpe4 | 0.4141804873 | 0.1596286301 | 2.59465039 | 0.009468717905 | 1.513130203 |
| il31 | 0.4076037356 | 0.1577129164 | 2.584466414 | 0.009752980284 | 1.503211374 |
| ep300 | 0.4074839145 | 0.1583286685 | 2.573658444 | 0.01006295594 | 1.503031268 |
| preb | 0.3553975465 | 0.1417174772 | 2.507789114 | 0.01214891497 | 1.42674774 |
| psg1 | 0.3698024062 | 0.1496971314 | 2.470337291 | 0.01349857106 | 1.447448579 |
| hrc | -0.380626993 | 0.154455857 | -2.464309225 | 0.01372776207 | 0.683432767 |
| ednrb | 0.386080773 | 0.1572685015 | 2.454914807 | 0.014091798 | 1.4712035 |
| gad2 | 0.3794834222 | 0.1550337735 | 2.447746795 | 0.01437526383 | 1.461529401 |
| klb | 0.3589523284 | 0.1468240925 | 2.444778116 | 0.01449412818 | 1.431828542 |
| ca14 | -0.381756336 | 0.1575840374 | -2.422557144 | 0.01541170166 | 0.6826613733 |
| serpina1 | 0.3645620781 | 0.1507486554 | 2.418343814 | 0.01559133732 | 1.439883314 |
| tor1aip1 | 0.3731464195 | 0.1548588145 | 2.409591089 | 0.01597040886 | 1.452296969 |
| naga | 0.3834388081 | 0.1597144374 | 2.400777378 | 0.0163602863 | 1.467321762 |
| ifit1 | -0.377174159 | 0.1573997551 | -2.39628174 | 0.01656235412 | 0.6857966259 |
| lpcat2 | 0.355202219 | 0.1483092171 | 2.395011085 | 0.01661986281 | 1.426469084 |
| kirrel2 | 0.3530906329 | 0.1482023985 | 2.382489329 | 0.01719603036 | 1.42346015 |
| lilra3 | 0.3690735091 | 0.1562299423 | 2.362373715 | 0.01815832688 | 1.446393923 |
| mmp7 | 0.3555656504 | 0.1505174459 | 2.362288625 | 0.01816249561 | 1.426987602 |
| raly | 0.3630610494 | 0.1539205853 | 2.358755645 | 0.01833632524 | 1.437723629 |
| fhip2a | -0.372958736 | 0.1585276302 | -2.352641842 | 0.01864057889 | 0.6886936507 |
| babam1 | 0.3680353805 | 0.1567113997 | 2.348491439 | 0.01884963251 | 1.444893159 |
| reg4 | 0.3511087947 | 0.1504233667 | 2.334137325 | 0.01958853242 | 1.420641876 |
| bhlhe40 | 0.3604473687 | 0.1546827223 | 2.33023678 | 0.01979364004 | 1.433970785 |
| hip1 | 0.3845236689 | 0.1653971298 | 2.324850917 | 0.02007993327 | 1.468914465 |
| clspn | 0.3343514163 | 0.1445624774 | 2.312850626 | 0.02073085325 | 1.397033998 |
| pnma2 | 0.348064322 | 0.1512938297 | 2.300585044 | 0.02141509718 | 1.416323348 |
| clec12a | 0.3561690416 | 0.1551903034 | 2.295047008 | 0.02173043049 | 1.427848894 |
| tpr | 0.3803388027 | 0.1658758612 | 2.292912302 | 0.02185305444 | 1.462780099 |
| mrpl46 | -0.33193309 | 0.1449143441 | -2.290546821 | 0.02198963763 | 0.7175353313 |
| tnfrsf1b | 0.3546500008 | 0.1548648805 | 2.290060856 | 0.0220177891 | 1.425681579 |
| ptprm | -0.348233397 | 0.1520994256 | -2.289511586 | 0.02204964554 | 0.705934094 |
| coch | 0.358011651 | 0.1568044792 | 2.283172348 | 0.02242021827 | 1.430482287 |
| gfra1 | 0.3529681137 | 0.1546588903 | 2.282236171 | 0.02247540065 | 1.423285759 |
| scn2a | 0.3518684897 | 0.1545617891 | 2.27655549 | 0.02281278338 | 1.42172154 |
| scarb1 | -0.336166112 | 0.1476737232 | -2.276411161 | 0.02282141232 | 0.7145044085 |
| mia | 0.3266220628 | 0.1438226296 | 2.271006056 | 0.02314661135 | 1.386277452 |
| ptpn9 | 0.3591972191 | 0.1583005177 | 2.269084298 | 0.02326320007 | 1.432179227 |
| arhgef5 | 0.3594720394 | 0.1587905942 | 2.263811916 | 0.02358568529 | 1.432572873 |
| cxcl9 | 0.3520545235 | 0.1561201838 | 2.255022476 | 0.02413191803 | 1.421986053 |
| cstb | 0.342209188 | 0.1531047596 | 2.235130958 | 0.02540875252 | 1.408054815 |
| saa4 | 0.3553429182 | 0.159057473 | 2.234053587 | 0.02547954738 | 1.426669801 |
| ctsh | 0.3258317318 | 0.1460562932 | 2.230864037 | 0.02569013658 | 1.385182267 |
| rbp5 | 0.3541613854 | 0.158846696 | 2.229579805 | 0.0257753518 | 1.42498514 |
| tubb3 | 0.3444679393 | 0.1546321699 | 2.227660256 | 0.02590317919 | 1.411238855 |
| vsig4 | 0.3584574812 | 0.1622074918 | 2.209870069 | 0.02711418101 | 1.431120181 |
| ache | -0.368384213 | 0.1672800681 | -2.202200283 | 0.02765116412 | 0.6918513123 |
| nfic | 0.3405042887 | 0.1555757684 | 2.188671745 | 0.02862070703 | 1.405656268 |
| crisp2 | -0.35940503 | 0.1648352427 | -2.180389486 | 0.02922860283 | 0.6980915459 |
| f7 | -0.313900936 | 0.1444814754 | -2.172603338 | 0.02981018448 | 0.7305914002 |
| anxa10 | 0.3268573089 | 0.1504466494 | 2.172579518 | 0.02981197884 | 1.386603607 |
| nme1 | 0.3400685904 | 0.1567926145 | 2.168906944 | 0.03008974942 | 1.40504396 |

| Protein | beta | se | z | p_val | HR |
|---|---|---|---|---|---|
| txndc15 | -0.313134577 | 0.1446585969 | -2.164645474 | 0.03041484573 | 0.7311515097 |
| rnase1 | 0.3649008479 | 0.1697463609 | 2.149682892 | 0.03158030663 | 1.440371185 |
| entpd2 | 0.3159346057 | 0.1471250612 | 2.1473881 | 0.03176239725 | 1.371540562 |
| fgf5 | 0.3290757801 | 0.1534906474 | 2.143946786 | 0.03203715084 | 1.389683162 |
| spint3 | -0.497060855 | 0.2319261537 | -2.143185868 | 0.03209817661 | 0.6083159636 |
| tnfrsf1a | 0.3437224756 | 0.1614539475 | 2.128919615 | 0.03326091167 | 1.41018722 |
| tnfrsf14 | 0.3186703792 | 0.1502672085 | 2.120691416 | 0.03394777941 | 1.375297923 |
| dpep2 | -0.306450161 | 0.1445932073 | -2.119395279 | 0.03405707544 | 0.7360552016 |
| cep164 | 0.3015690103 | 0.1425461677 | 2.11558834 | 0.03437983442 | 1.351978412 |
| lilrb4 | 0.3294283023 | 0.1558809987 | 2.113331997 | 0.03457236192 | 1.390173142 |
| mapre3 | -0.342912342 | 0.1625107368 | -2.11009038 | 0.03485057175 | 0.7097004195 |
| fga | 0.3553733861 | 0.1687631578 | 2.105752172 | 0.03522588539 | 1.42671327 |
| dpy30 | 0.3191127666 | 0.1518696184 | 2.101228475 | 0.03562091493 | 1.375906472 |
| vat1 | 0.3043860226 | 0.1452795388 | 2.095174759 | 0.03615546023 | 1.355792322 |
| pik3ip1 | 0.3294887653 | 0.1576086975 | 2.090549384 | 0.03656847714 | 1.390257199 |
| fstl3 | 0.3511085451 | 0.1681702857 | 2.087815595 | 0.03681447224 | 1.420641521 |
| serpinc1 | 0.332392933 | 0.159460433 | 2.084485328 | 0.03711604346 | 1.394300608 |
| ccl3 | 0.3395176819 | 0.1633953997 | 2.077890091 | 0.03771948344 | 1.404270122 |
| tnf | 0.3200827139 | 0.154277192 | 2.074724784 | 0.03801204909 | 1.377241677 |
| relb | 0.3293648227 | 0.1589709903 | 2.071854884 | 0.0382789765 | 1.390084898 |
| ppl | 0.3370245444 | 0.1627231214 | 2.071153389 | 0.03834446393 | 1.400773444 |
| muc13 | -0.304068954 | 0.1470043252 | -2.068435429 | 0.03859909659 | 0.7378099894 |
| colec12 | 0.3391392244 | 0.1640996348 | 2.066666539 | 0.03876558618 | 1.403738766 |
| matn3 | -0.304598862 | 0.1475973754 | -2.063714623 | 0.03904478204 | 0.737419122 |
| fgfbp1 | -0.3076421 | 0.1491707453 | -2.062348751 | 0.03917454474 | 0.7351783911 |
| pcsk9 | 0.3117048214 | 0.15164107 | 2.055543537 | 0.03982653499 | 1.365751493 |
| glp1r | 0.3161720923 | 0.1539519425 | 2.053706417 | 0.04000411513 | 1.371866323 |
| agbl2 | -0.318157062 | 0.1550046228 | -2.052564992 | 0.04011478578 | 0.7274885184 |
| sit1 | 0.3026642434 | 0.147524483 | 2.051620431 | 0.04020656501 | 1.353459955 |
| rcc1 | 0.3245318647 | 0.1586613943 | 2.045436863 | 0.0408118078 | 1.383382884 |
| shh | 0.3241545576 | 0.1587588347 | 2.041804844 | 0.0411708936 | 1.382861022 |
| ccl16 | 0.3199278976 | 0.1567909165 | 2.040474695 | 0.04130306893 | 1.377028474 |
| tcn1 | -0.317028669 | 0.1556139981 | -2.037276038 | 0.04162238772 | 0.7283098746 |
| kel | -0.304586629 | 0.1496340016 | -2.035544231 | 0.04179614275 | 0.7374281431 |
| crybb2 | 0.3130254638 | 0.1541837669 | 2.030210249 | 0.04233517251 | 1.367556354 |
| csf3r | 0.3189649571 | 0.1573938473 | 2.026540189 | 0.04270945862 | 1.375703115 |
| il13ra2 | 0.3077946156 | 0.1519953886 | 2.025026012 | 0.04286469328 | 1.360421551 |
| psap | 0.3299946635 | 0.1637183037 | 2.015624741 | 0.04383923068 | 1.390960706 |
| scn3b | 0.3030738327 | 0.1505244794 | 2.013452123 | 0.04406708689 | 1.354014431 |
| tnfrsf19 | 0.3059360262 | 0.1519941139 | 2.012814959 | 0.04413409958 | 1.357895434 |
| b2m | 0.3369993687 | 0.1674540714 | 2.012488355 | 0.04416848297 | 1.400738179 |
| dcbld2 | 0.2963744262 | 0.1476402872 | 2.007408898 | 0.04470614214 | 1.344973656 |
| klf4 | 0.3179313254 | 0.1584256399 | 2.006817367 | 0.04476911298 | 1.37428188 |
| rtn4r | 0.3201001168 | 0.1598348334 | 2.002693093 | 0.04521024066 | 1.377265645 |
| sirpa | 0.2775456761 | 0.1386885606 | 2.001215349 | 0.04536918758 | 1.319886405 |
| apom | -0.297874305 | 0.1492227296 | -1.996172474 | 0.04591515252 | 0.7423946489 |
| col18a1 | 0.301352038 | 0.1520574234 | 1.981830491 | 0.04749821567 | 1.351685102 |
| lmod2 | 0.3016732033 | 0.1524484779 | 1.978853495 | 0.04783250346 | 1.352119286 |
| lrrc25 | 0.2953803589 | 0.1495825127 | 1.974698469 | 0.04830237676 | 1.343637326 |
| tjp3 | 0.3014539662 | 0.1527046975 | 1.97409753 | 0.04837065413 | 1.351822884 |
| inpp5j | -0.313837559 | 0.1590418169 | -1.973302148 | 0.04846114833 | 0.7306377041 |
| afp | -0.297443026 | 0.1507422251 | -1.973189835 | 0.04847393818 | 0.7427148973 |
| cd276 | 0.2865963259 | 0.1455385732 | 1.969212145 | 0.04892873486 | 1.331886457 |
| gimap8 | 0.3233846577 | 0.165069618 | 1.959080427 | 0.05010336861 | 1.381796767 |
| kirrel1 | 0.2968457097 | 0.1516186699 | 1.957844043 | 0.05024831554 | 1.345607669 |
| mucl3 | 0.3120358457 | 0.1594010329 | 1.957552219 | 0.05028257869 | 1.366203665 |
| scrib | 0.296776707 | 0.1517614835 | 1.955546955 | 0.05051854683 | 1.345514822 |
| vstm2b | -0.307945705 | 0.1577762661 | -1.951787254 | 0.05096346812 | 0.7349552211 |
| clmp | 0.3239190333 | 0.1660444577 | 1.95079702 | 0.05108119654 | 1.382535363 |
| cda | 0.3043697956 | 0.1564642122 | 1.945299767 | 0.05173891019 | 1.355770321 |
| lilra6 | 0.2893758102 | 0.1487887734 | 1.944876643 | 0.05178982678 | 1.335593564 |
| caly | 0.3040736367 | 0.1568570499 | 1.938539816 | 0.052557398 | 1.355368857 |
| terf1 | 0.3011818993 | 0.1565571839 | 1.923782045 | 0.05438190897 | 1.351455148 |
| fgf23 | 0.3020994544 | 0.1574017823 | 1.91928865 | 0.05494781357 | 1.352695752 |
| map1lc3a | 0.2913214623 | 0.1518554013 | 1.918413569 | 0.05505859186 | 1.338194694 |
| prss8 | 0.3219199836 | 0.1678235548 | 1.918205009 | 0.05508502131 | 1.379774367 |
| tfpi | -0.290913628 | 0.1518468551 | -1.915835711 | 0.05538601071 | 0.7475802456 |
| il3 | -0.303894391 | 0.1589478382 | -1.9119127 | 0.0558873924 | 0.7379387956 |
| fam171a2 | -0.296529394 | 0.1555156663 | -1.906749337 | 0.05655305474 | 0.7433937759 |
| tppp2 | 0.2967807847 | 0.1559010399 | 1.903648526 | 0.05695597446 | 1.345520308 |

| Protein | beta | se | z | p_val | HR |
|---|---|---|---|---|---|
| il20rb | 0.2953625401 | 0.1554321756 | 1.900266395 | 0.05739816898 | 1.343613384 |
| snrpb2 | 0.3033716544 | 0.1596525096 | 1.900199722 | 0.05740691471 | 1.354417746 |
| adcyap1r1 | -0.272590267 | 0.1435433151 | -1.899010528 | 0.05756309174 | 0.7614046966 |
| evpl | -0.297846682 | 0.1574048856 | -1.892232766 | 0.05845997448 | 0.7424151565 |
| ralb | 0.2939475096 | 0.1560983484 | 1.883091734 | 0.0596879373 | 1.341713475 |
| sart1 | 0.311241994 | 0.1657385236 | 1.877909778 | 0.06039350857 | 1.365119532 |
| nos3 | 0.2873646557 | 0.1534119373 | 1.873157074 | 0.06104669798 | 1.332910178 |
| tarm1 | 0.2992943187 | 0.1600113897 | 1.870456343 | 0.06142047411 | 1.348906574 |
| itgb1 | -0.283145097 | 0.1515325297 | -1.868543325 | 0.06168637705 | 0.7534104624 |
| ager | -0.278915298 | 0.1492888464 | -1.868292939 | 0.0617212503 | 0.7566039866 |
| chp1 | 0.288762542 | 0.1549620926 | 1.863439873 | 0.06240040465 | 1.334774738 |
| layn | 0.2861832272 | 0.1536535663 | 1.862522518 | 0.06252947447 | 1.33133637 |
| creb3 | -0.300593047 | 0.161394154 | -1.862477911 | 0.06253575611 | 0.740379011 |
| acp1 | -0.29132369 | 0.1572693522 | -1.852386915 | 0.06397028293 | 0.7472737538 |
| ruvbl1 | 0.2608290693 | 0.1415761455 | 1.842323567 | 0.06542783453 | 1.298005777 |
| sh3bgrl2 | 0.279063427 | 0.1516599442 | 1.840060198 | 0.06575939988 | 1.321891185 |
| hnmt | 0.2930286701 | 0.1594832693 | 1.837363075 | 0.06615631403 | 1.340481222 |
| ceacam20 | 0.2989420494 | 0.1630700628 | 1.833212328 | 0.06677100122 | 1.348431479 |
| defb103a_defb103b | 0.2910350192 | 0.1590694795 | 1.829609427 | 0.06730836304 | 1.337811432 |
| ccn3 | 0.287208154 | 0.1570423881 | 1.82885753 | 0.06742095397 | 1.332701592 |
| il6r | 0.2631163359 | 0.1441139371 | 1.825752187 | 0.06788759836 | 1.300978061 |
| wfdc2 | 0.3151477058 | 0.1728769076 | 1.822960106 | 0.06830943334 | 1.370461721 |
| clec4m | -0.281505237 | 0.1545085974 | -1.821938986 | 0.06846424405 | 0.7546469634 |
| fcrl2 | 0.2573031353 | 0.1413207631 | 1.820702985 | 0.06865201813 | 1.293437154 |
| ranbp2 | 0.2771243942 | 0.1522517406 | 1.820172257 | 0.06873277665 | 1.319330478 |
| znf174 | 0.2846385581 | 0.1565748696 | 1.817907043 | 0.0690783417 | 1.329281483 |
| otoa | 0.2768193482 | 0.1523565816 | 1.816917557 | 0.06922973786 | 1.318928083 |
| rbp7 | 0.3001745132 | 0.1653364659 | 1.815537253 | 0.06944138646 | 1.350094396 |
| lamp2 | -0.279109554 | 0.1542073628 | -1.809962568 | 0.07030159222 | 0.7564570256 |
| cd48 | 0.2592216498 | 0.1433205275 | 1.808684732 | 0.07049999632 | 1.295921014 |
| dxo | 0.2770702308 | 0.1537260999 | 1.802362975 | 0.07148831643 | 1.31925902 |
| arntl | 0.2879943154 | 0.1598872939 | 1.801233284 | 0.07166611834 | 1.333749722 |
| mcam | -0.271164371 | 0.150708449 | -1.799264559 | 0.07197684155 | 0.7624911547 |
| gipc2 | 0.2956633273 | 0.1643444215 | 1.799046933 | 0.07201125699 | 1.344017587 |
| stx6 | 0.2639665784 | 0.146754081 | 1.798700088 | 0.07206613493 | 1.302084678 |
| slc12a2 | 0.2800607568 | 0.1557478179 | 1.798168094 | 0.07215037406 | 1.323210204 |
| cst7 | 0.2636288759 | 0.1466330413 | 1.797881799 | 0.07219574107 | 1.301645035 |
| nbl1 | 0.2687924978 | 0.1495959541 | 1.79678989 | 0.07236898199 | 1.30838362 |
| baiap2 | 0.2694620378 | 0.1500080977 | 1.796316611 | 0.07244417744 | 1.309259929 |
| gp2 | 0.2737728111 | 0.1525685875 | 1.794424498 | 0.07274543965 | 1.314916034 |
| vamp5 | 0.2671566271 | 0.1490175161 | 1.792786742 | 0.07300703031 | 1.306245024 |
| ninj1 | 0.273512777 | 0.1526021646 | 1.792325671 | 0.07308081347 | 1.314574155 |
| col6a3 | 0.2879821084 | 0.1608479986 | 1.790399078 | 0.07338977841 | 1.333733441 |
| traf3 | 0.2871689714 | 0.1605178976 | 1.789015279 | 0.07361235487 | 1.332649374 |
| ssbp1 | 0.2757702874 | 0.154206547 | 1.788317634 | 0.07372477638 | 1.317545173 |
| dennd2b | 0.2862839554 | 0.1601343076 | 1.787774023 | 0.07381247351 | 1.33147048 |
| abca2 | 0.2957499161 | 0.1654886659 | 1.787130947 | 0.07391632679 | 1.344133969 |
| ccl25 | 0.2685100831 | 0.1503395489 | 1.78602427 | 0.07409532852 | 1.308014166 |
| stau1 | 0.2921097665 | 0.1635681347 | 1.785859863 | 0.07412195121 | 1.339250014 |
| anp32c | 0.2707624118 | 0.1517533163 | 1.784227313 | 0.07438673579 | 1.310963564 |
| acta2 | 0.3008490367 | 0.1688191767 | 1.782078568 | 0.0747364202 | 1.351005374 |
| tgfbr1 | 0.2785657245 | 0.1563589111 | 1.781578821 | 0.07481794071 | 1.32123344 |
| sirt1 | 0.2761142633 | 0.1551948183 | 1.779146149 | 0.07521580439 | 1.317998454 |
| tgfbi | -0.258437482 | 0.1453884754 | -1.777565113 | 0.07547530787 | 0.7722573098 |
| sptlc1 | 0.2712730474 | 0.1526479799 | 1.777115214 | 0.07554928555 | 1.31163316 |
| steap4 | 0.2751242879 | 0.1550580703 | 1.774330658 | 0.07600847226 | 1.316694314 |
| itgal | 0.2820179741 | 0.1592251325 | 1.771190073 | 0.07652909943 | 1.32580255 |
| rpa2 | 0.2730396659 | 0.1542461082 | 1.770155948 | 0.07670116554 | 1.313952363 |
| ccnd2 | -0.282039418 | 0.1598365144 | -1.764549353 | 0.0776395338 | 0.754243953 |
| dbh | 0.2809739353 | 0.159307729 | 1.763718164 | 0.07777944145 | 1.324419083 |
| gast | 0.2757723574 | 0.1564567126 | 1.762611222 | 0.07796608302 | 1.3175479 |
| igsf8 | 0.259915817 | 0.1475122161 | 1.761995202 | 0.07807010822 | 1.296820912 |
| vwc2 | 0.263790411 | 0.1497425306 | 1.761626507 | 0.07813242241 | 1.301855313 |
| scgb3a2 | 0.2592813501 | 0.147298957 | 1.760238874 | 0.07836731338 | 1.295998383 |
| gsn | -0.271327058 | 0.1545963374 | -1.75506783 | 0.07924770626 | 0.762367117 |
| cdkl5 | 0.260133802 | 0.1482251283 | 1.754991242 | 0.07926080576 | 1.29710363 |
| nectin1 | 0.2700461182 | 0.1539106791 | 1.75456388 | 0.07933393436 | 1.310024865 |
| dsc2 | 0.2707304068 | 0.1544270682 | 1.753127932 | 0.07958004996 | 1.310921607 |
| eif2ak3 | 0.2748355601 | 0.1567691673 | 1.753122536 | 0.07958097605 | 1.316314202 |
| tpsg1 | 0.2714001666 | 0.1549117286 | 1.751966549 | 0.07977955996 | 1.311799904 |

| Protein | beta | se | z | p_val | HR |
|---|---|---|---|---|---|
| aif1l | 0.2857378534 | 0.1633313745 | 1.74943641 | 0.08021561182 | 1.33074356 |
| fam3b | 0.2506992029 | 0.1437481013 | 1.74401749 | 0.08115604005 | 1.284923525 |
| ecscr | 0.2653414081 | 0.1522806185 | 1.742450291 | 0.08142968237 | 1.303876054 |
| pigr | 0.2741780708 | 0.1574549334 | 1.741311402 | 0.08162900871 | 1.315449024 |
| cdh1 | -0.260718421 | 0.1500991296 | -1.736974904 | 0.08239160075 | 0.7704978449 |
| pdzd2 | 0.2789599666 | 0.1607284617 | 1.735597813 | 0.08263497296 | 1.321754429 |
| lmnb1 | 0.2715327043 | 0.1564816308 | 1.735236928 | 0.0826988482 | 1.311973778 |
| btn2a1 | 0.2571302866 | 0.1483914611 | 1.732783576 | 0.08313414309 | 1.293213604 |
| mpo | 0.2549346186 | 0.1475206846 | 1.728127952 | 0.08396528721 | 1.290377251 |
| c2 | -0.254849 | 0.147498618 | -1.727806021 | 0.08402300751 | 0.775033519 |
| itgav | -0.26780353 | 0.1550267239 | -1.727466875 | 0.08408384924 | 0.7650580771 |
| ssc5d | 0.2627123716 | 0.1521670307 | 1.726473668 | 0.08426223253 | 1.300452618 |
| s100a12 | 0.2508573258 | 0.1455055165 | 1.724039966 | 0.08470062844 | 1.285126717 |
| fap | -0.24982414 | 0.1454351299 | -1.717770253 | 0.08583852939 | 0.7789377551 |
| dgcr6 | 0.2682789089 | 0.1564132788 | 1.715192668 | 0.08630990878 | 1.307711822 |
| lrrc38 | -0.278670122 | 0.162736331 | -1.712402636 | 0.08682249355 | 0.75678951 |
| lair1 | 0.2698025191 | 0.1582887248 | 1.704496132 | 0.08828843808 | 1.309705783 |
| adamtsl5 | -0.263977196 | 0.1553644738 | -1.699083387 | 0.08930347318 | 0.7679910525 |
| ccl21 | 0.2492387986 | 0.1467185987 | 1.698753947 | 0.0893655542 | 1.283048387 |
| tmprss5 | 0.2537451175 | 0.1497040632 | 1.694978159 | 0.09007956418 | 1.288843259 |
| cep85 | 0.2445669012 | 0.1446865717 | 1.690322041 | 0.09096636065 | 1.277068097 |
| aplp1 | -0.252828708 | 0.1496167784 | -1.689841948 | 0.09105819609 | 0.7766008956 |
| hs3st3b1 | -0.246668174 | 0.1460137572 | -1.68934886 | 0.09115259507 | 0.781399939 |
| syngap1 | 0.2535333376 | 0.1505439281 | 1.684115333 | 0.09215938174 | 1.288570337 |
| serpinb8 | 0.2514606531 | 0.1494315834 | 1.68278116 | 0.09241746356 | 1.285902303 |
| scgb3a1 | 0.2771487176 | 0.1648589522 | 1.681126283 | 0.09273838828 | 1.319362569 |
| nmrk2 | 0.2653454394 | 0.1578992802 | 1.680472761 | 0.09286536966 | 1.30388131 |
| alms1 | -0.264533566 | 0.1578208628 | -1.676163475 | 0.09370617638 | 0.7675638846 |
| epha1 | 0.2692574628 | 0.1606450273 | 1.67610207 | 0.09371820128 | 1.308992114 |
| ly96 | -0.248940234 | 0.1486068626 | -1.675159746 | 0.09390289313 | 0.7796265669 |
| tnfrsf9 | 0.252117089 | 0.1508069526 | 1.671786908 | 0.09456634933 | 1.286746692 |
| vgf | -0.27017631 | 0.161895879 | -1.668827596 | 0.09515155199 | 0.7632449144 |
| vcpkmt | 0.2644122153 | 0.1584852129 | 1.668371518 | 0.09524199865 | 1.302665064 |
| plaur | 0.2639097965 | 0.1582341129 | 1.667843878 | 0.09534672292 | 1.302010745 |
| fdx2 | 0.2698998447 | 0.1618393778 | 1.667701942 | 0.09537490969 | 1.309833257 |
| il36a | 0.256153284 | 0.1541612677 | 1.661593004 | 0.09659440698 | 1.291950748 |
| gli2 | 0.2593612072 | 0.1561234714 | 1.661256984 | 0.09666184542 | 1.296101882 |
| ca8 | 0.2581775237 | 0.1554585136 | 1.660748696 | 0.09676392883 | 1.294568615 |
| tnfrsf11b | 0.2650982515 | 0.1596267505 | 1.660738258 | 0.09676602605 | 1.303559046 |
| lypd8 | -0.237424405 | 0.1430976868 | -1.659177098 | 0.09708011118 | 0.7886565074 |
| cenpf | 0.2650403066 | 0.1601982992 | 1.654451439 | 0.09803582232 | 1.303483514 |
| amdhd2 | 0.2489707436 | 0.1509831329 | 1.648997068 | 0.09914823534 | 1.282704505 |
| stx8 | 0.2531418773 | 0.1537105401 | 1.646873905 | 0.09958396663 | 1.288066011 |
| sema6c | 0.2551214808 | 0.1550063972 | 1.645877109 | 0.09978906276 | 1.290618397 |
| mad1l1 | 0.2565919821 | 0.1560199067 | 1.644610534 | 0.1000501532 | 1.292517649 |
| lrrn1 | -0.245885458 | 0.149588384 | -1.643747002 | 0.1002284727 | 0.7820117931 |
| cd5l | 0.2511159292 | 0.1529375707 | 1.641950556 | 0.1006002513 | 1.285459098 |
| hs6st2 | 0.2699541741 | 0.1647664389 | 1.638405102 | 0.1013372156 | 1.309904422 |
| ddr1 | 0.239033652 | 0.1461996773 | 1.634980709 | 0.1020530916 | 1.270021275 |
| f2 | 0.2480235553 | 0.1520114738 | 1.631610753 | 0.1027615115 | 1.281490118 |
| tspan1 | 0.2711026686 | 0.1662627198 | 1.630567989 | 0.1029815083 | 1.311409704 |
| gfap | 0.257726613 | 0.1583690764 | 1.627379655 | 0.1036564897 | 1.293985012 |
| prtn3 | 0.2399879433 | 0.1476658898 | 1.625209069 | 0.1041180187 | 1.271233823 |
| col1a1 | -0.233384511 | 0.1439246761 | -1.621573985 | 0.1048945966 | 0.7918490406 |
| kiaa1549l | 0.2528469427 | 0.1559937491 | 1.620878683 | 0.1050436594 | 1.287686172 |
| xcl1 | 0.2343173237 | 0.1445647666 | 1.620846692 | 0.1050505219 | 1.26404554 |
| dipk2b | -0.269945684 | 0.1667420525 | -1.618941831 | 0.1054597793 | 0.7634209594 |
| atp2b4 | 0.2608327561 | 0.161331699 | 1.616748338 | 0.1059326152 | 1.298010563 |
| fgf7 | 0.2639371487 | 0.163369761 | 1.615581409 | 0.1061848461 | 1.302046358 |
| cd8a | 0.2310573916 | 0.1434443697 | 1.610780486 | 0.1072275757 | 1.259931547 |
| cxcl14 | 0.2642680497 | 0.1646025593 | 1.605491742 | 0.108385629 | 1.302477278 |
| impg1 | 0.2454763599 | 0.152988754 | 1.604538592 | 0.108595385 | 1.278230066 |
| igfbp2 | 0.2511948077 | 0.1567044422 | 1.602984601 | 0.108938054 | 1.285560497 |
| shc1 | -0.265861018 | 0.1659694408 | -1.60186729 | 0.1091849592 | 0.7665456559 |
| b3gat3 | 0.2553682209 | 0.1599735317 | 1.596315454 | 0.1104183814 | 1.290936883 |
| tnfrsf11a | 0.2478301832 | 0.155295032 | 1.595866784 | 0.1105185389 | 1.281242337 |
| vcan | -0.234733399 | 0.1470939747 | -1.595805674 | 0.110532186 | 0.7907816444 |
| itih4 | 0.2595259927 | 0.162639601 | 1.595712183 | 0.1105530674 | 1.296315478 |
| rictor | 0.2485008444 | 0.1561201298 | 1.5917284 | 0.1114457441 | 1.282101905 |
| rbp1 | -0.253097577 | 0.15913038 | -1.590504444 | 0.1117211447 | 0.7763921204 |

| Protein | beta | se | z | p_val | HR |
|---|---|---|---|---|---|
| scg3 | 0.2313945502 | 0.1458061485 | 1.58700132 | 0.1125123457 | 1.260356415 |
| tgoln2 | 0.2504466069 | 0.157828611 | 1.586826402 | 0.1125519674 | 1.284598999 |
| selenop | 0.2412663524 | 0.1522753055 | 1.584408921 | 0.1131006931 | 1.27286002 |
| pnma1 | 0.239434697 | 0.1511430072 | 1.584159938 | 0.1131573274 | 1.270530712 |
| asah1 | 0.25331064 | 0.1599048784 | 1.584133283 | 0.1131633917 | 1.288283407 |
| itpa | 0.2562296543 | 0.1618029092 | 1.583591145 | 0.1132867903 | 1.292049419 |
| il6 | 0.2559940128 | 0.1618239248 | 1.581929329 | 0.1136657046 | 1.291744994 |
| agr2 | 0.2429739075 | 0.1537020857 | 1.580810737 | 0.1139213187 | 1.275035355 |
| xg | 0.2806503621 | 0.1777101663 | 1.579258902 | 0.1142766844 | 1.323990606 |
| cd5 | 0.2317298958 | 0.1468793803 | 1.577688409 | 0.1146372105 | 1.260779141 |
| kit | -0.234282113 | 0.1485331116 | -1.57730563 | 0.1147252175 | 0.7911385937 |
| chrdl1 | 0.2547029388 | 0.1615811287 | 1.576316126 | 0.114952967 | 1.290078332 |
| adgrb3 | 0.2252175402 | 0.142915698 | 1.575876852 | 0.1150541866 | 1.252595176 |
| folr1 | 0.2341755852 | 0.1487742953 | 1.574032561 | 0.1154799219 | 1.263866389 |
| agr3 | 0.2324610828 | 0.1478153084 | 1.572645522 | 0.1158009209 | 1.261701344 |
| hdac9 | 0.2417996414 | 0.1540317695 | 1.569803698 | 0.1164607869 | 1.273539003 |
| lypd3 | 0.2270083021 | 0.144628776 | 1.569592915 | 0.1165098479 | 1.254840286 |
| prune2 | 0.2518673637 | 0.160513119 | 1.569138805 | 0.1166155995 | 1.286425399 |
| ebi3_il27 | 0.2313260638 | 0.1478399996 | 1.564705522 | 0.117651973 | 1.260270101 |
| tyrp1 | 0.2365224369 | 0.1512025585 | 1.564275361 | 0.1177529157 | 1.266835979 |
| lair2 | 0.2280230861 | 0.1458638081 | 1.563260202 | 0.1179914051 | 1.256114324 |
| il11 | 0.2269174707 | 0.1452301067 | 1.562468526 | 0.1181776549 | 1.254726312 |
| cfi | 0.2707171388 | 0.1732976334 | 1.562151389 | 0.1182523291 | 1.310904214 |
| bche | -0.252552978 | 0.1618840039 | -1.56008606 | 0.1187395454 | 0.7768150578 |
| vstm2l | 0.2305619648 | 0.1478199088 | 1.559749067 | 0.1188191919 | 1.259307498 |
| irag2 | 0.2235639605 | 0.1436961014 | 1.555810898 | 0.1197530659 | 1.250525622 |
| ccn2 | 0.2264011239 | 0.1455279018 | 1.555723137 | 0.1197739424 | 1.254078605 |
| tk1 | -0.242144927 | 0.1559314372 | -1.552893575 | 0.120448562 | 0.78494241 |
| kif1c | 0.2661863725 | 0.1720003809 | 1.547591762 | 0.1217206161 | 1.304978248 |
| guk1 | 0.250836087 | 0.1621118832 | 1.547302283 | 0.1217903714 | 1.285099423 |
| immt | 0.2468821138 | 0.1605683819 | 1.537551235 | 0.1241583787 | 1.280028206 |
| scara5 | 0.2393314985 | 0.1557028217 | 1.53710444 | 0.124267736 | 1.270399602 |
| dclre1c | -0.242375842 | 0.1579169051 | -1.534831512 | 0.12482522 | 0.7847611757 |
| nectin4 | 0.2198446986 | 0.1436283878 | 1.530649352 | 0.1258560776 | 1.245883228 |
| klk11 | 0.2175877768 | 0.1424999593 | 1.526932202 | 0.126777871 | 1.243074538 |
| cacna1c | 0.2324389445 | 0.1525428058 | 1.523762089 | 0.127568153 | 1.261673412 |
| cxcl1 | 0.2198285739 | 0.1443328809 | 1.523066487 | 0.1277420721 | 1.245863139 |
| ncr1 | -0.230083849 | 0.151094526 | -1.522780837 | 0.1278135457 | 0.7944669846 |
| mki67 | 0.2390735791 | 0.15703038 | 1.522467048 | 0.1278920961 | 1.270071984 |
| pla2g10 | 0.232565612 | 0.15283809 | 1.52164694 | 0.1280975692 | 1.261833235 |
| cd84 | 0.2298303846 | 0.151539609 | 1.516635724 | 0.1293586806 | 1.25838655 |
| ivd | -0.218748606 | 0.144506347 | -1.513764696 | 0.1300855297 | 0.8035236935 |
| satb1 | 0.2370171151 | 0.1566050331 | 1.513470611 | 0.1301601609 | 1.26746281 |
| dll1 | 0.2249280259 | 0.1487323263 | 1.512300866 | 0.1304573408 | 1.252232585 |
| cdc123 | 0.2409247097 | 0.1594064497 | 1.511386209 | 0.130690081 | 1.27242523 |
| htr1b | 0.2271791966 | 0.1506553569 | 1.507939719 | 0.1315699557 | 1.255054749 |
| pnliprp2 | -0.214341775 | 0.1423533291 | -1.505702577 | 0.1321435421 | 0.8070725011 |
| ccl19 | -0.225841777 | 0.1500960173 | -1.504648698 | 0.132414419 | 0.7978443289 |
| micall2 | 0.2417643516 | 0.1608590398 | 1.502957819 | 0.1328499216 | 1.273494061 |
| cd74 | 0.2361396923 | 0.1572321282 | 1.501853946 | 0.1331348326 | 1.266351197 |
| sned1 | 0.2360617607 | 0.1571992391 | 1.501672414 | 0.1331817317 | 1.266252512 |
| crim1 | 0.2266272057 | 0.1511196866 | 1.499653757 | 0.1337041149 | 1.254362162 |
| fgf20 | 0.2294740513 | 0.1530766445 | 1.499079445 | 0.1338530233 | 1.257938225 |
| ctnna1 | 0.2280387983 | 0.1521819015 | 1.498462012 | 0.1340132553 | 1.25613406 |
| polr2f | 0.2339023037 | 0.1562611384 | 1.496868038 | 0.1344275982 | 1.263521045 |
| hao1 | 0.2306357814 | 0.1540841118 | 1.496817411 | 0.1344407745 | 1.259400459 |
| cd3d | 0.2292379606 | 0.1531507726 | 1.496812303 | 0.134442104 | 1.257641273 |
| lrrc59 | 0.2280401708 | 0.1523876954 | 1.496447402 | 0.1345371048 | 1.256135784 |
| glt8d2 | -0.216872418 | 0.1452891275 | -1.492695438 | 0.1355169281 | 0.8050326706 |
| brd2 | 0.2298363532 | 0.1540527911 | 1.491932418 | 0.1357168631 | 1.258394061 |
| gngt1 | 0.2392993793 | 0.1610508689 | 1.485862081 | 0.1373156051 | 1.270358799 |
| stx5 | 0.2332865636 | 0.157237087 | 1.48366119 | 0.1378988271 | 1.262743284 |
| apobr | 0.2255189502 | 0.1523970747 | 1.479811542 | 0.1389235475 | 1.252972778 |
| tgfb2 | -0.225352247 | 0.1525335181 | -1.477394935 | 0.1395698032 | 0.7982349934 |
| dsg2 | 0.2102575867 | 0.1425782082 | 1.474682488 | 0.1402979264 | 1.23399588 |
| sorbs1 | 0.2476952826 | 0.1680668333 | 1.473790383 | 0.1405380384 | 1.281069509 |
| ddx39a | 0.2307196131 | 0.1579873692 | 1.46036746 | 0.1441891108 | 1.259506041 |
| gusb | -0.232994599 | 0.1598990466 | -1.457135638 | 0.1450789427 | 0.7921578518 |
| hsd17b3 | 0.2301125304 | 0.1579453702 | 1.45691216 | 0.145140629 | 1.258741649 |
| rnaset2 | 0.2162937444 | 0.1484749329 | 1.456769437 | 0.1451800351 | 1.241466999 |

| Protein | beta | se | z | p_val | HR |
|---|---|---|---|---|---|
| apbb1ip | 0.2260251964 | 0.1553924381 | 1.454544372 | 0.1457954408 | 1.253607251 |
| h2ap | 0.2317203273 | 0.1599008064 | 1.449150461 | 0.1472955686 | 1.260767077 |
| stx3 | 0.219288154 | 0.1514601024 | 1.447827848 | 0.1476652025 | 1.245190032 |
| morc3 | 0.2230347442 | 0.1544698769 | 1.443872092 | 0.1487749583 | 1.249863999 |
| pla2g2a | 0.2207665482 | 0.1529405086 | 1.443479888 | 0.1488853341 | 1.247032275 |
| tspan15 | 0.2228830171 | 0.1545217664 | 1.442405315 | 0.1491880654 | 1.249674375 |
| bambi | 0.2126011436 | 0.1474255194 | 1.442091874 | 0.1492764571 | 1.236891211 |
| boc | 0.2185095062 | 0.151537996 | 1.441945333 | 0.149317796 | 1.244220844 |
| sytl4 | 0.220433023 | 0.1528890681 | 1.441784071 | 0.149363298 | 1.246616427 |
| cytl1 | 0.2325394946 | 0.161830358 | 1.436933697 | 0.150736834 | 1.26180028 |
| klk13 | 0.2103850811 | 0.1464315955 | 1.436746492 | 0.1507900394 | 1.234153218 |
| dgkz | 0.2082100161 | 0.1449409847 | 1.43651581 | 0.1508556209 | 1.231471771 |
| sh3gl3 | -0.223004528 | 0.1553949523 | -1.43508219 | 0.1512636781 | 0.8001112261 |
| cd99 | 0.2415411622 | 0.168466615 | 1.433762779 | 0.15163997 | 1.273209862 |
| lysmd3 | 0.2269947231 | 0.158580593 | 1.431415527 | 0.152311161 | 1.254823246 |
| cpox | 0.22653209 | 0.1583065863 | 1.430970719 | 0.1524386072 | 1.254242857 |
| btn1a1 | -0.222069291 | 0.155241747 | -1.43047405 | 0.1525810086 | 0.8008598704 |
| sparcl1 | -0.210666695 | 0.1473457228 | -1.429744219 | 0.152790444 | 0.8100440133 |
| ass1 | 0.2466920247 | 0.1726589998 | 1.428781731 | 0.1530669779 | 1.27978491 |
| atp6v1g2 | -0.22682373 | 0.1587984058 | -1.428375358 | 0.153183848 | 0.7970612681 |
| tnr | -0.216741062 | 0.1517612517 | -1.428171289 | 0.1532425624 | 0.8051384229 |
| bst1 | 0.2099259054 | 0.1471964587 | 1.426161385 | 0.1538217631 | 1.233586654 |
| cdon | 0.2119698875 | 0.1487984862 | 1.424543306 | 0.1542892582 | 1.236110662 |
| esam | 0.2098884283 | 0.1475373007 | 1.422612637 | 0.1548484787 | 1.233540424 |
| omp | 0.2204644378 | 0.1550170697 | 1.42219459 | 0.154969769 | 1.24665559 |
| gpc1 | -0.207516405 | 0.1467770403 | -1.413820609 | 0.1574145808 | 0.8125999114 |
| ambp | 0.2158278376 | 0.1528898975 | 1.411655323 | 0.1580514743 | 1.240888726 |
| rnf31 | 0.222409969 | 0.1577089975 | 1.410255423 | 0.1584642758 | 1.249083358 |
| igfbp4 | 0.2306051368 | 0.1635396248 | 1.410087232 | 0.1585139269 | 1.259361866 |
| fut1 | -0.223425944 | 0.1588979278 | -1.406097279 | 0.1596952402 | 0.7997741177 |
| pdzk1 | 0.2324419778 | 0.1653180889 | 1.406028701 | 0.1597156022 | 1.261677239 |
| slamf1 | 0.2044093046 | 0.1454977108 | 1.404897049 | 0.1600518942 | 1.226800186 |
| nudt15 | 0.2240576168 | 0.15951494 | 1.404618382 | 0.1601347876 | 1.251143104 |
| adgrg2 | -0.232146704 | 0.1657107947 | -1.400914797 | 0.1612395534 | 0.7928298032 |
| fgf12 | 0.2156556716 | 0.1541782774 | 1.398742256 | 0.1618902877 | 1.240675106 |
| tgfbr2 | 0.2214534889 | 0.1584902987 | 1.397268418 | 0.1623328691 | 1.247889206 |
| bap18 | 0.2219654227 | 0.1589088352 | 1.396809827 | 0.1624707663 | 1.248528206 |
| cyth3 | -0.224018855 | 0.1604369412 | -1.396304701 | 0.1626227588 | 0.7993000633 |
| uhrf2 | 0.2130134623 | 0.1526325881 | 1.395596215 | 0.1628361229 | 1.237401309 |
| lrrfip1 | 0.2135855769 | 0.1531543837 | 1.394576974 | 0.1631434428 | 1.238109447 |
| gpc5 | -0.203899786 | 0.1462371266 | -1.394309302 | 0.1632242231 | 0.8155440961 |
| cep20 | 0.2013783004 | 0.1445560448 | 1.393081145 | 0.1635952541 | 1.223087379 |
| lta | 0.2141026188 | 0.1537327336 | 1.39269376 | 0.1637124164 | 1.238749767 |
| lrp2 | 0.2244439535 | 0.1613954236 | 1.39064633 | 0.1643326999 | 1.25162656 |
| anxa5 | 0.2064449562 | 0.1484786536 | 1.390401592 | 0.1644069635 | 1.229300067 |
| azu1 | 0.2055070348 | 0.1478955699 | 1.389541519 | 0.1646681449 | 1.228147621 |
| cdk1 | 0.225318611 | 0.1622486169 | 1.388724387 | 0.1649165757 | 1.252721784 |
| vim | 0.2016849224 | 0.1455674033 | 1.385508828 | 0.165896935 | 1.223462462 |
| kcnc4 | 0.2116036931 | 0.1527791018 | 1.385030352 | 0.1660431867 | 1.235658088 |
| entr1 | 0.2205003303 | 0.1592069998 | 1.38499143 | 0.1660550879 | 1.246700337 |
| pth | -0.230784906 | 0.1666555764 | -1.38480158 | 0.1661131476 | 0.7939102133 |
| rbfox3 | 0.2500414243 | 0.1807493101 | 1.383360325 | 0.1665544094 | 1.284078608 |
| ccl18 | 0.217955559 | 0.1576454698 | 1.382567855 | 0.1667974111 | 1.243531803 |
| ntf4 | 0.1961833034 | 0.1421661929 | 1.379957495 | 0.1675997325 | 1.216749919 |
| yap1 | 0.2479063181 | 0.1799939352 | 1.377303729 | 0.1684183629 | 1.281339888 |
| pafah2 | -0.216997878 | 0.158211016 | -1.371572494 | 0.1701965613 | 0.8049316775 |
| il2ra | 0.2019966431 | 0.1475795228 | 1.368730832 | 0.1710834288 | 1.2238439 |
| xrcc4 | -0.1965569 | 0.1437497439 | -1.367354783 | 0.171514128 | 0.8215545836 |
| folh1 | 0.2177986297 | 0.1592937244 | 1.3672769 | 0.1715385293 | 1.243336671 |
| efna1 | 0.20089532 | 0.1470429187 | 1.366235938 | 0.1718649209 | 1.222496794 |
| lrig1 | 0.1960894608 | 0.1438283243 | 1.363357752 | 0.1727697891 | 1.216635742 |
| pcyt2 | 0.2129158891 | 0.156199683 | 1.363100648 | 0.1728507927 | 1.237280578 |
| apoe | 0.2098060042 | 0.1541243 | 1.361277904 | 0.1734258829 | 1.233438755 |
| gsap | 0.1936391841 | 0.1423103237 | 1.360682619 | 0.1736140096 | 1.213658297 |
| tfrc | -0.19952922 | 0.1470791164 | -1.356611497 | 0.1749046868 | 0.8191162857 |
| ctse | -0.217880175 | 0.1607979839 | -1.354993199 | 0.1754197239 | 0.8042218021 |
| mmp12 | 0.213436792 | 0.1575376529 | 1.35483034 | 0.1754716176 | 1.237925249 |
| lrpap1 | 0.2051248927 | 0.1516122304 | 1.352957425 | 0.1760692323 | 1.227678383 |
| gsta3 | 0.2108707593 | 0.1560624287 | 1.35119491 | 0.1766330047 | 1.234752764 |
| vti1a | 0.2176880871 | 0.1611916767 | 1.350492107 | 0.1768581838 | 1.243199237 |

| Protein | beta | se | z | p_val | HR |
|---|---|---|---|---|---|
| jchain | 0.1944152636 | 0.1440036884 | 1.350071417 | 0.1769930758 | 1.214600558 |
| nme3 | -0.19505322 | 0.1446132463 | -1.348792211 | 0.1774037172 | 0.8227908678 |
| tfpi2 | 0.2049044391 | 0.1519810884 | 1.348223264 | 0.1775865843 | 1.227407767 |
| nampt | 0.1944770804 | 0.1442959481 | 1.347765359 | 0.1777338628 | 1.214675643 |
| map3k5 | 0.194206143 | 0.1441956099 | 1.346824242 | 0.1780368454 | 1.214346586 |
| aif1 | 0.1979158792 | 0.1470839666 | 1.345597917 | 0.1784322237 | 1.218859858 |
| cst3 | 0.2172915675 | 0.1616665224 | 1.344072752 | 0.1789248624 | 1.242706382 |
| postn | 0.2095213122 | 0.1562626965 | 1.340827446 | 0.1799764831 | 1.233087655 |
| cfd | 0.2231068058 | 0.1664721115 | 1.340205298 | 0.1801786098 | 1.249954069 |
| dpep1 | -0.197190759 | 0.1475808737 | -1.33615389 | 0.1814989787 | 0.8210339991 |
| ido1 | 0.207821615 | 0.1555956273 | 1.335652027 | 0.1816630365 | 1.230993559 |
| spint2 | 0.1962072415 | 0.1469529715 | 1.33517029 | 0.1818206185 | 1.216779046 |
| kiaa2013 | -0.206115925 | 0.1549134519 | -1.330523091 | 0.1833459834 | 0.8137387382 |
| grk5 | -0.191839056 | 0.1442807288 | -1.329623561 | 0.1836423312 | 0.825439707 |
| krt17 | 0.2122560875 | 0.1596651199 | 1.329382946 | 0.1837216612 | 1.236464488 |
| rassf2 | 0.1958959229 | 0.147405844 | 1.328956286 | 0.1838623925 | 1.216400299 |
| cd63 | 0.1941843853 | 0.1465933829 | 1.32464632 | 0.185288485 | 1.214320165 |
| aldh2 | 0.2058239943 | 0.1555559436 | 1.323150948 | 0.1857851845 | 1.228536955 |
| myo9b | 0.189491875 | 0.1433118587 | 1.322234439 | 0.1860900964 | 1.208635304 |
| ly9 | -0.190785374 | 0.1443283156 | -1.321884577 | 0.1862065889 | 0.8263099164 |
| gfral | -0.216273573 | 0.1638421016 | -1.320012201 | 0.1868309443 | 0.8055149044 |
| heg1 | 0.2182948114 | 0.1655651569 | 1.318482799 | 0.1873420796 | 1.243953745 |
| dhrs4l2 | 0.2040787428 | 0.1547977245 | 1.318357511 | 0.1873839971 | 1.226394719 |
| casq2 | -0.208785273 | 0.1586036121 | -1.316396709 | 0.1880409263 | 0.8115694828 |
| cpxm2 | 0.2132924415 | 0.1623829066 | 1.313515357 | 0.1890093497 | 1.237746567 |
| nfx1 | 0.2091294671 | 0.1594727051 | 1.311380947 | 0.1897290926 | 1.23260457 |
| tnfsf12 | -0.190628652 | 0.1456092825 | -1.309179257 | 0.1904736369 | 0.8264394276 |
| itga11 | -0.203787021 | 0.1556923235 | -1.3089086 | 0.1905653133 | 0.8156360659 |
| cend1 | 0.2011514884 | 0.1540089135 | 1.306102899 | 0.1915175673 | 1.222809999 |
| tpp1 | 0.1957360096 | 0.1501246639 | 1.303823133 | 0.1922938934 | 1.216205796 |
| ccne1 | 0.2046972794 | 0.1571146785 | 1.302852676 | 0.192625063 | 1.227153524 |
| timd4 | 0.190705154 | 0.1464126354 | 1.302518416 | 0.1927392266 | 1.210102606 |
| prkcq | 0.1878636808 | 0.1443048342 | 1.301852996 | 0.1929666431 | 1.206669012 |
| dhodh | -0.207510584 | 0.1594323954 | -1.301558464 | 0.1930673665 | 0.8126046414 |
| sdc1 | -0.190968266 | 0.146905583 | -1.299938791 | 0.1936219485 | 0.826158805 |
| anxa2 | 0.2020915534 | 0.1555405941 | 1.299284952 | 0.1938461565 | 1.223960061 |
| epgn | -0.198626525 | 0.1529524659 | -1.298616039 | 0.1940757309 | 0.8198560315 |
| phykpl | 0.2076040565 | 0.160272887 | 1.295316135 | 0.1952111955 | 1.230725775 |
| pdia2 | 0.2097060377 | 0.1620415631 | 1.294149684 | 0.1956137227 | 1.233315458 |
| cpe | -0.183914604 | 0.1422630365 | -1.292778565 | 0.1960876559 | 0.8320068507 |
| s100p | 0.1963172292 | 0.1518745354 | 1.292627686 | 0.1961398594 | 1.216912884 |
| fn1 | 0.2075490539 | 0.1606145687 | 1.292218107 | 0.1962816228 | 1.230658084 |
| shbg | -0.228709137 | 0.1775185014 | -1.288367892 | 0.1976179299 | 0.7955598984 |
| ube2z | 0.1987276777 | 0.1542643038 | 1.288228533 | 0.1976664224 | 1.219849728 |
| il5 | -0.183934615 | 0.1428161187 | -1.287912156 | 0.1977765434 | 0.8319902015 |
| gabarap | 0.202650292 | 0.1574353303 | 1.287197045 | 0.1980256169 | 1.224644125 |
| arnt | 0.185695498 | 0.1442930292 | 1.286933257 | 0.1981175524 | 1.204055567 |
| cd59 | 0.2102218582 | 0.1634053356 | 1.286505471 | 0.1982667108 | 1.233951792 |
| fcrl3 | 0.1799483416 | 0.1398980191 | 1.28628227 | 0.1983445678 | 1.197155518 |
| sema4c | 0.1909967339 | 0.1487303094 | 1.284181649 | 0.1990784047 | 1.210455499 |
| nhlrc3 | 0.2202485154 | 0.1715468301 | 1.28389732 | 0.1991778852 | 1.246386438 |
| clu | 0.201852744 | 0.157329681 | 1.28299214 | 0.1994948299 | 1.223667802 |
| bhmt2 | 0.2013791188 | 0.1570107638 | 1.282581613 | 0.1996386953 | 1.22308838 |
| met | -0.193127365 | 0.1507722469 | -1.280921183 | 0.2002213517 | 0.8243769708 |
| stx4 | 0.1917151797 | 0.1497662014 | 1.28009643 | 0.2005112242 | 1.211325458 |
| tgfb1 | 0.1900507048 | 0.1485535446 | 1.279341434 | 0.2007768477 | 1.209310914 |
| c1qbp | -0.192745429 | 0.1507257538 | -1.278782319 | 0.200973722 | 0.8246918901 |
| mln | -0.205174112 | 0.160455572 | -1.278697333 | 0.2010036592 | 0.8145054889 |
| acrv1 | -0.306545446 | 0.2400850396 | -1.276820273 | 0.2016657065 | 0.73598507 |
| rcor1 | 0.1895637453 | 0.1485079975 | 1.276454794 | 0.2017947972 | 1.208722172 |
| m6pr | 0.1951047998 | 0.1528739196 | 1.276246467 | 0.2018684074 | 1.215438357 |
| sparc | 0.1824225831 | 0.1429879932 | 1.27578952 | 0.2020299333 | 1.200121238 |
| ccl7 | 0.2051170057 | 0.1607810345 | 1.275753738 | 0.2020425859 | 1.227668701 |
| cd3e | 0.201352021 | 0.1580646386 | 1.273858738 | 0.2027134841 | 1.223055237 |
| prkab1 | 0.187541042 | 0.1473532254 | 1.272731163 | 0.2031134554 | 1.206279756 |
| commd9 | 0.1998063516 | 0.1579044064 | 1.265362735 | 0.2057413272 | 1.221166258 |
| ceacam5 | -0.185352194 | 0.1466652691 | -1.263777004 | 0.2063100773 | 0.8308116252 |
| wasl | 0.1999100153 | 0.1585586616 | 1.2607953 | 0.2073826077 | 1.221292856 |
| hmgcs1 | 0.199605662 | 0.1583688931 | 1.260384272 | 0.2075307726 | 1.220921208 |
| insl4 | 0.2011953437 | 0.159710077 | 1.259753595 | 0.2077582646 | 1.222863627 |

| Protein | beta | se | z | p_val | HR |
|---|---|---|---|---|---|
| ssh3 | 0.2011842455 | 0.1598143941 | 1.258861861 | 0.2080802317 | 1.222850056 |
| ifng | 0.178814065 | 0.1420819754 | 1.258527441 | 0.2082010696 | 1.195798383 |
| mansc1 | 0.1809105987 | 0.143851433 | 1.257621109 | 0.2085288154 | 1.198308044 |
| nagk | 0.1996347254 | 0.1590268285 | 1.255352491 | 0.2093508277 | 1.220956692 |
| ccl24 | -0.18278665 | 0.1459295633 | -1.25256765 | 0.2103630943 | 0.8329458457 |
| atp1b4 | 0.1997647966 | 0.1595038292 | 1.252413799 | 0.2104191208 | 1.221115514 |
| lrg1 | 0.1972305023 | 0.1577183428 | 1.250523552 | 0.2111083573 | 1.218024766 |
| duox2 | -0.181055584 | 0.1448611021 | -1.249856457 | 0.2113519882 | 0.8343889789 |
| slc51b | -0.202590807 | 0.1620926074 | -1.24984606 | 0.2113557868 | 0.8166123253 |
| dipk1c | -0.200393302 | 0.1603689722 | -1.249576516 | 0.2114542862 | 0.8184088083 |
| ifnw1 | 0.1962603697 | 0.1570945475 | 1.249313696 | 0.2115503605 | 1.216843693 |
| fut3_fut5 | 0.1857578363 | 0.1489169682 | 1.24739201 | 0.2122537949 | 1.204130628 |
| cmip | 0.1896534073 | 0.1520876319 | 1.247000856 | 0.2123971836 | 1.208830553 |
| ptgds | 0.191385245 | 0.1536799846 | 1.245349195 | 0.2130034199 | 1.210925865 |
| chga | 0.1918232821 | 0.1542459747 | 1.243619371 | 0.2136396832 | 1.211456412 |
| hip1r | 0.2003177245 | 0.1614487583 | 1.2407511 | 0.2146977106 | 1.221790889 |
| mtr | 0.201496307 | 0.162751479 | 1.238061296 | 0.2156933335 | 1.22323172 |
| nfatc3 | -0.180722098 | 0.1459981519 | -1.237838256 | 0.2157760403 | 0.8346672825 |
| defa1_defa1b | 0.1815851836 | 0.1469029418 | 1.236089498 | 0.2164252981 | 1.199116677 |
| col28a1 | 0.2017690226 | 0.1633149092 | 1.235459908 | 0.2166593887 | 1.22356536 |
| akap12 | -0.19674127 | 0.1595699331 | -1.232946999 | 0.2175955391 | 0.8214031274 |
| spink1 | 0.1829608448 | 0.1485165883 | 1.231921948 | 0.2179782418 | 1.200767391 |
| cit | -0.200498238 | 0.162904115 | -1.230774544 | 0.2184071988 | 0.818322932 |
| creld1 | -0.207146498 | 0.1683083206 | -1.230756135 | 0.218414086 | 0.8129005528 |
| zbtb17 | 0.1853047798 | 0.1508804631 | 1.228156224 | 0.2193883283 | 1.203585213 |
| fgfbp3 | -0.194700033 | 0.1586025766 | -1.227596912 | 0.2195983216 | 0.823081518 |
| a1bg | 0.2032963521 | 0.1656425791 | 1.227319408 | 0.2197025639 | 1.225435575 |
| ptprb | 0.1922554922 | 0.1567125743 | 1.226803229 | 0.219896557 | 1.211980129 |
| lep | 0.3153414594 | 0.2575726141 | 1.224281784 | 0.2208459464 | 1.370727279 |
| vsig10l | -0.191680323 | 0.1566536015 | -1.223593465 | 0.2211056261 | 0.8255707423 |
| gchfr | -0.213834109 | 0.1748914591 | -1.222667531 | 0.2214552952 | 0.8074823283 |
| psapl1 | -0.191001243 | 0.1563178537 | -1.221877339 | 0.2217540161 | 0.8261315611 |
| pagr1 | 0.1902589947 | 0.1558829921 | 1.220524395 | 0.2222661475 | 1.209562827 |
| sod3 | -0.207679044 | 0.1703153861 | -1.219379228 | 0.2227002903 | 0.8124677613 |
| sumf1 | 0.1928468663 | 0.1582697668 | 1.218469391 | 0.2230456497 | 1.212697075 |
| mthfsd | -0.192876945 | 0.1583472888 | -1.218062822 | 0.2232001005 | 0.8245834366 |
| tsnax | 0.1978422438 | 0.1624332915 | 1.217990733 | 0.2232274944 | 1.21877011 |
| csh1 | 0.1858773449 | 0.1526452894 | 1.217707704 | 0.2233350683 | 1.204274541 |
| hsd11b1 | -0.193000097 | 0.1585050334 | -1.217627559 | 0.2233655368 | 0.8244818942 |
| cd6 | -0.174286272 | 0.1433740203 | -1.21560567 | 0.2241351734 | 0.8400563786 |
| hsbp1 | -0.186327064 | 0.1536085776 | -1.212999085 | 0.2251301704 | 0.8300020867 |
| gamt | 0.1923605625 | 0.1588220308 | 1.211170525 | 0.2258300566 | 1.212107479 |
| coro1a | 0.1739804017 | 0.1437805934 | 1.210040921 | 0.2262631909 | 1.190032243 |
| pglyrp1 | 0.1752456254 | 0.1448302552 | 1.210007019 | 0.2262761994 | 1.191538853 |
| clec7a | 0.1747047057 | 0.1446509818 | 1.207767162 | 0.227136836 | 1.1908945 |
| ppm1b | 0.1899699583 | 0.1573107766 | 1.20760931 | 0.2271975766 | 1.20921327 |
| prelp | 0.1792261655 | 0.1485155493 | 1.206783845 | 0.2275153993 | 1.196291273 |
| nfkb2 | -0.195498141 | 0.1620485436 | -1.206417145 | 0.2276566884 | 0.8224248721 |
| ghr | -0.212955653 | 0.176548873 | -1.206213609 | 0.2277351379 | 0.8081919771 |
| fgf21 | 0.187310434 | 0.1552973013 | 1.206140946 | 0.2277631492 | 1.206001611 |
| cox6b1 | 0.1843089951 | 0.152965176 | 1.204908202 | 0.228238742 | 1.202387297 |
| krt18 | 0.1951482132 | 0.1621705954 | 1.203351401 | 0.2288403659 | 1.215491125 |
| il17d | 0.192126219 | 0.1596802142 | 1.203193645 | 0.2289013935 | 1.211823462 |
| spink6 | 0.1814758203 | 0.1508367337 | 1.203127486 | 0.2289269903 | 1.198985545 |
| brdt | 0.1948489592 | 0.1621368571 | 1.201756113 | 0.2294580335 | 1.215127439 |
| tbc1d5 | 0.1787300248 | 0.1487630557 | 1.201440935 | 0.229580205 | 1.195697892 |
| omg | -0.178444475 | 0.1485547711 | -1.201203258 | 0.2296723661 | 0.8365705062 |
| clns1a | 0.1911236034 | 0.1591269195 | 1.201076499 | 0.2297215282 | 1.210609078 |
| scin | 0.1843544056 | 0.1537317595 | 1.199195314 | 0.2304520091 | 1.2024419 |
| bcl7a | 0.1895146108 | 0.1582521461 | 1.19754844 | 0.2310928589 | 1.208662783 |
| bcl2l15 | 0.1923682324 | 0.1606716691 | 1.197275372 | 0.2311992405 | 1.212116775 |
| scpep1 | 0.1900528885 | 0.158968879 | 1.195535187 | 0.2318779972 | 1.209313555 |
| pcdh1 | 0.1785040777 | 0.1493276049 | 1.195385661 | 0.2319363854 | 1.195427758 |
| lsm1 | 0.1700082193 | 0.1422307232 | 1.195298846 | 0.2319702903 | 1.185314594 |
| prg2 | 0.1850693331 | 0.1550386944 | 1.193697701 | 0.2325962401 | 1.203301866 |
| spink4 | 0.1848701086 | 0.1552844515 | 1.19052556 | 0.2338398899 | 1.203062163 |
| fam171b | 0.1842857573 | 0.1549526347 | 1.1893038 | 0.2343201404 | 1.202359357 |
| pdcl2 | 0.1869272467 | 0.1572163467 | 1.188980985 | 0.2344471497 | 1.205539575 |
| lxn | -0.172243722 | 0.1450900342 | -1.187150605 | 0.2351682205 | 0.8417739895 |
| ccdc50 | 0.1844042499 | 0.1554360314 | 1.186367461 | 0.235477216 | 1.202501836 |

| Protein | beta | se | z | p_val | HR |
|---|---|---|---|---|---|
| cdh22 | -0.186228584 | 0.1570168265 | -1.186042209 | 0.2356056311 | 0.8300838297 |
| lilra5 | 0.1764901583 | 0.1488555266 | 1.185647335 | 0.2357616009 | 1.193022685 |
| txndc9 | 0.1801544365 | 0.1521949294 | 1.183708533 | 0.23652846 | 1.197402272 |
| tagln3 | -0.192578844 | 0.1627121827 | -1.183555162 | 0.2365891984 | 0.824829283 |
| nt5e | 0.1732050629 | 0.1463659445 | 1.183369967 | 0.2366625547 | 1.189109922 |
| erbb2 | -0.186014774 | 0.1572905995 | -1.182618507 | 0.2369603744 | 0.8302613287 |
| lepr | -0.180817723 | 0.1530192639 | -1.1816664 | 0.2373380956 | 0.8345874712 |
| zp4 | 0.187233841 | 0.1584831501 | 1.181411657 | 0.2374392292 | 1.205909243 |
| scrn1 | 0.1810944287 | 0.1533345879 | 1.181040959 | 0.2375864526 | 1.198528349 |
| ca7 | 0.18612571 | 0.1576794898 | 1.180405329 | 0.2378390436 | 1.204573678 |
| nrcam | -0.169378971 | 0.1435153368 | -1.180215123 | 0.2379146656 | 0.8441889197 |
| myh4 | 0.1885267795 | 0.1598557723 | 1.17935547 | 0.2382566589 | 1.207469418 |
| jmjd1c | 0.1901282589 | 0.1612918777 | 1.178783839 | 0.238484261 | 1.209404705 |
| ndst1 | 0.181884657 | 0.154346816 | 1.178415349 | 0.2386310616 | 1.199475835 |
| tcl1b | 0.167732177 | 0.1423587481 | 1.178235825 | 0.2387026042 | 1.182619835 |
| upk3bl1 | 0.1847413196 | 0.1567993411 | 1.178202143 | 0.2387160286 | 1.202907231 |
| lrch4 | 0.1912111171 | 0.1624065054 | 1.177361194 | 0.2390513717 | 1.210715028 |
| morn4 | 0.1907370297 | 0.1622163057 | 1.175819094 | 0.2396671746 | 1.210141179 |
| il4 | -0.165671626 | 0.1411973696 | -1.173333656 | 0.2406620309 | 0.847324428 |
| eng | -0.167664039 | 0.1429292597 | -1.17305609 | 0.2407733134 | 0.8456378887 |
| tnfrsf10c | 0.1690496977 | 0.1442522551 | 1.171903327 | 0.2412358713 | 1.184178988 |
| egfl7 | 0.1772137354 | 0.1514637617 | 1.170007488 | 0.2419979554 | 1.193886242 |
| tlr4 | 0.1839228498 | 0.1572212956 | 1.169834208 | 0.2420676947 | 1.201923091 |
| lhb | -0.186359905 | 0.1593649372 | -1.169390885 | 0.2422461807 | 0.8299748292 |
| shisa5 | 0.2020011303 | 0.172793378 | 1.169032822 | 0.2423904076 | 1.223849391 |
| igfbp7 | 0.1774861714 | 0.1519060181 | 1.168394601 | 0.2426476318 | 1.194211543 |
| lilrb1 | 0.1706048857 | 0.1460374426 | 1.168227015 | 0.2427152062 | 1.186022042 |
| snap25 | 0.1807867058 | 0.1547570669 | 1.168196771 | 0.2427274027 | 1.198159592 |
| sod1 | -0.17176591 | 0.1471364233 | -1.16739218 | 0.2430520275 | 0.8421762951 |
| st3gal1 | 0.1777898572 | 0.1527110222 | 1.164224131 | 0.2443331933 | 1.194574264 |
| psmd1 | 0.1875240254 | 0.1610842747 | 1.164136138 | 0.2443688456 | 1.20625923 |
| gadd45b | -0.183475861 | 0.157732845 | -1.163206438 | 0.2447457553 | 0.8323719684 |
| cct5 | 0.1662328728 | 0.1429126912 | 1.163177821 | 0.244757363 | 1.180848057 |
| tmem106a | 0.1795145136 | 0.1543905402 | 1.162730005 | 0.2449390638 | 1.196636271 |
| map1lc3b2 | 0.1851120845 | 0.1593180324 | 1.161902903 | 0.2452749076 | 1.20335331 |
| mag | 0.1849172134 | 0.1593265999 | 1.160617333 | 0.2457975536 | 1.203118834 |
| calcoco2 | 0.1786570214 | 0.1539731067 | 1.160313156 | 0.2459213301 | 1.195610605 |
| arid3a | 0.1768757611 | 0.1524526833 | 1.160201036 | 0.2459669654 | 1.193482807 |
| il22 | 0.1885847775 | 0.1627220104 | 1.158938345 | 0.2464813173 | 1.207539451 |
| rrp15 | 0.1834412169 | 0.1582974005 | 1.158839099 | 0.246521777 | 1.201344345 |
| gh2 | 0.1656277077 | 0.1429997945 | 1.158237383 | 0.2467671767 | 1.180133665 |
| mcts1 | 0.1758553716 | 0.1518985968 | 1.157715577 | 0.246980125 | 1.192265611 |
| nmi | 0.1776994927 | 0.1537141948 | 1.156038276 | 0.2476655012 | 1.194466321 |
| itgb6 | -0.177502772 | 0.1537799389 | -1.154264812 | 0.248391618 | 0.8373586779 |
| brme1 | 0.1781482957 | 0.1544835945 | 1.153185853 | 0.2488341088 | 1.195002522 |
| galnt3 | 0.1665633664 | 0.1447947166 | 1.150341465 | 0.250003259 | 1.181238384 |
| cdh3 | 0.1671232691 | 0.1452927993 | 1.150251561 | 0.2500402754 | 1.181899948 |
| tff3 | 0.179638481 | 0.1562599828 | 1.14961283 | 0.2503033717 | 1.196784624 |
| amotl2 | 0.1790346615 | 0.1557828568 | 1.149257789 | 0.2504496982 | 1.196062201 |
| mmp10 | 0.1657995138 | 0.1442867928 | 1.149096952 | 0.2505160052 | 1.180336437 |
| khdc3l | 0.18075865 | 0.1573279813 | 1.148928808 | 0.2505853378 | 1.198125977 |
| ctsv | -0.174283945 | 0.151716155 | -1.148750081 | 0.2506590487 | 0.8400583332 |
| cd40 | 0.1694928541 | 0.1476066258 | 1.148274023 | 0.2508554593 | 1.184703881 |
| ly6d | 0.168769117 | 0.1470713589 | 1.147532179 | 0.2511617415 | 1.183846777 |
| edem2 | 0.1816780862 | 0.1584574762 | 1.146541587 | 0.2515711302 | 1.199228084 |
| ppp1cc | 0.1759104841 | 0.153473275 | 1.146196196 | 0.2517139814 | 1.192331321 |
| pamr1 | -0.17937223 | 0.1567851704 | -1.144063751 | 0.2525971991 | 0.8357947334 |
| bpifb2 | -0.189472414 | 0.1656321791 | -1.143934805 | 0.2526506756 | 0.8273955408 |
| grn | 0.167474389 | 0.1465004392 | 1.143166464 | 0.2529694833 | 1.18231501 |
| casc3 | 0.1850817586 | 0.1620810076 | 1.141908983 | 0.2534918542 | 1.203316818 |
| klk4 | 0.1740562545 | 0.1525044917 | 1.141318872 | 0.2537372515 | 1.190122514 |
| col9a1 | -0.169927541 | 0.1489589685 | -1.140767439 | 0.253966714 | 0.8437259499 |
| igfbp6 | 0.19590694 | 0.1718791183 | 1.139794886 | 0.2543717648 | 1.216413701 |
| efna4 | 0.1722194505 | 0.1512005277 | 1.139013554 | 0.2546975008 | 1.187938498 |
| kcnh2 | -0.177917833 | 0.1564645527 | -1.137112717 | 0.2554911683 | 0.8370111957 |
| stab2 | -0.183903874 | 0.1617654395 | -1.136855153 | 0.2555988426 | 0.8320157787 |
| npc2 | 0.1954213116 | 0.172064491 | 1.135744572 | 0.2560634808 | 1.215823119 |
| atraid | 0.1837143164 | 0.1617875684 | 1.135528015 | 0.2561541505 | 1.201672476 |
| mansc4 | -0.180554054 | 0.1591376201 | -1.134578069 | 0.2565521463 | 0.834807555 |
| s100g | 0.1792581788 | 0.1580609002 | 1.1341083 | 0.2567491226 | 1.196329571 |

| Protein | beta | se | z | p_val | HR |
|---|---|---|---|---|---|
| efemp1 | -0.178721818 | 0.1575949351 | -1.134058131 | 0.2567701646 | 0.8363385217 |
| col4a1 | 0.1665237833 | 0.1470181002 | 1.132675385 | 0.2573505983 | 1.181191628 |
| scn2b | -0.179433581 | 0.1584516761 | -1.132418324 | 0.2574586048 | 0.835743458 |
| pdxdc1 | 0.1808291486 | 0.159898887 | 1.130896856 | 0.2580985067 | 1.198210446 |
| fstl1 | -0.177094181 | 0.156683523 | -1.130266778 | 0.2583638291 | 0.8377008856 |
| c1galt1c1 | 0.1766410341 | 0.1563876482 | 1.12950758 | 0.2586837737 | 1.193202697 |
| fzd10 | -0.182212764 | 0.1614760223 | -1.128419945 | 0.2591426084 | 0.8334239988 |
| aoc1 | -0.162258687 | 0.1438023041 | -1.12834553 | 0.2591740222 | 0.8502212349 |
| adamts4 | 0.184975722 | 0.1640173789 | 1.127781234 | 0.2594123205 | 1.203189229 |
| crebzf | 0.1826421114 | 0.1620478901 | 1.127087254 | 0.2597055918 | 1.200384727 |
| chl1 | -0.166549754 | 0.1478059903 | -1.126813288 | 0.2598214311 | 0.8465806951 |
| pam | 0.1619459038 | 0.1438047719 | 1.126151112 | 0.2601015624 | 1.175796634 |
| cd82 | -0.174577031 | 0.1555117923 | -1.12259674 | 0.2616088008 | 0.8398121601 |
| rln2 | 0.1997624151 | 0.1781615168 | 1.121243345 | 0.262184294 | 1.221112606 |
| ca4 | 0.1607810913 | 0.1434194293 | 1.121055161 | 0.2622643834 | 1.174427848 |
| txnl1 | -0.174590596 | 0.1557513682 | -1.120957062 | 0.2623061397 | 0.839800768 |
| tada3 | 0.1737412264 | 0.1550937376 | 1.120233667 | 0.2626142005 | 1.189747651 |
| tie1 | 0.160343659 | 0.1432500491 | 1.119327078 | 0.2630006278 | 1.173914228 |
| dcdc2c | -0.175415563 | 0.1567997054 | -1.118723804 | 0.2632579867 | 0.8391082461 |
| dda1 | 0.1780595631 | 0.1592157334 | 1.118354068 | 0.2634158035 | 1.194896491 |
| rpe | -0.18001993 | 0.1612472865 | -1.116421456 | 0.2642417735 | 0.8352535643 |
| nfkb1 | 0.1789912233 | 0.1604279014 | 1.115711305 | 0.2645457298 | 1.196010247 |
| gfra3 | 0.163409026 | 0.1468257273 | 1.112945456 | 0.2657318559 | 1.177518227 |
| col5a1 | -0.174702927 | 0.1571214312 | -1.111897504 | 0.2661822219 | 0.8397064376 |
| il17a | 0.1589603579 | 0.1430763177 | 1.111017955 | 0.2665606209 | 1.172291474 |
| mme | 0.1678237561 | 0.1510568126 | 1.110997599 | 0.2665693828 | 1.182728144 |
| mfge8 | 0.1636897755 | 0.1473806628 | 1.110659786 | 0.2667148179 | 1.177848861 |
| il21r | 0.1704653956 | 0.1536819366 | 1.109209055 | 0.2673400073 | 1.185856615 |
| dcn | 0.1690529567 | 0.152412707 | 1.109178887 | 0.2673530188 | 1.184182848 |
| bmper | -0.174980529 | 0.1578149911 | -1.108770007 | 0.2675294124 | 0.8394733662 |
| tff1 | 0.1706085012 | 0.1538770721 | 1.108732437 | 0.2675456244 | 1.18602633 |
| fgr | 0.1627433336 | 0.1468022445 | 1.108588865 | 0.2676075837 | 1.176734623 |
| il6st | -0.160950198 | 0.1451913871 | -1.108538191 | 0.2676294548 | 0.8513344685 |
| ugdh | -0.176208598 | 0.1590075166 | -1.108177787 | 0.2677850419 | 0.8384430676 |
| tnpo1 | -0.174861502 | 0.1580209367 | -1.106571735 | 0.2684791335 | 0.8395732919 |
| bsnd | 0.1787922925 | 0.161792326 | 1.105072762 | 0.2691280621 | 1.195772348 |
| scarf2 | 0.1791364091 | 0.1622480259 | 1.104089915 | 0.2695541357 | 1.196183903 |
| serpind1 | 0.1801366722 | 0.1633405772 | 1.102828674 | 0.2701015733 | 1.197381001 |
| lama4 | 0.1597649168 | 0.1449156171 | 1.102468595 | 0.2702580043 | 1.173235031 |
| scrg1 | 0.1926126888 | 0.1747664854 | 1.102114564 | 0.2704118686 | 1.212413121 |
| arhgap5 | -0.173802639 | 0.1577454572 | -1.101791724 | 0.2705522292 | 0.8404627557 |
| spink8 | 0.1749181419 | 0.1591830659 | 1.098848932 | 0.2718339649 | 1.191148707 |
| f13b | 0.1708683236 | 0.1556749475 | 1.097596796 | 0.2723805926 | 1.186334526 |
| hepacam2 | -0.168772865 | 0.1539416731 | -1.09634293 | 0.272928729 | 0.8447007428 |
| mfap4 | -0.178900974 | 0.1632894887 | -1.095606184 | 0.2732511542 | 0.8361887 |
| nid2 | 0.1559754569 | 0.1425178306 | 1.094427668 | 0.2737674548 | 1.168797517 |
| ephx2 | 0.1604145603 | 0.1466646712 | 1.093750519 | 0.274064411 | 1.173997463 |
| c19orf12 | 0.1680844381 | 0.1537942131 | 1.092917833 | 0.2744298779 | 1.1830365 |
| insl3 | -0.265687663 | 0.2431138898 | -1.092852667 | 0.2744584931 | 0.7666785523 |
| furin | 0.1790773277 | 0.163931698 | 1.092389879 | 0.2746617698 | 1.196113233 |
| il7r | -0.161188935 | 0.1475975167 | -1.092084331 | 0.2747960361 | 0.8511312472 |
| guca2a | 0.1607078893 | 0.14715837 | 1.092074405 | 0.2748003986 | 1.174341881 |
| hddc2 | -0.168642884 | 0.1545188299 | -1.091406686 | 0.2750939717 | 0.8448105449 |
| ctag1a_ctag1b | 0.1708431478 | 0.1565816088 | 1.09108055 | 0.2752374405 | 1.18630466 |
| bag4 | 0.1737059583 | 0.1592265444 | 1.09093593 | 0.2753010757 | 1.189705691 |
| afm | 0.1796627309 | 0.1647618275 | 1.090439052 | 0.2755197875 | 1.196813647 |
| pdcd6 | -0.155412955 | 0.1425481052 | -1.090249178 | 0.2756033956 | 0.85606159 |
| npy | -0.158023615 | 0.1450962887 | -1.089094809 | 0.2761120787 | 0.8538296186 |
| psmd5 | 0.1725493719 | 0.1584617367 | 1.088902441 | 0.2761969097 | 1.188330489 |
| angpt2 | 0.1662764044 | 0.1528027133 | 1.088177041 | 0.2765169584 | 1.180899462 |
| hspa2 | 0.1741935352 | 0.1601374656 | 1.087775022 | 0.2766944388 | 1.190285906 |
| atp5f1d | -0.17293198 | 0.1591889156 | -1.086331793 | 0.2773322261 | 0.8411948309 |
| il17ra | -0.156865816 | 0.1444205019 | -1.08617415 | 0.2774019516 | 0.8548187542 |
| timp2 | -0.167505462 | 0.154326077 | -1.0853996 | 0.2777447094 | 0.8457719974 |
| camsap1 | 0.1672172795 | 0.1542215463 | 1.08426665 | 0.2782465874 | 1.182011064 |
| tnfrsf10a | 0.1698930833 | 0.1567148247 | 1.084090696 | 0.2783245878 | 1.185178129 |
| hpse | 0.1679605725 | 0.1549940889 | 1.083657923 | 0.2785164985 | 1.182889971 |
| acy3 | 0.1790605863 | 0.1652565949 | 1.083530654 | 0.2785729523 | 1.196093209 |
| kctd5 | 0.1729994291 | 0.1597025029 | 1.0832606 | 0.2786927684 | 1.188865426 |
| cd200 | -0.169388416 | 0.1565760865 | -1.081828135 | 0.2793289023 | 0.8441809466 |

| Protein | beta | se | z | p_val | HR |
|---|---|---|---|---|---|
| itpr1 | 0.1682313186 | 0.1555643 | 1.081426256 | 0.2795075474 | 1.183210277 |
| ada | -0.159843387 | 0.1478904145 | -1.080823175 | 0.2797757777 | 0.852277256 |
| doc2b | 0.1716203702 | 0.1587989254 | 1.080740123 | 0.2798127301 | 1.187227041 |
| rps10 | 0.1714368471 | 0.1586383435 | 1.080677239 | 0.279840711 | 1.187009177 |
| gpnmb | 0.1576191228 | 0.146142763 | 1.078528417 | 0.2807980073 | 1.170720209 |
| necap2 | -0.166553548 | 0.1544997771 | -1.078018047 | 0.2810257025 | 0.8465774833 |
| il1rn | 0.1826573853 | 0.1699838189 | 1.074557487 | 0.2825728941 | 1.200403062 |
| itga6 | 0.1625196534 | 0.1515074607 | 1.072684162 | 0.283412849 | 1.17647144 |
| fgf9 | 0.1716269949 | 0.1600518083 | 1.072321498 | 0.2835756539 | 1.187234906 |
| golm2 | 0.1631919894 | 0.1522597805 | 1.071799715 | 0.283810001 | 1.17726269 |
| zbp1 | 0.1712807917 | 0.1599465968 | 1.070862369 | 0.2842313182 | 1.186823953 |
| c1r | 0.1668162521 | 0.1558188871 | 1.070577869 | 0.2843592789 | 1.18153714 |
| gzma | -0.153906849 | 0.143771565 | -1.070495751 | 0.2843962206 | 0.8573518801 |
| hmmr | -0.171916733 | 0.1607425563 | -1.069515984 | 0.2848372302 | 0.8420492849 |
| gzmh | -0.160632303 | 0.1503398676 | -1.068461118 | 0.2853125605 | 0.8516051463 |
| ptprh | -0.16711942 | 0.1564967329 | -1.06787801 | 0.285575543 | 0.8460985644 |
| c2orf69 | -0.171923452 | 0.1611620379 | -1.066773875 | 0.2860739586 | 0.8420436278 |
| cdan1 | 0.1635730151 | 0.1535933743 | 1.064974423 | 0.2868875043 | 1.177711343 |
| mst1 | -0.159780785 | 0.1501231617 | -1.064331335 | 0.2871786276 | 0.852330612 |
| trim24 | -0.170250377 | 0.1599849997 | -1.064164624 | 0.2872541295 | 0.8434536087 |
| ctsb | 0.1551462061 | 0.1458838526 | 1.063491287 | 0.2875592145 | 1.167828692 |
| plscr3 | 0.1634019837 | 0.1536556001 | 1.063430058 | 0.2875869679 | 1.177509934 |
| rbpms | -0.169187915 | 0.1593040468 | -1.062044053 | 0.2882156868 | 0.844350222 |
| bnip3l | 0.1697110061 | 0.1598705589 | 1.061552591 | 0.2884388458 | 1.184962355 |
| rnf4 | 0.1672426649 | 0.158044588 | 1.058199253 | 0.2899646119 | 1.18204107 |
| slitrk6 | 0.1561741686 | 0.1476089978 | 1.058026076 | 0.2900435546 | 1.169029794 |
| agrn | 0.1619834635 | 0.1531528826 | 1.057658601 | 0.2902111156 | 1.175840797 |
| acsl1 | 0.1683173285 | 0.1592368144 | 1.057025218 | 0.2905000783 | 1.18331205 |
| pglyrp4 | 0.1708387852 | 0.1617637879 | 1.056100301 | 0.2909223927 | 1.186299485 |
| mgmt | 0.1596355284 | 0.1513710313 | 1.054597614 | 0.2916093954 | 1.173083238 |
| pag1 | 0.1577949301 | 0.149812082 | 1.053285743 | 0.2922100509 | 1.170926048 |
| padi4 | 0.1532297871 | 0.1455013501 | 1.053115912 | 0.292287871 | 1.165592786 |
| dnpep | -0.164995658 | 0.1568821869 | -1.05171697 | 0.2929294227 | 0.8478973854 |
| gpha2 | 0.1691108085 | 0.1608192057 | 1.051558536 | 0.2930021399 | 1.184251357 |
| ppie | 0.1647255209 | 0.1566800071 | 1.051349971 | 0.2930978842 | 1.179069444 |
| dkk4 | -0.158897707 | 0.1515446882 | -1.048520466 | 0.294398878 | 0.8530836189 |
| st6gal1 | 0.158286425 | 0.1510443755 | 1.047946502 | 0.2946632556 | 1.171501694 |
| bnip2 | 0.1614323111 | 0.1541139814 | 1.047486474 | 0.2948752672 | 1.175192908 |
| dnajb1 | -0.152801719 | 0.1458797657 | -1.047449715 | 0.2948922126 | 0.8582998895 |
| decr1 | 0.1572223433 | 0.150118878 | 1.047318934 | 0.2949525063 | 1.170255784 |
| cacnb1 | 0.1591066075 | 0.1520637842 | 1.046314929 | 0.2954156552 | 1.172462933 |
| pfdn6 | 0.1682188973 | 0.1609173457 | 1.045374547 | 0.295849896 | 1.183195581 |
| esyt2 | 0.1604072038 | 0.1534699611 | 1.04520261 | 0.2959293376 | 1.173988826 |
| il18bp | 0.1559115298 | 0.1496935866 | 1.041537806 | 0.2976260166 | 1.168722801 |
| qdpr | -0.15450664 | 0.1484051972 | -1.041113405 | 0.297822919 | 0.8568378026 |
| odam | 0.1457026187 | 0.1401459177 | 1.039649396 | 0.2985028187 | 1.156852111 |
| scarb2 | 0.1655914933 | 0.1599572796 | 1.03522324 | 0.3005646677 | 1.180090928 |
| ctf1 | 0.1503347672 | 0.1452956921 | 1.034681518 | 0.3008176703 | 1.162223252 |
| gdnf | -0.15555891 | 0.1504130071 | -1.034211822 | 0.3010371487 | 0.8559366521 |
| matn2 | 0.1521057415 | 0.147130788 | 1.033813137 | 0.3012235289 | 1.164283343 |
| dcun1d1 | 0.1574439392 | 0.1524659047 | 1.032650148 | 0.3017676508 | 1.170515136 |
| pga4 | 0.1555234614 | 0.1510634104 | 1.029524363 | 0.3032333372 | 1.168269345 |
| lbr | 0.1512155544 | 0.1469840986 | 1.028788528 | 0.3035790588 | 1.163247374 |
| med21 | -0.161412901 | 0.1569516222 | -1.028424547 | 0.3037501668 | 0.8509406444 |
| gstp1 | -0.151761972 | 0.1476242083 | -1.028029031 | 0.3039361721 | 0.8591927686 |
| kyat1 | -0.147215418 | 0.143358046 | -1.026907255 | 0.3044641376 | 0.8631080288 |
| eif4ebp1 | -0.152172243 | 0.1482514177 | -1.026447136 | 0.3046808696 | 0.8588403387 |
| hsdl2 | -0.157579198 | 0.1536068021 | -1.025860808 | 0.3049571987 | 0.8542091593 |
| gbp1 | 0.1593199993 | 0.1554926388 | 1.024614416 | 0.3055451598 | 1.172713154 |
| nucb2 | 0.1518954358 | 0.1483976673 | 1.023570238 | 0.3060383084 | 1.164038513 |
| spink5 | 0.1474692962 | 0.144333524 | 1.021725876 | 0.3069106604 | 1.158897702 |
| fcn1 | 0.1644231903 | 0.1611553419 | 1.02027763 | 0.3075968095 | 1.178713029 |
| nos2 | 0.1625342184 | 0.1593175294 | 1.020190428 | 0.3076381566 | 1.176488575 |
| myh9 | -0.154191395 | 0.1516678728 | -1.016638473 | 0.3093254448 | 0.8571079595 |
| prr4 | -0.165907581 | 0.1632886449 | -1.016038693 | 0.3096109614 | 0.8471245207 |
| srpx | -0.160582709 | 0.1582991023 | -1.014425901 | 0.3103795705 | 0.8516473814 |
| ifngr2 | 0.1487486166 | 0.1467315404 | 1.013746712 | 0.3107036285 | 1.160381252 |
| aldh3a1 | 0.155875756 | 0.1537623428 | 1.013744673 | 0.3107046015 | 1.168680992 |
| jam3 | 0.1628997993 | 0.1608077776 | 1.013009456 | 0.3110556443 | 1.176918756 |
| scn4b | -0.155566961 | 0.1536378992 | -1.012555896 | 0.3112723352 | 0.8559297614 |

| Protein | beta | se | z | p_val | HR |
| --- | --- | --- | --- | --- | --- |
| tyro3 | -0.145932723 | 0.1443832053 | -1.01073198 | 0.3121447265 | 0.8642158431 |
| mrpl58 | 0.1562577964 | 0.1546640723 | 1.010304424 | 0.3123494623 | 1.169127561 |
| fos | 0.1633676637 | 0.1619871436 | 1.008522405 | 0.3132037372 | 1.177469523 |
| enpp7 | -0.149796478 | 0.1486701272 | -1.007576174 | 0.313657972 | 0.8608831673 |
| slirp | 0.1602209069 | 0.1593019521 | 1.005768635 | 0.3145268784 | 1.173770136 |
| snx15 | -0.161055479 | 0.160225022 | -1.005183069 | 0.3148087061 | 0.8512448433 |
| klk1 | 0.1420918486 | 0.141487415 | 1.004271995 | 0.3152475282 | 1.152682516 |
| rapgef2 | -0.159484343 | 0.1590610782 | -1.002661022 | 0.3160244425 | 0.852583316 |
| kif22 | 0.1569155673 | 0.1565005318 | 1.002651975 | 0.3160288092 | 1.169896832 |
| bag6 | 0.1496373038 | 0.1496111008 | 1.000175141 | 0.3172257573 | 1.161412926 |
| gipr | -0.157780796 | 0.1579730443 | -0.99878303 | 0.3178998083 | 0.8540369699 |
| gdf15 | 0.1850615751 | 0.1853993397 | 0.9981781777 | 0.3181929663 | 1.203292531 |
| ca5a | 0.1563570062 | 0.1566605178 | 0.9980626155 | 0.3182489967 | 1.169243556 |
| mphosph8 | -0.146392725 | 0.1467752076 | -0.997394091 | 0.3185732585 | 0.8638183937 |
| serpina6 | 0.1795455023 | 0.1800280482 | 0.9973196069 | 0.3186093996 | 1.196673354 |
| spock1 | -0.146024624 | 0.1465154284 | -0.99665015 | 0.3189343545 | 0.8641364248 |
| cd38 | -0.177827273 | 0.1785869333 | -0.995746271 | 0.3193734419 | 0.8370869986 |
| il1r2 | -0.144098488 | 0.1447980931 | -0.995168411 | 0.3196543627 | 0.8658024725 |
| septin3 | -0.15772623 | 0.1585776334 | -0.994631 | 0.3199157646 | 0.8540835723 |
| dtx2 | 0.1609610851 | 0.1619211494 | 0.9940707915 | 0.3201884042 | 1.174639257 |
| c4bpb | 0.1458608041 | 0.1468085059 | 0.9935446397 | 0.3204446076 | 1.157035122 |
| lag3 | 0.1507406363 | 0.1520615987 | 0.9913129785 | 0.3215327775 | 1.162695058 |
| mycbp2 | 0.1534513287 | 0.154892944 | 0.9906928281 | 0.3218355942 | 1.165851042 |
| crhbp | 0.1477636919 | 0.1492590283 | 0.9899816012 | 0.3221831121 | 1.159238926 |
| lgals7_lgals7b | 0.1507512577 | 0.1522951382 | 0.989862575 | 0.3222412943 | 1.162707408 |
| tgfbr3 | 0.1398414412 | 0.141342674 | 0.9893787722 | 0.3224778566 | 1.150091427 |
| rab39b | -0.159025524 | 0.1607605601 | -0.989207325 | 0.3225617151 | 0.8529745876 |
| ak1 | -0.14699889 | 0.1487919547 | -0.987949182 | 0.3231775371 | 0.8632949358 |
| edil3 | 0.1546036995 | 0.1565227221 | 0.9877396544 | 0.3232801684 | 1.167195309 |
| hmgcl | 0.1514520213 | 0.1534437974 | 0.9870195076 | 0.3236330754 | 1.163522476 |
| cd14 | -0.151489973 | 0.1538940769 | -0.984378192 | 0.3249295968 | 0.8594264996 |
| ntrk2 | -0.146398786 | 0.1488061261 | -0.983822307 | 0.3252028895 | 0.8638131577 |
| znf830 | -0.151596292 | 0.1541619516 | -0.983357376 | 0.3254315805 | 0.859335131 |
| insl5 | -0.166612402 | 0.1694878836 | -0.983034292 | 0.3255905617 | 0.8465276605 |
| pon2 | -0.140986866 | 0.1435924522 | -0.981854294 | 0.3261716355 | 0.8685007187 |
| apoa4 | -0.153094985 | 0.1560259304 | -0.98121501 | 0.3264867235 | 0.8580482163 |
| il34 | -0.143175608 | 0.1459181115 | -0.981205185 | 0.3264915676 | 0.8666018736 |
| eif5a | 0.1418093035 | 0.1446407986 | 0.9804239532 | 0.3268768908 | 1.152356877 |
| gpihbp1 | -0.151323412 | 0.1543584737 | -0.980337575 | 0.3269195126 | 0.8595696587 |
| cd177 | -0.138372916 | 0.1411599083 | -0.98025649 | 0.3269595263 | 0.8707739055 |
| bcl2l11 | 0.1493941607 | 0.1525620101 | 0.9792356598 | 0.3274635528 | 1.161130571 |
| cnst | 0.1383688965 | 0.141509288 | 0.9778078773 | 0.3281693543 | 1.148399113 |
| tcl1a | -0.13915169 | 0.1423256536 | -0.977699287 | 0.3282230748 | 0.8700960336 |
| pof1b | 0.1602532076 | 0.1639918987 | 0.9772019773 | 0.3284691693 | 1.173808051 |
| abl1 | 0.1426979254 | 0.1460540169 | 0.9770215735 | 0.3285584721 | 1.153381342 |
| mapk9 | 0.151668263 | 0.155287735 | 0.9766918361 | 0.3287217381 | 1.163774105 |
| prg3 | 0.1490983955 | 0.15272426 | 0.9762587528 | 0.3289362546 | 1.1607872 |
| batf | -0.155765728 | 0.1597161178 | -0.975266179 | 0.3294282422 | 0.8557596474 |
| dner | -0.148019723 | 0.1518520394 | -0.974762828 | 0.3296779198 | 0.8624141051 |
| psma1 | 0.1410176387 | 0.144725188 | 0.9743821421 | 0.3298668329 | 1.151444958 |
| sfrp1 | -0.155051927 | 0.1594399299 | -0.972478644 | 0.3308124843 | 0.8563707078 |
| galnt5 | -0.150682974 | 0.1550398873 | -0.971898111 | 0.3311012396 | 0.8601203363 |
| letm1 | 0.154809106 | 0.1596875141 | 0.9694502846 | 0.3323205737 | 1.167435083 |
| tdrkh | 0.1428280602 | 0.1475695818 | 0.9678692487 | 0.3331096739 | 1.153531447 |
| palld | 0.1517284872 | 0.1567835954 | 0.9677574158 | 0.3331655359 | 1.163844195 |
| gnas | 0.1495146694 | 0.154507486 | 0.9676856009 | 0.3332014115 | 1.161270506 |
| mcemp1 | 0.1511052361 | 0.1564102107 | 0.9660829394 | 0.3340026807 | 1.163119054 |
| dpp6 | -0.14735217 | 0.1525272244 | -0.966071277 | 0.3340085161 | 0.8629900044 |
| ptn | -0.149548352 | 0.154924647 | -0.965297355 | 0.3343958961 | 0.8610968013 |
| pkd2 | 0.1603352782 | 0.1662228811 | 0.9645800696 | 0.334755186 | 1.17390439 |
| pcbd1 | 0.1705266971 | 0.1768380442 | 0.9643100154 | 0.3348905211 | 1.185929312 |
| ltbr | 0.1438559484 | 0.1492893147 | 0.9636051229 | 0.3352439374 | 1.154717758 |
| lypla2 | 0.1469980842 | 0.1525628564 | 0.9635247243 | 0.3352842627 | 1.158351744 |
| fst | -0.147354057 | 0.1529932491 | -0.963140907 | 0.3354768152 | 0.8629883764 |
| ccl4 | 0.1419017761 | 0.1475295712 | 0.9618531047 | 0.3361233979 | 1.152463444 |
| cdsn | 0.1393437682 | 0.1448934322 | 0.9616983055 | 0.3362011738 | 1.1495192 |
| dsg4 | -0.144089142 | 0.1498291725 | -0.9616895 | 0.3362055982 | 0.8658105645 |
| pdp1 | 0.1386726568 | 0.1444061317 | 0.9602961809 | 0.3369061716 | 1.148748004 |
| fes | 0.140448068 | 0.1463461035 | 0.9596980355 | 0.3372072123 | 1.150789315 |
| wfikkn2 | 0.1457782788 | 0.1519281551 | 0.9595211544 | 0.3372962679 | 1.156939642 |

| Protein | beta | se | z | p_val | HR |
|---|---|---|---|---|---|
| lmnb2 | 0.1611681718 | 0.1680328778 | 0.9591466493 | 0.3374848726 | 1.174882534 |
| fgd3 | 0.1484249543 | 0.1548055574 | 0.9587831134 | 0.3376680179 | 1.160005741 |
| capg | 0.1415435625 | 0.1477172993 | 0.9582057295 | 0.3379590287 | 1.152050689 |
| lyve1 | 0.1478925914 | 0.1545806111 | 0.9567344208 | 0.3387013199 | 1.159388361 |
| wfikkn1 | -0.1448998 | 0.1515601981 | -0.956054438 | 0.3390447321 | 0.8651089727 |
| ceacam6 | 0.1541792024 | 0.1613827885 | 0.955363356 | 0.3393939783 | 1.166699943 |
| tef | 0.1565499251 | 0.1639477618 | 0.9548768668 | 0.3396399696 | 1.169469147 |
| mtss2 | 0.1464659072 | 0.153451858 | 0.9544746421 | 0.3398434392 | 1.15773546 |
| fabp4 | 0.18173206 | 0.1906566329 | 0.9531903359 | 0.340493642 | 1.199292812 |
| jam2 | 0.1428366988 | 0.1502876597 | 0.9504220045 | 0.3418978675 | 1.153541412 |
| retn | 0.1365644072 | 0.144025664 | 0.9481949497 | 0.3430302177 | 1.146328707 |
| b4galt1 | 0.1397932133 | 0.1476589695 | 0.9467302514 | 0.3437762512 | 1.150035962 |
| mvk | 0.1426215772 | 0.1506630712 | 0.9466259786 | 0.3438294013 | 1.153293287 |
| nrp2 | 0.1409115803 | 0.1492285436 | 0.9442669407 | 0.3450332552 | 1.151322844 |
| oscar | 0.1347034977 | 0.142674454 | 0.9441318601 | 0.3451022702 | 1.144197477 |
| gfra2 | 0.1366170673 | 0.1448330197 | 0.9432729329 | 0.3455413168 | 1.146389075 |
| cd200r1 | -0.141793018 | 0.1504392313 | -0.94252687 | 0.3459229611 | 0.8678008573 |
| il36g | 0.1469243317 | 0.1562137067 | 0.9405341873 | 0.3469436221 | 1.158266316 |
| aprt | -0.136436488 | 0.1451260167 | -0.940124253 | 0.3471538299 | 0.8724617303 |
| mrps16 | 0.1469204752 | 0.1564071743 | 0.9393461384 | 0.3475530572 | 1.158261849 |
| usp25 | 0.1450030511 | 0.1544792907 | 0.9386568938 | 0.3479069319 | 1.156043097 |
| tafa5 | 0.1384134466 | 0.1477448444 | 0.9368411274 | 0.3488402863 | 1.148450275 |
| clstn1 | 0.1360618542 | 0.1452834335 | 0.9365269726 | 0.3490019318 | 1.145752761 |
| tpbgl | 0.1472641595 | 0.1573229912 | 0.9360625446 | 0.3492409863 | 1.158659994 |
| atxn2l | 0.1466459155 | 0.1568286005 | 0.9350712498 | 0.3497515819 | 1.15794388 |
| cpb2 | 0.1454327426 | 0.1558565908 | 0.9331189767 | 0.3507585421 | 1.156539946 |
| arl13b | 0.1485143147 | 0.1592374068 | 0.9326597169 | 0.3509956899 | 1.160109404 |
| egfr | -0.138117571 | 0.1481029734 | -0.932577978 | 0.3510379082 | 0.8709962814 |
| fdx1 | 0.1497278035 | 0.1605916406 | 0.9323511668 | 0.3511550728 | 1.161518039 |
| arl2bp | 0.1493720433 | 0.1603231905 | 0.9316933055 | 0.3514950471 | 1.16110489 |
| ophn1 | 0.140678751 | 0.1513830014 | 0.9292902751 | 0.3527386737 | 1.151054813 |
| cd209 | -0.134515589 | 0.1448437666 | -0.928694356 | 0.3530475066 | 0.8741392522 |
| plg | 0.1507686435 | 0.1625536672 | 0.9275007207 | 0.3536666178 | 1.162727622 |
| cd72 | -0.148173956 | 0.1599051656 | -0.926636457 | 0.3541153194 | 0.8622811028 |
| ptk7 | 0.1349169037 | 0.14571795 | 0.9258770362 | 0.3545098865 | 1.144441681 |
| plat | 0.151778522 | 0.1641053337 | 0.9248847593 | 0.3550258551 | 1.163902429 |
| frmd4b | 0.1469444403 | 0.1590662804 | 0.9237937794 | 0.3555936945 | 1.158289607 |
| pf4 | 0.1403150592 | 0.1519579115 | 0.9233810723 | 0.355808652 | 1.15063626 |
| lypd1 | -0.133845688 | 0.1449860505 | -0.923162521 | 0.3559225172 | 0.8747250349 |
| cntnap4 | -0.150501318 | 0.1630312904 | -0.923143756 | 0.3559322944 | 0.8602765963 |
| arfip1 | 0.1429375952 | 0.1549626119 | 0.9224005291 | 0.3563196939 | 1.153657806 |
| smtn | 0.1410588348 | 0.1530321413 | 0.9217595308 | 0.3566540209 | 1.151492394 |
| iqgap2 | 0.1353738563 | 0.1469859626 | 0.9209985353 | 0.3570511917 | 1.144964757 |
| app | 0.1333678997 | 0.1448637831 | 0.9206434957 | 0.3572365855 | 1.142670309 |
| pappa | -0.139694981 | 0.151738011 | -0.920632742 | 0.3572422021 | 0.8696234466 |
| nppb | 0.1320738889 | 0.143525485 | 0.9202121066 | 0.357461929 | 1.141192638 |
| fshb | -0.211037412 | 0.2295607635 | -0.919309591 | 0.3579336625 | 0.8097437724 |
| dmd | 0.1453940042 | 0.1582606427 | 0.9186996955 | 0.3582526691 | 1.156495144 |
| fcgr3b | 0.1354755944 | 0.1475385743 | 0.9182384679 | 0.3584940335 | 1.145081249 |
| lrfn2 | -0.146593903 | 0.1597195422 | -0.917820708 | 0.3587127391 | 0.8636446295 |
| hdgfl2 | 0.1424270241 | 0.1552090415 | 0.9176464383 | 0.3588039974 | 1.153068932 |
| cd2ap | -0.132191876 | 0.1442313909 | -0.916526387 | 0.3593908743 | 0.8761728628 |
| cpxm1 | 0.1294064087 | 0.1413147002 | 0.9157321111 | 0.3598074185 | 1.138152585 |
| scg2 | 0.1306031166 | 0.1426218504 | 0.9157300672 | 0.3598084908 | 1.139515437 |
| pgr | 0.1407920876 | 0.1538356266 | 0.9152111947 | 0.3600807688 | 1.151185277 |
| csf3 | 0.1359794824 | 0.1486019801 | 0.915058348 | 0.3601609997 | 1.145658387 |
| timp1 | 0.1399263525 | 0.1529195737 | 0.9150323213 | 0.3601746625 | 1.150189087 |
| slmap | 0.1436681495 | 0.1570634846 | 0.9147138806 | 0.3603418555 | 1.154500923 |
| pdgfb | 0.1308171738 | 0.1431056567 | 0.914129999 | 0.3606485411 | 1.139759384 |
| ctsl | 0.133724122 | 0.146461241 | 0.9130342003 | 0.3612245547 | 1.143077426 |
| metap1 | 0.1329382108 | 0.145801869 | 0.9117730226 | 0.3618882145 | 1.142179422 |
| gprc5c | 0.1388936957 | 0.1523366736 | 0.9117548152 | 0.3618978012 | 1.14900195 |
| il17f | 0.1319325063 | 0.1447524848 | 0.9114351746 | 0.3620661273 | 1.141031304 |
| mapt | 0.133714239 | 0.14679123 | 0.910914358 | 0.3623404999 | 1.143066129 |
| zhx2 | 0.1431331223 | 0.1571925352 | 0.9105592837 | 0.362527632 | 1.153883399 |
| stat2 | 0.1426565681 | 0.1566764462 | 0.910517002 | 0.3625499194 | 1.153333642 |
| milr1 | 0.1327592984 | 0.1460225668 | 0.9091697354 | 0.3632605369 | 1.14197509 |
| ahnak2 | 0.1434693856 | 0.1580186799 | 0.9079267447 | 0.3639169263 | 1.154271473 |
| gart | 0.1422918763 | 0.1567304014 | 0.9078766785 | 0.3639433804 | 1.152913107 |
| cspg4 | -0.150677876 | 0.1661864907 | -0.90667945 | 0.3645763337 | 0.8601247209 |

| Protein | beta | se | z | p_val | HR |
|---|---|---|---|---|---|
| rtbdn | -0.136232109 | 0.1502799554 | -0.906522154 | 0.3646595446 | 0.8726400616 |
| dnaja1 | 0.1477664354 | 0.1630448491 | 0.9062931835 | 0.3647806925 | 1.159242107 |
| nefl | 0.1548663317 | 0.170980286 | 0.9057554839 | 0.3650652879 | 1.167501893 |
| sdk2 | 0.1435805343 | 0.1585611712 | 0.9055214039 | 0.3651892259 | 1.154399776 |
| tex101 | -0.204573 | 0.2259969265 | -0.905202574 | 0.3653580785 | 0.8149952454 |
| lgals3bp | 0.1478332651 | 0.16360073 | 0.9036222826 | 0.3661957217 | 1.159319581 |
| orm1 | 0.1410130566 | 0.1562478427 | 0.9024960231 | 0.366793433 | 1.151439682 |
| clgn | -0.146540341 | 0.1624113932 | -0.9022787 | 0.3669088374 | 0.8636908897 |
| cntn5 | -0.139461346 | 0.154637547 | -0.901859533 | 0.3671314905 | 0.869826645 |
| apob | 0.1383146838 | 0.1533754338 | 0.9018046788 | 0.3671606339 | 1.148336856 |
| fh | 0.1387496634 | 0.1539016045 | 0.9015478683 | 0.3672970947 | 1.148836468 |
| amn | 0.1309094785 | 0.1453556124 | 0.9006152312 | 0.3677929334 | 1.139864594 |
| il10rb | 0.1367790927 | 0.1519150556 | 0.9003656166 | 0.3679257123 | 1.146574834 |
| acox1 | 0.1280290464 | 0.1422380432 | 0.9001041039 | 0.3680648523 | 1.136586016 |
| itm2a | -0.135546778 | 0.1506390819 | -0.899811498 | 0.3682205743 | 0.8732383136 |
| sncg | 0.1448189612 | 0.1611516008 | 0.8986504667 | 0.3688388686 | 1.155830301 |
| gga1 | -0.131853689 | 0.1468124882 | -0.89810949 | 0.3691271802 | 0.8764692229 |
| amfr | -0.132596577 | 0.147702605 | -0.89772673 | 0.3693312555 | 0.8758183465 |
| reps1 | 0.1388006751 | 0.1546873237 | 0.8972983161 | 0.3695597548 | 1.148895074 |
| ubqln3 | 0.1402577144 | 0.1563810602 | 0.896897068 | 0.3697738448 | 1.150570279 |
| icam5 | -0.132674362 | 0.1479736958 | -0.896607749 | 0.3699282616 | 0.8757502229 |
| fabp6 | 0.128251464 | 0.14306963 | 0.8964268933 | 0.3700248093 | 1.136838841 |
| esr1 | 0.1393125175 | 0.1554176835 | 0.8963749446 | 0.3700525444 | 1.149483277 |
| il31ra | 0.1399358316 | 0.1563170722 | 0.8952050447 | 0.3706774885 | 1.15019999 |
| ptgr1 | 0.1392920366 | 0.1557035732 | 0.89459756 | 0.3710022566 | 1.149459735 |
| pdia3 | 0.1424007016 | 0.1592181638 | 0.8943747262 | 0.3711214304 | 1.15303858 |
| adgrv1 | 0.1358617354 | 0.1519584686 | 0.8940714963 | 0.3712836388 | 1.145523497 |
| stk24 | 0.1310177908 | 0.1466062236 | 0.8936714114 | 0.3714977258 | 1.139988062 |
| il25 | 0.1446533199 | 0.1622580897 | 0.8915014358 | 0.3726602216 | 1.155638864 |
| tgm2 | 0.1266325797 | 0.1421089544 | 0.8910950071 | 0.3728782033 | 1.134999919 |
| chad | -0.144691648 | 0.1623774676 | -0.891082057 | 0.3728851502 | 0.8652890657 |
| pfdn2 | -0.130930435 | 0.1469466988 | -0.891006301 | 0.37292579 | 0.8772788006 |
| khk | 0.1430310448 | 0.1605344762 | 0.8909677737 | 0.3729464591 | 1.15376562 |
| fkbp14 | 0.139336047 | 0.15656728 | 0.8899435885 | 0.3734961773 | 1.149510325 |
| fabp1 | 0.1369284264 | 0.1538749064 | 0.8898684624 | 0.3735365199 | 1.146746069 |
| clc | 0.1320385031 | 0.1484183027 | 0.8896376034 | 0.3736605081 | 1.141152256 |
| ddi2 | -0.139604681 | 0.1569500156 | -0.889484977 | 0.3737424936 | 0.8697019771 |
| notch1 | 0.1281218457 | 0.1440772399 | 0.8892580523 | 0.37386441 | 1.136691495 |
| ca1 | -0.131225231 | 0.1476589519 | -0.888704882 | 0.3741617066 | 0.8770202196 |
| nudt2 | -0.127797594 | 0.1438034068 | -0.888696566 | 0.3741661773 | 0.880031485 |
| mmp3 | 0.1649337374 | 0.1856874944 | 0.8882328768 | 0.3744154974 | 1.179314972 |
| rnase3 | 0.1281136613 | 0.1442735426 | 0.8879913738 | 0.3745453915 | 1.136682192 |
| serpinf1 | 0.1555180343 | 0.1753609749 | 0.8868451742 | 0.3751622625 | 1.168263005 |
| ccl11 | -0.137939204 | 0.1555650364 | -0.886697985 | 0.3752415234 | 0.8711516523 |
| larp1 | 0.1359230016 | 0.1535249979 | 0.8853476858 | 0.3759691383 | 1.145593681 |
| ccl22 | 0.1315042134 | 0.1485725687 | 0.8851177208 | 0.3760931428 | 1.140542713 |
| ddt | 0.1418341492 | 0.1603856296 | 0.8843320289 | 0.3765170033 | 1.152385509 |
| ghrhr | 0.1268642381 | 0.1434940994 | 0.8841076991 | 0.3766380775 | 1.135262882 |
| vsig10 | -0.136728268 | 0.1546617976 | -0.884046803 | 0.3766709483 | 0.8722072008 |
| clec10a | -0.128968314 | 0.1459384432 | -0.883717214 | 0.3768488854 | 0.8790018168 |
| ppy | -0.131546261 | 0.1490880243 | -0.882339555 | 0.3775932123 | 0.8767387154 |
| lrp2bp | 0.1407880728 | 0.1597785129 | 0.8811452193 | 0.3782392249 | 1.151180656 |
| skap1 | 0.1279305165 | 0.145241474 | 0.8808125732 | 0.3784192734 | 1.136474034 |
| psip1 | 0.1367833201 | 0.1553930982 | 0.8802406392 | 0.3787289624 | 1.146579681 |
| mfap3l | -0.140847891 | 0.1602047541 | -0.879174229 | 0.3793068155 | 0.8686214267 |
| clasp1 | -0.137290571 | 0.1563782088 | -0.877939275 | 0.3799766738 | 0.8717168934 |
| ccar2 | 0.1338678773 | 0.1525893752 | 0.8773079849 | 0.3803193764 | 1.143241761 |
| hla_e | 0.139020941 | 0.158578835 | 0.87666769 | 0.380667161 | 1.149148164 |
| amigo1 | -0.137347083 | 0.1569443888 | -0.875132169 | 0.3815019951 | 0.8716676322 |
| jun | 0.1321800344 | 0.1511126843 | 0.8747117092 | 0.3817307874 | 1.141313777 |
| il15 | 0.1313003003 | 0.1503346757 | 0.8733866603 | 0.3824523595 | 1.140310165 |
| dnajb6 | 0.1377561873 | 0.1578192335 | 0.8728732505 | 0.3827321679 | 1.147695693 |
| dpp4 | -0.131276051 | 0.1504071473 | -0.872804608 | 0.3827695876 | 0.8769756508 |
| vcam1 | 0.1277960924 | 0.1464358238 | 0.8727105778 | 0.3828208507 | 1.136321275 |
| fgfr4 | -0.138312503 | 0.1585838651 | -0.872172605 | 0.3831142221 | 0.8708265135 |
| cd46 | 0.1303201041 | 0.1495307063 | 0.8715273757 | 0.3834662651 | 1.139192985 |
| calb2 | 0.1384155268 | 0.1588841659 | 0.8711725683 | 0.3836599356 | 1.148452664 |
| foxo3 | 0.1244742794 | 0.1430177895 | 0.870341234 | 0.3841139513 | 1.13255289 |
| mink1 | 0.1323122624 | 0.1523846985 | 0.8682778765 | 0.3852422304 | 1.1414647 |
| cnpy2 | 0.127198292 | 0.1465758504 | 0.8677984244 | 0.3855046927 | 1.135642184 |

| Protein | beta | se | z | p_val | HR |
|---|---|---|---|---|---|
| hebp1 | 0.1237793536 | 0.1427240293 | 0.867263587 | 0.385797603 | 1.131766123 |
| ca12 | 0.1285530132 | 0.148276788 | 0.8669800242 | 0.3859529548 | 1.137181705 |
| pdgfc | 0.1258854708 | 0.1452724055 | 0.8665477133 | 0.3861898728 | 1.134152267 |
| atp6v1d | -0.126672322 | 0.1462788204 | -0.865964886 | 0.386509418 | 0.8810223168 |
| cbs | 0.1382057372 | 0.1598817741 | 0.864424591 | 0.3873546889 | 1.148211756 |
| vipr1 | 0.1344753245 | 0.1556444567 | 0.8639904518 | 0.3875931358 | 1.143936431 |
| ngrn | 0.1366214579 | 0.1582105628 | 0.86354195 | 0.3878395651 | 1.146394108 |
| ccl5 | 0.1250589575 | 0.1450990515 | 0.8618868018 | 0.3887498125 | 1.133215263 |
| adamtsl4 | 0.1357028937 | 0.1574782417 | 0.8617247195 | 0.3888390194 | 1.145341555 |
| crtac1 | -0.125453055 | 0.1459920643 | -0.859314206 | 0.3901671887 | 0.8820971737 |
| pfdn4 | -0.139498392 | 0.1625482472 | -0.858196838 | 0.3907837815 | 0.869794422 |
| ppp1r14d | 0.1337669029 | 0.1560521457 | 0.8571936154 | 0.3913378904 | 1.143126329 |
| ddx4 | 0.1331778252 | 0.1556980615 | 0.8553595588 | 0.3923521248 | 1.142453137 |
| rnf43 | -0.139526808 | 0.1631674492 | -0.855114233 | 0.3924879107 | 0.8697697059 |
| cracr2a | 0.1227029483 | 0.1435901631 | 0.8545358934 | 0.3928081305 | 1.13054854 |
| adm | 0.1509935337 | 0.1768421345 | 0.8538323411 | 0.3931978922 | 1.162989138 |
| smc3 | 0.1365560516 | 0.1601736117 | 0.852550243 | 0.3939087654 | 1.146319129 |
| vstm1 | 0.1238752283 | 0.145464086 | 0.8515863378 | 0.3944437251 | 1.131874636 |
| enpp6 | -0.138745391 | 0.1629589541 | -0.851413119 | 0.3945399068 | 0.8704496242 |
| eppk1 | 0.1318178245 | 0.1549903795 | 0.8504903656 | 0.3950525146 | 1.140900456 |
| bcr | 0.1276129146 | 0.1500803804 | 0.8502971159 | 0.3951599197 | 1.136113145 |
| icam1 | -0.126041969 | 0.1482383474 | -0.850265609 | 0.3951774324 | 0.8815778473 |
| il12rb2 | 0.1318685235 | 0.1550963465 | 0.8502361693 | 0.3951937964 | 1.1409583 |
| pcdh12 | -0.131940971 | 0.155271682 | -0.849742655 | 0.3954681782 | 0.8763927259 |
| mdh1 | -0.133069517 | 0.1567255222 | -0.849060925 | 0.3958473918 | 0.8754042347 |
| mnda | 0.1214818286 | 0.1431536005 | 0.8486117579 | 0.3960973622 | 1.129168847 |
| mllt1 | 0.1390674451 | 0.1639428894 | 0.8482676229 | 0.3962889446 | 1.149201605 |
| nell2 | -0.125182608 | 0.1475755569 | -0.848261125 | 0.3962925627 | 0.8823357665 |
| tacstd2 | 0.1352734866 | 0.159510864 | 0.8480518705 | 0.3964090839 | 1.144849843 |
| siae | 0.1252079805 | 0.1476826917 | 0.8478175681 | 0.3965395776 | 1.13338415 |
| adgrd1 | 0.1511563307 | 0.1783955089 | 0.8473101798 | 0.3968222541 | 1.163178484 |
| swap70 | 0.1353825682 | 0.1597963777 | 0.8472192558 | 0.3968729225 | 1.144974731 |
| smpdl3b | -0.128773043 | 0.1520245106 | -0.847054481 | 0.396964755 | 0.8791734775 |
| lcn2 | 0.1282928812 | 0.1515295324 | 0.8466526572 | 0.3971887542 | 1.136885926 |
| clps | -0.120801502 | 0.1427636398 | -0.846164346 | 0.3974610689 | 0.8862098531 |
| kif20b | 0.129873602 | 0.1535793832 | 0.845644769 | 0.3977509428 | 1.138684447 |
| snapin | 0.1338245205 | 0.1583751719 | 0.8449842161 | 0.3981196518 | 1.143192195 |
| hdgf | 0.1201893782 | 0.1422893187 | 0.8446830675 | 0.3982878159 | 1.127710395 |
| echs1 | 0.1293931624 | 0.1534775482 | 0.8430755111 | 0.3991862137 | 1.138137509 |
| plpbp | -0.121614009 | 0.1443441208 | -0.842528317 | 0.3994922962 | 0.8854900936 |
| stc1 | 0.1293153629 | 0.1535605244 | 0.8421133193 | 0.3997245265 | 1.138048966 |
| vash1 | 0.1269928005 | 0.1509810892 | 0.8411172629 | 0.400282245 | 1.135408843 |
| tmprss15 | 0.1245448083 | 0.1482857188 | 0.8398975258 | 0.400965845 | 1.132632771 |
| nmt1 | 0.1308181477 | 0.1559973917 | 0.8385918907 | 0.4016983628 | 1.139760494 |
| tnfsf11 | -0.128415894 | 0.1532047245 | -0.838198004 | 0.4019195079 | 0.8794875292 |
| il33 | 0.1206928589 | 0.1440463004 | 0.8378754509 | 0.4021006576 | 1.128278318 |
| cd1c | -0.122834516 | 0.1466994462 | -0.837320927 | 0.4024121995 | 0.884410006 |
| angpt1 | 0.1188687567 | 0.1421283109 | 0.8363481977 | 0.4029590459 | 1.126222099 |
| ptpn1 | 0.1223213418 | 0.1463105285 | 0.8360392317 | 0.4031328328 | 1.130117197 |
| ctrl | 0.1251025443 | 0.1498210632 | 0.8350130592 | 0.4037103556 | 1.133264657 |
| blnk | 0.1317938889 | 0.1581445783 | 0.8333759547 | 0.4046327314 | 1.140873148 |
| gadd45gip1 | 0.12844793 | 0.1541552215 | 0.8332376213 | 0.4047107288 | 1.137062213 |
| get3 | 0.1292365959 | 0.1551846529 | 0.8327923769 | 0.4049618348 | 1.137959329 |
| tpk1 | 0.1292050295 | 0.1555885609 | 0.8304275634 | 0.4062970869 | 1.137923408 |
| alcam | 0.1271868622 | 0.1532147367 | 0.8301215988 | 0.4064700365 | 1.135629204 |
| sf3b4 | -0.119963552 | 0.1445320497 | -0.830013497 | 0.4065311529 | 0.8869527638 |
| ostn | 0.13512665 | 0.1628022392 | 0.8300048616 | 0.406536035 | 1.144681749 |
| pdcd1 | 0.1181914704 | 0.1425193006 | 0.829301504 | 0.40693382 | 1.125459583 |
| dkk1 | 0.1203504734 | 0.1452036288 | 0.8288392955 | 0.4071953492 | 1.127892078 |
| eno2 | -0.119342224 | 0.1441768775 | -0.827748706 | 0.4078128295 | 0.8875040239 |
| ccn5 | 0.1394435799 | 0.1687320731 | 0.8264201187 | 0.4085658147 | 1.149633941 |
| man1a2 | -0.135070796 | 0.1637258989 | -0.824981248 | 0.4093822369 | 0.8736540579 |
| camlg | 0.1319955778 | 0.1600220429 | 0.824858722 | 0.4094518036 | 1.141103273 |
| inhbb | 0.1374077022 | 0.1666083436 | 0.8247348197 | 0.4095221588 | 1.147295808 |
| fam3d | 0.1294739651 | 0.1571762362 | 0.8237502578 | 0.4100814764 | 1.138229477 |
| rab37 | -0.117460869 | 0.1427464182 | -0.822863863 | 0.4105854149 | 0.8891753053 |
| kir2dl3 | 0.1185206705 | 0.1441335937 | 0.8222973384 | 0.4109076912 | 1.125830145 |
| ighmbp2 | 0.1309764816 | 0.159348823 | 0.8219482212 | 0.4111063668 | 1.139940971 |
| spred2 | 0.1288843203 | 0.1573594353 | 0.8190441206 | 0.4127612415 | 1.137558524 |
| idua | 0.1160764331 | 0.1417320957 | 0.8189848069 | 0.4127950819 | 1.123081709 |

| Protein | beta | se | z | p_val | HR |
|---|---|---|---|---|---|
| cnpy4 | 0.1175339468 | 0.1435794451 | 0.818598698 | 0.4130154101 | 1.12471981 |
| shd | 0.1298202741 | 0.1587100789 | 0.8179712026 | 0.413373631 | 1.138623725 |
| casp9 | 0.1268937612 | 0.1552011831 | 0.8176082079 | 0.4135809393 | 1.135296399 |
| cgb3_cgb5_cgb8 | 0.1359321632 | 0.1663674992 | 0.8170596051 | 0.4138943661 | 1.145604177 |
| ifngr1 | 0.1180536184 | 0.1445653884 | 0.81661053 | 0.4141510355 | 1.125304447 |
| foxj3 | 0.1293128155 | 0.158624821 | 0.8152117347 | 0.414951122 | 1.138046067 |
| c1qa | 0.1215076799 | 0.1490862857 | 0.81501581 | 0.4150632604 | 1.129198038 |
| cd86 | 0.1233524141 | 0.1514769602 | 0.8143311959 | 0.415455243 | 1.131283031 |
| ppbp | 0.12369844 | 0.1519780904 | 0.8139228472 | 0.4156891512 | 1.131674552 |
| art3 | -0.128727286 | 0.1581653019 | -0.813878165 | 0.4157147508 | 0.8792137071 |
| lgmn | 0.1178458921 | 0.1448058215 | 0.8138201274 | 0.4157480028 | 1.125070716 |
| il17c | -0.119555133 | 0.146966211 | -0.813487209 | 0.4159387771 | 0.8873150864 |
| pdlim7 | 0.116279059 | 0.1431763091 | 0.8121389618 | 0.4167118988 | 1.123309298 |
| tnfrsf6b | 0.1238268101 | 0.1525163129 | 0.8118922344 | 0.4168534708 | 1.131819834 |
| tnfaip2 | 0.1274933293 | 0.1572905563 | 0.8105593391 | 0.4176187752 | 1.13597729 |
| myo6 | 0.1264834041 | 0.1561122424 | 0.810208105 | 0.41782058 | 1.134830617 |
| cd34 | -0.117611706 | 0.1452090713 | -0.809947372 | 0.4179704239 | 0.8890411953 |
| nrtn | 0.1158419213 | 0.1431623372 | 0.8091647814 | 0.4184203698 | 1.122818364 |
| il17rb | -0.128467737 | 0.1588099424 | -0.808940141 | 0.4185495784 | 0.8794419352 |
| nlgn2 | 0.123826638 | 0.1533549871 | 0.8074510023 | 0.4194066929 | 1.13181964 |
| vsir | 0.115481879 | 0.1431194306 | 0.8068916879 | 0.4197288881 | 1.122414175 |
| defb118 | 0.1242099086 | 0.1541457207 | 0.8057953736 | 0.4203608461 | 1.132253516 |
| casp8 | -0.117171236 | 0.1455741635 | -0.804890328 | 0.4208829704 | 0.8894328771 |
| mep1a | -0.130892169 | 0.1626359137 | -0.80481713 | 0.420925215 | 0.8773123705 |
| ccl23 | 0.1166139365 | 0.1452080965 | 0.8030815035 | 0.4219276278 | 1.123685532 |
| dtd1 | 0.1274888882 | 0.1587602301 | 0.803027862 | 0.4219586308 | 1.135972245 |
| cpq | -0.123272842 | 0.1537169626 | -0.801946905 | 0.4225836721 | 0.8840224309 |
| vsnl1 | 0.1365400869 | 0.1703155208 | 0.8016890432 | 0.4227328554 | 1.146300829 |
| chrdl2 | -0.12499134 | 0.1560171853 | -0.801138282 | 0.4230515961 | 0.8825045452 |
| usp47 | -0.130568421 | 0.1630536464 | -0.800769707 | 0.4232649794 | 0.8775964452 |
| pmm2 | 0.1274240973 | 0.1592955798 | 0.7999223673 | 0.4237557776 | 1.135898647 |
| btla | -0.131139305 | 0.1640222596 | -0.799521393 | 0.4239881473 | 0.8770955817 |
| ahnak | 0.133064945 | 0.166461716 | 0.7993726614 | 0.4240743577 | 1.142324184 |
| omd | -0.121195513 | 0.1518921871 | -0.797904854 | 0.4249257065 | 0.8858607451 |
| mtdh | 0.1244690223 | 0.1562492001 | 0.7966058211 | 0.4256799956 | 1.132546936 |
| enpp2 | -0.136194813 | 0.1711480124 | -0.795772101 | 0.4261645101 | 0.8726726075 |
| cxcl10 | 0.1227026957 | 0.1542221936 | 0.7956228145 | 0.4262513019 | 1.130548254 |
| slc27a4 | 0.1184850072 | 0.148957545 | 0.7954280346 | 0.4263645577 | 1.125789995 |
| uso1 | 0.1146860026 | 0.1444433658 | 0.7939859469 | 0.4272036126 | 1.121521227 |
| nup50 | 0.1260044767 | 0.1587773462 | 0.7935922831 | 0.4274328264 | 1.134287246 |
| f2r | 0.1156133099 | 0.1457318715 | 0.7933289313 | 0.427586205 | 1.122561705 |
| pikfyve | 0.1254033579 | 0.1581217957 | 0.7930807852 | 0.427730757 | 1.13360561 |
| rbm19 | 0.128557977 | 0.1621347241 | 0.7929083525 | 0.4278312206 | 1.13718735 |
| stx16 | 0.1126871443 | 0.1422748661 | 0.7920383084 | 0.4283383396 | 1.119281705 |
| tnfrsf17 | -0.121703961 | 0.1537493363 | -0.791573897 | 0.4286091724 | 0.8854104455 |
| entpd5 | 0.1159813519 | 0.1466279058 | 0.7909909869 | 0.4289492511 | 1.122974931 |
| plxdc1 | 0.1155249706 | 0.1463871693 | 0.7891741547 | 0.4300102255 | 1.122462543 |
| dpt | 0.1281757959 | 0.162478986 | 0.7888761436 | 0.4301844003 | 1.136752822 |
| folr2 | 0.1207736494 | 0.1531821112 | 0.7884318114 | 0.4304441695 | 1.128369476 |
| dynlt3 | 0.1206341979 | 0.1532564866 | 0.7871392625 | 0.4312003477 | 1.128212134 |
| lifr | 0.120618914 | 0.1533409296 | 0.7866061225 | 0.4315124741 | 1.128194891 |
| atg16l1 | 0.1209717146 | 0.1537939858 | 0.7865828691 | 0.4315260908 | 1.128592989 |
| amigo2 | -0.112287293 | 0.1428086012 | -0.786278223 | 0.431704508 | 0.8937874416 |
| kiaa0319 | 0.1244975537 | 0.1584311669 | 0.7858147868 | 0.4319760024 | 1.13257925 |
| ret | -0.118428297 | 0.1508312428 | -0.785170861 | 0.4323533974 | 0.8883155085 |
| ulbp2 | 0.1158000095 | 0.1474905031 | 0.7851353613 | 0.4323742089 | 1.122771306 |
| gcnt1 | 0.1170990296 | 0.1492367966 | 0.784652527 | 0.4326573238 | 1.124230756 |
| cxcl13 | 0.1167371513 | 0.1488951664 | 0.7840224375 | 0.4330269447 | 1.123823995 |
| fmnl1 | 0.1172854603 | 0.1498867503 | 0.7824938501 | 0.4339243982 | 1.124440367 |
| polr2a | 0.1221026954 | 0.1561185724 | 0.7821151166 | 0.4341469236 | 1.129870128 |
| spon2 | 0.1179398148 | 0.1508305084 | 0.7819360693 | 0.434252146 | 1.12517639 |
| siglec1 | 0.1155076009 | 0.1477650707 | 0.7816975985 | 0.4343923133 | 1.122443046 |
| nubp1 | -0.120272197 | 0.1539167565 | -0.781410677 | 0.4345609934 | 0.8866790526 |
| bex3 | 0.1262202514 | 0.1615869071 | 0.7811291995 | 0.4347265098 | 1.134532023 |
| cd69 | 0.1106537398 | 0.1417925969 | 0.780391517 | 0.4351604595 | 1.117008064 |
| gys1 | 0.1128397654 | 0.1447004029 | 0.779816525 | 0.4354988781 | 1.119452544 |
| dock9 | 0.1208489038 | 0.1549968348 | 0.7796862687 | 0.4355755632 | 1.128454394 |
| igfl4 | -0.120702304 | 0.1549492633 | -0.778979528 | 0.4359917742 | 0.8862977676 |
| susd5 | -0.134419939 | 0.1726180921 | -0.778712923 | 0.436148842 | 0.8742228672 |
| crhr1 | 0.1127686132 | 0.1448869802 | 0.7783212344 | 0.4363796602 | 1.119372895 |

| Protein | beta | se | z | p_val | HR |
|---|---|---|---|---|---|
| dmp1 | -0.124296009 | 0.1597961348 | -0.777841151 | 0.4366626647 | 0.8831183908 |
| btd | 0.1217743346 | 0.1565690118 | 0.7777677918 | 0.4367059188 | 1.129499184 |
| hnrnpul1 | 0.1266713943 | 0.1629648646 | 0.7772926676 | 0.4369861201 | 1.135043975 |
| qpct | 0.1120593478 | 0.1442149308 | 0.7770301392 | 0.4371409888 | 1.118579244 |
| tdp1 | 0.123735627 | 0.1593341427 | 0.7765794886 | 0.4374069068 | 1.131716636 |
| siglec8 | 0.122642329 | 0.1580735582 | 0.7758560659 | 0.4378339757 | 1.130480009 |
| cd302 | 0.1202626304 | 0.1550203115 | 0.7757862776 | 0.4378751876 | 1.127793005 |
| cngb3 | 0.1204428229 | 0.1553228578 | 0.7754352748 | 0.4380824979 | 1.127996244 |
| cdh6 | -0.11190961 | 0.1444628365 | -0.774660203 | 0.4385404732 | 0.8941250736 |
| ppcdc | -0.113810793 | 0.1469397994 | -0.774540275 | 0.4386113608 | 0.8924267936 |
| ptprf | 0.1139821738 | 0.1471865039 | 0.7744064213 | 0.4386904875 | 1.120732146 |
| cd109 | 0.1122280047 | 0.1449225196 | 0.7744000384 | 0.438694261 | 1.118767916 |
| mzb1 | -0.11378101 | 0.147130103 | -0.773336034 | 0.4393235354 | 0.8924533725 |
| bach1 | 0.1152818402 | 0.1491092974 | 0.7731364994 | 0.4394416021 | 1.122189671 |
| fcn2 | -0.112283685 | 0.1453291592 | -0.772616352 | 0.4397494642 | 0.8937906667 |
| glyr1 | 0.1212911518 | 0.1570308132 | 0.7724035134 | 0.4398754736 | 1.128953561 |
| anxa11 | 0.1163586593 | 0.1506830655 | 0.7722079378 | 0.4399912809 | 1.123398717 |
| phospho1 | 0.1215100126 | 0.1574415889 | 0.7717783688 | 0.4402457055 | 1.129200672 |
| serpina3 | 0.1181047523 | 0.1530325338 | 0.7717623788 | 0.4402551776 | 1.125361989 |
| ttr | 0.1244522258 | 0.1613083562 | 0.7715175377 | 0.4404002313 | 1.132527914 |
| pdlim5 | 0.1200585542 | 0.1558126797 | 0.7705313486 | 0.4409847668 | 1.127562873 |
| txk | -0.119364371 | 0.1551386957 | -0.769404242 | 0.4416533714 | 0.8874843687 |
| fam13a | -0.118849209 | 0.154605453 | -0.768725855 | 0.4420560729 | 0.8879416843 |
| tdgf1 | -0.109149817 | 0.1420602725 | -0.768334563 | 0.4422884458 | 0.8965960817 |
| krt19 | 0.1196608618 | 0.1557494127 | 0.7682909339 | 0.4423143596 | 1.127114539 |
| znrf4 | 0.1178173412 | 0.1533761384 | 0.768159522 | 0.4423924183 | 1.125038594 |
| pon1 | 0.1189697109 | 0.1549212571 | 0.7679366482 | 0.4425248233 | 1.126335802 |
| osbpl2 | 0.1188813846 | 0.1548470345 | 0.7677343325 | 0.4426450347 | 1.126236321 |
| reck | -0.125166503 | 0.163337756 | -0.766304777 | 0.4434949763 | 0.8823499766 |
| adamts15 | 0.125029317 | 0.1632192863 | 0.766020486 | 0.4436641128 | 1.133181674 |
| extl1 | 0.1216471039 | 0.1588976967 | 0.7655687053 | 0.4439329712 | 1.129355486 |
| ercc1 | -0.122450859 | 0.1600008482 | -0.765313809 | 0.4440847031 | 0.8847493817 |
| bin2 | 0.1097229278 | 0.1434204681 | 0.7650437157 | 0.4442455136 | 1.115968824 |
| ntf3 | 0.1163506684 | 0.152121694 | 0.7648525686 | 0.4443593406 | 1.12338974 |
| slamf6 | 0.1106371189 | 0.1447366038 | 0.7644031708 | 0.4446270198 | 1.116989499 |
| dusp3 | 0.1102504715 | 0.1442522844 | 0.7642892585 | 0.4446948851 | 1.116557701 |
| lrig3 | 0.1217428195 | 0.1594315709 | 0.7636054692 | 0.445102389 | 1.129463588 |
| cfhr4 | -0.113811613 | 0.1490680462 | -0.763487654 | 0.4451726223 | 0.8924260616 |
| serpina9 | 0.1151000802 | 0.1508269809 | 0.7631265941 | 0.4453879012 | 1.121985721 |
| eif4g1 | 0.1094132076 | 0.1433972432 | 0.7630077479 | 0.4454587752 | 1.115623239 |
| got1 | -0.120706728 | 0.1583315198 | -0.762367013 | 0.4458409881 | 0.8862938468 |
| adam12 | 0.129473214 | 0.1699277055 | 0.7619311614 | 0.4461010906 | 1.138228622 |
| cd2 | 0.1231664512 | 0.1617038841 | 0.7616789908 | 0.4462516173 | 1.131072674 |
| ace2 | 0.1272467988 | 0.1671859799 | 0.7611092678 | 0.4465918054 | 1.135697272 |
| proc | -0.113081706 | 0.1486197801 | -0.760879244 | 0.446729197 | 0.8930776874 |
| dag1 | 0.1122004252 | 0.1475216822 | 0.7605690467 | 0.4469145137 | 1.118737061 |
| thap12 | -0.121888708 | 0.1604707853 | -0.759569464 | 0.4475119777 | 0.8852468836 |
| septin9 | -0.109616446 | 0.1443332337 | -0.759467817 | 0.4475727591 | 0.8961778021 |
| plxdc2 | 0.1170931669 | 0.1543535099 | 0.7586038498 | 0.448089569 | 1.124224165 |
| sftpa2 | 0.111997588 | 0.1477113339 | 0.7582193257 | 0.4483196937 | 1.118510163 |
| dnajb8 | 0.1101915806 | 0.1453368094 | 0.7581808153 | 0.4483427445 | 1.116491948 |
| serpini1 | 0.1223088198 | 0.1617880495 | 0.7559817934 | 0.4496601117 | 1.130103046 |
| fcrl1 | 0.1075694619 | 0.1424314663 | 0.7552366389 | 0.4501070085 | 1.113568209 |
| il19 | 0.1109397371 | 0.1471931005 | 0.753702019 | 0.4510281693 | 1.117327571 |
| itih3 | 0.1139174038 | 0.1516175849 | 0.7513469095 | 0.4524439049 | 1.120659559 |
| ces1 | -0.111909181 | 0.1490084989 | -0.75102549 | 0.4526373157 | 0.8941254575 |
| atf4 | 0.1198969797 | 0.1596532811 | 0.7509834992 | 0.4526625864 | 1.127380703 |
| cd164 | 0.1087317906 | 0.1448356925 | 0.7507251061 | 0.4528181104 | 1.114863293 |
| s100a13 | -0.120991265 | 0.1613637251 | -0.749804611 | 0.4533723912 | 0.886041699 |
| ifnlr1 | -0.119430609 | 0.1595141799 | -0.748714684 | 0.4540291911 | 0.8874255852 |
| pnpt1 | -0.107915098 | 0.1442167938 | -0.748283853 | 0.454288962 | 0.89770381 |
| ltbp3 | 0.1173003596 | 0.1568417276 | 0.7478899996 | 0.45452651 | 1.12445712 |
| nudt10 | 0.1150426418 | 0.154081989 | 0.7466326375 | 0.4552853418 | 1.121921277 |
| nfat5 | 0.1142773442 | 0.1530970255 | 0.7464373906 | 0.4554032394 | 1.121063002 |
| mdk | 0.1101785357 | 0.1476275614 | 0.7463276816 | 0.4554694935 | 1.116477384 |
| stx1b | -0.119325189 | 0.1600502104 | -0.745548469 | 0.4559402215 | 0.887519142 |
| fyb1 | -0.107990944 | 0.1450043732 | -0.744742673 | 0.4564272968 | 0.897635725 |
| ift20 | -0.120579837 | 0.1620043693 | -0.744299905 | 0.4566950585 | 0.8864063167 |
| pacs2 | 0.1181382827 | 0.159074345 | 0.742660815 | 0.4576870592 | 1.125399724 |
| il16 | 0.1136234954 | 0.1530423657 | 0.7424316453 | 0.4578258522 | 1.120330236 |

| Protein | beta | se | z | p_val | HR |
|---|---|---|---|---|---|
| stc2 | 0.1144404097 | 0.1543274391 | 0.7415428545 | 0.4583643579 | 1.121245824 |
| mars1 | 0.1165679144 | 0.1573956467 | 0.7406044379 | 0.4589333164 | 1.123633819 |
| fzd8 | 0.114566449 | 0.1548659253 | 0.7397782872 | 0.459434536 | 1.121387154 |
| prrt3 | -0.117226296 | 0.1586010636 | -0.739126795 | 0.4598300077 | 0.8893839066 |
| maea | -0.107325541 | 0.1453055172 | -0.738619859 | 0.460137862 | 0.898233214 |
| crym | 0.1133763615 | 0.1536444602 | 0.737913761 | 0.4605668562 | 1.120053399 |
| htra2 | 0.1053320562 | 0.1427860713 | 0.7376913956 | 0.460702002 | 1.11107949 |
| ptprr | -0.115063562 | 0.156174455 | -0.736763011 | 0.4612664805 | 0.8913094889 |
| sat1 | 0.1140331316 | 0.1548522443 | 0.7363996057 | 0.4614875445 | 1.120789258 |
| cd27 | 0.1090281293 | 0.1481759655 | 0.7358017132 | 0.4618513785 | 1.115193719 |
| s100a4 | -0.106168759 | 0.144294728 | -0.735777119 | 0.4618663484 | 0.8992728747 |
| cpa2 | -0.107538827 | 0.1462463768 | -0.735326439 | 0.4621407103 | 0.8980416531 |
| lcn15 | 0.1174921436 | 0.1598230871 | 0.7351387445 | 0.4622550002 | 1.124672794 |
| sele | -0.11075487 | 0.1507229335 | -0.734824275 | 0.4624465208 | 0.8951581518 |
| pqbp1 | 0.1078417501 | 0.1467769806 | 0.7347320382 | 0.4625027043 | 1.113871461 |
| lamb1 | -0.111942899 | 0.1524121501 | -0.7344749 | 0.4626593523 | 0.89409531 |
| actn4 | 0.1054970891 | 0.1438146262 | 0.7335630028 | 0.4632151164 | 1.11126287 |
| sell | 0.1129340616 | 0.1539683392 | 0.7334888602 | 0.4632603197 | 1.119558109 |
| paip2b | 0.1173360045 | 0.1600024566 | 0.7333387686 | 0.463351835 | 1.124497202 |
| stam | 0.1136799618 | 0.1554181441 | 0.7314458838 | 0.4645068477 | 1.120393499 |
| ddc | -0.108230731 | 0.1480483949 | -0.731049674 | 0.4647488124 | 0.89742051 |
| pinlyp | -0.116935971 | 0.1600127232 | -0.730791703 | 0.4649063922 | 0.8896421547 |
| psmg3 | -0.113336705 | 0.1551424913 | -0.730532973 | 0.4650644659 | 0.8928499821 |
| nrxn3 | 0.1163601722 | 0.1593086523 | 0.7304071093 | 0.465141374 | 1.123400417 |
| phldb2 | 0.118943645 | 0.1629132379 | 0.7301042355 | 0.4653264726 | 1.126306443 |
| tgfa | 0.1150861524 | 0.1577360011 | 0.7296124637 | 0.4656271015 | 1.121970094 |
| serping1 | 0.1158154846 | 0.1589437508 | 0.7286570502 | 0.4662114714 | 1.122788681 |
| col24a1 | 0.1129514763 | 0.1550196798 | 0.728626691 | 0.466230047 | 1.119577606 |
| angptl2 | 0.1102787351 | 0.1514149737 | 0.7283211985 | 0.4664169887 | 1.11658926 |
| atp1b2 | 0.1151547965 | 0.1582805853 | 0.7275358268 | 0.4668977764 | 1.122047113 |
| mdga1 | 0.10771748 | 0.1480874236 | 0.7273911409 | 0.46698638 | 1.113733049 |
| lgals1 | 0.1152759439 | 0.1586763704 | 0.726484628 | 0.4675417277 | 1.122183054 |
| agrp | -0.108244551 | 0.1490199975 | -0.726376007 | 0.4676082955 | 0.8974081077 |
| pcdh7 | -0.11450352 | 0.157645244 | -0.726336661 | 0.4676324099 | 0.8918087991 |
| clstn2 | 0.1068817093 | 0.1473866918 | 0.7251788339 | 0.4683423261 | 1.112802613 |
| echdc3 | 0.1167923705 | 0.1614166164 | 0.7235461446 | 0.4693444149 | 1.123886054 |
| islr2 | -0.105667504 | 0.1461918815 | -0.722800084 | 0.4698027156 | 0.8997237526 |
| vav3 | 0.1109754361 | 0.1538347809 | 0.7213936633 | 0.4706673442 | 1.11736746 |
| eln | 0.1441476483 | 0.1998968993 | 0.7211099765 | 0.4708418534 | 1.155054638 |
| tspan7 | 0.1098290194 | 0.1526287256 | 0.7195828892 | 0.4717818503 | 1.116087225 |
| pdgfa | 0.1022865566 | 0.1423026564 | 0.7187958337 | 0.4722667251 | 1.107700845 |
| luzp2 | -0.113885798 | 0.1587054016 | -0.717592448 | 0.4730086155 | 0.8923598597 |
| acp5 | 0.1072139081 | 0.149643682 | 0.7164613077 | 0.4737065507 | 1.113172346 |
| eci2 | 0.112622337 | 0.1572394783 | 0.716247206 | 0.4738387193 | 1.119209169 |
| pdgfrb | -0.101780361 | 0.1421069761 | -0.716223538 | 0.4738533314 | 0.9032279138 |
| cd300e | 0.1100847342 | 0.1538202421 | 0.7156713101 | 0.4741943301 | 1.116372661 |
| chgb | 0.1064943344 | 0.1488235848 | 0.7155743127 | 0.4742542395 | 1.112371624 |
| cdh2 | -0.113772178 | 0.1596273203 | -0.712736251 | 0.47600898 | 0.8924612552 |
| adamts16 | 0.1133814994 | 0.1592080149 | 0.7121594942 | 0.4763660165 | 1.120059153 |
| thop1 | -0.108185831 | 0.1520102753 | -0.711700776 | 0.476650087 | 0.897460805 |
| tbc1d23 | 0.1023106689 | 0.1437960591 | 0.7114984198 | 0.4767754297 | 1.107727555 |
| ceacam18 | 0.1116327852 | 0.1569044649 | 0.7114697807 | 0.4767931706 | 1.118102202 |
| c2cd2l | -0.103989142 | 0.146247188 | -0.711050543 | 0.477052916 | 0.9012350826 |
| eno3 | -0.1193937 | 0.1680373924 | -0.710518641 | 0.4773825753 | 0.8874583398 |
| dctn6 | 0.102726934 | 0.1447800946 | 0.709537691 | 0.4779908705 | 1.108188759 |
| susd4 | -0.113279909 | 0.1598657057 | -0.70859418 | 0.4785763492 | 0.8929006946 |
| otud7b | 0.112097444 | 0.1584089356 | 0.7076459646 | 0.4791651412 | 1.118621858 |
| qsox1 | 0.1111547897 | 0.1574608193 | 0.7059203057 | 0.4802376991 | 1.117567881 |
| vwa1 | 0.106380503 | 0.1507943166 | 0.7054675892 | 0.4805192949 | 1.112245009 |
| nptx2 | -0.130310212 | 0.1848335443 | -0.705013871 | 0.4808016043 | 0.877823077 |
| efcab2 | -0.112568014 | 0.1598548136 | -0.704189077 | 0.4813150326 | 0.8935365724 |
| ddx25 | 0.1122987842 | 0.1595219115 | 0.7039709035 | 0.4814508938 | 1.118847105 |
| kiaa1549 | 0.1076265987 | 0.1528951784 | 0.7039240856 | 0.4814800511 | 1.113631836 |
| rnase6 | 0.119148568 | 0.1695188154 | 0.7028633826 | 0.4821408928 | 1.126537273 |
| ldlr | -0.109147037 | 0.1554080015 | -0.702325721 | 0.4824760561 | 0.8965985749 |
| pcsk7 | -0.106516018 | 0.1517555215 | -0.701892209 | 0.4827463881 | 0.8989606483 |
| fuca1 | -0.096417436 | 0.1374812906 | -0.701313146 | 0.4831076114 | 0.9080848692 |
| eda2r | 0.1281193677 | 0.1828070547 | 0.7008447672 | 0.4833998967 | 1.136688678 |
| fam3c | 0.105417125 | 0.1507979081 | 0.6990622506 | 0.4845131285 | 1.111174012 |
| cinp | -0.109453995 | 0.1573473781 | -0.695620074 | 0.4866667912 | 0.8963233989 |

| Protein | beta | se | z | p_val | HR |
|---|---|---|---|---|---|
| srp14 | 0.1040196609 | 0.1501553384 | 0.6927470044 | 0.4884683354 | 1.109622271 |
| ckap4 | 0.1055279008 | 0.1523336346 | 0.6927419609 | 0.488471501 | 1.11129711 |
| eif2ak2 | 0.1064203752 | 0.1538765146 | 0.6915959557 | 0.4891911022 | 1.112289357 |
| adgre2 | 0.1012821281 | 0.1464611603 | 0.691528921 | 0.4892332123 | 1.106588798 |
| cd70 | -0.107823465 | 0.1559308007 | -0.691482788 | 0.4892621935 | 0.8977860733 |
| prap1 | 0.126218017 | 0.1830121562 | 0.6896701272 | 0.4904016559 | 1.134529488 |
| megf11 | -0.107495344 | 0.1559993148 | -0.689075746 | 0.4907756018 | 0.8980807036 |
| sord | -0.103589594 | 0.1503644355 | -0.688923508 | 0.4908714053 | 0.9015952413 |
| clip2 | 0.1032899423 | 0.1500040119 | 0.6885811986 | 0.4910868561 | 1.108812854 |
| lamtor5 | 0.1076651093 | 0.156360532 | 0.6885696022 | 0.4910941558 | 1.113674724 |
| gkn1 | -0.100454326 | 0.146042817 | -0.687841607 | 0.4915525314 | 0.9044264202 |
| arhgef12 | 0.1030848543 | 0.1499132922 | 0.6876298477 | 0.4916859072 | 1.108585473 |
| dapk2 | -0.106851254 | 0.1555250802 | -0.68703552 | 0.4920603452 | 0.8986593347 |
| smad3 | 0.1052497025 | 0.1535574133 | 0.6854094523 | 0.493085581 | 1.110987992 |
| nos1 | -0.109606321 | 0.160068029 | -0.684748366 | 0.4935027227 | 0.8961868755 |
| dll4 | -0.105461544 | 0.154035464 | -0.684657553 | 0.4935600404 | 0.8999090793 |
| mmp13 | -0.1014435 | 0.1483373302 | -0.683870338 | 0.4940570459 | 0.9035322274 |
| fmr1 | 0.09945434065 | 0.1457013789 | 0.6825902502 | 0.4948657966 | 1.104568036 |
| snca | -0.108604127 | 0.1592514503 | -0.681966331 | 0.4952602405 | 0.8970854788 |
| lif | -0.098997961 | 0.1452083841 | -0.681764774 | 0.495387702 | 0.9057445547 |
| cdh5 | -0.097417269 | 0.1432799981 | -0.679908361 | 0.4965624873 | 0.9071773904 |
| atp5po | -0.098232545 | 0.144534125 | -0.679649496 | 0.4967264213 | 0.9064380914 |
| hras | -0.104702697 | 0.1541114652 | -0.679395895 | 0.4968870505 | 0.9005922318 |
| ccn4 | 0.102137615 | 0.1503914862 | 0.6791449276 | 0.497046038 | 1.107535875 |
| mydgf | 0.1050534473 | 0.1547193556 | 0.6789935679 | 0.4971419375 | 1.110769976 |
| bank1 | 0.1019893074 | 0.1502995469 | 0.6785736186 | 0.4974080632 | 1.107371631 |
| eps8l2 | 0.1026453286 | 0.151386597 | 0.6780344538 | 0.4977498482 | 1.108098329 |
| tlr1 | 0.1059732872 | 0.1565091433 | 0.677106046 | 0.4983386731 | 1.111792177 |
| gnly | 0.09739848918 | 0.1438972663 | 0.6768612893 | 0.4984939671 | 1.102299541 |
| ica1 | 0.09797248279 | 0.1447455587 | 0.676860027 | 0.4984947681 | 1.102932435 |
| dbn1 | 0.1064055675 | 0.1572273534 | 0.676762441 | 0.4985566919 | 1.112272887 |
| tnfsf8 | 0.1033623697 | 0.1527945711 | 0.6764793335 | 0.4987363629 | 1.108893166 |
| slc9a3r1 | 0.1062469974 | 0.1571890913 | 0.6759183891 | 0.4990924617 | 1.112096528 |
| cdh17 | -0.097370952 | 0.1442290443 | -0.675113343 | 0.4996037571 | 0.9072194087 |
| anxa1 | 0.1112400398 | 0.1647856635 | 0.6750589672 | 0.4996383018 | 1.117663158 |
| chchd6 | 0.1095743691 | 0.1627519401 | 0.6732599871 | 0.5007819038 | 1.115803049 |
| msr1 | 0.10579285 | 0.1573680815 | 0.6722637076 | 0.5014158298 | 1.111591586 |
| tomm20 | 0.1037397417 | 0.1543154928 | 0.6722574633 | 0.5014198044 | 1.10931171 |
| smpdl3a | -0.095032455 | 0.1414839947 | -0.67168343 | 0.5017852523 | 0.9093434214 |
| vnn2 | 0.09741032231 | 0.1451275196 | 0.6712050381 | 0.5020899196 | 1.102312584 |
| htr1a | -0.1018614 | 0.1522491426 | -0.66904416 | 0.5034673088 | 0.9031547205 |
| ccn1 | -0.098904519 | 0.14787807 | -0.66882479 | 0.503607251 | 0.9058291932 |
| myom3 | -0.114712041 | 0.1715899265 | -0.668524333 | 0.5037989551 | 0.8916228577 |
| hbz | -0.100846246 | 0.151053218 | -0.667620641 | 0.5043757788 | 0.9040720267 |
| acadsb | 0.1006289569 | 0.150895068 | 0.6668803596 | 0.5048485572 | 1.105866242 |
| pdcd5 | -0.099071559 | 0.1486072054 | -0.666667262 | 0.5049846947 | 0.9056778965 |
| btc | -0.095269885 | 0.1429152595 | -0.666618004 | 0.5050161658 | 0.9091275414 |
| tg | -0.102128905 | 0.1532246071 | -0.666530701 | 0.5050719472 | 0.9029131544 |
| rp2 | 0.09709801722 | 0.1457009352 | 0.6664200001 | 0.5051426823 | 1.10196838 |
| znrd2 | 0.1055370955 | 0.1585956644 | 0.6654475449 | 0.5057642835 | 1.111307328 |
| cat | -0.110417924 | 0.1659625557 | -0.665318294 | 0.5058469323 | 0.8954598226 |
| axl | 0.0950089832 | 0.1429970632 | 0.6644121288 | 0.5064265721 | 1.099668734 |
| igfbpl1 | -0.101387645 | 0.1526763707 | -0.664069002 | 0.5066461487 | 0.9035826956 |
| ceacam1 | -0.094962019 | 0.143065388 | -0.663766549 | 0.5068397382 | 0.9094074741 |
| tbr1 | 0.1064293741 | 0.1603463395 | 0.6637468269 | 0.5068523632 | 1.112299366 |
| cx3cl1 | 0.09533850075 | 0.143801593 | 0.6629864019 | 0.5073392631 | 1.100031153 |
| tinagl1 | -0.099508401 | 0.1501452946 | -0.662747382 | 0.5074923585 | 0.9052823447 |
| cep290 | -0.103885573 | 0.1568297336 | -0.662409929 | 0.5077085422 | 0.9013284281 |
| gpi | 0.1028646874 | 0.1555710464 | 0.661207145 | 0.5084794798 | 1.108341426 |
| gpr37 | 0.09975443955 | 0.1509324606 | 0.6609210448 | 0.5086629491 | 1.104899565 |
| ubac1 | -0.097986416 | 0.1485668666 | -0.659544203 | 0.5095463707 | 0.9066612201 |
| gbp2 | -0.096259448 | 0.1461202473 | -0.658768718 | 0.5100442981 | 0.9082283475 |
| gp1ba | -0.092149058 | 0.1400099097 | -0.658160973 | 0.5104346997 | 0.9119692027 |
| mthfd2 | 0.1004176083 | 0.152652161 | 0.6578197628 | 0.5106539537 | 1.105632543 |
| gas2 | -0.105462441 | 0.1604064191 | -0.657470202 | 0.5108786247 | 0.8999082721 |
| btn3a2 | -0.100203561 | 0.1524449134 | -0.657309967 | 0.5109816292 | 0.9046532472 |
| apof | -0.114068111 | 0.1736648546 | -0.656828989 | 0.5112908825 | 0.8921971856 |
| xpnpep2 | -0.094511476 | 0.1443190308 | -0.654878819 | 0.5125457783 | 0.9098172931 |
| adgre1 | 0.09982717525 | 0.1526557653 | 0.6539364896 | 0.5131527238 | 1.104979934 |
| itgb2 | -0.09874442 | 0.1511252747 | -0.653394475 | 0.5135019998 | 0.9059742278 |

| Protein | beta | se | z | p_val | HR |
|---|---|---|---|---|---|
| sod2 | 0.09457485636 | 0.1447961144 | 0.6531588004 | 0.5136539078 | 1.099191442 |
| pmch | -0.10313733 | 0.1579905558 | -0.652806934 | 0.513880753 | 0.9020030926 |
| lgals4 | 0.09821535075 | 0.1505598583 | 0.65233424 | 0.5141855762 | 1.103200335 |
| rap1a | 0.1018601763 | 0.1562597097 | 0.6518646203 | 0.5144885103 | 1.107228644 |
| ephb4 | 0.09384562725 | 0.1439840142 | 0.6517781002 | 0.5145443313 | 1.098390171 |
| efnb2 | -0.104668914 | 0.1606996088 | -0.651332725 | 0.5148317279 | 0.9006226567 |
| plxnb3 | -0.093381778 | 0.1434045964 | -0.651177024 | 0.5149322198 | 0.9108456927 |
| cfp | -0.102531921 | 0.1575381768 | -0.650838566 | 0.5151507021 | 0.902549339 |
| fabp3 | -0.118696506 | 0.182609253 | -0.650002692 | 0.5156904829 | 0.888077286 |
| il12b | 0.09541931862 | 0.146875665 | 0.6496605046 | 0.5159115411 | 1.100120059 |
| fus | 0.09650820519 | 0.1486524049 | 0.6492206114 | 0.516195791 | 1.101318618 |
| ctla4 | 0.09823690873 | 0.1513726685 | 0.6489738846 | 0.5163552564 | 1.103224118 |
| cd300lg | 0.09686364845 | 0.1493075484 | 0.6487525213 | 0.5164983505 | 1.101710143 |
| prkd2 | -0.101549334 | 0.1568315832 | -0.647505637 | 0.5173047476 | 0.903436608 |
| rgs8 | 0.09209298969 | 0.1425568208 | 0.6460090029 | 0.518273525 | 1.096466777 |
| gpkow | -0.096289418 | 0.1492755202 | -0.645044936 | 0.5188980657 | 0.9082011279 |
| enpp5 | -0.102509904 | 0.1593624301 | -0.64325013 | 0.52006181 | 0.9025692108 |
| vps4b | 0.09786052977 | 0.1521563961 | 0.6431575159 | 0.520121897 | 1.102808965 |
| ckb | 0.1119312818 | 0.1740639448 | 0.64304691 | 0.5201936617 | 1.118436001 |
| pvr | 0.09264358064 | 0.1441633116 | 0.6426293874 | 0.5204646096 | 1.097070648 |
| glb1 | 0.09834406127 | 0.1532332832 | 0.6417930834 | 0.5210075412 | 1.103342337 |
| adam22 | 0.09278699875 | 0.1448560987 | 0.6405460289 | 0.5218176747 | 1.097227999 |
| il20 | 0.08921795805 | 0.1393433829 | 0.6402740928 | 0.5219944205 | 1.093318928 |
| fcar | 0.09688151701 | 0.1515657991 | 0.6392043427 | 0.5226900074 | 1.10172983 |
| cd55 | -0.094549284 | 0.1481282251 | -0.638293503 | 0.5232826407 | 0.9097828961 |
| lats1 | 0.0993508588 | 0.155764748 | 0.6378263382 | 0.5235867326 | 1.104453739 |
| lyar | 0.09122716702 | 0.1430608251 | 0.6376809793 | 0.5236813697 | 1.095517843 |
| ccl17 | 0.09102395512 | 0.1429260223 | 0.6368606194 | 0.5242156361 | 1.095295243 |
| adam23 | -0.088979245 | 0.1400587307 | -0.635299522 | 0.5252330855 | 0.9148645617 |
| hspg2 | 0.1000726044 | 0.1576089256 | 0.6349424945 | 0.5254659208 | 1.105251161 |
| defb104a_defb104b | 0.1034154728 | 0.1629184357 | 0.6347683881 | 0.5255794834 | 1.108952053 |
| septin8 | 0.1062647051 | 0.1674822643 | 0.6344833323 | 0.525765441 | 1.11211622 |
| sema3f | -0.096973719 | 0.1529859473 | -0.633873374 | 0.5261634632 | 0.9075798583 |
| atox1 | 0.09188598031 | 0.1449970884 | 0.6337091408 | 0.5262706584 | 1.096239822 |
| ncf2 | 0.09605130187 | 0.1517647923 | 0.6328958146 | 0.5268016812 | 1.100815536 |
| sh2b3 | 0.09060745268 | 0.1433368506 | 0.6321295066 | 0.5273022558 | 1.094839145 |
| adra2a | -0.100669721 | 0.159520317 | -0.631077738 | 0.5279896964 | 0.9042316325 |
| nectin2 | 0.09238271542 | 0.1466055128 | 0.6301448945 | 0.528599789 | 1.096784498 |
| pth1r | 0.09580874027 | 0.1521189861 | 0.6298276284 | 0.5288073672 | 1.100548553 |
| maged1 | 0.0895961563 | 0.1423100273 | 0.6295842814 | 0.5289666104 | 1.093732498 |
| bmp4 | -0.093694049 | 0.1489783065 | -0.628910689 | 0.5294075278 | 0.9105613064 |
| ankra2 | -0.101910071 | 0.1620869497 | -0.628737054 | 0.5295212152 | 0.9031107636 |
| per3 | 0.09682488179 | 0.1540003492 | 0.628731573 | 0.5295248043 | 1.101667435 |
| apcs | 0.1123677671 | 0.1791357215 | 0.627277274 | 0.5304774976 | 1.118924288 |
| rasa1 | -0.09185681 | 0.1465347182 | -0.626860384 | 0.5307507576 | 0.9122357635 |
| tspan8 | -0.099881207 | 0.159338126 | -0.626850646 | 0.5307571416 | 0.9049449126 |
| dusp13 | -0.09761251 | 0.1557573527 | -0.626695999 | 0.530858527 | 0.9070002894 |
| lta4h | -0.091722732 | 0.1465041507 | -0.626075992 | 0.5312650983 | 0.9123580826 |
| gba | -0.099070101 | 0.1584058717 | -0.625419374 | 0.5316958496 | 0.9056792166 |
| phldb1 | 0.1028329516 | 0.1645434657 | 0.624959193 | 0.531997841 | 1.108306253 |
| wfdc12 | -0.098610403 | 0.157858716 | -0.624675059 | 0.5321843459 | 0.9060956517 |
| crygd | 0.09482647344 | 0.1518066117 | 0.6246531188 | 0.5321987485 | 1.099468052 |
| fgf3 | 0.09787169207 | 0.1567037188 | 0.6245652165 | 0.5322564548 | 1.102821275 |
| clint1 | 0.09879855823 | 0.15828099 | 0.6241972473 | 0.5324980545 | 1.103843917 |
| akt1s1 | -0.091563558 | 0.1467456488 | -0.623960977 | 0.5326532133 | 0.9125033171 |
| foxo1 | 0.09389764578 | 0.1506550145 | 0.6232626646 | 0.5331119282 | 1.098447309 |
| fgfr2 | 0.09724002617 | 0.156035049 | 0.6231934863 | 0.5331573816 | 1.102124881 |
| cxcl12 | 0.09040358836 | 0.145126857 | 0.6229280384 | 0.5333318118 | 1.094615969 |
| wfdc1 | -0.100581629 | 0.1614755484 | -0.622890771 | 0.5333563031 | 0.9043112915 |
| gbp4 | -0.090530075 | 0.1454607552 | -0.622367693 | 0.5337001187 | 0.9134468619 |
| bcat2 | 0.09533839323 | 0.1532608056 | 0.6220663715 | 0.5338982263 | 1.100031035 |
| atp1b3 | 0.09861260759 | 0.1587675744 | 0.6211130197 | 0.5345252634 | 1.103638675 |
| ebag9 | -0.088878056 | 0.1431615011 | -0.620823721 | 0.5347156137 | 0.9149571404 |
| cbx2 | 0.09876634691 | 0.1591060141 | 0.6207580994 | 0.5347587959 | 1.103808361 |
| ndufs6 | 0.09185310596 | 0.1480554057 | 0.6203968407 | 0.5349965519 | 1.096203784 |
| dpp10 | -0.094089684 | 0.1519414626 | -0.619249561 | 0.5357519671 | 0.9102011282 |
| clec4g | 0.09566750383 | 0.1545463386 | 0.6190214837 | 0.535902206 | 1.100393127 |
| kitlg | 0.08801384371 | 0.1423475719 | 0.6183023886 | 0.5363760277 | 1.092003239 |
| relt | 0.1003787501 | 0.1624583565 | 0.6178737264 | 0.5366585793 | 1.105589581 |
| thsd1 | 0.09735202574 | 0.1577782301 | 0.6170181125 | 0.537222779 | 1.102248325 |

| Protein | beta | se | z | p_val | HR |
|---|---|---|---|---|---|
| ppp3r1 | -0.091804322 | 0.1488070327 | -0.616935368 | 0.5372773572 | 0.9122836464 |
| hrg | -0.09601184 | 0.1557326511 | -0.616517087 | 0.537553298 | 0.9084532596 |
| kdm3a | 0.09632193671 | 0.1562448915 | 0.6164805504 | 0.5375774047 | 1.101113496 |
| ogn | 0.09646602992 | 0.1565311356 | 0.6162737498 | 0.5377138609 | 1.10127217 |
| ang | 0.09449736542 | 0.153405606 | 0.6159968198 | 0.5378966187 | 1.099106267 |
| tsc22d1 | -0.09628862 | 0.1563494251 | -0.615855285 | 0.5379900359 | 0.9082018533 |
| efcab14 | 0.09986120287 | 0.1622697726 | 0.6154023714 | 0.5382890257 | 1.105017534 |
| dhps | -0.095723963 | 0.1562780876 | -0.612523254 | 0.5401916176 | 0.9087148206 |
| tnfrsf10b | 0.1016563589 | 0.1659880693 | 0.6124317206 | 0.5402521601 | 1.107002995 |
| spaca5_spaca5b | 0.09576540296 | 0.1565233847 | 0.6118280864 | 0.5406515051 | 1.100500859 |
| grik2 | 0.09407595367 | 0.1539114239 | 0.6112343792 | 0.5410444265 | 1.098643189 |
| dctd | 0.09618042185 | 0.157626431 | 0.6101795316 | 0.541742887 | 1.100957683 |
| clic5 | 0.09721208247 | 0.1593281974 | 0.6101373394 | 0.5417708337 | 1.102094084 |
| slitrk1 | 0.1056526641 | 0.1732149647 | 0.609951134 | 0.5418941783 | 1.111435768 |
| wwp2 | 0.08927695219 | 0.146397441 | 0.6098259065 | 0.5419771383 | 1.093383429 |
| mxra8 | -0.108976542 | 0.178727844 | -0.609734551 | 0.5420376629 | 0.8967514535 |
| akr1b10 | 0.09745408068 | 0.1598313451 | 0.6097307173 | 0.542040203 | 1.102360821 |
| nars1 | 0.09362240979 | 0.1536822688 | 0.6091946098 | 0.5423954527 | 1.098145019 |
| brd3 | 0.09695781469 | 0.1592365705 | 0.6088916283 | 0.5425962736 | 1.101813892 |
| ap1g2 | 0.09521244485 | 0.1564847016 | 0.6084457066 | 0.542891905 | 1.099892497 |
| lilrb2 | 0.08664351678 | 0.1424171189 | 0.6083785253 | 0.5429364509 | 1.090507863 |
| fis1 | 0.08850916675 | 0.1455069184 | 0.608281501 | 0.5430007881 | 1.092544268 |
| sil1 | -0.094209803 | 0.1549559592 | -0.607977925 | 0.5432021151 | 0.9100918026 |
| vwa5a | 0.09931140836 | 0.1633993106 | 0.6077835213 | 0.5433310599 | 1.104410169 |
| cep350 | 0.09840824642 | 0.1621313017 | 0.6069663625 | 0.5438732356 | 1.103413158 |
| nell1 | -0.085099938 | 0.1403477622 | -0.606350519 | 0.5442820185 | 0.9184204943 |
| lbp | 0.08855798263 | 0.1461119775 | 0.6060966673 | 0.5444505639 | 1.092597602 |
| pfkfb2 | -0.087149752 | 0.1438058001 | -0.6060239 | 0.5444988825 | 0.9165398318 |
| golga3 | -0.093692641 | 0.1546102459 | -0.605992447 | 0.5445197688 | 0.9105625886 |
| il1r1 | 0.08760847169 | 0.1446748482 | 0.6055542673 | 0.5448107786 | 1.091560661 |
| agt | 0.09439313329 | 0.1559198573 | 0.6053952008 | 0.5449164391 | 1.098991711 |
| ereg | 0.08835131268 | 0.1459621411 | 0.6053029371 | 0.5449777303 | 1.092371819 |
| scp2 | -0.088237313 | 0.145977716 | -0.604457419 | 0.5455395707 | 0.9155435801 |
| cd80 | 0.09495712968 | 0.1573929132 | 0.6033126128 | 0.5463007439 | 1.099611713 |
| npr1 | 0.09686061203 | 0.1606809145 | 0.6028134227 | 0.5466328163 | 1.101706798 |
| dtx3 | 0.09580532265 | 0.1594054553 | 0.6010165866 | 0.5478289389 | 1.100544792 |
| pcdhb15 | 0.09279539156 | 0.154427639 | 0.6008988557 | 0.5479073554 | 1.097237208 |
| atg4a | -0.087424183 | 0.1455227913 | -0.600759388 | 0.5480002571 | 0.9162883392 |
| rbm25 | -0.095782165 | 0.1596332714 | -0.600013794 | 0.5484970428 | 0.9086619331 |
| mstn | -0.095790748 | 0.1597763985 | -0.599530021 | 0.5488194967 | 0.9086541343 |
| atrn | -0.094717975 | 0.1584901812 | -0.597626768 | 0.550088999 | 0.9096294367 |
| iglon5 | 0.09609187986 | 0.1608291391 | 0.5974780464 | 0.55018826 | 1.100860206 |
| shmt1 | -0.087158059 | 0.1460754165 | -0.596664802 | 0.5507311971 | 0.9165322176 |
| ltbp2 | 0.09999930735 | 0.1678875873 | 0.5955953926 | 0.5514455554 | 1.105163263 |
| vps53 | 0.08557484319 | 0.1438043521 | 0.5950782568 | 0.551791162 | 1.089343088 |
| sez6 | -0.096393014 | 0.1620200661 | -0.594944913 | 0.5518802942 | 0.908107047 |
| penk | -0.094631846 | 0.1590783072 | -0.594875871 | 0.5519264475 | 0.909707785 |
| sv2a | 0.09413054784 | 0.158255563 | 0.5948008783 | 0.5519765804 | 1.09870317 |
| ncam2 | -0.089141694 | 0.1500249378 | -0.594179178 | 0.5523922778 | 0.9147159544 |
| hpgds | -0.085950102 | 0.1447841462 | -0.593643047 | 0.5527508832 | 0.9176400188 |
| gsta1 | 0.09474132345 | 0.159609183 | 0.5935831615 | 0.552790946 | 1.099374436 |
| usp28 | 0.09443351421 | 0.1591095318 | 0.5935126144 | 0.5528381435 | 1.09903609 |
| ppme1 | 0.08574326059 | 0.1445138944 | 0.5933219153 | 0.5529657349 | 1.089526568 |
| artn | 0.08488169849 | 0.1432183919 | 0.5926731711 | 0.5533998997 | 1.088588277 |
| paep | 0.09826987706 | 0.1658828188 | 0.5924053966 | 0.5535791535 | 1.10326049 |
| il2rg | 0.09281788111 | 0.1566927685 | 0.5923558696 | 0.553612311 | 1.097261885 |
| asgr1 | 0.09677825237 | 0.1634353016 | 0.5921502357 | 0.5537499898 | 1.101616066 |
| lrtm2 | 0.1001983034 | 0.1692117476 | 0.5921474412 | 0.553751861 | 1.105390099 |
| scgn | 0.08729327713 | 0.1475122683 | 0.5917696077 | 0.5540048775 | 1.091216662 |
| igf2r | 0.08854141345 | 0.1496443976 | 0.5916787724 | 0.5540657139 | 1.092579499 |
| ftcd | 0.1014386062 | 0.1714763623 | 0.5915602875 | 0.5541450733 | 1.106761968 |
| klk10 | -0.083548081 | 0.1414095033 | -0.590823663 | 0.5546385784 | 0.9198468587 |
| nfya | 0.0949399183 | 0.1608071041 | 0.590396294 | 0.5549249946 | 1.099592788 |
| havcr2 | 0.09130862977 | 0.1550296058 | 0.5889754366 | 0.5558777514 | 1.09560709 |
| fndc1 | -0.092124162 | 0.1564688109 | -0.588770127 | 0.5560154879 | 0.9119919081 |
| spon1 | 0.09039064822 | 0.1535251687 | 0.5887676201 | 0.5560171698 | 1.094601804 |
| adgre5 | 0.08627328056 | 0.1468387919 | 0.5875373903 | 0.5568428464 | 1.090104192 |
| kynu | -0.090724552 | 0.1549325511 | -0.585574504 | 0.5581614862 | 0.9132692347 |
| mnat1 | 0.09352897091 | 0.1598215008 | 0.5852089389 | 0.5584072351 | 1.098042414 |
| pkn3 | 0.09232811367 | 0.1577730051 | 0.5851958869 | 0.5584160102 | 1.096724613 |

| Protein | beta | se | z | p_val | HR |
|---|---|---|---|---|---|
| tspyl1 | 0.09248993797 | 0.1583027201 | 0.5842599413 | 0.5590454389 | 1.096902104 |
| sowaha | 0.09036128152 | 0.1546612093 | 0.584253039 | 0.559050082 | 1.09456966 |
| spring1 | 0.09622045056 | 0.1648841248 | 0.5835640675 | 0.5595136412 | 1.101001754 |
| casp4 | 0.09212861276 | 0.1579191596 | 0.5833909766 | 0.5596301308 | 1.096505838 |
| pcdh17 | -0.083503723 | 0.1431550056 | -0.583309838 | 0.559684741 | 0.9198876618 |
| morf4l2 | -0.093111251 | 0.1597323923 | -0.58292028 | 0.5599469675 | 0.9110921348 |
| creg1 | -0.085616258 | 0.1469239744 | -0.582724898 | 0.5600785093 | 0.9179464182 |
| wif1 | 0.08528296629 | 0.1467390945 | 0.5811877644 | 0.5611139131 | 1.089025181 |
| sdc4 | -0.082731047 | 0.1423653348 | -0.581117916 | 0.5611609846 | 0.920598712 |
| erp29 | 0.09091720025 | 0.1571224128 | 0.5786392829 | 0.5628325927 | 1.095178321 |
| mamdc4 | 0.09269225937 | 0.1603876734 | 0.5779263293 | 0.5633138584 | 1.097124054 |
| cpvl | -0.081601497 | 0.1414525143 | -0.576882621 | 0.5640187514 | 0.9216391613 |
| lelp1 | 0.08846824249 | 0.1534817386 | 0.5764089153 | 0.5643388198 | 1.092499557 |
| dand5 | 0.08790744587 | 0.1526879571 | 0.5757326742 | 0.5647958866 | 1.091887059 |
| pros1 | 0.09064271366 | 0.1575090428 | 0.5754762522 | 0.564969247 | 1.09487775 |
| itgax | -0.090874378 | 0.1581012568 | -0.574785929 | 0.5654360844 | 0.9131324135 |
| rangap1 | -0.083872215 | 0.1459290613 | -0.574746487 | 0.565462763 | 0.9195487528 |
| epha4 | -0.090320694 | 0.1573829696 | -0.573891156 | 0.5660414579 | 0.9136381396 |
| sel1l | 0.09444005077 | 0.1645858576 | 0.573804166 | 0.5661003293 | 1.099043274 |
| rarres1 | 0.08387157911 | 0.1464338434 | 0.5727608942 | 0.5668066005 | 1.087489229 |
| leo1 | 0.08831874237 | 0.1545087566 | 0.5716099482 | 0.5675862543 | 1.09233624 |
| dpp7 | -0.081657651 | 0.1429194629 | -0.571354306 | 0.5677594968 | 0.9215874097 |
| thy1 | 0.08673129579 | 0.1518342501 | 0.5712235266 | 0.5678481324 | 1.090603591 |
| metap1d | 0.08583524436 | 0.1508736359 | 0.5689214279 | 0.5694094629 | 1.089626791 |
| cgn | 0.09130224883 | 0.1605953466 | 0.5685236264 | 0.5696794676 | 1.095600099 |
| txn | -0.089859176 | 0.1581604187 | -0.568152114 | 0.5699316837 | 0.9140598975 |
| dnajb14 | 0.08877552886 | 0.1565597921 | 0.5670391334 | 0.5706875944 | 1.092835319 |
| mylpf | 0.09634410048 | 0.1701082313 | 0.5663694212 | 0.5711426775 | 1.101137901 |
| fkbp1b | 0.08047746337 | 0.1421822859 | 0.5660161031 | 0.5713828338 | 1.083804421 |
| pecam1 | 0.08324853473 | 0.1470811948 | 0.566003933 | 0.5713911069 | 1.086811885 |
| adam8 | 0.08387200056 | 0.1482320929 | 0.5658153974 | 0.5715192783 | 1.087489687 |
| sema7a | -0.081745709 | 0.1447283335 | -0.564821741 | 0.5721950181 | 0.9215062594 |
| rnf149 | 0.08969758166 | 0.1589281988 | 0.5643906011 | 0.572488334 | 1.093843435 |
| itgbl1 | -0.090726055 | 0.1608116766 | -0.564175793 | 0.5726345005 | 0.9132678617 |
| tpd52l2 | 0.08865916195 | 0.1572875967 | 0.5636754823 | 0.5729750061 | 1.092708156 |
| tnni3 | 0.08263622395 | 0.1469796732 | 0.562228927 | 0.5739600551 | 1.086146623 |
| eef1d | 0.08526318378 | 0.1521221614 | 0.5604915353 | 0.5751442117 | 1.089003637 |
| yju2 | 0.08720013325 | 0.1557511384 | 0.5598683526 | 0.5755692366 | 1.091115026 |
| chi3l1 | -0.084702084 | 0.1514339087 | -0.559333668 | 0.5759340218 | 0.918785965 |
| oga | 0.08693704406 | 0.1557073621 | 0.5583361179 | 0.576614886 | 1.090828003 |
| mprip | 0.08681488525 | 0.1555506724 | 0.558113211 | 0.5767670799 | 1.090694757 |
| cfc1 | 0.08333081641 | 0.1493345612 | 0.5580142716 | 0.5768346388 | 1.086901314 |
| marco | 0.08115068064 | 0.1455970767 | 0.5573647665 | 0.5772782331 | 1.084534303 |
| bst2 | 0.08492800176 | 0.1525809885 | 0.5566093297 | 0.577794378 | 1.088638684 |
| comt | -0.082679184 | 0.1485762951 | -0.556476282 | 0.5778853041 | 0.9206464577 |
| lrrc37a2 | -0.084944504 | 0.1526850204 | -0.556338162 | 0.5779797038 | 0.91856326 |
| csf1 | 0.08494760338 | 0.1528431639 | 0.5557828117 | 0.578359337 | 1.088660023 |
| vit | 0.09581850637 | 0.1724829973 | 0.555524358 | 0.5785360541 | 1.100559301 |
| mavs | 0.08053280175 | 0.1450745355 | 0.5551132833 | 0.5788171776 | 1.083864399 |
| tnfrsf21 | 0.08145507699 | 0.1468819078 | 0.5545616761 | 0.5791945085 | 1.084864481 |
| stambp | -0.080629543 | 0.1456532612 | -0.553571837 | 0.5798719047 | 0.9225353876 |
| upk3a | -0.085579101 | 0.1546320488 | -0.553437024 | 0.5799641923 | 0.9179805271 |
| tst | -0.08001406 | 0.144579266 | -0.553426936 | 0.5799710988 | 0.9231033673 |
| psme2 | 0.08187054007 | 0.1479356914 | 0.5534197955 | 0.5799759869 | 1.085315296 |
| tpsd1 | -0.086597785 | 0.1565605248 | -0.553126563 | 0.5801767489 | 0.9170458711 |
| edn1 | 0.08578851303 | 0.1551623154 | 0.5528952876 | 0.5803351144 | 1.089575873 |
| cdc27 | 0.07976326049 | 0.1443388427 | 0.5526111961 | 0.5805296738 | 1.083030641 |
| smndc1 | 0.08772656621 | 0.1589567545 | 0.5518895155 | 0.5810240521 | 1.091689576 |
| prkg1 | 0.08658450129 | 0.1569676719 | 0.551607221 | 0.581217488 | 1.090443508 |
| plekho1 | 0.08298203737 | 0.1509208284 | 0.549838205 | 0.5824303517 | 1.086522292 |
| vsig2 | 0.08685925021 | 0.157997296 | 0.5497514982 | 0.5824898295 | 1.090743147 |
| rln1 | 0.08947798033 | 0.1628730488 | 0.5493725389 | 0.5827498157 | 1.093603252 |
| ccl15 | 0.08361417588 | 0.1522139786 | 0.5493199552 | 0.5827858952 | 1.087209341 |
| cmc1 | 0.08343841149 | 0.1519475381 | 0.5491264453 | 0.5829186779 | 1.087018265 |
| trpv3 | -0.086536773 | 0.1584576583 | -0.546119223 | 0.5849839866 | 0.9171018233 |
| crnn | 0.08409094836 | 0.1544818289 | 0.5443420041 | 0.5862061472 | 1.087727816 |
| naprt | -0.085407649 | 0.1569382869 | -0.544211682 | 0.5862958139 | 0.91813793 |
| afap1 | -0.088748127 | 0.1630959335 | -0.544146779 | 0.586340472 | 0.9150760276 |
| bola1 | 0.08451808191 | 0.1554182312 | 0.5438106021 | 0.5865718123 | 1.088192521 |
| hyal1 | -0.077795225 | 0.1430646451 | -0.543776728 | 0.5865951254 | 0.9251538559 |

| Protein | beta | se | z | p_val | HR |
|---|---|---|---|---|---|
| rab27b | 0.08190648441 | 0.1506431745 | 0.5437118852 | 0.5866397524 | 1.085354308 |
| septin7 | -0.085220279 | 0.1567465003 | -0.543682182 | 0.5866601957 | 0.9183099774 |
| hjv | 0.08977585344 | 0.16521382 | 0.5433919113 | 0.5868599937 | 1.093929056 |
| ampd3 | 0.08286972645 | 0.152543091 | 0.543254538 | 0.5869545609 | 1.08640027 |
| cga | -0.119243579 | 0.2197376544 | -0.542663383 | 0.58736159 | 0.8875915759 |
| lsp1 | 0.08567931007 | 0.1579533111 | 0.5424344034 | 0.5875192849 | 1.089456894 |
| tmprss11b | 0.08261807756 | 0.1523186131 | 0.542403032 | 0.5875408914 | 1.086126913 |
| man2b2 | -0.084604903 | 0.1560113917 | -0.542299524 | 0.5876121837 | 0.9188752572 |
| f10 | -0.083559858 | 0.1542058995 | -0.541871994 | 0.5879066908 | 0.9198360252 |
| thtpa | 0.08304940201 | 0.1535696243 | 0.5407931575 | 0.5886501597 | 1.086595487 |
| ece1 | -0.079103969 | 0.1463047309 | -0.540679498 | 0.5887285118 | 0.9239438584 |
| il12rb1 | 0.07860967935 | 0.1453936225 | 0.5406680017 | 0.5887364375 | 1.081781998 |
| stip1 | -0.078605853 | 0.1454095142 | -0.540582598 | 0.5887953151 | 0.9244042039 |
| mif | -0.07808109 | 0.1445415296 | -0.540198308 | 0.5890602791 | 0.9248894244 |
| rtkn2 | 0.08589260109 | 0.1590107322 | 0.5401685779 | 0.5890807803 | 1.089689291 |
| ace | 0.08206526895 | 0.1519367382 | 0.5401278844 | 0.5891088418 | 1.085526659 |
| impa1 | -0.078784389 | 0.1458729961 | -0.540088924 | 0.5891357088 | 0.9242391787 |
| slc1a4 | -0.088021253 | 0.1630224716 | -0.539933252 | 0.5892430648 | 0.915741414 |
| epn1 | 0.08436540648 | 0.1562869504 | 0.5398109456 | 0.5893274178 | 1.088026393 |
| lonp1 | 0.08202308914 | 0.1519804075 | 0.5396951522 | 0.5894072837 | 1.085480872 |
| anxa4 | -0.076970371 | 0.1431808512 | -0.537574476 | 0.5908708551 | 0.9259172871 |
| gstm4 | 0.08380678817 | 0.1559616827 | 0.5373549883 | 0.5910224289 | 1.087418771 |
| impact | -0.081598482 | 0.151972454 | -0.536929426 | 0.5913163642 | 0.9216419398 |
| arf6 | 0.08331575832 | 0.155309302 | 0.5364505362 | 0.5916472127 | 1.086884947 |
| gcg | 0.07748188724 | 0.1447147841 | 0.5354109997 | 0.5923656856 | 1.08056266 |
| izumo1 | 0.08620395617 | 0.1610114324 | 0.5353902817 | 0.5923800088 | 1.090028624 |
| klk6 | 0.07742391487 | 0.1447407191 | 0.5349145377 | 0.5927089551 | 1.080500019 |
| nfe2 | 0.08123019031 | 0.1521294188 | 0.5339545166 | 0.5933730026 | 1.084620537 |
| nsfl1c | -0.078789128 | 0.1475768658 | -0.533885356 | 0.5934208543 | 0.9242347996 |
| areg | 0.08315334201 | 0.1559992234 | 0.5330368973 | 0.5940080391 | 1.086708434 |
| camkk1 | 0.07893164676 | 0.148143369 | 0.5328058035 | 0.5941680161 | 1.082130352 |
| coro6 | 0.08296543389 | 0.1559550641 | 0.5319829423 | 0.5947378097 | 1.086504252 |
| cd207 | 0.07622178265 | 0.1434883998 | 0.5312051898 | 0.595276597 | 1.079201896 |
| serpina5 | -0.085350321 | 0.1607855226 | -0.530833368 | 0.5955342547 | 0.918190567 |
| ghrl | -0.080423251 | 0.1515530309 | -0.530660787 | 0.5956538639 | 0.9227257194 |
| acp6 | -0.075197733 | 0.1418404715 | -0.530157097 | 0.5960030145 | 0.9275600593 |
| gatd3 | 0.08151481373 | 0.154000398 | 0.5293156043 | 0.5965865326 | 1.084929289 |
| nrp1 | -0.075770024 | 0.1431751501 | -0.529212115 | 0.5966583136 | 0.9270293766 |
| chchd10 | 0.09387053744 | 0.1774948426 | 0.5288634648 | 0.5969001681 | 1.098417533 |
| il3ra | -0.078475501 | 0.1483973535 | -0.528820085 | 0.5969302634 | 0.9245247095 |
| cd164l2 | -0.084407066 | 0.1597508008 | -0.528367093 | 0.5972445724 | 0.9190570628 |
| npl | 0.09129053695 | 0.1729415933 | 0.5278691796 | 0.5975901375 | 1.095587268 |
| ptprn2 | 0.07593375315 | 0.1439276361 | 0.5275828548 | 0.5977888955 | 1.078891099 |
| ddx58 | 0.07706455374 | 0.1462786781 | 0.526833813 | 0.5983089997 | 1.080111799 |
| procr | 0.08304102183 | 0.1577189997 | 0.5265124809 | 0.5985321826 | 1.086586381 |
| cd79b | 0.07642942879 | 0.1451765928 | 0.5264583452 | 0.5985697865 | 1.079426011 |
| ywhaq | 0.08242966139 | 0.1568111331 | 0.525662048 | 0.5991230373 | 1.085922288 |
| prnd | 0.08025882477 | 0.1527207841 | 0.5255265369 | 0.5992172107 | 1.083567485 |
| alpp | 0.08260207649 | 0.1573110635 | 0.5250875217 | 0.5995223501 | 1.086109534 |
| f3 | 0.07939055621 | 0.151230388 | 0.5249643095 | 0.599608002 | 1.082627066 |
| hbegf | 0.07570737919 | 0.1445063074 | 0.5239036313 | 0.600345569 | 1.078646893 |
| xiap | 0.08168466822 | 0.1561691428 | 0.5230525491 | 0.6009376851 | 1.085113585 |
| sez6l2 | -0.085201796 | 0.1631364334 | -0.522273255 | 0.6014800881 | 0.918326951 |
| eri1 | 0.08411673961 | 0.1611232681 | 0.5220645074 | 0.6016254175 | 1.087755871 |
| aldh5a1 | 0.08257198101 | 0.1582997857 | 0.5216177689 | 0.601936489 | 1.086076848 |
| wdr46 | 0.08021565257 | 0.153868326 | 0.5213266088 | 0.6021392678 | 1.083520707 |
| fgf6 | 0.07769197697 | 0.1495803089 | 0.5193997628 | 0.6034819967 | 1.080789699 |
| sirpb1 | 0.07523660893 | 0.1449133634 | 0.5191833739 | 0.603632872 | 1.078139218 |
| ripk4 | 0.08298491692 | 0.1599718132 | 0.5187471171 | 0.6039371 | 1.08652542 |
| defb4a_defb4b | 0.08137687039 | 0.1568899523 | 0.5186875844 | 0.6039786211 | 1.084779641 |
| krt6c | 0.08338314427 | 0.1611722868 | 0.5173541056 | 0.6049089909 | 1.086958191 |
| kir3dl2 | 0.08114172275 | 0.1570245012 | 0.516745617 | 0.6053337476 | 1.084524587 |
| clec2l | 0.0829668587 | 0.1607201625 | 0.5162193556 | 0.6057012132 | 1.0865058 |
| meltf | -0.079536238 | 0.1544269332 | -0.515041235 | 0.6065242056 | 0.9235445516 |
| eif4g3 | 0.07949866067 | 0.1544213052 | 0.5148166607 | 0.6066811419 | 1.082744109 |
| bpifb1 | 0.07824092851 | 0.1519861344 | 0.5147899103 | 0.6066998367 | 1.081383163 |
| treml2 | 0.07410651224 | 0.1440537145 | 0.5144366635 | 0.6069467315 | 1.076921505 |
| cep112 | 0.08232948028 | 0.1600895968 | 0.51427127 | 0.6070623453 | 1.085813505 |
| ism2 | 0.07858888968 | 0.1530933013 | 0.5133398328 | 0.6077136252 | 1.081759508 |
| erbb4 | 0.07647476507 | 0.1490986685 | 0.512913803 | 0.6080116176 | 1.07947495 |

| Protein | beta | se | z | p_val | HR |
|---|---|---|---|---|---|
| krt8 | -0.078237688 | 0.1525775932 | -0.51277312 | 0.6081100349 | 0.9247445993 |
| col15a1 | 0.08416773152 | 0.1646448274 | 0.51120787 | 0.6092055085 | 1.087811339 |
| dctpp1 | 0.07513553102 | 0.1470461438 | 0.5109656675 | 0.6093750975 | 1.078030248 |
| ikbkg | 0.07681952365 | 0.1505294359 | 0.5103289146 | 0.6098210489 | 1.079847172 |
| gad1 | -0.084050478 | 0.1650249984 | -0.509319669 | 0.6105281731 | 0.9193848466 |
| stk11 | -0.074227113 | 0.1461009226 | -0.508053691 | 0.6114156902 | 0.9284608044 |
| smpd1 | 0.07422687613 | 0.1463386431 | 0.5072267622 | 0.6119957193 | 1.077051135 |
| atxn3 | 0.07724362713 | 0.1527072765 | 0.5058280711 | 0.6129773505 | 1.080305236 |
| cd22 | 0.07348268018 | 0.1455898028 | 0.5047240863 | 0.6137526413 | 1.076249896 |
| fcamr | 0.08416931944 | 0.1667682301 | 0.5047083571 | 0.6137636905 | 1.087813066 |
| cela2a | 0.0774984507 | 0.1537254252 | 0.5041355431 | 0.6141661316 | 1.080580558 |
| micb_mica | 0.07414400878 | 0.1471851417 | 0.5037465598 | 0.6144394854 | 1.076961886 |
| il7 | -0.076352434 | 0.1517677629 | -0.503087298 | 0.614902897 | 0.9264896229 |
| lap3 | 0.07346414974 | 0.1463728059 | 0.5018975301 | 0.6157396028 | 1.076229953 |
| itih1 | 0.07624412308 | 0.1521960952 | 0.5009597846 | 0.6163994259 | 1.079226006 |
| abhd14b | 0.07409319507 | 0.1479419018 | 0.5008262986 | 0.6164933755 | 1.076907163 |
| il2rb | 0.07179168423 | 0.1433555131 | 0.5007947212 | 0.6165156011 | 1.0744315 |
| atp6ap2 | 0.07274338944 | 0.1453047841 | 0.5006262518 | 0.6166341834 | 1.075454529 |
| eif1ax | 0.07622057873 | 0.1522556272 | 0.5006092722 | 0.6166461356 | 1.079200597 |
| sprr1b | -0.078325865 | 0.156911921 | -0.4991709 | 0.6176589933 | 0.9246630623 |
| txlna | -0.072668077 | 0.1460786839 | -0.497458458 | 0.6188657909 | 0.9299094373 |
| tp53inp1 | 0.07206926482 | 0.1449446926 | 0.497219067 | 0.6190345771 | 1.074729782 |
| ggt5 | 0.07183956689 | 0.1444897021 | 0.4971950654 | 0.6190515009 | 1.074482948 |
| med18 | 0.07153371179 | 0.1440509431 | 0.4965862094 | 0.61948088 | 1.074154362 |
| akr1c4 | 0.07246195134 | 0.1460091922 | 0.496283489 | 0.6196944137 | 1.075151897 |
| spesp1 | 0.08138737446 | 0.1641129317 | 0.4959229819 | 0.6199487509 | 1.084791036 |
| mindy1 | 0.07522000451 | 0.1517233674 | 0.4957707292 | 0.6200561786 | 1.078121316 |
| s100a14 | -0.076066202 | 0.1534639749 | -0.495661617 | 0.620133172 | 0.9267548516 |
| arid4b | -0.072521784 | 0.1463960791 | -0.495380643 | 0.6203314556 | 0.9300454865 |
| atp6v1g1 | -0.076193495 | 0.1540800129 | -0.494506024 | 0.6209488527 | 0.92663689 |
| tcn2 | 0.06912874969 | 0.1405421548 | 0.4918719923 | 0.6228098389 | 1.071574165 |
| dctn1 | 0.07017307998 | 0.1426923575 | 0.4917788255 | 0.622875707 | 1.072693827 |
| fcer2 | 0.0714890985 | 0.1453722308 | 0.491765849 | 0.6228848815 | 1.074106441 |
| lztfl1 | 0.07810856794 | 0.1589437054 | 0.4914228452 | 0.6231274096 | 1.08124004 |
| itga2 | -0.080217203 | 0.1633425104 | -0.491098142 | 0.6233570357 | 0.9229158642 |
| cd160 | 0.06948103658 | 0.1416086823 | 0.4906552017 | 0.6236703364 | 1.071951733 |
| ccl26 | -0.074383685 | 0.1517034585 | -0.490322933 | 0.6239054014 | 0.928315445 |
| hmox2 | 0.07394526682 | 0.1508894467 | 0.4900625486 | 0.6240896386 | 1.07674787 |
| smad1 | 0.07134097748 | 0.1455951497 | 0.489995564 | 0.6241370378 | 1.073947355 |
| reg3g | 0.07528584987 | 0.1536771002 | 0.4898963462 | 0.6242072487 | 1.078192308 |
| cert | 0.07075160887 | 0.1447998857 | 0.4886164691 | 0.6251132511 | 1.073314591 |
| myl6b | -0.07806796 | 0.1597777358 | -0.488603495 | 0.6251224382 | 0.924901568 |
| klrf1 | 0.07910808221 | 0.1619330606 | 0.4885233559 | 0.6251791864 | 1.082321295 |
| pard3 | 0.07647131468 | 0.1565816031 | 0.4883799448 | 0.6252807446 | 1.079471225 |
| uxs1 | -0.070954388 | 0.1456143028 | -0.487276224 | 0.6260625945 | 0.9315043793 |
| pbld | 0.0736192629 | 0.1511875363 | 0.4869400262 | 0.6263008327 | 1.076396903 |
| zbtb16 | 0.07053208353 | 0.1449747098 | 0.4865130175 | 0.6266034781 | 1.073078997 |
| casp7 | 0.07394761856 | 0.1520866993 | 0.4862201554 | 0.6268110825 | 1.076750402 |
| gage2a | -0.078756199 | 0.1622130408 | -0.485510896 | 0.6273139853 | 0.9242652339 |
| cd163 | 0.07225994805 | 0.1488895075 | 0.4853259929 | 0.6274451202 | 1.074934735 |
| ppp1r9b | 0.06910722847 | 0.1424141857 | 0.4852552303 | 0.6274953087 | 1.071551104 |
| gapdh | 0.07528825191 | 0.1553122161 | 0.4847542184 | 0.6278507015 | 1.078194898 |
| acvrl1 | 0.07559389201 | 0.1560483358 | 0.4844261339 | 0.628083475 | 1.078524488 |
| birc2 | 0.06990715777 | 0.144442676 | 0.4839785563 | 0.6284010877 | 1.072408612 |
| caps | 0.07945439516 | 0.1649683814 | 0.4816340833 | 0.6300659096 | 1.082696182 |
| comp | -0.072880667 | 0.1514280517 | -0.481289075 | 0.6303110603 | 0.9297117687 |
| rbm17 | 0.07391177916 | 0.1541236096 | 0.4795616929 | 0.6315390906 | 1.076711813 |
| tnfrsf4 | 0.07040251119 | 0.1468415086 | 0.4794455726 | 0.6316216795 | 1.072939965 |
| ehbp1 | 0.07318826337 | 0.1526644239 | 0.4794061477 | 0.6316497208 | 1.075933077 |
| fadd | -0.068531687 | 0.1432506662 | -0.478403965 | 0.6323627123 | 0.9337638718 |
| pspn | 0.1000083251 | 0.2093355823 | 0.4777416435 | 0.6328341008 | 1.105180119 |
| nagpa | -0.07664238 | 0.1606603307 | -0.477046075 | 0.6333293127 | 0.9262210294 |
| elavl4 | 0.07562732278 | 0.1585466877 | 0.4770034864 | 0.633359639 | 1.078560544 |
| ctbs | -0.075712238 | 0.1588798223 | -0.476537781 | 0.6336912978 | 0.9270829475 |
| acaa1 | 0.07125880084 | 0.1496575629 | 0.4761456721 | 0.633970601 | 1.073859106 |
| cxcl3 | 0.06808583286 | 0.1430351252 | 0.4760077832 | 0.634068833 | 1.070457185 |
| rab6b | 0.06887279829 | 0.1447546053 | 0.4757900319 | 0.6342239721 | 1.071299929 |
| snx2 | 0.07197868098 | 0.1513508944 | 0.4755748638 | 0.6343772866 | 1.074632434 |
| arsb | 0.06996388933 | 0.1471290424 | 0.4755273887 | 0.6344111163 | 1.072469453 |
| pomc | -0.075933227 | 0.1600224486 | -0.47451609 | 0.6351319272 | 0.9268780953 |

| Protein | beta | se | z | p_val | HR |
|---|---|---|---|---|---|
| trim26 | 0.0739471613 | 0.1559094937 | 0.4742954362 | 0.6352892455 | 1.07674991 |
| cd3g | 0.07340583678 | 0.1547690337 | 0.4742927899 | 0.6352911324 | 1.076167197 |
| vegfb | -0.078231372 | 0.1650411376 | -0.47401135 | 0.6354918133 | 0.92475044 |
| plin3 | 0.06989429451 | 0.147473688 | 0.473944169 | 0.635539721 | 1.072394817 |
| phactr2 | 0.07349909815 | 0.1551775661 | 0.473645128 | 0.6357529888 | 1.076267566 |
| fcrl5 | 0.07147566215 | 0.151102345 | 0.4730281462 | 0.6361930987 | 1.074092009 |
| dscam | -0.073714677 | 0.1558742112 | -0.47291131 | 0.6362764555 | 0.9289367026 |
| fbn2 | 0.07562300741 | 0.159943344 | 0.4728112189 | 0.6363478693 | 1.07855589 |
| il2 | -0.067609394 | 0.1431498948 | -0.472297898 | 0.6367141708 | 0.9346254723 |
| pdcd1lg2 | 0.06868350568 | 0.1455643673 | 0.4718428484 | 0.6370389641 | 1.071097159 |
| csnk1d | -0.073732572 | 0.1564824191 | -0.471187578 | 0.6375067889 | 0.9289200799 |
| cd93 | -0.067979552 | 0.144285843 | -0.471144991 | 0.6375371986 | 0.9342795774 |
| mfap5 | 0.07447705488 | 0.1582222318 | 0.4707116947 | 0.6378466319 | 1.077320624 |
| hbq1 | -0.06825609 | 0.1458985262 | -0.467832619 | 0.6399042919 | 0.9340212498 |
| prtfdc1 | -0.067566279 | 0.1445291901 | -0.467492268 | 0.6401477225 | 0.9346657699 |
| ifnl1 | 0.068408657 | 0.1463456928 | 0.4674456468 | 0.6401810708 | 1.07080281 |
| metap2 | -0.067169584 | 0.1437046017 | -0.467414285 | 0.6402035041 | 0.9350366209 |
| ceacam8 | 0.07063343815 | 0.1511842853 | 0.4672009263 | 0.640356131 | 1.073187764 |
| cbln4 | -0.066090583 | 0.1416429867 | -0.466599756 | 0.6407862618 | 0.9360460706 |
| anxa3 | -0.067365051 | 0.1444917016 | -0.466220901 | 0.6410573903 | 0.9348538695 |
| myl1 | 0.07542480711 | 0.1617796425 | 0.4662193955 | 0.6410584676 | 1.078342141 |
| ptges2 | 0.07367581332 | 0.1584206758 | 0.4650643796 | 0.6418853536 | 1.076457776 |
| prtg | -0.067561966 | 0.1453082334 | -0.464956217 | 0.6419628112 | 0.9346698007 |
| psmc3 | -0.074321978 | 0.1602456242 | -0.463800362 | 0.6427907842 | 0.9283727297 |
| reg1a | -0.068269408 | 0.1473293451 | -0.463379566 | 0.6430923226 | 0.9340088102 |
| mmp9 | 0.0698390289 | 0.1507662084 | 0.4632273347 | 0.643201425 | 1.072335552 |
| cpm | 0.0766103696 | 0.1660450634 | 0.4613830006 | 0.6445238456 | 1.079621341 |
| itgb7 | -0.06700135 | 0.145254479 | -0.461268737 | 0.6446058115 | 0.9351939387 |
| znf75d | -0.074117075 | 0.1607083951 | -0.461189817 | 0.6446624267 | 0.9285629756 |
| nt5c1a | 0.08300500352 | 0.1799864507 | 0.4611736229 | 0.6446740443 | 1.086547245 |
| kir2ds4 | 0.07033445115 | 0.1525441109 | 0.4610761487 | 0.6447439732 | 1.072866943 |
| apol1 | -0.072454732 | 0.1572657414 | -0.460715292 | 0.645002882 | 0.9301078498 |
| ank2 | -0.073103648 | 0.1588264332 | -0.46027381 | 0.6453196975 | 0.9295044842 |
| igf2bp3 | -0.073994528 | 0.1608099301 | -0.460136561 | 0.6454182031 | 0.9286767754 |
| zcchc8 | 0.07346092397 | 0.1596823716 | 0.4600440438 | 0.645484607 | 1.076226481 |
| ctsc | 0.06696436997 | 0.1457248902 | 0.4595259592 | 0.6458565149 | 1.06925738 |
| il32 | -0.069958096 | 0.1522549151 | -0.459480049 | 0.6458894755 | 0.9324328918 |
| timm10 | 0.0701174707 | 0.1526485607 | 0.4593392194 | 0.645990588 | 1.072634177 |
| gpr101 | 0.06952012367 | 0.1514528436 | 0.4590215808 | 0.6462186688 | 1.071993633 |
| podxl | -0.066154917 | 0.1441291095 | -0.458997612 | 0.6462358811 | 0.9359858529 |
| ism1 | -0.071285766 | 0.1553247943 | -0.458946469 | 0.646272608 | 0.9311957502 |
| ranbp1 | -0.069243739 | 0.1513468254 | -0.457516959 | 0.6472995152 | 0.9330992194 |
| tpt1 | 0.06535044447 | 0.143023778 | 0.4569201386 | 0.6477284484 | 1.06753307 |
| palm | 0.07837293529 | 0.1716643013 | 0.4565476613 | 0.6479962059 | 1.081525923 |
| mtus1 | -0.074282715 | 0.1632635433 | -0.454986542 | 0.6491189207 | 0.9284091815 |
| aamdc | -0.069596146 | 0.1529989774 | -0.45487981 | 0.649195709 | 0.9327704472 |
| prdx2 | -0.072588411 | 0.1595880205 | -0.454848748 | 0.6492180569 | 0.929983522 |
| rgma | -0.06556342 | 0.1442785651 | -0.454422454 | 0.6495247943 | 0.93653965 |
| ap3b1 | 0.0699473731 | 0.1540727067 | 0.4539893832 | 0.6498364683 | 1.07245174 |
| tmsb10 | 0.0685023036 | 0.1510682493 | 0.4534526872 | 0.6502228049 | 1.070903092 |
| sumf2 | -0.066240064 | 0.1462301471 | -0.452985004 | 0.6505595397 | 0.9359061601 |
| cyb5r2 | 0.0734051796 | 0.162247423 | 0.452427399 | 0.6509611123 | 1.076166489 |
| cant1 | -0.066571092 | 0.1471649882 | -0.452356863 | 0.6510119175 | 0.9355963997 |
| rspo3 | 0.07030801674 | 0.1558901504 | 0.4510100002 | 0.6519823416 | 1.072838583 |
| snx5 | -0.069518249 | 0.1541726086 | -0.450911804 | 0.6520531153 | 0.9328431096 |
| cdh15 | -0.075902851 | 0.168618801 | -0.450144647 | 0.652606146 | 0.9269062506 |
| epcam | 0.06422514885 | 0.1428013031 | 0.4497518401 | 0.6528893872 | 1.066332455 |
| scly | 0.07019486944 | 0.1563088136 | 0.4490781283 | 0.6533752976 | 1.072717201 |
| slc13a1 | 0.06831215727 | 0.1523980845 | 0.4482481357 | 0.6539741266 | 1.070699483 |
| nedd4l | -0.069922582 | 0.1563596224 | -0.447190781 | 0.654737317 | 0.9324660071 |
| dcc | -0.07174293 | 0.1608176066 | -0.446113655 | 0.6555151494 | 0.9307701378 |
| alpi | -0.069311869 | 0.1554430705 | -0.445898735 | 0.6556703958 | 0.9330356502 |
| kir3dl1 | -0.062012985 | 0.1391673601 | -0.445600065 | 0.6558861638 | 0.9398706828 |
| sh2d1a | 0.06825848723 | 0.1533538706 | 0.4451044303 | 0.6562442873 | 1.07064202 |
| podxl2 | 0.06457912421 | 0.145257767 | 0.4445829338 | 0.6566211829 | 1.066709977 |
| tp73 | -0.069445894 | 0.1562065145 | -0.44457745 | 0.656625147 | 0.9329106082 |
| setmar | 0.07023844775 | 0.1580205086 | 0.4444894423 | 0.6566887603 | 1.072763949 |
| appl2 | 0.06919568505 | 0.1560807615 | 0.4433325695 | 0.6575252012 | 1.071645894 |
| spink2 | 0.0766730454 | 0.1730141086 | 0.4431606534 | 0.6576495364 | 1.079689009 |
| lrtm1 | -0.071353596 | 0.1611324183 | -0.442825824 | 0.657891723 | 0.9311325892 |

| Protein | beta | se | z | p_val | HR |
|---|---|---|---|---|---|
| sftpa1 | 0.06531844233 | 0.1475994252 | 0.4425385957 | 0.6580995077 | 1.067498907 |
| ppp1r2 | -0.063550495 | 0.1437704168 | -0.442027619 | 0.65846922 | 0.9384267322 |
| klk3 | 0.1037182563 | 0.2350093633 | 0.4413366976 | 0.6589692626 | 1.109287876 |
| smad5 | -0.066640203 | 0.1510254067 | -0.441251604 | 0.6590308581 | 0.9355317423 |
| notch2 | -0.069927693 | 0.158592124 | -0.440927908 | 0.6592651891 | 0.9324612405 |
| adh4 | 0.07301440867 | 0.1656174604 | 0.4408617818 | 0.6593130632 | 1.075746037 |
| pdia5 | 0.07164292443 | 0.1631169364 | 0.4392120524 | 0.6605078928 | 1.074271679 |
| aspn | -0.068563191 | 0.1561420686 | -0.439107743 | 0.6605834686 | 0.9337344543 |
| thpo | -0.063893021 | 0.1456177887 | -0.438772087 | 0.6608266874 | 0.9381053516 |
| icam4 | 0.06653926413 | 0.1518497238 | 0.4381915388 | 0.6612474416 | 1.068802929 |
| crtap | -0.070681876 | 0.1613076078 | -0.438180673 | 0.661255318 | 0.9317582596 |
| arhgef1 | 0.06604019583 | 0.1509287451 | 0.4375587685 | 0.6617061657 | 1.068269656 |
| pms1 | 0.06858793541 | 0.1567846434 | 0.4374659017 | 0.6617734998 | 1.070994799 |
| pdia4 | 0.06784918278 | 0.155317563 | 0.4368416647 | 0.6622261804 | 1.070203891 |
| skap2 | 0.06246042885 | 0.1430069188 | 0.4367650837 | 0.6622817235 | 1.064452336 |
| fnta | -0.070439183 | 0.1613355583 | -0.436600484 | 0.6624011115 | 0.9319844184 |
| psmd9 | -0.064388323 | 0.1475398834 | -0.436412996 | 0.6625371113 | 0.9376408217 |
| ensa | -0.067352827 | 0.1544313387 | -0.43613445 | 0.6627391834 | 0.9348652974 |
| gne | 0.06218784345 | 0.1427991583 | 0.4354916667 | 0.6632055865 | 1.064162222 |
| sdhb | -0.067677968 | 0.1555177255 | -0.435178483 | 0.6634328792 | 0.9345613839 |
| cd226 | 0.06794070802 | 0.1562357676 | 0.4348601415 | 0.6636639477 | 1.070301846 |
| tnfaip8 | 0.06284259818 | 0.1447772392 | 0.4340640734 | 0.6642419134 | 1.064859215 |
| ifnar1 | 0.06745666896 | 0.1554878559 | 0.4338388265 | 0.6644054847 | 1.069783904 |
| sbsn | 0.06597041288 | 0.1521722939 | 0.4335244689 | 0.6646337937 | 1.068195112 |
| paxx | 0.07146572847 | 0.1649495307 | 0.4332581498 | 0.6648272381 | 1.07408134 |
| gnpda1 | -0.065965437 | 0.1525887251 | -0.432308725 | 0.6655170465 | 0.9361632201 |
| cfb | 0.07272482114 | 0.1683377736 | 0.4320172449 | 0.6657288796 | 1.075434559 |
| il13ra1 | 0.06531799389 | 0.1511997562 | 0.4319980106 | 0.665742859 | 1.067498428 |
| map2 | 0.06608355355 | 0.1529974081 | 0.4319259677 | 0.6657952207 | 1.068315975 |
| treml1 | 0.06627600831 | 0.153466706 | 0.4318591964 | 0.6658437524 | 1.068521597 |
| notch3 | -0.065577394 | 0.1520565602 | -0.431269744 | 0.6662722474 | 0.9365265627 |
| bcat1 | -0.069123641 | 0.1604225288 | -0.430884876 | 0.6665520807 | 0.9332112893 |
| tap1 | -0.067472626 | 0.1567542895 | -0.430435596 | 0.6668788062 | 0.9347533081 |
| mfap3 | -0.062527145 | 0.145338269 | -0.430218038 | 0.6670370419 | 0.9393875629 |
| exosc10 | 0.06787081498 | 0.1578432347 | 0.4299887486 | 0.6672038258 | 1.070227042 |
| akr7l | -0.070226352 | 0.1637085299 | -0.428971855 | 0.6679437072 | 0.9321827949 |
| spint1 | 0.06143181743 | 0.1432772932 | 0.4287617114 | 0.6680966456 | 1.063357992 |
| slitrk2 | 0.06302846624 | 0.1471114214 | 0.428440332 | 0.6683305662 | 1.065057157 |
| mocs2 | 0.06871322695 | 0.1609071568 | 0.4270364869 | 0.6693527522 | 1.071128994 |
| dusp29 | -0.071982549 | 0.1685739511 | -0.427008733 | 0.6693729666 | 0.9305471343 |
| il1b | -0.061571276 | 0.1442152257 | -0.426940193 | 0.6694228895 | 0.9402859233 |
| cdh23 | 0.06588838817 | 0.1543603564 | 0.4268478623 | 0.6694901427 | 1.068107497 |
| tnfsf10 | -0.062292815 | 0.1460119376 | -0.426628236 | 0.6696501281 | 0.9396077149 |
| gas6 | 0.06354891507 | 0.1489979806 | 0.4265085661 | 0.6697373074 | 1.065611609 |
| nudc | 0.06137519703 | 0.144331173 | 0.4252386769 | 0.670662692 | 1.063297786 |
| akr1b1 | 0.06158502616 | 0.1450270361 | 0.4246451408 | 0.6710953808 | 1.06352092 |
| clstn3 | -0.065460462 | 0.1542884772 | -0.424273172 | 0.6713666023 | 0.9366360791 |
| ptp4a3 | 0.06913807479 | 0.163285166 | 0.4234192025 | 0.6719894371 | 1.071584158 |
| ly75 | -0.0635829 | 0.150173245 | -0.423396987 | 0.6720056425 | 0.9383963235 |
| crisp3 | -0.065705538 | 0.155692925 | -0.422020062 | 0.6730103741 | 0.93640656 |
| scgb1a1 | -0.06167719 | 0.1461825094 | -0.421919084 | 0.6730840802 | 0.940186339 |
| tnfaip8l2 | 0.06706864414 | 0.15896175 | 0.4219168708 | 0.6730856954 | 1.069368882 |
| hmbs | -0.062027098 | 0.1471708323 | -0.421463252 | 0.67341684 | 0.9398574186 |
| slc28a1 | 0.06839735052 | 0.1623383438 | 0.4213259106 | 0.6735171123 | 1.070790703 |
| ncr3lg1 | -0.067376326 | 0.1600105242 | -0.421074344 | 0.6737007956 | 0.9348433289 |
| rest | 0.06748414396 | 0.1602988218 | 0.4209896443 | 0.6737626438 | 1.069813296 |
| nop56 | 0.06668484854 | 0.158905194 | 0.4196517866 | 0.674739851 | 1.068958541 |
| mpig6b | 0.0594545611 | 0.1417051453 | 0.419565295 | 0.6748030457 | 1.061257538 |
| mep1b | -0.05979698 | 0.1425283683 | -0.419544409 | 0.6748183064 | 0.94195575 |
| fcrlb | 0.06273200713 | 0.1498410733 | 0.4186569525 | 0.6754668604 | 1.064741458 |
| daam1 | 0.0648766256 | 0.1551254164 | 0.4182204768 | 0.6757859257 | 1.067027372 |
| lactb2 | -0.059923029 | 0.143560817 | -0.417405184 | 0.6763820634 | 0.9418370247 |
| igfbp3 | 0.06038119079 | 0.144892365 | 0.4167313493 | 0.6768749214 | 1.062241386 |
| dnajc9 | 0.06472192643 | 0.1553140035 | 0.416716619 | 0.676885697 | 1.066862317 |
| rras | 0.06619308571 | 0.1593278842 | 0.4154519848 | 0.6778110578 | 1.068432996 |
| ambn | 0.05916986508 | 0.1425464358 | 0.4150918593 | 0.6780746586 | 1.060955445 |
| sec31a | 0.06348968986 | 0.1531883653 | 0.4144550385 | 0.6785408883 | 1.0655485 |
| padi2 | 0.06086254862 | 0.1471704377 | 0.4135514549 | 0.6792026318 | 1.062752827 |
| sh3bp1 | 0.0643781088 | 0.155694994 | 0.4134886237 | 0.6792486557 | 1.066495574 |
| safb2 | 0.06385095991 | 0.1544951658 | 0.413287753 | 0.6793958016 | 1.06593352 |

| Protein | beta | se | z | p_val | HR |
|---|---|---|---|---|---|
| csf1r | 0.06545677664 | 0.158428756 | 0.4131622207 | 0.6794877654 | 1.067646589 |
| lcat | 0.06823745654 | 0.1654769911 | 0.4123682458 | 0.6800695341 | 1.070619504 |
| siglec10 | 0.06369949047 | 0.1544764971 | 0.4123571656 | 0.6800776542 | 1.065772076 |
| uros | 0.06570584399 | 0.1593558099 | 0.4123216093 | 0.6801037119 | 1.067912538 |
| ube2l6 | 0.0635474443 | 0.1541683303 | 0.4121951907 | 0.6801963619 | 1.065610042 |
| lcp1 | 0.06425518007 | 0.1559053696 | 0.4121421875 | 0.6802352085 | 1.066364479 |
| pde5a | 0.06405443033 | 0.1556966207 | 0.4114053988 | 0.6807752958 | 1.066150428 |
| prss53 | -0.063366464 | 0.1540697902 | -0.411284157 | 0.680864185 | 0.9385994479 |
| ctsz | 0.06088037612 | 0.1482534805 | 0.410650569 | 0.6813287776 | 1.062771774 |
| ube2b | 0.06335413018 | 0.1544445177 | 0.4102064036 | 0.6816545438 | 1.065404064 |
| thbs4 | -0.062103409 | 0.1514926349 | -0.409943423 | 0.6818474507 | 0.9397856991 |
| scarf1 | 0.05951230611 | 0.1452329425 | 0.4097713997 | 0.6819736482 | 1.061318822 |
| ppp2r5a | 0.06314001941 | 0.1541875223 | 0.4095014853 | 0.682171677 | 1.065175974 |
| ccdc134 | 0.06322856234 | 0.1544283442 | 0.4094362512 | 0.6822195407 | 1.065270292 |
| azi2 | 0.06292835732 | 0.1537044671 | 0.4094113756 | 0.6822377929 | 1.064950541 |
| ddah1 | 0.0613332195 | 0.1498334629 | 0.4093426017 | 0.6822882558 | 1.063253152 |
| serpinb9 | -0.058855523 | 0.1438672764 | -0.40909597 | 0.6824692337 | 0.9428429785 |
| inpp1 | 0.06071812825 | 0.1485712529 | 0.4086801925 | 0.6827743716 | 1.062599355 |
| fbln2 | -0.065714552 | 0.1615838243 | -0.406690162 | 0.684235567 | 0.9363981194 |
| calcb | 0.06370891251 | 0.1571400181 | 0.405427677 | 0.6851631702 | 1.065782118 |
| ctrc | -0.057417382 | 0.1416525767 | -0.405339479 | 0.6852279912 | 0.9441998955 |
| pebp1 | 0.06038537793 | 0.1491498675 | 0.4048637718 | 0.6855776495 | 1.062245834 |
| plau | 0.06008802799 | 0.148437786 | 0.4048027771 | 0.6856224873 | 1.061930022 |
| ccl13 | 0.05838020822 | 0.1445279785 | 0.4039370704 | 0.6862589955 | 1.060117985 |
| inpp5d | 0.06209817853 | 0.1543997528 | 0.4021909194 | 0.6875435246 | 1.064066808 |
| cd248 | -0.064281343 | 0.1601399918 | -0.401407182 | 0.6881203627 | 0.9377411357 |
| igsf3 | 0.06415543741 | 0.1600040361 | 0.4009613694 | 0.6884485657 | 1.066258122 |
| tax1bp1 | 0.06067639273 | 0.1516966894 | 0.3999849501 | 0.6891676017 | 1.062555008 |
| zpr1 | -0.061096896 | 0.1528471767 | -0.399725381 | 0.6893587955 | 0.9407320822 |
| arsa | 0.05935503293 | 0.1486049958 | 0.3994147883 | 0.6895875992 | 1.061151918 |
| psca | 0.06027516395 | 0.1509522946 | 0.3992994218 | 0.6896725931 | 1.062128766 |
| tank | 0.05738000174 | 0.1437294273 | 0.3992223641 | 0.689729366 | 1.059058178 |
| arhgef10 | -0.061009736 | 0.1528852082 | -0.399055848 | 0.6898520537 | 0.9408140795 |
| srpk2 | 0.06001223012 | 0.1506513568 | 0.3983517401 | 0.6903709264 | 1.061849533 |
| fkbp7 | -0.058534243 | 0.1469462316 | -0.398337832 | 0.6903811766 | 0.9431459433 |
| gja8 | 0.06422035475 | 0.1613174935 | 0.3980991359 | 0.6905571113 | 1.066327343 |
| cyb5a | -0.063764719 | 0.1603104577 | -0.397757704 | 0.6908087979 | 0.9382257198 |
| unc5d | 0.06234487867 | 0.1568217668 | 0.3975524568 | 0.6909601118 | 1.064329346 |
| tymp | 0.05745641994 | 0.1449814962 | 0.396301745 | 0.6918824401 | 1.059139112 |
| mn1 | 0.06374264632 | 0.1608458248 | 0.3962965554 | 0.691886268 | 1.065818071 |
| brsk2 | 0.06157689307 | 0.1553997736 | 0.3962482805 | 0.6919218773 | 1.06351227 |
| pak4 | -0.057059228 | 0.1441229579 | -0.395906586 | 0.6921739426 | 0.9445381245 |
| axin1 | 0.05932307883 | 0.1500952102 | 0.3952363219 | 0.6926684892 | 1.06111801 |
| prame | -0.063252947 | 0.1600655398 | -0.395169047 | 0.6927181347 | 0.938706001 |
| rarres2 | 0.06366241182 | 0.1611027662 | 0.3951664723 | 0.6927200345 | 1.065732559 |
| lect2 | 0.06999491619 | 0.1772969545 | 0.3947891625 | 0.6929984937 | 1.072502729 |
| fgfbp2 | -0.061945464 | 0.1569640482 | -0.394647464 | 0.6931030794 | 0.939934146 |
| pear1 | 0.05636947743 | 0.1432508508 | 0.3935018683 | 0.6939488444 | 1.057988514 |
| nfasc | -0.059948124 | 0.1525309065 | -0.3930228 | 0.6943026416 | 0.9418133898 |
| sct | 0.06085719615 | 0.1548688931 | 0.392959457 | 0.6943494262 | 1.062747139 |
| diablo | 0.05542595397 | 0.1410519122 | 0.3929472001 | 0.6943584791 | 1.056990748 |
| ocln | 0.06680065469 | 0.170364759 | 0.3921037138 | 0.6949815836 | 1.06908234 |
| naa80 | 0.06315519455 | 0.1611187105 | 0.3919792701 | 0.6950735307 | 1.065192138 |
| clec4c | -0.058826929 | 0.1506392299 | -0.390515331 | 0.6961555207 | 0.9428699387 |
| sms | 0.05841010102 | 0.1496075189 | 0.3904222292 | 0.696224353 | 1.060149675 |
| pilrb | -0.054879278 | 0.140599587 | -0.390323179 | 0.6962975858 | 0.9465994167 |
| cd28 | 0.0578253684 | 0.148215191 | 0.3901446808 | 0.6964295649 | 1.059529952 |
| csde1 | -0.062490994 | 0.1606381716 | -0.389017088 | 0.6972635057 | 0.9394215234 |
| cdc25a | -0.063839909 | 0.1644505032 | -0.388201363 | 0.6978670248 | 0.9381551773 |
| psrc1 | -0.055941379 | 0.1441058988 | -0.388196314 | 0.6978707609 | 0.9455945662 |
| ctso | 0.05756809074 | 0.1483887895 | 0.3879544468 | 0.6980497447 | 1.059257394 |
| adh1b | 0.06782414294 | 0.1748459358 | 0.3879080325 | 0.6980840936 | 1.070177094 |
| rrm2b | 0.05642923064 | 0.1455835285 | 0.3876072467 | 0.6983067053 | 1.058051735 |
| tex33 | 0.06155657271 | 0.1588120007 | 0.3876065564 | 0.6983072162 | 1.063490659 |
| hexim1 | 0.05880634312 | 0.1517714665 | 0.3874663958 | 0.698410958 | 1.060569834 |
| il10ra | 0.05670908208 | 0.1464621567 | 0.3871927286 | 0.6986135329 | 1.058347873 |
| chrm1 | -0.061857458 | 0.1597876924 | -0.387122793 | 0.6986653044 | 0.9400168694 |
| ofd1 | -0.061818482 | 0.1597142038 | -0.387056882 | 0.6987140978 | 0.9400535082 |
| pon3 | -0.063604893 | 0.1643793597 | -0.386939657 | 0.6988008813 | 0.938375685 |
| lpo | 0.05827817505 | 0.1507172829 | 0.3866721449 | 0.6989989403 | 1.060009823 |

| Protein | beta | se | z | p_val | HR |
|---|---|---|---|---|---|
| kdr | -0.056963646 | 0.1476657305 | -0.385760773 | 0.6996738491 | 0.9446284094 |
| il1rap | -0.056537372 | 0.1468235372 | -0.385070224 | 0.7001853883 | 0.9450311658 |
| chek2 | 0.0571961619 | 0.1486082955 | 0.3848786618 | 0.7003273158 | 1.058863499 |
| ryr1 | 0.06033235201 | 0.1567708232 | 0.3848442636 | 0.7003528024 | 1.062189509 |
| slit2 | 0.05669381645 | 0.1473233916 | 0.3848256265 | 0.7003666114 | 1.058331717 |
| ahsp | 0.05830142333 | 0.1516818025 | 0.3843666306 | 0.7007067294 | 1.060034467 |
| jpt2 | -0.059238071 | 0.1541893782 | -0.384190351 | 0.7008373691 | 0.9424823644 |
| serpine1 | 0.05588058842 | 0.1456061052 | 0.3837791578 | 0.701142137 | 1.057471402 |
| vegfd | -0.056778969 | 0.1480768767 | -0.383442508 | 0.7013916905 | 0.944802877 |
| adipoq | -0.069152231 | 0.1804687159 | -0.383181264 | 0.7015853692 | 0.93318461 |
| grin2b | 0.05900856239 | 0.1541181024 | 0.3828788538 | 0.7018095911 | 1.060784324 |
| nfkbie | 0.05570928317 | 0.1455683413 | 0.3827019162 | 0.7019407935 | 1.057290267 |
| eif2s2 | 0.05903902007 | 0.1543866378 | 0.3824101679 | 0.7021571496 | 1.060816633 |
| b3gnt7 | -0.061622492 | 0.1612076803 | -0.382255313 | 0.7022719973 | 0.9402377668 |
| cebpb | -0.056630315 | 0.1482859579 | -0.381899375 | 0.7025360041 | 0.9449433366 |
| dcun1d2 | -0.060906091 | 0.159666723 | -0.381457635 | 0.7028637011 | 0.9409115961 |
| cilp | 0.06130782668 | 0.1607692036 | 0.3813406133 | 0.7029505212 | 1.063226153 |
| pde4d | -0.062271612 | 0.1633491786 | -0.381217786 | 0.7030416522 | 0.9396276377 |
| nfatc1 | 0.05761332354 | 0.1511920368 | 0.3810605688 | 0.7031583054 | 1.059305308 |
| dnaja2 | 0.05483248258 | 0.1440746791 | 0.3805837565 | 0.7035121363 | 1.056363641 |
| igdcc3 | -0.057761612 | 0.1517986057 | -0.380514775 | 0.7035633309 | 0.9438749288 |
| osm | -0.060115544 | 0.158345342 | -0.379648327 | 0.7042064824 | 0.9416557245 |
| mapkapk2 | -0.058531535 | 0.1542093336 | -0.379558964 | 0.7042728272 | 0.9431484979 |
| cd300a | -0.060790247 | 0.1602714278 | -0.379295597 | 0.7044683691 | 0.9410206011 |
| mri1 | -0.060919477 | 0.1606178594 | -0.379282088 | 0.7044783993 | 0.9408990006 |
| cd4 | -0.058693599 | 0.1550832829 | -0.37846503 | 0.7050851672 | 0.9429956594 |
| ssna1 | 0.05838918786 | 0.1550302687 | 0.3766308887 | 0.7064479295 | 1.060127504 |
| ythdf3 | -0.054750574 | 0.1455241003 | -0.376230284 | 0.7067457029 | 0.9467212557 |
| pgf | 0.06375689582 | 0.1696050904 | 0.3759138106 | 0.706980973 | 1.065833259 |
| grp | 0.05630918645 | 0.1499530364 | 0.3755121458 | 0.7072796152 | 1.057924729 |
| casp10 | 0.05543716837 | 0.1477067685 | 0.3753190794 | 0.7074231783 | 1.057002602 |
| selplg | 0.0571439696 | 0.1523616728 | 0.3750547532 | 0.7076197466 | 1.058808236 |
| smad2 | 0.05741760617 | 0.1531143098 | 0.3749983019 | 0.7076617295 | 1.059098004 |
| susd2 | 0.05689966655 | 0.1521173317 | 0.3740511744 | 0.7083662434 | 1.058549597 |
| cr2 | 0.0538577656 | 0.1440600192 | 0.3738564378 | 0.7085111277 | 1.055334487 |
| pzp | 0.08171525638 | 0.21862821 | 0.3737635522 | 0.7085802385 | 1.085146777 |
| ppt1 | -0.060746847 | 0.1626273784 | -0.373533947 | 0.7087510846 | 0.9410614426 |
| armcx2 | 0.05766587217 | 0.1545514595 | 0.3731176164 | 0.7090609079 | 1.059360975 |
| irak1 | 0.05445755764 | 0.1460303904 | 0.3729193456 | 0.7092084732 | 1.055967658 |
| il1rl1 | -0.057209619 | 0.1534434338 | -0.372838498 | 0.7092686483 | 0.9443960849 |
| wnt9a | -0.05976127 | 0.1605973616 | -0.37211863 | 0.7098045263 | 0.9419893878 |
| itgb1bp1 | -0.05356621 | 0.1439643695 | -0.372079632 | 0.7098335609 | 0.9478431826 |
| snap29 | 0.05278897223 | 0.1420946296 | 0.371505752 | 0.7102608725 | 1.054207155 |
| pglyrp2 | 0.05772820797 | 0.1554173293 | 0.3714399689 | 0.7103098605 | 1.059427013 |
| spry2 | 0.05334626234 | 0.1438584991 | 0.370824544 | 0.7107682192 | 1.054794818 |
| prss2 | -0.051052496 | 0.1380625934 | -0.369777903 | 0.7115479806 | 0.9502287857 |
| ap2b1 | 0.06057854344 | 0.1643244829 | 0.3686519645 | 0.7123871573 | 1.062451043 |
| cacybp | 0.05810249137 | 0.1576626626 | 0.3685241033 | 0.712482476 | 1.059823613 |
| cabp2 | 0.05938790558 | 0.1613569422 | 0.3680529935 | 0.7128337201 | 1.061186801 |
| mzt1 | 0.05451359643 | 0.1483541917 | 0.3674557208 | 0.7132791149 | 1.056026835 |
| ceacam21 | 0.05540028114 | 0.1509193502 | 0.367085341 | 0.7135553614 | 1.056963613 |
| egln1 | 0.05782787909 | 0.1576922203 | 0.3667135828 | 0.7138326738 | 1.059532612 |
| ptprc | 0.05717455043 | 0.1559532559 | 0.3666133811 | 0.7139074255 | 1.058840615 |
| scamp3 | 0.0531478099 | 0.1451524631 | 0.3661516227 | 0.7142519387 | 1.054585512 |
| tnn | 0.0555468561 | 0.1518012616 | 0.3659182771 | 0.7144260576 | 1.057118548 |
| erbin | 0.05265249352 | 0.144003452 | 0.3656335511 | 0.7146385359 | 1.054063288 |
| washc3 | 0.05653657816 | 0.1550689969 | 0.3645898232 | 0.7154176126 | 1.05816532 |
| vegfc | 0.05184551773 | 0.1424767621 | 0.3638875348 | 0.7159419933 | 1.053213027 |
| masp1 | -0.053118812 | 0.1461437435 | -0.363469631 | 0.7162540951 | 0.9482673398 |
| eif4b | -0.052479799 | 0.1444017314 | -0.363429152 | 0.7162843279 | 0.9488734892 |
| ccl14 | 0.05385980221 | 0.1482574636 | 0.3632856038 | 0.7163915463 | 1.055336636 |
| tnfrsf13c | 0.0531188606 | 0.146311664 | 0.3630528091 | 0.7165654358 | 1.054554983 |
| slc4a1 | -0.057238155 | 0.1577359593 | -0.362873219 | 0.716699593 | 0.944369136 |
| atf2 | -0.053729251 | 0.1481184906 | -0.36274506 | 0.7167953361 | 0.9476886577 |
| kifbp | 0.05223058958 | 0.1441034317 | 0.3624520871 | 0.7170142218 | 1.053618668 |
| akt3 | 0.05301637195 | 0.1462790794 | 0.3624330435 | 0.7170284504 | 1.054446908 |
| cadps | -0.057929831 | 0.1600949328 | -0.361846748 | 0.7174665558 | 0.9437161649 |
| galnt7 | 0.05144270509 | 0.1421828952 | 0.3618065663 | 0.7174965849 | 1.052788865 |
| traf3ip2 | -0.057500884 | 0.1592888829 | -0.360984918 | 0.7181107215 | 0.9441210554 |
| l1cam | -0.052651765 | 0.1458996639 | -0.360876532 | 0.7181917479 | 0.9487103294 |

| Protein | beta | se | z | p_val | HR |
|---|---|---|---|---|---|
| crtam | 0.05248567627 | 0.1455248291 | 0.3606647512 | 0.7183500781 | 1.053887466 |
| dnajc6 | -0.055240493 | 0.1531919799 | -0.360596511 | 0.7184010978 | 0.9462575518 |
| rnaseh2a | 0.0569343714 | 0.1581247111 | 0.3600599236 | 0.7188023219 | 1.058586335 |
| megf9 | -0.053515076 | 0.1488828973 | -0.359444077 | 0.719262906 | 0.9478916508 |
| dtnb | 0.06209129719 | 0.1732038895 | 0.3584867371 | 0.7199790916 | 1.064059486 |
| s100a3 | 0.05543237677 | 0.1551151194 | 0.3573628218 | 0.7208202056 | 1.056997537 |
| twf2 | 0.05499829592 | 0.1539076148 | 0.3573461651 | 0.7208326735 | 1.056538814 |
| rgl2 | -0.056859506 | 0.1592742437 | -0.356991218 | 0.7210983804 | 0.9447267883 |
| col2a1 | 0.05465651692 | 0.1533374028 | 0.356446085 | 0.7215065218 | 1.056177773 |
| kazald1 | -0.051738817 | 0.145340251 | -0.355984093 | 0.7218524777 | 0.9495768474 |
| ccs | -0.051929268 | 0.1461263938 | -0.35537227 | 0.72231072 | 0.9493960169 |
| clul1 | 0.05375030465 | 0.1514366991 | 0.3549357915 | 0.7226376941 | 1.055221085 |
| evi2b | 0.05673857938 | 0.1603713637 | 0.3537949549 | 0.7234925547 | 1.058379092 |
| acadm | 0.05591683994 | 0.1587165383 | 0.3523063226 | 0.724608547 | 1.057509738 |
| dsg3 | 0.05362624992 | 0.1522434254 | 0.3522401692 | 0.7246581543 | 1.055090188 |
| ndufb7 | 0.05356137721 | 0.1522214077 | 0.3518649447 | 0.7249395501 | 1.055021744 |
| cntn4 | -0.052155494 | 0.1482871217 | -0.351719649 | 0.7250485234 | 0.949181263 |
| dnm3 | 0.05730272838 | 0.1630711072 | 0.3513971871 | 0.7252903924 | 1.058976344 |
| rwdd1 | -0.050755665 | 0.1444437613 | -0.351387034 | 0.725298008 | 0.9505108853 |
| rab33a | 0.05523905943 | 0.1573676446 | 0.3510191664 | 0.7255739695 | 1.056793221 |
| mepe | -0.052774314 | 0.1504067823 | -0.350877224 | 0.7256804591 | 0.9485940725 |
| siglec7 | -0.052393506 | 0.1495753175 | -0.350281762 | 0.7261272508 | 0.9489553739 |
| pla2g15 | 0.0507597029 | 0.1449888878 | 0.3500937464 | 0.726268344 | 1.052070054 |
| pibf1 | 0.05483705214 | 0.1566363288 | 0.3500915308 | 0.7262700066 | 1.056368468 |
| siglec5 | -0.049782717 | 0.1422164414 | -0.350048956 | 0.7263019575 | 0.9514361331 |
| chac2 | 0.05061153987 | 0.1446158346 | 0.3499723251 | 0.7263594672 | 1.051914187 |
| aifm1 | 0.05099544114 | 0.1457756483 | 0.3498213983 | 0.7264727389 | 1.052318096 |
| espl1 | -0.056011557 | 0.1601193661 | -0.349811259 | 0.726480349 | 0.9455282084 |
| il1rl2 | -0.054026963 | 0.1545933627 | -0.349477897 | 0.726730562 | 0.947406561 |
| sirt2 | -0.050496479 | 0.1447124616 | -0.348943542 | 0.7271316956 | 0.9507572764 |
| zp3 | -0.052099433 | 0.1493573609 | -0.348824008 | 0.7272214386 | 0.9492344767 |
| lacrt | 0.05612188615 | 0.1609979217 | 0.3485876437 | 0.7273989055 | 1.057726598 |
| crip2 | 0.05556249882 | 0.1595020237 | 0.3483498047 | 0.7275774945 | 1.057135085 |
| pttg1 | -0.054671137 | 0.1569792356 | -0.348269862 | 0.7276375251 | 0.9467964633 |
| cacnb3 | 0.05404473677 | 0.1551993225 | 0.3482279168 | 0.7276690237 | 1.055531822 |
| gmfg | 0.05266239754 | 0.1528291756 | 0.3445834039 | 0.7304075842 | 1.054073727 |
| pi3 | -0.054280902 | 0.1578592078 | -0.343856422 | 0.7309542661 | 0.947166008 |
| mmp1 | 0.05105723305 | 0.1485171123 | 0.3437801359 | 0.73101164 | 1.052383123 |
| tmem25 | -0.053522765 | 0.1557075918 | -0.343738955 | 0.7310426125 | 0.9478843623 |
| chm | 0.05505451495 | 0.160385868 | 0.34326288 | 0.7314007036 | 1.056598214 |
| pdap1 | 0.05205823941 | 0.1517193485 | 0.3431219545 | 0.7315067153 | 1.053437092 |
| spag1 | -0.054697235 | 0.1595850537 | -0.342746605 | 0.7317890983 | 0.9467717536 |
| mmp8 | 0.05037878416 | 0.1471597284 | 0.3423408341 | 0.7320944088 | 1.051669377 |
| mmp15 | -0.051126307 | 0.1493613365 | -0.342299476 | 0.7321255295 | 0.950158651 |
| cntnap2 | -0.052340747 | 0.1531705176 | -0.341715543 | 0.7325649739 | 0.9490054414 |
| dnph1 | -0.049442182 | 0.1446945696 | -0.341700331 | 0.7325764231 | 0.9517601851 |
| optc | -0.053479989 | 0.15653257 | -0.341654065 | 0.7326112451 | 0.9479249099 |
| tslp | 0.04973288617 | 0.1457221946 | 0.3412855968 | 0.7328885898 | 1.050990325 |
| prss27 | 0.04826055054 | 0.1414194177 | 0.3412583034 | 0.7329091349 | 1.049444053 |
| icoslg | -0.049301678 | 0.1445505173 | -0.341068843 | 0.7330517563 | 0.9518939212 |
| plb1 | -0.055881652 | 0.1641330155 | -0.340465642 | 0.7335058928 | 0.9456510447 |
| skiv2l | 0.05506354659 | 0.1619030274 | 0.3401020194 | 0.733779701 | 1.056607756 |
| ca13 | -0.048331176 | 0.1421747148 | -0.339942133 | 0.7339001068 | 0.9528181845 |
| spp1 | -0.050623999 | 0.1490245756 | -0.339702351 | 0.7340806913 | 0.9506360438 |
| akt2 | 0.052561231 | 0.1550677983 | 0.3389564537 | 0.7346425352 | 1.053967096 |
| calcoco1 | 0.0478827364 | 0.1413397926 | 0.3387774633 | 0.7347773801 | 1.049047633 |
| pcna | 0.05462672553 | 0.1612740825 | 0.3387198034 | 0.7348208207 | 1.056146309 |
| l3hypdh | 0.0512964488 | 0.1514490712 | 0.3387042813 | 0.7348325151 | 1.052634899 |
| ggt1 | 0.05586981455 | 0.1650192706 | 0.338565395 | 0.7349371553 | 1.057460009 |
| glrx | -0.050751423 | 0.1501184836 | -0.338075776 | 0.7353060846 | 0.9505149174 |
| tars1 | -0.052204728 | 0.1546021652 | -0.337671391 | 0.7356108363 | 0.9491345324 |
| tcof1 | 0.0529409338 | 0.1570323654 | 0.3371339001 | 0.7360159632 | 1.054367366 |
| nomo1 | -0.052169189 | 0.1547646919 | -0.337087148 | 0.7360512051 | 0.9491682647 |
| coq7 | 0.05322420076 | 0.1581319277 | 0.3365809899 | 0.7364327887 | 1.054666076 |
| plcb2 | 0.05134118937 | 0.1527889449 | 0.3360268597 | 0.7368506118 | 1.052681996 |
| add1 | -0.052367122 | 0.1562786709 | -0.335088093 | 0.7375586347 | 0.9489804115 |
| calb1 | 0.04940068551 | 0.1476310973 | 0.3346224909 | 0.7379098768 | 1.050641243 |
| fxn | 0.04756684958 | 0.1427064207 | 0.3333196176 | 0.7388930325 | 1.048716305 |
| calca | 0.06167009895 | 0.185332002 | 0.3327547228 | 0.7393194383 | 1.0636114 |
| cope | -0.048177703 | 0.1448332968 | -0.332642449 | 0.7394041969 | 0.9529644279 |

| Protein | beta | se | z | p_val | HR |
|---|---|---|---|---|---|
| eloa | -0.050844654 | 0.1531403908 | -0.332013347 | 0.739879181 | 0.9504263043 |
| il1a | -0.046939578 | 0.1418829095 | -0.330833204 | 0.7407704787 | 0.9541450477 |
| enpep | 0.05123988061 | 0.1551971252 | 0.3301599856 | 0.74127908 | 1.052575355 |
| oplah | 0.05062348456 | 0.1534426967 | 0.3299178498 | 0.7414620357 | 1.051926752 |
| lat | 0.04725883012 | 0.1432460449 | 0.329913682 | 0.741465185 | 1.04839333 |
| acyp1 | 0.05238704214 | 0.1588202386 | 0.3298511738 | 0.7415124182 | 1.053783522 |
| pecr | 0.05016947189 | 0.1525488291 | 0.3288748409 | 0.7422502915 | 1.051449272 |
| fli1 | 0.04756377273 | 0.1447042764 | 0.3286963862 | 0.7423851861 | 1.048713078 |
| rabepk | -0.047458592 | 0.1445932789 | -0.328221288 | 0.7427443534 | 0.9536499609 |
| actn2 | 0.05471351631 | 0.1668166155 | 0.3279860111 | 0.7429222396 | 1.056237976 |
| acot13 | 0.05002142223 | 0.152822451 | 0.3273172358 | 0.7434279575 | 1.051293617 |
| tnfrsf13b | 0.04755734971 | 0.1453992188 | 0.3270811913 | 0.7436064773 | 1.048706342 |
| mecr | 0.04930765213 | 0.1508814286 | 0.3267973574 | 0.7438211583 | 1.050543503 |
| ptprs | 0.04602946619 | 0.1411321776 | 0.3261443773 | 0.7443151228 | 1.047105265 |
| ccl20 | -0.04816383 | 0.1476945021 | -0.32610442 | 0.7443453528 | 0.952977648 |
| tmod4 | 0.05295051146 | 0.162468317 | 0.3259128453 | 0.7444902968 | 1.054377464 |
| myom2 | 0.05306613964 | 0.1630292304 | 0.3255007676 | 0.7448021018 | 1.054499387 |
| c1qtnf5 | 0.05147928526 | 0.1583434127 | 0.3251116317 | 0.7450965859 | 1.052827377 |
| c1rl | 0.05268082192 | 0.1620945744 | 0.325000526 | 0.7451806736 | 1.054093148 |
| obp2b | -0.060050638 | 0.1849227003 | -0.324733729 | 0.7453826047 | 0.9417168457 |
| crx | -0.046591313 | 0.1434971433 | -0.324684603 | 0.7454197888 | 0.9544774004 |
| clec1b | 0.0466947419 | 0.1438621481 | 0.3245797628 | 0.7454991456 | 1.04780211 |
| enoph1 | 0.05041222761 | 0.1560938934 | 0.3229609212 | 0.7467248378 | 1.051704549 |
| atxn10 | -0.046655546 | 0.1447088784 | -0.322409702 | 0.7471423354 | 0.9544160931 |
| dynlt1 | -0.052033107 | 0.1619237845 | -0.321343198 | 0.7479503233 | 0.9492974381 |
| ptpn6 | 0.04588152679 | 0.142788497 | 0.321325091 | 0.7479640439 | 1.046950368 |
| il18 | 0.0472178428 | 0.1469712553 | 0.3212726373 | 0.7480037905 | 1.04835036 |
| ceacam3 | 0.04623616345 | 0.1439290153 | 0.321242825 | 0.7480263809 | 1.047321721 |
| ciapin1 | 0.04633033391 | 0.1444698612 | 0.3206920359 | 0.7484437832 | 1.047420352 |
| vasp | 0.04875456228 | 0.152057571 | 0.3206322577 | 0.7484890891 | 1.049962619 |
| esm1 | 0.04745235338 | 0.1480465805 | 0.3205231302 | 0.7485717992 | 1.048596238 |
| ppm1f | 0.04906036717 | 0.1531476474 | 0.3203468549 | 0.748705408 | 1.050283751 |
| ogfr | 0.04821884699 | 0.1507742279 | 0.3198082833 | 0.7491136679 | 1.049400288 |
| cdhr2 | -0.059867505 | 0.1874036716 | -0.31945748 | 0.7493796296 | 0.9418893213 |
| cxcl11 | 0.04730828897 | 0.1482312119 | 0.3191520084 | 0.7496112471 | 1.048445183 |
| prdx6 | 0.04535898695 | 0.1422217578 | 0.3189314184 | 0.749778519 | 1.046403438 |
| gabarapl1 | 0.05035312486 | 0.1581382566 | 0.3184120399 | 0.7501724067 | 1.051642392 |
| gh1 | 0.05055964032 | 0.1589442369 | 0.3180967193 | 0.7504115721 | 1.051859595 |
| adam15 | 0.04577494141 | 0.1439785881 | 0.3179288117 | 0.7505389371 | 1.046838784 |
| efhd1 | -0.05391685 | 0.1696260783 | -0.317857078 | 0.7505933519 | 0.9475108891 |
| bcl2 | 0.05091198952 | 0.1603753554 | 0.3174551937 | 0.7508982327 | 1.052230282 |
| rnf168 | 0.04930387003 | 0.1553480228 | 0.3173768752 | 0.7509576519 | 1.05053953 |
| npdc1 | -0.047271594 | 0.1490553896 | -0.317141126 | 0.75113652 | 0.9538283082 |
| ecm1 | -0.047819307 | 0.1508162711 | -0.317069947 | 0.7511905279 | 0.9533060271 |
| angptl3 | -0.048864154 | 0.1543598507 | -0.316559996 | 0.7515774946 | 0.9523104888 |
| reg1b | 0.04674732414 | 0.1478158626 | 0.3162537723 | 0.7518098961 | 1.047857207 |
| tnfsf13 | 0.04767125054 | 0.150887215 | 0.3159396277 | 0.7520483327 | 1.048825798 |
| tmed10 | -0.05069968 | 0.1605785335 | -0.31573137 | 0.7522064141 | 0.9505641008 |
| rad23b | -0.045912744 | 0.1456396822 | -0.31524886 | 0.7525727102 | 0.9551252991 |
| gsr | -0.04970161 | 0.1577906546 | -0.314984497 | 0.7527734246 | 0.9515133042 |
| pde1c | -0.048864682 | 0.1552685851 | -0.314710682 | 0.7529813331 | 0.9523099854 |
| cr1 | -0.049770935 | 0.1582039951 | -0.314599737 | 0.7530655785 | 0.9514473425 |
| adamts8 | -0.04551762 | 0.1447891432 | -0.314371779 | 0.7532386877 | 0.955502766 |
| pmvk | -0.046054934 | 0.1466649676 | -0.31401455 | 0.753509988 | 0.9549894996 |
| tbcb | -0.045418296 | 0.1448363551 | -0.313583537 | 0.7538373652 | 0.9555976751 |
| frzb | 0.04669954239 | 0.1489952322 | 0.3134297769 | 0.7539541644 | 1.04780714 |
| lmod1 | -0.056667716 | 0.1810019988 | -0.313077844 | 0.7542215212 | 0.9449079954 |
| selp | -0.045481655 | 0.1457975804 | -0.311950687 | 0.7550780004 | 0.9555371314 |
| grpel1 | 0.04717270717 | 0.1512569229 | 0.3118713925 | 0.7551382644 | 1.048303043 |
| ikzf2 | 0.04504453962 | 0.144663956 | 0.3113736197 | 0.7555166062 | 1.046074451 |
| pdrg1 | -0.049838627 | 0.1601803686 | -0.311140669 | 0.7556936853 | 0.9513829396 |
| tp53bp1 | 0.04930631304 | 0.1586504062 | 0.3107859237 | 0.7559633714 | 1.050542096 |
| klrb1 | 0.04487657372 | 0.1444076653 | 0.3107630999 | 0.7559807237 | 1.045898761 |
| c9orf40 | -0.04820162 | 0.1552640521 | -0.310449326 | 0.7562192891 | 0.9529416353 |
| myom1 | 0.04846518621 | 0.1563399828 | 0.3099986666 | 0.7565619704 | 1.049658829 |
| bmp10 | -0.05223014 | 0.1687400244 | -0.30953024 | 0.7569182127 | 0.9491104132 |
| dars1 | 0.04431786566 | 0.1432151473 | 0.3094495694 | 0.7569795685 | 1.045314572 |
| adamtsl2 | 0.05094911143 | 0.1648135699 | 0.3091317751 | 0.7572212891 | 1.052269343 |
| hdac8 | -0.048094915 | 0.1556230112 | -0.309047577 | 0.7572853356 | 0.9530433251 |
| klk14 | -0.045045543 | 0.1459418629 | -0.308654023 | 0.7575847216 | 0.9559539437 |

| Protein | beta | se | z | p_val | HR |
|---|---|---|---|---|---|
| top1mt | 0.04888997608 | 0.1585669977 | 0.3083237797 | 0.7578359733 | 1.050104808 |
| vwc2l | 0.05181819063 | 0.1681791042 | 0.3081131327 | 0.7579962486 | 1.053184246 |
| cblif | 0.04294936715 | 0.139417411 | 0.3080631525 | 0.7580342786 | 1.043885039 |
| ncln | -0.044458453 | 0.1443642285 | -0.307960312 | 0.7581125322 | 0.9565153398 |
| igsf9 | -0.054746436 | 0.1778767457 | -0.307777363 | 0.7582517477 | 0.9467251732 |
| cdnf | 0.04556557699 | 0.1482766769 | 0.3073010398 | 0.7586142443 | 1.046619637 |
| asah2 | -0.045915467 | 0.1496597057 | -0.306799126 | 0.7589962734 | 0.9551226982 |
| taldo1 | 0.0492499274 | 0.1607253595 | 0.3064228791 | 0.7592826907 | 1.050482862 |
| syt1 | -0.047280328 | 0.1543121052 | -0.306394164 | 0.7593045512 | 0.9538199772 |
| lamp3 | 0.04779229077 | 0.1561988937 | 0.3059707378 | 0.7596269258 | 1.048952756 |
| rab3gap1 | 0.04577885167 | 0.1496580371 | 0.3058896974 | 0.7596886305 | 1.046842878 |
| bloc1s3 | 0.04689446922 | 0.153333821 | 0.3058325222 | 0.759732165 | 1.048011406 |
| fam20a | 0.05108686171 | 0.1670875157 | 0.3057491249 | 0.759795667 | 1.052414304 |
| ppm1a | -0.04354924 | 0.1428123149 | -0.30494037 | 0.7604115682 | 0.9573854111 |
| cdh4 | 0.04575175587 | 0.1503931069 | 0.3042144472 | 0.7609645189 | 1.046814513 |
| csnk2a1 | 0.04693863868 | 0.1545707934 | 0.3036708141 | 0.7613786955 | 1.048057697 |
| nit1 | -0.04718342 | 0.1554055322 | -0.303614805 | 0.7614213713 | 0.9539124147 |
| atp6v1f | -0.044024413 | 0.1451934108 | -0.303212197 | 0.7617281548 | 0.9569305956 |
| fgl1 | 0.0497344963 | 0.1641014256 | 0.3030716894 | 0.7618352287 | 1.050992017 |
| tfap2a | 0.04660055184 | 0.1538489664 | 0.3028980494 | 0.7619675579 | 1.047703422 |
| arhgap45 | 0.04746126549 | 0.1570685843 | 0.3021690537 | 0.7625231938 | 1.048605583 |
| cdhr1 | 0.04457301523 | 0.1477356388 | 0.30170794 | 0.762874715 | 1.045581317 |
| ccer2 | -0.046170085 | 0.1531108016 | -0.301546885 | 0.7629975036 | 0.9548795374 |
| f12 | 0.04720390256 | 0.157002283 | 0.3006574277 | 0.7636757353 | 1.048335746 |
| insr | 0.04682778845 | 0.1563791856 | 0.2994502641 | 0.7645965153 | 1.047941526 |
| dnmbp | 0.04486564598 | 0.1500178929 | 0.2990686318 | 0.7648876797 | 1.045887331 |
| fkbp4 | -0.043672159 | 0.1464014891 | -0.298304066 | 0.7654711012 | 0.9572677372 |
| hspb6 | 0.05315436959 | 0.1783909603 | 0.2979656004 | 0.7657294187 | 1.05459243 |
| epb41l5 | -0.047214495 | 0.1584864359 | -0.297908743 | 0.7657728147 | 0.9538827726 |
| smpd3 | 0.04671294959 | 0.1568593056 | 0.2978015834 | 0.765854606 | 1.047821188 |
| rad51 | 0.04691244476 | 0.1579168506 | 0.2970705442 | 0.7664126521 | 1.048030245 |
| cst5 | 0.04433554993 | 0.1495314643 | 0.2964964606 | 0.7668509695 | 1.045333057 |
| ing1 | 0.04352958801 | 0.1469043748 | 0.296312401 | 0.7669915163 | 1.044490898 |
| f9 | 0.04751720009 | 0.1603814889 | 0.2962760878 | 0.7670192458 | 1.048664238 |
| hgf | 0.04900274403 | 0.1655590418 | 0.2959834962 | 0.7672426851 | 1.050223233 |
| tmed8 | 0.04550364521 | 0.1538694153 | 0.2957289799 | 0.7674370638 | 1.04655482 |
| cradd | -0.042761535 | 0.1448485689 | -0.29521545 | 0.7678293006 | 0.9581398453 |
| hhex | 0.04660831948 | 0.1579410223 | 0.2950995175 | 0.7679178583 | 1.04771156 |
| dync1h1 | -0.046396405 | 0.1572274605 | -0.295090975 | 0.7679243841 | 0.9546634542 |
| cntf | 0.04443263254 | 0.1511550284 | 0.2939540484 | 0.7687930169 | 1.045434546 |
| ubxn1 | -0.046627952 | 0.1588638173 | -0.293508948 | 0.7691331608 | 0.95444243 |
| trim25 | -0.044765902 | 0.152564387 | -0.29342301 | 0.7691988397 | 0.9562213055 |
| sorcs2 | 0.0446971229 | 0.1523677146 | 0.2933503533 | 0.7692543693 | 1.04571109 |
| ccdc80 | 0.04615261588 | 0.1576572428 | 0.2927402197 | 0.769720725 | 1.047234223 |
| mtss1 | 0.04423833327 | 0.1511482623 | 0.2926817193 | 0.7697654442 | 1.045231439 |
| tmed4 | 0.04732695166 | 0.1617358787 | 0.2926187562 | 0.7698135757 | 1.04846475 |
| igfbp1 | -0.046895675 | 0.1603043916 | -0.292541427 | 0.7698726902 | 0.9541869375 |
| gdf2 | -0.045604851 | 0.1559413829 | -0.292448675 | 0.7699435971 | 0.9554194209 |
| tpm3 | -0.045970955 | 0.1572409584 | -0.292359927 | 0.7700114438 | 0.9550697016 |
| copb2 | 0.04603081222 | 0.1576185386 | 0.2920393288 | 0.770256554 | 1.047106674 |
| bid | -0.045336181 | 0.1553379901 | -0.291855074 | 0.7703974344 | 0.955676148 |
| ntrk3 | 0.04526533735 | 0.1551108723 | 0.29182569 | 0.770419902 | 1.046305447 |
| pnlip | -0.043843648 | 0.1504134426 | -0.291487563 | 0.7706784545 | 0.957103591 |
| bcam | 0.04265677308 | 0.1466157785 | 0.2909425815 | 0.7710952339 | 1.043579649 |
| ermap | 0.04911683885 | 0.1689006656 | 0.2908031101 | 0.7712019066 | 1.050343064 |
| adamts13 | -0.041788237 | 0.1437851636 | -0.290629688 | 0.7713345516 | 0.9590728549 |
| gstt2b | 0.04461664626 | 0.1539427367 | 0.2898262511 | 0.7719491639 | 1.045626938 |
| blvrb | -0.042864354 | 0.1480367842 | -0.289552045 | 0.7721589588 | 0.9580413362 |
| s100a16 | 0.0423144245 | 0.1464211543 | 0.288991196 | 0.7725881149 | 1.043222442 |
| dok1 | 0.0435430463 | 0.1507482935 | 0.2888460314 | 0.7726992048 | 1.044504955 |
| dlk1 | -0.042694999 | 0.1480152753 | -0.288449951 | 0.7730023367 | 0.9582035987 |
| creld2 | 0.04192971926 | 0.1453699883 | 0.2884344957 | 0.7730141654 | 1.042821186 |
| bcan | -0.044788548 | 0.1553254977 | -0.288352841 | 0.773076663 | 0.9561996503 |
| apod | 0.0471755117 | 0.163727055 | 0.2881351021 | 0.7732433239 | 1.048305983 |
| fcer1a | -0.044788786 | 0.1555579791 | -0.287923423 | 0.773405357 | 0.9561994234 |
| cntn2 | -0.043439742 | 0.1511029451 | -0.287484414 | 0.773741434 | 0.9574902491 |
| ern1 | 0.0456225602 | 0.158707369 | 0.2874634019 | 0.7737575209 | 1.046679278 |
| shpk | -0.045264428 | 0.1576191265 | -0.287175987 | 0.7739775721 | 0.9557447226 |
| cptp | 0.04548407672 | 0.1584160935 | 0.2871177778 | 0.7740221403 | 1.04653434 |
| csf2ra | -0.042040085 | 0.1464978573 | -0.286967238 | 0.7741374063 | 0.9588313447 |

| Protein | beta | se | z | p_val | HR |
|---|---|---|---|---|---|
| cxcl17 | -0.050991335 | 0.1777594177 | -0.286855885 | 0.7742226705 | 0.9502869047 |
| lrp11 | 0.04201624998 | 0.1464880726 | 0.2868236931 | 0.7742473204 | 1.042911426 |
| ctsf | 0.04229484001 | 0.1476477596 | 0.2864577162 | 0.7745275746 | 1.043202011 |
| tnip1 | 0.04322507431 | 0.1509942437 | 0.2862696831 | 0.7746715763 | 1.044172885 |
| tmprss11d | 0.04333540928 | 0.1523011163 | 0.2845376997 | 0.7759983472 | 1.0442881 |
| tcp11 | -0.044995241 | 0.1582597794 | -0.284312549 | 0.7761708699 | 0.9560020311 |
| gid8 | 0.04373418458 | 0.1538549762 | 0.2842558989 | 0.7762142802 | 1.044704619 |
| hgfac | -0.044518637 | 0.1566879247 | -0.284122961 | 0.7763161511 | 0.9564577742 |
| sugt1 | 0.04081145896 | 0.1439850144 | 0.2834424064 | 0.776837725 | 1.041655692 |
| sort1 | 0.0410143699 | 0.1447671164 | 0.2833127503 | 0.7769371042 | 1.041867077 |
| fetub | -0.043401991 | 0.1535633728 | -0.282632444 | 0.7774586074 | 0.9575263954 |
| gcc1 | 0.04286138384 | 0.1517097935 | 0.2825221948 | 0.7775431305 | 1.043793198 |
| smoc1 | 0.04347575456 | 0.1544160239 | 0.2815495016 | 0.7782889661 | 1.044434671 |
| klhl41 | -0.045703627 | 0.162380554 | -0.281459978 | 0.7783576205 | 0.9553250526 |
| cyp24a1 | 0.04480964764 | 0.1592964471 | 0.2812972194 | 0.7784824429 | 1.045828765 |
| ahsg | 0.04328905507 | 0.1548438256 | 0.2795659104 | 0.7798105641 | 1.044239694 |
| serpinb5 | 0.04049826547 | 0.144963548 | 0.2793686138 | 0.7799619551 | 1.041329503 |
| rab10 | -0.042181996 | 0.1517214047 | -0.278022708 | 0.7809949268 | 0.9586952861 |
| dkkl1 | -0.040202232 | 0.1446455043 | -0.277936268 | 0.7810612823 | 0.9605951567 |
| arhgap25 | -0.040372709 | 0.1452589582 | -0.277936103 | 0.7810614095 | 0.9604314113 |
| bax | -0.040797101 | 0.1468194498 | -0.277872592 | 0.781110164 | 0.960023898 |
| mtpn | -0.040343423 | 0.145400653 | -0.277463834 | 0.7814239727 | 0.960459539 |
| trim40 | 0.04392417019 | 0.1583229403 | 0.2774340226 | 0.7814468603 | 1.044903117 |
| igdcc4 | -0.044799531 | 0.1616924947 | -0.277066238 | 0.7817292455 | 0.9561891487 |
| capn3 | -0.042517843 | 0.1535105742 | -0.276970123 | 0.7818030474 | 0.9583733655 |
| pydc1 | 0.04232254281 | 0.1532404143 | 0.2761839493 | 0.7824067846 | 1.043230911 |
| clec3b | 0.04276666609 | 0.1548625958 | 0.2761587837 | 0.7824261125 | 1.043694337 |
| megf10 | -0.040689517 | 0.1476613534 | -0.27555969 | 0.7828862738 | 0.960127187 |
| becn1 | 0.04238238398 | 0.1540610449 | 0.2751012368 | 0.7832384614 | 1.043293341 |
| dffa | -0.039426181 | 0.1433156067 | -0.2751004 | 0.7832391046 | 0.9613409169 |
| ap3s2 | -0.041863353 | 0.1522509282 | -0.274962876 | 0.7833447602 | 0.9590008162 |
| arhgap1 | -0.039927161 | 0.1455479889 | -0.274323 | 0.7838364109 | 0.9608594247 |
| serpinf2 | 0.04350569144 | 0.1586595736 | 0.2742077924 | 0.7839249401 | 1.044465939 |
| pyy | 0.04372788764 | 0.1596344115 | 0.2739251972 | 0.7841421073 | 1.044698041 |
| epha10 | -0.039633654 | 0.1456347504 | -0.272144209 | 0.785511138 | 0.961141485 |
| lpl | 0.04438058559 | 0.1637633698 | 0.2710043499 | 0.7863876867 | 1.045380136 |
| ssc4d | 0.04525280197 | 0.1671175561 | 0.2707842493 | 0.7865569747 | 1.046292331 |
| mamdc2 | -0.041866514 | 0.1546349333 | -0.270744214 | 0.7865877682 | 0.9589977852 |
| fgf16 | 0.042397603 | 0.1567052294 | 0.2705564017 | 0.7867322318 | 1.043309219 |
| il12a_il12b | 0.04009749762 | 0.1483038398 | 0.2703739679 | 0.7868725651 | 1.040912256 |
| wasf1 | 0.0393801519 | 0.1457369557 | 0.270213905 | 0.7869956959 | 1.04016583 |
| gata3 | 0.04323699212 | 0.1600479 | 0.2701503245 | 0.7870446075 | 1.044185329 |
| serpinh1 | 0.04225405105 | 0.1564371823 | 0.270102353 | 0.7870815119 | 1.043159461 |
| hla_dra | -0.040492029 | 0.1499678276 | -0.27000477 | 0.7871565839 | 0.9603168194 |
| nfu1 | 0.04117166805 | 0.1525573406 | 0.2698766766 | 0.787255131 | 1.042030974 |
| prl | -0.039953399 | 0.1480518354 | -0.269860883 | 0.7872672821 | 0.960834214 |
| enah | 0.04002719806 | 0.1484274495 | 0.2696751726 | 0.7874101625 | 1.040839083 |
| tet2 | 0.04166834799 | 0.1546880592 | 0.2693701647 | 0.787644843 | 1.042548658 |
| serpine2 | 0.04135204239 | 0.1537464956 | 0.2689625036 | 0.7879585375 | 1.042218946 |
| tbca | -0.042934286 | 0.1597061665 | -0.268832987 | 0.7880582078 | 0.9579743406 |
| timp4 | 0.04091022082 | 0.1522130302 | 0.2687695052 | 0.7881070614 | 1.041758573 |
| c1s | 0.04229102291 | 0.1574103536 | 0.2686673521 | 0.7881856775 | 1.043198029 |
| dbi | 0.04013757781 | 0.1495402205 | 0.2684065711 | 0.7883863821 | 1.040953977 |
| cxcl5 | -0.039254367 | 0.1464982373 | -0.267951123 | 0.7887369415 | 0.9615061024 |
| yod1 | 0.04352203338 | 0.1624386445 | 0.2679290603 | 0.7887539243 | 1.044483008 |
| eno1 | -0.038445988 | 0.1437882278 | -0.267379243 | 0.7891771793 | 0.9622836787 |
| ccdc28a | -0.043664771 | 0.1635857865 | -0.266922769 | 0.789528625 | 0.9572748098 |
| rabep1 | 0.04171432863 | 0.156354566 | 0.2667931592 | 0.7896284216 | 1.042596596 |
| abraxas2 | -0.042287879 | 0.1585370501 | -0.266738144 | 0.7896707826 | 0.9585937822 |
| palm2 | 0.0485429647 | 0.1821270838 | 0.2665334759 | 0.7898283814 | 1.049740473 |
| fcgr2b | 0.03832002113 | 0.1439211636 | 0.2662570268 | 0.7900412665 | 1.039063702 |
| fgf2 | -0.039668267 | 0.1494447802 | -0.265437622 | 0.7906723571 | 0.9611082175 |
| ceacam16 | -0.042802166 | 0.1612668278 | -0.265412092 | 0.7906920217 | 0.958100916 |
| ddhd2 | -0.041074298 | 0.1557640206 | -0.263695669 | 0.7920144334 | 0.9597578196 |
| parp1 | 0.04083517592 | 0.1549293765 | 0.263572841 | 0.7921090884 | 1.041680397 |
| fhit | -0.03828612 | 0.145289145 | -0.263516724 | 0.7921523349 | 0.9624375293 |
| c8b | 0.04078031288 | 0.1551672696 | 0.2628151735 | 0.7926930398 | 1.041623249 |
| myh7b | 0.04219706459 | 0.160873575 | 0.2622995392 | 0.7930905173 | 1.043100017 |
| clec4d | 0.04053569037 | 0.1548793714 | 0.2617242697 | 0.793534028 | 1.041368476 |
| prr5 | 0.04076458567 | 0.1559104031 | 0.2614616143 | 0.7937365475 | 1.041606867 |

| Protein | beta | se | z | p_val | HR |
|---|---|---|---|---|---|
| scgb2a2 | -0.041557531 | 0.159312997 | -0.260854616 | 0.7942046245 | 0.9592941449 |
| itih5 | 0.03961161067 | 0.1527312872 | 0.2593549193 | 0.7953614091 | 1.040406613 |
| sh3glb2 | -0.040058854 | 0.1546074188 | -0.259100464 | 0.7955577267 | 0.9607328946 |
| klrc1 | -0.039491984 | 0.1525295447 | -0.258913671 | 0.7957018495 | 0.9612776592 |
| slc16a1 | -0.038387224 | 0.1483333003 | -0.25879033 | 0.7957970195 | 0.9623402278 |
| nenf | 0.03989832478 | 0.154312672 | 0.2585550769 | 0.7959785482 | 1.040704955 |
| pcare | 0.0397534663 | 0.1537836696 | 0.258502521 | 0.7960191035 | 1.040554211 |
| defb116 | 0.03958904248 | 0.1535781976 | 0.2577777516 | 0.796578436 | 1.040383133 |
| gigyf2 | 0.04050325496 | 0.1573939099 | 0.2573368626 | 0.7969187381 | 1.041334699 |
| yes1 | 0.03673164471 | 0.1428455686 | 0.2571423466 | 0.7970688885 | 1.037414588 |
| tnc | -0.03696115 | 0.1437833102 | -0.257061479 | 0.7971313134 | 0.9637135745 |
| gpr15l | 0.04259872782 | 0.166009503 | 0.2566041525 | 0.7974843691 | 1.043519076 |
| elob | 0.04119308169 | 0.1605680603 | 0.2565459258 | 0.797529323 | 1.042053288 |
| cgref1 | -0.037119286 | 0.144711545 | -0.256505351 | 0.797560649 | 0.9635611895 |
| serpina11 | 0.04041712574 | 0.1583212943 | 0.2552854682 | 0.7985026229 | 1.041245014 |
| wasf3 | 0.03688132565 | 0.1444806783 | 0.2552682205 | 0.7985159434 | 1.037569881 |
| nbn | -0.037258742 | 0.1460413501 | -0.255124608 | 0.7986268585 | 0.9634268239 |
| src | 0.03611818125 | 0.1426114384 | 0.2532628635 | 0.8000650914 | 1.036778367 |
| mgll | 0.03612462452 | 0.1427150255 | 0.253124185 | 0.8001722504 | 1.036785047 |
| plxna4 | 0.03795644911 | 0.1500926188 | 0.2528868468 | 0.8003556539 | 1.038685996 |
| ceacam19 | -0.039645288 | 0.1570593645 | -0.252422311 | 0.8007146568 | 0.9611303034 |
| siglec9 | 0.03712544207 | 0.1473310294 | 0.2519865789 | 0.8010514376 | 1.037823199 |
| ids | 0.03600085266 | 0.1432614142 | 0.2512948295 | 0.8015861724 | 1.03665673 |
| mrpl24 | 0.040924053 | 0.1633739634 | 0.250493115 | 0.8022060285 | 1.041772983 |
| cpa4 | 0.03963419335 | 0.1585715635 | 0.2499451508 | 0.8026297658 | 1.040430108 |
| snx9 | -0.035604453 | 0.1435208972 | -0.248078532 | 0.8040736456 | 0.9650219291 |
| txnrd1 | -0.036759901 | 0.1482514207 | -0.247956487 | 0.8041680741 | 0.9639075404 |
| casp2 | 0.03749496442 | 0.151485964 | 0.2475144457 | 0.8045101132 | 1.038206769 |
| gfer | 0.03715800034 | 0.150481952 | 0.2469266236 | 0.8049650115 | 1.03785699 |
| rilpl2 | 0.03723486676 | 0.151199134 | 0.2462637568 | 0.805478064 | 1.037936769 |
| cd7 | 0.03855343247 | 0.1568055147 | 0.2458678353 | 0.8057845434 | 1.03930626 |
| cela3a | 0.03445731058 | 0.1401535149 | 0.2458540594 | 0.8057952077 | 1.035057841 |
| klk7 | -0.040351587 | 0.1642132586 | -0.24572673 | 0.8058937791 | 0.9604516974 |
| igsf21 | 0.03841237091 | 0.1567614754 | 0.2450370591 | 0.8064277369 | 1.039159664 |
| sestd1 | -0.035504841 | 0.1449222336 | -0.244992366 | 0.8064623421 | 0.9651180621 |
| ngfr | 0.03677005652 | 0.1508234172 | 0.243795408 | 0.807389277 | 1.037454438 |
| chmp1a | 0.03476623659 | 0.1427688778 | 0.2435141127 | 0.8076071538 | 1.035377647 |
| slamf7 | -0.03678073 | 0.1511797937 | -0.243291308 | 0.8077797374 | 0.963887464 |
| cndp1 | -0.036331029 | 0.1495087685 | -0.243002662 | 0.808003335 | 0.9643210226 |
| tff2 | 0.03719808154 | 0.1531186394 | 0.242936338 | 0.808054715 | 1.037898589 |
| smarca2 | 0.03539789184 | 0.1463614129 | 0.2418526246 | 0.8088943605 | 1.036031855 |
| nlgn1 | -0.038533771 | 0.1595996195 | -0.241440244 | 0.8092139247 | 0.9621992096 |
| hcg22 | 0.03861586599 | 0.1602525125 | 0.2409688646 | 0.809579248 | 1.039371149 |
| stx7 | 0.03857103648 | 0.1600904392 | 0.2409327919 | 0.8096072064 | 1.039324556 |
| glo1 | -0.035726677 | 0.1489695763 | -0.239825327 | 0.8104656718 | 0.9649039876 |
| flt3 | 0.03512774352 | 0.1471445148 | 0.2387295481 | 0.811315303 | 1.035752011 |
| gm2a | -0.038687088 | 0.1625831691 | -0.237952602 | 0.8119178568 | 0.9620516995 |
| ppp1r12a | 0.0338799549 | 0.1431998258 | 0.2365921516 | 0.8129732095 | 1.034460417 |
| tmed1 | 0.03690384124 | 0.1560138034 | 0.2365421549 | 0.8130120003 | 1.037593242 |
| ccl27 | 0.03527822666 | 0.1492299601 | 0.2364017697 | 0.8131209231 | 1.035907886 |
| b4gat1 | 0.03638355406 | 0.1539586303 | 0.2363203282 | 0.8131841139 | 1.037053536 |
| aarsd1 | 0.03509534164 | 0.1485624913 | 0.2362328562 | 0.8132519854 | 1.035718451 |
| rexo2 | 0.03689709919 | 0.1572251079 | 0.234676889 | 0.8144595267 | 1.037586247 |
| agxt | -0.035653621 | 0.1528118973 | -0.233317047 | 0.815515222 | 0.9649744828 |
| osmr | -0.034960132 | 0.1500243889 | -0.233029656 | 0.8157383776 | 0.9656439141 |
| pla2g4a | 0.03313561815 | 0.1422696504 | 0.2329071453 | 0.8158335098 | 1.033690717 |
| commd1 | 0.0359006706 | 0.1542972419 | 0.2326721474 | 0.8160159988 | 1.036552881 |
| gfod2 | -0.033272696 | 0.1432140683 | -0.232328403 | 0.8162829538 | 0.967274752 |
| snx18 | -0.035285572 | 0.1521484813 | -0.231915377 | 0.8166037409 | 0.9653297054 |
| bloc1s2 | 0.03544907326 | 0.1528997131 | 0.2318452569 | 0.8166582047 | 1.036084882 |
| ahcy | -0.03370759 | 0.1455031867 | -0.23166221 | 0.8168003848 | 0.9668541813 |
| slc39a14 | 0.03382761165 | 0.1460645349 | 0.2315936011 | 0.816853678 | 1.034406272 |
| stxbp1 | 0.0359145424 | 0.1555114834 | 0.2309446326 | 0.817357816 | 1.03656726 |
| casp3 | -0.03284496 | 0.1422939188 | -0.230824761 | 0.8174509443 | 0.9676885786 |
| hcls1 | -0.033411154 | 0.144797454 | -0.230744071 | 0.8175136338 | 0.967140834 |
| nrn1 | -0.035763534 | 0.1551610791 | -0.230492946 | 0.8177087439 | 0.9648684248 |
| cd40lg | 0.03269534946 | 0.1418543314 | 0.2304853799 | 0.8177146228 | 1.033235715 |
| rabgap1l | 0.03336969358 | 0.1447960611 | 0.230459954 | 0.8177343781 | 1.033932707 |
| ak2 | -0.034892333 | 0.1515403936 | -0.23025104 | 0.8178967032 | 0.9657093855 |
| smoc2 | -0.036688625 | 0.1593519672 | -0.23023641 | 0.8179080703 | 0.9639762468 |

| Protein | beta | se | z | p_val | HR |
|---|---|---|---|---|---|
| gpd1 | 0.03996681892 | 0.1742908367 | 0.2293110737 | 0.8186271472 | 1.04077624 |
| il18r1 | -0.035616929 | 0.1557994452 | -0.228607547 | 0.8191739586 | 0.9650098901 |
| itga5 | -0.035342043 | 0.1550740589 | -0.227904285 | 0.8197206514 | 0.9652751946 |
| mb | -0.039769046 | 0.1745290714 | -0.227864882 | 0.8197512846 | 0.9610113627 |
| sftpd | 0.03370281415 | 0.1482378341 | 0.2273563585 | 0.8201466525 | 1.034277189 |
| gucy2c | 0.03580924835 | 0.1576515004 | 0.2271418176 | 0.820313468 | 1.036458122 |
| dtymk | -0.035299836 | 0.1554521812 | -0.227078421 | 0.8203627636 | 0.9653159366 |
| cdk5rap3 | -0.035052344 | 0.1547473496 | -0.226513369 | 0.8208021623 | 0.9655548744 |
| arg2 | -0.034758942 | 0.1536457179 | -0.226227859 | 0.8210242036 | 0.9658382113 |
| myoc | 0.03391638544 | 0.1500679277 | 0.2260068888 | 0.8211960625 | 1.034498104 |
| prc1 | -0.034888872 | 0.1544252909 | -0.225927193 | 0.8212580476 | 0.9657127275 |
| eif4e | -0.035111208 | 0.1554652176 | -0.225846066 | 0.8213211473 | 0.9654980394 |
| rgmb | -0.033140026 | 0.1469788176 | -0.225474844 | 0.8216098934 | 0.9674030886 |
| inhbc | 0.03569250031 | 0.1583114667 | 0.2254574546 | 0.8216234196 | 1.036337124 |
| map2k1 | 0.03539156339 | 0.1574344157 | 0.2248019484 | 0.8221333503 | 1.036025299 |
| stxbp3 | 0.03227700671 | 0.143809503 | 0.2244427944 | 0.8224127748 | 1.032803559 |
| tigit | 0.03487980087 | 0.156101207 | 0.2234435053 | 0.8231903478 | 1.035495236 |
| fabp9 | -0.034045893 | 0.1529589377 | -0.222581916 | 0.8238609119 | 0.9665271463 |
| hmcn2 | 0.03551883197 | 0.1596866706 | 0.2224282831 | 0.8239804965 | 1.036157161 |
| il24 | 0.0315804823 | 0.1420277698 | 0.2223542785 | 0.8240381013 | 1.032084437 |
| hyou1 | -0.031436565 | 0.1416514236 | -0.221929045 | 0.8243691197 | 0.9690524262 |
| ctrb1 | 0.03116691793 | 0.1408566701 | 0.2212668943 | 0.8248846256 | 1.031657692 |
| cspg5 | -0.033608095 | 0.152014004 | -0.221085522 | 0.8250258434 | 0.9669503827 |
| ncs1 | 0.03429211081 | 0.1551498029 | 0.2210258097 | 0.8250723367 | 1.034886864 |
| crh | 0.03175485968 | 0.1445481714 | 0.2196835793 | 0.8261175941 | 1.032264425 |
| tppp3 | 0.03218735296 | 0.1472009742 | 0.2186626354 | 0.8269128572 | 1.032710969 |
| was | -0.031463307 | 0.144130486 | -0.218297378 | 0.8271974173 | 0.9690265121 |
| pnliprp1 | -0.033117379 | 0.1518237573 | -0.218130415 | 0.8273275002 | 0.9674249974 |
| rgs10 | -0.033463689 | 0.1536740056 | -0.21775764 | 0.8276179513 | 0.9670900269 |
| tjap1 | 0.03107093987 | 0.1426893414 | 0.2177523532 | 0.8276220703 | 1.03155868 |
| flt3lg | 0.0322287419 | 0.1480231449 | 0.2177277204 | 0.8276412641 | 1.032753712 |
| dnajb2 | 0.03473931517 | 0.1595883817 | 0.2176807284 | 0.8276778801 | 1.035349774 |
| ror1 | 0.03157979641 | 0.1456192216 | 0.2168655764 | 0.8283131053 | 1.032083729 |
| hnf1a | 0.03467681254 | 0.160035853 | 0.2166815241 | 0.8284565476 | 1.035285064 |
| vtcn1 | -0.031994123 | 0.1478882171 | -0.216339904 | 0.8287228066 | 0.9685122743 |
| heph | 0.03425314637 | 0.1585945061 | 0.2159794007 | 0.8290038045 | 1.034846541 |
| urod | -0.034181042 | 0.1588674583 | -0.215154459 | 0.8296468959 | 0.9663965304 |
| hs1bp3 | -0.033451978 | 0.155546412 | -0.215061072 | 0.8297197041 | 0.9671013522 |
| edf1 | 0.03391354241 | 0.1583043856 | 0.2142299614 | 0.8303677326 | 1.034495163 |
| fosb | 0.03100642006 | 0.1447643794 | 0.2141854245 | 0.8304024619 | 1.031492126 |
| nxph3 | -0.034900376 | 0.1633237189 | -0.213688351 | 0.8307900961 | 0.9657016184 |
| celsr2 | -0.03354452 | 0.1572900114 | -0.213265415 | 0.831119948 | 0.9670118594 |
| tigar | 0.03142801077 | 0.1475474351 | 0.2130027591 | 0.8313248106 | 1.031927085 |
| inppl1 | 0.03072908063 | 0.1447035322 | 0.2123588842 | 0.8318270599 | 1.031206092 |
| tab2 | 0.03320049449 | 0.1570419253 | 0.2114116624 | 0.8325660571 | 1.033757781 |
| sla2 | -0.032518714 | 0.1544724893 | -0.210514601 | 0.8332660574 | 0.9680043341 |
| cemip2 | 0.03323343552 | 0.1580656468 | 0.2102508432 | 0.8334718992 | 1.033791835 |
| ilkap | -0.030083961 | 0.1442663268 | -0.208530717 | 0.8348146014 | 0.9703640579 |
| nxph1 | -0.030886501 | 0.1481474927 | -0.208484802 | 0.8348504485 | 0.9695856142 |
| apex1 | -0.030378163 | 0.1462947728 | -0.207650367 | 0.8355019741 | 0.9700786161 |
| trdmt1 | 0.03182123716 | 0.1533215264 | 0.207545789 | 0.8355836366 | 1.032332946 |
| pdgfra | -0.030028705 | 0.1447127486 | -0.207505594 | 0.8356150241 | 0.9704176774 |
| siglec6 | -0.02973232 | 0.1437777018 | -0.206793683 | 0.8361709898 | 0.9707053367 |
| clpp | 0.02989556543 | 0.1446066169 | 0.2067371886 | 0.8362151125 | 1.030346924 |
| nexn | -0.031338707 | 0.1517989751 | -0.206448738 | 0.8364404034 | 0.9691472607 |
| elac1 | 0.03195895075 | 0.1549685804 | 0.206228583 | 0.8366123617 | 1.032475122 |
| clta | 0.03074784561 | 0.1491106874 | 0.2062081944 | 0.8366282872 | 1.031225443 |
| ttn | 0.0343606103 | 0.1669500132 | 0.2058137621 | 0.8369363908 | 1.034957756 |
| f11r | 0.03048056992 | 0.1483003171 | 0.205532736 | 0.8371559244 | 1.030949858 |
| angptl4 | 0.0308894821 | 0.1507029162 | 0.2049693721 | 0.8375960545 | 1.031371513 |
| cacna1h | -0.032633569 | 0.1593539559 | -0.204786692 | 0.8377387849 | 0.9678931602 |
| gp5 | 0.03203683637 | 0.1564726199 | 0.2047440402 | 0.8377721101 | 1.03255554 |
| adam9 | -0.032883842 | 0.1617704403 | -0.203274727 | 0.8389203083 | 0.9676509534 |
| sost | 0.03117943036 | 0.1535422942 | 0.2030673732 | 0.8390823728 | 1.0316706 |
| ndrg1 | -0.029986091 | 0.1478268712 | -0.202846013 | 0.8392553923 | 0.9704590312 |
| ptprk | -0.03208108 | 0.1583528004 | -0.202592437 | 0.839453602 | 0.968428059 |
| angptl7 | 0.0292779425 | 0.1451028059 | 0.201773786 | 0.8400935758 | 1.029710755 |
| cntn1 | -0.030553204 | 0.1514885665 | -0.201686534 | 0.8401617909 | 0.9699088278 |
| fas | 0.03225798519 | 0.1599966885 | 0.2016165802 | 0.8402164823 | 1.032783914 |
| nit2 | 0.03162492653 | 0.1570648237 | 0.2013495179 | 0.8404252856 | 1.032130308 |

| Protein | beta | se | z | p_val | HR |
|---|---|---|---|---|---|
| cxcl6 | 0.02880048648 | 0.1432140702 | 0.2011009564 | 0.8406196342 | 1.029219231 |
| snap23 | -0.028737221 | 0.1429363584 | -0.201049061 | 0.8406602123 | 0.9716717662 |
| drg2 | 0.0287886678 | 0.1435344051 | 0.2005698061 | 0.8410349696 | 1.029207067 |
| lpa | 0.0306171864 | 0.1530505212 | 0.2000462733 | 0.8414443916 | 1.031090713 |
| nacc1 | 0.03106128197 | 0.1559214308 | 0.1992111143 | 0.8420976057 | 1.031548717 |
| nap1l4 | 0.03105772288 | 0.1561700695 | 0.1988711601 | 0.8423635298 | 1.031545046 |
| eif5 | 0.0321218322 | 0.1619736465 | 0.1983151759 | 0.8427984788 | 1.032643307 |
| brk1 | -0.028984719 | 0.1463925757 | -0.197993097 | 0.8430504647 | 0.9714313084 |
| slc39a5 | 0.03224908386 | 0.1629682122 | 0.1978857314 | 0.8431344681 | 1.032774721 |
| rasgrf1 | 0.03153563365 | 0.1599284837 | 0.1971858478 | 0.843682105 | 1.03203815 |
| lama1 | 0.03080452795 | 0.1571441456 | 0.196027207 | 0.844588871 | 1.031283897 |
| cc2d1a | -0.028837491 | 0.1473658148 | -0.195686435 | 0.8448556021 | 0.9715743412 |
| thrap3 | -0.031517833 | 0.1611203788 | -0.195616675 | 0.8449102076 | 0.9689736768 |
| nub1 | 0.02949963316 | 0.1508283752 | 0.1955841075 | 0.8449357004 | 1.029939058 |
| reg3a | -0.028927845 | 0.1479320631 | -0.195548175 | 0.8449638271 | 0.9714865595 |
| ccl2 | 0.02808570438 | 0.1442841735 | 0.1946554754 | 0.8456626705 | 1.028483826 |
| ppp1r14a | -0.029754763 | 0.153153364 | -0.194280832 | 0.8459559938 | 0.9706835519 |
| ppp1r12b | 0.03063159566 | 0.1578581822 | 0.1940450298 | 0.8461406233 | 1.03110557 |
| apoc1 | 0.03184446808 | 0.1645329388 | 0.193544638 | 0.8465324508 | 1.032356928 |
| hsp90b1 | 0.02767332288 | 0.1437123001 | 0.1925605732 | 0.847303125 | 1.028059786 |
| faslg | -0.029402682 | 0.1528823009 | -0.19232234 | 0.84748972 | 0.9710253714 |
| rac3 | -0.030810089 | 0.1603125843 | -0.192187587 | 0.8475952692 | 0.969659705 |
| bag3 | -0.028254733 | 0.1471930964 | -0.191956915 | 0.8477759543 | 0.9721406992 |
| lpp | -0.030506316 | 0.1592069981 | -0.191614163 | 0.8480444475 | 0.9699543061 |
| hars1 | -0.027481406 | 0.1436821413 | -0.191265284 | 0.8483177583 | 0.9728927728 |
| muc2 | -0.02884108 | 0.1509087095 | -0.191116074 | 0.8484346547 | 0.9715708542 |
| aldh1a1 | -0.02920494 | 0.1532155932 | -0.190613364 | 0.8488285188 | 0.9712174031 |
| dlgap5 | -0.03003623 | 0.157643709 | -0.190532375 | 0.8488919759 | 0.9704103746 |
| id4 | -0.029583097 | 0.1553166453 | -0.190469585 | 0.8489411742 | 0.9708501995 |
| hadh | 0.03002154011 | 0.1584908865 | 0.1894212392 | 0.8497626793 | 1.03047673 |
| stk4 | 0.02693191327 | 0.1426255194 | 0.1888295544 | 0.8502264075 | 1.027297855 |
| clec14a | 0.02821640635 | 0.1496375909 | 0.1885649601 | 0.8504337979 | 1.02861826 |
| acan | -0.029638971 | 0.1582160492 | -0.187332264 | 0.8514001273 | 0.970795956 |
| rfc4 | -0.029541555 | 0.1578284685 | -0.187175073 | 0.8515233687 | 0.9708905314 |
| havcr1 | 0.03057771929 | 0.1635700708 | 0.1869395736 | 0.8517080109 | 1.031050019 |
| flt4 | -0.027479514 | 0.1473931753 | -0.186436811 | 0.8521022275 | 0.9728946135 |
| cxcl8 | -0.027363461 | 0.1470663162 | -0.186062052 | 0.8523961001 | 0.9730075273 |
| igbp1 | -0.028886042 | 0.1567285423 | -0.184306203 | 0.8537732484 | 0.971527171 |
| mcfd2 | -0.026609822 | 0.1444191627 | -0.184254092 | 0.8538141265 | 0.9737411001 |
| reep4 | 0.02823639418 | 0.1532854805 | 0.1842078852 | 0.8538503739 | 1.02863882 |
| pgm2 | -0.02926696 | 0.1589435286 | -0.184134327 | 0.8539080775 | 0.97115717 |
| pts | -0.028195158 | 0.1532758787 | -0.183950393 | 0.8540523715 | 0.9721986159 |
| chmp6 | -0.028263633 | 0.1539454767 | -0.183595083 | 0.8543311205 | 0.9721320473 |
| pxn | -0.027960126 | 0.1524255962 | -0.183434584 | 0.8544570419 | 0.9724271407 |
| nphs2 | 0.0281283462 | 0.1536830505 | 0.1830282917 | 0.85477582 | 1.028527684 |
| pgd | -0.028607159 | 0.1564331779 | -0.182871428 | 0.8548989021 | 0.971798152 |
| spart | 0.02889695725 | 0.1585179286 | 0.1822945676 | 0.8553515617 | 1.029318525 |
| flrt2 | -0.026186022 | 0.1437816423 | -0.182123541 | 0.8554857743 | 0.9741538588 |
| cryzl1 | 0.02774424665 | 0.1525288939 | 0.1818950229 | 0.8556651105 | 1.028132702 |
| cdc37 | -0.025730829 | 0.142387887 | -0.180709393 | 0.8565956855 | 0.974597388 |
| plxnb2 | 0.02695230016 | 0.1491572452 | 0.1806972241 | 0.8566052378 | 1.027318799 |
| nmnat1 | -0.026564703 | 0.1472991253 | -0.180345288 | 0.8568815036 | 0.9737850348 |
| siglec15 | -0.026043163 | 0.1447758735 | -0.179886073 | 0.8572420087 | 0.9742930349 |
| prdx1 | -0.025732561 | 0.1431050116 | -0.179815932 | 0.8572970752 | 0.9745956996 |
| park7 | -0.026327229 | 0.1469051341 | -0.179212451 | 0.8577708872 | 0.9740163109 |
| oxt | 0.02827878066 | 0.1578896399 | 0.1791047258 | 0.8578554714 | 1.028682421 |
| bsg | -0.026471806 | 0.1485037188 | -0.178256857 | 0.8585212593 | 0.9738755007 |
| rnase4 | -0.030625658 | 0.172184698 | -0.177865156 | 0.8588288761 | 0.9698385563 |
| dnm1 | -0.02741085 | 0.1542386794 | -0.177717095 | 0.8589451592 | 0.9729614182 |
| gclm | -0.027907256 | 0.1573251362 | -0.177385872 | 0.859205304 | 0.9724785538 |
| kir2dl2 | -0.026577851 | 0.1505688835 | -0.176516223 | 0.8598884045 | 0.9737722321 |
| ca9 | -0.025156423 | 0.1429100231 | -0.1760298 | 0.8602705312 | 0.9751573633 |
| st13 | -0.027786984 | 0.1578674448 | -0.176014655 | 0.860282429 | 0.9725955233 |
| fgf19 | 0.02492124387 | 0.1415929375 | 0.1760062635 | 0.8602890217 | 1.025234374 |
| c1ql2 | 0.02811229875 | 0.1603467636 | 0.1753218968 | 0.8608267071 | 1.028511178 |
| traf2 | 0.02678336834 | 0.1528881139 | 0.175182803 | 0.8609359966 | 1.027145266 |
| umod | -0.026679901 | 0.1527494896 | -0.174664421 | 0.8613433255 | 0.9736728631 |
| brap | 0.02656827787 | 0.1525201378 | 0.1741952129 | 0.8617120476 | 1.026924361 |
| s100a11 | 0.02633967947 | 0.1515246394 | 0.1738309992 | 0.8619982815 | 1.026689635 |
| cpa1 | 0.02439641202 | 0.1403905543 | 0.1737753095 | 0.8620420493 | 1.024696439 |

| Protein | beta | se | z | p_val | HR |
|---|---|---|---|---|---|
| pbxip1 | -0.027875624 | 0.1606207217 | -0.173549365 | 0.862219629 | 0.9725093159 |
| lsm8 | 0.02683920958 | 0.1547751874 | 0.173407702 | 0.8623309715 | 1.027202625 |
| cdcp1 | 0.02844738551 | 0.1641026267 | 0.1733511893 | 0.8623753894 | 1.028855877 |
| robo4 | 0.02618711928 | 0.1514940431 | 0.1728590692 | 0.8627622053 | 1.026533015 |
| mertk | -0.026019493 | 0.150703217 | -0.172653864 | 0.8629235104 | 0.9743160974 |
| yars1 | 0.02664055348 | 0.1545319942 | 0.172395067 | 0.8631269501 | 1.026998585 |
| serpini2 | -0.026224352 | 0.1524408221 | -0.172029721 | 0.8634141633 | 0.97411652 |
| lilrb5 | 0.02449418159 | 0.1425145047 | 0.1718714992 | 0.8635385534 | 1.024796628 |
| gtpbp2 | 0.02738984489 | 0.1597779163 | 0.1714244717 | 0.8638900139 | 1.027768395 |
| ren | 0.02462695805 | 0.1450436097 | 0.1697900246 | 0.8651752723 | 1.024932706 |
| aspscr1 | 0.02648080263 | 0.1566219745 | 0.1690746315 | 0.8657379387 | 1.026834535 |
| pxdnl | 0.02574447672 | 0.1523900924 | 0.1689379953 | 0.8658454127 | 1.026078728 |
| egf | 0.02396662199 | 0.1418722281 | 0.1689310326 | 0.8658508895 | 1.02425613 |
| serpinb1 | 0.02436460919 | 0.1447971123 | 0.1682672313 | 0.8663730519 | 1.024663852 |
| cd274 | 0.02509237561 | 0.149374355 | 0.1679831562 | 0.8665965302 | 1.025409839 |
| ung | 0.02619438533 | 0.1561177343 | 0.1677860971 | 0.8667515604 | 1.026540473 |
| tbcc | 0.02413515429 | 0.144655985 | 0.166845183 | 0.867491866 | 1.024428764 |
| ca6 | 0.02626776411 | 0.1578168013 | 0.1664446617 | 0.8678070291 | 1.026615803 |
| stoml2 | -0.026536043 | 0.1594734429 | -0.166397879 | 0.8678438432 | 0.9738129444 |
| acy1 | -0.027088875 | 0.163243147 | -0.165941879 | 0.8682026897 | 0.9732747383 |
| vwf | 0.02418083404 | 0.1458340678 | 0.1658105983 | 0.8683060053 | 1.024475561 |
| ces3 | 0.02460130845 | 0.1487337603 | 0.1654050055 | 0.8686252141 | 1.024906418 |
| sarg | 0.02514460502 | 0.1520392776 | 0.1653822974 | 0.8686430864 | 1.025463397 |
| amot | 0.02942452186 | 0.1790004672 | 0.1643823746 | 0.8694301378 | 1.0298617 |
| tlr2 | -0.025056289 | 0.1532729415 | -0.163474967 | 0.8701444818 | 0.9752550144 |
| edar | -0.024668943 | 0.1518102751 | -0.162498506 | 0.8709133046 | 0.9756328487 |
| lefty2 | -0.025130956 | 0.1549892424 | -0.162146454 | 0.8711905254 | 0.9751821977 |
| wars | -0.023403493 | 0.144850998 | -0.161569432 | 0.8716449308 | 0.9768682443 |
| ppib | 0.02291466715 | 0.141969775 | 0.1614052509 | 0.8717742313 | 1.023179225 |
| morf4l1 | -0.025689079 | 0.1593249723 | -0.161236991 | 0.8719067477 | 0.9746380778 |
| egflam | -0.024858272 | 0.1542856579 | -0.161118491 | 0.8720000767 | 0.9754481502 |
| rspo1 | 0.02465317501 | 0.1531751002 | 0.160947667 | 0.8721346189 | 1.024959577 |
| slc44a4 | 0.02554664157 | 0.1594092344 | 0.1602582288 | 0.8726776621 | 1.025875754 |
| gbp6 | -0.02550446 | 0.1592859105 | -0.160117487 | 0.8727885264 | 0.9748180316 |
| tlr3 | 0.02300802898 | 0.1442754932 | 0.1594728839 | 0.8732963205 | 1.023274755 |
| cetn2 | 0.02330547627 | 0.1464732909 | 0.1591107575 | 0.8735816129 | 1.023579171 |
| sox9 | 0.02477634629 | 0.1571560848 | 0.1576543875 | 0.8747291444 | 1.025085831 |
| bdnf | 0.02421975597 | 0.1536782652 | 0.1576003993 | 0.8747716889 | 1.024515437 |
| art5 | 0.02420607854 | 0.1540604042 | 0.1571206999 | 0.875149724 | 1.024501424 |
| pcbp2 | 0.02423560438 | 0.1546996302 | 0.1566623291 | 0.8755109775 | 1.024531674 |
| apoh | 0.02211220808 | 0.1417172271 | 0.1560304879 | 0.8760089897 | 1.022358495 |
| hsd17b14 | -0.023755744 | 0.1522858052 | -0.155994471 | 0.8760373794 | 0.9765242029 |
| cox5b | 0.02253091767 | 0.1457833341 | 0.1545507092 | 0.8771755313 | 1.022786656 |
| col4a4 | -0.023983219 | 0.1552819942 | -0.154449451 | 0.877255365 | 0.9763020932 |
| manf | 0.02201729312 | 0.142621982 | 0.1543751728 | 0.8773139282 | 1.022261462 |
| asrgl1 | -0.023566656 | 0.1532053499 | -0.153823979 | 0.8777485266 | 0.9767088685 |
| nppc | 0.02646786138 | 0.1720765315 | 0.1538144751 | 0.8777560201 | 1.026821246 |
| hla_a | -0.024186773 | 0.1574400189 | -0.153625318 | 0.8779051731 | 0.976103383 |
| neo1 | -0.024198293 | 0.1576490188 | -0.153494726 | 0.8780081492 | 0.9760921384 |
| lto1 | 0.02240917153 | 0.1460143939 | 0.1534723456 | 0.8780257969 | 1.022662143 |
| fen1 | 0.02220221959 | 0.1447568554 | 0.1533759456 | 0.8781018131 | 1.022450523 |
| clec4a | 0.02337041747 | 0.1523738955 | 0.1533754676 | 0.87810219 | 1.023645646 |
| ifit3 | -0.024331083 | 0.1588020278 | -0.153216452 | 0.8782275837 | 0.9759625313 |
| gabra4 | -0.023392222 | 0.1526928107 | -0.153197925 | 0.8782421937 | 0.9768792553 |
| lyzl2 | 0.028309109 | 0.1848116136 | 0.1531781929 | 0.8782577542 | 1.02871362 |
| mmut | 0.0247072578 | 0.1614007831 | 0.1530801606 | 0.878335061 | 1.025015011 |
| scn3a | 0.02427372845 | 0.1586864285 | 0.1529666316 | 0.8784245897 | 1.024570734 |
| cd83 | -0.023809378 | 0.1557084205 | -0.15291002 | 0.8784692337 | 0.9764718293 |
| map4k5 | -0.021909681 | 0.1433518564 | -0.152838487 | 0.8785256464 | 0.9783285929 |
| epha2 | 0.02387312011 | 0.156696875 | 0.1523522412 | 0.8789091232 | 1.024160364 |
| mybpc2 | -0.024698916 | 0.1623747966 | -0.152110526 | 0.8790997621 | 0.9756036067 |
| magea3 | -0.02387399 | 0.1570365296 | -0.152028256 | 0.8791646495 | 0.9764087395 |
| cpb1 | 0.02130550543 | 0.1408557165 | 0.1512576554 | 0.879772471 | 1.021534088 |
| casp1 | -0.022167753 | 0.1472393163 | -0.150555935 | 0.8803260241 | 0.9780761462 |
| gal | -0.022427651 | 0.1491531823 | -0.150366563 | 0.8804754202 | 0.9778219787 |
| ncan | -0.024028831 | 0.1598216435 | -0.15034779 | 0.8804902302 | 0.9762575629 |
| bglap | -0.023413591 | 0.1558691739 | -0.150213097 | 0.8805964932 | 0.97685838 |
| col9a2 | -0.023389073 | 0.1569093314 | -0.149061073 | 0.8815054418 | 0.976882331 |
| tp53i3 | 0.02407499102 | 0.1618110241 | 0.1487846156 | 0.8817235905 | 1.024367133 |
| krt5 | -0.022871753 | 0.1540623181 | -0.148457804 | 0.8819814841 | 0.9773878223 |

| Protein | beta | se | z | p_val | HR |
|---|---|---|---|---|---|
| klrk1 | -0.022334735 | 0.1505712479 | -0.148333335 | 0.8820797086 | 0.9779128382 |
| dlg4 | -0.022210175 | 0.1499527921 | -0.148114447 | 0.8822524478 | 0.9780346551 |
| cfhr5 | 0.02275015867 | 0.1536585506 | 0.1480565747 | 0.88229812 | 1.023010917 |
| hnrnpk | 0.0216817316 | 0.1468696599 | 0.1476256676 | 0.8826381973 | 1.021918488 |
| mrc1 | -0.024381502 | 0.1656093495 | -0.147222982 | 0.8829560213 | 0.9759133256 |
| vps37a | -0.021026216 | 0.1433593151 | -0.146667947 | 0.8833941199 | 0.9791932934 |
| cenpj | -0.023172002 | 0.1581666625 | -0.146503704 | 0.8835237661 | 0.9770944072 |
| nptxr | 0.02160698083 | 0.1477987395 | 0.1461919154 | 0.8837698875 | 1.021842102 |
| entpd6 | 0.02059099622 | 0.1413067875 | 0.1457183804 | 0.8841437108 | 1.020804453 |
| cthrc1 | 0.02615256743 | 0.1799490868 | 0.1453331489 | 0.8844478435 | 1.026497547 |
| pbk | 0.02294924098 | 0.1582593229 | 0.1450103575 | 0.8847026942 | 1.023214601 |
| cd58 | 0.02083063209 | 0.1437954528 | 0.1448629402 | 0.8848190871 | 1.021049104 |
| flt1 | 0.02126983371 | 0.147047117 | 0.1446463837 | 0.8849900734 | 1.021497649 |
| sema3g | -0.022944847 | 0.1589821816 | -0.144323387 | 0.8852451117 | 0.9773163843 |
| myl3 | -0.024403689 | 0.1697316642 | -0.143778058 | 0.8856757302 | 0.9758916735 |
| ces2 | -0.021866927 | 0.152196588 | -0.143675543 | 0.8857566845 | 0.9783704206 |
| fabp2 | 0.02045168696 | 0.1424212667 | 0.1435999513 | 0.8858163791 | 1.020662256 |
| tpsab1 | -0.020703831 | 0.1442965966 | -0.143481075 | 0.885910257 | 0.9795090221 |
| fuom | -0.024374658 | 0.1709419966 | -0.142590229 | 0.8866138146 | 0.9759200046 |
| blmh | 0.02077134426 | 0.146483436 | 0.1417999524 | 0.8872380216 | 1.02098857 |
| cdhr5 | 0.02088964874 | 0.1476591053 | 0.1414721341 | 0.8874969724 | 1.021109365 |
| frmd7 | 0.02245766931 | 0.1591337824 | 0.1411244612 | 0.8877716198 | 1.022711741 |
| cpped1 | 0.02103772163 | 0.1495127173 | 0.1407085765 | 0.8881001694 | 1.021260575 |
| sirt5 | 0.02011366276 | 0.143041755 | 0.1406139261 | 0.8881749461 | 1.020317306 |
| cdc26 | -0.021863133 | 0.1555190958 | -0.140581665 | 0.8882004339 | 0.9783741327 |
| nudt5 | 0.02065873479 | 0.1469630237 | 0.14057097 | 0.8882088831 | 1.020873604 |
| fxyd5 | -0.020089787 | 0.143738541 | -0.139766178 | 0.8888447369 | 0.9801106686 |
| top2b | 0.02223822918 | 0.1593804346 | 0.1395292291 | 0.8890319603 | 1.022487342 |
| ldlrap1 | 0.02144368419 | 0.1538387173 | 0.139390685 | 0.8891414328 | 1.021675252 |
| nphs1 | 0.02190104118 | 0.1573371393 | 0.1391981657 | 0.889293558 | 1.022142629 |
| draxin | 0.02011811195 | 0.1449895071 | 0.1387556407 | 0.8896432484 | 1.020321845 |
| hmox1 | -0.020438894 | 0.1474960304 | -0.138572505 | 0.8897879718 | 0.979768564 |
| lgals8 | -0.020283119 | 0.1464400201 | -0.138508029 | 0.8898389242 | 0.9799212001 |
| ssb | 0.02019744786 | 0.145849853 | 0.1384810985 | 0.8898602069 | 1.020402796 |
| cd36 | -0.022228718 | 0.1614549278 | -0.137677546 | 0.8904952664 | 0.9780165193 |
| fkbp5 | -0.019795748 | 0.1439833357 | -0.137486384 | 0.8906463546 | 0.9803989011 |
| clec6a | 0.02030392224 | 0.1479289377 | 0.1372545667 | 0.890829581 | 1.020511449 |
| erc2 | -0.021592706 | 0.1576611746 | -0.136956396 | 0.8910652609 | 0.9786387473 |
| lgals9 | -0.022703558 | 0.1662442751 | -0.13656746 | 0.8913726986 | 0.977552228 |
| dnajc21 | 0.02041748239 | 0.1499079795 | 0.1362001039 | 0.891663093 | 1.020627345 |
| icam3 | -0.019465973 | 0.1429255738 | -0.136196571 | 0.8916658856 | 0.9807222656 |
| usp8 | 0.0201354834 | 0.1479351835 | 0.1361101729 | 0.8917341856 | 1.02033957 |
| c9 | -0.020809968 | 0.1545264754 | -0.134669271 | 0.8928733701 | 0.9794050654 |
| gip | 0.02100169929 | 0.1574584886 | 0.1333792765 | 0.8938934339 | 1.021223787 |
| rnf5 | 0.02027675186 | 0.1521565371 | 0.133262443 | 0.8939858288 | 1.020483722 |
| enox2 | 0.02104563481 | 0.1580641456 | 0.133146165 | 0.8940777858 | 1.021268656 |
| erbb3 | 0.02039700279 | 0.1545663942 | 0.1319627264 | 0.8950137739 | 1.020606443 |
| samd9l | 0.02022422604 | 0.1532571197 | 0.1319627178 | 0.8950137807 | 1.020430121 |
| itgb1bp2 | 0.01923050514 | 0.1457780702 | 0.1319163103 | 0.8950504876 | 1.019416602 |
| fabp5 | -0.019991479 | 0.1517911441 | -0.131703859 | 0.8952185331 | 0.9802070252 |
| slamf8 | 0.01873493977 | 0.1423011288 | 0.1316570004 | 0.8952555981 | 1.01891154 |
| ckmt1a_ckmt1b | -0.018599352 | 0.1415373671 | -0.131409484 | 0.8954513865 | 0.9815725482 |
| vps28 | -0.020953296 | 0.1597591757 | -0.13115551 | 0.8956522901 | 0.979264699 |
| prkag3 | 0.02086346915 | 0.1591560119 | 0.1310881625 | 0.8957055653 | 1.021082633 |
| tacc3 | -0.018807712 | 0.1437155127 | -0.130867654 | 0.8958800026 | 0.9813680494 |
| lipf | 0.02019881464 | 0.1550230982 | 0.1302955165 | 0.8963326271 | 1.020404191 |
| sema4d | 0.01877335178 | 0.1446240525 | 0.1298079501 | 0.8967183729 | 1.018950679 |
| mesd | 0.01844535459 | 0.1431531293 | 0.1288505161 | 0.8974759326 | 1.018616521 |
| ada2 | -0.018791362 | 0.1458576863 | -0.128833539 | 0.8974893665 | 0.981384095 |
| lrp1 | 0.01968752518 | 0.152976188 | 0.1286966648 | 0.8975976746 | 1.019882603 |
| pten | -0.01866195 | 0.1457025541 | -0.128082516 | 0.898083672 | 0.9815111062 |
| gtf2ird1 | -0.020252425 | 0.158150087 | -0.128058263 | 0.8981028655 | 0.9799512775 |
| gmpr | -0.018412199 | 0.1444157622 | -0.127494387 | 0.8985491151 | 0.9817562699 |
| tprkb | 0.02040836344 | 0.1604674806 | 0.1271806809 | 0.8987973956 | 1.020618038 |
| il4r | 0.01961082544 | 0.1542908416 | 0.1271029779 | 0.8988588945 | 1.019804381 |
| cuzd1 | -0.02024891 | 0.1603965661 | -0.126242787 | 0.8995397433 | 0.9799547229 |
| ppif | 0.01925487575 | 0.1526849432 | 0.1261085432 | 0.899646005 | 1.019441446 |
| cd33 | -0.018065019 | 0.1435714476 | -0.125825985 | 0.899869672 | 0.9820971755 |
| sag | 0.01926347835 | 0.1560968075 | 0.1234072539 | 0.9017846132 | 1.019450216 |
| pilra | 0.01816224534 | 0.1471813517 | 0.1234004521 | 0.9017899991 | 1.018328182 |

| Protein | beta | se | z | p_val | HR |
|---|---|---|---|---|---|
| ca11 | -0.017740935 | 0.1439814071 | -0.123216845 | 0.9019353869 | 0.9824155092 |
| bcl7b | 0.01906425051 | 0.15491924 | 0.1230592824 | 0.9020601541 | 1.019247134 |
| hspa1a | 0.01790415206 | 0.1471920678 | 0.1216380225 | 0.9031856995 | 1.018065392 |
| chit1 | 0.0176510847 | 0.1455616645 | 0.1212619048 | 0.9034835929 | 1.017807786 |
| rnase10 | 0.01835484778 | 0.1524182585 | 0.1204242062 | 0.9041471172 | 1.018524333 |
| mitd1 | 0.01731391472 | 0.1439599686 | 0.1202689532 | 0.9042700973 | 1.017464669 |
| klkb1 | -0.019546076 | 0.1626849682 | -0.120146786 | 0.9043668707 | 0.9806437099 |
| gp6 | -0.017198334 | 0.1435281828 | -0.119825485 | 0.9046213932 | 0.982948713 |
| gmpr2 | 0.01883973429 | 0.1575853835 | 0.1195525491 | 0.9048376104 | 1.019018322 |
| pvalb | 0.01686492791 | 0.1411326474 | 0.1194969996 | 0.904881617 | 1.017007944 |
| sap18 | -0.018715534 | 0.1566393976 | -0.119481652 | 0.9048937755 | 0.9814585141 |
| cd300c | -0.017649202 | 0.1479938703 | -0.119256303 | 0.9050723012 | 0.982505633 |
| nck2 | -0.0167801 | 0.1414339494 | -0.118642659 | 0.9055584666 | 0.9833599018 |
| oxct1 | 0.01805744043 | 0.1525552554 | 0.1183665577 | 0.9057772226 | 1.018221462 |
| sult1a1 | 0.01686105528 | 0.1427420295 | 0.1181225693 | 0.9059705409 | 1.017004005 |
| aoc3 | -0.0176952 | 0.1501611277 | -0.117841419 | 0.9061933103 | 0.9824604403 |
| il10 | 0.01652815937 | 0.1403495275 | 0.117764268 | 0.9062544425 | 1.016665505 |
| cdkn1a | -0.017039678 | 0.144840619 | -0.117644336 | 0.9063494737 | 0.9831046758 |
| suox | 0.0195341741 | 0.1663498933 | 0.1174282334 | 0.9065207118 | 1.019726214 |
| il9 | -0.018333588 | 0.157159842 | -0.116655681 | 0.907132912 | 0.9818334495 |
| sfrp4 | -0.019336166 | 0.1658543037 | -0.11658525 | 0.9071887265 | 0.980849579 |
| pltp | 0.01733281933 | 0.1507687973 | 0.1149629078 | 0.9084745224 | 1.017483904 |
| sez6l | 0.01758501137 | 0.1534839438 | 0.1145723189 | 0.9087841215 | 1.017740538 |
| vnn1 | -0.018430807 | 0.1612373152 | -0.114308568 | 0.9089931906 | 0.981738002 |
| ment | -0.019345287 | 0.1694863096 | -0.114140703 | 0.9091262564 | 0.9808406326 |
| trem2 | -0.017838455 | 0.1565981245 | -0.113912313 | 0.9093073044 | 0.9823197087 |
| tnfrsf8 | 0.01672267927 | 0.1468899319 | 0.1138449657 | 0.9093606928 | 1.016863286 |
| rnf41 | -0.016551235 | 0.1465444967 | -0.112943412 | 0.9100754186 | 0.9835849836 |
| tp53 | 0.01653853302 | 0.14669927 | 0.1127376641 | 0.91023854 | 1.016676052 |
| il22ra1 | 0.01649981537 | 0.1467721521 | 0.112417888 | 0.9104920726 | 1.016636689 |
| cd244 | -0.015935252 | 0.1419495131 | -0.112259997 | 0.9106172589 | 0.9841910425 |
| vamp8 | 0.01730177742 | 0.1545669819 | 0.1119370852 | 0.9108732917 | 1.01745232 |
| nptx1 | -0.016918223 | 0.1515528326 | -0.111632509 | 0.9111147947 | 0.9832240865 |
| sptbn2 | 0.01705424276 | 0.1533482928 | 0.1112124723 | 0.9114478616 | 1.017200497 |
| cd101 | 0.01703905078 | 0.1547620964 | 0.1100983456 | 0.9123313802 | 1.017185043 |
| pi16 | -0.016954751 | 0.1543259375 | -0.109863262 | 0.912517819 | 0.983188172 |
| top1 | 0.01690257475 | 0.1539503845 | 0.1097923516 | 0.9125740569 | 1.017046232 |
| ufd1 | -0.016946123 | 0.1548780562 | -0.109415905 | 0.9128726193 | 0.9831966552 |
| crlf1 | 0.01670726509 | 0.1529554374 | 0.1092296252 | 0.9130203634 | 1.016847612 |
| dkk3 | 0.01657662647 | 0.152414565 | 0.1087601206 | 0.9133927553 | 1.016714781 |
| tdo2 | -0.017301288 | 0.1598985454 | -0.108201657 | 0.9138357305 | 0.9828475203 |
| il18rap | -0.015413912 | 0.1428580287 | -0.107896716 | 0.9140776219 | 0.9847042741 |
| pm20d1 | -0.015611646 | 0.1447512785 | -0.107851524 | 0.9141134711 | 0.9845095841 |
| nadk | 0.01588506172 | 0.1485466949 | 0.1069364871 | 0.9148393666 | 1.0160119 |
| dcxr | 0.01676395282 | 0.1578361897 | 0.1062110841 | 0.9154148769 | 1.016905256 |
| ltb | -0.016688781 | 0.1576152319 | -0.105883048 | 0.9156751445 | 0.9834497051 |
| sat2 | 0.01706734305 | 0.1617304411 | 0.1055295647 | 0.9159556117 | 1.017213822 |
| hagh | 0.01554829239 | 0.147927282 | 0.1051076731 | 0.9162903708 | 1.015669796 |
| csf2rb | 0.01652199364 | 0.1576098371 | 0.1048284419 | 0.916511941 | 1.016659237 |
| hpcal1 | 0.01500854349 | 0.1432337344 | 0.1047835801 | 0.9165475395 | 1.015121737 |
| galnt2 | -0.014918595 | 0.1429528308 | -0.104360261 | 0.9168834573 | 0.9851921361 |
| cd300lf | 0.01468451384 | 0.141148307 | 0.1040360607 | 0.9171407315 | 1.014792861 |
| rilp | -0.014852115 | 0.1430362137 | -0.103834649 | 0.9173005695 | 0.9852576337 |
| il15ra | 0.01518528379 | 0.1467029382 | 0.1035104271 | 0.9175578744 | 1.015301166 |
| ca3 | 0.01558379407 | 0.1515312624 | 0.1028421055 | 0.918088287 | 1.015705855 |
| rrm2 | 0.01471290021 | 0.1450971302 | 0.1014003529 | 0.9192326563 | 1.014821668 |
| trim5 | -0.015182157 | 0.1516630513 | -0.100104519 | 0.9202613479 | 0.9849325111 |
| msmb | -0.01509636 | 0.151686349 | -0.099523524 | 0.9207226112 | 0.9850170188 |
| tnfsf14 | 0.01460396658 | 0.1472371757 | 0.09918667963 | 0.9209900508 | 1.014711126 |
| mrpl28 | -0.015150269 | 0.1536442814 | -0.098606135 | 0.9214509984 | 0.9849639191 |
| rida | 0.01710648943 | 0.1736835884 | 0.09849226166 | 0.9215414164 | 1.017253643 |
| atp1b1 | -0.015535691 | 0.1585081432 | -0.098011943 | 0.9219228096 | 0.9845843652 |
| tmem132a | -0.014726497 | 0.1503804651 | -0.097928257 | 0.9219892618 | 0.9853814077 |
| ttf2 | 0.0155072197 | 0.1590961751 | 0.09747072605 | 0.9223525807 | 1.015628081 |
| robo1 | 0.01431864843 | 0.1469532343 | 0.09743676959 | 0.9223795457 | 1.014421651 |
| denr | -0.01532756 | 0.1574534198 | -0.097346631 | 0.9224511255 | 0.9847893092 |
| grhpr | -0.01507779 | 0.1552398277 | -0.097125784 | 0.9226265053 | 0.9850353108 |
| gla | 0.01661015046 | 0.1714366022 | 0.0968880055 | 0.9228153342 | 1.016748866 |
| clec1a | 0.01421116557 | 0.1478188677 | 0.0961390504 | 0.9234101372 | 1.014312624 |
| rbpms2 | 0.01457226033 | 0.1515827307 | 0.09613404024 | 0.9234141163 | 1.014678953 |

| Protein | beta | se | z | p_val | HR |
|---|---|---|---|---|---|
| pla2g1b | 0.01371412995 | 0.1428081511 | 0.09603184301 | 0.9234952824 | 1.0138086 |
| cwc15 | 0.01558558972 | 0.1624511313 | 0.09594017349 | 0.9235680879 | 1.015707678 |
| naa10 | -0.01509733 | 0.1582025445 | -0.095430386 | 0.9239729818 | 0.9850160635 |
| il5ra | 0.01422765261 | 0.1495625801 | 0.09512842452 | 0.9242128209 | 1.014329347 |
| rab11fip3 | 0.01420589088 | 0.1510628761 | 0.09403958965 | 0.9250777081 | 1.014307274 |
| hk2 | -0.01385342 | 0.1477019127 | -0.093793096 | 0.9252735161 | 0.9862420973 |
| tnxb | -0.013620318 | 0.1455008389 | -0.093609893 | 0.9254190505 | 0.9864720188 |
| vegfa | 0.01406545157 | 0.1505118119 | 0.09345081553 | 0.9255454221 | 1.014164835 |
| lhpp | 0.01380254975 | 0.1478509659 | 0.093354478 | 0.9256219538 | 1.013898245 |
| crkl | -0.01329521 | 0.1427742386 | -0.093120513 | 0.9258078215 | 0.9867927806 |
| cebpa | 0.01470884229 | 0.1582088092 | 0.09297107007 | 0.9259265444 | 1.01481755 |
| fam172a | -0.014136413 | 0.1530647591 | -0.092355768 | 0.9264153812 | 0.9859630365 |
| tbl1x | -0.013217038 | 0.1436476873 | -0.092010099 | 0.9266900157 | 0.9868699236 |
| mapk13 | 0.01478518634 | 0.1610657871 | 0.09179594626 | 0.9268601649 | 1.014895028 |
| psmg4 | -0.014593913 | 0.1595795626 | -0.091452269 | 0.9271332308 | 0.9855120619 |
| fkbpl | 0.01430691266 | 0.1570326485 | 0.09110788615 | 0.9274068665 | 1.014409746 |
| mslnl | 0.01436393662 | 0.1588958225 | 0.09039845351 | 0.9279705856 | 1.014467594 |
| tarbp2 | 0.0132574614 | 0.1473603795 | 0.0899662545 | 0.9283140311 | 1.013345731 |
| dgka | 0.01385350878 | 0.1543776142 | 0.08973780851 | 0.9284955703 | 1.013949913 |
| col3a1 | 0.01381560661 | 0.1541417214 | 0.08962924826 | 0.9285818412 | 1.013911483 |
| naaa | -0.012676031 | 0.1418373228 | -0.089370206 | 0.9287877006 | 0.9874039717 |
| tshb | 0.01263033964 | 0.1415619136 | 0.08922131181 | 0.9289060285 | 1.012710439 |
| timm8a | -0.013516682 | 0.1524983178 | -0.088634957 | 0.9293720254 | 0.9865742582 |
| cst6 | -0.013064595 | 0.1474656002 | -0.088594188 | 0.9294044269 | 0.9870203762 |
| thbd | -0.013043089 | 0.1479896936 | -0.088135119 | 0.9297692839 | 0.9870416032 |
| robo2 | -0.013232974 | 0.1518993942 | -0.087116698 | 0.9305787526 | 0.9868541972 |
| serpina12 | 0.0132729775 | 0.1540322149 | 0.08617013982 | 0.9313311674 | 1.013361454 |
| ddx53 | -0.013718055 | 0.1595730966 | -0.085967215 | 0.931492479 | 0.986375609 |
| ddx1 | 0.01305444719 | 0.1536865778 | 0.08494201237 | 0.9323074914 | 1.013140028 |
| itprip | -0.012925498 | 0.1550230954 | -0.083377889 | 0.9335510692 | 0.987157677 |
| cfhr2 | -0.012543785 | 0.1510525773 | -0.083042506 | 0.9338177413 | 0.9875345608 |
| prkar2a | -0.01254159 | 0.1538098862 | -0.081539558 | 0.9350128667 | 0.9875367278 |
| asgr2 | -0.01286574 | 0.1582000199 | -0.081325781 | 0.9351828712 | 0.9872166696 |
| ogt | 0.01281599269 | 0.1577925216 | 0.08122053286 | 0.9352665706 | 1.012898469 |
| mbl2 | 0.01242407356 | 0.1553255105 | 0.07998733448 | 0.9362473293 | 1.012501573 |
| psme1 | 0.01186514129 | 0.1497125719 | 0.07925280513 | 0.9368315442 | 1.011935811 |
| lilra4 | 0.01247290018 | 0.1576112685 | 0.0791371093 | 0.9369235671 | 1.012551011 |
| max | 0.01130879249 | 0.1429597309 | 0.07910474104 | 0.9369493125 | 1.011372979 |
| maneal | 0.01259552183 | 0.1597365937 | 0.07885182435 | 0.9371504825 | 1.01267518 |
| atp5if1 | -0.011173923 | 0.1417437727 | -0.078831843 | 0.9371663756 | 0.9888882735 |
| carhsp1 | 0.01175779127 | 0.1497609614 | 0.07851038855 | 0.9374220669 | 1.011827186 |
| c1qtnf6 | -0.012244235 | 0.1585520733 | -0.077225324 | 0.9384442964 | 0.9878304204 |
| fcgr2a | -0.010872427 | 0.1408621128 | -0.077184892 | 0.93847646 | 0.9891864642 |
| zfyve19 | -0.01194772 | 0.1550754827 | -0.077044547 | 0.9385881067 | 0.9881233703 |
| dok2 | 0.0109377864 | 0.1428481011 | 0.07656935105 | 0.9389661417 | 1.010997823 |
| lamp1 | -0.012690344 | 0.166128482 | -0.076388731 | 0.9391098347 | 0.9873898389 |
| adamts1 | 0.01223933774 | 0.1607254023 | 0.07615061196 | 0.9392992745 | 1.012314545 |
| nptn | 0.01103800244 | 0.1450333344 | 0.07610665839 | 0.9393342429 | 1.011099146 |
| grap2 | -0.010757769 | 0.1418858111 | -0.075819906 | 0.9395623786 | 0.9892998889 |
| igf1r | -0.011263506 | 0.1488732162 | -0.075658381 | 0.9396908884 | 0.9887996893 |
| susd1 | 0.01084235118 | 0.1453219087 | 0.07460919878 | 0.940525655 | 1.010901342 |
| tmco5a | -0.012088958 | 0.1621123306 | -0.074571489 | 0.9405556591 | 0.9879838199 |
| erp44 | -0.011101821 | 0.1489843505 | -0.074516691 | 0.9405992607 | 0.988959577 |
| nt5c3a | -0.011139488 | 0.1497784524 | -0.074373102 | 0.9407135111 | 0.9889223263 |
| nt5c | 0.01131751268 | 0.1531507805 | 0.07389784523 | 0.9410916704 | 1.011381798 |
| il20ra | 0.01069163746 | 0.145360836 | 0.07355239385 | 0.9413665527 | 1.010748997 |
| unc79 | -0.011933869 | 0.1623532972 | -0.073505556 | 0.9414038232 | 0.9881370568 |
| tia1 | -0.010699962 | 0.1456734211 | -0.07345171 | 0.9414466703 | 0.9893570791 |
| tf | -0.011310549 | 0.1563907669 | -0.07232236 | 0.9423453708 | 0.9887531745 |
| abo | 0.01110216217 | 0.1537688861 | 0.07220031599 | 0.9424424937 | 1.01116402 |
| prdx3 | 0.01036960955 | 0.1438576424 | 0.07208243776 | 0.9425363025 | 1.01042356 |
| tnfrsf12a | 0.01149685745 | 0.1603219492 | 0.07171106328 | 0.9428318516 | 1.0115632 |
| prkar1a | -0.010121459 | 0.1433397028 | -0.070611694 | 0.9437068033 | 0.9899295904 |
| mybpc1 | 0.01203544257 | 0.1712817127 | 0.07026694434 | 0.9439811921 | 1.01210816 |
| pithd1 | 0.01104729767 | 0.1573537906 | 0.07020674637 | 0.9440291048 | 1.011108544 |
| klrd1 | 0.01035251266 | 0.1474891726 | 0.07019167899 | 0.9440410972 | 1.010406285 |
| ggct | -0.010607913 | 0.1525566362 | -0.069534264 | 0.9445643601 | 0.9894481521 |
| rbp2 | 0.01017653263 | 0.146754261 | 0.06934403512 | 0.9447157751 | 1.01022849 |
| ngf | -0.009963435 | 0.1450451667 | -0.068691947 | 0.9452348282 | 0.9900860356 |
| cxcl16 | -0.010203661 | 0.1487805189 | -0.068581969 | 0.9453223717 | 0.9898482198 |

| Protein | beta | se | z | p_val | HR |
|---|---|---|---|---|---|
| otud6b | -0.01060641 | 0.1556425122 | -0.068145969 | 0.9456694377 | 0.9894496399 |
| pafah1b3 | -0.010647456 | 0.1569253068 | -0.067850469 | 0.9459046682 | 0.9894090279 |
| evi5 | 0.0105906065 | 0.156133204 | 0.06783058457 | 0.9459204969 | 1.010646885 |
| ncam1 | -0.010286642 | 0.1521356625 | -0.06761493 | 0.9460921704 | 0.9897660844 |
| snu13 | 0.01083862937 | 0.1621305497 | 0.06685124666 | 0.9467001257 | 1.01089758 |
| pklr | -0.009547939 | 0.1432176241 | -0.066667349 | 0.9468465283 | 0.9904974975 |
| dnaja4 | 0.01014801139 | 0.1537228055 | 0.06601500253 | 0.9473658812 | 1.010199677 |
| gp1bb | -0.010337511 | 0.1584444719 | -0.065243746 | 0.9479799314 | 0.9897157376 |
| itgb5 | 0.0094506656 | 0.1457861315 | 0.06482554618 | 0.9483129013 | 1.009495464 |
| apoa1 | -0.010785485 | 0.1664690512 | -0.06478973 | 0.9483414188 | 0.98927247 |
| fcrl6 | 0.009698207305 | 0.1504729858 | 0.06445148447 | 0.9486107365 | 1.009745387 |
| idi2 | -0.0097819 | 0.1522554696 | -0.064246623 | 0.9487738544 | 0.9902657874 |
| nudt16 | -0.009906685 | 0.1545237321 | -0.06411109 | 0.9488817713 | 0.9901422246 |
| bola2_bola2b | -0.009987896 | 0.1560534743 | -0.064003036 | 0.9489678093 | 0.9900618172 |
| mcee | 0.00994922542 | 0.1555317687 | 0.06396908813 | 0.9489948405 | 1.009998884 |
| arhgap30 | 0.0101548363 | 0.1610092761 | 0.0630698836 | 0.9497108559 | 1.010206572 |
| ccl28 | -0.009785522 | 0.1568513144 | -0.062387252 | 0.9502544469 | 0.9902622 |
| nek7 | -0.009596 | 0.1550526413 | -0.061888658 | 0.9506515002 | 0.9904498949 |
| serpinb6 | 0.008943896002 | 0.1465266275 | 0.06103939029 | 0.9513278386 | 1.008984012 |
| glrx5 | 0.009630648207 | 0.1586580034 | 0.06070067693 | 0.9515975926 | 1.009677172 |
| serpina4 | -0.00932032 | 0.1543538605 | -0.060382812 | 0.9518507477 | 0.9907229794 |
| msln | -0.008993746 | 0.1493462855 | -0.060220754 | 0.9519798166 | 0.9910465769 |
| rab6a | -0.009324897 | 0.1555448931 | -0.059949874 | 0.9521955577 | 0.9907184453 |
| bcl2l1 | -0.009025431 | 0.151189097 | -0.059696309 | 0.9523975118 | 0.991015176 |
| cd99l2 | 0.008973835471 | 0.1522275287 | 0.05895014882 | 0.9529918145 | 1.009014221 |
| serpina7 | -0.008944039 | 0.1526813505 | -0.058579776 | 0.9532868198 | 0.9910958397 |
| gc | 0.009050814742 | 0.1548768641 | 0.05843877842 | 0.9533991268 | 1.009091897 |
| pik3ap1 | -0.008818285 | 0.1514780165 | -0.058214947 | 0.9535774151 | 0.9912204824 |
| ervv_1 | 0.009191417511 | 0.1586530021 | 0.05793409132 | 0.9538011278 | 1.009233788 |
| map2k6 | -0.00868312 | 0.150470109 | -0.057706613 | 0.9539823262 | 0.9913544691 |
| atxn2 | 0.008597488234 | 0.151403033 | 0.05678544255 | 0.9547161104 | 1.008634553 |
| tnfsf13b | 0.008204518268 | 0.1466199942 | 0.05595770421 | 0.9553755015 | 1.008238268 |
| cirbp | 0.008642379545 | 0.1552239118 | 0.05567685701 | 0.9555992363 | 1.008679833 |
| dnlz | 0.008269013187 | 0.1522851904 | 0.05429952291 | 0.9566965297 | 1.008303296 |
| amy2a | -0.007766637 | 0.1447081579 | -0.053671035 | 0.9571972601 | 0.9922634458 |
| cplx2 | 0.008525080728 | 0.1612238195 | 0.05287730284 | 0.9578296688 | 1.008561523 |
| ankrd54 | -0.007593163 | 0.1437230202 | -0.052831921 | 0.9578658281 | 0.9924355921 |
| ifi30 | 0.009235474206 | 0.1761415623 | 0.05243211249 | 0.9581843872 | 1.009278253 |
| dapp1 | -0.007753617 | 0.1494621642 | -0.051876785 | 0.9586268722 | 0.9922763652 |
| timp3 | -0.00737182 | 0.1442699375 | -0.051097411 | 0.9592478991 | 0.9926552849 |
| sdccag8 | 0.007787722915 | 0.1527106307 | 0.05099659977 | 0.95932823 | 1.007818126 |
| cntn3 | 0.008296296921 | 0.1628275962 | 0.05095141803 | 0.959364233 | 1.008330807 |
| amy2b | 0.007254301265 | 0.1445319798 | 0.05019166881 | 0.9599696505 | 1.007280677 |
| irak4 | -0.00738703 | 0.1492193229 | -0.049504511 | 0.960517242 | 0.9926401874 |
| cbln1 | -0.007337187 | 0.1507301978 | -0.048677619 | 0.9611762123 | 0.9926896643 |
| clec5a | -0.007206839 | 0.1482039027 | -0.048627859 | 0.9612158678 | 0.9928190685 |
| kcnip4 | -0.007082331 | 0.1467562995 | -0.048259129 | 0.961509727 | 0.9929426894 |
| c7orf50 | -0.007343786 | 0.1533199651 | -0.047898435 | 0.9617971865 | 0.9926831133 |
| dctn2 | 0.006836370239 | 0.1434280719 | 0.04766410194 | 0.9619839441 | 1.006859792 |
| dab2 | -0.00677602 | 0.1425535546 | -0.047533157 | 0.9620883049 | 0.993246885 |
| mog | 0.007069199917 | 0.1497806928 | 0.04719700372 | 0.9623562155 | 1.007094246 |
| gimap7 | 0.007393162731 | 0.15866252 | 0.04659678121 | 0.9628345975 | 1.00742056 |
| rab2b | 0.007018414443 | 0.1516786104 | 0.04627161616 | 0.9630937621 | 1.007043101 |
| ehd3 | 0.006804789537 | 0.1502183739 | 0.04529931565 | 0.9638687329 | 1.006827995 |
| lyn | -0.006433323 | 0.1429064917 | -0.045017708 | 0.9640931944 | 0.9935873268 |
| kazn | 0.00683818528 | 0.1526023027 | 0.04481049867 | 0.9642583568 | 1.006861619 |
| fbp1 | 0.006593314671 | 0.1486770921 | 0.04434654041 | 0.9646281743 | 1.006615098 |
| gpa33 | -0.006233367 | 0.1431013649 | -0.043559102 | 0.9652558524 | 0.9937860201 |
| krt14 | 0.006266193914 | 0.1453107105 | 0.04312272572 | 0.9656037036 | 1.006285868 |
| ntprobnp | -0.006380141 | 0.1481271894 | -0.043072047 | 0.965644102 | 0.9936401686 |
| vasn | 0.006216183437 | 0.1444150488 | 0.04304387589 | 0.9656665583 | 1.006235544 |
| c5 | -0.006701777 | 0.1564811239 | -0.042828024 | 0.9658386246 | 0.9933206295 |
| palm3 | -0.006861922 | 0.160381731 | -0.042784935 | 0.9658729734 | 0.9931615673 |
| ca2 | 0.006374922433 | 0.1490520769 | 0.04276976589 | 0.9658850653 | 1.006395285 |
| adgrg1 | -0.006368085 | 0.1503204774 | -0.04236339 | 0.9662090128 | 0.9936521483 |
| tbc1d17 | 0.006142966713 | 0.1484194995 | 0.04138921593 | 0.9669856099 | 1.006161873 |
| lilra2 | 0.00604781478 | 0.1483099436 | 0.0407782151 | 0.9674727067 | 1.00606614 |
| rgcc | -0.006175821 | 0.1519190437 | -0.040652053 | 0.9675732858 | 0.9938432101 |
| brd1 | 0.006290935384 | 0.1573320811 | 0.03998507704 | 0.9681050236 | 1.006310765 |
| klk8 | -0.0060198 | 0.152254262 | -0.039537807 | 0.9684616111 | 0.993998283 |

| Protein | beta | se | z | p_val | HR |
|---|---|---|---|---|---|
| epo | 0.006037411665 | 0.1546548205 | 0.0390379792 | 0.9688601087 | 1.006055674 |
| f11 | -0.006271998 | 0.1613875156 | -0.038862968 | 0.9689996416 | 0.9937476301 |
| il13 | 0.00557314336 | 0.1448683366 | 0.03847040346 | 0.9693126286 | 1.005588702 |
| rtn4ip1 | -0.005821904 | 0.15372424 | -0.03787239 | 0.9697894266 | 0.99419501 |
| btnl10 | -0.006064034 | 0.1601961108 | -0.037853815 | 0.9698042372 | 0.9939543153 |
| folr3 | -0.005605851 | 0.1495606859 | -0.037482115 | 0.9701005999 | 0.9944098326 |
| ptx3 | 0.005754435152 | 0.1535672478 | 0.03747176066 | 0.9701088561 | 1.005771024 |
| prkra | 0.005452964061 | 0.1459455349 | 0.03736300712 | 0.9701955681 | 1.005467859 |
| prss22 | 0.005856750307 | 0.1581153974 | 0.03704098654 | 0.9704523256 | 1.005873935 |
| lat2 | -0.005290576 | 0.1432986168 | -0.03691994 | 0.9705488409 | 0.9947233941 |
| cnp | -0.005605782 | 0.1521460255 | -0.036844751 | 0.9706087924 | 0.9944099007 |
| upb1 | 0.00614729111 | 0.1675272442 | 0.03669427704 | 0.9707287718 | 1.006166224 |
| nrgn | 0.005556932955 | 0.1519836 | 0.03656271436 | 0.9708336733 | 1.005572401 |
| glod4 | 0.005320140483 | 0.1462529218 | 0.03637630223 | 0.9709823098 | 1.005334318 |
| msra | -0.005233963 | 0.1439822251 | -0.03635145 | 0.9710021257 | 0.9947797106 |
| apoa2 | -0.005795056 | 0.1596682803 | -0.036294348 | 0.9710476566 | 0.9942217028 |
| arg1 | 0.005217772258 | 0.1442258985 | 0.03617777606 | 0.9711406065 | 1.005231409 |
| sprr3 | -0.005909557 | 0.163743974 | -0.036090228 | 0.9712104144 | 0.9941078698 |
| syap1 | -0.005678734 | 0.1575910986 | -0.036034613 | 0.97125476 | 0.9943373593 |
| rhoc | -0.005017378 | 0.14355707 | -0.034950405 | 0.9721192877 | 0.9949951883 |
| pepd | -0.005265845 | 0.1519009475 | -0.034666307 | 0.9723458276 | 0.9947479953 |
| sox2 | -0.005383882 | 0.1555867657 | -0.034603726 | 0.9723957305 | 0.9946305853 |
| vmo1 | -0.004999633 | 0.1455476108 | -0.0343505 | 0.9725976557 | 0.9950128442 |
| ankmy2 | 0.005283063029 | 0.1538987156 | 0.03432818143 | 0.9726154526 | 1.005297043 |
| sugp1 | -0.005270694 | 0.1564388526 | -0.033691718 | 0.9731229832 | 0.994743172 |
| ggact | -0.005062247 | 0.1522497942 | -0.033249614 | 0.973475534 | 0.9949505447 |
| myl4 | -0.005248871 | 0.1582196329 | -0.03317459 | 0.9735353613 | 0.9947648798 |
| trim58 | -0.004963745 | 0.1519148555 | -0.03267452 | 0.9739341431 | 0.995048554 |
| ggh | 0.00500080131 | 0.1531697519 | 0.03264875243 | 0.9739546917 | 1.005013326 |
| mpi | -0.004769401 | 0.1468196164 | -0.032484768 | 0.9740854633 | 0.9952419544 |
| sgsh | 0.005167582235 | 0.1598598474 | 0.0323257048 | 0.9742123104 | 1.005180957 |
| prcp | -0.004952234 | 0.153469024 | -0.032268624 | 0.9742578307 | 0.9950600079 |
| git1 | 0.004896941847 | 0.1534218931 | 0.03191814251 | 0.9745373304 | 1.004908951 |
| olr1 | -0.004650751 | 0.1466190857 | -0.031719955 | 0.9746953811 | 0.9953600472 |
| sult2a1 | -0.004946403 | 0.1573989486 | -0.031425897 | 0.9749298883 | 0.9950658101 |
| ptrhd1 | 0.00498403349 | 0.1586345873 | 0.03141832796 | 0.9749359248 | 1.004996474 |
| dut | 0.004729180136 | 0.1521160345 | 0.03108929412 | 0.9751983276 | 1.00474038 |
| klk15 | 0.004562549666 | 0.1507128483 | 0.03027313011 | 0.9758492258 | 1.004572974 |
| cxadr | -0.004470418 | 0.1535212909 | -0.029119203 | 0.9767695206 | 0.9955395598 |
| prdx5 | -0.004115236 | 0.1432302924 | -0.028731602 | 0.9770786521 | 0.9958932202 |
| gipc3 | 0.004418610661 | 0.1540654933 | 0.02868007993 | 0.9771197437 | 1.004428387 |
| pkd1 | 0.004503145271 | 0.1626277753 | 0.02768989038 | 0.9779094869 | 1.0045133 |
| plin1 | -0.004503292 | 0.1628270635 | -0.027656903 | 0.9779357968 | 0.9955068323 |
| hspb1 | 0.00391213545 | 0.1461111632 | 0.02677506198 | 0.9786391437 | 1.003919798 |
| csrp3 | 0.004455508674 | 0.1670326752 | 0.02667447354 | 0.9787193731 | 1.004465449 |
| acrbp | -0.004119495 | 0.1560018471 | -0.026406707 | 0.9789329445 | 0.9958889784 |
| icam2 | 0.003579824236 | 0.1402587423 | 0.02552300254 | 0.9796378011 | 1.003586239 |
| ist1 | -0.003920548 | 0.1544954003 | -0.02537647 | 0.9797546796 | 0.9960871275 |
| rpgr | 0.003977086489 | 0.1574188073 | 0.0252643668 | 0.979844096 | 1.003985006 |
| klk12 | -0.003528271 | 0.1431081374 | -0.024654582 | 0.9803304825 | 0.9964779458 |
| gopc | -0.003605534 | 0.1497208007 | -0.02408172 | 0.9807874241 | 0.9964009577 |
| c1qtnf1 | 0.003489540965 | 0.1475669264 | 0.02364717522 | 0.9811340423 | 1.003495637 |
| itgam | -0.003381305 | 0.1454358845 | -0.023249456 | 0.9814512887 | 0.9966244049 |
| prok1 | -0.004799246 | 0.2106701196 | -0.022780856 | 0.9818250786 | 0.9952122523 |
| cetn3 | -0.003413673 | 0.1554417436 | -0.021961108 | 0.982478979 | 0.996592147 |
| eddm3b | 0.005184255753 | 0.237335187 | 0.02184360364 | 0.9825727118 | 1.005197717 |
| ccl8 | 0.00307404281 | 0.1441507971 | 0.02132518774 | 0.9829862515 | 1.003078773 |
| stat5b | 0.003024605752 | 0.1434101348 | 0.02109059974 | 0.9831733836 | 1.003029184 |
| txndc5 | 0.00304917002 | 0.1451415017 | 0.02100825734 | 0.9832390687 | 1.003053823 |
| bmp6 | 0.003020403364 | 0.1444682704 | 0.02090703623 | 0.9833198138 | 1.003024969 |
| cdc42bpb | 0.003044208116 | 0.1512911643 | 0.02012151953 | 0.9839464335 | 1.003048846 |
| triap1 | -0.002948556 | 0.1466661774 | -0.020103858 | 0.9839605229 | 0.9970557868 |
| muc16 | -0.002812937 | 0.1445695362 | -0.019457327 | 0.9844762786 | 0.9971910158 |
| angptl1 | -0.002898978 | 0.1502114397 | -0.019299317 | 0.9846023286 | 0.9971052198 |
| gnpda2 | 0.003057110061 | 0.1598313421 | 0.01912709998 | 0.9847397127 | 1.003061788 |
| aida | 0.00292033131 | 0.1585810677 | 0.01841538434 | 0.9853074796 | 1.0029246 |
| tctn3 | -0.002843718 | 0.1588087533 | -0.017906555 | 0.9857133994 | 0.9971603218 |
| slurp1 | 0.002889800234 | 0.1628543061 | 0.01774469649 | 0.9858425236 | 1.00289398 |
| crybb1 | 0.002743070589 | 0.1554719192 | 0.01764351146 | 0.9859232449 | 1.002746836 |
| galnt10 | 0.00261870427 | 0.1485373113 | 0.01762994258 | 0.9859340697 | 1.002622136 |

| Protein | beta | se | z | p_val | HR |
|---|---|---|---|---|---|
| treh | -0.002750589 | 0.1569132998 | -0.017529354 | 0.9860143154 | 0.9972531906 |
| tpmt | 0.002508849551 | 0.1436385501 | 0.01746640821 | 0.9860645311 | 1.002511999 |
| anpep | -0.002499871 | 0.144321216 | -0.017321574 | 0.9861800749 | 0.9975032515 |
| ahsa1 | -0.002471484 | 0.1555727192 | -0.015886358 | 0.9873250531 | 0.9975315676 |
| clec11a | 0.002209215323 | 0.1433903882 | 0.01540699729 | 0.9877074811 | 1.002211657 |
| slc34a3 | -0.002364961 | 0.1588373594 | -0.014889198 | 0.9881205777 | 0.9976378334 |
| gorasp2 | 0.002273563656 | 0.1559264838 | 0.0145809974 | 0.9883664595 | 1.00227615 |
| gpr158 | 0.002351077988 | 0.1616049624 | 0.01454830318 | 0.988392543 | 1.002353844 |
| araf | 0.002186461945 | 0.157394813 | 0.01389157561 | 0.9889164828 | 1.002188854 |
| cep43 | -0.00204785 | 0.1474953045 | -0.013884171 | 0.9889223901 | 0.9979542454 |
| hs6st1 | -0.001992387 | 0.1438703233 | -0.013848495 | 0.9889508532 | 0.9980095961 |
| fut8 | -0.001782937 | 0.1442789051 | -0.012357571 | 0.9901403358 | 0.9982186517 |
| ndufa5 | -0.001887497 | 0.1597111643 | -0.011818188 | 0.9905706701 | 0.9981142837 |
| hgs | -0.001672453 | 0.1420647266 | -0.011772469 | 0.9906071461 | 0.9983289452 |
| numb | -0.001851515 | 0.1577176229 | -0.011739432 | 0.9906335039 | 0.9981501978 |
| rbks | -0.001731127 | 0.1516708994 | -0.011413703 | 0.99089338 | 0.9982703709 |
| ipcef1 | 0.001540642895 | 0.1465017421 | 0.0105162087 | 0.9916094341 | 1.00154183 |
| tek | 0.001454653329 | 0.1442625966 | 0.01008337132 | 0.99195477 | 1.001455712 |
| neb | 0.001588912262 | 0.1623599961 | 0.009786353161 | 0.9921917445 | 1.001590175 |
| dbnl | 0.001188429314 | 0.142440328 | 0.008343348626 | 0.9933430482 | 1.001189136 |
| iglc2 | -0.001273394 | 0.1561286069 | -0.008156061 | 0.9934924772 | 0.998727416 |
| pcdh9 | 0.001232707215 | 0.1561414986 | 0.007894808397 | 0.9937009197 | 1.001233467 |
| ctsd | -0.00115573 | 0.1590765926 | -0.007265243 | 0.9942032259 | 0.9988449375 |
| tsc1 | -0.00111024 | 0.1562750234 | -0.007104398 | 0.9943315581 | 0.9988903761 |
| ptprz1 | -0.001205229 | 0.1725238274 | -0.006985867 | 0.9944261296 | 0.9987954974 |
| bgn | 0.0009517804353 | 0.1460028122 | 0.006518918512 | 0.9947986924 | 1.000952234 |
| cdkn2d | -0.000935544 | 0.1439069376 | -0.006501033 | 0.9948129629 | 0.9990648938 |
| rpl14 | 0.001003664357 | 0.1578252518 | 0.006359339497 | 0.9949260154 | 1.001004168 |
| farsa | -0.000904381 | 0.1619093374 | -0.005585725 | 0.9955432597 | 0.9990960278 |
| ezr | 0.0008332357783 | 0.1516810195 | 0.005493342418 | 0.9956169689 | 1.000833583 |
| slk | 0.000857637083 | 0.1587971391 | 0.005400834597 | 0.9956907784 | 1.000858005 |
| nid1 | 0.0007744628464 | 0.146879024 | 0.005272794068 | 0.9957929385 | 1.000774763 |
| lgals3 | 0.0007314301365 | 0.1524481826 | 0.004797893448 | 0.9961718496 | 1.000731698 |
| trim21 | 0.0006227069414 | 0.1523977671 | 0.00408606342 | 0.9967398022 | 1.000622901 |
| adgrf5 | -0.000482652 | 0.1572317963 | -0.003069682 | 0.997550752 | 0.9995174649 |
| cep170 | -0.00044962 | 0.150041988 | -0.002996631 | 0.997609038 | 0.9995504806 |
| mtif3 | 0.0004116665731 | 0.1530102121 | 0.002690451621 | 0.9978533328 | 1.000411751 |
| vta1 | -0.000309763 | 0.144040404 | -0.002150525 | 0.9982841304 | 0.9996902854 |
| ephb6 | -0.000219411 | 0.1437695796 | -0.001526131 | 0.9987823244 | 0.9997806129 |
| p4hb | 0.0001788094907 | 0.1487415272 | 0.001202149085 | 0.999040824 | 1.000178825 |
| thbs2 | -3.71e-05 | 0.1451565429 | -0.000255551 | 0.9997961001 | 0.9999629058 |

**Supplementary Table 13. Associations between circulating metabolites and incident age-related macular degeneration (AMD) in the UK Biobank**

| Metabolite | beta | se | z | p_val | HR |
|---|---|---|---|---|---|
| Chol.to.TLs.ratio.in.M.HDL | 0.09547135908 | 0.01986406464 | 4.806234818 | 1.53799e-06 | 1.100177311 |
| CE.to.TLs.ratio.in.M.HDL | 0.09279211885 | 0.01953301947 | 4.750526101 | 2.02888e-06 | 1.097233617 |
| PL.to.TLs.ratio.in.M.HDL | -0.0938213453 | 0.02015527469 | -4.654927642 | 3.24095e-06 | 0.9104454031 |
| PL.to.TLs.ratio.in.L.VLDL | -0.08662757884 | 0.01892871715 | -4.576516102 | 4.72783e-06 | 0.9170185492 |
| TG.to.TLs.ratio.in.M.HDL | -0.08747183271 | 0.01945493043 | -4.496126729 | 6.92025e-06 | 0.9162446794 |
| Free.Chol.in.V..L.VLDL | -0.08338438402 | 0.01932377553 | -4.315118641 | 1.59517e-05 | 0.9199974469 |
| TG.to.TLs.ratio.in.S.HDL | -0.08350776349 | 0.01964913377 | -4.249946306 | 2.13822e-05 | 0.9198839451 |
| Chol.in.V..L.VLDL | -0.08062479092 | 0.01901601099 | -4.239837207 | 2.23682e-05 | 0.9225397718 |
| PL.in.V..L.VLDL | -0.08247703796 | 0.01946382822 | -4.237452008 | 2.26071e-05 | 0.9208325818 |
| Ratio.of.ApoB.to.ApoA1 | -0.07639007442 | 0.01828831014 | -4.176989227 | 2.95393e-05 | 0.9264547498 |
| PL.in.VLDL | -0.07731592696 | 0.01859664758 | -4.157519609 | 3.21722e-05 | 0.9255973862 |
| CE.in.V..L.VLDL | -0.07655680861 | 0.01872290906 | -4.088937695 | 4.33353e-05 | 0.926300291 |
| TG.to.TLs.ratio.in.L.HDL | -0.08193167182 | 0.02009353622 | -4.077513828 | 4.55198e-05 | 0.9213349097 |
| TLs.in.VLDL | -0.07646902469 | 0.01887351696 | -4.051657401 | 5.08561e-05 | 0.9263816088 |
| CE.in.HDL | 0.0841307926 | 0.02080144336 | 4.044468988 | 5.24418e-05 | 1.087771157 |
| PL.in.L.VLDL | -0.07800573557 | 0.01936880207 | -4.027390816 | 5.63992e-05 | 0.9249591214 |
| Free.Chol.in.VLDL | -0.0739088539 | 0.0183998348 | -4.016821601 | 5.89883e-05 | 0.9287563425 |
| CE.to.TLs.ratio.in.L.HDL | 0.083042339 | 0.02069186047 | 4.013285279 | 5.98795e-05 | 1.086587813 |
| CE.in.L.VLDL | -0.07443831193 | 0.01855862276 | -4.010982543 | 6.04666e-05 | 0.9282647351 |
| Chol.in.L.VLDL | -0.07598185008 | 0.01894576919 | -4.0104917 | 6.05924e-05 | 0.9268330284 |
| TLs.in.V..L.VLDL | -0.07798476312 | 0.01955507814 | -3.987954565 | 6.66454e-05 | 0.9249785202 |
| VLDL_C | -0.07104004913 | 0.01806335149 | -3.932827702 | 8.39524e-05 | 0.9314245886 |
| Free.Chol.in.L.VLDL | -0.07605682799 | 0.01934723204 | -3.931147765 | 8.45413e-05 | 0.926763539 |
| PL.to.TLs.ratio.in.V..L.VLDL | -0.07072088184 | 0.01799146575 | -3.930801572 | 8.46631e-05 | 0.9317219163 |
| Con.of.V..L.VLDL.particles | -0.07576423464 | 0.01950035794 | -3.885274048 | 0.000102214521 | 0.9270347435 |
| HDL_C | 0.07969020412 | 0.02090369606 | 3.812254249 | 0.0001377051187 | 1.082951522 |
| Con.of.VLDL.particles | -0.06967896261 | 0.01832164146 | -3.803096068 | 0.0001428989082 | 0.9326932012 |
| CE.in.VLDL | -0.06801370509 | 0.01789433241 | -3.800851774 | 0.0001441995465 | 0.9342476695 |
| PL.in.V..S.VLDL | -0.06934559692 | 0.01829486772 | -3.790439919 | 0.0001503807058 | 0.933004181 |
| Ratio.of.TG.to.PG | -0.07752032399 | 0.02047528794 | -3.786043166 | 0.0001530650968 | 0.9254082162 |
| TLs.in.L.VLDL | -0.07288919096 | 0.01927188824 | -3.782150977 | 0.000155479009 | 0.9297038439 |
| CE.in.L.HDL | 0.08125817505 | 0.02152261402 | 3.775478898 | 0.0001597005445 | 1.08465089 |
| Con.of.L.VLDL.particles | -0.07252929134 | 0.01932616853 | -3.752905871 | 0.0001747965025 | 0.9300385042 |
| CE.in.M.HDL | 0.07481241106 | 0.01993605435 | 3.752618734 | 0.0001749969016 | 1.077681971 |
| CE.in.S.VLDL | -0.0672550513 | 0.01805033034 | -3.725973432 | 0.0001945628981 | 0.934956709 |
| Con.of.chylomicrons.and.extremely.L.VLDL.particles | -0.07167391623 | 0.01945489868 | -3.684106375 | 0.0002295063875 | 0.9308343763 |
| PL.in.chylomicrons.and.extremely.L.VLDL | -0.07184350449 | 0.01965585557 | -3.655068804 | 0.0002571130142 | 0.9306765311 |
| TLs.in.V..S.VLDL | -0.06635145774 | 0.01830789555 | -3.624199054 | 0.0002898583088 | 0.9358019117 |
| Chol.in.M.HDL | 0.07252836271 | 0.02002802006 | 3.621344621 | 0.0002930758052 | 1.075223302 |
| Free.Chol.in.chylomicrons.and.extremely.L.VLDL | -0.07070414694 | 0.01953375355 | -3.619588359 | 0.0002950720433 | 0.9317375087 |
| Chol.in.L.HDL | 0.07752867213 | 0.02155270629 | 3.597166457 | 0.0003217026239 | 1.080613215 |
| TG.in.S.HDL | -0.06919728691 | 0.01936288241 | -3.573707955 | 0.0003519614741 | 0.9331425651 |
| TG.in.V..L.VLDL | -0.07018379913 | 0.01969924558 | -3.562765836 | 0.0003669679929 | 0.9322224625 |
| TLs.in.M.VLDL | -0.06500431568 | 0.01828094969 | -3.555850039 | 0.0003767589462 | 0.9370634193 |
| TLs.in.S.VLDL | -0.06572526011 | 0.01851746387 | -3.549366186 | 0.0003861596733 | 0.9363880921 |
| Con.of.S.VLDL.particles | -0.06598589917 | 0.01859148643 | -3.549253548 | 0.0003863249051 | 0.9361440646 |
| TG.in.VLDL | -0.06887387656 | 0.01946841432 | -3.537723999 | 0.0004035916916 | 0.9334444019 |
| Chol.in.S.VLDL | -0.06335947323 | 0.01798498286 | -3.522909847 | 0.0004268365244 | 0.9386060093 |
| TG | -0.06806860883 | 0.0194289511 | -3.503462872 | 0.0004592506719 | 0.9341963773 |
| Con.of.V..S.VLDL.particles | -0.06358625387 | 0.01818875951 | -3.495909318 | 0.0004724492285 | 0.9383931758 |
| PL.in.M.VLDL | -0.06294073494 | 0.01802266953 | -3.492309218 | 0.0004788635415 | 0.9389991219 |
| Chol.to.TLs.ratio.in.L.HDL | 0.07165969867 | 0.02069146732 | 3.463248766 | 0.0005336947279 | 1.0742897 |
| Con.of.L.HDL.particles | 0.07480455181 | 0.02167140642 | 3.451762676 | 0.0005569373561 | 1.077673501 |
| TLs.in.L.HDL | 0.07455765214 | 0.02164374538 | 3.444766645 | 0.000571552592 | 1.077407457 |
| PL.in.L.HDL | 0.07340432865 | 0.02154801286 | 3.406547469 | 0.00065790109 | 1.076165574 |
| TG.in.L.VLDL | -0.06542646401 | 0.01925721917 | -3.397503214 | 0.0006800378909 | 0.936667923 |
| Free.Chol.to.TLs.ratio.in.V..L.HDL | -0.07220095588 | 0.02125737191 | -3.396513745 | 0.0006825012956 | 0.9303439189 |
| Free.Chol.in.V..S.VLDL | -0.06165826907 | 0.01824377826 | -3.379687485 | 0.0007256829364 | 0.9402041287 |
| Con.of.M.VLDL.particles | -0.06075435057 | 0.01810561777 | -3.355552478 | 0.000792066274 | 0.9410543809 |
| TLs.in.chylomicrons.and.extremely.L.VLDL | -0.06476544337 | 0.01942195738 | -3.334650679 | 0.0008540661469 | 0.9372872845 |
| TG.in.V..S.VLDL | -0.06330025144 | 0.01904202191 | -3.324240028 | 0.0008865985207 | 0.9386615969 |
| PL.in.S.VLDL | -0.06009205091 | 0.01811414991 | -3.317409384 | 0.0009085638229 | 0.9416778473 |
| TG.in.M.VLDL | -0.0628393022 | 0.0190149835 | -3.304725571 | 0.0009506943829 | 0.939094372 |
| Chol.in.chylomicrons.and.extremely.L.VLDL | -0.06438622821 | 0.01952239558 | -3.298070052 | 0.000973518436 | 0.9376427855 |
| TG.to.TLs.ratio.in.V..L.HDL | -0.06628994535 | 0.02024367094 | -3.274601012 | 0.001058113024 | 0.9358594768 |
| TG.in.chylomicrons.and.extremely.L.VLDL | -0.06280175041 | 0.01921473711 | -3.268415802 | 0.001081513509 | 0.9391296373 |
| Free.Chol.in.M.VLDL | -0.05816879306 | 0.01793016952 | -3.244185339 | 0.001177870774 | 0.9434906793 |
| TG.in.S.LDL | -0.06219480675 | 0.01928270488 | -3.22541921 | 0.001257883325 | 0.9396998091 |

| Metabolite | beta | se | z | p_val | HR |
|---|---|---|---|---|---|
| Free.Chol.to.TLs.ratio.in.M.LDL | 0.06125559469 | 0.01976466618 | 3.099247624 | 0.001940127913 | 1.06317062 |
| Chol.in.V..S.VLDL | -0.05662967227 | 0.0183286995 | -3.089672143 | 0.002003775507 | 0.9449439435 |
| Free.Chol.in.S.VLDL | -0.05522919673 | 0.01791265983 | -3.083249347 | 0.002047535158 | 0.9462682415 |
| Average.diameter.for.VLDL.particles | -0.06138717911 | 0.01994730704 | -3.077466998 | 0.002087679536 | 0.9404590432 |
| CE.in.chylomicrons.and.extremely.L.VLDL | -0.05964646859 | 0.01938503754 | -3.076933355 | 0.002091420548 | 0.9420975358 |
| TG.in.IDL | -0.05864069445 | 0.01909191841 | -3.071493036 | 0.002129911326 | 0.9430455498 |
| TLs.in.HDL | 0.0633460993 | 0.02067621184 | 3.063718819 | 0.002186042693 | 1.065395508 |
| Con.of.M.HDL.particles | 0.06096475037 | 0.0199723968 | 3.052450388 | 0.002269812485 | 1.062861448 |
| Remnant_C | -0.05470283424 | 0.01793897239 | -3.049385051 | 0.002293103677 | 0.9467664527 |
| Chol.to.TLs.ratio.in.S.HDL | 0.05604059653 | 0.01878697249 | 2.982949837 | 0.002854847801 | 1.057640619 |
| TG.in.M.HDL | -0.05550072674 | 0.01870537178 | -2.96710097 | 0.003006222174 | 0.9460113362 |
| TG.in.S.VLDL | -0.05685057695 | 0.01917109032 | -2.965432639 | 0.003022575297 | 0.944735224 |
| Free.Chol.in.M.HDL | 0.06010378125 | 0.02029213193 | 2.961925413 | 0.003057218334 | 1.061946751 |
| Con.of.S.LDL.particles | -0.05268403418 | 0.01783718068 | -2.953607699 | 0.00314082993 | 0.9486797155 |
| Free.Chol.in.L.HDL | 0.06342494883 | 0.02154410034 | 2.943959034 | 0.003240428885 | 1.065479517 |
| ApoB | -0.05237137022 | 0.01784366328 | -2.935012245 | 0.003335345255 | 0.9489763798 |
| TG.in.HDL | -0.05505047926 | 0.0187653034 | -2.933631185 | 0.003350220433 | 0.9464373713 |
| CE.in.V..S.VLDL | -0.05393785362 | 0.01841812207 | -2.928520802 | 0.003405790417 | 0.9474909878 |
| ApoA1 | 0.05866016688 | 0.02013088159 | 2.913939294 | 0.003568993018 | 1.060414815 |
| Average.diameter.for.HDL.particles | 0.06262346452 | 0.02154068618 | 2.907217718 | 0.003646592977 | 1.064625894 |
| Con.of.IDL.particles | -0.05152046136 | 0.01790003977 | -2.87823167 | 0.003999113344 | 0.9497842159 |
| Chol.in.M.VLDL | -0.0509683761 | 0.01787583617 | -2.851244306 | 0.004354849558 | 0.9503087225 |
| CE.to.TLs.ratio.in.M.LDL | -0.05170737535 | 0.01847347933 | -2.799005776 | 0.005126022049 | 0.9496067045 |
| Free.Chol.in.HDL | 0.05859969488 | 0.0210216975 | 2.787581492 | 0.005310309632 | 1.060350692 |
| Free.Chol.to.TLs.ratio.in.M.HDL | 0.05819761029 | 0.0209838394 | 2.77344909 | 0.005546551814 | 1.059924427 |
| CE.to.TLs.ratio.in.S.HDL | 0.05159776973 | 0.01886906154 | 2.734517009 | 0.006247186763 | 1.052952128 |
| TLs.in.M.HDL | 0.05388969462 | 0.01978302906 | 2.724036569 | 0.006448936538 | 1.055368183 |
| Con.of.LDL.particles | -0.04838322704 | 0.01784789701 | -2.710864312 | 0.006710808195 | 0.9527685904 |
| PL.in.HDL | 0.05532252619 | 0.02047596914 | 2.701826995 | 0.006895963396 | 1.056881432 |
| Con.of.HDL.particles | 0.05225893605 | 0.01945207639 | 2.686547956 | 0.007219458393 | 1.053648535 |
| TG.in.LDL | -0.05109395162 | 0.01917647323 | -2.664408152 | 0.007712392409 | 0.9501893945 |
| Con.of.L.LDL.particles | -0.04757424796 | 0.01787984235 | -2.660775584 | 0.007796089939 | 0.9535396721 |
| Free.Chol.to.TLs.ratio.in.S.LDL | 0.05030686724 | 0.0192303868 | 2.616009119 | 0.008896418167 | 1.051593747 |
| CE.in.S.LDL | -0.04630483185 | 0.0179449043 | -2.580388899 | 0.009868910617 | 0.9547508794 |
| TG.in.M.LDL | -0.04928614736 | 0.01922103062 | -2.564178183 | 0.01034204366 | 0.9519087046 |
| TG.in.L.LDL | -0.04814547071 | 0.01913470576 | -2.516133319 | 0.01186502917 | 0.9529951441 |
| TG.to.TLs.ratio.in.L.VLDL | 0.04464826034 | 0.01781871645 | 2.50569453 | 0.01222111513 | 1.045659995 |
| CE.to.TLs.ratio.in.S.LDL | -0.04473643369 | 0.01817420152 | -2.46153503 | 0.01383438872 | 0.9562494838 |
| Con.of.M.LDL.particles | -0.04402398021 | 0.01791750601 | -2.457037279 | 0.01400881489 | 0.9569310098 |
| CE.in.V..L.HDL | 0.05159220705 | 0.02105623808 | 2.450210092 | 0.01427728826 | 1.052946271 |
| TC.minus.HDL.C | -0.04302104969 | 0.01796765753 | -2.394360512 | 0.01664937494 | 0.9578912265 |
| CE.in.M.VLDL | -0.04307173754 | 0.01803633778 | -2.388053388 | 0.01693788104 | 0.9578426743 |
| TLs.in.S.LDL | -0.04260284212 | 0.01786672339 | -2.384479862 | 0.01710328404 | 0.9582919077 |
| Tyrosine | -0.04297141824 | 0.01823357598 | -2.356719181 | 0.01843718356 | 0.9579387692 |
| Linoleic.acid | -0.04252250244 | 0.01815664022 | -2.341980781 | 0.01918170265 | 0.9583688996 |
| CE.in.M.LDL | -0.04216171835 | 0.01804269351 | -2.336775178 | 0.01945087715 | 0.9587147263 |
| Valine | -0.04468250643 | 0.01912761962 | -2.336020233 | 0.01949018707 | 0.9563010531 |
| Monounsaturated.FA | -0.04383651552 | 0.01914819024 | -2.289329434 | 0.02206021876 | 0.9571104174 |
| CE.to.TLs.ratio.in.V..L.HDL | 0.04230151523 | 0.01848732555 | 2.288136004 | 0.02212960209 | 1.043208975 |
| TG.in.V..L.HDL | -0.04223157627 | 0.01869292456 | -2.259227877 | 0.02386921363 | 0.9586477548 |
| Chol.in.S.LDL | -0.04024100139 | 0.01786456899 | -2.252559322 | 0.02428694841 | 0.9605579155 |
| PL.in.M.HDL | 0.04411270013 | 0.01962074699 | 2.248268129 | 0.02455909768 | 1.045100131 |
| Free.Chol.to.TLs.ratio.in.IDL | 0.04023237112 | 0.01810445902 | 2.222235476 | 0.02626739612 | 1.041052657 |
| Albumin | -0.04001592561 | 0.01841843389 | -2.172601962 | 0.02981028811 | 0.9607741381 |
| Total.FA | -0.04047908033 | 0.01872379505 | -2.161905758 | 0.03062544107 | 0.9603292541 |
| Chol.to.TLs.ratio.in.chylomicrons.and.extremely.L.VLDL | 0.03979587081 | 0.0184895824 | 2.152340164 | 0.03137057269 | 1.040598336 |
| PL.in.IDL | -0.03976770788 | 0.01849843669 | -2.149787495 | 0.03157202778 | 0.9610126489 |
| TLs.in.M.LDL | -0.03860475223 | 0.01798892253 | -2.146029155 | 0.03187065268 | 0.9621309141 |
| PL.in.S.LDL | -0.03803471003 | 0.01777939652 | -2.139257651 | 0.03241480745 | 0.9626795257 |
| PL.in.M.LDL | -0.03806141551 | 0.01794494286 | -2.121010682 | 0.03392090347 | 0.9626538172 |
| Total.Con.of.LP.particles | 0.04109612497 | 0.01940013451 | 2.118342269 | 0.03414609128 | 1.041952258 |
| Chol.in.V..L.HDL | 0.04396298264 | 0.0208220947 | 2.11136215 | 0.03474119578 | 1.044943673 |
| Chol.in.M.LDL | -0.0372286345 | 0.01796325346 | -2.072488405 | 0.03821991651 | 0.9634558309 |
| PL.to.TLs.ratio.in.L.HDL | -0.04115419068 | 0.02019618237 | -2.037721284 | 0.04157781435 | 0.9596811447 |
| PL.in.V..L.HDL | 0.04269551817 | 0.02100385309 | 2.032746944 | 0.04207809587 | 1.043620083 |
| Total.Con.of.branched.chain.AAs | -0.03828230446 | 0.0189159832 | -2.023807277 | 0.04298998516 | 0.9624412011 |
| Omega.6.FA | -0.03721364524 | 0.01849139282 | -2.012484706 | 0.04416886724 | 0.9634702725 |
| Polyunsaturated.FA | -0.0371997725 | 0.01870351968 | -1.988918296 | 0.04671022192 | 0.9634836386 |
| Pyruvate | 0.03738516069 | 0.01883807305 | 1.984553334 | 0.04719418863 | 1.038092776 |
| TLs.in.V..L.HDL | 0.04122813264 | 0.02095057281 | 1.967876154 | 0.04908228915 | 1.042089813 |

| Metabolite | beta | se | z | p_val | HR |
|---|---|---|---|---|---|
| TLs.in.IDL | -0.03614442982 | 0.01842895272 | -1.96128507 | 0.04984577793 | 0.9645009807 |
| Histidine | -0.03514547638 | 0.01802207917 | -1.95013439 | 0.05116010322 | 0.9654649537 |
| PL.to.TLs.ratio.in.V..S.VLDL | -0.03582108514 | 0.01855679033 | -1.930349188 | 0.05356358622 | 0.9648128974 |
| PL.in.LDL | -0.03450680194 | 0.01791224825 | -1.926436116 | 0.05404994154 | 0.9660817684 |
| CE.to.TLs.ratio.in.chylomicrons.and.extremely.L.VLDL | 0.03521869614 | 0.01828486371 | 1.92611204 | 0.05409038558 | 1.03584622 |
| CE.in.LDL | -0.03407056102 | 0.01800235436 | -1.892561403 | 0.05841622071 | 0.9665033048 |
| Citrate | -0.03445866286 | 0.01831660767 | -1.881279737 | 0.05993387591 | 0.9661282758 |
| Saturated.FA | -0.03522020719 | 0.01877028943 | -1.876380613 | 0.06060303475 | 0.9653928064 |
| Free.Chol.to.TLs.ratio.in.L.LDL | 0.03631112248 | 0.01942253589 | 1.86953561 | 0.0615483342 | 1.036978424 |
| TLs.in.LDL | -0.03328492125 | 0.01798497078 | -1.850707552 | 0.06421163707 | 0.9672629266 |
| Creatinine | -0.03867879212 | 0.02090851055 | -1.849906622 | 0.06432700929 | 0.9620596807 |
| X3.Hydroxybutyrate | 0.0335982619 | 0.01820295787 | 1.845758374 | 0.0649272972 | 1.034169058 |
| CE.in.IDL | -0.03280582891 | 0.01843768574 | -1.77928127 | 0.07519366007 | 0.9677264458 |
| Ratio.of.monounsaturated.FA.to.total.FA | -0.03567715025 | 0.02026817855 | -1.760254389 | 0.07836468394 | 0.9649517776 |
| Lactate | 0.0324054984 | 0.01841994856 | 1.759261069 | 0.07853317585 | 1.032936274 |
| Clinical.LDL_C | -0.03150128828 | 0.01797353953 | -1.75264801 | 0.07966244491 | 0.9689897081 |
| Con.of.V..L.HDL.particles | 0.03574115719 | 0.02082568414 | 1.716205669 | 0.08612440597 | 1.03638755 |
| LDL_C | -0.0308827898 | 0.01800754492 | -1.714991685 | 0.08634675149 | 0.9695892122 |
| TLs.in.LP.particles | -0.03130070978 | 0.01833205655 | -1.707430353 | 0.08774209273 | 0.9691840861 |
| PL.in.L.LDL | -0.03074091042 | 0.01802082955 | -1.705854347 | 0.08803520158 | 0.9697267866 |
| CE.to.TLs.ratio.in.S.VLDL | -0.03086054916 | 0.01810074487 | -1.704932553 | 0.08820700428 | 0.9696107767 |
| Leucine | -0.03181485534 | 0.01867215424 | -1.703866352 | 0.08840605838 | 0.9686859125 |
| PL.to.TLs.ratio.in.V..L.HDL | 0.03460858158 | 0.02036336939 | 1.699550842 | 0.08921544361 | 1.035214427 |
| Chol.in.IDL | -0.03078711758 | 0.0184435195 | -1.669264783 | 0.09506491632 | 0.9696819794 |
| PL.to.TLs.ratio.in.S.LDL | 0.03019666167 | 0.01819322973 | 1.659774659 | 0.09695979342 | 1.030657205 |
| Free.Chol.to.TLs.ratio.in.L.VLDL | -0.02963152245 | 0.01787737972 | -1.657486887 | 0.09742107785 | 0.9708031868 |
| Total.free.Chol | -0.02983340756 | 0.01832478971 | -1.628035466 | 0.1035173657 | 0.9706072159 |
| TLs.in.L.LDL | -0.02907943397 | 0.01811576158 | -1.605200744 | 0.1084496338 | 0.9713393041 |
| Phenylalanine | -0.02869972457 | 0.01795219704 | -1.598674775 | 0.1098928853 | 0.9717082008 |
| CE.in.L.LDL | -0.02863073792 | 0.01813297866 | -1.578931871 | 0.1143516848 | 0.971775238 |
| PL.to.TLs.ratio.in.chylomicrons.and.extremely.L.VLDL | -0.0279354499 | 0.01791029879 | -1.559742259 | 0.1188208014 | 0.9724511366 |
| TG.to.TLs.ratio.in.S.LDL | -0.02807186405 | 0.01924978468 | -1.458294964 | 0.1447592578 | 0.9723184895 |
| Chol.in.L.LDL | -0.02646758682 | 0.01816092536 | -1.457391971 | 0.145008212 | 0.9738796099 |
| Ratio.of.polyunsaturated.FA.to.monounsaturated.FA | 0.02898215751 | 0.0202236689 | 1.433081092 | 0.1518346642 | 1.029406227 |
| Isoleucine | -0.02593089803 | 0.01841017779 | -1.408508833 | 0.1589804533 | 0.9744024204 |
| CE.to.TLs.ratio.in.V..L.VLDL | 0.0271125938 | 0.01942577336 | 1.395702158 | 0.1628042041 | 1.027483485 |
| Acetate | 0.02463171556 | 0.01790918706 | 1.375367596 | 0.1690175097 | 1.024937582 |
| Free.Chol.to.TLs.ratio.in.chylomicrons.and.extremely.L..VLDL | 0.02540084525 | 0.01867541914 | 1.36012183 | 0.1737913742 | 1.025726196 |
| CE.in.S.HDL | 0.0239708108 | 0.01807562546 | 1.326140047 | 0.1847933132 | 1.02426042 |
| Free.Chol.in.IDL | -0.02433967501 | 0.01843099308 | -1.320584024 | 0.1866401026 | 0.9759541462 |
| Ratio.of.docosahexaenoic.acid.to.total.FA | 0.0244988801 | 0.01878526985 | 1.304153749 | 0.1921811659 | 1.024801443 |
| TG.in.L.HDL | -0.02483279417 | 0.01915328608 | -1.296529174 | 0.1947932358 | 0.9754730032 |
| Free.Chol.in.M.LDL | -0.02295956801 | 0.01794854543 | -1.279188228 | 0.2008307804 | 0.9773019972 |
| Chol.in.S.HDL | 0.02287771831 | 0.01817320929 | 1.258870569 | 0.2080770859 | 1.02314142 |
| Free.Chol.in.S.LDL | -0.02154316114 | 0.01785812463 | -1.2063507 | 0.2276822966 | 0.9786872353 |
| Omega.3.FAs | -0.02199822304 | 0.01881385641 | -1.169256455 | 0.2423003218 | 0.9782419733 |
| Free.Chol.in.LDL | -0.02114800108 | 0.01809947301 | -1.168431869 | 0.2426326062 | 0.9790740498 |
| TG.to.TLs.ratio.in.chylomicrons.and.extremely.L.VLDL | -0.02060009903 | 0.01826989071 | -1.127543638 | 0.2595127014 | 0.9796106335 |
| Glycoprotein.acetyls | -0.02199530177 | 0.01957286758 | -1.123764909 | 0.2611127716 | 0.9782448311 |
| Ratio.of.omega.6.FA.to.total.FA | 0.02224670475 | 0.02014237255 | 1.104472906 | 0.2693880502 | 1.022496008 |
| Chol.to.TLs.ratio.in.L.LDL | 0.02139683673 | 0.01941301221 | 1.102190454 | 0.2703788812 | 1.02162739 |
| Free.Chol.in.L.LDL | -0.01990512255 | 0.01825922747 | -1.090140456 | 0.2756512779 | 0.9802916765 |
| PL.to.TLs.ratio.in.M.VLDL | -0.01884356801 | 0.01874394241 | -1.005315083 | 0.3147451547 | 0.9813328621 |
| PL.to.TLs.ratio.in.M.LDL | 0.01823723772 | 0.01820337508 | 1.001860239 | 0.3164110982 | 1.018404552 |
| TG.to.TLs.ratio.in.IDL | -0.0193911201 | 0.01936457607 | -1.001370752 | 0.3166475987 | 0.9807956783 |
| TG.to.TLs.ratio.in.L.LDL | -0.01915428867 | 0.01936931437 | -0.9888986417 | 0.3227127352 | 0.9810279891 |
| Chol.to.TLs.ratio.in.V..L.VLDL | 0.01836410123 | 0.01940777381 | 0.9462239927 | 0.3440343512 | 1.018533758 |
| Free.Chol.to.TLs.ratio.in.V..L.VLDL | -0.01777715921 | 0.0190370849 | -0.9338172998 | 0.3503981441 | 0.9823799223 |
| Free.Chol.in.V..L.HDL | 0.01814957404 | 0.01976113463 | 0.9184479724 | 0.3583843853 | 1.018315279 |
| Free.Chol.to.TLs.ratio.in.S.HDL | 0.01761371374 | 0.01920379555 | 0.9171996078 | 0.3590380523 | 1.01776975 |
| TC | -0.01657304944 | 0.01855009866 | -0.8934210942 | 0.3716317104 | 0.983563528 |
| Ratio.of.polyunsaturated.FA.to.total.FA | 0.01762030551 | 0.0198511473 | 0.8876215183 | 0.3747443747 | 1.017776459 |
| PL.to.TLs.ratio.in.S.VLDL | 0.01614237078 | 0.01927001433 | 0.8376937611 | 0.4022027183 | 1.016273363 |
| Chol.to.TLs.ratio.in.IDL | 0.01516185481 | 0.01869542374 | 0.8109928407 | 0.4173697822 | 1.015277379 |
| Sphingomyelins | 0.01560563192 | 0.01926409773 | 0.8100889093 | 0.417889078 | 1.015728036 |
| Glutamine | -0.01445673979 | 0.01829822655 | -0.790062346 | 0.4294913587 | 0.9856472571 |
| Chol.to.TLs.ratio.in.S.VLDL | -0.01468711486 | 0.01860650972 | -0.7893535696 | 0.429905385 | 0.9854202147 |
| Chol.to.TLs.ratio.in.L.VLDL | -0.01443414667 | 0.01841810471 | -0.7836933763 | 0.4332200501 | 0.9856695262 |
| Glucose | -0.014478245 | 0.01960602721 | -0.7384588855 | 0.4602356427 | 0.9856260608 |
| Con.of.S.HDL.particles | 0.01335310152 | 0.01818779098 | 0.7341794031 | 0.4628394049 | 1.013442652 |

| Metabolite | beta | se | z | p_val | HR |
|---|---|---|---|---|---|
| Free.Chol.in.S.HDL | 0.01355276052 | 0.01858924165 | 0.7290647339 | 0.4659620656 | 1.013645015 |
| Ratio.of.linoleic.acid.to.total.FA | -0.01411144019 | 0.01962215541 | -0.7191585173 | 0.4720432555 | 0.9859876595 |
| Acetoacetate | -0.01214135096 | 0.01830765831 | -0.6631842671 | 0.5072125464 | 0.9879320578 |
| Glycine | 0.01219798011 | 0.0191553847 | 0.6367911847 | 0.5242608689 | 1.012272679 |
| Free.Chol.to.TLs.ratio.in.V..S.VLDL | 0.01126973656 | 0.01836872642 | 0.6135284671 | 0.5395269658 | 1.011333479 |
| PL.to.TLs.ratio.in.IDL | -0.01078706593 | 0.01763148075 | -0.6118071466 | 0.5406653609 | 0.9892709058 |
| Free.Chol.to.TLs.ratio.in.S.VLDL | 0.01162816226 | 0.01928864961 | 0.6028499917 | 0.5466084864 | 1.011696032 |
| CE.to.TLs.ratio.in.L.LDL | -0.01075267952 | 0.01794528573 | -0.5991924391 | 0.5490445642 | 0.9893049239 |
| Total.esterified.Chol | -0.01116930021 | 0.01864584222 | -0.5990236364 | 0.5491571228 | 0.9888928448 |
| Total.PL.in.LP.particles | -0.01064501519 | 0.01903475194 | -0.5592410778 | 0.5759972021 | 0.9894114425 |
| Chol.to.TLs.ratio.in.V..S.VLDL | 0.01044215056 | 0.01955977013 | 0.5338585521 | 0.5934394001 | 1.01049686 |
| CE.to.TLs.ratio.in.V..S.VLDL | 0.0102035632 | 0.01973061242 | 0.5171437655 | 0.6050558042 | 1.010255797 |
| Degree.of.unsaturation | 0.009831720276 | 0.01909987807 | 0.5147530387 | 0.6067256053 | 1.00988021 |
| Ratio.of.omega.6.FA.to.omega.3.FA | 0.008883531767 | 0.01849367195 | 0.4803552151 | 0.6309748335 | 1.008923107 |
| CE.to.TLs.ratio.in.M.VLDL | 0.008856053604 | 0.01959887106 | 0.4518654965 | 0.6513658823 | 1.008895384 |
| Acetone | 0.008047984215 | 0.01799952397 | 0.4471220587 | 0.6547869329 | 1.008080456 |
| Spectrometer.corrected.alanine | 0.0077577145 | 0.01836853801 | 0.4223370688 | 0.6727790049 | 1.007787884 |
| TG.to.TLs.ratio.in.M.LDL | -0.007749872379 | 0.01894586197 | -0.4090535649 | 0.6825003523 | 0.9922800805 |
| PL.to.TLs.ratio.in.S.HDL | -0.007002205613 | 0.01873881588 | -0.3736738573 | 0.7086469775 | 0.9930222527 |
| Chol.to.TLs.ratio.in.S.LDL | -0.006700651414 | 0.01815442627 | -0.3690918851 | 0.7120592374 | 0.9933217479 |
| TG.to.TLs.ratio.in.V..L.VLDL | 0.006953033452 | 0.01896823403 | 0.3665619815 | 0.7139457714 | 1.006977262 |
| TLs.in.S.HDL | 0.00594039659 | 0.01834157178 | 0.3238760919 | 0.7460318451 | 1.005958076 |
| Chol.to.TLs.ratio.in.M.VLDL | 0.006208905698 | 0.01942747845 | 0.3195940078 | 0.7492761174 | 1.006228221 |
| PL.to.TLs.ratio.in.L.LDL | -0.005621078365 | 0.01770175112 | -0.3175436333 | 0.7508311366 | 0.9943946903 |
| TG.to.TLs.ratio.in.S.VLDL | 0.005562252349 | 0.01882830257 | 0.2954197453 | 0.7676732515 | 1.00557775 |
| PL.in.S.HDL | 0.004878461652 | 0.01845550976 | 0.2643363263 | 0.7915207711 | 1.004890381 |
| PG | -0.004783179292 | 0.01929042943 | -0.2479560816 | 0.8041683876 | 0.9952282419 |
| PC | -0.004777263873 | 0.01942962384 | -0.245875263 | 0.8057787933 | 0.9952341291 |
| Ratio.of.omega.3.FA.to.total.FA | -0.004346522537 | 0.01858590098 | -0.2338612769 | 0.8150926762 | 0.9956629099 |
| Free.Chol.to.TLs.ratio.in.L.HDL | -0.004503598391 | 0.01956664937 | -0.2301670718 | 0.8179619478 | 0.9955065276 |
| Glucose.lactate | 0.004011638204 | 0.01975841509 | 0.2030344127 | 0.8391081349 | 1.004019696 |
| TG.to.TLs.ratio.in.V..S.VLDL | -0.003661759925 | 0.01950827951 | -0.1877028634 | 0.8511095855 | 0.9963449361 |
| Ratio.of.saturated.FA.to.total.FA | 0.002916421364 | 0.01844664292 | 0.1581003859 | 0.8743776965 | 1.002920678 |
| CE.to.TLs.ratio.in.L.VLDL | -0.002995826279 | 0.01910008813 | -0.1568488197 | 0.8753639964 | 0.9970086567 |
| CE.to.TLs.ratio.in.IDL | -0.00168824342 | 0.01856396167 | -0.09094197944 | 0.9275386936 | 0.9983131809 |
| Alanine | 0.001507527059 | 0.01819536841 | 0.08285224159 | 0.9339690291 | 1.001508664 |
| Total.cholines | -0.0015314952 | 0.01937604556 | -0.07904064821 | 0.9370002916 | 0.9984696769 |
| Docosahexaenoic.acid | 0.0008697080633 | 0.0190058583 | 0.04575999934 | 0.9635015413 | 1.000870086 |
| Average.diameter.for.LDL.particles | -0.0006747329823 | 0.01894813393 | -0.03560946872 | 0.9715937582 | 0.9993254946 |
| TG.to.TLs.ratio.in.M.VLDL | -0.000490610082 | 0.01928539428 | -0.0254394634 | 0.979704434 | 0.9995095102 |
| Free.Chol.to.TLs.ratio.in.M.VLDL | -0.0003170603727 | 0.01896303751 | -0.01671991486 | 0.9866600596 | 0.9996829899 |
| Chol.to.TLs.ratio.in.M.LDL | 0.0002560017078 | 0.01793661208 | 0.01427257872 | 0.9886125164 | 1.000256034 |
| Chol.to.TLs.ratio.in.V..L.HDL | 0.0002180667713 | 0.01907960389 | 0.01142931334 | 0.9908809259 | 1.000218091 |

## Supplementary Table 14. Associations between circulating metabolites and incident cataract in the UK Biobank

| Metabolite | beta | se | z | p_val | HR |
|---|---|---|---|---|---|
| Alanine | 0.04665483341 | 0.01044508861 | 4.466676652 | 7.94441e-06 | 1.047760295 |
| Docosahexaenoic.acid | -0.04366546384 | 0.01084333146 | -4.02694172 | 5.6507e-05 | 0.9572741467 |
| Lactate | 0.04195644564 | 0.01049025813 | 3.999562751 | 6.34596e-05 | 1.042849057 |
| Spectrometer.corrected.alanine | 0.0404667895 | 0.01054438856 | 3.837755909 | 0.0001241638107 | 1.041296727 |
| Ratio.of.omega.3.FA.to.total.FA | -0.04061529051 | 0.01059859017 | -3.832140864 | 0.0001270329642 | 0.9601984564 |
| Omega.3.FAs | -0.03880102448 | 0.01071777396 | -3.620250309 | 0.0002943181532 | 0.961942093 |
| Ratio.of.docosahexaenoic.acid.to.total.FA | -0.03840431185 | 0.01073306721 | -3.578130194 | 0.0003460610688 | 0.9623237833 |
| Ratio.of.omega.6.FA.to.omega.3.FA | 0.03771441683 | 0.01054515898 | 3.576467354 | 0.000348268787 | 1.038434631 |
| Glucose.lactate | 0.03981275343 | 0.0113727547 | 3.500713281 | 0.0004640147753 | 1.040615904 |
| Glycoprotein.acetyls | 0.03608465651 | 0.01116945975 | 3.230653704 | 0.001235074782 | 1.03674361 |
| X3.Hydroxybutyrate | 0.03063135721 | 0.01046679862 | 2.926525897 | 0.003427709673 | 1.031105324 |
| CE.in.L.LDL | -0.02701712834 | 0.01037822244 | -2.603252002 | 0.009234404934 | 0.9733445696 |
| CE.to.TLs.ratio.in.L.LDL | -0.02669788036 | 0.01026483872 | -2.600905975 | 0.009297793356 | 0.9736553575 |
| Chol.to.TLs.ratio.in.M.LDL | -0.02659485892 | 0.01030720174 | -2.580221053 | 0.009873708934 | 0.97375567 |
| Chol.in.L.LDL | -0.02671586771 | 0.01039744267 | -2.569465258 | 0.01018556044 | 0.9736378442 |
| Chol.to.TLs.ratio.in.L.LDL | -0.0284737121 | 0.01112603625 | -2.559196417 | 0.01049144453 | 0.9719278437 |
| CE.in.IDL | -0.02700962901 | 0.01056351411 | -2.556879153 | 0.01056159018 | 0.9733518691 |
| Clinical.LDL_C | -0.02594492461 | 0.01029534113 | -2.520064589 | 0.01173333021 | 0.974388753 |
| LDL_C | -0.02591849638 | 0.01030846209 | -2.514293224 | 0.01192712215 | 0.9744145047 |
| CE.in.LDL | -0.02586161845 | 0.01030219607 | -2.51030152 | 0.0120628113 | 0.9744699289 |
| TLs.in.L.LDL | -0.02595669026 | 0.0103706598 | -2.502896706 | 0.01231814894 | 0.9743772887 |
| TC.minus.HDL.C | -0.02565350676 | 0.01028146558 | -2.495121591 | 0.01259139755 | 0.9746727486 |
| Chol.in.IDL | -0.02628023281 | 0.01056704611 | -2.486998972 | 0.01288257711 | 0.9740620872 |
| CE.in.V..S.VLDL | -0.02611951501 | 0.01053341361 | -2.479681893 | 0.01314996405 | 0.9742186489 |
| PL.in.IDL | -0.02615090736 | 0.0105853144 | -2.470489431 | 0.01349283059 | 0.9741880664 |
| Con.of.L.LDL.particles | -0.02520463261 | 0.01022271312 | -2.465552178 | 0.01368022486 | 0.9751103522 |
| PL.in.L.LDL | -0.02544816461 | 0.01032363759 | -2.465038548 | 0.0136998512 | 0.9748729106 |
| TLs.in.IDL | -0.02596397689 | 0.01055075851 | -2.460863537 | 0.01386030734 | 0.9743701888 |
| Citrate | 0.02585113006 | 0.01051403777 | 2.458725242 | 0.01394312814 | 1.026188169 |
| Con.of.LDL.particles | -0.02499780874 | 0.01021080902 | -2.448171216 | 0.0143583407 | 0.9753120492 |
| TLs.in.LDL | -0.02512423548 | 0.01029456284 | -2.440534471 | 0.01466554626 | 0.9751887515 |
| Free.Chol.in.LDL | -0.02509338743 | 0.01037006541 | -2.41979066 | 0.01552944429 | 0.9752188346 |
| Free.Chol.in.M.LDL | -0.02486967762 | 0.01028220773 | -2.418709899 | 0.01557565648 | 0.975437025 |
| Total.free.Chol | -0.02535001659 | 0.01048685607 | -2.417313294 | 0.01563555303 | 0.9749685971 |
| Chol.in.V..S.VLDL | -0.02517839088 | 0.01047790099 | -2.402999504 | 0.01626120947 | 0.9751359412 |
| Free.Chol.in.L.LDL | -0.0251407159 | 0.01046394497 | -2.402603986 | 0.01627880557 | 0.9751726801 |
| PL.in.LDL | -0.02462291095 | 0.01025929872 | -2.400057902 | 0.01639247866 | 0.9756777601 |
| ApoB | -0.02448438577 | 0.01020890153 | -2.39833695 | 0.01646970711 | 0.9758129254 |
| Chol.in.M.VLDL | -0.02436892525 | 0.01022624484 | -2.382978857 | 0.01717318061 | 0.9759255997 |
| Remnant_C | -0.02434437049 | 0.01025974312 | -2.372805069 | 0.01765357792 | 0.9759495636 |
| Degree.of.unsaturation | -0.02589155775 | 0.01094040325 | -2.366599947 | 0.01795232625 | 0.9744407544 |
| Con.of.M.LDL.particles | -0.02419761645 | 0.01025722597 | -2.359079981 | 0.01832030676 | 0.9760927987 |
| Con.of.IDL.particles | -0.02415994402 | 0.01024130219 | -2.359069537 | 0.01832082238 | 0.9761295712 |
| Chol.in.M.LDL | -0.02411584808 | 0.01028559867 | -2.344622695 | 0.0190463427 | 0.9761726155 |
| TC | -0.02468692533 | 0.01062921797 | -2.322553306 | 0.02020316161 | 0.9756153047 |
| CE.in.M.VLDL | -0.02377420034 | 0.01032013546 | -2.303671346 | 0.02124109949 | 0.9765061796 |
| Chol.in.S.LDL | -0.02345861178 | 0.01022720478 | -2.293746169 | 0.02180508312 | 0.9768144024 |
| CE.to.TLs.ratio.in.IDL | -0.02435894459 | 0.01063047921 | -2.291424885 | 0.02193885168 | 0.9759353402 |
| Total.esterified.Chol | -0.0243327001 | 0.01068959333 | -2.276298018 | 0.0228281787 | 0.9759609534 |
| CE.in.M.LDL | -0.02334030838 | 0.01032799149 | -2.259907785 | 0.02382697462 | 0.9769299697 |
| Free.Chol.in.S.LDL | -0.02308306785 | 0.01022829458 | -2.256785593 | 0.02402147542 | 0.9771813081 |
| TLs.in.M.LDL | -0.02314460627 | 0.01029896826 | -2.24727426 | 0.02462250483 | 0.9771211757 |
| Free.Chol.in.IDL | -0.0235702308 | 0.01055880921 | -2.232281154 | 0.02559638667 | 0.9767053775 |
| TG.to.TLs.ratio.in.M.LDL | 0.02410414374 | 0.01082665633 | 2.226370081 | 0.02598940279 | 1.024396997 |
| PL.to.TLs.ratio.in.V..S.VLDL | 0.0235763358 | 0.01062262325 | 2.219445729 | 0.02645641553 | 1.023856455 |
| Free.Chol.in.M.VLDL | -0.02269272739 | 0.01025359763 | -2.213147834 | 0.02688745521 | 0.9775628159 |
| TLs.in.S.LDL | -0.0224272549 | 0.01022359298 | -2.193676425 | 0.0282586798 | 0.9778223664 |
| CE.in.S.LDL | -0.02249203671 | 0.01026902031 | -2.190280672 | 0.0285038877 | 0.9777590233 |
| PL.in.M.LDL | -0.02246402271 | 0.01027833873 | -2.185569408 | 0.02884712279 | 0.9777864147 |
| Leucine | -0.02310197435 | 0.01072745433 | -2.153537423 | 0.03127646623 | 0.9771628331 |
| Free.Chol.in.S.VLDL | -0.02203374091 | 0.01024902647 | -2.149837449 | 0.03156807484 | 0.9782072289 |
| PL.in.S.LDL | -0.02164910136 | 0.01017323553 | -2.128044839 | 0.03333336613 | 0.9785835584 |
| Ratio.of.monounsaturated.FA.to.total.FA | 0.02461275201 | 0.01158824457 | 2.123941366 | 0.03367504682 | 1.024918146 |
| Con.of.S.LDL.particles | -0.02163624053 | 0.01021174481 | -2.118760401 | 0.03411072084 | 0.9785961439 |
| CE.to.TLs.ratio.in.V..S.VLDL | -0.02383882759 | 0.01129280768 | -2.110974371 | 0.03477451484 | 0.9764430728 |
| Chol.to.TLs.ratio.in.V..S.VLDL | -0.02351379938 | 0.01119519797 | -2.100346903 | 0.03569833612 | 0.9767604959 |
| Free.Chol.in.V..S.VLDL | -0.02184248826 | 0.01041922091 | -2.096364829 | 0.03604983989 | 0.9783943315 |
| Chol.to.TLs.ratio.in.S.LDL | -0.02158273729 | 0.01041245842 | -2.072780166 | 0.03819274312 | 0.9786485034 |

| Metabolite | beta | se | z | p_val | HR |
|---|---|---|---|---|---|
| PL.in.M.VLDL | -0.02117423073 | 0.01030390958 | -2.054970549 | 0.03988184941 | 0.9790483694 |
| TG.to.TLs.ratio.in.L.LDL | 0.02268398187 | 0.01107500533 | 2.048214082 | 0.04053902726 | 1.02294322 |
| TLs.in.LP.particles | -0.02129658958 | 0.01046983476 | -2.034090323 | 0.0419424895 | 0.9789285815 |
| Albumin | -0.02133164083 | 0.01051682348 | -2.028334969 | 0.04252607234 | 0.9788942694 |
| CE.in.VLDL | -0.02075831129 | 0.0102408427 | -2.027012023 | 0.0426611831 | 0.9794556593 |
| Con.of.V..S.VLDL.particles | -0.02045573989 | 0.01038890455 | -1.968998732 | 0.0489532367 | 0.9797520595 |
| Free.Chol.to.TLs.ratio.in.M.HDL | -0.02321958733 | 0.01203507647 | -1.929326114 | 0.05369038978 | 0.9770479129 |
| TLs.in.V..S.VLDL | -0.02006471798 | 0.01045292123 | -1.919532113 | 0.05491702623 | 0.9801352389 |
| Chol.in.S.VLDL | -0.01976193017 | 0.01029900377 | -1.918819589 | 0.05500716988 | 0.9804320568 |
| PC | -0.02109632518 | 0.01111540508 | -1.897935796 | 0.05770454016 | 0.9791246457 |
| Chol.to.TLs.ratio.in.IDL | -0.02026858533 | 0.01071757846 | -1.891153435 | 0.05860386516 | 0.9799354417 |
| TG.to.TLs.ratio.in.V..S.VLDL | 0.02103829746 | 0.01115701422 | 1.885656597 | 0.05934124526 | 1.021261163 |
| Con.of.M.VLDL.particles | -0.01950562791 | 0.01034930498 | -1.884728293 | 0.05946653047 | 0.980683376 |
| TG.to.TLs.ratio.in.IDL | 0.02085836299 | 0.01108850498 | 1.881079823 | 0.05996106125 | 1.021077419 |
| PL.in.S.VLDL | -0.01933508973 | 0.01035919846 | -1.866465809 | 0.06197622331 | 0.9808506342 |
| PL.to.TLs.ratio.in.S.VLDL | -0.02032039367 | 0.01103580412 | -1.841315183 | 0.06557538406 | 0.9798846742 |
| TLs.in.M.VLDL | -0.01921764098 | 0.01044367106 | -1.840123158 | 0.06575015806 | 0.9809658406 |
| VLDL_C | -0.01888907683 | 0.01033087315 | -1.828410489 | 0.06748796837 | 0.9812882038 |
| Total.PL.in.LP.particles | -0.0197172453 | 0.01088946965 | -1.810670854 | 0.07019181707 | 0.9804758683 |
| Free.Chol.to.TLs.ratio.in.S.VLDL | -0.01984244612 | 0.01104844384 | -1.795949403 | 0.07250256435 | 0.9803531196 |
| TG.to.TLs.ratio.in.S.VLDL | 0.01927070163 | 0.0107735204 | 1.788709809 | 0.07366156234 | 1.01945758 |
| PL.to.TLs.ratio.in.M.VLDL | -0.01918494365 | 0.01075065475 | -1.78453723 | 0.07433641076 | 0.9809979161 |
| CE.in.S.VLDL | -0.01820643531 | 0.01034046755 | -1.760697495 | 0.07828961701 | 0.9819583006 |
| Ratio.of.ApoB.to.ApoA1 | -0.01833896955 | 0.01046566238 | -1.752298983 | 0.0797224108 | 0.9818281661 |
| Ratio.of.polyunsaturated.FA.to.monounsaturated.FA | -0.02019380034 | 0.01156276854 | -1.746450279 | 0.08073273968 | 0.9800087289 |
| Chol.to.TLs.ratio.in.S.VLDL | -0.01839674807 | 0.01064608814 | -1.728028909 | 0.08398304155 | 0.9817714392 |
| Polyunsaturated.FA | -0.01838195717 | 0.01066635784 | -1.72335838 | 0.08482373647 | 0.9817859605 |
| TG.to.TLs.ratio.in.S.LDL | 0.01871035178 | 0.01100132222 | 1.700736639 | 0.08899245182 | 1.018886487 |
| Free.Chol.to.TLs.ratio.in.M.VLDL | -0.01835437146 | 0.01088957688 | -1.685499048 | 0.09189232841 | 0.9818130442 |
| TG.to.TLs.ratio.in.M.VLDL | 0.01856318161 | 0.01106589238 | 1.677513297 | 0.09344215158 | 1.018736549 |
| Free.Chol.to.TLs.ratio.in.V..S.VLDL | -0.0175461913 | 0.01047369424 | -1.675262892 | 0.09388266283 | 0.9826068467 |
| Free.Chol.to.TLs.ratio.in.L.HDL | -0.01857265332 | 0.01119697819 | -1.658720147 | 0.0971721977 | 0.9815987556 |
| PL.in.V..S.VLDL | -0.01714357116 | 0.01044142809 | -1.641879924 | 0.1006148913 | 0.9830025437 |
| Con.of.V..L.HDL.particles | -0.01955452238 | 0.01191617939 | -1.641006042 | 0.1007961609 | 0.9806354271 |
| CE.to.TLs.ratio.in.chylomicrons.and.extremely.L.VLDL | -0.01714508209 | 0.01047398963 | -1.636919903 | 0.1016472067 | 0.9830010584 |
| Free.Chol.to.TLs.ratio.in.S.HDL | -0.01794658858 | 0.0110039062 | -1.630928895 | 0.1029053239 | 0.9822134924 |
| Chol.to.TLs.ratio.in.M.VLDL | -0.01803698667 | 0.01114675158 | -1.618138391 | 0.1056327764 | 0.9821247062 |
| Acetone | 0.01651218619 | 0.01031877889 | 1.600207386 | 0.1095525842 | 1.016649266 |
| CE.to.TLs.ratio.in.M.VLDL | -0.01797150022 | 0.01123730157 | -1.599271863 | 0.1097602086 | 0.9821890241 |
| Con.of.VLDL.particles | -0.01663307981 | 0.01046213888 | -1.589835502 | 0.1118718891 | 0.9835044861 |
| Total.Con.of.branched.chain.AAs | -0.01706811423 | 0.01084901502 | -1.573240907 | 0.1156630465 | 0.9830767208 |
| Chol.in.V..L.HDL | -0.01870899173 | 0.01192350749 | -1.56908458 | 0.1166282324 | 0.9814649351 |
| CE.in.V..L.HDL | -0.01846221459 | 0.01206304854 | -1.530476689 | 0.1258987792 | 0.9817071681 |
| PL.to.TLs.ratio.in.IDL | 0.01538726666 | 0.01011600133 | 1.521081914 | 0.1282392825 | 1.01550626 |
| Free.Chol.in.VLDL | -0.01592281648 | 0.01051192047 | -1.514739055 | 0.1298384998 | 0.9842032814 |
| CE.to.TLs.ratio.in.S.VLDL | -0.01563791289 | 0.01036468453 | -1.508768825 | 0.1313578701 | 0.9844837244 |
| Free.Chol.in.V..L.HDL | -0.01696684386 | 0.01132289239 | -1.498454924 | 0.1340150955 | 0.9831762824 |
| Total.cholines | -0.01661277508 | 0.01108858115 | -1.498187626 | 0.1340845095 | 0.9835244561 |
| Sphingomyelins | -0.01606157782 | 0.01104620843 | -1.454035376 | 0.1459364985 | 0.9840667215 |
| PG | -0.01586126237 | 0.01102765259 | -1.438317198 | 0.1503440744 | 0.984263865 |
| Chol.to.TLs.ratio.in.chylomicrons.and.extremely.L.VLDL | -0.0151468476 | 0.01057652352 | -1.432119692 | 0.1521095698 | 0.9849672889 |
| CE.in.L.VLDL | -0.01507665165 | 0.01060018494 | -1.422300811 | 0.1549389434 | 0.985036432 |
| CE.to.TLs.ratio.in.M.LDL | -0.01503525271 | 0.01057713898 | -1.421485786 | 0.1551755829 | 0.9850772124 |
| Free.Chol.in.HDL | -0.01695787974 | 0.01205428663 | -1.406792477 | 0.1594889341 | 0.9831850958 |
| TLs.in.S.VLDL | -0.0148404787 | 0.01057335147 | -1.403573763 | 0.1604458125 | 0.9852690985 |
| TLs.in.V..L.HDL | -0.01666575632 | 0.01198275303 | -1.390811968 | 0.164282453 | 0.9834723491 |
| CE.in.V..L.VLDL | -0.01429481896 | 0.01070425645 | -1.335433155 | 0.1817346195 | 0.9858068669 |
| PL.in.VLDL | -0.0138026082 | 0.01061911446 | -1.299789003 | 0.1936732956 | 0.986292211 |
| CE.to.TLs.ratio.in.V..L.VLDL | -0.0143498837 | 0.01110920834 | -1.291710738 | 0.1964573374 | 0.9857525852 |
| Pyruvate | 0.01367813387 | 0.01064233626 | 1.28525669 | 0.1987025994 | 1.013772108 |
| Valine | -0.0140267423 | 0.01094837463 | -1.281171203 | 0.2001335384 | 0.9860711741 |
| Total.Con.of.LP.particles | -0.01425370394 | 0.01113210304 | -1.280414302 | 0.2003994667 | 0.9858473992 |
| Free.Chol.to.TLs.ratio.in.S.LDL | -0.01395275094 | 0.01096328346 | -1.272679941 | 0.2031316386 | 0.9861441375 |
| Con.of.S.VLDL.particles | -0.01333337882 | 0.01061024742 | -1.256651074 | 0.2088800117 | 0.9867551169 |
| PL.to.TLs.ratio.in.L.HDL | 0.01432400067 | 0.011586635 | 1.236251998 | 0.2163649079 | 1.014427081 |
| Free.Chol.in.S.HDL | -0.01270277954 | 0.01064235388 | -1.193606197 | 0.2326320488 | 0.9873775602 |
| Average.diameter.for.LDL.particles | -0.01289835841 | 0.01082540273 | -1.191489936 | 0.2334613051 | 0.9871844689 |
| PL.in.V..L.HDL | -0.01423694175 | 0.01200670907 | -1.185748873 | 0.2357214877 | 0.9858639243 |
| Free.Chol.in.L.HDL | -0.01422543029 | 0.01234440413 | -1.152378855 | 0.2491654263 | 0.9858752731 |
| Chol.in.L.VLDL | -0.01233831625 | 0.01081403014 | -1.140954491 | 0.2538888618 | 0.9877374887 |

| Metabolite | beta | se | z | p_val | HR |
|---|---|---|---|---|---|
| Free.Chol.to.TLs.ratio.in.M.LDL | -0.01278515264 | 0.01130327043 | -1.131102075 | 0.258012131 | 0.9872962302 |
| Acetoacetate | 0.01182300423 | 0.01047456235 | 1.128734914 | 0.2590096762 | 1.011893172 |
| TLs.in.VLDL | -0.01214022387 | 0.01077245742 | -1.126968842 | 0.2597556548 | 0.9879331713 |
| TG.in.M.VLDL | -0.01218104939 | 0.01085541367 | -1.122117476 | 0.2618124942 | 0.9878928393 |
| PL.to.TLs.ratio.in.M.LDL | 0.01142621331 | 0.01041696084 | 1.096885501 | 0.2726914476 | 1.011491742 |
| Con.of.S.HDL.particles | -0.01131194631 | 0.0104395093 | -1.083570691 | 0.2785551918 | 0.9887517932 |
| Chol.in.V..L.VLDL | -0.01156036491 | 0.01086237861 | -1.064257225 | 0.2872121899 | 0.9885061994 |
| Saturated.FA | -0.01137100206 | 0.01068497623 | -1.064204713 | 0.2872359725 | 0.9886934034 |
| CE.to.TLs.ratio.in.L.VLDL | -0.01163897192 | 0.01096302743 | -1.061656736 | 0.2883915468 | 0.9884284989 |
| Chol.in.S.HDL | -0.01105550009 | 0.0104286732 | -1.060106102 | 0.2890963321 | 0.9890053874 |
| Con.of.L.HDL.particles | -0.01298757322 | 0.01243125155 | -1.044751863 | 0.296137668 | 0.9870964014 |
| Chol.to.TLs.ratio.in.V..L.VLDL | -0.01154350105 | 0.01111369702 | -1.038673362 | 0.2989566738 | 0.9885228695 |
| HDL_C | -0.01239850135 | 0.01200535794 | -1.03274733 | 0.3017221579 | 0.9876780434 |
| CE.to.TLs.ratio.in.S.LDL | -0.01063127556 | 0.01042609373 | -1.019679646 | 0.3078804181 | 0.9894250367 |
| Ratio.of.saturated.FA.to.total.FA | -0.01066679876 | 0.01052978115 | -1.013012389 | 0.3110542431 | 0.9893898898 |
| Con.of.HDL.particles | -0.01120985359 | 0.01116240671 | -1.004250596 | 0.3152578402 | 0.9888527427 |
| Isoleucine | -0.01052878365 | 0.01057258016 | -0.9958575382 | 0.319319369 | 0.98952645 |
| Free.Chol.to.TLs.ratio.in.L.LDL | -0.01103395864 | 0.01115224799 | -0.9893932285 | 0.3224707863 | 0.9890266922 |
| TG.to.TLs.ratio.in.chylomicrons.and.extremely.L.VLDL | 0.01018304465 | 0.01046346984 | 0.9731995983 | 0.3304541104 | 1.010235068 |
| Chol.in.L.HDL | -0.01197625334 | 0.01236461253 | -0.9685910744 | 0.3327492584 | 0.9880951765 |
| TLs.in.L.VLDL | -0.0105434208 | 0.01100243239 | -0.9582808985 | 0.3379211332 | 0.9895119662 |
| TG.to.TLs.ratio.in.V..L.VLDL | 0.01030159179 | 0.01088467028 | 0.9464312214 | 0.3439286872 | 1.010354836 |
| CE.in.S.HDL | -0.009701851026 | 0.01037717431 | -0.934922238 | 0.3498283757 | 0.9903450601 |
| Glucose | 0.01033596635 | 0.01126273856 | 0.9177134226 | 0.3587689186 | 1.010389567 |
| Omega.6.FA | -0.00957682522 | 0.01055830549 | -0.9070418761 | 0.3643846529 | 0.9904688865 |
| Linoleic.acid | -0.009401814804 | 0.01037762663 | -0.9059696534 | 0.364951915 | 0.9906422441 |
| TG.in.L.VLDL | -0.009962406883 | 0.01100662634 | -0.9051281085 | 0.3653975225 | 0.9900870535 |
| TLs.in.HDL | -0.01065447057 | 0.01185590709 | -0.8986634672 | 0.3688319417 | 0.9894020873 |
| TG.to.TLs.ratio.in.V..L.HDL | 0.01036315239 | 0.0115558042 | 0.896791968 | 0.3698299347 | 1.010417036 |
| CE.in.HDL | -0.01067090086 | 0.01194793525 | -0.8931167304 | 0.3717946644 | 0.9893858312 |
| CE.in.L.HDL | -0.01100757087 | 0.01235065875 | -0.8912537453 | 0.3727930571 | 0.9890527908 |
| Ratio.of.polyunsaturated.FA.to.total.FA | -0.01009775412 | 0.01134359791 | -0.8901720772 | 0.3733734955 | 0.989953057 |
| Free.Chol.in.M.HDL | -0.01032930768 | 0.01163569352 | -0.8877260013 | 0.374688156 | 0.9897238564 |
| TLs.in.S.HDL | -0.009254604211 | 0.0105222763 | -0.8795249194 | 0.3791167278 | 0.9907880878 |
| Total.FA | -0.009368315027 | 0.01065400663 | -0.8793231832 | 0.3792260693 | 0.9906754309 |
| Ratio.of.omega.6.FA.to.total.FA | 0.00998641746 | 0.01146225164 | 0.8712439557 | 0.3836209642 | 1.010036448 |
| ApoA1 | -0.009997768227 | 0.0115427596 | -0.8661506063 | 0.3864075763 | 0.9900520433 |
| TLs.in.L.HDL | -0.01070497512 | 0.01240746058 | -0.8627853421 | 0.3882554997 | 0.9893521192 |
| Free.Chol.in.L.VLDL | -0.00938144661 | 0.01104085508 | -0.8497029029 | 0.3954902841 | 0.9906624219 |
| PL.to.TLs.ratio.in.L.VLDL | 0.0090505817 | 0.01080390808 | 0.8377136894 | 0.4021915232 | 1.009091662 |
| CE.to.TLs.ratio.in.V..L.HDL | -0.008608507442 | 0.01050627717 | -0.8193680126 | 0.4125764793 | 0.9914284397 |
| Chol.to.TLs.ratio.in.L.HDL | -0.009597451031 | 0.01185292405 | -0.8097116785 | 0.4181059043 | 0.9904484575 |
| Con.of.L.VLDL.particles | -0.008771655788 | 0.01103347678 | -0.7950037839 | 0.426611301 | 0.9912667029 |
| PL.to.TLs.ratio.in.chylomicrons.and.extremely.L.VLDL | 0.008180925157 | 0.01029154873 | 0.7949168172 | 0.426661891 | 1.00821448 |
| PL.in.L.VLDL | -0.008768015661 | 0.01105814393 | -0.7929012062 | 0.4278353845 | 0.9912703113 |
| PL.in.S.HDL | -0.008306683327 | 0.01058051458 | -0.7850925647 | 0.4323992988 | 0.9917277218 |
| PL.to.TLs.ratio.in.S.LDL | 0.008183902797 | 0.01044132176 | 0.7837995025 | 0.4331577658 | 1.008217482 |
| Chol.to.TLs.ratio.in.L.VLDL | -0.008069008327 | 0.01058320316 | -0.7624353616 | 0.4458002079 | 0.9919634587 |
| Free.Chol.to.TLs.ratio.in.V..L.HDL | 0.009066294734 | 0.01218096107 | 0.7443004441 | 0.4566947326 | 1.009107518 |
| PL.in.HDL | -0.008717197896 | 0.01173457296 | -0.7428645188 | 0.457563709 | 0.9913206867 |
| Free.Chol.in.V..L.VLDL | -0.008044498601 | 0.01102463408 | -0.7296839552 | 0.4655833908 | 0.9919877718 |
| Average.diameter.for.HDL.particles | -0.00877076268 | 0.01233160822 | -0.7112424046 | 0.4769340354 | 0.9912675883 |
| TG.in.L.LDL | -0.007690863946 | 0.01088741487 | -0.7063994567 | 0.4799397587 | 0.9923386351 |
| PL.in.L.HDL | -0.008371144041 | 0.01234396134 | -0.6781570204 | 0.4976721403 | 0.9916637964 |
| TG.in.IDL | -0.00726955995 | 0.01086127248 | -0.6693101532 | 0.5032976514 | 0.9927567994 |
| PL.to.TLs.ratio.in.S.HDL | 0.007150752859 | 0.01071710852 | 0.667227811 | 0.5046266293 | 1.007176381 |
| Con.of.V..L.VLDL.particles | -0.007389137583 | 0.01114154983 | -0.6632055413 | 0.5071989231 | 0.992638095 |
| TG.to.TLs.ratio.in.L.VLDL | 0.006693299822 | 0.01027024184 | 0.6517178395 | 0.5145832121 | 1.00671575 |
| PL.to.TLs.ratio.in.M.HDL | 0.007513508906 | 0.01155870445 | 0.650030368 | 0.5156726058 | 1.007541806 |
| PL.to.TLs.ratio.in.L.LDL | 0.006579875708 | 0.01013373429 | 0.6493041476 | 0.5161418054 | 1.006601571 |
| PL.in.V..L.VLDL | -0.007076230514 | 0.01110680865 | -0.6371074478 | 0.5240548576 | 0.9929487471 |
| TLs.in.V..L.VLDL | -0.007101441202 | 0.01116476697 | -0.6360581659 | 0.524738511 | 0.9929237144 |
| Acetate | 0.006421589203 | 0.01026506566 | 0.6255770219 | 0.5315924143 | 1.006442252 |
| Con.of.M.HDL.particles | -0.007050166549 | 0.01145090449 | -0.6156864335 | 0.5381014928 | 0.9929746276 |
| Chol.to.TLs.ratio.in.S.HDL | -0.006572323857 | 0.0107187899 | -0.613159127 | 0.5397711276 | 0.9934492266 |
| CE.to.TLs.ratio.in.L.HDL | -0.006774007719 | 0.01182547777 | -0.5728316313 | 0.5667586997 | 0.9932488842 |
| TG.to.TLs.ratio.in.L.HDL | 0.006506594782 | 0.01148014294 | 0.5667694921 | 0.5708708004 | 1.006527809 |
| TG.in.LDL | -0.00617911457 | 0.01091250078 | -0.5662418444 | 0.571229388 | 0.9938399369 |
| TG.in.VLDL | -0.006054793467 | 0.011105245 | -0.5452192606 | 0.5856027262 | 0.9939634999 |
| Tyrosine | -0.005483687533 | 0.0104597356 | -0.5242663621 | 0.6000932896 | 0.9945313204 |

| Metabolite | beta | se | z | p_val | HR |
|---|---|---|---|---|---|
| TLs.in.chylomicrons.and.extremely.L.VLDL | 0.005767708354 | 0.01110682537 | 0.5192940523 | 0.6035557004 | 1.005784374 |
| Chol.in.M.HDL | -0.005843620634 | 0.01149032047 | -0.5085689865 | 0.6110543722 | 0.9941734201 |
| TG.in.V..L.HDL | -0.005341932874 | 0.01061624118 | -0.5031849584 | 0.6148342391 | 0.9946723099 |
| TG | -0.005430175134 | 0.0110725824 | -0.490416322 | 0.6238393291 | 0.9945845416 |
| TG.in.S.VLDL | -0.005268133441 | 0.01092658991 | -0.4821388453 | 0.6297073165 | 0.9947457188 |
| TLs.in.M.HDL | -0.005410511 | 0.01133786808 | -0.4772070869 | 0.6332146651 | 0.9946040995 |
| Free.Chol.to.TLs.ratio.in.chylomicrons.and.extremely.L.VLDL | -0.005079065153 | 0.01068673833 | -0.4752680373 | 0.6345959379 | 0.9949338115 |
| PL.to.TLs.ratio.in.V..L.HDL | -0.005396985247 | 0.01164451945 | -0.4634785721 | 0.6430213706 | 0.9946175523 |
| Histidine | -0.004465504879 | 0.01031549437 | -0.432892959 | 0.6650925354 | 0.9955444507 |
| TG.in.V..L.VLDL | -0.004805774023 | 0.01125481142 | -0.426997294 | 0.6693812986 | 0.9952057552 |
| PL.in.M.HDL | -0.004565603933 | 0.01123673227 | -0.4063106447 | 0.6845143651 | 0.9954448026 |
| CE.in.M.HDL | -0.004582172223 | 0.01143733314 | -0.4006329245 | 0.6886904014 | 0.9954283099 |
| Chol.to.TLs.ratio.in.M.HDL | -0.004483858964 | 0.01136851247 | -0.3944103484 | 0.6932781048 | 0.9955261785 |
| TG.in.M.LDL | -0.004297571705 | 0.01094429116 | -0.392677026 | 0.6945580411 | 0.9957116496 |
| TG.in.L.HDL | -0.004231454368 | 0.0108972489 | -0.3883048287 | 0.6977904643 | 0.9957774856 |
| PL.to.TLs.ratio.in.V..L.VLDL | -0.00391105395 | 0.0103437528 | -0.3781078325 | 0.70535049 | 0.9960965843 |
| TG.in.V..S.VLDL | -0.003631899072 | 0.01085217686 | -0.3346700961 | 0.7378739617 | 0.9963746883 |
| TG.to.TLs.ratio.in.S.HDL | 0.003574966053 | 0.01124241589 | 0.3179891303 | 0.7504931821 | 1.003581364 |
| Glycine | -0.003460454601 | 0.01099818108 | -0.3146388094 | 0.753035909 | 0.9965455259 |
| Free.Chol.to.TLs.ratio.in.IDL | -0.003161744357 | 0.01034200544 | -0.3057186902 | 0.7598188415 | 0.9968432487 |
| Ratio.of.linoleic.acid.to.total.FA | 0.00321632911 | 0.01122140462 | 0.2866244662 | 0.7743998788 | 1.003221507 |
| CE.to.TLs.ratio.in.S.HDL | -0.003008815102 | 0.01072654375 | -0.2805018255 | 0.7790925251 | 0.9969957068 |
| Free.Chol.to.TLs.ratio.in.L.VLDL | 0.002779147965 | 0.01028101454 | 0.2703184547 | 0.7869152689 | 1.002783013 |
| TG.in.M.HDL | -0.002829867338 | 0.01069665703 | -0.2645562375 | 0.7913513366 | 0.997174133 |
| TG.in.HDL | -0.00280459854 | 0.01071321066 | -0.2617887979 | 0.7934842759 | 0.9971993307 |
| TG.in.chylomicrons.and.extremely.L.VLDL | 0.002788682288 | 0.01100448755 | 0.2534131894 | 0.7999489366 | 1.002792574 |
| PL.in.chylomicrons.and.extremely.L.VLDL | 0.002715957875 | 0.01122743552 | 0.2419036717 | 0.808854805 | 1.002719649 |
| CE.in.chylomicrons.and.extremely.L.VLDL | -0.002597306863 | 0.0110810485 | -0.2343917962 | 0.8146808269 | 0.9974060632 |
| Ratio.of.TG.to.PG | 0.002700784449 | 0.01169615561 | 0.2309121508 | 0.8173830508 | 1.002704435 |
| Free.Chol.to.TLs.ratio.in.V..L.VLDL | -0.002523441496 | 0.01093496174 | -0.2307682053 | 0.817494883 | 0.9974797397 |
| Free.Chol.in.chylomicrons.and.extremely.L.VLDL | 0.002535907143 | 0.01115075761 | 0.2274201657 | 0.8200970411 | 1.002539125 |
| TG.in.S.LDL | -0.002471941782 | 0.01098504566 | -0.2250279022 | 0.821957568 | 0.997531111 |
| Monounsaturated.FA | 0.001787631728 | 0.01089846414 | 0.1640260229 | 0.8697106579 | 1.00178923 |
| Glutamine | 0.001484127265 | 0.01046822577 | 0.1417744801 | 0.8872581424 | 1.001485229 |
| TG.in.S.HDL | -0.001446457409 | 0.01108149369 | -0.130529101 | 0.8961478318 | 0.9985545882 |
| TG.to.TLs.ratio.in.M.HDL | 0.001254104688 | 0.01112060341 | 0.1127730791 | 0.9102104619 | 1.001254891 |
| Creatinine | 0.0006170666546 | 0.01201348581 | 0.0513644969 | 0.9590350748 | 1.000617257 |
| Average.diameter.for.VLDL.particles | 0.0004864061959 | 0.01141976802 | 0.04259335171 | 0.9660256953 | 1.000486525 |
| Phenylalanine | -0.0002850401576 | 0.01029700975 | -0.02768183817 | 0.9779159092 | 0.9997150005 |
| Con.of.chylomicrons.and.extremely.L.VLDL.particles | -0.0001107912795 | 0.01112187725 | -0.009961562868 | 0.9920519542 | 0.9998892149 |
| CE.to.TLs.ratio.in.M.HDL | -5.03669e-05 | 0.011149645 | -0.004517354664 | 0.9963956847 | 0.9999496344 |
| Chol.to.TLs.ratio.in.V..L.HDL | 3.03399e-05 | 0.01084086989 | 0.002798657447 | 0.9977669973 | 1.00003034 |
| Chol.in.chylomicrons.and.extremely.L.VLDL | 2.46422e-05 | 0.0111432115 | 0.002211410399 | 0.9982355512 | 1.000024643 |

## Supplementary Table 15. Associations between circulating metabolites and incident diabetic retinopathy (DR) in the UK Biobank

| Metabolite | beta | se | z | p_val | HR |
|---|---|---|---|---|---|
| CE.in.IDL | -0.6824733242 | 0.03463754184 | -19.703284 | 2.02078e-86 | 0.5053655126 |
| Chol.in.IDL | -0.675214474 | 0.03457032864 | -19.5316186 | 5.91351e-85 | 0.5090472315 |
| TLs.in.IDL | -0.6551006567 | 0.03427333728 | -19.11400256 | 1.93076e-81 | 0.5193897799 |
| Con.of.IDL.particles | -0.62020594 | 0.03286130845 | -18.8734402 | 1.88611e-79 | 0.5378336647 |
| PL.in.IDL | -0.6353719739 | 0.03411634021 | -18.62368501 | 2.06502e-77 | 0.5297384028 |
| Free.Chol.in.IDL | -0.6351161862 | 0.03422631792 | -18.55636904 | 7.24382e-77 | 0.5298739207 |
| Free.Chol.in.L.LDL | -0.6255597504 | 0.03378414601 | -18.51637008 | 1.5237e-76 | 0.5349618996 |
| Clinical.LDL_C | -0.5967000091 | 0.03267380823 | -18.26233431 | 1.6508e-74 | 0.550625701 |
| PL.in.L.LDL | -0.5991756398 | 0.03286277327 | -18.23265599 | 2.84179e-74 | 0.5492642411 |
| TC | -0.6193796708 | 0.03406756775 | -18.18091844 | 7.30992e-74 | 0.5382782437 |
| Remnant_C | -0.591472211 | 0.03255756894 | -18.16696486 | 9.42709e-74 | 0.5535117984 |
| Total.esterified.Chol | -0.6222054638 | 0.03429542339 | -18.14252172 | 1.47123e-73 | 0.5367593279 |
| TC.minus.HDL.C | -0.5899766472 | 0.0325483897 | -18.12613935 | 1.98195e-73 | 0.55434023 |
| Total.free.Chol | -0.6031690903 | 0.03346189677 | -18.02554991 | 1.22785e-72 | 0.5470751554 |
| Free.Chol.in.LDL | -0.5945841583 | 0.03309297099 | -17.96708306 | 3.52783e-72 | 0.5517919763 |
| Chol.in.L.LDL | -0.5900784466 | 0.03287614504 | -17.94852912 | 4.92783e-72 | 0.5542838013 |
| CE.in.M.VLDL | -0.5949792341 | 0.03332831912 | -17.85206244 | 2.78557e-71 | 0.5515740197 |
| CE.in.V..S.VLDL | -0.5986249679 | 0.03360602569 | -17.81302477 | 5.60001e-71 | 0.5495667888 |
| TLs.in.L.LDL | -0.5784437873 | 0.03263099688 | -17.72681936 | 2.60353e-70 | 0.5607703659 |
| CE.in.L.LDL | -0.5710064213 | 0.03251230686 | -17.56277781 | 4.74931e-69 | 0.5649565682 |
| Chol.in.M.VLDL | -0.5725006773 | 0.0326363024 | -17.5418364 | 6.86719e-69 | 0.5641130088 |
| LDL_C | -0.5639817838 | 0.03217902979 | -17.52637626 | 9.01339e-69 | 0.568939155 |
| PL.in.LDL | -0.5626012656 | 0.03225595066 | -17.44178219 | 3.97483e-68 | 0.5697251282 |
| TLs.in.LDL | -0.5495468492 | 0.03203012174 | -17.1571889 | 5.55375e-66 | 0.5772113149 |
| CE.in.LDL | -0.5449232455 | 0.03184860836 | -17.10979768 | 1.25434e-65 | 0.5798862905 |
| ApoB | -0.5448792394 | 0.0320000628 | -17.02744282 | 5.14019e-65 | 0.5799118096 |
| Chol.in.V..S.VLDL | -0.5623948071 | 0.0331351366 | -16.97276259 | 1.30639e-64 | 0.569842765 |
| Free.Chol.in.M.LDL | -0.5412789373 | 0.03208803823 | -16.8685581 | 7.66445e-64 | 0.5820034303 |
| Con.of.LDL.particles | -0.5313327318 | 0.03191398718 | -16.64889846 | 3.08223e-62 | 0.5878210396 |
| Con.of.L.LDL.particles | -0.5356212746 | 0.03220862838 | -16.62974493 | 4.24397e-62 | 0.5853055417 |
| Chol.in.S.LDL | -0.5199798761 | 0.03148885409 | -16.51314064 | 2.95117e-61 | 0.5945325121 |
| CE.to.TLs.ratio.in.V..S.VLDL | -0.5910937702 | 0.03627734353 | -16.2937446 | 1.09326e-59 | 0.5537213095 |
| Chol.in.M.LDL | -0.5089277809 | 0.03150585926 | -16.15343282 | 1.07414e-58 | 0.6011397869 |
| CE.in.S.LDL | -0.5028235793 | 0.03120316089 | -16.11450779 | 2.01764e-58 | 0.6048204878 |
| CE.to.TLs.ratio.in.IDL | -0.5383077917 | 0.03347309366 | -16.0818058 | 3.42255e-58 | 0.5837352187 |
| Chol.to.TLs.ratio.in.V..S.VLDL | -0.5784888601 | 0.03611212695 | -16.01924088 | 9.37883e-58 | 0.5607450909 |
| TG.to.TLs.ratio.in.IDL | 0.5710143709 | 0.03568625255 | 16.000962 | 1.25816e-57 | 1.77006164 |
| Free.Chol.in.S.LDL | -0.516329879 | 0.03237281471 | -15.94948983 | 2.87234e-57 | 0.5967065193 |
| Chol.to.TLs.ratio.in.M.LDL | -0.5395152689 | 0.03387741409 | -15.92551508 | 4.21528e-57 | 0.583030797 |
| Chol.to.TLs.ratio.in.IDL | -0.5431688194 | 0.03421019024 | -15.87739839 | 9.087e-57 | 0.5809045511 |
| Chol.to.TLs.ratio.in.L.LDL | -0.5576185407 | 0.03515840235 | -15.86017861 | 1.19553e-56 | 0.572570996 |
| PL.in.M.LDL | -0.5006536798 | 0.03166840169 | -15.80925001 | 2.68646e-56 | 0.6061343124 |
| TLs.in.S.LDL | -0.4924044979 | 0.03119905604 | -15.78267295 | 4.09474e-56 | 0.6111551048 |
| CE.in.VLDL | -0.5099089448 | 0.03236470107 | -15.75509515 | 6.33639e-56 | 0.6005502596 |
| Sphingomyelins | -0.5485364773 | 0.03482638857 | -15.75059889 | 6.80339e-56 | 0.5777948077 |
| Con.of.M.LDL.particles | -0.4967199375 | 0.03158460892 | -15.72664518 | 9.93367e-56 | 0.6085233845 |
| TLs.in.M.LDL | -0.4937803163 | 0.03143810638 | -15.70642679 | 1.36668e-55 | 0.6103148446 |
| Con.of.S.LDL.particles | -0.4905509528 | 0.03129032479 | -15.67740048 | 2.15911e-55 | 0.6122889589 |
| TG.to.TLs.ratio.in.L.LDL | 0.5508540449 | 0.03527668917 | 15.61524218 | 5.7327e-55 | 1.734733926 |
| Free.Chol.in.M.VLDL | -0.4921979648 | 0.03167572422 | -15.53864914 | 1.89945e-54 | 0.6112813416 |
| CE.in.M.LDL | -0.4870671631 | 0.03136811635 | -15.52745972 | 2.26163e-54 | 0.6144257648 |
| Free.Chol.to.TLs.ratio.in.S.VLDL | -0.549809547 | 0.03571667607 | -15.39363702 | 1.8059e-53 | 0.5770597027 |
| Chol.to.TLs.ratio.in.S.LDL | -0.5093062193 | 0.03316185682 | -15.35819367 | 3.12151e-53 | 0.6009123356 |
| TG.to.TLs.ratio.in.M.LDL | 0.5281049981 | 0.03472988656 | 15.20606746 | 3.22333e-52 | 1.695715877 |
| TG.to.TLs.ratio.in.V..S.VLDL | 0.5473715165 | 0.03601306348 | 15.19924893 | 3.57699e-52 | 1.728703173 |
| TLs.in.LP.particles | -0.4775569728 | 0.03154564829 | -15.13860069 | 9.01107e-52 | 0.6202969445 |
| PL.to.TLs.ratio.in.S.VLDL | -0.5393532831 | 0.03572984314 | -15.09531629 | 1.73852e-51 | 0.5831252474 |
| Free.Chol.in.S.VLDL | -0.4833165278 | 0.03203749449 | -15.08596523 | 2.00324e-51 | 0.6167345789 |
| PL.in.S.LDL | -0.4753270128 | 0.03153226148 | -15.07430773 | 2.39008e-51 | 0.6216817253 |
| PL.to.TLs.ratio.in.M.VLDL | -0.5264773151 | 0.03528217898 | -14.92190478 | 2.37399e-50 | 0.5906820959 |
| Total.PL.in.LP.particles | -0.4916446256 | 0.03319641472 | -14.81017241 | 1.25917e-49 | 0.6116196812 |
| TG.to.TLs.ratio.in.S.VLDL | 0.502460828 | 0.03483614386 | 14.423549 | 3.67926e-47 | 1.652783486 |
| PL.in.M.VLDL | -0.4532214735 | 0.03150930757 | -14.3837332 | 6.54658e-47 | 0.6355773545 |
| VLDL_C | -0.4548722064 | 0.03196505977 | -14.23029426 | 5.94312e-46 | 0.6345290515 |
| Free.Chol.in.V..S.VLDL | -0.4591414947 | 0.0323078378 | -14.21145846 | 7.77884e-46 | 0.6318258386 |
| Linoleic.acid | -0.4557004697 | 0.03221925 | -14.143733 | 2.04161e-45 | 0.634003712 |
| Con.of.V..S.VLDL.particles | -0.4469798283 | 0.03203027257 | -13.95491803 | 2.93639e-44 | 0.6395568091 |
| Omega.6.FA | -0.4503755281 | 0.03228057616 | -13.95190488 | 3.06313e-44 | 0.6373887493 |

| Metabolite | beta | se | z | p_val | HR |
|---|---|---|---|---|---|
| Con.of.M.VLDL.particles | -0.4393593774 | 0.03152688926 | -13.93602057 | 3.82691e-44 | 0.6444491375 |
| Chol.to.TLs.ratio.in.S.VLDL | -0.4766419007 | 0.03437082638 | -13.86762993 | 9.95108e-44 | 0.6208648207 |
| Chol.in.S.VLDL | -0.4523032016 | 0.03291397307 | -13.74198127 | 5.68972e-43 | 0.6361612554 |
| Glucose.lactate | 0.6271007902 | 0.0458196636 | 13.68628097 | 1.22627e-42 | 1.872174876 |
| Free.Chol.to.TLs.ratio.in.M.VLDL | -0.4790113842 | 0.03521236447 | -13.60349955 | 3.81722e-42 | 0.6193954333 |
| TG.to.TLs.ratio.in.M.VLDL | 0.4790725941 | 0.03557565954 | 13.46630253 | 2.46925e-41 | 1.61457634 |
| TLs.in.V..S.VLDL | -0.4277948596 | 0.03210080488 | -13.32660851 | 1.62101e-40 | 0.6519451414 |
| Total.cholines | -0.4488895649 | 0.03372784801 | -13.30916709 | 2.04752e-40 | 0.6383365896 |
| Polyunsaturated.FA | -0.4209779631 | 0.0321538109 | -13.09263044 | 3.62822e-39 | 0.6564045663 |
| Chol.to.TLs.ratio.in.M.VLDL | -0.4568394685 | 0.03556073005 | -12.84674043 | 8.97113e-38 | 0.6332819936 |
| PL.in.S.VLDL | -0.4116258312 | 0.03227304269 | -12.75447856 | 2.94338e-37 | 0.6625721435 |
| CE.in.S.VLDL | -0.4274129328 | 0.03366400154 | -12.69643873 | 6.18822e-37 | 0.6521941842 |
| PL.to.TLs.ratio.in.V..S.VLDL | 0.4084309921 | 0.0322938806 | 12.64731846 | 1.15759e-36 | 1.50445543 |
| Glucose | 0.5650258145 | 0.04501082144 | 12.55311049 | 3.82196e-36 | 1.759493203 |
| CE.to.TLs.ratio.in.M.VLDL | -0.4414935979 | 0.03551735796 | -12.43036147 | 1.78824e-35 | 0.6430752076 |
| PC | -0.4110374948 | 0.03340525307 | -12.30457659 | 8.55841e-35 | 0.6629620735 |
| TLs.in.M.VLDL | -0.3785633668 | 0.03120880709 | -12.1300172 | 7.32341e-34 | 0.6848445732 |
| Ratio.of.ApoB.to.ApoA1 | -0.3868987611 | 0.03248008303 | -11.91187722 | 1.0264e-32 | 0.6791598488 |
| Free.Chol.in.VLDL | -0.3745111791 | 0.03168125793 | -11.82122187 | 3.03242e-32 | 0.6876253223 |
| CE.to.TLs.ratio.in.S.VLDL | -0.3937704483 | 0.03347457955 | -11.76326794 | 6.03522e-32 | 0.6745088731 |
| PG | -0.3854610072 | 0.0328862363 | -11.72104353 | 9.94395e-32 | 0.6801370159 |
| PL.in.V..S.VLDL | -0.3718688938 | 0.03189287984 | -11.65993462 | 2.04199e-31 | 0.6894446271 |
| Spectrometer.corrected.alanine | 0.3771987316 | 0.03248148504 | 11.61273049 | 3.55092e-31 | 1.45819407 |
| Free.Chol.to.TLs.ratio.in.L.LDL | -0.4061761242 | 0.03527980517 | -11.51299227 | 1.13472e-30 | 0.6661928244 |
| Con.of.VLDL.particles | -0.3631407919 | 0.03175613615 | -11.43529522 | 2.78588e-30 | 0.6954885075 |
| Total.Con.of.LP.particles | -0.390587543 | 0.03440534014 | -11.35252671 | 7.205e-30 | 0.6766591913 |
| Free.Chol.to.TLs.ratio.in.M.HDL | -0.413891859 | 0.03730709258 | -11.09418693 | 1.33872e-28 | 0.6610724364 |
| PL.to.TLs.ratio.in.V..L.VLDL | -0.4231123268 | 0.03857894184 | -10.96744251 | 5.48005e-28 | 0.6550050544 |
| CE.to.TLs.ratio.in.S.LDL | -0.3366543923 | 0.03149719931 | -10.68839134 | 1.15354e-26 | 0.7141556149 |
| Free.Chol.to.TLs.ratio.in.V..S.VLDL | -0.3441556771 | 0.03235714728 | -10.63615634 | 2.02303e-26 | 0.7088185726 |
| CE.in.V..L.VLDL | -0.359494577 | 0.03390793388 | -10.60207851 | 2.9143e-26 | 0.6980290368 |
| TG.to.TLs.ratio.in.S.LDL | 0.3587950768 | 0.03480755573 | 10.3079653 | 6.48599e-25 | 1.431603403 |
| TG.to.TLs.ratio.in.V..L.VLDL | 0.3816014588 | 0.03719290011 | 10.26006194 | 1.06638e-24 | 1.464628254 |
| PL.to.TLs.ratio.in.IDL | 0.2987519126 | 0.02927342844 | 10.20556623 | 1.87226e-24 | 1.348175117 |
| Free.Chol.to.TLs.ratio.in.L.HDL | -0.3423459975 | 0.03369464823 | -10.16024845 | 2.98315e-24 | 0.7101024685 |
| CE.in.L.VLDL | -0.3441480684 | 0.0339719971 | -10.13034551 | 4.05215e-24 | 0.7088239658 |
| PL.in.VLDL | -0.3199887909 | 0.03174941989 | -10.07857126 | 6.8718e-24 | 0.7261571766 |
| Alanine | 0.302904918 | 0.03150295797 | 9.615126246 | 6.90246e-22 | 1.353785738 |
| Total.FA | -0.286570042 | 0.02983061866 | -9.606573876 | 7.50032e-22 | 0.7508344868 |
| Free.Chol.in.HDL | -0.3480491201 | 0.0363079484 | -9.586031032 | 9.15387e-22 | 0.7060641934 |
| PL.to.TLs.ratio.in.L.HDL | 0.3401468496 | 0.0361792998 | 9.401697975 | 5.36895e-21 | 1.405153922 |
| Free.Chol.in.S.HDL | -0.2951856637 | 0.03187172376 | -9.261678657 | 2.01243e-20 | 0.7443933678 |
| Saturated.FA | -0.2711591845 | 0.0292939 | -9.256506799 | 2.1123e-20 | 0.7624951093 |
| Con.of.HDL.particles | -0.315625953 | 0.0344667637 | -9.157400324 | 5.31626e-20 | 0.7293322036 |
| Con.of.S.HDL.particles | -0.2919955529 | 0.03217999101 | -9.073823321 | 1.14912e-19 | 0.746771857 |
| TLs.in.S.VLDL | -0.2968283496 | 0.03285685693 | -9.033984907 | 1.65532e-19 | 0.7431715671 |
| Con.of.V..L.HDL.particles | -0.3205580321 | 0.03572194272 | -8.973700971 | 2.86715e-19 | 0.7257439356 |
| Chol.in.V..L.VLDL | -0.2911177063 | 0.03348046577 | -8.695151027 | 3.46369e-18 | 0.7474276959 |
| Chol.in.S.HDL | -0.2783614602 | 0.03212662905 | -8.664508801 | 4.53464e-18 | 0.7570231383 |
| TLs.in.VLDL | -0.2693718127 | 0.03160809184 | -8.522242155 | 1.56494e-17 | 0.7638591903 |
| PL.to.TLs.ratio.in.S.LDL | 0.2676002839 | 0.0316651028 | 8.450952636 | 2.88935e-17 | 1.306824677 |
| CE.to.TLs.ratio.in.L.LDL | -0.2510978266 | 0.02983890606 | -8.415115021 | 3.9251e-17 | 0.777946264 |
| Chol.in.V..L.HDL | -0.3048071187 | 0.03623594363 | -8.41173399 | 4.03993e-17 | 0.7372655654 |
| Free.Chol.to.TLs.ratio.in.V..L.VLDL | -0.310901923 | 0.03712946459 | -8.373455596 | 5.59528e-17 | 0.7327857417 |
| CE.to.TLs.ratio.in.M.LDL | -0.2663461645 | 0.03196231175 | -8.33313205 | 7.87313e-17 | 0.7661738594 |
| Chol.in.L.VLDL | -0.2818859471 | 0.03388374442 | -8.319208868 | 8.85524e-17 | 0.7543597165 |
| CE.in.V..L.HDL | -0.3020828094 | 0.03634483637 | -8.311574341 | 9.44407e-17 | 0.7392768433 |
| Chol.to.TLs.ratio.in.V..L.VLDL | -0.3001832845 | 0.03624806467 | -8.281360322 | 1.21776e-16 | 0.7406824526 |
| Ratio.of.linoleic.acid.to.total.FA | -0.2829273259 | 0.03428115972 | -8.253143366 | 1.54282e-16 | 0.7535745512 |
| HDL_C | -0.2982245767 | 0.0365830979 | -8.151977109 | 3.58022e-16 | 0.7421346549 |
| Free.Chol.in.V..L.HDL | -0.2827199215 | 0.03512987238 | -8.047849375 | 8.42616e-16 | 0.7537308621 |
| PL.to.TLs.ratio.in.chylomicrons.and.extremely.L.VLDL | 0.2727029826 | 0.03399148864 | 8.022684312 | 1.03459e-15 | 1.313510052 |
| Con.of.S.VLDL.particles | -0.2640332045 | 0.03293579597 | -8.016603113 | 1.0871e-15 | 0.7679480399 |
| CE.in.S.HDL | -0.2570152855 | 0.03216983576 | -7.989325386 | 1.35679e-15 | 0.7733563925 |
| CE.to.TLs.ratio.in.L.VLDL | -0.28339695 | 0.03547385725 | -7.988895822 | 1.36153e-15 | 0.7532207376 |
| Free.Chol.to.TLs.ratio.in.S.HDL | -0.2633971794 | 0.03307110566 | -7.964571312 | 1.65798e-15 | 0.7684366295 |
| CE.to.TLs.ratio.in.V..L.VLDL | -0.2810602285 | 0.03580962556 | -7.84873408 | 4.20258e-15 | 0.7549828626 |
| Chol.to.TLs.ratio.in.L.VLDL | -0.2712881958 | 0.03533597216 | -7.677394429 | 1.62357e-14 | 0.7623967452 |
| CE.in.HDL | -0.275403775 | 0.03649356254 | -7.546639896 | 4.46631e-14 | 0.7592654889 |
| ApoA1 | -0.265817445 | 0.03548944354 | -7.490042627 | 6.88512e-14 | 0.7665790576 |

| Metabolite | beta | se | z | p_val | HR |
|---|---|---|---|---|---|
| Free.Chol.in.L.HDL | -0.2736560633 | 0.03696088227 | -7.403937527 | 1.32205e-13 | 0.7605936263 |
| TLs.in.HDL | -0.2685038405 | 0.03627557266 | -7.401780889 | 1.3437e-13 | 0.7645224867 |
| Chol.to.TLs.ratio.in.L.HDL | -0.2644659027 | 0.03639695524 | -7.266154571 | 3.69865e-13 | 0.7676158221 |
| Free.Chol.in.M.HDL | -0.2582731192 | 0.03577248452 | -7.21988206 | 5.20327e-13 | 0.7723842503 |
| TLs.in.S.HDL | -0.2292229207 | 0.03197147509 | -7.169607284 | 7.52132e-13 | 0.7951512581 |
| TLs.in.V..L.HDL | -0.2558005529 | 0.03610461202 | -7.084982737 | 1.39062e-12 | 0.7742963846 |
| TG.to.TLs.ratio.in.L.VLDL | 0.2529657132 | 0.03639100998 | 6.951324334 | 3.61873e-12 | 1.28783912 |
| Con.of.L.HDL.particles | -0.2520749849 | 0.03697701924 | -6.817071524 | 9.2915e-12 | 0.7771864587 |
| Free.Chol.in.V..L.VLDL | -0.2190602578 | 0.03333651096 | -6.57118131 | 4.99176e-11 | 0.8032733132 |
| Free.Chol.in.L.VLDL | -0.2218635383 | 0.03405042769 | -6.51573426 | 7.23349e-11 | 0.8010246661 |
| Chol.in.L.HDL | -0.2414694155 | 0.03706178517 | -6.515320683 | 7.25345e-11 | 0.7854728267 |
| PL.in.HDL | -0.2328639054 | 0.03596469124 | -6.474792286 | 9.49425e-11 | 0.7922613887 |
| PL.in.S.HDL | -0.205076595 | 0.0318938238 | -6.429978301 | 1.27622e-10 | 0.8145849209 |
| Free.Chol.to.TLs.ratio.in.IDL | -0.1941216031 | 0.03062134453 | -6.339421278 | 2.3063e-10 | 0.823557751 |
| CE.in.L.HDL | -0.2282171004 | 0.03703516304 | -6.162173504 | 7.17532e-10 | 0.7959514397 |
| TG.in.M.VLDL | -0.1995981064 | 0.03247170506 | -6.146831712 | 7.90459e-10 | 0.8190598618 |
| TLs.in.L.VLDL | -0.2030562397 | 0.03322213341 | -6.112077065 | 9.83427e-10 | 0.8162323355 |
| TLs.in.L.HDL | -0.2270603578 | 0.03760601268 | -6.037873775 | 1.56158e-09 | 0.7968726834 |
| X3.Hydroxybutyrate | 0.2223165237 | 0.03714766396 | 5.984670367 | 2.16829e-09 | 1.248966643 |
| Con.of.M.HDL.particles | -0.2096487667 | 0.03533659024 | -5.932908787 | 2.97614e-09 | 0.8108690001 |
| Con.of.L.VLDL.particles | -0.195389869 | 0.0334071457 | -5.84874478 | 4.95296e-09 | 0.8225139229 |
| PL.in.V..L.VLDL | -0.1928072049 | 0.03363632561 | -5.732112573 | 9.91874e-09 | 0.8246409455 |
| PL.in.V..L.HDL | -0.202437555 | 0.03607784208 | -5.611132575 | 2.01007e-08 | 0.8167374822 |
| Tyrosine | -0.178571343 | 0.03188812224 | -5.599932841 | 2.14435e-08 | 0.8364643789 |
| CE.to.TLs.ratio.in.L.HDL | -0.1989709074 | 0.03577498924 | -5.561732139 | 2.6711e-08 | 0.8195737365 |
| Chol.in.M.HDL | -0.1961167336 | 0.03565828395 | -5.499892644 | 3.80023e-08 | 0.8219162838 |
| CE.to.TLs.ratio.in.V..L.HDL | -0.1573474644 | 0.02901823956 | -5.422364238 | 5.88159e-08 | 0.8544071311 |
| PL.to.TLs.ratio.in.M.LDL | 0.1749970815 | 0.03232544219 | 5.413602093 | 6.17693e-08 | 1.19124274 |
| Monounsaturated.FA | -0.1652282983 | 0.03052124625 | -5.413550185 | 6.17873e-08 | 0.8477001535 |
| Free.Chol.to.TLs.ratio.in.S.LDL | -0.1782655602 | 0.03328658085 | -5.355478262 | 8.53304e-08 | 0.8367201944 |
| PL.in.L.VLDL | -0.1821058967 | 0.03409469408 | -5.341179957 | 9.23435e-08 | 0.8335130695 |
| TG.in.L.VLDL | -0.1709052326 | 0.03268035461 | -5.229601534 | 1.69876e-07 | 0.8429014492 |
| Ratio.of.monounsaturated.FA.to.total.FA | 0.1801977563 | 0.03460610074 | 5.207109512 | 1.91805e-07 | 1.197454144 |
| TLs.in.M.HDL | -0.1806377563 | 0.03483254158 | -5.185890783 | 2.14985e-07 | 0.8347376824 |
| PL.in.L.HDL | -0.1933746205 | 0.03804044841 | -5.083394875 | 3.70748e-07 | 0.8241731641 |
| PL.to.TLs.ratio.in.M.HDL | 0.1801749464 | 0.03563102077 | 5.056687755 | 4.26601e-07 | 1.19742683 |
| TLs.in.V..L.VLDL | -0.1698770903 | 0.03363371747 | -5.050797328 | 4.3997e-07 | 0.8437685175 |
| Average.diameter.for.LDL.particles | -0.1676095159 | 0.03329190946 | -5.034541984 | 4.78993e-07 | 0.8456839964 |
| CE.in.M.HDL | -0.1787072176 | 0.0355234445 | -5.030683823 | 4.88734e-07 | 0.8363507323 |
| Free.Chol.to.TLs.ratio.in.M.LDL | -0.1672534092 | 0.03391476991 | -4.931580244 | 8.1567e-07 | 0.8459852037 |
| Docosahexaenoic.acid | -0.1580330005 | 0.03234214861 | -4.886286389 | 1.02756e-06 | 0.8538216049 |
| Chol.to.TLs.ratio.in.S.HDL | -0.1580225114 | 0.03289023179 | -4.804542347 | 1.55106e-06 | 0.8538305608 |
| Con.of.V..L.VLDL.particles | -0.1591518433 | 0.03375511032 | -4.714896256 | 2.41834e-06 | 0.852866847 |
| PL.in.M.HDL | -0.1606488001 | 0.03422720097 | -4.693600282 | 2.68438e-06 | 0.8515910973 |
| CE.in.chylomicrons.and.extremely.L.VLDL | -0.1563289789 | 0.03383437105 | -4.620419238 | 3.82965e-06 | 0.8552777758 |
| TG.in.L.LDL | -0.1449454448 | 0.03192433692 | -4.540280512 | 5.61794e-06 | 0.8650694859 |
| Omega.3.FAs | -0.1441589927 | 0.03278716298 | -4.396812034 | 1.09852e-05 | 0.8657500891 |
| Free.Chol.to.TLs.ratio.in.L.VLDL | -0.1557624598 | 0.03555651139 | -4.380701415 | 1.18298e-05 | 0.8557624443 |
| TG.in.LDL | -0.1367280766 | 0.03175451879 | -4.305783296 | 1.66396e-05 | 0.8722073675 |
| TG.in.V..L.HDL | -0.1282416756 | 0.03024073465 | -4.240693127 | 2.22831e-05 | 0.8796407657 |
| Valine | 0.1473219154 | 0.03518575531 | 4.186976068 | 2.82696e-05 | 1.158726915 |
| Free.Chol.to.TLs.ratio.in.V..L.HDL | 0.149106763 | 0.03562707164 | 4.185209622 | 2.84903e-05 | 1.160796913 |
| TG.in.VLDL | -0.1377086872 | 0.03306686046 | -4.164552827 | 3.11963e-05 | 0.8713524909 |
| Acetone | 0.138453351 | 0.03410752185 | 4.059320158 | 4.92158e-05 | 1.148496104 |
| TG | -0.1317556957 | 0.03293774031 | -4.000143742 | 6.3304e-05 | 0.8765551151 |
| TG.in.IDL | -0.1248760853 | 0.03231743378 | -3.864047069 | 0.0001115237168 | 0.8826062637 |
| CE.to.TLs.ratio.in.chylomicrons.and.extremely.L.VLDL | -0.1372957553 | 0.03574721238 | -3.840740191 | 0.0001226638853 | 0.8717123744 |
| PL.to.TLs.ratio.in.S.HDL | 0.1253257843 | 0.03282776653 | 3.81767624 | 0.0001347145497 | 1.133517675 |
| TG.in.M.LDL | -0.1202053527 | 0.03165586047 | -3.797254313 | 0.0001463076681 | 0.8867383239 |
| Average.diameter.for.HDL.particles | -0.1406430008 | 0.03705958449 | -3.795050666 | 0.0001476133085 | 0.868799417 |
| TG.in.S.LDL | -0.119583872 | 0.03169325488 | -3.773164744 | 0.0001611897732 | 0.887289586 |
| Ratio.of.polyunsaturated.FA.to.monounsaturated.FA | -0.1281160149 | 0.03431681819 | -3.733330234 | 0.0001889645755 | 0.879751309 |
| Chol.in.chylomicrons.and.extremely.L.VLDL | -0.1242486137 | 0.03402846803 | -3.651313764 | 0.0002609022585 | 0.8831602479 |
| Albumin | -0.1173607946 | 0.03229217124 | -3.634342013 | 0.0002786909675 | 0.8892642935 |
| Total.Con.of.branched.chain.AAs | 0.1264590271 | 0.03485665919 | 3.627973249 | 0.0002856548497 | 1.134802954 |
| TG.to.TLs.ratio.in.L.HDL | 0.1199787553 | 0.03455795805 | 3.471812632 | 0.0005169570089 | 1.127472899 |
| Citrate | 0.1106828278 | 0.03267057782 | 3.387844208 | 0.0007044426495 | 1.117040556 |
| Ratio.of.saturated.FA.to.total.FA | -0.09869467247 | 0.02965277914 | -3.328344773 | 0.0008736367521 | 0.9060192984 |
| Degree.of.unsaturation | -0.106906187 | 0.03255789253 | -3.283572082 | 0.001025004283 | 0.8986099704 |
| TG.to.TLs.ratio.in.S.HDL | 0.1160349655 | 0.03533994516 | 3.283394044 | 0.001025651921 | 1.123035139 |

| Metabolite | beta | se | z | p_val | HR |
|---|---|---|---|---|---|
| TG.in.V..L.VLDL | -0.1090200292 | 0.03414881166 | -3.192498477 | 0.001410476883 | 0.8967124569 |
| Chol.to.TLs.ratio.in.M.HDL | -0.1116060006 | 0.03530332828 | -3.161345009 | 0.001570423689 | 0.8943965798 |
| Lactate | 0.1013112186 | 0.03359783176 | 3.015409426 | 0.00256632575 | 1.106620989 |
| TG.to.TLs.ratio.in.V..L.HDL | 0.1009204677 | 0.0345451537 | 2.921407401 | 0.003484538414 | 1.106188661 |
| Isoleucine | 0.09893801164 | 0.03409117912 | 2.902158687 | 0.003706007589 | 1.103997862 |
| CE.to.TLs.ratio.in.S.HDL | -0.0906114494 | 0.03227939379 | -2.807098857 | 0.004998989803 | 0.9133725334 |
| TG.in.chylomicrons.and.extremely.L.VLDL | -0.08883470849 | 0.03259028862 | -2.725803061 | 0.006414525878 | 0.9149968023 |
| TLs.in.chylomicrons.and.extremely.L.VLDL | -0.09001520789 | 0.03333027635 | -2.700703917 | 0.006919290594 | 0.9139172864 |
| Leucine | 0.09043728316 | 0.03355628487 | 2.695092246 | 0.00703691466 | 1.094652852 |
| TG.in.L.HDL | -0.08761505924 | 0.03252314678 | -2.693929337 | 0.00706151339 | 0.9161134583 |
| Free.Chol.to.TLs.ratio.in.chylomicrons.and.extremely.L.VLDL | 0.08782349818 | 0.03376371908 | 2.601120391 | 0.009291983867 | 1.091795401 |
| Free.Chol.in.chylomicrons.and.extremely.L.VLDL | -0.08241962839 | 0.03373370723 | -2.443242536 | 0.01455595143 | 0.9208854479 |
| Con.of.chylomicrons.and.extremely.L.VLDL.particles | -0.07852060172 | 0.03346630044 | -2.346258794 | 0.01896293565 | 0.9244830138 |
| Glycine | 0.08135461357 | 0.03487580136 | 2.332695176 | 0.01966414958 | 1.084755497 |
| Pyruvate | 0.07808199963 | 0.03445606254 | 2.266132398 | 0.02344327816 | 1.081211314 |
| TG.in.M.HDL | -0.05920978563 | 0.03311343279 | -1.788089625 | 0.07376154914 | 0.9425090236 |
| TG.in.HDL | -0.05719689953 | 0.03285856907 | -1.740699646 | 0.08173624046 | 0.9444080975 |
| Ratio.of.TG.to.PG | 0.06043192957 | 0.03524222643 | 1.714759131 | 0.08638939734 | 1.062295284 |
| Ratio.of.omega.6.FA.to.total.FA | -0.05526511916 | 0.03294747494 | -1.677370398 | 0.09347007418 | 0.9462342499 |
| TG.in.S.VLDL | -0.05626154988 | 0.03369091656 | -1.669932303 | 0.09493275834 | 0.9452918626 |
| Histidine | -0.05214918641 | 0.03149132421 | -1.655985822 | 0.09772468999 | 0.9491872504 |
| Ratio.of.polyunsaturated.FA.to.total.FA | -0.05341209828 | 0.03303156491 | -1.61700175 | 0.1058779031 | 0.9479892672 |
| TG.to.TLs.ratio.in.M.HDL | 0.05594728646 | 0.03478704622 | 1.608279303 | 0.1077740214 | 1.057541935 |
| PL.in.chylomicrons.and.extremely.L.VLDL | -0.05522049116 | 0.03440177229 | -1.605164138 | 0.1084576874 | 0.9462764794 |
| Average.diameter.for.VLDL.particles | -0.05040991496 | 0.0327242657 | -1.540444496 | 0.1234520424 | 0.9508395812 |
| Phenylalanine | -0.04940489431 | 0.03229707699 | -1.529701722 | 0.1260905771 | 0.951795675 |
| Acetoacetate | 0.05259513796 | 0.03525346084 | 1.491914175 | 0.1357216461 | 1.054002833 |
| TG.in.V..S.VLDL | -0.04890235497 | 0.03296423245 | -1.483497456 | 0.1379422919 | 0.95227411 |
| Ratio.of.omega.6.FA.to.omega.3.FA | -0.04590959946 | 0.03253449102 | -1.411105508 | 0.1582135063 | 0.9551283024 |
| Chol.to.TLs.ratio.in.chylomicrons.and.extremely.L.VLDL | -0.04471844181 | 0.03458979289 | -1.292821901 | 0.1960726638 | 0.9562666887 |
| Creatinine | 0.04025375839 | 0.03562107092 | 1.130054694 | 0.2584531788 | 1.041074922 |
| Ratio.of.docosahexaenoic.acid.to.total.FA | 0.03493471357 | 0.03139799729 | 1.112641461 | 0.2658624463 | 1.035552099 |
| TG.to.TLs.ratio.in.chylomicrons.and.extremely.L.VLDL | -0.03714452492 | 0.0346644528 | -1.071545111 | 0.2839243984 | 0.9635368702 |
| Glutamine | -0.02797147323 | 0.03132099799 | -0.8930581727 | 0.3718260208 | 0.9724161063 |
| Chol.to.TLs.ratio.in.V..L.HDL | -0.02426859379 | 0.02794850343 | -0.8683324975 | 0.3852123367 | 0.9760235207 |
| CE.to.TLs.ratio.in.M.HDL | -0.02776318475 | 0.03398143866 | -0.817010281 | 0.4139225527 | 0.9726186705 |
| Ratio.of.omega.3.FA.to.total.FA | 0.02456651698 | 0.03322428548 | 0.7394144562 | 0.4596553667 | 1.02487076 |
| Glycoprotein.acetyls | 0.02010296464 | 0.03408025848 | 0.5898712493 | 0.5552769707 | 1.02030639 |
| TG.in.S.HDL | -0.01453809154 | 0.03484536932 | -0.4172173181 | 0.6765194591 | 0.9855670763 |
| PL.to.TLs.ratio.in.L.LDL | -0.011353819 | 0.02891618961 | -0.3926457514 | 0.6945811431 | 0.9887103924 |
| PL.to.TLs.ratio.in.L.VLDL | -0.011709588 | 0.03605235506 | -0.3247939832 | 0.7453369986 | 0.9883587024 |
| PL.to.TLs.ratio.in.V..L.HDL | -0.005636943859 | 0.03384021711 | -0.1665752864 | 0.8677042405 | 0.9943789139 |
| Acetate | -0.001320918127 | 0.03290444398 | -0.04014406466 | 0.9679782716 | 0.9986799539 |

## Supplementary Table 16. Associations between circulating metabolites and incident glaucoma in the UK Biobank

| Metabolite | beta | se | z | p_val | HR |
|---|---|---|---|---|---|
| Lactate | 0.05832668942 | 0.01994182233 | 2.924842497 | 0.003446306038 | 1.06006125 |
| Average.diameter.for.HDL.particles | 0.06571738486 | 0.02328889412 | 2.82183364 | 0.004774994006 | 1.067924863 |
| Creatinine | -0.06338879531 | 0.02289647845 | -2.768495402 | 0.005631578027 | 0.9385784878 |
| PL.to.TLs.ratio.in.V..L.HDL | 0.06116708077 | 0.02210538145 | 2.767067418 | 0.00565630565 | 1.063076519 |
| Chol.to.TLs.ratio.in.V..L.HDL | -0.05382488252 | 0.02032097176 | -2.648735659 | 0.008079349874 | 0.947598033 |
| Free.Chol.to.TLs.ratio.in.V..L.HDL | -0.06065820772 | 0.02300760843 | -2.636441241 | 0.008378070879 | 0.9411448609 |
| PL.in.L.HDL | 0.06008334052 | 0.02334713824 | 2.573477738 | 0.01006821239 | 1.061925044 |
| TLs.in.L.HDL | 0.05996396403 | 0.02347248582 | 2.554649068 | 0.01062949042 | 1.061798283 |
| Con.of.L.HDL.particles | 0.0576467876 | 0.02352781303 | 2.450154952 | 0.01427947496 | 1.059340757 |
| Chol.in.L.HDL | 0.05704262627 | 0.02345487387 | 2.432015903 | 0.01501504803 | 1.058700938 |
| CE.in.L.HDL | 0.05652018525 | 0.02344647989 | 2.410604301 | 0.01592161687 | 1.058147974 |
| Free.Chol.in.L.HDL | 0.05633062047 | 0.02338514281 | 2.408820888 | 0.01600415019 | 1.057947405 |
| Average.diameter.for.VLDL.particles | -0.05029791546 | 0.02160794427 | -2.327751073 | 0.01992532546 | 0.9509460808 |
| PL.in.V..L.HDL | 0.05115102935 | 0.02287096738 | 2.236504844 | 0.02531872033 | 1.052481837 |
| Citrate | -0.04322734535 | 0.01992065839 | -2.169975736 | 0.03000868412 | 0.9576936381 |
| Con.of.M.LDL.particles | -0.04135123585 | 0.01946335493 | -2.12456876 | 0.03362261291 | 0.9594920627 |
| Ratio.of.TG.to.PG | -0.0467876999 | 0.02218740579 | -2.108750358 | 0.0349661356 | 0.9542899719 |
| TG.in.L.VLDL | -0.04347163586 | 0.02091091738 | -2.078896639 | 0.03762685169 | 0.9574597112 |
| Ratio.of.ApoB.to.ApoA1 | -0.04121034289 | 0.01990189971 | -2.07067383 | 0.03838928744 | 0.9596272579 |
| TG.to.TLs.ratio.in.L.HDL | -0.04453950321 | 0.02161132901 | -2.060933097 | 0.03930942327 | 0.956437817 |
| Con.of.L.VLDL.particles | -0.04314898607 | 0.02101072575 | -2.053664713 | 0.04000815406 | 0.9577686852 |
| TLs.in.L.VLDL | -0.04301172135 | 0.02095600555 | -2.052477093 | 0.04012331906 | 0.9579001621 |
| CE.in.M.LDL | -0.04011337783 | 0.01957178409 | -2.049551418 | 0.04040822536 | 0.9606805131 |
| TLs.in.V..L.HDL | 0.04666834606 | 0.02284463484 | 2.042858045 | 0.04106649259 | 1.047774453 |
| TG.to.TLs.ratio.in.V..L.HDL | -0.04440188571 | 0.02185422226 | -2.031730307 | 0.04218096599 | 0.9565694486 |
| Chol.in.M.LDL | -0.03921268735 | 0.01949144645 | -2.011789502 | 0.0442421311 | 0.9615461787 |
| Con.of.V..L.VLDL.particles | -0.04268093582 | 0.0212224977 | -2.011117467 | 0.04431305058 | 0.958217074 |
| TLs.in.M.LDL | -0.03874886194 | 0.01951769006 | -1.985320077 | 0.04710887164 | 0.9619922717 |
| Free.Chol.in.L.VLDL | -0.04169828587 | 0.02107569722 | -1.978500898 | 0.04787222738 | 0.9591591288 |
| PL.to.TLs.ratio.in.S.HDL | 0.03993372955 | 0.02026573652 | 1.970504724 | 0.04878055408 | 1.040741801 |
| PL.in.M.LDL | -0.03818904627 | 0.01949287945 | -1.959128017 | 0.05009779647 | 0.9625309608 |
| TG.in.M.VLDL | -0.04008041919 | 0.02062815879 | -1.942995475 | 0.05201670503 | 0.9607121764 |
| CE.in.V..L.HDL | 0.0438118789 | 0.02301067009 | 1.903981011 | 0.05691265735 | 1.04478579 |
| Chol.in.L.VLDL | -0.03929281037 | 0.02065211611 | -1.902604564 | 0.05709216313 | 0.9614691398 |
| PL.in.L.VLDL | -0.04015771403 | 0.02111769649 | -1.901614319 | 0.05722159457 | 0.9606379211 |
| CE.in.HDL | 0.04299629345 | 0.02262876114 | 1.900072796 | 0.05742356715 | 1.043934025 |
| PL.in.HDL | 0.04201921001 | 0.02214014805 | 1.89787394 | 0.05771268998 | 1.042914513 |
| TLs.in.HDL | 0.04238796123 | 0.02238254033 | 1.893795816 | 0.05825211724 | 1.04329916 |
| HDL_C | 0.04291398913 | 0.02271336021 | 1.889372102 | 0.05884198636 | 1.043848109 |
| TLs.in.V..L.VLDL | -0.04010989528 | 0.02127480067 | -1.88532414 | 0.05938608891 | 0.9606838587 |
| PL.in.V..L.VLDL | -0.03956698245 | 0.02117885099 | -1.868230834 | 0.06172990264 | 0.9612055679 |
| TG.in.V..L.VLDL | -0.03998473462 | 0.02143956069 | -1.864997851 | 0.06218170545 | 0.9608041061 |
| TG.in.VLDL | -0.03932865249 | 0.02109370707 | -1.864473246 | 0.06225527527 | 0.9614346793 |
| TLs.in.M.VLDL | -0.03609578378 | 0.01983884377 | -1.819449974 | 0.06884280845 | 0.964547901 |
| TLs.in.S.LDL | -0.03527653341 | 0.01939324645 | -1.819011247 | 0.06890971415 | 0.965338431 |
| Chol.in.S.LDL | -0.03528213054 | 0.0194025648 | -1.818426115 | 0.06899902987 | 0.9653330279 |
| Free.Chol.in.V..L.VLDL | -0.03806911483 | 0.02102248777 | -1.810875822 | 0.07016007596 | 0.9626464055 |
| TLs.in.VLDL | -0.03686801792 | 0.02046538103 | -1.801482116 | 0.07162692357 | 0.9638033317 |
| Ratio.of.omega.6.FA.to.total.FA | 0.03847098207 | 0.02143363907 | 1.794888024 | 0.07267154243 | 1.039220572 |
| Ratio.of.polyunsaturated.FA.to.total.FA | 0.03801927034 | 0.02125651725 | 1.788593583 | 0.07368029211 | 1.03875125 |
| CE.in.M.HDL | 0.03850366345 | 0.02165807947 | 1.777796757 | 0.07543724124 | 1.039254536 |
| Chol.in.V..L.VLDL | -0.03669839832 | 0.02073262688 | -1.770079524 | 0.07671389406 | 0.9639668255 |
| CE.in.L.VLDL | -0.03584359079 | 0.02025152318 | -1.769920735 | 0.07674034611 | 0.9647911839 |
| Chol.in.M.HDL | 0.03843440133 | 0.02174383605 | 1.76759985 | 0.07712782319 | 1.039182557 |
| CE.in.S.LDL | -0.03433676478 | 0.01947028535 | -1.763547075 | 0.07780826506 | 0.9662460522 |
| Free.Chol.in.M.LDL | -0.03434874833 | 0.01948391492 | -1.76292847 | 0.07791255459 | 0.9662344732 |
| Chol.in.V..L.HDL | 0.04008888798 | 0.02277559091 | 1.760168951 | 0.07837916467 | 1.040903294 |
| Con.of.M.VLDL.particles | -0.0345062248 | 0.01966854769 | -1.754386005 | 0.0793643878 | 0.966082326 |
| TLs.in.M.HDL | 0.0374194272 | 0.02140639141 | 1.748049285 | 0.08045549361 | 1.038128349 |
| PL.in.S.LDL | -0.03372683598 | 0.01931706158 | -1.745960991 | 0.08081773045 | 0.9668355733 |
| PL.in.M.HDL | 0.03689073015 | 0.02119532237 | 1.740512812 | 0.0817690123 | 1.037579638 |
| Con.of.S.VLDL.particles | -0.03509186096 | 0.02021071075 | -1.736300193 | 0.08251076922 | 0.9655167189 |
| Free.Chol.in.HDL | 0.03953330597 | 0.02276947385 | 1.736241524 | 0.08252113797 | 1.040325147 |
| Free.Chol.in.S.LDL | -0.03369088958 | 0.01942511103 | -1.734398817 | 0.08284734486 | 0.9668703281 |
| TG | -0.03639064633 | 0.02099465061 | -1.733329456 | 0.08303712808 | 0.9642635339 |
| Con.of.M.HDL.particles | 0.03728407381 | 0.02163538595 | 1.723291366 | 0.0848358484 | 1.037987844 |
| CE.in.V..L.VLDL | -0.03513873535 | 0.0204470294 | -1.718525203 | 0.08570086143 | 0.9654714619 |
| Con.of.S.LDL.particles | -0.03295614234 | 0.01939428713 | -1.699270621 | 0.08926820542 | 0.9675809945 |

| Metabolite | beta | se | z | p_val | HR |
|---|---|---|---|---|---|
| Ratio.of.saturated.FA.to.total.FA | -0.03347008682 | 0.01977284409 | -1.692730022 | 0.09050686792 | 0.9670838394 |
| Chol.to.TLs.ratio.in.L.HDL | 0.03769843914 | 0.02231880887 | 1.68908831 | 0.09120250782 | 1.038418039 |
| CE.to.TLs.ratio.in.L.HDL | 0.0372278315 | 0.02216520756 | 1.679561601 | 0.09304264397 | 1.037929467 |
| CE.in.LDL | -0.03271248695 | 0.01950570647 | -1.677072655 | 0.09352827521 | 0.9678167795 |
| Free.Chol.in.VLDL | -0.03342906896 | 0.02000381445 | -1.671134725 | 0.09469506984 | 0.9671235079 |
| PL.in.M.VLDL | -0.03264777725 | 0.01958541243 | -1.666943567 | 0.09552562681 | 0.9678794087 |
| Free.Chol.in.M.HDL | 0.03650770525 | 0.02198145293 | 1.660841318 | 0.09674532047 | 1.037182296 |
| PL.in.VLDL | -0.0333851661 | 0.02020113716 | -1.652637959 | 0.09840456777 | 0.9671659683 |
| Con.of.V..L.HDL.particles | 0.03750042801 | 0.0227083004 | 1.65139739 | 0.09865745743 | 1.038212441 |
| Degree.of.unsaturation | 0.03387682713 | 0.02066824731 | 1.639075952 | 0.1011974427 | 1.034457182 |
| LDL_C | -0.03192227218 | 0.01952068248 | -1.635305129 | 0.1019850989 | 0.9685818649 |
| TLs.in.LDL | -0.03178959627 | 0.01949864606 | -1.630348906 | 0.1030277768 | 0.9687103809 |
| TLs.in.S.VLDL | -0.03272714151 | 0.02014279354 | -1.624756836 | 0.1042143815 | 0.9678025967 |
| PL.in.LDL | -0.03157913244 | 0.01945332841 | -1.623327987 | 0.1045193099 | 0.9689142809 |
| Free.Chol.in.M.VLDL | -0.03120077953 | 0.01948457224 | -1.601306877 | 0.1093089666 | 0.9692809418 |
| PL.in.S.VLDL | -0.03098948276 | 0.01970939085 | -1.572320677 | 0.1158762 | 0.9694857693 |
| Con.of.VLDL.particles | -0.03125502613 | 0.01989744061 | -1.570806354 | 0.1162276358 | 0.969228363 |
| Con.of.chylomicrons.and.extremely.L.VLDL.particles | -0.03268297334 | 0.02107968957 | -1.550448513 | 0.1210339023 | 0.9678453437 |
| TG.in.S.VLDL | -0.03205797071 | 0.0208093764 | -1.540554128 | 0.1234253395 | 0.9684504387 |
| Con.of.LDL.particles | -0.02971791731 | 0.01937811187 | -1.533581678 | 0.1251325987 | 0.970719318 |
| ApoA1 | 0.03324781731 | 0.02180225385 | 1.524971571 | 0.1272661886 | 1.033806703 |
| CE.to.TLs.ratio.in.M.LDL | -0.0305496825 | 0.020072246 | -1.521986254 | 0.1280125249 | 0.9699122432 |
| Free.Chol.to.TLs.ratio.in.chylomicrons.and.extremely.L.VLDL | 0.03038628859 | 0.02022962653 | 1.502068688 | 0.1330793704 | 1.030852664 |
| Clinical.LDL_C | -0.02916636848 | 0.01952233908 | -1.493999687 | 0.135175701 | 0.9712548648 |
| Free.Chol.in.S.VLDL | -0.02868406244 | 0.01948038127 | -1.472458985 | 0.1408969748 | 0.9717234199 |
| Free.Chol.in.LDL | -0.02891908796 | 0.01965334129 | -1.471459104 | 0.1411669997 | 0.9714950669 |
| CE.to.TLs.ratio.in.M.HDL | 0.03091356514 | 0.02111721802 | 1.4639033 | 0.1432203716 | 1.031396351 |
| CE.in.L.LDL | -0.02871842946 | 0.01964052601 | -1.462202664 | 0.1436856818 | 0.9716900252 |
| TG.to.TLs.ratio.in.M.HDL | -0.03087002266 | 0.02116267116 | -1.458701618 | 0.1446472507 | 0.9696015911 |
| Chol.to.TLs.ratio.in.M.HDL | 0.03141642999 | 0.02161171046 | 1.45367624 | 0.1460360887 | 1.031915135 |
| Chol.in.S.VLDL | -0.02834569245 | 0.01962963031 | -1.444025792 | 0.1487317205 | 0.9720522776 |
| Ratio.of.polyunsaturated.FA.to.monounsaturated.FA | 0.03124999628 | 0.02171433618 | 1.439141221 | 0.150110515 | 1.031743404 |
| Chol.in.L.LDL | -0.02793215322 | 0.01968198034 | -1.419173921 | 0.155848317 | 0.9724543425 |
| VLDL_C | -0.02775404104 | 0.01967730952 | -1.410459139 | 0.1584041536 | 0.9726275638 |
| TLs.in.L.LDL | -0.02764124356 | 0.01963694401 | -1.407614318 | 0.1592453053 | 0.97273728 |
| ApoB | -0.02727816875 | 0.01939113285 | -1.406734148 | 0.1595062359 | 0.9730905205 |
| Average.diameter.for.LDL.particles | 0.02880230397 | 0.02049169821 | 1.40555964 | 0.1598549277 | 1.029221101 |
| CE.in.S.VLDL | -0.02772562421 | 0.01974433489 | -1.404231866 | 0.1602498158 | 0.9726552032 |
| Chol.to.TLs.ratio.in.M.LDL | -0.02734252492 | 0.01949810271 | -1.402317207 | 0.1608205445 | 0.9730278981 |
| TLs.in.chylomicrons.and.extremely.L.VLDL | -0.02919225732 | 0.02109270727 | -1.383997651 | 0.1663591739 | 0.9712297205 |
| CE.to.TLs.ratio.in.L.LDL | -0.02680915293 | 0.01944407811 | -1.378782412 | 0.167961851 | 0.9735470224 |
| Chol.to.TLs.ratio.in.V..L.VLDL | 0.02912400294 | 0.02115061581 | 1.376981323 | 0.1685180222 | 1.029552254 |
| TG.in.chylomicrons.and.extremely.L.VLDL | -0.02857798858 | 0.02080764683 | -1.373436834 | 0.1696165856 | 0.9718264998 |
| TC.minus.HDL.C | -0.02665120478 | 0.01950109932 | -1.366651405 | 0.1717345965 | 0.9737008045 |
| PL.in.L.LDL | -0.0267147258 | 0.01957403541 | -1.364804203 | 0.1723145974 | 0.973638956 |
| CE.to.TLs.ratio.in.V..L.VLDL | 0.0284080217 | 0.02114285211 | 1.343622968 | 0.1790703385 | 1.028815378 |
| TG.to.TLs.ratio.in.S.HDL | -0.02884693919 | 0.02147034193 | -1.343571485 | 0.1790869955 | 0.9715651616 |
| TG.in.S.HDL | -0.02751925784 | 0.02119032124 | -1.298671102 | 0.1940568255 | 0.9728559473 |
| TG.in.S.LDL | -0.0264732218 | 0.02071745842 | -1.277821887 | 0.2013122353 | 0.9738741221 |
| Free.Chol.in.L.LDL | -0.02524519522 | 0.01983207284 | -1.272947887 | 0.2030365348 | 0.9750708 |
| Pyruvate | 0.02516592524 | 0.02010043377 | 1.252009062 | 0.2105665618 | 1.02548526 |
| CE.in.chylomicrons.and.extremely.L.VLDL | -0.02613462334 | 0.02109894617 | -1.238669606 | 0.2154678791 | 0.9742039302 |
| TG.in.M.LDL | -0.02546553149 | 0.02060839009 | -1.235687571 | 0.2165747194 | 0.9748559802 |
| Free.Chol.to.TLs.ratio.in.M.HDL | 0.02768127723 | 0.02277923285 | 1.215197957 | 0.2242905993 | 1.028067964 |
| CE.in.VLDL | -0.02349379557 | 0.01951191477 | -1.204074323 | 0.2285608532 | 0.976780035 |
| Chol.in.M.VLDL | -0.02309553716 | 0.01942789952 | -1.188781996 | 0.2345254647 | 0.9771691234 |
| Con.of.L.LDL.particles | -0.02299744293 | 0.01939334473 | -1.185842012 | 0.235684697 | 0.9772649827 |
| Free.Chol.in.V..L.HDL | 0.02542187998 | 0.0216885847 | 1.172131808 | 0.2411441414 | 1.025747772 |
| CE.to.TLs.ratio.in.M.VLDL | 0.02468470353 | 0.02133089452 | 1.157227771 | 0.2471793143 | 1.024991893 |
| Ratio.of.monounsaturated.FA.to.total.FA | -0.02511046888 | 0.0217865493 | -1.15256751 | 0.2490879451 | 0.9752021766 |
| PL.in.chylomicrons.and.extremely.L.VLDL | -0.0245587238 | 0.0213601635 | -1.149744186 | 0.2502492493 | 0.9757403881 |
| Chol.in.chylomicrons.and.extremely.L.VLDL | -0.02424384722 | 0.02122165758 | -1.142410631 | 0.2532833745 | 0.9760476742 |
| PL.to.TLs.ratio.in.M.HDL | -0.02500389278 | 0.02190468554 | -1.141486041 | 0.2536677176 | 0.9753061154 |
| Ratio.of.linoleic.acid.to.total.FA | 0.02405558003 | 0.02108611066 | 1.140825846 | 0.2539424029 | 1.02434725 |
| Free.Chol.in.chylomicrons.and.extremely.L.VLDL | -0.02399922351 | 0.02121286504 | -1.131352293 | 0.2579068426 | 0.9762864678 |
| Chol.to.TLs.ratio.in.S.LDL | -0.02201714943 | 0.01966154452 | -1.119807724 | 0.2627957069 | 0.9782234589 |
| TG.to.TLs.ratio.in.chylomicrons.and.extremely.L.VLDL | -0.02133910063 | 0.01985354651 | -1.07482563 | 0.2824528032 | 0.9788869671 |
| Acetone | 0.02093882837 | 0.01956543627 | 1.070194811 | 0.2845316296 | 1.021159584 |
| Chol.to.TLs.ratio.in.chylomicrons.and.extremely.L.VLDL | 0.02115743789 | 0.0200428325 | 1.055611171 | 0.2911458951 | 1.021382843 |
| PL.to.TLs.ratio.in.chylomicrons.and.extremely.L.VLDL | 0.020611494 | 0.01956283533 | 1.053604636 | 0.2920639656 | 1.020825378 |

| Metabolite | beta | se | z | p_val | HR |
|---|---|---|---|---|---|
| Remnant_C | -0.02007349439 | 0.01949820311 | -1.029504836 | 0.3032425085 | 0.9801266368 |
| TG.in.LDL | -0.02080639402 | 0.02054094412 | -1.012922965 | 0.3110969584 | 0.9794085656 |
| X3.Hydroxybutyrate | 0.0198668284 | 0.01988825486 | 0.9989226578 | 0.3178321592 | 1.020065487 |
| Tyrosine | -0.01956000193 | 0.01988268882 | -0.9837704602 | 0.3252283867 | 0.9806300537 |
| PL.to.TLs.ratio.in.M.LDL | 0.01895175498 | 0.019754292 | 0.9593740428 | 0.3373703467 | 1.019132479 |
| Free.Chol.to.TLs.ratio.in.V..L.VLDL | 0.01959470596 | 0.02082816698 | 0.9407791853 | 0.3468180297 | 1.019787942 |
| CE.to.TLs.ratio.in.L.VLDL | 0.01956301517 | 0.02084335022 | 0.9385734518 | 0.3479497886 | 1.019755625 |
| Chol.to.TLs.ratio.in.M.VLDL | 0.01940696581 | 0.02116721099 | 0.9168409489 | 0.3592259913 | 1.019596505 |
| Saturated.FA | -0.01810848101 | 0.02006110279 | -0.9026662791 | 0.3667030384 | 0.9820544923 |
| Chol.to.TLs.ratio.in.L.LDL | -0.01889656919 | 0.02094986367 | -0.9019900789 | 0.367062138 | 0.9812808517 |
| Glucose | -0.01899585667 | 0.02117746317 | -0.8969845216 | 0.3697271764 | 0.9811834276 |
| Con.of.HDL.particles | 0.01878063535 | 0.02108068058 | 0.8908932173 | 0.3729864594 | 1.018958101 |
| Monounsaturated.FA | -0.01794555413 | 0.02046233613 | -0.8770041709 | 0.3804843726 | 0.9822145084 |
| TG.to.TLs.ratio.in.M.LDL | 0.01762419414 | 0.02040980393 | 0.863516093 | 0.3878537752 | 1.017780417 |
| TLs.in.LP.particles | -0.01693921451 | 0.01979748433 | -0.8556245953 | 0.392205461 | 0.9832034473 |
| TG.in.L.LDL | -0.01735839064 | 0.02049320125 | -0.8470316777 | 0.3969774649 | 0.9827913983 |
| TG.to.TLs.ratio.in.V..L.VLDL | -0.01755319916 | 0.02073269826 | -0.8466432557 | 0.397193996 | 0.9825999608 |
| PL.to.TLs.ratio.in.L.HDL | -0.01840424568 | 0.02174001337 | -0.8465609183 | 0.3972399053 | 0.9817640782 |
| Glucose.lactate | 0.01761391472 | 0.02135399592 | 0.8248533335 | 0.4094548631 | 1.017769955 |
| CE.in.M.VLDL | -0.01590841303 | 0.01960074981 | -0.8116226766 | 0.4170081752 | 0.9842174574 |
| TG.in.V..S.VLDL | -0.01660104982 | 0.02059604559 | -0.806030932 | 0.420225014 | 0.9835359882 |
| CE.to.TLs.ratio.in.V..L.HDL | -0.01581598757 | 0.01965968047 | -0.8044885364 | 0.4211148872 | 0.9843084284 |
| Con.of.S.HDL.particles | -0.01577701373 | 0.01975888948 | -0.7984767434 | 0.4245938839 | 0.9843467914 |
| Total.free.Chol | -0.01583449497 | 0.01987425484 | -0.7967340208 | 0.4256055212 | 0.9842902116 |
| PL.to.TLs.ratio.in.S.LDL | 0.01558534371 | 0.01980916796 | 0.7867742725 | 0.4314140167 | 1.015707429 |
| TG.to.TLs.ratio.in.M.VLDL | -0.01630112884 | 0.02102507585 | -0.7753184323 | 0.4381515203 | 0.9838310155 |
| Glycoprotein.acetyls | 0.01549244085 | 0.02103642202 | 0.7364579791 | 0.4614520313 | 1.015613071 |
| Free.Chol.to.TLs.ratio.in.M.LDL | 0.01519397167 | 0.0213829166 | 0.7105659137 | 0.477353272 | 1.015309987 |
| Free.Chol.to.TLs.ratio.in.L.HDL | 0.01491398319 | 0.02116142734 | 0.7047720813 | 0.4809520855 | 1.015025752 |
| Chol.to.TLs.ratio.in.L.VLDL | 0.01396249621 | 0.02014233878 | 0.6931914096 | 0.4881894384 | 1.014060427 |
| CE.in.S.HDL | -0.01359933652 | 0.01966443756 | -0.6915700731 | 0.489207361 | 0.9864927167 |
| TC | -0.01390112086 | 0.02013414342 | -0.6904252429 | 0.489926807 | 0.9861950536 |
| Valine | 0.01429840117 | 0.02089181691 | 0.684401995 | 0.4937213566 | 1.014401112 |
| Con.of.IDL.particles | -0.01286640867 | 0.01950682973 | -0.6595848147 | 0.5095203017 | 0.9872160097 |
| Ratio.of.omega.3.FA.to.total.FA | 0.01292200967 | 0.02003845834 | 0.6448604702 | 0.5190176105 | 1.01300586 |
| Phenylalanine | 0.01257438801 | 0.01954748742 | 0.6432738764 | 0.5200464041 | 1.012653778 |
| TG.to.TLs.ratio.in.V..S.VLDL | -0.01354350684 | 0.02109350969 | -0.6420698612 | 0.5208278237 | 0.9865477938 |
| Total.esterified.Chol | -0.01293189678 | 0.02024543574 | -0.6387561595 | 0.5229815724 | 0.9871513609 |
| Glutamine | 0.01261066544 | 0.01983592472 | 0.6357488054 | 0.5249401602 | 1.012690515 |
| CE.to.TLs.ratio.in.chylomicrons.and.extremely.L.VLDL | 0.01235550252 | 0.01987329702 | 0.6217137753 | 0.5341300921 | 1.012432147 |
| PL.to.TLs.ratio.in.L.VLDL | -0.01278728191 | 0.02073783311 | -0.6166161066 | 0.5374879682 | 0.987294128 |
| Total.Con.of.LP.particles | 0.0127526252 | 0.02101685324 | 0.6067809035 | 0.5439963229 | 1.012834287 |
| Total.Con.of.branched.chain.AAs | 0.01257827915 | 0.02074559474 | 0.6063108486 | 0.544308356 | 1.012657718 |
| Con.of.V..S.VLDL.particles | -0.01193633476 | 0.01975454993 | -0.6042321794 | 0.5456892894 | 0.9881346207 |
| Chol.in.S.HDL | -0.01186913866 | 0.01973371497 | -0.6014649892 | 0.5475303239 | 0.9882010217 |
| PL.to.TLs.ratio.in.L.LDL | 0.01132759506 | 0.01932322961 | 0.5862164499 | 0.5577300686 | 1.011391995 |
| TG.in.L.HDL | 0.01199716401 | 0.02061011861 | 0.5821006778 | 0.5604988686 | 1.012069419 |
| TG.in.HDL | -0.01159556487 | 0.02034435989 | -0.5699645962 | 0.5687017109 | 0.9884714046 |
| CE.to.TLs.ratio.in.V..S.VLDL | 0.01193796243 | 0.0213709406 | 0.5586072537 | 0.5764297885 | 1.012009504 |
| Free.Chol.in.IDL | -0.01098628589 | 0.0200472952 | -0.5480183626 | 0.5836792944 | 0.9890738429 |
| Free.Chol.to.TLs.ratio.in.V..S.VLDL | -0.01078113485 | 0.01971136069 | -0.5469503102 | 0.5844128703 | 0.9892767733 |
| Leucine | 0.01107438577 | 0.0205518742 | 0.5388504065 | 0.5899900794 | 1.011135934 |
| Total.FA | -0.01059303744 | 0.02004058862 | -0.528579157 | 0.5970974223 | 0.9894628712 |
| TG.in.IDL | -0.01078066862 | 0.02049643236 | -0.5259778104 | 0.5989036244 | 0.9892772345 |
| CE.to.TLs.ratio.in.S.LDL | -0.01032985241 | 0.01975588734 | -0.5228746361 | 0.6010614962 | 0.9897233173 |
| TG.to.TLs.ratio.in.L.LDL | 0.01086603775 | 0.02082810934 | 0.5217006294 | 0.6018787864 | 1.010925288 |
| Chol.in.IDL | -0.01020928504 | 0.02007951221 | -0.508442881 | 0.6111427869 | 0.9898426528 |
| TLs.in.IDL | -0.01017155292 | 0.02003994278 | -0.50756397 | 0.6117591637 | 0.9898800024 |
| Free.Chol.in.V..S.VLDL | -0.0100487737 | 0.01981367263 | -0.5071636081 | 0.6120400272 | 0.9900015465 |
| PL.in.IDL | -0.01003187392 | 0.02009977602 | -0.4991037664 | 0.6177062844 | 0.9900182775 |
| CE.in.IDL | -0.009813787812 | 0.02007768246 | -0.4887908666 | 0.6249897648 | 0.9902342103 |
| TG.to.TLs.ratio.in.L.VLDL | -0.009473948905 | 0.01958959282 | -0.4836215327 | 0.6286544902 | 0.9905707876 |
| PL.to.TLs.ratio.in.V..S.VLDL | 0.009360959032 | 0.0200573063 | 0.4667106785 | 0.6407068887 | 1.00940491 |
| Spectrometer.corrected.alanine | -0.009263817592 | 0.01997535656 | -0.4637623145 | 0.6428180461 | 0.9907789594 |
| Ratio.of.docosahexaenoic.acid.to.total.FA | 0.009338463565 | 0.02019547743 | 0.462403704 | 0.6437918436 | 1.009382203 |
| Histidine | 0.008883293704 | 0.01958037142 | 0.4536836158 | 0.6500565611 | 1.008922867 |
| Total.cholines | 0.009419167198 | 0.02096434745 | 0.4492945569 | 0.6532191838 | 1.009463667 |
| TLs.in.V..S.VLDL | -0.008444320481 | 0.0198802157 | -0.4247600031 | 0.6710116375 | 0.9915912326 |
| PG | 0.008644917999 | 0.02082756099 | 0.4150710687 | 0.6780898779 | 1.008682393 |
| Chol.to.TLs.ratio.in.V..S.VLDL | 0.008755704287 | 0.02117364737 | 0.4135189434 | 0.6792264463 | 1.008794148 |

| Metabolite | beta | se | z | p_val | HR |
|---|---|---|---|---|---|
| PL.to.TLs.ratio.in.V..L.VLDL | -0.007638432247 | 0.01973503641 | -0.387049311 | 0.6987197025 | 0.9923906664 |
| TLs.in.S.HDL | -0.007603389408 | 0.01991032113 | -0.3818818068 | 0.7025490355 | 0.9924254432 |
| Free.Chol.to.TLs.ratio.in.S.VLDL | 0.008003757962 | 0.02096273572 | 0.3818088473 | 0.7026031558 | 1.008035874 |
| TG.in.M.HDL | -0.007736903648 | 0.0203405549 | -0.3803683668 | 0.7036719928 | 0.9922929492 |
| PL.to.TLs.ratio.in.S.VLDL | 0.00792544498 | 0.0209411367 | 0.378462979 | 0.7050866905 | 1.007956934 |
| Omega.3.FAs | 0.007274150482 | 0.02025429315 | 0.3591411672 | 0.7194894862 | 1.007300671 |
| PL.in.V..S.VLDL | -0.0069059642 | 0.01985272006 | -0.3478598488 | 0.7279454403 | 0.9931178272 |
| TG.to.TLs.ratio.in.S.VLDL | -0.00708077916 | 0.02043689679 | -0.3464703685 | 0.7289892493 | 0.9929442305 |
| Ratio.of.omega.6.FA.to.omega.3.FA | -0.00660448918 | 0.01993521488 | -0.3312976169 | 0.7404196912 | 0.9934172725 |
| Chol.to.TLs.ratio.in.S.VLDL | 0.006690218253 | 0.02019534402 | 0.3312752804 | 0.7404365615 | 1.006712648 |
| Free.Chol.in.S.HDL | -0.006364778916 | 0.02009120053 | -0.3167943551 | 0.7513996477 | 0.9936554334 |
| PL.to.TLs.ratio.in.IDL | 0.005869361425 | 0.01920856305 | 0.3055596303 | 0.7599399615 | 1.00588662 |
| CE.to.TLs.ratio.in.S.VLDL | 0.005772090705 | 0.01967908795 | 0.2933108851 | 0.7692845342 | 1.005788781 |
| Acetoacetate | -0.00578172222 | 0.01978568596 | -0.2922174259 | 0.7701203889 | 0.9942349598 |
| Alanine | 0.005728513561 | 0.01975916825 | 0.2899167357 | 0.771879938 | 1.005744953 |
| Acetate | 0.00551921081 | 0.01941119802 | 0.2843312815 | 0.7761565158 | 1.00553447 |
| TG.in.V..L.HDL | -0.005435366639 | 0.02002858536 | -0.2713804566 | 0.7860984316 | 0.9945793782 |
| PC | 0.005673485657 | 0.02103013972 | 0.2697787905 | 0.7873304407 | 1.00568961 |
| Free.Chol.to.TLs.ratio.in.M.VLDL | 0.005483340403 | 0.02069844943 | 0.2649155156 | 0.7910745455 | 1.005498401 |
| PL.to.TLs.ratio.in.M.VLDL | 0.005364553543 | 0.02046185435 | 0.2621733814 | 0.7931877744 | 1.005378969 |
| Chol.in.V..S.VLDL | -0.005181483558 | 0.01994912062 | -0.2597349356 | 0.7950682425 | 0.9948319172 |
| Isoleucine | 0.005153771473 | 0.02022074547 | 0.2548754437 | 0.7988193031 | 1.005167075 |
| Glycine | 0.005101159959 | 0.02124223194 | 0.2401423718 | 0.8102198871 | 1.005114193 |
| Free.Chol.to.TLs.ratio.in.L.VLDL | 0.004605227204 | 0.01960893343 | 0.2348535284 | 0.8143224198 | 1.004615848 |
| Free.Chol.to.TLs.ratio.in.L.LDL | 0.004664562686 | 0.02119281367 | 0.2201011512 | 0.8257923783 | 1.004675459 |
| Chol.to.TLs.ratio.in.S.HDL | -0.004415782006 | 0.02027398283 | -0.2178053539 | 0.8275807729 | 0.9955939532 |
| Free.Chol.to.TLs.ratio.in.IDL | -0.003905610724 | 0.01950364024 | -0.2002503469 | 0.8412847934 | 0.9961020063 |
| Sphingomyelins | 0.004134340336 | 0.0209119392 | 0.1977023889 | 0.8432779205 | 1.004142899 |
| CE.to.TLs.ratio.in.S.HDL | -0.003833300432 | 0.02024218766 | -0.1893718454 | 0.8498013893 | 0.9961740373 |
| Docosahexaenoic.acid | 0.003771716372 | 0.02042282414 | 0.1846814303 | 0.8534789121 | 1.003778838 |
| CE.in.V..S.VLDL | -0.00330188673 | 0.02006310571 | -0.1645750553 | 0.8692784665 | 0.9967035585 |
| Polyunsaturated.FA | 0.003203096615 | 0.0201258818 | 0.1591531068 | 0.8735482482 | 1.003208232 |
| Albumin | 0.002002632708 | 0.01987235697 | 0.1007747954 | 0.9197292353 | 1.002004639 |
| CE.to.TLs.ratio.in.IDL | 0.001621580093 | 0.02013276898 | 0.08054431533 | 0.9358043522 | 1.001622896 |
| TG.to.TLs.ratio.in.IDL | -0.001566287104 | 0.02093675698 | -0.07481039711 | 0.9403655693 | 0.9984349389 |
| Omega.6.FA | 0.00125423859 | 0.01992857149 | 0.06293670324 | 0.9498169078 | 1.001255025 |
| PL.in.S.HDL | -0.001127114338 | 0.02000458397 | -0.05634280319 | 0.955068721 | 0.9988735206 |
| Linoleic.acid | 0.0009334231349 | 0.01962309507 | 0.04756757951 | 0.9620608706 | 1.000933859 |
| TG.to.TLs.ratio.in.S.LDL | 0.0009543064063 | 0.02080383422 | 0.04587165981 | 0.9634125426 | 1.000954762 |
| Free.Chol.to.TLs.ratio.in.S.HDL | -0.0006171845345 | 0.02088855064 | -0.02954654659 | 0.9764286963 | 0.9993830059 |
| Chol.to.TLs.ratio.in.IDL | -0.0003341288309 | 0.02029726224 | -0.01646176844 | 0.9868660023 | 0.999665927 |
| Total.PL.in.LP.particles | -0.0002617457019 | 0.02058993013 | -0.01271231618 | 0.9898573124 | 0.9997382886 |
| Free.Chol.to.TLs.ratio.in.S.LDL | 5.93476e-05 | 0.0206697542 | 0.002871226846 | 0.9977090956 | 1.000059349 |

## Supplementary Table 17. Associations between circulating metabolites and incident retinal vein occlusion (RVO) in the UK Biobank

| Metabolite | beta | se | z | p_val | HR |
|---|---|---|---|---|---|
| PL.in.S.LDL | -0.1786154848 | 0.04482491868 | -3.984736393 | 6.75551e-05 | 0.8364274567 |
| Con.of.L.LDL.particles | -0.1786485026 | 0.04515919219 | -3.955972061 | 7.62241e-05 | 0.8363998401 |
| Chol.in.M.VLDL | -0.1744719141 | 0.04535405447 | -3.846886814 | 0.0001196281997 | 0.8399004433 |
| Con.of.LDL.particles | -0.1734009383 | 0.04509921295 | -3.844877259 | 0.0001206127946 | 0.8408004382 |
| Con.of.S.LDL.particles | -0.1727124407 | 0.04504169187 | -3.834501626 | 0.0001258191398 | 0.8413795265 |
| ApoB | -0.1724982864 | 0.04512105508 | -3.823010922 | 0.0001318319375 | 0.8415597309 |
| PL.in.IDL | -0.1767148977 | 0.0470381265 | -3.756843882 | 0.0001720697662 | 0.8380186715 |
| Remnant_C | -0.1698481647 | 0.04541494192 | -3.739918131 | 0.0001840802003 | 0.8437929244 |
| CE.in.M.VLDL | -0.1706471412 | 0.04584581356 | -3.722196816 | 0.0001974969815 | 0.8431190229 |
| Free.Chol.in.M.VLDL | -0.1677622444 | 0.04538826753 | -3.696158799 | 0.000218886092 | 0.8455548462 |
| TLs.in.S.LDL | -0.1658305686 | 0.04499380302 | -3.685631299 | 0.0002281364291 | 0.8471897625 |
| Chol.in.V..S.VLDL | -0.1706390077 | 0.04646317581 | -3.67256445 | 0.0002401285627 | 0.8431258805 |
| TC.minus.HDL.C | -0.1669163839 | 0.04550175527 | -3.668350439 | 0.0002441204306 | 0.8462703702 |
| CE.in.V..S.VLDL | -0.1716378232 | 0.04680482993 | -3.667096397 | 0.0002453203311 | 0.8422841737 |
| CE.in.VLDL | -0.1642772584 | 0.04549131194 | -3.611178737 | 0.0003048084963 | 0.8485067337 |
| Total.free.Chol | -0.1670522912 | 0.04643872757 | -3.597262456 | 0.0003215839561 | 0.8461553637 |
| CE.in.L.LDL | -0.1641728274 | 0.04591677811 | -3.575443099 | 0.0003496352171 | 0.8485953486 |
| Free.Chol.in.S.VLDL | -0.1613183556 | 0.04544522986 | -3.549731317 | 0.0003856245111 | 0.8510211006 |
| PL.in.M.VLDL | -0.161161872 | 0.04555366245 | -3.537846649 | 0.0004034042746 | 0.8511542819 |
| Chol.in.L.LDL | -0.162808699 | 0.04604505699 | -3.535856173 | 0.0004064559183 | 0.8497537316 |
| Chol.in.S.LDL | -0.1587846038 | 0.04512513281 | -3.518762027 | 0.0004335654277 | 0.8531801109 |
| CE.in.LDL | -0.1596684364 | 0.04549824388 | -3.509331851 | 0.000449234014 | 0.8524263757 |
| TLs.in.L.LDL | -0.1610228407 | 0.04589578468 | -3.508445095 | 0.0004507342595 | 0.8512726273 |
| TLs.in.IDL | -0.1642579533 | 0.04685621593 | -3.505574448 | 0.0004556230591 | 0.8485231143 |
| LDL_C | -0.1594513441 | 0.04557371554 | -3.498756734 | 0.0004674328387 | 0.8526114509 |
| Free.Chol.in.V..S.VLDL | -0.1610073174 | 0.04605468583 | -3.496002947 | 0.0004722834844 | 0.8512858419 |
| TLs.in.LDL | -0.1586494206 | 0.04547831306 | -3.488463181 | 0.000485805723 | 0.8532954543 |
| Con.of.M.VLDL.particles | -0.1589416697 | 0.04577768766 | -3.472033601 | 0.0005165316813 | 0.8530461159 |
| PL.in.LDL | -0.1575474957 | 0.04538861128 | -3.471079886 | 0.000518369761 | 0.8542362401 |
| Con.of.IDL.particles | -0.1574610275 | 0.04541670818 | -3.467028628 | 0.0005262458387 | 0.8543101076 |
| CE.in.S.LDL | -0.1565029679 | 0.04521612024 | -3.461220624 | 0.000537731928 | 0.8551289797 |
| VLDL_C | -0.158363567 | 0.04582209491 | -3.456052528 | 0.0005481484887 | 0.8535394068 |
| Clinical.LDL_C | -0.1566610732 | 0.04560570896 | -3.435119786 | 0.0005922919574 | 0.85499379 |
| TLs.in.V..S.VLDL | -0.1566791286 | 0.04616675472 | -3.393765267 | 0.0006893875581 | 0.8549783529 |
| Con.of.V..S.VLDL.particles | -0.1551164032 | 0.04586491047 | -3.382027821 | 0.0007195285724 | 0.8563154938 |
| TC | -0.1590865445 | 0.04715288971 | -3.373845069 | 0.0007412607517 | 0.8529225401 |
| Ratio.of.ApoB.to.ApoA1 | -0.1556942651 | 0.04635973283 | -3.358394356 | 0.0007839668147 | 0.8558208047 |
| PL.in.L.LDL | -0.1528803647 | 0.04575109752 | -3.341567154 | 0.0008330686154 | 0.8582323905 |
| Free.Chol.in.S.LDL | -0.1512044135 | 0.04529997928 | -3.3378473 | 0.0008443013491 | 0.8596719521 |
| Free.Chol.in.L.LDL | -0.1549573793 | 0.04645322549 | -3.335772224 | 0.0008506282692 | 0.8564516793 |
| Chol.in.IDL | -0.1566339168 | 0.04699119581 | -3.333260924 | 0.0008583440434 | 0.8550170089 |
| Free.Chol.in.LDL | -0.1528302752 | 0.0459626852 | -3.325094574 | 0.0008838854705 | 0.8582753801 |
| CE.in.IDL | -0.1561521751 | 0.04699349582 | -3.322846542 | 0.0008910391183 | 0.8554290055 |
| PL.in.V..S.VLDL | -0.1529073838 | 0.04609322005 | -3.317350873 | 0.000908754135 | 0.8582092022 |
| Linoleic.acid | -0.1521168878 | 0.04590711676 | -3.313579648 | 0.0009210986874 | 0.8588878814 |
| PL.to.TLs.ratio.in.chylomicrons.and.extremely.L.VLDL | 0.1554356005 | 0.04698314001 | 3.308327209 | 0.0009385508168 | 1.168166704 |
| Chol.in.S.VLDL | -0.1516518592 | 0.04596545857 | -3.299256962 | 0.0009694112947 | 0.8592873817 |
| Free.Chol.in.IDL | -0.1544148285 | 0.04687097172 | -3.294466123 | 0.0009860883008 | 0.8569164739 |
| TLs.in.M.VLDL | -0.1510280634 | 0.04608530544 | -3.277141422 | 0.001048638317 | 0.8598235687 |
| PL.in.M.LDL | -0.1485528054 | 0.04544886765 | -3.268570001 | 0.001080924356 | 0.8619544901 |
| Con.of.M.LDL.particles | -0.1482625094 | 0.04538536382 | -3.266747182 | 0.001087907878 | 0.8622047484 |
| Total.esterified.Chol | -0.1548249693 | 0.04744505811 | -3.263247542 | 0.001101432676 | 0.8565650896 |
| PL.in.S.VLDL | -0.1493472805 | 0.04603589416 | -3.244148577 | 0.00117802281 | 0.8612699607 |
| TLs.in.M.LDL | -0.1471735414 | 0.04544968818 | -3.238163942 | 0.001203016577 | 0.8631441731 |
| Chol.in.M.LDL | -0.1469125331 | 0.04541963341 | -3.234560081 | 0.001218302851 | 0.8633694904 |
| TLs.in.LP.particles | -0.1485135844 | 0.04595908083 | -3.231430693 | 0.001231721906 | 0.8619882975 |
| Free.Chol.in.M.LDL | -0.1451325195 | 0.04549665005 | -3.189960566 | 0.001422922083 | 0.8649076684 |
| CE.in.M.LDL | -0.1447899488 | 0.04557316054 | -3.177088162 | 0.001487618066 | 0.8652040111 |
| Con.of.VLDL.particles | -0.1468617071 | 0.04624957801 | -3.175417235 | 0.001496211966 | 0.8634133731 |
| Free.Chol.in.VLDL | -0.1467844637 | 0.04648668403 | -3.157559348 | 0.001590958484 | 0.8634800686 |
| CE.in.S.VLDL | -0.1441490461 | 0.04636825912 | -3.108787107 | 0.00187857027 | 0.8657587005 |
| Chol.to.TLs.ratio.in.L.LDL | -0.1501114115 | 0.04928807678 | -3.045592795 | 0.002322221069 | 0.860612089 |
| Omega.6.FA | -0.140911538 | 0.04657989042 | -3.025158211 | 0.002485030984 | 0.8685661434 |
| PL.in.VLDL | -0.1353795069 | 0.04688480582 | -2.887492111 | 0.00388326344 | 0.8733843934 |
| Polyunsaturated.FA | -0.1332751873 | 0.0469717403 | -2.837348296 | 0.004548995856 | 0.8752242084 |
| CE.to.TLs.ratio.in.L.LDL | -0.1258952776 | 0.04521768001 | -2.784204707 | 0.005365915629 | 0.8817071765 |
| Total.PL.in.LP.particles | -0.1331331223 | 0.04799894165 | -2.77366787 | 0.005542823481 | 0.875348556 |
| CE.in.L.VLDL | -0.1313001931 | 0.04757647287 | -2.759771483 | 0.005784180553 | 0.8769544792 |

| Metabolite | beta | se | z | p_val | HR |
|---|---|---|---|---|---|
| CE.in.V..L.VLDL | -0.1321872965 | 0.04795170757 | -2.756675481 | 0.005839227141 | 0.8761768748 |
| TLs.in.S.VLDL | -0.1273713106 | 0.04707881091 | -2.705491242 | 0.006820344666 | 0.8804067075 |
| Con.of.S.VLDL.particles | -0.1255926938 | 0.04721998412 | -2.659736045 | 0.007820191131 | 0.8819740071 |
| TLs.in.VLDL | -0.124548127 | 0.04741283525 | -2.626886291 | 0.008617012376 | 0.8828957693 |
| Free.Chol.to.TLs.ratio.in.L.HDL | -0.1239482587 | 0.04941513012 | -2.508305826 | 0.01213116217 | 0.8834255493 |
| Chol.in.V..L.VLDL | -0.1205934412 | 0.0484354097 | -2.489778489 | 0.01278227332 | 0.8863942577 |
| Chol.in.L.VLDL | -0.1201774118 | 0.04838851784 | -2.483593571 | 0.01300641589 | 0.8867631005 |
| Free.Chol.to.TLs.ratio.in.chylomicrons.and.extremely.L.VLDL | 0.1184880352 | 0.04784055537 | 2.476727837 | 0.01325929675 | 1.125793404 |
| Free.Chol.to.TLs.ratio.in.S.HDL | -0.1205246768 | 0.04873594857 | -2.47301387 | 0.0133978943 | 0.8864552122 |
| Total.FA | -0.1139597452 | 0.04621378504 | -2.465925375 | 0.01366598024 | 0.8922938743 |
| Sphingomyelins | -0.1205608261 | 0.04896425716 | -2.462221079 | 0.01380795249 | 0.8864231681 |
| Free.Chol.to.TLs.ratio.in.S.VLDL | -0.118885211 | 0.04929656616 | -2.4116327 | 0.01588127145 | 0.8879097172 |
| TG.to.TLs.ratio.in.chylomicrons.and.extremely.L.VLDL | -0.1102349335 | 0.04739721862 | -2.325767983 | 0.02003093155 | 0.8956236985 |
| TG.to.TLs.ratio.in.V..S.VLDL | 0.1108565941 | 0.04957037731 | 2.236347596 | 0.02532901096 | 1.117234677 |
| Total.cholines | -0.1093525104 | 0.04892311255 | -2.235191195 | 0.02540479932 | 0.8964143664 |
| PC | -0.1094807147 | 0.0490100411 | -2.23384254 | 0.02549343538 | 0.8962994496 |
| PL.to.TLs.ratio.in.S.VLDL | -0.1082422709 | 0.04917655421 | -2.201095067 | 0.02772929445 | 0.8974101537 |
| PL.to.TLs.ratio.in.M.VLDL | -0.1055205603 | 0.04795700002 | -2.200316122 | 0.0277844742 | 0.8998559713 |
| TG.in.M.VLDL | -0.1051040066 | 0.04794958329 | -2.191969135 | 0.02838173534 | 0.9002308877 |
| Free.Chol.in.L.VLDL | -0.1064828545 | 0.04929040514 | -2.160316073 | 0.0307482095 | 0.8989904617 |
| TG.to.TLs.ratio.in.S.VLDL | 0.1024428669 | 0.04795438778 | 2.136256381 | 0.03265852211 | 1.107874004 |
| Monounsaturated.FA | -0.1006048102 | 0.04721525686 | -2.130769097 | 0.03310816939 | 0.9042903286 |
| Saturated.FA | -0.09811964561 | 0.04612422742 | -2.127290821 | 0.03339592697 | 0.9065404337 |
| TG.to.TLs.ratio.in.L.LDL | 0.1039273651 | 0.04890455147 | 2.125106192 | 0.03357775306 | 1.109519862 |
| Free.Chol.in.V..L.VLDL | -0.1035251773 | 0.04895082914 | -2.114880976 | 0.03444009303 | 0.9016533213 |
| Con.of.L.VLDL.particles | -0.1025307218 | 0.04893026266 | -2.095445971 | 0.03613136656 | 0.9025504214 |
| Free.Chol.to.TLs.ratio.in.M.VLDL | -0.1017075986 | 0.0486068353 | -2.092454651 | 0.03639786443 | 0.9032936374 |
| TLs.in.L.VLDL | -0.101877634 | 0.0488386101 | -2.086006005 | 0.03697807907 | 0.9031400586 |
| Chol.to.TLs.ratio.in.V..S.VLDL | -0.1034726621 | 0.04976926432 | -2.07904745 | 0.03761298937 | 0.901700673 |
| Chol.to.TLs.ratio.in.S.VLDL | -0.0970942278 | 0.04736676236 | -2.04983881 | 0.04038016298 | 0.9074704932 |
| CE.to.TLs.ratio.in.V..S.VLDL | -0.1026515402 | 0.05023605188 | -2.043383913 | 0.04101444873 | 0.9024413832 |
| PG | -0.09611790785 | 0.04848636252 | -1.982369946 | 0.04743785075 | 0.9083569073 |
| TG.to.TLs.ratio.in.M.VLDL | 0.0974076362 | 0.04935073215 | 1.973783001 | 0.04840642258 | 1.102309623 |
| TG.in.L.VLDL | -0.09467428325 | 0.04854305722 | -1.950315631 | 0.05113851079 | 0.9096691807 |
| PL.in.L.VLDL | -0.09481822903 | 0.04934767638 | -1.921432497 | 0.05467720493 | 0.9095382471 |
| PL.in.V..L.VLDL | -0.09474299696 | 0.04933791618 | -1.920287769 | 0.0548215605 | 0.9096066761 |
| Ratio.of.linoleic.acid.to.total.FA | -0.09465278452 | 0.04933592724 | -1.918536649 | 0.05504299971 | 0.9096887377 |
| Spectrometer.corrected.alanine | 0.08934284849 | 0.04701004163 | 1.900505624 | 0.05736679766 | 1.093455482 |
| Chol.to.TLs.ratio.in.M.VLDL | -0.09417049611 | 0.04968629596 | -1.895301195 | 0.05805250994 | 0.9101275758 |
| Con.of.V..L.HDL.particles | -0.09940310479 | 0.05274858484 | -1.884469604 | 0.05950148254 | 0.9053776724 |
| TG.to.TLs.ratio.in.M.LDL | 0.09015689919 | 0.04791873627 | 1.881454024 | 0.05991018377 | 1.094345972 |
| Free.Chol.to.TLs.ratio.in.V..S.VLDL | -0.08590310679 | 0.04566262592 | -1.88125639 | 0.05993705025 | 0.9176831442 |
| TG.in.V..L.HDL | -0.08572663422 | 0.04614615821 | -1.857719852 | 0.06320880781 | 0.9178451044 |
| Citrate | -0.08610326019 | 0.04663507402 | -1.846319793 | 0.06484578553 | 0.9174994852 |
| Con.of.V..L.VLDL.particles | -0.09117501433 | 0.04941200651 | -1.845199593 | 0.06500850964 | 0.9128579337 |
| CE.to.TLs.ratio.in.M.VLDL | -0.09124403931 | 0.05002772596 | -1.823869416 | 0.06817181643 | 0.9127949259 |
| Glucose.lactate | 0.09072925187 | 0.05049245077 | 1.796887465 | 0.072353487 | 1.094972503 |
| TG.to.TLs.ratio.in.IDL | 0.08804631033 | 0.04909289519 | 1.793463392 | 0.07289885916 | 1.092038694 |
| TLs.in.V..L.VLDL | -0.0884078777 | 0.04957147306 | -1.783442618 | 0.07451428096 | 0.9153874345 |
| Chol.to.TLs.ratio.in.M.LDL | -0.08196325327 | 0.04597997268 | -1.78258595 | 0.07465372866 | 0.921305813 |
| Free.Chol.in.V..L.HDL | -0.08941647468 | 0.05043642922 | -1.772854979 | 0.07625274039 | 0.9144646429 |
| TG.in.VLDL | -0.08617688691 | 0.04895570087 | -1.760303405 | 0.07835637723 | 0.9174319352 |
| TG.in.S.LDL | -0.08383343198 | 0.04775339458 | -1.755549165 | 0.07916541874 | 0.9195844167 |
| Acetoacetate | -0.08160140811 | 0.04665375858 | -1.749085403 | 0.08027625808 | 0.9216392434 |
| TG.in.IDL | -0.08222134383 | 0.04759579472 | -1.727491774 | 0.08407938132 | 0.9210680634 |
| PL.to.TLs.ratio.in.L.LDL | 0.0758522564 | 0.04461982525 | 1.699967581 | 0.08913702361 | 1.078803176 |
| Chol.to.TLs.ratio.in.chylomicrons.and.extremely.L.VLDL | 0.08079697952 | 0.04754080164 | 1.699529178 | 0.08921952175 | 1.084150769 |
| TG.in.LDL | -0.07987982114 | 0.04751836318 | -1.68103057 | 0.09275697691 | 0.9232272921 |
| TG.in.L.LDL | -0.07954792157 | 0.04749165425 | -1.674987381 | 0.09393670755 | 0.9235337617 |
| TG | -0.08131278656 | 0.04871157361 | -1.669270371 | 0.09506380929 | 0.9219052868 |
| TG.in.M.LDL | -0.07700145617 | 0.04759457711 | -1.617861967 | 0.1056923482 | 0.9258885054 |
| CE.to.TLs.ratio.in.S.VLDL | -0.07295759624 | 0.0461062312 | -1.582380393 | 0.113562758 | 0.9296402494 |
| TG.in.chylomicrons.and.extremely.L.VLDL | -0.07510153945 | 0.04786731614 | -1.568952377 | 0.116659036 | 0.9276492885 |
| X3.Hydroxybutyrate | -0.07366388585 | 0.04737616416 | -1.554872311 | 0.119976483 | 0.928983886 |
| Free.Chol.to.TLs.ratio.in.M.HDL | -0.08317992775 | 0.05358339965 | -1.552345097 | 0.1205796727 | 0.9201855654 |
| Chol.in.V..L.HDL | -0.07959287121 | 0.05291053512 | -1.504291556 | 0.1325063123 | 0.9234922501 |
| TG.in.V..L.VLDL | -0.07502236135 | 0.04994625418 | -1.502061818 | 0.1330811446 | 0.927722741 |
| Glucose | 0.07200602356 | 0.05012613882 | 1.436496512 | 0.1508611083 | 1.074661817 |
| TG.in.V..S.VLDL | -0.06770964481 | 0.04793006102 | -1.412675957 | 0.1577510242 | 0.9345317803 |
| TLs.in.V..L.HDL | -0.07441455767 | 0.05310902143 | -1.401166048 | 0.1611644246 | 0.9282867857 |

| Metabolite | beta | se | z | p_val | HR |
|---|---|---|---|---|---|
| Con.of.chylomicrons.and.extremely.L.VLDL.particles | -0.06798424502 | 0.04886089279 | -1.391383602 | 0.1641091344 | 0.9342751929 |
| PL.to.TLs.ratio.in.V..L.VLDL | -0.06594075795 | 0.04759125831 | -1.385564498 | 0.165879925 | 0.9361863242 |
| CE.in.V..L.HDL | -0.07321887555 | 0.05349146178 | -1.368795563 | 0.1710631882 | 0.9293973854 |
| PL.to.TLs.ratio.in.L.HDL | 0.06902138113 | 0.05075388971 | 1.359922984 | 0.173854297 | 1.071459118 |
| TG.in.S.VLDL | -0.06564538008 | 0.04849797172 | -1.353569598 | 0.1758737317 | 0.9364628938 |
| CE.to.TLs.ratio.in.M.LDL | -0.06385503375 | 0.04718849061 | -1.353190851 | 0.1759946675 | 0.9381409885 |
| Average.diameter.for.LDL.particles | -0.06450255825 | 0.04795293383 | -1.34512225 | 0.178585759 | 0.9375337158 |
| TLs.in.chylomicrons.and.extremely.L.VLDL | -0.06458166998 | 0.04891736021 | -1.32021985 | 0.1867616263 | 0.9374595488 |
| PL.to.TLs.ratio.in.S.HDL | 0.06219165367 | 0.04743144091 | 1.311190478 | 0.1897934186 | 1.064166277 |
| Acetate | 0.0577277303 | 0.04554051229 | 1.267612668 | 0.2049363056 | 1.059426507 |
| TG.to.TLs.ratio.in.S.LDL | 0.06022640229 | 0.04856631071 | 1.240086006 | 0.2149435843 | 1.062076976 |
| CE.in.chylomicrons.and.extremely.L.VLDL | -0.06019050895 | 0.0491290832 | -1.225150258 | 0.2205186122 | 0.9415851361 |
| Glycoprotein.acetyls | -0.05998221473 | 0.04947885938 | -1.212279658 | 0.2254053479 | 0.9417812833 |
| TG.to.TLs.ratio.in.V..L.VLDL | 0.05629217056 | 0.04910111274 | 1.14645407 | 0.2516073211 | 1.057906728 |
| Omega.3.FAs | -0.05313361506 | 0.04743271861 | -1.120189114 | 0.2626331817 | 0.9482533031 |
| Ratio.of.docosahexaenoic.acid.to.total.FA | 0.05177054388 | 0.04693508509 | 1.103024396 | 0.270016571 | 1.053134067 |
| Acetone | -0.05057847756 | 0.04590867552 | -1.101719381 | 0.2705836887 | 0.9506793188 |
| PL.in.V..L.HDL | -0.05849642357 | 0.05322041084 | -1.099135137 | 0.2717091256 | 0.9431816136 |
| Free.Chol.in.S.HDL | -0.05088153885 | 0.04677429551 | -1.087809839 | 0.2766790652 | 0.9503912483 |
| CE.to.TLs.ratio.in.IDL | -0.04774671315 | 0.04674124514 | -1.021511366 | 0.3070122271 | 0.9533752339 |
| Average.diameter.for.VLDL.particles | -0.05109244218 | 0.05017071977 | -1.01837172 | 0.3085013365 | 0.9501908288 |
| Total.Con.of.LP.particles | -0.04957861659 | 0.04914208686 | -1.008883012 | 0.3130307435 | 0.9516303413 |
| PL.to.TLs.ratio.in.L.VLDL | -0.04939448998 | 0.04920507553 | -1.00384949 | 0.3154511657 | 0.9518055779 |
| Chol.in.chylomicrons.and.extremely.L.VLDL | -0.0492381365 | 0.0494524456 | -0.9956663599 | 0.3194122802 | 0.9519544076 |
| Chol.to.TLs.ratio.in.IDL | -0.04660352563 | 0.04723173125 | -0.9866995006 | 0.323789975 | 0.9544657438 |
| Chol.to.TLs.ratio.in.L.HDL | -0.05049171965 | 0.05203281082 | -0.9703823195 | 0.3318559583 | 0.9507618013 |
| CE.to.TLs.ratio.in.chylomicrons.and.extremely.L.VLDL | 0.0458922253 | 0.04750681345 | 0.9660135455 | 0.3340374031 | 1.046961569 |
| CE.to.TLs.ratio.in.V..L.VLDL | -0.04557672724 | 0.04968796804 | -0.9172588262 | 0.3590070275 | 0.955446291 |
| Alanine | 0.04233322915 | 0.04639564903 | 0.9124396367 | 0.3615373321 | 1.04324206 |
| Ratio.of.TG.to.PG | -0.04639003776 | 0.05158104524 | -0.8993621115 | 0.3684598136 | 0.9546695324 |
| Chol.to.TLs.ratio.in.V..L.VLDL | -0.04393519642 | 0.0497724195 | -0.8827217335 | 0.3773866366 | 0.9570159735 |
| Leucine | -0.04266349275 | 0.04856060605 | -0.8785617852 | 0.3796389237 | 0.9582337884 |
| Free.Chol.in.HDL | -0.04684418943 | 0.05342182904 | -0.8768735604 | 0.3805553185 | 0.9542360661 |
| Free.Chol.to.TLs.ratio.in.V..L.VLDL | -0.04246543811 | 0.04943188828 | -0.8590697136 | 0.3903020554 | 0.9584235899 |
| Free.Chol.in.chylomicrons.and.extremely.L.VLDL | -0.04190501751 | 0.04936588134 | -0.8488659854 | 0.3959558679 | 0.9589608607 |
| CE.to.TLs.ratio.in.M.HDL | 0.04097170353 | 0.0491377555 | 0.8338130857 | 0.4043863202 | 1.041822625 |
| Valine | -0.04033609608 | 0.04938629999 | -0.8167466705 | 0.4140732143 | 0.9604665758 |
| Total.Con.of.branched.chain.AAs | -0.03891428165 | 0.04914314131 | -0.7918558037 | 0.4284447597 | 0.9618331524 |
| Free.Chol.to.TLs.ratio.in.L.LDL | -0.03829115079 | 0.04952915736 | -0.7731032151 | 0.4394612985 | 0.9624326871 |
| TG.to.TLs.ratio.in.L.VLDL | 0.03387355999 | 0.04682013883 | 0.7234826902 | 0.469383385 | 1.034453802 |
| Chol.to.TLs.ratio.in.S.HDL | -0.03399046406 | 0.04723532217 | -0.7195984383 | 0.4717722739 | 0.9665807218 |
| Lactate | 0.03369563944 | 0.04689165578 | 0.7185849781 | 0.4723966718 | 1.034269768 |
| Free.Chol.in.L.HDL | -0.03838432072 | 0.05465439103 | -0.7023099151 | 0.4824859112 | 0.9623430214 |
| CE.to.TLs.ratio.in.L.VLDL | -0.03427898094 | 0.04892473292 | -0.7006472778 | 0.483523166 | 0.9663018872 |
| PL.in.chylomicrons.and.extremely.L.VLDL | -0.03477751773 | 0.04974173244 | -0.6991617706 | 0.4844509389 | 0.9658202702 |
| Tyrosine | -0.03252419343 | 0.04689064686 | -0.6936179304 | 0.4879218461 | 0.9679990303 |
| Ratio.of.monounsaturated.FA.to.total.FA | -0.03477843101 | 0.0508775906 | -0.6835707154 | 0.494246282 | 0.9658193882 |
| CE.in.M.HDL | 0.03334999546 | 0.05051852716 | 0.6601537561 | 0.5091551648 | 1.033912341 |
| CE.to.TLs.ratio.in.S.LDL | -0.03016569571 | 0.04638322485 | -0.6503578785 | 0.5154610788 | 0.9702847482 |
| Con.of.S.HDL.particles | -0.02936361218 | 0.0463183685 | -0.6339517805 | 0.5261122913 | 0.9710633098 |
| Free.Chol.to.TLs.ratio.in.V..L.HDL | 0.03307581562 | 0.05357119182 | 0.6174179536 | 0.536959083 | 1.033628901 |
| Chol.to.TLs.ratio.in.L.VLDL | -0.02921514994 | 0.0475280425 | -0.6146928929 | 0.5387575543 | 0.9712074868 |
| Chol.to.TLs.ratio.in.S.LDL | -0.02759480168 | 0.04613195624 | -0.5981710712 | 0.549725792 | 0.9727824568 |
| Ratio.of.polyunsaturated.FA.to.monounsaturated.FA | 0.02942527981 | 0.0506557133 | 0.5808876807 | 0.5613161557 | 1.029862481 |
| CE.to.TLs.ratio.in.L.HDL | -0.02659899175 | 0.05146114993 | -0.5168751919 | 0.6052432864 | 0.9737516457 |
| Glutamine | 0.02377133219 | 0.04645628329 | 0.5116925097 | 0.6088662292 | 1.024056122 |
| Chol.in.M.HDL | 0.02588121446 | 0.05075933529 | 0.5098808783 | 0.6101349191 | 1.026219041 |
| TG.in.L.HDL | -0.02278244146 | 0.04795892863 | -0.4750406675 | 0.6347579872 | 0.9774751187 |
| Albumin | -0.02201526957 | 0.04657873485 | -0.472646362 | 0.6364655001 | 0.9782252979 |
| TG.to.TLs.ratio.in.M.HDL | -0.02311595881 | 0.04943405723 | -0.467612009 | 0.6400620755 | 0.9771491681 |
| TG.in.S.HDL | -0.02307050838 | 0.04962203228 | -0.4649246981 | 0.6419853829 | 0.977193581 |
| Chol.in.S.HDL | -0.02140873284 | 0.04621521406 | -0.4632399369 | 0.6431923929 | 0.9788188074 |
| Free.Chol.to.TLs.ratio.in.IDL | -0.01997130678 | 0.04500446399 | -0.4437627961 | 0.6572140891 | 0.9802267988 |
| Docosahexaenoic.acid | -0.02077338683 | 0.04752659149 | -0.4370897676 | 0.6620462478 | 0.9794408936 |
| PL.to.TLs.ratio.in.M.LDL | 0.02011919019 | 0.04660155858 | 0.4317278392 | 0.6659392314 | 1.020322945 |
| Degree.of.unsaturation | 0.0202930913 | 0.04818809168 | 0.4211225344 | 0.6736656073 | 1.020500396 |
| Chol.to.TLs.ratio.in.M.HDL | 0.0209080448 | 0.05048820822 | 0.4141173858 | 0.6787881408 | 1.021128149 |
| CE.to.TLs.ratio.in.V..L.HDL | -0.01795707679 | 0.04447381463 | -0.4037674064 | 0.6863837665 | 0.9822031908 |
| TLs.in.M.HDL | 0.02012006188 | 0.04990957191 | 0.4031303237 | 0.6868523539 | 1.020323835 |
| Con.of.L.HDL.particles | -0.02192856046 | 0.05499784494 | -0.3987167223 | 0.6901019449 | 0.9783101226 |

| Metabolite | beta | se | z | p_val | HR |
|---|---|---|---|---|---|
| Con.of.HDL.particles | -0.01954062882 | 0.04925510802 | -0.3967228903 | 0.6915718185 | 0.9806490518 |
| PL.in.M.HDL | 0.01871067086 | 0.04933733494 | 0.3792395937 | 0.7045099521 | 1.018886812 |
| Isoleucine | -0.01794877068 | 0.04777180448 | -0.3757189179 | 0.7071258722 | 0.9822113491 |
| Ratio.of.polyunsaturated.FA.to.total.FA | 0.01703544106 | 0.04948975876 | 0.3442215416 | 0.7306796832 | 1.017181372 |
| TG.to.TLs.ratio.in.S.HDL | -0.01715747698 | 0.05026669941 | -0.3413288954 | 0.7328559972 | 0.9829888743 |
| PL.to.TLs.ratio.in.M.HDL | -0.01729733599 | 0.05123611604 | -0.3376004532 | 0.7356643006 | 0.9828514041 |
| TG.in.HDL | -0.01601558017 | 0.04746472488 | -0.337420689 | 0.7357997906 | 0.9841119873 |
| Free.Chol.to.TLs.ratio.in.L.VLDL | -0.01555936669 | 0.0468403173 | -0.3321789345 | 0.7397541493 | 0.9845610549 |
| PL.to.TLs.ratio.in.IDL | -0.01465557736 | 0.04453628444 | -0.3290704993 | 0.7421024017 | 0.9854512929 |
| Free.Chol.to.TLs.ratio.in.S.LDL | -0.01562121832 | 0.04813118173 | -0.3245550547 | 0.7455178483 | 0.9845001601 |
| Con.of.M.HDL.particles | 0.01605762958 | 0.05053426242 | 0.3177572762 | 0.7506690609 | 1.016187246 |
| Glycine | -0.01563562975 | 0.05003423975 | -0.3124985975 | 0.7546616287 | 0.9844859721 |
| Creatinine | -0.01606091902 | 0.05323466671 | -0.3017003771 | 0.7628804808 | 0.9840673698 |
| Chol.in.L.HDL | -0.01538996067 | 0.0547412898 | -0.2811398987 | 0.7786031 | 0.9847278596 |
| TLs.in.S.HDL | -0.01228318834 | 0.04656988854 | -0.2637581648 | 0.7919662729 | 0.9877919421 |
| CE.in.S.HDL | -0.01072436636 | 0.04612231106 | -0.2325201429 | 0.8161340441 | 0.9893329346 |
| Free.Chol.to.TLs.ratio.in.M.LDL | -0.01142889604 | 0.04985750165 | -0.2292312222 | 0.8186892068 | 0.9886361657 |
| Ratio.of.saturated.FA.to.total.FA | 0.009159669687 | 0.04578848521 | 0.2000430817 | 0.8414468877 | 1.009201748 |
| TLs.in.L.HDL | -0.01089575112 | 0.05495556362 | -0.1982647507 | 0.8428379291 | 0.9891633926 |
| Histidine | -0.008180730245 | 0.04581502056 | -0.1785600038 | 0.858283202 | 0.9918526409 |
| TLs.in.HDL | -0.008690012789 | 0.05246519749 | -0.1656338526 | 0.8684451047 | 0.9913476362 |
| PL.to.TLs.ratio.in.V..L.HDL | -0.008422622274 | 0.05141120945 | -0.1638285184 | 0.8698661405 | 0.9916127486 |
| HDL_C | -0.007425233387 | 0.0531938008 | -0.1395883219 | 0.8889852681 | 0.9926022656 |
| CE.in.L.HDL | -0.007539956918 | 0.05468532633 | -0.1378789782 | 0.8903360647 | 0.9924883972 |
| Free.Chol.in.M.HDL | -0.00697984799 | 0.05144079251 | -0.1356870229 | 0.8920687071 | 0.9930444546 |
| Ratio.of.omega.3.FA.to.total.FA | 0.005530887891 | 0.04721071666 | 0.1171532288 | 0.9067386296 | 1.005546211 |
| TG.to.TLs.ratio.in.L.HDL | -0.00511897482 | 0.05004688273 | -0.1022835897 | 0.9185315805 | 0.9948941048 |
| PL.to.TLs.ratio.in.V..S.VLDL | 0.004693341056 | 0.046909545 | 0.1000508757 | 0.9203039351 | 1.004704372 |
| TG.in.M.HDL | -0.004029269509 | 0.04756585234 | -0.08470928852 | 0.9324925113 | 0.9959788371 |
| Phenylalanine | 0.003806127088 | 0.04612399054 | 0.0825194664 | 0.9342336392 | 1.00381338 |
| CE.in.HDL | 0.003781784912 | 0.05293651343 | 0.07144000741 | 0.9430475696 | 1.003788945 |
| TG.to.TLs.ratio.in.V..L.HDL | 0.003558198696 | 0.05067667003 | 0.07021374321 | 0.9440235358 | 1.003564537 |
| Chol.to.TLs.ratio.in.V..L.HDL | -0.003065810874 | 0.04640687005 | -0.06606372873 | 0.947327088 | 0.9969388839 |
| ApoA1 | -0.003352520606 | 0.05102523565 | -0.06570318712 | 0.9476141349 | 0.9966530928 |
| PL.in.HDL | -0.003405102887 | 0.05184726053 | -0.06567565677 | 0.9476360536 | 0.9966006879 |
| PL.in.S.HDL | -0.002385683364 | 0.04669734832 | -0.05108819771 | 0.9592552406 | 0.9976171601 |
| Average.diameter.for.HDL.particles | 0.002779820457 | 0.05462417916 | 0.0508899264 | 0.9594132327 | 1.002783688 |
| Ratio.of.omega.6.FA.to.omega.3.FA | -0.002185586887 | 0.04679266974 | -0.04670789034 | 0.9627460416 | 0.9978167998 |
| Pyruvate | 0.001970793686 | 0.04774974922 | 0.04127338296 | 0.9670779523 | 1.001972737 |
| PL.to.TLs.ratio.in.S.LDL | -0.001478327979 | 0.04653502933 | -0.03176806807 | 0.9746570118 | 0.9985227642 |
| PL.in.L.HDL | -0.00118880585 | 0.05474949414 | -0.02171354948 | 0.9826764554 | 0.9988119005 |
| CE.to.TLs.ratio.in.S.HDL | 0.0009116411971 | 0.04707514457 | 0.019365659 | 0.9845494054 | 1.000912057 |
| Ratio.of.omega.6.FA.to.total.FA | -7.85325e-05 | 0.04962908589 | -0.001582387701 | 0.9987374378 | 0.9999214706 |

**Supplementary Table 18. Harmonized food-group definitions and cohort-specific mapping used to construct PDI-H and PDI-U in the UK Biobank and Tianjin datasets.**

| PDI domain | Harmonized food group | UKB operational definition / coding scheme | TJ operational definition / mapping |
|---|---|---|---|
| Healthy plant foods | Whole grains | Whole-grain staple foods and cereals, including wholemeal/wholegrain bread and high-fiber or whole-grain cereal products; coded using the UKB plant-based diet grouping framework | Whole grains and coarse cereals, including oats, brown rice, corn, millet, whole-wheat products, and other minimally refined grain staples |
| Healthy plant foods | Fruits | Fresh fruits and dried fruits | Fresh fruits; dried or unsweetened preserved fruits when recorded |
| Healthy plant foods | Vegetables | Cooked vegetables and salad/raw vegetables; potatoes excluded | Leafy vegetables, cruciferous vegetables, gourds, root vegetables, and other non-potato vegetables |
| Healthy plant foods | Nuts | Nuts and seeds grouped under the UKB plant-based diet coding framework | Peanuts, walnuts, almonds, melon seeds, mixed nuts, and other edible nuts/seeds |
| Healthy plant foods | Legumes and vegetarian protein alternatives | Beans, lentils, peas, soy products, and vegetarian protein alternatives grouped under the UKB plant-based diet coding framework | Soybeans, tofu, bean curd products, lentils, red beans, mung beans, peas, and other legumes/soy-based protein foods |
| Healthy plant foods | Tea and coffee | Tea and coffee beverages | Tea and coffee beverages |
| Unhealthy plant foods | Fruit juices | Fruit juice variables grouped under the UKB plant-based diet coding framework | Fruit juice or sweetened fruit-based beverages, where separately recorded |
| Unhealthy plant foods | Refined grains | Refined grain products, including white or refined bread and lower-fiber refined breakfast cereals, coded using the UKB plant-based diet framework | White rice, refined wheat noodles, white bread, steamed buns made from refined flour, refined breakfast grains, and other refined staple foods |
| Unhealthy plant foods | Potatoes | Potato intake grouped as a separate plant-food category in the UKB plant-based diet framework | Potatoes and other potato-based staple dishes recorded as a separate category |
| Unhealthy plant foods | Sugar-sweetened beverages | Sugar-sweetened beverages coded under the UKB plant-based diet grouping framework | Sugar-sweetened soft drinks, sweetened tea drinks, sweetened fruit drinks, and other sugar-containing beverages |
| Unhealthy plant foods | Sweets and desserts | Cakes, biscuits, confectionery, desserts, and similar items coded under the UKB plant-based diet grouping framework | Cakes, pastries, biscuits, candies, ice cream, sweet desserts, and other sugary snack foods |
| Animal foods | Animal fat | Butter, lard, and other animal-fat–based spreads or cooking fats as defined in the UKB plant-based diet grouping framework | Not retained as an independent scored group in TJ; animal-fat use was not sufficiently captured as a standalone FFQ category |
| Animal foods | Dairy | Milk, cheese, yogurt, and other dairy foods | Milk, yogurt, cheese, milk powder, and other dairy foods |
| Animal foods | Eggs | Eggs and egg-containing foods | Eggs and egg-containing foods |
| Animal foods | Fish or seafood | Oily fish, non-oily fish, and seafood | Fish, shrimp, shellfish, crab, and other seafood |
| Animal foods | Meat | Red meat, processed meat, poultry, pork, lamb/mutton, and beef grouped under the UKB plant-based diet framework | Pork, beef, lamb/mutton, poultry, processed meats, and mixed meat dishes |
| Animal foods | Miscellaneous animal-derived foods | Animal-derived foods not captured by the major dairy/egg/fish/meat categories, coded under the UKB plant-based diet framework | Not retained as an independent scored group in TJ because no direct standalone FFQ counterpart was available |

## Supplementary Table 19. Detailed exploratory mediation results for proteins and metabolites

| Feature_type | Exposure | Mediator | Outcome | a_path_beta | b_path_beta | Indirect_effect | SE | CI_lower | CI_upper | Proportion_mediated_percent | P | FDR_q | Effect_direction |
|---|---|---|---|---|---|---|---|---|---|---|---|---|---|
| Protein | PDI_H | FGF21 | AMD | 0.0474 | 0.079 | 0.003745 | 0.001387 | 0.001026 | 0.006463 | 3.77 | 0.00694 | 0.0606 | Positive indirect effect |
| Protein | PDI_H | FGF21 | Cataract | 0.0337 | 0.05016 | 0.00169 | 0.001458 | -0.001167 | 0.004547 | 1.38 | 0.246 | 0.428 | Positive indirect effect |
| Protein | PDI_H | FGF21 | Glaucoma | 0.0508 | 0.04132 | 0.002099 | 0.001018 | 0.000104 | 0.004093 | 1.51 | 0.0392 | 0.123 | Positive indirect effect |
| Protein | PDI_H | FGF21 | DR | 0.08725 | 0.04425 | 0.003861 | 0.001888 | 0.00016 | 0.007561 | 3.35 | 0.0409 | 0.128 | Positive indirect effect |
| Protein | PDI_H | FGF21 | RVO | -0.04576 | -0.02219 | 0.001016 | 0.001132 | -0.001202 | 0.003233 | 1.06 | 0.369 | 0.639 | Positive indirect effect |
| Protein | PDI_H | CHI3L1 | AMD | -0.04234 | -0.07014 | 0.002969 | 0.001106 | 0.000801 | 0.005138 | 2.06 | 0.00728 | 0.0635 | Positive indirect effect |
| Protein | PDI_H | CHI3L1 | Cataract | 0.06154 | -0.07601 | -0.004678 | 0.001385 | -0.007393 | -0.001963 | 4.51 | 0.000733 | 0.0173 | Negative indirect effect |
| Protein | PDI_H | CHI3L1 | Glaucoma | 0.03471 | 0.0238 | 0.000826 | 0.0006 | -0.00035 | 0.002003 | 0.75 | 0.169 | 0.295 | Positive indirect effect |
| Protein | PDI_H | CHI3L1 | DR | 0.07052 | -0.10924 | -0.007704 | 0.002254 | -0.012122 | -0.003286 | 7.72 | 0.000632 | 0.015 | Negative indirect effect |
| Protein | PDI_H | CHI3L1 | RVO | -0.02888 | 0.02362 | -0.000682 | 0.000429 | -0.001524 | 0.000159 | 0.63 | 0.112 | 0.198 | Negative indirect effect |
| Protein | PDI_H | IGFBP4 | AMD | 0.0489 | -0.0257 | -0.001257 | 0.001266 | -0.003739 | 0.001225 | 1.21 | 0.321 | 0.556 | Negative indirect effect |
| Protein | PDI_H | IGFBP4 | Cataract | -0.05085 | -0.03111 | 0.001582 | 0.000863 | -0.000109 | 0.003273 | 1.61 | 0.0667 | 0.12 | Positive indirect effect |
| Protein | PDI_H | IGFBP4 | Glaucoma | 0.03025 | 0.03418 | 0.001034 | 0.000575 | -9.2e-05 | 0.00216 | 0.74 | 0.0719 | 0.129 | Positive indirect effect |
| Protein | PDI_H | IGFBP4 | DR | -0.07213 | 0.04573 | -0.003298 | 0.001609 | -0.006451 | -0.000146 | 2.78 | 0.0403 | 0.126 | Negative indirect effect |
| Protein | PDI_H | IGFBP4 | RVO | -0.02761 | -0.02569 | 0.000709 | 0.000786 | -0.000831 | 0.002249 | 0.6 | 0.367 | 0.635 | Positive indirect effect |
| Protein | PDI_H | TNFRSF10B | AMD | 0.05818 | 0.04873 | 0.002835 | 0.001012 | 0.000851 | 0.004818 | 2.84 | 0.00509 | 0.0449 | Positive indirect effect |
| Protein | PDI_H | TNFRSF10B | Cataract | -0.08967 | 0.06775 | -0.006075 | 0.001778 | -0.009559 | -0.002591 | 5.4 | 0.000632 | 0.015 | Negative indirect effect |
| Protein | PDI_H | TNFRSF10B | Glaucoma | 0.04462 | 0.02321 | 0.001036 | 0.001223 | -0.001362 | 0.003433 | 0.99 | 0.397 | 0.686 | Positive indirect effect |
| Protein | PDI_H | TNFRSF10B | DR | 0.10213 | -0.07426 | -0.007584 | 0.002204 | -0.011904 | -0.003264 | 5.83 | 0.00058 | 0.0138 | Negative indirect effect |
| Protein | PDI_H | TNFRSF10B | RVO | -0.03463 | 0.02547 | -0.000882 | 0.000667 | -0.002189 | 0.000425 | 0.85 | 0.186 | 0.324 | Negative indirect effect |
| Protein | PDI_H | GPR37 | AMD | 0.06271 | 0.03497 | 0.002193 | 0.001082 | 7.2e-05 | 0.004314 | 2.22 | 0.0427 | 0.133 | Positive indirect effect |
| Protein | PDI_H | GPR37 | Cataract | 0.05287 | 0.0799 | 0.004224 | 0.001015 | 0.002235 | 0.006214 | 3.11 | 3.16e-05 | 0.00149 | Positive indirect effect |
| Protein | PDI_H | GPR37 | Glaucoma | 0.02455 | -0.04155 | -0.00102 | 0.000752 | -0.002494 | 0.000454 | 0.94 | 0.175 | 0.306 | Negative indirect effect |
| Protein | PDI_H | GPR37 | DR | 0.05372 | 0.0616 | 0.003309 | 0.001633 | 0.000109 | 0.006509 | 2.18 | 0.0427 | 0.133 | Positive indirect effect |
| Protein | PDI_H | GPR37 | RVO | -0.02359 | -0.03271 | 0.000772 | 0.00089 | -0.000972 | 0.002515 | 0.79 | 0.386 | 0.667 | Positive indirect effect |
| Protein | PDI_H | STC1 | AMD | -0.05765 | 0.09441 | -0.005443 | 0.001472 | -0.008329 | -0.002557 | 4.05 | 0.000219 | 0.0057 | Negative indirect effect |
| Protein | PDI_H | STC1 | Cataract | 0.03502 | 0.04454 | 0.00156 | 0.001657 | -0.001687 | 0.004807 | 1.52 | 0.346 | 0.6 | Positive indirect effect |
| Protein | PDI_H | STC1 | Glaucoma | 0.02787 | -0.02844 | -0.000793 | 0.000669 | -0.002105 | 0.000519 | 0.64 | 0.236 | 0.411 | Negative indirect effect |
| Protein | PDI_H | STC1 | DR | -0.08804 | 0.05666 | -0.004989 | 0.001666 | -0.008254 | -0.001723 | 4.88 | 0.00275 | 0.0251 | Negative indirect effect |
| Protein | PDI_H | STC1 | RVO | -0.02909 | 0.03443 | -0.001001 | 0.001158 | -0.003272 | 0.001269 | 0.69 | 0.387 | 0.67 | Negative indirect effect |
| Protein | PDI_H | ASGR1 | AMD | -0.0346 | -0.03957 | 0.001369 | 0.000789 | -0.000177 | 0.002915 | 1.28 | 0.0826 | 0.147 | Positive indirect effect |
| Protein | PDI_H | ASGR1 | Cataract | -0.08277 | 0.04603 | -0.00381 | 0.001417 | -0.006587 | -0.001033 | 2.58 | 0.00717 | 0.0625 | Negative indirect effect |
| Protein | PDI_H | ASGR1 | Glaucoma | -0.03612 | 0.02935 | -0.00106 | 0.00114 | -0.003295 | 0.001175 | 0.76 | 0.352 | 0.61 | Negative indirect effect |
| Protein | PDI_H | ASGR1 | DR | 0.07484 | 0.03905 | 0.002922 | 0.001283 | 0.000407 | 0.005438 | 2.16 | 0.0228 | 0.0758 | Positive indirect effect |
| Protein | PDI_H | ASGR1 | RVO | -0.04015 | -0.03999 | 0.001606 | 0.000759 | 0.000118 | 0.003094 | 1.57 | 0.0344 | 0.109 | Positive indirect effect |
| Protein | PDI_H | DDC | AMD | -0.03617 | 0.03242 | -0.001172 | 0.000992 | -0.003117 | 0.000772 | 1.16 | 0.237 | 0.413 | Negative indirect effect |
| Protein | PDI_H | DDC | Cataract | 0.04596 | -0.02903 | -0.001334 | 0.001223 | -0.003731 | 0.001062 | 0.89 | 0.275 | 0.478 | Negative indirect effect |
| Protein | PDI_H | DDC | Glaucoma | 0.03529 | 0.02326 | 0.000821 | 0.000737 | -0.000624 | 0.002266 | 0.68 | 0.265 | 0.461 | Positive indirect effect |
| Protein | PDI_H | DDC | DR | -0.05181 | 0.03761 | -0.001949 | 0.001766 | -0.00541 | 0.001512 | 1.39 | 0.27 | 0.468 | Negative indirect effect |
| Protein | PDI_H | DDC | RVO | 0.02228 | -0.02882 | -0.000642 | 0.000413 | -0.001452 | 0.000167 | 0.54 | 0.12 | 0.211 | Negative indirect effect |
| Protein | PDI_H | POLR2F | AMD | 0.03711 | 0.05429 | 0.002015 | 0.000919 | 0.000215 | 0.003815 | 1.47 | 0.0283 | 0.0915 | Positive indirect effect |
| Protein | PDI_H | POLR2F | Cataract | 0.05856 | 0.04128 | 0.002417 | 0.001118 | 0.000226 | 0.004609 | 1.68 | 0.0306 | 0.0982 | Positive indirect effect |
| Protein | PDI_H | POLR2F | Glaucoma | -0.02346 | 0.03926 | -0.000921 | 0.000555 | -0.002009 | 0.000167 | 0.61 | 0.0969 | 0.172 | Negative indirect effect |
| Protein | PDI_H | POLR2F | DR | -0.03267 | 0.05282 | -0.001726 | 0.000985 | -0.003657 | 0.000206 | 1.4 | 0.0799 | 0.143 | Negative indirect effect |
| Protein | PDI_H | POLR2F | RVO | 0.0365 | 0.02401 | 0.000876 | 0.000627 | -0.000352 | 0.002104 | 0.74 | 0.162 | 0.283 | Positive indirect effect |
| Protein | PDI_H | GRPEL1 | AMD | -0.04715 | -0.03422 | 0.001613 | 0.001823 | -0.00196 | 0.005187 | 1.6 | 0.376 | 0.651 | Positive indirect effect |
| Protein | PDI_H | GRPEL1 | Cataract | -0.03688 | 0.02828 | -0.001043 | 0.000585 | -0.00219 | 0.000104 | 1.01 | 0.0746 | 0.134 | Negative indirect effect |
| Protein | PDI_H | GRPEL1 | Glaucoma | 0.04254 | -0.0333 | -0.001417 | 0.00061 | -0.002613 | -0.00022 | 1.16 | 0.0203 | 0.0687 | Negative indirect effect |
| Protein | PDI_H | GRPEL1 | DR | -0.05196 | 0.0312 | -0.001621 | 0.001359 | -0.004285 | 0.001043 | 1.29 | 0.233 | 0.405 | Negative indirect effect |
| Protein | PDI_H | GRPEL1 | RVO | -0.03657 | -0.02104 | 0.00077 | 0.000533 | -0.000276 | 0.001815 | 0.55 | 0.149 | 0.261 | Positive indirect effect |
| Protein | PDI_H | BST2 | AMD | 0.0427 | 0.06189 | 0.002642 | 0.001219 | 0.000253 | 0.005032 | 1.79 | 0.0302 | 0.097 | Positive indirect effect |
| Protein | PDI_H | BST2 | Cataract | 0.04963 | -0.03163 | -0.00157 | 0.001379 | -0.004272 | 0.001133 | 1.27 | 0.255 | 0.443 | Negative indirect effect |
| Protein | PDI_H | BST2 | Glaucoma | 0.04475 | 0.02426 | 0.001086 | 0.00096 | -0.000796 | 0.002967 | 0.72 | 0.258 | 0.448 | Positive indirect effect |
| Protein | PDI_H | BST2 | DR | 0.04125 | 0.03438 | 0.001418 | 0.001156 | -0.000847 | 0.003683 | 0.93 | 0.22 | 0.383 | Positive indirect effect |
| Protein | PDI_H | BST2 | RVO | 0.02155 | -0.03206 | -0.000691 | 0.000756 | -0.002172 | 0.00079 | 0.61 | 0.361 | 0.624 | Negative indirect effect |
| Protein | PDI_H | RELT | AMD | 0.03419 | 0.03361 | 0.001149 | 0.000749 | -0.000319 | 0.002617 | 0.96 | 0.125 | 0.22 | Positive indirect effect |
| Protein | PDI_H | RELT | Cataract | -0.04673 | 0.02563 | -0.001198 | 0.000894 | -0.00295 | 0.000555 | 0.82 | 0.181 | 0.315 | Negative indirect effect |
| Protein | PDI_H | RELT | Glaucoma | 0.03827 | 0.02013 | 0.00077 | 0.000549 | -0.000306 | 0.001846 | 0.55 | 0.161 | 0.281 | Positive indirect effect |
| Protein | PDI_H | RELT | DR | 0.07427 | 0.08301 | 0.006165 | 0.001769 | 0.002697 | 0.009633 | 6.14 | 0.000493 | 0.0119 | Positive indirect effect |
| Protein | PDI_H | RELT | RVO | -0.03238 | 0.0236 | -0.000764 | 0.000506 | -0.001755 | 0.000227 | 0.68 | 0.131 | 0.23 | Negative indirect effect |
| Protein | PDI_H | ADAMTS8 | AMD | 0.04065 | 0.07198 | 0.002926 | 0.000955 | 0.001053 | 0.004798 | 2.05 | 0.00219 | 0.0204 | Positive indirect effect |
| Protein | PDI_H | ADAMTS8 | Cataract | 0.03239 | 0.03955 | 0.001281 | 0.001119 | -0.000912 | 0.003474 | 1.18 | 0.252 | 0.438 | Positive indirect effect |
| Protein | PDI_H | ADAMTS8 | Glaucoma | 0.03057 | 0.02871 | 0.000878 | 0.001044 | -0.001169 | 0.002925 | 0.75 | 0.401 | 0.692 | Positive indirect effect |
| Protein | PDI_H | ADAMTS8 | DR | -0.06115 | 0.07183 | -0.004393 | 0.001606 | -0.00754 | -0.001245 | 4.3 | 0.00624 | 0.0546 | Negative indirect effect |
| Protein | PDI_H | ADAMTS8 | RVO | -0.02288 | 0.02675 | -0.000612 | 0.000564 | -0.001717 | 0.000493 | 0.54 | 0.278 | 0.482 | Negative indirect effect |
| Protein | PDI_H | SELENOP | AMD | -0.03761 | -0.06521 | 0.002453 | 0.001142 | 0.000214 | 0.004691 | 2.15 | 0.0317 | 0.101 | Positive indirect effect |
| Protein | PDI_H | SELENOP | Cataract | 0.04841 | -0.06109 | -0.002957 | 0.001419 | -0.005739 | -0.000176 | 2.66 | 0.0372 | 0.117 | Negative indirect effect |
| Protein | PDI_H | SELENOP | Glaucoma | -0.03534 | 0.0307 | -0.001085 | 0.001125 | -0.00329 | 0.001119 | 1.06 | 0.335 | 0.579 | Negative indirect effect |
| Protein | PDI_H | SELENOP | DR | 0.05709 | -0.0586 | -0.003346 | 0.001222 | -0.00574 | -0.000951 | 2.23 | 0.00618 | 0.0541 | Negative indirect effect |
| Protein | PDI_H | SELENOP | RVO | -0.0475 | 0.06494 | -0.003084 | 0.000896 | -0.00484 | -0.001328 | 2.68 | 0.000576 | 0.0137 | Negative indirect effect |
| Protein | PDI_H | TNFRSF6B | AMD | -0.04188 | -0.03497 | 0.001465 | 0.000869 | -0.000239 | 0.003168 | 1.06 | 0.0919 | 0.163 | Positive indirect effect |
| Protein | PDI_H | TNFRSF6B | Cataract | -0.04918 | 0.03009 | -0.00148 | 0.000847 | -0.00314 | 0.00018 | 1.25 | 0.0805 | 0.144 | Negative indirect effect |
| Protein | PDI_H | TNFRSF6B | Glaucoma | 0.01956 | 0.03439 | 0.000673 | 0.000422 | -0.000155 | 0.0015 | 0.48 | 0.111 | 0.196 | Positive indirect effect |
| Protein | PDI_H | TNFRSF6B | DR | 0.06243 | -0.07924 | -0.004947 | 0.001435 | -0.00776 | -0.002133 | 4.36 | 0.000569 | 0.0136 | Negative indirect effect |
| Protein | PDI_H | TNFRSF6B | RVO | -0.03244 | 0.02374 | -0.00077 | 0.000603 | -0.001951 | 0.000411 | 0.67 | 0.201 | 0.35 | Negative indirect effect |
| Protein | PDI_H | LY6D | AMD | -0.04981 | -0.02451 | 0.001221 | 0.001159 | -0.00105 | 0.003492 | 0.88 | 0.292 | 0.506 | Positive indirect effect |
| Protein | PDI_H | LY6D | Cataract | 0.08035 | -0.05697 | -0.004578 | 0.001325 | -0.007176 | -0.00198 | 4.26 | 0.000552 | 0.0132 | Negative indirect effect |
| Protein | PDI_H | LY6D | Glaucoma | 0.02484 | 0.02965 | 0.000736 | 0.000716 | -0.000667 | 0.00214 | 0.69 | 0.304 | 0.527 | Positive indirect effect |
| Protein | PDI_H | LY6D | DR | 0.04611 | -0.02617 | -0.001207 | 0.001389 | -0.003929 | 0.001516 | 1 | 0.385 | 0.666 | Negative indirect effect |
| Protein | PDI_H | LY6D | RVO | -0.03856 | -0.01938 | 0.000747 | 0.000526 | -0.000284 | 0.001779 | 0.51 | 0.156 | 0.272 | Positive indirect effect |
| Protein | PDI_H | CCL16 | AMD | -0.043 | -0.05677 | 0.002442 | 0.000796 | 0.000882 | 0.004001 | 1.73 | 0.00215 | 0.02 | Positive indirect effect |
| Protein | PDI_H | CCL16 | Cataract | -0.03404 | 0.04196 | -0.001428 | 0.001464 | -0.004299 | 0.001442 | 1.09 | 0.329 | 0.57 | Negative indirect effect |
| Protein | PDI_H | CCL16 | Glaucoma | 0.03975 | -0.01892 | -0.000752 | 0.000422 | -0.00158 | 7.6e-05 | 0.51 | 0.0749 | 0.134 | Negative indirect effect |
| Protein | PDI_H | CCL16 | DR | 0.068 | 0.06399 | 0.004351 | 0.001293 | 0.001817 | 0.006885 | 3.55 | 0.000763 | 0.0179 | Positive indirect effect |
| Protein | PDI_H | CCL16 | RVO | 0.02318 | 0.02456 | 0.000569 | 0.000555 | -0.000518 | 0.001656 | 0.58 | 0.305 | 0.528 | Positive indirect effect |
| Protein | PDI_H | CD99L2 | AMD | -0.02924 | 0.03556 | -0.00104 | 0.001048 | -0.003094 | 0.001014 | 0.72 | 0.321 | 0.556 | Negative indirect effect |
| Protein | PDI_H | CD99L2 | Cataract | -0.05792 | 0.06564 | -0.003802 | 0.001075 | -0.005909 | -0.001695 | 2.81 | 0.000405 | 0.00989 | Negative indirect effect |
| Protein | PDI_H | CD99L2 | Glaucoma | -0.05381 | -0.03352 | 0.001804 | 0.000824 | 0.000189 | 0.003419 | 1.64 | 0.0286 | 0.0923 | Positive indirect effect |
| Protein | PDI_H | CD99L2 | DR | -0.06427 | 0.06249 | -0.004016 | 0.000985 | -0.005947 | -0.002084 | 4.07 | 4.6e-05 | 0.00181 | Negative indirect effect |
| Protein | PDI_H | CD99L2 | RVO | 0.02306 | 0.02422 | 0.000558 | 0.000514 | -0.000449 | 0.001566 | 0.53 | 0.277 | 0.481 | Positive indirect effect |
| Protein | PDI_H | FSTL3 | AMD | 0.07514 | 0.05939 | 0.004462 | 0.001168 | 0.002174 | 0.006751 | 3.06 | 0.000132 | 0.00375 | Positive indirect effect |
| Protein | PDI_H | FSTL3 | Cataract | -0.04684 | 0.02804 | -0.001313 | 0.001444 | -0.004144 | 0.001518 | 1.08 | 0.363 | 0.628 | Negative indirect effect |
| Protein | PDI_H | FSTL3 | Glaucoma | -0.03745 | -0.03454 | 0.001294 | 0.000639 | 4.2e-05 | 0.002545 | 1.17 | 0.0428 | 0.133 | Positive indirect effect |
| Protein | PDI_H | FSTL3 | DR | -0.0603 | -0.03006 | 0.001813 | 0.001553 | -0.001232 | 0.004857 | 1.63 | 0.243 | 0.423 | Positive indirect effect |
| Protein | PDI_H | FSTL3 | RVO | 0.0319 | 0.04278 | 0.001365 | 0.000657 | 7.6e-05 | 0.002653 | 1.02 | 0.0379 | 0.119 | Positive indirect effect |
| Protein | PDI_H | CCL3 | AMD | -0.05875 | -0.07709 | 0.004529 | 0.001275 | 0.00203 | 0.007028 | 3.6 | 0.000382 | 0.00937 | Positive indirect effect |
| Protein | PDI_H | CCL3 | Cataract | 0.0341 | 0.03938 | 0.001343 | 0.000898 | -0.000418 | 0.003103 | 1.15 | 0.135 | 0.237 | Positive indirect effect |
| Protein | PDI_H | CCL3 | Glaucoma | -0.04292 | -0.02715 | 0.001165 | 0.000531 | 0.000124 | 0.002207 | 0.92 | 0.0283 | 0.0916 | Positive indirect effect |

| Feature_type | Exposure | Mediator | Outcome | a_path_beta | b_path_beta | Indirect_effect | SE | CI_lower | CI_upper | Proportion_mediated_percent | P | FDR_q | Effect_direction |
|---|---|---|---|---|---|---|---|---|---|---|---|---|---|
| Protein | PDI_H | CCL3 | DR | -0.07848 | 0.07133 | -0.005598 | 0.00164 | -0.008813 | -0.002383 | 5.03 | 0.000644 | 0.0153 | Negative indirect effect |
| Protein | PDI_H | CCL3 | RVO | 0.02172 | -0.02939 | -0.000639 | 0.000726 | -0.002062 | 0.000785 | 0.53 | 0.379 | 0.656 | Negative indirect effect |
| Protein | PDI_H | FABP4 | AMD | 0.05782 | 0.05028 | 0.002907 | 0.001021 | 0.000905 | 0.004909 | 2.37 | 0.00442 | 0.0393 | Positive indirect effect |
| Protein | PDI_H | FABP4 | Cataract | 0.08551 | 0.04219 | 0.003608 | 0.000916 | 0.001812 | 0.005404 | 3.33 | 8.26e-05 | 0.00263 | Positive indirect effect |
| Protein | PDI_H | FABP4 | Glaucoma | -0.02446 | -0.03397 | 0.000831 | 0.000771 | -0.00068 | 0.002342 | 0.56 | 0.281 | 0.488 | Positive indirect effect |
| Protein | PDI_H | FABP4 | DR | -0.10666 | 0.05264 | -0.005614 | 0.001376 | -0.008311 | -0.002917 | 5.78 | 4.5e-05 | 0.00179 | Negative indirect effect |
| Protein | PDI_H | FABP4 | RVO | -0.02266 | 0.03594 | -0.000814 | 0.000883 | -0.002545 | 0.000916 | 0.74 | 0.356 | 0.616 | Negative indirect effect |
| Protein | PDI_H | CNTN1 | AMD | -0.04669 | -0.03506 | 0.001637 | 0.0007 | 0.000265 | 0.003009 | 1.16 | 0.0194 | 0.0661 | Positive indirect effect |
| Protein | PDI_H | CNTN1 | Cataract | 0.03282 | 0.03292 | 0.00108 | 0.001139 | -0.001152 | 0.003313 | 0.79 | 0.343 | 0.593 | Positive indirect effect |
| Protein | PDI_H | CNTN1 | Glaucoma | -0.03564 | -0.02268 | 0.000808 | 0.000493 | -0.000158 | 0.001775 | 0.8 | 0.101 | 0.179 | Positive indirect effect |
| Protein | PDI_H | CNTN1 | DR | -0.10581 | 0.05177 | -0.005478 | 0.001603 | -0.008619 | -0.002337 | 3.65 | 0.00063 | 0.015 | Negative indirect effect |
| Protein | PDI_H | CNTN1 | RVO | 0.03379 | -0.02396 | -0.00081 | 0.000557 | -0.001902 | 0.000282 | 0.59 | 0.146 | 0.256 | Negative indirect effect |
| Protein | PDI_H | CFB | AMD | 0.06546 | 0.05562 | 0.003641 | 0.001025 | 0.001632 | 0.00565 | 3.11 | 0.000382 | 0.00938 | Positive indirect effect |
| Protein | PDI_H | CFB | Cataract | -0.02571 | 0.03773 | -0.00097 | 0.001088 | -0.003102 | 0.001162 | 0.81 | 0.373 | 0.644 | Negative indirect effect |
| Protein | PDI_H | CFB | Glaucoma | -0.03504 | -0.01825 | 0.00064 | 0.000443 | -0.000228 | 0.001508 | 0.44 | 0.149 | 0.26 | Positive indirect effect |
| Protein | PDI_H | CFB | DR | -0.04977 | -0.03511 | 0.001747 | 0.00135 | -0.000899 | 0.004394 | 1.26 | 0.196 | 0.341 | Positive indirect effect |
| Protein | PDI_H | CFB | RVO | 0.0228 | -0.02698 | -0.000615 | 0.000348 | -0.001297 | 6.7e-05 | 0.4 | 0.0771 | 0.138 | Negative indirect effect |
| Protein | PDI_H | TNFRSF10A | AMD | -0.02765 | 0.04052 | -0.001121 | 0.00128 | -0.00363 | 0.001389 | 1.11 | 0.382 | 0.66 | Negative indirect effect |
| Protein | PDI_H | TNFRSF10A | Cataract | -0.03495 | 0.02949 | -0.001031 | 0.000713 | -0.002428 | 0.000366 | 0.89 | 0.148 | 0.26 | Negative indirect effect |
| Protein | PDI_H | TNFRSF10A | Glaucoma | -0.04414 | 0.02239 | -0.000988 | 0.000909 | -0.00277 | 0.000793 | 0.78 | 0.277 | 0.48 | Negative indirect effect |
| Protein | PDI_H | TNFRSF10A | DR | -0.07668 | 0.07603 | -0.00583 | 0.001429 | -0.00863 | -0.00303 | 5.61 | 4.48e-05 | 0.00178 | Negative indirect effect |
| Protein | PDI_H | TNFRSF10A | RVO | -0.03165 | 0.0198 | -0.000626 | 0.000394 | -0.001399 | 0.000146 | 0.49 | 0.112 | 0.197 | Negative indirect effect |
| Protein | PDI_H | SPON2 | AMD | -0.05033 | -0.03564 | 0.001794 | 0.000829 | 0.000169 | 0.003419 | 1.22 | 0.0305 | 0.0979 | Positive indirect effect |
| Protein | PDI_H | SPON2 | Cataract | -0.03 | 0.03428 | -0.001028 | 0.000673 | -0.002348 | 0.000291 | 1.01 | 0.127 | 0.223 | Negative indirect effect |
| Protein | PDI_H | SPON2 | Glaucoma | -0.02163 | 0.02972 | -0.000643 | 0.000632 | -0.001882 | 0.000597 | 0.44 | 0.309 | 0.536 | Negative indirect effect |
| Protein | PDI_H | SPON2 | DR | -0.04564 | -0.03429 | 0.001565 | 0.001759 | -0.001883 | 0.005013 | 1.41 | 0.374 | 0.646 | Positive indirect effect |
| Protein | PDI_H | SPON2 | RVO | 0.02651 | -0.02402 | -0.000637 | 0.000681 | -0.001972 | 0.000698 | 0.61 | 0.35 | 0.605 | Negative indirect effect |
| Protein | PDI_H | TFF1 | AMD | 0.0587 | 0.05593 | 0.003283 | 0.001199 | 0.000932 | 0.005634 | 2.99 | 0.0062 | 0.0543 | Positive indirect effect |
| Protein | PDI_H | TFF1 | Cataract | 0.05527 | 0.04108 | 0.00227 | 0.000906 | 0.000494 | 0.004047 | 1.87 | 0.0123 | 0.0457 | Positive indirect effect |
| Protein | PDI_H | TFF1 | Glaucoma | 0.02583 | -0.03131 | -0.000809 | 0.000833 | -0.002442 | 0.000824 | 0.73 | 0.332 | 0.574 | Negative indirect effect |
| Protein | PDI_H | TFF1 | DR | 0.05137 | -0.03236 | -0.001663 | 0.001918 | -0.005423 | 0.002098 | 1.34 | 0.386 | 0.668 | Negative indirect effect |
| Protein | PDI_H | TFF1 | RVO | 0.02151 | -0.02584 | -0.000556 | 0.000456 | -0.001449 | 0.000337 | 0.56 | 0.222 | 0.387 | Negative indirect effect |
| Protein | PDI_H | CNTN5 | AMD | -0.03551 | -0.02935 | 0.001042 | 0.000996 | -0.00091 | 0.002995 | 0.77 | 0.295 | 0.512 | Positive indirect effect |
| Protein | PDI_H | CNTN5 | Cataract | 0.07096 | -0.05974 | -0.004239 | 0.001254 | -0.006698 | -0.001781 | 4.05 | 0.000726 | 0.0171 | Negative indirect effect |
| Protein | PDI_H | CNTN5 | Glaucoma | 0.03697 | -0.0189 | -0.000699 | 0.000485 | -0.001649 | 0.000251 | 0.49 | 0.149 | 0.262 | Negative indirect effect |
| Protein | PDI_H | CNTN5 | DR | -0.05623 | 0.05339 | -0.003002 | 0.001319 | -0.005588 | -0.000416 | 2 | 0.0229 | 0.0761 | Negative indirect effect |
| Protein | PDI_H | CNTN5 | RVO | 0.04021 | 0.01925 | 0.000774 | 0.0008 | -0.000794 | 0.002342 | 0.76 | 0.333 | 0.577 | Positive indirect effect |
| Protein | PDI_H | CD302 | AMD | -0.04305 | -0.02932 | 0.001262 | 0.000983 | -0.000665 | 0.003189 | 1.3 | 0.199 | 0.347 | Positive indirect effect |
| Protein | PDI_H | CD302 | Cataract | 0.03585 | 0.05782 | 0.002073 | 0.000931 | 0.000248 | 0.003898 | 1.56 | 0.026 | 0.085 | Positive indirect effect |
| Protein | PDI_H | CD302 | Glaucoma | 0.03068 | -0.02939 | -0.000902 | 0.000954 | -0.002772 | 0.000969 | 0.69 | 0.345 | 0.597 | Negative indirect effect |
| Protein | PDI_H | CD302 | DR | 0.08139 | 0.07113 | 0.005789 | 0.001352 | 0.00314 | 0.008439 | 5.94 | 1.85e-05 | 0.00119 | Positive indirect effect |
| Protein | PDI_H | CD302 | RVO | 0.0314 | -0.01712 | -0.000538 | 0.00052 | -0.001557 | 0.000481 | 0.43 | 0.301 | 0.522 | Negative indirect effect |
| Protein | PDI_H | B2M | AMD | 0.02948 | -0.04073 | -0.001201 | 0.000766 | -0.002702 | 0.000301 | 0.86 | 0.117 | 0.206 | Negative indirect effect |
| Protein | PDI_H | B2M | Cataract | 0.03042 | 0.04509 | 0.001372 | 0.001537 | -0.00164 | 0.004384 | 0.99 | 0.372 | 0.644 | Positive indirect effect |
| Protein | PDI_H | B2M | Glaucoma | 0.04499 | 0.04948 | 0.002226 | 0.000579 | 0.001091 | 0.003361 | 1.6 | 0.000121 | 0.0035 | Positive indirect effect |
| Protein | PDI_H | B2M | DR | -0.04887 | 0.04711 | -0.002302 | 0.00107 | -0.004399 | -0.000206 | 1.66 | 0.0314 | 0.1 | Negative indirect effect |
| Protein | PDI_H | B2M | RVO | -0.03201 | 0.03902 | -0.001249 | 0.000584 | -0.002393 | -0.000105 | 1.21 | 0.0324 | 0.103 | Negative indirect effect |
| Protein | PDI_H | ADM | AMD | -0.07298 | -0.05524 | 0.004032 | 0.001178 | 0.001722 | 0.006341 | 2.76 | 0.000622 | 0.0148 | Positive indirect effect |
| Protein | PDI_H | ADM | Cataract | 0.04558 | -0.04813 | -0.002194 | 0.000818 | -0.003798 | -0.00059 | 1.94 | 0.00734 | 0.064 | Negative indirect effect |
| Protein | PDI_H | ADM | Glaucoma | -0.03558 | -0.01789 | 0.000636 | 0.000466 | -0.000277 | 0.00155 | 0.63 | 0.172 | 0.3 | Positive indirect effect |
| Protein | PDI_H | ADM | DR | -0.02927 | -0.03566 | 0.001044 | 0.000793 | -0.00051 | 0.002598 | 0.72 | 0.188 | 0.328 | Positive indirect effect |
| Protein | PDI_H | ADM | RVO | 0.03212 | 0.01903 | 0.000611 | 0.000709 | -0.000779 | 0.002001 | 0.45 | 0.389 | 0.672 | Positive indirect effect |
| Protein | PDI_U | IGFBP1 | AMD | -0.03238 | 0.04893 | -0.001585 | 0.001713 | -0.004941 | 0.001772 | 1.54 | 0.355 | 0.614 | Negative indirect effect |
| Protein | PDI_U | IGFBP1 | Cataract | -0.07214 | 0.0526 | -0.003795 | 0.001368 | -0.006477 | -0.001113 | 2.58 | 0.00555 | 0.0488 | Negative indirect effect |
| Protein | PDI_U | IGFBP1 | Glaucoma | 0.036 | 0.03034 | 0.001092 | 0.000771 | -0.000419 | 0.002604 | 0.86 | 0.157 | 0.274 | Positive indirect effect |
| Protein | PDI_U | IGFBP1 | DR | -0.07446 | -0.05132 | 0.003822 | 0.001897 | 0.000103 | 0.00754 | 2.49 | 0.044 | 0.136 | Positive indirect effect |
| Protein | PDI_U | IGFBP1 | RVO | 0.03589 | 0.05121 | 0.001838 | 0.000843 | 0.000185 | 0.003491 | 1.86 | 0.0293 | 0.0944 | Positive indirect effect |
| Protein | PDI_U | INHBC | AMD | 0.03506 | 0.04658 | 0.001633 | 0.001821 | -0.001936 | 0.005202 | 1.22 | 0.37 | 0.64 | Positive indirect effect |
| Protein | PDI_U | INHBC | Cataract | -0.04701 | -0.02814 | 0.001323 | 0.001108 | -0.000848 | 0.003494 | 1.26 | 0.232 | 0.404 | Positive indirect effect |
| Protein | PDI_U | INHBC | Glaucoma | -0.04301 | -0.02681 | 0.001153 | 0.001143 | -0.001088 | 0.003394 | 1.14 | 0.313 | 0.543 | Positive indirect effect |
| Protein | PDI_U | INHBC | DR | 0.03994 | 0.03802 | 0.001519 | 0.001529 | -0.001479 | 0.004516 | 1.57 | 0.321 | 0.556 | Positive indirect effect |
| Protein | PDI_U | INHBC | RVO | 0.03963 | -0.02055 | -0.000814 | 0.000484 | -0.001764 | 0.000135 | 0.73 | 0.0927 | 0.165 | Negative indirect effect |
| Protein | PDI_U | TFF2 | AMD | -0.04292 | 0.07363 | -0.00316 | 0.001162 | -0.005438 | -0.000882 | 2.41 | 0.00654 | 0.0572 | Negative indirect effect |
| Protein | PDI_U | TFF2 | Cataract | 0.07439 | -0.05088 | -0.003785 | 0.001111 | -0.005963 | -0.001607 | 2.9 | 0.00066 | 0.0156 | Negative indirect effect |
| Protein | PDI_U | TFF2 | Glaucoma | -0.02822 | 0.04193 | -0.001183 | 0.001243 | -0.00362 | 0.001253 | 0.94 | 0.341 | 0.591 | Negative indirect effect |
| Protein | PDI_U | TFF2 | DR | 0.07789 | 0.07251 | 0.005648 | 0.001511 | 0.002687 | 0.008609 | 5.66 | 0.000185 | 0.00495 | Positive indirect effect |
| Protein | PDI_U | TFF2 | RVO | -0.02988 | 0.03445 | -0.001029 | 0.001003 | -0.002996 | 0.000937 | 0.79 | 0.305 | 0.528 | Negative indirect effect |
| Protein | PDI_U | LEP | AMD | -0.03292 | -0.03763 | 0.001239 | 0.00108 | -0.000877 | 0.003355 | 1.26 | 0.251 | 0.436 | Positive indirect effect |
| Protein | PDI_U | LEP | Cataract | 0.10856 | -0.05495 | -0.005966 | 0.00161 | -0.00912 | -0.002811 | 4.87 | 0.00021 | 0.0055 | Negative indirect effect |
| Protein | PDI_U | LEP | Glaucoma | 0.0413 | -0.02408 | -0.000994 | 0.000753 | -0.002471 | 0.000482 | 0.67 | 0.187 | 0.326 | Negative indirect effect |
| Protein | PDI_U | LEP | DR | -0.05574 | -0.09002 | 0.005018 | 0.001845 | 0.001402 | 0.008634 | 4.39 | 0.00653 | 0.0571 | Positive indirect effect |
| Protein | PDI_U | LEP | RVO | -0.02609 | 0.03117 | -0.000813 | 0.000578 | -0.001947 | 0.00032 | 0.57 | 0.16 | 0.279 | Negative indirect effect |
| Protein | PDI_U | VSIG2 | AMD | 0.03344 | 0.03856 | 0.00129 | 0.00102 | -0.00071 | 0.003289 | 1.15 | 0.206 | 0.359 | Positive indirect effect |
| Protein | PDI_U | VSIG2 | Cataract | 0.08361 | 0.04997 | 0.004178 | 0.001231 | 0.001766 | 0.006591 | 4.22 | 0.000688 | 0.0163 | Positive indirect effect |
| Protein | PDI_U | VSIG2 | Glaucoma | -0.06439 | -0.04098 | 0.002639 | 0.000905 | 0.000864 | 0.004413 | 1.98 | 0.00356 | 0.032 | Positive indirect effect |
| Protein | PDI_U | VSIG2 | DR | 0.06841 | -0.07521 | -0.005145 | 0.001471 | -0.008029 | -0.002261 | 4.79 | 0.000471 | 0.0114 | Negative indirect effect |
| Protein | PDI_U | VSIG2 | RVO | -0.06552 | 0.03132 | -0.002052 | 0.000755 | -0.003533 | -0.000571 | 1.36 | 0.0066 | 0.0577 | Negative indirect effect |
| Protein | PDI_U | OXT | AMD | 0.0383 | 0.02707 | 0.001036 | 0.001229 | -0.001372 | 0.003445 | 1.06 | 0.399 | 0.69 | Positive indirect effect |
| Protein | PDI_U | OXT | Cataract | 0.0806 | 0.05679 | 0.004578 | 0.001716 | 0.001214 | 0.007941 | 4.33 | 0.00765 | 0.0666 | Positive indirect effect |
| Protein | PDI_U | OXT | Glaucoma | -0.04852 | 0.02406 | -0.001167 | 0.001078 | -0.00328 | 0.000945 | 0.98 | 0.279 | 0.484 | Negative indirect effect |
| Protein | PDI_U | OXT | DR | 0.08519 | 0.05765 | 0.004911 | 0.001829 | 0.001327 | 0.008495 | 3.94 | 0.00723 | 0.063 | Positive indirect effect |
| Protein | PDI_U | OXT | RVO | 0.03899 | -0.02547 | -0.000993 | 0.000677 | -0.002321 | 0.000335 | 0.75 | 0.143 | 0.25 | Negative indirect effect |
| Protein | PDI_U | RLN2 | AMD | 0.05173 | 0.03209 | 0.00166 | 0.001126 | -0.000546 | 0.003866 | 1.41 | 0.14 | 0.246 | Positive indirect effect |
| Protein | PDI_U | RLN2 | Cataract | -0.06126 | -0.0339 | 0.002076 | 0.001001 | 0.000114 | 0.004039 | 1.88 | 0.0381 | 0.12 | Positive indirect effect |
| Protein | PDI_U | RLN2 | Glaucoma | -0.0506 | 0.04916 | -0.002487 | 0.00087 | -0.004192 | -0.000783 | 2.42 | 0.00424 | 0.0377 | Negative indirect effect |
| Protein | PDI_U | RLN2 | DR | -0.09119 | 0.05743 | -0.005237 | 0.001525 | -0.008226 | -0.002249 | 4.6 | 0.000593 | 0.0141 | Negative indirect effect |
| Protein | PDI_U | RLN2 | RVO | 0.04548 | 0.05258 | 0.002391 | 0.00061 | 0.001196 | 0.003586 | 1.85 | 8.79e-05 | 0.00275 | Positive indirect effect |
| Protein | PDI_U | CDHR2 | AMD | 0.03468 | 0.03282 | 0.001138 | 0.000691 | -0.000215 | 0.002492 | 0.98 | 0.0992 | 0.176 | Positive indirect effect |
| Protein | PDI_U | CDHR2 | Cataract | -0.04994 | -0.028 | 0.001398 | 0.001289 | -0.001128 | 0.003924 | 1.09 | 0.278 | 0.482 | Positive indirect effect |
| Protein | PDI_U | CDHR2 | Glaucoma | -0.04031 | 0.01953 | -0.000787 | 0.000513 | -0.001793 | 0.000218 | 0.7 | 0.125 | 0.22 | Negative indirect effect |
| Protein | PDI_U | CDHR2 | DR | 0.06916 | 0.05478 | 0.003789 | 0.001379 | 0.001085 | 0.006492 | 2.51 | 0.00601 | 0.0527 | Positive indirect effect |
| Protein | PDI_U | CDHR2 | RVO | -0.04973 | 0.03047 | -0.001515 | 0.000739 | -0.002965 | -6.6e-05 | 1.47 | 0.0404 | 0.126 | Negative indirect effect |
| Protein | PDI_U | TFF1 | AMD | 0.05819 | 0.05545 | 0.003227 | 0.001186 | 0.000902 | 0.005551 | 2.94 | 0.00651 | 0.0569 | Positive indirect effect |
| Protein | PDI_U | TFF1 | Cataract | 0.06047 | 0.04494 | 0.002718 | 0.000986 | 0.000786 | 0.00465 | 2.24 | 0.00584 | 0.0512 | Positive indirect effect |
| Protein | PDI_U | TFF1 | Glaucoma | -0.02666 | -0.03231 | 0.000861 | 0.001025 | -0.001147 | 0.00287 | 0.77 | 0.401 | 0.693 | Positive indirect effect |
| Protein | PDI_U | TFF1 | DR | -0.0613 | -0.03862 | 0.002367 | 0.000964 | 0.000477 | 0.004257 | 1.9 | 0.0141 | 0.0509 | Positive indirect effect |
| Protein | PDI_U | TFF1 | RVO | -0.02677 | -0.03217 | 0.000861 | 0.000697 | -0.000504 | 0.002227 | 0.86 | 0.216 | 0.377 | Positive indirect effect |
| Protein | PDI_U | FABP2 | AMD | -0.07276 | -0.03651 | 0.002656 | 0.000937 | 0.00082 | 0.004492 | 2.01 | 0.00458 | 0.0406 | Positive indirect effect |
| Protein | PDI_U | FABP2 | Cataract | -0.06448 | 0.05718 | -0.003687 | 0.00117 | -0.00598 | -0.001394 | 3.51 | 0.00163 | 0.0156 | Negative indirect effect |
| Protein | PDI_U | FABP2 | Glaucoma | -0.0502 | 0.06014 | -0.003019 | 0.000834 | -0.004654 | -0.001384 | 2.36 | 0.000295 | 0.00742 | Negative indirect effect |

| Feature_type | Exposure | Mediator | Outcome | a_path_beta | b_path_beta | Indirect_effect | SE | CI_lower | CI_upper | Proportion_mediated_percent | P | FDR_q | Effect_direction |
|---|---|---|---|---|---|---|---|---|---|---|---|---|---|
| Protein | PDI_U | FABP2 | DR | 0.04872 | -0.03199 | -0.001559 | 0.001328 | -0.004162 | 0.001045 | 1.31 | 0.241 | 0.418 | Negative indirect effect |
| Protein | PDI_U | FABP2 | RVO | 0.03678 | -0.02145 | -0.000789 | 0.000501 | -0.001771 | 0.000194 | 0.56 | 0.116 | 0.204 | Negative indirect effect |
| Protein | PDI_U | IGSF9 | AMD | 0.04069 | -0.03643 | -0.001482 | 0.001067 | -0.003574 | 0.00061 | 1.23 | 0.165 | 0.288 | Negative indirect effect |
| Protein | PDI_U | IGSF9 | Cataract | 0.05117 | 0.03189 | 0.001632 | 0.001467 | -0.001244 | 0.004507 | 1.48 | 0.266 | 0.462 | Positive indirect effect |
| Protein | PDI_U | IGSF9 | Glaucoma | 0.03389 | -0.02542 | -0.000861 | 0.000994 | -0.002809 | 0.001086 | 0.74 | 0.386 | 0.667 | Negative indirect effect |
| Protein | PDI_U | IGSF9 | DR | -0.06829 | -0.05796 | 0.003958 | 0.001452 | 0.001112 | 0.006805 | 3.29 | 0.00642 | 0.0562 | Positive indirect effect |
| Protein | PDI_U | IGSF9 | RVO | -0.03377 | 0.02391 | -0.000807 | 0.000794 | -0.002363 | 0.000748 | 0.55 | 0.309 | 0.535 | Negative indirect effect |
| Protein | PDI_U | SSC4D | AMD | 0.04419 | 0.03554 | 0.001571 | 0.001535 | -0.001437 | 0.004579 | 1.37 | 0.306 | 0.53 | Positive indirect effect |
| Protein | PDI_U | SSC4D | Cataract | 0.0563 | -0.03444 | -0.001939 | 0.000808 | -0.003522 | -0.000355 | 1.35 | 0.0164 | 0.0576 | Negative indirect effect |
| Protein | PDI_U | SSC4D | Glaucoma | -0.02524 | 0.0434 | -0.001095 | 0.001308 | -0.003658 | 0.001468 | 0.8 | 0.402 | 0.695 | Negative indirect effect |
| Protein | PDI_U | SSC4D | DR | 0.03387 | -0.05799 | -0.001964 | 0.001421 | -0.00475 | 0.000821 | 1.38 | 0.167 | 0.292 | Negative indirect effect |
| Protein | PDI_U | SSC4D | RVO | 0.03275 | -0.02234 | -0.000732 | 0.000478 | -0.001668 | 0.000205 | 0.53 | 0.126 | 0.221 | Negative indirect effect |
| Protein | PDI_U | SLC39A5 | AMD | 0.03317 | 0.02914 | 0.000967 | 0.000583 | -0.000175 | 0.002109 | 0.91 | 0.0971 | 0.172 | Positive indirect effect |
| Protein | PDI_U | SLC39A5 | Cataract | 0.07813 | -0.05391 | -0.004212 | 0.00156 | -0.007269 | -0.001155 | 4.01 | 0.00693 | 0.0605 | Negative indirect effect |
| Protein | PDI_U | SLC39A5 | Glaucoma | 0.05877 | 0.05369 | 0.003156 | 0.000891 | 0.001409 | 0.004903 | 2.81 | 0.000399 | 0.00976 | Positive indirect effect |
| Protein | PDI_U | SLC39A5 | DR | -0.04829 | 0.06194 | -0.002991 | 0.001264 | -0.005469 | -0.000514 | 2.27 | 0.018 | 0.062 | Negative indirect effect |
| Protein | PDI_U | SLC39A5 | RVO | -0.03677 | 0.01866 | -0.000686 | 0.000493 | -0.001653 | 0.00028 | 0.68 | 0.164 | 0.287 | Negative indirect effect |
| Protein | PDI_U | IGFBP2 | AMD | -0.05644 | 0.06978 | -0.003939 | 0.001017 | -0.005932 | -0.001946 | 3.77 | 0.000107 | 0.00319 | Negative indirect effect |
| Protein | PDI_U | IGFBP2 | Cataract | 0.06226 | -0.06397 | -0.003983 | 0.001089 | -0.006118 | -0.001848 | 3.38 | 0.000256 | 0.00652 | Negative indirect effect |
| Protein | PDI_U | IGFBP2 | Glaucoma | -0.03537 | 0.02996 | -0.00106 | 0.000759 | -0.002548 | 0.000428 | 0.88 | 0.163 | 0.285 | Negative indirect effect |
| Protein | PDI_U | IGFBP2 | DR | 0.0335 | 0.04873 | 0.001632 | 0.001659 | -0.001619 | 0.004884 | 1.34 | 0.325 | 0.563 | Positive indirect effect |
| Protein | PDI_U | IGFBP2 | RVO | -0.04048 | 0.02196 | -0.000889 | 0.000664 | -0.00219 | 0.000412 | 0.71 | 0.181 | 0.315 | Negative indirect effect |
| Protein | PDI_U | CKB | AMD | 0.03039 | 0.04017 | 0.001221 | 0.001172 | -0.001077 | 0.003519 | 0.92 | 0.298 | 0.516 | Positive indirect effect |
| Protein | PDI_U | CKB | Cataract | 0.0346 | 0.03335 | 0.001154 | 0.001078 | -0.00096 | 0.003267 | 1.08 | 0.285 | 0.494 | Positive indirect effect |
| Protein | PDI_U | CKB | Glaucoma | 0.02643 | -0.03488 | -0.000922 | 0.000592 | -0.002082 | 0.000238 | 0.76 | 0.119 | 0.21 | Negative indirect effect |
| Protein | PDI_U | CKB | DR | -0.07918 | 0.06201 | -0.00491 | 0.001371 | -0.007598 | -0.002223 | 4.53 | 0.000342 | 0.00848 | Negative indirect effect |
| Protein | PDI_U | CKB | RVO | -0.02232 | 0.02983 | -0.000666 | 0.000642 | -0.001925 | 0.000593 | 0.54 | 0.3 | 0.52 | Negative indirect effect |
| Protein | PDI_U | GUCA2A | AMD | 0.03606 | 0.02656 | 0.000958 | 0.000674 | -0.000364 | 0.00228 | 0.67 | 0.156 | 0.272 | Positive indirect effect |
| Protein | PDI_U | GUCA2A | Cataract | 0.07957 | 0.04587 | 0.00365 | 0.001082 | 0.00153 | 0.005771 | 2.56 | 0.00074 | 0.0174 | Positive indirect effect |
| Protein | PDI_U | GUCA2A | Glaucoma | 0.03064 | 0.0343 | 0.001051 | 0.00115 | -0.001203 | 0.003305 | 0.92 | 0.361 | 0.624 | Positive indirect effect |
| Protein | PDI_U | GUCA2A | DR | 0.06511 | 0.09617 | 0.006261 | 0.001864 | 0.002607 | 0.009915 | 4.97 | 0.000783 | 0.0184 | Positive indirect effect |
| Protein | PDI_U | GUCA2A | RVO | -0.0346 | 0.02224 | -0.00077 | 0.000614 | -0.001973 | 0.000433 | 0.66 | 0.21 | 0.365 | Negative indirect effect |
| Protein | PDI_U | CNTN4 | AMD | 0.0325 | 0.05612 | 0.001824 | 0.000822 | 0.000214 | 0.003434 | 1.23 | 0.0264 | 0.0862 | Positive indirect effect |
| Protein | PDI_U | CNTN4 | Cataract | -0.03163 | -0.03605 | 0.00114 | 0.000956 | -0.000733 | 0.003013 | 1.04 | 0.233 | 0.405 | Positive indirect effect |
| Protein | PDI_U | CNTN4 | Glaucoma | 0.03876 | -0.02383 | -0.000924 | 0.000977 | -0.002838 | 0.000991 | 0.64 | 0.344 | 0.596 | Negative indirect effect |
| Protein | PDI_U | CNTN4 | DR | -0.05619 | -0.03398 | 0.001909 | 0.001361 | -0.000759 | 0.004578 | 1.5 | 0.161 | 0.281 | Positive indirect effect |
| Protein | PDI_U | CNTN4 | RVO | -0.04343 | 0.02077 | -0.000902 | 0.000877 | -0.002621 | 0.000817 | 0.64 | 0.304 | 0.527 | Negative indirect effect |
| Protein | PDI_U | XPNPEP2 | AMD | -0.04952 | -0.0287 | 0.001421 | 0.00116 | -0.000853 | 0.003696 | 1.24 | 0.221 | 0.384 | Positive indirect effect |
| Protein | PDI_U | XPNPEP2 | Cataract | -0.05063 | 0.03073 | -0.001556 | 0.001082 | -0.003678 | 0.000566 | 1.22 | 0.151 | 0.264 | Negative indirect effect |
| Protein | PDI_U | XPNPEP2 | Glaucoma | -0.03525 | 0.02231 | -0.000786 | 0.000577 | -0.001917 | 0.000345 | 0.82 | 0.173 | 0.302 | Negative indirect effect |
| Protein | PDI_U | XPNPEP2 | DR | -0.08788 | 0.04762 | -0.004185 | 0.001371 | -0.006873 | -0.001498 | 3.3 | 0.00227 | 0.021 | Negative indirect effect |
| Protein | PDI_U | XPNPEP2 | RVO | -0.03344 | 0.0234 | -0.000783 | 0.000748 | -0.002248 | 0.000683 | 0.5 | 0.295 | 0.512 | Negative indirect effect |
| Protein | PDI_U | PPY | AMD | 0.02953 | 0.04437 | 0.00131 | 0.001367 | -0.00137 | 0.00399 | 0.95 | 0.338 | 0.585 | Positive indirect effect |
| Protein | PDI_U | PPY | Cataract | -0.06792 | 0.06966 | -0.004731 | 0.001397 | -0.00747 | -0.001993 | 4.58 | 0.000707 | 0.0167 | Negative indirect effect |
| Protein | PDI_U | PPY | Glaucoma | 0.02439 | 0.02905 | 0.000709 | 0.00057 | -0.000408 | 0.001825 | 0.47 | 0.214 | 0.372 | Positive indirect effect |
| Protein | PDI_U | PPY | DR | 0.04187 | 0.03974 | 0.001664 | 0.001971 | -0.002199 | 0.005527 | 1.55 | 0.399 | 0.689 | Positive indirect effect |
| Protein | PDI_U | PPY | RVO | -0.03679 | 0.02239 | -0.000824 | 0.000856 | -0.002502 | 0.000854 | 0.78 | 0.336 | 0.582 | Negative indirect effect |
| Protein | PDI_U | CDHR5 | AMD | -0.03686 | 0.02464 | -0.000908 | 0.000582 | -0.00205 | 0.000233 | 0.68 | 0.119 | 0.209 | Negative indirect effect |
| Protein | PDI_U | CDHR5 | Cataract | -0.03683 | -0.03642 | 0.001341 | 0.000817 | -0.000259 | 0.002942 | 1.04 | 0.1 | 0.178 | Positive indirect effect |
| Protein | PDI_U | CDHR5 | Glaucoma | -0.0341 | 0.02303 | -0.000785 | 0.00082 | -0.002392 | 0.000822 | 0.79 | 0.338 | 0.585 | Negative indirect effect |
| Protein | PDI_U | CDHR5 | DR | 0.03866 | 0.03495 | 0.001351 | 0.000832 | -0.00028 | 0.002982 | 1.28 | 0.104 | 0.185 | Positive indirect effect |
| Protein | PDI_U | CDHR5 | RVO | -0.02929 | 0.02639 | -0.000773 | 0.000784 | -0.00231 | 0.000765 | 0.79 | 0.325 | 0.562 | Negative indirect effect |
| Protein | PDI_U | CES1 | AMD | 0.05825 | 0.03168 | 0.001845 | 0.000746 | 0.000383 | 0.003308 | 1.42 | 0.0134 | 0.0489 | Positive indirect effect |
| Protein | PDI_U | CES1 | Cataract | 0.07416 | 0.04883 | 0.003621 | 0.001136 | 0.001394 | 0.005849 | 2.69 | 0.00144 | 0.014 | Positive indirect effect |
| Protein | PDI_U | CES1 | Glaucoma | 0.04729 | 0.02672 | 0.001264 | 0.000535 | 0.000215 | 0.002312 | 1.19 | 0.0181 | 0.0625 | Positive indirect effect |
| Protein | PDI_U | CES1 | DR | 0.07413 | 0.04567 | 0.003386 | 0.001143 | 0.001145 | 0.005626 | 2.21 | 0.00305 | 0.0277 | Positive indirect effect |
| Protein | PDI_U | CES1 | RVO | -0.03919 | 0.02063 | -0.000809 | 0.000564 | -0.001915 | 0.000297 | 0.59 | 0.152 | 0.266 | Negative indirect effect |
| Protein | PDI_U | MMP12 | AMD | 0.04375 | 0.0645 | 0.002822 | 0.00094 | 0.000979 | 0.004665 | 2.75 | 0.00269 | 0.0246 | Positive indirect effect |
| Protein | PDI_U | MMP12 | Cataract | -0.07352 | 0.04627 | -0.003402 | 0.001149 | -0.005654 | -0.001149 | 2.84 | 0.00308 | 0.0279 | Negative indirect effect |
| Protein | PDI_U | MMP12 | Glaucoma | -0.02643 | 0.02704 | -0.000715 | 0.000495 | -0.001684 | 0.000255 | 0.65 | 0.148 | 0.26 | Negative indirect effect |
| Protein | PDI_U | MMP12 | DR | 0.0654 | 0.07017 | 0.004589 | 0.001202 | 0.002232 | 0.006946 | 3.22 | 0.000135 | 0.00382 | Positive indirect effect |
| Protein | PDI_U | MMP12 | RVO | -0.03414 | 0.0177 | -0.000604 | 0.000576 | -0.001733 | 0.000525 | 0.56 | 0.294 | 0.51 | Negative indirect effect |
| Protein | PDI_U | CTSZ | AMD | -0.04734 | 0.02528 | -0.001197 | 0.000718 | -0.002604 | 0.000211 | 1.03 | 0.0956 | 0.17 | Negative indirect effect |
| Protein | PDI_U | CTSZ | Cataract | -0.04521 | -0.02245 | 0.001015 | 0.001086 | -0.001113 | 0.003144 | 0.87 | 0.35 | 0.605 | Positive indirect effect |
| Protein | PDI_U | CTSZ | Glaucoma | -0.02841 | -0.03389 | 0.000963 | 0.000591 | -0.000196 | 0.002122 | 0.69 | 0.103 | 0.183 | Positive indirect effect |
| Protein | PDI_U | CTSZ | DR | -0.10141 | -0.05647 | 0.005727 | 0.001561 | 0.002667 | 0.008786 | 4.15 | 0.000244 | 0.00627 | Positive indirect effect |
| Protein | PDI_U | CTSZ | RVO | -0.02683 | 0.02948 | -0.000791 | 0.000612 | -0.00199 | 0.000408 | 0.6 | 0.196 | 0.342 | Negative indirect effect |
| Protein | PDI_U | RBP2 | AMD | 0.04721 | -0.03467 | -0.001637 | 0.000653 | -0.002916 | -0.000358 | 1.12 | 0.0121 | 0.0454 | Negative indirect effect |
| Protein | PDI_U | RBP2 | Cataract | -0.0706 | 0.0454 | -0.003205 | 0.001139 | -0.005439 | -0.000972 | 3.1 | 0.00491 | 0.0434 | Negative indirect effect |
| Protein | PDI_U | RBP2 | Glaucoma | 0.05465 | -0.0439 | -0.0024 | 0.000857 | -0.004079 | -0.00072 | 2.22 | 0.00511 | 0.0451 | Negative indirect effect |
| Protein | PDI_U | RBP2 | DR | -0.04615 | 0.02995 | -0.001383 | 0.000797 | -0.002944 | 0.000179 | 1.39 | 0.0827 | 0.148 | Negative indirect effect |
| Protein | PDI_U | RBP2 | RVO | 0.03355 | 0.0348 | 0.001167 | 0.000525 | 0.000139 | 0.002196 | 0.77 | 0.0261 | 0.0854 | Positive indirect effect |
| Protein | PDI_U | SPINK4 | AMD | 0.04191 | 0.0478 | 0.002004 | 0.00092 | 0.0002 | 0.003807 | 1.5 | 0.0294 | 0.0948 | Positive indirect effect |
| Protein | PDI_U | SPINK4 | Cataract | -0.03596 | 0.02792 | -0.001004 | 0.001045 | -0.003051 | 0.001044 | 0.81 | 0.337 | 0.583 | Negative indirect effect |
| Protein | PDI_U | SPINK4 | Glaucoma | -0.04399 | -0.05631 | 0.002477 | 0.000809 | 0.000892 | 0.004062 | 2.18 | 0.00219 | 0.0204 | Positive indirect effect |
| Protein | PDI_U | SPINK4 | DR | -0.09894 | -0.05278 | 0.005222 | 0.001437 | 0.002406 | 0.008038 | 4.52 | 0.000278 | 0.00703 | Positive indirect effect |
| Protein | PDI_U | SPINK4 | RVO | -0.02671 | 0.0211 | -0.000564 | 0.00057 | -0.001682 | 0.000554 | 0.52 | 0.323 | 0.56 | Negative indirect effect |
| Protein | PDI_U | PLAU | AMD | -0.03272 | 0.02539 | -0.000831 | 0.00071 | -0.002222 | 0.00056 | 0.81 | 0.242 | 0.42 | Negative indirect effect |
| Protein | PDI_U | PLAU | Cataract | -0.04447 | 0.03203 | -0.001424 | 0.001097 | -0.003575 | 0.000726 | 1.03 | 0.194 | 0.339 | Negative indirect effect |
| Protein | PDI_U | PLAU | Glaucoma | -0.03296 | 0.02828 | -0.000932 | 0.000996 | -0.002884 | 0.00102 | 0.88 | 0.349 | 0.605 | Negative indirect effect |
| Protein | PDI_U | PLAU | DR | 0.05514 | -0.08074 | -0.004452 | 0.001239 | -0.006881 | -0.002023 | 3.46 | 0.000328 | 0.00815 | Negative indirect effect |
| Protein | PDI_U | PLAU | RVO | -0.03492 | -0.02098 | 0.000733 | 0.000694 | -0.000628 | 0.002093 | 0.49 | 0.291 | 0.505 | Positive indirect effect |
| Protein | PDI_U | EPHA1 | AMD | 0.0261 | 0.03788 | 0.000989 | 0.000922 | -0.000819 | 0.002796 | 0.65 | 0.284 | 0.492 | Positive indirect effect |
| Protein | PDI_U | EPHA1 | Cataract | 0.03485 | 0.03529 | 0.00123 | 0.000998 | -0.000727 | 0.003186 | 1.08 | 0.218 | 0.379 | Positive indirect effect |
| Protein | PDI_U | EPHA1 | Glaucoma | -0.04925 | 0.03308 | -0.001629 | 0.000689 | -0.00298 | -0.000278 | 1.61 | 0.0181 | 0.0624 | Negative indirect effect |
| Protein | PDI_U | EPHA1 | DR | 0.07285 | -0.05301 | -0.003862 | 0.001402 | -0.00661 | -0.001114 | 3.53 | 0.00588 | 0.0516 | Negative indirect effect |
| Protein | PDI_U | EPHA1 | RVO | 0.03365 | -0.02061 | -0.000694 | 0.000427 | -0.001531 | 0.000144 | 0.61 | 0.105 | 0.185 | Negative indirect effect |
| Protein | PDI_U | SUSD2 | AMD | -0.03653 | -0.02499 | 0.000913 | 0.00068 | -0.00042 | 0.002246 | 0.72 | 0.18 | 0.314 | Positive indirect effect |
| Protein | PDI_U | SUSD2 | Cataract | 0.06638 | 0.07269 | 0.004825 | 0.001412 | 0.002058 | 0.007592 | 3.19 | 0.000631 | 0.015 | Positive indirect effect |
| Protein | PDI_U | SUSD2 | Glaucoma | 0.03177 | -0.02109 | -0.00067 | 0.000603 | -0.001852 | 0.000512 | 0.63 | 0.267 | 0.463 | Negative indirect effect |
| Protein | PDI_U | SUSD2 | DR | -0.03677 | -0.03818 | 0.001404 | 0.001242 | -0.001031 | 0.003838 | 1.12 | 0.258 | 0.449 | Positive indirect effect |
| Protein | PDI_U | SUSD2 | RVO | -0.02251 | 0.02208 | -0.000497 | 0.000382 | -0.001245 | 0.000251 | 0.34 | 0.193 | 0.336 | Negative indirect effect |
| Protein | PDI_U | B3GLAP | AMD | 0.02813 | 0.02867 | 0.000806 | 0.000623 | -0.000415 | 0.002028 | 0.75 | 0.196 | 0.341 | Positive indirect effect |
| Protein | PDI_U | B3GLAP | Cataract | 0.05861 | -0.03784 | -0.002218 | 0.000973 | -0.004126 | -0.00031 | 1.73 | 0.0227 | 0.0755 | Negative indirect effect |
| Protein | PDI_U | B3GLAP | Glaucoma | 0.06152 | -0.03104 | -0.00191 | 0.000699 | -0.00328 | -0.00054 | 1.77 | 0.0063 | 0.0551 | Negative indirect effect |
| Protein | PDI_U | B3GLAP | DR | -0.04461 | 0.03248 | -0.001449 | 0.001433 | -0.004258 | 0.00136 | 1.02 | 0.312 | 0.541 | Negative indirect effect |
| Protein | PDI_U | B3GLAP | RVO | -0.02322 | 0.03017 | -0.000701 | 0.00052 | -0.001719 | 0.000318 | 0.68 | 0.178 | 0.31 | Negative indirect effect |
| Protein | PDI_U | MET | AMD | -0.03997 | -0.02918 | 0.001166 | 0.000923 | -0.000642 | 0.002975 | 0.79 | 0.206 | 0.359 | Positive indirect effect |
| Protein | PDI_U | MET | Cataract | 0.05083 | 0.04366 | 0.00222 | 0.001009 | 0.000242 | 0.004198 | 1.88 | 0.0279 | 0.0903 | Positive indirect effect |
| Protein | PDI_U | MET | Glaucoma | 0.04501 | 0.05189 | 0.002336 | 0.000856 | 0.000657 | 0.004014 | 1.95 | 0.00639 | 0.0559 | Positive indirect effect |

| Feature_type | Exposure | Mediator | Outcome | a_path_beta | b_path_beta | Indirect_effect | SE | CI_lower | CI_upper | Proportion_mediated_percent | P | FDR_q | Effect_direction |
|---|---|---|---|---|---|---|---|---|---|---|---|---|---|
| Protein | PDI_U | MET | DR | -0.05534 | 0.09083 | -0.005027 | 0.001334 | -0.007642 | -0.002411 | 3.68 | 0.000165 | 0.00449 | Negative indirect effect |
| Protein | PDI_U | MET | RVO | -0.03009 | -0.01628 | 0.00049 | 0.000583 | -0.000652 | 0.001632 | 0.4 | 0.401 | 0.692 | Positive indirect effect |
| Metabolite | PDI_U | Ratio.of.polyunsaturated.FA.to.total.FA | AMD | 0.0926 | -0.06254 | -0.005792 | 0.001963 | -0.00964 | -0.001943 | 4.4 | 0.00318 | 0.0287 | Negative indirect effect |
| Metabolite | PDI_U | Ratio.of.polyunsaturated.FA.to.total.FA | Cataract | 0.05946 | 0.03181 | 0.001891 | 0.001727 | -0.001494 | 0.005276 | 1.05 | 0.273 | 0.475 | Positive indirect effect |
| Metabolite | PDI_U | Ratio.of.polyunsaturated.FA.to.total.FA | Glaucoma | 0.07753 | -0.04808 | -0.003728 | 0.001226 | -0.006131 | -0.001324 | 2.02 | 0.00237 | 0.0219 | Negative indirect effect |
| Metabolite | PDI_U | Ratio.of.polyunsaturated.FA.to.total.FA | DR | -0.08466 | -0.10499 | 0.008888 | 0.002481 | 0.004025 | 0.013751 | 5.92 | 0.000341 | 0.00844 | Positive indirect effect |
| Metabolite | PDI_U | Ratio.of.polyunsaturated.FA.to.total.FA | RVO | -0.07053 | 0.04654 | -0.003282 | 0.000912 | -0.005069 | -0.001496 | 1.86 | 0.000317 | 0.00791 | Negative indirect effect |
| Metabolite | PDI_U | Ratio.of.saturated.FA.to.total.FA | AMD | -0.09733 | 0.0673 | -0.00655 | 0.001839 | -0.010155 | -0.002945 | 3.62 | 0.000369 | 0.00908 | Negative indirect effect |
| Metabolite | PDI_U | Ratio.of.saturated.FA.to.total.FA | Cataract | -0.0739 | -0.04637 | 0.003427 | 0.001591 | 0.000308 | 0.006546 | 1.81 | 0.0313 | 0.1 | Positive indirect effect |
| Metabolite | PDI_U | Ratio.of.saturated.FA.to.total.FA | Glaucoma | -0.03228 | -0.04031 | 0.001301 | 0.001065 | -0.000786 | 0.003388 | 0.81 | 0.222 | 0.386 | Positive indirect effect |
| Metabolite | PDI_U | Ratio.of.saturated.FA.to.total.FA | DR | -0.04891 | -0.0619 | 0.003028 | 0.003094 | -0.003037 | 0.009093 | 2.54 | 0.328 | 0.568 | Positive indirect effect |
| Metabolite | PDI_U | Ratio.of.saturated.FA.to.total.FA | RVO | 0.0772 | 0.04958 | 0.003828 | 0.001311 | 0.001257 | 0.006398 | 2.37 | 0.00351 | 0.0316 | Positive indirect effect |
| Metabolite | PDI_U | TG.in.L.VLDL | AMD | -0.08103 | 0.0389 | -0.003152 | 0.001444 | -0.005981 | -0.000322 | 2 | 0.029 | 0.0936 | Negative indirect effect |
| Metabolite | PDI_U | TG.in.L.VLDL | Cataract | 0.09755 | 0.06156 | 0.006005 | 0.002186 | 0.001721 | 0.010289 | 3.32 | 0.00601 | 0.0527 | Positive indirect effect |
| Metabolite | PDI_U | TG.in.L.VLDL | Glaucoma | -0.03492 | 0.03803 | -0.001328 | 0.001135 | -0.003553 | 0.000896 | 0.84 | 0.242 | 0.42 | Negative indirect effect |
| Metabolite | PDI_U | TG.in.L.VLDL | DR | -0.07474 | 0.0507 | -0.00379 | 0.001627 | -0.006978 | -0.000601 | 2.98 | 0.0198 | 0.0674 | Negative indirect effect |
| Metabolite | PDI_U | TG.in.L.VLDL | RVO | -0.04143 | 0.04508 | -0.001867 | 0.0008 | -0.003436 | -0.000299 | 1.21 | 0.0196 | 0.0668 | Negative indirect effect |
| Metabolite | PDI_U | TG.in.VLDL | AMD | 0.04893 | 0.05229 | 0.002559 | 0.001036 | 0.000528 | 0.004589 | 1.46 | 0.0135 | 0.0494 | Positive indirect effect |
| Metabolite | PDI_U | TG.in.VLDL | Cataract | 0.04762 | 0.03272 | 0.001558 | 0.001865 | -0.002097 | 0.005213 | 0.86 | 0.403 | 0.697 | Positive indirect effect |
| Metabolite | PDI_U | TG.in.VLDL | Glaucoma | -0.05916 | 0.03404 | -0.002014 | 0.000814 | -0.003609 | -0.000419 | 1.32 | 0.0134 | 0.0489 | Negative indirect effect |
| Metabolite | PDI_U | TG.in.VLDL | DR | 0.09584 | 0.10723 | 0.010276 | 0.003058 | 0.004282 | 0.016271 | 5.75 | 0.000779 | 0.0183 | Positive indirect effect |
| Metabolite | PDI_U | TG.in.VLDL | RVO | -0.02838 | 0.03575 | -0.001014 | 0.000784 | -0.002552 | 0.000523 | 0.55 | 0.196 | 0.342 | Negative indirect effect |
| Metabolite | PDI_U | TG | AMD | 0.10184 | 0.06171 | 0.006285 | 0.001869 | 0.002622 | 0.009948 | 3.59 | 0.000771 | 0.0181 | Positive indirect effect |
| Metabolite | PDI_U | TG | Cataract | 0.04418 | 0.03867 | 0.001708 | 0.001467 | -0.001167 | 0.004583 | 1.22 | 0.244 | 0.424 | Positive indirect effect |
| Metabolite | PDI_U | TG | Glaucoma | 0.0652 | -0.0399 | -0.002601 | 0.001227 | -0.005007 | -0.000195 | 1.68 | 0.0341 | 0.108 | Negative indirect effect |
| Metabolite | PDI_U | TG | DR | 0.09976 | 0.06779 | 0.006762 | 0.001947 | 0.002946 | 0.010579 | 5.63 | 0.000516 | 0.0124 | Positive indirect effect |
| Metabolite | PDI_U | TG | RVO | -0.04099 | 0.02622 | -0.001075 | 0.000771 | -0.002585 | 0.000436 | 0.57 | 0.163 | 0.285 | Negative indirect effect |
| Metabolite | PDI_U | TG.in.V.L.VLDL | AMD | -0.04041 | 0.04927 | -0.001991 | 0.001087 | -0.004121 | 0.000139 | 1.23 | 0.0669 | 0.12 | Negative indirect effect |
| Metabolite | PDI_U | TG.in.V.L.VLDL | Cataract | 0.03865 | 0.04717 | 0.001823 | 0.001535 | -0.001185 | 0.004831 | 1.29 | 0.235 | 0.408 | Positive indirect effect |
| Metabolite | PDI_U | TG.in.V.L.VLDL | Glaucoma | -0.04239 | -0.02468 | 0.001046 | 0.001232 | -0.001369 | 0.003461 | 0.8 | 0.396 | 0.684 | Positive indirect effect |
| Metabolite | PDI_U | TG.in.V.L.VLDL | DR | -0.0765 | -0.09566 | 0.007318 | 0.001726 | 0.003936 | 0.0107 | 4.27 | 2.23e-05 | 0.00128 | Positive indirect effect |
| Metabolite | PDI_U | TG.in.V.L.VLDL | RVO | -0.04218 | 0.02902 | -0.001224 | 0.000844 | -0.002878 | 0.00043 | 0.86 | 0.147 | 0.258 | Negative indirect effect |
| Metabolite | PDI_U | Ratio.of.omega.6.FA.to.total.FA | AMD | 0.04662 | -0.03707 | -0.001728 | 0.001183 | -0.004048 | 0.000591 | 1.07 | 0.144 | 0.253 | Negative indirect effect |
| Metabolite | PDI_U | Ratio.of.omega.6.FA.to.total.FA | Cataract | -0.0336 | 0.04766 | -0.001601 | 0.00177 | -0.00507 | 0.001867 | 0.86 | 0.366 | 0.632 | Negative indirect effect |
| Metabolite | PDI_U | Ratio.of.omega.6.FA.to.total.FA | Glaucoma | -0.04287 | -0.0239 | 0.001024 | 0.001065 | -0.001062 | 0.003111 | 0.69 | 0.336 | 0.582 | Positive indirect effect |
| Metabolite | PDI_U | Ratio.of.omega.6.FA.to.total.FA | DR | 0.09723 | 0.09132 | 0.008878 | 0.00262 | 0.003744 | 0.014013 | 5.65 | 0.000701 | 0.0166 | Positive indirect effect |
| Metabolite | PDI_U | Ratio.of.omega.6.FA.to.total.FA | RVO | 0.041 | -0.02133 | -0.000875 | 0.00062 | -0.002091 | 0.000342 | 0.49 | 0.159 | 0.278 | Negative indirect effect |
| Metabolite | PDI_U | Saturated.FA | AMD | 0.06319 | 0.03249 | 0.002053 | 0.001626 | -0.001134 | 0.00524 | 1.31 | 0.207 | 0.36 | Positive indirect effect |
| Metabolite | PDI_U | Saturated.FA | Cataract | -0.06175 | 0.10217 | -0.006308 | 0.001564 | -0.009373 | -0.003244 | 4.87 | 5.47e-05 | 0.00201 | Negative indirect effect |
| Metabolite | PDI_U | Saturated.FA | Glaucoma | 0.03725 | 0.03157 | 0.001176 | 0.000892 | -0.000573 | 0.002925 | 0.89 | 0.188 | 0.327 | Positive indirect effect |
| Metabolite | PDI_U | Saturated.FA | DR | 0.1057 | 0.06038 | 0.006382 | 0.001995 | 0.002471 | 0.010293 | 5.35 | 0.00138 | 0.0135 | Positive indirect effect |
| Metabolite | PDI_U | Saturated.FA | RVO | -0.04715 | 0.0257 | -0.001212 | 0.001259 | -0.00368 | 0.001257 | 1.02 | 0.336 | 0.582 | Negative indirect effect |
| Metabolite | PDI_U | TG.in.M.VLDL | AMD | -0.08423 | 0.04146 | -0.003492 | 0.001559 | -0.006547 | -0.000437 | 2.9 | 0.0251 | 0.0824 | Negative indirect effect |
| Metabolite | PDI_U | TG.in.M.VLDL | Cataract | 0.0735 | 0.06622 | 0.004867 | 0.001399 | 0.002125 | 0.007609 | 3.44 | 0.000503 | 0.0121 | Positive indirect effect |
| Metabolite | PDI_U | TG.in.M.VLDL | Glaucoma | -0.07315 | 0.07142 | -0.005224 | 0.001511 | -0.008186 | -0.002263 | 3.55 | 0.000545 | 0.013 | Negative indirect effect |
| Metabolite | PDI_U | TG.in.M.VLDL | DR | -0.0906 | 0.07536 | -0.006828 | 0.002491 | -0.011711 | -0.001945 | 4.99 | 0.00613 | 0.0537 | Negative indirect effect |
| Metabolite | PDI_U | TG.in.M.VLDL | RVO | -0.04241 | -0.02054 | 0.000871 | 0.00088 | -0.000854 | 0.002597 | 0.6 | 0.322 | 0.558 | Positive indirect effect |
| Metabolite | PDI_U | TLs.in.V.L.VLDL | AMD | 0.07511 | 0.0661 | 0.004964 | 0.001384 | 0.002251 | 0.007677 | 3.29 | 0.000335 | 0.00832 | Positive indirect effect |
| Metabolite | PDI_U | TLs.in.V.L.VLDL | Cataract | -0.08594 | -0.08753 | 0.007522 | 0.002214 | 0.003183 | 0.011862 | 4.7 | 0.00068 | 0.0161 | Positive indirect effect |
| Metabolite | PDI_U | TLs.in.V.L.VLDL | Glaucoma | 0.03701 | 0.02692 | 0.000996 | 0.000689 | -0.000354 | 0.002347 | 0.7 | 0.148 | 0.26 | Positive indirect effect |
| Metabolite | PDI_U | TLs.in.V.L.VLDL | DR | 0.10385 | 0.08443 | 0.008768 | 0.002587 | 0.003697 | 0.01384 | 6.54 | 0.000702 | 0.0166 | Positive indirect effect |
| Metabolite | PDI_U | TLs.in.V.L.VLDL | RVO | 0.05302 | 0.039 | 0.002068 | 0.000892 | 0.000319 | 0.003817 | 1.49 | 0.0205 | 0.0692 | Positive indirect effect |
| Metabolite | PDI_U | TG.in.HDL | AMD | -0.04427 | -0.06967 | 0.003084 | 0.001255 | 0.000624 | 0.005544 | 1.91 | 0.014 | 0.0507 | Positive indirect effect |
| Metabolite | PDI_U | TG.in.HDL | Cataract | 0.05119 | 0.02924 | 0.001497 | 0.001034 | -0.00053 | 0.003524 | 1.1 | 0.148 | 0.259 | Positive indirect effect |
| Metabolite | PDI_U | TG.in.HDL | Glaucoma | 0.06645 | 0.0323 | 0.002146 | 0.000886 | 0.000409 | 0.003884 | 1.4 | 0.0155 | 0.0549 | Positive indirect effect |
| Metabolite | PDI_U | TG.in.HDL | DR | 0.09901 | 0.08271 | 0.008189 | 0.002416 | 0.003453 | 0.012925 | 4.45 | 0.000702 | 0.0166 | Positive indirect effect |
| Metabolite | PDI_U | TG.in.HDL | RVO | 0.03564 | -0.02457 | -0.000876 | 0.000725 | -0.002297 | 0.000545 | 0.6 | 0.227 | 0.395 | Negative indirect effect |
| Metabolite | PDI_U | TLs.in.L.VLDL | AMD | 0.03624 | 0.03724 | 0.00135 | 0.001003 | -0.000616 | 0.003315 | 1.1 | 0.178 | 0.311 | Positive indirect effect |
| Metabolite | PDI_U | TLs.in.L.VLDL | Cataract | -0.02966 | -0.04523 | 0.001341 | 0.001091 | -0.000796 | 0.003479 | 1.07 | 0.219 | 0.381 | Positive indirect effect |
| Metabolite | PDI_U | TLs.in.L.VLDL | Glaucoma | 0.03413 | 0.0272 | 0.000928 | 0.000593 | -0.000234 | 0.00209 | 0.53 | 0.117 | 0.207 | Positive indirect effect |
| Metabolite | PDI_U | TLs.in.L.VLDL | DR | -0.06973 | 0.08527 | -0.005946 | 0.002173 | -0.010204 | -0.001688 | 4.29 | 0.0062 | 0.0543 | Negative indirect effect |
| Metabolite | PDI_U | TLs.in.L.VLDL | RVO | -0.09039 | -0.04798 | 0.004337 | 0.001179 | 0.002026 | 0.006648 | 3.39 | 0.000235 | 0.00606 | Positive indirect effect |
| Metabolite | PDI_U | Con.of.V.L.VLDL.particles | AMD | -0.05422 | -0.08414 | 0.004562 | 0.001607 | 0.001413 | 0.007712 | 2.38 | 0.00453 | 0.0401 | Positive indirect effect |
| Metabolite | PDI_U | Con.of.V.L.VLDL.particles | Cataract | -0.05525 | 0.06158 | -0.003402 | 0.001665 | -0.006665 | -0.000138 | 2.82 | 0.041 | 0.128 | Negative indirect effect |
| Metabolite | PDI_U | Con.of.V.L.VLDL.particles | Glaucoma | 0.04698 | 0.02946 | 0.001384 | 0.000812 | -0.000208 | 0.002976 | 0.73 | 0.0885 | 0.157 | Positive indirect effect |
| Metabolite | PDI_U | Con.of.V.L.VLDL.particles | DR | 0.08779 | 0.05501 | 0.004829 | 0.001773 | 0.001354 | 0.008305 | 3.54 | 0.00646 | 0.0565 | Positive indirect effect |
| Metabolite | PDI_U | Con.of.V.L.VLDL.particles | RVO | 0.03358 | 0.02429 | 0.000816 | 0.000797 | -0.000746 | 0.002377 | 0.63 | 0.306 | 0.53 | Positive indirect effect |
| Metabolite | PDI_U | Con.of.L.VLDL.particles | AMD | -0.0483 | 0.06493 | -0.003136 | 0.001556 | -0.006186 | -8.5e-05 | 2.26 | 0.0439 | 0.136 | Negative indirect effect |
| Metabolite | PDI_U | Con.of.L.VLDL.particles | Cataract | -0.0392 | 0.04918 | -0.001928 | 0.001234 | -0.004345 | 0.00049 | 1.4 | 0.118 | 0.208 | Negative indirect effect |
| Metabolite | PDI_U | Con.of.L.VLDL.particles | Glaucoma | 0.03301 | 0.05678 | 0.001874 | 0.000776 | 0.000354 | 0.003394 | 1.29 | 0.0157 | 0.0554 | Positive indirect effect |
| Metabolite | PDI_U | Con.of.L.VLDL.particles | DR | -0.0742 | -0.04924 | 0.003653 | 0.001644 | 0.000431 | 0.006875 | 3 | 0.0263 | 0.0858 | Positive indirect effect |
| Metabolite | PDI_U | Con.of.L.VLDL.particles | RVO | 0.04498 | -0.02495 | -0.001122 | 0.001198 | -0.003471 | 0.001226 | 0.84 | 0.349 | 0.604 | Negative indirect effect |
| Metabolite | PDI_U | TG.in.M.HDL | AMD | 0.09002 | 0.05304 | 0.004775 | 0.00115 | 0.00252 | 0.007029 | 3.55 | 3.3e-05 | 0.00152 | Positive indirect effect |
| Metabolite | PDI_U | TG.in.M.HDL | Cataract | 0.0455 | 0.03958 | 0.001801 | 0.001117 | -0.000388 | 0.00399 | 1.49 | 0.107 | 0.189 | Positive indirect effect |
| Metabolite | PDI_U | TG.in.M.HDL | Glaucoma | -0.03117 | 0.04027 | -0.001255 | 0.00089 | -0.003 | 0.00049 | 0.95 | 0.159 | 0.277 | Negative indirect effect |
| Metabolite | PDI_U | TG.in.M.HDL | DR | 0.0552 | -0.09726 | -0.005369 | 0.001553 | -0.008412 | -0.002325 | 3.15 | 0.000545 | 0.013 | Negative indirect effect |
| Metabolite | PDI_U | TG.in.M.HDL | RVO | -0.04505 | 0.02284 | -0.001029 | 0.001013 | -0.003014 | 0.000956 | 0.84 | 0.31 | 0.536 | Negative indirect effect |
| Metabolite | PDI_U | TG.in.S.VLDL | AMD | -0.03268 | -0.04171 | 0.001363 | 0.001111 | -0.000814 | 0.00354 | 0.73 | 0.22 | 0.382 | Positive indirect effect |
| Metabolite | PDI_U | TG.in.S.VLDL | Cataract | 0.03909 | -0.05214 | -0.002038 | 0.001202 | -0.004393 | 0.000317 | 1.15 | 0.0898 | 0.16 | Negative indirect effect |
| Metabolite | PDI_U | TG.in.S.VLDL | Glaucoma | 0.0254 | -0.04148 | -0.001054 | 0.000853 | -0.002726 | 0.000619 | 0.7 | 0.217 | 0.378 | Negative indirect effect |
| Metabolite | PDI_U | TG.in.S.VLDL | DR | -0.10922 | -0.05204 | 0.005684 | 0.001483 | 0.002777 | 0.008591 | 4.13 | 0.000127 | 0.00363 | Positive indirect effect |
| Metabolite | PDI_U | TG.in.S.VLDL | RVO | -0.0302 | 0.03676 | -0.00111 | 0.001092 | -0.003251 | 0.001031 | 0.6 | 0.31 | 0.536 | Negative indirect effect |
| Metabolite | PDI_U | TLs.in.VLDL | AMD | -0.07094 | -0.03504 | 0.002485 | 0.001089 | 0.00035 | 0.004621 | 1.48 | 0.0225 | 0.075 | Positive indirect effect |
| Metabolite | PDI_U | TLs.in.VLDL | Cataract | -0.05161 | -0.03277 | 0.001691 | 0.001175 | -0.000612 | 0.003994 | 1.15 | 0.15 | 0.263 | Positive indirect effect |
| Metabolite | PDI_U | TLs.in.VLDL | Glaucoma | 0.04547 | 0.02545 | 0.001157 | 0.001215 | -0.001223 | 0.003538 | 0.93 | 0.341 | 0.59 | Positive indirect effect |
| Metabolite | PDI_U | TLs.in.VLDL | DR | -0.03324 | 0.05731 | -0.001905 | 0.00186 | -0.00555 | 0.00174 | 1 | 0.306 | 0.53 | Negative indirect effect |
| Metabolite | PDI_U | TLs.in.VLDL | RVO | -0.03086 | 0.04409 | -0.001361 | 0.000582 | -0.002502 | -0.00022 | 1.04 | 0.0194 | 0.0662 | Negative indirect effect |
| Metabolite | PDI_U | TG.in.S.HDL | AMD | -0.04114 | -0.03114 | 0.001281 | 0.000865 | -0.000415 | 0.002977 | 0.94 | 0.139 | 0.243 | Positive indirect effect |
| Metabolite | PDI_U | TG.in.S.HDL | Cataract | -0.03682 | -0.03748 | 0.00138 | 0.000911 | -0.000406 | 0.003166 | 1.12 | 0.13 | 0.228 | Positive indirect effect |
| Metabolite | PDI_U | TG.in.S.HDL | Glaucoma | -0.02811 | 0.03603 | -0.001013 | 0.001091 | -0.003151 | 0.001125 | 0.78 | 0.353 | 0.611 | Negative indirect effect |
| Metabolite | PDI_U | TG.in.S.HDL | DR | -0.06197 | 0.03485 | -0.002159 | 0.002029 | -0.006137 | 0.001818 | 1.71 | 0.287 | 0.498 | Negative indirect effect |
| Metabolite | PDI_U | TG.in.S.HDL | RVO | -0.0483 | 0.03201 | -0.001546 | 0.000771 | -0.003057 | -3.5e-05 | 0.95 | 0.0449 | 0.139 | Negative indirect effect |
| Metabolite | PDI_U | PL.in.V.L.VLDL | AMD | 0.0411 | 0.0322 | 0.001323 | 0.001542 | -0.0017 | 0.004346 | 0.88 | 0.391 | 0.676 | Positive indirect effect |
| Metabolite | PDI_U | PL.in.V.L.VLDL | Cataract | 0.03898 | 0.03772 | 0.00147 | 0.001377 | -0.001228 | 0.004169 | 0.9 | 0.286 | 0.495 | Positive indirect effect |
| Metabolite | PDI_U | PL.in.V.L.VLDL | Glaucoma | 0.05149 | -0.03072 | -0.001582 | 0.000761 | -0.003074 | -9e-05 | 1.13 | 0.0376 | 0.118 | Negative indirect effect |
| Metabolite | PDI_U | PL.in.V.L.VLDL | DR | 0.07803 | 0.0965 | 0.007529 | 0.001943 | 0.003721 | 0.011338 | 5.32 | 0.000107 | 0.00318 | Positive indirect effect |
| Metabolite | PDI_U | PL.in.V.L.VLDL | RVO | 0.03866 | -0.04592 | -0.001776 | 0.000861 | -0.003463 | -8.8e-05 | 0.96 | 0.0391 | 0.123 | Negative indirect effect |
| Metabolite | PDI_U | PL.in.L.VLDL | AMD | -0.09384 | -0.04861 | 0.004561 | 0.001118 | 0.002369 | 0.006754 | 3.79 | 4.53e-05 | 0.0018 | Positive indirect effect |
| Metabolite | PDI_U | PL.in.L.VLDL | Cataract | -0.05637 | 0.03001 | -0.001692 | 0.001299 | -0.004237 | 0.000853 | 0.88 | 0.193 | 0.336 | Negative indirect effect |
| Metabolite | PDI_U | PL.in.L.VLDL | Glaucoma | 0.03242 | 0.02754 | 0.000893 | 0.000634 | -0.00035 | 0.002136 | 0.63 | 0.159 | 0.279 | Positive indirect effect |

| Feature_type | Exposure | Mediator | Outcome | a_path_beta | b_path_beta | Indirect_effect | SE | CI_lower | CI_upper | Proportion_mediated_percent | P | FDR_q | Effect_direction |
|---|---|---|---|---|---|---|---|---|---|---|---|---|---|
| Metabolite | PDI_U | PL.in.L.VLDL | DR | -0.09479 | 0.06952 | -0.006589 | 0.001894 | -0.010301 | -0.002877 | 3.48 | 0.000503 | 0.0121 | Negative indirect effect |
| Metabolite | PDI_U | PL.in.L.VLDL | RVO | -0.0466 | 0.02319 | -0.001081 | 0.001109 | -0.003255 | 0.001093 | 0.84 | 0.33 | 0.571 | Negative indirect effect |
| Metabolite | PDI_H | Ratio.of.omega.3.FA.to.total.FA | AMD | -0.06317 | -0.07304 | 0.004614 | 0.001694 | 0.001294 | 0.007935 | 3.18 | 0.00645 | 0.0565 | Positive indirect effect |
| Metabolite | PDI_H | Ratio.of.omega.3.FA.to.total.FA | Cataract | -0.08565 | 0.09417 | -0.008066 | 0.002074 | -0.012131 | -0.004001 | 5.32 | 0.000101 | 0.00304 | Negative indirect effect |
| Metabolite | PDI_H | Ratio.of.omega.3.FA.to.total.FA | Glaucoma | -0.03212 | 0.04708 | -0.001512 | 0.001378 | -0.004212 | 0.001188 | 0.86 | 0.272 | 0.473 | Negative indirect effect |
| Metabolite | PDI_H | Ratio.of.omega.3.FA.to.total.FA | DR | 0.10801 | -0.06554 | -0.007079 | 0.002258 | -0.011505 | -0.002653 | 4.18 | 0.00172 | 0.0164 | Negative indirect effect |
| Metabolite | PDI_H | Ratio.of.omega.3.FA.to.total.FA | RVO | 0.0527 | 0.026 | 0.00137 | 0.001555 | -0.001678 | 0.004418 | 0.9 | 0.378 | 0.654 | Positive indirect effect |
| Metabolite | PDI_H | Docosahexaenoic.acid | AMD | -0.08283 | 0.04002 | -0.003315 | 0.001643 | -0.006536 | -9.4e-05 | 2.2 | 0.0437 | 0.136 | Negative indirect effect |
| Metabolite | PDI_H | Docosahexaenoic.acid | Cataract | 0.05572 | 0.09345 | 0.005208 | 0.001941 | 0.001404 | 0.009011 | 4.07 | 0.00728 | 0.0635 | Positive indirect effect |
| Metabolite | PDI_H | Docosahexaenoic.acid | Glaucoma | -0.08787 | 0.04779 | -0.004199 | 0.001419 | -0.006979 | -0.001418 | 3.15 | 0.00308 | 0.0279 | Negative indirect effect |
| Metabolite | PDI_H | Docosahexaenoic.acid | DR | -0.08135 | 0.13088 | -0.010647 | 0.003011 | -0.016549 | -0.004746 | 7.17 | 0.000406 | 0.0099 | Negative indirect effect |
| Metabolite | PDI_H | Docosahexaenoic.acid | RVO | -0.06083 | 0.06184 | -0.003762 | 0.000981 | -0.005685 | -0.001838 | 2.64 | 0.000127 | 0.00363 | Negative indirect effect |
| Metabolite | PDI_H | Omega.3.FAs | AMD | -0.06918 | -0.10824 | 0.007488 | 0.002231 | 0.003116 | 0.01186 | 5.96 | 0.000788 | 0.0185 | Positive indirect effect |
| Metabolite | PDI_H | Omega.3.FAs | Cataract | -0.12436 | 0.06667 | -0.008291 | 0.002414 | -0.013022 | -0.00356 | 5.3 | 0.000592 | 0.0141 | Negative indirect effect |
| Metabolite | PDI_H | Omega.3.FAs | Glaucoma | -0.03101 | -0.05422 | 0.001682 | 0.00097 | -0.000219 | 0.003582 | 1.27 | 0.0828 | 0.148 | Positive indirect effect |
| Metabolite | PDI_H | Omega.3.FAs | DR | 0.06363 | 0.09832 | 0.006256 | 0.002254 | 0.001837 | 0.010674 | 3.3 | 0.00552 | 0.0486 | Positive indirect effect |
| Metabolite | PDI_H | Omega.3.FAs | RVO | 0.02272 | -0.03924 | -0.000891 | 0.001 | -0.002851 | 0.001068 | 0.67 | 0.373 | 0.644 | Negative indirect effect |
| Metabolite | PDI_H | Ratio.of.omega.6.FA.to.omega.3.FA | AMD | 0.09468 | -0.08044 | -0.007617 | 0.00221 | -0.011949 | -0.003285 | 4.34 | 0.000568 | 0.0136 | Negative indirect effect |
| Metabolite | PDI_H | Ratio.of.omega.6.FA.to.omega.3.FA | Cataract | -0.0771 | -0.05044 | 0.003889 | 0.001826 | 0.000309 | 0.007469 | 3.06 | 0.0332 | 0.106 | Positive indirect effect |
| Metabolite | PDI_H | Ratio.of.omega.6.FA.to.omega.3.FA | Glaucoma | 0.05231 | 0.05664 | 0.002963 | 0.001233 | 0.000546 | 0.005379 | 2.09 | 0.0163 | 0.0572 | Positive indirect effect |
| Metabolite | PDI_H | Ratio.of.omega.6.FA.to.omega.3.FA | DR | 0.13335 | 0.07587 | 0.010118 | 0.002991 | 0.004255 | 0.015981 | 8.5 | 0.000719 | 0.0169 | Positive indirect effect |
| Metabolite | PDI_H | Ratio.of.omega.6.FA.to.omega.3.FA | RVO | 0.04639 | 0.026 | 0.001206 | 0.001355 | -0.001451 | 0.003863 | 0.63 | 0.374 | 0.646 | Positive indirect effect |
| Metabolite | PDI_H | Ratio.of.docosahexaenoic.acid.to.total.FA | AMD | 0.08528 | -0.05639 | -0.004809 | 0.001247 | -0.007253 | -0.002365 | 3.61 | 0.000115 | 0.00336 | Negative indirect effect |
| Metabolite | PDI_H | Ratio.of.docosahexaenoic.acid.to.total.FA | Cataract | 0.08688 | 0.06767 | 0.00588 | 0.001592 | 0.002758 | 0.009001 | 4.41 | 0.000222 | 0.00578 | Positive indirect effect |
| Metabolite | PDI_H | Ratio.of.docosahexaenoic.acid.to.total.FA | Glaucoma | -0.03273 | -0.03209 | 0.00105 | 0.00109 | -0.001086 | 0.003187 | 0.55 | 0.335 | 0.581 | Positive indirect effect |
| Metabolite | PDI_H | Ratio.of.docosahexaenoic.acid.to.total.FA | DR | -0.09862 | 0.04863 | -0.004796 | 0.001793 | -0.008311 | -0.001281 | 4.05 | 0.00749 | 0.0652 | Negative indirect effect |
| Metabolite | PDI_H | Ratio.of.docosahexaenoic.acid.to.total.FA | RVO | -0.02904 | -0.03749 | 0.001089 | 0.001064 | -0.000996 | 0.003173 | 0.88 | 0.306 | 0.53 | Positive indirect effect |
| Metabolite | PDI_H | Degree.of.unsaturation | AMD | 0.04892 | 0.03721 | 0.001821 | 0.001231 | -0.000593 | 0.004234 | 1.5 | 0.139 | 0.245 | Positive indirect effect |
| Metabolite | PDI_H | Degree.of.unsaturation | Cataract | 0.06183 | 0.08833 | 0.005462 | 0.001394 | 0.00273 | 0.008194 | 3.49 | 8.93e-05 | 0.00278 | Positive indirect effect |
| Metabolite | PDI_H | Degree.of.unsaturation | Glaucoma | 0.08697 | -0.06255 | -0.00544 | 0.001482 | -0.008344 | -0.002535 | 4.37 | 0.000242 | 0.00622 | Negative indirect effect |
| Metabolite | PDI_H | Degree.of.unsaturation | DR | -0.10001 | 0.07095 | -0.007096 | 0.002056 | -0.011126 | -0.003066 | 4.31 | 0.000559 | 0.0134 | Negative indirect effect |
| Metabolite | PDI_H | Degree.of.unsaturation | RVO | 0.03385 | 0.02936 | 0.000994 | 0.001178 | -0.001315 | 0.003302 | 0.63 | 0.399 | 0.69 | Positive indirect effect |
| Metabolite | PDI_H | Ratio.of.polyunsaturated.FA.to.total.FA | AMD | -0.05376 | -0.03631 | 0.001952 | 0.001749 | -0.001476 | 0.005379 | 1.48 | 0.264 | 0.459 | Positive indirect effect |
| Metabolite | PDI_H | Ratio.of.polyunsaturated.FA.to.total.FA | Cataract | 0.115 | 0.06151 | 0.007074 | 0.00206 | 0.003037 | 0.011112 | 3.92 | 0.000594 | 0.0141 | Positive indirect effect |
| Metabolite | PDI_H | Ratio.of.polyunsaturated.FA.to.total.FA | Glaucoma | -0.04375 | -0.02714 | 0.001187 | 0.001209 | -0.001183 | 0.003557 | 0.64 | 0.326 | 0.565 | Positive indirect effect |
| Metabolite | PDI_H | Ratio.of.polyunsaturated.FA.to.total.FA | DR | -0.08256 | -0.10238 | 0.008452 | 0.002473 | 0.003605 | 0.013299 | 5.63 | 0.000631 | 0.015 | Positive indirect effect |
| Metabolite | PDI_H | Ratio.of.polyunsaturated.FA.to.total.FA | RVO | -0.07294 | 0.04813 | -0.003511 | 0.001273 | -0.006005 | -0.001017 | 1.98 | 0.0058 | 0.0509 | Negative indirect effect |
| Metabolite | PDI_H | Ratio.of.saturated.FA.to.total.FA | AMD | 0.04998 | 0.03456 | 0.001728 | 0.001686 | -0.001576 | 0.005032 | 0.95 | 0.305 | 0.529 | Positive indirect effect |
| Metabolite | PDI_H | Ratio.of.saturated.FA.to.total.FA | Cataract | -0.05194 | -0.03259 | 0.001693 | 0.001602 | -0.001448 | 0.004834 | 0.89 | 0.291 | 0.504 | Positive indirect effect |
| Metabolite | PDI_H | Ratio.of.saturated.FA.to.total.FA | Glaucoma | -0.02826 | -0.03528 | 0.000997 | 0.000729 | -0.000431 | 0.002425 | 0.62 | 0.171 | 0.299 | Positive indirect effect |
| Metabolite | PDI_H | Ratio.of.saturated.FA.to.total.FA | DR | 0.06027 | -0.07628 | -0.004598 | 0.00214 | -0.008792 | -0.000404 | 3.86 | 0.0317 | 0.101 | Negative indirect effect |
| Metabolite | PDI_H | Ratio.of.saturated.FA.to.total.FA | RVO | 0.07192 | 0.04618 | 0.003321 | 0.001214 | 0.000942 | 0.005701 | 2.06 | 0.00623 | 0.0545 | Positive indirect effect |
| Metabolite | PDI_H | Ratio.of.linoleic.acid.to.total.FA | AMD | -0.0527 | 0.02956 | -0.001558 | 0.000905 | -0.003332 | 0.000217 | 0.86 | 0.0854 | 0.152 | Negative indirect effect |
| Metabolite | PDI_H | Ratio.of.linoleic.acid.to.total.FA | Cataract | 0.0345 | -0.05803 | -0.002002 | 0.001753 | -0.005438 | 0.001433 | 1.66 | 0.253 | 0.44 | Negative indirect effect |
| Metabolite | PDI_H | Ratio.of.linoleic.acid.to.total.FA | Glaucoma | -0.0438 | 0.03033 | -0.001328 | 0.000842 | -0.002978 | 0.000322 | 1.03 | 0.115 | 0.202 | Negative indirect effect |
| Metabolite | PDI_H | Ratio.of.linoleic.acid.to.total.FA | DR | -0.06523 | 0.07839 | -0.005114 | 0.001671 | -0.008389 | -0.001838 | 3.41 | 0.00221 | 0.0206 | Negative indirect effect |
| Metabolite | PDI_H | Ratio.of.linoleic.acid.to.total.FA | RVO | -0.04417 | 0.05546 | -0.00245 | 0.001197 | -0.004796 | -0.000104 | 1.51 | 0.0407 | 0.127 | Negative indirect effect |
| Metabolite | PDI_H | Ratio.of.polyunsaturated.FA.to.monounsaturated.FA | AMD | -0.04255 | 0.03851 | -0.001639 | 0.001649 | -0.004871 | 0.001594 | 0.89 | 0.32 | 0.555 | Negative indirect effect |
| Metabolite | PDI_H | Ratio.of.polyunsaturated.FA.to.monounsaturated.FA | Cataract | -0.07392 | 0.04454 | -0.003293 | 0.001374 | -0.005986 | -0.000599 | 2 | 0.0166 | 0.0581 | Negative indirect effect |
| Metabolite | PDI_H | Ratio.of.polyunsaturated.FA.to.monounsaturated.FA | Glaucoma | 0.06474 | 0.03943 | 0.002552 | 0.001136 | 0.000326 | 0.004779 | 1.48 | 0.0247 | 0.0812 | Positive indirect effect |
| Metabolite | PDI_H | Ratio.of.polyunsaturated.FA.to.monounsaturated.FA | DR | 0.11099 | -0.05922 | -0.006573 | 0.002133 | -0.010754 | -0.002392 | 4.37 | 0.00206 | 0.0193 | Negative indirect effect |
| Metabolite | PDI_H | Ratio.of.polyunsaturated.FA.to.monounsaturated.FA | RVO | 0.0349 | 0.03325 | 0.001161 | 0.000871 | -0.000546 | 0.002867 | 0.96 | 0.183 | 0.319 | Positive indirect effect |
| Metabolite | PDI_H | Polyunsaturated.FA | AMD | 0.0552 | 0.09737 | 0.005375 | 0.001804 | 0.001838 | 0.008911 | 3.67 | 0.00289 | 0.0263 | Positive indirect effect |
| Metabolite | PDI_H | Polyunsaturated.FA | Cataract | -0.05678 | 0.09914 | -0.00563 | 0.00186 | -0.009275 | -0.001984 | 4.11 | 0.00247 | 0.0228 | Negative indirect effect |
| Metabolite | PDI_H | Polyunsaturated.FA | Glaucoma | 0.04394 | 0.03196 | 0.001404 | 0.000791 | -0.000146 | 0.002955 | 1.16 | 0.0758 | 0.136 | Positive indirect effect |
| Metabolite | PDI_H | Polyunsaturated.FA | DR | 0.06749 | 0.10138 | 0.006842 | 0.002002 | 0.002918 | 0.010767 | 5.6 | 0.000633 | 0.015 | Positive indirect effect |
| Metabolite | PDI_H | Polyunsaturated.FA | RVO | 0.03065 | 0.02837 | 0.00087 | 0.000657 | -0.000417 | 0.002157 | 0.6 | 0.185 | 0.323 | Positive indirect effect |
| Metabolite | PDI_H | Linoleic.acid | AMD | -0.03347 | 0.04042 | -0.001353 | 0.001016 | -0.003344 | 0.000639 | 0.88 | 0.183 | 0.32 | Negative indirect effect |
| Metabolite | PDI_H | Linoleic.acid | Cataract | -0.05075 | 0.03104 | -0.001575 | 0.001503 | -0.004522 | 0.001371 | 0.98 | 0.295 | 0.511 | Negative indirect effect |
| Metabolite | PDI_H | Linoleic.acid | Glaucoma | 0.02561 | 0.04204 | 0.001077 | 0.000913 | -0.000713 | 0.002867 | 0.81 | 0.238 | 0.414 | Positive indirect effect |
| Metabolite | PDI_H | Linoleic.acid | DR | -0.10574 | -0.08453 | 0.008938 | 0.002527 | 0.003985 | 0.01389 | 6.87 | 0.000404 | 0.00987 | Positive indirect effect |
| Metabolite | PDI_H | Linoleic.acid | RVO | 0.04142 | -0.02913 | -0.001206 | 0.001221 | -0.003599 | 0.001186 | 0.75 | 0.323 | 0.559 | Negative indirect effect |
| Metabolite | PDI_H | Ratio.of.monounsaturated.FA.to.total.FA | AMD | -0.0366 | -0.03809 | 0.001394 | 0.00098 | -0.000526 | 0.003314 | 0.92 | 0.155 | 0.271 | Positive indirect effect |
| Metabolite | PDI_H | Ratio.of.monounsaturated.FA.to.total.FA | Cataract | -0.08637 | 0.04127 | -0.003564 | 0.001615 | -0.006729 | -0.000399 | 2.68 | 0.0273 | 0.0888 | Negative indirect effect |
| Metabolite | PDI_H | Ratio.of.monounsaturated.FA.to.total.FA | Glaucoma | -0.03876 | 0.02512 | -0.000974 | 0.000888 | -0.002713 | 0.000766 | 0.55 | 0.273 | 0.473 | Negative indirect effect |
| Metabolite | PDI_H | Ratio.of.monounsaturated.FA.to.total.FA | DR | -0.11799 | 0.06001 | -0.007081 | 0.002069 | -0.011136 | -0.003026 | 5.57 | 0.00062 | 0.0147 | Negative indirect effect |
| Metabolite | PDI_H | Ratio.of.monounsaturated.FA.to.total.FA | RVO | -0.05104 | 0.04361 | -0.002226 | 0.000737 | -0.003671 | -0.000781 | 1.86 | 0.00253 | 0.0232 | Negative indirect effect |
| Metabolite | PDI_H | PL.in.V.L.HDL | AMD | 0.03469 | 0.05716 | 0.001983 | 0.001349 | -0.000662 | 0.004628 | 1.39 | 0.142 | 0.249 | Positive indirect effect |
| Metabolite | PDI_H | PL.in.V.L.HDL | Cataract | 0.04045 | 0.03895 | 0.001575 | 0.000937 | -0.00026 | 0.003411 | 1.3 | 0.0925 | 0.164 | Positive indirect effect |
| Metabolite | PDI_H | PL.in.V.L.HDL | Glaucoma | 0.04072 | 0.02222 | 0.000905 | 0.001038 | -0.00113 | 0.00294 | 0.54 | 0.384 | 0.663 | Positive indirect effect |
| Metabolite | PDI_H | PL.in.V.L.HDL | DR | 0.04057 | 0.06352 | 0.002577 | 0.001824 | -0.000999 | 0.006152 | 2.05 | 0.158 | 0.276 | Positive indirect effect |
| Metabolite | PDI_H | PL.in.V.L.HDL | RVO | -0.05482 | 0.0306 | -0.001678 | 0.000827 | -0.003299 | -5.6e-05 | 1.15 | 0.0426 | 0.132 | Negative indirect effect |
| Metabolite | PDI_H | Con.of.V.L.HDL.particles | AMD | 0.05015 | 0.02495 | 0.001251 | 0.000825 | -0.000366 | 0.002868 | 0.92 | 0.129 | 0.227 | Positive indirect effect |
| Metabolite | PDI_H | Con.of.V.L.HDL.particles | Cataract | -0.0887 | -0.06272 | 0.005563 | 0.001415 | 0.002789 | 0.008337 | 3.05 | 8.46e-05 | 0.00268 | Positive indirect effect |
| Metabolite | PDI_H | Con.of.V.L.HDL.particles | Glaucoma | -0.04308 | 0.02651 | -0.001142 | 0.000998 | -0.003098 | 0.000813 | 0.68 | 0.252 | 0.438 | Negative indirect effect |
| Metabolite | PDI_H | Con.of.V.L.HDL.particles | DR | 0.05906 | 0.06151 | 0.003633 | 0.001544 | 0.000606 | 0.00666 | 2.2 | 0.0187 | 0.064 | Positive indirect effect |
| Metabolite | PDI_H | Con.of.V.L.HDL.particles | RVO | -0.04392 | 0.024 | -0.001054 | 0.00106 | -0.003131 | 0.001023 | 0.71 | 0.32 | 0.554 | Negative indirect effect |
| Metabolite | PDI_H | TLs.in.V.L.HDL | AMD | -0.06346 | -0.06564 | 0.004166 | 0.001417 | 0.001389 | 0.006942 | 2.35 | 0.00328 | 0.0296 | Positive indirect effect |
| Metabolite | PDI_H | TLs.in.V.L.HDL | Cataract | 0.07569 | -0.04777 | -0.003615 | 0.001675 | -0.006899 | -0.000332 | 2.31 | 0.0309 | 0.0991 | Negative indirect effect |
| Metabolite | PDI_H | TLs.in.V.L.HDL | Glaucoma | 0.03198 | -0.02789 | -0.000892 | 0.001056 | -0.002961 | 0.001177 | 0.69 | 0.398 | 0.688 | Negative indirect effect |
| Metabolite | PDI_H | TLs.in.V.L.HDL | DR | -0.11476 | -0.07436 | 0.008534 | 0.002479 | 0.003676 | 0.013392 | 5.67 | 0.000576 | 0.0137 | Positive indirect effect |
| Metabolite | PDI_H | TLs.in.V.L.HDL | RVO | -0.03658 | 0.05211 | -0.001906 | 0.000852 | -0.003576 | -0.000237 | 0.99 | 0.0252 | 0.0828 | Negative indirect effect |
| Metabolite | PDI_H | Valine | AMD | 0.0426 | 0.03396 | 0.001447 | 0.001166 | -0.000838 | 0.003732 | 0.95 | 0.215 | 0.374 | Positive indirect effect |
| Metabolite | PDI_H | Valine | Cataract | 0.04811 | 0.03606 | 0.001735 | 0.001599 | -0.0014 | 0.00487 | 1.08 | 0.278 | 0.482 | Positive indirect effect |
| Metabolite | PDI_H | Valine | Glaucoma | 0.02163 | 0.03819 | 0.000826 | 0.000651 | -0.00045 | 0.002102 | 0.65 | 0.204 | 0.356 | Positive indirect effect |
| Metabolite | PDI_H | Valine | DR | 0.07127 | -0.03408 | -0.002429 | 0.002835 | -0.007986 | 0.003128 | 1.74 | 0.392 | 0.677 | Negative indirect effect |
| Metabolite | PDI_H | Valine | RVO | -0.07544 | 0.04361 | -0.003289 | 0.000922 | -0.005097 | -0.001482 | 2.44 | 0.00036 | 0.00888 | Negative indirect effect |
| Metabolite | PDI_H | Albumin | AMD | -0.03813 | -0.03176 | 0.001211 | 0.001169 | -0.001081 | 0.003503 | 0.72 | 0.3 | 0.521 | Positive indirect effect |
| Metabolite | PDI_H | Albumin | Cataract | 0.06558 | -0.04959 | -0.003252 | 0.001128 | -0.005464 | -0.001041 | 1.94 | 0.00395 | 0.0353 | Negative indirect effect |
| Metabolite | PDI_H | Albumin | Glaucoma | 0.03688 | 0.02978 | 0.001098 | 0.0009 | -0.000665 | 0.002862 | 0.71 | 0.222 | 0.387 | Positive indirect effect |
| Metabolite | PDI_H | Albumin | DR | 0.079 | 0.05412 | 0.004275 | 0.001859 | 0.000632 | 0.007919 | 3.34 | 0.0215 | 0.072 | Positive indirect effect |
| Metabolite | PDI_H | Albumin | RVO | 0.03056 | -0.02837 | -0.000867 | 0.000534 | -0.001914 | 0.000181 | 0.72 | 0.105 | 0.185 | Negative indirect effect |
| Metabolite | PDI_H | Tyrosine | AMD | 0.0613 | 0.04046 | 0.00248 | 0.001222 | 8.5e-05 | 0.004875 | 1.96 | 0.0424 | 0.132 | Positive indirect effect |
| Metabolite | PDI_H | Tyrosine | Cataract | 0.05406 | 0.04429 | 0.002394 | 0.00109 | 0.000259 | 0.00453 | 1.42 | 0.028 | 0.0907 | Positive indirect effect |
| Metabolite | PDI_H | Tyrosine | Glaucoma | 0.02712 | -0.0396 | -0.001074 | 0.000748 | -0.002539 | 0.000391 | 0.74 | 0.151 | 0.264 | Negative indirect effect |
| Metabolite | PDI_H | Tyrosine | DR | -0.07573 | -0.08256 | 0.006253 | 0.002309 | 0.001728 | 0.010777 | 4.36 | 0.00676 | 0.0591 | Positive indirect effect |
| Metabolite | PDI_H | Tyrosine | RVO | -0.0735 | 0.03929 | -0.002888 | 0.001049 | -0.004944 | -0.000831 | 1.65 | 0.00593 | 0.052 | Negative indirect effect |

| Feature_type | Exposure | Mediator | Outcome | a_path_beta | b_path_beta | Indirect_effect | SE | CI_lower | CI_upper | Proportion_mediated_percent | P | FDR_q | Effect_direction |
|---|---|---|---|---|---|---|---|---|---|---|---|---|---|
| Metabolite | PDI_H | Free.chol.to.TLs.ratio.in.V.L.HDL | AMD | -0.05558 | 0.02796 | -0.001554 | 0.000987 | -0.003489 | 0.000381 | 0.91 | 0.116 | 0.204 | Negative indirect effect |
| Metabolite | PDI_H | Free.chol.to.TLs.ratio.in.V.L.HDL | Cataract | 0.06215 | -0.05966 | -0.003707 | 0.001239 | -0.006137 | -0.001278 | 2.54 | 0.00278 | 0.0254 | Negative indirect effect |
| Metabolite | PDI_H | Free.chol.to.TLs.ratio.in.V.L.HDL | Glaucoma | 0.05525 | 0.03398 | 0.001878 | 0.000839 | 0.000233 | 0.003522 | 1.13 | 0.0252 | 0.0828 | Positive indirect effect |
| Metabolite | PDI_H | Free.chol.to.TLs.ratio.in.V.L.HDL | DR | 0.04569 | -0.05983 | -0.002734 | 0.001305 | -0.005292 | -0.000176 | 2.2 | 0.0362 | 0.114 | Negative indirect effect |
| Metabolite | PDI_H | Free.chol.to.TLs.ratio.in.V.L.HDL | RVO | 0.0326 | -0.04076 | -0.001329 | 0.000625 | -0.002554 | -0.000104 | 0.95 | 0.0335 | 0.106 | Negative indirect effect |

## Supplementary Table 20. Associations between PDIs, ocular metrics, and ARED status

| Exposure_or_outcome | Ocular_metric | Standardized_beta | SE | CI_lower | CI_upper | P | FDR_q | FDR_significance |
|---|---|---|---|---|---|---|---|---|
| PDI_H | BCVA | -0.01 | 0.0071 | -0.0239 | 0.0039 | 0.159 | 0.537 | |
| PDI_H | IRT | -0.01 | 0.0104 | -0.0303 | 0.0103 | 0.334 | 0.52 | |
| PDI_H | INL | -0.03 | 0.0081 | -0.0459 | -0.0141 | 0.000218 | 0.000508 | *** |
| PDI_H | RGC | 0.03 | 0.0083 | 0.0138 | 0.0462 | 0.000296 | 0.000429 | *** |
| PDI_H | ORT | -0.01 | 0.0051 | -0.0201 | 0.0001 | 0.0519 | 0.0802 | |
| PDI_H | RNFL | 0.01 | 0.0074 | -0.0045 | 0.0245 | 0.177 | 0.255 | |
| PDI_H | RPE | -0.01 | 0.0098 | -0.0292 | 0.0092 | 0.307 | 0.635 | |
| PDI_H | MT | -0.01 | 0.0057 | -0.0212 | 0.0012 | 0.0806 | 0.158 | |
| PDI_H | CRAE | -0.05 | 0.0139 | -0.0773 | -0.0227 | 0.000328 | 0.000449 | *** |
| PDI_H | CRVE | -0.07 | 0.0168 | -0.103 | -0.037 | 3.16e-05 | 7.61e-05 | *** |
| PDI_H | CFDV | 0.05 | 0.0134 | 0.0237 | 0.0763 | 0.000192 | 0.000431 | *** |
| PDI_H | FDA | 0.04 | 0.0104 | 0.0196 | 0.0604 | 0.000117 | 0.000289 | *** |
| PDI_H | FDV | 0.05 | 0.012 | 0.0264 | 0.0736 | 3.17e-05 | 6.34e-05 | *** |
| PDI_H | TDA | 0.01 | 0.0094 | -0.0085 | 0.0285 | 0.29 | 0.54 | |
| PDI_H | TDV | 0 | 0.01 | -0.0196 | 0.0196 | 0.17 | 0.26 | |
| PDI_H | AODVL | 0 | 0.0092 | -0.018 | 0.018 | 0.065 | 0.102 | |
| PDI_H | CPAA | 0.01 | 0.0058 | -0.0014 | 0.0214 | 0.0844 | 0.0864 | |
| PDI_U | BCVA | -0.01 | 0.0066 | -0.0229 | 0.0029 | 0.13 | 0.461 | |
| PDI_U | IRT | -0.02 | 0.0084 | -0.0364 | -0.0036 | 0.0166 | 0.0406 | * |
| PDI_U | INL | -0.02 | 0.0092 | -0.038 | -0.002 | 0.0293 | 0.0406 | * |
| PDI_U | RGC | -0.02 | 0.0062 | -0.0322 | -0.0078 | 0.00134 | 0.00382 | ** |
| PDI_U | ORT | -0.01 | 0.0064 | -0.0226 | 0.0026 | 0.121 | 0.212 | |
| PDI_U | RNFL | 0.01 | 0.0086 | -0.0069 | 0.0269 | 0.246 | 0.576 | |
| PDI_U | RPE | 0 | 0.01 | -0.0195 | 0.0195 | 0.265 | 0.0723 | |
| PDI_U | MT | -0.02 | 0.0128 | -0.045 | 0.005 | 0.117 | 0.459 | |
| PDI_U | CRAE | -0.03 | 0.0081 | -0.0459 | -0.0141 | 0.00022 | 0.000323 | *** |
| PDI_U | CRVE | -0.02 | 0.0061 | -0.032 | -0.008 | 0.00104 | 0.00189 | ** |
| PDI_U | CFDV | -0.01 | 0.0092 | -0.0279 | 0.0079 | 0.275 | 0.413 | |
| PDI_U | FDA | -0.01 | 0.0067 | -0.0232 | 0.0032 | 0.138 | 0.099 | |
| PDI_U | FDV | -0.01 | 0.006 | -0.0217 | 0.0017 | 0.0938 | 0.283 | |
| PDI_U | TDA | 0 | 0.0076 | -0.0149 | 0.0149 | 0.201 | 0.0806 | |
| PDI_U | TDV | -0.01 | 0.0073 | -0.0243 | 0.0043 | 0.171 | 0.566 | |
| PDI_U | AODVL | -0.01 | 0.0108 | -0.0311 | 0.0111 | 0.353 | 0.561 | |
| PDI_U | CPAA | 0 | 0.0066 | -0.013 | 0.013 | 0.137 | 0.403 | |
| RVO | BCVA | 0 | 0.0064 | -0.0124 | 0.0124 | 0.206 | 0.518 | |
| RVO | IRT | 0 | 0.0097 | -0.019 | 0.019 | 0.263 | 0.611 | |
| RVO | INL | 0.01 | 0.0073 | -0.0044 | 0.0244 | 0.172 | 0.278 | |
| RVO | RGC | 0 | 0.0087 | -0.0171 | 0.0171 | 0.148 | 0.586 | |
| RVO | ORT | 0 | 0.0091 | -0.0179 | 0.0179 | 0.322 | 0.475 | |
| RVO | RNFL | -0.02 | 0.0075 | -0.0348 | -0.0052 | 0.00803 | 0.0163 | * |
| RVO | RPE | -0.01 | 0.0074 | -0.0244 | 0.0044 | 0.175 | 0.383 | |
| RVO | MT | 0 | 0.0085 | -0.0166 | 0.0166 | 0.284 | 0.475 | |
| RVO | CRAE | -0.01 | 0.0067 | -0.0232 | 0.0032 | 0.138 | 0.635 | |
| RVO | CRVE | 0.01 | 0.0071 | -0.0039 | 0.0239 | 0.16 | 0.237 | |
| RVO | CFDV | -0.03 | 0.0094 | -0.0484 | -0.0116 | 0.00136 | 0.00276 | ** |
| RVO | FDA | -0.03 | 0.0085 | -0.0466 | -0.0134 | 0.000408 | 0.000709 | *** |
| RVO | FDV | -0.02 | 0.0091 | -0.0378 | -0.0022 | 0.0274 | 0.0372 | * |
| RVO | TDA | 0.02 | 0.0086 | 0.0032 | 0.0368 | 0.0194 | 0.0357 | * |
| RVO | TDV | 0 | 0.0098 | -0.0192 | 0.0192 | 0.0693 | 0.13 | |
| RVO | AODVL | 0 | 0.0099 | -0.0194 | 0.0194 | 0.22 | 0.359 | |
| RVO | CPAA | -0.01 | 0.0079 | -0.0254 | 0.0054 | 0.204 | 0.33 | |
| DR | BCVA | 0.03 | 0.0087 | 0.0129 | 0.0471 | 0.000589 | 0.000801 | *** |
| DR | IRT | 0 | 0.0076 | -0.0148 | 0.0148 | 0.187 | 0.348 | |
| DR | INL | -0.01 | 0.006 | -0.0217 | 0.0017 | 0.0952 | 0.471 | |
| DR | RGC | -0.02 | 0.0125 | -0.0445 | 0.0045 | 0.109 | 0.161 | |
| DR | ORT | -0.01 | 0.0081 | -0.0259 | 0.0059 | 0.218 | 0.3 | |
| DR | RNFL | -0.01 | 0.0065 | -0.0227 | 0.0027 | 0.121 | 0.216 | |
| DR | RPE | -0.02 | 0.008 | -0.0357 | -0.0043 | 0.0125 | 0.0395 | * |
| DR | MT | -0.01 | 0.007 | -0.0238 | 0.0038 | 0.155 | 0.266 | |
| DR | CRAE | 0 | 0.0079 | -0.0154 | 0.0154 | 0.118 | 0.158 | |
| DR | CRVE | 0 | 0.006 | -0.0119 | 0.0119 | 0.0666 | 0.0931 | |
| DR | CFDV | -0.03 | 0.0079 | -0.0455 | -0.0145 | 0.000149 | 0.000237 | *** |
| DR | FDA | -0.03 | 0.0075 | -0.0447 | -0.0153 | 6.09e-05 | 0.000199 | *** |
| DR | FDV | -0.03 | 0.0082 | -0.0461 | -0.0139 | 0.000254 | 0.000707 | *** |
| DR | TDA | -0.01 | 0.0088 | -0.0272 | 0.0072 | 0.254 | 0.37 | |
| DR | TDV | 0 | 0.008 | -0.0157 | 0.0157 | 0.0779 | 0.152 | |
| DR | AODVL | -0.01 | 0.007 | -0.0237 | 0.0037 | 0.153 | 0.391 | |
| DR | CPAA | -0.01 | 0.0057 | -0.0212 | 0.0012 | 0.0807 | 0.213 | |
| Glaucoma | BCVA | 0.03 | 0.0084 | 0.0136 | 0.0464 | 0.000333 | 0.000474 | *** |
| Glaucoma | IRT | 0 | 0.0091 | -0.0178 | 0.0178 | 0.0551 | 0.092 | |
| Glaucoma | INL | 0 | 0.0081 | -0.0159 | 0.0159 | 0.0938 | 0.287 | |
| Glaucoma | RGC | -0.06 | 0.0158 | -0.0909 | -0.0291 | 0.000141 | 0.00023 | *** |
| Glaucoma | ORT | 0 | 0.0068 | -0.0133 | 0.0133 | 0.182 | 0.399 | |
| Glaucoma | RNFL | -0.05 | 0.0138 | -0.077 | -0.023 | 0.000279 | 0.000749 | *** |
| Glaucoma | RPE | -0.01 | 0.008 | -0.0257 | 0.0057 | 0.212 | 0.112 | |
| Glaucoma | MT | -0.05 | 0.0121 | -0.0737 | -0.0263 | 3.59e-05 | 4.93e-05 | *** |
| Glaucoma | CRAE | -0.04 | 0.011 | -0.0615 | -0.0185 | 0.000265 | 0.000645 | *** |
| Glaucoma | CRVE | -0.03 | 0.0084 | -0.0464 | -0.0136 | 0.000333 | 0.000533 | *** |
| Glaucoma | CFDV | -0.05 | 0.0125 | -0.0745 | -0.0255 | 6.42e-05 | 0.00019 | *** |
| Glaucoma | FDA | -0.04 | 0.0115 | -0.0625 | -0.0175 | 0.000482 | 0.000811 | *** |
| Glaucoma | FDV | -0.05 | 0.0135 | -0.0764 | -0.0236 | 0.000205 | 0.000369 | *** |
| Glaucoma | TDA | 0.01 | 0.0058 | -0.0014 | 0.0214 | 0.0843 | 0.168 | |
| Glaucoma | TDV | 0.02 | 0.0082 | 0.004 | 0.036 | 0.0144 | 0.0232 | * |
| Glaucoma | AODVL | -0.01 | 0.0062 | -0.0222 | 0.0022 | 0.108 | 0.465 | |
| Glaucoma | CPAA | 0 | 0.0082 | -0.0161 | 0.0161 | 0.372 | 0.633 | |
| AMD | BCVA | 0.11 | 0.0299 | 0.0515 | 0.1685 | 0.000229 | 0.000692 | *** |
| AMD | IRT | 0 | 0.009 | -0.0176 | 0.0176 | 0.0613 | 0.303 | |
| AMD | INL | -0.03 | 0.0091 | -0.0478 | -0.0122 | 0.000944 | 0.00197 | ** |
| AMD | RGC | -0.07 | 0.0186 | -0.1065 | -0.0335 | 0.000169 | 0.0003 | *** |
| AMD | ORT | -0.02 | 0.0082 | -0.036 | -0.004 | 0.0142 | 0.0191 | * |

| Exposure_or_outcome | Ocular_metric | Standardized_beta | SE | CI_lower | CI_upper | P | FDR_q | FDR_significance |
|---|---|---|---|---|---|---|---|---|
| AMD | RNFL | -0.03 | 0.0076 | -0.045 | -0.015 | 8.63e-05 | 0.000332 | *** |
| AMD | RPE | -0.03 | 0.0074 | -0.0446 | -0.0154 | 5.37e-05 | 0.000141 | *** |
| AMD | MT | -0.05 | 0.0145 | -0.0784 | -0.0216 | 0.00055 | 0.000785 | *** |
| AMD | CRAE | -0.07 | 0.0194 | -0.1079 | -0.0321 | 0.000299 | 0.000516 | *** |
| AMD | CRVE | -0.05 | 0.0138 | -0.0771 | -0.0229 | 0.000301 | 0.000667 | *** |
| AMD | CFDV | -0.12 | 0.028 | -0.1749 | -0.0651 | 1.82e-05 | 3.53e-05 | *** |
| AMD | FDA | -0.12 | 0.0322 | -0.1832 | -0.0568 | 0.000198 | 0.000667 | *** |
| AMD | FDV | -0.11 | 0.0293 | -0.1674 | -0.0526 | 0.000171 | 0.000312 | *** |
| AMD | TDA | 0.01 | 0.0056 | -0.0009 | 0.0209 | 0.0731 | 0.197 | |
| AMD | TDV | 0.01 | 0.0081 | -0.0059 | 0.0259 | 0.217 | 0.295 | |
| AMD | AODVL | 0 | 0.0098 | -0.0192 | 0.0192 | 0.151 | 0.223 | |
| AMD | CPAA | 0.01 | 0.0068 | -0.0033 | 0.0233 | 0.14 | 0.196 | |
| Cataract | BCVA | 0.06 | 0.0154 | 0.0299 | 0.0901 | 9.35e-05 | 0.00013 | *** |
| Cataract | IRT | 0.01 | 0.0082 | -0.0061 | 0.0261 | 0.223 | 0.298 | |
| Cataract | INL | -0.02 | 0.0125 | -0.0444 | 0.0044 | 0.109 | 0.519 | |
| Cataract | RGC | -0.04 | 0.0107 | -0.0609 | -0.0191 | 0.000178 | 0.000836 | *** |
| Cataract | ORT | -0.01 | 0.0077 | -0.0251 | 0.0051 | 0.193 | 0.362 | |
| Cataract | RNFL | 0 | 0.0082 | -0.0161 | 0.0161 | 0.175 | 0.322 | |
| Cataract | RPE | 0.01 | 0.0064 | -0.0026 | 0.0226 | 0.121 | 0.236 | |
| Cataract | MT | -0.02 | 0.0081 | -0.0358 | -0.0042 | 0.0133 | 0.0247 | * |
| Cataract | CRAE | -0.03 | 0.0079 | -0.0455 | -0.0145 | 0.00015 | 0.000383 | *** |
| Cataract | CRVE | -0.01 | 0.0069 | -0.0235 | 0.0035 | 0.147 | 0.229 | |
| Cataract | CFDV | -0.07 | 0.0191 | -0.1074 | -0.0326 | 0.000245 | 0.000449 | *** |
| Cataract | FDA | -0.07 | 0.0182 | -0.1057 | -0.0343 | 0.000123 | 0.000197 | *** |
| Cataract | FDV | -0.07 | 0.0198 | -0.1087 | -0.0313 | 0.000396 | 0.000686 | *** |
| Cataract | TDA | 0 | 0.0077 | -0.0151 | 0.0151 | 0.0935 | 0.236 | |
| Cataract | TDV | -0.02 | 0.0078 | -0.0352 | -0.0048 | 0.0101 | 0.0298 | * |
| Cataract | AODVL | 0 | 0.0087 | -0.0171 | 0.0171 | 0.364 | 0.498 | |
| Cataract | CPAA | 0 | 0.0069 | -0.0135 | 0.0135 | 0.0526 | 0.217 | |

## Supplementary Table 21. Incremental predictive performance after adding PDI-H and PDI-U

| Outcome | Dataset | Model | N_cases | N_controls | AUC_base_without_PDI | AUC base_95CI | AUC_full_with_PDI_H_PDI_U | AUC full_95CI | Delta_AUC | Delta AUC_95CI | DeLong_P_value | Accuracy_without_PDI_percent | Accuracy_with_PDI_percent | Delta_accuracy_percentage_points | Sensitivity_without_PDI_percent | Sensitivity_with_PDI_percent | Delta_sensitivity_percentage_points | Specificity_without_PDI_percent | Specificity_with_PDI_percent | Delta_specificity_percentage_points | Precision_without_PDI_percent | Precision_with_PDI_percent | Delta_precision_percentage_points | F1_without_PDI_percent | F1_with_PDI_percent | Delta_F1_percentage_points |
|---|---|---|---|---|---|---|---|---|---|---|---|---|---|---|---|---|---|---|---|---|---|---|---|---|---|---|
| AMD | UKB validation | Logistic regression | 946 | 37755 | 0.786 | 0.769–0.803 | 0.827 | 0.811–0.843 | 0.041 | 0.024–0.058 | 1.94e-06 | | | | | | | | | | | | | | | |
| AMD | UKB validation | XGBoost | 946 | 37755 | 0.812 | 0.795–0.829 | 0.855 | 0.840–0.870 | 0.043 | 0.027–0.059 | 1.55e-07 | 77.5 | 77.6 | 0.1 | 77.7 | 83.5 | 5.8 | 77.5 | 77.5 | 0 | 8 | 8.5 | 0.5 | 14.4 | 15.4 | 1 |
| AMD | Tianjin | Logistic regression | 1328 | 4649 | 0.736 | 0.720–0.752 | 0.775 | 0.759–0.791 | 0.039 | 0.023–0.055 | 2.09e-06 | | | | | | | | | | | | | | | |
| AMD | Tianjin | XGBoost | 1328 | 4649 | 0.778 | 0.762–0.794 | 0.819 | 0.804–0.834 | 0.041 | 0.026–0.056 | 1.08e-07 | 75 | 76.9 | 1.9 | 75 | 79.8 | 4.8 | 75 | 76.1 | 1.1 | 46.1 | 48.8 | 2.7 | 57.1 | 60.6 | 3.5 |
| Cataract | UKB validation | Logistic regression | 2888 | 35812 | 0.8 | 0.790–0.810 | 0.842 | 0.833–0.851 | 0.042 | 0.032–0.052 | 4.89e-18 | | | | | | | | | | | | | | | |
| Cataract | UKB validation | XGBoost | 2888 | 35812 | 0.813 | 0.803–0.823 | 0.856 | 0.847–0.865 | 0.043 | 0.034–0.052 | 8.48e-20 | 80.1 | 81.1 | 1 | 77.9 | 83.5 | 5.6 | 80.3 | 80.9 | 0.6 | 24.2 | 26 | 1.8 | 36.9 | 39.7 | 2.8 |
| Cataract | Tianjin | Logistic regression | 1687 | 4290 | 0.754 | 0.739–0.769 | 0.794 | 0.780–0.808 | 0.04 | 0.026–0.054 | 3.61e-08 | | | | | | | | | | | | | | | |
| Cataract | Tianjin | XGBoost | 1687 | 4290 | 0.798 | 0.784–0.812 | 0.84 | 0.827–0.853 | 0.042 | 0.029–0.055 | 3.65e-10 | 80.5 | 83.3 | 2.8 | 79.5 | 84.3 | 4.8 | 80.9 | 82.9 | 2 | 62.1 | 66 | 3.9 | 69.7 | 74 | 4.3 |

Supplementary Figures

Supplementary Figure 1

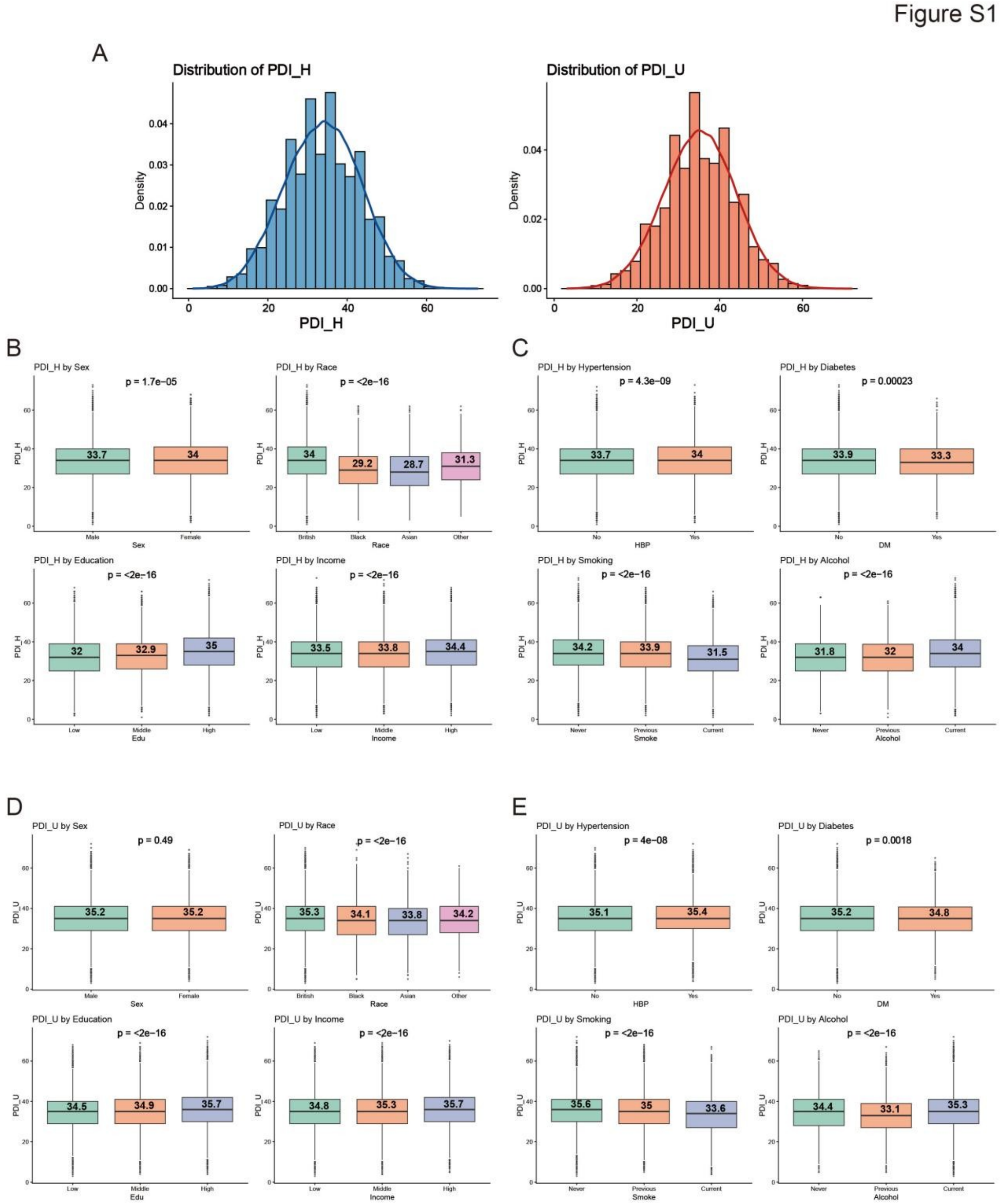


**Supplementary Figure 1. Distribution and determinants of PDI-H and PDI-U in the UK Biobank**

(A) Distribution of PDI-H and PDI-U in the UK Biobank cohort.

(B) PDI-H stratified by sex, race, education, and income.

(C) PDI-H stratified by hypertension, diabetes, smoking status, and alcohol consumption.

(D) PDI-U stratified by sex, race, education, and income.

(E) PDI-U stratified by hypertension, diabetes, smoking status, and alcohol consumption.

Boxplots show median and interquartile range; P values are derived from group comparisons.

Supplementary Figure 2

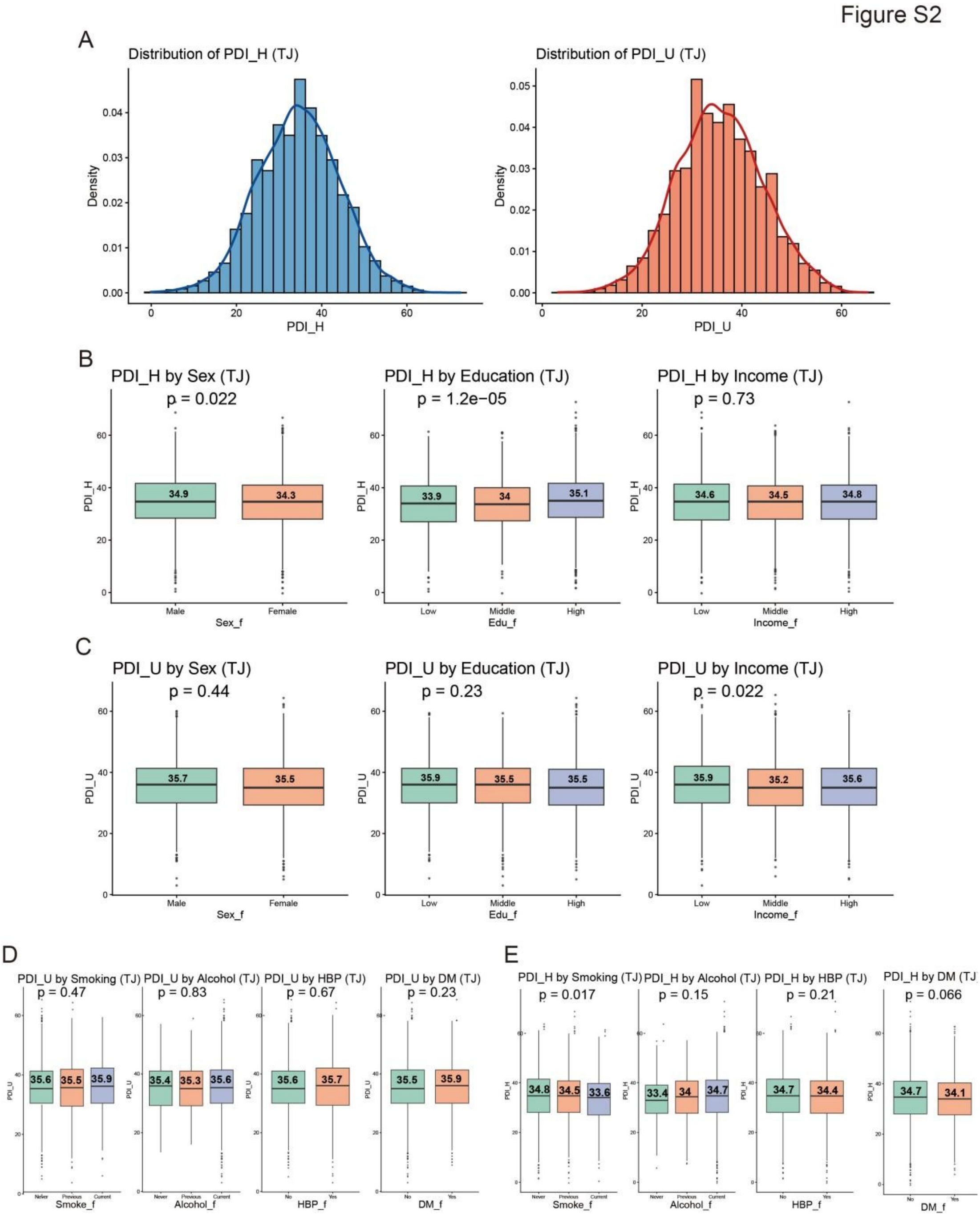


**Supplementary Figure 2. Distribution and determinants of PDI-H and PDI-U in the Tianjin cohort**

(A) Distribution of PDI-H and PDI-U in the Tianjin cohort.
(B) PDI-H stratified by sex, education, and income.
(C) PDI-U stratified by sex, education, and income.
(D) PDI-U stratified by smoking, alcohol consumption, hypertension, and diabetes.
(E) PDI-H stratified by smoking, alcohol consumption, hypertension, and diabetes. Boxplots show median and interquartile range.

Supplementary Figure 3

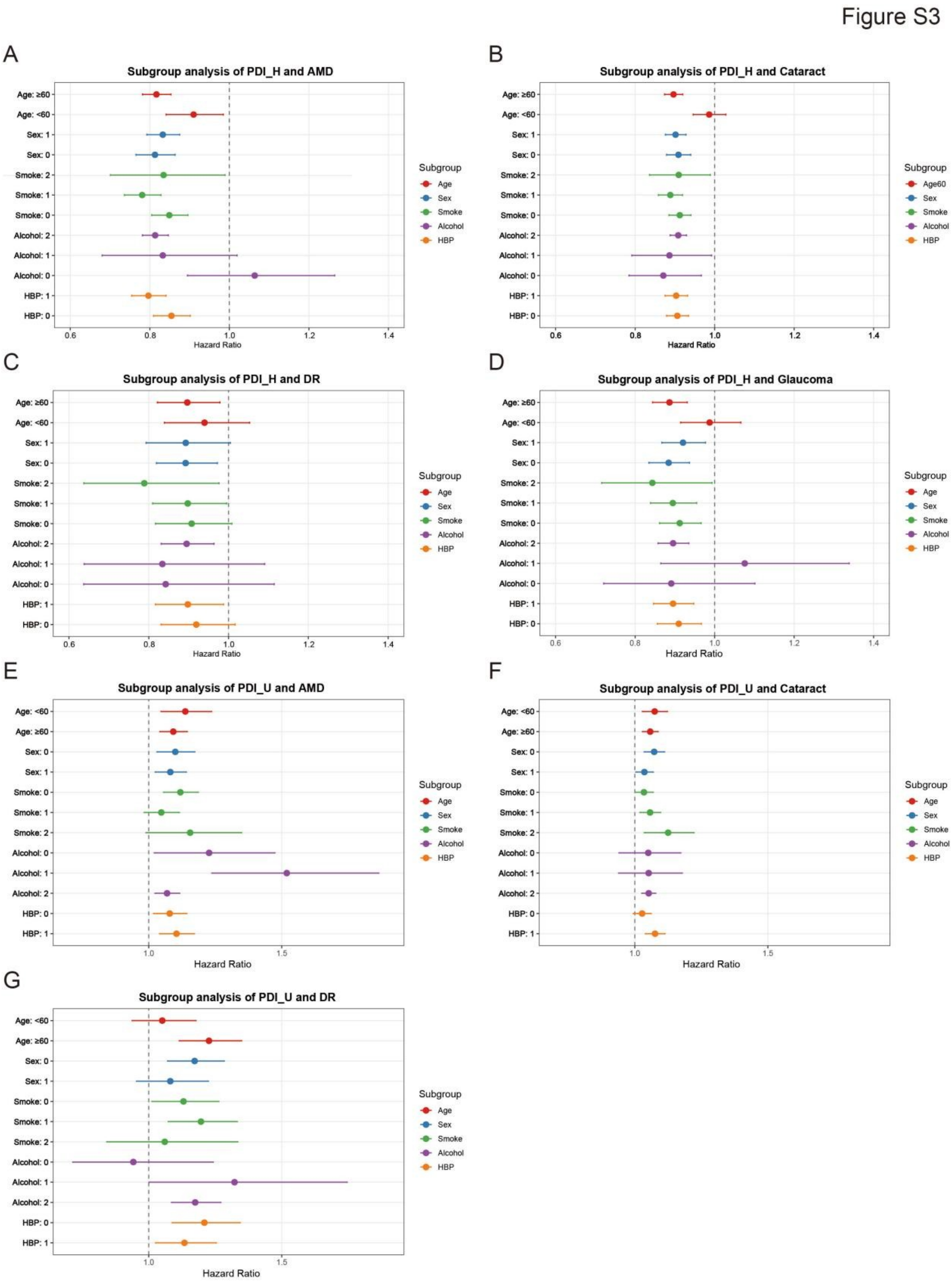


**Supplementary Figure 3. Subgroup analyses of PDI-H associations with AREDs**

(A–D) Subgroup analyses of associations between PDI-H and AMD, cataract, DR, and glaucoma in the UK Biobank, stratified by age, sex, smoking, alcohol consumption, and hypertension. Points

represent hazard ratios and bars indicate 95% confidence intervals.

Supplementary Figure 4

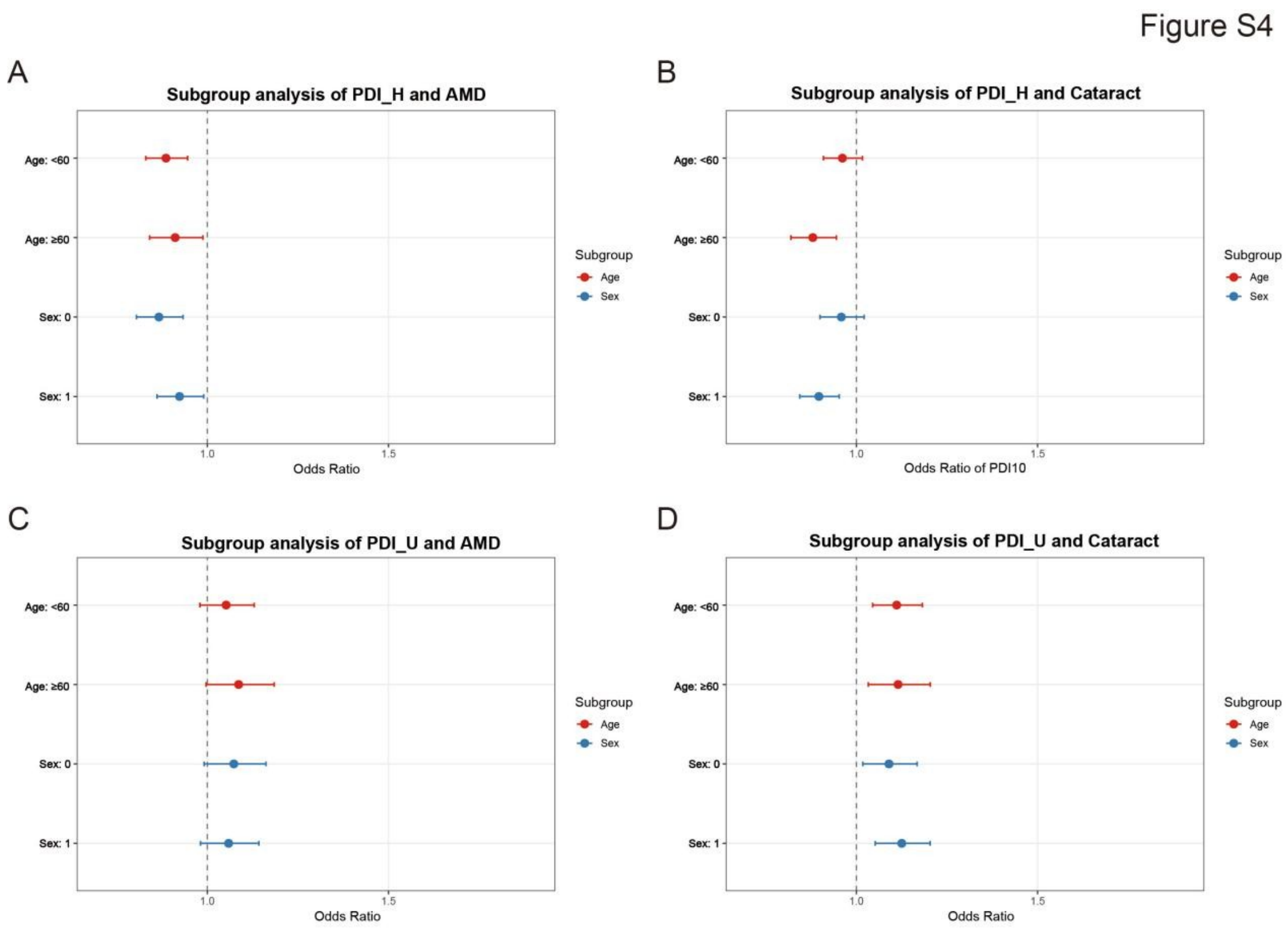


**Supplementary Figure 4. Subgroup analyses of PDI-H and PDI-U in the Tianjin cohort**

(A–B) Subgroup analyses of associations between PDI-H and AMD and cataract in the Tianjin cohort.
(C–D) Subgroup analyses of associations between PDI-U and AMD and cataract in the Tianjin cohort. Odds ratios and 95% confidence intervals are shown.

Supplementary Figure 5

Figure S5

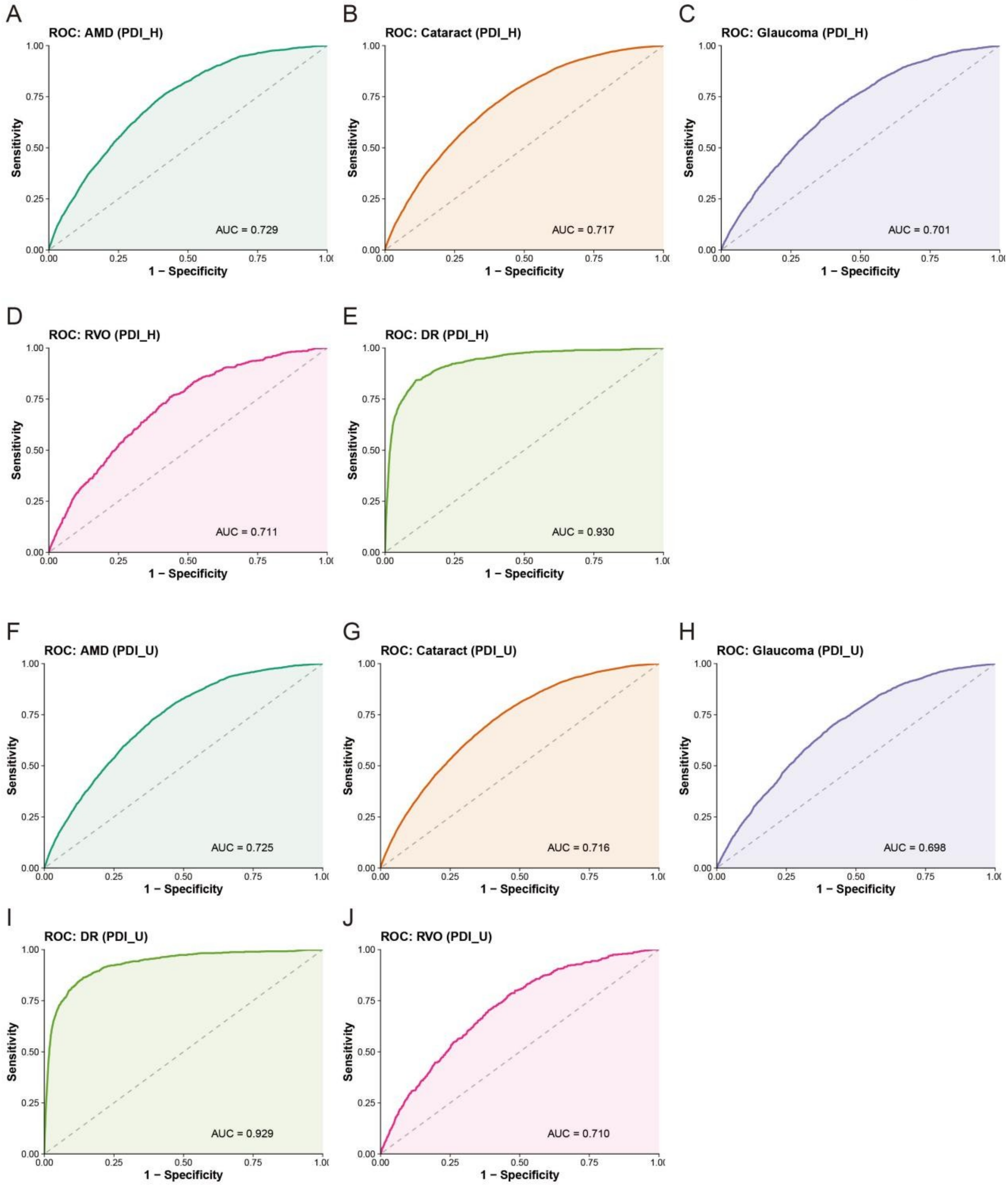


**Supplementary Figure 5. Predictive performance of PDI-H and PDI-U alone**

(A–E) ROC curves showing discrimination of PDI-H alone for AMD, cataract, glaucoma, RVO, and DR.
(F–J) ROC curves showing discrimination of PDI-U alone for the same outcomes. AUC values are shown in each panel.

Supplementary Figure 6

Figure S6

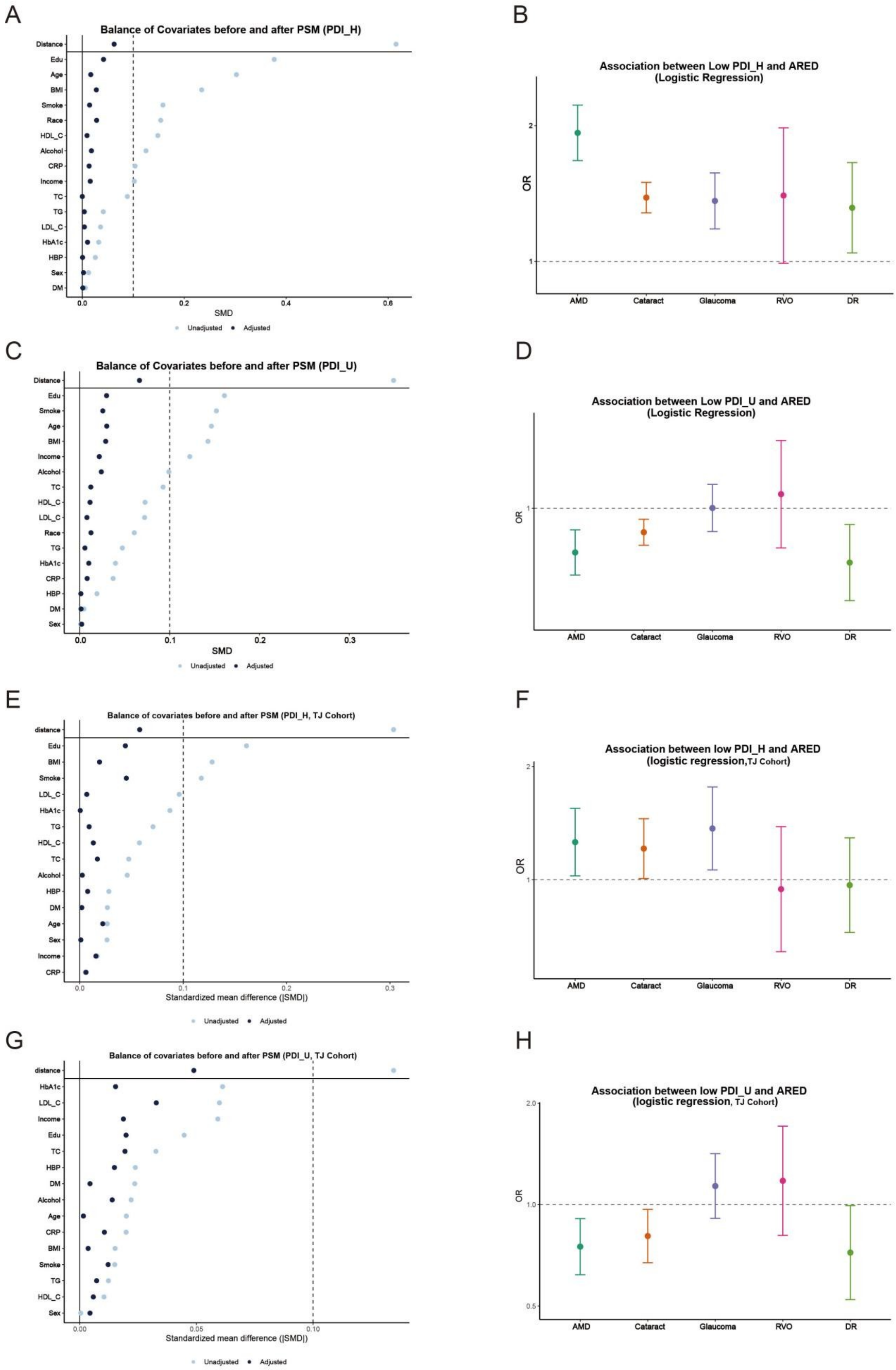


**Supplementary Figure 6. Covariate balance and PSM sensitivity analyses**

(A–C) Standardized mean differences of covariates before and after propensity score matching (PSM) for PDI-H and PDI-U in the UK Biobank.
(D) Logistic regression estimates of associations between low vs high PDI-H and AREDs after PSM in UKB.
(E–G) Covariate balance before and after PSM in the Tianjin cohort.
(H) Logistic regression estimates of associations between low vs high PDI-U and AREDs after PSM in the Tianjin cohort.

Supplementary Figure 7

Figure S7

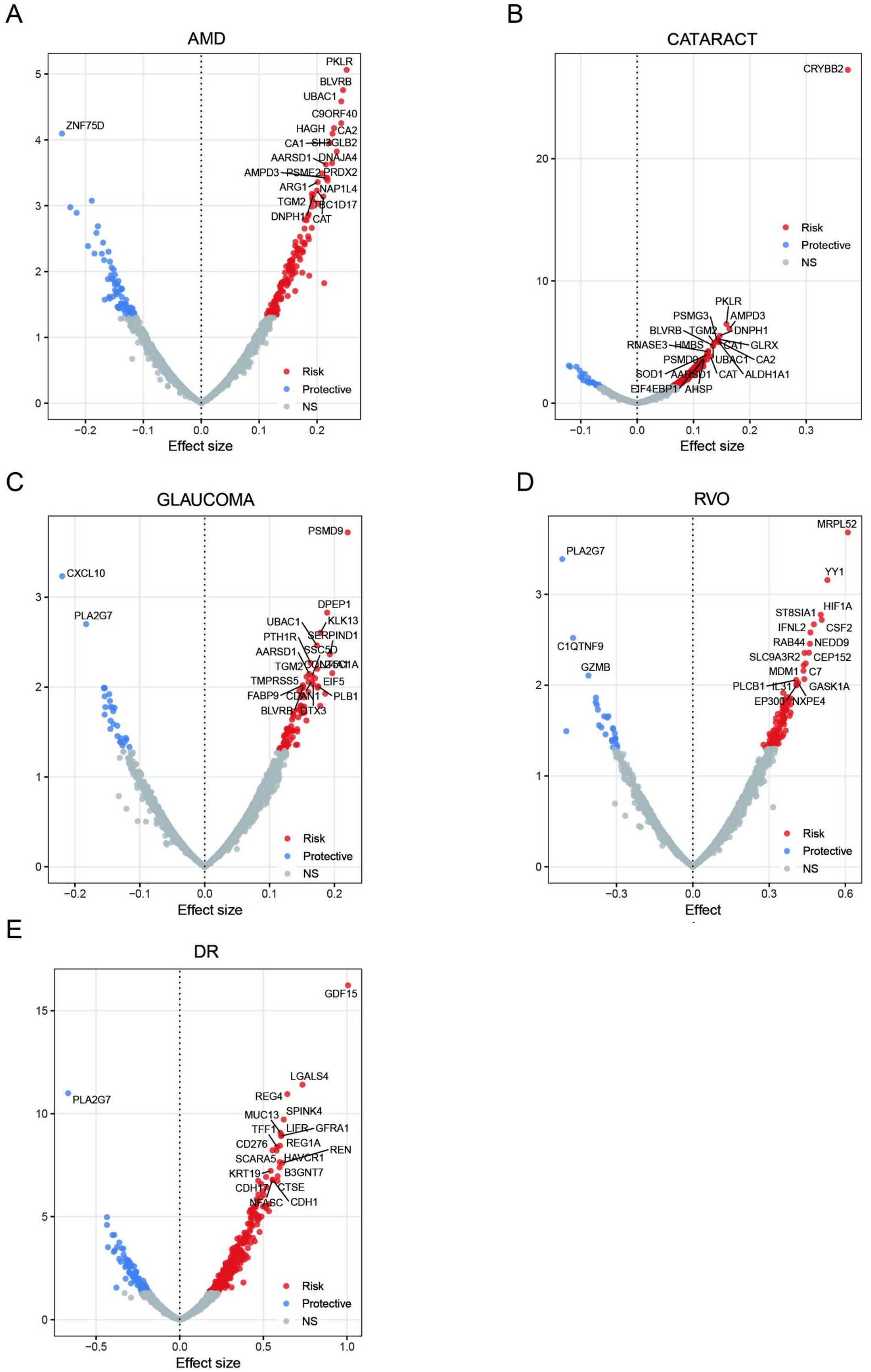


**Supplementary Figure 7. Disease-specific proteomic signatures**

(A–E) Volcano plots showing associations between circulating proteins and AMD, cataract, glaucoma, RVO, and DR, respectively. Red points indicate risk-associated proteins and blue points indicate protective proteins.

Supplementary Figure 8

Figure S8

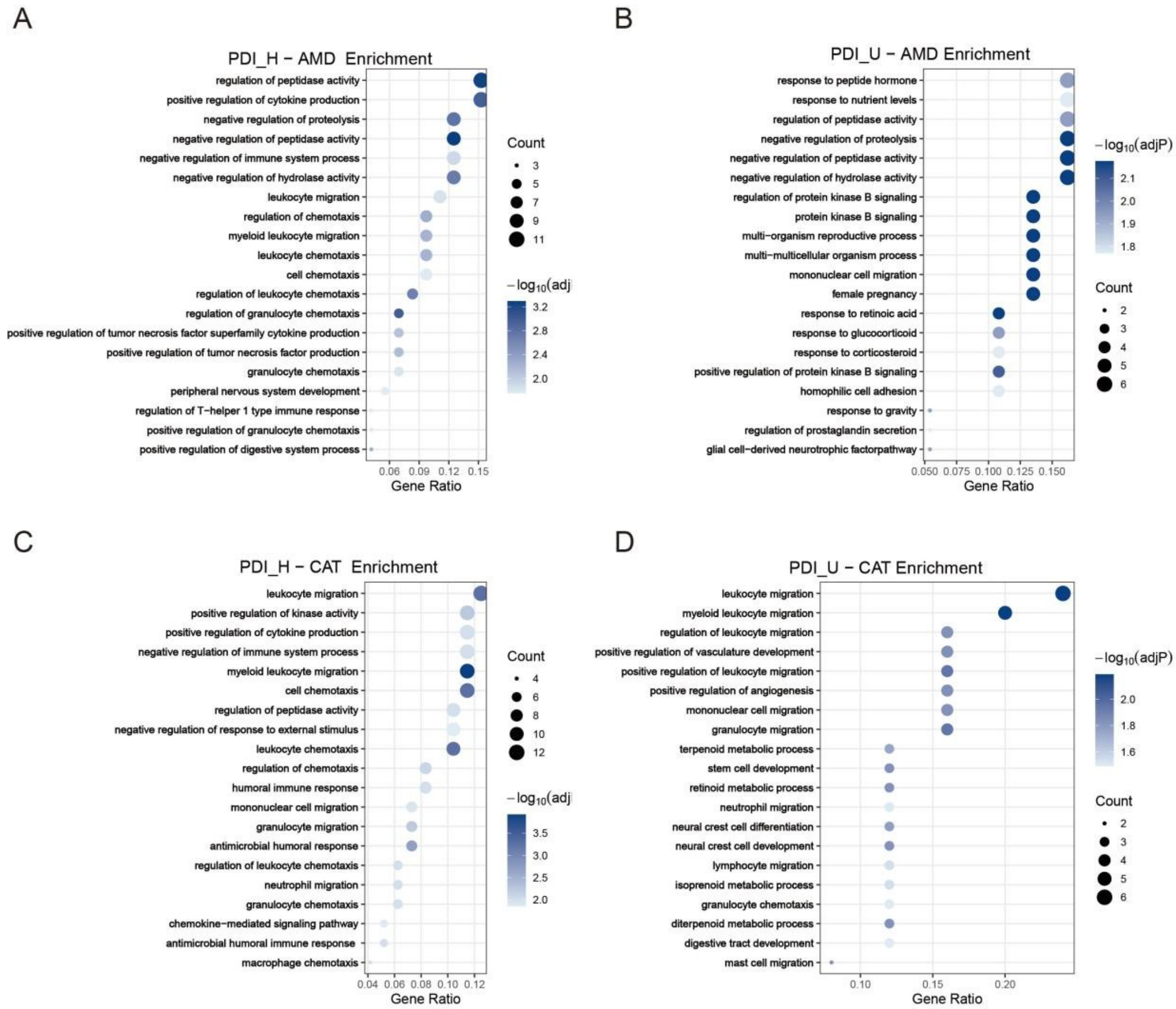


**Supplementary Figure 8. Functional enrichment of diet-associated proteins**

(A–B) Gene Ontology biological process enrichment of PDI-H– and PDI-U–associated proteins for AMD.

(C–D) Enrichment results for cataract. Bubble size represents gene count and color indicates adjusted P value.

Supplementary Figure 9

Figure S9

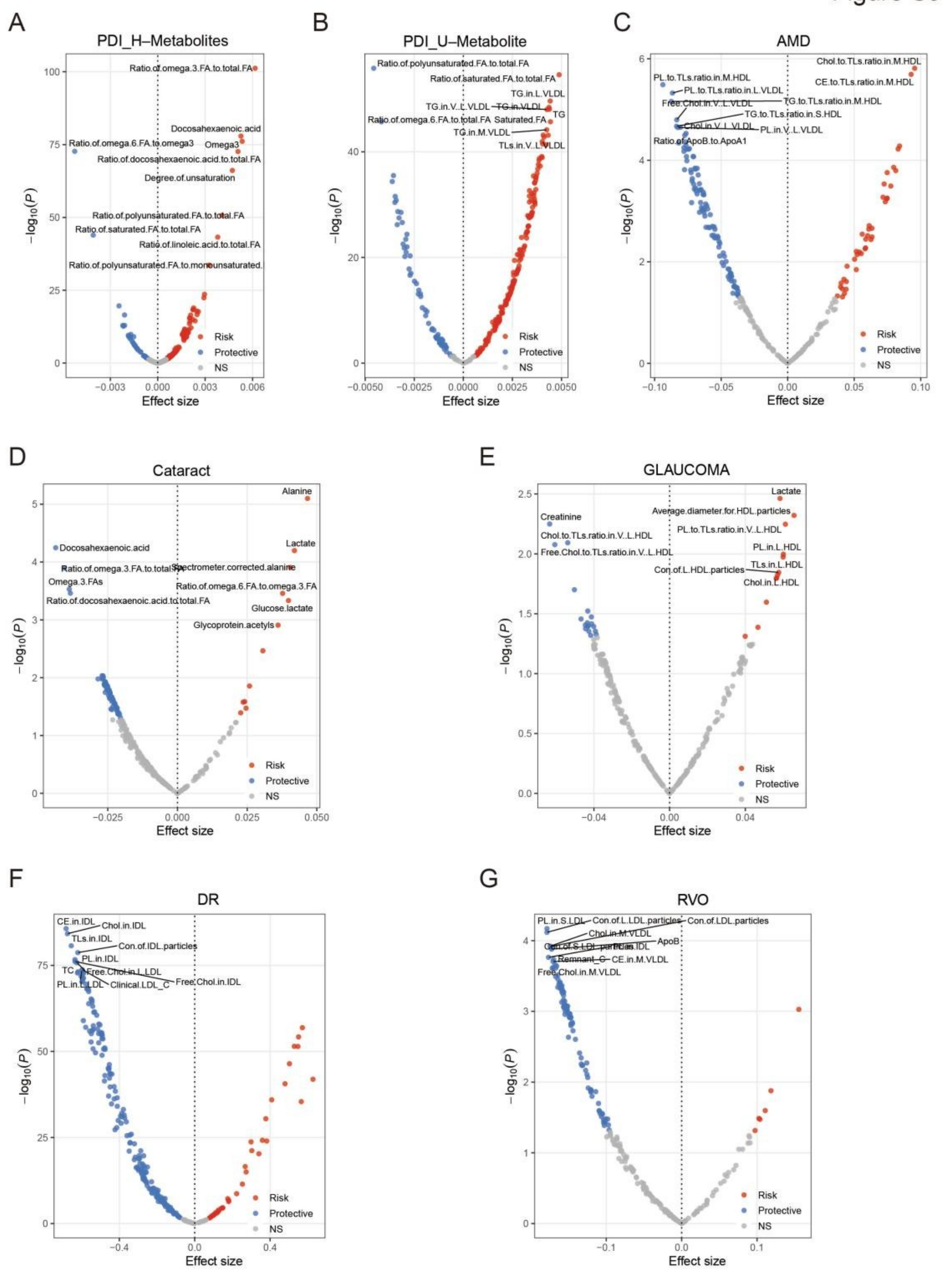


**Supplementary Figure 9. Disease-specific metabolomic signatures**

(A–B) Associations between PDI-H and PDI-U and circulating metabolites.

(C–G) Volcano plots showing metabolite associations with AMD, cataract, glaucoma, DR, and RVO, respectively. Red indicates risk-associated metabolites and blue indicates protective metabolites.

Supplementary Figure 10

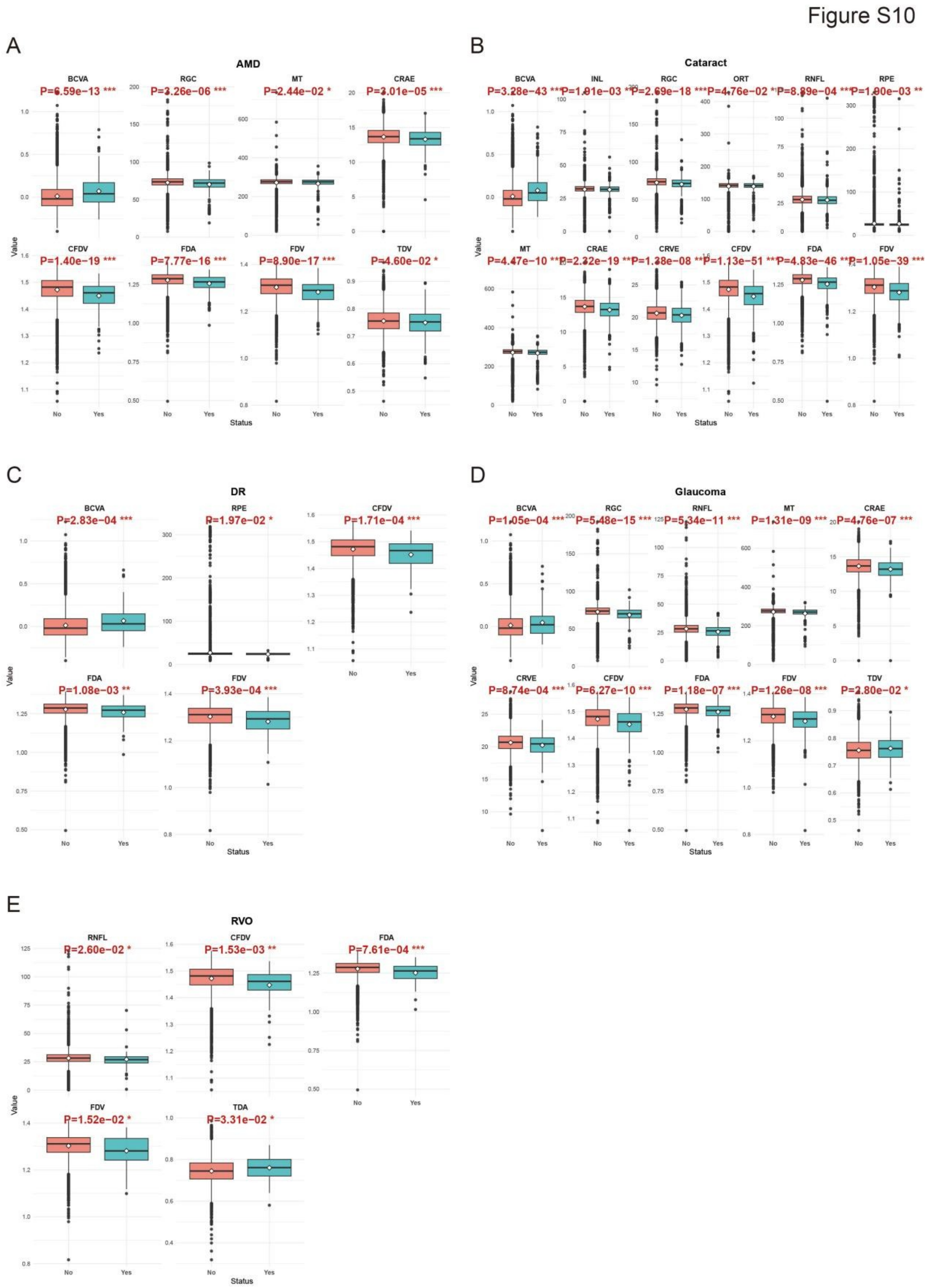


**Supplementary Figure 10. Differences in ocular metrics by disease status**

(A–E) Comparisons of ocular structural and vascular metrics between disease cases and controls for AMD, cataract, DR, glaucoma, and RVO, respectively. Boxplots show distribution of metrics by disease status, with P values from group comparisons.

Supplementary Figure 11

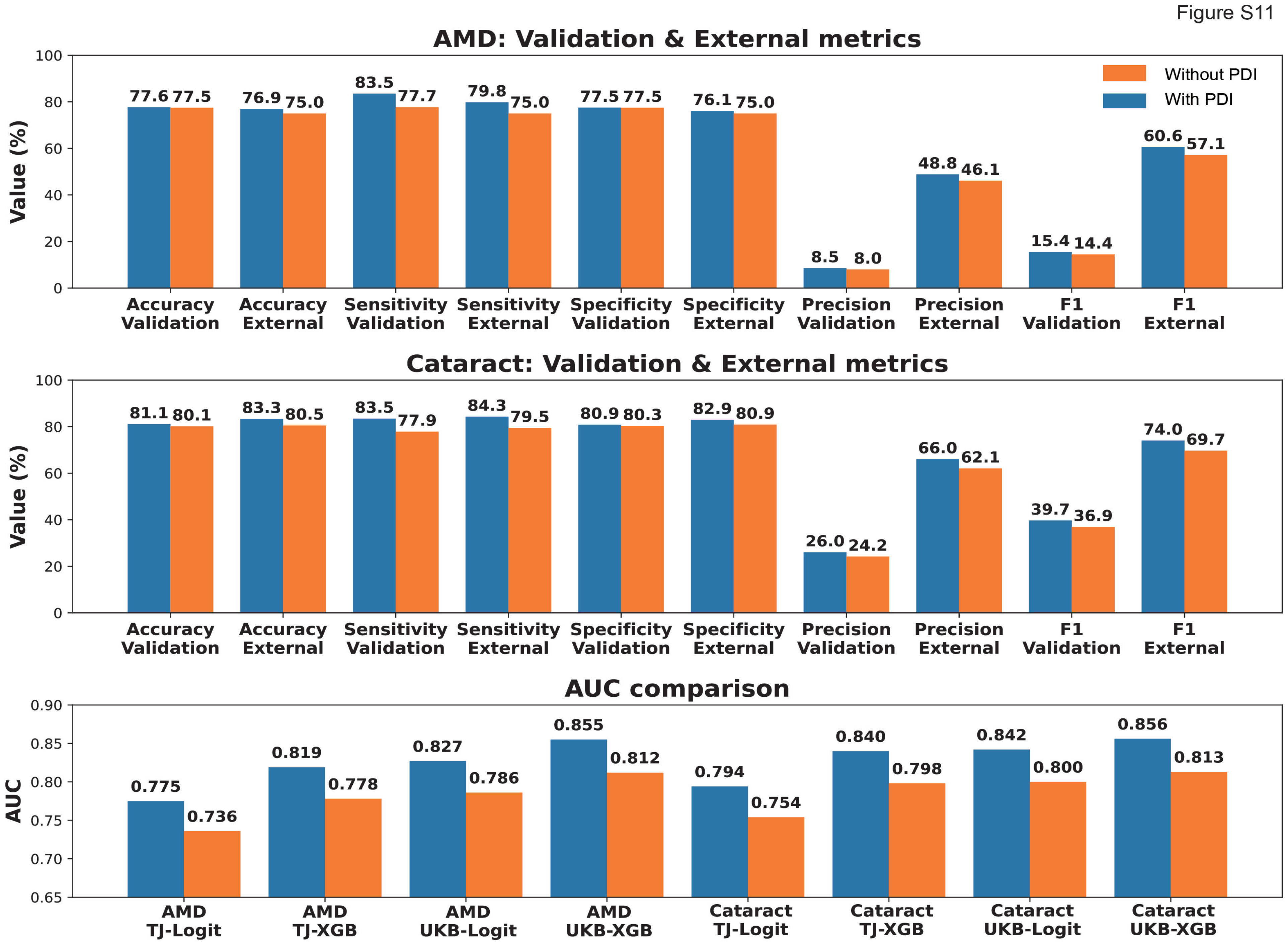


Supplementary Figure 11. Incremental predictive value of dietary indices for AMD and cataract models.

The upper panel shows validation and external-validation classification metrics for AMD models with and without PDI-H/PDI-U. The middle panel shows the corresponding metrics for cataract models. The lower panel summarizes AUC comparisons for logistic regression and XGBoost models in the UKB and TJ datasets. Bars indicate model performance with and without dietary indices.
